# Expression of concern on: Comparative genomics and characterization of SARS-CoV-2 P.1 (Gamma) variant of concern from Amazonas, Brazil

**DOI:** 10.3389/fmed.2024.1393692

**Published:** 2024-03-26

**Authors:** Frontiers Editorial Office

With this notice, Frontiers states its awareness of ethics concerns surrounding the article “Comparative Genomics and Characterization of SARS-CoV-2 P.1 (Gamma) Variant of Concern From Amazonas, Brazil” published on 15 February 2022. An investigation is currently being conducted by The Federal University of Health Sciences of Porto Alegre, in accordance with applicable institutional policies and procedures. This expression of concern has been posted while Frontiers awaits the outcome of that investigation. It will be updated accordingly after that time.

