# Supplementary figures and images for: Comparative Genomics and Characterization of SARS-CoV-2 P.1 (Gamma) Variant of Concern From Amazonas, Brazil
Source: Front Med (Lausanne). 2022 Feb 15;9:806611. doi: 10.3389/fmed.2022.806611 (PMC8885995; doi:10.3389/fmed.2022.806611)

38966

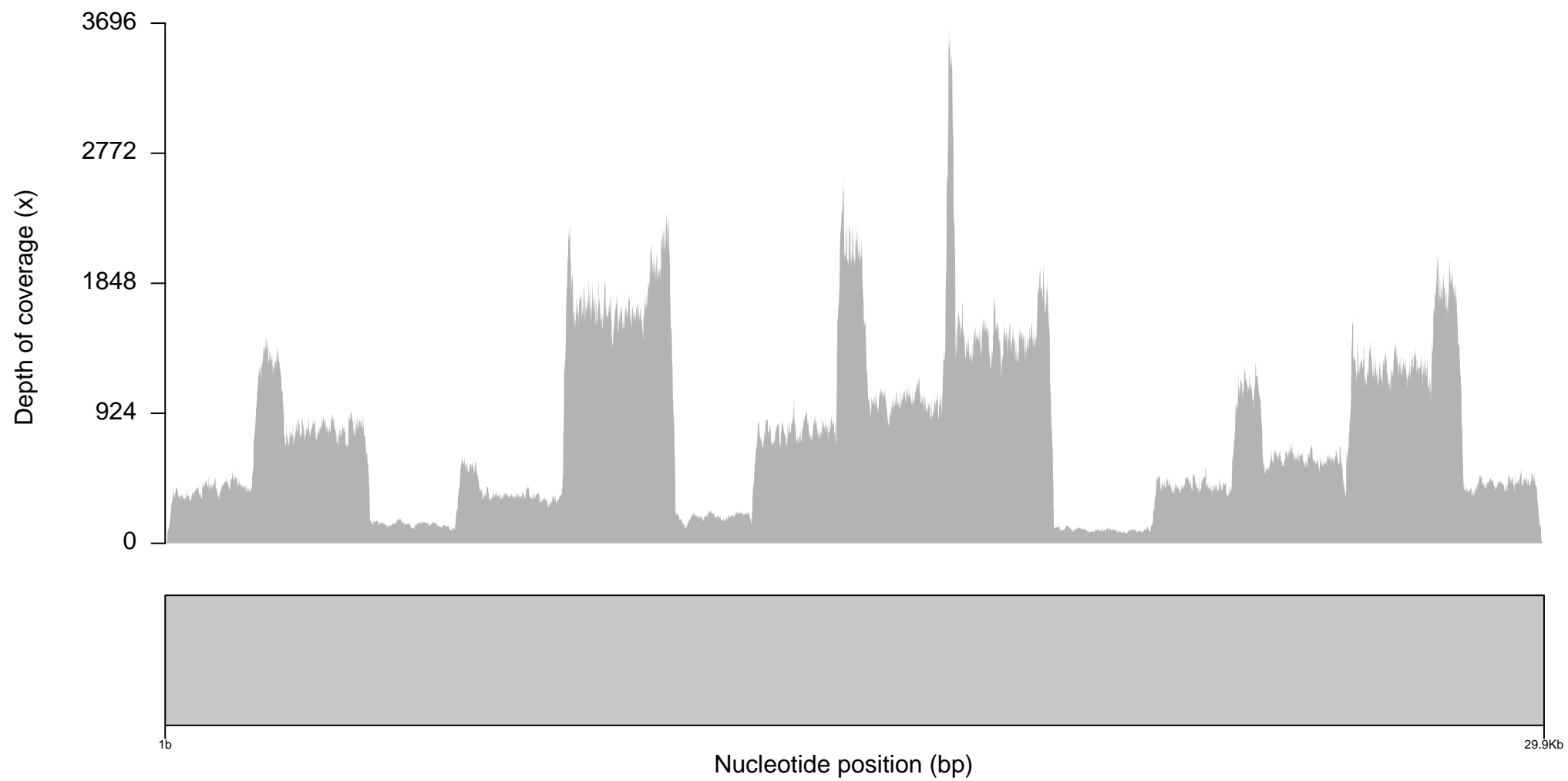

38967

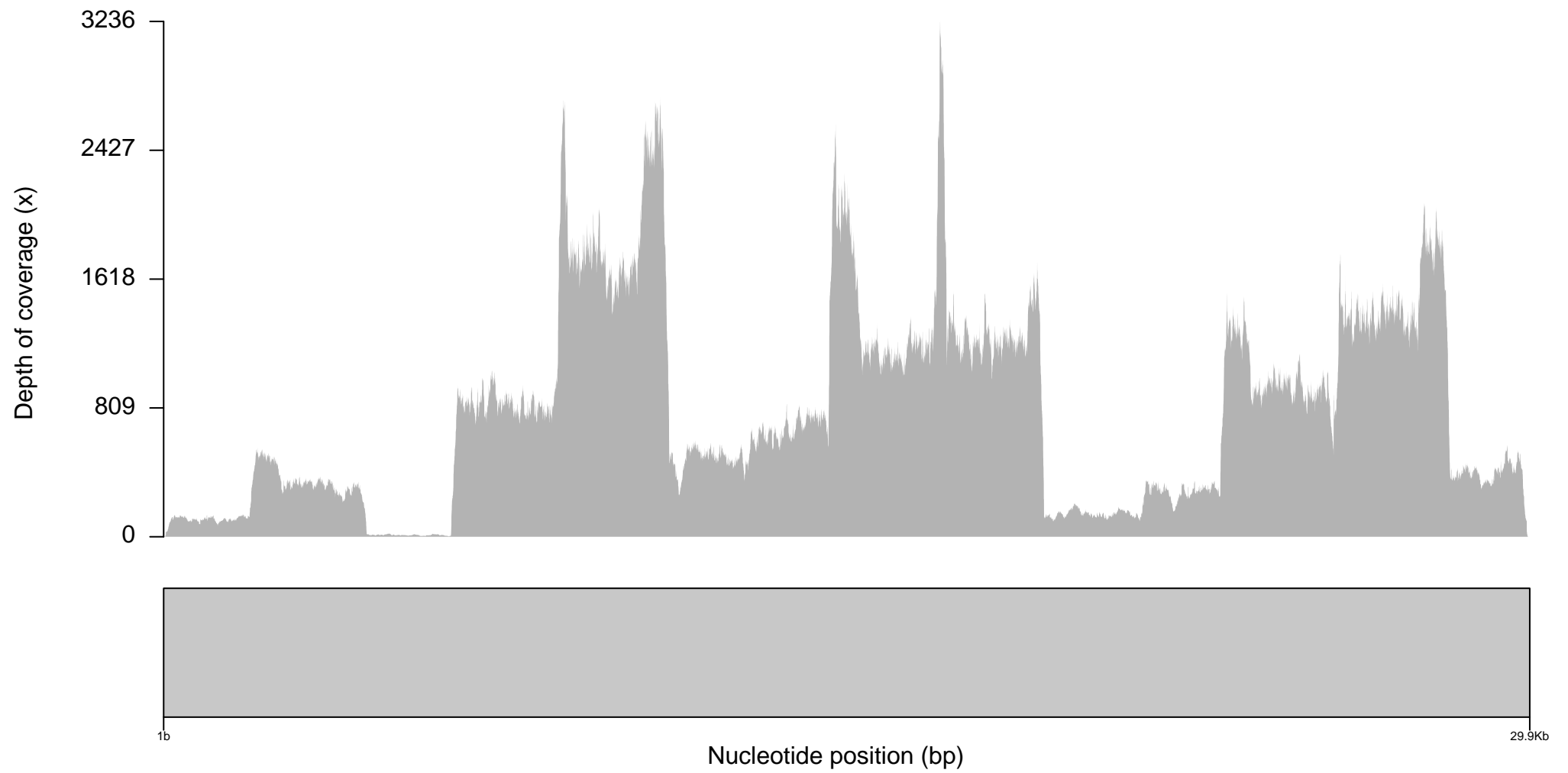

**38968**

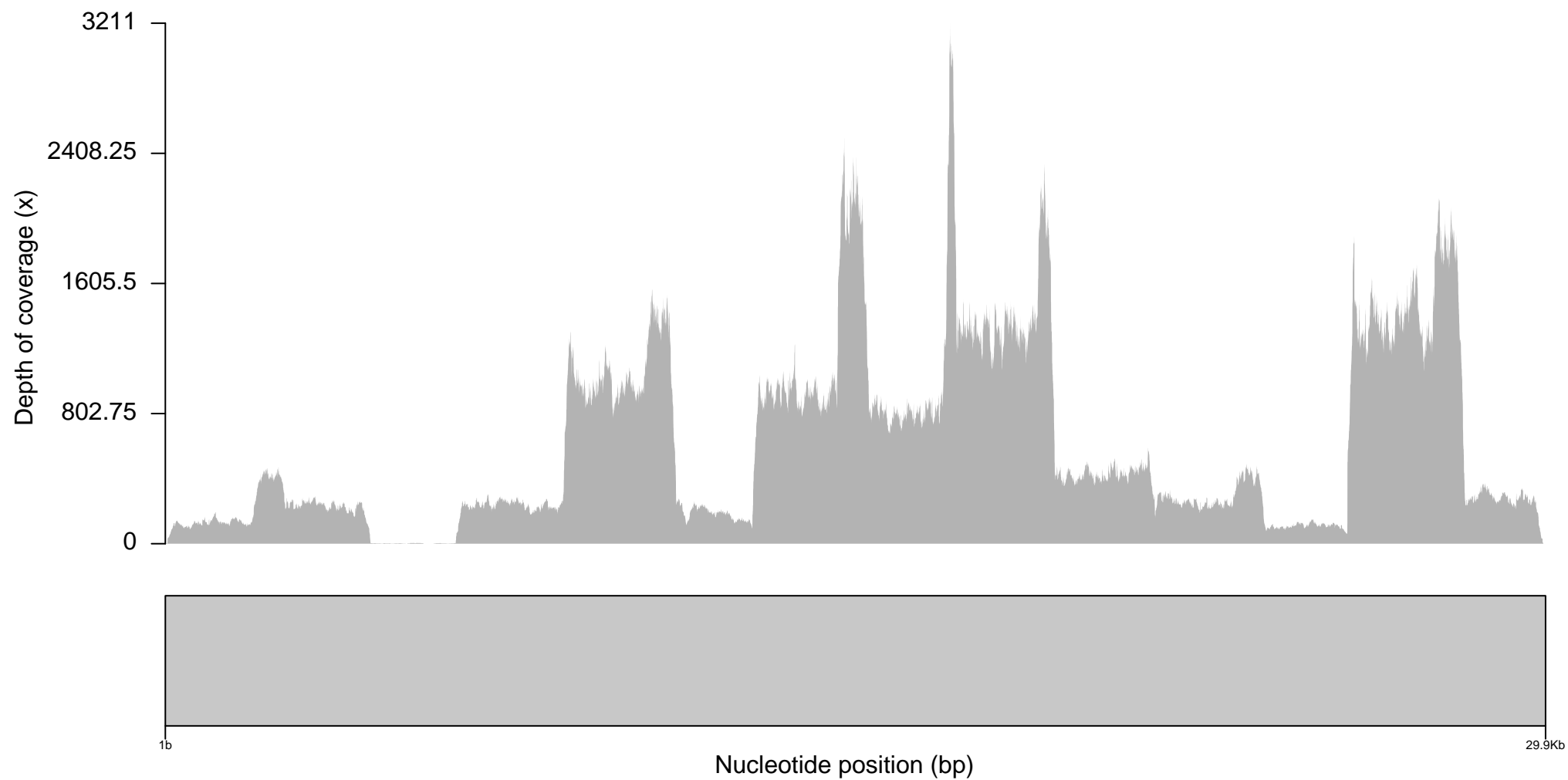

**38969**

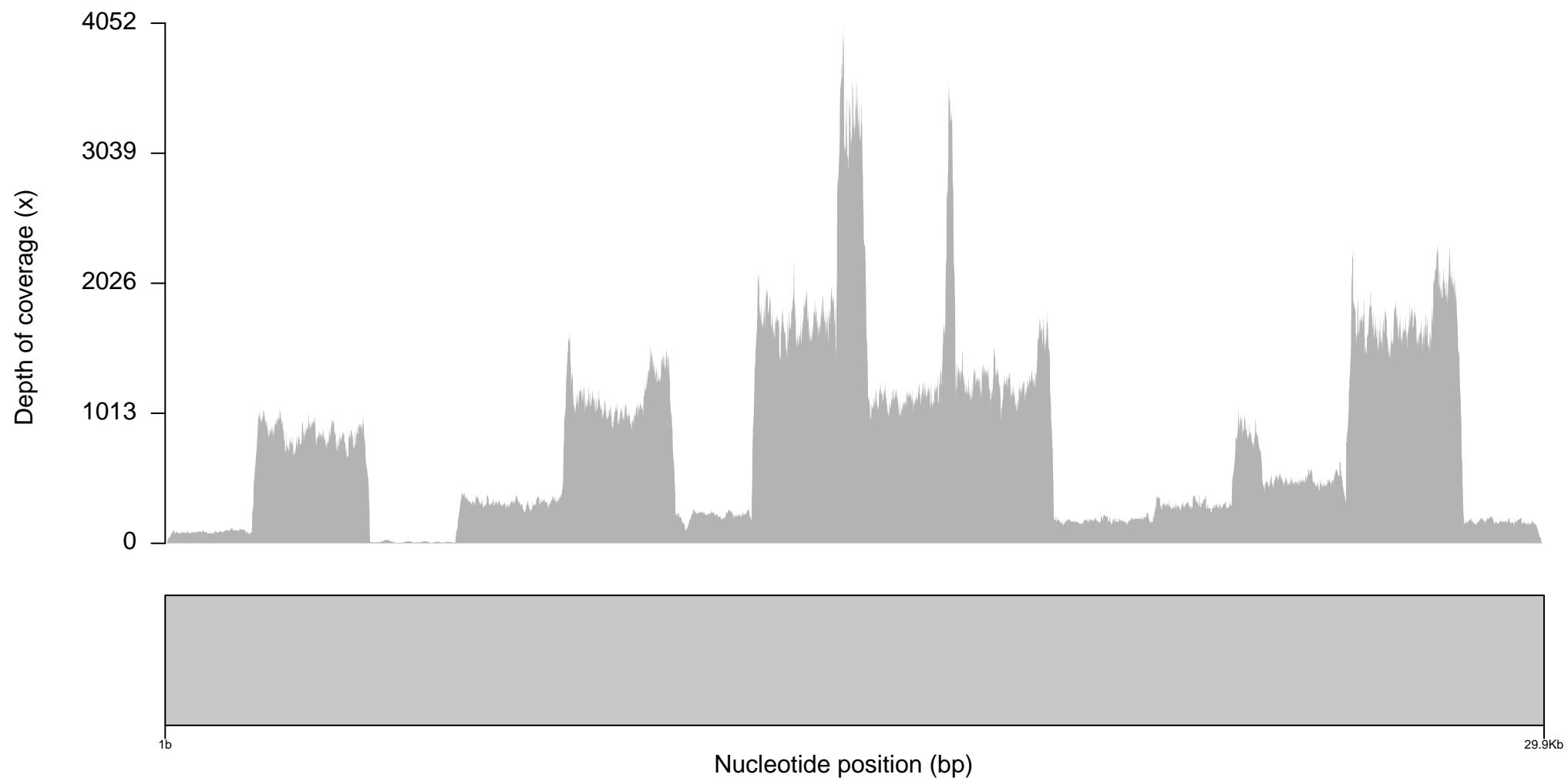

39428

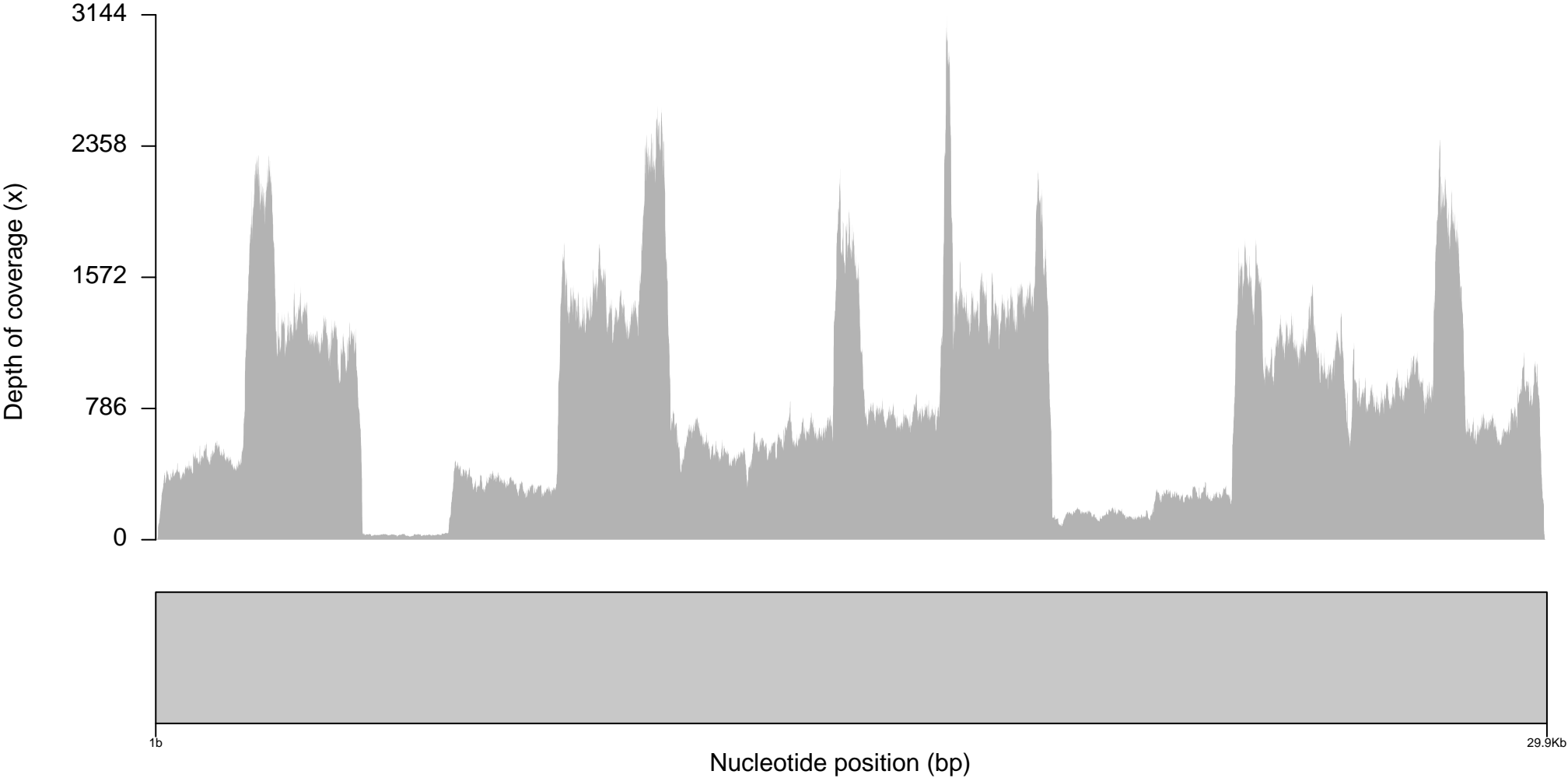

**39429**

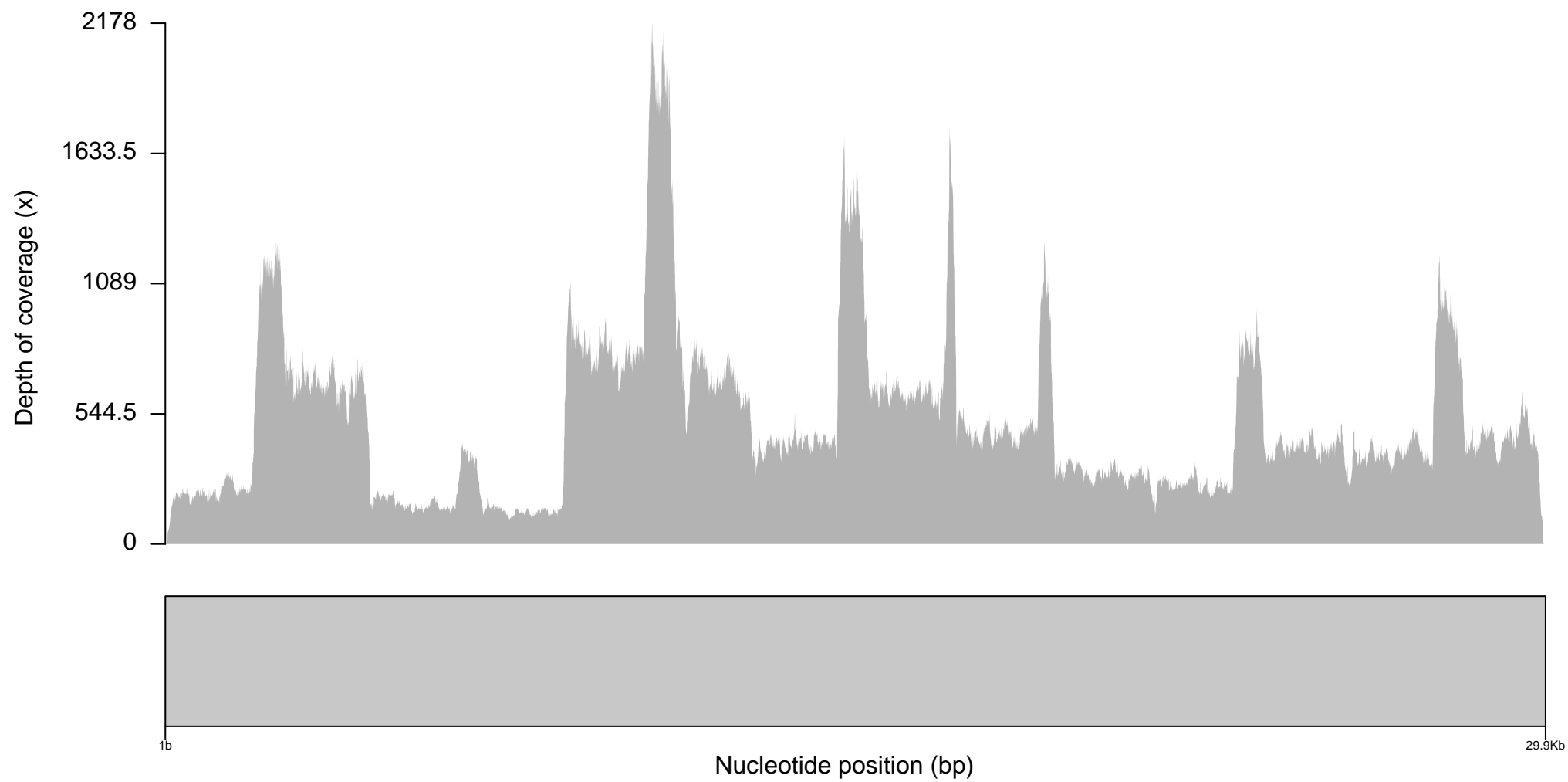

**39430**

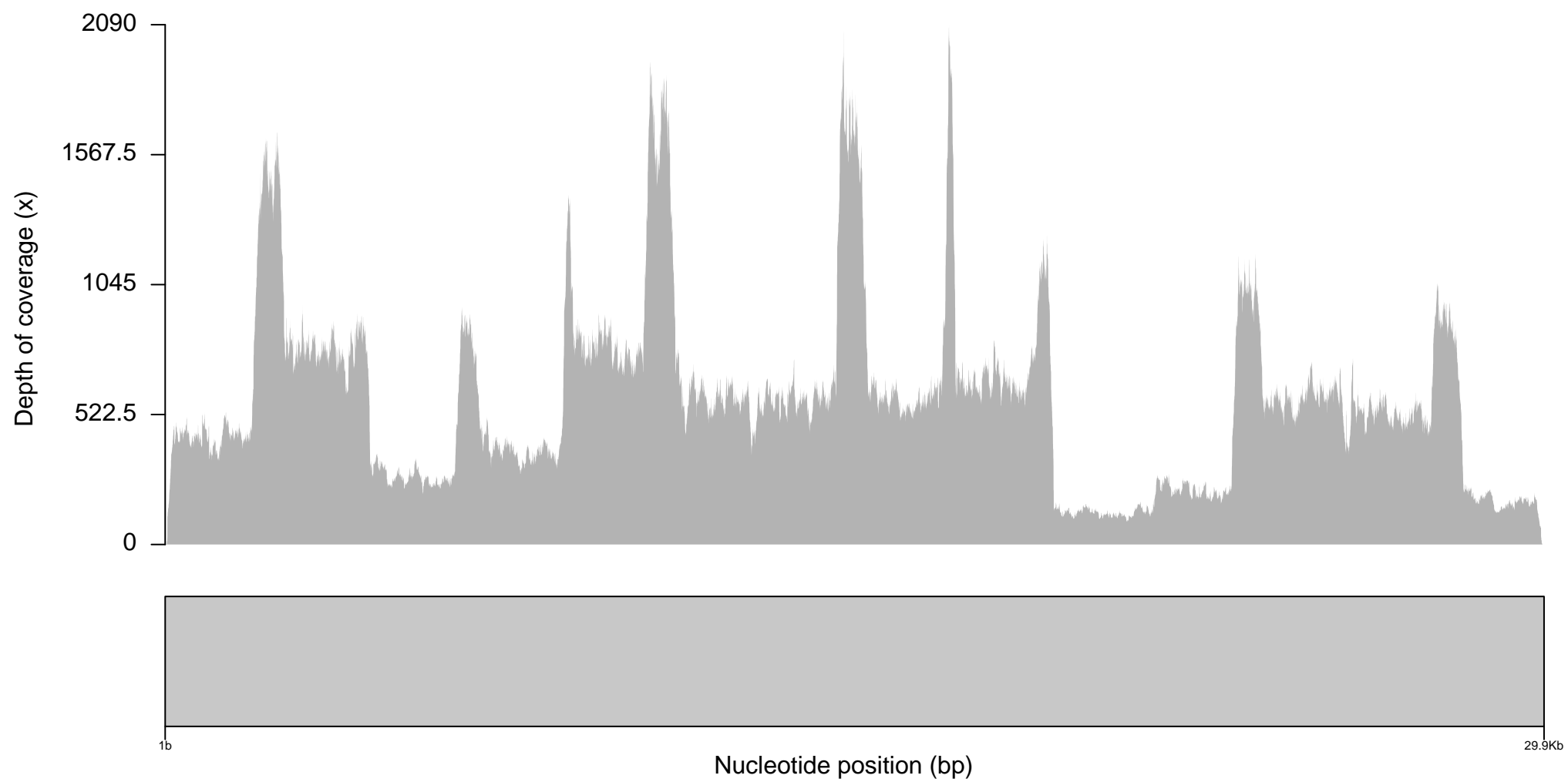

**39431**

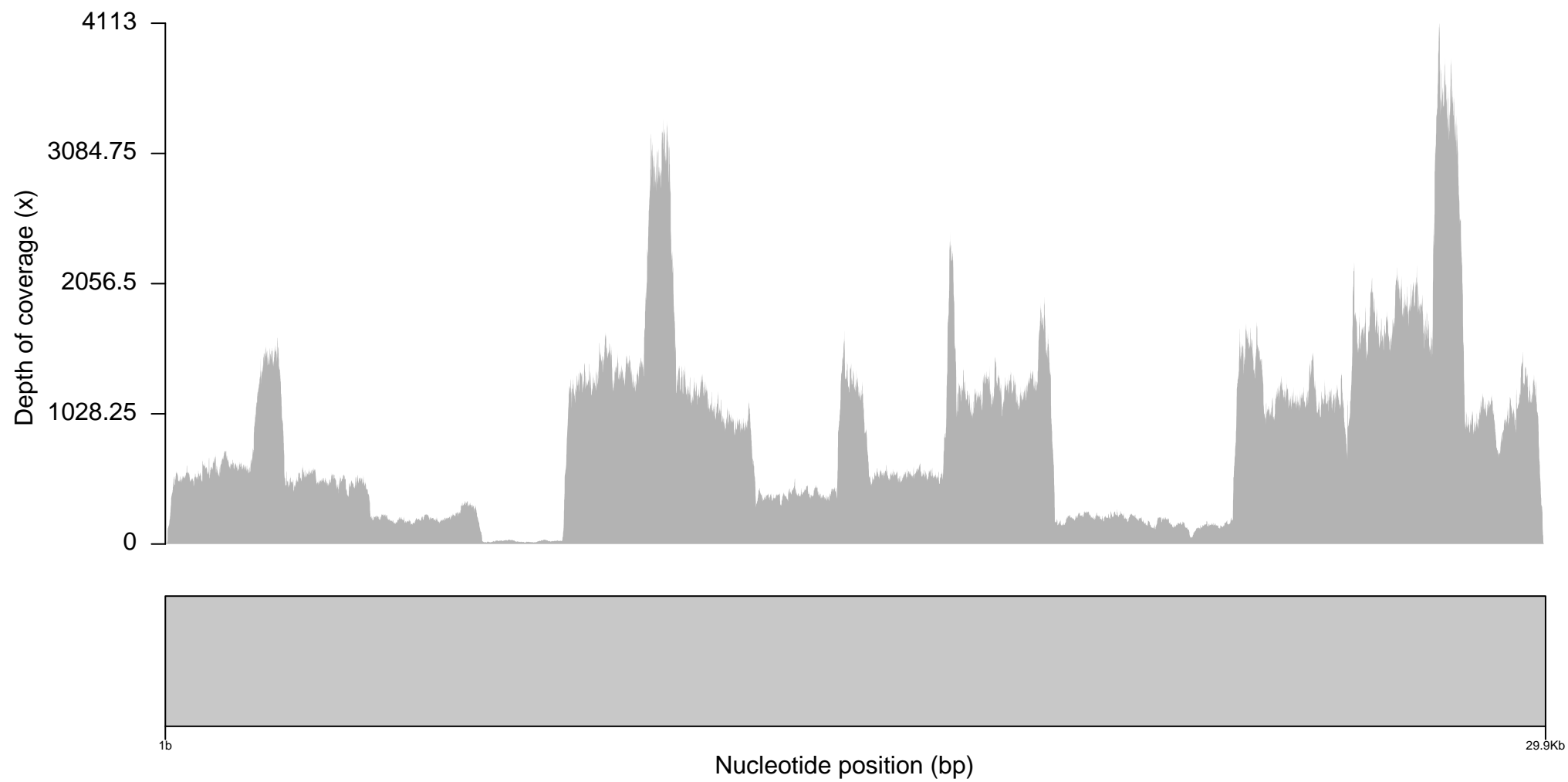

**39432**

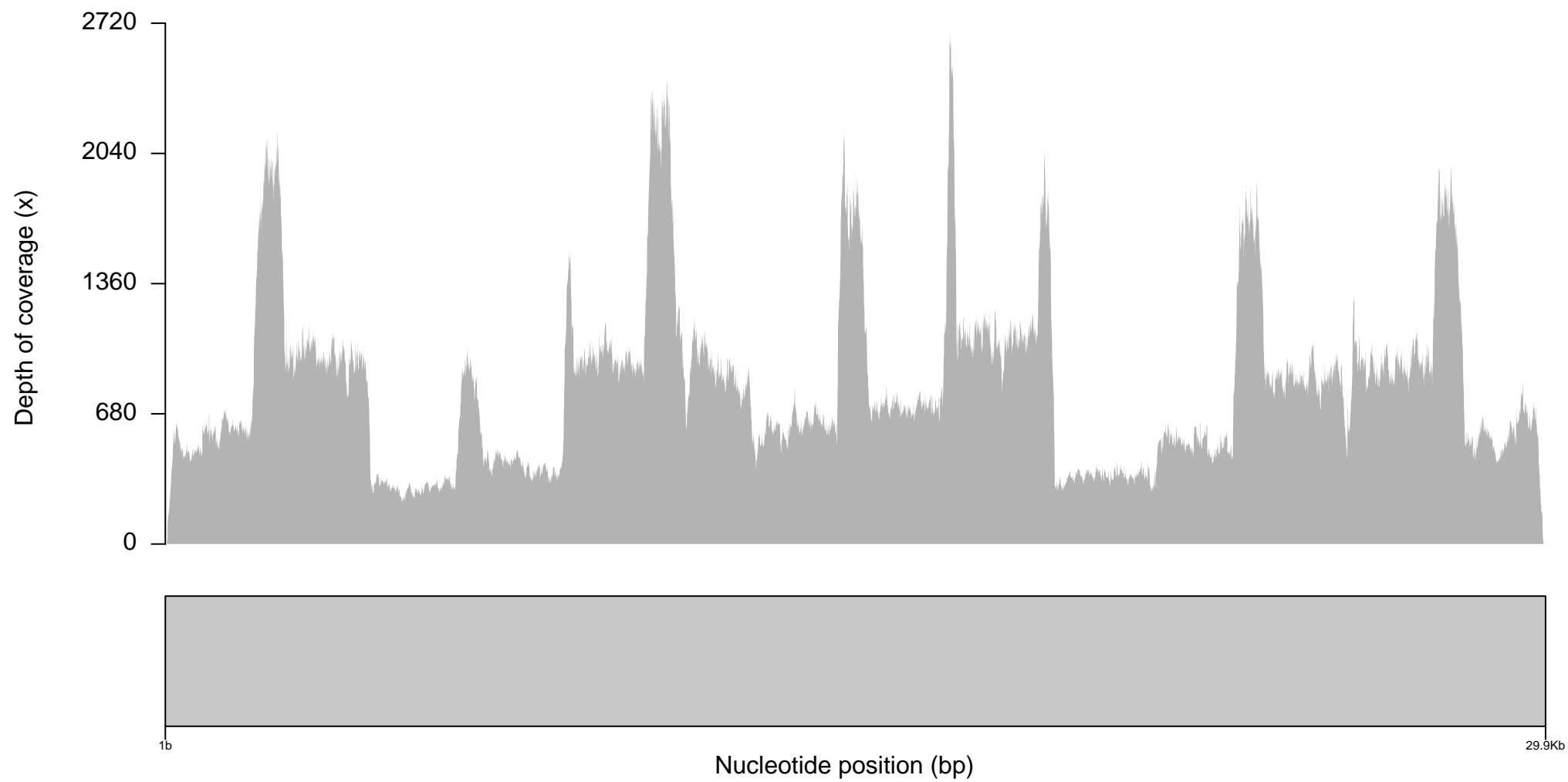

**39433**

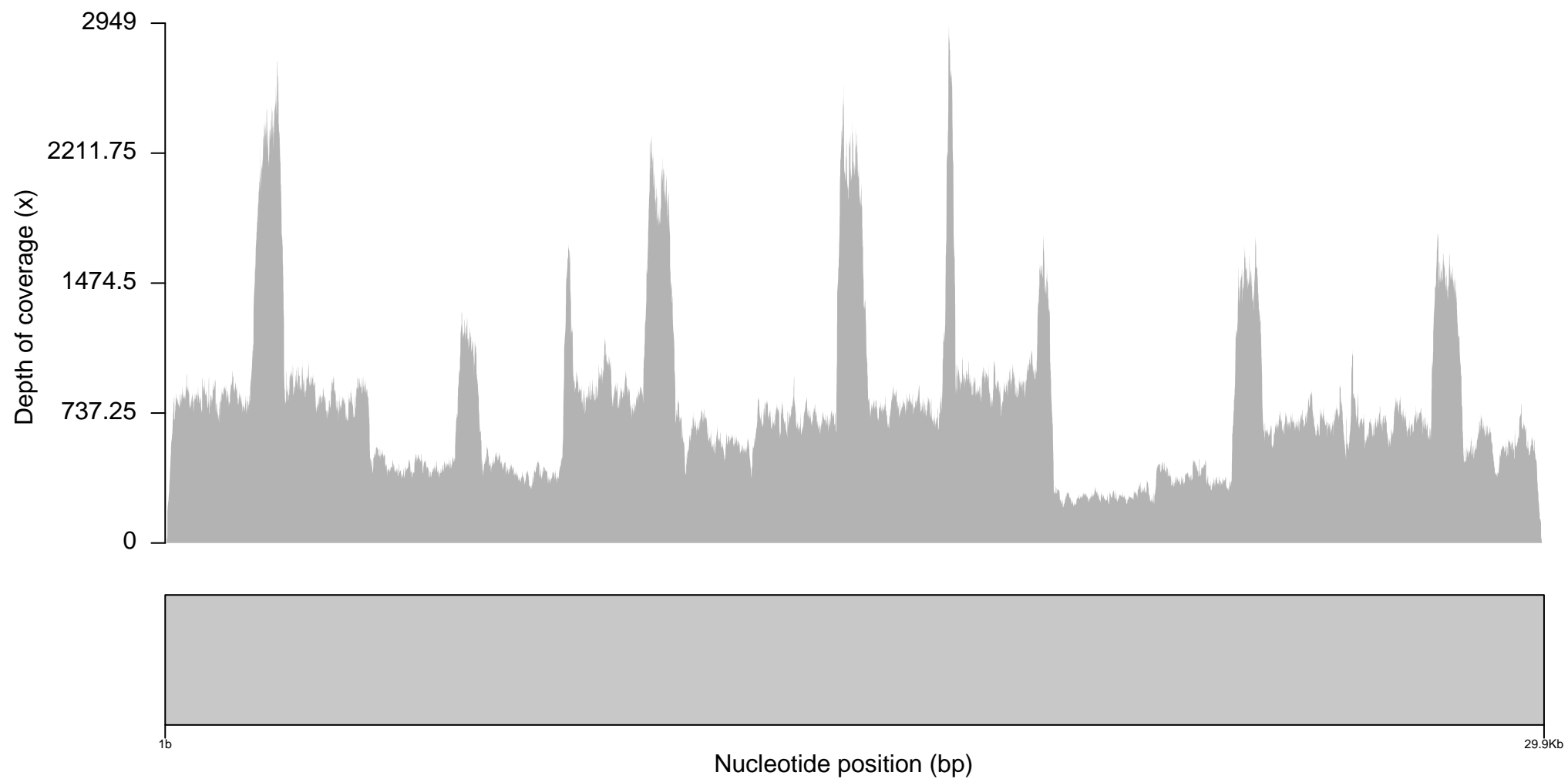

**39434**

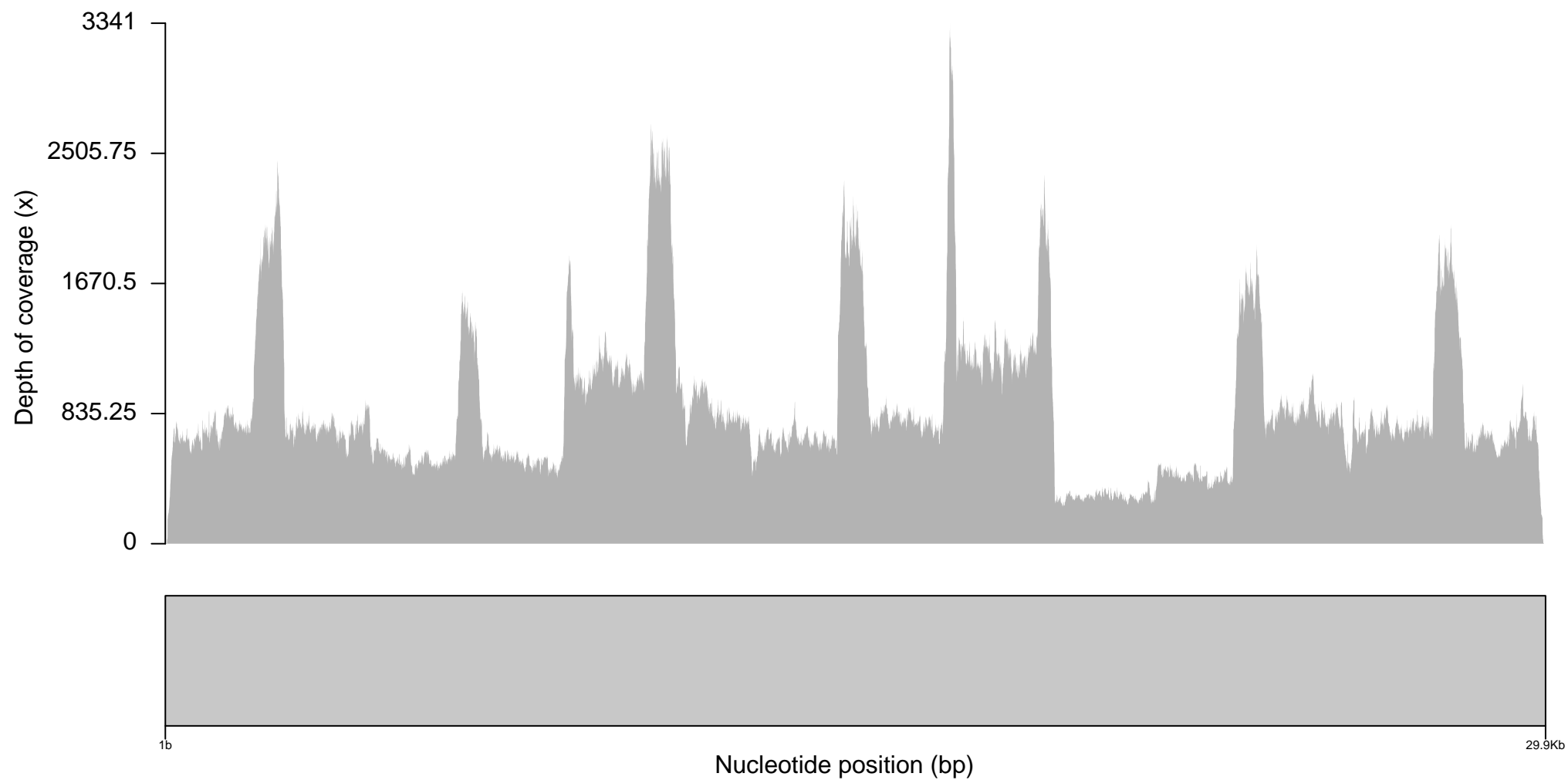

**39435**

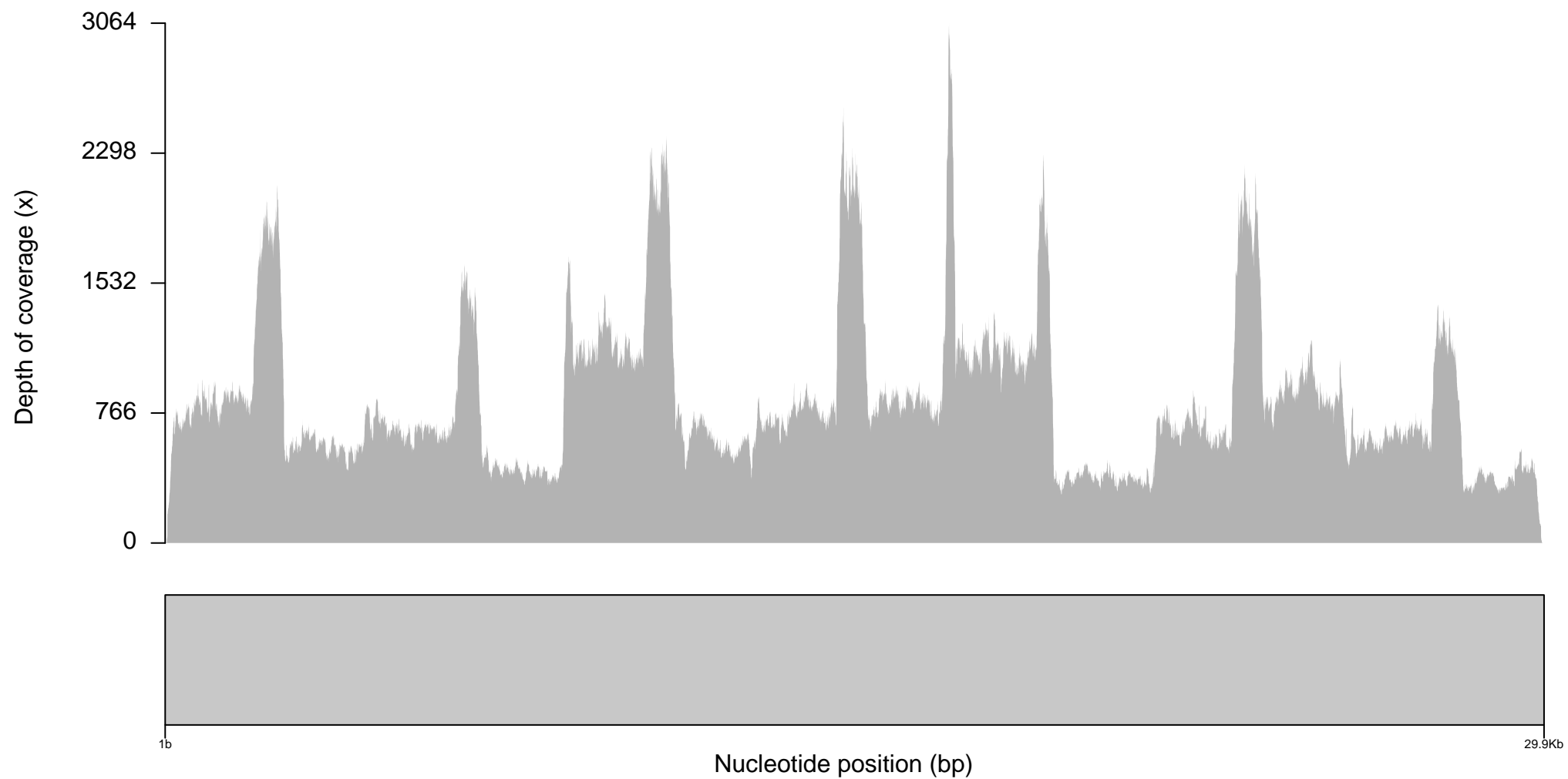

**39436**

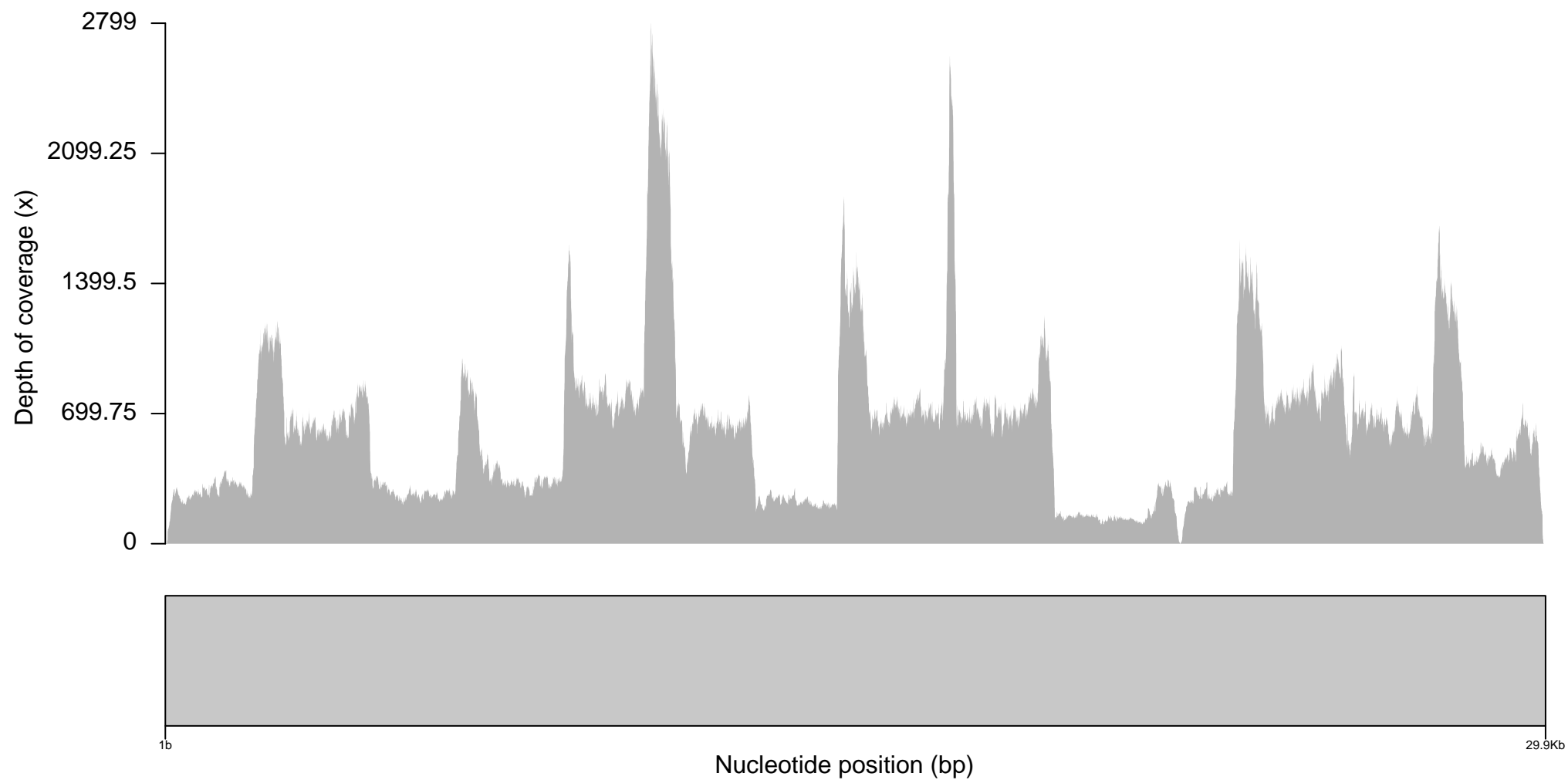

**39437**

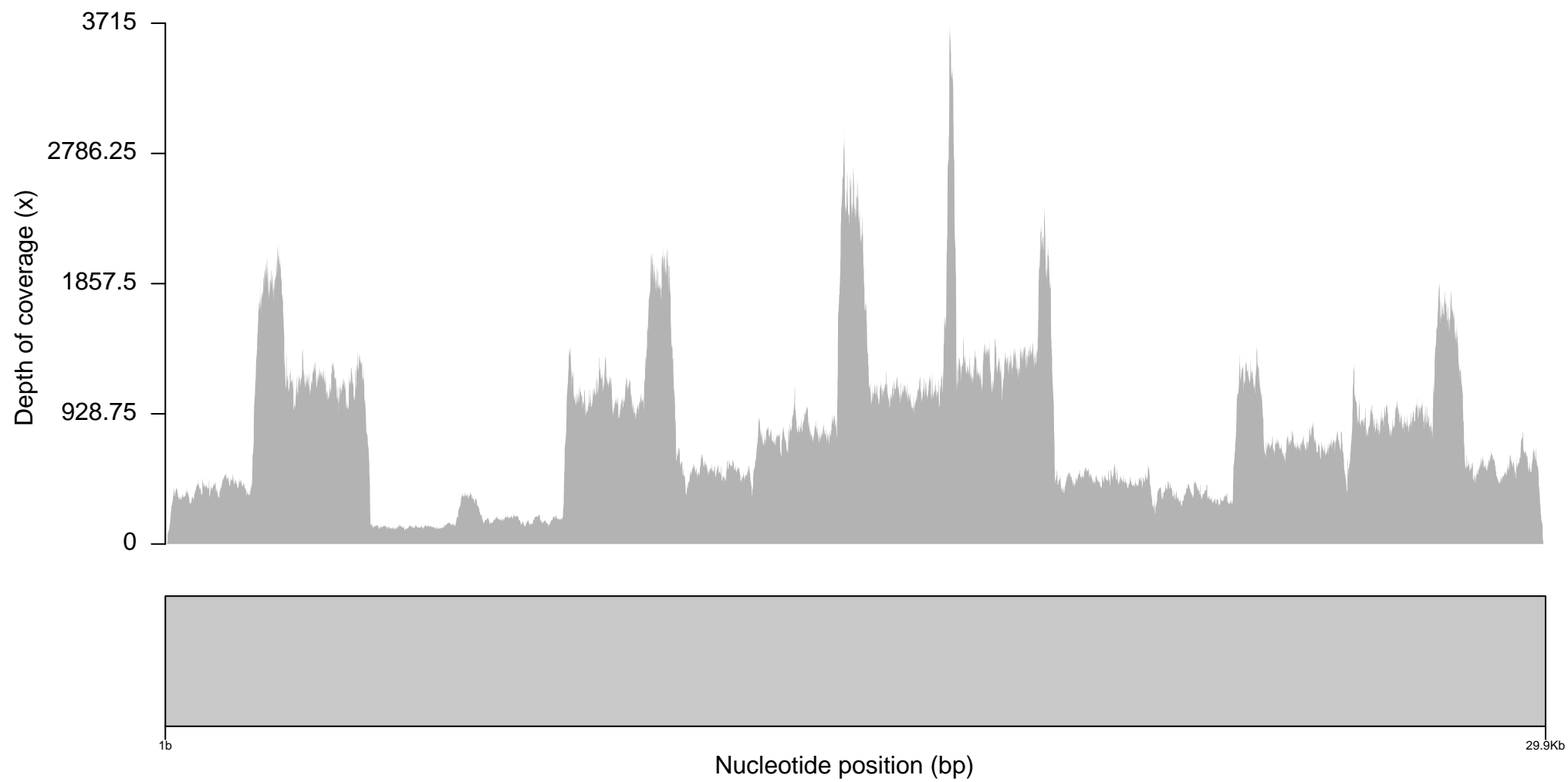

**39438**

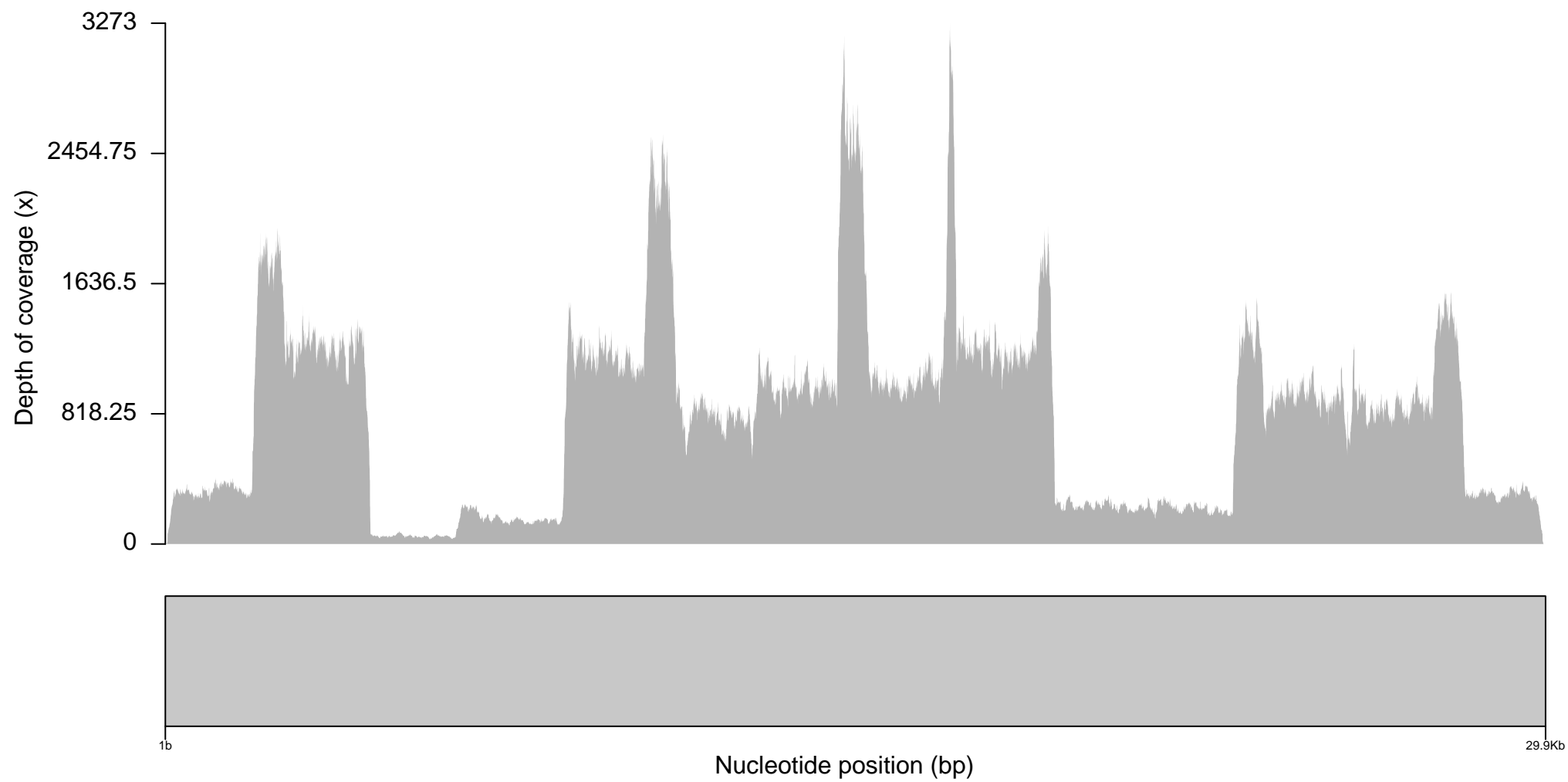

**39439**

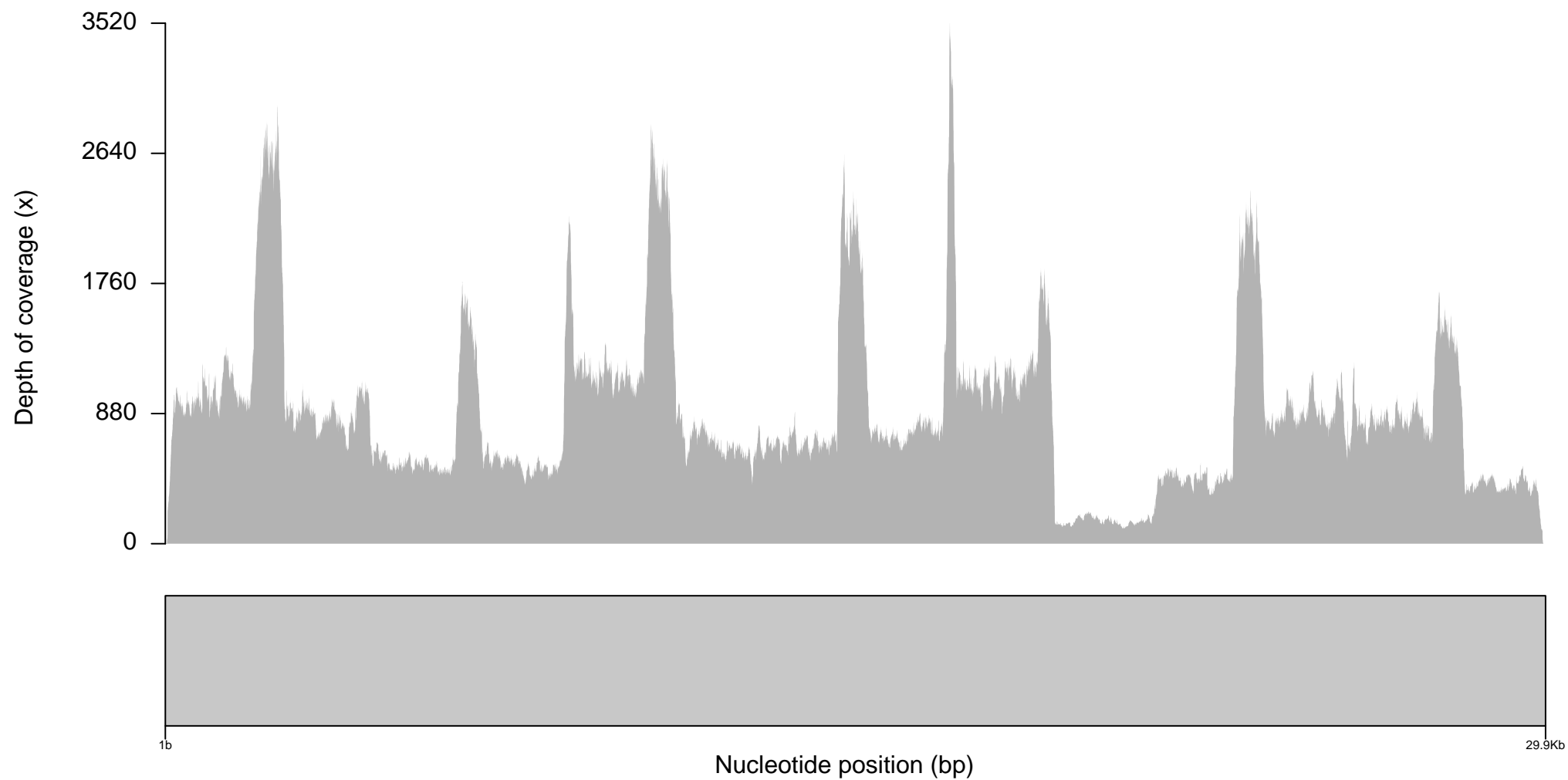

**39440**

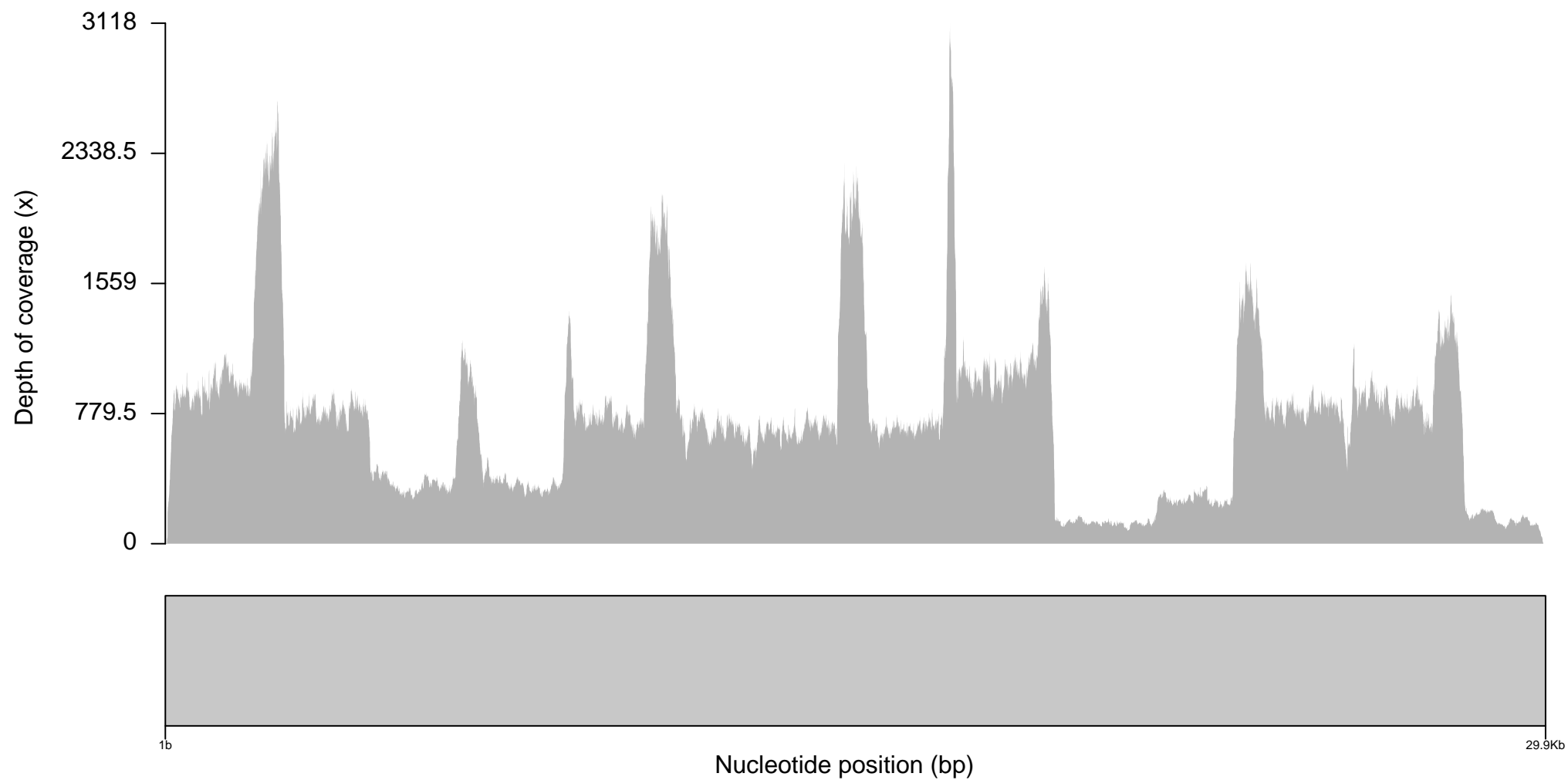

**39441**

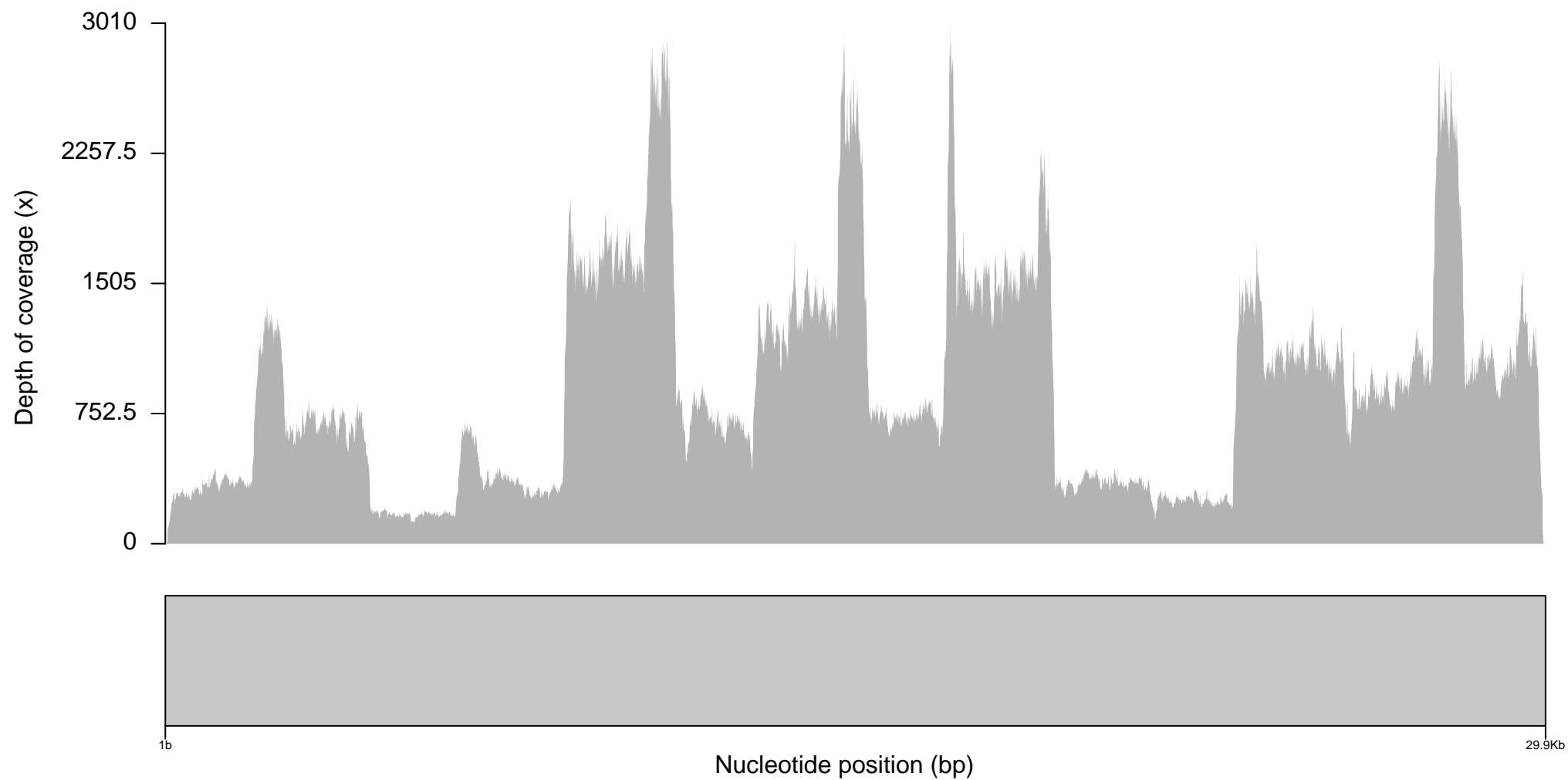

**39442**

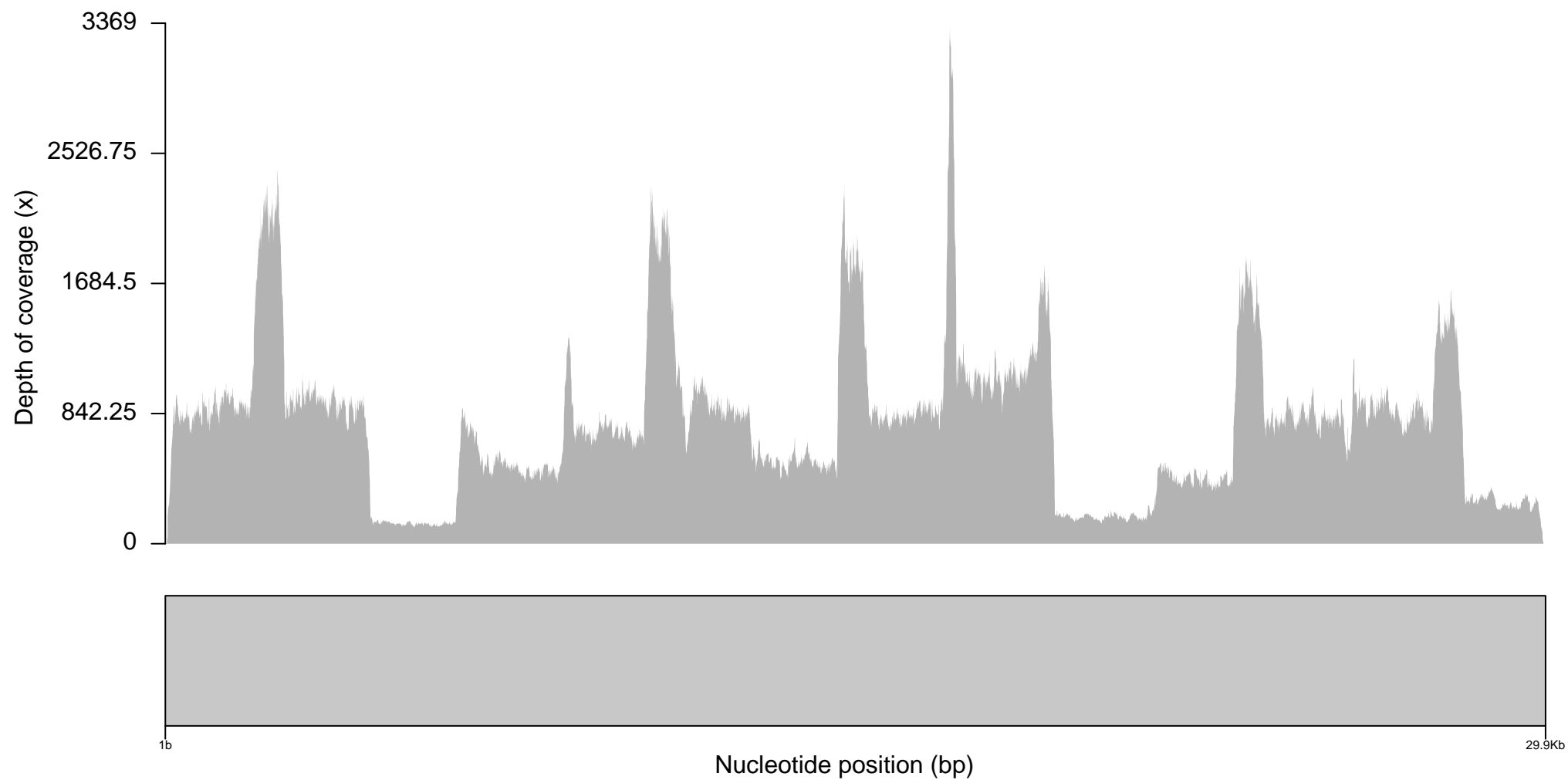

**39443**

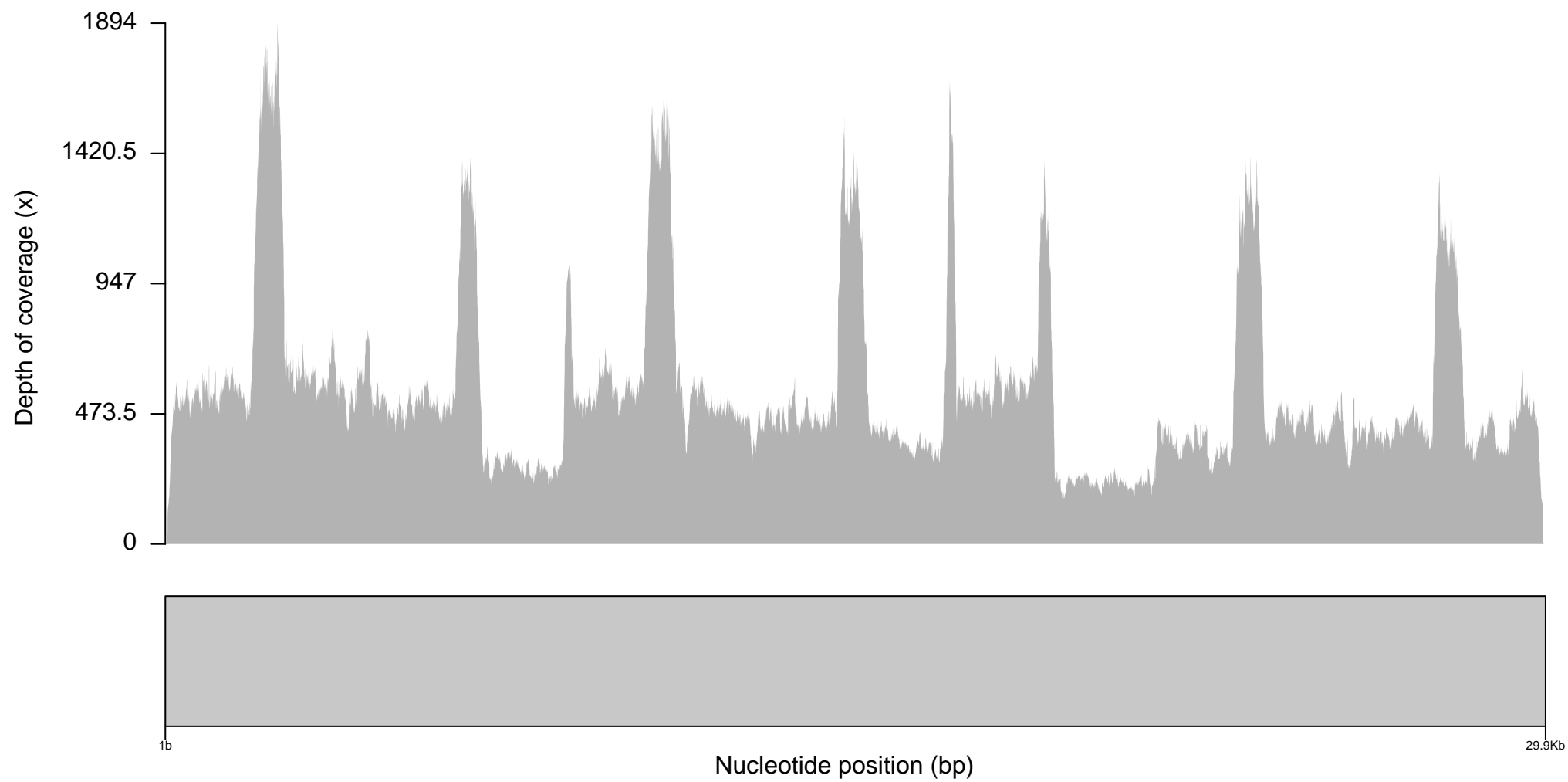

**39444**

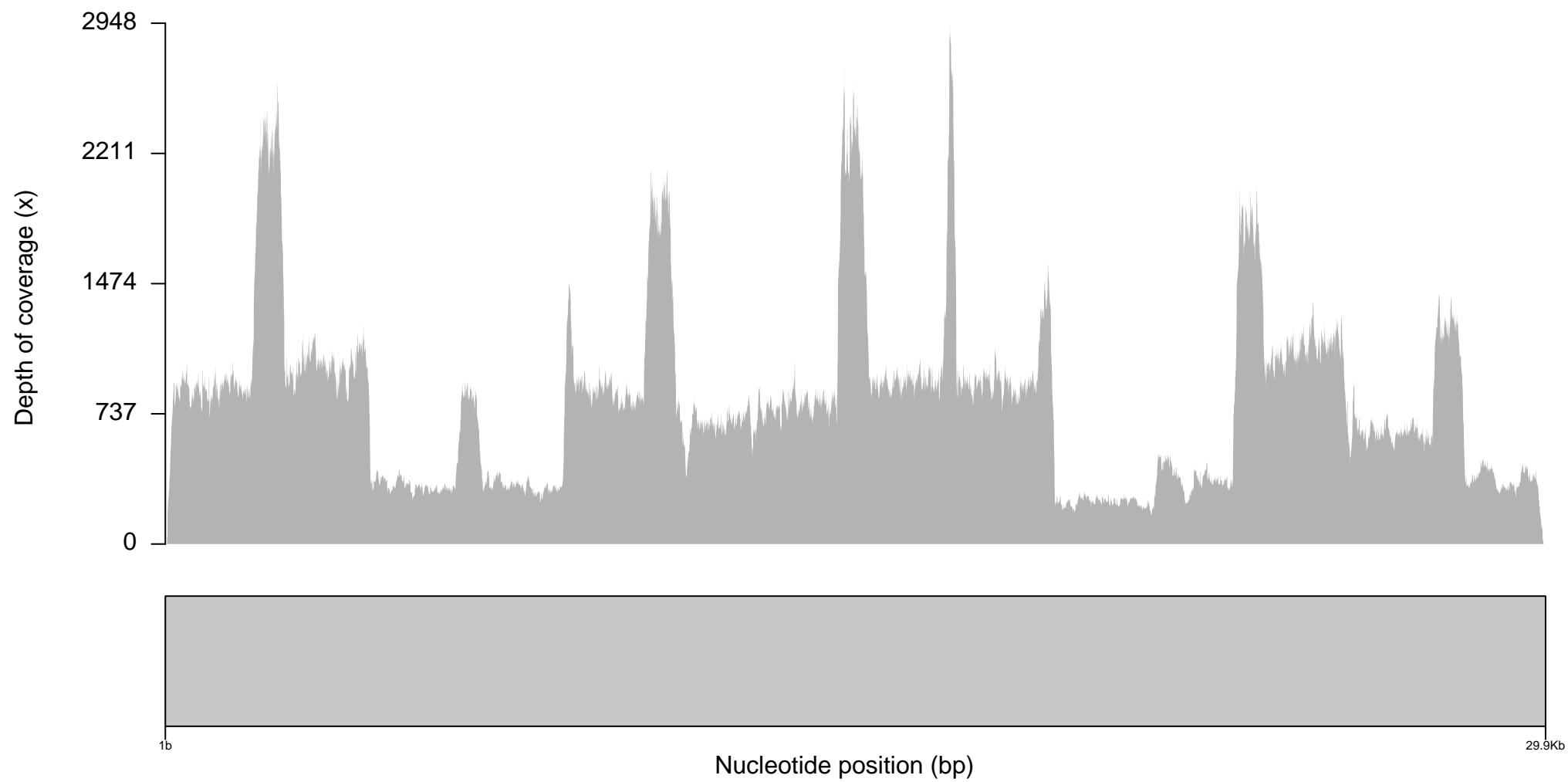

**39445**

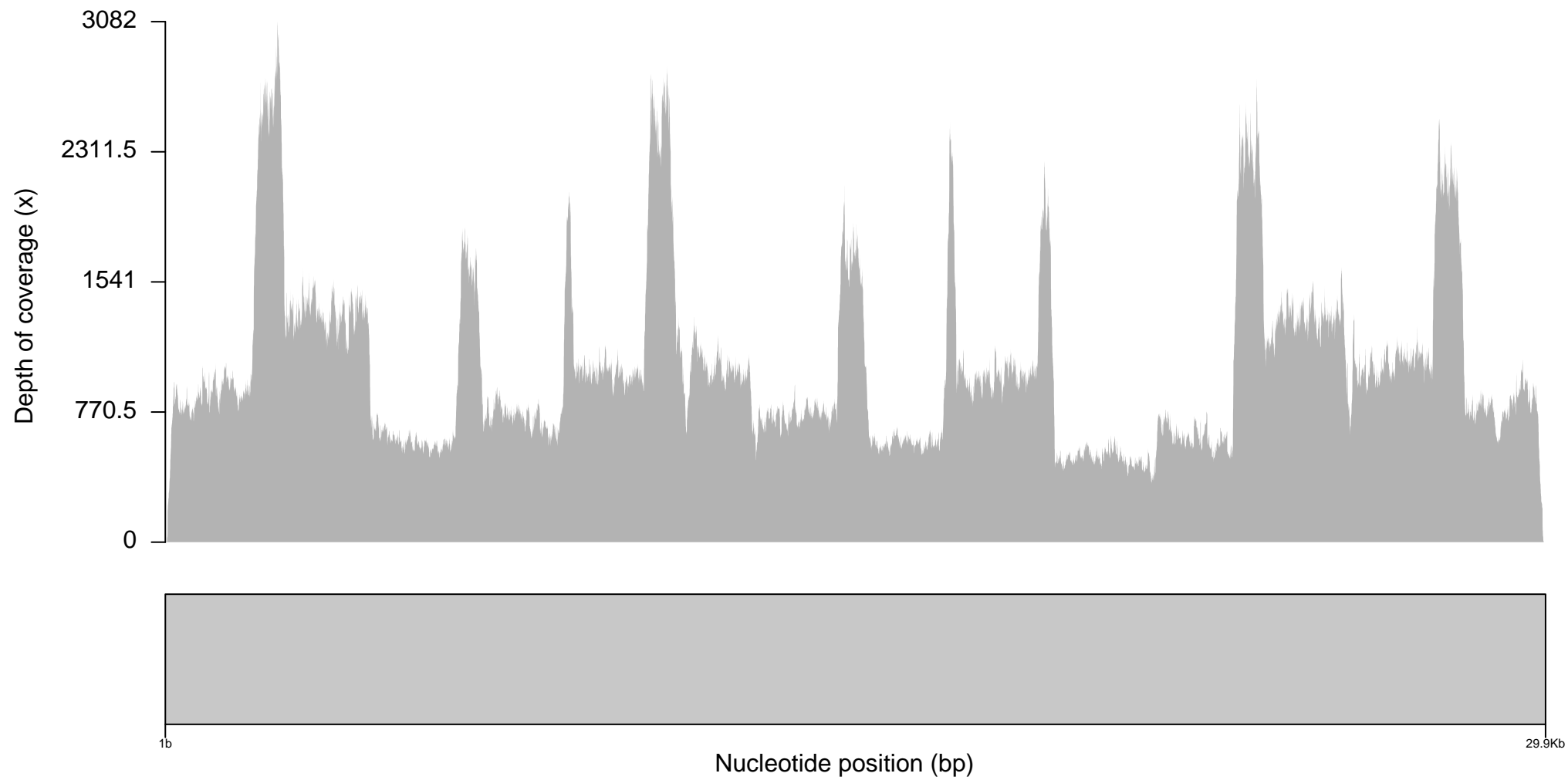

**39446**

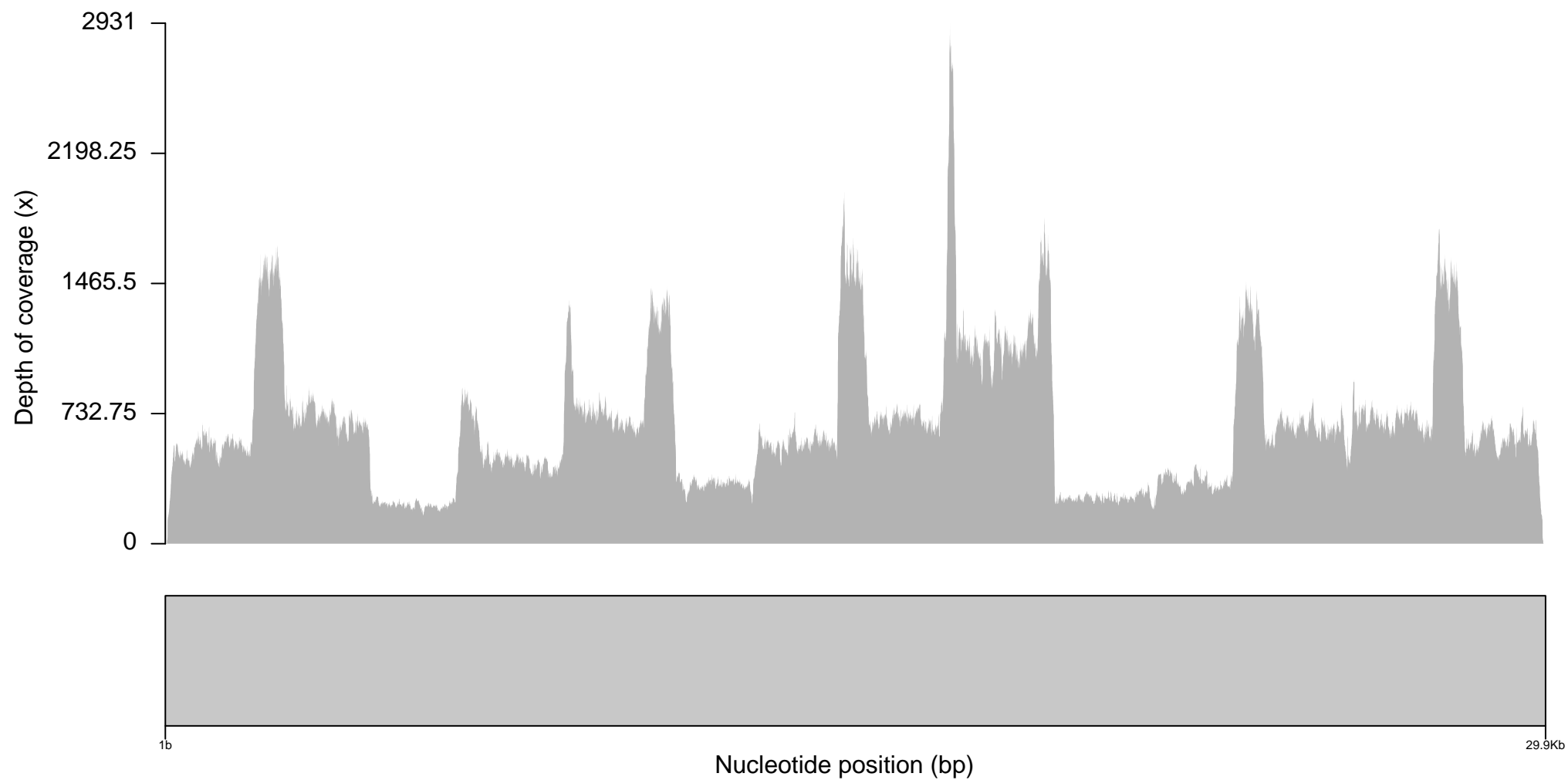

**39447**

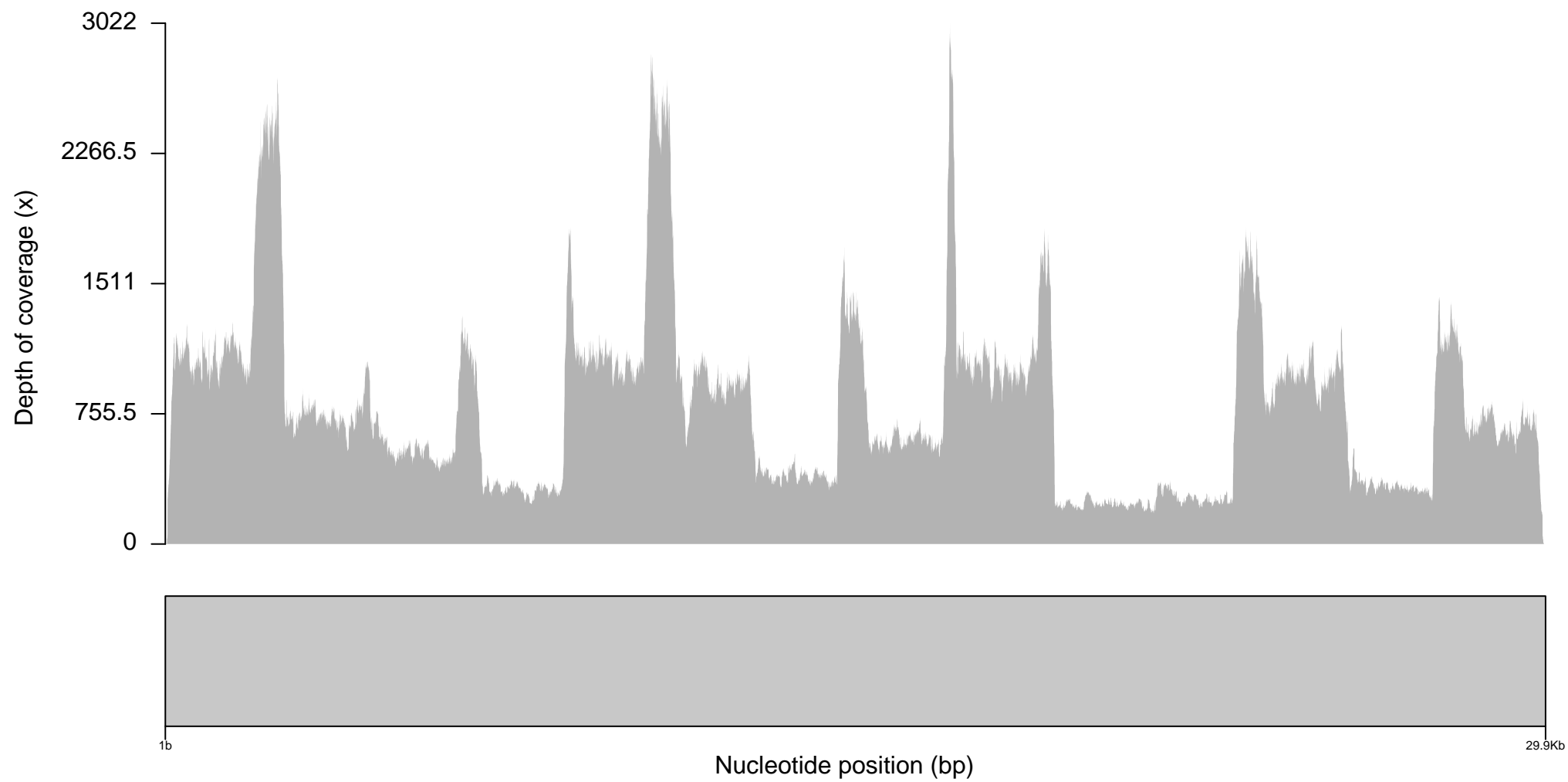

**39448**

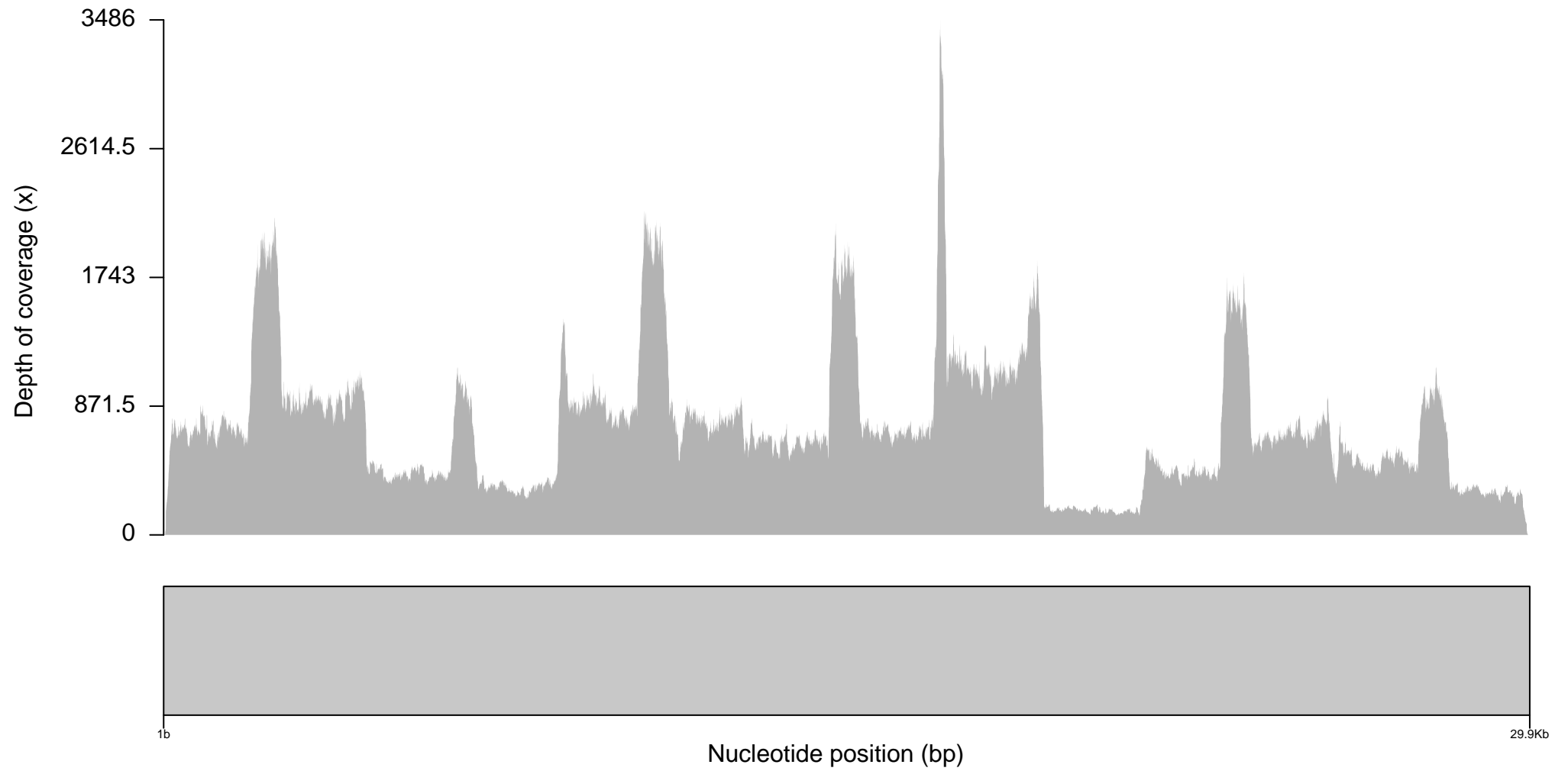

**39449**

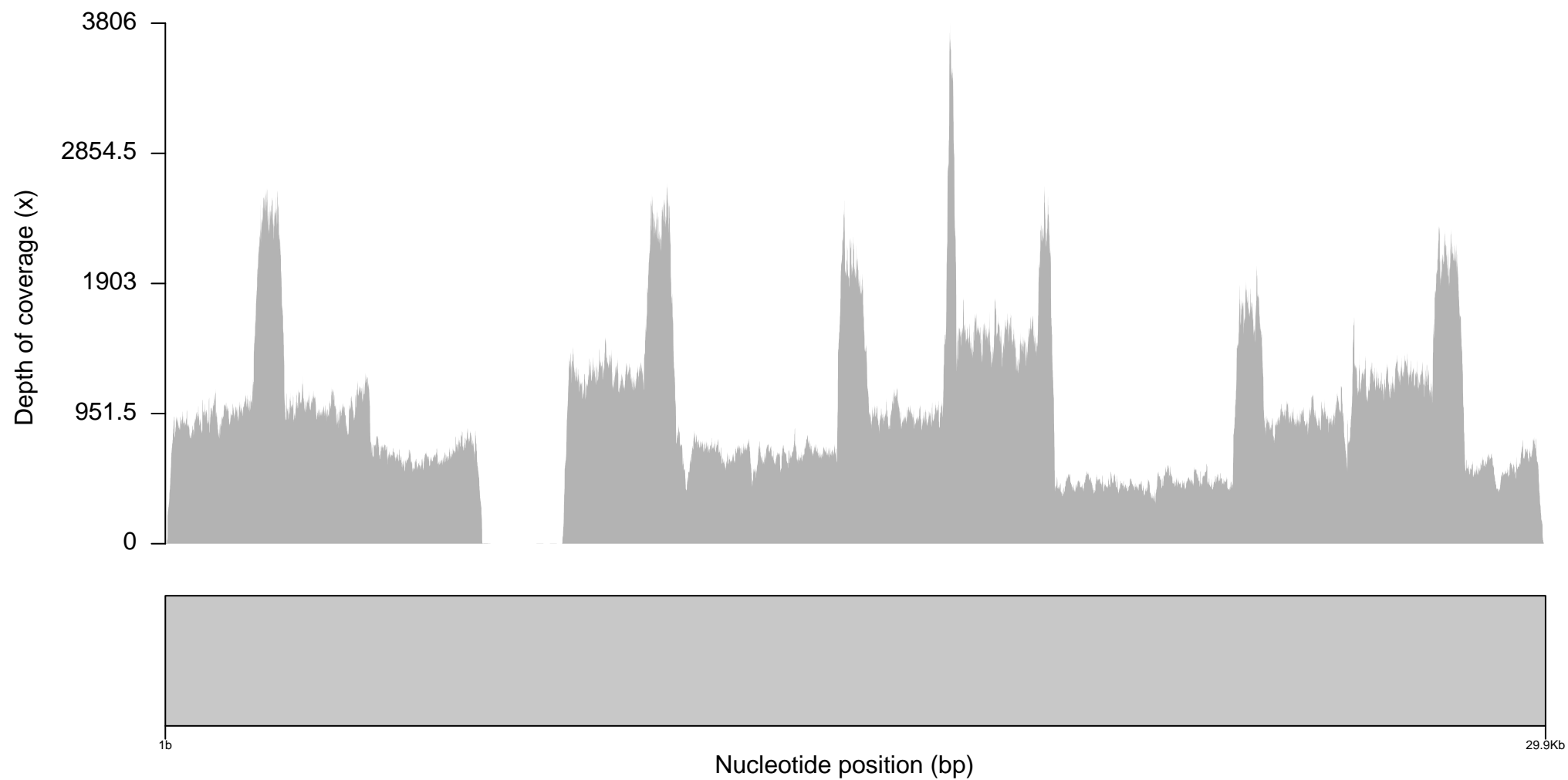

**39450**

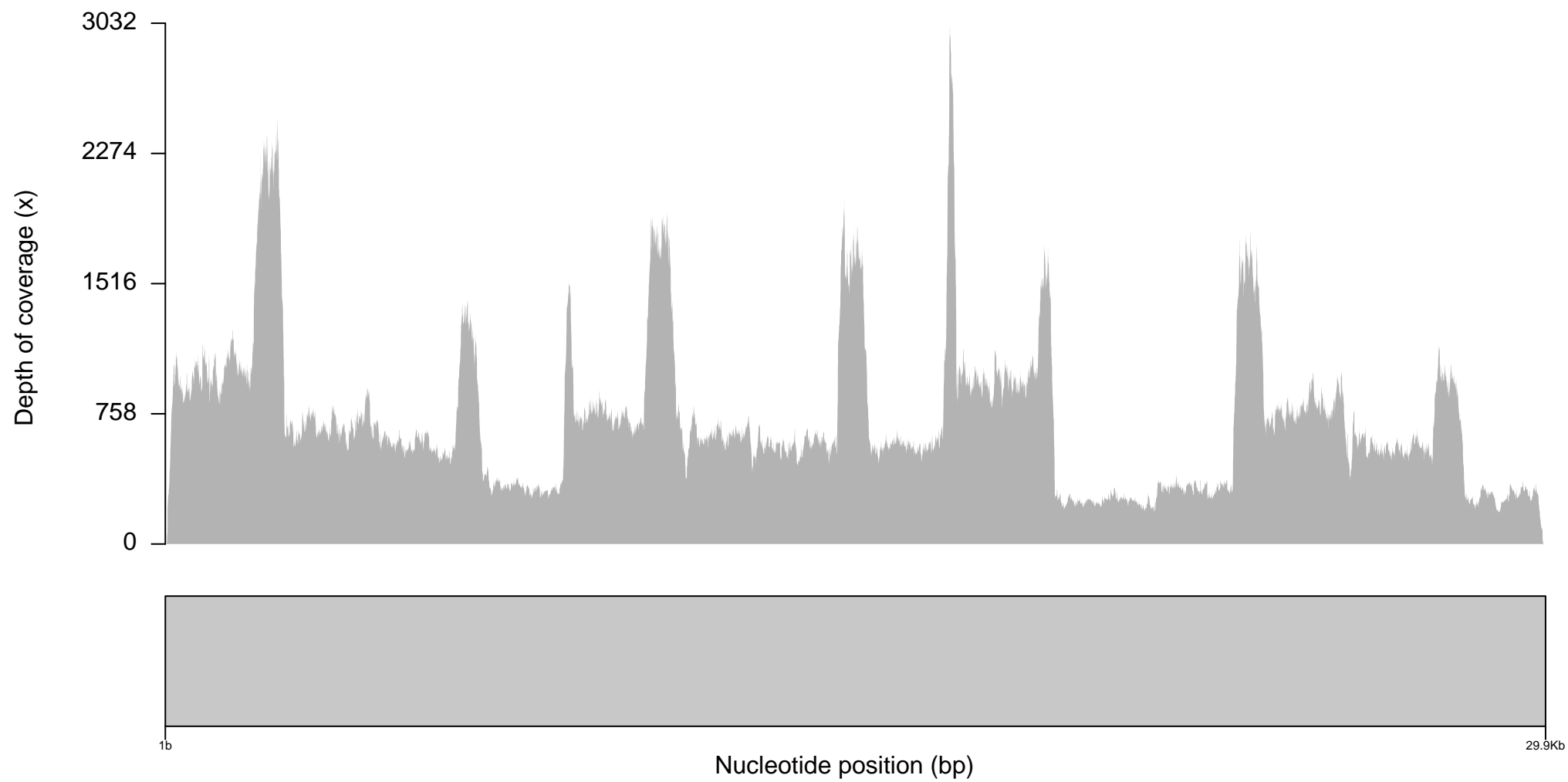

**39451**

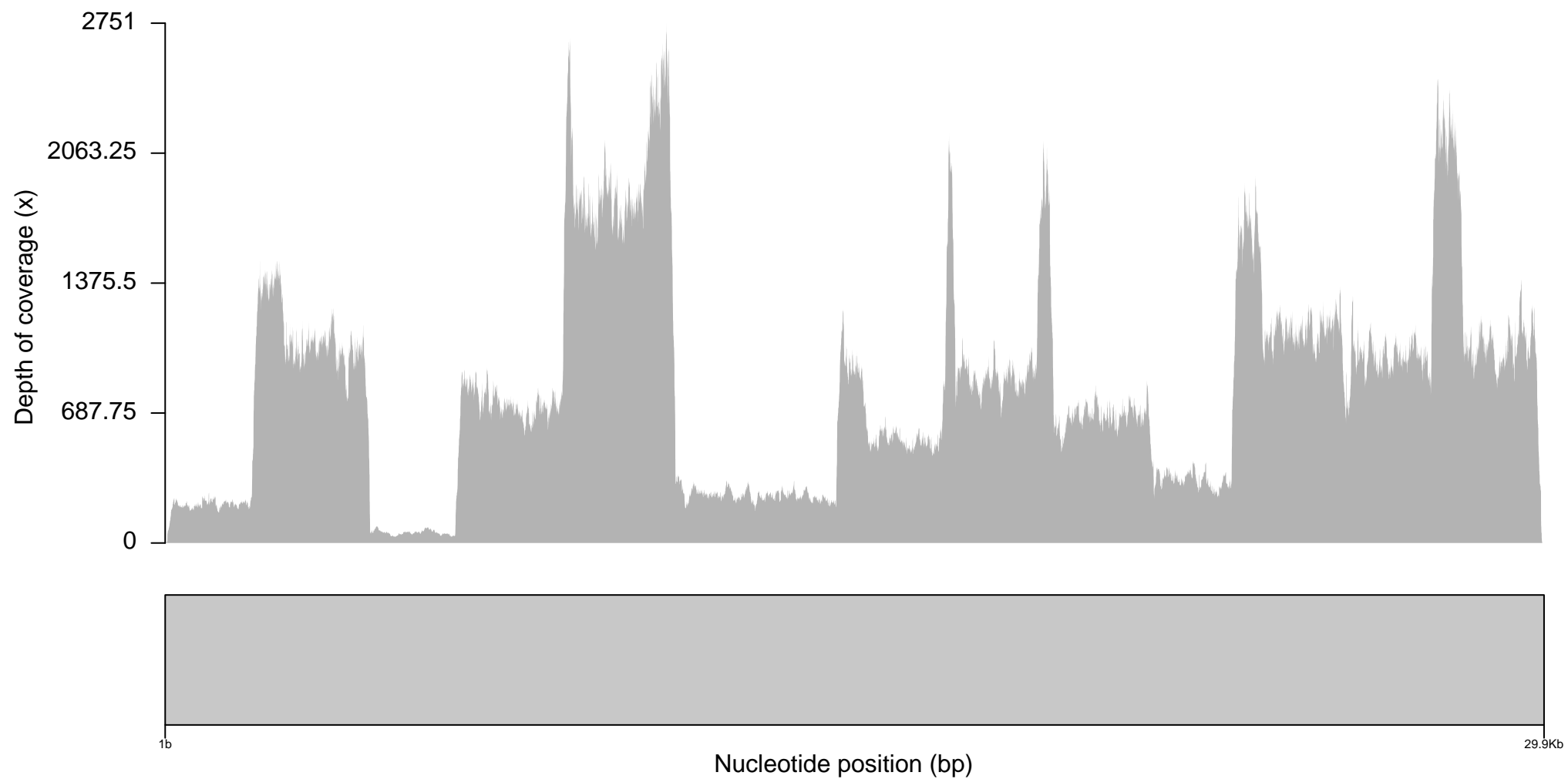

**39452**

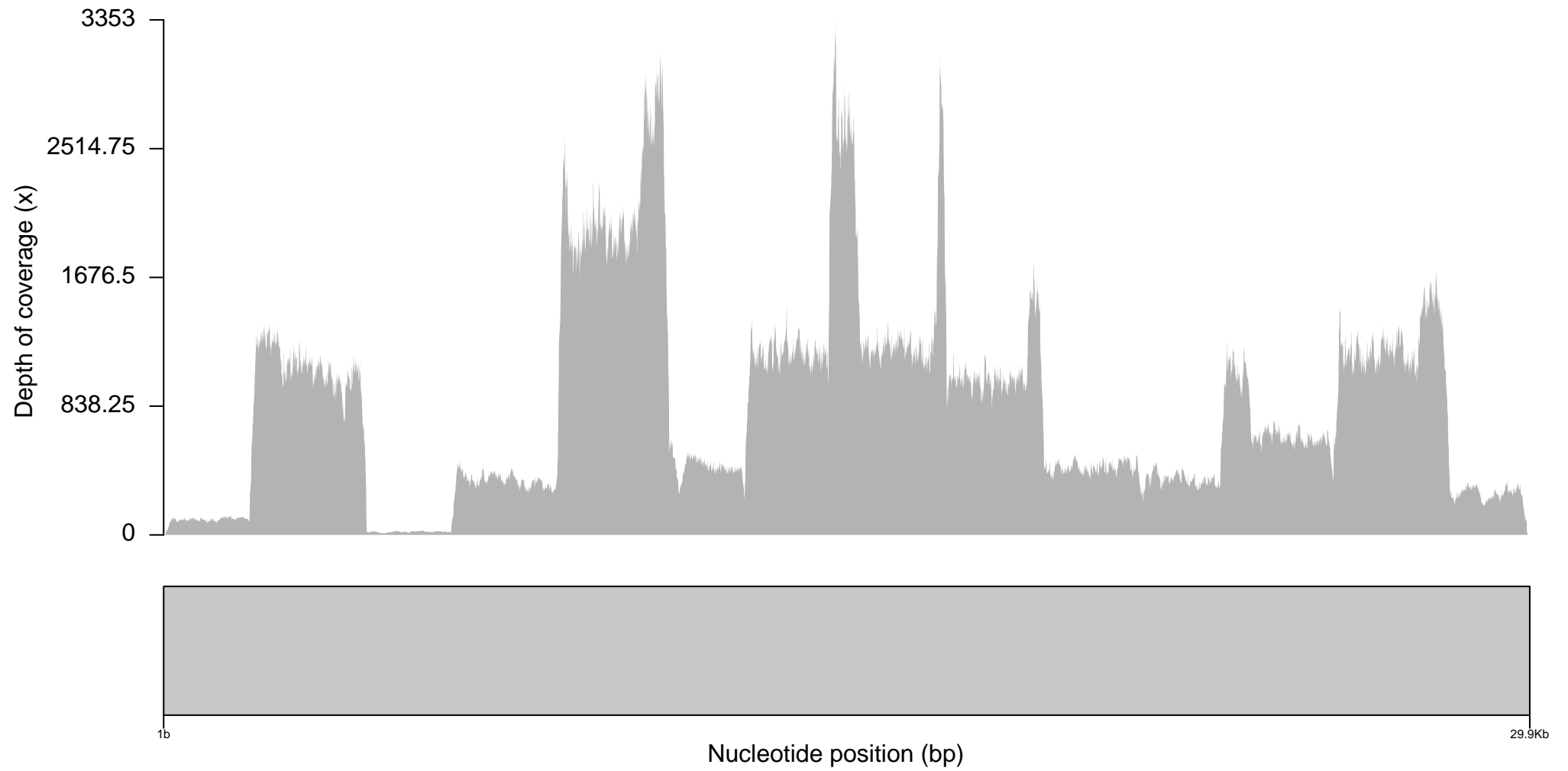

**39453**

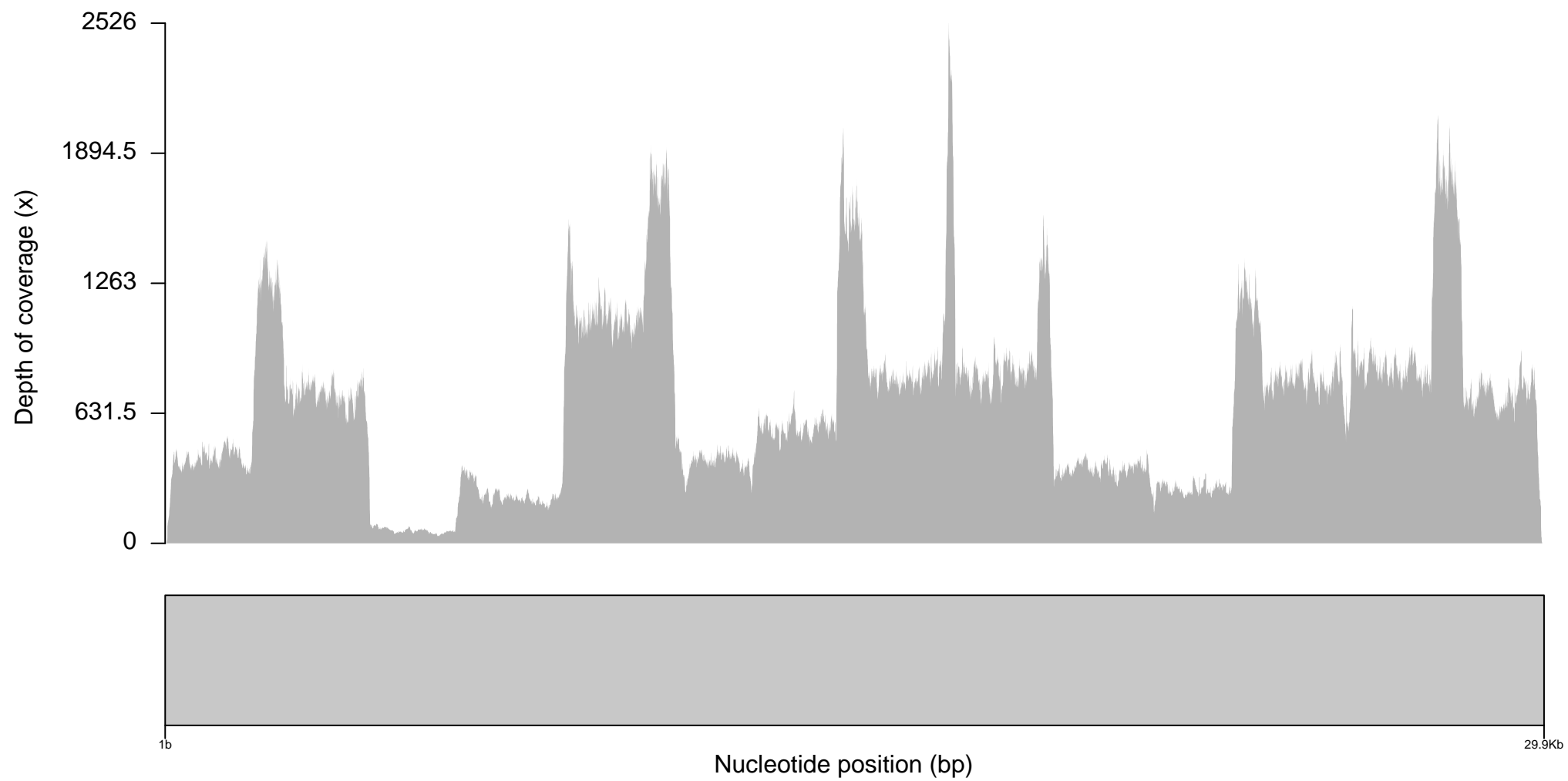

**39454**

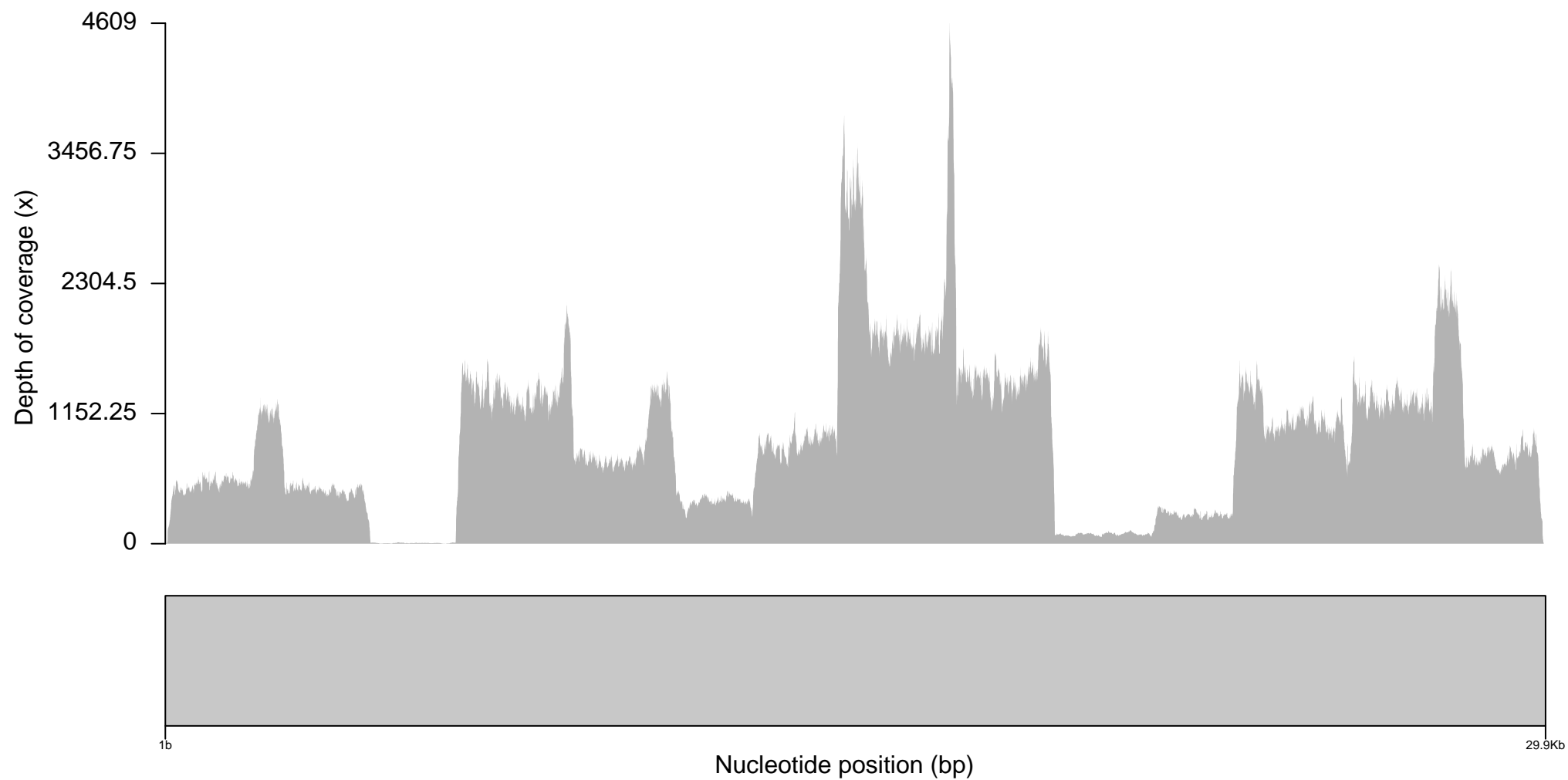

**39455**

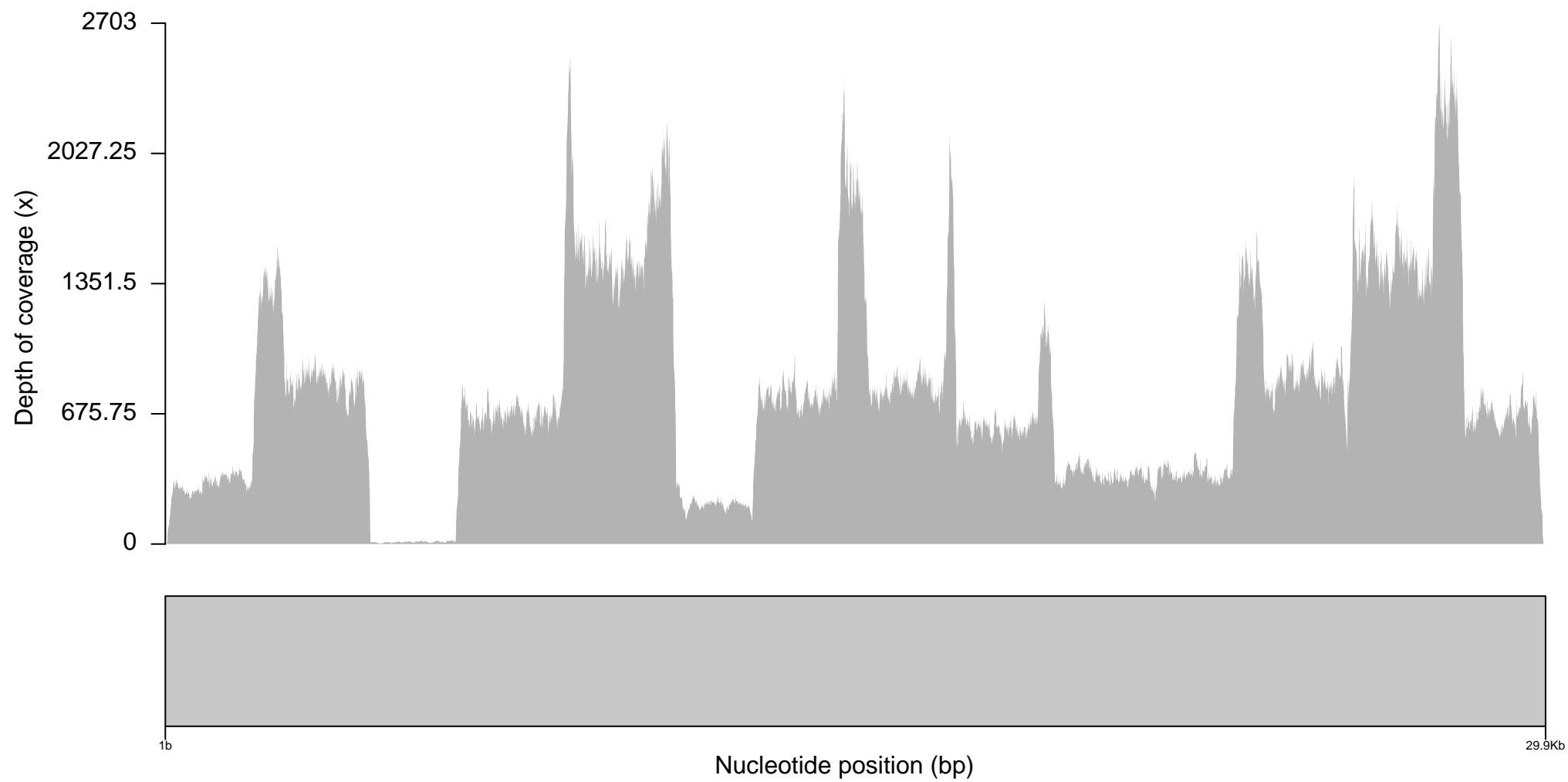

**39456**

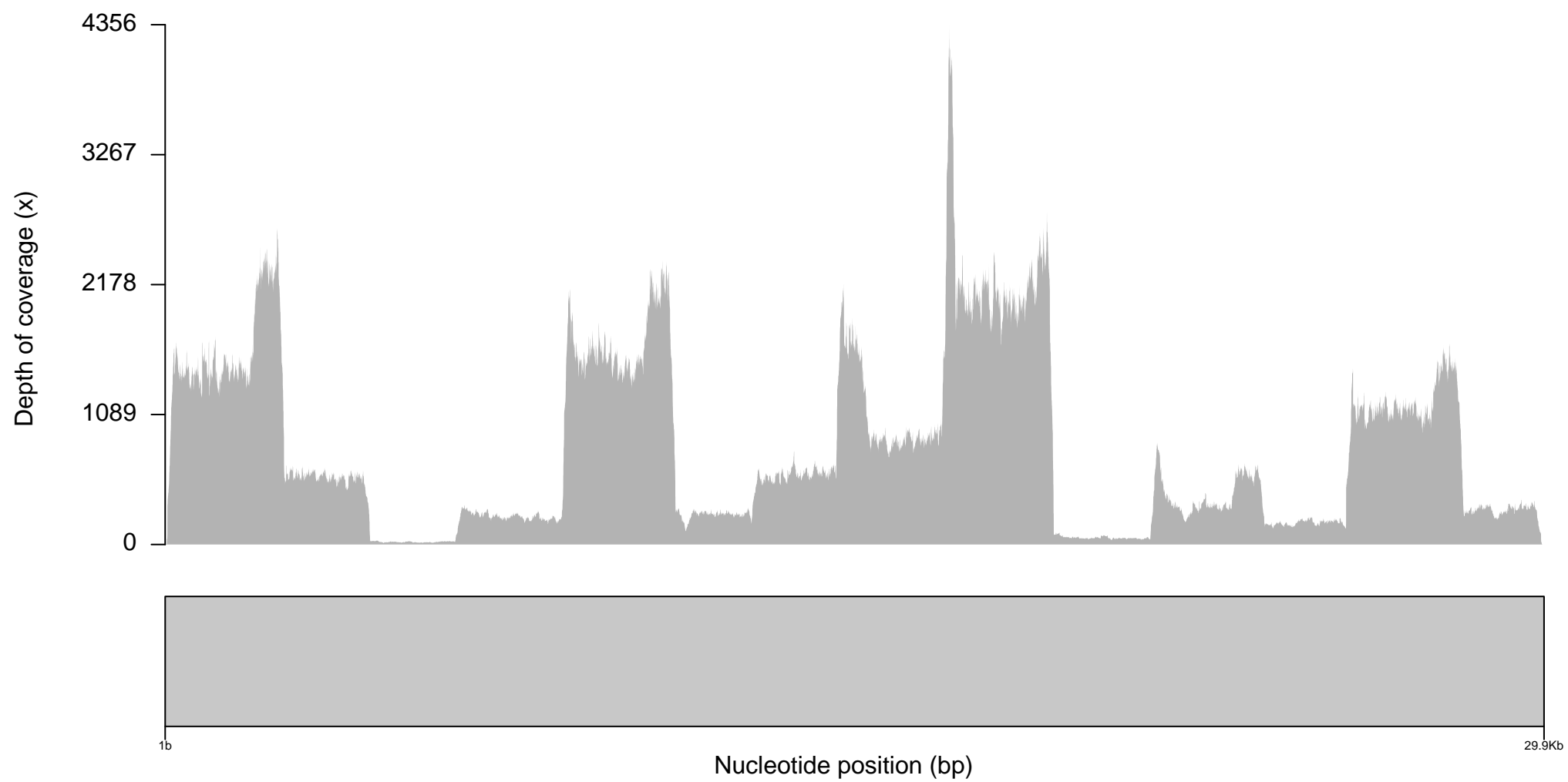

**39457**

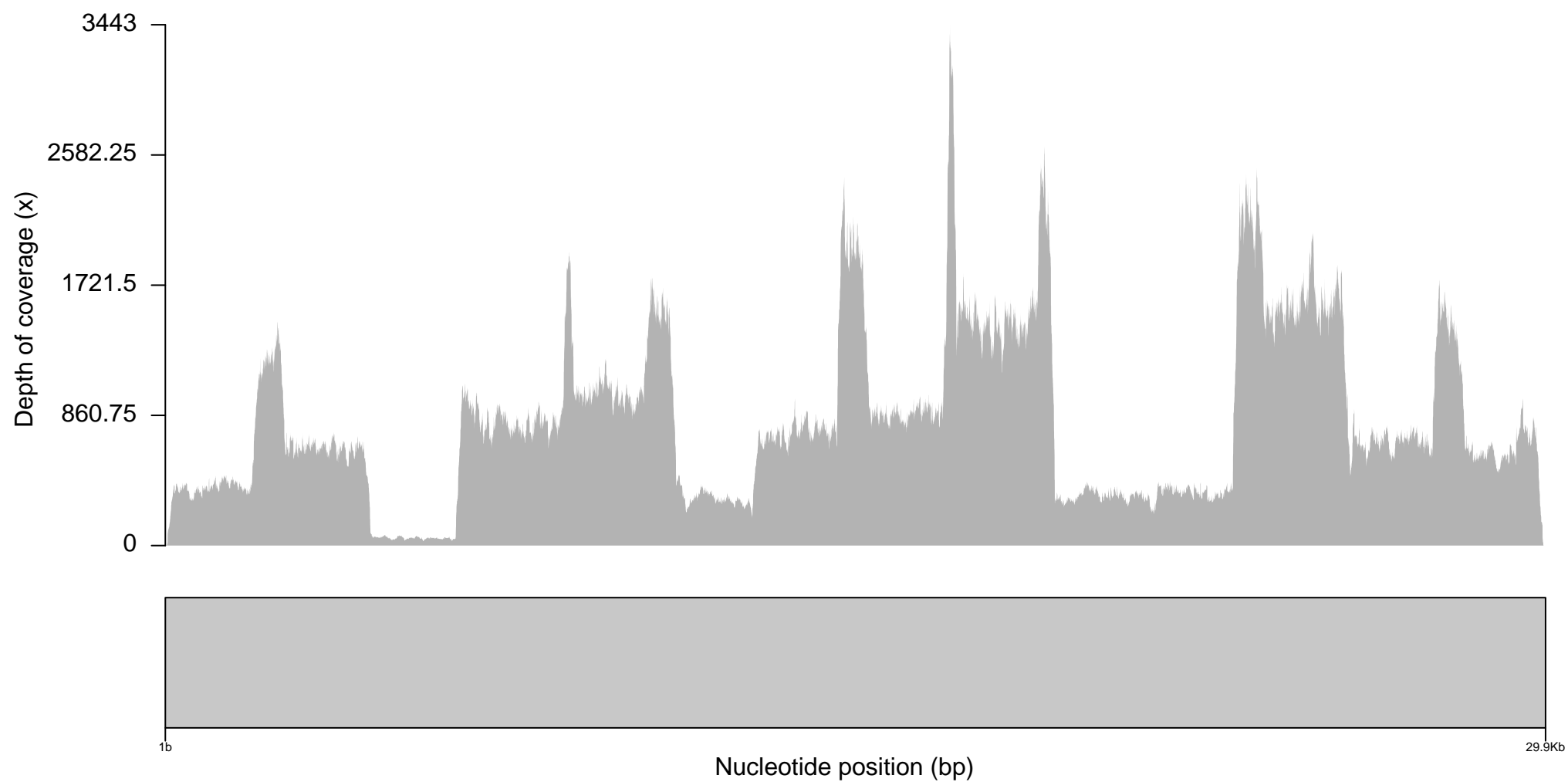

**39458**

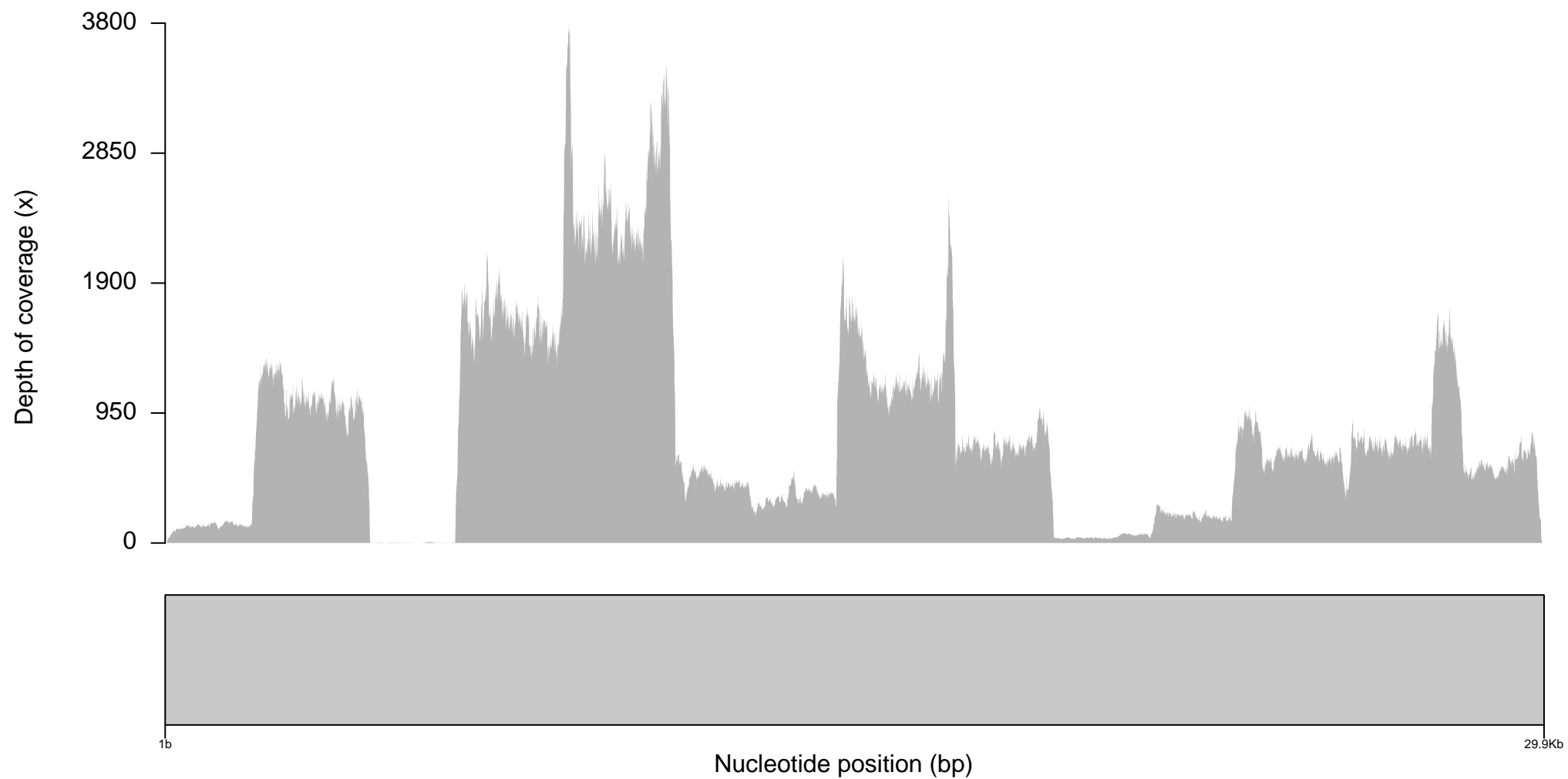

**39459**

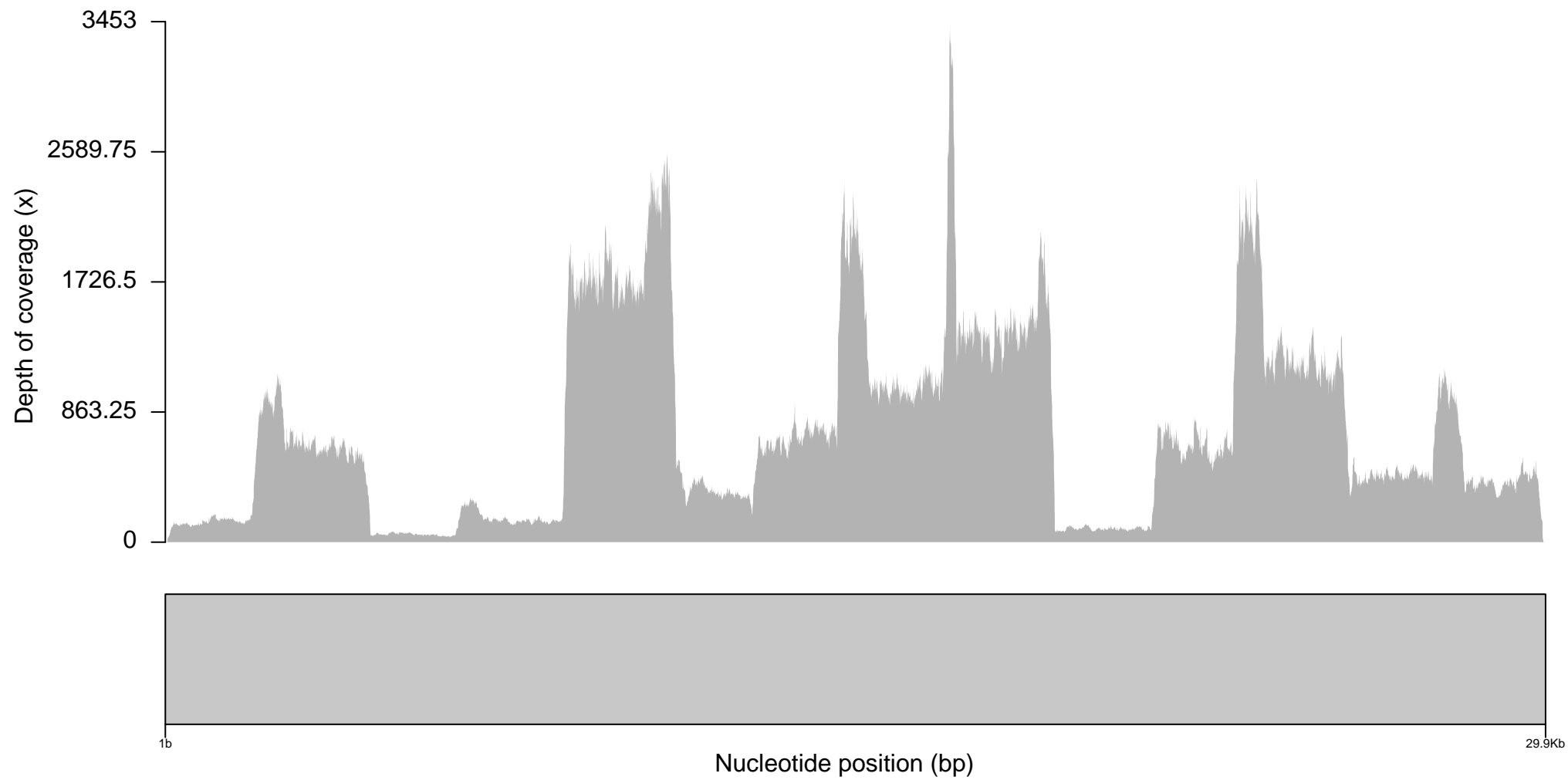

39460

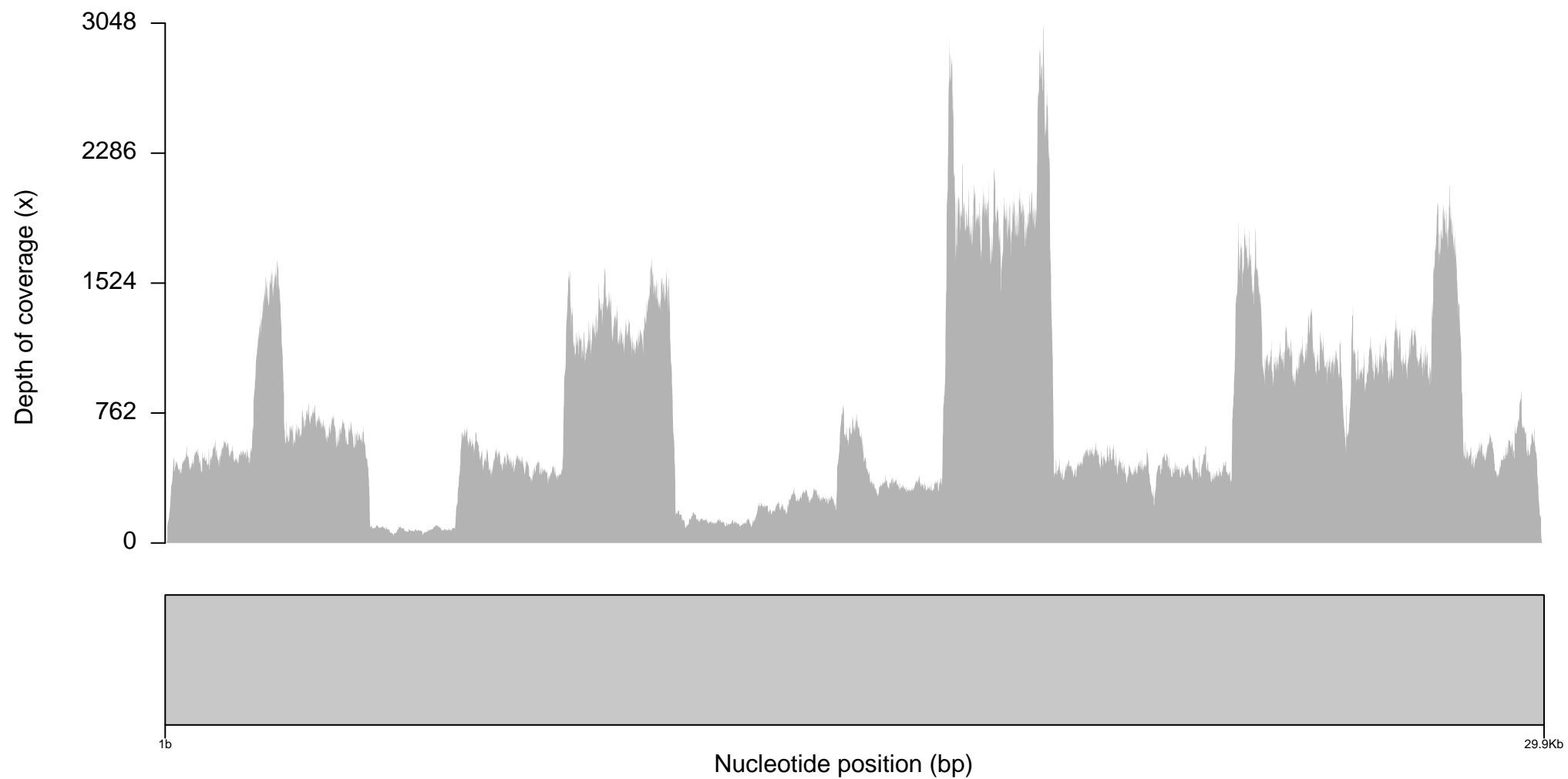

**39461**

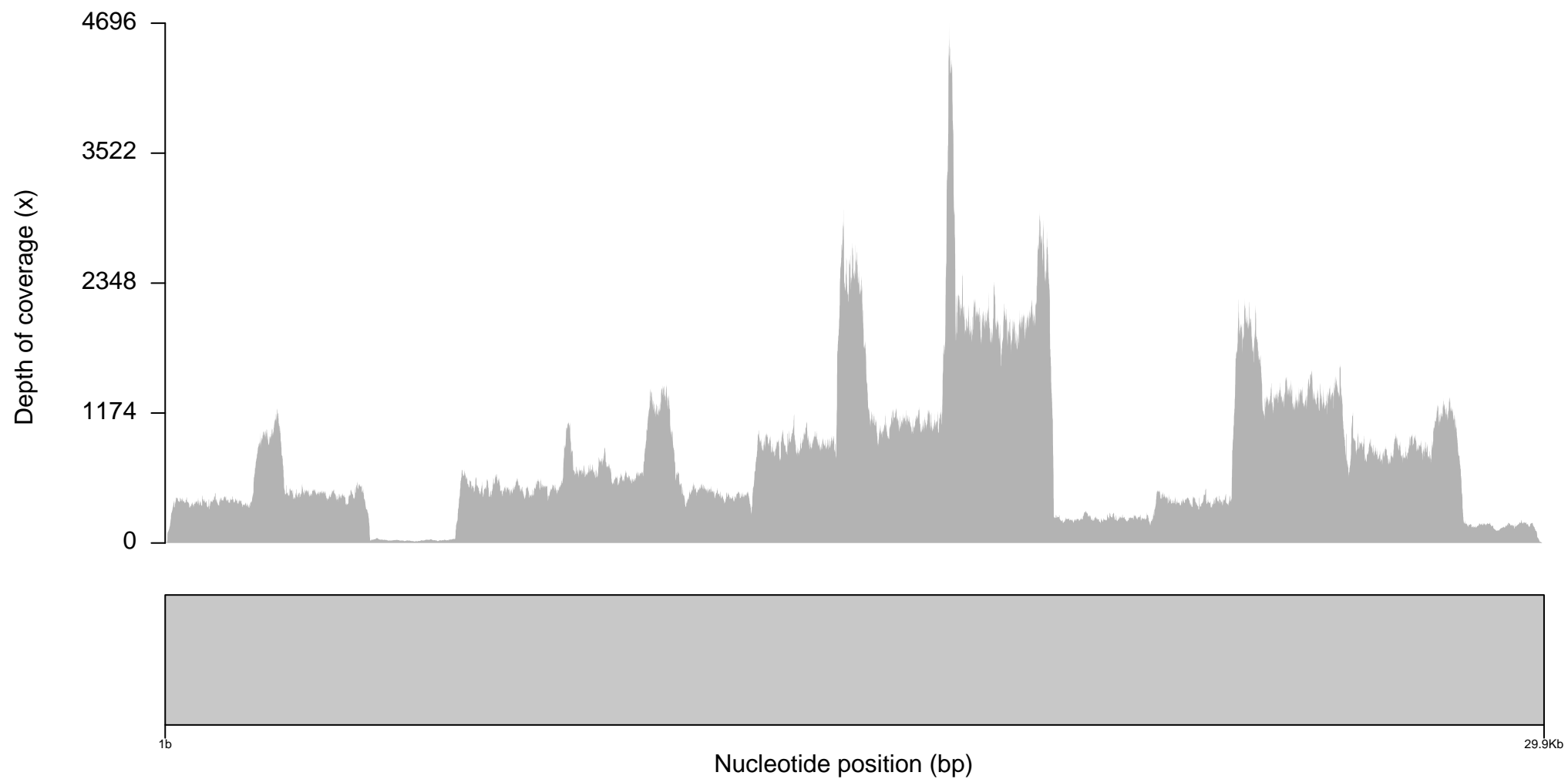

**39462**

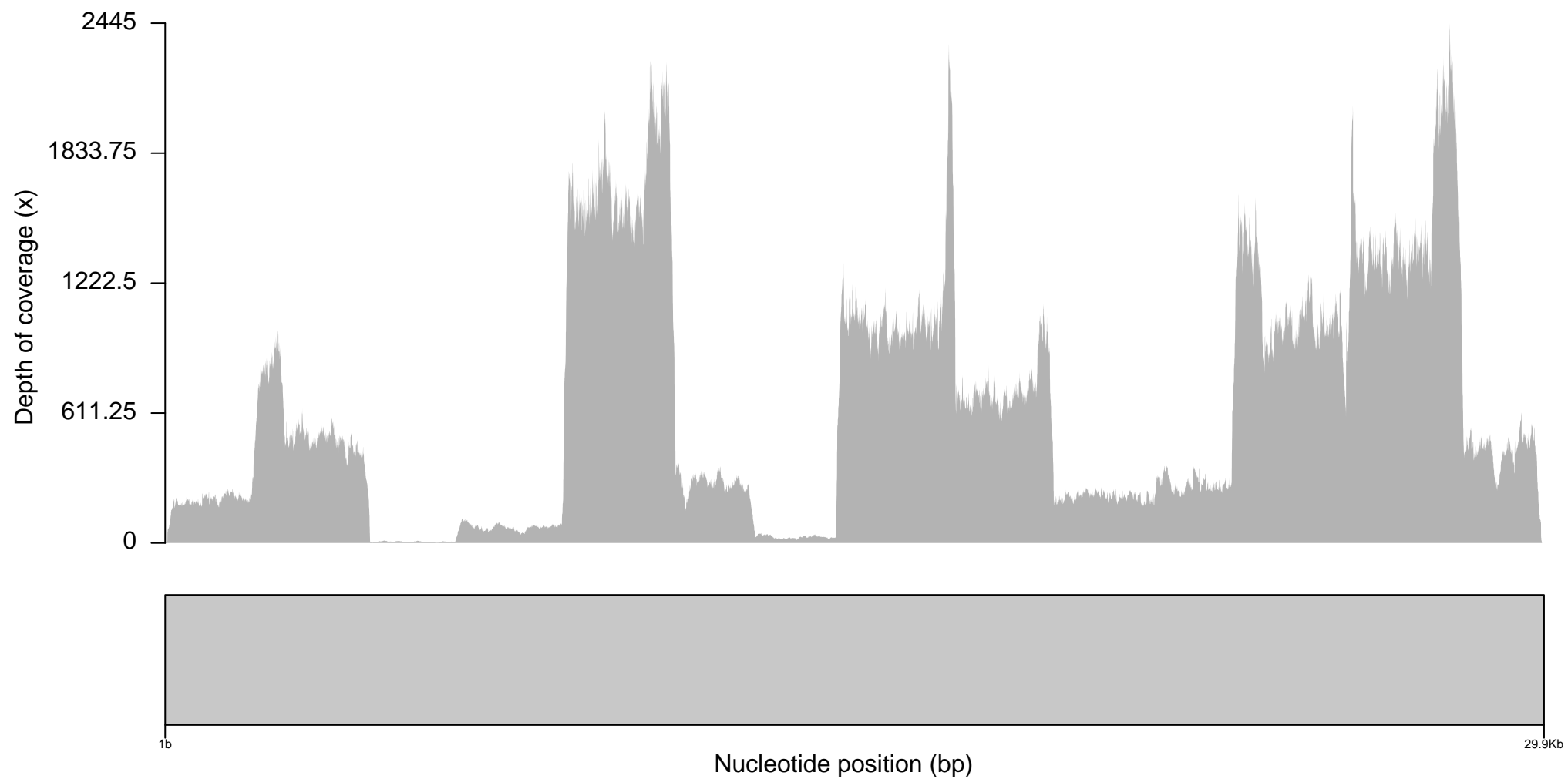

**39463**

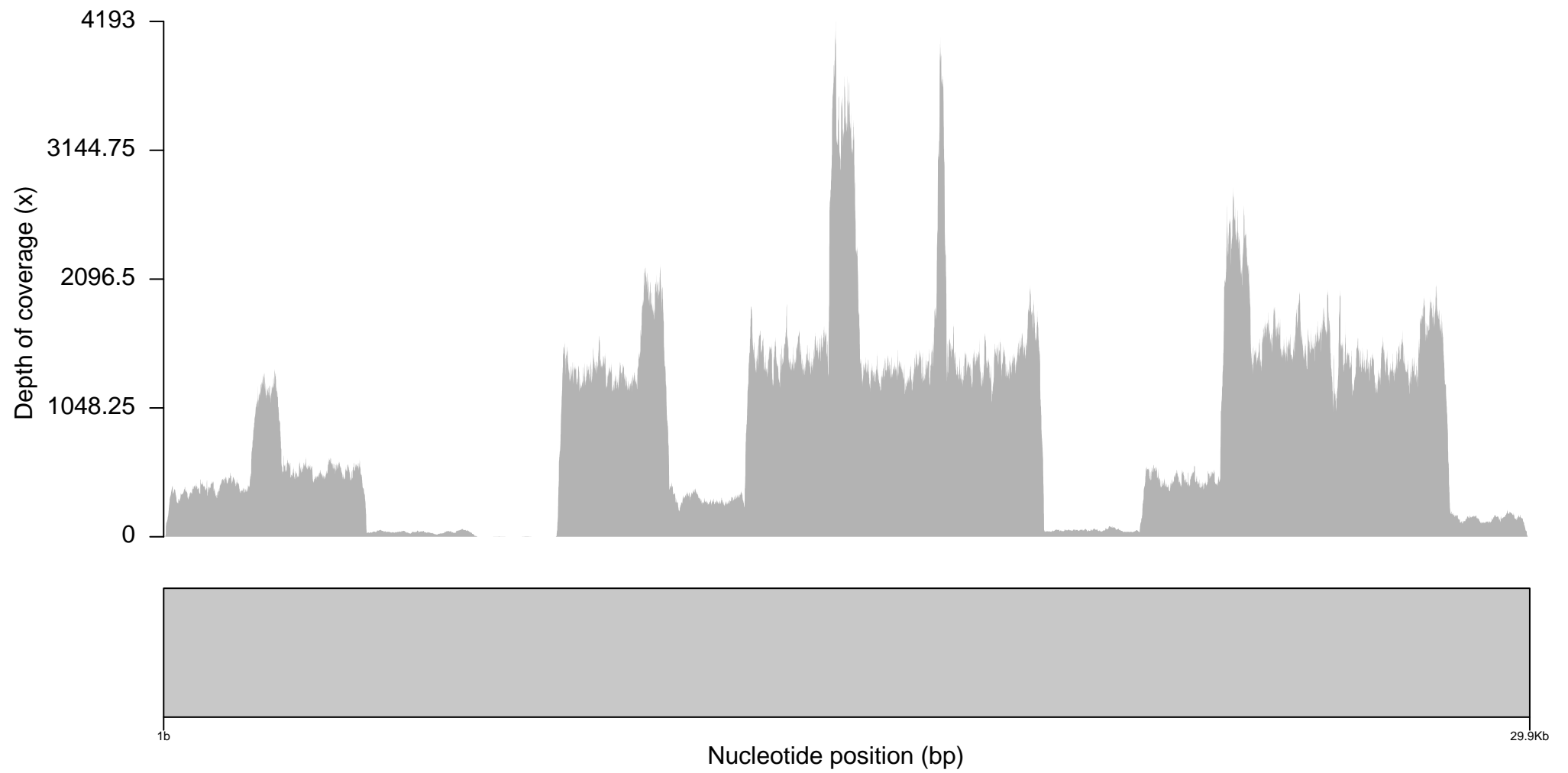

**39464**

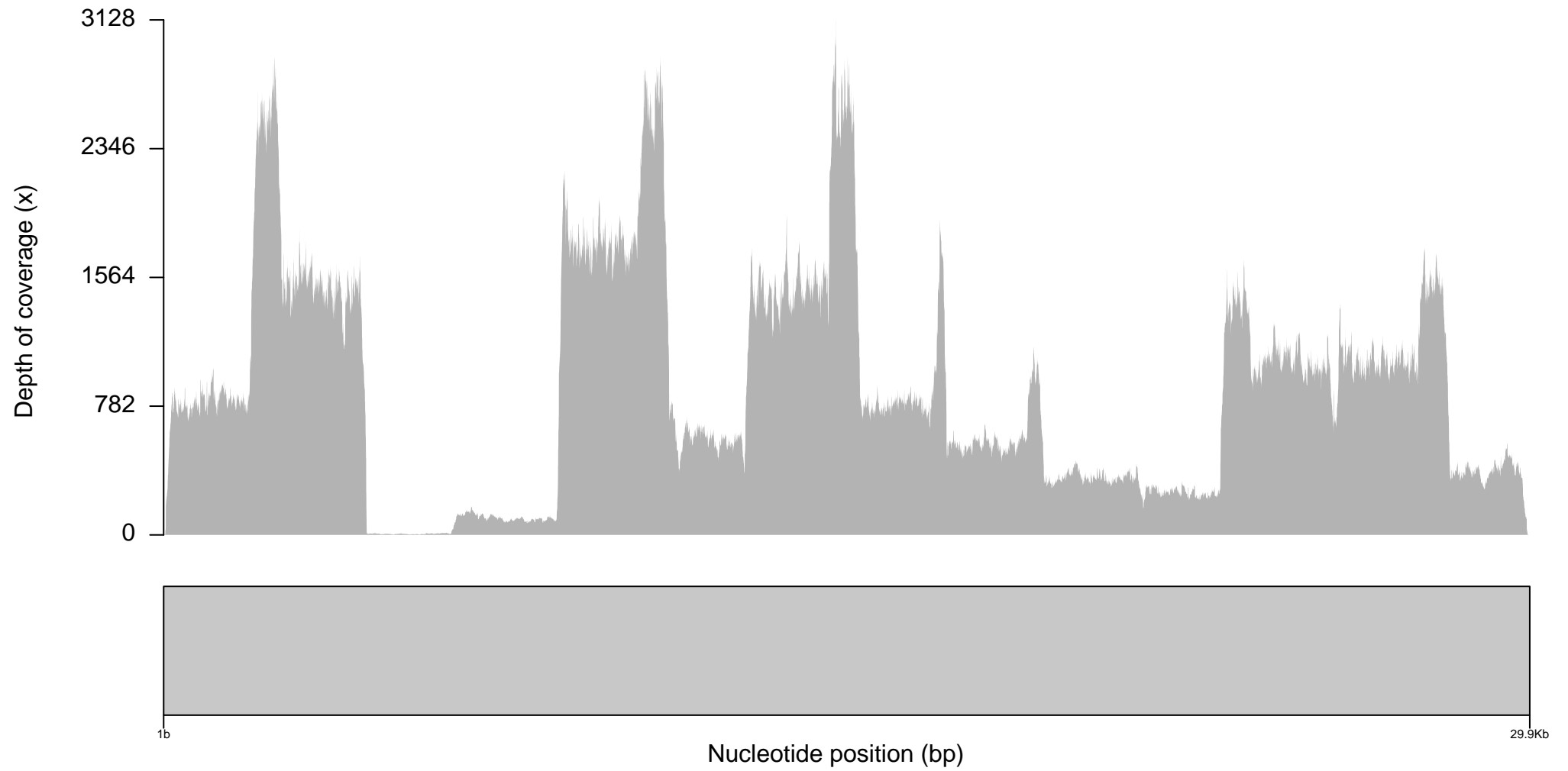

**39465**

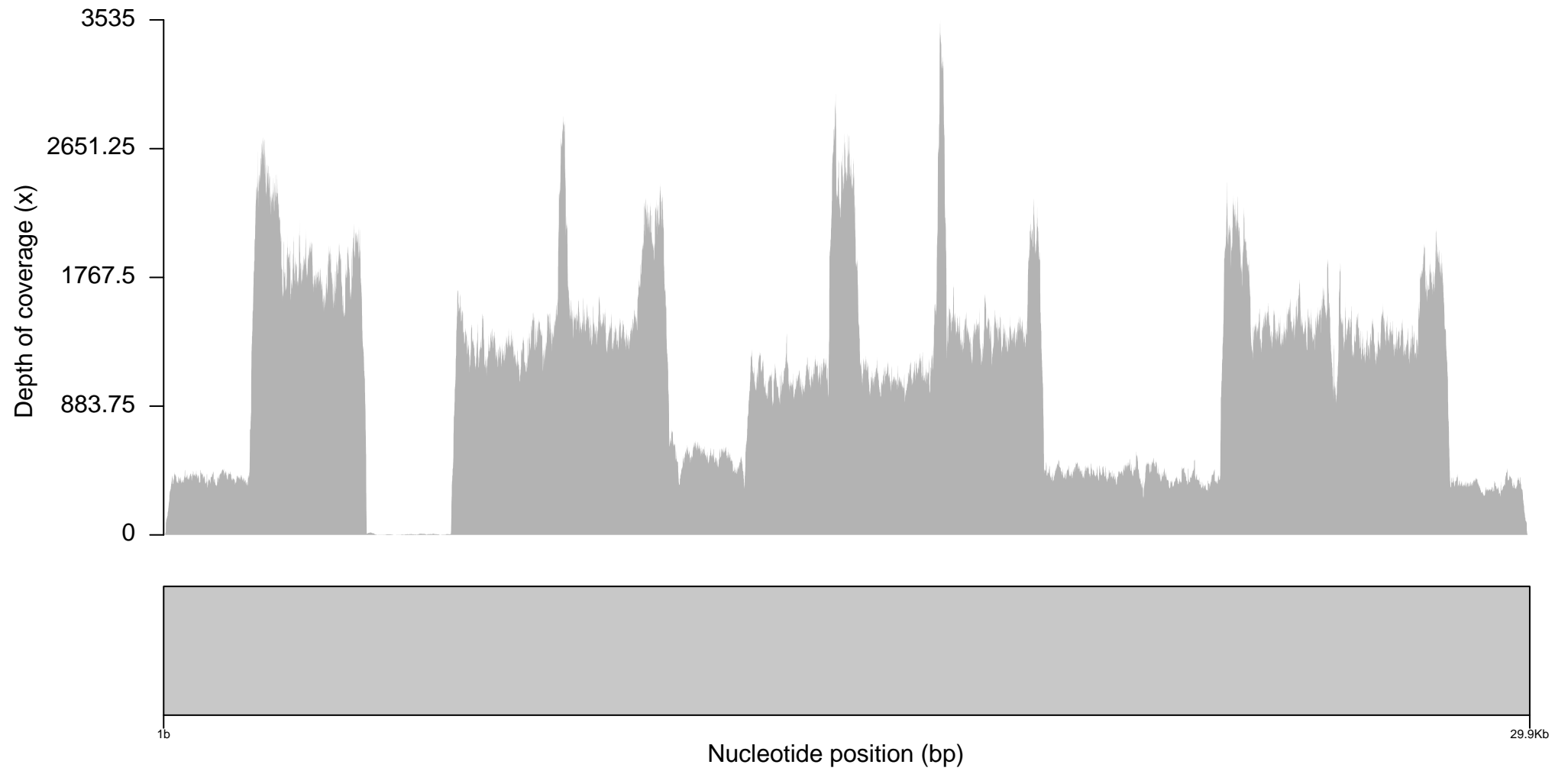

**39466**

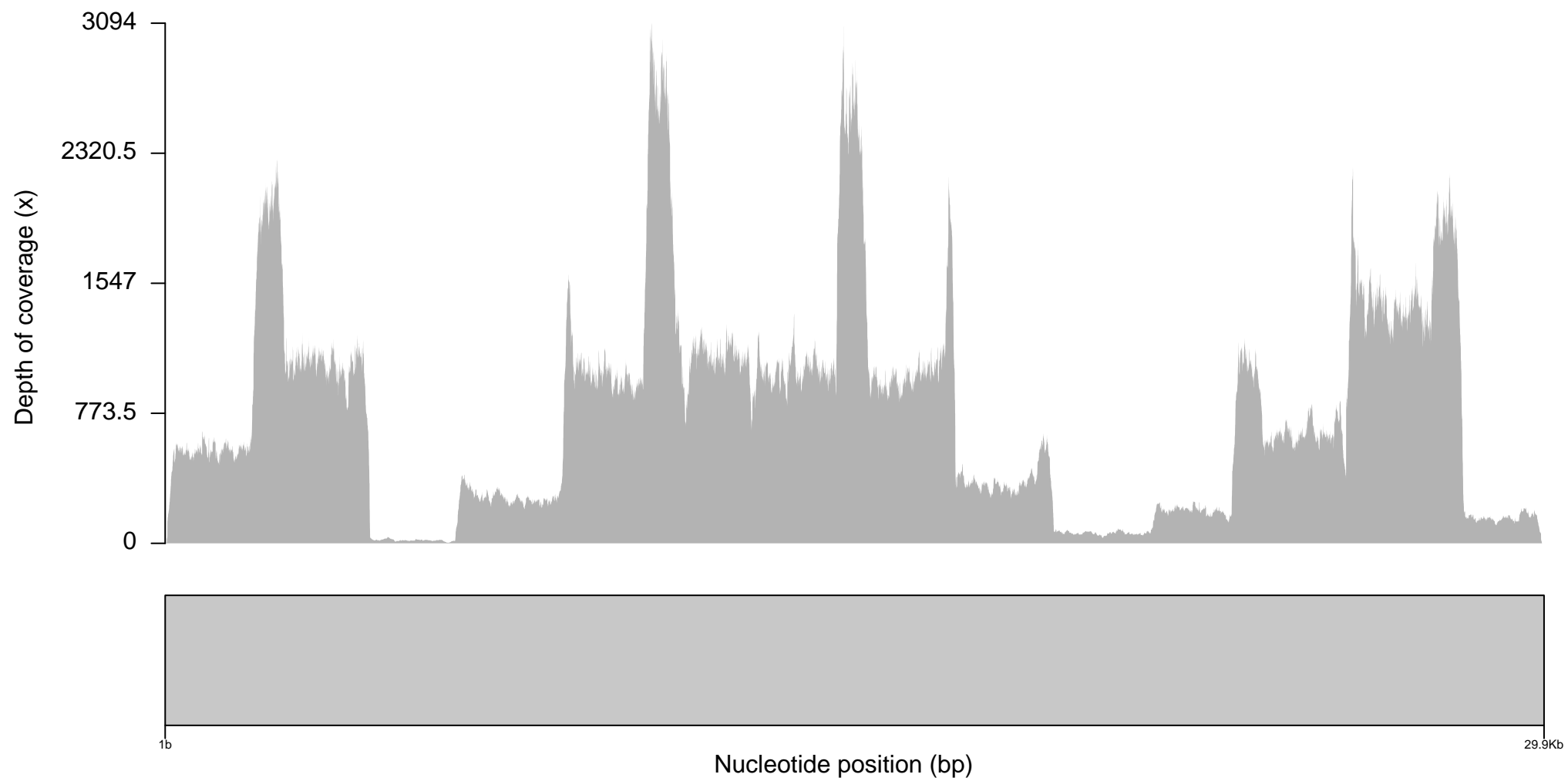

**39467**

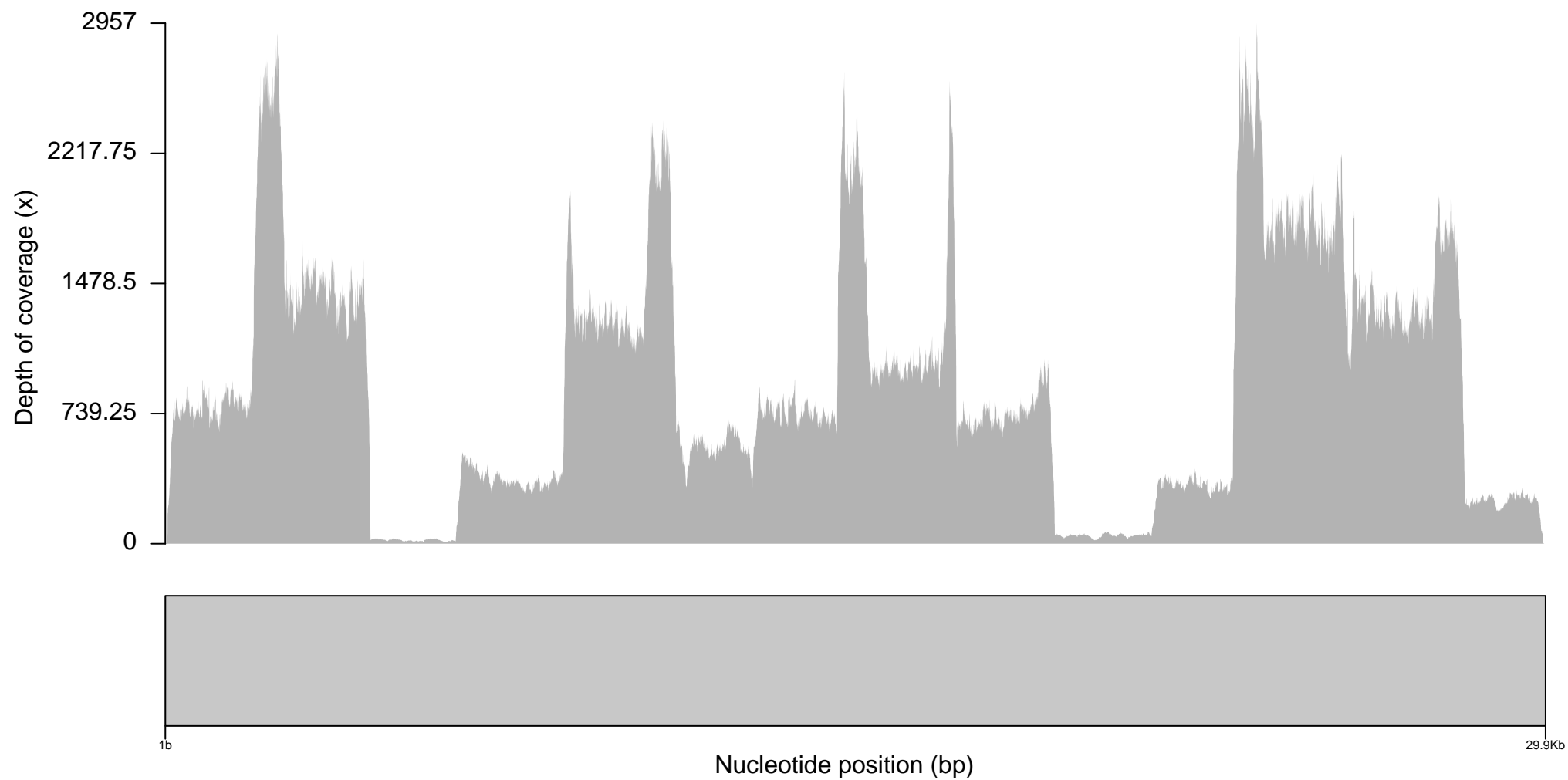

Supplement: Supplementary File 1 — Sequencing depth and coverage plots for the forty-four sequenced genomes from this study. [file Data_Sheet_1.PDF]

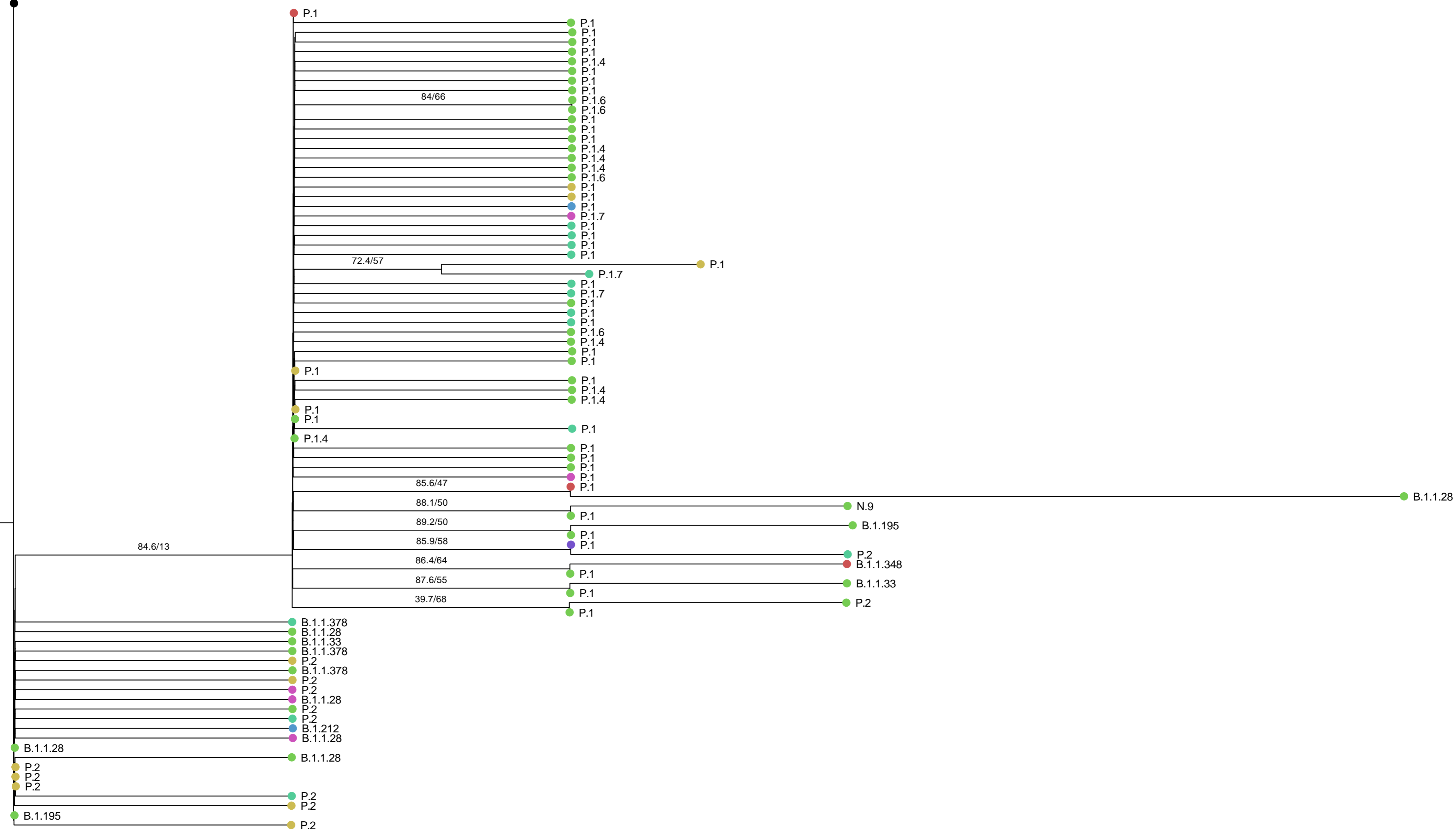

B



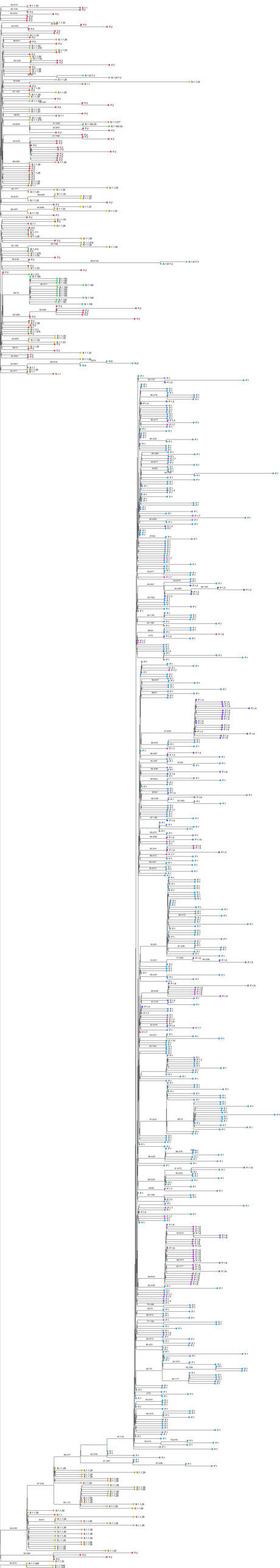

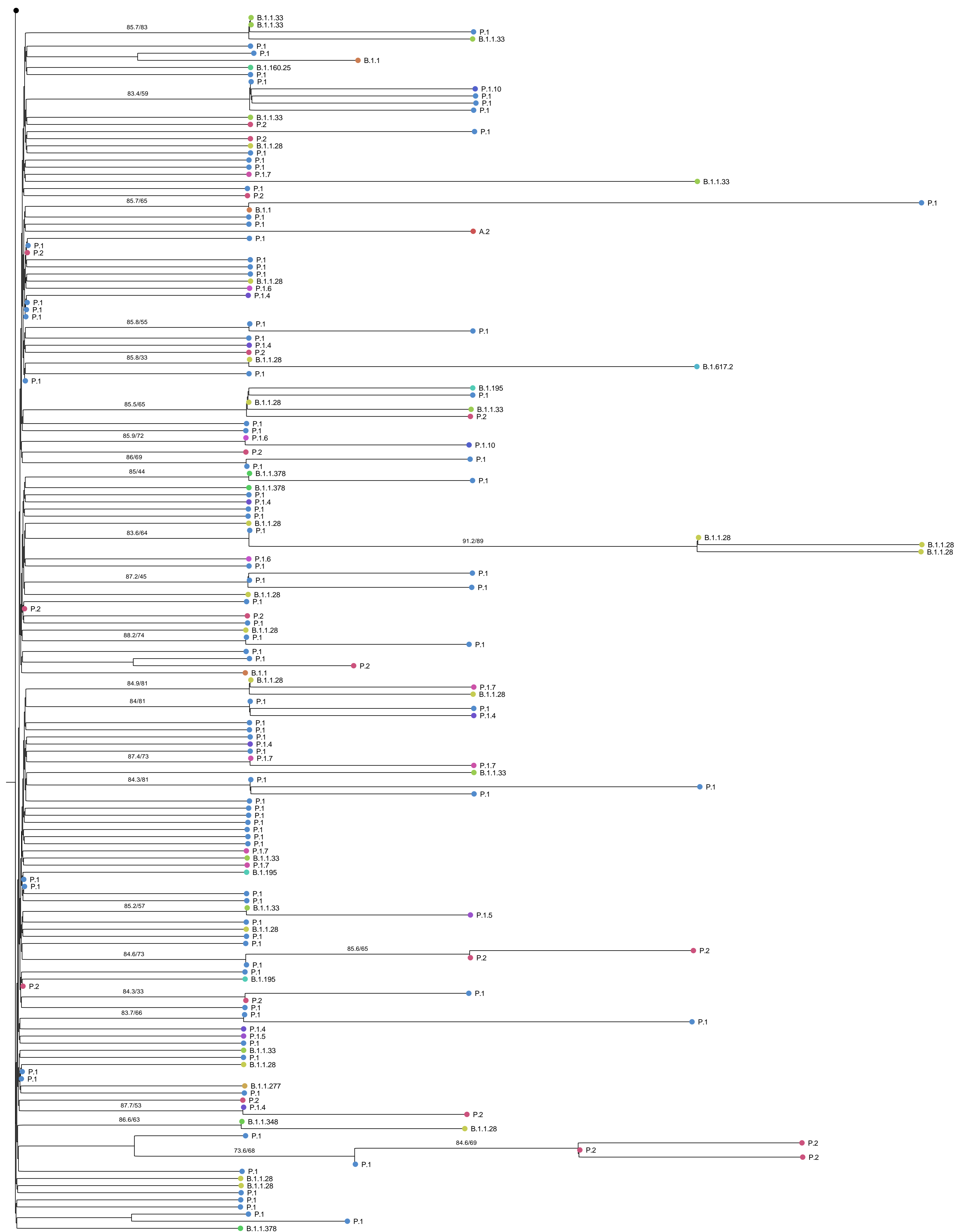

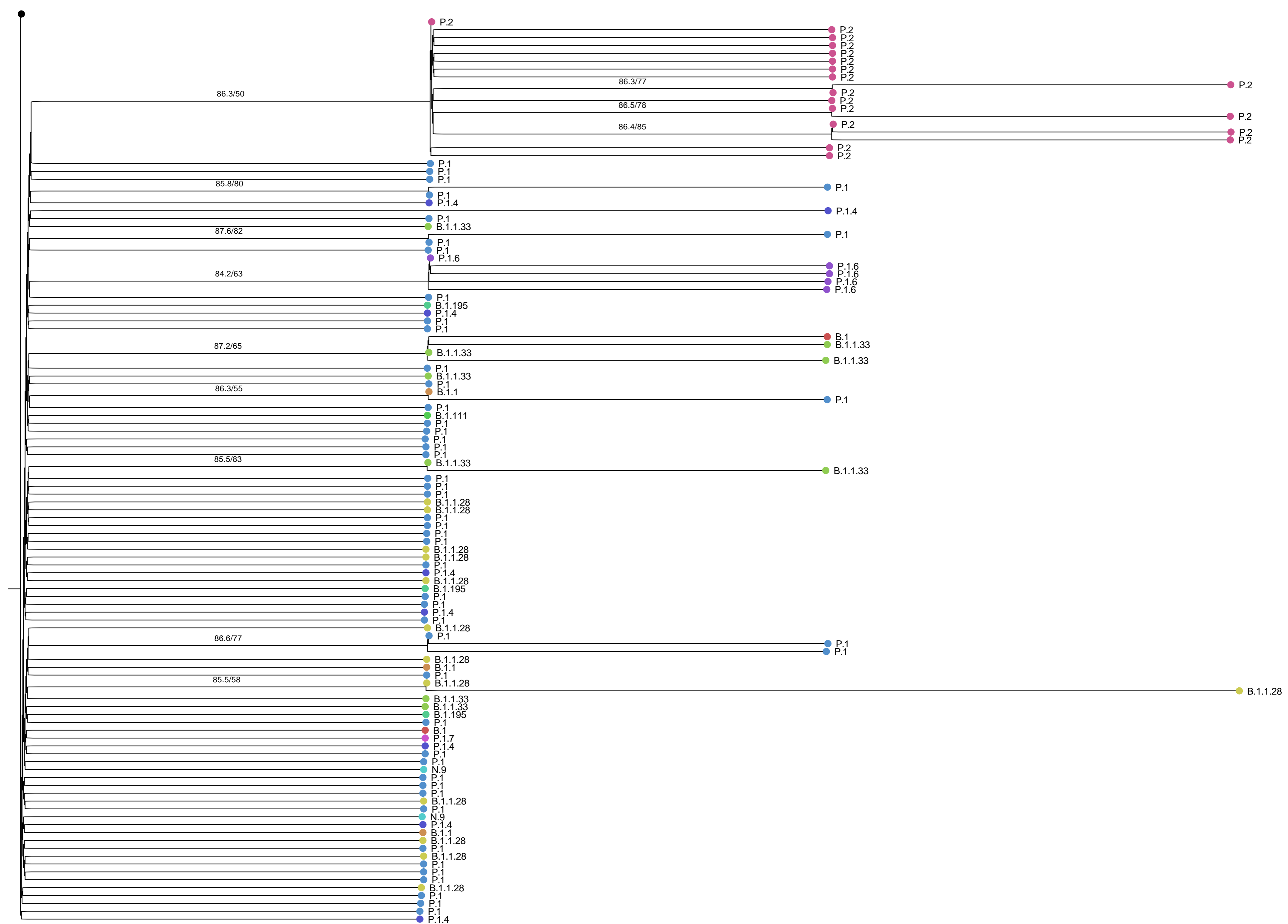

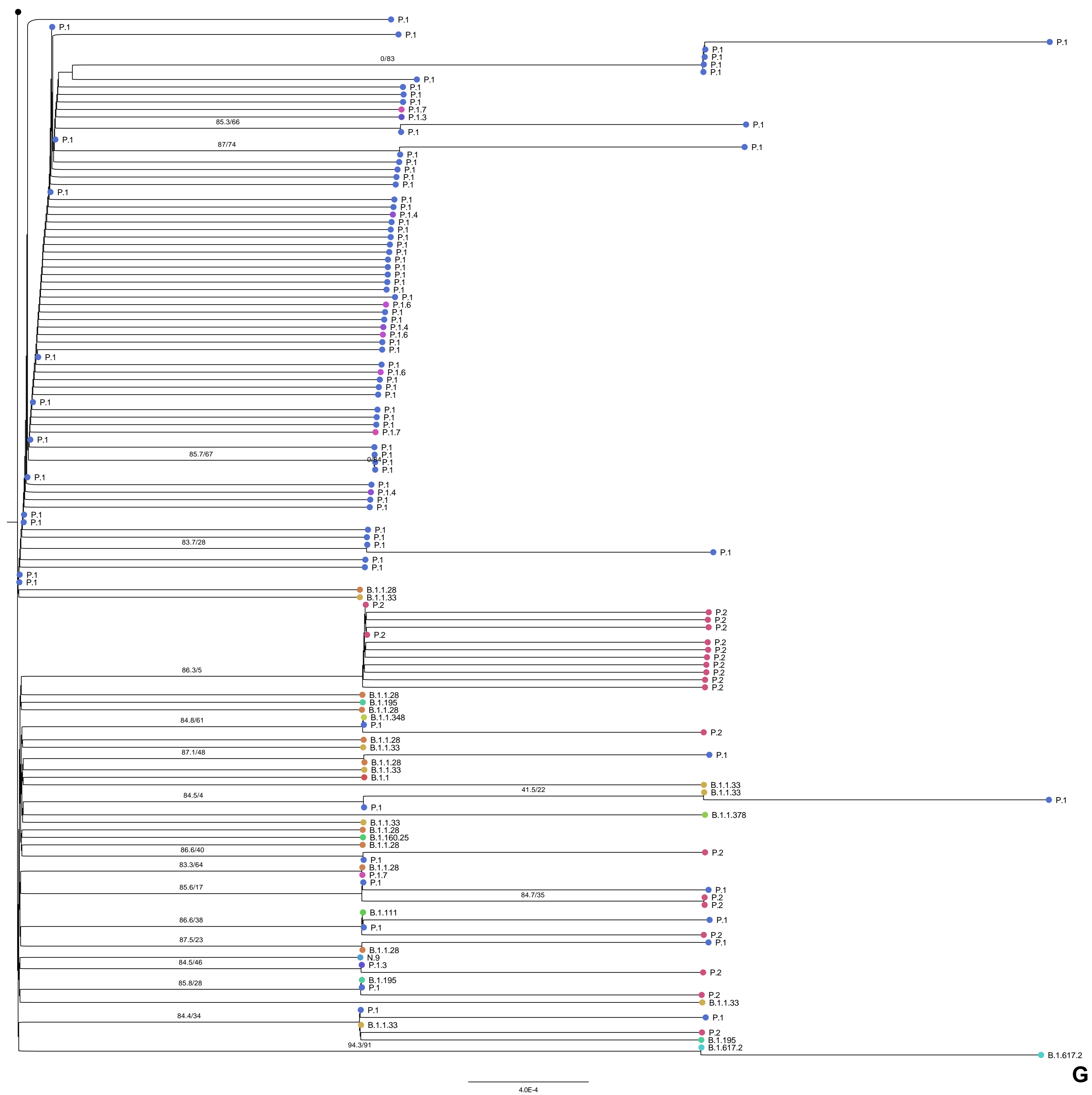

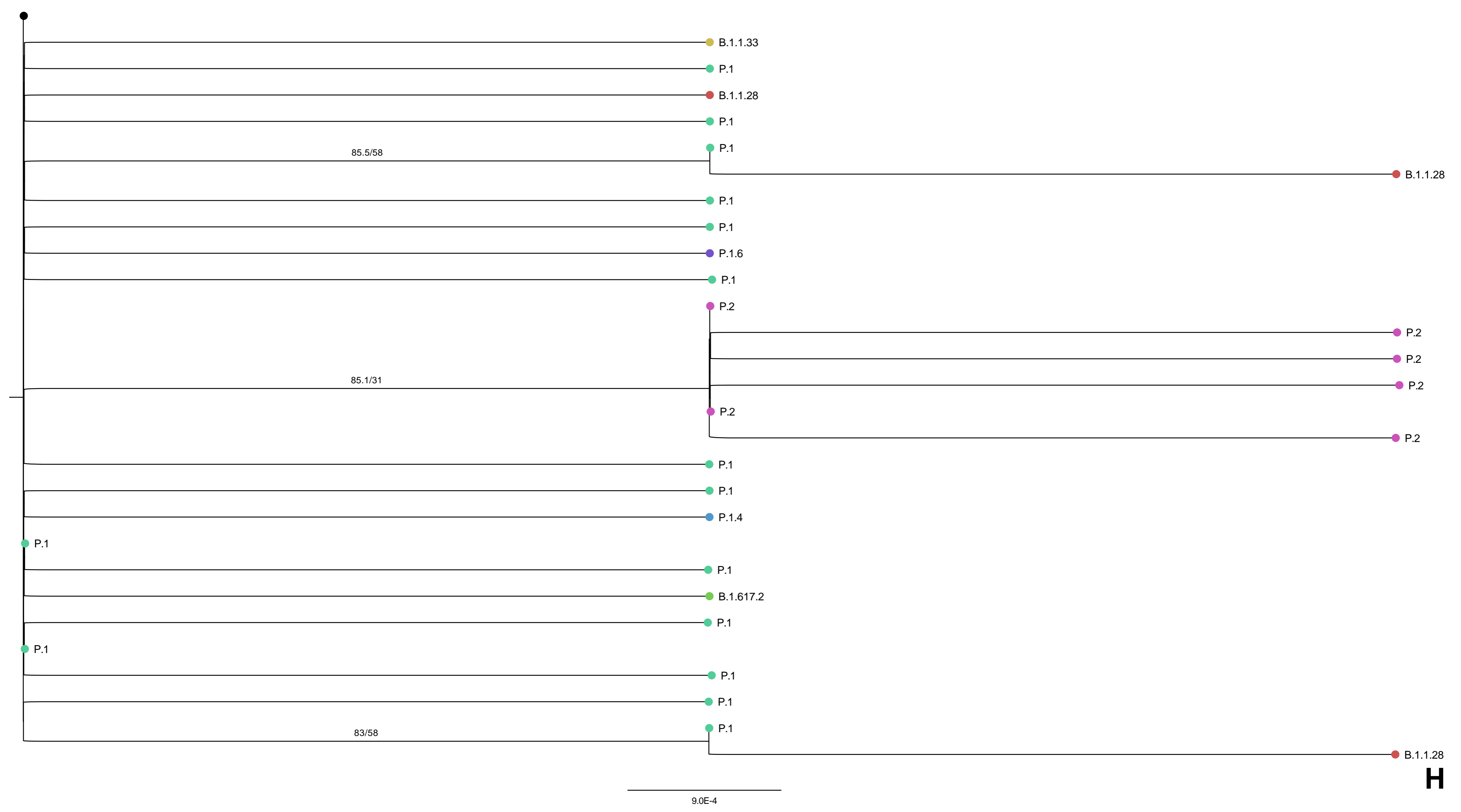

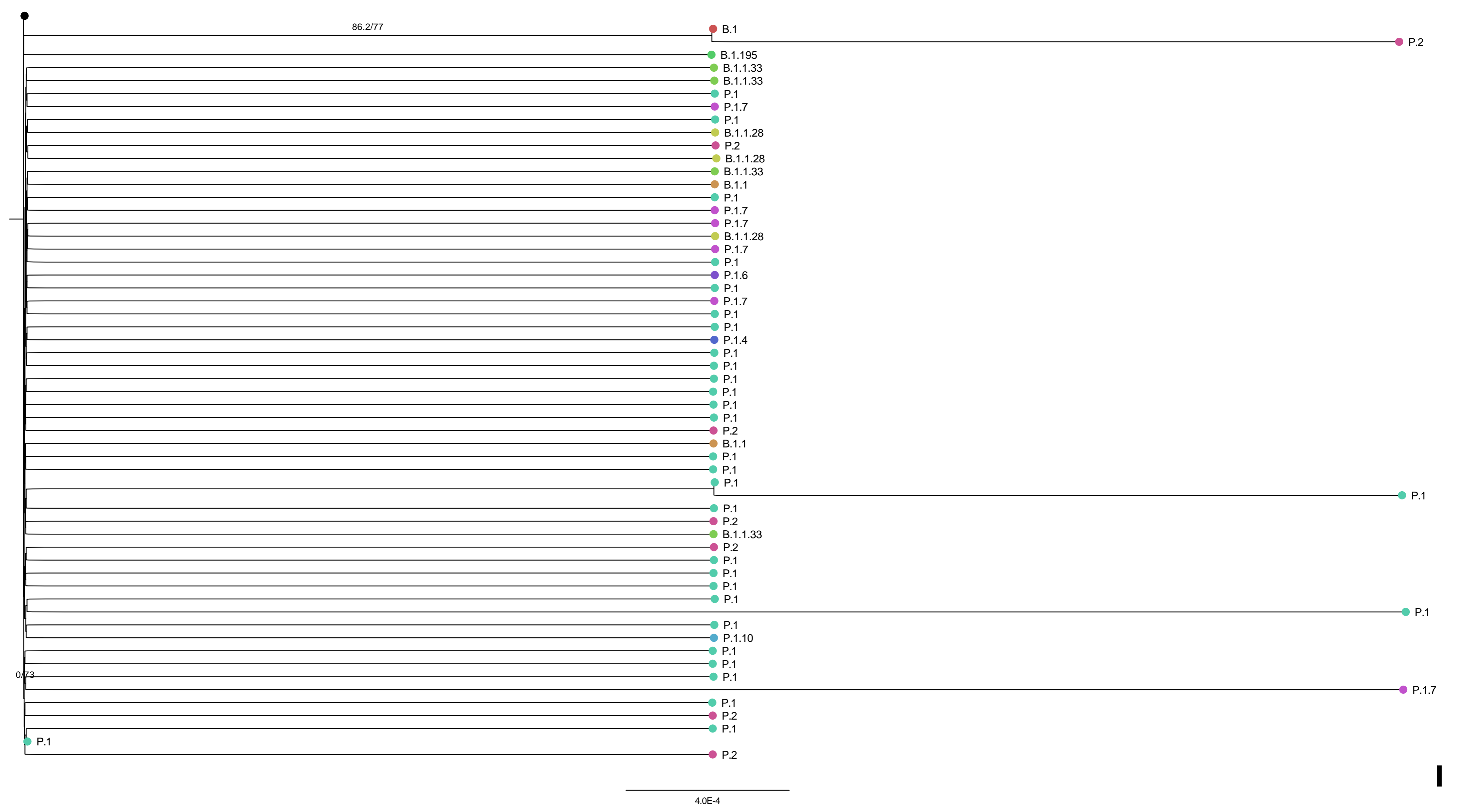

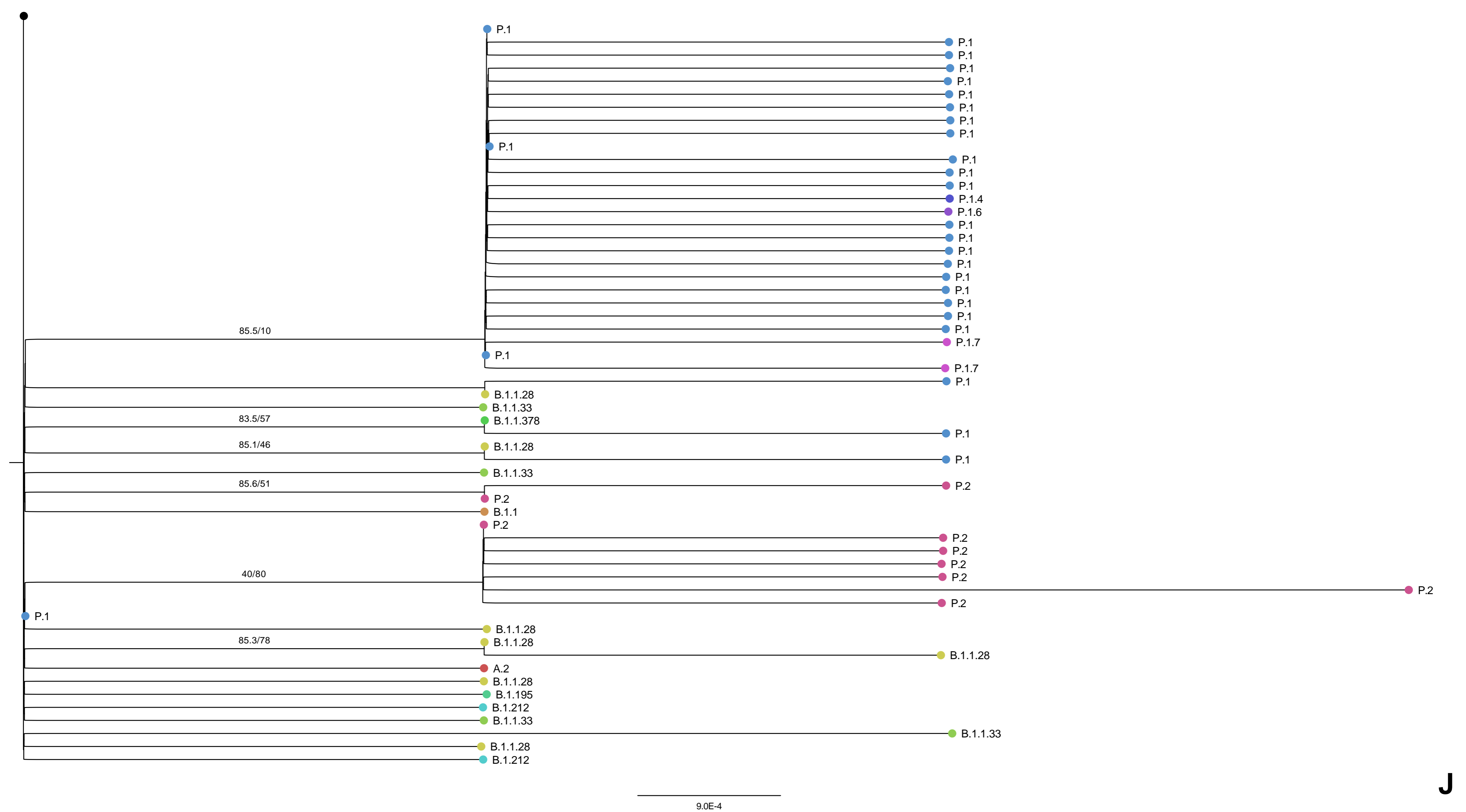

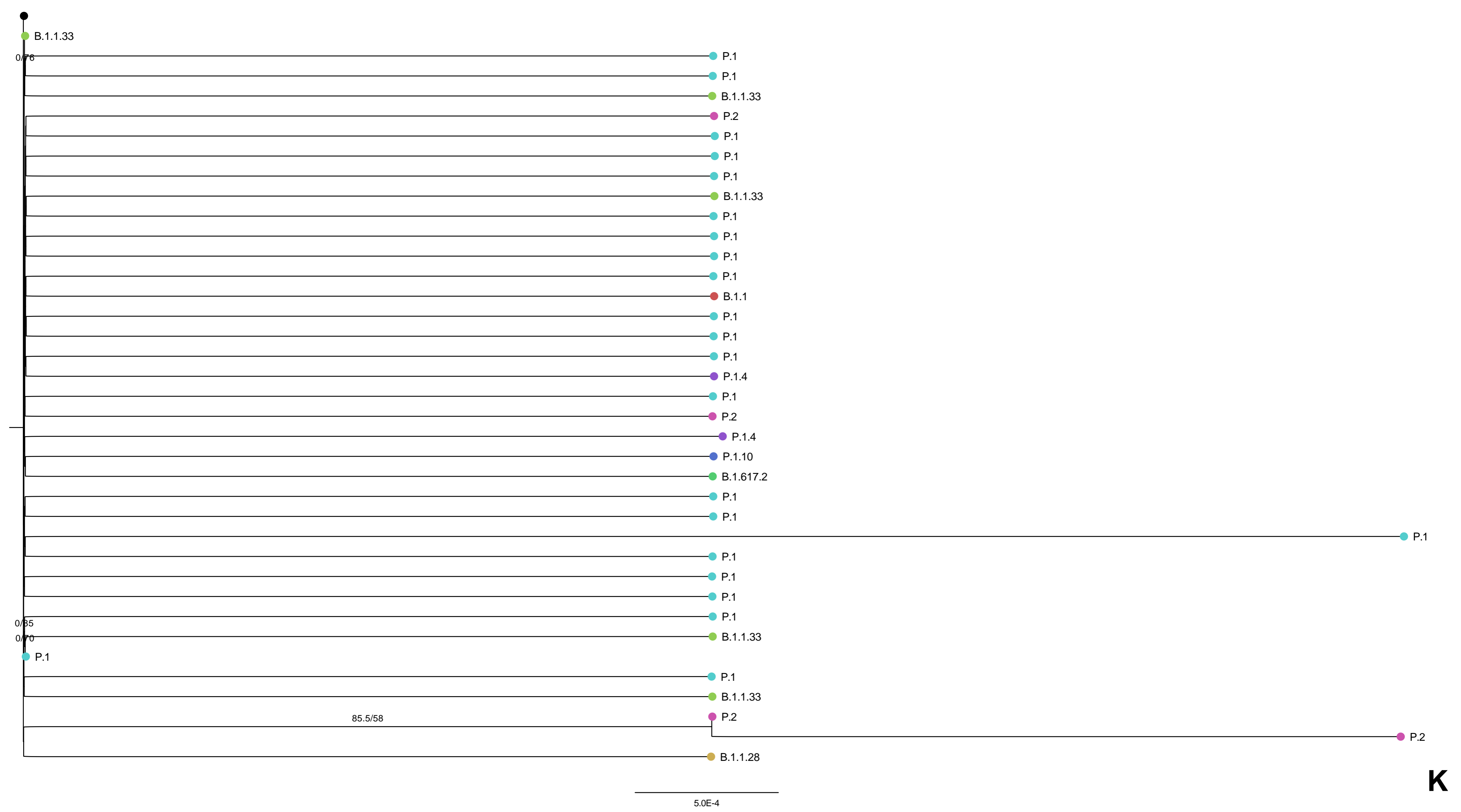

Supplement: Supplementary File 4 — Phylogenetic analysis of spike and non-structural proteins from ORF1a. (A) Spike, (B) NSP1, (C) NSP2, (D) NSP3, (E) NSP4, (F) NSP5, (G) NSP6, (H) NSP7, (I) NSP8, (J) NSP9, and (K) NSP10. Branch support values below 70 for both SH-aLRT and ultrafast bootstrap tests were not labeled in the trees. [file Data_Sheet_4.PDF]
