# Supplementary material for: Comparative Genomics and Characterization of SARS-CoV-2 P.1 (Gamma) Variant of Concern From Amazonas, Brazil
Source: Front Med (Lausanne). 2022 Feb 15;9:806611. doi: 10.3389/fmed.2022.806611 (PMC8885995; doi:10.3389/fmed.2022.806611)
Supplement: Supplementary File 2 — Frequency of mutations identified in the P.1 sequenced genomes (this study) compared with sequences from Amazonas, Brazil and World (up to September 12, 2021) available on GISAID. [file Data_Sheet_2.PDF]

Supplementary File 2. Frequency of mutations identified in the P.1 sequenced genomes (this study) compared with sequences from Amazonas, Brazil and World (up to September 12, 2021) available on GISAID.

**Codon**	**ORF codon**	**% Sequenced genomes (n=44)**	**% AM genomes (n=840)**	**% BR genomes (n=19,827)**	**% World genomes (n=53,570)**
N:P80R	N:P80R	100.00 (44)	99.52 (836)	99.09 (19,646)	99.20 (53,139)
N:T135I	N:T135I	2.27 (1)	0.95 (8)	0.63 (125)	0.14 (73)
N:P199L	N:P199L	2.27 (1)	0.00 (0)	0.02 (4)	0.02 (11)
N:R203K	N:R203K	100.00 (44)	99.29 (834)	98.54 (19,538)	92.17 (49,376)
N:G204R	N:G204R	100.00 (44)	99.17 (833)	98.32 (19,493)	93.24 (49,950)
NSP1:T170I	ORF1a:T170I	2.27 (1)	0.00 (0)	0.00 (0)	0.01 (4)
NSP2:N9S	ORF1a:N189S	2.27 (1)	0.00 (0)	0.00 (0)	0.00 (0)
NSP2:L113F	ORF1a:L293F	2.27 (1)	0.24 (2)	0.02 (3)	0.01 (7)
NSP2:L400F	ORF1a:L580F	2.27 (1)	0.00 (0)	0.10 (20)	0.06 (30)
NSP2:K456R	ORF1a:K636R	2.27 (1)	0.60 (5)	0.03 (5)	0.004 (2)
NSP2:V469F	ORF1a:V649F	2.27 (1)	0.00 (0)	0.05 (9)	0.03 (17)
NSP3:A41V	ORF1a:A859V	2.27 (1)	0.12 (1)	0.07 (14)	0.04 (14)
NSP3:T133I	ORF1a:T951I	25.00 (11)	11.79 (99)	1.59 (316)	0.99 (530)
NSP3:T186P	ORF1a:T1004P	2.27 (1)	0.60 (5)	0.02 (4)	0.002 (1)
NSP3:A231V	ORF1a:A1049V	2.27 (1)	0.12 (1)	0.37 (74)	0.04 (19)
NSP3:S370L	ORF1a:S1188L	97.73 (43)	95.95 (806)	97.63 (19,358)	97.60 (52,286)
NSP3:K977Q	ORF1a:K1795Q	56.82 (25)	99.29 (834)	99.80 (19,788)	99.89 (52,976)

NSP3:T1189I	ORF1a:T2007I	2.27 (1)	0.60 (5)	0.17 (33)	0.03 (14)
NSP3:T1365A	ORF1a:T2183A	2.27 (1)	0.12 (1)	0.07 (14)	0.01 (3)
NSP3:S1437F	ORF1a:S2255F	2.27 (1)	0.00 (0)	0.03 (6)	0.002 (1)
NSP3:S1670F	ORF1a:S2488F	2.27 (1)	0.00 (0)	0.11 (21)	0.21 (110)
NSP4:V30A	ORF1a:V2793A	2.27 (1)	0.00 (0)	0.00 (0)	0.00 (0)
NSP4:T83I	ORF1a:T2846I	2.27 (1)	0.60 (5)	1.45 (288)	1.26 (676)
NSP4:H313Y	ORF1a:H3076Y	2.27 (1)	0.00 (0)	0.05 (9)	0.04 (23)
NSP4:S481L	ORF1a:S3244L	2.27 (1)	0.24 (2)	0.15 (29)	0.10 (51)
NSP5:A70V	ORF1a:A3333V	6.82 (3)	2.14 (18)	0.06 (12)	0.002 (1)
NSP5:V86I	ORF1a:V3349I	6.82 (3)	2.14 (18)	0.01 (1)	0.00 (0)
NSP5:P241L	ORF1a:P3504L	2.27 (1)	0.24 (2)	0.65 (128)	0.13 (72)
NSP6:A46V	ORF1a:A3615V	2.27 (1)	0.00 (0)	0.13 (25)	0.02 (13)
NSP6:A51V	ORF1a:A3620V	6.82 (3)	2.14 (18)	0.07 (14)	0.03 (14)
NSP6:S106del	ORF1a:S3675del	27.27 (12)	98.33 (826)	96.55 (19,142)	97.22 (52,081)
NSP6:G107S	ORF1a:G3676S	4.55 (2)	0.24 (2)	1.06 (210)	0.81 (436)
NSP6:G107del	ORF1a:G3676del	27.27 (12)	98.45 (827)	96.81 (19,195)	97.23 (52,085)
NSP6:F108L	ORF1a:F3677L	11.36 (5)	0.24 (2)	1.85 (367)	1.02 (546)
NSP6:F108del	ORF1a:F3677del	27.27 (12)	98.57 (828)	96.86 (19,205)	97.21 (52,077)
NSP6:V149A	ORF1a:V3718A	2.27 (1)	0.60 (5)	0.02 (4)	0.01 (4)
NSP8:E155G	ORF1a:E4097G	2.27 (1)	0.00 (0)	0.06 (11)	0.01 (8)

NSP12:P323L	ORF1b:P314L	97.73 (43)	99.64 (837)	98.56 (19,541)	99.51 (53,310)
NSP12:I548V	ORF1b:I539V	4.55 (2)	1.19 (10)	0.99 (196)	0.02 (12)
NSP12:Q822H	ORF1b:Q813H	2.27 (1)	0.12 (1)	0.13 (25)	0.06 (30)
NSP13:S74L	ORF1b:S997L	2.27 (1)	0.60 (5)	0.13 (25)	0.10 (52)
NSP13:M274I	ORF1b:M1197I	2.27 (1)	0.24 (2)	0.03 (5)	0.02 (11)
NSP13:E341D	ORF1b:E1264D	97.73 (43)	99.05 (832)	98.32 (19,494)	99.33 (53,211)
NSP13:L581F	ORF1b:L1504F	4.55 (2)	0.00 (0)	0.20 (39)	0.10 (55)
NSP14:P158H	ORF1b:P1682H	2.27 (1)	0.12 (1)	0.00 (0)	0.01 (5)
NSP15:D39Y	ORF1b:D2090Y	2.27 (1)	0.00 (0)	0.06 (11)	0.03 (14)
NSP15:E170D	ORF1b:E2221D	4.55 (2)	0.00 (0)	0.00 (0)	0.01 (5)
NSP16:K160R	ORF1b:K2557R	2.27 (1)	0.24 (2)	0.86 (170)	0.35 (186)
NS3:P25L	ORF3a:P25L	4.55 (2)	0.12 (1)	0.04 (8)	0.02 (11)
NS3:P104S	ORF3a:P104S	4.55 (2)	0.00 (0)	0.04 (7)	0.02 (10)
NS3:S117I	ORF3a:S117I	2.27 (1)	0.00 (0)	0.02 (4)	0.00 (0)
NS3:W131C	ORF3a:W131C	2.27 (1)	0.12 (1)	0.24 (47)	0.15 (81)
NS3:E181V	ORF3a:E181V	2.27 (1)	0.00 (0)	0.00 (0)	0.002 (1)
NS3:S253P	ORF3a:S253P	100.00 (44)	99.76 (838)	98.99 (19,626)	98.85 (52,954)
NS7a:E22D	ORF7a:E22D	2.27 (1)	0.12 (1)	0.14 (27)	0.09 (46)
NS7b:S31L	ORF7b:S31L	2.27 (1)	0.83 (7)	0.07 (14)	0.06 (32)
NS8:E59D	ORF8:E59D	2.27 (1)	0.48 (4)	0.01 (2)	0.00 (0)

NS8:E92K	ORF8:E92K	97.73 (43)	98.93 (831)	97.59 (19,350)	98.84 (52,951)
ORF9b:Q77E	ORF9b:Q77E	100.00 (44)	-	-	-

AM: Amazonas, BR: Brazil. The genomes sequenced in this study are not included in AM counts. AM counts are not included in BR and BR counts are not included in World counts. This analysis included 74,281 P.1 genome sequences available on the GISAID database up to September 12, 2021. Mutations were verified on the GISAID database on September 23, 2021. There is no data on GISAID for mutations in ORF9b (alternative N gene ORF).
