# Supplementary material for: Comparative Genomics and Characterization of SARS-CoV-2 P.1 (Gamma) Variant of Concern From Amazonas, Brazil
Source: Front Med (Lausanne). 2022 Feb 15;9:806611. doi: 10.3389/fmed.2022.806611 (PMC8885995; doi:10.3389/fmed.2022.806611)
Supplement: Supplementary File 3 — Collection dates (first occurrence) on GISAID database for SARS-CoV-2 mutations identified in the forty-four sequenced genomes from this study. [file Data_Sheet_3.PDF]

Supplementary File 3. First occurrence (by collection date) registered on GISAID for SARS-CoV-2 mutations identified in the sequenced genomes from this study.

Mutation	Amazonas	Brazil	Brazil (outside Amazonas)	World (outside Brazil)
N:P80R	2020-12-03	2020-09-11 / SP	2020-09-11 / SP	2020-12-17 / Peru
N:T135I	2021-01-20	2021-01-10 / AP	2021-01-10 / AP	2021-01-02 / Peru
N:P199L	**2021-02-22**	**2021-02-22 / AM**	2021-03-09 / SE	2021-02-11 / USA
N:R203K	2020-12-03	2020-09-11 / SP	2020-09-11 / SP	2020-12-17 / Peru
N:G204R	2020-12-03	2020-09-11 / SP	2020-09-11 / SP	2020-12-17 / Peru
NSP1:T170I	**2021-02-25**	**2021-02-25 / AM**	--	2021-06-28 / Canada
NSP2:N9S	**2021-02-25**	**2021-02-25 / AM**	--	--
NSP2:L113F	2021-02-11	2021-02-11 / AM	2021-04-01 / PR	2021-04-23 / Paraguay
NSP2:L400F	**2021-03-05**	2021-02-26 / SE	2021-02-26 / SE	2021-04-10 / USA
NSP2:K456R	2020-12-23	2020-12-23 / AM	2021-01-21 / PR	2021-01-18 / Guyana
NSP2:V469F	**2021-02-25**	2021-02-22 / SP	2021-02-22 / SP	2021-03-27 / Colombia
NSP3:A41V	2021-02-11	2021-02-11 / AM	2021-02-23 / SP	2021-04-11 / USA
NSP3:T133I	2020-12-29	2020-12-29 / AM	2021-01-03 / PA	2021-01-18 / Italy
NSP3:T186P	2020-12-23	2020-12-23 / AM	2021-01-21 / PR	2021-01-18 / Guyana
NSP3:A231V	**2021-03-01**	2021-02-22 / RJ	2021-02-22 / RJ	2021-06-07 / USA
NSP3:S370L	2020-12-03	2020-09-11 / SP	2020-09-11 / SP	2020-12-17 / Peru
NSP3:K977Q	2020-12-03	2020-09-11 / SP	2020-09-11 / SP	2020-12-17 / Peru
NSP3:T1189I	2020-12-23	2020-12-23 / AM	2021-01-21 / PR	2021-01-18 / Guyana
NSP3:T1365A	**2021-02-24**	**2021-02-24 / AM**	2021-03-23 / SP	2021-06-04 / Mexico
NSP3:S1437F	**2021-03-01**	**2021-03-01 / AM**	2021-03-25 / RJ	2021-06-21 / Mexico
NSP3:S1670F	**2021-03-08**	**2021-03-08 / AM**	2021-03-17 / SP	2021-03-17 / Peru
NSP4:V30A	**2021-02-23**	**2021-02-23 / AM**	--	--
NSP4:T83I	2020-12-23	2020-12-23 / AM	2021-01-07 / PB	2021-01-06 / Colombia
NSP4:H313Y	**2021-02-26**	2021-02-19 / PA	2021-02-19 / PA	2021-02-20 / USA
NSP4:S481L	**2021-02-23**	**2021-02-23 / AM**	2021-03-31 / RJ	2021-03-18 / Canada
NSP5:A70V	**2021-02-23**	**2021-02-23 / AM**	2021-03-25 / RJ	2021-04-19 / Argentina
NSP5:V86I	**2021-02-23**	2021-02-22 / TO	2021-02-22 / TO	--
NSP5:P241L	2021-01-07	2021-01-07 / AM	2021-01-28 / AP	2021-01-22 / Italy
NSP6:A46V	**2021-03-01**	2021-02-19 / SC	2021-02-19 / SC	2021-05-09 / Colombia
NSP6:A51V	**2021-02-23**	**2021-02-23 / AM**	2021-03-04 / GO	2021-04-27 / USA
NSP6:S106del	2020-12-03	2020-09-11 / SP	2020-09-11 / SP	2020-11-11 / USA
NSP6:G107S	2020-12-21	2020-12-21 / AM	2021-01-11 / RJ	2021-01-04 / Italy
NSP6:G107del	2020-12-03	2020-09-11 / SP	2020-09-11 / SP	2020-11-11 / USA
NSP6:F108L	2020-12-03	2020-09-11 / SP	2020-09-11 / SP	2020-11-11 / USA
NSP6:F108del	2020-12-21	2020-12-21 / AM	2021-01-11 / RJ	2020-12-17 / Peru
NSP6:V149A	2020-12-23	2020-12-23 / AM	2021-01-21 / PR	2021-01-18 / Guyana
NSP8:E155G	**2021-02-23**	**2021-02-23 / AM**	2021-03-05 / PR	2021-04-22 / USA
NSP12:P323L	2020-12-03	2020-09-11 / SP	2020-09-11 / SP	2020-11-11 / USA
NSP12:I548V	2021-01-04	2021-01-04 / AM	2021-01-27 / AC	2021-03-04 / Ireland
NSP12:Q822H	**2021-03-01**	2021-02-01 / RN	2021-02-01 / RN	2021-01-23 / Venezuela
NSP13:S74L	2020-12-23	2020-12-23 / AM	2021-01-21 / PR	2021-01-18 / Guyana
NSP13:M274I	**2021-03-01**	**2021-03-01 / AM**	2021-04-19 / SP	2021-04-16 / Colombia
NSP13:E341D	2020-12-03	2020-09-11 / SP	2020-09-11 / SP	2020-12-17 / Peru
NSP13:L581F	**2021-02-22**	2021-02-11 / RJ	2021-02-11 / RJ	2021-03-26 / USA
NSP14:P158H	**2021-02-23**	**2021-02-23 / AM**	--	2021-06-28 / Chile

NSP15:D39Y	**2021-02-23**	**2021-02-23 / AM**	2021-03-23 / SP	2021-03-08 / Switzerland
NSP15:E170D	**2021-02-22**	**2021-02-22 / AM**	--	2021-05-14 / USA
NSP16:K160R	**2021-02-22**	**2021-02-22 / AM**	2021-02-22 / PA	2021-01-04 / Italy
NS3:P25L	**2021-02-25**	2021-01-27 / AL	2021-01-27 / AL	2021-03-30 / Germany
NS3:P104S	**2021-02-22**	**2021-02-22 / AM**	2021-03-21 / GO	2021-03-29 / USA
NS3:S117I	**2021-02-22**	2021-01-02 / PR	2021-01-02 / PR	--
NS3:W131C	2021-01-19	2021-01-19 / AM	2021-03-08 / RS	2021-04-09 / USA
NS3:E181V	**2021-02-25**	**2021-02-25 / AM**	--	--
NS3:S253P	2020-12-03	2020-09-11 / SP	2020-09-11 / SP	2020-12-17 / Peru
NS7a:E22D	2021-02-02	2021-02-02 / AM	2021-02-23 / CE	2021-04-14 / USA
NS7b:S31L	2021-02-21	2021-02-21 / AM	2021-05-06 / MS	2021-03-31 / USA
NS8:E59D	2021-02-21	2021-02-21 / AM	2021-03-04 / CE	--
NS8:E92K	2020-12-03	2020-09-11 / SP	2020-09-11 / SP	2021-04-07 / USA

Only complete collection dates were considered for this analysis. Collection dates related to genomes from this study are labeled in bold. AC: Acre, AL: Alagoas, AM: Amazonas, AP: Amapá, CE: Ceará, GO: Goiás, MS: Mato Grosso do Sul, PB: Paraíba, PR: Paraná, SC: Santa Catarina, SE: Sergipe, SP: São Paulo, RJ: Rio de Janeiro, RN: Rio Grande do Norte, RS: Rio Grande do Sul, TO: Tocantins. Date verification on September 24, 2021. *According to the GISAID database there are three P.1 genomes from the USA carrying these mutations with collection dates from april and may, 2020 (before its first occurrence in Brazil), however we do not find any publication confirming this USA origin of P.1 lineage.
