# Supplementary material for: Comparative Genomics and Characterization of SARS-CoV-2 P.1 (Gamma) Variant of Concern From Amazonas, Brazil
Source: Front Med (Lausanne). 2022 Feb 15;9:806611. doi: 10.3389/fmed.2022.806611 (PMC8885995; doi:10.3389/fmed.2022.806611)
Supplement: Supplementary File 5 — Sites under adaptive or purifying pressure according to the HyPhy tests FUBAR, FEL, MEME, and SLAC. [file Data_Sheet_5.PDF]

SPIKE

FEL

Codon	alpha	beta	LRT	Selection detected?
4	6.741	0.000	3.571	Neg. p = 0.0588
5	0.000	7.119	2.805	Pos. p = 0.0940
11	4.037	0.000	3.268	Neg. p = 0.0706
32	6.982	0.000	5.919	Neg. p = 0.0150
43	4.605	0.000	3.940	Neg. p = 0.0471
66	6.694	0.000	3.139	Neg. p = 0.0764
84	5.302	0.000	3.026	Neg. p = 0.0820
130	6.097	0.000	4.921	Neg. p = 0.0265
138	0.000	13.185	3.371	Pos. p = 0.0664
168	6.694	0.000	3.578	Neg. p = 0.0585
170	6.694	0.000	2.751	Neg. p = 0.0972
198	13.486	0.000	5.844	Neg. p = 0.0156
224	23.025	0.000	4.176	Neg. p = 0.0410
287	6.741	0.000	2.919	Neg. p = 0.0876
291	6.788	0.000	3.485	Neg. p = 0.0619
293	8.465	0.000	5.612	Neg. p = 0.0178
295	4.369	0.000	2.836	Neg. p = 0.0922
296	8.080	0.000	7.085	Neg. p = 0.0078
300	17.587	0.000	6.824	Neg. p = 0.0090
306	4.645	0.000	3.943	Neg. p = 0.0471
313	6.741	0.000	2.753	Neg. p = 0.0971
351	6.694	0.000	2.753	Neg. p = 0.0971
364	6.741	0.000	2.926	Neg. p = 0.0872
369	6.694	0.000	2.753	Neg. p = 0.0971
375	4.025	0.000	3.424	Neg. p = 0.0643
392	6.694	0.000	3.573	Neg. p = 0.0587
395	4.028	0.000	3.273	Neg. p = 0.0704
413	10.812	0.000	7.648	Neg. p = 0.0057
417	0.000	7.902	3.469	Pos. p = 0.0625
421	6.694	0.000	2.753	Neg. p = 0.0971
432	6.788	0.000	5.515	Neg. p = 0.0189
441	8.756	0.000	5.617	Neg. p = 0.0178
469	10.392	0.000	3.947	Neg. p = 0.0470
491	4.333	0.000	2.835	Neg. p = 0.0922
507	10.465	0.000	4.277	Neg. p = 0.0386
518	5.320	0.000	3.024	Neg. p = 0.0821
562	4.605	0.000	3.939	Neg. p = 0.0472
578	6.694	0.000	2.919	Neg. p = 0.0876
618	10.328	0.000	3.645	Neg. p = 0.0562
673	6.694	0.000	3.077	Neg. p = 0.0794
680	4.333	0.000	2.736	Neg. p = 0.0981
681	0.000	7.119	2.777	Pos. p = 0.0956

SLAC

Codon	S	N	dS	dN	Selection detected?
32	3.000	0.000	3.575	0.000	Neg. p = 0.022
43	2.000	0.000	2.382	0.000	Neg. p = 0.078
130	3.000	0.000	3.000	0.000	Neg. p = 0.037
146	3.000	1.000	3.576	0.463	Neg. p = 0.069
198	2.000	0.000	3.005	0.000	Neg. p = 0.049
296	4.000	0.000	4.000	0.000	Neg. p = 0.012
300	2.000	0.000	5.077	0.000	Neg. p = 0.019
306	2.000	0.000	2.382	0.000	Neg. p = 0.078
354	3.000	1.000	3.576	0.463	Neg. p = 0.069
413	4.000	0.000	4.000	0.000	Neg. p = 0.012
432	3.000	0.000	3.573	0.000	Neg. p = 0.024
543	3.000	1.000	3.573	0.463	Neg. p = 0.069
562	2.000	0.000	2.381	0.000	Neg. p = 0.078
682	3.000	0.000	2.707	0.000	Neg. p = 0.050
692	3.000	1.000	3.171	0.487	Neg. p = 0.096
707	5.000	0.000	5.958	0.000	Neg. p = 0.002
821	5.000	0.000	2.722	0.000	Neg. p = 0.086
856	2.000	0.000	2.382	0.000	Neg. p = 0.078
936	2.000	0.000	2.382	0.000	Neg. p = 0.078
960	2.000	0.000	2.384	0.000	Neg. p = 0.078
1018	2.000	0.000	2.109	0.000	Neg. p = 0.100
1101	2.000	0.000	2.382	0.000	Neg. p = 0.078
1178	6.000	0.000	7.161	0.000	Neg. p = 0.000
1215	3.000	0.000	3.575	0.000	Neg. p = 0.026

Significant sites at p <= 0.1

MEME

Codon	alpha	beta+	p+	LRT	Episodic selection detected?	# branches	Most common codon substitutions at this site
138	0.000	13.028	1.000	3.371	Yes, p = 0.0879	6	[4]TAT>GAT | [2]GAT>TAT
417	0.000	7.889	0.990	3.471	Yes, p = 0.0834	4	[2]ACG>AAG | [1]AAG>ACG,ACG>ATG

Significant sites at p <= 0.1

682	6.982	0.000	5.473	Neg. p = 0.0193
707	11.629	0.000	7.037	Neg. p = 0.0080
718	6.694	0.000	3.578	Neg. p = 0.0585
743	6.694	0.000	3.477	Neg. p = 0.0622
756	6.694	0.000	2.751	Neg. p = 0.0972
793	10.392	0.000	4.265	Neg. p = 0.0389
803	10.262	0.000	3.935	Neg. p = 0.0473
817	6.694	0.000	3.576	Neg. p = 0.0586
818	5.935	0.000	2.907	Neg. p = 0.0882
819	23.146	0.000	4.177	Neg. p = 0.0410
821	13.347	0.000	7.579	Neg. p = 0.0059
881	10.392	0.000	3.653	Neg. p = 0.0560
893	10.392	0.000	4.068	Neg. p = 0.0437
897	20.554	0.000	8.491	Neg. p = 0.0036
934	5.935	0.000	2.907	Neg. p = 0.0882
936	4.605	0.000	2.978	Neg. p = 0.0844
948	4.273	0.000	2.804	Neg. p = 0.0940
972	10.392	0.000	4.080	Neg. p = 0.0434
979	6.694	0.000	2.916	Neg. p = 0.0877
988	8.684	0.000	3.419	Neg. p = 0.0645
1018	4.154	0.000	2.859	Neg. p = 0.0908
1030	10.392	0.000	3.947	Neg. p = 0.0470
1034	4.333	0.000	2.807	Neg. p = 0.0939
1047	6.694	0.000	2.753	Neg. p = 0.0971
1055	20.205	0.000	7.888	Neg. p = 0.0050
1067	6.694	0.000	2.753	Neg. p = 0.0971
1070	10.262	0.000	4.068	Neg. p = 0.0437
1087	10.262	0.000	4.068	Neg. p = 0.0437
1101	4.617	0.000	3.293	Neg. p = 0.0696
1122	5.278	0.000	3.919	Neg. p = 0.0477
1129	10.262	0.000	4.032	Neg. p = 0.0446
1136	10.262	0.000	3.641	Neg. p = 0.0564
1159	6.694	0.000	3.144	Neg. p = 0.0762
1175	10.324	0.000	3.931	Neg. p = 0.0474
1178	13.995	0.000	6.911	Neg. p = 0.0086
1196	4.333	0.000	2.736	Neg. p = 0.0981
1213	10.245	0.000	4.261	Neg. p = 0.0390
1215	6.788	0.000	4.225	Neg. p = 0.0398
1239	6.694	0.000	3.077	Neg. p = 0.0794
1264	0.000	4.771	2.827	Pos. p = 0.0927

Significant sites at p <= 0.1

NSP1

FEL				
Codon	alpha	beta	LRT	Selection detected?
5	0	6.771	3.021	Pos. p = 0.0822
36	11.333	0	2.814	Neg. p = 0.0934
42	8.352	0	2.914	Neg. p = 0.0878
74	6.097	0	2.999	Neg. p = 0.0833
156	31.027	3.885	5.935	Neg. p = 0.0148

Significant sites at p <= 0.1

SLAC					
Codon	S	N	dS	dN	Selection detected?
156	8.000	1.000	9.188	0.470	Neg. p = 0.000

Significant sites at p <= 0.1

MEME							
Codon	alpha	beta+	p+	LRT	Episodic selection detected?	# branches	Most common codon substitutions at this site

No sites found at p <= 0.1

NSP2

FEL				
Codon	alpha	beta	LRT	Selection detected?
86	4.698	0	3.263	Neg. p = 0.0709
136	0	5.146	2.988	Pos. p = 0.0839
165	0	6.407	3.718	Pos. p = 0.0538
205	4.223	0	2.899	Neg. p = 0.0886
207	7.056	0	3.392	Neg. p = 0.0655
256	10.891	0	3.749	Neg. p = 0.0528
339	0	9.624	3.236	Pos. p = 0.0720
347	18.887	0	3.710	Neg. p = 0.0541
369	13.010	0	9.759	Neg. p = 0.0018
414	10.812	0	3.644	Neg. p = 0.0563
446	4.210	0	3.602	Neg. p = 0.0577
447	0	4.804	3.045	Pos. p = 0.0810
473	11.034	0	3.856	Neg. p = 0.0496
483	18.887	0	3.079	Neg. p = 0.0793
569	10.922	0	3.757	Neg. p = 0.0526
597	10.934	0	4.300	Neg. p = 0.0381
636	18.887	0	3.705	Neg. p = 0.0543

Significant sites at p <= 0.1

SLAC					
Codon	S	N	dS	dN	Selection detected?
8	2.000	0	2.253	0	Neg. p = 0.088
40	2.000	0	2.253	0	Neg. p = 0.088
86	2.000	0	2.254	0	Neg. p = 0.088
207	3.000	0	3.381	0	Neg. p = 0.029
369	6.000	0	6.000	0	Neg. p = 0.001
414	2.000	0	2.359	0	Neg. p = 0.080
606	2.000	0	2.257	0	Neg. p = 0.087

Significant sites at p <= 0.1

MEME							
Codon	alpha	beta+	p+	LRT	Episodic selection detected?	# branches	Most common codon substitutions at this site
165	0	6.341	0.990	3.718	Yes, p = 0.0733	2	[1]GGC>AGC,GGC>GAC
339	0	10.038	0.960	3.236	Yes, p = 0.0943	3	[2]GGT>AGT | [1]GGT>GAT
447	0	1407.863	0.005	11.733	Yes, p = 0.0012	1	[1]GTC>TCC
458	0	790.947	0.006	3.376	Yes, p = 0.0876	1	[1]GGT>GCT

Significant sites at p <= 0.1

NSP3

FEL

Codon	alpha	beta	LRT	Selection detected?
5	10.392	0	5.360	Neg. p = 0.0206
21	7.464	0	4.384	Neg. p = 0.0363
27	5.357	0	3.056	Neg. p = 0.0804
48	11.754	0	3.918	Neg. p = 0.0478
100	11.754	0	4.133	Neg. p = 0.0420
106	7.212	0	3.417	Neg. p = 0.0645
146	5.473	0	4.319	Neg. p = 0.0377
231	0	8.571	2.794	Pos. p = 0.0946
266	34.200	0	4.801	Neg. p = 0.0284
268	31.517	0	6.458	Neg. p = 0.0110
300	7.401	0	5.447	Neg. p = 0.0196
329	11.754	0	4.490	Neg. p = 0.0341
348	11.754	0	3.970	Neg. p = 0.0463
368	5.357	0	3.057	Neg. p = 0.0804
422	11.754	0	3.929	Neg. p = 0.0474
446	7.238	0	3.027	Neg. p = 0.0819
472	5.428	0	2.712	Neg. p = 0.0996
497	15.762	0	3.282	Neg. p = 0.0700
504	11.754	0	3.916	Neg. p = 0.0478
512	7.464	0	5.206	Neg. p = 0.0225
524	11.754	0	4.122	Neg. p = 0.0423
527	11.148	0	6.575	Neg. p = 0.0103
528	5.357	0	3.058	Neg. p = 0.0804
538	7.401	0	3.792	Neg. p = 0.0515
564	11.754	0	4.110	Neg. p = 0.0426
597	7.466	0	4.382	Neg. p = 0.0363
613	11.754	0	3.970	Neg. p = 0.0463
618	0	7.272	4.236	Pos. p = 0.0396
620	5.357	0	3.056	Neg. p = 0.0804
631	11.754	0	4.490	Neg. p = 0.0341
665	5.402	0	2.712	Neg. p = 0.0996
680	15.762	0	3.258	Neg. p = 0.0711
685	15.762	0	3.259	Neg. p = 0.0710
692	3.663	0	3.040	Neg. p = 0.0813
707	43.945	1.881	3.704	Neg. p = 0.0543
727	0	4.230	2.955	Pos. p = 0.0856
827	6.097	0	2.907	Neg. p = 0.0882
850	15.762	0	3.282	Neg. p = 0.0701
885	6.097	0	2.907	Neg. p = 0.0882
903	11.754	0	3.918	Neg. p = 0.0478
955	11.754	0	3.905	Neg. p = 0.0482
970	11.257	1.929	2.792	Neg. p = 0.0947
973	15.762	0	3.277	Neg. p = 0.0702

SLAC

Codon	S	N	dS	dN	Selection detected?
146	3.000	0.000	3.000	0.000	Neg. p = 0.037
262	2.000	0.000	2.150	0.000	Neg. p = 0.096
268	2.000	0.000	2.528	0.000	Neg. p = 0.088
300	4.000	0.000	4.000	0.000	Neg. p = 0.012
356	3.000	1.000	3.225	0.500	Neg. p = 0.097
455	2.000	0.000	2.152	0.000	Neg. p = 0.096
527	3.000	0.000	3.000	0.000	Neg. p = 0.037
621	2.000	0.000	2.150	0.000	Neg. p = 0.096
736	2.000	0.000	2.152	0.000	Neg. p = 0.096
760	2.000	0.000	2.150	0.000	Neg. p = 0.096
855	3.000	0.000	3.226	0.000	Neg. p = 0.030
931	2.000	0.000	2.150	0.000	Neg. p = 0.096
953	0.000	2.000	0.000	0.667	Neg. p = 1.000
1031	3.000	0.000	3.226	0.000	Neg. p = 0.030
1038	3.000	0.000	3.000	0.000	Neg. p = 0.037
1089	2.000	0.000	2.150	0.000	Neg. p = 0.096
1099	2.000	0.000	2.150	0.000	Neg. p = 0.096
1104	2.000	0.000	2.150	0.000	Neg. p = 0.096
1107	3.000	1.000	3.230	0.483	Neg. p = 0.091
1117	4.000	0.000	4.055	0.000	Neg. p = 0.012
1228	0.000	7.000	0.000	3.519	Pos. p = 0.056
1238	3.000	0.000	3.000	0.000	Neg. p = 0.037
1298	3.000	0.000	3.000	0.000	Neg. p = 0.037
1329	3.000	0.000	3.226	0.000	Neg. p = 0.030
1354	3.000	0.000	3.226	0.000	Neg. p = 0.030
1516	2.000	0.000	2.150	0.000	Neg. p = 0.096
1567	3.000	0.000	3.042	0.000	Neg. p = 0.036
1603	6.000	0.000	6.000	0.000	Neg. p = 0.001
1705	2.000	0.000	2.150	0.000	Neg. p = 0.096
1713	2.000	0.000	2.150	0.000	Neg. p = 0.096
1739	3.000	0.000	3.000	0.000	Neg. p = 0.037
1820	4.000	0.000	3.788	0.000	Neg. p = 0.015
1858	3.000	0.000	3.000	0.000	Neg. p = 0.037
1860	2.000	0.000	2.150	0.000	Neg. p = 0.096
1875	2.000	0.000	2.150	0.000	Neg. p = 0.096
1891	2.000	0.000	2.406	0.000	Neg. p = 0.077

Significant sites at p <= 0.1

MEME

Codon	alpha	beta+	p+	LRT	Episodic selection detected?	# branches	Most common codon substitutions at this site
618	0.000	7.275	0.990	4.237	Yes, p = 0.0560	2	[2]AAC>AGC

Significant sites at p <= 0.1

1024	15.762	0	3.283	Neg. p = 0.0700
1031	5.829	0	3.005	Neg. p = 0.0830
1038	5.647	0	4.656	Neg. p = 0.0310
1060	15.762	0	3.283	Neg. p = 0.0700
1065	15.762	0	3.281	Neg. p = 0.0701
1089	3.890	0	3.115	Neg. p = 0.0776
1117	7.401	0	5.049	Neg. p = 0.0246
1118	15.762	0	3.283	Neg. p = 0.0700
1159	3.629	0	3.341	Neg. p = 0.0676
1207	7.215	0	3.422	Neg. p = 0.0643
1211	0	11.198	2.737	Pos. p = 0.0981
1222	3.646	0	2.887	Neg. p = 0.0893
1235	15.762	0	3.259	Neg. p = 0.0710
1238	5.580	0	4.055	Neg. p = 0.0440
1249	15.791	0	3.257	Neg. p = 0.0711
1298	14.732	0	7.731	Neg. p = 0.0054
1299	13.165	0	5.889	Neg. p = 0.0152
1303	0	3.871	2.899	Pos. p = 0.0886
1354	5.795	0	4.679	Neg. p = 0.0305
1368	23.920	0	7.858	Neg. p = 0.0051
1440	0	4.296	3.118	Pos. p = 0.0774
1470	7.277	0	3.207	Neg. p = 0.0733
1487	15.762	0	3.259	Neg. p = 0.0710
1491	6.694	0	2.976	Neg. p = 0.0845
1494	11.754	0	4.137	Neg. p = 0.0419
1497	15.762	0	3.279	Neg. p = 0.0702
1516	3.890	0	3.118	Neg. p = 0.0774
1519	7.277	0	3.419	Neg. p = 0.0644
1544	5.357	0	2.940	Neg. p = 0.0864
1558	7.464	0	5.201	Neg. p = 0.0226
1567	5.529	0	3.791	Neg. p = 0.0515
1569	7.186	0	3.414	Neg. p = 0.0646
1572	7.215	0	3.422	Neg. p = 0.0643
1603	11.034	0	8.168	Neg. p = 0.0043
1634	7.186	0	3.205	Neg. p = 0.0734
1665	5.357	0	3.156	Neg. p = 0.0757
1728	15.762	0	3.282	Neg. p = 0.0700
1739	11.148	0	6.900	Neg. p = 0.0086
1799	15.762	0	3.259	Neg. p = 0.0710
1807	3.649	0	3.039	Neg. p = 0.0813
1820	13.654	0	8.737	Neg. p = 0.0031
1858	5.473	0	3.986	Neg. p = 0.0459
1891	13.679	0	5.999	Neg. p = 0.0143
1914	5.357	0	2.826	Neg. p = 0.0928
1915	5.357	0	2.826	Neg. p = 0.0928

Significant sites at p <= 0.1

NSP4

FEL				
Codon	alpha	beta	LRT	Selection detected?
69	7.402	0.000	2.887	Neg. p = 0.0893
76	8.670	0.049	3.715	Neg. p = 0.0539
121	5.487	0.000	2.847	Neg. p = 0.0916
145	8.670	0.000	2.899	Neg. p = 0.0886
154	7.402	0.000	2.887	Neg. p = 0.0893
213	14.541	0.000	3.671	Neg. p = 0.0554
214	14.409	0.000	3.893	Neg. p = 0.0485
228	18.887	0.000	3.768	Neg. p = 0.0522
229	14.541	0.000	3.834	Neg. p = 0.0502
252	14.523	0.000	4.473	Neg. p = 0.0344
258	14.541	0.000	3.671	Neg. p = 0.0554
298	5.170	0.000	3.026	Neg. p = 0.0820
363	8.251	0.000	2.718	Neg. p = 0.0992
390	5.484	0.000	2.855	Neg. p = 0.0911
413	21.710	0.000	3.685	Neg. p = 0.0549
456	14.702	0.000	3.253	Neg. p = 0.0713
475	5.170	0.000	3.315	Neg. p = 0.0686
489	14.418	0.000	4.465	Neg. p = 0.0346

Significant sites at p <= 0.1

SLAC					
Codon	S	N	dS	dN	Selection detected?
76	3.000	0.000	3.227	0.000	Neg. p = 0.030
121	2.000	0.000	2.150	0.000	Neg. p = 0.096
390	2.000	0.000	2.151	0.000	Neg. p = 0.096

Significant sites at p <= 0.1

MEME							
Codon	alpha	beta+	p+	LRT	Episodic selection detected?	# branches	Most common codon substitutions at this site

No sites found at p <= 0.1

NSP5

FEL

Codon	alpha	beta	LRT	Selection detected?
3	6.426	0.000	3.204	Neg. p = 0.0735
18	11.754	0.000	3.784	Neg. p = 0.0517
24	11.754	0.000	3.972	Neg. p = 0.0463
55	17.723	0.000	3.553	Neg. p = 0.0594
105	3.464	0.000	2.741	Neg. p = 0.0978
132	3.464	0.000	3.291	Neg. p = 0.0697
134	3.649	0.000	2.982	Neg. p = 0.0842
178	17.723	0.000	3.553	Neg. p = 0.0594
194	5.233	0.000	2.743	Neg. p = 0.0977
226	5.293	0.000	2.712	Neg. p = 0.0996
269	17.723	0.000	3.665	Neg. p = 0.0556
272	5.491	0.000	2.918	Neg. p = 0.0876
285	5.083	0.000	2.718	Neg. p = 0.0992
293	5.205	0.000	3.123	Neg. p = 0.0772
304	5.293	0.000	2.712	Neg. p = 0.0996

Significant sites at p <= 0.1

SLAC

Codon	S	N	dS	dN	Selection detected?
151	3.000	0	3.162	0	Neg. p = 0.032

Significant sites at p <= 0.1

MEME

Codon	alpha	beta+	p+	LRT	Episodic selection detected?	# branches	Most common codon substitutions at this site

No sites found at p <= 0.1

NSP6

FEL				
Codon	alpha	beta	LRT	Selection detected?
118	7.536	0.000	3.556	Neg. p = 0.0593
120	7.400	0.000	3.184	Neg. p = 0.0743
149	0.000	7.689	2.739	Pos. p = 0.0980
200	4.238	0.000	2.984	Neg. p = 0.0841
262	12.358	0.000	12.426	Neg. p = 0.0004
270	17.723	0.000	5.302	Neg. p = 0.0213
284	18.001	0.000	10.773	Neg. p = 0.0010
286	7.627	0.000	3.203	Neg. p = 0.0735

Significant sites at p <= 0.1

SLAC					
Codon	S	N	dS	dN	Selection detected?
200	2.000	0.000	2.140	0.000	Neg. p = 0.097
262	6.000	0.000	6.000	0.000	Neg. p = 0.001
270	2.000	0.000	2.409	0.000	Neg. p = 0.078
284	8.000	0.000	8.259	0.000	Neg. p = 0.000

Significant sites at p <= 0.1

MEME							
Codon	alpha	beta+	p+	LRT	Episodic selection detected?	# branches	Most common codon substitutions at this site
106	0.000	2198.575	0.021	4.790	Yes, p = 0.0420	1	[1]TCT>ACT,TCT>TNT
194	0.020	216.548	0.015	7.676	Yes, p = 0.0096	1	[1]GTT>ACT

Significant sites at p <= 0.1

NSP7

FEL				
Codon	alpha	beta	LRT	Selection detected?
No sites found at p <= 0.1				

SLAC					
Codon	S	N	dS	dN	Selection detected?
No sites found at p <= 0.1					

MEME							
Codon	alpha	beta+	p+	LRT	Episodic selection detected?	# branches	Most common codon substitutions at this site
No sites found at p <= 0.1							

NSP8

FEL				
Codon	alpha	beta	LRT	Selection detected?
18	7.186	0	2.861	Neg. p = 0.0908
91	7.186	0	3.166	Neg. p = 0.0752
111	13.785	0	3.219	Neg. p = 0.0728
121	7.401	0	3.245	Neg. p = 0.0717
137	11.754	0	3.567	Neg. p = 0.0590
162	11.754	0	3.681	Neg. p = 0.0550
183	7.186	0	3.209	Neg. p = 0.0732

Significant sites at p <= 0.1

SLAC					
Codon	S	N	dS	dN	Selection detected?

No sites found at p <= 0.1

MEME							
Codon	alpha	beta+	p+	LRT	Episodic selection detected?	# branches	Most common codon substitutions at this site

No sites found at p <= 0.1

NSP9

FEL				
Codon	alpha	beta	LRT	Selection detected?
6	3.886	0	2.989	Neg. p = 0.0838
28	4.605	0	2.739	Neg. p = 0.0979
31	8.729	0	5.680	Neg. p = 0.0172
33	0	4.341	2.850	Pos. p = 0.0914
42	8.077	0	6.241	Neg. p = 0.0125
68	18.887	0	3.519	Neg. p = 0.0607
93	9.695	0	2.781	Neg. p = 0.0954
103	3.906	0	3.104	Neg. p = 0.0781
112	3.951	0	2.900	Neg. p = 0.0886

Significant sites at p <= 0.1

SLAC					
Codon	S	N	dS	dN	Selection detected?
31	4.000	0.000	4.366	0.000	Neg. p = 0.010
95	3.000	0.000	3.087	0.000	Neg. p = 0.034

Significant sites at p <= 0.1

MEME							
Codon	alpha	beta+	p+	LRT	Episodic selection detected?	# branches	Most common codon substitutions at this site
47	0.010	987.683	0.020	8.976	Yes, p = 0.0049	1	[1]GAT>TTT

Significant sites at p <= 0.1

NSP10

FEL

Codon	alpha	beta	LRT	Selection detected?
30	9.738	0	2.853	Neg. p = 0.0912
59	8.624	0	3.993	Neg. p = 0.0457
64	9.738	0	2.762	Neg. p = 0.0965
65	32.365	0	3.516	Neg. p = 0.0608
66	30.699	0	3.907	Neg. p = 0.0481
83	9.738	0	2.993	Neg. p = 0.0836

Significant sites at p <= 0.1

SLAC

Codon	S	N	dS	dN	Selection detected?

No sites found at p <= 0.1

MEME

Codon	alpha	beta+	p+	LRT	Episodic selection detected?	# branches	Most common codon substitutions at this site

No sites found at p <= 0.1
