# Supplementary material for: Comparative Genomics and Characterization of SARS-CoV-2 P.1 (Gamma) Variant of Concern From Amazonas, Brazil
Source: Front Med (Lausanne). 2022 Feb 15;9:806611. doi: 10.3389/fmed.2022.806611 (PMC8885995; doi:10.3389/fmed.2022.806611)
Supplement: Supplementary File 6 — Authors acknowledgement lists for GISAID genomes used in the genomic and phylogenomic analyses. [file Data_Sheet_6.PDF]

We gratefully acknowledge the following Authors from the Originating laboratories responsible for obtaining the specimens, as well as the Submitting laboratories where the genome data were generated and shared via GISAID, on which this research is based.

All Submitters of data may be contacted directly via www.gisaid.org

Authors are sorted alphabetically.

Accession ID	Originating Laboratory	Submitting Laboratory	Authors
EPI_ISL_1061031, EPI_ISL_1061032	CDL Laboratorio Santos e Vidal LTDA.	Instituto de Medicina Tropical de Sao Paulo	Brazil-UK Centre for Arbovirus Discovery Diagnosis Genomics and Epidemiology (CADDE) Genomic Network - Instituto de Medicina Tropical
EPI_ISL_833167, EPI_ISL_833168, EPI_ISL_833169, EPI_ISL_833170, EPI_ISL_833171, EPI_ISL_833172, EPI_ISL_833173, EPI_ISL_833174, EPI_ISL_833175, EPI_ISL_833176			
see above	DB Diagnosticos do Brasil	Instituto Adolfo Lutz, Interdiciplinary Procedures Center, Strategic Laboratory	Claudia Regina Gonçalves; Claudio Tavares Sacchi; Erica Valessa Ramos Gomes; Karoline Rodrigues Campos
EPI_ISL_1708318, EPI_ISL_2499748	Fundação Hospitalar de Hematologia e Hemoterapia do Amazonas	Laboratório de Estudos de Vírus Emergentes	Adriana S. S. Duarte; Alessandro S. Farias; Arilson Bernardo S. P. Gomes; Audrey B. Zangirolami; Bruno D. Benites; Camila A.M. Silva; Camila L. Simeoni; Camila Lopes Simeoni; Carolina Costa-Lima; Cecilia C. Camilo; Chieh-Hsi Wu; Christopher Dye; Clarice W. Arns; Daniel A. Toledo-Teixeira; Daniel Augusto de Toledo-Teixeira; Darlan S. Candido; Erika R. Manuli; Esmenia C. Rocha; Ester C. Sabino; Ester Cerdeira Sabino; Fabia; Fabiana Granja; Fernando R. Spilki; Flavia C. Sales; Gabriela Fabiano de Souza; Giulia M. Ferreira; Grazielle C. Maktura; Henrique Marques-Souza; Ingra M. Claro; Jaqueline G. de Jesus; José Luiz Proença Módena; Julia Forato; Karina Bispo dos Santos; Karina Bispo-dos-Santos; Lais D. Coimbra; Lais Durço Coimbra; Leandro M. Souza; Lucas A.M. Franco; Lucas I. Buscaratti; Luciana S. Mofatto; Luciana Souto Mofatto; Magnun N.N. Santos; Marcelo A.S. Mori; Marcelo Addas-Carvalho; Maria L. Moretti; Maria Luiza Moretti; Mariana C. Pinho; Mariana S. Ramundo; Mariene R. Amorim; Mariene Ribeiro Amorim; Michael S. Diamond; Myuki A.E. Crispim; Natalia S. Brunetti; Nelson Gaburo; Nuno R. Faria; Nuno Rodrigues Faria; Oliver G. Pybus; Pamela S. Andrade; Pierina L. Parise; Pierina Lorencini Parise; Priscilla P. Barbosa; Priscilla Paschoal Barbosa; Rafael E. Marques; Renata Sesti-Costa; Rodrigo N. Angerami; Rodrigo Nogueira Angerami; Stefanie Primon Muraro; Thais M. Coletti; Vitor A. Costa; William M. Souza; William Marciel de Souza
EPI_ISL_3149997, EPI_ISL_3149998, EPI_ISL_3149999, EPI_ISL_3150000, EPI_ISL_3150001, EPI_ISL_3150002, EPI_ISL_3150003, EPI_ISL_3150004, EPI_ISL_3150005, EPI_ISL_3150006, EPI_ISL_3150007, EPI_ISL_3150008, EPI_ISL_3150009, EPI_ISL_3150010, EPI_ISL_3150011, EPI_ISL_3150012, EPI_ISL_3150013, EPI_ISL_3150014, EPI_ISL_3150015, EPI_ISL_3150016, EPI_ISL_3150017			
see above	Hermes Pardini	Universidade Federal de Ciencias da Saude de Porto Alegre	Andrea Roberto de Souza; Andy Goren; Carlos Gustavo Wambier; Claudia Elizabeth Thompson; Daniel do Nascimento Fonseca; Emilyn Oliveira Guerreiro; Flávio Adsuara Cadegiani; Liane Nanci Rotta; Patrícia Aline Gröhs Ferrareze; Ricardo Ariel Zimerman; Zhihua Ren
EPI_ISL_906080, EPI_ISL_906081	Hospital Beneficiencia Portuguesa	Instituto Adolfo Lutz, Interdiciplinary Procedures Center, Strategic Laboratory	Claudia Regina Gonçalves; Claudio Tavares Sacchi; Erica Valessa Ramos Gomes; Karoline Rodrigues Campos
EPI_ISL_940626, EPI_ISL_940627	Hospital Central Sao Caetano do Sul	Instituto Adolfo Lutz, Interdiciplinary Procedures Center, Strategic Laboratory	Claudia Regina Gonçalves; Claudio Tavares Sacchi; Erica Valessa Ramos Gomes; Karoline Rodrigues Campos
EPI_ISL_906075	Hospital Geral de Vila Penteado Dr Jose Pangella Sao Paulo	Instituto Adolfo Lutz, Interdiciplinary Procedures Center, Strategic Laboratory	Claudia Regina Gonçalves; Claudio Tavares Sacchi; Erica Valessa Ramos Gomes; Karoline Rodrigues Campos
EPI_ISL_940620, EPI_ISL_940623, EPI_ISL_940624	Hospital Sao Joaquim - Beneficiencia Portuguesa	Instituto Adolfo Lutz, Interdiciplinary Procedures Center, Strategic Laboratory	Claudia Regina Gonçalves; Claudio Tavares Sacchi; Erica Valessa Ramos Gomes; Karoline Rodrigues Campos
EPI_ISL_906076, EPI_ISL_906077	Hospital Sao Luiz Sao Caetano	Instituto Adolfo Lutz, Interdiciplinary Procedures Center, Strategic Laboratory	Claudia Regina Gonçalves; Claudio Tavares Sacchi; Erica Valessa Ramos Gomes; Karoline Rodrigues Campos
EPI_ISL_906069	Instituto Adolfo Lutz - Regional de Campinas	Instituto Adolfo Lutz, Interdiciplinary Procedures Center, Strategic Laboratory	Claudia Regina Gonçalves; Claudio Tavares Sacchi; Erica Valessa Ramos Gomes; Karoline Rodrigues Campos
EPI_ISL_925846, EPI_ISL_925916, EPI_ISL_926446	LACEN - Laboratório Central de Saúde Pública do Amazonas	Evandro Chagas Institute Virology	A.M.; Barbagelata; E.C.; E.M.A.; Ferreira; J.A.; Junior; K.C.; L.C.; L.S.; M.C.; P.S.; Pinheiro; Santos; Silva; Sousa; Sousa Junior; W.D.C.; da Silva
EPI_ISL_918499, EPI_ISL_918500, EPI_ISL_918501, EPI_ISL_918502, EPI_ISL_918503, EPI_ISL_918504, EPI_ISL_918505, EPI_ISL_918506, EPI_ISL_918507, EPI_ISL_918508, EPI_ISL_918509, EPI_ISL_918510, EPI_ISL_918511, EPI_ISL_918512, EPI_ISL_918531, EPI_ISL_918532, EPI_ISL_918533, EPI_ISL_918534, EPI_ISL_918535, EPI_ISL_1261683, EPI_ISL_1261685, EPI_ISL_1261690, EPI_ISL_1261694, EPI_ISL_2488774, EPI_ISL_2488775			
see above	LACEN - Laboratório Central de Saúde Pública do Amazonas	Evandro Chagas Institute	A.M.; Barbagelata; E.C.; E.M.A.; Ferreira; J.A.; Junior; K.C.; L.C.; L.S.; M.C.; P.S.; Pinheiro; Santos; Silva; Sousa; Sousa Junior; W.D.C.; da Silva
EPI_ISL_1166615	LACEN - Laboratório Central de Saúde Pública do Rio Grande do Norte	Evandro Chagas Institute Virology	A.M.; Barbagelata; E.C.; E.M.A.; Ferreira; J.A.; Junior; K.C.; L.C.; L.S.; M.C.; P.S.; Pinheiro; Santos; Silva; Sousa; Sousa Junior; W.D.C.; da Silva
EPI_ISL_943990	LACEN do Estado de Goias	Instituto Adolfo Lutz, Interdiciplinary Procedures Center, Strategic Laboratory	Claudia Regina Gonçalves; Claudio Tavares Sacchi; Erica Valessa Ramos Gomes; Karoline Rodrigues Campos
EPI_ISL_906071, EPI_ISL_940614, EPI_ISL_940615, EPI_ISL_940616, EPI_ISL_940617, EPI_ISL_940618	LACEN-PI DR. Costa Alvarenga	Instituto Adolfo Lutz, Interdiciplinary Procedures Center, Strategic Laboratory	Claudia Regina Gonçalves; Claudio Tavares Sacchi; Erica Valessa Ramos Gomes; Karoline Rodrigues Campos
EPI_ISL_1213190, EPI_ISL_1213202, EPI_ISL_1213204, EPI_ISL_1213206	LBM/UFPB	Bioinformatics Laboratory / LNCC	Alessandra P Lamarca; Alexandra L Gerber; Ana Paula Melo Mariano; Ana Paula de C Guimarães; Ana Tereza R Vasconcelos; Angela Maria Guimarães Santos; Bianca Mendes Maciel; Danielle Angst Secco; Eduardo Sérgio Soares Sousa; Eloiza Helena Campana; Francisco Paulo Freire Neto; George Rego Albuquerque; Kátia Castanho Scortecci; Lucymara Fassarella Agnez Lima; Luiz G P de Almeida; Luís Cristóvão Porto; Otavio J. Brustolini; Paulo Ricardo Nascimento; Ronaldo da Silva Francisco Jr; Sandra Rocha Gadelha; Selma Maria Bezerra Jeronimo; Vinícius Pietta Perez
EPI_ISL_4030305, EPI_ISL_4030306, EPI_ISL_4030311, EPI_ISL_4030312, EPI_ISL_4030313, EPI_ISL_4030318, EPI_ISL_4030320, EPI_ISL_4030321, EPI_ISL_4030322, EPI_ISL_4030323, EPI_ISL_4030368, EPI_ISL_4030369, EPI_ISL_4030370, EPI_ISL_4030371, EPI_ISL_4030372, EPI_ISL_4030373, EPI_ISL_4030374, EPI_ISL_4030375, EPI_ISL_4030376, EPI_ISL_4030377, EPI_ISL_4030378, EPI_ISL_4030379, EPI_ISL_4030380, EPI_ISL_4030381, EPI_ISL_4030382			
see above	Laboratorio Central de Saude Publica do Amazonas - LACEN-AM	Laboratorio de Ecologia de Doencas Transmissiveis na Amazonia, Instituto Leonidas e Maria Deane - Fiocruz Amazonia	André Corado; Debora Duarte; Felipe Naveca; Fernanda Nascimento; George Silva; Karina Pessoa; Luciana Gonçalves; Maria Júlia Brandão; Matilde Mejía; Michele Jesus; Valdinete Nascimento; Victor Souza; Ágatha Costa
EPI_ISL_417034, EPI_ISL_792560, EPI_ISL_801386, EPI_ISL_801387, EPI_ISL_801388, EPI_ISL_801389, EPI_ISL_801390, EPI_ISL_801391, EPI_ISL_801392, EPI_ISL_801393, EPI_ISL_801394, EPI_ISL_801395, EPI_ISL_801396, EPI_ISL_811148, EPI_ISL_811149, EPI_ISL_833131, EPI_ISL_833132, EPI_ISL_833133, EPI_ISL_833134, EPI_ISL_833135, EPI_ISL_833136, EPI_ISL_833137, EPI_ISL_833138, EPI_ISL_833139, EPI_ISL_833140, EPI_ISL_1034304, EPI_ISL_1034305, EPI_ISL_1034306, EPI_ISL_1068078, EPI_ISL_1068079, EPI_ISL_1068080, EPI_ISL_1068081, EPI_ISL_1068082, EPI_ISL_1068083, EPI_ISL_1068084, EPI_ISL_1068085, EPI_ISL_1068086, EPI_ISL_1068087, EPI_ISL_1068088, EPI_ISL_1068089, EPI_ISL_1068090, EPI_ISL_1068091, EPI_ISL_1068092, EPI_ISL_1068093, EPI_ISL_1068094, EPI_ISL_1068095, EPI_ISL_1068096, EPI_ISL_1068097, EPI_ISL_1068098, EPI_ISL_1068099, EPI_ISL_1068100, EPI_ISL_1068101, EPI_ISL_1068102, EPI_ISL_1068103, EPI_ISL_1068104, EPI_ISL_1068105, EPI_ISL_1068106, EPI_ISL_1068107, EPI_ISL_1068108, EPI_ISL_1068109, EPI_ISL_1068110, EPI_ISL_1068111, EPI_ISL_1068112, EPI_ISL_1068113, EPI_ISL_1068114, EPI_ISL_1068115, EPI_ISL_1068117, EPI_ISL_1068118, EPI_ISL_1068119, EPI_ISL_1068120, EPI_ISL_1068121, EPI_ISL_1068122, EPI_ISL_1068123, EPI_ISL_1068124, EPI_ISL_1068125, EPI_ISL_1068126, EPI_ISL_1068127, EPI_ISL_1068128, EPI_ISL_1068129, EPI_ISL_1068130, EPI_ISL_1068131, EPI_ISL_1068132, EPI_ISL_1068133, EPI_ISL_1068134, EPI_ISL_1068135, EPI_ISL_1068136, EPI_ISL_1068137, EPI_ISL_1068138, EPI_ISL_1068139, EPI_ISL_1068140, EPI_ISL_1068141, EPI_ISL_1068142, EPI_ISL_1068143, EPI_ISL_1068144, EPI_ISL_1068145, EPI_ISL_1068146, EPI_ISL_1068147, EPI_ISL_1068148, EPI_ISL_1068149, EPI_ISL_1068150, EPI_ISL_1068151, EPI_ISL_1068152, EPI_ISL_1068153, EPI_ISL_1068154, EPI_ISL_1068155, EPI_ISL_1068156, EPI_ISL_1068157, EPI_ISL_1068158, EPI_ISL_1068159, EPI_ISL_1068160, EPI_ISL_1068161, EPI_ISL_1068162, EPI_ISL_1068163, EPI_ISL_1068164, EPI_ISL_1068165, EPI_ISL_1068166, EPI_ISL_1068167, EPI_ISL_1068168, EPI_ISL_1068169, EPI_ISL_1068170, EPI_ISL_1068171, EPI_ISL_1068172, EPI_ISL_1068173, EPI_ISL_1068174, EPI_ISL_1068175, EPI_ISL_1068176, EPI_ISL_1068177, EPI_ISL_1068178, EPI_ISL_1068179, EPI_ISL_1068180, EPI_ISL_1068181, EPI_ISL_1068182, EPI_ISL_1068183, EPI_ISL_1068184, EPI_ISL_1068185, EPI_ISL_1068186, EPI_ISL_1068187, EPI_ISL_1068188, EPI_ISL_1068189, EPI_ISL_1068190, EPI_ISL_1068191, EPI_ISL_1068192, EPI_ISL_1068193, EPI_ISL_1068194, EPI_ISL_1068195, EPI_ISL_1068196, EPI_ISL_1068197, EPI_ISL_1068198, EPI_ISL_1068201, EPI_ISL_1068203, EPI_ISL_1068204, EPI_ISL_1068205, EPI_ISL_1068206, EPI_ISL_1068207, EPI_ISL_1068208, EPI_ISL_1068209, EPI_ISL_1068210, EPI_ISL_1068211, EPI_ISL_1068212, EPI_ISL_1068213, EPI_ISL_1068214, EPI_ISL_1068215, EPI_ISL_1068216, EPI_ISL_1068217, EPI_ISL_1068218, EPI_ISL_1068219, EPI_ISL_1068221, EPI_ISL_1068222, EPI_ISL_1068223, EPI_ISL_1068224, EPI_ISL_1068225, EPI_ISL_1068226, EPI_ISL_1068227, EPI_ISL_1068228, EPI_ISL_1068229, EPI_ISL_1068230, EPI_ISL_1068231, EPI_ISL_1068232, EPI_ISL_1068233, EPI_ISL_1068234, EPI_ISL_1068235, EPI_ISL_1068236, EPI_ISL_1068237, EPI_ISL_1068238, EPI_ISL_1068239, EPI_ISL_1068240, EPI_ISL_1068241, EPI_ISL_1068242, EPI_ISL_1068243, EPI_ISL_1068244, EPI_ISL_1068245, EPI_ISL_1068246, EPI_ISL_1068247, EPI_ISL_1068248, EPI_ISL_1068249, EPI_ISL_1068250, EPI_ISL_1068251, EPI_ISL_1068252, EPI_ISL_1068253, EPI_ISL_1068254, EPI_ISL_1068255, EPI_ISL_1068256, EPI_ISL_1068257, EPI_ISL_1068258, EPI_ISL_1068259, EPI_ISL_1068260, EPI_ISL_1068261, EPI_ISL_1068262, EPI_ISL_1068263, EPI_ISL_1068264, EPI_ISL_1068265, EPI_ISL_1068266, EPI_ISL_1068268, EPI_ISL_1068269, EPI_ISL_1068270, EPI_ISL_1068271, EPI_ISL_1068272, EPI_ISL_1068273, EPI_ISL_1068274, EPI_ISL_1068275, EPI_ISL_1068276, EPI_ISL_1068277, EPI_ISL_1068278, EPI_ISL_1068279, EPI_ISL_1068280, EPI_ISL_1068281, EPI_ISL_1068282, EPI_ISL_1068283, EPI_ISL_1068284, EPI_ISL_1068285, EPI_ISL_1068286, EPI_ISL_1068287, EPI_ISL_1068288, EPI_ISL_1068289, EPI_ISL_1068290, EPI_ISL_1068291, EPI_ISL_1068292, EPI_ISL_1261122, EPI_ISL_1261123, EPI_ISL_1533609, EPI_ISL_1661250, EPI_ISL_1661251, EPI_ISL_1661252, EPI_ISL_2777360, EPI_ISL_2777361, EPI_ISL_2777362, EPI_ISL_2777363, EPI_ISL_2777364, EPI_ISL_2777365, EPI_ISL_2777366, EPI_ISL_2777367, EPI_ISL_2777368, EPI_ISL_2777369, EPI_ISL_2777370, EPI_ISL_2777371, EPI_ISL_2777372, EPI_ISL_2777373, EPI_ISL_2777374, EPI_ISL_2777375, EPI_ISL_2777376, EPI_ISL_2777377, EPI_ISL_2777378, EPI_ISL_2777379, EPI_ISL_2777380, EPI_ISL_2777381, EPI_ISL_2777382, EPI_ISL_2777383, EPI_ISL_2777384, EPI_ISL_2777385, EPI_ISL_2777386, EPI_ISL_2777387, EPI_ISL_2777388, EPI_ISL_2777389, EPI_ISL_2777390, EPI_ISL_2777391, EPI_ISL_2777392, EPI_ISL_2777393, EPI_ISL_2777394, EPI_ISL_2777395, EPI_ISL_2777396, EPI_ISL_2777397, EPI_ISL_2777398, EPI_ISL_2777399, EPI_ISL_2777400, EPI_ISL_2777401, EPI_ISL_2777402, EPI_ISL_2777403, EPI_ISL_2777404, EPI_ISL_2777405, EPI_ISL_2777406, EPI_ISL_2777407, EPI_ISL_2777408, EPI_ISL_2777409, EPI_ISL_2777410, EPI_ISL_2777411, EPI_ISL_2777412, EPI_ISL_2777413, EPI_ISL_2777414, EPI_ISL_2777415, EPI_ISL_2777416, EPI_ISL_2777417, EPI_ISL_2777418, EPI_ISL_2777419, EPI_ISL_2777420, EPI_ISL_2777421, EPI_ISL_2777423, EPI_ISL_2777424, EPI_ISL_2777425, EPI_ISL_2777426, EPI_ISL_2777427, EPI_ISL_2777428, EPI_ISL_2777429, EPI_ISL_2777430, EPI_ISL_2777431, EPI_ISL_2777432, EPI_ISL_2777433, EPI_ISL_2777436, EPI_ISL_2777437, EPI_ISL_2777438, EPI_ISL_2777439, EPI_ISL_2777440, EPI_ISL_2777441, EPI_ISL_2777442, EPI_ISL_2777443, EPI_ISL_2777444, EPI_ISL_2777445, EPI_ISL_2777446, EPI_ISL_2777447, EPI_ISL_2777448, EPI_ISL_2777449, EPI_ISL_2777450, EPI_ISL_2777451, EPI_ISL_2777452, EPI_ISL_2777453, EPI_ISL_2777454, EPI_ISL_2777455, EPI_ISL_2777456, EPI_ISL_2777457, EPI_ISL_2777458, EPI_ISL_2777459, EPI_ISL_2777460, EPI_ISL_2777461, EPI_ISL_2777462, EPI_ISL_2777463, EPI_ISL_2777464, EPI_ISL_2777465, EPI_ISL_2777466, EPI_ISL_2777467, EPI_ISL_2777468, EPI_ISL_2777469, EPI_ISL_2777470, EPI_ISL_2777471, EPI_ISL_2777472, EPI_ISL_2777473, EPI_ISL_2777474, EPI_ISL_2777475, EPI_ISL_2777476, EPI_ISL_2777477, EPI_ISL_2777478, EPI_ISL_2777479, EPI_ISL_2777480, EPI_ISL_2777481, EPI_ISL_2777482, EPI_ISL_2777483, EPI_ISL_2777484, EPI_ISL_2777485, EPI_ISL_2777486, EPI_ISL_2777487, EPI_ISL_2777488, EPI_ISL_2777489, EPI_ISL_2777490, EPI_ISL_2777491, EPI_ISL_2777492, EPI_ISL_2777493, EPI_ISL_2777494, EPI_ISL_2777995,			

EPI_ISL_2777996, EPI_ISL_2777997, EPI_ISL_2777998, EPI_ISL_2777999, EPI_ISL_2778000, EPI_ISL_2778001, EPI_ISL_2778003, EPI_ISL_3050307, EPI_ISL_3050457, EPI_ISL_3050515, EPI_ISL_3050516, EPI_ISL_3050517, EPI_ISL_3050574, EPI_ISL_3050591, EPI_ISL_3050615, EPI_ISL_3050616, EPI_ISL_3050617, EPI_ISL_3050618, EPI_ISL_3050641, EPI_ISL_4030308, EPI_ISL_4030309, EPI_ISL_4030310, EPI_ISL_4030314, EPI_ISL_4030315, EPI_ISL_4030316, EPI_ISL_4030317, EPI_ISL_4030319, EPI_ISL_4030324, EPI_ISL_4030325, EPI_ISL_4030326, EPI_ISL_4030327, EPI_ISL_4030328, EPI_ISL_4030329, EPI_ISL_4030330, EPI_ISL_4030331, EPI_ISL_4030332, EPI_ISL_4030333, EPI_ISL_4030334, EPI_ISL_4030335, EPI_ISL_4030336, EPI_ISL_4030337, EPI_ISL_4030338, EPI_ISL_4030339, EPI_ISL_4030340, EPI_ISL_4030341, EPI_ISL_4030342, EPI_ISL_4030343, EPI_ISL_4030344, EPI_ISL_4030345, EPI_ISL_4030346, EPI_ISL_4030347, EPI_ISL_4030348, EPI_ISL_4030349, EPI_ISL_4030350, EPI_ISL_4030351, EPI_ISL_4030352, EPI_ISL_4030353, EPI_ISL_4030354, EPI_ISL_4030355, EPI_ISL_4030356, EPI_ISL_4030357, EPI_ISL_4030358, EPI_ISL_4030359, EPI_ISL_4030360, EPI_ISL_4030361, EPI_ISL_4030362, EPI_ISL_4030363, EPI_ISL_4030364, EPI_ISL_4030365, EPI_ISL_4030366, EPI_ISL_4030367			
see above	Laboratorio de Ecologia de Doencas Transmissiveis na Amazonia, Instituto Leonidas e Maria Deane - Fiocruz Amazonia	Laboratorio de Ecologia de Doencas Transmissiveis na Amazonia, Instituto Leonidas e Maria Deane - Fiocruz Amazonia	Alex Martins; André Corado; Debora Duarte; Fabiola Mendonça da Silva Chui; Felipe Naveca; Felipe Naveca on behalf of the Fiocruz COVID-19 Genomic Surveillance Network; Fernanda Nascimento; Fernando Fonseca de Almeida Val; George Silva; Gisely Cardoso de Melo; Karina Pessoa; Luciana Gonçalves; Marcus Vinicius Guimarães de Lacerda; Maria Júlia Brandão; Maria Paula Gomes Mourão; Mariana Xavier Simão; Matilde Mejía; Michele Jesus; Sérgio Luz; Valdinete Nascimento; Vanderson de Souza Sampaio; Victor Souza; Ágatha Costa
EPI_ISL_1402431	Laboratory of Respiratory Viruses and Measles, Oswaldo Cruz Institute, FIOCRUZ	Laboratory of Respiratory Viruses and Measles, Oswaldo Cruz Institute, FIOCRUZ	Alex Pauvolid-Correa; Alice Sampaio Rocha; Ana Beatriz Machado Lima; Ana Carolina Mendonca; Anna Carolina Paixao; Felipe Naveca; Fernando Motta; Luciana Appolinario; Marilda Siqueira on behalf of the Fiocruz COVID-19 Genomic Surveillance Network; Mia Ferreira Araujo; Paola Resende; Renata Serrano Lopes
EPI_ISL_2241532, EPI_ISL_2241605	Laboratório Central de Saúde Pública de Pernambuco	Coordenação Geral de Laboratórios de Saúde Pública (CGLAB/DAEVS/SVS/MS)	Vagner Fonseca; et al.
EPI_ISL_2298850, EPI_ISL_2298852, EPI_ISL_2298854, EPI_ISL_2298856, EPI_ISL_2298859, EPI_ISL_2298861, EPI_ISL_2298863, EPI_ISL_2298865, EPI_ISL_2298867, EPI_ISL_2298869, EPI_ISL_2298871			
see above	Laboratório Central de Saúde Pública do Amazonas	Coordenação Geral de Laboratórios de Saúde Pública (CGLAB/DAEVS/SVS/MS)	Vagner Fonseca; et al.
EPI_ISL_2777236, EPI_ISL_2777237, EPI_ISL_2777238, EPI_ISL_2777239, EPI_ISL_2777240, EPI_ISL_2777241, EPI_ISL_2777242, EPI_ISL_2777243, EPI_ISL_2777244, EPI_ISL_2777245, EPI_ISL_2777246, EPI_ISL_2777247, EPI_ISL_2777248, EPI_ISL_2777249, EPI_ISL_2777250, EPI_ISL_2777251, EPI_ISL_2777252, EPI_ISL_2777253, EPI_ISL_2777254, EPI_ISL_2777255, EPI_ISL_2777256, EPI_ISL_2777257, EPI_ISL_2777258, EPI_ISL_2777259, EPI_ISL_2777260, EPI_ISL_2777261, EPI_ISL_2777262, EPI_ISL_2777263, EPI_ISL_2777264, EPI_ISL_2777265, EPI_ISL_2777266, EPI_ISL_2777267, EPI_ISL_2777268, EPI_ISL_2777269, EPI_ISL_2777270, EPI_ISL_2777271, EPI_ISL_2777272, EPI_ISL_2777273, EPI_ISL_2777274, EPI_ISL_2777275, EPI_ISL_2777276, EPI_ISL_2777277, EPI_ISL_2777278, EPI_ISL_2777279, EPI_ISL_2777280, EPI_ISL_2777281, EPI_ISL_2777282, EPI_ISL_2777283, EPI_ISL_2777284, EPI_ISL_2777285, EPI_ISL_2777287, EPI_ISL_2777288, EPI_ISL_2777290, EPI_ISL_2777292, EPI_ISL_2777293, EPI_ISL_2777295, EPI_ISL_2777297, EPI_ISL_2777299, EPI_ISL_2777301, EPI_ISL_2777302, EPI_ISL_2777304, EPI_ISL_2777306, EPI_ISL_2777309, EPI_ISL_2777311, EPI_ISL_2777313, EPI_ISL_2777314, EPI_ISL_2777316, EPI_ISL_2777318, EPI_ISL_2777320, EPI_ISL_2777321, EPI_ISL_2777324, EPI_ISL_2777327, EPI_ISL_2777328, EPI_ISL_2777329, EPI_ISL_2777330, EPI_ISL_2777331, EPI_ISL_2777332, EPI_ISL_2777333, EPI_ISL_2777334, EPI_ISL_2777335, EPI_ISL_2777337, EPI_ISL_2777338, EPI_ISL_2777339, EPI_ISL_2777340, EPI_ISL_2777341, EPI_ISL_2777342, EPI_ISL_2777343, EPI_ISL_2777344, EPI_ISL_2777345, EPI_ISL_2777346, EPI_ISL_2777347, EPI_ISL_2777348, EPI_ISL_2777349, EPI_ISL_2777350, EPI_ISL_2777351, EPI_ISL_2777352, EPI_ISL_2777353, EPI_ISL_2777354, EPI_ISL_2777355, EPI_ISL_2777356, EPI_ISL_2777357, EPI_ISL_2777358, EPI_ISL_2777359, EPI_ISL_2777495, EPI_ISL_2777496, EPI_ISL_2777497, EPI_ISL_2777498, EPI_ISL_2777499, EPI_ISL_2777500, EPI_ISL_2777501, EPI_ISL_2777502, EPI_ISL_2777503, EPI_ISL_2777504, EPI_ISL_2777505, EPI_ISL_2777506, EPI_ISL_2777507, EPI_ISL_2777508, EPI_ISL_2777509, EPI_ISL_2777510, EPI_ISL_2777511, EPI_ISL_2777512, EPI_ISL_2777513, EPI_ISL_2777514, EPI_ISL_2777515, EPI_ISL_2777516, EPI_ISL_2777517, EPI_ISL_2777518, EPI_ISL_2777519, EPI_ISL_2777520, EPI_ISL_2777521, EPI_ISL_2777522, EPI_ISL_2777523, EPI_ISL_2777524, EPI_ISL_2777525, EPI_ISL_2777526, EPI_ISL_2777527, EPI_ISL_2777528, EPI_ISL_2777529, EPI_ISL_2777530, EPI_ISL_2777531, EPI_ISL_2777532, EPI_ISL_2777533, EPI_ISL_2777534, EPI_ISL_2777535, EPI_ISL_2777536, EPI_ISL_2777537, EPI_ISL_2777538, EPI_ISL_2777539, EPI_ISL_2777540, EPI_ISL_2777541, EPI_ISL_2777542, EPI_ISL_2777543, EPI_ISL_2777544, EPI_ISL_2777545, EPI_ISL_2777546, EPI_ISL_2777547, EPI_ISL_2777548, EPI_ISL_2777549, EPI_ISL_2777550, EPI_ISL_2777551, EPI_ISL_2777552, EPI_ISL_2777553, EPI_ISL_2777554, EPI_ISL_2777555, EPI_ISL_2777556, EPI_ISL_2777557, EPI_ISL_2777558, EPI_ISL_2777559, EPI_ISL_2777560, EPI_ISL_2777561, EPI_ISL_2777562, EPI_ISL_2777563, EPI_ISL_2777564, EPI_ISL_2777565, EPI_ISL_2777566, EPI_ISL_2777567, EPI_ISL_2777568, EPI_ISL_2777569, EPI_ISL_2777570, EPI_ISL_2777571, EPI_ISL_2777572, EPI_ISL_2777573, EPI_ISL_2777574, EPI_ISL_2777575, EPI_ISL_2777576, EPI_ISL_2777577, EPI_ISL_2777578, EPI_ISL_2777579, EPI_ISL_2777580, EPI_ISL_2777581, EPI_ISL_2777582, EPI_ISL_2777583, EPI_ISL_2777584, EPI_ISL_2777585, EPI_ISL_2777586, EPI_ISL_2777587, EPI_ISL_2777588, EPI_ISL_2777589, EPI_ISL_2777590, EPI_ISL_2777591, EPI_ISL_2777592, EPI_ISL_2777593, EPI_ISL_2777594, EPI_ISL_2777596, EPI_ISL_2777597, EPI_ISL_2777598, EPI_ISL_2777599, EPI_ISL_2777600, EPI_ISL_2777601, EPI_ISL_2777602, EPI_ISL_2777603, EPI_ISL_2777604, EPI_ISL_2777605, EPI_ISL_2777606, EPI_ISL_2777607, EPI_ISL_2777608, EPI_ISL_2777609, EPI_ISL_2777610, EPI_ISL_2777611, EPI_ISL_2777612, EPI_ISL_2777613, EPI_ISL_2777614, EPI_ISL_2777615, EPI_ISL_2777616, EPI_ISL_2777617, EPI_ISL_2777618, EPI_ISL_2777619, EPI_ISL_2777620, EPI_ISL_2777621, EPI_ISL_2777622, EPI_ISL_2777623, EPI_ISL_2777624, EPI_ISL_2777625, EPI_ISL_2777626, EPI_ISL_2777627, EPI_ISL_2777628, EPI_ISL_2777629, EPI_ISL_2777630, EPI_ISL_2777631, EPI_ISL_2777632, EPI_ISL_2777633, EPI_ISL_2777634, EPI_ISL_2777635, EPI_ISL_2777636, EPI_ISL_2777637, EPI_ISL_2777638, EPI_ISL_2777639, EPI_ISL_2777640, EPI_ISL_2777641, EPI_ISL_2777642, EPI_ISL_2777643, EPI_ISL_2777644, EPI_ISL_2777645, EPI_ISL_2777646, EPI_ISL_2777648, EPI_ISL_2777649, EPI_ISL_2777650, EPI_ISL_2777651, EPI_ISL_2777652, EPI_ISL_2777653, EPI_ISL_2777654, EPI_ISL_2777655, EPI_ISL_2777656, EPI_ISL_2777657, EPI_ISL_2777658, EPI_ISL_2777659, EPI_ISL_2777660, EPI_ISL_2777661, EPI_ISL_2777662, EPI_ISL_2777663, EPI_ISL_2777664, EPI_ISL_2777665, EPI_ISL_2777666, EPI_ISL_2777667, EPI_ISL_2777668, EPI_ISL_2777669, EPI_ISL_2777670, EPI_ISL_2777671, EPI_ISL_2777672, EPI_ISL_2777673, EPI_ISL_2777674, EPI_ISL_2777675, EPI_ISL_2777676, EPI_ISL_2777677, EPI_ISL_2777678, EPI_ISL_2777679, EPI_ISL_2777680, EPI_ISL_2777681, EPI_ISL_2777682, EPI_ISL_2777683, EPI_ISL_2777684, EPI_ISL_2777685, EPI_ISL_2777686, EPI_ISL_2777687, EPI_ISL_2777688, EPI_ISL_2777689, EPI_ISL_2777690, EPI_ISL_2777691, EPI_ISL_2777692, EPI_ISL_2777693, EPI_ISL_2777694, EPI_ISL_2777695, EPI_ISL_2777696, EPI_ISL_2777697, EPI_ISL_2777698, EPI_ISL_2777699, EPI_ISL_2777700, EPI_ISL_2777701, EPI_ISL_2777702, EPI_ISL_2777703, EPI_ISL_2777704, EPI_ISL_2777705, EPI_ISL_2777706, EPI_ISL_2777707, EPI_ISL_2777708, EPI_ISL_2777709, EPI_ISL_2777710, EPI_ISL_2777711, EPI_ISL_2777712, EPI_ISL_2777713, EPI_ISL_2777714, EPI_ISL_2777715, EPI_ISL_2777716, EPI_ISL_2777717, EPI_ISL_2777718, EPI_ISL_2777719, EPI_ISL_2777720, EPI_ISL_2777721, EPI_ISL_2777722, EPI_ISL_2777723, EPI_ISL_2777724, EPI_ISL_2777725, EPI_ISL_2777726, EPI_ISL_2777727, EPI_ISL_2777728, EPI_ISL_2777729, EPI_ISL_2777730, EPI_ISL_2777731, EPI_ISL_2777732, EPI_ISL_2777733, EPI_ISL_2777734, EPI_ISL_2777735, EPI_ISL_2777736, EPI_ISL_2777737, EPI_ISL_2777738, EPI_ISL_2777739, EPI_ISL_2777740, EPI_ISL_2777741, EPI_ISL_2777742, EPI_ISL_2777743, EPI_ISL_2777744, EPI_ISL_2777745, EPI_ISL_2777746, EPI_ISL_2777747, EPI_ISL_2777748, EPI_ISL_2777749, EPI_ISL_2777750, EPI_ISL_2777751, EPI_ISL_2777752, EPI_ISL_2777753, EPI_ISL_2777754, EPI_ISL_2777755, EPI_ISL_2777756, EPI_ISL_2777757, EPI_ISL_2777758, EPI_ISL_2777759, EPI_ISL_2777760, EPI_ISL_2777761, EPI_ISL_2777762, EPI_ISL_2777763, EPI_ISL_2777764, EPI_ISL_2777765, EPI_ISL_2777766, EPI_ISL_2777767, EPI_ISL_2777768, EPI_ISL_2777769, EPI_ISL_2777770, EPI_ISL_2777771, EPI_ISL_2777772, EPI_ISL_2777773, EPI_ISL_2777774, EPI_ISL_2777775, EPI_ISL_2777776, EPI_ISL_2777777, EPI_ISL_2777778, EPI_ISL_2777779, EPI_ISL_2777780, EPI_ISL_2777781, EPI_ISL_2777782, EPI_ISL_2777783, EPI_ISL_2777784, EPI_ISL_2777785, EPI_ISL_2777786, EPI_ISL_2777787, EPI_ISL_2777788, EPI_ISL_2777789, EPI_ISL_2777790, EPI_ISL_2777791, EPI_ISL_2777792, EPI_ISL_2777793, EPI_ISL_2777794, EPI_ISL_2777795, EPI_ISL_2777796, EPI_ISL_2777797, EPI_ISL_2777798, EPI_ISL_2777799, EPI_ISL_2777800, EPI_ISL_2777801, EPI_ISL_2777802, EPI_ISL_2777803, EPI_ISL_2777804, EPI_ISL_2777805, EPI_ISL_2777806, EPI_ISL_2777807, EPI_ISL_2777808, EPI_ISL_2777809, EPI_ISL_2777810, EPI_ISL_2777811, EPI_ISL_2777812, EPI_ISL_2777813, EPI_ISL_2777814, EPI_ISL_2777815, EPI_ISL_2777816, EPI_ISL_2777817, EPI_ISL_2777818, EPI_ISL_2777819, EPI_ISL_2777820, EPI_ISL_2777821, EPI_ISL_2777822, EPI_ISL_2777823, EPI_ISL_2777824, EPI_ISL_2777825, EPI_ISL_2777826, EPI_ISL_2777827, EPI_ISL_2777828, EPI_ISL_2777829, EPI_ISL_2777830, EPI_ISL_2777831, EPI_ISL_2777832, EPI_ISL_2777833, EPI_ISL_2777834, EPI_ISL_2777835, EPI_ISL_2777836, EPI_ISL_2777837, EPI_ISL_2777838, EPI_ISL_2777839, EPI_ISL_2777840, EPI_ISL_2777841, EPI_ISL_2777842, EPI_ISL_2777843, EPI_ISL_2777844, EPI_ISL_2777845, EPI_ISL_2777846, EPI_ISL_2777847, EPI_ISL_2777848, EPI_ISL_2777849, EPI_ISL_2777850, EPI_ISL_2777851, EPI_ISL_2777852, EPI_ISL_2777853, EPI_ISL_2777854, EPI_ISL_2777855, EPI_ISL_2777856, EPI_ISL_2777857, EPI_ISL_2777858, EPI_ISL_2777859, EPI_ISL_2777860, EPI_ISL_2777861, EPI_ISL_2777862, EPI_ISL_2777863, EPI_ISL_2777864, EPI_ISL_2777865, EPI_ISL_2777866, EPI_ISL_2777867, EPI_ISL_2777868, EPI_ISL_2777869, EPI_ISL_2777870, EPI_ISL_2777871, EPI_ISL_2777872, EPI_ISL_2777873, EPI_ISL_2777874, EPI_ISL_2777875, EPI_ISL_2777876, EPI_ISL_2777877, EPI_ISL_2777878, EPI_ISL_2777879, EPI_ISL_2777880, EPI_ISL_2777881, EPI_ISL_2777882, EPI_ISL_2777883, EPI_ISL_2777884, EPI_ISL_2777885, EPI_ISL_2777886, EPI_ISL_2777887, EPI_ISL_2777888, EPI_ISL_2777889, EPI_ISL_2777890, EPI_ISL_2777891, EPI_ISL_2777892, EPI_ISL_2777893, EPI_ISL_2777894, EPI_ISL_2777895, EPI_ISL_2777896, EPI_ISL_2777897, EPI_ISL_2777898, EPI_ISL_2777899, EPI_ISL_2777900, EPI_ISL_2777901, EPI_ISL_2777902, EPI_ISL_2777903, EPI_ISL_2777904, EPI_ISL_2777905, EPI_ISL_2777906, EPI_ISL_2777907, EPI_ISL_2777908, EPI_ISL_2777909, EPI_ISL_2777910, EPI_ISL_2777911, EPI_ISL_2777912, EPI_ISL_2777913, EPI_ISL_2777914, EPI_ISL_2777915, EPI_ISL_2777916, EPI_ISL_2777917, EPI_ISL_2777918, EPI_ISL_2777919, EPI_ISL_2777920, EPI_ISL_2777921, EPI_ISL_2777922, EPI_ISL_2777923, EPI_ISL_2777924, EPI_ISL_2777925, EPI_ISL_2777926, EPI_ISL_2777927, EPI_ISL_2777928, EPI_ISL_2777929, EPI_ISL_2777930, EPI_ISL_2777931, EPI_ISL_2777932, EPI_ISL_2777933, EPI_ISL_2777934, EPI_ISL_2777935, EPI_ISL_2777936, EPI_ISL_2777937, EPI_ISL_2777938, EPI_ISL_2777939, EPI_ISL_2777940, EPI_ISL_2777941, EPI_ISL_2777942, EPI_ISL_2777943, EPI_ISL_2777944, EPI_ISL_2777945, EPI_ISL_2777946, EPI_ISL_2777947, EPI_ISL_2777948, EPI_ISL_2777949, EPI_ISL_2777950, EPI_ISL_2777951, EPI_ISL_2777952, EPI_ISL_2777953, EPI_ISL_2777954, EPI_ISL_2777955, EPI_ISL_2777956, EPI_ISL_2777957, EPI_ISL_2777958, EPI_ISL_2777959, EPI_ISL_2777960, EPI_ISL_2777961, EPI_ISL_2777962, EPI_ISL_2777963, EPI_ISL_2777964, EPI_ISL_2777965, EPI_ISL_2777966, EPI_ISL_2777967, EPI_ISL_2777968, EPI_ISL_2777969, EPI_ISL_2777970, EPI_ISL_2777971, EPI_ISL_2777972, EPI_ISL_2777973, EPI_ISL_2777974, EPI_ISL_2777975, EPI_ISL_2777976, EPI_ISL_2777977, EPI_ISL_2777978, EPI_ISL_2777979, EPI_ISL_2777980, EPI_ISL_2777981, EPI_ISL_2777982, EPI_ISL_2777983, EPI_ISL_2777984, EPI_ISL_2777985, EPI_ISL_2777986, EPI_ISL_2777987, EPI_ISL_2777988, EPI_ISL_2777989, EPI_ISL_2777990, EPI_ISL_2777991, EPI_ISL_2777992, EPI_ISL_2777993, EPI_ISL_2777994, EPI_ISL_2778002, EPI_ISL_2778004, EPI_ISL_2778005, EPI_ISL_3050295, EPI_ISL_3050296, EPI_ISL_3050297, EPI_ISL_3050298, EPI_ISL_3050299, EPI_ISL_3050300, EPI_ISL_3050302, EPI_ISL_3050303, EPI_ISL_3050304, EPI_ISL_3050305, EPI_ISL_3050306, EPI_ISL_3050308, EPI_ISL_3050309, EPI_ISL_3050310, EPI_ISL_3050311, EPI_ISL_3050312, EPI_ISL_3050313, EPI_ISL_3050314, EPI_ISL_3050315, EPI_ISL_3050316, EPI_ISL_3050317, EPI_ISL_3050318, EPI_ISL_3050319, EPI_ISL_3050320, EPI_ISL_3050321, EPI_ISL_3050322, EPI_ISL_3050323, EPI_ISL_3050324, EPI_ISL_3050325, EPI_ISL_3050326, EPI_ISL_3050327, EPI_ISL_3050328, EPI_ISL_3050329, EPI_ISL_3050330, EPI_ISL_3050331, EPI_ISL_3050332, EPI_ISL_3050333, EPI_ISL_3050334, EPI_ISL_3050335, EPI_ISL_3050336, EPI_ISL_3050337, EPI_ISL_3050338, EPI_ISL_3050339, EPI_ISL_3050340, EPI_ISL_3050341, EPI_ISL_3050342, EPI_ISL_3050343, EPI_ISL_3050344, EPI_ISL_3050345, EPI_ISL_3050346, EPI_ISL_3050347, EPI_ISL_3050348, EPI_ISL_3050349, EPI_ISL_3050350, EPI_ISL_3050351, EPI_ISL_3050352, EPI_ISL_3050353, EPI_ISL_3050354, EPI_ISL_3050355, EPI_ISL_3050356, EPI_ISL_3050358, EPI_ISL_3050359, EPI_ISL_3050360, EPI_ISL_3050361, EPI_ISL_3050362, EPI_ISL_3050363, EPI_ISL_3050364, EPI_ISL_3050365, EPI_ISL_3050366, EPI_ISL_3050367, EPI_ISL_3050368, EPI_ISL_3050369, EPI_ISL_3050370, EPI_ISL_3050371, EPI_ISL_3050372, EPI_ISL_3050373, EPI_ISL_3050374, EPI_ISL_3050375, EPI_ISL_3050376, EPI_ISL_3050377, EPI_ISL_3050378, EPI_ISL_3050379, EPI_ISL_3050380, EPI_ISL_3050381, EPI_ISL_3050382, EPI_ISL_3050383, EPI_ISL_3050384, EPI_ISL_3050385, EPI_ISL_3050386, EPI_ISL_3050387, EPI_ISL_3050388, EPI_ISL_3050389, EPI_ISL_3050390, EPI_ISL_3050391, EPI_ISL_3050392, EPI_ISL_3050393, EPI_ISL_3050394, EPI_ISL_3050395, EPI_ISL_3050396, EPI_ISL_3050397, EPI_ISL_3050398, EPI_ISL_3050399, EPI_ISL_3050400, EPI_ISL_3050401, EPI_ISL_3050402, EPI_ISL_3050403, EPI_ISL_3050404, EPI_ISL_3050405, EPI_ISL_3050406, EPI_ISL_3050407, EPI_ISL_3050408, EPI_ISL_3050409, EPI_ISL_3050410, EPI_ISL_3050411, EPI_ISL_3050412, EPI_ISL_3050413, EPI_ISL_3050414, EPI_ISL_3050415, EPI_ISL_3050416, EPI_ISL_3050417, EPI_ISL_3050418, EPI_ISL_3050419, EPI_ISL_3050420, EPI_ISL_3050421, EPI_ISL_3050422, EPI_ISL_3050423, EPI_ISL_3050424, EPI_ISL_3050425, EPI_ISL_3050426, EPI_ISL_3050427, EPI_ISL_3050428, EPI_ISL_3050429, EPI_ISL_3050430, EPI_ISL_3050431, EPI_ISL_3050432, EPI_ISL_3050433, EPI_ISL_3050434, EPI_ISL_3050435, EPI_ISL_3050436, EPI_ISL_3050437, EPI_ISL_3050438, EPI_ISL_3050439, EPI_ISL_3050440, EPI_ISL_3050441, EPI_ISL_3050442, EPI_ISL_3050443, EPI_ISL_3050444, EPI_ISL_3050445, EPI_ISL_3050446, EPI_ISL_3050447, EPI_ISL_3050448, EPI_ISL_3050449, EPI_ISL_3050450, EPI_ISL_3050451, EPI_ISL_3050452, EPI_ISL_3050453, EPI_ISL_3050454, EPI_ISL_3050455, EPI_ISL_3050456, EPI_ISL_3050458, EPI_ISL_3050459, EPI_ISL_3050460, EPI_ISL_3050461, EPI_ISL_3050462, EPI_ISL_3050463, EPI_ISL_3050464, EPI_ISL_3050465, EPI_ISL_3050466, EPI_ISL_3050467, EPI_ISL_3050468, EPI_ISL_3050469, EPI_ISL_3050470, EPI_ISL_3050471, EPI_ISL_3050472, EPI_ISL_3050473, EPI_ISL_3050474, EPI_ISL_3050475, EPI_ISL_3050476, EPI_ISL_3050477, EPI_ISL_3050478, EPI_ISL_3050479, EPI_ISL_3050480, EPI_ISL_3050481, EPI_ISL_3050482, EPI_ISL_3050483, EPI_ISL_3050484, EPI_ISL_3050485, EPI_ISL_3050486, EPI_ISL_3050487, EPI_ISL_3050488, EPI_ISL_3050489, EPI_ISL_3050490, EPI_ISL_3050491, EPI_ISL_3050492, EPI_ISL_3050493, EPI_ISL_3050494, EPI_ISL_3050495, EPI_ISL_3050496, EPI_ISL_3050497, EPI_ISL_3050498, EPI_ISL_3050499, EPI_ISL_3050500, EPI_ISL_3050501, EPI_ISL_3050502, EPI_ISL_3050503, EPI_ISL_3050504, EPI_ISL_3050505, EPI_ISL_3050506, EPI_ISL_3050507, EPI_ISL_3050508, EPI_ISL_3050509, EPI_ISL_3050510, EPI_ISL_3050511, EPI_ISL_3050512, EPI_ISL_3050513, EPI_ISL_3050514, EPI_ISL_3050518, EPI_ISL_3050519, EPI_ISL_3050520, EPI_ISL_3050521, EPI_ISL_3050522, EPI_ISL_3050523, EPI_ISL_3050524, EPI_ISL_3050525, EPI_ISL_3050526, EPI_ISL_3050527, EPI_ISL_3050528, EPI_ISL_3050529, EPI_ISL_3050530, EPI_ISL_3050531, EPI_ISL_3050532, EPI_ISL_3050533, EPI_ISL_3050534, EPI_ISL_3050535, EPI_ISL_3050536, EPI_ISL_3050537, EPI_ISL_3050538, EPI_ISL_3050539, EPI_ISL_3050540, EPI_ISL_3050541, EPI_ISL_3050542, EPI_ISL_3050543, EPI_ISL_3050544, EPI_ISL_3050545, EPI_ISL_3050546, EPI_ISL_3050547, EPI_ISL_3050548, EPI_ISL_3050549, EPI_ISL_3050550, EPI_ISL_3050551, EPI_ISL_3050552, EPI_ISL_3050553, EPI_ISL_3050554, EPI_ISL_3050555, EPI_ISL_3050556, EPI_ISL_3050557, EPI_ISL_3050558, EPI_ISL_3050559, EPI_ISL_3050560, EPI_ISL_3050561, EPI_ISL_3050562, EPI_ISL_3050563, EPI_ISL_3050564, EPI_ISL_3050565, EPI_ISL_3050566, EPI_ISL_3050567, EPI_ISL_3050568, EPI_ISL_3050569, EPI_ISL_3050570, EPI_ISL_3050571, EPI_ISL_3050572, EPI_ISL_3050573, EPI_ISL_3050575, EPI_ISL_3050576, EPI_ISL_3050577, EPI_ISL_3050578, EPI_ISL_3050579, EPI_ISL_3050580, EPI_ISL_3050581, EPI_ISL_3050582, EPI_ISL_3050583, EPI_ISL_3050584, EPI_ISL_3050585, EPI_ISL_3050586, EPI_ISL_3050587, EPI_ISL_3050588, EPI_ISL_3050589, EPI_ISL_3050590, EPI_ISL_3050592, EPI_ISL_3050593, EPI_ISL_3050594, EPI_ISL_3050595, EPI_ISL_3050596, EPI_ISL_3050597, EPI_ISL_3050598, EPI_ISL_3050599, EPI_ISL_3050600, EPI_ISL_3050601, EPI_ISL_3050602, EPI_ISL_3050603, EPI_ISL_3050604, EPI_ISL_3050605, EPI_ISL_3050606, EPI_ISL_3050607, EPI_ISL_3050608, EPI_ISL_3050609, EPI_ISL_3050610, EPI_ISL_3050611, EPI_ISL_3050612, EPI_ISL_3050613, EPI_ISL_3050614, EPI_ISL_3050619, EPI_ISL_3050620, EPI_ISL_3050621, EPI_ISL_3050622, EPI_ISL_3050623, EPI_ISL_3050624, EPI_ISL_3050625, EPI_ISL_3050626, EPI_ISL_3050627, EPI_ISL_3050628, EPI_ISL_3050629, EPI_ISL_3050630, EPI_ISL_3050631, EPI_ISL_3050632, EPI_ISL_3050633, EPI_ISL_3050634, EPI_ISL_3050635, EPI_ISL_3050636, EPI_ISL_3050637, EPI_ISL_3050638, EPI_ISL_3050639, EPI_ISL_3050640, EPI_ISL_3050642, EPI_ISL_3050643, EPI_ISL_3050644, EPI_ISL_3050645, EPI_ISL_3050646, EPI_ISL_3050647, EPI_ISL_3050648, EPI_ISL_3050649, EPI_ISL_3050650			
see above	Laboratório Central de Saúde Pública do Amazonas - LACEN-AM	Laboratorio de Ecologia de Doencas Transmissiveis na Amazonia, Instituto Leonidas e Maria Deane - Fiocruz Amazonia	André Corado; Debora Duarte; Felipe Naveca; Fernanda Nascimento; George Silva; Karina Pessoa; Luciana Gonçalves; Maria Júlia Brandão; Matilde Mejía; Michele Jesus; Valdinete Nascimento; Victor Souza; Ágatha Costa
EPI_ISL_1239012	Laboratório Central de Saúde Pública do Estado de Pernambuco (LACEN-PE)	WallauLab, Aggeu Magalhaes Institute	Alexandre Freitas da Silva; Antonio Mauro Rezende; Constância Flávia Junqueira Ayres; Cássia Docena; Duschinka Ribeiro Duarte Guedes; Elisama Helvecio; Filipe Zimmer Dezordi; Gabriel Luz Wallau on behalf of the Fiocruz COVID-19 Genomic Surveillance Network; Larissa Krokovsky; Laís Ceschini Machado; Marcelo Henrique dos Santos Paiva; Matheus Filgueira Bezerra; Sinval Pinto Brandão Filho
EPI_ISL_801397, EPI_ISL_801398, EPI_ISL_801399, EPI_ISL_801400, EPI_ISL_801401, EPI_ISL_801402, EPI_ISL_801403			
see above	Laboratório Central de Saúde Pública do Estado do Amazonas (LACEN-AM)	Laboratorio de Ecologia de Doencas Transmissiveis na Amazonia, Instituto Leonidas e Maria Deane - Fiocruz Amazonia	André Corado; Debora Duarte; Felipe Naveca on behalf of the Fiocruz COVID-19 Genomic Surveillance Network; Fernanda Nascimento; George Silva; Luciana Gonçalves; Maria Júlia Brandão; Michele Jesus; Valdinete Nascimento; Victor Souza; Ágatha Costa
EPI_ISL_2466265, EPI_ISL_2466266, EPI_ISL_2466267, EPI_ISL_3061877	Laboratório de Ecologia de Doenças Transmissíveis na Amazônia (EDTA), Instituto Leônidas e Maria Deane, FIOCRUZ, Manaus, Amazonas, Brazil.	Laboratory of Respiratory Viruses and Measles, Oswaldo Cruz Institute, FIOCRUZ	Alex Pauvolid-Corrêa; Alice Sampaio Rocha; Ana Beatriz Machado Lima; Ana Carolina Mendonca; Anna Carolina Paixao; Elisa Cavalcante Pereira; Felipe Naveca; Fernando Motta; Luciana Appolinario; Marilda Siqueira on behalf of the Fiocruz COVID-19 Genomic Surveillance Network; Mia Ferreira de Araujo; Paola Resende; Renata Serrano Lopes; Taina Venas
EPI_ISL_2233906	Scientific Platform Pasteur USP, University of Sao Paulo	Scientific Platform Pasteur USP, University of Sao Paulo	Cunha; M.P.; Muxel; S.M. and Minoprio, P.
EPI_ISL_906070	UPA Dr. Akira Tada	Instituto Adolfo Lutz, Interdiciplinary Procedures Center, Strategic Laboratory	Claudia Regina Gonçalves; Claudio Tavares Sacchi; Erica Valessa Ramos Gomes; Karoline Rodrigues Campos
EPI_ISL_2535159	Unidade de apoio ao diagnostico da COVID - UNADIG	Bioinformatics Laboratory / LNCC	Alessandra P Lamarca; Alexandra L Gerber; Amilcar Tanuri; Ana Paula de C Guimaraes; Ana Tereza R Vasconcelos; Andrea Cony Cavalcanti; Caio Luiz Pereira Ribeiro; Cassia Alves; Cintia Policarpo; Claudia Maria Braga de Mello; Cristiane Gomes da Silva; Diana Mariani; Douglas Terra Machado; Flavio Dias da Silva; Gleidson da Silva de Oliveira; Leandro Magalhaes de Souza; Liliane Cavalcante; Luiz G P de Almeida; Marcio Henrique de Oliveira Garcia; Mario Sergio Ribeiro; Ronaldo da Silva F Jr; Silvia Carvalho

We gratefully acknowledge the following Authors from the Originating laboratories responsible for obtaining the specimens, as well as the Submitting laboratories where the genome data were generated and shared via GISAID, on which this research is based.

All Submitters of data may be contacted directly via www.gisaid.org

Authors are sorted alphabetically.

Accession ID	Originating Laboratory	Submitting Laboratory	Authors
EPI_ISL_2612324, EPI_ISL_2612325, EPI_ISL_2612326, EPI_ISL_2612328, EPI_ISL_2612330, EPI_ISL_2612343, EPI_ISL_2612347, EPI_ISL_2612348, EPI_ISL_2612349, EPI_ISL_2612350, EPI_ISL_2612351, EPI_ISL_2612352, EPI_ISL_2612353, EPI_ISL_2612355, EPI_ISL_2612358, EPI_ISL_2612360, EPI_ISL_2612361, EPI_ISL_2612362, EPI_ISL_2612363, EPI_ISL_2612364, EPI_ISL_2612365, EPI_ISL_2612366, EPI_ISL_2612372, EPI_ISL_2612373, EPI_ISL_2612377, EPI_ISL_2612378, EPI_ISL_2612379, EPI_ISL_2612380, EPI_ISL_2612391, EPI_ISL_2612392, EPI_ISL_2612393, EPI_ISL_2612394, EPI_ISL_2612395, EPI_ISL_2612396, EPI_ISL_2612398, EPI_ISL_2612399, EPI_ISL_2612400, EPI_ISL_2612402, EPI_ISL_2612407, EPI_ISL_2612408			
see above	Centro de Infectologia Charles Mérieux/ Laboratório Rodolphe Mérieux, FUNDHACRE	Bioinformatics Laboratory / LNCC	Alessandra P Lamarca; Alexandra L Gerber; Ana Paula de C Guimarães; Ana Tereza R Vasconcelos; Andreas Stocker; Cirley Maria de Oliveira Lobato; Douglas Terra Machado; Luiz Fellype Alves de Souza; Luiz G P de Almeida; Ronaldo da Silva F Jr
EPI_ISL_2245062, EPI_ISL_2248780, EPI_ISL_2248781	Laboratório Central de Saúde Pública do Acre	Coordenação Geral de Laboratórios de Saúde Pública (CGLAB/DAEVS/SVS/MS)	Vagner Fonseca; et al.
EPI_ISL_1251222, EPI_ISL_1251223, EPI_ISL_1251224, EPI_ISL_1251225	Raimundo Reginaldo de Almeida	MOA Lab	Marcelo U. Ferreira; Priscila T. Rodrigues; Vanessa C. Nicolete
EPI_ISL_458139, EPI_ISL_848573, EPI_ISL_848574, EPI_ISL_848599, EPI_ISL_848600, EPI_ISL_848601, EPI_ISL_848602, EPI_ISL_848612, EPI_ISL_848613, EPI_ISL_848627			
see above	Evandro Chagas Institute	Evandro Chagas Institute	A.M.; Barbagelata; E.C.; E.M.A.; Ferreira; G.M.R; H.R; J.A.; Junior; K.C.; L.C.; L.S.; M.C.; Martins; P.S.; Pinheiro; Resque; Santos; Silva; Sousa; Sousa Junior; Viana; W.D.C.; da Silva

We gratefully acknowledge the following Authors from the Originating laboratories responsible for obtaining the specimens, as well as the Submitting laboratories where the genome data were generated and shared via GISAID, on which this research is based.

All Submitters of data may be contacted directly via www.gisaid.org

Authors are sorted alphabetically.

Accession ID	Originating Laboratory	Submitting Laboratory	Authors
EPI_ISL_450873	Evandro Chagas Institute	Evandro Chagas Institute	A.M.; Barbagelata; E.C.; E.M.A.; Ferreira; G.M.R; J.A.; Junior; L.C.; L.S.; M.C.; Martins; P.S.; Santos; Silva; Sousa; Sousa Junior; Viana; W.D.C.; da Silva
EPI_ISL_918551, EPI_ISL_918553, EPI_ISL_918554, EPI_ISL_918555, EPI_ISL_918556, EPI_ISL_918557, EPI_ISL_918558, EPI_ISL_918559, EPI_ISL_918560, EPI_ISL_918561			
see above	LACEN - Laboratório Central de Saúde Pública do Amapa	Evandro Chagas Institute	A.M.; Barbagelata; E.C.; E.M.A.; Ferreira; J.A.; Junior; K.C.; L.C.; L.S.; M.C.; P.S.; Pinheiro; Santos; Silva; Sousa; Sousa Junior; W.D.C.; da Silva
EPI_ISL_1164976, EPI_ISL_1164981, EPI_ISL_1164982, EPI_ISL_1164984, EPI_ISL_1164985, EPI_ISL_1261686, EPI_ISL_1261688, EPI_ISL_1261689, EPI_ISL_1261692, EPI_ISL_1261696, EPI_ISL_2488776, EPI_ISL_2488777, EPI_ISL_2488792, EPI_ISL_2488793, EPI_ISL_2488794, EPI_ISL_2488804, EPI_ISL_2488805, EPI_ISL_2488806, EPI_ISL_2488807			
see above	LACEN - Laboratório Central de Saúde Pública do Amapá	Evandro Chagas Institute	A.M.; Barbagelata; E.C.; E.M.A.; Ferreira; J.A.; Junior; K.C.; L.C.; L.S.; M.C.; P.S.; Pinheiro; Santos; Silva; Sousa; Sousa Junior; W.D.C.; da Silva
EPI_ISL_4030307	Laboratorio Central de Saude Publica do Amazonas - LACEN-AM	Laboratorio de Ecologia de Doencas Transmissiveis na Amazonia, Instituto Leonidas e Maria Deane - Fiocruz Amazonia	André Corado; Debora Duarte; Felipe Naveca; Fernanda Nascimento; George Silva; Karina Pessoa; Luciana Gonçalves; Maria Júlia Brandão; Matilde Mejía; Michele Jesus; Valdinete Nascimento; Victor Souza; Ágatha Costa
EPI_ISL_2983086, EPI_ISL_2983087, EPI_ISL_2983088, EPI_ISL_2983089, EPI_ISL_2983090, EPI_ISL_2983091, EPI_ISL_2983092, EPI_ISL_2983093, EPI_ISL_2983094, EPI_ISL_2983095, EPI_ISL_2983096, EPI_ISL_2983097, EPI_ISL_2983098, EPI_ISL_2983099, EPI_ISL_2983100, EPI_ISL_2983101, EPI_ISL_2983102, EPI_ISL_2983103, EPI_ISL_2983104, EPI_ISL_2983105, EPI_ISL_2983106, EPI_ISL_2983107, EPI_ISL_2983108, EPI_ISL_2983109, EPI_ISL_2983110, EPI_ISL_2983111, EPI_ISL_2983112, EPI_ISL_2983113, EPI_ISL_2983114, EPI_ISL_2983116, EPI_ISL_2983117, EPI_ISL_2983118, EPI_ISL_2983119, EPI_ISL_2983120, EPI_ISL_2983121, EPI_ISL_2983122, EPI_ISL_2983123, EPI_ISL_2983124, EPI_ISL_2983125, EPI_ISL_2983126, EPI_ISL_2983128, EPI_ISL_2983129, EPI_ISL_2983131, EPI_ISL_2983132, EPI_ISL_2983133, EPI_ISL_2983134, EPI_ISL_2983135, EPI_ISL_2983136, EPI_ISL_2983137, EPI_ISL_2983139, EPI_ISL_2983140, EPI_ISL_2983141, EPI_ISL_2983142, EPI_ISL_2983143, EPI_ISL_2983144, EPI_ISL_2983145, EPI_ISL_2983146, EPI_ISL_2983147, EPI_ISL_2983149, EPI_ISL_2983150, EPI_ISL_2983151, EPI_ISL_2983152, EPI_ISL_2983153, EPI_ISL_2983154, EPI_ISL_2983155, EPI_ISL_2983156, EPI_ISL_2983157, EPI_ISL_2983158, EPI_ISL_2983159, EPI_ISL_2983160, EPI_ISL_2983162, EPI_ISL_2983163, EPI_ISL_2983164, EPI_ISL_2983165, EPI_ISL_2983166, EPI_ISL_2983167, EPI_ISL_2983169, EPI_ISL_2983170, EPI_ISL_2983171, EPI_ISL_2983172, EPI_ISL_2983173, EPI_ISL_2983174, EPI_ISL_2983175, EPI_ISL_2983177, EPI_ISL_2983178, EPI_ISL_2983476, EPI_ISL_3434898, EPI_ISL_3434899, EPI_ISL_3434900, EPI_ISL_3434901, EPI_ISL_3434902, EPI_ISL_3434903, EPI_ISL_3434904, EPI_ISL_3434948, EPI_ISL_3434956, EPI_ISL_3434957, EPI_ISL_3434958, EPI_ISL_3434959, EPI_ISL_3434960, EPI_ISL_3434961, EPI_ISL_3434962, EPI_ISL_3434963, EPI_ISL_3434964, EPI_ISL_3434965, EPI_ISL_3434966, EPI_ISL_3434967, EPI_ISL_3434968, EPI_ISL_3434969, EPI_ISL_3434970, EPI_ISL_3434971, EPI_ISL_3434972, EPI_ISL_3434973, EPI_ISL_3434974, EPI_ISL_3434975, EPI_ISL_3434976, EPI_ISL_3434977, EPI_ISL_3434978, EPI_ISL_3434979, EPI_ISL_3434980, EPI_ISL_3434981, EPI_ISL_3434982, EPI_ISL_3434983, EPI_ISL_3435067, EPI_ISL_3539742, EPI_ISL_3539743, EPI_ISL_3539744, EPI_ISL_3539745, EPI_ISL_3539746, EPI_ISL_3539747, EPI_ISL_3539748, EPI_ISL_3539749, EPI_ISL_3539795, EPI_ISL_3539796, EPI_ISL_3539797, EPI_ISL_3539798, EPI_ISL_3539799, EPI_ISL_3539800, EPI_ISL_3539801, EPI_ISL_3539802, EPI_ISL_3539803, EPI_ISL_3539804, EPI_ISL_3539805, EPI_ISL_3539806, EPI_ISL_3539807, EPI_ISL_3539808, EPI_ISL_3539809, EPI_ISL_3539811, EPI_ISL_3539812, EPI_ISL_3539813, EPI_ISL_3539814, EPI_ISL_3539815, EPI_ISL_3539816, EPI_ISL_3539817, EPI_ISL_3539818, EPI_ISL_3539819, EPI_ISL_3539820, EPI_ISL_3539821, EPI_ISL_3539822, EPI_ISL_3539823, EPI_ISL_3539824, EPI_ISL_3539825, EPI_ISL_3539826, EPI_ISL_3539827, EPI_ISL_3539828, EPI_ISL_3539931, EPI_ISL_3539932, EPI_ISL_4080931, EPI_ISL_4080932, EPI_ISL_4080933, EPI_ISL_4081029, EPI_ISL_4081030, EPI_ISL_4081031, EPI_ISL_4081032, EPI_ISL_4081033, EPI_ISL_4081034, EPI_ISL_4081035, EPI_ISL_4081036, EPI_ISL_4081037, EPI_ISL_4081038, EPI_ISL_4081039, EPI_ISL_4081040, EPI_ISL_4081041, EPI_ISL_4081042, EPI_ISL_4081043, EPI_ISL_4081044, EPI_ISL_4081045, EPI_ISL_4081046, EPI_ISL_4081047, EPI_ISL_4081048, EPI_ISL_4081049, EPI_ISL_4081050, EPI_ISL_4081051, EPI_ISL_4081052, EPI_ISL_4081053, EPI_ISL_4081054, EPI_ISL_4081055, EPI_ISL_4081056, EPI_ISL_4081057, EPI_ISL_4081058, EPI_ISL_4081059, EPI_ISL_4081060, EPI_ISL_4081061, EPI_ISL_4081062, EPI_ISL_4081063, EPI_ISL_4081064, EPI_ISL_4081065, EPI_ISL_4081066			
see above	Laboratorio Central de Saude Publica do Estado do Amapa (LACEN/AP)	Laboratory of Respiratory Viruses and Measles, Oswaldo Cruz Institute, FIOCRUZ	Agatha Cristinne Prudencio; Alice Sampaio Rocha; Ana Carolina Mendonca; Andreia Santos Costa; Anna Carolina Paixao; Anne Caroline da Silva Soledade; Elisa Cavalcante Pereira; Fernando Motta; Ighor Leonardo Arantes Gomes; Lindomar dos Anjos Silva; Luciana Appolinario; Marcia Socorro Pereira Cavalcante; Marilda Siqueira on behalf of the Fiocruz COVID-19 Genomic Surveillance Network; Paola Resende; Renata Serrano Lopes; Taina Venas
EPI_ISL_2241547	Laboratório Central de Saúde Pública de Pernambuco	Coordenação Geral de Laboratórios de Saúde Pública (CGLAB/DAEVS/SVS/MS)	Vagner Fonseca; et al.
EPI_ISL_2245064, EPI_ISL_2245065, EPI_ISL_2245066, EPI_ISL_2245067, EPI_ISL_2245068, EPI_ISL_2245069, EPI_ISL_2245070, EPI_ISL_2245071, EPI_ISL_2245072, EPI_ISL_2245073, EPI_ISL_2245074, EPI_ISL_2245075, EPI_ISL_2245076, EPI_ISL_2245077, EPI_ISL_2245078, EPI_ISL_2245079, EPI_ISL_2248784, EPI_ISL_2248785, EPI_ISL_2248786, EPI_ISL_2248787, EPI_ISL_2298747, EPI_ISL_2298760, EPI_ISL_2298790, EPI_ISL_2298791, EPI_ISL_2298793, EPI_ISL_2298795, EPI_ISL_2298797, EPI_ISL_2298799, EPI_ISL_2298800, EPI_ISL_2298801, EPI_ISL_2298803, EPI_ISL_2298804, EPI_ISL_2298806, EPI_ISL_2298808, EPI_ISL_2298816, EPI_ISL_2298817, EPI_ISL_2298819, EPI_ISL_2298821, EPI_ISL_2298823, EPI_ISL_2298824, EPI_ISL_2298826, EPI_ISL_2298828, EPI_ISL_2298830, EPI_ISL_2298832			
see above	Laboratório Central de Saúde Pública do Amapá	Coordenação Geral de Laboratórios de Saúde Pública (CGLAB/DAEVS/SVS/MS)	Vagner Fonseca; et al.
EPI_ISL_458138, EPI_ISL_458142, EPI_ISL_458143, EPI_ISL_458144, EPI_ISL_458145, EPI_ISL_524784, EPI_ISL_524792, EPI_ISL_524793, EPI_ISL_524795, EPI_ISL_524796, EPI_ISL_848575, EPI_ISL_848576, EPI_ISL_848577, EPI_ISL_848578, EPI_ISL_848579, EPI_ISL_848584			
see above	Evandro Chagas Institute	Evandro Chagas Institute	A.M.; Barbagelata; E.C.; E.M.A.; Ferreira; G.M.R; H.R; J.A.; Junior; K.C.; L.C.; L.S.; M.C.; Martins; P.S.; Pinheiro; Resque; Santos; Silva; Sousa; Sousa Junior; Viana; W.D.C.; da Silva

We gratefully acknowledge the following Authors from the Originating laboratories responsible for obtaining the specimens, as well as the Submitting laboratories where the genome data were generated and shared via GISAID, on which this research is based.

All Submitters of data may be contacted directly via www.gisaid.org

Authors are sorted alphabetically.

Accession ID	Originating Laboratory	Submitting Laboratory	Authors
EPI_ISL_1293052, EPI_ISL_1293053, EPI_ISL_1293054, EPI_ISL_1293055, EPI_ISL_1303499, EPI_ISL_1303500, EPI_ISL_1303501, EPI_ISL_1303502, EPI_ISL_1303503, EPI_ISL_1303504, EPI_ISL_1303505			
see above	LACEN de Rondonia	Instituto Adolfo Lutz, Interdiciplinary Procedures Center, Strategic Laboratory	Caio Vinicius Dias Lopes; Claudia Regina Gonçalves; Claudio Tavares Sacchi; Erica Valessa Ramos Gomes; Karoline Rodrigues Campos
EPI_ISL_1493578, EPI_ISL_1493579, EPI_ISL_1493584, EPI_ISL_1493595, EPI_ISL_1493596, EPI_ISL_1493597, EPI_ISL_1493598, EPI_ISL_1493599, EPI_ISL_1493600, EPI_ISL_1494924, EPI_ISL_1520107, EPI_ISL_1520108, EPI_ISL_1520109			
see above	LACEN do Estado de Rondonia	Instituto Adolfo Lutz, Interdiciplinary Procedures Center, Strategic Laboratory	Caio Vinicius Dias Lopes; Claudia Regina Gonçalves; Claudio Tavares Sacchi; Erica Valessa Ramos Gomes; Karoline Rodrigues Campos
EPI_ISL_514131, EPI_ISL_514132, EPI_ISL_514133, EPI_ISL_514134, EPI_ISL_514135, EPI_ISL_514136, EPI_ISL_514137, EPI_ISL_514138			
see above	Rondônia Central Public Health Laboratory (LACEN/RO), vinculated to State Health Secretariat of Rondônia (SESAU/RO)	Molecular Virology Laboratory of Oswaldo Cruz Foundation of Rondônia	Adriana Cristina Salvador Maia; Alcione de Oliveira dos Santos; Alice Paula Di Sabatino Guimarães; Aline Linhares Ferreira de Melo Mendonça; Caio Henrique Nemeth Santos; Camila Flávia Gomes Azzi; Celina Aparecida Bertoni Lugtenburg; Cicileia Correia da Silva; Felipe Gomes Naveca; Felipe Souza Nogueira-Lima; Fernando Rodrigues Máximo; Jansen Fernandes de Medeiros; Juan Miguel Vilallobos-Salcedo and Deusilene Souza Vieira; Juan Miguel Vilallobos-Salcedo and Deusilene Souza Vieira.; Juan Miguel Vilallobos-Salcedo and Deusilene Souza Vieira1; Juliana Loca Furtado; Luan Felipe Botelho-Souza; Suelen Cavalcante; Tárcio Peixoto Roca; Rita de Cássia Pontello Rampazzo

We gratefully acknowledge the following Authors from the Originating laboratories responsible for obtaining the specimens, as well as the Submitting laboratories where the genome data were generated and shared via GISAID, on which this research is based.

All Submitters of data may be contacted directly via www.gisaid.org

Authors are sorted alphabetically.

Accession ID	Originating Laboratory	Submitting Laboratory	Authors
EPI_ISL_940630, EPI_ISL_943967, EPI_ISL_943968, EPI_ISL_943969, EPI_ISL_943970, EPI_ISL_943971	Hospital Geral de Sao Paulo	Instituto Adolfo Lutz, Interdiciplinary Procedures Center, Strategic Laboratory	Claudia Regina Gonçalves; Claudio Tavares Sacchi; Erica Valessa Ramos Gomes; Karoline Rodrigues Campos
EPI_ISL_1261687	LACEN - Laboratório Central de Saúde Pública de Roraima	Evandro Chagas Institute	A.M.; Barbagelata; E.C.; E.M.A.; Ferreira; J.A.; Junior; K.C.; L.C.; L.S.; M.C.; P.S.; Pinheiro; Santos; Silva; Sousa; Sousa Junior; W.D.C.; da Silva
EPI_ISL_918513, EPI_ISL_2488772, EPI_ISL_2488780, EPI_ISL_2488781, EPI_ISL_2488782, EPI_ISL_2488783, EPI_ISL_2488784, EPI_ISL_2488785, EPI_ISL_2488786			
see above	LACEN - Laboratório Central de Saúde Pública do Roraima	Evandro Chagas Institute	A.M.; Barbagelata; E.C.; E.M.A.; Ferreira; J.A.; Junior; K.C.; L.C.; L.S.; M.C.; P.S.; Pinheiro; Santos; Silva; Sousa; Sousa Junior; W.D.C.; da Silva
EPI_ISL_2245107, EPI_ISL_2245108, EPI_ISL_2245109, EPI_ISL_2245110, EPI_ISL_2245111, EPI_ISL_2245112, EPI_ISL_2245113, EPI_ISL_2298748, EPI_ISL_2298762, EPI_ISL_2298764, EPI_ISL_2298765, EPI_ISL_2298767, EPI_ISL_2298769, EPI_ISL_2298788, EPI_ISL_2298847, EPI_ISL_2298849			
see above	Laboratório Central de Saúde Pública de Roraima	Coordenação Geral de Laboratórios de Saúde Pública (CGLAB/DAEVS/SVS/MS)	Vagner Fonseca; et al.
EPI_ISL_2778007, EPI_ISL_2778008	Laboratório Central de Saúde Pública do Amazonas - LACEN-AM	Laboratorio de Ecologia de Doencas Transmissiveis na Amazonia, Instituto Leonidas e Maria Deane - Fiocruz Amazonia	André Corado; Debora Duarte; Felipe Naveca; Fernanda Nascimento; George Silva; Karina Pessoa; Luciana Gonçalves; Maria Júlia Brandão; Matilde Mejía; Michele Jesus; Valdinete Nascimento; Victor Souza; Ágatha Costa
EPI_ISL_848556, EPI_ISL_848586, EPI_ISL_848610, EPI_ISL_848616	Evandro Chagas Institute	Evandro Chagas Institute	A.M.; Barbagelata; E.C.; E.M.A.; Ferreira; J.A.; Junior; K.C.; L.C.; L.S.; M.C.; P.S.; Pinheiro; Santos; Silva; Sousa; Sousa Junior; W.D.C.; da Silva

We gratefully acknowledge the following Authors from the Originating laboratories responsible for obtaining the specimens, as well as the Submitting laboratories where the genome data were generated and shared via GISAID, on which this research is based.

All Submitters of data may be contacted directly via www.gisaid.org

Authors are sorted alphabetically.

Accession ID	Originating Laboratory	Submitting Laboratory	Authors
EPI_ISL_3663878, EPI_ISL_3663887, EPI_ISL_3663893, EPI_ISL_3664065, EPI_ISL_3664168, EPI_ISL_3664170	DASA	DASA	Adriano Bonaldi; Angelica Hristov; Annelise Lopes; Bianca Cota; Cristina Oliveira; Jose Levi; Lidia Yamamoto; Paulo Pierry; Rodrigo Guarischi; Rodrigo Salazar
EPI_ISL_2756435, EPI_ISL_2756436, EPI_ISL_2756437, EPI_ISL_2756438, EPI_ISL_2756439, EPI_ISL_2756440, EPI_ISL_2756441, EPI_ISL_2756442, EPI_ISL_2756443, EPI_ISL_2756444, EPI_ISL_2756445, EPI_ISL_2756446, EPI_ISL_2756447, EPI_ISL_2756448, EPI_ISL_2756449, EPI_ISL_2756450, EPI_ISL_2756451, EPI_ISL_2756452, EPI_ISL_2756453, EPI_ISL_2756454, EPI_ISL_2756455, EPI_ISL_2756456, EPI_ISL_2756457, EPI_ISL_2756458, EPI_ISL_2756459, EPI_ISL_2756460, EPI_ISL_2756461, EPI_ISL_2756462, EPI_ISL_2756463, EPI_ISL_2756464, EPI_ISL_2756465, EPI_ISL_2756466, EPI_ISL_2756467, EPI_ISL_2756468, EPI_ISL_2756469, EPI_ISL_2756470, EPI_ISL_2756471			
see above	Instituto Adolfo Lutz Central	Instituto Adolfo Lutz, Interdiciplinary Procedures Center, Strategic Laboratory	Caio Vinicius Dias Lopes; Claudia Regina Gonçalves; Claudio Tavares Sacchi; Erica Valessa Ramos Gomes; Karoline Rodrigues Campos; Leonardo Jose Tadeu de Araujo
EPI_ISL_943973, EPI_ISL_943974, EPI_ISL_943975, EPI_ISL_943976, EPI_ISL_943977, EPI_ISL_943978, EPI_ISL_943979, EPI_ISL_943980, EPI_ISL_943981, EPI_ISL_943982, EPI_ISL_943983, EPI_ISL_943985, EPI_ISL_943986, EPI_ISL_943987, EPI_ISL_943991, EPI_ISL_1303509, EPI_ISL_2919205, EPI_ISL_2919206, EPI_ISL_2919207, EPI_ISL_2919208, EPI_ISL_2919209, EPI_ISL_2919210, EPI_ISL_2919211, EPI_ISL_2919212, EPI_ISL_2919213, EPI_ISL_2919214, EPI_ISL_2919215, EPI_ISL_2919216, EPI_ISL_2919217, EPI_ISL_2919218, EPI_ISL_2919219, EPI_ISL_2919220, EPI_ISL_2919221, EPI_ISL_2919222, EPI_ISL_2919223, EPI_ISL_2919224, EPI_ISL_2919225, EPI_ISL_2919226, EPI_ISL_2919227, EPI_ISL_2958854, EPI_ISL_3316155, EPI_ISL_3316213			
see above	LACEN do Estado de Tocantins	Instituto Adolfo Lutz, Interdiciplinary Procedures Center, Strategic Laboratory	Caio Vinicius Dias Lopes; Claudia Regina Gonçalves; Claudio Tavares Sacchi; Erica Valessa Ramos Gomes; Karoline Rodrigues Campos; Leonardo Tadeu de Araujo; Marlon Benedito Nascomento Santos
EPI_ISL_3235304, EPI_ISL_3235305, EPI_ISL_3235306, EPI_ISL_3235307, EPI_ISL_3235308, EPI_ISL_3235310, EPI_ISL_3235311, EPI_ISL_3235312, EPI_ISL_3235313, EPI_ISL_3235314, EPI_ISL_4080794, EPI_ISL_4080795, EPI_ISL_4080796, EPI_ISL_4080797, EPI_ISL_4080798, EPI_ISL_4080799, EPI_ISL_4080800, EPI_ISL_4080801, EPI_ISL_4080802, EPI_ISL_4080803, EPI_ISL_4080804, EPI_ISL_4080805			
see above	Laboratorio Central de Saude Publica do Estado do Tocantins (LACEN/TO)	Laboratory of Respiratory Viruses and Measles, Oswaldo Cruz Institute, FIOCRUZ	Agatha Soares; Alice Sampaio Rocha; Ana Carolina Mendonca; Anna Carolina Paixao; Elisa Cavalcante Pereira; Fernando Motta; Ighor Arantes; Jucimaria Dantas Galvao; Luciana Appolinario; Marilda Siqueira on behalf of the Fiocruz COVID-19 Genomic Surveillance Network; Paola Resende; Renata Serrano Lopes; Taina Venas
EPI_ISL_3426438, EPI_ISL_3584949, EPI_ISL_3585952, EPI_ISL_3587057, EPI_ISL_3587490, EPI_ISL_3602957, EPI_ISL_3602958, EPI_ISL_3602960, EPI_ISL_3602961, EPI_ISL_3602964, EPI_ISL_3610172, EPI_ISL_3610352, EPI_ISL_3610353, EPI_ISL_3610354, EPI_ISL_3610355, EPI_ISL_3610356, EPI_ISL_3610357, EPI_ISL_3610358, EPI_ISL_3610359			
see above	Laboratório Central de Saúde Pública do Estado do Tocantins (LACEN/TO)	Bioinformatics and Biotechnology Laboratory (Labinftec/Federal University of Tocantins, Campus of Gurupi)	Bergmann Morais Ribeiro; Chácaras 69/72; Fabrício Souza Campos; Fernando Lucas Melo; Gurupi/TO Ueric José Borges de Souza; Jucimária Dantas Galvão; Laboratório de Bioinformática e Biotecnologia; Lote 7; Raíssa Nunes dos Santos; Rua Badejós; Ueric José Borges de Souza; Zona Rural | 77.402-970| UFT Campus de Gurupi
EPI_ISL_2645507, EPI_ISL_2645508, EPI_ISL_2645509, EPI_ISL_2645510, EPI_ISL_2645511, EPI_ISL_2645512, EPI_ISL_2645513, EPI_ISL_2863595, EPI_ISL_2863596, EPI_ISL_2863598, EPI_ISL_2863600, EPI_ISL_2983228, EPI_ISL_2983229, EPI_ISL_2983417, EPI_ISL_2983418, EPI_ISL_2983419, EPI_ISL_2983420, EPI_ISL_2983421, EPI_ISL_2983422, EPI_ISL_2983423, EPI_ISL_3190409, EPI_ISL_3190410, EPI_ISL_3190411			
see above	Labortorio Central de Saude Publica do Estado do Tocantins (LACEN/TO)	Laboratory of Respiratory Viruses and Measles, Oswaldo Cruz Institute, FIOCRUZ	Agatha Soares; Alice Sampaio Rocha; Ana Carolina Mendonca; Anna Carolina Paixao; Elisa Cavalcante Pereira; Fernando Motta; Ighor Arantes; Jucimaria Dantas Galvao; Luciana Appolinario; Marilda Siqueira on behalf of the Fiocruz COVID-19 Genomic Surveillance Network; Paola Resende; Renata Serrano Lopes; Taina Venas
EPI_ISL_1358300, EPI_ISL_1358301, EPI_ISL_1358302, EPI_ISL_1358303	Lacen de Tocantins	Instituto Adolfo Lutz, Interdiciplinary Procedures Center, Strategic Laboratory	Caio Vinicius Dias Lopes; Claudia Regina Gonçalves; Claudio Tavares Sacchi; Erica Valessa Ramos Gomes; Karoline Rodrigues Campos
EPI_ISL_3014467	Unidade de apoio ao diagnostico da COVID - UNADIG	Bioinformatics Laboratory / LNCC	Alessandra P Lamarca; Alexandra L Gerber; Amilcar Tanuri; Ana Paula de C Guimaraes; Ana Tereza R Vasconcelos; Andrea Cony Cavalcanti; Caio Luiz Pereira Ribeiro; Cassia Alves; Cintia Policarpo; Claudia Maria Braga de Mello; Cristiane Gomes da Silva; Diana Mariani; Douglas Terra Machado; Flavio Dias da Silva; Gleidson da Silva de Oliveira; Leandro Magalhaes de Souza; Liliane Cavalcante; Luiz G P de Almeida; Marcio Henrique de Oliveira Garcia; Mario Sergio Ribeiro; Ronaldo da Silva F Jr; Silvia Carvalho
EPI_ISL_1272236	Universidade Federal do Norte do Tocantins (UFNT)	Laboratório de Bioinformática e Biotecnologia (Labinftec/UFT)	Bergmann Morais Ribeiro; Fabrício Souza Campos; Fernando Lucas Melo; José Carlos Ribeiro Júnior; Monike da Silva Oliveira; Raíssa Nunes dos Santos; Rogério Fernandes Carvalho; Ueric José Borges de Souza

We gratefully acknowledge the following Authors from the Originating laboratories responsible for obtaining the specimens, as well as the Submitting laboratories where the genome data were generated and shared via GISAID, on which this research is based.

All Submitters of data may be contacted directly via www.gisaid.org

Authors are sorted alphabetically.

Accession ID	Originating Laboratory	Submitting Laboratory	Authors
EPI_ISL_1499114, EPI_ISL_1499205, EPI_ISL_1499298, EPI_ISL_1499299, EPI_ISL_1499300	Associação Fundo de Incentivo à Pesquisa (AFIP)	Associação Fundo de Incentivo à Pesquisa (AFIP)	Debora R. Ramadan; Erika Rodrigues de Oliveira; Juliana Nogueira Martins Rodrigues; Priscila Farias Tempaku; Sergio Tufik.; Soraya Sgambatti de Andrade
EPI_ISL_3663810, EPI_ISL_3663939, EPI_ISL_3663955, EPI_ISL_3663957, EPI_ISL_3663959, EPI_ISL_3663968, EPI_ISL_3663969, EPI_ISL_3663977, EPI_ISL_3663978, EPI_ISL_3663980, EPI_ISL_3663982, EPI_ISL_3663991, EPI_ISL_3663997, EPI_ISL_3664007, EPI_ISL_3664027, EPI_ISL_3664028, EPI_ISL_3664030, EPI_ISL_3664048, EPI_ISL_3664049, EPI_ISL_3664058, EPI_ISL_3664077, EPI_ISL_3664084, EPI_ISL_3664087, EPI_ISL_3664097, EPI_ISL_3664106, EPI_ISL_3664107, EPI_ISL_3664108, EPI_ISL_3664109, EPI_ISL_3664110, EPI_ISL_3664111, EPI_ISL_3664113, EPI_ISL_3664114, EPI_ISL_3664132, EPI_ISL_3664133, EPI_ISL_3664134, EPI_ISL_3664135, EPI_ISL_3664136, EPI_ISL_3664137, EPI_ISL_3664138, EPI_ISL_3664139, EPI_ISL_3664140, EPI_ISL_3664141, EPI_ISL_3664142, EPI_ISL_3664143, EPI_ISL_3664144, EPI_ISL_3664148, EPI_ISL_3664149, EPI_ISL_3664150, EPI_ISL_3664151, EPI_ISL_3664154, EPI_ISL_3664155, EPI_ISL_3664158, EPI_ISL_3664159, EPI_ISL_3664161, EPI_ISL_3664222			
see above	DASA	DASA	Adriano Bonaldi; Angelica Hristov; Annelise Lopes; Bianca Cota; Cristina Oliveira; Jose Levi; Lidia Yamamoto; Paulo Pierry; Rodrigo Guarischi; Rodrigo Salazar
EPI_ISL_476282	DB Diagnósticos do Brasil	Instituto de Medicina Tropical da Univesidade de São Paulo	Camila Alves Maia da Silva; Darlan da Silva Candido; Erika Regina Manuli; Ester Sabino; Flavia Cristina da Silva Sales; Giula Magalhaes Ferreira; Jaqueline Goes de Jesus; Julien Theze; Mariana Severo Ramundo; Nuno Faria; Samples: Nelson Gaburo Jr; Sequencing: Ingra Morales Claro; Thais de Moura Coletti
EPI_ISL_450874	Evandro Chagas Institute	Evandro Chagas Institute	A.M.; Barbagelata; E.C.; E.M.A.; Ferreira; G.M.R; J.A.; Junior; L.C.; L.S.; M.C.; Martins; P.S.; Santos; Silva; Sousa; Sousa Junior; Viana; W.D.C.; da Silva
EPI_ISL_1063789	Evandro Chagas Institute	Evandro Chagas Institute Virology	A.M.; Barbagelata; E.C.; E.M.A.; Ferreira; J.A.; Junior; K.C.; L.C.; L.S.; M.C.; P.S.; Pinheiro; Santos; Silva; Sousa; Sousa Junior; W.D.C.; da Silva
EPI_ISL_2758675, EPI_ISL_2758676, EPI_ISL_2758692, EPI_ISL_2758698, EPI_ISL_2758699, EPI_ISL_2758700, EPI_ISL_2758702, EPI_ISL_2758703, EPI_ISL_2758704, EPI_ISL_2758706, EPI_ISL_2758707, EPI_ISL_2758708, EPI_ISL_2758709, EPI_ISL_2758710, EPI_ISL_2758711, EPI_ISL_2758712, EPI_ISL_2758767, EPI_ISL_2758768, EPI_ISL_2758769, EPI_ISL_2758770, EPI_ISL_2758772, EPI_ISL_2758773, EPI_ISL_2758774, EPI_ISL_2758775			
see above	HUEM/IBMP	IPEC Guarapuava	NAPI-Genômica (Novos Arranjo de Pesquisa e Inovação em Genômica): Ademar Dantas da Cunha Júnior Adriano Ferrasa Adriano Mondini Aldo Przybysz Alessandra Lourenço Cecchini Armani Alex Sandro Jorge Alexandra Ivo de Medeiros Alexandre Maller Aline Cristina Batista Rodrigues Johann Ana Lucia Ferreira Ana Marisa Fusco Almeida Anderson Joel Martino Andrade André Luís Laforga Vanzela Andrea Duarte Doetzer Andrea Name Colado Simao Andressa Pereira de Souza Anelisa Ramão Angelica Beate Winter Boldt Anna Hermínia Castro Gomes de Amorim Anna Silvia Penteado Setti da Rocha Antonio Camilo da Silva Filho Antonio Stabelini Neto Arthur Hirata Bertachi Barbara Mendes Paz Chao Betty Cristiane Kuhn Bruno Ambrozio Galindo Bruno Ribeiro Cruz Camilla Reginatto De Pierri Carla Fredrichsen Moya Araujo Carla Fredrichsen Moya Araujo Carlos Alberto Oliveira de Biagi Junior Carlos Augusto Nassar Carlos Eduardo Buss Carlos Gilberto Carlotti Junior Carlos Henrique Schneider Carolina Panis Carolina Weigert Galvão Caroline de Jesus Coelho Donha Caroline Guisantes de Salvo Toni Caryna Eurich Mazur Catiuscie Cabreira da Silva Tortorella Celso F. D. Doliveira Cesar Luiz Boguszewski Christiane Pienna Soares Chung Man Chin Claudia Moro Cleverson Busso Cristiane Cominetti Daiane Priscila Simão-Silva Dalila Luciola Zanette Daniel de Paula Daniel de Paula Daniel Rech Daniela Fiori Gradia Daniela Pretti da Cunha Tirapelli Daniela Viganó Zanoti Jeronymo Daniele Ukan Danielle Malheiros Ferreira Danielle Venturini Deborah Catharine de Assis Leite Deivid Calebe de Souza Dennis Armando Bertolini Edenir Inez Pamero Edna Maria Vissoci Reiche Edson Roberto Arpini Miguel Eduardo José de Almeida Araújo Eliana Carolina Vespero Eliandro Reis Tavares Elza Kimura Grimshaw Emanuel Maltempi de Souza Emanuele Cristina Gustani Buss Émerson Carraro Emiliana Cristina Melo ENILze Maria de Souza Fonseca Ribeiro Enilze Maria de Souza Fonseca Ribeiro Erika Izumi Erika Seki Kioshima Cotica Evani Marques Pereira Fabio Negretti Fábio Rodrigues Ferreira Seiva Felipe Dunin dos Santos Felipe Tuon Fernanda Andreia Rosa Fernanda Cestaro Prado Cortez Fernanda Ivanski Fernanda Maris Peria Flavia Regina Oliveira de Barros Franciele Aní Caovilla Follador Franciele Mara Lucca Zanardo Bohm Francinete Ramos Campos Fulviana Silva Nishiyama GABRIEL RIBEIRO CORDEIRO Gabriela Datsch Bennemann Gisele Santos de Oliveira Glaucio Valdameri Glauco Akelinghton Freire Vitiello Glauco Vieira Miranda Glaura Scantamburlo ALves Fernandes Guilherme Ferreira Silveira Gustavo Bianchini Porfírio Gustavo Lenci Marques Hélito Volpato Hildebrando Masshiro Nagai Huei Diana Lee Ilce Mara de Syllos Cólus Iris Rabinovich Israel Gomy Jackson Kawakami Jacques Duílio Brancher Jaime Luís Lopes Rocha Jaqueline Carvalho de Oliveira Jean Henrique da Silva Rodrigues Jean Leandro dos Santos Jeane Eliete Laguila Visentainer João Paulo Bianchi Ximenez Joaquim Manoel da Silva Jociani Ascari Joel Donazzolo Jorge Luis Maria Ruiz Jose Knopfholz José Luis da Conceição Silva José Sebastião dos Santos Joseane Carla Schabarum Juliana Cheleski Wiggers Juliana Mara Serpeloni Juliana Morini Küpper Cardoso Perseguini Karen Brajão de Oliveira Karin Braun Prado Karine Aparecida de Lima Katiany Rizzieri Caleffi Ferracioli Katiuscia de Oliveira Francisco Gabriel Kelvinson Fernandes Viana Larissa Beatriz Cossalter Larissa Danielle Bahls Pinto Laurival Antonio Vilas Boas Léia Carolina Lucio Líbero Mezzadri Neto Ligia Carla Faccin Galhardi Lirane Elize Defante Ferreto Luciana Furlaneto Maia Luciana Oliveira de Fariña Luciana Reis Azevedo Alanis Luciane Regina Cavalli Lucy Megumi Yamauchi Lioni Luis Paulo Gomes Mascarenhas Luis Paulo Gomes Mascarenhas Luís Paulo Mascarenhas Lupe Furtado Alle Lyvia Regina Biagi Silva Bertachi Mara Antonia Ramos Costa Mara L. Cordeiro Marcela Maria Birolim Marcelo Ricardo Vicari Marcia Edilaine Lopes Consolaro Marcia Holsbach Beltrame Marcia Regina Eches Perugini Marcos Abdo Arbex Marcos Pileggi MARCOS TADEU GRZELCZAK Marcus Peikriszwili Tartaruga Maria Angelica Ehara Watanabe Maria Antonia Ramos Costa Maria Claudia Gross Maria José Soares Mendes Giannini Maria Leandra Terencio Maria Lúcia Bonfleur Maria Luiza Guimarães de Oliveira Maria Luiza Petzl-Erler Mariana Abe Vicente Cavagnari Marina Kimiko Kadowaki Marise Fonseca dos Santos Marla Karine Amarante Maurício Turkiewicz Mauro Antonio Alves Castro Michel Rodrigo Zambrano Passarini Michele Potrich Michelle Orane Schemberger Milena Massumi Kozonoe Mônica Degraf Cavallin Monica Tereza Suldofski Mucio Luiz de Assis Cirino Nadia Graciele Krohn Najeh Maissar Khalil Nédia de Castilhos Ghisi Neide Tomimura Costa Neiva Leite Neyva Maria Lopes Romeiro Patrícia Amâncio da Rosa Patricia Dayane Carvalho Schaker Patricia Oehlmeyer Nassar Patrícia Savio de Araújo-Souza Patricia Silva Lucio Paulo Henrique Couto Souza Paulo Roberto Donadio Percy Nohama Quirino Alves de Lima Neto Rafael Deminice Rafael dos Santos Bezerra Raquel Alves dos Santos Renan Manozzo Galante Renata Ernlund Freitas de Macedo Rita de Cássia Garcia Simão Roberta Losi Guembarovski Roberto H. Herai Roberto Rosati Rodrigo Ferreira Rodrigo Rodrigues Matiello Rogério Neri Shinsato Rogério Pincela Mateus Rosane Aparecida Ribeiro Rosilene Fressatti Cardoso Rosilene Fressatti Cardoso Sandra Mara Guse Scós Venske Selene Elifio Esposito Sérgio Ossamu Ioshii Silvana Giuliatti Silvia Mara de Souza Halick Silvio Henrique Maia de Almeida Simone Neumann Wendt Spencer Luiz Marques Payão Stefan Wolanski Negrão Stephane Janaina de Moura Escobar Sueli Fumie Yamada Ogatta SUELI PERCIO QUINAIA Taciane Finatto Tatiana Mayumi Veiga Iriyoda Tayza Katelline Danilau Ostroski Tony Alexander Hild Valeria Valente Vanessa Nascimento Kozak Vanessa Santos Sotomaior Victor Breno Pedrosa Victoria Zeghbi Cochenski Borba Vivian Rotuno Moure Valdameri Wander Rogerio Pavanelli Weber Cláudio Francisco Nunes da Silva Willian Augusto de Melo Yohandra Reyes Torres
EPI_ISL_2758771, EPI_ISL_2758797, EPI_ISL_2758798	HUEM/LACEN	IPEC Guarapuava	NAPI-Genômica (Novos Arranjo de Pesquisa e Inovação em Genômica): Ademar Dantas da Cunha Júnior Adriano Ferrasa Adriano Mondini Aldo Przybysz Alessandra Lourenço Cecchini Armani Alex Sandro Jorge Alexandra Ivo de Medeiros Alexandre Maller Aline Cristina Batista Rodrigues Johann Ana Lucia Ferreira Ana Marisa Fusco Almeida Anderson Joel Martino Andrade André Luís Laforga Vanzela Andrea Duarte Doetzer Andrea Name Colado Simao Andressa Pereira de Souza Anelisa Ramão Angelica Beate Winter Boldt Anna Hermínia Castro Gomes de Amorim Anna Silvia Penteado Setti da Rocha Antonio Camilo da Silva Filho Antonio Stabelini Neto Arthur Hirata Bertachi Barbara Mendes Paz Chao Betty Cristiane Kuhn Bruno Ambrozio Galindo Bruno Ribeiro Cruz Camilla Reginatto De Pierri Carla Fredrichsen Moya Araujo Carla Fredrichsen Moya Araujo Carlos Alberto Oliveira de Biagi Junior Carlos Augusto Nassar Carlos Eduardo Buss Carlos Gilberto Carlotti Junior Carlos Henrique Schneider Carolina Panis Carolina Weigert Galvão Caroline de Jesus Coelho Donha Caroline Guisantes de Salvo Toni Caryna Eurich Mazur Catiuscie Cabreira da Silva Tortorella Celso F. D. Doliveira Cesar Luiz Boguszewski Christiane Pienna Soares Chung Man Chin Claudia Moro Cleverson Busso Cristiane Cominetti Daiane Priscila Simão-Silva Dalila Luciola Zanette Daniel de Paula Daniel de Paula Daniel Rech Daniela Fiori Gradia Daniela Pretti da Cunha Tirapelli Daniela Viganó Zanoti Jeronymo Daniele Ukan Danielle Malheiros Ferreira Danielle Venturini Deborah Catharine de Assis Leite Deivid Calebe de Souza Dennis Armando Bertolini Edenir Inez Pamero Edna Maria Vissoci Reiche Edson Roberto Arpini Miguel Eduardo José de Almeida Araújo Eliana Carolina Vespero Eliandro Reis Tavares Elza Kimura Grimshaw Emanuel Maltempi de Souza Emanuele Cristina Gustani Buss Émerson Carraro Emiliana Cristina Melo ENILze Maria de Souza Fonseca Ribeiro Enilze Maria de Souza Fonseca Ribeiro Erika Izumi Erika Seki Kioshima Cotica Evani Marques Pereira Fabio Negretti Fábio Rodrigues Ferreira Seiva Felipe Dunin dos Santos Felipe Tuon Fernanda Andreia Rosa Fernanda Cestaro Prado Cortez Fernanda Ivanski Fernanda Maris Peria Flavia Regina Oliveira de Barros Franciele Aní Caovilla Follador Franciele Mara Lucca Zanardo Bohm Francinete Ramos Campos Fulviana Silva Nishiyama GABRIEL RIBEIRO CORDEIRO Gabriela Datsch Bennemann Gisele Santos de Oliveira Glaucio Valdameri Glauco Akelinghton Freire Vitiello Glauco Vieira Miranda Glaura Scantamburlo ALves Fernandes Guilherme Ferreira Silveira Gustavo Bianchini Porfírio Gustavo Lenci Marques Hélito Volpato Hildebrando Masshiro Nagai Huei Diana Lee Ilce Mara de Syllos Cólus Iris Rabinovich Israel Gomy Jackson Kawakami Jacques Duílio Brancher Jaime Luís Lopes Rocha Jaqueline Carvalho de Oliveira Jean Henrique da Silva Rodrigues Jean Leandro dos Santos Jeane Eliete Laguila Visentainer João Paulo Bianchi Ximenez Joaquim Manoel da Silva Jociani Ascari Joel Donazzolo Jorge Luis Maria Ruiz Jose Knopfholz José Luis da Conceição Silva José Sebastião dos Santos Joseane Carla Schabarum Juliana Cheleski Wiggers Juliana Mara Serpeloni Juliana Morini Küpper Cardoso Perseguini Karen Brajão de Oliveira Karin Braun Prado Karine Aparecida de Lima Katiany Rizzieri Caleffi Ferracioli Katiuscia de Oliveira Francisco Gabriel Kelvinson Fernandes Viana Larissa Beatriz Cossalter Larissa Danielle Bahls Pinto Laurival Antonio Vilas Boas Léia Carolina Lucio Líbero Mezzadri Neto Ligia Carla Faccin Galhardi Lirane Elize Defante Ferreto Luciana Furlaneto Maia Luciana Oliveira de Fariña Luciana Reis Azevedo Alanis Luciane Regina Cavalli Lucy Megumi Yamauchi Lioni Luis Paulo Gomes Mascarenhas Luis Paulo Gomes Mascarenhas Luís Paulo Mascarenhas Lupe Furtado Alle Lyvia Regina Biagi Silva Bertachi Mara Antonia Ramos Costa Mara L. Cordeiro Marcela Maria Birolim Marcelo Ricardo Vicari Marcia Edilaine Lopes Consolaro Marcia Holsbach Beltrame Marcia Regina Eches Perugini Marcos Abdo Arbex Marcos Pileggi MARCOS TADEU GRZELCZAK Marcus Peikriszwili Tartaruga Maria Angelica Ehara Watanabe Maria Antonia Ramos Costa Maria Claudia Gross Maria José Soares Mendes Giannini Maria Leandra Terencio Maria Lúcia Bonfleur Maria Luiza Guimarães de Oliveira Maria Luiza Petzl-Erler Mariana Abe Vicente Cavagnari Marina Kimiko Kadowaki Marise Fonseca dos Santos Marla Karine Amarante Maurício Turkiewicz Mauro Antonio Alves Castro Michel Rodrigo Zambrano Passarini Michele Potrich Michelle Orane Schemberger Milena Massumi Kozonoe Mônica Degraf Cavallin Monica Tereza Suldofski Mucio Luiz de Assis Cirino Nadia Graciele Krohn Najeh Maissar Khalil Nédia de Castilhos Ghisi Neide Tomimura Costa Neiva Leite Neyva Maria Lopes Romeiro Patrícia Amâncio da Rosa Patricia Dayane Carvalho Schaker Patricia Oehlmeyer Nassar Patrícia Savio de Araújo-Souza Patricia Silva Lucio Paulo Henrique Couto Souza Paulo Roberto Donadio Percy Nohama Quirino Alves de Lima Neto Rafael Deminice Rafael dos Santos Bezerra Raquel Alves dos Santos Renan Manozzo Galante Renata Ernlund Freitas de Macedo Rita de Cássia Garcia Simão Roberta Losi Guembarovski Roberto H. Herai Roberto Rosati Rodrigo Ferreira Rodrigo Rodrigues Matiello Rogério Neri Shinsato Rogério Pincela Mateus Rosane Aparecida Ribeiro Rosilene Fressatti Cardoso Rosilene Fressatti Cardoso Sandra Mara Guse Scós Venske Selene Elifio Esposito Sérgio Ossamu Ioshii Silvana Giuliatti Silvia Mara de Souza Halick Silvio Henrique Maia de Almeida Simone Neumann Wendt Spencer Luiz Marques Payão Stefan Wolanski Negrão Stephane Janaina de Moura Escobar Sueli Fumie Yamada Ogatta SUELI PERCIO QUINAIA Taciane Finatto Tatiana Mayumi Veiga Iriyoda Tayza Katelline Danilau Ostroski Tony Alexander Hild Valeria Valente Vanessa Nascimento Kozak Vanessa Santos Sotomaior Victor Breno Pedrosa Victoria Zeghbi Cochenski Borba Vivian Rotuno Moure Valdameri Wander Rogerio Pavanelli Weber Cláudio Francisco Nunes da Silva Willian Augusto de Melo Yohandra Reyes Torres
EPI_ISL_2758668, EPI_ISL_2758669, EPI_ISL_2758670, EPI_ISL_2758671, EPI_ISL_2758672, EPI_ISL_2758806	IPEC Guarapuava	IPEC Guarapuava	NAPI-Genômica (Novos Arranjo de Pesquisa e Inovação em Genômica): Ademar Dantas da Cunha Júnior Adriano Ferrasa Adriano Mondini Aldo Przybysz Alessandra Lourenço Cecchini Armani Alex Sandro Jorge Alexandra Ivo de Medeiros Alexandre Maller Aline Cristina Batista Rodrigues Johann Ana Lucia Ferreira Ana Marisa Fusco Almeida Anderson Joel Martino Andrade André Luís Laforga Vanzela Andrea Duarte Doetzer Andrea Name Colado Simao Andressa Pereira de Souza Anelisa Ramão Angelica Beate Winter Boldt Anna Hermínia Castro Gomes de Amorim Anna Silvia Penteado Setti da Rocha Antonio Camilo da Silva Filho Antonio Stabelini Neto Arthur Hirata Bertachi Barbara Mendes Paz Chao Betty Cristiane Kuhn Bruno Ambrozio Galindo Bruno Ribeiro Cruz Camilla Reginatto De Pierri Carla Fredrichsen Moya Araujo Carla Fredrichsen Moya Araujo Carlos Alberto Oliveira de Biagi Junior Carlos Augusto Nassar Carlos Eduardo Buss Carlos Gilberto Carlotti Junior Carlos Henrique Schneider Carolina Panis Carolina Weigert Galvão Caroline de Jesus Coelho Donha Caroline Guisantes de Salvo Toni Caryna Eurich Mazur Catiuscie Cabreira da Silva Tortorella Celso F. D. Doliveira Cesar Luiz Boguszewski Christiane Pienna Soares Chung Man Chin Claudia Moro Cleverson Busso Cristiane Cominetti Daiane Priscila Simão-Silva Dalila Luciola Zanette Daniel de Paula Daniel de Paula Daniel Rech Daniela Fiori Gradia Daniela Pretti da Cunha Tirapelli Daniela Viganó Zanoti Jeronymo Daniele Ukan Danielle Malheiros Ferreira Danielle Venturini Deborah Catharine de Assis Leite Deivid Calebe de Souza Dennis Armando Bertolini Edenir Inez Pamero Edna Maria Vissoci Reiche Edson Roberto Arpini Miguel Eduardo José de Almeida Araújo Eliana Carolina Vespero Eliandro Reis Tavares Elza Kimura Grimshaw Emanuel Maltempi de Souza Emanuele Cristina Gustani Buss Émerson Carraro Emiliana Cristina Melo ENILze Maria de Souza Fonseca Ribeiro Enilze Maria de Souza Fonseca Ribeiro Erika Izumi Erika Seki Kioshima Cotica Evani Marques Pereira Fabio Negretti Fábio Rodrigues Ferreira Seiva Felipe Dunin dos Santos Felipe Tuon Fernanda Andreia Rosa Fernanda Cestaro Prado Cortez Fernanda Ivanski Fernanda Maris Peria Flavia Regina Oliveira de Barros Franciele Aní Caovilla Follador Franciele Mara Lucca Zanardo Bohm Francinete Ramos Campos Fulviana Silva Nishiyama GABRIEL RIBEIRO CORDEIRO Gabriela Datsch Bennemann Gisele Santos de Oliveira Glaucio Valdameri Glauco Akelinghton Freire Vitiello Glauco Vieira Miranda Glaura Scantamburlo ALves Fernandes Guilherme Ferreira Silveira Gustavo Bianchini Porfírio Gustavo Lenci Marques Hélito Volpato Hildebrando Masshiro Nagai Huei Diana Lee Ilce Mara de Syllos Cólus Iris Rabinovich Israel Gomy Jackson Kawakami Jacques Duílio Brancher Jaime Luís Lopes Rocha Jaqueline Carvalho de Oliveira Jean Henrique da Silva Rodrigues Jean Leandro dos Santos Jeane Eliete Laguila Visentainer João Paulo Bianchi Ximenez Joaquim Manoel da Silva Jociani Ascari Joel Donazzolo Jorge Luis Maria Ruiz Jose Knopfholz José Luis da Conceição Silva José Sebastião dos Santos Joseane Carla Schabarum Juliana Cheleski Wiggers Juliana Mara Serpeloni Juliana Morini Küpper Cardoso Perseguini Karen Brajão de Oliveira Karin Braun Prado Karine Aparecida de Lima Katiany Rizzieri Caleffi Ferracioli Katiuscia de Oliveira Francisco Gabriel Kelvinson Fernandes Viana Larissa Beatriz Cossalter Larissa Danielle Bahls Pinto Laurival Antonio Vilas Boas Léia Carolina Lucio Líbero Mezzadri Neto Ligia Carla Faccin Galhardi Lirane Elize Defante Ferreto Luciana Furlaneto Maia Luciana Oliveira de Fariña Luciana Reis Azevedo Alanis Luciane Regina Cavalli Lucy Megumi Yamauchi Lioni Luis Paulo Gomes Mascarenhas Luis Paulo Gomes Mascarenhas Luís Paulo Mascarenhas Lupe Furtado Alle Lyvia Regina Biagi Silva Bertachi Mara Antonia Ramos Costa Mara L. Cordeiro Marcela Maria Birolim Marcelo Ricardo Vicari Marcia Edilaine Lopes Consolaro Marcia Holsbach Beltrame Marcia Regina Eches Perugini Marcos Abdo Arbex Marcos Pileggi MARCOS TADEU GRZELCZAK Marcus Peikriszwili Tartaruga Maria Angelica Ehara Watanabe Maria Antonia Ramos Costa Maria Claudia Gross Maria José Soares Mendes Giannini Maria Leandra Terencio Maria Lúcia Bonfleur Maria Luiza Guimarães de Oliveira Maria Luiza Petzl-Erler Mariana Abe Vicente Cavagnari Marina Kimiko Kadowaki Marise Fonseca dos Santos Marla Karine Amarante Maurício Turkiewicz Mauro Antonio Alves Castro Michel Rodrigo Zambrano Passarini Michele Potrich Michelle Orane Schemberger Milena Massumi Kozonoe Mônica Degraf Cavallin Monica Tereza Suldofski Mucio Luiz de Assis Cirino Nadia Graciele Krohn Najeh Maissar Khalil Nédia de Castilhos Ghisi Neide Tomimura Costa Neiva Leite Neyva Maria Lopes Romeiro Patrícia Amâncio da Rosa Patricia Dayane Carvalho Schaker Patricia Oehlmeyer Nassar Patrícia Savio de Araújo-Souza Patricia Silva Lucio Paulo Henrique Couto Souza Paulo Roberto Donadio Percy Nohama Quirino Alves de Lima Neto Rafael Deminice Rafael dos Santos Bezerra Raquel Alves dos Santos Renan Manozzo Galante Renata Ernlund Freitas de Macedo Rita de Cássia Garcia Simão Roberta Losi Guembarovski Roberto H. Herai Roberto Rosati Rodrigo Ferreira Rodrigo Rodrigues Matiello Rogério Neri Shinsato Rogério Pincela Mateus Rosane Aparecida Ribeiro Rosilene Fressatti Cardoso Rosilene Fressatti Cardoso Sandra Mara Guse Scós Venske Selene Elifio Esposito Sérgio Ossamu Ioshii Silvana Giuliatti Silvia Mara de Souza Halick Silvio Henrique Maia de Almeida Simone Neumann Wendt Spencer Luiz Marques Payão Stefan Wolanski Negrão Stephane Janaina de Moura Escobar Sueli Fumie Yamada Ogatta SUELI PERCIO QUINAIA Taciane Finatto Tatiana Mayumi Veiga Iriyoda Tayza Katelline Danilau Ostroski Tony Alexander Hild Valeria Valente Vanessa Nascimento Kozak Vanessa Santos Sotomaior Victor Breno Pedrosa Victoria Zeghbi Cochenski Borba Vivian Rotuno Moure Valdameri Wander Rogerio Pavanelli Weber Cláudio Francisco Nunes da Silva Willian Augusto de Melo Yohandra Reyes Torres
EPI_ISL_2344420, EPI_ISL_2344421, EPI_ISL_2344425, EPI_ISL_2344426, EPI_ISL_2344428, EPI_ISL_2344431, EPI_ISL_2344440, EPI_ISL_2344450, EPI_ISL_2344451, EPI_ISL_2344455, EPI_ISL_2344456, EPI_ISL_2344457, EPI_ISL_2344458			
see above	Instituto Butantan	Instituto de Medicina Tropical de Sao Paulo	Brazil-UK Centre for Arbovirus Discovery Diagnosis Genomics and Epidemiology (CADDE) Genomic Network - Instituto de Medicina Tropical
EPI_ISL_3418670, EPI_ISL_3418671, EPI_ISL_3418673, EPI_ISL_3418676, EPI_ISL_3418678, EPI_ISL_3418679, EPI_ISL_3418680, EPI_ISL_3418681, EPI_ISL_3418682, EPI_ISL_3418683			
see above	Instituto Carlos Chagas - ICC, FIOCRUZ Parana	Instituto Carlos Chagas - ICC, FIOCRUZ Parana	A.A.; A.M.; A.R.; Aguiar; Albrecht, L.; Alves; Avila; Balsanelli, E.; Becker, G.; Blanes, L.; Dallagiovanna, B.; Debur; E.M.; F.K.; F.O.; Faoro, H.; Graef, T.; H.G.; I.N.; L.G.; L.R.; M.M.; M.O.; Marchini; Md.C.; Morello; Nardeli; Oliveira; P.C.; Passetti, F.; Pedrosa; Resende; Riediger; S.C.; Schemberger; Suzukawa; V.A.; Zanette, D.; de Baura; de Souza; dos Santos
EPI_ISL_2758987, EPI_ISL_2758988, EPI_ISL_2758992, EPI_ISL_2758994, EPI_ISL_2758995, EPI_ISL_2758997, EPI_ISL_2759001, EPI_ISL_2759002, EPI_ISL_2759004, EPI_ISL_2759006, EPI_ISL_2759008, EPI_ISL_2759011, EPI_ISL_2759012, EPI_ISL_2759016, EPI_ISL_2759017, EPI_ISL_2759020, EPI_ISL_2759021, EPI_ISL_2759022, EPI_ISL_2759024, EPI_ISL_2759025, EPI_ISL_2759026, EPI_ISL_2759028, EPI_ISL_2759030, EPI_ISL_2759033, EPI_ISL_2759035, EPI_ISL_2759037, EPI_ISL_2759040, EPI_ISL_2759041, EPI_ISL_2759045, EPI_ISL_2759046, EPI_ISL_2759047, EPI_ISL_2759050, EPI_ISL_2759051, EPI_ISL_2759052, EPI_ISL_2759054, EPI_ISL_2759056, EPI_ISL_2759057, EPI_ISL_2759059, EPI_ISL_2759061, EPI_ISL_2759063, EPI_ISL_2759065, EPI_ISL_2759066, EPI_ISL_2759068, EPI_ISL_2775419			
see above	Instituto de Biologia Molecular do Paraná (LAC)	Instituto Carlos Chagas - Fiocruz	Alessandra De Melo Aguiar; Andreia Akemi Suzukawa; Andréa Rodrigues Ávila; Bruno Dallagiovanna; Dalila Zanette; Eduardo Balsanelli; Emanuel Maltempi de Souza; Fabio Passetti; Fabricio Klerynton Marchini; Fábio de Oliveira Pedrosa; Guilherme Becker; Helisson Faoro; Hellen Geremias dos Santos; Irina Nastassja Riediger; Letusa Albrecht; Lucas Blanes; Luis Gustavo Morello; Lysangela Ronalte Alves; Maria do Carmo Debur; Mauro de Medeiros Oliveira; Michelle Orane Schemberger; Paola Cristina Resende; Sheila Cristina Nardeli; Tiago Gräf; Valter Antônio de Baura

EPI_ISL_918515, EPI_ISL_918516, EPI_ISL_918517, EPI_ISL_918522, EPI_ISL_918523, EPI_ISL_918524, EPI_ISL_918525, EPI_ISL_918526, EPI_ISL_918527, EPI_ISL_918528, EPI_ISL_918529, EPI_ISL_918530, EPI_ISL_918545, EPI_ISL_918546, EPI_ISL_918547, EPI_ISL_918548, EPI_ISL_918549, EPI_ISL_918550, EPI_ISL_918552, EPI_ISL_2488814, EPI_ISL_2488815, EPI_ISL_2488816, EPI_ISL_2488817, EPI_ISL_2488818, EPI_ISL_2488819

see above	LACEN - Laboratório Central de Saúde Pública do Para	Evandro Chagas Institute	A.M.; Barbagelata; E.C.; E.M.A.; Ferreira; J.A.; Junior; K.C.; L.C.; L.S.; M.C.; P.S.; Pinheiro; Santos; Silva; Sousa; Sousa Junior; W.D.C.; da Silva

EPI_ISL_1086377, EPI_ISL_1164989, EPI_ISL_1164990, EPI_ISL_1164991, EPI_ISL_1164992, EPI_ISL_1164994, EPI_ISL_1164999

see above	LACEN - Laboratório Central de Saúde Pública do Paraiba	Evandro Chagas Institute	A.M.; Barbagelata; E.C.; E.M.A.; Ferreira; J.A.; Junior; K.C.; L.C.; L.S.; M.C.; P.S.; Pinheiro; Santos; Silva; Sousa; Sousa Junior; W.D.C.; da Silva

EPI_ISL_904120, EPI_ISL_904121, EPI_ISL_1164972, EPI_ISL_1164974, EPI_ISL_1164975, EPI_ISL_1164978, EPI_ISL_1164983

see above	LACEN - Laboratório Central de Saúde Pública do Pará	Evandro Chagas Institute	A.M.; Barbagelata; E.C.; E.M.A.; Ferreira; J.A.; Junior; K.C.; L.C.; L.S.; M.C.; P.S.; Pinheiro; Santos; Silva; Sousa; Sousa Junior; W.D.C.; da Silva

EPI_ISL_3835195, EPI_ISL_3835196, EPI_ISL_3835197, EPI_ISL_3835198, EPI_ISL_3835199, EPI_ISL_3835200, EPI_ISL_3835201, EPI_ISL_3835202, EPI_ISL_3835204, EPI_ISL_3835205, EPI_ISL_3835209, EPI_ISL_3835210, EPI_ISL_3835211, EPI_ISL_3835212, EPI_ISL_3835213, EPI_ISL_3835214, EPI_ISL_3835215, EPI_ISL_3835216, EPI_ISL_3835217, EPI_ISL_3835218, EPI_ISL_3835219, EPI_ISL_3835220, EPI_ISL_3835222, EPI_ISL_3835224, EPI_ISL_3835225, EPI_ISL_3835228, EPI_ISL_3835229, EPI_ISL_3835230, EPI_ISL_3835231, EPI_ISL_3835232, EPI_ISL_3835233, EPI_ISL_3835234, EPI_ISL_3835235, EPI_ISL_3835237, EPI_ISL_3835238, EPI_ISL_3835239

see above	LACEN/PB	WallauLab on behalf of Fiocruz COVID-19 Genomic Surveillance Network	Alexandre Freitas da Silva; Cassia Docena; Constância Flávia Junqueira Ayres; Filipe Zimmer Dezordi; Gabriel Luz Wallau; Gustavo Barbosa de Lima; Lais Ceschini Machado; Lilian Caroliny Amorim Silva; Maira Galdino da Rocha Pitta; Marcelo Henrique dos Santos Paiva; Matheus Filgueira Bezerra; Michelly Cristiny Pereira; Rômulo Pessoa e Silva; Sinval Pinto Brandão Filho

EPI_ISL_1213160, EPI_ISL_1213161, EPI_ISL_1213189, EPI_ISL_1213201, EPI_ISL_1213207, EPI_ISL_1213213, EPI_ISL_1213336, EPI_ISL_1213343, EPI_ISL_1213344, EPI_ISL_1213367, EPI_ISL_1213369, EPI_ISL_1213370, EPI_ISL_1213388, EPI_ISL_1213392, EPI_ISL_1213404, EPI_ISL_1213406, EPI_ISL_1213427, EPI_ISL_1213429, EPI_ISL_1213431, EPI_ISL_1213433, EPI_ISL_1213434, EPI_ISL_1213436, EPI_ISL_1213438, EPI_ISL_1213439, EPI_ISL_1213441, EPI_ISL_1213443, EPI_ISL_1213444, EPI_ISL_1213446, EPI_ISL_1213448, EPI_ISL_1213449, EPI_ISL_1213451, EPI_ISL_1213453, EPI_ISL_1213454, EPI_ISL_1213456, EPI_ISL_1213458, EPI_ISL_1213459, EPI_ISL_1213461, EPI_ISL_1213463

see above	LBM/UFPB	Bioinformatics Laboratory / LNCC	Alessandra P Lamarca; Alexandra L Gerber; Ana Paula Melo Mariano; Ana Paula de C Guimarães; Ana Tereza R Vasconcelos; Angela Maria Guimarães Santos; Bianca Mendes Maciel; Danielle Angst Secco; Eduardo Sérgio Soares Sousa; Eloiza Helena Campana; Francisco Paulo Freire Neto; George Rego Albuquerque; Kátia Castanho Scortecci; Lucymara Fassarella Agnez Lima; Luiz G P de Almeida; Luís Cristóvão Porto; Otavio J. Brustolini; Paulo Ricardo Nascimento; Ronaldo da Silva Francisco Jr; Sandra Rocha Gadelha; Selma Maria Bezerra Jeronimo; Vinícius Pietta Perez

EPI_ISL_2196243, EPI_ISL_2196281, EPI_ISL_2196282, EPI_ISL_2196283, EPI_ISL_2196284, EPI_ISL_2196285, EPI_ISL_2196286, EPI_ISL_2196287, EPI_ISL_2196288, EPI_ISL_2196289, EPI_ISL_2196290, EPI_ISL_2196291, EPI_ISL_2196292, EPI_ISL_2196293, EPI_ISL_2196294, EPI_ISL_2196295, EPI_ISL_2196296, EPI_ISL_2196297, EPI_ISL_2196298, EPI_ISL_2196299, EPI_ISL_2196300, EPI_ISL_2196301, EPI_ISL_2196302, EPI_ISL_2196303, EPI_ISL_2196304, EPI_ISL_2196305, EPI_ISL_2443557, EPI_ISL_2443558, EPI_ISL_2443559, EPI_ISL_2443560, EPI_ISL_2443561, EPI_ISL_2443562, EPI_ISL_2443563, EPI_ISL_2443564, EPI_ISL_2645421, EPI_ISL_2645422, EPI_ISL_2645423, EPI_ISL_2645424, EPI_ISL_2645425, EPI_ISL_2645426, EPI_ISL_2645427, EPI_ISL_2645428, EPI_ISL_2645429, EPI_ISL_2645430, EPI_ISL_2645431, EPI_ISL_2645432, EPI_ISL_2645433, EPI_ISL_2645434, EPI_ISL_2645435, EPI_ISL_2645436, EPI_ISL_2645437, EPI_ISL_2645438, EPI_ISL_2645439, EPI_ISL_2645819, EPI_ISL_2645820, EPI_ISL_2645821, EPI_ISL_2645822, EPI_ISL_2645823, EPI_ISL_2645824, EPI_ISL_2645825, EPI_ISL_2645826, EPI_ISL_2645827, EPI_ISL_2645828, EPI_ISL_2645829, EPI_ISL_2645830, EPI_ISL_2645831, EPI_ISL_2645832, EPI_ISL_2645833, EPI_ISL_2645834, EPI_ISL_2645835, EPI_ISL_2645836, EPI_ISL_2645837, EPI_ISL_2645838, EPI_ISL_2645839, EPI_ISL_2645840, EPI_ISL_2645841, EPI_ISL_2645842, EPI_ISL_2645843, EPI_ISL_2645844, EPI_ISL_2645845, EPI_ISL_2645846, EPI_ISL_2645847, EPI_ISL_2645848, EPI_ISL_2645849, EPI_ISL_2645850, EPI_ISL_2645851, EPI_ISL_2645852, EPI_ISL_2645853, EPI_ISL_2645854, EPI_ISL_2645855, EPI_ISL_2645856, EPI_ISL_2645857, EPI_ISL_2645858, EPI_ISL_2645859, EPI_ISL_2645860, EPI_ISL_2645861, EPI_ISL_2645862, EPI_ISL_2645863, EPI_ISL_2645864, EPI_ISL_2645865, EPI_ISL_2645866, EPI_ISL_2645867, EPI_ISL_2645868, EPI_ISL_2645869, EPI_ISL_2645870, EPI_ISL_2645871, EPI_ISL_2645872, EPI_ISL_2645873, EPI_ISL_2645874, EPI_ISL_2645875, EPI_ISL_2645876, EPI_ISL_2645877, EPI_ISL_2645878, EPI_ISL_2645879, EPI_ISL_2645880, EPI_ISL_2645882, EPI_ISL_2645883, EPI_ISL_2645884, EPI_ISL_2645886, EPI_ISL_2645887, EPI_ISL_2645888, EPI_ISL_2645889, EPI_ISL_2645890, EPI_ISL_2645891, EPI_ISL_2645892, EPI_ISL_2645893, EPI_ISL_2645894, EPI_ISL_2645895, EPI_ISL_2645896, EPI_ISL_2645897, EPI_ISL_2645898, EPI_ISL_2645899, EPI_ISL_2645900, EPI_ISL_2645901, EPI_ISL_2645902, EPI_ISL_2645903, EPI_ISL_2645904, EPI_ISL_2645905, EPI_ISL_2645906, EPI_ISL_2645907, EPI_ISL_2645908, EPI_ISL_2645909, EPI_ISL_2645910, EPI_ISL_2645911, EPI_ISL_2645912, EPI_ISL_2645913, EPI_ISL_2645914, EPI_ISL_2645915, EPI_ISL_2645916, EPI_ISL_2645917, EPI_ISL_2645918, EPI_ISL_2645919, EPI_ISL_2645920, EPI_ISL_2645921, EPI_ISL_2645922, EPI_ISL_2645923, EPI_ISL_2645924, EPI_ISL_2645925, EPI_ISL_2645926, EPI_ISL_2645927, EPI_ISL_2645928, EPI_ISL_2645929, EPI_ISL_2645930, EPI_ISL_2645931, EPI_ISL_2645932, EPI_ISL_2731453, EPI_ISL_2731454, EPI_ISL_2731455, EPI_ISL_2731456, EPI_ISL_2863578, EPI_ISL_2863580, EPI_ISL_2863582, EPI_ISL_2863706, EPI_ISL_2863707, EPI_ISL_2863709, EPI_ISL_2863711, EPI_ISL_2863801, EPI_ISL_2863802, EPI_ISL_2863803, EPI_ISL_2863804, EPI_ISL_2863805, EPI_ISL_2863806, EPI_ISL_2863807, EPI_ISL_2863808, EPI_ISL_2863809, EPI_ISL_2863810, EPI_ISL_2863811, EPI_ISL_2863812, EPI_ISL_2863813, EPI_ISL_2863814, EPI_ISL_2863815, EPI_ISL_2863816, EPI_ISL_2863817, EPI_ISL_2863818, EPI_ISL_2863819, EPI_ISL_2863820, EPI_ISL_2863821, EPI_ISL_2863822, EPI_ISL_2863823, EPI_ISL_2863824, EPI_ISL_2863825, EPI_ISL_2863826, EPI_ISL_2863827, EPI_ISL_2863828, EPI_ISL_2863829, EPI_ISL_2863830, EPI_ISL_2863831, EPI_ISL_2863832, EPI_ISL_2863833, EPI_ISL_2863834, EPI_ISL_2863835, EPI_ISL_2863836, EPI_ISL_2863837, EPI_ISL_2863838, EPI_ISL_2863839, EPI_ISL_2863840, EPI_ISL_2863841, EPI_ISL_2863842, EPI_ISL_2863843, EPI_ISL_2863844, EPI_ISL_2863845, EPI_ISL_2863846, EPI_ISL_2863847, EPI_ISL_2863848, EPI_ISL_2863849, EPI_ISL_2863850, EPI_ISL_2863851, EPI_ISL_2863852, EPI_ISL_2863853, EPI_ISL_2863854, EPI_ISL_2863894, EPI_ISL_3539238, EPI_ISL_3539239, EPI_ISL_3539240, EPI_ISL_3801916, EPI_ISL_3802965, EPI_ISL_3802966, EPI_ISL_4061287, EPI_ISL_4061289, EPI_ISL_4061290, EPI_ISL_4061291, EPI_ISL_4061293, EPI_ISL_4061295, EPI_ISL_4080766, EPI_ISL_4080767, EPI_ISL_4080768, EPI_ISL_4080769, EPI_ISL_4080770

see above	Laboratorio Central de Saude Publica do Estado do Para (LACEN/PA)	Laboratory of Respiratory Viruses and Measles, Oswaldo Cruz Institute, FIOCRUZ	Agatha Cristinne Prudencio; Agatha Cristinne Prudencio Soares; Agatha Soares; Alice Sampaio Rocha; Ana Carolina Mendonca; Anna Carolina Paixao; Elisa Cavalcante Pereira; Fernando Motta; Ighor Arantes; Ighor Leonardo Arantes Gomes; Luciana Appolinario; Marilda Siqueira on behalf of the Fiocruz COVID-19 Genomic Surveillance Network; Paola Resende; Renata Serrano Lopes; Taina Venas; Valnete Andrade
EPI_ISL_2535017	Laboratorio Central Noel Nutels	Bioinformatics Laboratory / LNCC	Alessandra P Lamarca; Alexandra L Gerber; Amilcar Tanuri; Ana Paula de C Guimaraes; Ana Tereza R Vasconcelos; Andrea Cony Cavalcanti; Caio Luiz Pereira Ribeiro; Cassia Alves; Cintia Policarpo; Claudia Maria Braga de Mello; Cristiane Gomes da Silva; Diana Mariani; Douglas Terra Machado; Flavio Dias da Silva; Gleidson da Silva de Oliveira; Leandro Magalhaes de Souza; Liliane Cavalcante; Luiz G P de Almeida; Marcio Henrique de Oliveira Garcia; Mario Sergio Ribeiro; Ronaldo da Silva F Jr; Silvia Carvalho

EPI_ISL_1181416, EPI_ISL_1181422, EPI_ISL_2157379, EPI_ISL_2157380, EPI_ISL_2157383, EPI_ISL_2157394, EPI_ISL_2157395, EPI_ISL_2157401, EPI_ISL_2157425, EPI_ISL_2157550, EPI_ISL_2157578, EPI_ISL_2157581, EPI_ISL_2157582, EPI_ISL_2157589, EPI_ISL_2157591, EPI_ISL_2274092, EPI_ISL_2274093, EPI_ISL_2536303, EPI_ISL_2536304, EPI_ISL_2536305, EPI_ISL_2536306, EPI_ISL_2536307, EPI_ISL_2536308, EPI_ISL_2536309, EPI_ISL_2536310, EPI_ISL_2536311, EPI_ISL_2536312, EPI_ISL_2536313, EPI_ISL_2536314, EPI_ISL_2536315, EPI_ISL_2536316, EPI_ISL_2536317, EPI_ISL_2536318, EPI_ISL_2536319, EPI_ISL_2536320, EPI_ISL_2536321, EPI_ISL_2536322, EPI_ISL_2536323, EPI_ISL_2536324, EPI_ISL_2536325, EPI_ISL_2536326, EPI_ISL_2536327, EPI_ISL_2536328, EPI_ISL_2536329, EPI_ISL_2536330, EPI_ISL_2536331, EPI_ISL_2536332, EPI_ISL_2536333, EPI_ISL_2536334, EPI_ISL_2536335, EPI_ISL_2536336, EPI_ISL_2536337, EPI_ISL_2536338, EPI_ISL_2536339, EPI_ISL_2536340, EPI_ISL_2536341, EPI_ISL_2536342, EPI_ISL_2536343, EPI_ISL_2536344, EPI_ISL_2536345, EPI_ISL_2536346, EPI_ISL_2536347, EPI_ISL_2536348, EPI_ISL_2536349, EPI_ISL_2536350, EPI_ISL_2536351, EPI_ISL_2536352, EPI_ISL_2536353, EPI_ISL_2731457, EPI_ISL_3061880, EPI_ISL_3061881, EPI_ISL_3061882, EPI_ISL_3061883, EPI_ISL_3061884, EPI_ISL_3061885, EPI_ISL_3061886, EPI_ISL_3061887, EPI_ISL_3061888, EPI_ISL_3061889, EPI_ISL_3061890, EPI_ISL_3061891

see above	Laboratorio Central de Saude Publica do Estado da Paraiba (LACEN-PB)	Laboratory of Respiratory Viruses and Measles, Oswaldo Cruz Institute, FIOCRUZ	Alice Sampaio Rocha; Ana Carolina Mendonca; Anna Carolina Paixao; Dalane Loudal Florentino Teixeira; Elisa Cavalcante Pereira; Fernando Motta; Irina Riediger; Joao Felipe Bezerra; Luciana Appolinario; Marilda Siqueira on behalf of the Fiocruz COVID-19 Genomic Surveillance Network; Paola Resende; Renata Serrano Lopes; Taina Venas

EPI_ISL_3190174, EPI_ISL_3190175, EPI_ISL_3190176, EPI_ISL_3190178, EPI_ISL_3190179, EPI_ISL_3190180, EPI_ISL_3190181, EPI_ISL_3190182, EPI_ISL_3190183, EPI_ISL_3190184, EPI_ISL_3190185, EPI_ISL_3190186, EPI_ISL_3190187, EPI_ISL_3190188, EPI_ISL_3190189, EPI_ISL_3235238, EPI_ISL_3235239, EPI_ISL_3235240, EPI_ISL_3235241, EPI_ISL_3235242, EPI_ISL_3235243, EPI_ISL_3235244, EPI_ISL_3235245, EPI_ISL_3235246, EPI_ISL_3235247, EPI_ISL_3235248, EPI_ISL_3235249, EPI_ISL_3235250, EPI_ISL_3235251, EPI_ISL_3235252, EPI_ISL_3235253, EPI_ISL_3235254, EPI_ISL_3235255, EPI_ISL_3235256, EPI_ISL_3434726, EPI_ISL_3434759, EPI_ISL_3434760, EPI_ISL_3434761, EPI_ISL_3434762, EPI_ISL_3434763, EPI_ISL_3434764, EPI_ISL_3434765, EPI_ISL_3434766, EPI_ISL_3434767, EPI_ISL_3434768, EPI_ISL_3434769, EPI_ISL_3434770, EPI_ISL_3434771, EPI_ISL_3434772, EPI_ISL_3434773, EPI_ISL_3434774, EPI_ISL_3434775, EPI_ISL_3434776, EPI_ISL_3434777, EPI_ISL_3434778, EPI_ISL_3434779, EPI_ISL_3434780, EPI_ISL_3434781, EPI_ISL_3434782, EPI_ISL_3434783, EPI_ISL_3434784, EPI_ISL_3434785, EPI_ISL_3434786, EPI_ISL_3434787, EPI_ISL_3434788, EPI_ISL_3434789, EPI_ISL_3434802, EPI_ISL_3434905, EPI_ISL_3539936, EPI_ISL_3832350, EPI_ISL_3832351, EPI_ISL_3832352, EPI_ISL_3832353, EPI_ISL_3832354, EPI_ISL_3832355, EPI_ISL_3832356, EPI_ISL_3832357, EPI_ISL_3832358, EPI_ISL_3832359, EPI_ISL_3832360, EPI_ISL_3832361, EPI_ISL_3832362, EPI_ISL_3832363, EPI_ISL_3832364, EPI_ISL_3832365, EPI_ISL_3832394, EPI_ISL_3832397, EPI_ISL_4080930, EPI_ISL_4080936, EPI_ISL_4080937, EPI_ISL_4080938, EPI_ISL_4080939, EPI_ISL_4080940, EPI_ISL_4080941, EPI_ISL_4080942, EPI_ISL_4080943, EPI_ISL_4080944, EPI_ISL_4080945, EPI_ISL_4080946, EPI_ISL_4080947, EPI_ISL_4080948, EPI_ISL_4080949, EPI_ISL_4080950, EPI_ISL_4080951, EPI_ISL_4080952, EPI_ISL_4080953, EPI_ISL_4080954, EPI_ISL_4080955, EPI_ISL_4080956, EPI_ISL_4080957, EPI_ISL_4080958, EPI_ISL_4080959, EPI_ISL_4080960, EPI_ISL_4080961, EPI_ISL_4080962, EPI_ISL_4080963, EPI_ISL_4080964, EPI_ISL_4080965, EPI_ISL_4080966, EPI_ISL_4080967, EPI_ISL_4080968, EPI_ISL_4080969, EPI_ISL_4080970, EPI_ISL_4080971, EPI_ISL_4080972, EPI_ISL_4080973, EPI_ISL_4080974, EPI_ISL_4080975, EPI_ISL_4080976, EPI_ISL_4080977, EPI_ISL_4080978, EPI_ISL_4080979, EPI_ISL_4080980, EPI_ISL_4080981, EPI_ISL_4080982, EPI_ISL_4080983, EPI_ISL_4080984, EPI_ISL_4080985, EPI_ISL_4080986, EPI_ISL_4080987, EPI_ISL_4080988, EPI_ISL_4080989, EPI_ISL_4080990, EPI_ISL_4080991, EPI_ISL_4080992, EPI_ISL_4080993, EPI_ISL_4080994, EPI_ISL_4080995, EPI_ISL_4080996, EPI_ISL_4080997, EPI_ISL_4080998, EPI_ISL_4080999, EPI_ISL_4081000, EPI_ISL_4081001, EPI_ISL_4081002, EPI_ISL_4081003, EPI_ISL_4081004, EPI_ISL_4081005, EPI_ISL_4081006, EPI_ISL_4081007, EPI_ISL_4081008, EPI_ISL_4081009, EPI_ISL_4081010, EPI_ISL_4081011, EPI_ISL_4081012, EPI_ISL_4081013, EPI_ISL_4081014, EPI_ISL_4081015, EPI_ISL_4081016, EPI_ISL_4081017, EPI_ISL_4081018, EPI_ISL_4081019, EPI_ISL_4081020, EPI_ISL_4081021, EPI_ISL_4081022, EPI_ISL_4081023, EPI_ISL_4081024, EPI_ISL_4081025, EPI_ISL_4081026, EPI_ISL_4081027, EPI_ISL_4081069, EPI_ISL_4081070, EPI_ISL_4081071, EPI_ISL_4081072, EPI_ISL_4081073, EPI_ISL_4081074, EPI_ISL_4081075, EPI_ISL_4081076, EPI_ISL_4081077, EPI_ISL_4081078, EPI_ISL_4081079, EPI_ISL_4081080, EPI_ISL_4081081, EPI_ISL_4081082, EPI_ISL_4081083, EPI_ISL_4081084, EPI_ISL_4081085, EPI_ISL_4081086, EPI_ISL_4081087, EPI_ISL_4081088, EPI_ISL_4081089, EPI_ISL_4081091, EPI_ISL_4081092, EPI_ISL_4081093, EPI_ISL_4081094, EPI_ISL_4081095, EPI_ISL_4081097, EPI_ISL_4081098, EPI_ISL_4081099, EPI_ISL_4081100, EPI_ISL_4081101

see above	Laboratorio Central de Saude Publica do Estado da Paraiba (LACEN/PB)	Laboratory of Respiratory Viruses and Measles, Oswaldo Cruz Institute, FIOCRUZ	Alice Sampaio Rocha; Ana Carolina Mendonca; Anna Carolina Paixao; Dalane Loudal Florentino Teixeira; Elisa Cavalcante Pereira; Fernando Motta; Joao Felipe Bezerra; Luciana Appolinario; Marilda Siqueira on behalf of the Fiocruz COVID-19 Genomic Surveillance Network; Paola Resende; Renata Serrano Lopes; Taina Venas
EPI_ISL_2536354	Laboratorio Central de Saude Publica do Estado do Para (LACEN/PA)	Laboratory of Respiratory Viruses and Measles, Oswaldo Cruz Institute, FIOCRUZ	Alice Sampaio Rocha; Ana Carolina Mendonca; Anna Carolina Paixao; Elisa Cavalcante Pereira; Fernando Motta; Luciana Appolinario; Marilda Siqueira on behalf of the Fiocruz COVID-19 Genomic Surveillance Network; Paola Resende; Renata Serrano Lopes; Taina Venas; Valnete Andrade

EPI_ISL_2758939, EPI_ISL_2758940, EPI_ISL_2758942, EPI_ISL_2758945, EPI_ISL_2758947, EPI_ISL_2758948, EPI_ISL_2758949, EPI_ISL_2758950, EPI_ISL_2758951, EPI_ISL_2758952, EPI_ISL_2758956, EPI_ISL_2758958, EPI_ISL_2758964, EPI_ISL_2758990, EPI_ISL_2758998, EPI_ISL_2759007, EPI_ISL_2759010, EPI_ISL_2759013, EPI_ISL_2759023, EPI_ISL_2759027, EPI_ISL_2759029, EPI_ISL_2759032, EPI_ISL_2759036, EPI_ISL_2759038, EPI_ISL_2759039, EPI_ISL_2759042, EPI_ISL_2759044, EPI_ISL_2759053, EPI_ISL_2759058, EPI_ISL_2759064, EPI_ISL_2759069, EPI_ISL_2759070, EPI_ISL_2759071, EPI_ISL_2775416, EPI_ISL_2775417, EPI_ISL_2775423, EPI_ISL_2775424, EPI_ISL_2775425, EPI_ISL_2775426, EPI_ISL_2775427, EPI_ISL_2775428, EPI_ISL_2775429, EPI_ISL_2775430, EPI_ISL_2775431, EPI_ISL_2775432, EPI_ISL_2775433, EPI_ISL_2775434, EPI_ISL_2775435, EPI_ISL_2775436, EPI_ISL_2775437, EPI_ISL_2775438, EPI_ISL_2775439, EPI_ISL_2775440, EPI_ISL_2775441, EPI_ISL_2775442, EPI_ISL_2775443, EPI_ISL_2775444, EPI_ISL_2775445, EPI_ISL_2775446, EPI_ISL_2775447, EPI_ISL_2775448, EPI_ISL_2775449, EPI_ISL_2775450, EPI_ISL_2775451, EPI_ISL_2775452, EPI_ISL_2775453, EPI_ISL_2775454, EPI_ISL_2775455, EPI_ISL_2775456, EPI_ISL_2775457, EPI_ISL_2775458, EPI_ISL_2775459, EPI_ISL_2775460, EPI_ISL_2775461, EPI_ISL_2775462, EPI_ISL_2775463, EPI_ISL_2775464, EPI_ISL_2775465, EPI_ISL_2775466, EPI_ISL_2775467, EPI_ISL_2775468, EPI_ISL_2775469, EPI_ISL_2775470, EPI_ISL_2775471, EPI_ISL_2775472, EPI_ISL_2775473, EPI_ISL_2775474, EPI_ISL_2775475, EPI_ISL_2775476, EPI_ISL_2775477, EPI_ISL_2775478, EPI_ISL_2775479, EPI_ISL_2775480, EPI_ISL_2775481, EPI_ISL_2775482, EPI_ISL_2775483, EPI_ISL_2775485, EPI_ISL_2775486, EPI_ISL_2775487, EPI_ISL_2775488, EPI_ISL_2775489, EPI_ISL_2775490, EPI_ISL_2775491, EPI_ISL_2775492, EPI_ISL_2775493, EPI_ISL_2775494, EPI_ISL_2775495, EPI_ISL_2775496, EPI_ISL_2775497, EPI_ISL_2775498, EPI_ISL_2775499

see above	Laboratorio Central de Saude Publica do Estado do Parana (Instituto de	Instituto Carlos Chagas - Fiocruz	Alessandra De Melo Aguiar; Andreia Akemi Suzukawa; Andréa Rodrigues Ávila; Bruno Dallagiovanna; Dalila Zanette; Eduardo Balsanelli; Emanuel Maltempi de Souza; Fabio Passetti; Fabricio Klerynton Marchini; Fábio de Oliveira Pedrosa; Guilherme Becker; Helisson Faoro; Hellen Geremias dos Santos; Irina Nastassja Riediger; Letusa Albrecht; Lucas Blanes; Luis Gustavo Morello; Lysangela Ronalte Alves; Maria do Carmo Debur; Mauro de Medeiros Oliveira; Michelle Orane Schemberger; Paola Cristina Resende; Sheila Cristina Nardeli; Tiago Gräf; Valter Antônio de Baura

Biologia Molecular do Paraná (LAC)EN-PR)

EPI_ISL_3913651, EPI_ISL_3913656, EPI_ISL_3913660, EPI_ISL_3913664, EPI_ISL_3913669, EPI_ISL_3913672, EPI_ISL_3913676, EPI_ISL_3913681, EPI_ISL_3913685, EPI_ISL_3913690, EPI_ISL_3913694, EPI_ISL_3913698, EPI_ISL_3913703, EPI_ISL_3913707, EPI_ISL_3913712, EPI_ISL_3913715, EPI_ISL_3913720, EPI_ISL_3913724, EPI_ISL_3913729, EPI_ISL_3913733, EPI_ISL_3913738, EPI_ISL_3913741, EPI_ISL_3913746, EPI_ISL_3913751, EPI_ISL_3913755, EPI_ISL_3913759, EPI_ISL_3913764, EPI_ISL_3913767, EPI_ISL_3913772, EPI_ISL_3913775, EPI_ISL_3913780, EPI_ISL_3913784, EPI_ISL_3913789, EPI_ISL_3913792, EPI_ISL_3913797, EPI_ISL_3913801, EPI_ISL_3913806, EPI_ISL_3913809, EPI_ISL_3913815, EPI_ISL_3913818, EPI_ISL_3913823, EPI_ISL_3913827, EPI_ISL_3913832, EPI_ISL_3913836, EPI_ISL_3913841, EPI_ISL_3913845, EPI_ISL_3913849, EPI_ISL_3913854, EPI_ISL_3913858, EPI_ISL_3913862, EPI_ISL_3913866, EPI_ISL_3913870, EPI_ISL_3913874, EPI_ISL_3913879, EPI_ISL_3913882, EPI_ISL_3913891, EPI_ISL_3913896, EPI_ISL_3913900, EPI_ISL_3913905, EPI_ISL_3913909, EPI_ISL_3913913, EPI_ISL_3913917, EPI_ISL_3913921, EPI_ISL_3913925, EPI_ISL_3913930, EPI_ISL_3913934, EPI_ISL_3913938, EPI_ISL_3913943, EPI_ISL_3913947, EPI_ISL_3913951, EPI_ISL_3913955, EPI_ISL_3913960, EPI_ISL_3913964, EPI_ISL_3913969, EPI_ISL_3913973, EPI_ISL_3913977, EPI_ISL_3913981, EPI_ISL_3913985, EPI_ISL_3913989, EPI_ISL_3913994, EPI_ISL_3913997, EPI_ISL_3914002, EPI_ISL_3914007, EPI_ISL_3914010, EPI_ISL_3914014, EPI_ISL_3914019, EPI_ISL_3914023, EPI_ISL_3914027, EPI_ISL_3914032, EPI_ISL_3914036, EPI_ISL_3914040, EPI_ISL_3914045, EPI_ISL_3914048, EPI_ISL_3914053, EPI_ISL_3914056, EPI_ISL_3914061, EPI_ISL_3914064, EPI_ISL_3914069, EPI_ISL_3914073, EPI_ISL_3914078, EPI_ISL_3914082, EPI_ISL_3914090, EPI_ISL_3914095, EPI_ISL_3914099, EPI_ISL_3914103, EPI_ISL_3914107, EPI_ISL_3914111, EPI_ISL_3914116, EPI_ISL_3914119, EPI_ISL_3914124, EPI_ISL_3914129, EPI_ISL_3914134, EPI_ISL_3914138, EPI_ISL_3914142, EPI_ISL_3914147, EPI_ISL_3914150, EPI_ISL_3914155, EPI_ISL_3914158, EPI_ISL_3914163, EPI_ISL_3914166, EPI_ISL_3914171, EPI_ISL_3914175, EPI_ISL_3914179, EPI_ISL_3914184, EPI_ISL_3914193, EPI_ISL_3914198, EPI_ISL_3914202, EPI_ISL_3914206, EPI_ISL_3914211, EPI_ISL_3914214, EPI_ISL_3914219, EPI_ISL_3914223, EPI_ISL_3914228, EPI_ISL_3914232, EPI_ISL_3914236, EPI_ISL_3914240, EPI_ISL_3914244, EPI_ISL_3914249, EPI_ISL_3914254, EPI_ISL_3914258, EPI_ISL_3914262, EPI_ISL_3914267, EPI_ISL_3914271, EPI_ISL_3914275, EPI_ISL_3914279, EPI_ISL_3914282, EPI_ISL_3914287, EPI_ISL_3914291, EPI_ISL_3914296, EPI_ISL_3914300, EPI_ISL_3914304, EPI_ISL_3914308, EPI_ISL_3914313, EPI_ISL_3914317, EPI_ISL_3914321, EPI_ISL_3914326, EPI_ISL_3914331, EPI_ISL_3914334, EPI_ISL_3914339, EPI_ISL_3914342, EPI_ISL_3914347, EPI_ISL_3914351, EPI_ISL_3914355, EPI_ISL_3914359, EPI_ISL_3914363, EPI_ISL_3914368, EPI_ISL_3914372, EPI_ISL_3914381, EPI_ISL_3914384, EPI_ISL_3914389, EPI_ISL_3914394, EPI_ISL_3914397, EPI_ISL_3914402, EPI_ISL_3914407, EPI_ISL_3914410, EPI_ISL_3914415, EPI_ISL_3914418, EPI_ISL_3914423, EPI_ISL_3914427, EPI_ISL_3914430, EPI_ISL_3914434

see above | Laboratorio Central de Saude Publica do Estado do Parana (Instituto de Biologia Molecular do Paraná (LACEN-PR) | Instituto Carlos Chagas - Fiocruz | Alessandra De Melo Aguiar; Andreia Akemi Suzukawa; Andréa Rodrigues Ávila; Bruno Dallagiovanna; Dalila Zanette; Eduardo Balsanelli; Emanuel Maltempi de Souza; Fabio Passetti; Fabricio Klerynton Marchini; Fábio de Oliveira Pedrosa; Guilherme Becker; Helisson Faoro; Hellen Geremias dos Santos; Irina Nastassja Riediger; Letusa Albrecht; Lucas Blanes; Luis Gustavo Morello; Lysangela Ronalte Alves; Maria do Carmo Debur; Mauro de Medeiros Oliveira; Michelle Orane Schemberger; Paola Cristina Resende; Sheila Cristina Nardeli; Tiago Gräf; Valter Antônio de Baura

EPI_ISL_1181390, EPI_ISL_1181392, EPI_ISL_1181394, EPI_ISL_1181474, EPI_ISL_1181498, EPI_ISL_1181517, EPI_ISL_1181526, EPI_ISL_1181527, EPI_ISL_1181528, EPI_ISL_1181530, EPI_ISL_1181533, EPI_ISL_1181534, EPI_ISL_1181536, EPI_ISL_1181537, EPI_ISL_1181538, EPI_ISL_1181539, EPI_ISL_1181540, EPI_ISL_1181541, EPI_ISL_1181542, EPI_ISL_1181543, EPI_ISL_1181544, EPI_ISL_1181545, EPI_ISL_1181551, EPI_ISL_1181552, EPI_ISL_1181553, EPI_ISL_1181554, EPI_ISL_1181555, EPI_ISL_1181556, EPI_ISL_1181557, EPI_ISL_1181558, EPI_ISL_1181559, EPI_ISL_1181563, EPI_ISL_1181564, EPI_ISL_1181565, EPI_ISL_1181566, EPI_ISL_1181568, EPI_ISL_1181570, EPI_ISL_1181576, EPI_ISL_1181580, EPI_ISL_1181581, EPI_ISL_1181584, EPI_ISL_1181605, EPI_ISL_1181609, EPI_ISL_1181610, EPI_ISL_1181614, EPI_ISL_1181615, EPI_ISL_1181616, EPI_ISL_1181617, EPI_ISL_1181618, EPI_ISL_1181619, EPI_ISL_1181623, EPI_ISL_1219132, EPI_ISL_1219133, EPI_ISL_1533978, EPI_ISL_1533999, EPI_ISL_1534000, EPI_ISL_1534001, EPI_ISL_2038955

see above | Laboratorio Central de Saude Publica do Estado do Parana (LACEN-PR) | Laboratory of Respiratory Viruses and Measles, Oswaldo Cruz Institute, FIOCRUZ | Alice Sampaio Rocha; Ana Carolina Mendonca; Anna Carolina Paixao; Elisa Cavalcante Pereira; Fernando Motta; Irina Nastassja Riediger; Irina Riediger; Luciana Appolinario; Maria do Carmo Debur; Marilda Siqueira on behalf of the Fiocruz COVID-19 Genomic Surveillance Network; Paola Resende; Renata Serrano Lopes; Taina Venas

EPI_ISL_2157482, EPI_ISL_2645445, EPI_ISL_2645446, EPI_ISL_2645447, EPI_ISL_2645448, EPI_ISL_2645449, EPI_ISL_2645450, EPI_ISL_2645451, EPI_ISL_2645452, EPI_ISL_2645453, EPI_ISL_2645454, EPI_ISL_2645455, EPI_ISL_2645456, EPI_ISL_2645457, EPI_ISL_2645458, EPI_ISL_2645459, EPI_ISL_2645460, EPI_ISL_2645461, EPI_ISL_2645462, EPI_ISL_2645463, EPI_ISL_2645464, EPI_ISL_2645465, EPI_ISL_2645466, EPI_ISL_2645467, EPI_ISL_2645468, EPI_ISL_2645469, EPI_ISL_2645470, EPI_ISL_2645471, EPI_ISL_2645472, EPI_ISL_2645473, EPI_ISL_2645474, EPI_ISL_2645475, EPI_ISL_2645476, EPI_ISL_2645477, EPI_ISL_2645478, EPI_ISL_2645479, EPI_ISL_2645480, EPI_ISL_2645481, EPI_ISL_2645482, EPI_ISL_2645483, EPI_ISL_2645484, EPI_ISL_2645485, EPI_ISL_2645486, EPI_ISL_2645487, EPI_ISL_2645491, EPI_ISL_2645492, EPI_ISL_2645493, EPI_ISL_2645494, EPI_ISL_2645495, EPI_ISL_2645496, EPI_ISL_2645497, EPI_ISL_2645498, EPI_ISL_2645499, EPI_ISL_2645500, EPI_ISL_2645501, EPI_ISL_2645502, EPI_ISL_2645503, EPI_ISL_2645504, EPI_ISL_2731458, EPI_ISL_2731459, EPI_ISL_2731461, EPI_ISL_2731466, EPI_ISL_2731467, EPI_ISL_2983373, EPI_ISL_2983426, EPI_ISL_2983427, EPI_ISL_2983428, EPI_ISL_2983430, EPI_ISL_2983431, EPI_ISL_2983432, EPI_ISL_2983433, EPI_ISL_2983434, EPI_ISL_2983435, EPI_ISL_2983437, EPI_ISL_2983438, EPI_ISL_2983439, EPI_ISL_2983440, EPI_ISL_2983441, EPI_ISL_2983442, EPI_ISL_2983443, EPI_ISL_2983444, EPI_ISL_2983445, EPI_ISL_2983446, EPI_ISL_2983447, EPI_ISL_2983448, EPI_ISL_2983449, EPI_ISL_2983450, EPI_ISL_2983451, EPI_ISL_2983452, EPI_ISL_3061892, EPI_ISL_3061893, EPI_ISL_3190190, EPI_ISL_3190191, EPI_ISL_3190192, EPI_ISL_3190264, EPI_ISL_3190266, EPI_ISL_3190267, EPI_ISL_3190268, EPI_ISL_3190360, EPI_ISL_3190361, EPI_ISL_3190362, EPI_ISL_3190363, EPI_ISL_3190364, EPI_ISL_3190365, EPI_ISL_3190366, EPI_ISL_3190367, EPI_ISL_3190368, EPI_ISL_3190369, EPI_ISL_3190370, EPI_ISL_3190371, EPI_ISL_3190372, EPI_ISL_3190373, EPI_ISL_3190374, EPI_ISL_3190375, EPI_ISL_3190376, EPI_ISL_3190377, EPI_ISL_3190378, EPI_ISL_3190379, EPI_ISL_3190380, EPI_ISL_3190381, EPI_ISL_3190382, EPI_ISL_3190383, EPI_ISL_3190384, EPI_ISL_3190385, EPI_ISL_3190386, EPI_ISL_3190387, EPI_ISL_3190388, EPI_ISL_3190389, EPI_ISL_3190390, EPI_ISL_3190391, EPI_ISL_3235257, EPI_ISL_3243095, EPI_ISL_3243096, EPI_ISL_3243097, EPI_ISL_3243098, EPI_ISL_3243099, EPI_ISL_3243100, EPI_ISL_3243101, EPI_ISL_3243102, EPI_ISL_3243103, EPI_ISL_3243104, EPI_ISL_3243105, EPI_ISL_3243106, EPI_ISL_3243107, EPI_ISL_3243108, EPI_ISL_3243109, EPI_ISL_3243110, EPI_ISL_3243111, EPI_ISL_3243112, EPI_ISL_3435048, EPI_ISL_3540001, EPI_ISL_3540002, EPI_ISL_3801799, EPI_ISL_3801800, EPI_ISL_3801801, EPI_ISL_3801802, EPI_ISL_3801823, EPI_ISL_3801824, EPI_ISL_3801825, EPI_ISL_3801839, EPI_ISL_3801840, EPI_ISL_3801841, EPI_ISL_3801842, EPI_ISL_3801843, EPI_ISL_3801844, EPI_ISL_3801852, EPI_ISL_3801853, EPI_ISL_3801854, EPI_ISL_3801855, EPI_ISL_3801856, EPI_ISL_3801857, EPI_ISL_3801858, EPI_ISL_3801859, EPI_ISL_3801860, EPI_ISL_3801861, EPI_ISL_3801862, EPI_ISL_3801863, EPI_ISL_3801864, EPI_ISL_3801865, EPI_ISL_3801866, EPI_ISL_3801867, EPI_ISL_3801868, EPI_ISL_3801869, EPI_ISL_3801870, EPI_ISL_3801875, EPI_ISL_3801920, EPI_ISL_3801921, EPI_ISL_3801922, EPI_ISL_3801923, EPI_ISL_3801925, EPI_ISL_3801926, EPI_ISL_3801927, EPI_ISL_3801928, EPI_ISL_3801929, EPI_ISL_3801930, EPI_ISL_3801931, EPI_ISL_3802976, EPI_ISL_3802977, EPI_ISL_3827988, EPI_ISL_3827989, EPI_ISL_3827992, EPI_ISL_3828024, EPI_ISL_3828025, EPI_ISL_3828026, EPI_ISL_3832366, EPI_ISL_3832367, EPI_ISL_3832368, EPI_ISL_3832369, EPI_ISL_3832370, EPI_ISL_3832371, EPI_ISL_3832372, EPI_ISL_3832373, EPI_ISL_3832374, EPI_ISL_3832375, EPI_ISL_3832376, EPI_ISL_3832377, EPI_ISL_3832378, EPI_ISL_3832379, EPI_ISL_3832380, EPI_ISL_3832381, EPI_ISL_3832382, EPI_ISL_3832393, EPI_ISL_4061328, EPI_ISL_4061329, EPI_ISL_4061330, EPI_ISL_4061424, EPI_ISL_4061425, EPI_ISL_4061426, EPI_ISL_4061427, EPI_ISL_4061428, EPI_ISL_4061429, EPI_ISL_4061430, EPI_ISL_4061431, EPI_ISL_4061432, EPI_ISL_4061433, EPI_ISL_4061437, EPI_ISL_4061438

see above | Laboratorio Central de Saude Publica do Estado do Parana (LACEN/PR) | Laboratory of Respiratory Viruses and Measles, Oswaldo Cruz Institute, FIOCRUZ | Agatha Soares; Alice Sampaio Rocha; Ana Carolina Mendonca; Anna Carolina Paixao; Elisa Cavalcante Pereira; Fernando Motta; Ighor Arantes; Ighor Leonardo Arantes; Irina Riediger; Luciana Appolinario; Marilda Siqueira on behalf of the Fiocruz COVID-19 Genomic Surveillance Network; Paola Resende; Renata Serrano Lopes; Taina Venas

EPI_ISL_3997110, EPI_ISL_3997227, EPI_ISL_3997228, EPI_ISL_3997230, EPI_ISL_3997240, EPI_ISL_3997739, EPI_ISL_3997833, EPI_ISL_3997877, EPI_ISL_3998018, EPI_ISL_3998051, EPI_ISL_3998169, EPI_ISL_3999620, EPI_ISL_4000067, EPI_ISL_4000069, EPI_ISL_4000229, EPI_ISL_4000270, EPI_ISL_4000384, EPI_ISL_4000695, EPI_ISL_4000717, EPI_ISL_4000718, EPI_ISL_4000719, EPI_ISL_4000801, EPI_ISL_4000802, EPI_ISL_4000803, EPI_ISL_4000804, EPI_ISL_4001402, EPI_ISL_4001403, EPI_ISL_4001404, EPI_ISL_4001405, EPI_ISL_4001562, EPI_ISL_4001708, EPI_ISL_4009398

see above | Laboratorio Central do Estado do Parana | Laboratorio Central do Estado do Parana | Guilherme Nardi Becker; Irina Nastassja Riediger; Maria do Carmo Debur Rossa; Mayra Presibella Giacomini; Nelson Quallio Marques

EPI_ISL_1402429, EPI_ISL_2731462, EPI_ISL_2731463, EPI_ISL_2731464, EPI_ISL_2731465 | Laboratory of Respiratory Viruses and Measles, Oswaldo Cruz Institute, FIOCRUZ | Laboratory of Respiratory Viruses and Measles, Oswaldo Cruz Institute, FIOCRUZ | Alex Pauvolid-Correa; Alice Sampaio Rocha; Ana Beatriz Machado Lima; Ana Carolina Mendonca; Anna Carolina Paixao; Elisa Cavalcante Pereira; Fernando Motta; Luciana Appolinario; Marilda Siqueira on behalf of the Fiocruz COVID-19 Genomic Surveillance Network; Mia Ferreira Araujo; Paola Resende; Renata Serrano Lopes; Taina Venas

EPI_ISL_2157506, EPI_ISL_2157516, EPI_ISL_2157517 | Laboratório Central de Saude Publica do Estado do Paraná (LACEN/PR) | Laboratory of Respiratory Viruses and Measles, Oswaldo Cruz Institute, FIOCRUZ | Alice Sampaio Rocha; Ana Carolina Mendonca; Anna Carolina Paixao; Elisa Cavalcante Pereira; Fernando Motta; Irina Nastassja Riediger; Luciana Appolinario; Maria do Carmo Debur; Marilda Siqueira on behalf of the Fiocruz COVID-19 Genomic Surveillance Network; Paola Resende; Renata Serrano Lopes; Taina Venas

EPI_ISL_2241495, EPI_ISL_2241496, EPI_ISL_2241497, EPI_ISL_2241498, EPI_ISL_2241499, EPI_ISL_2241514, EPI_ISL_2241516, EPI_ISL_2241517, EPI_ISL_2241518, EPI_ISL_2241519, EPI_ISL_2241520, EPI_ISL_2241522, EPI_ISL_2241523, EPI_ISL_2241525, EPI_ISL_2241526, EPI_ISL_2241527, EPI_ISL_2241529, EPI_ISL_2241556, EPI_ISL_2241558, EPI_ISL_2241559, EPI_ISL_2241561, EPI_ISL_2241562, EPI_ISL_2241563, EPI_ISL_2241564, EPI_ISL_2241566, EPI_ISL_2241567, EPI_ISL_2241568, EPI_ISL_2241569, EPI_ISL_2241570, EPI_ISL_2241571, EPI_ISL_2241572, EPI_ISL_2241573, EPI_ISL_2241576, EPI_ISL_2241581, EPI_ISL_2241583, EPI_ISL_2241585, EPI_ISL_2241586, EPI_ISL_2241588, EPI_ISL_2241594, EPI_ISL_2241596, EPI_ISL_2246285

see above | Laboratório Central de Saúde Pública da Paraiba | Coordenação Geral de Laboratórios de Saúde Pública (CGLAB/DAEVS/SVS/MS) | Vagner Fonseca; et al.

EPI_ISL_792561, EPI_ISL_792562, EPI_ISL_792563, EPI_ISL_792564, EPI_ISL_792565, EPI_ISL_792566, EPI_ISL_792567, EPI_ISL_792568, EPI_ISL_792569, EPI_ISL_792570, EPI_ISL_792571, EPI_ISL_792572, EPI_ISL_792573, EPI_ISL_792574, EPI_ISL_792575, EPI_ISL_792576, EPI_ISL_792577, EPI_ISL_792578, EPI_ISL_792579, EPI_ISL_792580, EPI_ISL_792581, EPI_ISL_792582, EPI_ISL_792583, EPI_ISL_792584, EPI_ISL_792585, EPI_ISL_792586, EPI_ISL_792587, EPI_ISL_792588, EPI_ISL_792589, EPI_ISL_792590, EPI_ISL_792591, EPI_ISL_792592, EPI_ISL_792593, EPI_ISL_792594, EPI_ISL_792595, EPI_ISL_792596, EPI_ISL_792597, EPI_ISL_792598, EPI_ISL_792599, EPI_ISL_792600, EPI_ISL_792601, EPI_ISL_792602, EPI_ISL_792603, EPI_ISL_792604, EPI_ISL_792605, EPI_ISL_792606, EPI_ISL_792607, EPI_ISL_792608, EPI_ISL_792609, EPI_ISL_792610, EPI_ISL_792611, EPI_ISL_792612, EPI_ISL_792613, EPI_ISL_792614, EPI_ISL_792615, EPI_ISL_792616, EPI_ISL_792617, EPI_ISL_792618, EPI_ISL_792619, EPI_ISL_792620, EPI_ISL_792621, EPI_ISL_792622, EPI_ISL_792623, EPI_ISL_792624, EPI_ISL_792625, EPI_ISL_792626, EPI_ISL_792627, EPI_ISL_792628, EPI_ISL_792629, EPI_ISL_792630, EPI_ISL_792631, EPI_ISL_792632, EPI_ISL_792633, EPI_ISL_792634, EPI_ISL_792635, EPI_ISL_792636, EPI_ISL_792637, EPI_ISL_792638

see above | Laboratório Central de Saúde Pública do Estado da Paraíba (LACEN-PB) | Laboratory of Respiratory Viruses and Measles, Oswaldo Cruz Institute, FIOCRUZ | Ana Carolina Mendonca; Anna Carolina Paixao; Dalane Loudal Florentino Teixeira; Fernando Motta; João Felipe Bezerra; Luciana Appolinario; Marilda Siqueira on behalf of the Fiocruz COVID-19 Genomic Surveillance Network; Paola Resende; Romero Henrique Teixeira de Vasconcelos; Thiago Franco de Oliveira Carneiro

EPI_ISL_541340, EPI_ISL_541341, EPI_ISL_541342, EPI_ISL_541343, EPI_ISL_541344, EPI_ISL_541345, EPI_ISL_541346, EPI_ISL_792645, EPI_ISL_792646, EPI_ISL_792647, EPI_ISL_792648, EPI_ISL_792649, EPI_ISL_792650, EPI_ISL_792651, EPI_ISL_792652, EPI_ISL_792653, EPI_ISL_792654

see above | Laboratório Central de Saúde Pública do Estado do Paraná (LACEN-PR) | Laboratory of Respiratory Viruses and Measles, Oswaldo Cruz Institute, FIOCRUZ | Ana Carolina Mendonca; Ana Carolina Mendonça; Anna Carolina Paixao; Anna Carolina Paixão; Fernando Motta; Irina Nastassja Riediger; Irina Riediger; Jonathan Lopes; Luciana Appolinario; Maria do Carmo Debur; Marilda Siqueira on behalf of the Fiocruz COVID-19 Genomic Surveillance Network; Paola Resende

EPI_ISL_2249446, EPI_ISL_2249447, EPI_ISL_2249448, EPI_ISL_2249449, EPI_ISL_2249450, EPI_ISL_2249451, EPI_ISL_2249452, EPI_ISL_2249453, EPI_ISL_2249454, EPI_ISL_2249455, EPI_ISL_2249456, EPI_ISL_2249457

see above | Laboratório | Coordenação Geral de | Vagner Fonseca; et al.

	Central de Saúde Pública do Paraá	Laboratórios de Saúde Pública (CGLAB/DAEVS/SVS/MS)	
EPI_ISL_2245087, EPI_ISL_2245089, EPI_ISL_2245090, EPI_ISL_2245092, EPI_ISL_2245093, EPI_ISL_2245094, EPI_ISL_2245095, EPI_ISL_2245098, EPI_ISL_2245101, EPI_ISL_2245102, EPI_ISL_2245103, EPI_ISL_2245104, EPI_ISL_2245105, EPI_ISL_2248758, EPI_ISL_2248759, EPI_ISL_2248760, EPI_ISL_2248761, EPI_ISL_2248762, EPI_ISL_2248763, EPI_ISL_2248764, EPI_ISL_2248765, EPI_ISL_2248766, EPI_ISL_2248767, EPI_ISL_2248782, EPI_ISL_2248783, EPI_ISL_2298758, EPI_ISL_2298783, EPI_ISL_2298787, EPI_ISL_2298810, EPI_ISL_2298812, EPI_ISL_2298814			
see above	Laboratório Central de Saúde Pública do Pará	Coordenação Geral de Laboratórios de Saúde Pública (CGLAB/DAEVS/SVS/MS)	Vagner Fonseca; et al.
EPI_ISL_1182546, EPI_ISL_1182562, EPI_ISL_1182563, EPI_ISL_1182565, EPI_ISL_1182567, EPI_ISL_1182568, EPI_ISL_1182571, EPI_ISL_1182572, EPI_ISL_1182575, EPI_ISL_1182576, EPI_ISL_1182580, EPI_ISL_1182581, EPI_ISL_1182582, EPI_ISL_1182583, EPI_ISL_1182584, EPI_ISL_1182588, EPI_ISL_1182589, EPI_ISL_1182592, EPI_ISL_1182594, EPI_ISL_1182595, EPI_ISL_1182596, EPI_ISL_1182603, EPI_ISL_1182604, EPI_ISL_1182613, EPI_ISL_1182614, EPI_ISL_1182617, EPI_ISL_1182622, EPI_ISL_1182624			
see above	Laboratório Central do Estado do Paraná	Coordenação Geral de Laboratórios de Saúde Pública (CGLAB/DAEVS/SVS/MS)	Vagner Fonseca; et al.
EPI_ISL_1133256	Laboratório Hermes Pardini	Laboratório de Biologia Integrativa, Instituto de Ciências Biológicas, Universidade Federal de Minas Gerais	Alessandro Clayton de Souza Ferreira; Aline Brito de Lima; Carolina Moreira Voloch; Daniel Costa Queiroz; Danielle Alves Gomes Zauli; Diego Menezes Bonfim; Filipe Romero Rebello Moreira; Frederico Scott Varella Malta; Joice do Prado Silva; Rafael Marques de Souza; Renan Pedra de Souza; Renato Santana Aguiar.; Rennan Garcias Moreira; Victor Cavalcanti Pardini; Victor Emmanuel Viana Geddes; Wagner Carlos Santos Magalhaes; Walyson Coelho Costa
EPI_ISL_1261691	Laboratório Paulo C. Azevedo	Evandro Chagas Institute	A.M.; Barbagelata; E.C.; E.M.A.; Ferreira; J.A.; Junior; K.C.; L.C.; L.S.; M.C.; P.S.; Pinheiro; Santos; Silva; Sousa; Sousa Junior; W.D.C.; da Silva
EPI_ISL_2157343, EPI_ISL_2157386, EPI_ISL_2157387, EPI_ISL_2157388, EPI_ISL_2157397, EPI_ISL_2157402, EPI_ISL_2157403, EPI_ISL_2157404, EPI_ISL_2157408, EPI_ISL_2157413, EPI_ISL_2157416, EPI_ISL_2157417, EPI_ISL_2157419, EPI_ISL_2157420, EPI_ISL_2157421, EPI_ISL_2157424, EPI_ISL_2157431, EPI_ISL_2157452, EPI_ISL_2157479, EPI_ISL_2157483, EPI_ISL_2157485, EPI_ISL_2157486, EPI_ISL_2157487, EPI_ISL_2157488, EPI_ISL_2157492, EPI_ISL_2157496, EPI_ISL_2157504, EPI_ISL_2157527, EPI_ISL_2157593, EPI_ISL_2157595, EPI_ISL_2157597, EPI_ISL_2196244, EPI_ISL_2196252, EPI_ISL_2196259, EPI_ISL_2196306, EPI_ISL_2196307, EPI_ISL_2196308, EPI_ISL_2196309, EPI_ISL_2196310, EPI_ISL_2196311, EPI_ISL_2196312, EPI_ISL_2196362, EPI_ISL_2274094, EPI_ISL_2274095, EPI_ISL_2274096, EPI_ISL_2443528, EPI_ISL_2443530, EPI_ISL_2443531, EPI_ISL_2443532, EPI_ISL_2443533, EPI_ISL_2443534, EPI_ISL_2443535, EPI_ISL_2443536, EPI_ISL_2443537, EPI_ISL_2443538, EPI_ISL_2443539, EPI_ISL_2443540, EPI_ISL_2443541, EPI_ISL_2443542, EPI_ISL_2443543, EPI_ISL_2443544, EPI_ISL_2443545, EPI_ISL_2443546, EPI_ISL_2443547, EPI_ISL_2443548, EPI_ISL_2443549, EPI_ISL_2443550, EPI_ISL_2443553, EPI_ISL_2443554, EPI_ISL_2443565, EPI_ISL_2443566, EPI_ISL_2443567, EPI_ISL_2443568, EPI_ISL_2443569, EPI_ISL_2443570, EPI_ISL_2443571, EPI_ISL_2443572, EPI_ISL_2443573, EPI_ISL_2443574, EPI_ISL_2443575, EPI_ISL_2443576, EPI_ISL_2443577, EPI_ISL_2557359, EPI_ISL_2557360, EPI_ISL_2557361, EPI_ISL_2557362, EPI_ISL_2557363, EPI_ISL_2557364, EPI_ISL_2557365, EPI_ISL_2557366, EPI_ISL_2557367, EPI_ISL_2557368, EPI_ISL_2557369, EPI_ISL_2557370, EPI_ISL_2557371, EPI_ISL_2557372, EPI_ISL_2557373, EPI_ISL_2557374, EPI_ISL_2557375, EPI_ISL_2603424, EPI_ISL_2603425, EPI_ISL_2603426, EPI_ISL_2603427, EPI_ISL_2603428, EPI_ISL_2603429, EPI_ISL_2603430, EPI_ISL_2603431, EPI_ISL_2603437, EPI_ISL_2603438, EPI_ISL_2603439, EPI_ISL_2603440, EPI_ISL_2603451, EPI_ISL_2603452, EPI_ISL_2603453, EPI_ISL_2603454, EPI_ISL_2603455, EPI_ISL_2603456, EPI_ISL_2603457, EPI_ISL_2603458, EPI_ISL_2603459, EPI_ISL_2603460, EPI_ISL_2603461, EPI_ISL_2603462, EPI_ISL_2603463, EPI_ISL_2603464, EPI_ISL_2603465, EPI_ISL_2603466, EPI_ISL_2603467, EPI_ISL_2603468, EPI_ISL_2603469, EPI_ISL_2603470, EPI_ISL_2603471, EPI_ISL_2603543, EPI_ISL_2603544, EPI_ISL_2603545, EPI_ISL_2603546, EPI_ISL_2613809, EPI_ISL_2613810, EPI_ISL_2613811, EPI_ISL_2613812, EPI_ISL_2613813, EPI_ISL_2613814, EPI_ISL_2613815, EPI_ISL_2613816, EPI_ISL_2613817, EPI_ISL_2613819, EPI_ISL_2613820, EPI_ISL_2613821, EPI_ISL_2613822, EPI_ISL_2613823, EPI_ISL_2613824, EPI_ISL_2645440, EPI_ISL_2645441, EPI_ISL_2645442, EPI_ISL_2645443, EPI_ISL_2645488, EPI_ISL_2645489, EPI_ISL_2645490, EPI_ISL_2863571, EPI_ISL_2863576, EPI_ISL_2863682, EPI_ISL_2863684, EPI_ISL_2863685, EPI_ISL_2863687, EPI_ISL_2863725, EPI_ISL_2863795, EPI_ISL_2863855, EPI_ISL_2863856, EPI_ISL_2863857, EPI_ISL_2863858, EPI_ISL_2863859, EPI_ISL_2863860, EPI_ISL_2863861, EPI_ISL_2863862, EPI_ISL_2863863, EPI_ISL_2863864, EPI_ISL_2863865, EPI_ISL_2863866, EPI_ISL_2863867, EPI_ISL_2863868, EPI_ISL_2863869, EPI_ISL_2863870, EPI_ISL_2863871, EPI_ISL_2863872, EPI_ISL_2863873, EPI_ISL_2863874, EPI_ISL_2863875, EPI_ISL_2863876, EPI_ISL_2863877, EPI_ISL_2863878, EPI_ISL_2863879, EPI_ISL_2863880, EPI_ISL_2863881, EPI_ISL_2863882, EPI_ISL_2863883, EPI_ISL_2863884, EPI_ISL_2863885, EPI_ISL_2863886, EPI_ISL_2863887, EPI_ISL_2863888, EPI_ISL_2863889, EPI_ISL_2863890, EPI_ISL_2863893, EPI_ISL_2863895, EPI_ISL_2863896, EPI_ISL_2863898, EPI_ISL_2863899			
see above	Lboratorio Central de Saude Publica do Estado do Parana (LACEN/PR)	Laboratory of Respiratory Viruses and Measles, Oswaldo Cruz Institute, FIOCRUZ	Agatha Cristinne Prudencio; Agatha Cristinne Prudencio Soares; Alice Sampaio Rocha; Ana Carolina Mendonca; Anna Carolina Paixao; Elisa Cavalcante Pereira; Fernando Motta; Ighor Leonardo Arantes Gomes; Irina Riediger; Luciana Appolinario; Marilda Siqueira on behalf of the Fiocruz COVID-19 Genomic Surveillance Network; Paola Resende; Renata Serrano Lopes; Taina Venas
EPI_ISL_510535	Molecular Virology, Instituto Carlos Chagas / Fiocruz Paraná	Universidade Federal do Parana (UFPR)	Duarte dos Santos, C.; Raboni, S.; Suzukawa, A.; Tscha, M.; Zanluca, C.
EPI_ISL_3982748, EPI_ISL_3982751, EPI_ISL_3982756, EPI_ISL_3982761, EPI_ISL_3982764, EPI_ISL_3982767, EPI_ISL_3982769, EPI_ISL_3982771			
see above	Parana	LACEN PR	Guilherme Nardi Becker; Irina Nastassja Riediger; Maria do Carmo Debur Rossa; Mayra Presibella Giacomini; Nelson Quallio Marques
EPI_ISL_3982759, EPI_ISL_3982768	Parana	Lacen-PR	Guilherme Nardi Becker; Irina Nastassja Riediger; Maria do Carmo Debur Rossa; Mayra Presibella Giacomini; Nelson Quallio Marques
EPI_ISL_3982749	Parana	Lacen/PR	Guilherme Nardi Becker; Irina Nastassja Riediger; Maria do Carmo Debur Rossa; Mayra Presibella Giacomini; Nelson Quallio Marques
EPI_ISL_3982745, EPI_ISL_3982763	Parana	Parana	Guilherme Nardi Becker; Irina Nastassja Riediger; Maria do Carmo Debur Rossa; Mayra Presibella Giacomini; Nelson Quallio Marques
EPI_ISL_2758648, EPI_ISL_2758649, EPI_ISL_2758650, EPI_ISL_2758651, EPI_ISL_2758652, EPI_ISL_2758653, EPI_ISL_2758654, EPI_ISL_2758655, EPI_ISL_2758656, EPI_ISL_2758657, EPI_ISL_2758658, EPI_ISL_2758659, EPI_ISL_2758660, EPI_ISL_2758661, EPI_ISL_2758662, EPI_ISL_2758663, EPI_ISL_2758664, EPI_ISL_2758665, EPI_ISL_2758666, EPI_ISL_2758667, EPI_ISL_2758673, EPI_ISL_2758674, EPI_ISL_2758677, EPI_ISL_2758678, EPI_ISL_2758679, EPI_ISL_2758680, EPI_ISL_2758681, EPI_ISL_2758682, EPI_ISL_2758683, EPI_ISL_2758684, EPI_ISL_2758685, EPI_ISL_2758686, EPI_ISL_2758687, EPI_ISL_2758688, EPI_ISL_2758689, EPI_ISL_2758690, EPI_ISL_2758691, EPI_ISL_2758693, EPI_ISL_2758694, EPI_ISL_2758695, EPI_ISL_2758696, EPI_ISL_2758697, EPI_ISL_2758701, EPI_ISL_2758705, EPI_ISL_2758713, EPI_ISL_2758714, EPI_ISL_2758715, EPI_ISL_2758716, EPI_ISL_2758717, EPI_ISL_2758718, EPI_ISL_2758719, EPI_ISL_2758720, EPI_ISL_2758721, EPI_ISL_2758722, EPI_ISL_2758723, EPI_ISL_2758724, EPI_ISL_2758725, EPI_ISL_2758726, EPI_ISL_2758727, EPI_ISL_2758728, EPI_ISL_2758729, EPI_ISL_2758730, EPI_ISL_2758731, EPI_ISL_2758732, EPI_ISL_2758733, EPI_ISL_2758734, EPI_ISL_2758735, EPI_ISL_2758736, EPI_ISL_2758737, EPI_ISL_2758738, EPI_ISL_2758739, EPI_ISL_2758740, EPI_ISL_2758741, EPI_ISL_2758742, EPI_ISL_2758743, EPI_ISL_2758744, EPI_ISL_2758745, EPI_ISL_2758746, EPI_ISL_2758747, EPI_ISL_2758748, EPI_ISL_2758749, EPI_ISL_2758750, EPI_ISL_2758751, EPI_ISL_2758752, EPI_ISL_2758753, EPI_ISL_2758754, EPI_ISL_2758755, EPI_ISL_2758756, EPI_ISL_2758757, EPI_ISL_2758758, EPI_ISL_2758759, EPI_ISL_2758760, EPI_ISL_2758761, EPI_ISL_2758762, EPI_ISL_2758763, EPI_ISL_2758764, EPI_ISL_2758765, EPI_ISL_2758766, EPI_ISL_2758776, EPI_ISL_2758777, EPI_ISL_2758778, EPI_ISL_2758779, EPI_ISL_2758780, EPI_ISL_2758781, EPI_ISL_2758782, EPI_ISL_2758783, EPI_ISL_2758784, EPI_ISL_2758785, EPI_ISL_2758786, EPI_ISL_2758787, EPI_ISL_2758788, EPI_ISL_2758789, EPI_ISL_2758790, EPI_ISL_2758791, EPI_ISL_2758792, EPI_ISL_2758793, EPI_ISL_2758794, EPI_ISL_2758795, EPI_ISL_2758796, EPI_ISL_2758799, EPI_ISL_2758801, EPI_ISL_2758802, EPI_ISL_2758803, EPI_ISL_2758804, EPI_ISL_2758805			
see above	UEL	IPEC Guarapuava	NAPI-Genômica (Novos Arranjo de Pesquisa e Inovação em Genômica): Ademar Dantas da Cunha Júnior Adriano Ferrasa Adriano Mondini Aldo Przybysz Alessandra Lourenço Cecchini Armani Alex Sandro Jorge Alexandra Ivo de Medeiros Alexandre Maller Aline Cristina Batista Rodrigues Johann Ana Lucia Ferreira Ana Marisa Fusco Almeida Anderson Joel Martino Andrade André Luís Laforga Vanzela Andrea Duarte Doetzer Andrea Name Colado Simao Andressa Pereira de Souza Anelisa Ramão Angelica Beate Winter Boldt Anna Hermínia Castro Gomes de Amorim Anna Silvia Penteado Setti da Rocha Antonio Camilo da Silva Filho Antonio Stabelini Neto Arthur Hirata Bertachi Barbara Mendes Paz Chao Betty Cristiane Kuhn Bruno Ambrozio Galindo Bruno Ribeiro Cruz Camilla Reginatto De Pierri Carla Fredrichsen Moya Araujo Carla Fredrichsen Moya Araujo Carlos Alberto Oliveira de Biagi Junior Carlos Augusto Nassar Carlos Eduardo Buss Carlos Gilberto Carlotti Junior Carlos Henrique Schneider Carolina Panis Carolina Weigert Galvão Caroline de Jesus Coelho Donha Caroline Guisantes de Salvo Toni Caryna Eurich Mazur Catiuscie Cabreira da Silva Tortorella Celso F. D. Doliveira Cesar Luiz Boguszewski Christiane Pienna Soares Chung Man Chin Claudia Moro Cleverson Busso Cristiane Cominetti Daiane Priscila Simão-Silva Dalila Luciola Zanette Daniel de Paula Daniel de Paula Daniel Rech Daniela Fiori Gradia Daniela Pretti da Cunha Tirapelli Daniela Viganó Zanoti Jeronymo Daniele Ukan Danielle Malheiros Ferreira Danielle Venturini Deborah Catharine de Assis Leite Deivid Calebe de Souza Dennis Armando Bertolini Edenir Inez Pamero Edna Maria Vissoci Reiche Edson Roberto Arpini Miguel Eduardo José de Almeida Araújo Eliana Carolina Vespero Eliandro Reis Tavares Elza Kimura Grimshaw Emanuel Maltempi de Souza Emanuele Cristina Gustani Buss Émerson Carraro Emiliana Cristina Melo ENILze Maria de Souza Fonseca Ribeiro Enilze Maria de Souza Fonseca Ribeiro Erika Izumi Erika Seki Kioshima Cotica Evani Marques Pereira Fabio Negretti Fábio Rodrigues Ferreira Seiva Felipe Dunin dos Santos Felipe Tuon Fernanda Andreia Rosa Fernanda Cestaro Prado Cortez Fernanda Ivanski Fernanda Maris Peria Flavia Regina Oliveira de Barros Franciele Aní Caovilla Follador Franciele Mara Lucca Zanardo Bohm Francinete Ramos Campos Fulviana Silva Nishiyama GABRIEL RIBEIRO CORDEIRO Gabriela Datsch Bennemann Gisele Santos de Oliveira Glaucio Valdameri Glauco Akelinghton Freire Vitiello Glauco Vieira Miranda Glaura Scantamburlo ALves Fernandes Guilherme Ferreira Silveira Gustavo Bianchini Porfírio Gustavo Lenci Marques Hélito Volpato Hildebrando Masshiro Nagai Huei Diana Lee Ilce Mara de Syllos Cólus Iris Rabinovich Israel Gomy Jackson Kawakami Jacques Duílio Brancher Jaime Luís Lopes Rocha Jaqueline Carvalho de Oliveira Jean Henrique da Silva Rodrigues Jean Leandro dos Santos Jeane Eliete Laguila Visentainer João Paulo Bianchi Ximenez Joaquim Manoel da Silva Jociani Ascari Joel Donazzolo Jorge Luis Maria Ruiz Jose Knopfholz José Luis da Conceição Silva José Sebastião dos Santos Joseane Carla Schabarum Juliana Cheleski Wiggers Juliana Mara Serpeloni Juliana Morini Küpper Cardoso Perseguini Karen Brajão de Oliveira Karin Braun Prado Karine Aparecida de Lima Katiany Rizzieri Caleffi Ferracioli Katiuscia de Oliveira Francisco Gabriel Kelvinson Fernandes Viana Larissa Beatriz Cossalter Larissa Danielle Bahls Pinto Laurival Antonio Vilas Boas Léia Carolina Lucio Líbero Mezzadri Neto Ligia Carla Faccin Galhardi Lirane Elize Defante Ferreto Luciana Furlaneto Maia Luciana Oliveira de Fariña Luciana Reis Azevedo Alanis Luciane Regina Cavalli Lucy Megumi Yamauchi Lioni Luis Paulo Gomes Mascarenhas Luis Paulo Gomes Mascarenhas Luís Paulo Mascarenhas Lupe Furtado Alle Lyvia Regina Biagi Silva Bertachi Mara Antonia Ramos Costa Mara L. Cordeiro Marcela Maria Birolim Marcelo Ricardo Vicari Marcia Edilaine Lopes Consolaro Marcia Holsbach Beltrame Marcia Regina Eches Perugini Marcos Abdo Arbex Marcos Pileggi MARCOS TADEU GRZELCZAK Marcus Peikriszwili Tartaruga Maria Angelica Ehara Watanabe Maria Antonia Ramos Costa Maria Claudia Gross Maria José Soares Mendes Giannini Maria Leandra Terencio Maria Lúcia Bonfleur Maria Luiza Guimarães de Oliveira Maria Luiza Petzl-Erler Mariana Abe Vicente Cavagnari Marina Kimiko Kadowaki Marise Fonseca dos Santos Marla Karine Amarante Maurício Turkiewicz Mauro Antonio Alves Castro Michel Rodrigo Zambrano Passarini Michele Potrich Michelle Orane Schemberger Milena Massumi Kozonoe Mônica Degraf Cavallin Monica Tereza Suldofski Mucio Luiz de Assis Cirino Nadia Graciele Krohn Najeh Maissar Khalil Nédia de Castilhos Ghisi Neide Tomimura Costa Neiva Leite Neyva Maria Lopes Romeiro Patrícia Amâncio da Rosa Patricia Dayane Carvalho Schaker Patricia Oehlmeyer Nassar Patrícia Savio de Araújo-Souza Patricia Silva Lucio Paulo Henrique Couto Souza Paulo Roberto Donadio Percy Nohama Quirino Alves de Lima Neto Rafael Deminice Rafael dos Santos Bezerra Raquel Alves dos Santos Renan Manozzo Galante Renata Ernlund Freitas de Macedo Rita de Cássia Garcia Simão Roberta Losi Guembarovski Roberto H. Herai Roberto Rosati Rodrigo Ferreira Rodrigo Rodrigues Matiello Rogério Neri Shinsato Rogério Pincela Mateus Rosane Aparecida Ribeiro Rosilene Fressatti Cardoso Rosilene Fressatti Cardoso Sandra Mara Guse Scós Venske Selene Elifio Esposito Sérgio Ossamu Ioshii Silvana Giuliatti Silvia Mara de Souza Halick Silvio Henrique Maia de Almeida Simone Neumann Wendt Spencer Luiz Marques Payão Stefan Wolanski Negrão Stephane Janaina de Moura Escobar Sueli Fumie Yamada Ogatta SUELI PERCIO QUINAIA Taciane Finatto Tatiana Mayumi Veiga Iriyoda Tayza Katelline Danilau Ostroski Tony Alexander Hild Valeria Valente Vanessa Nascimento Kozak Vanessa Santos Sotomaior Victor Breno Pedrosa Victoria Zeghbi Cochenski Borba Vivian Rotuno Moure Valdameri Wander Rogerio Pavanelli Weber Cláudio Francisco Nunes da Silva Willian Augusto de Melo Yohandra Reyes Torres
EPI_ISL_3369438, EPI_ISL_3369439, EPI_ISL_3369440, EPI_ISL_3369441	UFPA-Federal University of Pará	ITV-Vale Institute of Technology	Amanda Vidal; Guilherme Oliveira; Tatianne Costa Negri; Ândrea Kely Campos Ribeiro dos Santos
EPI_ISL_3072192	Unidade de apoio ao diagnostico da COVID - UNADIG	Bioinformatics Laboratory / LNCC	Alessandra P Lamarca; Alexandra L Gerber; Amilcar Tanuri; Ana Paula de C Guimaraes; Ana Tereza R Vasconcelos; Andrea Cony Cavalcanti; Caio Luiz Pereira Ribeiro; Cassia Alves; Cintia Policarpo; Claudia Maria Braga de Mello; Cristiane Gomes da Silva; Diana Mariani; Douglas Terra Machado; Flavio Dias da Silva; Gleidson da Silva de Oliveira; Leandro Magalhaes de Souza; Liliane Cavalcante; Luiz G P de Almeida; Marcio Henrique de Oliveira Garcia; Mario Sergio Ribeiro; Ronaldo da Silva F Jr; Silvia Carvalho
EPI_ISL_458140, EPI_ISL_458141, EPI_ISL_458146, EPI_ISL_458147, EPI_ISL_458148, EPI_ISL_524783, EPI_ISL_524785, EPI_ISL_524786, EPI_ISL_524787, EPI_ISL_524800, EPI_ISL_524801, EPI_ISL_848555, EPI_ISL_848557, EPI_ISL_848561, EPI_ISL_848562, EPI_ISL_848563, EPI_ISL_848564, EPI_ISL_848565, EPI_ISL_848566, EPI_ISL_848567, EPI_ISL_848568, EPI_ISL_848569, EPI_ISL_848570, EPI_ISL_848571, EPI_ISL_848572, EPI_ISL_848582, EPI_ISL_848583, EPI_ISL_848585, EPI_ISL_848587, EPI_ISL_848588, EPI_ISL_848589, EPI_ISL_848590, EPI_ISL_848591, EPI_ISL_848592, EPI_ISL_848593, EPI_ISL_848594, EPI_ISL_848595, EPI_ISL_848596, EPI_ISL_848597, EPI_ISL_848598, EPI_ISL_848606, EPI_ISL_848607, EPI_ISL_848608, EPI_ISL_848609, EPI_ISL_848611, EPI_ISL_848614, EPI_ISL_848615, EPI_ISL_848617, EPI_ISL_848618, EPI_ISL_848619, EPI_ISL_848620, EPI_ISL_848621, EPI_ISL_848622, EPI_ISL_848623, EPI_ISL_848624, EPI_ISL_848625, EPI_ISL_848628, EPI_ISL_848629, EPI_ISL_918514, EPI_ISL_918518, EPI_ISL_918519, EPI_ISL_918520, EPI_ISL_918521, EPI_ISL_2488795, EPI_ISL_2488796, EPI_ISL_2488813			
see above	Evandro Chagas Institute	Evandro Chagas Institute	A.M.; Barbagelata; E.C.; E.M.A.; Ferreira; G.M.R; H.R; J.A.; Junior; K.C.; L.C.; L.S.; M.C.; Martins; P.S.; Pinheiro; Resque; Santos; Silva; Sousa; Sousa Junior; Viana; W.D.C.; da Silva

We gratefully acknowledge the following Authors from the Originating laboratories responsible for obtaining the specimens, as well as the Submitting laboratories where the genome data were generated and shared via GISAID, on which this research is based.

All Submitters of data may be contacted directly via www.gisaid.org

Authors are sorted alphabetically.

Accession ID	Originating Laboratory	Submitting Laboratory	Authors
EPI_ISL_2645298, EPI_ISL_2645301, EPI_ISL_2645304, EPI_ISL_2645306, EPI_ISL_2645307	"CO Dept. of Public Health and Environment, Lab Services Division"	Centers for Disease Control and Prevention Division of Viral Diseases, Pathogen Discovery	Alison Laufer Halpin; Ben L. Rambo-Martin; Clinton R. Paden; Dakota Howard; Darlene Wagner; Dave Wentworth; Dhwani Batra; Jasmine Padilla; Justin Lee; Katie Dillon; Krista Queen; Kristen Knipe; Kristine Lacek; Mark Burroughs; Matthew Schmerer; Mili Sheth; Peter Cook; Sam Shepard; Sarah Nobles; Shoshona Le; Suxiang Tong; Vivien Dugan; Yvette Unoarumhi
EPI_ISL_471546	AMA DR Jose Soares Hungria	Instituto Adolfo Lutz, Interdiciplinary Procedures Center, Strategic Laboratory	Claudia Regina Gonçalves; Claudio Tavares Sacchi; Erica Valessa Ramos Gomes
EPI_ISL_523989	AMA Jardim Joamar	Instituto Adolfo Lutz, Interdiciplinary Procedures Center, Strategic Laboratory	Claudia Regina Gonçalves; Claudio Tavares Sacchi; Erica Valessa Ramos Gomes
EPI_ISL_523990	AMA Jardim Peri	Instituto Adolfo Lutz, Interdiciplinary Procedures Center, Strategic Laboratory	Claudia Regina Gonçalves; Claudio Tavares Sacchi; Erica Valessa Ramos Gomes
EPI_ISL_861652	AMA Wamberto Dias da Costa	Instituto Adolfo Lutz, Interdiciplinary Procedures Center, Strategic Laboratory	Claudia Regina Gonçalves; Claudio Tavares Sacchi; Erica Valessa Ramos Gomes; Karoline Rodrigues Campos
EPI_ISL_1966699	AMBULATORIO MEDICO DE ESPECIALIDADES DE PERUIBE	Instituto Butantan / Mendelics	Antonio Jorge Martins; Bianca Cechetto Carlos. Mendelics: Bibiana Santos; Claudia Renata dos Santos Barros; Cíntia Bittar; David Schlesinger. Hemocentro Ribeirão Preto: Simone Kashima; Debora Botequio Moretti; Elaine Cristina Marqueze; Elaine Vieira dos Santos; Elisangela Chicaroni Mattos; Erika Freitas; Evandra Strazza Rodrigues; Felipe Allan da Silva da Costa; Flavia Aburjaile; Fábio Sossai Possebon; Guilherme Campos; Guilherme Targino Valente; Heidge Fukumasu. USP-Botucatu: Rejane Maria Tommasini Grotto; Helena Lage Ferreira; Instituto Butantan: Dimas Tadeu Covas; Jardelina de Souza Todao Bernardino; Jayme A. Souza-Neto; Jessika Cristina Chagas Lesbon; Jorge A. Petroli Marchesi; José Salvatore Leister Patané; João Paulo Kitajima; João Pessoa Araújo Jr.; Leila Sabrina Ullmann; Loyze Paola Oliveira de Lima; Luiz Aurelio de Campos Crispin. Centro de Genômica Funcional da ESALQ: Luiz Lehmann Coutinho; Luiz Carlos Junior de Alcantara; Lívia Sacchetto; Maisa C. Pereira Parra; Maria Carolina Elias; Marta Giovanetti; Marília Moraes; Maurício Lacerda Nogueira. Prefeitura de Sao Paulo: Melissa Palmieri.; Patricia Akemi Assato; Paula Rahal; Paulo Inacio da Costa; Rafael dos Santos Bezerra; Raquel de Lello Rocha Campos Cassano. NGS Soluções Genômicas: Pilar Drummond Sampaio Corrêa Mariani. FZEA-USP Pirassununga: Mirele Daiana Poleti; Raul Machado Neto; Ricardo Augusto Brassaloti; Ricardo Haddad; Rodrigo Tocantins Calado. FAMERP-SJRP: Cecília Artico Banho; Sandra Coccuzzo Sampaio; Svetoslav Nanev Slavov; Vagner Fonseca; Vincent Louis Viala
EPI_ISL_2150721, EPI_ISL_2421940	Aegis Sciences Corporation	Centers for Disease Control and Prevention Division of Viral Diseases, Pathogen Discovery	Adrian Paskey; Alec Vest; Benjamin Rambo-Martin; Christopher Gulvick; Clinton R. Paden; Cyndi Clark; Dakota Howard; Darlene Wagner; Dhwani Batra; Dillon Nall; Duncan MacCannell; Ethan Sanders; Holly Houdeshell; Jason Caravas; Kara Moser; Matthew Hardison; Matthew Schmerer; Ola Kvalvaag; Patrick Campbell; Peter W. Cook; Rob Case; Scott Sammons; Shatavia Morrison; Shaun Westlund; Vikramsinha Ghorpade; Yvette Unoarumhi
EPI_ISL_1201886, EPI_ISL_1219028, EPI_ISL_1219032	Aeroporto Internacional de Guarulhos	Instituto Adolfo Lutz, Interdiciplinary Procedures Center, Strategic Laboratory	Caio Vinicius Dias Lopes; Claudia Regina Gonçalves; Claudio Tavares Sacchi; Erica Valessa Ramos Gomes; Karoline Rodrigues Campos
EPI_ISL_549084	Akershus University Hospital, Department for Microbiology and Infectious Disease Control	Norwegian Institute of Public Health, Department of Virology	Hilde Elshaug; Hilde Synnøve Vollan; Kamilla Heddeland Instefjord; Karoline Bragstad; Kathrine Stene-Johansen; Olav Hungnes; Rasmus Riis Kopperud
EPI_ISL_515544, EPI_ISL_523984, EPI_ISL_523986	Ama Dr Jose Soares Hungria	Instituto Adolfo Lutz, Interdiciplinary Procedures Center, Strategic Laboratory	Claudia Regina Gonçalves; Claudio Tavares Sacchi; Erica Valessa Ramos Gomes
EPI_ISL_2156551	Amosup Seamen's Hospital	Philippine Genome Center	Alethea R. de Guzman; Anna Ong-Lim; Arianne A. Zamora; Asia Louisa U. Chong; Benedict A. Maralit; Candice Francheska B. Tambaoan; Carlo M. Lapid; Celia Carlos; Devon Ray Pacial; Edsel Maurice Salvaña; El King D. Morado; Elcid Aaron R. Pangilinan; Eva Maria Cutiongco-de la Paz; Francis A. Tablizo; Irish Coleen A. Asin; Jaime C. Montoya; Jan Michael C. Yap; Jo-Hannah S. Llames; John Q. Wong; Joshua Gregor A. Dizon; Juan Antonio R. Magalang; Karol Sophia Agape R. Padilla; Kenneth M. Kim; Kris P. Punayan; Marc Edsel C. Ayes; Marc Jerrone R. Castro; Maria Rosario Singh-Vergeire and Cynthia P. Saloma; Maria Sofia L. Yangzon; Marissa Alejandria; Razel Nikka M. Hao; Rianna Patricia S. Cruz; Sheila Mae M. Araiza
EPI_ISL_509500, EPI_ISL_527019, EPI_ISL_527032	Area of Virology, Serology and Virology Division (SAViD), New South Wales Health Pathology Randwick	Area of Virology, Serology and Virology Division (SAViD), New South Wales Health Pathology Randwick	Rawlinson, W.
EPI_ISL_678320	Area of Virology, Serology and Virology Division (SAViD), New South Wales Health Pathology Randwick	Virology Research Laboratory; Area of Virology, Serology and Virology Division (SAViD), New South Wales Health Pathology Randwick	Au, J.; Bull, R.; Deveson, I.; Foster, C.; Rawlinson, W.; Ruiz Silva, M.; Van Hal, S.
EPI_ISL_2105513, EPI_ISL_2105518, EPI_ISL_2105520	Army General Hospital Molecular Laboratory	Philippine Genome Center	Alethea R. de Guzman; Anna Ong-Lim; Arianne A. Zamora; Asia Louisa U. Chong; Benedict A. Maralit; Candice Francheska B. Tambaoan; Carlo M. Lapid; Celia Carlos; Devon Ray Pacial; Edsel Maurice Salvaña; El King D. Morado; Eva Maria Cutiongco-de la Paz; Francis A. Tablizo; Irish Coleen A. Asin; Jaime C. Montoya; Jan Michael C. Yap; Jo-Hannah S. Llames; John Q. Wong; Joshua Gregor A. Dizon; Juan Antonio R. Magalang; Karol Sophia Agape R. Padilla; Kenneth M. Kim; Kris P. Punayan; Marc Edsel C. Ayes; Marc Jerrone R. Castro; Maria Rosario Singh-Vergeire and Cynthia P. Saloma; Maria Sofia L. Yangzon; Marissa Alejandria; Razel Nikka M. Hao; Rianna Patricia S. Cruz; Sheila Mae M. Araiza
EPI_ISL_1499020, EPI_ISL_1499297	Associação Fundo de Incentivo à Pesquisa (AFIP)	Associação Fundo de Incentivo à Pesquisa (AFIP)	Debora R. Ramadan; Erika Rodrigues de Oliveira; Juliana Nogueira Martins Rodrigues; Priscila Farias Tempaku; Sergio Tufik.; Soraya Sgambatti de Andrade
EPI_ISL_2499718, EPI_ISL_2499719	BCCDC Public Health Laboratory	BCCDC Public Health Laboratory	Ana Pacagnella; Corrinne Ng; Dan Fornika; John Tyson; Kim Macdonald; Kimia Kamelian; Linda Hoang; Loretta Janz; Mel Krajden; Prystajecky Natalie; Robert Azana; Shannon Russell
EPI_ISL_2156319	BULACAN MEDICAL CENTER	Philippine Genome Center	Alethea R. de Guzman; Anna Ong-Lim; Arianne A. Zamora; Asia Louisa U. Chong; Benedict A. Maralit; Candice Francheska B. Tambaoan; Carlo M. Lapid; Celia Carlos; Devon Ray Pacial; Edsel Maurice Salvaña; El King D. Morado; Elcid Aaron R. Pangilinan; Eva Maria Cutiongco-de la Paz; Francis A. Tablizo; Irish Coleen A. Asin; Jaime C. Montoya; Jan Michael C. Yap; Jo-Hannah S. Llames; John Q. Wong; Joshua Gregor A. Dizon; Juan Antonio R. Magalang; Karol Sophia Agape R. Padilla; Kenneth M. Kim; Kris P. Punayan; Marc Edsel C. Ayes; Marc Jerrone R. Castro; Maria Rosario Singh-Vergeire and Cynthia P. Saloma; Maria Sofia L. Yangzon; Marissa Alejandria; Razel Nikka M. Hao; Rianna Patricia S. Cruz; Sheila Mae M. Araiza
EPI_ISL_2105421, EPI_ISL_2188197	Baguio General Hospital Medical Center (BGHMC)	Philippine Genome Center	Alethea R. de Guzman; Anna Ong-Lim; Arianne A. Zamora; Asia Louisa U. Chong; Benedict A. Maralit; Candice Francheska B. Tambaoan; Carlo M. Lapid; Celia Carlos; Devon Ray Pacial; Edsel Maurice Salvaña; El King D. Morado; Elcid Aaron R. Pangilinan; Eva Maria Cutiongco-de la Paz; Francis A. Tablizo; Irish Coleen A. Asin; Jaime C. Montoya; Jan Michael C. Yap; Jo-Hannah S. Llames; John Q. Wong; Joshua Gregor A. Dizon; Juan Antonio R. Magalang; Karol Sophia Agape R. Padilla; Kenneth M. Kim; Kris P. Punayan; Marc Edsel C. Ayes; Marc Jerrone R. Castro; Maria Rosario Singh-Vergeire and Cynthia P. Saloma; Maria Sofia L. Yangzon; Marissa Alejandria; Razel Nikka M. Hao; Rianna Patricia S. Cruz; Sheila Mae M. Araiza
EPI_ISL_2156577	Bataan General Hospital and Medical Center	Philippine Genome Center	Alethea R. de Guzman; Anna Ong-Lim; Arianne A. Zamora; Asia Louisa U. Chong; Benedict A. Maralit; Candice Francheska B. Tambaoan; Carlo M. Lapid; Celia Carlos; Devon Ray Pacial; Edsel Maurice Salvaña; El King D. Morado; Elcid Aaron R. Pangilinan; Eva Maria Cutiongco-de la Paz; Francis A. Tablizo; Irish Coleen A. Asin; Jaime C. Montoya; Jan Michael C. Yap; Jo-Hannah S. Llames; John Q. Wong; Joshua Gregor A. Dizon; Juan Antonio R. Magalang; Karol Sophia Agape R. Padilla; Kenneth M. Kim; Kris P. Punayan; Marc Edsel C. Ayes; Marc Jerrone R. Castro; Maria Rosario Singh-Vergeire and Cynthia P. Saloma; Maria Sofia L. Yangzon; Marissa Alejandria; Razel Nikka M. Hao; Rianna Patricia S. Cruz; Sheila Mae M. Araiza
EPI_ISL_2551528	Belo Horizonte center-south emergency care unit - UPA-BH	Laboratório de Virologia Clínica e Molecular	Alex Fiorini; Ana Paula Salles Fernandes; Bruna Larotonda Telezynski; Danielle Bruna Leal Oliveira; Edison Luiz Durigon; Erick Gustavo Dorlass; Flavio Fonseca e Santuza Teixeira; Guilherme Pereira Scagion; Helena Perez Coelho; Hugo Sato; Karine Lima Lourenço; Luciano Matsumiya Thomazelli; Renata Peixoto; Rubens Daniel Miserani Magalhães; Tatiana Ometto
EPI_ISL_2698098	Biology, UFLA, Universidade Federal de Lavras	Biology, UFLA	Barcante, J.; Cherem, J.; Fernandes, G.; Luciano, P.; Melo, D.; Pylro, V.
EPI_ISL_3364406	Biotia	Biotia	Christopher Mason; David Danko; Dorottya Nagy-Szakal; Mara Couto-Rodriguez; Marilyne Debieu; Niamh O'Hara; Xavier Jirau Serrano
EPI_ISL_976950, EPI_ISL_976998, EPI_ISL_1121645, EPI_ISL_1121665, EPI_ISL_1121671, EPI_ISL_1121679, EPI_ISL_1121680			
see above	Broad Institute	Infectious Disease	Adams, G.; B.L.; B.W.; Bauer, M.; Birren; Carter, A.; Chaluvadi, S.; D.J.; DeRuff, K.; Gallagher, G.; Gladden-Young, A.; J.E.; K.J.; Lagerborg, K.; Lemieux; Loreth, C.; MacInnis; Normandin, E.; P.C.; Park; Reilly, S.; Rudy, M.; Siddle; Smole, S.; Tomkins-Tinch, C.; and Sabeti

	Clinical Research Sequencing Platform	Program, Broad Institute of Harvard and MIT	
EPI_ISL_2105414, EPI_ISL_2105456, EPI_ISL_2105522, EPI_ISL_2105524, EPI_ISL_2105544, EPI_ISL_2105548, EPI_ISL_2156340, EPI_ISL_2156343, EPI_ISL_2156380, EPI_ISL_2156397, EPI_ISL_2156422, EPI_ISL_2156445, EPI_ISL_2188097, EPI_ISL_2188994, EPI_ISL_2189115			
see above	Butuan Medical Center	Philippine Genome Center	Alethea R. de Guzman; Anna Ong-Lim; Arianne A. Zamora; Asia Louisa U. Chong; Benedict A. Maralit; Candice Francheska B. Tambaoan; Carlo M. Lapid; Celia Carlos; Devon Ray Pacial; Edsel Maurice Salvaña; El King D. Morado; Elcid Aaron R. Pangilinan; Eva Maria Cutiongco-de la Paz; Francis A. Tablizo; Irish Coleen A. Asin; Jaime C. Montoya; Jan Michael C. Yap; Jo-Hannah S. Llames; John Q. Wong; Joshua Gregor A. Dizon; Juan Antonio R. Magalang; Karol Sophia Agape R. Padilla; Kenneth M. Kim; Kris P. Punayan; Marc Edsel C. Ayes; Marc Jerrone R. Castro; Maria Rosario Singh-Vergeire and Cynthia P. Saloma; Maria Sofia L. Yangzon; Marissa Alejandria; Razel Nikka M. Hao; Renato Jacinto Q. Mantaring; Rianna Patricia S. Cruz; Sheila Mae M. Araiza
EPI_ISL_2797603	CDPH VBL	California Department of Public Health	CDPH-COVIDNet; UCSF Center for Advanced Technology
EPI_ISL_3912410	CENTRO DE ESPECIALIDADES MEDICAS DR NESTOR DE PAULA PESSOA	Analytical Competence Molecular Epidemiology Lab/ACME, Oswaldo Cruz Foundation, Ceara (FIOCRUZ CE)	Cleber Furtado Aksenen; Fabio Miyajima; Fernando Braga Stehling; Francisco Eder de Moura Lopes; Jamille Maria Mendes Bezerra; Joaquim Cesar do Nascimento Sousa Junior; Pedro Miguel Carneiro Jeronimo; Suzana Porto Almeida & Lucas Delerino on behalf of COVID-19 FIOCRUZ Genomic Network; Thais Ferreira de Oliveira; Thais de Oliveira Costa; Ticiane Cavalcante de Souza; Veridiana Pessoa Miyajima
EPI_ISL_1469658	CENTRO DE ESPECIALIDADES TRIUNFO	Epiclin	Ana Paula Muterle; Carolina Comerlato; Eliana Márcia Da Ros Wendland; Fernando Hayashi Sant'Anna; Janira Prichula; Juliana Comerlato
EPI_ISL_1469642, EPI_ISL_1469737	CENTRO DE REFERENCIA EM SINDROMES GRIPAIS	Epiclin	Ana Paula Muterle; Carolina Comerlato; Eliana Márcia Da Ros Wendland; Fernando Hayashi Sant'Anna; Janira Prichula; Juliana Comerlato
EPI_ISL_1445195, EPI_ISL_1966219	CENTRO DE SAUDE DE BORA	Instituto Butantan / Mendelics	Antonio Jorge Martins; Bianca Cechetto Carlos. Mendelics: Bibiana Santos; Bibiana Santos; Claudia Renata dos Santos Barros; Cíntia Bittar; David Schlesinger; David Schlesinger. Hemocentro Ribeirão Preto: Simone Kashima; Debora Botequio Moretti; Dimas Tadeu Covas; Elaine Cristina Marqueze; Elaine Vieira dos Santos; Elisangela Chicaroni Mattos; Erika Freitas; Evandra Strazza Rodrigues; Felipe Allan da Silva da Costa; Flavia Aburjaile; Fábio Sossai Possebon; Guilherme Campos; Guilherme Targino Valente; Heidge Fukumasu. USP-Botucatu: Rejane Maria Tommasini Grotto; Helena Lage Ferreira; Instituto Butantan: Dimas Tadeu Covas; Jardelina de Souza Todao Bernardino; Jayme A. Souza-Neto; Jessika Cristina Chagas Lesbon; Jorge A. Petroli Marchesi; José Salvatore Leister Patané; João Paulo Kitajima; João Pessoa Araújo Jr.; Leila Sabrina Ullmann; Loyze Paola Oliveira de Lima; Luiz Aurelio de Campos Crispin. Centro de Genômica Funcional da ESALQ: Luiz Lehmann Coutinho; Luiz Carlos Junior de Alcantara; Lívia Sacchetto; Maisa C. Pereira Parra; Maria Carolina Elias; Marta Giovanetti; Marília Moraes; Maurício Lacerda Nogueira. Prefeitura de Sao Paulo: Melissa Palmieri.; Patricia Akemi Assato; Paula Rahal; Paulo Inacio da Costa; Rafael dos Santos Bezerra; Raquel de Lello Rocha Campos Cassano. NGS Soluções Genômicas: Pilar Drummond Sampaio Corrêa Mariani. FZEA-USP Pirassununga: Mirele Daiana Poleti; Raul Machado Neto; Ricardo Augusto Brassaloti; Ricardo Haddad; Rodrigo Tocantins Calado.; Rodrigo Tocantins Calado. FAMERP-SJRP: Cecília Artico Banho; Sandra Coccuzzo Sampaio; Simone Kashima; Svetoslav Nanev Slavov; Vagner Fonseca; Vincent Louis Viala
EPI_ISL_1966239	CENTRO DE SAUDE DR MARIO DIAS DE AGUIAR CAPIVARI	Instituto Butantan / Mendelics	Antonio Jorge Martins; Bianca Cechetto Carlos. Mendelics: Bibiana Santos; Claudia Renata dos Santos Barros; Cíntia Bittar; David Schlesinger. Hemocentro Ribeirão Preto: Simone Kashima; Debora Botequio Moretti; Elaine Cristina Marqueze; Elaine Vieira dos Santos; Elisangela Chicaroni Mattos; Erika Freitas; Evandra Strazza Rodrigues; Felipe Allan da Silva da Costa; Flavia Aburjaile; Fábio Sossai Possebon; Guilherme Campos; Guilherme Targino Valente; Heidge Fukumasu. USP-Botucatu: Rejane Maria Tommasini Grotto; Helena Lage Ferreira; Instituto Butantan: Dimas Tadeu Covas; Jardelina de Souza Todao Bernardino; Jayme A. Souza-Neto; Jessika Cristina Chagas Lesbon; Jorge A. Petroli Marchesi; José Salvatore Leister Patané; João Paulo Kitajima; João Pessoa Araújo Jr.; Leila Sabrina Ullmann; Loyze Paola Oliveira de Lima; Luiz Aurelio de Campos Crispin. Centro de Genômica Funcional da ESALQ: Luiz Lehmann Coutinho; Luiz Carlos Junior de Alcantara; Lívia Sacchetto; Maisa C. Pereira Parra; Maria Carolina Elias; Marta Giovanetti; Marília Moraes; Maurício Lacerda Nogueira. Prefeitura de Sao Paulo: Melissa Palmieri.; Patricia Akemi Assato; Paula Rahal; Paulo Inacio da Costa; Rafael dos Santos Bezerra; Raquel de Lello Rocha Campos Cassano. NGS Soluções Genômicas: Pilar Drummond Sampaio Corrêa Mariani. FZEA-USP Pirassununga: Mirele Daiana Poleti; Raul Machado Neto; Ricardo Augusto Brassaloti; Ricardo Haddad; Rodrigo Tocantins Calado. FAMERP-SJRP: Cecília Artico Banho; Sandra Coccuzzo Sampaio; Svetoslav Nanev Slavov; Vagner Fonseca; Vincent Louis Viala
EPI_ISL_3912412	CENTRO DE SAUDE DR MIRANDA TAVARES	Analytical Competence Molecular Epidemiology Lab/ACME, Oswaldo Cruz Foundation, Ceara (FIOCRUZ CE)	Cleber Furtado Aksenen; Fabio Miyajima; Fernando Braga Stehling; Francisco Eder de Moura Lopes; Jamille Maria Mendes Bezerra; Joaquim Cesar do Nascimento Sousa Junior; Pedro Miguel Carneiro Jeronimo; Suzana Porto Almeida & Lucas Delerino on behalf of COVID-19 FIOCRUZ Genomic Network; Thais Ferreira de Oliveira; Thais de Oliveira Costa; Ticiane Cavalcante de Souza; Veridiana Pessoa Miyajima
EPI_ISL_1445089, EPI_ISL_1966131	CENTRO DE SAUDE II MAIRINQUE MAIRINQUE	Instituto Butantan / Mendelics	Antonio Jorge Martins; Bianca Cechetto Carlos. Mendelics: Bibiana Santos; Bibiana Santos; Claudia Renata dos Santos Barros; Cíntia Bittar; David Schlesinger; David Schlesinger. Hemocentro Ribeirão Preto: Simone Kashima; Debora Botequio Moretti; Dimas Tadeu Covas; Elaine Cristina Marqueze; Elaine Vieira dos Santos; Elisangela Chicaroni Mattos; Erika Freitas; Evandra Strazza Rodrigues; Felipe Allan da Silva da Costa; Flavia Aburjaile; Fábio Sossai Possebon; Guilherme Campos; Guilherme Targino Valente; Heidge Fukumasu. USP-Botucatu: Rejane Maria Tommasini Grotto; Helena Lage Ferreira; Instituto Butantan: Dimas Tadeu Covas; Jardelina de Souza Todao Bernardino; Jayme A. Souza-Neto; Jessika Cristina Chagas Lesbon; Jorge A. Petroli Marchesi; José Salvatore Leister Patané; João Paulo Kitajima; João Pessoa Araújo Jr.; Leila Sabrina Ullmann; Loyze Paola Oliveira de Lima; Luiz Aurelio de Campos Crispin. Centro de Genômica Funcional da ESALQ: Luiz Lehmann Coutinho; Luiz Carlos Junior de Alcantara; Lívia Sacchetto; Maisa C. Pereira Parra; Maria Carolina Elias; Marta Giovanetti; Marília Moraes; Maurício Lacerda Nogueira. Prefeitura de Sao Paulo: Melissa Palmieri.; Patricia Akemi Assato; Paula Rahal; Paulo Inacio da Costa; Rafael dos Santos Bezerra; Raquel de Lello Rocha Campos Cassano. NGS Soluções Genômicas: Pilar Drummond Sampaio Corrêa Mariani. FZEA-USP Pirassununga: Mirele Daiana Poleti; Raul Machado Neto; Ricardo Augusto Brassaloti; Ricardo Haddad; Rodrigo Tocantins Calado.; Rodrigo Tocantins Calado. FAMERP-SJRP: Cecília Artico Banho; Sandra Coccuzzo Sampaio; Simone Kashima; Svetoslav Nanev Slavov; Vagner Fonseca; Vincent Louis Viala
EPI_ISL_3912414	CENTRO DE SAUDE OTAVIO LOBO	Analytical Competence Molecular Epidemiology Lab/ACME, Oswaldo Cruz Foundation, Ceara (FIOCRUZ CE)	Cleber Furtado Aksenen; Fabio Miyajima; Fernando Braga Stehling; Francisco Eder de Moura Lopes; Jamille Maria Mendes Bezerra; Joaquim Cesar do Nascimento Sousa Junior; Pedro Miguel Carneiro Jeronimo; Suzana Porto Almeida & Lucas Delerino on behalf of COVID-19 FIOCRUZ Genomic Network; Thais Ferreira de Oliveira; Thais de Oliveira Costa; Ticiane Cavalcante de Souza; Veridiana Pessoa Miyajima
EPI_ISL_1469584, EPI_ISL_1469629	CENTRO MUNICIPAL DE SAUDE DE ROLANTE	Epiclin	Ana Paula Muterle; Carolina Comerlato; Eliana Márcia Da Ros Wendland; Fernando Hayashi Sant'Anna; Janira Prichula; Juliana Comerlato
EPI_ISL_3912415	CENTRO ODONTOLOGICO DA POLICIA MILITAR DO CEARA CEOP PMCE	Analytical Competence Molecular Epidemiology Lab/ACME, Oswaldo Cruz Foundation, Ceara (FIOCRUZ CE)	Cleber Furtado Aksenen; Fabio Miyajima; Fernando Braga Stehling; Francisco Eder de Moura Lopes; Jamille Maria Mendes Bezerra; Joaquim Cesar do Nascimento Sousa Junior; Pedro Miguel Carneiro Jeronimo; Suzana Porto Almeida & Lucas Delerino on behalf of COVID-19 FIOCRUZ Genomic Network; Thais Ferreira de Oliveira; Thais de Oliveira Costa; Ticiane Cavalcante de Souza; Veridiana Pessoa Miyajima
EPI_ISL_732252	CH Porto - H Sto Antonio	Instituto Nacional de Saude (INSA) and Instituto Gulbenkian de Ciencia (IGC)	Borges et al
EPI_ISL_2017667, EPI_ISL_2516562, EPI_ISL_2516565, EPI_ISL_2516577	CLILAB	Microbiology Department	Aida Gonzalez-Diaz; Carmen Ardanuy; Jordi Camara; Jordi Niubó; Laura Calatayud; M Angeles Domínguez; Miguel Fernandez-Huerta; Sara Marti
EPI_ISL_2383909, EPI_ISL_2383915	CO Dept. of Public Health and Environment, Lab Services Division	Centers for Disease Control and Prevention Division of Viral Diseases, Pathogen Discovery	Alison Laufer Halpin; Ben L. Rambo-Martin; Clinton R. Paden; Dakota Howard; Darlene Wagner; Dave Wentworth; Dhwani Batra; Jasmine Padilla; Justin Lee; Katie Dillon; Krista Queen; Kristen Knipe; Kristine Lacek; Mark Burroughs; Matthew Schmerer; Mili Sheth; Peter Cook; Sam Shepard; Sarah Nobles; Shoshona Le; Suxiang Tong; Vivien Dugan; Yvette Unoarumhi
EPI_ISL_1469604, EPI_ISL_1469608, EPI_ISL_1469636, EPI_ISL_1469641	COORDENADORIA GERAL DE VIGILANCIA EM SAUDE	Epiclin	Ana Paula Muterle; Carolina Comerlato; Eliana Márcia Da Ros Wendland; Fernando Hayashi Sant'Anna; Janira Prichula; Juliana Comerlato
EPI_ISL_735408	COVID 19 Centro de Combate ao Coronavirus CCC Jandira	Instituto Adolfo Lutz, Interdiciplinary Procedures Center, Strategic Laboratory	Claudia Regina Gonçalves; Claudio Tavares Sacchi; Erica Valessa Ramos Gomes; Karoline Rodrigues Campos
EPI_ISL_2170967	CS DE NIPOA	Instituto Butantan / Mendelics	Antonio Jorge Martins; Bianca Cechetto Carlos. Mendelics: Bibiana Santos; Claudia Renata dos Santos Barros; Cíntia Bittar; David Schlesinger. Hemocentro Ribeirão Preto: Simone Kashima; Debora Botequio Moretti; Elaine Cristina Marqueze; Elaine Vieira dos Santos; Elisangela Chicaroni Mattos; Erika Freitas; Evandra Strazza Rodrigues; Felipe Allan da Silva da Costa; Flavia Aburjaile; Fábio Sossai Possebon; Guilherme Campos; Guilherme Targino Valente; Heidge Fukumasu. USP-Botucatu: Rejane Maria Tommasini Grotto; Helena Lage Ferreira; Instituto Butantan: Dimas Tadeu Covas; Jardelina de Souza Todao Bernardino; Jayme A. Souza-Neto; Jessika Cristina Chagas Lesbon; Jorge A. Petroli Marchesi; José Salvatore Leister Patané; João Paulo Kitajima; João Pessoa Araújo Jr.; Leila Sabrina Ullmann; Loyze Paola Oliveira de Lima; Luiz Aurelio de Campos Crispin. Centro de Genômica Funcional da ESALQ: Luiz Lehmann Coutinho; Luiz Carlos Junior de Alcantara; Lívia Sacchetto; Maisa C. Pereira Parra; Maria Carolina Elias; Marta Giovanetti; Marília Moraes; Maurício Lacerda Nogueira. Prefeitura de Sao Paulo: Melissa Palmieri.; Patricia Akemi Assato; Paula Rahal; Paulo Inacio da Costa; Rafael dos Santos Bezerra; Raquel de Lello Rocha Campos Cassano. NGS Soluções Genômicas: Pilar Drummond Sampaio Corrêa Mariani. FZEA-USP Pirassununga: Mirele Daiana Poleti; Raul Machado Neto; Ricardo Augusto Brassaloti; Ricardo Haddad; Rodrigo Tocantins Calado. FAMERP-SJRP: Cecília Artico Banho; Sandra Coccuzzo Sampaio; Svetoslav Nanev Slavov; Vagner Fonseca; Vincent Louis Viala
EPI_ISL_2344853	CS DE PALMARES PAULISTA	Instituto Butantan / ESALQ-Piracicaba	Antonio Jorge Martins; Claudia Renata dos Santos Barros; David Schlesinger; Debora Botequio Moretti; Dimas Tadeu Covas; Elaine Cristina Marqueze; Elaine Vieira Santos; Evandra Strazza Rodrigues; Heidge Fukumasu; Jayme Augusto de Souza-Neto; José Salvatore Leister Patané; Luiz Alcantara; Luiz Lehmann Coutinho; Maria Carolina Elias; Maurício Lacerda Nogueira; Rafael dos Santos Bezerra; Raul Machado Neto; Rejane Maria Tommasini Grotto; Ricardo Haddad; Sandra Coccuzzo Sampaio Vessoni; Simone Kashima; Svetoslav Nanev Slavov; Vincent Louis Viala
EPI_ISL_583494	CS II Dr. Antonio Vicoso Moreira de Rezende Sumare	Instituto Adolfo Lutz, Interdiciplinary Procedures Center, Strategic Laboratory	Claudia Regina Gonçalves; Claudio Tavares Sacchi; Erica Valessa Ramos Gomes; Karoline Rodrigues Campos
EPI_ISL_468314, EPI_ISL_583503	CTA Centro de Testagem e Aconselhamento	Instituto Adolfo Lutz, Interdiciplinary Procedures Center, Strategic Laboratory	Claudia Regina Gonçalves; Claudio Tavares Sacchi; Erica Valessa Ramos Gomes; Karoline Rodrigues Campos
EPI_ISL_2105218, EPI_ISL_2105225, EPI_ISL_2105226, EPI_ISL_2105231, EPI_ISL_2105242, EPI_ISL_2105245, EPI_ISL_2105253, EPI_ISL_2105254, EPI_ISL_2105255, EPI_ISL_2105270, EPI_ISL_2105276, EPI_ISL_2105282, EPI_ISL_2105291, EPI_ISL_2105299, EPI_ISL_2105322, EPI_ISL_2105409, EPI_ISL_2105448, EPI_ISL_2105453, EPI_ISL_2105529, EPI_ISL_2105532, EPI_ISL_2156415, EPI_ISL_2156431, EPI_ISL_2156432, EPI_ISL_2156433, EPI_ISL_2156460, EPI_ISL_2156515, EPI_ISL_2156553, EPI_ISL_2156581, EPI_ISL_2156625, EPI_ISL_2156650, EPI_ISL_2156679, EPI_ISL_2156700, EPI_ISL_2156705, EPI_ISL_2156712, EPI_ISL_2156733, EPI_ISL_2188061			
see above	Cagayan Valley Medical Center Molecular Laboratory	Philippine Genome Center	Alethea R. de Guzman; Anna Ong-Lim; Arianne A. Zamora; Asia Louisa U. Chong; Benedict A. Maralit; Candice Francheska B. Tambaoan; Carlo M. Lapid; Celia Carlos; Devon Ray Pacial; Edsel Maurice Salvaña; El King D. Morado; Elcid Aaron R. Pangilinan; Eva Maria Cutiongco-de la Paz; Francis A. Tablizo; Irish Coleen A. Asin; Jaime C. Montoya; Jan Michael C. Yap; Jo-Hannah S. Llames; John Q. Wong; Joshua Gregor A. Dizon; Juan Antonio R. Magalang; Karol Sophia Agape R. Padilla; Kenneth M. Kim; Kris P. Punayan; Marc Edsel C. Ayes; Marc Jerrone R. Castro; Maria Rosario Singh-Vergeire and Cynthia P. Saloma; Maria Sofia L. Yangzon; Marissa Alejandria; Razel Nikka M. Hao; Rianna Patricia S. Cruz; Sheila Mae M. Araiza
EPI_ISL_4005510	California	California Department	CDPH IDLB COVIDNet et al

	Department of Public Health	of Public Health	
EPI_ISL_2837812	California Department of Public Health Valencia Branch Laboratory (CDPH VBL)	California Department of Public Health	CDPH-COVIDNet
EPI_ISL_579220	Canterbury Health Laboratories	Institute of Environmental Science and Research (ESR)	Anja Werno; Antje van der Linden; Arlo Upton; Chris Mansell; David Hammer; Dragana Drinkovic; Erasmus Smit; Gary McAuliffe; Hana Sofia Andersson; Hermes Perez; James Ussher; Jill Sherwood; Jing Wang; Joep de Ligt; Josh Freeman; Julia Howard; Juliet Elvy; Lauren Jelly; Mary DeAlmeida; Matt Blakiston; Matt Storey; Matthew Rogers; Max Bloomfield; Michael Addidle; Michelle Balm; Muhammad Faisal; Nikki Freed; Olin Silander; Sally Roberts; Sarah Jefferies; Sharmini Muttaiyah; Susan Morpeth; Susan Taylor; Timothy Blackmore; Vani Sathyendran; Veronica Playle; Virginia Hope; Xiaoyun Ren
EPI_ISL_583504, EPI_ISL_583505	Casa de Saude Stella Maris	Instituto Adolfo Lutz, Interdiciplinary Procedures Center, Strategic Laboratory	Claudia Regina Gonçalves; Claudio Tavares Sacchi; Erica Valessa Ramos Gomes; Karoline Rodrigues Campos
EPI_ISL_693235	Casmi Centro Atendimento Saude da Mulher e Infancia	Instituto Adolfo Lutz, Interdiciplinary Procedures Center, Strategic Laboratory	Claudia Regina Gonçalves; Claudio Tavares Sacchi; Erica Valessa Ramos Gomes; Karoline Rodrigues Campos
EPI_ISL_2105383	Cebu TB Reference Laboratory	Philippine Genome Center	Alethea R. de Guzman; Anna Ong-Lim; Arianne A. Zamora; Asia Louisa U. Chong; Benedict A. Maralit; Candice Francheska B. Tambaoan; Carlo M. Lapid; Celia Carlos; Devon Ray Pacial; Edsel Maurice Salvaña; El King D. Morado; Eva Maria Cutiongco-de la Paz; Francis A. Tablizo; Irish Coleen A. Asin; Jaime C. Montoya; Jan Michael C. Yap; Jo-Hannah S. Llames; John Q. Wong; Joshua Gregor A. Dizon; Juan Antonio R. Magalang; Karol Sophia Agape R. Padilla; Kenneth M. Kim; Kris P. Punayan; Marc Edsel C. Ayes; Marc Jerrone R. Castro; Maria Rosario Singh-Vergeire and Cynthia P. Saloma; Maria Sofia L. Yangzon; Marissa Alejandria; Razel Nikka M. Hao; Rianna Patricia S. Cruz; Sheila Mae M. Araiza
EPI_ISL_522491	Center for Laboratory Control of Infectious Diseases, Korea Centers for Diseases Control and Prevention	Center for Laboratory Control of Infectious Diseases, Korea Centers for Diseases Control and Prevention	Ae Kyung Park; Eunkyung Shin; Heui Man Kim; Jeong-Min Kim; Jin Sun No; Junyoung Kim; Myung Guk Han; Yoon-Seok Chung
EPI_ISL_940924	Centers for Disease Control and Prevention, Dengue Branch	Centers for Disease Control and Prevention, Dengue Branch	Betzabel Flores; Gabriela Paz-Bailey; Gilberto A. Santiago; Glenda Gonzalez; Jorge L. Munoz-Jordan; Keyla Charriez
EPI_ISL_930857, EPI_ISL_942898, EPI_ISL_943581, EPI_ISL_943584, EPI_ISL_943606	Central Laboratory of Public Health of Rio Grande do Sul (Lacen-RS)	State Center for Health Surveillance of the Health Department of the State of Rio Grande do Sul (CEVS/SES-RS)	; Aline Campos; Amanda da Silva; Anelise Schaurich; Barcellos R; Campos A; Claudia Dornelles; Crescente L; Cynthia Molina; Da Silva A; Dornelles C; Fernanda Godinho; Fonseca V; Garay L; Godinho F; Gonzalez A; Gregianini T; Lara Crescente; Leticia Garay; Molina C; Regina Barcellos; Richard Salvato; Salvato R; Schaurich A; Tatiana Gregianini; Vagner Fonseca
EPI_ISL_978498, EPI_ISL_978506, EPI_ISL_978515, EPI_ISL_978517, EPI_ISL_978518, EPI_ISL_978525, EPI_ISL_978529, EPI_ISL_1068319, EPI_ISL_1068363, EPI_ISL_1068364, EPI_ISL_1068369, EPI_ISL_1068371, EPI_ISL_1068373, EPI_ISL_1068376, EPI_ISL_1068377, EPI_ISL_1068378, EPI_ISL_1068380, EPI_ISL_1068394, EPI_ISL_1583642, EPI_ISL_1583643, EPI_ISL_1583648, EPI_ISL_1583649, EPI_ISL_1583651, EPI_ISL_1583657, EPI_ISL_1583658, EPI_ISL_1583659, EPI_ISL_1583660, EPI_ISL_1583668, EPI_ISL_1583671, EPI_ISL_1583686, EPI_ISL_1583727, EPI_ISL_3255056, EPI_ISL_3255082, EPI_ISL_3255084, EPI_ISL_3266080, EPI_ISL_3266082, EPI_ISL_3266083, EPI_ISL_3266085, EPI_ISL_3266086, EPI_ISL_3266096, EPI_ISL_3266097, EPI_ISL_3266121, EPI_ISL_3506972, EPI_ISL_3506975, EPI_ISL_3506976, EPI_ISL_3506981, EPI_ISL_3506982, EPI_ISL_3506983, EPI_ISL_3506986, EPI_ISL_3631208			
see above	Central Public Health Laboratory - LACEN -Bahia, Salvador, Brazil	Central Public Health Laboratory - LACEN - Bahia, Salvador, Brazil	Arabela Leal; Breno Dominguez; Felicidade Pereira; Jaqueline Gomes; Luciana Oliveira; Luiz Alcantara; Marcela Gómez; Marta Giovanetti; Patrícia Cajado; Stephane Tosta; Vagner Fonseca; Vanessa Nardy
EPI_ISL_515547	Centro Medico da Policia Militar do Estado de Sao Paulo	Instituto Adolfo Lutz, Interdiciplinary Procedures Center, Strategic Laboratory	Claudia Regina Gonçalves; Claudio Tavares Sacchi; Erica Valessa Ramos Gomes
EPI_ISL_693244	Centro Médico da Polícia Militar do Estado de Sao Paulo	Instituto Adolfo Lutz, Interdiciplinary Procedures Center, Strategic Laboratory	Claudia Regina Gonçalves; Claudio Tavares Sacchi; Erica Valessa Ramos Gomes; Karoline Rodrigues Campos
EPI_ISL_2612324, EPI_ISL_2612328, EPI_ISL_2612348, EPI_ISL_2612349, EPI_ISL_2612353, EPI_ISL_2612355, EPI_ISL_2612361, EPI_ISL_2612364, EPI_ISL_2612393, EPI_ISL_2612394, EPI_ISL_2612395			
see above	Centro de Infectologia Charles Mérieux/ Laboratório Rodolphe Mérieux, FUNDHACRE	Bioinformatics Laboratory / LNCC	Alessandra P Lamarca; Alexandra L Gerber; Ana Paula de C Guimarães; Ana Tereza R Vasconcelos; Andreas Stocker; Cirley Maria de Oliveira Lobato; Douglas Terra Machado; Luiz Fellype Alves de Souza; Luiz G P de Almeida; Ronaldo da Silva F Jr
EPI_ISL_2491693, EPI_ISL_2491756, EPI_ISL_2491757, EPI_ISL_2491758, EPI_ISL_2491760, EPI_ISL_2491762, EPI_ISL_2491772, EPI_ISL_2491780			
see above	Centro de Pesquisa Gonçalo Moniz (CPqGM - FIOCRUZ/BA)	Laboratory of Respiratory Viruses and Measles, Oswaldo Cruz Institute, FIOCRUZ	Alice Sampaio Rocha; Ana Carolina Mendonca; Anna Carolina Paixao; Camila I. de Oliveira; Elisa Cavalcante Pereira; Fernando Motta; Luciana Appolinario; Marilda Siqueira on behalf of the Fiocruz COVID-19 Genomic Surveillance Network; Paola Resende; Renata Serrano Lopes; Ricardo Khouri; Taina Venas
EPI_ISL_583500	Centro de Saude I Tacito Leite de Carvalho e Silva	Instituto Adolfo Lutz, Interdiciplinary Procedures Center, Strategic Laboratory	Claudia Regina Gonçalves; Claudio Tavares Sacchi; Erica Valessa Ramos Gomes; Karoline Rodrigues Campos
EPI_ISL_1493591	Centro de Saude II Dr Alcides Facundo Arroyo	Instituto Adolfo Lutz, Interdiciplinary Procedures Center, Strategic Laboratory	Caio Vinicius Dias Lopes; Claudia Regina Gonçalves; Claudio Tavares Sacchi; Erica Valessa Ramos Gomes; Karoline Rodrigues Campos
EPI_ISL_1520134, EPI_ISL_1520135	Centro de Saude II Dr Jose Paione Mococa	Instituto Adolfo Lutz, Interdiciplinary Procedures Center, Strategic Laboratory	Caio Vinicius Dias Lopes; Claudia Regina Gonçalves; Claudio Tavares Sacchi; Erica Valessa Ramos Gomes; Karoline Rodrigues Campos
EPI_ISL_1533691	Centro de Saude II Dr. Jose Paione Mococa	Instituto Adolfo Lutz, Interdiciplinary Procedures Center, Strategic Laboratory	Caio Vinicius Dias Lopes; Claudia Regina Gonçalves; Claudio Tavares Sacchi; Erica Valessa Ramos Gomes; Karoline Rodrigues Campos; Leonardo Jose Tadeu de Araujo
EPI_ISL_1396459	Centro de Tecnología en Salud Pública de la Universidad Nacional de Rosario	Laboratorio Mixto de Biotecnología Acuática (LMBA) on behalf of 'Proyecto Argentino Interinstitucional de genomica de SARS-CoV-2' (PAIS Consortium)	Adriana Giri; Agustina Cerri; Ana Cavatorta; Ana Paletta; Diego Chouhy; Elisa Bolatti; Elizabeth Tapia (argenTAG); Federico Remes Lenicov; Flavio Spetale; Gastón Viarengo; Ignacio García Labari; Javier Murillo; Joaquín Ezpeleta; Julian Acosta; Laura Angelone; Leandro Ciappina; María Re; Pablo Casal; Pilar Bulacio; Silvana Spinelli; Silvia Arranz; Sofía Lavista Llanos; Vanina Villanova; Victoria Posner
EPI_ISL_882659	Centro de Triagem Covid19	Instituto Adolfo Lutz, Interdiciplinary Procedures Center, Strategic Laboratory	Claudia Regina Gonçalves; Claudio Tavares Sacchi; Erica Valessa Ramos Gomes; Karoline Rodrigues Campos
EPI_ISL_468305, EPI_ISL_468307, EPI_ISL_735411, EPI_ISL_735424, EPI_ISL_735426	Centro de Vigilancia a Saude de Diadema	Instituto Adolfo Lutz, Interdiciplinary Procedures Center, Strategic Laboratory	Claudia Regina Gonçalves; Claudio Tavares Sacchi; Erica Valessa Ramos Gomes; Karoline Rodrigues Campos
EPI_ISL_693240,	Centro de Vigilância	Instituto Adolfo Lutz,	Claudia Regina Gonçalves; Claudio Tavares Sacchi; Erica Valessa Ramos Gomes; Karoline Rodrigues Campos

EPI_ISL_693242	a Saude de Diadema	Interdiciplinary Procedures Center, Strategic Laboratory	
EPI_ISL_1164590	Charité Universitätsmedizin Berlin, Institut für Virologie/Labor Berlin	Charité Universitätsmedizin Berlin, Institut für Virologie	Barbara Mühlemann; Christian Drosten; Julia Schneider; Jörn Beheim-Schwarzbach; Talitha Veith; Terry Jones; Victor M Corman
EPI_ISL_581703	Clinical Virology	Clinical Bacteriology	Adrian Egli; Alexander Gensch; Alfredo Mari; Christian Nickel; Hans Hirsch; Hans Pargger; Helena MB Seth-Smith; Julia Bielicki; Karoline Leuzinger; Kirstine K. Soegaard; Madlen Stange; Manuel Battegay; Martin Siegemund; Michael Osthoff; Michael Schweitzer; Myrta Brunner; Rita Schneider-SliFemalea; Roland Bingisser; Sarah Tschudin-Sutter; Simon Fuchs; Stefano Bassetti; Tim Roloff
EPI_ISL_2277174, EPI_ISL_2309973	Colorado Department of Public Health and Environment	Colorado Department of Public Health and Environment	Alexandria Rossheim; Diana Ir; Emily A. Travanty; Laura Bankers; Molly C. Hetherington-Rauth; Sarah Elizabeth Totten; Shannon R. Matzinger
EPI_ISL_583497	Complexo Hospitalar Ouro Verde de Campinas	Instituto Adolfo Lutz, Interdiciplinary Procedures Center, Strategic Laboratory	Claudia Regina Gonçalves; Claudio Tavares Sacchi; Erica Valessa Ramos Gomes; Karoline Rodrigues Campos
EPI_ISL_1121323	Complexo Hospitalar Padre Bentode Guarulhos	Instituto Adolfo Lutz, Interdiciplinary Procedures Center, Strategic Laboratory	Caio Vinicius Dias Lopes; Claudia Regina Gonçalves; Claudio Tavares Sacchi; Erica Valessa Ramos Gomes; Karoline Rodrigues Campos
EPI_ISL_523970	Conjunto Hospitalar do Mandaqui	Instituto Adolfo Lutz, Interdiciplinary Procedures Center, Strategic Laboratory	Claudia Regina Gonçalves; Claudio Tavares Sacchi; Erica Valessa Ramos Gomes
EPI_ISL_2105543, EPI_ISL_2156282, EPI_ISL_2156299, EPI_ISL_2171229, EPI_ISL_2188677, EPI_ISL_2188728, EPI_ISL_2188775			
see above	Cotabato Regional and Medical Center	Philippine Genome Center	Alethea R. de Guzman; Anna Ong-Lim; Arianne A. Zamora; Asia Louisa U. Chong; Benedict A. Maralit; Candice Francheska B. Tambaoan; Carlo M. Lapid; Celia Carlos; Devon Ray Pacial; Edsel Maurice Salvaña; El King D. Morado; Elcid Aaron R. Pangilinan; Eva Maria Cutiongco-de la Paz; Francis A. Tablizo; Irish Coleen A. Asin; Jaime C. Montoya; Jan Michael C. Yap; Jo-Hannah S. Llames; John Q. Wong; Joshua Gregor A. Dizon; Juan Antonio R. Magalang; Karol Sophia Agape R. Padilla; Kenneth M. Kim; Kris P. Punayan; Marc Edsel C. Ayes; Marc Jerrone R. Castro; Maria Rosario Singh-Vergeire and Cynthia P. Saloma; Maria Sofia L. Yangzon; Marissa Alejandria; Razel Nikka M. Hao; Renato Jacinto Q. Mantaring; Rianna Patricia S. Cruz; Sheila Mae M. Araiza
EPI_ISL_693207	Cs II Doutor Antonio Vicoso Moreira de Rezende	Instituto Adolfo Lutz, Interdiciplinary Procedures Center, Strategic Laboratory	Claudia Regina Gonçalves; Claudio Tavares Sacchi; Erica Valessa Ramos Gomes; Karoline Rodrigues Campos
EPI_ISL_833168	DB Diagnosticos do Brasil	Instituto Adolfo Lutz, Interdiciplinary Procedures Center, Strategic Laboratory	Claudia Regina Gonçalves; Claudio Tavares Sacchi; Erica Valessa Ramos Gomes; Karoline Rodrigues Campos
EPI_ISL_476282	DB Diagnósticos do Brasil	Instituto de Medicina Tropical da Univesidade de São Paulo	Camila Alves Maia da Silva; Darlan da Silva Candido; Erika Regina Manuli; Ester Sabino; Flavia Cristina da Silva Sales; Giula Magalhaes Ferreira; Jaqueline Goes de Jesus; Julien Theze; Mariana Severo Ramundo; Nuno Faria; Samples: Nelson Gaburo Jr; Sequencing: Ingra Morales Claro; Thais de Moura Coletti
EPI_ISL_1469579, EPI_ISL_1469633, EPI_ISL_1469637, EPI_ISL_1469638, EPI_ISL_1469675, EPI_ISL_1469677, EPI_ISL_1469684, EPI_ISL_1469692, EPI_ISL_1469731, EPI_ISL_1469781, EPI_ISL_1479121			
see above	DIRETORIA DE VIGILANCIA EM SAUDE	Epiclin	Ana Paula Muterle; Carolina Comerlato; Eliana Márcia Da Ros Wendland; Fernando Hayashi Sant'Anna; Janira Prichula; Juliana Comerlato
EPI_ISL_2344691	DIRETORIA MUNICIPAL DE SAUDE DE ENGENHEIRO COELHO	Instituto Butantan / FZEA-USP-Pirassununga	Antonio Jorge Martins; Claudia Renata dos Santos Barros; David Schlesinger; Debora Botequio Moretti; Dimas Tadeu Covas; Elaine Cristina Marqueze; Elaine Vieira Santos; Evandra Strazza Rodrigues; Heidge Fukumasu; Jayme Augusto de Souza-Neto; José Salvatore Leister Patané; Luiz Alcantara; Luiz Lehmann Coutinho; Maria Carolina Elias; Maurício Lacerda Nogueira; Rafael dos Santos Bezerra; Raul Machado Neto; Rejane Maria Tommasini Grotto; Ricardo Haddad; Sandra Coccuzzo Sampaio Vessoni; Simone Kashima; Svetoslav Nanev Slavov; Vincent Louis Viala
EPI_ISL_857239	DOHMH Corona	New York City Public Health Laboratory	Jade Wang; et al.
EPI_ISL_937224	DOHMH Jamaica	New York City Public Health Laboratory	Jade Wang; et al.
EPI_ISL_2105331, EPI_ISL_2156371, EPI_ISL_2156403, EPI_ISL_2156405, EPI_ISL_2156406	Davao One World Diagnostic Center Incorporated	Philippine Genome Center	Alethea R. de Guzman; Anna Ong-Lim; Arianne A. Zamora; Asia Louisa U. Chong; Benedict A. Maralit; Candice Francheska B. Tambaoan; Carlo M. Lapid; Celia Carlos; Devon Ray Pacial; Edsel Maurice Salvaña; El King D. Morado; Elcid Aaron R. Pangilinan; Eva Maria Cutiongco-de la Paz; Francis A. Tablizo; Irish Coleen A. Asin; Jaime C. Montoya; Jan Michael C. Yap; Jo-Hannah S. Llames; John Q. Wong; Joshua Gregor A. Dizon; Juan Antonio R. Magalang; Karol Sophia Agape R. Padilla; Kenneth M. Kim; Kris P. Punayan; Marc Edsel C. Ayes; Marc Jerrone R. Castro; Maria Rosario Singh-Vergeire and Cynthia P. Saloma; Maria Sofia L. Yangzon; Marissa Alejandria; Razel Nikka M. Hao; Rianna Patricia S. Cruz; Sheila Mae M. Araiza
EPI_ISL_906065	Day Hospital de Ermelino Matarazzo	Instituto Adolfo Lutz, Interdiciplinary Procedures Center, Strategic Laboratory	Claudia Regina Gonçalves; Claudio Tavares Sacchi; Erica Valessa Ramos Gomes; Karoline Rodrigues Campos
EPI_ISL_1939017	Departamento de Microbiología, CDB, Hospital Clínic, Barcelona	SeqCOVID-SPAIN consortium/IBV(CSIC)	Aida Peiró and SeqCOVID-SPAIN consortium; Andrea Vergara; Elisa Rubio; Jéssica Navero; Mikel Martínez
EPI_ISL_603034	Departamento de Vigilância à Saúde	Instituto Adolfo Lutz, Interdiciplinary Procedures Center, Strategic Laboratory	Claudia Regina Gonçalves; Claudio Tavares Sacchi; Erica Valessa Ramos Gomes; Karoline Rodrigues Campos
EPI_ISL_1340751, EPI_ISL_1340754, EPI_ISL_1340756, EPI_ISL_1340757, EPI_ISL_1340764	Departamento de Virologia, Laboratorio Central de Salud Pública, Avenida Venezuela y Teniente Escurra, Asunción, Paraguay	Laboratory of Respiratory Viruses and Measles, Oswaldo Cruz Institute, FIOCRUZ	Alice Sampaio Rocha; Ana Carolina Mendonca; Anna Carolina Paixao; Cynthia Vazquez; Fernando Motta; Luciana Appolinario; Marilda Siqueira on behalf of the Fiocruz COVID-19 Genomic Surveillance Network; Paola Resende; Renata Serrano Lopes
EPI_ISL_1667472	Department of Laboratory Medicine, National Taiwan University Hospital	Microbial Genomics Core Lab, National Taiwan University Centers of Genomic and Precision Medicine	Chiao-Ling Li; Pei-Jer Chen; Shan-Chwen Chang; Shiou-Hwei Yeh; Sui-Yuan Chang; Ya-Yun Lai; You-Yu Lin
EPI_ISL_1034353	Department of Medical Microbiology, St. Olavs hospital	Norwegian Institute of Public Health, Department of Virology	Atiya R Ali; Hilde Elshaug; Hilde Vollan; Ignacio Garcia Llorente; Kamilla Heddeland Instefjord; Karoline Bragstad; Kathrine Stene-Johansen; Marie Paulsen Madsen; Olav Hungnes; Rasmus Riis Kopperud; Serina B Engebretsen
EPI_ISL_619577	Department of Virus and Microbiological Special Diagnostics, Statens Serum Institut, Denmark	Albertsen lab, Department of Chemistry and Bioscience, Aalborg University, Denmark	Danish Covid-19 Genome Consortia
EPI_ISL_1092725	Diagnosticos da America - DASA	Instituto Adolfo Lutz, Interdiciplinary Procedures Center, Strategic Laboratory	Claudia Regina Gonçalves; Claudio Tavares Sacchi; Erica Valessa Ramos Gomes; Karoline Rodrigues Campos
EPI_ISL_1533724	Diretoria Municipal de Saude	Instituto Adolfo Lutz, Interdiciplinary	Caio Vinicius Dias Lopes; Claudia Regina Gonçalves; Claudio Tavares Sacchi; Erica Valessa Ramos Gomes; Karoline Rodrigues Campos; Leonardo Jose Tadeu de Araujo

		Procedures Center, Strategic Laboratory	
EPI_ISL_1469560, EPI_ISL_1469609, EPI_ISL_1469610, EPI_ISL_1469616, EPI_ISL_1469623, EPI_ISL_1469625, EPI_ISL_1469627, EPI_ISL_1469656, EPI_ISL_1469669, EPI_ISL_1469708, EPI_ISL_1469748, EPI_ISL_1469783, EPI_ISL_1469792, EPI_ISL_1469796, EPI_ISL_1469802, EPI_ISL_1469808, EPI_ISL_1469823, EPI_ISL_1469835			
see above	Diretoria de Vigilância em Saúde	Epiclin	Ana Paula Muterle; Carolina Comerlato; Eliana Márcia Da Ros Wendland; Fernando Hayashi Sant'Anna; Janira Prichula; Juliana Comerlato
EPI_ISL_583499	Distrito Sanitario Sul Campinas	Instituto Adolfo Lutz, Interdiciplinary Procedures Center, Strategic Laboratory	Claudia Regina Gonçalves; Claudio Tavares Sacchi; Erica Valessa Ramos Gomes; Karoline Rodrigues Campos
EPI_ISL_747337, EPI_ISL_850198, EPI_ISL_2161051, EPI_ISL_2161155	Division of Emerging Infectious Diseases, Bureau of Infectious Diseases Diagnosis Control, Korea Disease Control and Prevention Agency	Division of Emerging Infectious Diseases, Bureau of Infectious Diseases Diagnosis Control, Korea Disease Control and Prevention Agency	Ae Kyung Park; Chae Young Lee; Chaeyoung Lee; Eun-Jin Kim; Heui Man Kim; Il-Hwan Kim; Jeong-Ah Kim; Jeong-Min Kim; Jin Sun No; Namjoo Lee; Sang Hee Woo
EPI_ISL_2105306, EPI_ISL_2105360, EPI_ISL_2105365, EPI_ISL_2105433, EPI_ISL_2105541, EPI_ISL_2188278	Dr. Jorge P. Royeca Hospital	Philippine Genome Center	Alethea R. de Guzman; Anna Ong-Lim; Arianne A. Zamora; Asia Louisa U. Chong; Benedict A. Maralit; Candice Francheska B. Tambaoan; Carlo M. Lapid; Celia Carlos; Devon Ray Pacial; Edsel Maurice Salvaña; El King D. Morado; Elcid Aaron R. Pangilinan; Eva Maria Cutiongco-de la Paz; Francis A. Tablizo; Irish Coleen A. Asin; Jaime C. Montoya; Jan Michael C. Yap; Jo-Hannah S. Llames; John Q. Wong; Joshua Gregor A. Dizon; Juan Antonio R. Magalang; Karol Sophia Agape R. Padilla; Kenneth M. Kim; Kris P. Punayan; Marc Edsel C. Ayes; Marc Jerrone R. Castro; Maria Rosario Singh-Vergeire and Cynthia P. Saloma; Maria Sofia L. Yangzon; Marissa Alejandria; Razel Nikka M. Hao; Rianna Patricia S. Cruz; Sheila Mae M. Araiza
EPI_ISL_1063789	Evandro Chagas Institute	Evandro Chagas Institute Virology	A.M.; Barbagelata; E.C.; E.M.A.; Ferreira; J.A.; Junior; K.C.; L.C.; L.S.; M.C.; P.S.; Pinheiro; Santos; Silva; Sousa; Sousa Junior; W.D.C.; da Silva
EPI_ISL_1469555, EPI_ISL_1469572, EPI_ISL_1469620, EPI_ISL_1469661	FUNDACAO DE SAUDE PUBLICA DE NOVO HAMBURGO FSNH	Epiclin	Ana Paula Muterle; Carolina Comerlato; Eliana Márcia Da Ros Wendland; Fernando Hayashi Sant'Anna; Janira Prichula; Juliana Comerlato
EPI_ISL_1469704, EPI_ISL_1469723	FUNDACAO DE SAUDE PUBLICA SAO CAMILO DE ESTEIO	Epiclin	Ana Paula Muterle; Carolina Comerlato; Eliana Márcia Da Ros Wendland; Fernando Hayashi Sant'Anna; Janira Prichula; Juliana Comerlato
EPI_ISL_1469586	FUNDACAO HOSPITALAR SAO JOSE	Epiclin	Ana Paula Muterle; Carolina Comerlato; Eliana Márcia Da Ros Wendland; Fernando Hayashi Sant'Anna; Janira Prichula; Juliana Comerlato
EPI_ISL_2105553	First Aide Diagnostic Center	Philippine Genome Center	Alethea R. de Guzman; Anna Ong-Lim; Arianne A. Zamora; Asia Louisa U. Chong; Benedict A. Maralit; Candice Francheska B. Tambaoan; Carlo M. Lapid; Celia Carlos; Devon Ray Pacial; Edsel Maurice Salvaña; El King D. Morado; Eva Maria Cutiongco-de la Paz; Francis A. Tablizo; Irish Coleen A. Asin; Jaime C. Montoya; Jan Michael C. Yap; Jo-Hannah S. Llames; John Q. Wong; Joshua Gregor A. Dizon; Juan Antonio R. Magalang; Karol Sophia Agape R. Padilla; Kenneth M. Kim; Kris P. Punayan; Marc Edsel C. Ayes; Marc Jerrone R. Castro; Maria Rosario Singh-Vergeire and Cynthia P. Saloma; Maria Sofia L. Yangzon; Marissa Alejandria; Razel Nikka M. Hao; Rianna Patricia S. Cruz; Sheila Mae M. Araiza
EPI_ISL_431180, EPI_ISL_431240	Fujian Center for Disease Control and Prevention	Fujian Center for Disease Control and Prevention	Huang Zhimiao; Lin Qi; Weng Yuwei; Zhang Yanhua
EPI_ISL_1182550, EPI_ISL_1182587, EPI_ISL_1182601, EPI_ISL_1182602, EPI_ISL_1182609, EPI_ISL_1182612	Fundação Ezequiel Dias (FUNED)	Coordenação Geral de Laboratórios de Saúde Pública (CGLAB/DAEVS/SVS/MS)	Vagner Fonseca; et al.
EPI_ISL_1469713, EPI_ISL_1469845	Fundação Hospitalar de Sapucaia do Sul	Epiclin	Ana Paula Muterle; Carolina Comerlato; Eliana Márcia Da Ros Wendland; Fernando Hayashi Sant'Anna; Janira Prichula; Juliana Comerlato
EPI_ISL_1469561	Fundação de Saúde Pública São Camilo de Esteio	Epiclin	Ana Paula Muterle; Carolina Comerlato; Eliana Márcia Da Ros Wendland; Fernando Hayashi Sant'Anna; Janira Prichula; Juliana Comerlato
EPI_ISL_3320805	Furst Medical Laboratory	Norwegian Institute of Public Health, Department of Virology	Atiya R Ali; Debech Nadia; Engebretsen Serina Beate; Garcia Llorente Ignacio; Hilde Elshaug; Hilde Vollan; Jon Bråte; Kamilla Heddeland Instefjord; Karoline Bragstad; Kathrine Stene-Johansen; Line Victoria Moen; Marie Paulsen Madsen; Olav Hungnes; Pedersen Benedikte Nevjen; Rasmus Riis Kopperud
EPI_ISL_746686, EPI_ISL_1167743, EPI_ISL_1321466	Genetica Molecular and Subdepartamento de Virologia ISP Chile	Instituto de Salud Publica de Chile	Andres Castillo; Barbara Parra; Gisselle Barra; Jaime Lagos; Javier Tognarelli; Jorge Fernandez; Karen Orostica; Loredana Arata; Patricia Bustos; Rodrigo Fasce
EPI_ISL_861914	Genomika Einstein	LATE - Laboratório de Técnicas Especiais - Hospital Israelita Albert Einstein	Ana Paula Moreira Salles; Deyvid Amgarten; Fernanda de Mello Malta; João Bosco Oliveira Filho; João Renato Rebello Pinho; Pedro Henrique Sebe Rodrigues; Raquel Riyuzo
EPI_ISL_960776	Germano de sousa	Instituto Gulbenkian de Ciencia	Cathy Paulino; João Costa; João Sobral; Maria Costa; Ricardo Leite; Susana Ladeiro
EPI_ISL_745827, EPI_ISL_745855	Ginkgo Bioworks Clinical Laboratory	Utah Public Health Laboratory	Alex Plocik; Becky Schilling; Birgitte Simen; David R. Hillyard; E. Susan Slechta; Erin L. Young; James McGann; Jeffrey B. Stevenson; Jim Griffin; Keith Robison; Kelly Oakeson; Malaika McKenzie-Bennett; Martha Pierson; Melanie A. Mallory; Michael T. Pyne; Michelle Spencer; Rebecca Littlefield; Salika M. Shakir; Tara Gallagher
EPI_ISL_1503139	Gorgas Memorial Laboratory of Health Studies	Gorgas Memorial Laboratory of Health Studies	Castillo Jorge; Franco Danilo; Gonzalez Claudia; Jessica Gondola; Leyda Abrego; Lopez-Verges Sandra; Marlenne Castillo; Martinez Alexander; Moreno Ambar; Moreno Brechla; Oris Chavarria; Ortiz Alma
EPI_ISL_508266	Government Medical College	National Institute of Biomedical Genomics	Arindam Maitra; Dhaval Khatri; Jyoti Iravane; Maitrik Dave; Saumitra Das
EPI_ISL_2105471, EPI_ISL_2105483, EPI_ISL_2105502, EPI_ISL_2105555	Governor Celestino Gallares Memorial Hospital	Philippine Genome Center	Alethea R. de Guzman; Anna Ong-Lim; Arianne A. Zamora; Asia Louisa U. Chong; Benedict A. Maralit; Candice Francheska B. Tambaoan; Carlo M. Lapid; Celia Carlos; Devon Ray Pacial; Edsel Maurice Salvaña; El King D. Morado; Eva Maria Cutiongco-de la Paz; Francis A. Tablizo; Irish Coleen A. Asin; Jaime C. Montoya; Jan Michael C. Yap; Jo-Hannah S. Llames; John Q. Wong; Joshua Gregor A. Dizon; Juan Antonio R. Magalang; Karol Sophia Agape R. Padilla; Kenneth M. Kim; Kris P. Punayan; Marc Edsel C. Ayes; Marc Jerrone R. Castro; Maria Rosario Singh-Vergeire and Cynthia P. Saloma; Maria Sofia L. Yangzon; Marissa Alejandria; Razel Nikka M. Hao; Rianna Patricia S. Cruz; Sheila Mae M. Araiza
EPI_ISL_1040815	Groote Schuur Hospital wc GSH	NHLS/UCT	Arash Iranzadeh; Bruna Galvao; Carolyn Williamson; Deelan Doolabh; Diana Hardie; Innocent Mudau; Kruger Marais; Lynn Tyers; Marvin Hsiao; Stephen Korsman
EPI_ISL_3127913	Guam Public Health Laboratory	Centers for Disease Control and Prevention Division of Viral Diseases, Pathogen Discovery	Alex Burgin; Ben L. Rambo-Martin; Clinton R. Paden; Dakota Howard; Dave Wentworth; Dhwani Batra; Jasmine Padilla; Justin Lee; Krista Queen; Kristen Knipe; Kristine Lacek; Mark Burroughs; Matthew Schmerer; Meghan Bentz; Mili Sheth; Peter Cook; Sam Shepard; Sarah Nobles; Suxiang Tong; Vivien Dugan; Yvette Unoarumhi
EPI_ISL_732044, EPI_ISL_732052	H Beatriz Angelo	Instituto Nacional de Saude (INSA)	Borges et al
EPI_ISL_1117216	H Guimaraes	Instituto Nacional de Saude (INSA)	Borges et al
EPI_ISL_1172014	HC_FMUSP	Laboratório de Parasitologia Médica - Instituto de Medicina Tropical - Universidade de São Paulo	Brazil-UK Centre for Arbovirus Discovery Diagnosis Genomics and Epidemiology (CADDE) Genomic Network - Instituto de Medicina Tropical
EPI_ISL_3912417	HGF HOSPITAL GERAL DE	Analytical Competence Molecular Epidemiology	Cleber Furtado Aksenen; Fabio Miyajima; Fernando Braga Stehling; Francisco Eder de Moura Lopes; Jamille Maria Mendes Bezerra; Joaquim Cesar do Nascimento Sousa Junior; Pedro Miguel Carneiro Jeronimo; Suzana Porto Almeida & Lucas Delerino on behalf of COVID-19 FIOCRUZ Genomic Network; Thais Ferreira de Oliveira; Thais de Oliveira Costa; Ticiane Cavalcante de Souza; Veridiana Pessoa Miyajima

	FORTALEZA	Lab/ACME, Oswaldo Cruz Foundation, Ceara (FIOCRUZ CE)	
EPI_ISL_2017243, EPI_ISL_2017251, EPI_ISL_2017253, EPI_ISL_2017258, EPI_ISL_2017259, EPI_ISL_2017264, EPI_ISL_2017266, EPI_ISL_2017271, EPI_ISL_2017275, EPI_ISL_2017276, EPI_ISL_2017309, EPI_ISL_2017457, EPI_ISL_2102517, EPI_ISL_2187714, EPI_ISL_2187726, EPI_ISL_2187746, EPI_ISL_2187747, EPI_ISL_2187783, EPI_ISL_2227560, EPI_ISL_2348593, EPI_ISL_2497434, EPI_ISL_2617626, EPI_ISL_3259351			
see above	HLAGYN - Laboratorio de Imunologia de Transplantes de Goias	HLAGYN - Laboratorio de Imunologia de Transplantes de Goias	Alessandro Leonardo Alvares Magalhaes; Daniel Ferreira de Sousa; Danielle de Paiva Rezende; Erika Lopes Rocha Batista; Fernando Antonio Vinhal dos Santos; Frederico Rodrigues Vinhal; Lucas Carlos Gomes Pereira; Paola Cristina Resende Silva; Raphael Bessa Parmigiane; Sabrina Sara Moreira Duarte
EPI_ISL_1912832, EPI_ISL_1912892	HMH	New York Genome Center	Andre Corvelo; Barry Kreiswirth; David Perlin; Dayna M. Oschwald; Jose Mediavilla; Kaelea Composto; Kar Chow; Liang Chen; Marcus Cunningham; Michael Zody; Samantha Fennessey; Tom Maniatis
EPI_ISL_1684086	HOSPITAL CLINIC	Instituto de Salud Carlos III	A. Monzón; F. Casas; I. Jiménez; I.VILA ESTAPE; Iglesias-Caballero; JORDI; M. Sandonís; P. Zaballos; S. Camarero; S. Cuesta; S. Pozo; S. Varona; V. Vázquez-Morón
EPI_ISL_3912420	HOSPITAL DE CAMPANHA COVID 19 DR EUDASIO BARROSO HMEB	Analytical Competence Molecular Epidemiology Lab/ACME, Oswaldo Cruz Foundation, Ceara (FIOCRUZ CE)	Cleber Furtado Aksenen; Fabio Miyajima; Fernando Braga Stehling; Francisco Eder de Moura Lopes; Jamille Maria Mendes Bezerra; Joaquim Cesar do Nascimento Sousa Junior; Pedro Miguel Carneiro Jeronimo; Suzana Porto Almeida & Lucas Delerino on behalf of COVID-19 FIOCRUZ Genomic Network; Thais Ferreira de Oliveira; Thais de Oliveira Costa; Ticiane Cavalcante de Souza; Veridiana Pessoa Miyajima
EPI_ISL_3912421	HOSPITAL E MATERNIDADE NOSSA SENHORA DOS MILAGRES	Analytical Competence Molecular Epidemiology Lab/ACME, Oswaldo Cruz Foundation, Ceara (FIOCRUZ CE)	Cleber Furtado Aksenen; Fabio Miyajima; Fernando Braga Stehling; Francisco Eder de Moura Lopes; Jamille Maria Mendes Bezerra; Joaquim Cesar do Nascimento Sousa Junior; Pedro Miguel Carneiro Jeronimo; Suzana Porto Almeida & Lucas Delerino on behalf of COVID-19 FIOCRUZ Genomic Network; Thais Ferreira de Oliveira; Thais de Oliveira Costa; Ticiane Cavalcante de Souza; Veridiana Pessoa Miyajima
EPI_ISL_1469746	HOSPITAL MONTENEGRO	Epiclin	Ana Paula Muterle; Carolina Comerlato; Eliana Márcia Da Ros Wendland; Fernando Hayashi Sant'Anna; Janira Prichula; Juliana Comerlato
EPI_ISL_3912424	HOSPITAL MUNICIPAL DE CAMPOS SALES	Analytical Competence Molecular Epidemiology Lab/ACME, Oswaldo Cruz Foundation, Ceara (FIOCRUZ CE)	Cleber Furtado Aksenen; Fabio Miyajima; Fernando Braga Stehling; Francisco Eder de Moura Lopes; Jamille Maria Mendes Bezerra; Joaquim Cesar do Nascimento Sousa Junior; Pedro Miguel Carneiro Jeronimo; Suzana Porto Almeida & Lucas Delerino on behalf of COVID-19 FIOCRUZ Genomic Network; Thais Ferreira de Oliveira; Thais de Oliveira Costa; Ticiane Cavalcante de Souza; Veridiana Pessoa Miyajima
EPI_ISL_2345448	HOSPITAL MUNICIPAL DE IBIUNA IBIUNA SP	Instituto Butantan / ESALQ-Piracicaba	Antonio Jorge Martins; Claudia Renata dos Santos Barros; David Schlesinger; Debora Botequio Moretti; Dimas Tadeu Covas; Elaine Cristina Marqueze; Elaine Vieira Santos; Evandra Strazza Rodrigues; Heidge Fukumasu; Jayme Augusto de Souza-Neto; José Salvatore Leister Patané; Luiz Alcantara; Luiz Lehmann Coutinho; Maria Carolina Elias; Maurício Lacerda Nogueira; Rafael dos Santos Bezerra; Raul Machado Neto; Rejane Maria Tommasini Grotto; Ricardo Haddad; Sandra Coccuzzo Sampaio Vessoni; Simone Kashima; Svetoslav Nanev Slavov; Vincent Louis Viala
EPI_ISL_1966922	HOSPITAL MUNICIPAL DE ITABERA	Instituto Butantan / ESALQ-USP (Piracicaba)	Antonio Jorge Martins; Bianca Cechetto Carlos. Mendelics: Bibiana Santos; Claudia Renata dos Santos Barros; Cíntia Bittar; David Schlesinger. Hemocentro Ribeirão Preto: Simone Kashima; Debora Botequio Moretti; Elaine Cristina Marqueze; Elaine Vieira dos Santos; Elisangela Chicaroni Mattos; Erika Freitas; Evandra Strazza Rodrigues; Felipe Allan da Silva da Costa; Flavia Aburjaile; Fábio Sossai Possebon; Guilherme Campos; Guilherme Targino Valente; Heidge Fukumasu. USP-Botucatu: Rejane Maria Tommasini Grotto; Helena Lage Ferreira; Instituto Butantan: Dimas Tadeu Covas; Jardelina de Souza Todao Bernardino; Jayme A. Souza-Neto; Jessika Cristina Chagas Lesbon; Jorge A. Petroli Marchesi; José Salvatore Leister Patané; João Paulo Kitajima; João Pessoa Araújo Jr.; Leila Sabrina Ullmann; Loyze Paola Oliveira de Lima; Luiz Aurelio de Campos Crispin. Centro de Genômica Funcional da ESALQ: Luiz Lehmann Coutinho; Luiz Carlos Junior de Alcantara; Lívia Sacchetto; Maisa C. Pereira Parra; Maria Carolina Elias; Marta Giovanetti; Marília Moraes; Maurício Lacerda Nogueira. Prefeitura de Sao Paulo: Melissa Palmieri.; Patricia Akemi Assato; Paula Rahal; Paulo Inacio da Costa; Rafael dos Santos Bezerra; Raquel de Lello Rocha Campos Cassano. NGS Soluções Genômicas: Pilar Drummond Sampaio Corrêa Mariani. FZEA-USP Pirassununga: Mirele Daiana Poleti; Raul Machado Neto; Ricardo Augusto Brassaloti; Ricardo Haddad; Rodrigo Tocantins Calado. FAMERP-SJRP: Cecília Artico Banho; Sandra Coccuzzo Sampaio; Svetoslav Nanev Slavov; Vagner Fonseca; Vincent Louis Viala
EPI_ISL_1469624, EPI_ISL_1469631	HOSPITAL MUNICIPAL GETULIO VARGAS	Epiclin	Ana Paula Muterle; Carolina Comerlato; Eliana Márcia Da Ros Wendland; Fernando Hayashi Sant'Anna; Janira Prichula; Juliana Comerlato
EPI_ISL_1510080, EPI_ISL_1510081	HOSPITAL SAN PEDRO DE ALCANTARA - C.D. 05	Instituto de Salud Carlos III	A. Monzón; F. Casas; GUADALUPE; I. Jiménez; I.RODRIGUEZ RODRIGUEZ; Iglesias-Caballero; M. Sandonís; P. Zaballos; S. Camarero; S. Cuesta; S. Pozo; S. Varona; V. Vázquez-Morón
EPI_ISL_2758675, EPI_ISL_2758699, EPI_ISL_2758700, EPI_ISL_2758702, EPI_ISL_2758703, EPI_ISL_2758704, EPI_ISL_2758708, EPI_ISL_2758712, EPI_ISL_2758767, EPI_ISL_2758768, EPI_ISL_2758769, EPI_ISL_2758770, EPI_ISL_2758774, EPI_ISL_2758775			
see above	HUEM/IBMP	IPEC Guarapuava	NAPI-Genômica (Novos Arranjo de Pesquisa e Inovação em Genômica): Ademar Dantas da Cunha Júnior Adriano Ferrasa Adriano Mondini Aldo Przybysz Alessandra Lourenço Cecchini Armani Alex Sandro Jorge Alexandra Ivo de Medeiros Alexandre Maller Aline Cristina Batista Rodrigues Johann Ana Lucia Ferreira Ana Marisa Fusco Almeida Anderson Joel Martino Andrade André Luís Laforga Vanzela Andrea Duarte Doetzer Andrea Name Colado Simao Andressa Pereira de Souza Anelisa Ramão Angelica Beate Winter Boldt Anna Hermínia Castro Gomes de Amorim Anna Silvia Penteado Setti da Rocha Antonio Camilo da Silva Filho Antonio Stabelini Neto Arthur Hirata Bertachi Barbara Mendes Paz Chao Betty Cristiane Kuhn Bruno Ambrozio Galindo Bruno Ribeiro Cruz Camilla Reginatto De Pierri Carla Fredrichsen Moya Araujo Carla Fredrichsen Moya Araujo Carlos Alberto Oliveira de Biagi Junior Carlos Augusto Nassar Carlos Eduardo Buss Carlos Gilberto Carlotti Junior Carlos Henrique Schneider Carolina Panis Carolina Weigert Galvão Caroline de Jesus Coelho Donha Caroline Guisantes de Salvo Toni Caryna Eurich Mazur Catiuscie Cabreira da Silva Tortorella Celso F. D. Doliveira Cesar Luiz Boguszewski Christiane Pienna Soares Chung Man Chin Claudia Moro Cleverson Busso Cristiane Cominetti Daiane Priscila Simão-Silva Dalila Luciola Zanette Daniel de Paula Daniel de Paula Daniel Rech Daniela Fiori Gradia Daniela Pretti da Cunha Tirapelli Daniela Viganó Zanoti Jeronymo Daniele Ukan Danielle Malheiros Ferreira Danielle Venturini Deborah Catharine de Assis Leite Deivid Calebe de Souza Dennis Armando Bertolini Edenir Inez Pamero Edna Maria Vissoci Reiche Edson Roberto Arpini Miguel Eduardo José de Almeida Araújo Eliana Carolina Vespero Eliandro Reis Tavares Elza Kimura Grimshaw Emanuel Maltempi de Souza Emanuele Cristina Gustani Buss Émerson Carraro Emiliana Cristina Melo ENILze Maria de Souza Fonseca Ribeiro Enilze Maria de Souza Fonseca Ribeiro Erika Izumi Erika Seki Kioshima Cotica Evani Marques Pereira Fabio Negretti Fábio Rodrigues Ferreira Seiva Felipe Dunin dos Santos Felipe Tuon Fernanda Andreia Rosa Fernanda Cestaro Prado Cortez Fernanda Ivanski Fernanda Maris Peria Flavia Regina Oliveira de Barros Franciele Aní Caovilla Follador Franciele Mara Lucca Zanardo Bohm Francinete Ramos Campos Fulviana Silva Nishiyama GABRIEL RIBEIRO CORDEIRO Gabriela Datsch Bennemann Gisele Santos de Oliveira Glaucio Valdameri Glauco Akelinghton Freire Vitiello Glauco Vieira Miranda Glaura Scantamburlo ALves Fernandes Guilherme Ferreira Silveira Gustavo Bianchini Porfírio Gustavo Lenci Marques Hélito Volpato Hildebrando Masshiro Nagai Huei Diana Lee Ilce Mara de Syllos Cólus Iris Rabinovich Israel Gomy Jackson Kawakami Jacques Duílio Brancher Jaime Luís Lopes Rocha Jaqueline Carvalho de Oliveira Jean Henrique da Silva Rodrigues Jean Leandro dos Santos Jeane Eliete Laguila Visentainer João Paulo Bianchi Ximenez Joaquim Manoel da Silva Jociani Ascari Joel Donazzolo Jorge Luis Maria Ruiz Jose Knopfholz José Luis da Conceição Silva José Sebastião dos Santos Joseane Carla Schabarum Juliana Cheleski Wiggers Juliana Mara Serpeloni Juliana Morini Küpper Cardoso Perseguini Karen Brajão de Oliveira Karin Braun Prado Karine Aparecida de Lima Katiany Rizzieri Caleffi Ferracioli Katiuscia de Oliveira Francisco Gabriel Kelvinson Fernandes Viana Larissa Beatriz Cossalter Larissa Danielle Bahls Pinto Laurival Antonio Vilas Boas Léia Carolina Lucio Líbero Mezzadri Neto Ligia Carla Faccin Galhardi Lirane Elize Defante Ferreto Luciana Furlaneto Maia Luciana Oliveira de Fariña Luciana Reis Azevedo Alanis Luciane Regina Cavalli Lucy Megumi Yamauchi Lioni Luis Paulo Gomes Mascarenhas Luis Paulo Gomes Mascarenhas Luís Paulo Mascarenhas Lupe Furtado Alle Lyvia Regina Biagi Silva Bertachi Mara Antonia Ramos Costa Mara L. Cordeiro Marcela Maria Birolim Marcelo Ricardo Vicari Marcia Edilaine Lopes Consolaro Marcia Holsbach Beltrame Marcia Regina Eches Perugini Marcos Abdo Arbex Marcos Pileggi MARCOS TADEU GRZELCZAK Marcus Peikriszwili Tartaruga Maria Angelica Ehara Watanabe Maria Antonia Ramos Costa Maria Claudia Gross Maria José Soares Mendes Giannini Maria Leandra Terencio Maria Lúcia Bonfleur Maria Luiza Guimarães de Oliveira Maria Luiza Petzl-Erler Mariana Abe Vicente Cavagnari Marina Kimiko Kadowaki Marise Fonseca dos Santos Marla Karine Amarante Maurício Turkiewicz Mauro Antonio Alves Castro Michel Rodrigo Zambrano Passarini Michele Potrich Michelle Orane Schemberger Milena Massumi Kozonoe Mônica Degraf Cavallin Monica Tereza Suldofski Mucio Luiz de Assis Cirino Nadia Graciele Krohn Najeh Maissar Khalil Nédia de Castilhos Ghisi Neide Tomimura Costa Neiva Leite Neyva Maria Lopes Romeiro Patrícia Amâncio da Rosa Patricia Dayane Carvalho Schaker Patricia Oehlmeyer Nassar Patrícia Savio de Araújo-Souza Patricia Silva Lucio Paulo Henrique Couto Souza Paulo Roberto Donadio Percy Nohama Quirino Alves de Lima Neto Rafael Deminice Rafael dos Santos Bezerra Raquel Alves dos Santos Renan Manozzo Galante Renata Ernlund Freitas de Macedo Rita de Cássia Garcia Simão Roberta Losi Guembarovski Roberto H. Herai Roberto Rosati Rodrigo Ferreira Rodrigo Rodrigues Matiello Rogério Neri Shinsato Rogério Pincela Mateus Rosane Aparecida Ribeiro Rosilene Fressatti Cardoso Rosilene Fressatti Cardoso Sandra Mara Guse Scós Venske Selene Elifio Esposito Sérgio Ossamu Ioshii Silvana Giuliatti Silvia Mara de Souza Halick Silvio Henrique Maia de Almeida Simone Neumann Wendt Spencer Luiz Marques Payão Stefan Wolanski Negrão Stephane Janaina de Moura Escobar Sueli Fumie Yamada Ogatta SUELI PERCIO QUINAIA Taciane Finatto Tatiana Mayumi Veiga Iriyoda Tayza Katelline Danilau Ostroski Tony Alexander Hild Valeria Valente Vanessa Nascimento Kozak Vanessa Santos Sotomaior Victor Breno Pedrosa Victoria Zeghbi Cochenski Borba Vivian Rotuno Moure Valdameri Wander Rogerio Pavanelli Weber Cláudio Francisco Nunes da Silva Willian Augusto de Melo Yohandra Reyes Torres
EPI_ISL_2758771	HUEM/LACEN	IPEC Guarapuava	NAPI-Genômica (Novos Arranjo de Pesquisa e Inovação em Genômica): Ademar Dantas da Cunha Júnior Adriano Ferrasa Adriano Mondini Aldo Przybysz Alessandra Lourenço Cecchini Armani Alex Sandro Jorge Alexandra Ivo de Medeiros Alexandre Maller Aline Cristina Batista Rodrigues Johann Ana Lucia Ferreira Ana Marisa Fusco Almeida Anderson Joel Martino Andrade André Luís Laforga Vanzela Andrea Duarte Doetzer Andrea Name Colado Simao Andressa Pereira de Souza Anelisa Ramão Angelica Beate Winter Boldt Anna Hermínia Castro Gomes de Amorim Anna Silvia Penteado Setti da Rocha Antonio Camilo da Silva Filho Antonio Stabelini Neto Arthur Hirata Bertachi Barbara Mendes Paz Chao Betty Cristiane Kuhn Bruno Ambrozio Galindo Bruno Ribeiro Cruz Camilla Reginatto De Pierri Carla Fredrichsen Moya Araujo Carla Fredrichsen Moya Araujo Carlos Alberto Oliveira de Biagi Junior Carlos Augusto Nassar Carlos Eduardo Buss Carlos Gilberto Carlotti Junior Carlos Henrique Schneider Carolina Panis Carolina Weigert Galvão Caroline de Jesus Coelho Donha Caroline Guisantes de Salvo Toni Caryna Eurich Mazur Catiuscie Cabreira da Silva Tortorella Celso F. D. Doliveira Cesar Luiz Boguszewski Christiane Pienna Soares Chung Man Chin Claudia Moro Cleverson Busso Cristiane Cominetti Daiane Priscila Simão-Silva Dalila Luciola Zanette Daniel de Paula Daniel de Paula Daniel Rech Daniela Fiori Gradia Daniela Pretti da Cunha Tirapelli Daniela Viganó Zanoti Jeronymo Daniele Ukan Danielle Malheiros Ferreira Danielle Venturini Deborah Catharine de Assis Leite Deivid Calebe de Souza Dennis Armando Bertolini Edenir Inez Pamero Edna Maria Vissoci Reiche Edson Roberto Arpini Miguel Eduardo José de Almeida Araújo Eliana Carolina Vespero Eliandro Reis Tavares Elza Kimura Grimshaw Emanuel Maltempi de Souza Emanuele Cristina Gustani Buss Émerson Carraro Emiliana Cristina Melo ENILze Maria de Souza Fonseca Ribeiro Enilze Maria de Souza Fonseca Ribeiro Erika Izumi Erika Seki Kioshima Cotica Evani Marques Pereira Fabio Negretti Fábio Rodrigues Ferreira Seiva Felipe Dunin dos Santos Felipe Tuon Fernanda Andreia Rosa Fernanda Cestaro Prado Cortez Fernanda Ivanski Fernanda Maris Peria Flavia Regina Oliveira de Barros Franciele Aní Caovilla Follador Franciele Mara Lucca Zanardo Bohm Francinete Ramos Campos Fulviana Silva Nishiyama GABRIEL RIBEIRO CORDEIRO Gabriela Datsch Bennemann Gisele Santos de Oliveira Glaucio Valdameri Glauco Akelinghton Freire Vitiello Glauco Vieira Miranda Glaura Scantamburlo ALves Fernandes Guilherme Ferreira Silveira Gustavo Bianchini Porfírio Gustavo Lenci Marques Hélito Volpato Hildebrando Masshiro Nagai Huei Diana Lee Ilce Mara de Syllos Cólus Iris Rabinovich Israel Gomy Jackson Kawakami Jacques Duílio Brancher Jaime Luís Lopes Rocha Jaqueline Carvalho de Oliveira Jean Henrique da Silva Rodrigues Jean Leandro dos Santos Jeane Eliete Laguila Visentainer João Paulo Bianchi Ximenez Joaquim Manoel da Silva Jociani Ascari Joel Donazzolo Jorge Luis Maria Ruiz Jose Knopfholz José Luis da Conceição Silva José Sebastião dos Santos Joseane Carla Schabarum Juliana Cheleski Wiggers Juliana Mara Serpeloni Juliana Morini Küpper Cardoso Perseguini Karen Brajão de Oliveira Karin Braun Prado Karine Aparecida de Lima Katiany Rizzieri Caleffi Ferracioli Katiuscia de Oliveira Francisco Gabriel Kelvinson Fernandes Viana Larissa Beatriz Cossalter Larissa Danielle Bahls Pinto Laurival Antonio Vilas Boas Léia Carolina Lucio Líbero Mezzadri Neto Ligia Carla Faccin Galhardi Lirane Elize Defante Ferreto Luciana Furlaneto Maia Luciana Oliveira de Fariña Luciana Reis Azevedo Alanis Luciane Regina Cavalli Lucy Megumi Yamauchi Lioni Luis Paulo Gomes Mascarenhas Luis Paulo Gomes Mascarenhas Luís Paulo Mascarenhas Lupe Furtado Alle Lyvia Regina Biagi Silva Bertachi Mara Antonia Ramos Costa Mara L. Cordeiro Marcela Maria Birolim Marcelo Ricardo Vicari Marcia Edilaine Lopes Consolaro Marcia Holsbach Beltrame Marcia Regina Eches Perugini Marcos Abdo Arbex Marcos Pileggi MARCOS TADEU GRZELCZAK Marcus Peikriszwili Tartaruga Maria Angelica Ehara Watanabe Maria Antonia Ramos Costa Maria Claudia Gross Maria José Soares Mendes Giannini Maria Leandra Terencio Maria Lúcia Bonfleur Maria Luiza Guimarães de Oliveira Maria Luiza Petzl-Erler Mariana Abe Vicente Cavagnari Marina Kimiko Kadowaki Marise Fonseca dos Santos Marla Karine Amarante Maurício Turkiewicz Mauro Antonio Alves Castro Michel Rodrigo Zambrano Passarini Michele Potrich Michelle Orane Schemberger Milena Massumi Kozonoe Mônica Degraf Cavallin Monica Tereza Suldofski Mucio Luiz de Assis Cirino Nadia Graciele Krohn Najeh Maissar Khalil Nédia de Castilhos Ghisi Neide Tomimura Costa Neiva Leite Neyva Maria Lopes Romeiro Patrícia Amâncio da Rosa Patricia Dayane Carvalho Schaker Patricia Oehlmeyer Nassar Patrícia Savio de Araújo-Souza Patricia Silva Lucio Paulo Henrique Couto Souza Paulo Roberto Donadio Percy Nohama Quirino Alves de Lima Neto Rafael Deminice Rafael dos Santos Bezerra Raquel Alves dos Santos Renan Manozzo Galante Renata Ernlund Freitas de Macedo Rita de Cássia Garcia Simão Roberta Losi Guembarovski Roberto H. Herai Roberto Rosati Rodrigo Ferreira Rodrigo Rodrigues Matiello Rogério Neri Shinsato Rogério Pincela Mateus Rosane Aparecida Ribeiro Rosilene Fressatti Cardoso Rosilene Fressatti Cardoso Sandra Mara Guse Scós Venske Selene Elifio Esposito Sérgio Ossamu Ioshii Silvana Giuliatti Silvia Mara de Souza Halick Silvio Henrique Maia de Almeida Simone Neumann Wendt Spencer Luiz Marques Payão Stefan Wolanski Negrão Stephane Janaina de Moura Escobar Sueli Fumie Yamada Ogatta SUELI PERCIO QUINAIA Taciane Finatto Tatiana Mayumi Veiga Iriyoda Tayza Katelline Danilau Ostroski Tony Alexander Hild Valeria Valente Vanessa Nascimento Kozak Vanessa Santos Sotomaior Victor Breno Pedrosa Victoria Zeghbi Cochenski Borba Vivian Rotuno Moure Valdameri Wander Rogerio Pavanelli Weber Cláudio Francisco Nunes da Silva Willian Augusto de Melo Yohandra Reyes Torres
EPI_ISL_1268114, EPI_ISL_1269948, EPI_ISL_1270371, EPI_ISL_1297937, EPI_ISL_2320148	Helix/Illumina	Centers for Disease Control and Prevention Division of Viral Diseases, Pathogen Discovery	Adrian Paskey; Alexandre Bolze; Ary Ascencio; Ben L. Rambo-Martin; Benjamin Rambo-Martin; Brad Sickler; Charlotte Rivera-Garcia; Christine Tran; Christopher Gulvick; Clinton R. Paden; Dakota Howard; Darlene Wagner; David Becker; Dhwani Batra; Duncan MacCannell; Efren Sandoval; Eileen de Feo; Elizabeth Cirulli; Eric Allen; Geraint Levan; James Lu; Jan Antico; Jason Caravas; Jason Nguyen; Jimmy Ramirez; Jingtao Liu; Kara Moser; Kelly Schiabor Barrett; Kim Gietzen; Magnus Isaksson; Marc Laurent; Matthew Schmerer; Matthew Tolentino; Nicole L. Washington; Peter W. Cook; Phil Febbo; Ryan Cho; Scott Sammons; Shannon Wickline; Shatavia Morrison; Sherry Wang; Simon White; Summer Galloway; Suxiang Tong; Tyler Cassens; William Lee; Yvette Unoarumhi
EPI_ISL_471562, EPI_ISL_471581, EPI_ISL_515564, EPI_ISL_523977	Hosp. Municipal Prof. Dr. Alípio Corrêa Netto	Instituto Adolfo Lutz, Interdiciplinary Procedures Center, Strategic Laboratory	Claudia Regina Gonçalves; Claudio Tavares Sacchi; Erica Valessa Ramos Gomes
EPI_ISL_693229	Hospital 8 de Maio	Instituto Adolfo Lutz, Interdiciplinary Procedures Center, Strategic Laboratory	Claudia Regina Gonçalves; Claudio Tavares Sacchi; Erica Valessa Ramos Gomes; Karoline Rodrigues Campos
EPI_ISL_574580	Hospital Cidade Tiradentes Carmen Prudente	Instituto Adolfo Lutz, Interdiciplinary Procedures Center, Strategic Laboratory	Claudia Regina Gonçalves; Claudio Tavares Sacchi; Erica Valessa Ramos Gomes; Karoline Rodrigues Campos

EPI_ISL_574591, EPI_ISL_574592, EPI_ISL_603030	Hospital Domingos Leonardo Ceravolo Presidente Prudente	Instituto Adolfo Lutz, Interdiciplinary Procedures Center, Strategic Laboratory	Claudia Regina Gonçalves; Claudio Tavares Sacchi; Erica Valessa Ramos Gomes; Karoline Rodrigues Campos
EPI_ISL_574594	Hospital Escola da Universidade de Taubate	Instituto Adolfo Lutz, Interdiciplinary Procedures Center, Strategic Laboratory	Claudia Regina Gonçalves; Claudio Tavares Sacchi; Erica Valessa Ramos Gomes; Karoline Rodrigues Campos
EPI_ISL_574588, EPI_ISL_583490	Hospital Estadual Sumare	Instituto Adolfo Lutz, Interdiciplinary Procedures Center, Strategic Laboratory	Claudia Regina Gonçalves; Claudio Tavares Sacchi; Erica Valessa Ramos Gomes; Karoline Rodrigues Campos
EPI_ISL_583501	Hospital Estadual de CampanhaCOVID 19 Barradas	Instituto Adolfo Lutz, Interdiciplinary Procedures Center, Strategic Laboratory	Claudia Regina Gonçalves; Claudio Tavares Sacchi; Erica Valessa Ramos Gomes; Karoline Rodrigues Campos
EPI_ISL_1647843, EPI_ISL_1647850	Hospital General Universitario Gregorio Marañón	Hospital General Universitario Gregorio Marañón	Cristina Rodriguez-Grande; Darío García de Viedma; Laura Pérez-Lago; Patricia Muñoz; Pedro Sola Campoy; Pilar Catalán; Sergio Buenestado Serrano
EPI_ISL_852976	Hospital General Universitario Gregorio Marañón	SeqCOVID-SPAIN consortium/IBV(CSIC)	Darío García de Viedma; Julia Suárez; Laura Pérez-Lago; Marta Herranz; Patricia Muñoz and SeqCOVID-SPAIN consortium; Pedro J Sola-Campoy; Pilar Catalán; Sergio Buenestado-Serrano; Victor Manuel de la Cueva
EPI_ISL_471541, EPI_ISL_523971	Hospital Geral Santa Marcelina	Instituto Adolfo Lutz, Interdiciplinary Procedures Center, Strategic Laboratory	Claudia Regina Gonçalves; Claudio Tavares Sacchi; Erica Valessa Ramos Gomes
EPI_ISL_534317	Hospital Geral de Itapevi	Instituto Adolfo Lutz, Interdiciplinary Procedures Center, Strategic Laboratory	Claudia Regina Gonçalves; Claudio Tavares Sacchi; Erica Valessa Ramos Gomes
EPI_ISL_603037	Hospital Geral de Pedreira	Instituto Adolfo Lutz, Interdiciplinary Procedures Center, Strategic Laboratory	Claudia Regina Gonçalves; Claudio Tavares Sacchi; Erica Valessa Ramos Gomes; Karoline Rodrigues Campos
EPI_ISL_861636	Hospital Geral de Sao Mateus São Paulo	Instituto Adolfo Lutz, Interdiciplinary Procedures Center, Strategic Laboratory	Claudia Regina Gonçalves; Claudio Tavares Sacchi; Erica Valessa Ramos Gomes; Karoline Rodrigues Campos
EPI_ISL_515555	Hospital Geral de Vila Nova Cachoeirinha	Instituto Adolfo Lutz, Interdiciplinary Procedures Center, Strategic Laboratory	Claudia Regina Gonçalves; Claudio Tavares Sacchi; Erica Valessa Ramos Gomes
EPI_ISL_574595	Hospital Geral de Vila Penteado Dr. Jose Pamgella	Instituto Adolfo Lutz, Interdiciplinary Procedures Center, Strategic Laboratory	Claudia Regina Gonçalves; Claudio Tavares Sacchi; Erica Valessa Ramos Gomes; Karoline Rodrigues Campos
EPI_ISL_523957	Hospital Itamaraty	Instituto Adolfo Lutz, Interdiciplinary Procedures Center, Strategic Laboratory	Claudia Regina Gonçalves; Claudio Tavares Sacchi; Erica Valessa Ramos Gomes
EPI_ISL_524462	Hospital Metropolitano	Instituto Adolfo Lutz, Interdiciplinary Procedures Center, Strategic Laboratory	Claudia Regina Gonçalves; Claudio Tavares Sacchi; Erica Valessa Ramos Gomes
EPI_ISL_3031336	Hospital Metropolitano Dr. Célio de Castro	Instituto René Rachou / Fiocruz Minas	Anna Salim; Cristina Fonseca; Gabriel Fernandes; Mariana Melo; Núbia Fernandes; Pedro Alves; Rosiane Pereira; Rubens do Monte Neto; Sandra Gava; Thaís Santos; Thaís Silva; Wilma Patrícia Bernardes
EPI_ISL_515541, EPI_ISL_515561	Hospital Montemagno	Instituto Adolfo Lutz, Interdiciplinary Procedures Center, Strategic Laboratory	Claudia Regina Gonçalves; Claudio Tavares Sacchi; Erica Valessa Ramos Gomes
EPI_ISL_534318, EPI_ISL_693208, EPI_ISL_693209	Hospital Municipal Antonio Giglio	Instituto Adolfo Lutz, Interdiciplinary Procedures Center, Strategic Laboratory	Claudia Regina Gonçalves; Claudio Tavares Sacchi; Erica Valessa Ramos Gomes; Karoline Rodrigues Campos
EPI_ISL_861658	Hospital Municipal Antônio Giglio	Instituto Adolfo Lutz, Interdiciplinary Procedures Center, Strategic Laboratory	Claudia Regina Gonçalves; Claudio Tavares Sacchi; Erica Valessa Ramos Gomes; Karoline Rodrigues Campos
EPI_ISL_471549	Hospital Municipal Carmen Prudente	Instituto Adolfo Lutz, Interdiciplinary Procedures Center, Strategic Laboratory	Claudia Regina Gonçalves; Claudio Tavares Sacchi; Erica Valessa Ramos Gomes
EPI_ISL_1303544	Hospital Municipal Cidade Tiradentes Carmen Prudente	Instituto Adolfo Lutz, Interdiciplinary Procedures Center, Strategic Laboratory	Caio Vinicius Dias Lopes; Claudia Regina Gonçalves; Claudio Tavares Sacchi; Erica Valessa Ramos Gomes; Karoline Rodrigues Campos
EPI_ISL_515562	Hospital Municipal Doutor Alexandre Zaio	Instituto Adolfo Lutz, Interdiciplinary Procedures Center, Strategic Laboratory	Claudia Regina Gonçalves; Claudio Tavares Sacchi; Erica Valessa Ramos Gomes
EPI_ISL_693203	Hospital Municipal Doutor Arthur Ribeiro de Saboya	Instituto Adolfo Lutz, Interdiciplinary Procedures Center, Strategic Laboratory	Claudia Regina Gonçalves; Claudio Tavares Sacchi; Erica Valessa Ramos Gomes; Karoline Rodrigues Campos
EPI_ISL_468311, EPI_ISL_468312	Hospital Municipal Dr Ignacio Proenca de Gouvea	Instituto Adolfo Lutz, Interdiciplinary Procedures Center, Strategic Laboratory	Claudia Regina Gonçalves; Claudio Tavares Sacchi; Erica Valessa Ramos Gomes
EPI_ISL_523985	Hospital Municipal Dr. Benedicto Montenegro	Instituto Adolfo Lutz, Interdiciplinary Procedures Center, Strategic Laboratory	Claudia Regina Gonçalves; Claudio Tavares Sacchi; Erica Valessa Ramos Gomes
EPI_ISL_882672	Hospital Municipal Dr. Guido Guida	Instituto Adolfo Lutz, Interdiciplinary Procedures Center, Strategic Laboratory	Claudia Regina Gonçalves; Claudio Tavares Sacchi; Erica Valessa Ramos Gomes; Karoline Rodrigues Campos
EPI_ISL_515553,	Hospital Municipal	Instituto Adolfo Lutz,	Claudia Regina Gonçalves; Claudio Tavares Sacchi; Erica Valessa Ramos Gomes; Karoline Rodrigues Campos

EPI_ISL_574577, EPI_ISL_574579	Dr. Ignacio Proença de Gouvea	Interdiciplinary Procedures Center, Strategic Laboratory	
EPI_ISL_515548, EPI_ISL_515563, EPI_ISL_574589	Hospital Municipal Dr. Jose Soares Hungria	Instituto Adolfo Lutz, Interdiciplinary Procedures Center, Strategic Laboratory	Claudia Regina Gonçalves; Claudio Tavares Sacchi; Erica Valessa Ramos Gomes; Karoline Rodrigues Campos
EPI_ISL_861640, EPI_ISL_861641	Hospital Municipal Dr. Moyses Deutsch	Instituto Adolfo Lutz, Interdiciplinary Procedures Center, Strategic Laboratory	Claudia Regina Gonçalves; Claudio Tavares Sacchi; Erica Valessa Ramos Gomes; Karoline Rodrigues Campos
EPI_ISL_583498	Hospital Municipal Dr. Waldemar Tebaldi	Instituto Adolfo Lutz, Interdiciplinary Procedures Center, Strategic Laboratory	Claudia Regina Gonçalves; Claudio Tavares Sacchi; Erica Valessa Ramos Gomes; Karoline Rodrigues Campos
EPI_ISL_1469754, EPI_ISL_1469778	Hospital Municipal Getúlio Vargas	Epiclin	Ana Paula Muterle; Carolina Comerlato; Eliana Márcia Da Ros Wendland; Fernando Hayashi Sant'Anna; Janira Prichula; Juliana Comerlato
EPI_ISL_693206	Hospital Municipal Mario Gatti	Instituto Adolfo Lutz, Interdiciplinary Procedures Center, Strategic Laboratory	Claudia Regina Gonçalves; Claudio Tavares Sacchi; Erica Valessa Ramos Gomes; Karoline Rodrigues Campos
EPI_ISL_527870, EPI_ISL_574578	Hospital Municipal Mário Gatti	Instituto Adolfo Lutz, Interdiciplinary Procedures Center, Strategic Laboratory	Claudia Regina Gonçalves; Claudio Tavares Sacchi; Erica Valessa Ramos Gomes; Karoline Rodrigues Campos
EPI_ISL_861671, EPI_ISL_882660	Hospital Municipal Prefeito Waldemar Costa Filho	Instituto Adolfo Lutz, Interdiciplinary Procedures Center, Strategic Laboratory	Claudia Regina Gonçalves; Claudio Tavares Sacchi; Erica Valessa Ramos Gomes; Karoline Rodrigues Campos
EPI_ISL_527856	Hospital Municipal Prof. Waldomiro de Paula	Instituto Adolfo Lutz, Interdiciplinary Procedures Center, Strategic Laboratory	Claudia Regina Gonçalves; Claudio Tavares Sacchi; Erica Valessa Ramos Gomes
EPI_ISL_1520110	Hospital Municipal Reynaldo Guerra Cajati	Instituto Adolfo Lutz, Interdiciplinary Procedures Center, Strategic Laboratory	Caio Vinicius Dias Lopes; Claudia Regina Gonçalves; Claudio Tavares Sacchi; Erica Valessa Ramos Gomes; Karoline Rodrigues Campos
EPI_ISL_603028	Hospital Municipal Santa Ana	Instituto Adolfo Lutz, Interdiciplinary Procedures Center, Strategic Laboratory	Claudia Regina Gonçalves; Claudio Tavares Sacchi; Erica Valessa Ramos Gomes; Karoline Rodrigues Campos
EPI_ISL_524468, EPI_ISL_527859	Hospital Municipal Vereador Jose Storopolli	Instituto Adolfo Lutz, Interdiciplinary Procedures Center, Strategic Laboratory	Claudia Regina Gonçalves; Claudio Tavares Sacchi; Erica Valessa Ramos Gomes
EPI_ISL_471647	Hospital Municipal de Barueri Dr. Francisco Moran	Instituto Adolfo Lutz, Interdiciplinary Procedures Center, Strategic Laboratory	Claudia Regina Gonçalves; Claudio Tavares Sacchi; Erica Valessa Ramos Gomes
EPI_ISL_527860	Hospital Municipal de Parelheiros Josanias Castanha Braga	Instituto Adolfo Lutz, Interdiciplinary Procedures Center, Strategic Laboratory	Claudia Regina Gonçalves; Claudio Tavares Sacchi; Erica Valessa Ramos Gomes
EPI_ISL_527862	Hospital Municipal de Urgência	Instituto Adolfo Lutz, Interdiciplinary Procedures Center, Strategic Laboratory	Claudia Regina Gonçalves; Claudio Tavares Sacchi; Erica Valessa Ramos Gomes
EPI_ISL_468308, EPI_ISL_468315, EPI_ISL_515520, EPI_ISL_515546, EPI_ISL_515552, EPI_ISL_523955, EPI_ISL_523974, EPI_ISL_527863			
see above	Hospital Municipal do Tatuape Carmino Caricchio	Instituto Adolfo Lutz, Interdiciplinary Procedures Center, Strategic Laboratory	Claudia Regina Gonçalves; Claudio Tavares Sacchi; Erica Valessa Ramos Gomes
EPI_ISL_735399	Hospital Municiplal Dr Ignacio de gouvea	Instituto Adolfo Lutz, Interdiciplinary Procedures Center, Strategic Laboratory	Claudia Regina Gonçalves; Claudio Tavares Sacchi; Erica Valessa Ramos Gomes; Karoline Rodrigues Campos
EPI_ISL_735428, EPI_ISL_735429, EPI_ISL_735431, EPI_ISL_906066	Hospital Nipo Brasileiro	Instituto Adolfo Lutz, Interdiciplinary Procedures Center, Strategic Laboratory	Claudia Regina Gonçalves; Claudio Tavares Sacchi; Erica Valessa Ramos Gomes; Karoline Rodrigues Campos
EPI_ISL_527857	Hospital Regional Vale do Ribeira	Instituto Adolfo Lutz, Interdiciplinary Procedures Center, Strategic Laboratory	Claudia Regina Gonçalves; Claudio Tavares Sacchi; Erica Valessa Ramos Gomes
EPI_ISL_524463	Hospital Regional de Cotia	Instituto Adolfo Lutz, Interdiciplinary Procedures Center, Strategic Laboratory	Claudia Regina Gonçalves; Claudio Tavares Sacchi; Erica Valessa Ramos Gomes
EPI_ISL_735418	Hospital Regional do Vale do Paraiba	Instituto Adolfo Lutz, Interdiciplinary Procedures Center, Strategic Laboratory	Claudia Regina Gonçalves; Claudio Tavares Sacchi; Erica Valessa Ramos Gomes; Karoline Rodrigues Campos
EPI_ISL_861649	Hospital Renascença Campinas	Instituto Adolfo Lutz, Interdiciplinary Procedures Center, Strategic Laboratory	Claudia Regina Gonçalves; Claudio Tavares Sacchi; Erica Valessa Ramos Gomes; Karoline Rodrigues Campos
EPI_ISL_833165	Hospital Samaritano	Instituto Adolfo Lutz, Interdiciplinary Procedures Center, Strategic Laboratory	Claudia Regina Gonçalves; Claudio Tavares Sacchi; Erica Valessa Ramos Gomes; Karoline Rodrigues Campos
EPI_ISL_471552	Hospital Sancta Maggiore	Instituto Adolfo Lutz, Interdiciplinary Procedures Center, Strategic Laboratory	Claudia Regina Gonçalves; Claudio Tavares Sacchi; Erica Valessa Ramos Gomes
EPI_ISL_603036	Hospital Santa Ana	Instituto Adolfo Lutz, Interdiciplinary Procedures Center, Strategic Laboratory	Claudia Regina Gonçalves; Claudio Tavares Sacchi; Erica Valessa Ramos Gomes; Karoline Rodrigues Campos
EPI_ISL_693196	Hospital Santa	Instituto Adolfo Lutz,	Claudia Regina Gonçalves; Claudio Tavares Sacchi; Erica Valessa Ramos Gomes; Karoline Rodrigues Campos

	Clara	Interdiciplinary Procedures Center, Strategic Laboratory	
EPI_ISL_693233	Hospital Santa Cruz	Instituto Adolfo Lutz, Interdiciplinary Procedures Center, Strategic Laboratory	Claudia Regina Gonçalves; Claudio Tavares Sacchi; Erica Valessa Ramos Gomes; Karoline Rodrigues Campos
EPI_ISL_693236, EPI_ISL_861646, EPI_ISL_861647, EPI_ISL_861650, EPI_ISL_861654, EPI_ISL_861655	Hospital Santa Marcelina Sao Paulo	Instituto Adolfo Lutz, Interdiciplinary Procedures Center, Strategic Laboratory	Claudia Regina Gonçalves; Claudio Tavares Sacchi; Erica Valessa Ramos Gomes; Karoline Rodrigues Campos
EPI_ISL_861644	Hospital Santa Virginia	Instituto Adolfo Lutz, Interdiciplinary Procedures Center, Strategic Laboratory	Claudia Regina Gonçalves; Claudio Tavares Sacchi; Erica Valessa Ramos Gomes; Karoline Rodrigues Campos
EPI_ISL_1493590	Hospital Sao Marcos da Samamorro Agudo	Instituto Adolfo Lutz, Interdiciplinary Procedures Center, Strategic Laboratory	Caio Vinicius Dias Lopes; Claudia Regina Gonçalves; Claudio Tavares Sacchi; Erica Valessa Ramos Gomes; Karoline Rodrigues Campos
EPI_ISL_471545, EPI_ISL_515545, EPI_ISL_515559, EPI_ISL_515560, EPI_ISL_523969, EPI_ISL_523981, EPI_ISL_523988, EPI_ISL_693201, EPI_ISL_861639			
see above	Hospital Sao Paulo de Ensino da Unifesp	Instituto Adolfo Lutz, Interdiciplinary Procedures Center, Strategic Laboratory	Claudia Regina Gonçalves; Claudio Tavares Sacchi; Erica Valessa Ramos Gomes; Karoline Rodrigues Campos
EPI_ISL_1469593	Hospital Sapiranga	Epiclin	Ana Paula Muterle; Carolina Comerlato; Eliana Márcia Da Ros Wendland; Fernando Hayashi Sant'Anna; Janira Prichula; Juliana Comerlato
EPI_ISL_2352015, EPI_ISL_2516467, EPI_ISL_2516544	Hospital Universitari Bellvitge	Microbiology Department	Aida Gonzalez-Diaz; Carmen Ardanuy; Jordi Camara; Jordi Niubó; Laura Calatayud; M Angeles Domínguez; Miguel Fernandez-Huerta; Sara Marti
EPI_ISL_468318, EPI_ISL_468321	Hospital Universitario da USP	Instituto Adolfo Lutz, Interdiciplinary Procedures Center, Strategic Laboratory	Claudia Regina Gonçalves; Claudio Tavares Sacchi; Erica Valessa Ramos Gomes
EPI_ISL_471539	Hospital Universitario da USP Sao Paulo	Instituto Adolfo Lutz, Interdiciplinary Procedures Center, Strategic Laboratory	Claudia Regina Gonçalves; Claudio Tavares Sacchi; Erica Valessa Ramos Gomes
EPI_ISL_534314	Hospital Universitario da USP de SP	Instituto Adolfo Lutz, Interdiciplinary Procedures Center, Strategic Laboratory	Claudia Regina Gonçalves; Claudio Tavares Sacchi; Erica Valessa Ramos Gomes
EPI_ISL_1469570	Hospital Universitário	Epiclin	Ana Paula Muterle; Carolina Comerlato; Eliana Márcia Da Ros Wendland; Fernando Hayashi Sant'Anna; Janira Prichula; Juliana Comerlato
EPI_ISL_1469774	Hospital Universitário de Canoas	Epiclin	Ana Paula Muterle; Carolina Comerlato; Eliana Márcia Da Ros Wendland; Fernando Hayashi Sant'Anna; Janira Prichula; Juliana Comerlato
EPI_ISL_3031311	Hospital da Baleia	Instituto René Rachou / Fiocruz Minas	Alana Oliveira; Anna Salim; Camila Corsini; Daniel Miranda; Gabriel Fernandes; Mozar de Castro; Nathalie Almeida; Pedro Alves; Priscilla Filgueiras; Rafaella Fortini; Raphael Silva; Raquel Vilela; Rubens do Monte Neto; Sarah Gomes; Thaís Silva; Wander Jeremias
EPI_ISL_476373, EPI_ISL_476445, EPI_ISL_476446, EPI_ISL_476469	Hospital da Clínicas da Faculdade de Medicina da Universidade de São Paulo	Instituto de Medicina Tropical da Univesidade de São Paulo	Camila Alves Maia da Silva; Carolina S. Lazar; Cecilia Salete Alencar; Darlan da Silva Candido; Erika Regina Manuli; Ester Sabino; Flavia Cristina da Silva Sales; Giula Magalhaes Ferreira; Jaqueline Goes de Jesus; Julien Theze; Mariana Severo Ramundo; Nuno Faria; Samples: Ingra Morales Claro; Sequencing: Ingra Morales Claro; Silvia F. Costa; Thais de Moura Coletti
EPI_ISL_3031319, EPI_ISL_3031335	Hospital das Clínicas da UFMG	Instituto René Rachou / Fiocruz Minas	Anna Salim; Cristina Fonseca; Gabriel Fernandes; Matheus Westin; Núbia Fernandes; Pedro Alves; Rosiane Pereira; Rubens do Monte Neto; Sandra Gava; Thaís Santos; Thaís Silva; Wilma Patrícia Bernardes
EPI_ISL_693205	Hospital de Campanha Covid-19 Assis	Instituto Adolfo Lutz, Interdiciplinary Procedures Center, Strategic Laboratory	Claudia Regina Gonçalves; Claudio Tavares Sacchi; Erica Valessa Ramos Gomes; Karoline Rodrigues Campos
EPI_ISL_735396	Hospital de Camplanha COVID 19 SER	Instituto Adolfo Lutz, Interdiciplinary Procedures Center, Strategic Laboratory	Claudia Regina Gonçalves; Claudio Tavares Sacchi; Erica Valessa Ramos Gomes; Karoline Rodrigues Campos
EPI_ISL_882661, EPI_ISL_882662	Hospital de Santa Barbara de Goias	Instituto Adolfo Lutz, Interdiciplinary Procedures Center, Strategic Laboratory	Claudia Regina Gonçalves; Claudio Tavares Sacchi; Erica Valessa Ramos Gomes; Karoline Rodrigues Campos
EPI_ISL_534319, EPI_ISL_534320	Hospital do Serv Pub ESTAFCO Morato de Oliveira	Instituto Adolfo Lutz, Interdiciplinary Procedures Center, Strategic Laboratory	Claudia Regina Gonçalves; Claudio Tavares Sacchi; Erica Valessa Ramos Gomes
EPI_ISL_693199	Hospital do Servidor Publico Estadual Francisco Morato de Oliveira	Instituto Adolfo Lutz, Interdiciplinary Procedures Center, Strategic Laboratory	Claudia Regina Gonçalves; Claudio Tavares Sacchi; Erica Valessa Ramos Gomes; Karoline Rodrigues Campos
EPI_ISL_471548, EPI_ISL_515565, EPI_ISL_523965, EPI_ISL_523978, EPI_ISL_523982	Hospital do Servidor Público Estadual Francisco Morato de Oliveira	Instituto Adolfo Lutz, Interdiciplinary Procedures Center, Strategic Laboratory	Claudia Regina Gonçalves; Claudio Tavares Sacchi; Erica Valessa Ramos Gomes
EPI_ISL_527861	Hospital e Maternidade Celso Pierro	Instituto Adolfo Lutz, Interdiciplinary Procedures Center, Strategic Laboratory	01246-1301; 355 - Brazil; Av. Dr. Arnaldo; Cerqueira Cesar; São Paulo - SP
EPI_ISL_693200	Hospital e Maternidade Mairipora	Instituto Adolfo Lutz, Interdiciplinary Procedures Center, Strategic Laboratory	Claudia Regina Gonçalves; Claudio Tavares Sacchi; Erica Valessa Ramos Gomes; Karoline Rodrigues Campos
EPI_ISL_693241	Hospital e Maternidade Sao Lucas	Instituto Adolfo Lutz, Interdiciplinary Procedures Center, Strategic Laboratory	Claudia Regina Gonçalves; Claudio Tavares Sacchi; Erica Valessa Ramos Gomes; Karoline Rodrigues Campos
EPI_ISL_861657	Hospital e Maternidade Sino Brasileiro	Instituto Adolfo Lutz, Interdiciplinary Procedures Center, Strategic Laboratory	Claudia Regina Gonçalves; Claudio Tavares Sacchi; Erica Valessa Ramos Gomes; Karoline Rodrigues Campos

EPI_ISL_527865	Hospital e Maternidade São Cristóvão	Instituto Adolfo Lutz, Interdiciplinary Procedures Center, Strategic Laboratory	Claudia Regina Gonçalves; Claudio Tavares Sacchi; Erica Valessa Ramos Gomes
EPI_ISL_527868	Hospital e Maternidade do Braz	Instituto Adolfo Lutz, Interdiciplinary Procedures Center, Strategic Laboratory	Claudia Regina Gonçalves; Claudio Tavares Sacchi; Erica Valessa Ramos Gomes
EPI_ISL_861645	Hospital e Pronto Socorro Comunitario Vila Iolanda	Instituto Adolfo Lutz, Interdiciplinary Procedures Center, Strategic Laboratory	Claudia Regina Gonçalves; Claudio Tavares Sacchi; Erica Valessa Ramos Gomes; Karoline Rodrigues Campos
EPI_ISL_693195, EPI_ISL_693230, EPI_ISL_693232, EPI_ISL_735412, EPI_ISL_861648, EPI_ISL_861680	Hospital e Pronto Socorro Portinari	Instituto Adolfo Lutz, Interdiciplinary Procedures Center, Strategic Laboratory	Claudia Regina Gonçalves; Claudio Tavares Sacchi; Erica Valessa Ramos Gomes; Karoline Rodrigues Campos
EPI_ISL_790234, EPI_ISL_1074736, EPI_ISL_1075791, EPI_ISL_1076528, EPI_ISL_1079280, EPI_ISL_1304728, EPI_ISL_2191321, EPI_ISL_2223909			
see above	Houston Methodist Hospital	Houston Methodist Hospital	David W. Bernard; Heather Hendrickson; Ilya J. Finkelstein; James J. Davis; Jessica Cambric; Jimmy Gollihar; Kristina Reppond; Layne Pruitt; Madison N. Shyer; Marcus Nguyen; Matthew Ojeda Saavedra; Maulik Shukla; Paul A. Christensen; Prasanti Yerramilli; Randall J. Olsen; Robert Olson; Ryan Gadd; S. Wesley Long; Sishir Subedi; and James M. Musser
EPI_ISL_984263, EPI_ISL_1078981, EPI_ISL_1078983, EPI_ISL_1078984, EPI_ISL_1078991, EPI_ISL_1078996, EPI_ISL_1079003, EPI_ISL_1079006, EPI_ISL_1079158, EPI_ISL_1079163, EPI_ISL_1079166, EPI_ISL_1086051, EPI_ISL_1086056, EPI_ISL_1121326			
see above	IAL Regional de Bauru	Instituto Adolfo Lutz, Interdiciplinary Procedures Center, Strategic Laboratory	Caio Vinicius Dias Lopes; Claudia Regina Gonçalves; Claudio Tavares Sacchi; Erica Valessa Ramos Gomes; Karoline Rodrigues Campos
EPI_ISL_984248, EPI_ISL_984254, EPI_ISL_1171646, EPI_ISL_1171647, EPI_ISL_1196297, EPI_ISL_1196298	IAL Regional de Marilia	Instituto Adolfo Lutz, Interdiciplinary Procedures Center, Strategic Laboratory	Caio Vinicius Dias Lopes; Claudia Regina Gonçalves; Claudio Tavares Sacchi; Erica Valessa Ramos Gomes; Karoline Rodrigues Campos
EPI_ISL_1171664, EPI_ISL_1171668, EPI_ISL_1171669, EPI_ISL_1171671	IAL Regional de Presidente Prudente	Instituto Adolfo Lutz, Interdiciplinary Procedures Center, Strategic Laboratory	Caio Vinicius Dias Lopes; Claudia Regina Gonçalves; Claudio Tavares Sacchi; Erica Valessa Ramos Gomes; Karoline Rodrigues Campos
EPI_ISL_1358296, EPI_ISL_1358297, EPI_ISL_1381044, EPI_ISL_1381046, EPI_ISL_1381049, EPI_ISL_1381064	IAL Regional de Santo Andre	Instituto Adolfo Lutz, Interdiciplinary Procedures Center, Strategic Laboratory	Caio Vinicius Dias Lopes; Claudia Regina Gonçalves; Claudio Tavares Sacchi; Erica Valessa Ramos Gomes; Karoline Rodrigues Campos
EPI_ISL_1123374, EPI_ISL_1171623, EPI_ISL_1171625, EPI_ISL_1171627, EPI_ISL_1171631, EPI_ISL_1171633, EPI_ISL_1171635, EPI_ISL_1171636			
see above	IAL Regional de Santos	Instituto Adolfo Lutz, Interdiciplinary Procedures Center, Strategic Laboratory	Caio Vinicius Dias Lopes; Claudia Regina Gonçalves; Claudio Tavares Sacchi; Erica Valessa Ramos Gomes; Karoline Rodrigues Campos
EPI_ISL_1293056, EPI_ISL_1293057, EPI_ISL_1293064, EPI_ISL_1293072, EPI_ISL_1293081	IAL Regional de Sorocaba	Instituto Adolfo Lutz, Interdiciplinary Procedures Center, Strategic Laboratory	Caio Vinicius Dias Lopes; Claudia Regina Gonçalves; Claudio Tavares Sacchi; Erica Valessa Ramos Gomes; Karoline Rodrigues Campos
EPI_ISL_1303527, EPI_ISL_1303528	IAL Regional de São Jose do Rio Preto	Instituto Adolfo Lutz, Interdiciplinary Procedures Center, Strategic Laboratory	Caio Vinicius Dias Lopes; Claudia Regina Gonçalves; Claudio Tavares Sacchi; Erica Valessa Ramos Gomes; Karoline Rodrigues Campos
EPI_ISL_2234880, EPI_ISL_2234881, EPI_ISL_2234884, EPI_ISL_2234887, EPI_ISL_2234888, EPI_ISL_2234892, EPI_ISL_2234894, EPI_ISL_2234896, EPI_ISL_2234898, EPI_ISL_2444785, EPI_ISL_2444788, EPI_ISL_2444796, EPI_ISL_2444839, EPI_ISL_4071901			
see above	IICS-UNA	IICS-UNA	Adriana Valenzuela; Alejandra Rojas; Chyntia Diaz; Eva Nara; Fatima Cardozo; Florencia del Puerto; Joel Ortiz; Jonas Fernandez; Laura Franco; Laura Mendoza; Leticia Rojas; Magaly Martinez; Maria Eugenia Galeano.
EPI_ISL_1213317	IMT-UFRN/RN	Bioinformatics Laboratory / LNCC	Alessandra P Lamarca; Alexandra L Gerber; Ana Paula Melo Mariano; Ana Paula de C Guimarães; Ana Tereza R Vasconcelos; Angela Maria Guimarães Santos; Bianca Mendes Maciel; Danielle Angst Secco; Eduardo Sérgio Soares Sousa; Eloiza Helena Campana; Francisco Paulo Freire Neto; George Rego Albuquerque; Kátia Castanho Scortecci; Lucymara Fassarella Agnez Lima; Luiz G P de Almeida; Luís Cristóvão Porto; Otavio J. Brustolini; Paulo Ricardo Nascimento; Ronaldo da Silva Francisco Jr; Sandra Rocha Gadelha; Selma Maria Bezerra Jeronimo; Vinícius Pietta Perez
EPI_ISL_732116	INSA	Instituto Nacional de Saude (INSA)	Borges et al
EPI_ISL_2758668, EPI_ISL_2758669, EPI_ISL_2758671, EPI_ISL_2758672, EPI_ISL_2758806	IPEC Guarapuava	IPEC Guarapuava	NAPI-Genômica (Novos Arranjo de Pesquisa e Inovação em Genômica): Ademar Dantas da Cunha Júnior Adriano Ferrasa Adriano Mondini Aldo Przybysz Alessandra Lourenço Cecchini Armani Alex Sandro Jorge Alexandra Ivo de Medeiros Alexandre Maller Aline Cristina Batista Rodrigues Johann Ana Lucia Ferreira Ana Marisa Fusco Almeida Anderson Joel Martino Andrade André Luís Laforga Vanzela Andrea Duarte Doetzer Andrea Name Colado Simao Andressa Pereira de Souza Anelisa Ramão Angelica Beate Winter Boldt Anna Hermínia Castro Gomes de Amorim Anna Silvia Penteado Setti da Rocha Antonio Camilo da Silva Filho Antonio Stabelini Neto Arthur Hirata Bertachi Barbara Mendes Paz Chao Betty Cristiane Kuhn Bruno Ambrozio Galindo Bruno Ribeiro Cruz Camilla Reginatto De Pierri Carla Fredrichsen Moya Araujo Carla Fredrichsen Moya Araujo Carlos Alberto Oliveira de Biagi Junior Carlos Augusto Nassar Carlos Eduardo Buss Carlos Gilberto Carlotti Junior Carlos Henrique Schneider Carolina Panis Carolina Weigert Galvão Caroline de Jesus Coelho Donha Caroline Guisantes de Salvo Toni Caryna Eurich Mazur Catiuscie Cabreira da Silva Tortorella Celso F. D. Doliveira Cesar Luiz Boguszewski Christiane Pienna Soares Chung Man Chin Claudia Moro Cleverson Busso Cristiane Cominetti Daiane Priscila Simão-Silva Dalila Luciola Zanette Daniel de Paula Daniel de Paula Daniel Rech Daniela Fiori Gradia Daniela Pretti da Cunha Tirapelli Daniela Viganó Zanoti Jeronymo Daniele Ukan Danielle Malheiros Ferreira Danielle Venturini Deborah Catharine de Assis Leite Deivid Calebe de Souza Dennis Armando Bertolini Edenir Inez Pamero Edna Maria Vissoci Reiche Edson Roberto Arpini Miguel Eduardo José de Almeida Araújo Eliana Carolina Vespero Eliandro Reis Tavares Elza Kimura Grimshaw Emanuel Maltempi de Souza Emanuele Cristina Gustani Buss Émerson Carraro Emiliana Cristina Melo ENILze Maria de Souza Fonseca Ribeiro Enilze Maria de Souza Fonseca Ribeiro Erika Izumi Erika Seki Kioshima Cotica Evani Marques Pereira Fabio Negretti Fábio Rodrigues Ferreira Seiva Felipe Dunin dos Santos Felipe Tuon Fernanda Andreia Rosa Fernanda Cestaro Prado Cortez Fernanda Ivanski Fernanda Maris Peria Flavia Regina Oliveira de Barros Franciele Aní Caovilla Follador Franciele Mara Lucca Zanardo Bohm Francinete Ramos Campos Fulviana Silva Nishiyama GABRIEL RIBEIRO CORDEIRO Gabriela Datsch Bennemann Gisele Santos de Oliveira Glaucio Valdameri Glauco Akelinghton Freire Vitiello Glauco Vieira Miranda Glaura Scantamburlo ALves Fernandes Guilherme Ferreira Silveira Gustavo Bianchini Porfírio Gustavo Lenci Marques Hélito Volpato Hildebrando Masshiro Nagai Huei Diana Lee Ilce Mara de Syllos Cólus Iris Rabinovich Israel Gomy Jackson Kawakami Jacques Duílio Brancher Jaime Luís Lopes Rocha Jaqueline Carvalho de Oliveira Jean Henrique da Silva Rodrigues Jean Leandro dos Santos Jeane Eliete Laguila Visentainer João Paulo Bianchi Ximenez Joaquim Manoel da Silva Jociani Ascari Joel Donazzolo Jorge Luis Maria Ruiz Jose Knopfholz José Luis da Conceição Silva José Sebastião dos Santos Joseane Carla Schabarum Juliana Cheleski Wiggers Juliana Mara Serpeloni Juliana Morini Küpper Cardoso Perseguini Karen Brajão de Oliveira Karin Braun Prado Karine Aparecida de Lima Katiany Rizzieri Caleffi Ferracioli Katiuscia de Oliveira Francisco Gabriel Kelvinson Fernandes Viana Larissa Beatriz Cossalter Larissa Danielle Bahls Pinto Laurival Antonio Vilas Boas Léia Carolina Lucio Líbero Mezzadri Neto Ligia Carla Faccin Galhardi Lirane Elize Defante Ferreto Luciana Furlaneto Maia Luciana Oliveira de Fariña Luciana Reis Azevedo Alanis Luciane Regina Cavalli Lucy Megumi Yamauchi Lioni Luis Paulo Gomes Mascarenhas Luis Paulo Gomes Mascarenhas Luís Paulo Mascarenhas Lupe Furtado Alle Lyvia Regina Biagi Silva Bertachi Mara Antonia Ramos Costa Mara L. Cordeiro Marcela Maria Birolim Marcelo Ricardo Vicari Marcia Edilaine Lopes Consolaro Marcia Holsbach Beltrame Marcia Regina Eches Perugini Marcos Abdo Arbex Marcos Pileggi MARCOS TADEU GRZELCZAK Marcus Peikriszwili Tartaruga Maria Angelica Ehara Watanabe Maria Antonia Ramos Costa Maria Claudia Gross Maria José Soares Mendes Giannini Maria Leandra Terencio Maria Lúcia Bonfleur Maria Luiza Guimarães de Oliveira Maria Luiza Petzl-Erler Mariana Abe Vicente Cavagnari Marina Kimiko Kadowaki Marise Fonseca dos Santos Marla Karine Amarante Maurício Turkiewicz Mauro Antonio Alves Castro Michel Rodrigo Zambrano Passarini Michele Potrich Michelle Orane Schemberger Milena Massumi Kozonoe Mônica Degraf Cavallin Monica Tereza Suldofski Mucio Luiz de Assis Cirino Nadia Graciele Krohn Najeh Maissar Khalil Nédia de Castilhos Ghisi Neide Tomimura Costa Neiva Leite Neyva Maria Lopes Romeiro Patrícia Amâncio da Rosa Patricia Dayane Carvalho Schaker Patricia Oehlmeyer Nassar Patrícia Savio de Araújo-Souza Patricia Silva Lucio Paulo Henrique Couto Souza Paulo Roberto Donadio Percy Nohama Quirino Alves de Lima Neto Rafael Deminice Rafael dos Santos Bezerra Raquel Alves dos Santos Renan Manozzo Galante Renata Ernlund Freitas de Macedo Rita de Cássia Garcia Simão Roberta Losi Guembarovski Roberto H. Herai Roberto Rosati Rodrigo Ferreira Rodrigo Rodrigues Matiello Rogério Neri Shinsato Rogério Pincela Mateus Rosane Aparecida Ribeiro Rosilene Fressatti Cardoso Rosilene Fressatti Cardoso Sandra Mara Guse Scós Venske Selene Elifio Esposito Sérgio Ossamu Ioshii Silvana Giuliatti Silvia Mara de Souza Halick Silvio Henrique Maia de Almeida Simone Neumann Wendt Spencer Luiz Marques Payão Stefan Wolanski Negrão Stephane Janaina de Moura Escobar Sueli Fumie Yamada Ogatta SUELI PERCIO QUINAIA Taciane Finatto Tatiana Mayumi Veiga Iriyoda Tayza Katelline Danilau Ostroski Tony Alexander Hild Valeria Valente Vanessa Nascimento Kozak Vanessa Santos Sotomaior Victor Breno Pedrosa Victoria Zeghbi Cochenski Borba Vivian Rotuno Moure Valdameri Wander Rogerio Pavanelli Weber Cláudio Francisco Nunes da Silva Willian Augusto de Melo Yohandra Reyes Torres
EPI_ISL_2464654, EPI_ISL_2465722	IdISSC/Hospital Clínico San Carlos de Madrid	SeqCOVID-SPAIN consortium/IBV(CSIC)	Alberto Delgado-Iribarren; Esther Culebras López; Jorge Matias-Guiu; Luis Ortega Medina; Silvia Sánchez Ramón; Ulises Gómez-Pinedo and SeqCOVID-SPAIN consortium; Vicente Estrada Pérez
EPI_ISL_1827855	Institute for Health Research, Epidemiological Surveillance and Training (IRESSEF)	Abbott Laboratories	Adbou Padane; Ambroise Ahouidi; Aminata Dia; Aminata Mboup; Ana Olivo; Anna julienne selbe Ndiaye; Barbara Harris; Cyrille Diedhiou; Gavin Cloherty; Mary Rodgers; Moustapha Mbow; Nafissatou Leye; Ndeye Diabou Diagne; Papa Alassane Diaw; Souleymane Mboup; Todd Meyer
EPI_ISL_672705, EPI_ISL_672711, EPI_ISL_672719, EPI_ISL_672720, EPI_ISL_672748	Institute of Tropical Medicine at the University of São Paulo (IMT-USP)	Laboratório de Parasitologia Médica - Instituto de Medicina Tropical - Universidade de São Paulo	Brazil-UK Centre for Arbovirus Discovery Diagnosis Genomics and Epidemiology (CADDE) Genomic Network - Instituto de Medicina Tropical
EPI_ISL_2245187	Instituto Adolfo Lutz	Coordenação Geral de Laboratórios de Saúde Pública (CGLAB/DAEVS/SVS/MS)	Vagner Fonseca; et al.
EPI_ISL_755640, EPI_ISL_755643, EPI_ISL_755654, EPI_ISL_776750, EPI_ISL_776752, EPI_ISL_776753, EPI_ISL_776755, EPI_ISL_776756, EPI_ISL_776761, EPI_ISL_792101, EPI_ISL_792104, EPI_ISL_792106, EPI_ISL_792107, EPI_ISL_792108, EPI_ISL_792109, EPI_ISL_792110, EPI_ISL_792111, EPI_ISL_792112, EPI_ISL_792113, EPI_ISL_792114, EPI_ISL_833152, EPI_ISL_833153, EPI_ISL_833154, EPI_ISL_861625, EPI_ISL_861626, EPI_ISL_861627, EPI_ISL_861629, EPI_ISL_861630, EPI_ISL_861631, EPI_ISL_861632, EPI_ISL_861633, EPI_ISL_861634, EPI_ISL_861643, EPI_ISL_861663			

see above	Instituto Adolfo Lutz - Central	Instituto Adolfo Lutz, Interdiciplinary Procedures Center, Strategic Laboratory	Claudia Regina Gonçalves; Claudio Tavares Sacchi; Erica Valessa Ramos Gomes; Karoline Rodrigues Campos
EPI_ISL_776768, EPI_ISL_1039702	Instituto Adolfo Lutz - Regional de Aracatuba	Instituto Adolfo Lutz, Interdiciplinary Procedures Center, Strategic Laboratory	Claudia Regina Gonçalves; Claudio Tavares Sacchi; Erica Valessa Ramos Gomes; Karoline Rodrigues Campos
EPI_ISL_2919249	Instituto Adolfo Lutz - Regional de Bauru	Instituto Adolfo Lutz, Interdiciplinary Procedures Center, Strategic Laboratory	Caio Vinicius Dias Lopes; Claudia Regina Gonçalves; Claudio Tavares Sacchi; Erica Valessa Ramos Gomes; Karoline Rodrigues Campos
EPI_ISL_755655	Instituto Adolfo Lutz - Regional de Campinas	Instituto Adolfo Lutz, Interdiciplinary Procedures Center, Strategic Laboratory	Claudia Regina Gonçalves; Claudio Tavares Sacchi; Erica Valessa Ramos Gomes; Karoline Rodrigues Campos
EPI_ISL_776757, EPI_ISL_776758, EPI_ISL_776767, EPI_ISL_984243, EPI_ISL_1821229	Instituto Adolfo Lutz - Regional de Marilia	Instituto Adolfo Lutz, Interdiciplinary Procedures Center, Strategic Laboratory	Caio Vinicius Dias Lopes; Claudia Regina Gonçalves; Claudio Tavares Sacchi; Erica Valessa Ramos Gomes; Karoline Rodrigues Campos; Leonardo Jose Tadeu de Araujo
EPI_ISL_977471, EPI_ISL_977475, EPI_ISL_977478, EPI_ISL_977480, EPI_ISL_977481, EPI_ISL_977485, EPI_ISL_977488, EPI_ISL_985170, EPI_ISL_1039696			
see above	Instituto Adolfo Lutz - Regional de Presidente Prudente	Instituto Adolfo Lutz, Interdiciplinary Procedures Center, Strategic Laboratory	Claudia Regina Gonçalves; Claudio Tavares Sacchi; Erica Valessa Ramos Gomes; Karoline Rodrigues Campos
EPI_ISL_735401, EPI_ISL_735402, EPI_ISL_735403, EPI_ISL_735404, EPI_ISL_861667, EPI_ISL_1625985, EPI_ISL_1625996, EPI_ISL_1626008			
see above	Instituto Adolfo Lutz - Regional de Rio Claro	Instituto Adolfo Lutz, Interdiciplinary Procedures Center, Strategic Laboratory	Caio Vinicius Dias Lopes; Claudia Regina Gonçalves; Claudio Tavares Sacchi; Erica Valessa Ramos Gomes; Karoline Rodrigues Campos; Katia Correa de Oliveira Santos; Leonardo Jose Tadeu de Araujo
EPI_ISL_755647, EPI_ISL_776765, EPI_ISL_776769, EPI_ISL_792103, EPI_ISL_833157	Instituto Adolfo Lutz - Regional de Santo Andre	Instituto Adolfo Lutz, Interdiciplinary Procedures Center, Strategic Laboratory	Claudia Regina Gonçalves; Claudio Tavares Sacchi; Erica Valessa Ramos Gomes; Karoline Rodrigues Campos
EPI_ISL_735400	Instituto Adolfo Lutz - Regional de Santos	Instituto Adolfo Lutz, Interdiciplinary Procedures Center, Strategic Laboratory	Claudia Regina Gonçalves; Claudio Tavares Sacchi; Erica Valessa Ramos Gomes; Karoline Rodrigues Campos
EPI_ISL_833156	Instituto Adolfo Lutz - Regional de Sorocaba	Instituto Adolfo Lutz, Interdiciplinary Procedures Center, Strategic Laboratory	Claudia Regina Gonçalves; Claudio Tavares Sacchi; Erica Valessa Ramos Gomes; Karoline Rodrigues Campos
EPI_ISL_792115, EPI_ISL_792116, EPI_ISL_985171, EPI_ISL_985172, EPI_ISL_985173, EPI_ISL_1039699, EPI_ISL_1039703			
see above	Instituto Adolfo Lutz - Regional de Taubate	Instituto Adolfo Lutz, Interdiciplinary Procedures Center, Strategic Laboratory	Claudia Regina Gonçalves; Claudio Tavares Sacchi; Erica Valessa Ramos Gomes; Karoline Rodrigues Campos
EPI_ISL_977473, EPI_ISL_977474, EPI_ISL_977476, EPI_ISL_977477, EPI_ISL_977483, EPI_ISL_977484, EPI_ISL_977487, EPI_ISL_984242, EPI_ISL_984246, EPI_ISL_985175, EPI_ISL_1039697, EPI_ISL_1039705, EPI_ISL_1039707, EPI_ISL_1039708, EPI_ISL_1039709, EPI_ISL_1039710, EPI_ISL_1171620, EPI_ISL_1731593, EPI_ISL_2003160, EPI_ISL_2614514, EPI_ISL_2614516, EPI_ISL_2614517, EPI_ISL_2756442, EPI_ISL_2756452			
see above	Instituto Adolfo Lutz Central	Instituto Adolfo Lutz, Interdiciplinary Procedures Center, Strategic Laboratory	Caio Vinicius Dias Lopes; Claudia Regina Gonçalves; Claudio Tavares Sacchi; Erica Valessa Ramos Gomes; Karoline Rodrigues Campos; Katia Correa de Oliveira Santos; Leonardo Jose Tadeu de Araujo
EPI_ISL_4104676, EPI_ISL_4104692, EPI_ISL_4104697	Instituto Butantan	Instituto Butantan	Antonio Jorge Martins; Claudia Renata dos Santos Barros; David Schlesinger; Debora Botequio Moretti; Dimas Tadeu Covas; Elaine Cristina Marqueze; Elaine Vieira Santos; Evandra Strazza Rodrigues; Heidge Fukumasu; Jayme Augusto de Souza-Neto; José Salvatore Leister Patané; Luiz Alcantara; Luiz Lehmann Coutinho; Maria Carolina Elias; Maurício Lacerda Nogueira; Rafael dos Santos Bezerra; Raul Machado Neto; Rejane Maria Tommasini Grotto; Ricardo Haddad; Sandra Coccuzzo Sampaio Vessoni; Simone Kashima; Svetoslav Nanev Slavov; Vincent Louis Viala
EPI_ISL_2344421, EPI_ISL_2344422, EPI_ISL_2344426, EPI_ISL_2344429, EPI_ISL_2344430, EPI_ISL_2344431, EPI_ISL_2344435, EPI_ISL_2344438, EPI_ISL_2344439, EPI_ISL_2344445, EPI_ISL_2344448, EPI_ISL_2344450, EPI_ISL_2344453, EPI_ISL_2344454, EPI_ISL_2344457, EPI_ISL_2344459, EPI_ISL_2425442			
see above	Instituto Butantan	Instituto de Medicina Tropical de Sao Paulo	Brazil-UK Centre for Arbovirus Discovery Diagnosis Genomics and Epidemiology (CADDE) Genomic Network - Instituto de Medicina Tropical
EPI_ISL_3478866	Instituto Nacional de Enfermedades Virales Humanas Dr. Julio I. Maiztegui	Laboratorio Mixto de Biotecnología Acuática (LMBA) on behalf of 'Proyecto Argentino Interinstitucional de genomica de SARS-CoV-2' (PAIS Consortium)	Ada Nazar; Adriana Giri; Agustina Cerri; Agustina Pacual; Anabel Sinchi; COFECyT SF-11; Camila Gonzalez; Carina Bonacalza; Carina Sen; Carlos Figueroa; Cintia Fabbri; Diego Chouhy; Elisa Bolatti; Elizabeth Tapia; Flavio Spetale; Florencia Mascali; Focem COF 03/11 COVID-19); Gastón Viarengo; Germán R. Perez; Ignacio García Labari; Javier Murillo; Joaquín Ezpeleta; Julia Brignone; Leandro Ciappina; Maria Laura Casela; Mariana Viegas (Financiamiento: argenTAG; Mariel Feroci; María Alejandra Morales; María Laura Martin; María Re; María de los Angeles Conti; Matías Abalo; Pablo Casal; Pilar Bulacio; Proyecto IP COVID-19 N°08; Silvana Spinelli; Silvia Arranz; Sofía Lavista Llanos; Sylvia Garcia; Vanina Villanova; Victoria Luppo; Victoria Posner
EPI_ISL_955192, EPI_ISL_1262637, EPI_ISL_1298462	Instituto Nacional de Medicina Genomica	Instituto Nacional de Medicina Genomica	Alcaraz N; Cedro-Tanda A; Cisneros-Villanueva M; Gonzalez-Barrera D; Herrera-Montalvo LA; Hidalgo-Miranda A; Mendoza-Vargas A; Peñaloza-Figueroa F; Ramirez-Vega O; Rangel-DeLeon D; Reyes-Grajeda JP
EPI_ISL_492036	Instituto de Biologia do Exército	Laboratório Metabolismo Macromolecular FirminoTorres de Castro, Instituto de Biofísica Carlos Chagas Filho, Universidade Federal do Rio de Janeiro	Aline Rosa Vianna de Souza; Bianca Catarina Azevedo Cabral; Caleb GM Santos; Clarissa Damaso; Elizabeth Valentin; Marcio da Costa Cipitelli; Marcos Dornelas-Ribeiro; Nádia Vaez Gonçalves da Cruz; Rodrigo Soares de Moura Neto; Rosane Silva; Tatiana LS Nogueira; Virginia Sara Grancieri do Amaral
EPI_ISL_861242, EPI_ISL_875540, EPI_ISL_875541, EPI_ISL_875542, EPI_ISL_875543, EPI_ISL_875544, EPI_ISL_875545, EPI_ISL_875546, EPI_ISL_875547, EPI_ISL_875548, EPI_ISL_875549, EPI_ISL_875550, EPI_ISL_888671, EPI_ISL_888672, EPI_ISL_1000670			
see above	Instituto de Biotecnologia - UNESP-Botucatu-SP	Instituto de Biotecnologia - UNESP-Botucatu-SP	Camila Dantas Malossi; Fábio Sossai Possebon; João Pessoa Araújo Jr.; Leila Sabrina Ullmann; Paula Rahal; Paulo Inacio da Costa
EPI_ISL_836978	Irmandade da Santa Casa de Misericordia de Lorena	Instituto Adolfo Lutz, Interdiciplinary Procedures Center, Strategic Laboratory	Claudia Regina Gonçalves; Claudio Tavares Sacchi; Erica Valessa Ramos Gomes; Karoline Rodrigues Campos
EPI_ISL_1287622	Istituto Zooprofilattico Sperimentale del Mezzogiorno	TIGEM	Antonio Grimaldi Patrizia Annunziata Francesco Panariello Biancamaria Pierri Claudia Tiberio Valentina Bouche Chiara Colantuono Maria Concetta Cuomo Denise Di Concilio Lucio Di Filippo Anna Manfredi Marcello Salvi Antonio Limone Luigi Atripaldi Pellegrino Cerino Andrea Ballabio Davide Cacchiarelli
EPI_ISL_887535, EPI_ISL_981183	Johns Hopkins Hospital Department of Pathology	Johns Hopkins Hospital Department of Pathology	Adannaya Amadi; C. Paul Morris; Chun Huai Luo; Heba H. Mostafa; Matthew Schwartz; Nicholas Gallagher

EPI_ISL_890322	KU Leuven, Rega Institute, Clinical and Epidemiological Virology	KU Leuven, Rega Institute, Clinical and Epidemiological Virology	Bert Vanmechelen; Joan Marti-Carerras; Piet Maes; Tony Wawina-Bokalanga
EPI_ISL_690818	Kanagawa Prefectural Institute of Public Health	Pathogen Genomics Center, National Institute of Infectious Diseases	Kentaro Itokawa; Makoto Kuroda; Masanori Hashino; Rina Tanaka; Tsuyoshi Sekizuka
EPI_ISL_766625, EPI_ISL_1008415	Klinisk mikrobiologi	The Public Health Agency of Sweden	Anna Risberg; Anna-Malin Linde; Carlo Berg; Department of Microbiology; Karin Tegmark-Wisell; Maria Lind Karlberg; Mattias Haukland; Mia Brytting; Noura Walai; Oskar Karlsson Lindsjo; Petra Edquist; Petra Holmstrom; Reza Advani; Sofia Stamouli; The Public Health Agency of Sweden
EPI_ISL_1795418, EPI_ISL_1795420, EPI_ISL_1795421, EPI_ISL_1795422, EPI_ISL_2345552, EPI_ISL_2345573, EPI_ISL_2345574, EPI_ISL_2345580			
see above	LABORATORIO DE FRANCA	Instituto Butantan / ESALQ-Piracicaba	Antonio Jorge Martins; Bianca Cechetto Carlos. Mendelics: Bibiana Santos; Claudia Renata dos Santos Barros; David Schlesinger; David Schlesinger. Hemocentro Ribeirão Preto: Simone Kashima; Debora Botequio Moretti; Debora Botequio Moretti. Centro de Genômica Funcional da ESALQ: Luiz Lehmann Coutinho; Dimas Tadeu Covas; Elaine Cristina Marqueze; Elaine Vieira Santos; Elaine Vieira dos Santos; Elisangela Chicaroni Mattos; Erika Freitas; Evandra Strazza Rodrigues; Felipe Allan da Silva da Costa; Flavia Aburjaile; Guilherme Targino Valente; Heidge Fukumasu; Heidge Fukumasu. USP-Botucatu: Rejane Maria Tommasini Grotto; Instituto Butantan: Alexander Roberto Precioso; Jayme A. Souza-Neto; Jayme Augusto de Souza-Neto; Jessika Cristina Chagas Lesbon; José Salvatore Leister Patané; João Paulo Kitajima; Luiz Alcantara; Luiz Carlos Junior de Alcantara; Luiz Lehmann Coutinho; Maria Carolina Elias; Marta Giovanetti; Maurício Lacerda Nogueira; Patricia Akemi Assato; Rafael dos Santos Bezerra; Raquel de Lello Rocha Campos Cassano. NGS Soluções Genômicas: Pilar Drummond Sampaio Corrêa Mariani. FZEA-USP Pirassununga: Mirele Daiana Poleti; Raul Machado Neto; Rejane Maria Tommasini Grotto; Ricardo Augusto Brassaloti; Ricardo Haddad; Rodrigo Tocantins Calado.; Sandra Coccuzzo Sampaio; Sandra Coccuzzo Sampaio Vessoni; Simone Kashima; Svetoslav Nanev Slavov; Vagner Fonseca; Vincent Louis Viala
EPI_ISL_1261698	LACEN - Laboratório Central de Saúde Pública de Pernambuco	Evandro Chagas Institute	A.M.; Barbagelata; E.C.; E.M.A.; Ferreira; J.A.; Junior; K.C.; L.C.; L.S.; M.C.; P.S.; Pinheiro; Santos; Silva; Sousa; Sousa Junior; W.D.C.; da Silva
EPI_ISL_925916, EPI_ISL_926446	LACEN - Laboratório Central de Saúde Pública do Amazonas	Evandro Chagas Institute Virology	A.M.; Barbagelata; E.C.; E.M.A.; Ferreira; J.A.; Junior; K.C.; L.C.; L.S.; M.C.; P.S.; Pinheiro; Santos; Silva; Sousa; Sousa Junior; W.D.C.; da Silva
EPI_ISL_918512	LACEN - Laboratório Central de Saúde Pública do Amazonas	Evandro Chagas Institute	A.M.; Barbagelata; E.C.; E.M.A.; Ferreira; J.A.; Junior; K.C.; L.C.; L.S.; M.C.; P.S.; Pinheiro; Santos; Silva; Sousa; Sousa Junior; W.D.C.; da Silva
EPI_ISL_1164995, EPI_ISL_2488770	LACEN - Laboratório Central de Saúde Pública do Ceará	Evandro Chagas Institute	A.M.; Barbagelata; E.C.; E.M.A.; Ferreira; J.A.; Junior; K.C.; L.C.; L.S.; M.C.; P.S.; Pinheiro; Santos; Silva; Sousa; Sousa Junior; W.D.C.; da Silva
EPI_ISL_2488773, EPI_ISL_2488810	LACEN - Laboratório Central de Saúde Pública do Maranhao	Evandro Chagas Institute	A.M.; Barbagelata; E.C.; E.M.A.; Ferreira; J.A.; Junior; K.C.; L.C.; L.S.; M.C.; P.S.; Pinheiro; Santos; Silva; Sousa; Sousa Junior; W.D.C.; da Silva
EPI_ISL_918515, EPI_ISL_918550	LACEN - Laboratório Central de Saúde Pública do Para	Evandro Chagas Institute	A.M.; Barbagelata; E.C.; E.M.A.; Ferreira; J.A.; Junior; K.C.; L.C.; L.S.; M.C.; P.S.; Pinheiro; Santos; Silva; Sousa; Sousa Junior; W.D.C.; da Silva
EPI_ISL_1086377, EPI_ISL_1164994	LACEN - Laboratório Central de Saúde Pública do Paraiba	Evandro Chagas Institute	A.M.; Barbagelata; E.C.; E.M.A.; Ferreira; J.A.; Junior; K.C.; L.C.; L.S.; M.C.; P.S.; Pinheiro; Santos; Silva; Sousa; Sousa Junior; W.D.C.; da Silva
EPI_ISL_1086376	LACEN - Laboratório Central de Saúde Pública do Rio Grande do Norte	Evandro Chagas Institute	A.M.; Barbagelata; E.C.; E.M.A.; Ferreira; J.A.; Junior; K.C.; L.C.; L.S.; M.C.; P.S.; Pinheiro; Santos; Silva; Sousa; Sousa Junior; W.D.C.; da Silva
EPI_ISL_3912433	LACEN LABORATORIO CENTRAL DE SAUDE PUBLICA	Analytical Competence Molecular Epidemiology Lab/ACME, Oswaldo Cruz Foundation, Ceara (FIOCRUZ CE)	Cleber Furtado Aksenen; Fabio Miyajima; Fernando Braga Stehling; Francisco Eder de Moura Lopes; Jamille Maria Mendes Bezerra; Joaquim Cesar do Nascimento Sousa Junior; Pedro Miguel Carneiro Jeronimo; Suzana Porto Almeida & Lucas Delerino on behalf of COVID-19 FIOCRUZ Genomic Network; Thais Ferreira de Oliveira; Thais de Oliveira Costa; Ticiane Cavalcante de Souza; Veridiana Pessoa Miyajima
EPI_ISL_1293052, EPI_ISL_1303499	LACEN de Rondonia	Instituto Adolfo Lutz, Interdiciplinary Procedures Center, Strategic Laboratory	Caio Vinicius Dias Lopes; Claudia Regina Gonçalves; Claudio Tavares Sacchi; Erica Valessa Ramos Gomes; Karoline Rodrigues Campos
EPI_ISL_1196287, EPI_ISL_1196288, EPI_ISL_1196291, EPI_ISL_1196293	LACEN do Distrito Federal	Instituto Adolfo Lutz, Interdiciplinary Procedures Center, Strategic Laboratory	Caio Vinicius Dias Lopes; Claudia Regina Gonçalves; Claudio Tavares Sacchi; Erica Valessa Ramos Gomes; Karoline Rodrigues Campos
EPI_ISL_943988	LACEN do Estado de Goias	Instituto Adolfo Lutz, Interdiciplinary Procedures Center, Strategic Laboratory	Claudia Regina Gonçalves; Claudio Tavares Sacchi; Erica Valessa Ramos Gomes; Karoline Rodrigues Campos
EPI_ISL_3316170, EPI_ISL_3316222	LACEN do Estado de Mato Grosso	Instituto Adolfo Lutz, Interdiciplinary Procedures Center, Strategic Laboratory	Caio Vinicius Dias Lopes; Claudia Regina Gonçalves; Claudio Tavares Sacchi; Karoline Rodrigues Campos; Leonardo Tadeu de Araujo; Marlon Benedito Nascimento Santos
EPI_ISL_943974, EPI_ISL_943975, EPI_ISL_943976, EPI_ISL_943977, EPI_ISL_943978, EPI_ISL_943979, EPI_ISL_943981, EPI_ISL_943983, EPI_ISL_943985, EPI_ISL_943991, EPI_ISL_2919221			
see above	LACEN do Estado de Tocantins	Instituto Adolfo Lutz, Interdiciplinary Procedures Center, Strategic Laboratory	Caio Vinicius Dias Lopes; Claudia Regina Gonçalves; Claudio Tavares Sacchi; Erica Valessa Ramos Gomes; Karoline Rodrigues Campos
EPI_ISL_1040825, EPI_ISL_1040826, EPI_ISL_1040827, EPI_ISL_1040828, EPI_ISL_1040830, EPI_ISL_1040832, EPI_ISL_1040834, EPI_ISL_1040838, EPI_ISL_1040841, EPI_ISL_1040846, EPI_ISL_1040847, EPI_ISL_1040849, EPI_ISL_1040850, EPI_ISL_1121329, EPI_ISL_1139052, EPI_ISL_1139054, EPI_ISL_1139056, EPI_ISL_1139057, EPI_ISL_1139060, EPI_ISL_1139067, EPI_ISL_1171621, EPI_ISL_1358305, EPI_ISL_1358308, EPI_ISL_1358311, EPI_ISL_1358314, EPI_ISL_1468437			
see above	LACEN do Mato Grosso do Sul	Instituto Adolfo Lutz, Interdiciplinary Procedures Center, Strategic Laboratory	Caio Vinicius Dias Lopes; Claudia Regina Gonçalves; Claudio Tavares Sacchi; Erica Valessa Ramos Gomes; Karoline Rodrigues Campos
EPI_ISL_502875	LACEN/PE	LABBE, Federal University of Pernambuco	ANTONIO CARLOS DE FREITAS; BRUNO SAMPAIO; HEIDI LACERDA ALVES DA CRUZ; MAIRA GALDINO DA ROCHA PITTA; MARCOS ANTONIO DE MORAIS JUNIOR; MARCOS DA SILVEIRA REGUEIRA NETO; MICHELLY CRISTINY PEREIRA; REGINALDO GONCALVES DE LIMA NETO; SERGIO DE SA LEITAO PAIVA JUNIOR; VALDIR DE QUEIROZ BALBINO.; WILSON JOSE DA SILVA JUNIOR; ZILDENE DE SOUSA SILVEIRA
EPI_ISL_2821285, EPI_ISL_2821287, EPI_ISL_2821291, EPI_ISL_2821293, EPI_ISL_2821298, EPI_ISL_2821305, EPI_ISL_2821311, EPI_ISL_2821316, EPI_ISL_2821318, EPI_ISL_2821320, EPI_ISL_2821321, EPI_ISL_2821323, EPI_ISL_2821324, EPI_ISL_2821325, EPI_ISL_3046171, EPI_ISL_3060274, EPI_ISL_3704467, EPI_ISL_3704474, EPI_ISL_3704487, EPI_ISL_3704496, EPI_ISL_3704499, EPI_ISL_3704504, EPI_ISL_3704511, EPI_ISL_3704514, EPI_ISL_3704518, EPI_ISL_3704530, EPI_ISL_3704534, EPI_ISL_3704536, EPI_ISL_3704539, EPI_ISL_3704543, EPI_ISL_3704546, EPI_ISL_3704549, EPI_ISL_3704553, EPI_ISL_3704582, EPI_ISL_3704585, EPI_ISL_3704590, EPI_ISL_3704597, EPI_ISL_3704614, EPI_ISL_3704622, EPI_ISL_3704631, EPI_ISL_3704640, EPI_ISL_3704647, EPI_ISL_3704651, EPI_ISL_3704654, EPI_ISL_3704658, EPI_ISL_3704662, EPI_ISL_3704666, EPI_ISL_3704669, EPI_ISL_3704673, EPI_ISL_3704676, EPI_ISL_3704680, EPI_ISL_3704687, EPI_ISL_3704691, EPI_ISL_3704694, EPI_ISL_3704698, EPI_ISL_3704706, EPI_ISL_3704709, EPI_ISL_3704746, EPI_ISL_3704749, EPI_ISL_3704753, EPI_ISL_3704756, EPI_ISL_3704764, EPI_ISL_3704771, EPI_ISL_3704775, EPI_ISL_3704778, EPI_ISL_3704785, EPI_ISL_3704807, EPI_ISL_3704814, EPI_ISL_3704818, EPI_ISL_3704821, EPI_ISL_3704833, EPI_ISL_3704848, EPI_ISL_3704880, EPI_ISL_3704884, EPI_ISL_3704917, EPI_ISL_3704922, EPI_ISL_3704937, EPI_ISL_3704952, EPI_ISL_3704963, EPI_ISL_3705014, EPI_ISL_3705017, EPI_ISL_3705032, EPI_ISL_3705077, EPI_ISL_3705083, EPI_ISL_3705090, EPI_ISL_3705092, EPI_ISL_3705095, EPI_ISL_3705127, EPI_ISL_3835305, EPI_ISL_3835306, EPI_ISL_3835307, EPI_ISL_3835309, EPI_ISL_3835311, EPI_ISL_3835313, EPI_ISL_3835315, EPI_ISL_3835316, EPI_ISL_3835317, EPI_ISL_3835318, EPI_ISL_3835319, EPI_ISL_3835320, EPI_ISL_3835321, EPI_ISL_3835324, EPI_ISL_3835325, EPI_ISL_3835326, EPI_ISL_3835327, EPI_ISL_3835328, EPI_ISL_3835329, EPI_ISL_3835331, EPI_ISL_3835332, EPI_ISL_3835335, EPI_ISL_3835336, EPI_ISL_3835337, EPI_ISL_3835338, EPI_ISL_3835339, EPI_ISL_3835340			
see above	LACEN/PE	WallauLab on behalf of Fiocruz COVID-19 Genomic Surveillance Network	Alexandre Freitas da Silva; Antonio Mauro Rezende; Cassia Docena; Constância Flávia Junqueira Ayres; Cássia Docena; Duschinka Ribeiro Duarte Guedes; Elisama Helvecio; Filipe Zimmer Dezordi; Gabriel Luz Wallau; Gustavo Barbosa de Lima; Lais Ceschini Machado; Larissa Krokovsky; Laís Ceschini Machado; Lilian Caroliny Amorim Silva; Marcelo Henrique dos Santos Paiva; Matheus Filgueira Bezerra; Sinval Pinto Brandão Filho
EPI_ISL_1213220, EPI_ISL_1213226, EPI_ISL_1213269, EPI_ISL_1213294, EPI_ISL_1213401	LAFEM/UESC	Bioinformatics Laboratory / LNCC	Alessandra P Lamarca; Alexandra L Gerber; Ana Paula Melo Mariano; Ana Paula de C Guimarães; Ana Tereza R Vasconcelos; Angela Maria Guimarães Santos; Bianca Mendes Maciel; Danielle Angst Secco; Eduardo Sérgio Soares Sousa; Eloiza Helena Campana; Francisco Paulo Freire Neto; George Rego Albuquerque; Kátia Castanho Scortecci; Lucymara Fassarella Agnez Lima; Luiz G P de Almeida; Luís Cristóvão Porto; Otavio J. Brustolini; Paulo Ricardo Nascimento; Ronaldo da Silva Francisco Jr; Sandra Rocha Gadelha; Selma Maria Bezerra Jeronimo; Vinícius Pietta Perez
EPI_ISL_861867, EPI_ISL_861868, EPI_ISL_861875, EPI_ISL_861876, EPI_ISL_861879, EPI_ISL_861885, EPI_ISL_861890, EPI_ISL_861892, EPI_ISL_861894, EPI_ISL_861895, EPI_ISL_861896, EPI_ISL_861900, EPI_ISL_861902, EPI_ISL_861903, EPI_ISL_861905, EPI_ISL_861906, EPI_ISL_861909, EPI_ISL_861912, EPI_ISL_861913, EPI_ISL_3457423			
see above	LATE - Laboratório de Técnicas	LATE - Laboratório de Técnicas Especiais -	Alexandre Hideaki Takara; Ana Paula Moreira Salles; Anelisie da Silva Santos; Deyvid Amgarten; Erick Gustavo Dorlass; Fernanda de Mello Malta; João Renato Rebello Pinho; Marcio Anunciacao Menezes; Pedro Henrique Sebe Rodrigues; Raquel Riyuzo

	Especiais - Hospital Israelita Albert Einstein	Hospital Israelita Albert Einstein	
EPI_ISL_1213453, EPI_ISL_1213454, EPI_ISL_1213458, EPI_ISL_1213459, EPI_ISL_1213461	LBM/UFPB	Bioinformatics Laboratory / LNCC	Alessandra P Lamarca; Alexandra L Gerber; Ana Paula Melo Mariano; Ana Paula de C Guimarães; Ana Tereza R Vasconcelos; Angela Maria Guimarães Santos; Bianca Mendes Maciel; Danielle Angst Secco; Eduardo Sérgio Soares Sousa; Eloiza Helena Campana; Francisco Paulo Freire Neto; George Rego Albuquerque; Kátia Castanho Scortecci; Lucymara Fassarella Agnez Lima; Luiz G P de Almeida; Luís Cristóvão Porto; Otavio J. Brustolini; Paulo Ricardo Nascimento; Ronaldo da Silva Francisco Jr; Sandra Rocha Gadelha; Selma Maria Bezerra Jeronimo; Vinícius Pietta Perez
EPI_ISL_755644, EPI_ISL_833162, EPI_ISL_861669	Lab LOC - Itapecerica da Serra	Instituto Adolfo Lutz, Interdiciplinary Procedures Center, Strategic Laboratory	Claudia Regina Gonçalves; Claudio Tavares Sacchi; Erica Valessa Ramos Gomes; Karoline Rodrigues Campos
EPI_ISL_985178, EPI_ISL_1039698, EPI_ISL_1039704	Lab Loc - Itapecerica da Serra	Instituto Adolfo Lutz, Interdiciplinary Procedures Center, Strategic Laboratory	Claudia Regina Gonçalves; Claudio Tavares Sacchi; Erica Valessa Ramos Gomes; Karoline Rodrigues Campos
EPI_ISL_511103	Labeto - CAB - Leiria	Instituto Nacional de Saude (INSA)	Borges et al
EPI_ISL_2557340, EPI_ISL_2557341, EPI_ISL_2557342, EPI_ISL_2557346, EPI_ISL_2557350, EPI_ISL_2557353, EPI_ISL_2557358			
see above	Laboratorio Central de Saude Publica do Estado de Minas Gerais (LACEN/MG)	Laboratory of Respiratory Viruses and Measles, Oswaldo Cruz Institute, FIOCRUZ	Alice Sampaio Rocha; Ana Carolina Mendonca; Andre Felipe Leal Bernardes; Anna Carolina Paixao; Elisa Cavalcante Pereira; Fernando Motta; Luciana Appolinario; Marilda Siqueira on behalf of the Fiocruz COVID-19 Genomic Surveillance Network; Paola Resende; Renata Serrano Lopes; Taina Venas
EPI_ISL_2645855, EPI_ISL_2645860, EPI_ISL_2645861, EPI_ISL_2645904	Laboratorio Central de Saude Publica do Estado do Para (LACEN/PA)	Laboratory of Respiratory Viruses and Measles, Oswaldo Cruz Institute, FIOCRUZ	Alice Sampaio Rocha; Ana Carolina Mendonca; Anna Carolina Paixao; Elisa Cavalcante Pereira; Fernando Motta; Luciana Appolinario; Marilda Siqueira on behalf of the Fiocruz COVID-19 Genomic Surveillance Network; Paola Resende; Renata Serrano Lopes; Taina Venas; Valnete Andrade
EPI_ISL_1416434	Laboratorio Central de Epidemiología (LCE)	Instituto de Biotecnología de la UNAM	Alejandro Sanchez-Flores; Alfredo Herrera-Estrella; Alicia Ocaña-Mondragón; Angel Gustavo Salas-Lais; Bernardo Martínez-Miguel; Blanca Taboada; Brenda Irasema Maldonado-Meza; Carla Ivón Herrera-Najera; Carlos F. Arias; Celia Boukadida; Clara Esperanza Santacruz-Tinoco; Concepción Grajales-Muñiz; Consorcio Mexicano de Vigilancia Genómica (CoViGen-Mex). Authors (in alphabetical order): Julio Elias Alvarado-Yaah; Fernando Fontove-Herrera; Francisco Pulido; Gloria Elena Espinoza-Ayala; Gloria María Molina-Salinas; Gloria Vazquez; Hector Esteban Paz-Juárez; Hector Montoya-Fuentes; Helen Haydee Fernanda Ramírez-Plascencia; Jorge Ivan Salinal-Nevarez; José Antonio Enciso-Moreno; José Esteban Muñoz-Medina; José de Jesús Nuñez-Contreras; Juan Bautista Chale-Dzul; Luis Alberto Ochoa-Carrera; Margarita Matías-Florentino; María Guadalupe Santiago-Mauricio; María Guadalupe de Jesús Mireles-Rivera; Nelly Sélem-Mojica; Pavel Isa; Ricardo Grande; Santiago Ávila-Ríos; Víctor Hugo Borja-Aburto
EPI_ISL_2491721, EPI_ISL_2491723, EPI_ISL_2491724, EPI_ISL_2491725, EPI_ISL_2491730, EPI_ISL_2491731, EPI_ISL_2491751			
see above	Laboratorio Central de Saude Publica do Estado da Bahia (LACEN/BA)	Laboratory of Respiratory Viruses and Measles, Oswaldo Cruz Institute, FIOCRUZ	Alice Sampaio Rocha; Ana Carolina Mendonca; Anna Carolina Paixao; Elisa Cavalcante Pereira; Felicidade Pereira; Fernando Motta; Luciana Appolinario; Marilda Siqueira on behalf of the Fiocruz COVID-19 Genomic Surveillance Network; Paola Resende; Renata Serrano Lopes; Taina Venas
EPI_ISL_2157550, EPI_ISL_2536352	Laboratorio Central de Saude Publica do Estado da Paraiba (LACEN-PB)	Laboratory of Respiratory Viruses and Measles, Oswaldo Cruz Institute, FIOCRUZ	Alice Sampaio Rocha; Ana Carolina Mendonca; Anna Carolina Paixao; Dalane Loudal Florentino Teixeira; Elisa Cavalcante Pereira; Fernando Motta; Joao Felipe Bezerra; Luciana Appolinario; Marilda Siqueira on behalf of the Fiocruz COVID-19 Genomic Surveillance Network; Paola Resende; Renata Serrano Lopes; Taina Venas
EPI_ISL_2645637, EPI_ISL_2645638, EPI_ISL_2645656, EPI_ISL_2645685, EPI_ISL_2645688, EPI_ISL_3434817	Laboratorio Central de Saude Publica do Estado de Alagoas (LACEN/AL)	Laboratory of Respiratory Viruses and Measles, Oswaldo Cruz Institute, FIOCRUZ	Agatha Soares; Alice Sampaio Rocha; Ana Carolina Mendonca; Anderson Brandao Leite; Anna Carolina Paixao; Elisa Cavalcante Pereira; Fernando Motta; Ighor Arantes; Luciana Appolinario; Marilda Siqueira on behalf of the Fiocruz COVID-19 Genomic Surveillance Network; Paola Resende; Renata Serrano Lopes; Taina Venas
EPI_ISL_2645420	Laboratorio Central de Saude Publica do Estado de Minas Gerais (LACEN-MG)	Laboratory of Respiratory Viruses and Measles, Oswaldo Cruz Institute, FIOCRUZ	Alice Sampaio Rocha; Ana Carolina Mendonca; Andre Felipe Leal Bernardes; Anna Carolina Paixao; Elisa Cavalcante Pereira; Fernando Motta; Luciana Appolinario; Marilda Siqueira on behalf of the Fiocruz COVID-19 Genomic Surveillance Network; Paola Resende; Renata Serrano Lopes; Taina Venas
EPI_ISL_2660459, EPI_ISL_2660473, EPI_ISL_2660476, EPI_ISL_2660493, EPI_ISL_2660496, EPI_ISL_2660498, EPI_ISL_2660500, EPI_ISL_2660502, EPI_ISL_2660504			
see above	Laboratorio Central de Saude Publica do Estado de Minas Gerais (LACEN/MG)	Laboratory of Respiratory Viruses and Measles, Oswaldo Cruz Institute, FIOCRUZ	Alice Sampaio Rocha; Ana Carolina Mendonca; Andre Felipe Leal Bernardes; Anna Carolina Paixao; Elisa Cavalcante Pereira; Fernando Motta; Luciana Appolinario; Marilda Siqueira on behalf of the Fiocruz COVID-19 Genomic Surveillance Network; Paola Resende; Renata Serrano Lopes; Taina Venas
EPI_ISL_2157587, EPI_ISL_2196238, EPI_ISL_2660596, EPI_ISL_2660600, EPI_ISL_2660663, EPI_ISL_2660664, EPI_ISL_2660670, EPI_ISL_2660678, EPI_ISL_2660689, EPI_ISL_2660690			
see above	Laboratorio Central de Saude Publica do Estado de Sergipe (LACEN/SE)	Laboratory of Respiratory Viruses and Measles, Oswaldo Cruz Institute, FIOCRUZ	Alice Sampaio Rocha; Ana Carolina Mendonca; Anna Carolina Paixao; Cliomar Alves dos Santos; Elisa Cavalcante Pereira; Fernando Motta; Luciana Appolinario; Marilda Siqueira on behalf of the Fiocruz COVID-19 Genomic Surveillance Network; Paola Resende; Renata Serrano Lopes; Tainá Moreira Martins Venas
EPI_ISL_2645546, EPI_ISL_2645549, EPI_ISL_2645551, EPI_ISL_2645554, EPI_ISL_2645561, EPI_ISL_2645564, EPI_ISL_2645565, EPI_ISL_2645569, EPI_ISL_2645574, EPI_ISL_2645578, EPI_ISL_2645579, EPI_ISL_2645586, EPI_ISL_2645588, EPI_ISL_2645599, EPI_ISL_2645606, EPI_ISL_2645613, EPI_ISL_2645614, EPI_ISL_2645626, EPI_ISL_2645627, EPI_ISL_2645633			
see above	Laboratorio Central de Saude Publica do Estado do Espirito Santo (LACEN/ES)	Laboratory of Respiratory Viruses and Measles, Oswaldo Cruz Institute, FIOCRUZ	Alice Sampaio Rocha; Ana Carolina Mendonca; Anna Carolina Paixao; Eilisa Cavalcante Pereira; Fernando Motta; Luciana Appolinario; Marilda Siqueira on behalf of the Fiocruz COVID-19 Genomic Surveillance Network; Paola Resende; Renata Serrano Lopes; Rodrigo Ribeiro Rodrigues; Taina Venas
EPI_ISL_1465225, EPI_ISL_2983247, EPI_ISL_2983248, EPI_ISL_2983249, EPI_ISL_2983255, EPI_ISL_2983316, EPI_ISL_2983318, EPI_ISL_2983472			
see above	Laboratorio Central de Saude Publica do Estado do Maranhao (LACEN-MA)	Laboratory of Respiratory Viruses and Measles, Oswaldo Cruz Institute, FIOCRUZ	Agatha Cristinne Prudencio; Alice Sampaio Rocha; Ana Carolina Mendonca; Anna Carolina Paixao; Elisa Cavalcante Pereira; Fernando Motta; Ighor Leonardo Arantes Gomes; Lidio Gonçalves Lima Neto; Luciana Appolinario; Marilda Siqueira on behalf of the Fiocruz COVID-19 Genomic Surveillance Network; Paola Resende; Renata Serrano Lopes; Taina Moreira Venas; Tainá Venas
EPI_ISL_2775424, EPI_ISL_2775425, EPI_ISL_2775437, EPI_ISL_2775444, EPI_ISL_2775452, EPI_ISL_2775454, EPI_ISL_2775455, EPI_ISL_2775456, EPI_ISL_2775458, EPI_ISL_2775463, EPI_ISL_2775466, EPI_ISL_2775469, EPI_ISL_2775470, EPI_ISL_2775472, EPI_ISL_2775473, EPI_ISL_2775474, EPI_ISL_2775476, EPI_ISL_2775477, EPI_ISL_2775478, EPI_ISL_2775479, EPI_ISL_2775480, EPI_ISL_2775481			
see above	Laboratorio Central de Saude Publica do Estado do Parana (Instituto de Biologia Molecular do Paraná (LAC)EN-PR)	Instituto Carlos Chagas - Fiocruz	Alessandra De Melo Aguiar; Andreia Akemi Suzukawa; Andréa Rodrigues Ávila; Bruno Dallagiovanna; Dalila Zanette; Eduardo Balsanelli; Emanuel Maltempi de Souza; Fabio Passetti; Fabricio Klerynton Marchini; Fábio de Oliveira Pedrosa; Guilherme Becker; Helisson Faoro; Hellen Geremias dos Santos; Irina Nastassja Riediger; Letusa Albrecht; Lucas Blanes; Luis Gustavo Morello; Lysangela Ronalte Alves; Maria do Carmo Debur; Mauro de Medeiros Oliveira; Michelle Orane Schemberger; Paola Cristina Resende; Sheila Cristina Nardeli; Tiago Gräf; Valter Antônio de Baura
EPI_ISL_2731459, EPI_ISL_2731461, EPI_ISL_2731467, EPI_ISL_3061893, EPI_ISL_3243095, EPI_ISL_3243097, EPI_ISL_3243098, EPI_ISL_3243099, EPI_ISL_3243101, EPI_ISL_3243102, EPI_ISL_3243104, EPI_ISL_3243105, EPI_ISL_3243106, EPI_ISL_3243107, EPI_ISL_3243108, EPI_ISL_3243109, EPI_ISL_3243110, EPI_ISL_3243111, EPI_ISL_3243112			
see above	Laboratorio Central de Saude Publica do Estado do Parana (LACEN/PR)	Laboratory of Respiratory Viruses and Measles, Oswaldo Cruz Institute, FIOCRUZ	Agatha Soares; Alice Sampaio Rocha; Ana Carolina Mendonca; Anna Carolina Paixao; Elisa Cavalcante Pereira; Fernando Motta; Ighor Leonardo Arantes; Irina Riediger; Luciana Appolinario; Marilda Siqueira on behalf of the Fiocruz COVID-19 Genomic Surveillance Network; Paola Resende; Renata Serrano Lopes; Taina Venas
EPI_ISL_2603526, EPI_ISL_2661786, EPI_ISL_2661795, EPI_ISL_2661797, EPI_ISL_2661798, EPI_ISL_2661799, EPI_ISL_2661801, EPI_ISL_2661819, EPI_ISL_2661827, EPI_ISL_2661865			
see above	Laboratorio Central de Saude Publica do Estado do Rio Grande do Sul (LACEN-RS)	Laboratory of Respiratory Viruses and Measles, Oswaldo Cruz Institute, FIOCRUZ	Alice Sampaio Rocha; Ana Carolina Mendonca; Anderson Brandao Leite; Anna Carolina Paixao; Elisa Cavalcante Pereira; Fernando Motta; Luciana Appolinario; Marilda Siqueira on behalf of the Fiocruz COVID-19 Genomic Surveillance Network; Paola Resende; Renata Serrano Lopes; Richard Salvato; Taina Venas; Tatiana Schaffer Gregianini
EPI_ISL_3048754, EPI_ISL_3048755, EPI_ISL_3048772, EPI_ISL_3048797, EPI_ISL_3048810, EPI_ISL_3048813, EPI_ISL_3048815, EPI_ISL_3048820, EPI_ISL_3048821, EPI_ISL_3048824, EPI_ISL_3048826, EPI_ISL_3048827, EPI_ISL_3048831, EPI_ISL_3048834			
see above	Laboratorio Central de Saude Publica do Estado do Rio Grande do Sul (LACEN-RS)	Laboratório de Biologia Molecular da Universidade Federal de Ciências da Saúde de Porto Alegre	Adriana Seixas; Ana B. G. Veiga; Ana Paula Muterle Varela; Fabiana Quoos Mayer; Fernando Hayashi Sant'Anna; Janira Prichula; Letícia Garay Martins; Richard Steiner Salvato; Tatiana Schäffer Gregianini
EPI_ISL_2731471	Laboratorio Central	Laboratory of	Alice Sampaio Rocha; Ana Carolina Mendonca; Andrea Cony Cavalcanti; Anna Carolina Paixao; Elisa Cavalcante Pereira; Fernando Motta; Luciana Appolinario; Marilda Siqueira on behalf of the Fiocruz COVID-19 Genomic Surveillance Network; Paola Resende; Renata Serrano Lopes; Taina Venas

	de Saude Publica do Estado do Rio de Janeiro (LACEN/RJ)	Respiratory Viruses and Measles, Oswaldo Cruz Institute, FIOCRUZ	
EPI_ISL_979352	Laboratorio Estatal de Salud Pública de Nuevo León	Laboratorio de Infectología Molecular, Departamento de Bioquímica y Medicina Molecular,Facultad de Medicina - Universidad Autónoma de Nuevo León	Ana M. Rivas-Estilla; Consuelo Treviño-Garza; Daniel Arellanos-Soto; Else del Carmen García-García; Gloria A. Jasso-de-la-Peña; Kame A. Galán-Huerta; Manuel E. de-la-O-Cavazos; María F. Herrera-Saldivar; Natalia Martínez-Acuña; Roberto Montes-de-Oca; Samuel Buentello-Wong; Sonia A. Lozano-Sepúlveda
EPI_ISL_735398	Laboratorio Fleury	Instituto Adolfo Lutz, Interdiciplinary Procedures Center, Strategic Laboratory	Claudia Regina Gonçalves; Claudio Tavares Sacchi; Erica Valessa Ramos Gomes; Karoline Rodrigues Campos
EPI_ISL_861628	Laboratorio Municipal de Guarulhos	Instituto Adolfo Lutz, Interdiciplinary Procedures Center, Strategic Laboratory	Claudia Regina Gonçalves; Claudio Tavares Sacchi; Erica Valessa Ramos Gomes; Karoline Rodrigues Campos
EPI_ISL_801386, EPI_ISL_801387, EPI_ISL_801388, EPI_ISL_801389, EPI_ISL_801390, EPI_ISL_801391, EPI_ISL_801392, EPI_ISL_801393, EPI_ISL_801394, EPI_ISL_801395, EPI_ISL_801396, EPI_ISL_833131, EPI_ISL_1068082, EPI_ISL_1068089, EPI_ISL_1068090, EPI_ISL_1068093, EPI_ISL_1068095, EPI_ISL_1068096, EPI_ISL_1068101, EPI_ISL_1068102, EPI_ISL_1068107, EPI_ISL_1068127, EPI_ISL_1068129, EPI_ISL_1068130, EPI_ISL_1068132, EPI_ISL_1068133, EPI_ISL_1068134, EPI_ISL_1068135, EPI_ISL_1068137, EPI_ISL_1068146, EPI_ISL_1068148, EPI_ISL_1068152, EPI_ISL_1068161, EPI_ISL_1068168, EPI_ISL_1068172, EPI_ISL_1068175, EPI_ISL_1068182, EPI_ISL_1068190, EPI_ISL_1068192, EPI_ISL_1068197, EPI_ISL_1068205, EPI_ISL_1068206, EPI_ISL_1068208, EPI_ISL_1068209, EPI_ISL_1068210, EPI_ISL_1068211, EPI_ISL_1068213, EPI_ISL_1068214, EPI_ISL_1068217, EPI_ISL_1068218, EPI_ISL_1068242, EPI_ISL_1068244, EPI_ISL_1068245, EPI_ISL_1068246, EPI_ISL_1068247, EPI_ISL_1068250, EPI_ISL_1068253, EPI_ISL_1068254, EPI_ISL_1068257, EPI_ISL_1661251, EPI_ISL_2777360, EPI_ISL_2777371, EPI_ISL_2777387, EPI_ISL_2777390, EPI_ISL_2777391, EPI_ISL_2777392, EPI_ISL_2777394			
see above	Laboratorio de Ecologia de Doencas Transmissiveis na Amazonia, Instituto Leonidas e Maria Deane - Fiocruz Amazonia	Laboratorio de Ecologia de Doencas Transmissiveis na Amazonia, Instituto Leonidas e Maria Deane - Fiocruz Amazonia	André Corado; Debora Duarte; Felipe Naveca; Felipe Naveca on behalf of the Fiocruz COVID-19 Genomic Surveillance Network; Fernanda Nascimento; George Silva; Karina Pessoa; Luciana Gonçalves; Maria Júlia Brandão; Matilde Mejía; Michele Jesus; Valdinete Nascimento; Victor Souza; Ágatha Costa
EPI_ISL_1511641	Laboratorio de Patologia Clinica - UNICAMP	Laboratorio de Estudos de Virus Emergentes	Alessandro S. Farias; Aline Vieira; André S. Vieira; Angelica Schreiber; Antonio C. G. Carlos Jr; Barbara F. N. Carvalho; Camila L. Simeoni; Daniel A. Toledo-Teixeira; Emerson S.S. França; Ester C. Sabino; Fabiana Granja; Fernando R. Spilki; Gisele A. Pedroso; Ingra M. Claro; José Luiz Proenca-Modena; Julia Forato; Kamila C. S. Krywacz; Karina Bispo-dos-Santos; Luciana S. Mofatto; Luís Felipe Bachur; Luís G. O. Cardoso; Magnun N. N. Santos; Marcelo A. Mori; Maria H. P. Pavan; Maria L. Moretti; Mariene R. Amorim; Natalia S. Brunetti; Nuno R. Faria; Patricia A. F. Leme; Pierina L. Parise; Rodrigo Angerami; Tania R. Zaccariotto; William M. Souza
EPI_ISL_2008937, EPI_ISL_2008939, EPI_ISL_2107304, EPI_ISL_2544838, EPI_ISL_2544848, EPI_ISL_2544849, EPI_ISL_2544851, EPI_ISL_2544857, EPI_ISL_2544858, EPI_ISL_2544862, EPI_ISL_2544873, EPI_ISL_2544879, EPI_ISL_2544880, EPI_ISL_2544894, EPI_ISL_2544895, EPI_ISL_3048949, EPI_ISL_3048950			
see above	Laboratorio de Pesquisa em Virologia, FAMERP, SJRP	Laboratorio de Pesquisa em Virologia, FAMERP, SJRP	Cecília Artico Banho; Cíntia Bittar; Fábio Sossai Possebon; Guilherme Campos; Helena Lage Ferreira; Jorge A. Petroli Marchesi; João Pessoa Araújo Jr.; Leila Sabrina Ullmann; Lívia Sacchetto; Maisa C. Pereira Parra; Marília Moraes; Maurício L. Nogueira.; Paula Rahal; Paulo Inacio da Costa
EPI_ISL_1534655	Laboratorio de Referencia Nacional de Virus Respiratorio. Instituto Nacional de Salud Perú	Laboratorio de Referencia Nacional de Biotecnología y Biología Molecular. Instituto Nacional de Salud Perú	Carlos Padilla Rojas; Henri Bailon Calderon; Johanna Balbuena Torrez; Karolyn Vega Chozo; Luis Barcena; Marco Galarza Perez; Maribel Huaringa Nuñez; Nancy Rojas Serrano; Omar Caceres Rey; Priscila Lope Pari
EPI_ISL_1111146	Laboratorio de Referencia Nacional de Virus Respiratorio. Instituto Nacional de Salud Perú	Laboratorio de Referencia Nacional de Enteropatógenos. Instituto Nacional de Salud del Perú	Fiorella Orellana Peralta; Iris Silva Molina; Junior Caro Castro; Ronnie Gavilan Chavez; Veronica Hurtado Vela; Willi Quino Sifuentes
EPI_ISL_623130, EPI_ISL_717807, EPI_ISL_717808, EPI_ISL_717810, EPI_ISL_717811, EPI_ISL_717812, EPI_ISL_717813, EPI_ISL_717814, EPI_ISL_717815, EPI_ISL_717818, EPI_ISL_717819, EPI_ISL_717820, EPI_ISL_717821, EPI_ISL_717822, EPI_ISL_717823, EPI_ISL_717824, EPI_ISL_717825, EPI_ISL_717826, EPI_ISL_717827, EPI_ISL_717828, EPI_ISL_717829, EPI_ISL_717830			
see above	Laboratorio de Virologia Molecular / UFRJ	Bioinformatics Laboratory / LNCC	Alexandra L Gerber; Amilcar Tanuri; Ana Paula de C Guimarães; Ana Tereza R de Vasconcelos; Andréa Cony Cavalcanti; Carolina M Voloch; Claudia dos Santos Rodrigues; Covid19-UFRJ Workgroup; Cynthia C Cardoso; Diana Mariani; Luiz G P de Almeida; Luís Cristóvão Pôrto; Orlando C. Ferreira; Otavio Bustrolini; Otavio J. Brustolini; Renato S Aguiar; Ronaldo S Francisco Jr; Ronaldo da Silva F Jr; Terezinha M P P Castiñeira; Terezinha M P P Castiñeiras
EPI_ISL_3090038, EPI_ISL_3090039	Laboratorio de Virología Molecular, Centro Universitario Regional del Litoral Norte. Universidad de la República, Salto, Uruguay	Centro de Innovación en Vigilancia Epidemiológica (CiVE), Institut Pasteur Montevideo, Uruguay	Alicia Costábile; Alvaro Fajardo; Ana Moller; Andrés Lizasoain; Belén González; Bernardina Rivera; Cecilia Alonso; Cecilia Salazar; Gonzalo Bello; Gonzalo Moratorio; Gregorio Iraola; Henry Albornoz; Ignacio Ferrés; Javier Hurtado; Juan Zanetti; Julio Medina; Luciana Griffero; Lucía Spangenberg; Ma Noel Bentancor; Ma Pía Techera; Mailen Arleo; Martina Alonso; Matías Maidana; Mauricio Méndez; Melissa Duquía; Mercedes Paz; Natalia Rego; Natalia Reyes; Nicolas Nin; Odhille Chappos; Paula Perbolianachis; Pilar Moreno; Rodney Colina; Rodrigo Arce; Tamara Fernández-Calero; Tania Possi; Veronica Noya; Viviana Bortagaray
EPI_ISL_1798980, EPI_ISL_1799147, EPI_ISL_1928690	Laboratory Corporation of America	Centers for Disease Control and Prevention Division of Viral Diseases, Pathogen Discovery	Adrian Paskey; Amanda Douglas; Amanda Suchanek; Andrea Throop; Ayla Burns; Benjamin Rambo-Martin; Bobbi Croy; Brian Krueger; Brian Norvell; Christopher Gulvick; Christos Petropoulos; Clinton R. Paden; Craig Lukasik; Dakota Howard; Darlene Wagner; Debbie Boles; Dhwani Batra; Duncan MacCannell; Eyad Almasri; Goran Stevovic; Howard Engler; Hrushikesh Deshmukh; Jake Humphrey; Jana Schroth; Jason Caravas; Joe Voshell; John Pruitt; Jonathan Meltzer; Jonathan Williams; Kara Moser; Kimberly Wagner; Lax Iyer; Lyndon Tilson; Manoj Jain; Marcia Eisenberg; Mary Ann Cristobal; Mary Williamson; Matthew Schmerer; Michael Levandoski; Mike Sapeta; Mindy Nye; Minoo Agarwal; Mohan Kolli; Nuthawin Charoensri; Oren Cohen; Peter W. Cook; Prashant Gupta; Qian Zeng; Rama Ghatti; Scott Parker; Scott Ryan; Scott Sammons; Shatavia Morrison; Stanley Letovsky; Steven Ragan; Suresh Babu Selvaraju; Susan Countryman; Susan Hicks; Suzanne Dale; Thomas Urban; Tim Kuphal; Tricia Zwiefelhofer; Vincent Drouillon; Yvette Unoarumhi
EPI_ISL_1021947	Laboratory Corporation of America	Respiratory Viruses Branch, Division of Viral Diseases, Centers for Disease Control and Prevention	Ben L. Rambo-Martin; Clinton R. Paden; Dakota Howard; Dhwani Batra; Duncan MacCannell; Peter W. Cook; Suxiang Tong
EPI_ISL_754913	Laboratory Diagnostics and Clinical Immunology of Developmental Age, Medical University of Warsaw	genXone SA, Research & Development Laboratory; The Faculty of Mathematics, Informatics and Mechanics of the University of Warsaw	Anna Gambin; Grzegorz Nowicki; Jakub Grabowski; Maciej Sykulski; Michał Kaszuba; Monika Mańkowska-Woźniak; Natalia Drwęska-Matelska; Urszula Demkow; Łukasz Krych
EPI_ISL_2614072	Laboratory of Molecular Virology, Federal University of Rio de Janeito, UFRJ	Laboratory of Respiratory Viruses and Measles, Oswaldo Cruz Institute, FIOCRUZ	Alice Sampaio Rocha; Amilcar Tanuri; Ana Carolina Mendonca; Anna Carolina Paixao; Elisa Cavalcante Pereira; Fernando Motta; Luciana Appolinario; Marilda Siqueira on behalf of the Fiocruz COVID-19 Genomic Surveillance Network; Paola Resende; Renata Serrano Lopes; Taina Venas
EPI_ISL_467356, EPI_ISL_467359, EPI_ISL_467366, EPI_ISL_541354, EPI_ISL_541355, EPI_ISL_541359, EPI_ISL_2443595, EPI_ISL_2443635, EPI_ISL_2443636, EPI_ISL_2557388, EPI_ISL_2557400, EPI_ISL_2603477, EPI_ISL_2603487, EPI_ISL_2603499, EPI_ISL_2603501, EPI_ISL_2603505, EPI_ISL_2603512, EPI_ISL_2603516, EPI_ISL_2603517, EPI_ISL_2603520, EPI_ISL_2614095, EPI_ISL_2614103, EPI_ISL_2614104, EPI_ISL_2614144, EPI_ISL_2614156, EPI_ISL_2614160, EPI_ISL_2614169, EPI_ISL_2614181, EPI_ISL_2677218, EPI_ISL_2731462, EPI_ISL_2731463, EPI_ISL_2731464, EPI_ISL_2731465, EPI_ISL_2731477, EPI_ISL_2731479, EPI_ISL_2731484, EPI_ISL_2731485, EPI_ISL_2731489, EPI_ISL_2731490, EPI_ISL_2731496, EPI_ISL_2731497, EPI_ISL_2731500, EPI_ISL_2731502, EPI_ISL_2731503, EPI_ISL_2731505, EPI_ISL_3190277, EPI_ISL_3190280, EPI_ISL_3190284, EPI_ISL_3243093, EPI_ISL_3434944, EPI_ISL_3434945			
see above	Laboratory of Respiratory Viruses and Measles, Oswaldo Cruz Institute, FIOCRUZ	Laboratory of Respiratory Viruses and Measles, Oswaldo Cruz Institute, FIOCRUZ	Agatha Cristinne Prudencio; Alice Sampaio Rocha; Aline Mattos; Ana Carolina Mendonca; Ana Carolina Mendonça; Anna Carolina Paixao; Anna Carolina Paixão; Braulia Caetano; Cinthia Avila; Cristiana Garcia; Elisa Cavalcante Pereira; Fernando Motta; Ighor Leonardo Arantes Gomes; Jonathan Lopes; Luciana Appolinario; Maria Ogrzewalska; Marilda Siqueira on behalf of the Fiocruz COVID-19 Genomic Surveillance Network; Milene Miranda; Paola Resende; Renata Serrano Lopes; Roxana Loayza; Taina Venas
EPI_ISL_456088	Laboratório Central de Saúde Pública Noel Nutels (LACEN-RJ)	Laboratory of Respiratory Viruses and Measles, Oswaldo Cruz Institute, FIOCRUZ	Aline Mattos; Braulia Caetano; Cristiana Garcia; Fernando Motta; Jonathan Lopes; Luciana Appolinario; Maria Ogrzewalska; Marilda Siqueira on behalf of the Fiocruz COVID-19 Genomic Surveillance Network; Milene Miranda; Paola Resende
EPI_ISL_2241528	Laboratório Central de Saúde Pública da Bahia	Coordenação Geral de Laboratórios de Saúde Pública (CGLAB/DAEVS/SVS/MS)	Vagner Fonseca; et al.
EPI_ISL_2241498, EPI_ISL_2241529,	Laboratório Central de Saúde Pública da	Coordenação Geral de Laboratórios de Saúde	Vagner Fonseca; et al.

EPI_ISL_2241566, EPI_ISL_2241572, EPI_ISL_2241596	Paraiba	Pública (CGLAB/DAEVS/SVS/MS)	
EPI_ISL_2308426, EPI_ISL_2308452, EPI_ISL_2308469	Laboratório Central de Saúde Pública de Alagoas	Coordenação Geral de Laboratórios de Saúde Pública (CGLAB/DAEVS/SVS/MS)	Vagner Fonseca; et al.
EPI_ISL_2298748	Laboratório Central de Saúde Pública de Roraima	Coordenação Geral de Laboratórios de Saúde Pública (CGLAB/DAEVS/SVS/MS)	Vagner Fonseca; et al.
EPI_ISL_2293009	Laboratório Central de Saúde Pública de Santa Catarina	Coordenação Geral de Laboratórios de Saúde Pública (CGLAB/DAEVS/SVS/MS)	Vagner Fonseca; et al.
EPI_ISL_2241557, EPI_ISL_2241609	Laboratório Central de Saúde Pública de Sergipe	Coordenação Geral de Laboratórios de Saúde Pública (CGLAB/DAEVS/SVS/MS)	Vagner Fonseca; et al.
EPI_ISL_2245069	Laboratório Central de Saúde Pública do Amapá	Coordenação Geral de Laboratórios de Saúde Pública (CGLAB/DAEVS/SVS/MS)	Vagner Fonseca; et al.
EPI_ISL_2298865	Laboratório Central de Saúde Pública do Amazonas	Coordenação Geral de Laboratórios de Saúde Pública (CGLAB/DAEVS/SVS/MS)	Vagner Fonseca; et al.
EPI_ISL_1239117	Laboratório Central de Saúde Pública do Espírito Santo	Coordenação Geral de Laboratórios de Saúde Pública (CGLAB)	; Vagner Fonseca et al
EPI_ISL_792605	Laboratório Central de Saúde Pública do Estado da Paraíba (LACEN-PB)	Laboratory of Respiratory Viruses and Measles, Oswaldo Cruz Institute, FIOCRUZ	Ana Carolina Mendonca; Anna Carolina Paixao; Dalane Loudal Florentino Teixeira; Fernando Motta; João Felipe Bezerra; Luciana Appolinario; Marilda Siqueira on behalf of the Fiocruz COVID-19 Genomic Surveillance Network; Paola Resende; Romero Henrique Teixeira de Vasconcelos; Thiago Franco de Oliveira Carneiro
EPI_ISL_427292, EPI_ISL_792643	Laboratório Central de Saúde Pública do Estado de Alagoas (LACEN-AL)	Laboratory of Respiratory Viruses and Measles, Oswaldo Cruz Institute, FIOCRUZ	Aline Mattos; Ana Carolina Mendonca; Anderson Brandao Leite; Anna Carolina Paixao; Braulia Caetano; Cristiana Garcia; Fernando Motta; Jonathan Lopes; Luciana Appolinario; Maria Ogrzewalska; Marilda Siqueira on behalf of the Fiocruz COVID-19 Genomic Surveillance Network; Milene Miranda; Paola Resende; Priscila Born; Sunando Roy
EPI_ISL_500483, EPI_ISL_572371	Laboratório Central de Saúde Pública do Estado de Pernambuco (LACEN-PE)	WallauLab, Aggeu Magalhaes Institute	Alexandre Freitas da Silva; Antonio Mauro Rezende; Armando de Menezes Neto; Bruna Santos Lima Figueiredo de Sá; Caroline Targino Alves da Silva; Claudio Eduardo Cavalcanti; Constância Flávia Junqueira Ayres; Cássia Docena; Derciliano Lopes da Cruz; Duschinka Ribeiro Duarte Guedes; Elisama Helvecio; Filipe Zimmer Dezordi; Gabriel Luz Wallau on behalf of the Fiocruz COVID-19 Genomic Surveillance Network; Gonzalo Bello; Kamila Gaudêncio da Silva Sales; Larissa Krokovsky; Laís Ceschini Machado; Luciane Caroline Albuquerque Bezerra; Luydson Richardson Silva Vasconcelos; Marcelo Henrique Santos Paiva; Maria Almerice Lopes da Silva; Matheus Filgueira Bezerra; Michelle da Silva Barros; Paola Cristina Resende; Renata Pessôa Germano Mendes; Rodrigo Moraes Loyo Arcoverde; Severino Jefferson Ribeiro da Silva; Sinval Pinto Brandão Filho; Tiago Gräf; Wheverton Ricardo Correia do Nascimento
EPI_ISL_541372, EPI_ISL_541386	Laboratório Central de Saúde Pública do Estado de Sergipe (LACEN-SE)	Laboratory of Respiratory Viruses and Measles, Oswaldo Cruz Institute, FIOCRUZ	Ana Carolina Mendonça; Anna Carolina Paixão; Clioma Santos; Fernando Motta; Jonathan Lopes; Luciana Appolinario; Marilda Siqueira on behalf of the Fiocruz COVID-19 Genomic Surveillance Network; Paola Resende
EPI_ISL_801397, EPI_ISL_801398, EPI_ISL_801399, EPI_ISL_801400, EPI_ISL_801401, EPI_ISL_801402, EPI_ISL_801403			
see above	Laboratório Central de Saúde Pública do Estado do Amazonas (LACEN-AM)	Laboratorio de Ecologia de Doencas Transmissiveis na Amazonia, Instituto Leonidas e Maria Deane - Fiocruz Amazonia	André Corado; Debora Duarte; Felipe Naveca on behalf of the Fiocruz COVID-19 Genomic Surveillance Network; Fernanda Nascimento; George Silva; Luciana Gonçalves; Maria Júlia Brandão; Michele Jesus; Valdinete Nascimento; Victor Souza; Ágatha Costa
EPI_ISL_541343, EPI_ISL_541344, EPI_ISL_792647, EPI_ISL_792649, EPI_ISL_792653, EPI_ISL_792654	Laboratório Central de Saúde Pública do Estado do Paraná (LACEN-PR)	Laboratory of Respiratory Viruses and Measles, Oswaldo Cruz Institute, FIOCRUZ	Ana Carolina Mendonca; Ana Carolina Mendonça; Anna Carolina Paixao; Anna Carolina Paixão; Fernando Motta; Irina Nastassja Riediger; Irina Riediger; Jonathan Lopes; Luciana Appolinario; Maria do Carmo Debur; Marilda Siqueira on behalf of the Fiocruz COVID-19 Genomic Surveillance Network; Paola Resende
EPI_ISL_729801, EPI_ISL_729803, EPI_ISL_729805, EPI_ISL_729806, EPI_ISL_729808, EPI_ISL_729813, EPI_ISL_729840, EPI_ISL_729845, EPI_ISL_729852, EPI_ISL_729853, EPI_ISL_729854, EPI_ISL_729856, EPI_ISL_729861			
see above	Laboratório Central de Saúde Pública do Estado do Rio Grande do Sul (LACEN-RS)	Laboratory of Respiratory Viruses and Measles, Oswaldo Cruz Institute, FIOCRUZ	Ana Carolina Mendonça; Anna Carolina Paixão; Fernando Motta; Luciana Appolinario; Marilda Siqueira on behalf of the Fiocruz COVID-19 Genomic Surveillance Network; Marilda Tereza Mar da Rosa; Paola Resende; Tatiana Schaffer Gregianini
EPI_ISL_2248770, EPI_ISL_2298750	Laboratório Central de Saúde Pública do Maranhão	Coordenação Geral de Laboratórios de Saúde Pública (CGLAB/DAEVS/SVS/MS)	Vagner Fonseca; et al.
EPI_ISL_2241593, EPI_ISL_2241607, EPI_ISL_2246287	Laboratório Central de Saúde Pública do Piaui	Coordenação Geral de Laboratórios de Saúde Pública (CGLAB/DAEVS/SVS/MS)	Vagner Fonseca; et al.
EPI_ISL_2241508	Laboratório Central de Saúde Pública do Rio Grande do Norte	Coordenação Geral de Laboratórios de Saúde Pública (CGLAB/DAEVS/SVS/MS)	Vagner Fonseca; et al.
EPI_ISL_1182547, EPI_ISL_1182610, EPI_ISL_1182621, EPI_ISL_1182623, EPI_ISL_2249348, EPI_ISL_2249352, EPI_ISL_2249353, EPI_ISL_2249355, EPI_ISL_2249379, EPI_ISL_2249382			
see above	Laboratório Central de Saúde Pública do Rio Grande do Sul	Coordenação Geral de Laboratórios de Saúde Pública (CGLAB/DAEVS/SVS/MS)	Vagner Fonseca; et al.
EPI_ISL_2249404, EPI_ISL_2249405, EPI_ISL_2249407, EPI_ISL_2249409, EPI_ISL_2249426	Laboratório Central de Saúde Pública do Rio de Janeiro	Coordenação Geral de Laboratórios de Saúde Pública (CGLAB/DAEVS/SVS/MS)	Vagner Fonseca; et al.
EPI_ISL_1182562, EPI_ISL_1182567, EPI_ISL_1182584, EPI_ISL_1182613, EPI_ISL_1182614	Laboratório Central do Estado do Paraná	Coordenação Geral de Laboratórios de Saúde Pública (CGLAB/DAEVS/SVS/MS)	Vagner Fonseca; et al.
EPI_ISL_1182554	Laboratório Central do Estado do Rio de Janeiro	Coordenação Geral de Laboratórios de Saúde Pública (CGLAB/DAEVS/SVS/MS)	Vagner Fonseca; et al.
EPI_ISL_1213309,	Laboratório	Bioinformatics	Alessandra P Lamarca; Alexandra L Gerber; Ana Paula Melo Mariano; Ana Paula de C Guimarães; Ana Tereza R Vasconcelos; Angela Maria Guimarães Santos; Bianca Mendes Maciel; Danielle Angst Secco; Eduardo Sérgio Soares Sousa; Eloiza Helena Campana; Francisco Paulo Freire Neto; George Rego Albuquerque; Kátia Castanho

EPI_ISL_1213397	HLA/UERJ	Laboratory / LNCC	Scortecci; Lucymara Fassarella Agnez Lima; Luiz G P de Almeida; Luís Cristóvão Porto; Otavio J. Brustolini; Paulo Ricardo Nascimento; Ronaldo da Silva Francisco Jr; Sandra Rocha Gadelha; Selma Maria Bezerra Jeronimo; Vinícius Pietta Perez
EPI_ISL_693220, EPI_ISL_693223, EPI_ISL_693224, EPI_ISL_693243	Laboratório Municipal de Piracicaba	Instituto Adolfo Lutz, Interdiciplinary Procedures Center, Strategic Laboratory	Claudia Regina Gonçalves; Claudio Tavares Sacchi; Erica Valessa Ramos Gomes; Karoline Rodrigues Campos
EPI_ISL_940608	Laboratório Sao Lucas	Instituto Adolfo Lutz, Interdiciplinary Procedures Center, Strategic Laboratory	Claudia Regina Gonçalves; Claudio Tavares Sacchi; Erica Valessa Ramos Gomes; Karoline Rodrigues Campos
EPI_ISL_1494970, EPI_ISL_1495004, EPI_ISL_1497548	Laboratório de Biologia Integrativa	Laboratório de Biologia Integrativa	Alessandro Clayton de Souza Ferreira; Aline Brito de Lima; Carolina Moreira Voloch; Daniel Costa Queiroz; Danielle Alves Gomes Zauli; Diego Menezes Bonfim; Filipe Romero Rebello Moreira; Frederico Scott Varella Malta; Joice do Prado Silva; Lucyene Miguita Luiz; Nuno Rodrigues Faria; Paula Luize Camargos Fonseca; Rafael Marques de Souza; Renan Pedra de Souza; Renato Santana Aguiar; Rennan Garcias Moreira; Victor Cavalcanti Pardini; Victor Emmanuel Viana Geddes
EPI_ISL_2466150, EPI_ISL_2466153, EPI_ISL_2466157, EPI_ISL_2466161, EPI_ISL_2466172, EPI_ISL_2466176, EPI_ISL_2466186, EPI_ISL_2466188, EPI_ISL_2466189, EPI_ISL_2466229			
see above	Laboratório de Biologia Molecular de Doenças Infecciosas e do Câncer (LADIC - UFRN)	Laboratory of Respiratory Viruses and Measles, Oswaldo Cruz Institute, FIOCRUZ	Alice Sampaio Rocha; Ana Carolina Mendonca; Anna Carolina Paixao; Elisa Cavalcante Pereira; Fernando Motta; Josélio Araújo; Luciana Appolinario; Marilda Siqueira on behalf of the Fiocruz COVID-19 Genomic Surveillance Network; Paola Resende; Renata Serrano Lopes; Taina Venas
EPI_ISL_770558, EPI_ISL_770562, EPI_ISL_770569, EPI_ISL_770572, EPI_ISL_770576, EPI_ISL_770577, EPI_ISL_770582, EPI_ISL_770585, EPI_ISL_770586, EPI_ISL_770588, EPI_ISL_770590, EPI_ISL_770614, EPI_ISL_770623, EPI_ISL_770626, EPI_ISL_770627, EPI_ISL_770629, EPI_ISL_779156, EPI_ISL_779160, EPI_ISL_779161, EPI_ISL_779163, EPI_ISL_779165, EPI_ISL_779166, EPI_ISL_779167, EPI_ISL_779168			
see above	Laboratório de Microbiologia Molecular - Universidade FEEVALE	Bioinformatics Laboratory / LNCC	Alana Witt Hansen; Alessandra Pavan Lamarca da Silva; Alexandra L Gerber; Ana Karolina Eisen Antunes; Ana Luiza Ziulkoski; Ana Paula de C Guimarães; Ana Tereza R de Vasconcelos; Bruna Hermann; Fagner Henrique Heldt; Felipe Benites; Fernando Rosado Spilki; Juliana Schons; Juliane Deise Fleck; Karoline Schallenberger; Larissa Mallmann; Luiz G P de Almeida; Matheus Nunes Weber; Meriane Demoliner; Paula Rodrigues de Almeida; Ronaldo da Silva F Jr; Vyctoria Goes
EPI_ISL_2918996, EPI_ISL_2918997, EPI_ISL_2918998, EPI_ISL_2918999, EPI_ISL_2919001, EPI_ISL_2919002, EPI_ISL_2919003, EPI_ISL_2919004, EPI_ISL_2919005, EPI_ISL_2919006, EPI_ISL_2919007, EPI_ISL_2919008, EPI_ISL_2919009, EPI_ISL_2919010, EPI_ISL_2919011, EPI_ISL_2919015, EPI_ISL_2919016, EPI_ISL_2925736, EPI_ISL_2928507, EPI_ISL_2928508			
see above	Laboratório de Microbiologia Molecular - Universidade FEEVALE	Laboratório de Microbiologia Molecular - Universidade FEEVALE	Alana Witt Hansen; Fernando Rosado Spilki; Flávio Silveira; Fágner Henrique Heldt; Juliana Schons Gulart; Juliana Schons Gularte; Juliane Deise Fleck; Mariana Soares da Silva; Matheus Nunes Weber; Meriane Demoliner; Michele Filippi.; Micheli Filippi.; Paula Rodrigues de Almeida
EPI_ISL_1799499, EPI_ISL_1799505, EPI_ISL_2431431, EPI_ISL_2928344	Laboratório de Microbiologia Molecular - Universidade FEEVALE	Molecular Microbiology Laboratory	Alana Witt Hansen; Fernando Rosado Spilki; Flávio Silveira; Fágner Henrique Heldt; Juliana Schons Gulart; Juliana Schons Gularte; Juliane Deise Fleck; Mariana Soares da Silva; Matheus Nunes Weber; Meriane Demoliner; Michele Filippi.; Micheli Filippi.; Paula Rodrigues de Almeida
EPI_ISL_831645, EPI_ISL_831660, EPI_ISL_831688, EPI_ISL_831689, EPI_ISL_831938, EPI_ISL_832009, EPI_ISL_832011			
see above	Laboratório de Microbiologia Molecular - Universidade FEEVALE	Universidade Federal de Ciências da Saúde de Porto Alegre	Amanda de Menezes Mayer; Carla Andretta Moreira Neves; Claudia Elizabeth Thompson; Fernando Rosado Spilki; Gabriel Dickin Caldana; Gabriela Bettella Cybis; Lívia Kmetzsch; Patrícia Aline Gröhs Ferrareze; Ricardo Ariel Zimerman; Vinícius Bonetti Franceschi
EPI_ISL_476341, EPI_ISL_476395, EPI_ISL_476398	Laboratório de Patologia Clínica - UNICAMP	Laboratório de Estudos de Vírus Emergentes - UNICAMP	Angelica Schreiber; Camila Simeoni; Darlan da Silva Candido; Jaqueline Goes Jesus e William Marciel de Souza; José Luiz Proença-Modena; Julia Forato; Julien Theze; Luiz Gonzaga; Magnun Nueldo Nunes dos Santos; Marcilio Jorge Fumagalli; Mariene Ribeiro Amorim; Nuno Rodrigues Faria
EPI_ISL_1785610, EPI_ISL_1785612	Laboratório de Pesquisa em Virologia, FAMERP, SJRP	Laboratório de Pesquisa em Virologia, FAMERP, SJRP	Cecília Artico Banho; Cíntia Bittar; Fábio Sossai Possebon; Guilherme Campos; Helena Lage Ferreira; Jorge A. Petroli Marchesi; João Pessoa Araújo Jr.; Leila Sabrina Ullmann; Lívia Sacchetto; Maisa C. Pereira Parra; Marília Moraes; Maurício L. Nogueira; Paula Rahal; Paulo Inacio da Costa
EPI_ISL_1464675, EPI_ISL_1464677	Laboratório de Virologia - UNIFESP	Laboratory of Respiratory Viruses and Measles, Oswaldo Cruz Institute, FIOCRUZ	Alice Sampaio Rocha; Ana Carolina Mendonca; Anna Carolina Paixao; Fernando Motta; Luciana Appolinario; Marilda Siqueira on behalf of the Fiocruz COVID-19 Genomic Surveillance Network; Nancy Belei; Paola Resende; Renata Serrano Lopes
EPI_ISL_2629608, EPI_ISL_2629624, EPI_ISL_2629634	Laboratório de Virologia Molecular - Universidade Federal do Rio de Janeiro	Laboratório de Virologia Molecular - Universidade Federal do Rio de Janeiro	; Alice Laschuk Herlinger; Amilcar Tanuri; André Felipe Andrade dos Santos; Carolina Moreira Voloch; Cássia Cristina Alves Gonçalves; Diana Mariani; Débora Souza Faffe; Filipe Romero Rebello Moreira; Francine Bittencourt Schiffler; Isabela de Carvalho Leitão; Marcelo Calado de Paula Tôrres; Matheus Augusto Calvano Cosentino; Mirela D'arc; Orlando da Costa Ferreira Junior; Rafael Mello Galliez; Raíssa Mirella dos Santos Cunha da Costa; Renato Santana de Aguiar; Terezinha Marta Pereira Pinto Castineiras; Thamiris dos Santos Miranda; Átila Duque Rossi
EPI_ISL_2196357, EPI_ISL_2196360, EPI_ISL_2677096, EPI_ISL_2677099, EPI_ISL_2677126, EPI_ISL_2677234, EPI_ISL_2677244, EPI_ISL_2677289, EPI_ISL_2677291, EPI_ISL_2677292, EPI_ISL_2677299, EPI_ISL_2677300, EPI_ISL_2677307, EPI_ISL_2677308, EPI_ISL_3061901			
see above	Labortorio Central de Saude Publica do Estado de Santa Catarina (LACEN/SC)	Laboratory of Respiratory Viruses and Measles, Oswaldo Cruz Institute, FIOCRUZ	Alice Sampaio Rocha; Ana Carolina Mendonca; Anna Carolina Paixao; Darcita Buerger Rovaris; Elisa Cavalcante Pereira; Fernando Motta; Luciana Appolinario; Marilda Siqueira on behalf of the Fiocruz COVID-19 Genomic Surveillance Network; Paola Resende; Renata Serrano Lopes; Sandra Bianchini Fernandes; Taina Venas
EPI_ISL_2614351	Labortorio Central de Saude Publica do Estado do Rio de Janeiro (LACEN/RJ)	Laboratory of Respiratory Viruses and Measles, Oswaldo Cruz Institute, FIOCRUZ	Alice Sampaio Rocha; Ana Carolina Mendonca; Andrea Cony Cavalcanti; Anna Carolina Paixao; Elisa Cavalcante Pereira; Fernando Motta; Luciana Appolinario; Marilda Siqueira on behalf of the Fiocruz COVID-19 Genomic Surveillance Network; Paola Resende; Renata Serrano Lopes; Taina Venas
EPI_ISL_1358302	Lacen de Tocantins	Instituto Adolfo Lutz, Interdiciplinary Procedures Center, Strategic Laboratory	Caio Vinicius Dias Lopes; Claudia Regina Gonçalves; Claudio Tavares Sacchi; Erica Valessa Ramos Gomes; Karoline Rodrigues Campos
EPI_ISL_2157343, EPI_ISL_2196362	Lboratorio Central de Saude Publica do Estado do Parana (LACEN/PR)	Laboratory of Respiratory Viruses and Measles, Oswaldo Cruz Institute, FIOCRUZ	Alice Sampaio Rocha; Ana Carolina Mendonca; Anna Carolina Paixao; Elisa Cavalcante Pereira; Fernando Motta; Irina Riediger; Luciana Appolinario; Marilda Siqueira on behalf of the Fiocruz COVID-19 Genomic Surveillance Network; Paola Resende; Renata Serrano Lopes; Taina Venas
EPI_ISL_703236, EPI_ISL_730661	Lighthouse Lab in Alderley Park	Wellcome Sanger Institute for the COVID-19 Genomics UK (COG-UK) Consortium	Cordelia Langford; David K. Jackson; Dominic Kwiatkowski; Ewan Harrison; Ian Johnston; Jacquelyn Wynn; John Sillitoe on behalf of the Wellcome Sanger Institute COVID-19 Surveillance Team; Mairead Hyland; Roberto Amato; Sonia Goncalves; The Lighthouse Lab in Alderley Park and Alex Alderton
EPI_ISL_551467	Lighthouse Lab in Alderley Park	Wellcome Sanger Institute for the COVID-19 Genomics UK (COG-UK) consortium	Cordelia Langford; David K. Jackson; Dominic Kwiatkowski; Ewan Harrison; Ian Johnston; John Sillitoe on behalf of the Wellcome Sanger Institute COVID-19 Surveillance Team (http://www.sanger.ac.uk/covid-team); Roberto Amato; Sonia Goncalves; The Lighthouse Lab in Alderley Park and Alex Alderton
EPI_ISL_673528, EPI_ISL_857679	Lighthouse Lab in Cambridge	Wellcome Sanger Institute for the COVID-19 Genomics UK (COG-UK) Consortium	Cordelia Langford; David K. Jackson; Dominic Kwiatkowski; Ewan Harrison; Ian Johnston; John Sillitoe on behalf of the Wellcome Sanger Institute COVID-19 Surveillance Team; Rob Howes; Roberto Amato; Sonia Goncalves; The Lighthouse Lab in Cambridge and Alex Alderton
EPI_ISL_590506	Lighthouse Lab in Glasgow	Wellcome Sanger Institute for the COVID-19 Genomics UK (COG-UK) consortium	Anna Dominiczak and Alex Alderton; Carol Clugston; Cordelia Langford; David Gray; David K. Jackson; Dominic Kwiatkowski; Ewan Harrison; Harper VanSteenhouse; Ian Johnston; John Sillitoe on behalf of the Wellcome Sanger Institute COVID-19 Surveillance Team (http://www.sanger.ac.uk/covid-team); Roberto Amato; Sonia Goncalves; Yumi Kasai
EPI_ISL_760881, EPI_ISL_760963, EPI_ISL_3954939	Lighthouse Lab in Milton Keynes	Wellcome Sanger Institute for the COVID-19 Genomics UK (COG-UK) Consortium	Cordelia Langford; David K. Jackson; Dominic Kwiatkowski; Ewan Harrison; Ian Johnston; Jeffrey Barrett; John Sillitoe on behalf of the Wellcome Sanger Institute COVID-19 Surveillance Team; Roberto Amato; Sonia Goncalves; The Lighthouse Lab in Milton Keynes and Alex Alderton
EPI_ISL_549833, EPI_ISL_629164,	Lighthouse Lab in Milton Keynes	Wellcome Sanger Institute for the COVID-	Cordelia Langford; David K. Jackson; Dominic Kwiatkowski; Ewan Harrison; Ian Johnston; John Sillitoe on behalf of the Wellcome Sanger Institute COVID-19 Surveillance Team; John Sillitoe on behalf of the Wellcome Sanger Institute COVID-19 Surveillance Team (http://www.sanger.ac.uk/covid-team); Roberto Amato; Sonia Goncalves; The Lighthouse Lab in Milton Keynes and Alex Alderton

EPI_ISL_630998, EPI_ISL_631036		19 Genomics UK (COG-UK) consortium	
EPI_ISL_1227371, EPI_ISL_1227396, EPI_ISL_1227400, EPI_ISL_1227427, EPI_ISL_1227445, EPI_ISL_1227446, EPI_ISL_1227933, EPI_ISL_1228176, EPI_ISL_1228572, EPI_ISL_1397621, EPI_ISL_1623976, EPI_ISL_2278731			
see above	MONTEFIORE MEDICAL CENTER LABORATORIES	Wadsworth Center, New York State Department of Health	Alexis Russel; Alexis Russell; Catharine Prussing; Daryl M. Lamson; Erasmus Schneider; Erica Lasek-Nesselquist; John Kelly; Jonathan Plitnick; Kirsten St. George; Matthew Shudt; Melissa A Leisner; Navjot Singh
EPI_ISL_802105, EPI_ISL_802106, EPI_ISL_1300937, EPI_ISL_1301233, EPI_ISL_1301238, EPI_ISL_1709347, EPI_ISL_1709917			
see above	MSHS Clinical Microbiology Laboratories	MSHS Pathogen Surveillance Program	Adolfo García-Sastre; Adriana van de Guchte; Ajay Obla; Alberto Paniz-Mondolfi; Ana S. Gonzalez-Reiche; Andrew Kasarskis; Angela Amoako; Ashley S. Salimbangon; Ashley Salimbangon; Betsaida Salom Melo; Bremy Alburquerque; Brianne Ciferri; Charles Gleason; Daniel Floda; Deena R. Altman; Denise Jurczyszak; Elena Hirsch; Emilia Mia Sordillo; Emily Ferreri; Gintaras Deikus; Giulio Kleiner; Gopi Patel; Hala Alshammary; Harm van Bakel; Irina Oussenko; Jayeeta Dutta; Juan Soto; Julia Matthews; Katherine Beach; Kathryn Twyman; Kayla Russo; Komal Srivastava; Levy Sominsky; Mahmoud Awawda; Marta Luksza; Matthew M. Hernandez; Melissa Gitman; Michael D. Nowak; Mitchell J. Sullivan; Nancy Francoeur; Rachel Chernet; Robert Sebra; Sarah Schaefer; Shelcie Fabre; Shwetha Hara Sridhar; Viviana Simon; Ying-Chih Wang; Zenab Khan
EPI_ISL_2691009	MSK Microbiology Lab	MSK Microbiology Lab	Esther Babady; Krupa Jani; Tracy McMillen
EPI_ISL_1152591	MVZ Medizinisches Labor Hannover GmbH	Robert Koch Institute	
EPI_ISL_1719809	Microbiology Department, Laboratori Clínic Metropolitana Nord. Hospital Universitari Germans Trias i Pujol.	Can Ruti SARS-CoV-2 Sequencing Hub (HUGTiP/IrsiCaixa/IGTP)	Alba Sánchez; Anna Not; Antoni E Bordoy; Bonaventura Clotet; Cristina Casañ; Cristina Esteban; Francesc Catala-Moll; Gemma Clara; Ignacio Blanco; Marc Noguera-Julian; Maria Casadellà; Mariona Parera; Mercedes Guerrero; Montserrat Giménez; Pere-Joan Cardona; Pilar Armengol; Roger Paredes; Verónica Saludes; and Elisa Martró on behalf of the Can Ruti SARS-CoV-2 Sequencing Hub.
EPI_ISL_547433, EPI_ISL_547434, EPI_ISL_547435, EPI_ISL_547437	Microbiology, Department of Pathology, St. Bernard's Hospital, Gibraltar Health Authority	Respiratory Virus Unit, Microbiology Services Colindale, Public Health England	Charlotte Gillborn-Jones (Gibraltar); Dr Nicholas Cortes (Gibraltar); PHE Covid Sequencing Team
EPI_ISL_735413	Militello Centro de Diagnosticos e Biopesquisa Clinica	Instituto Adolfo Lutz, Interdiciplinary Procedures Center, Strategic Laboratory	Claudia Regina Gonçalves; Claudio Tavares Sacchi; Erica Valessa Ramos Gomes; Karoline Rodrigues Campos
EPI_ISL_1534512, EPI_ISL_1911798, EPI_ISL_3256939, EPI_ISL_3406510, EPI_ISL_3406753, EPI_ISL_3414403, EPI_ISL_3414677			
see above	Ministry of Health Turkey	Ministry of Health Turkey	Fatma Bayrakdar; Gulay Korukluoglu; Gülay Korukluoğlu; Suleyman Yalcin; Süleyman Yalcin; Yasemin Cosgun; Yasemin Coşgun
EPI_ISL_1714420, EPI_ISL_1714447, EPI_ISL_1714459, EPI_ISL_1714483, EPI_ISL_1714533, EPI_ISL_1714591, EPI_ISL_1714616, EPI_ISL_1714620			
see above	Ministry of Public Health / Hamad Medical Corporation	Weill Cornell Medical College - Qatar (WCM-Q), Genomics Core Laboratory / Qatar Genome Project (QGP)	Chadi Saad MOPH and HMC: Abdullatif Al-Khal; Dina Elgakhlab; Einas A. E. Al-Kuwari; Hamad E. Al-Romaihi; Hamda Alromaihi; Joel A Malek. QGP: Fatima H. Al-Kuwari; Laith Abu-Raddad; Mashael A. Al-Bader; Meryem Bensaad; Mohammed Al-Thani; Muna A. S. Al-Maslamani; Peter V. Coyle; Reham A. El-Kahlout. QBB: Tasneem Al-Hamad; Roberto Bertollini; Salih Al-Marri; Shameem Younuskunju; WCMQ: Ayeda A. Ahmed; Yasmin Mohamoud
EPI_ISL_1597713, EPI_ISL_1597714	Montefiore Medical Center	Abbott	Amy Fox; Ana Olivo; Ana Vallari; Barbara Harris; Gavin Cloherty; Mary Rodgers; Todd Meyer; Yitz Goldstein
EPI_ISL_1017531	Murphy Medical Associates	Grubaugh Lab - Yale School of Public Health	Annie Watkins; Caleb Neal; Chantal Vogels; Joseph Fauver; Mallery Breban; Mary Petrone; Nathan Grubaugh; Steven Murphy; Tara Alpert
EPI_ISL_1966563	NUCLEO DE SAUDE VILA FALCAO DE BAURU	Instituto Butantan / Mendelics	Antonio Jorge Martins; Bianca Cechetto Carlos. Mendelics: Bibiana Santos; Claudia Renata dos Santos Barros; Cíntia Bittar; David Schlesinger. Hemocentro Ribeirão Preto: Simone Kashima; Debora Botequio Moretti; Elaine Cristina Marqueze; Elaine Vieira dos Santos; Elisangela Chicaroni Mattos; Erika Freitas; Evandra Strazza Rodrigues; Felipe Allan da Silva da Costa; Flavia Aburjaile; Fábio Sossai Possebon; Guilherme Campos; Guilherme Targino Valente; Heidge Fukumasu. USP-Botucatu: Rejane Maria Tommasini Grotto; Helena Lage Ferreira; Instituto Butantan: Dimas Tadeu Covas; Jardelina de Souza Todao Bernardino; Jayme A. Souza-Neto; Jessika Cristina Chagas Lesbon; Jorge A. Petroli Marchesi; José Salvatore Leister Patané; João Paulo Kitajima; João Pessoa Araújo Jr.; Leila Sabrina Ullmann; Loyze Paola Oliveira de Lima; Luiz Aurelio de Campos Crispin. Centro de Genômica Funcional da ESALQ: Luiz Lehmann Coutinho; Luiz Carlos Junior de Alcantara; Lívia Sacchetto; Maisa C. Pereira Parra; Maria Carolina Elias; Marta Giovanetti; Marília Moraes; Maurício Lacerda Nogueira. Prefeitura de Sao Paulo: Melissa Palmieri.; Patricia Akemi Assato; Paula Rahal; Paulo Inacio da Costa; Rafael dos Santos Bezerra; Raquel de Lello Rocha Campos Cassano. NGS Soluções Genômicas: Pilar Drummond Sampaio Corrêa Mariani. FZEA-USP Pirassununga: Mirele Daiana Poleti; Raul Machado Neto; Ricardo Augusto Brassaloti; Ricardo Haddad; Rodrigo Tocantins Calado. FAMERP-SJRP: Cecília Artico Banho; Sandra Coccuzzo Sampaio; Svetoslav Nanev Slavov; Vagner Fonseca; Vincent Louis Viala
EPI_ISL_3046174, EPI_ISL_3046188, EPI_ISL_3046190, EPI_ISL_3046193, EPI_ISL_3046196, EPI_ISL_3046199, EPI_ISL_3046200, EPI_ISL_3046201, EPI_ISL_3046204, EPI_ISL_3046207, EPI_ISL_3046214, EPI_ISL_3046216, EPI_ISL_3046219, EPI_ISL_3046228, EPI_ISL_3046230, EPI_ISL_3046232, EPI_ISL_3046238, EPI_ISL_3046239, EPI_ISL_3046244, EPI_ISL_3046245, EPI_ISL_3046260, EPI_ISL_3046262, EPI_ISL_3060269, EPI_ISL_3060271, EPI_ISL_3060272, EPI_ISL_3060273, EPI_ISL_3134727, EPI_ISL_3134738, EPI_ISL_3134741, EPI_ISL_3134742, EPI_ISL_3134743, EPI_ISL_3134744, EPI_ISL_3134748, EPI_ISL_3134751, EPI_ISL_3134819, EPI_ISL_3134821, EPI_ISL_3134822, EPI_ISL_3134827, EPI_ISL_3134828, EPI_ISL_3134829, EPI_ISL_3703673			
see above	NUPIT/UFPE	WallauLab on behalf of Fiocruz COVID-19 Genomic Surveillance Network	Alexandre Freitas da Silva; Cassia Docena; Constância Flávia Junqueira Ayres; Filipe Zimmer Dezordi; Gabriel Luz Wallau; Gustavo Barbosa de Lima; Lais Ceschini Machado; Lilian Caroliny Amorim Silva; Maira Galdino da Rocha Pitta; Marcelo Henrique dos Santos Paiva; Matheus Filgueira Bezerra; Michelly Cristiny Pereira; Rômulo Pessoa e Silva; Sinval Pinto Brandão Filho
EPI_ISL_1227698	NYC Pandemic Response Lab	Wadsworth Center, New York State Department of Health	Alexis Russel; Daryl M. Lamson; Erasmus Schneider; Erica Lasek-Nesselquist; John Kelly; Jonathan Plitnick; Kirsten St. George; Matthew Shudt; Melissa A Leisner; Navjot Singh
EPI_ISL_1379204	NYP-WCM	New York Genome Center	Andre Corvelo; Arryn Craney; Chris Mason; Dayna M. Oschwald; Hanna Rennert; Lars F Westblade; Margaret Elizabeth Ross; Melissa Cushing; Michael Zody; Olivier Elemento; Priya Velu; Samantha Fennessey; Tom Maniatis
EPI_ISL_1415196	National Centre For Cell Science	National Centre For Cell Science – INSACOG	Ajay Pillai; Dhiraj Paul; INSACOG Consortium team; Manoj Kumar Bhat; Mitali Inamdar; Mohak P Gujare; Shivang P. Bhanushali; Sonal Manik Chavan; Yogesh Shouche.
EPI_ISL_416036	National Influenza Center - Instituto Adolfo Lutz	Instituto Adolfo Lutz, Interdiciplinary Procedures Center, Strategic Laboratory	Adriana Bugno; Adriano Abbud; Carlos Henrique Camargo; Claudia Regina Gonçalves; Claudio Tavares Sacchi; Daniela Bernardes Borges da Silva; Erica Valessa Ramos Gomes; Fabiana Cristina Pereira dos Santos; Maria do Carmo Sampaio Tavares Timenetsky; Simone Guadagnucci Morillo; Terezinha Maria de Paiva
EPI_ISL_1447440	National Institute of Infectious Diseases (NIID)	National Institute of Infectious Diseases (NIID)	Hideka Miura; Kentaro Itokawa; Kiyoko Okamoto; Kumiko Araki; Makoto Kuroda; Masanori Hashino; Rina Tanaka; Seiichiro Fujisaki; Shinichiro Hirai; Tsuyoshi Sekizuka
EPI_ISL_728187	National Public Health Laboratory, National Centre for Infectious Diseases	National Public Health Laboratory, National Centre for Infectious Diseases	Lin Cui; Raymond Tzer Pin Lin; Sophie Octavia; Tze Minn Mak; Zhenyang Zhou
EPI_ISL_875351	National Virus Reference Laboratory	National Virus Reference Laboratory	Cillian F De Gascun; Gabriel Gonzalez; Jonathan Dean; Michael Carr
EPI_ISL_2105404, EPI_ISL_2105410, EPI_ISL_2105412, EPI_ISL_2105416, EPI_ISL_2105419, EPI_ISL_2105422, EPI_ISL_2105423, EPI_ISL_2105427, EPI_ISL_2105431, EPI_ISL_2105435, EPI_ISL_2105439, EPI_ISL_2105444, EPI_ISL_2105450, EPI_ISL_2105451, EPI_ISL_2105452, EPI_ISL_2105460			
see above	Negros Oriental Provincial Hospital	Philippine Genome Center	Alethea R. de Guzman; Anna Ong-Lim; Arianne A. Zamora; Asia Louisa U. Chong; Benedict A. Maralit; Candice Francheska B. Tambaoan; Carlo M. Lapid; Celia Carlos; Devon Ray Pacial; Edsel Maurice Salvaña; El King D. Morado; Eva Maria Cutiongco-de la Paz; Francis A. Tablizo; Irish Coleen A. Asin; Jaime C. Montoya; Jan Michael C. Yap; Jo-Hannah S. Llames; John Q. Wong; Joshua Gregor A. Dizon; Juan Antonio R. Magalang; Karol Sophia Agape R. Padilla; Kenneth M. Kim; Kris P. Punayan; Marc Edsel C. Ayes; Marc Jerrone R. Castro; Maria Rosario Singh-Vergeire and Cynthia P. Saloma; Maria Sofia L. Yangzon; Marissa Alejandria; Razel Nikka M. Hao; Rianna Patricia S. Cruz; Sheila Mae M. Araiza
EPI_ISL_896351, EPI_ISL_1016306, EPI_ISL_1397496, EPI_ISL_1397501, EPI_ISL_1624013, EPI_ISL_1624022, EPI_ISL_1624056, EPI_ISL_1624076, EPI_ISL_1624081			
see above	New York Presbyterian Hospital	Wadsworth Center, New York State Department of Health	Alexis Russel; Alexis Russell; Catharine Prussing; Daryl M. Lamson; Erasmus Schneider; Erica Lasek-Nesselquist; John Kelly; Jonathan Plitnick; Kirsten St. George; Matthew Shudt; Melissa A Leisner; Navjot Singh
EPI_ISL_2105401, EPI_ISL_2105556, EPI_ISL_2156485, EPI_ISL_2156584, EPI_ISL_2156591, EPI_ISL_2156592, EPI_ISL_2156593, EPI_ISL_2189279			
see above	Northern Mindanao TB Regional Center	Philippine Genome Center	Alethea R. de Guzman; Anna Ong-Lim; Arianne A. Zamora; Asia Louisa U. Chong; Benedict A. Maralit; Candice Francheska B. Tambaoan; Carlo M. Lapid; Celia Carlos; Devon Ray Pacial; Edsel Maurice Salvaña; El King D. Morado; Elcid Aaron R. Pangilinan; Eva Maria Cutiongco-de la Paz; Francis A. Tablizo; Irish Coleen A. Asin; Jaime C. Montoya; Jan Michael C. Yap; Jo-Hannah S. Llames; John Q. Wong; Joshua Gregor A. Dizon; Juan Antonio R. Magalang; Karol Sophia Agape R. Padilla; Kenneth M. Kim; Kris P. Punayan; Marc Edsel C. Ayes; Marc Jerrone R. Castro; Maria Rosario Singh-Vergeire and Cynthia P. Saloma; Maria Sofia L. Yangzon; Marissa Alejandria; Razel Nikka M. Hao; Renato Jacinto Q. Mantaring; Rianna Patricia S. Cruz; Sheila Mae M. Araiza
EPI_ISL_534326	Notre Dame Intermedica Saude AS	Instituto Adolfo Lutz, Interdiciplinary Procedures Center, Strategic Laboratory	Claudia Regina Gonçalves; Claudio Tavares Sacchi; Erica Valessa Ramos Gomes
EPI_ISL_1117384, EPI_ISL_1117388,	Nucleo de Pesquisa em Inovacao	LABBE, Federal University of	Bruno Sampaio; Heidi Lacerda Alves da Cruz; Maira Galdino da Rocha Pitta; Marco Katzenberger; Marcos da Silveira Regueira Neto; Michelly Cristiny Pereira; Reginaldo Goncalves de Lima Neto; Valdir de Queiroz Balbino; Wilson Jose da Silva Junior

EPI_ISL_1117408, EPI_ISL_1117429	Terapeutica - UFPE	Pernambuco	
EPI_ISL_534316	OS Mun Santana Lauro Ribas Braga	Instituto Adolfo Lutz, Interdiciplinary Procedures Center, Strategic Laboratory	Claudia Regina Gonçalves; Claudio Tavares Sacchi; Erica Valessa Ramos Gomes
EPI_ISL_1131143, EPI_ISL_1430700	Osaka Institute of Public Health, Morinomiya Center	Pathogen Genomics Center, National Institute of Infectious Diseases	Kentaro Itokawa; Makoto Kuroda; Masanori Hashino; Rina Tanaka; Tsuyoshi Sekizuka
EPI_ISL_2171170	Ospital ng ParaÃ±aque II	Philippine Genome Center	Alethea R. de Guzman; Anna Ong-Lim; Arianne A. Zamora; Asia Louisa U. Chong; Benedict A. Maralit; Candice Francheska B. Tambaoan; Carlo M. Lapid; Celia Carlos; Devon Ray Pacial; Edsel Maurice Salvaña; El King D. Morado; Elcid Aaron R. Pangilinan; Eva Maria Cutiongco-de la Paz; Francis A. Tablizo; Irish Coleen A. Asin; Jaime C. Montoya; Jan Michael C. Yap; Jo-Hannah S. Llames; John Q. Wong; Joshua Gregor A. Dizon; Juan Antonio R. Magalang; Karol Sophia Agape R. Padilla; Kenneth M. Kim; Kris P. Punayan; Marc Edsel C. Ayes; Marc Jerrone R. Castro; Maria Rosario Singh-Vergeire and Cynthia P. Saloma; Maria Sofia L. Yangzon; Marissa Alejandria; Razel Nikka M. Hao; Rianna Patricia S. Cruz; Sheila Mae M. Araiza
EPI_ISL_2661879, EPI_ISL_2661903	Oswaldo Cruz Institute, FIOCRUZ/CE	Analytical Competence Molecular Epidemiology Lab/ACME, Oswaldo Cruz Foundation, Ceara (FIOCRUZ CE)	Alice Sampaio Rocha; Ana Carolina Mendonca; Anna Carolina Paixao; Elisa Cavalcante Pereira; Fabio Miyajima; Fernando Motta; Luciana Appolinario; Marilda Siqueira on behalf of the Fiocruz COVID-19 Genomic Surveillance Network; Paola Resende; Renata Serrano Lopes; Taina Venas
EPI_ISL_638782, EPI_ISL_651545, EPI_ISL_652142	Oxford Viromics, NDM, University of Oxford; Oxford University Hospitals; Basingstoke and North Hampshire Hospital	COVID-19 Genomics UK (COG-UK) Consortium	Alex Mobbs; Amy Trebes; Anita Justice; Catrin Moore; Christophe Fraser; David Bonsall; David Buck; Emma Wise; George Macintyre; Jessica Lynch; John Todd; Mariateresa de Cesare; Matilde Mori; Monique Andersson; Nathan Moore; Nick Cortes; Robert Shaw; Stephen Kidd; Tanya Golubchik; Timothy Peto
EPI_ISL_861673	PA Novo Osasco	Instituto Adolfo Lutz, Interdiciplinary Procedures Center, Strategic Laboratory	Claudia Regina Gonçalves; Claudio Tavares Sacchi; Erica Valessa Ramos Gomes; Karoline Rodrigues Campos
EPI_ISL_2156328	PHILIPPINE AIRPORT DIAGNOSTIC LABORATORY	Philippine Genome Center	Alethea R. de Guzman; Anna Ong-Lim; Arianne A. Zamora; Asia Louisa U. Chong; Benedict A. Maralit; Candice Francheska B. Tambaoan; Carlo M. Lapid; Celia Carlos; Devon Ray Pacial; Edsel Maurice Salvaña; El King D. Morado; Elcid Aaron R. Pangilinan; Eva Maria Cutiongco-de la Paz; Francis A. Tablizo; Irish Coleen A. Asin; Jaime C. Montoya; Jan Michael C. Yap; Jo-Hannah S. Llames; John Q. Wong; Joshua Gregor A. Dizon; Juan Antonio R. Magalang; Karol Sophia Agape R. Padilla; Kenneth M. Kim; Kris P. Punayan; Marc Edsel C. Ayes; Marc Jerrone R. Castro; Maria Rosario Singh-Vergeire and Cynthia P. Saloma; Maria Sofia L. Yangzon; Marissa Alejandria; Razel Nikka M. Hao; Rianna Patricia S. Cruz; Sheila Mae M. Araiza
EPI_ISL_1445090, EPI_ISL_1966104	POLICLINICA COVID 19 ITAPETININGA	Instituto Butantan / Mendelics	Antonio Jorge Martins; Bianca Cechetto Carlos. Mendelics: Bibiana Santos; Bibiana Santos; Claudia Renata dos Santos Barros; Cíntia Bittar; David Schlesinger; David Schlesinger. Hemocentro Ribeirão Preto: Simone Kashima; Debora Botequio Moretti; Dimas Tadeu Covas; Elaine Cristina Marqueze; Elaine Vieira dos Santos; Elisangela Chicaroni Mattos; Erika Freitas; Evandra Strazza Rodrigues; Felipe Allan da Silva da Costa; Flavia Aburjaile; Fábio Sossai Possebon; Guilherme Campos; Guilherme Targino Valente; Heidge Fukumasu. USP-Botucatu: Rejane Maria Tommasini Grotto; Helena Lage Ferreira; Instituto Butantan: Dimas Tadeu Covas; Jardelina de Souza Todao Bernardino; Jayme A. Souza-Neto; Jessika Cristina Chagas Lesbon; Jorge A. Petroli Marchesi; José Salvatore Leister Patané; João Paulo Kitajima; João Pessoa Araújo Jr.; Leila Sabrina Ullmann; Loyze Paola Oliveira de Lima; Luiz Aurelio de Campos Crispin. Centro de Genômica Funcional da ESALQ: Luiz Lehmann Coutinho; Luiz Carlos Junior de Alcantara; Lívia Sacchetto; Maisa C. Pereira Parra; Maria Carolina Elias; Marta Giovanetti; Marília Moraes; Maurício Lacerda Nogueira. Prefeitura de Sao Paulo: Melissa Palmieri.; Patricia Akemi Assato; Paula Rahal; Paulo Inacio da Costa; Rafael dos Santos Bezerra; Raquel de Lello Rocha Campos Cassano. NGS Soluções Genômicas: Pilar Drummond Sampaio Corrêa Mariani. FZEA-USP Pirassununga: Mirele Daiana Poleti; Raul Machado Neto; Ricardo Augusto Brassaloti; Ricardo Haddad; Rodrigo Tocantins Calado.; Rodrigo Tocantins Calado. FAMERP-SJRP: Cecília Artico Banho; Sandra Coccuzzo Sampaio; Simone Kashima; Svetoslav Nanev Slavov; Vagner Fonseca; Vincent Louis Viala
EPI_ISL_1795399, EPI_ISL_2345349	POLICLINICA HORTOLANDIA	Instituto Butantan / ESALQ-Piracicaba	Antonio Jorge Martins; Bianca Cechetto Carlos. Mendelics: Bibiana Santos; Claudia Renata dos Santos Barros; David Schlesinger; David Schlesinger. Hemocentro Ribeirão Preto: Simone Kashima; Debora Botequio Moretti; Debora Botequio Moretti. Centro de Genômica Funcional da ESALQ: Luiz Lehmann Coutinho; Dimas Tadeu Covas; Elaine Cristina Marqueze; Elaine Vieira Santos; Elaine Vieira dos Santos; Elisangela Chicaroni Mattos; Erika Freitas; Evandra Strazza Rodrigues; Felipe Allan da Silva da Costa; Flavia Aburjaile; Guilherme Targino Valente; Heidge Fukumasu; Heidge Fukumasu. USP-Botucatu: Rejane Maria Tommasini Grotto; Instituto Butantan: Alexander Roberto Precioso; Jayme A. Souza-Neto; Jayme Augusto de Souza-Neto; Jessika Cristina Chagas Lesbon; José Salvatore Leister Patané; João Paulo Kitajima; Luiz Alcantara; Luiz Carlos Junior de Alcantara; Luiz Lehmann Coutinho; Maria Carolina Elias; Marta Giovanetti; Maurício Lacerda Nogueira; Patricia Akemi Assato; Rafael dos Santos Bezerra; Raquel de Lello Rocha Campos Cassano. NGS Soluções Genômicas: Pilar Drummond Sampaio Corrêa Mariani. FZEA-USP Pirassununga: Mirele Daiana Poleti; Raul Machado Neto; Rejane Maria Tommasini Grotto; Ricardo Augusto Brassaloti; Ricardo Haddad; Rodrigo Tocantins Calado.; Sandra Coccuzzo Sampaio; Sandra Coccuzzo Sampaio Vessoni; Simone Kashima; Svetoslav Nanev Slavov; Vagner Fonseca; Vincent Louis Viala
EPI_ISL_1966691	PRONTO ATENDIMENTO MUNICIPAL DE JACUPIRANGA	Instituto Butantan / Mendelics	Antonio Jorge Martins; Bianca Cechetto Carlos. Mendelics: Bibiana Santos; Claudia Renata dos Santos Barros; Cíntia Bittar; David Schlesinger. Hemocentro Ribeirão Preto: Simone Kashima; Debora Botequio Moretti; Elaine Cristina Marqueze; Elaine Vieira dos Santos; Elisangela Chicaroni Mattos; Erika Freitas; Evandra Strazza Rodrigues; Felipe Allan da Silva da Costa; Flavia Aburjaile; Fábio Sossai Possebon; Guilherme Campos; Guilherme Targino Valente; Heidge Fukumasu. USP-Botucatu: Rejane Maria Tommasini Grotto; Helena Lage Ferreira; Instituto Butantan: Dimas Tadeu Covas; Jardelina de Souza Todao Bernardino; Jayme A. Souza-Neto; Jessika Cristina Chagas Lesbon; Jorge A. Petroli Marchesi; José Salvatore Leister Patané; João Paulo Kitajima; João Pessoa Araújo Jr.; Leila Sabrina Ullmann; Loyze Paola Oliveira de Lima; Luiz Aurelio de Campos Crispin. Centro de Genômica Funcional da ESALQ: Luiz Lehmann Coutinho; Luiz Carlos Junior de Alcantara; Lívia Sacchetto; Maisa C. Pereira Parra; Maria Carolina Elias; Marta Giovanetti; Marília Moraes; Maurício Lacerda Nogueira. Prefeitura de Sao Paulo: Melissa Palmieri.; Patricia Akemi Assato; Paula Rahal; Paulo Inacio da Costa; Rafael dos Santos Bezerra; Raquel de Lello Rocha Campos Cassano. NGS Soluções Genômicas: Pilar Drummond Sampaio Corrêa Mariani. FZEA-USP Pirassununga: Mirele Daiana Poleti; Raul Machado Neto; Ricardo Augusto Brassaloti; Ricardo Haddad; Rodrigo Tocantins Calado. FAMERP-SJRP: Cecília Artico Banho; Sandra Coccuzzo Sampaio; Svetoslav Nanev Slavov; Vagner Fonseca; Vincent Louis Viala
EPI_ISL_2209413	PRONTO ATENDIMENTO MUNICIPAL ITALO SANTUCCI	Instituto Butantan	Antonio Jorge Martins; Claudia Renata dos Santos Barros; David Schlesinger; Debora Botequio Moretti; Dimas Tadeu Covas; Elaine Cristina Marqueze; Elaine Vieira Santos; Evandra Strazza Rodrigues; Heidge Fukumasu; Jayme Augusto de Souza-Neto; José Salvatore Leister Patané; Luiz Alcantara; Luiz Lehmann Coutinho; Maria Carolina Elias; Maurício Lacerda Nogueira; Rafael dos Santos Bezerra; Raul Machado Neto; Rejane Maria Tommasini Grotto; Ricardo Haddad; Sandra Coccuzzo Sampaio Vessoni; Simone Kashima; Svetoslav Nanev Slavov; Vincent Louis Viala.
EPI_ISL_2344592	PRONTO ATENDIMENTO MUNICIPAL ITALO SANTUCCI	Instituto Butantan / UNESP-Botucatu	Antonio Jorge Martins; Claudia Renata dos Santos Barros; David Schlesinger; Debora Botequio Moretti; Dimas Tadeu Covas; Elaine Cristina Marqueze; Elaine Vieira Santos; Evandra Strazza Rodrigues; Heidge Fukumasu; Jayme Augusto de Souza-Neto; José Salvatore Leister Patané; Luiz Alcantara; Luiz Lehmann Coutinho; Maria Carolina Elias; Maurício Lacerda Nogueira; Rafael dos Santos Bezerra; Raul Machado Neto; Rejane Maria Tommasini Grotto; Ricardo Haddad; Sandra Coccuzzo Sampaio Vessoni; Simone Kashima; Svetoslav Nanev Slavov; Vincent Louis Viala
EPI_ISL_2209422	PRONTO SOCORRO MUNICIPAL DE TAUBATE	Instituto Butantan	Antonio Jorge Martins; Claudia Renata dos Santos Barros; David Schlesinger; Debora Botequio Moretti; Dimas Tadeu Covas; Elaine Cristina Marqueze; Elaine Vieira Santos; Evandra Strazza Rodrigues; Heidge Fukumasu; Jayme Augusto de Souza-Neto; José Salvatore Leister Patané; Luiz Alcantara; Luiz Lehmann Coutinho; Maria Carolina Elias; Maurício Lacerda Nogueira; Rafael dos Santos Bezerra; Raul Machado Neto; Rejane Maria Tommasini Grotto; Ricardo Haddad; Sandra Coccuzzo Sampaio Vessoni; Simone Kashima; Svetoslav Nanev Slavov; Vincent Louis Viala.
EPI_ISL_2344686	PRONTO SOCORRO MUNICIPAL DE TAUBATE	Instituto Butantan / UNESP-Botucatu	Antonio Jorge Martins; Claudia Renata dos Santos Barros; David Schlesinger; Debora Botequio Moretti; Dimas Tadeu Covas; Elaine Cristina Marqueze; Elaine Vieira Santos; Evandra Strazza Rodrigues; Heidge Fukumasu; Jayme Augusto de Souza-Neto; José Salvatore Leister Patané; Luiz Alcantara; Luiz Lehmann Coutinho; Maria Carolina Elias; Maurício Lacerda Nogueira; Rafael dos Santos Bezerra; Raul Machado Neto; Rejane Maria Tommasini Grotto; Ricardo Haddad; Sandra Coccuzzo Sampaio Vessoni; Simone Kashima; Svetoslav Nanev Slavov; Vincent Louis Viala
EPI_ISL_2105557	PROVIDERS MULTI-PURPOSE COOPERATIVE MEDICAL CENTER	Philippine Genome Center	Alethea R. de Guzman; Anna Ong-Lim; Arianne A. Zamora; Asia Louisa U. Chong; Benedict A. Maralit; Candice Francheska B. Tambaoan; Carlo M. Lapid; Celia Carlos; Devon Ray Pacial; Edsel Maurice Salvaña; El King D. Morado; Eva Maria Cutiongco-de la Paz; Francis A. Tablizo; Irish Coleen A. Asin; Jaime C. Montoya; Jan Michael C. Yap; Jo-Hannah S. Llames; John Q. Wong; Joshua Gregor A. Dizon; Juan Antonio R. Magalang; Karol Sophia Agape R. Padilla; Kenneth M. Kim; Kris P. Punayan; Marc Edsel C. Ayes; Marc Jerrone R. Castro; Maria Rosario Singh-Vergeire and Cynthia P. Saloma; Maria Sofia L. Yangzon; Marissa Alejandria; Razel Nikka M. Hao; Rianna Patricia S. Cruz; Sheila Mae M. Araiza
EPI_ISL_534322	PS Mun Julio Tupy	Instituto Adolfo Lutz, Interdiciplinary Procedures Center, Strategic Laboratory	Claudia Regina Gonçalves; Claudio Tavares Sacchi; Erica Valessa Ramos Gomes
EPI_ISL_515524, EPI_ISL_515566, EPI_ISL_524466, EPI_ISL_527866	PS Municipal Dr Lauro Ribas Braga	Instituto Adolfo Lutz, Interdiciplinary Procedures Center, Strategic Laboratory	01246-1301; 355 - Brazil; Av. Dr. Arnaldo; Cerqueira Cesar; Claudia Regina Gonçalves; Claudio Tavares Sacchi; Erica Valessa Ramos Gomes; São Paulo - SP
EPI_ISL_524465	PS Municipal Dr. Caetano Virgílio Neto	Instituto Adolfo Lutz, Interdiciplinary Procedures Center, Strategic Laboratory	Claudia Regina Gonçalves; Claudio Tavares Sacchi; Erica Valessa Ramos Gomes
EPI_ISL_534321, EPI_ISL_861659, EPI_ISL_861660, EPI_ISL_861661, EPI_ISL_906067	PS e Maternidade Nair Fonseca Leitao Arantes	Instituto Adolfo Lutz, Interdiciplinary Procedures Center, Strategic Laboratory	Claudia Regina Gonçalves; Claudio Tavares Sacchi; Erica Valessa Ramos Gomes; Karoline Rodrigues Campos
EPI_ISL_861666	PSF Dr. Antonio Pires de Almeida	Instituto Adolfo Lutz, Interdiciplinary Procedures Center, Strategic Laboratory	Claudia Regina Gonçalves; Claudio Tavares Sacchi; Erica Valessa Ramos Gomes; Karoline Rodrigues Campos
EPI_ISL_984932, EPI_ISL_984938, EPI_ISL_1041373, EPI_ISL_1097913, EPI_ISL_1098162, EPI_ISL_1258936	Pandemic Response Lab - NYC	Pandemic Response Lab, R&D	Cybill del Castillo; Haiping Hao; Henry Lee; Jon Laurent; Melissa Hopkins; Michael Hammerling; Pradeep Bugga; Shinyoung Clair Kang; William Ward
EPI_ISL_2156364	Pasig City Children's Hospital - Child's Hope	Philippine Genome Center	Alethea R. de Guzman; Anna Ong-Lim; Arianne A. Zamora; Asia Louisa U. Chong; Benedict A. Maralit; Candice Francheska B. Tambaoan; Carlo M. Lapid; Celia Carlos; Devon Ray Pacial; Edsel Maurice Salvaña; El King D. Morado; Elcid Aaron R. Pangilinan; Eva Maria Cutiongco-de la Paz; Francis A. Tablizo; Irish Coleen A. Asin; Jaime C. Montoya; Jan Michael C. Yap; Jo-Hannah S. Llames; John Q. Wong; Joshua Gregor A. Dizon; Juan Antonio R. Magalang; Karol Sophia Agape R. Padilla; Kenneth M. Kim; Kris P. Punayan; Marc Edsel C. Ayes; Marc Jerrone R. Castro; Maria Rosario Singh-Vergeire and Cynthia P. Saloma; Maria Sofia L. Yangzon; Marissa Alejandria; Razel Nikka M. Hao; Rianna Patricia S. Cruz; Sheila Mae M. Araiza
EPI_ISL_591352, EPI_ISL_591372, EPI_ISL_591402, EPI_ISL_591411, EPI_ISL_591449, EPI_ISL_591450, EPI_ISL_591537, EPI_ISL_591538, EPI_ISL_667561, EPI_ISL_667578, EPI_ISL_667626, EPI_ISL_667633, EPI_ISL_667634, EPI_ISL_667639, EPI_ISL_667649, EPI_ISL_667650, EPI_ISL_667659, EPI_ISL_667663, EPI_ISL_685539, EPI_ISL_686307, EPI_ISL_690635, EPI_ISL_736895, EPI_ISL_768652, EPI_ISL_768709, EPI_ISL_779209, EPI_ISL_779216, EPI_ISL_779245, EPI_ISL_779246, EPI_ISL_901583, EPI_ISL_901605, EPI_ISL_1127124, EPI_ISL_1127125			
see above	Pathogen Genomics	Pathogen Genomics	Kentaro Itokawa; Makoto Kuroda; Masanori Hashino; Rina Tanaka; Tsuyoshi Sekizuka

	Center, National Institute of Infectious Diseases	Center, National Institute of Infectious Diseases	
EPI_ISL_2105405, EPI_ISL_2105420, EPI_ISL_2105432, EPI_ISL_2105446, EPI_ISL_2105459	Philippine Red Cross - Misamis Oriental	Philippine Genome Center	Alethea R. de Guzman; Anna Ong-Lim; Arianne A. Zamora; Asia Louisa U. Chong; Benedict A. Maralit; Candice Francheska B. Tambaoan; Carlo M. Lapid; Celia Carlos; Devon Ray Pacial; Edsel Maurice Salvaña; El King D. Morado; Eva Maria Cutiongco-de la Paz; Francis A. Tablizo; Irish Coleen A. Asin; Jaime C. Montoya; Jan Michael C. Yap; Jo-Hannah S. Llames; John Q. Wong; Joshua Gregor A. Dizon; Juan Antonio R. Magalang; Karol Sophia Agape R. Padilla; Kenneth M. Kim; Kris P. Punayan; Marc Edsel C. Ayes; Marc Jerrone R. Castro; Maria Rosario Singh-Vergeire and Cynthia P. Saloma; Maria Sofia L. Yangzon; Marissa Alejandria; Razel Nikka M. Hao; Rianna Patricia S. Cruz; Sheila Mae M. Araiza
EPI_ISL_2171200, EPI_ISL_2189213	Philippine Red Cross - Port Area	Philippine Genome Center	Alethea R. de Guzman; Anna Ong-Lim; Arianne A. Zamora; Asia Louisa U. Chong; Benedict A. Maralit; Candice Francheska B. Tambaoan; Carlo M. Lapid; Celia Carlos; Devon Ray Pacial; Edsel Maurice Salvaña; El King D. Morado; Elcid Aaron R. Pangilinan; Eva Maria Cutiongco-de la Paz; Francis A. Tablizo; Irish Coleen A. Asin; Jaime C. Montoya; Jan Michael C. Yap; Jo-Hannah S. Llames; John Q. Wong; Joshua Gregor A. Dizon; Juan Antonio R. Magalang; Karol Sophia Agape R. Padilla; Kenneth M. Kim; Kris P. Punayan; Marc Edsel C. Ayes; Marc Jerrone R. Castro; Maria Rosario Singh-Vergeire and Cynthia P. Saloma; Maria Sofia L. Yangzon; Marissa Alejandria; Razel Nikka M. Hao; Renato Jacinto Q. Mantaring; Rianna Patricia S. Cruz; Sheila Mae M. Araiza
EPI_ISL_2105381	Philippine Red Cross Logistics and Multipurpose Center	Philippine Genome Center	Alethea R. de Guzman; Anna Ong-Lim; Arianne A. Zamora; Asia Louisa U. Chong; Benedict A. Maralit; Candice Francheska B. Tambaoan; Carlo M. Lapid; Celia Carlos; Devon Ray Pacial; Edsel Maurice Salvaña; El King D. Morado; Eva Maria Cutiongco-de la Paz; Francis A. Tablizo; Irish Coleen A. Asin; Jaime C. Montoya; Jan Michael C. Yap; Jo-Hannah S. Llames; John Q. Wong; Joshua Gregor A. Dizon; Juan Antonio R. Magalang; Karol Sophia Agape R. Padilla; Kenneth M. Kim; Kris P. Punayan; Marc Edsel C. Ayes; Marc Jerrone R. Castro; Maria Rosario Singh-Vergeire and Cynthia P. Saloma; Maria Sofia L. Yangzon; Marissa Alejandria; Razel Nikka M. Hao; Rianna Patricia S. Cruz; Sheila Mae M. Araiza
EPI_ISL_2663256	Plataforma de Vigilancia Molecular (PVM) - FIOCRUZ/BA	Plataforma de Vigilancia Molecular (PVM) - FIOCRUZ/BA	Bruno Bezerril Andrade; Camila I. de Oliveira on behalf of the Fiocruz COVID-19 Genomic Surveillance Network.; Clarissa Araújo Gurgel; Leonardo Paiva Farias; Marina Cucco; Ricardo Khouri; Tiago Graf
EPI_ISL_513514, EPI_ISL_513532, EPI_ISL_513546, EPI_ISL_513557, EPI_ISL_513578	Programa de Oncovirologia, Instituto Nacional de Câncer	Programa de Oncovirologia, Instituto Nacional de Câncer	Andreia C. de Melo; Brunna M. Alves; Claudia Cicala; James Arthos; João P.B. Viola; Juliana D. Siqueira; Livia R. Goes; Marcelo A. Soares
EPI_ISL_1469657	Pronto Atendimento Campo Bom	Epiclin	Ana Paula Muterle; Carolina Comerlato; Eliana Márcia Da Ros Wendland; Fernando Hayashi Sant'Anna; Janira Prichula; Juliana Comerlato
EPI_ISL_1469721	Pronto Atendimento Cruzeiro do Sul	Epiclin	Ana Paula Muterle; Carolina Comerlato; Eliana Márcia Da Ros Wendland; Fernando Hayashi Sant'Anna; Janira Prichula; Juliana Comerlato
EPI_ISL_603021, EPI_ISL_693204	Pronto Socorro Dr. Conrado Cesarino Nuvolini	Instituto Adolfo Lutz, Interdiciplinary Procedures Center, Strategic Laboratory	Claudia Regina Gonçalves; Claudio Tavares Sacchi; Erica Valessa Ramos Gomes; Karoline Rodrigues Campos
EPI_ISL_471556	Pronto Socorro Jose Ibrahin	Instituto Adolfo Lutz, Interdiciplinary Procedures Center, Strategic Laboratory	Claudia Regina Gonçalves; Claudio Tavares Sacchi; Erica Valessa Ramos Gomes
EPI_ISL_515529	Pronto Socorro Municipal Julio Tupy	Instituto Adolfo Lutz, Interdiciplinary Procedures Center, Strategic Laboratory	Claudia Regina Gonçalves; Claudio Tavares Sacchi; Erica Valessa Ramos Gomes
EPI_ISL_693202	Pronto Socorro Municipal Prof. Joao Catarin Mezomo	Instituto Adolfo Lutz, Interdiciplinary Procedures Center, Strategic Laboratory	Claudia Regina Gonçalves; Claudio Tavares Sacchi; Erica Valessa Ramos Gomes; Karoline Rodrigues Campos
EPI_ISL_515554, EPI_ISL_523958	Pronto Socorro Municipal de Perus	Instituto Adolfo Lutz, Interdiciplinary Procedures Center, Strategic Laboratory	Claudia Regina Gonçalves; Claudio Tavares Sacchi; Erica Valessa Ramos Gomes
EPI_ISL_693231	Pronto Socorro Municipal de Santa Branca	Instituto Adolfo Lutz, Interdiciplinary Procedures Center, Strategic Laboratory	Claudia Regina Gonçalves; Claudio Tavares Sacchi; Erica Valessa Ramos Gomes; Karoline Rodrigues Campos
EPI_ISL_693210	Pronto-Socorro Dr. Osmar Mesquita	Instituto Adolfo Lutz, Interdiciplinary Procedures Center, Strategic Laboratory	Claudia Regina Gonçalves; Claudio Tavares Sacchi; Erica Valessa Ramos Gomes; Karoline Rodrigues Campos
EPI_ISL_693257, EPI_ISL_693278	Queensland Health Forensic and Scientific Services	Queensland Health Forensic and Scientific Services	Son Nguyen et al
EPI_ISL_854921	Quest Diagnostics	Quest Diagnostics	Anderson, B.; Bernstein; D.F.; Gerasimova, A.; Hua, M.; I.A.; K.E.; Kagan; L.E.; Lacbawan, F.; Liu Y.; Livingston; Owen, R.; Perez, A.; R.M.; Rosenthal; S.H.; Shalhout; Shlyakhter; Tanpaiboon, P.
EPI_ISL_1266775	Quest Diagnostics Incorporated	Centers for Disease Control and Prevention Division of Viral Diseases, Pathogen Discovery	A. Gerasimova; A. Perez; B. Anderson; Ben L. Rambo-Martin; Clinton R. Paden; Dakota Howard; Dhwani Batra; Duncan MacCannell; F. Lacbawan; I. A. Shlyakhter; K.E. Livingston; L.E. Bernstein; M. Hua; P. Tanpaiboon; Peter W. Cook; R. M. Kagan; R. Owen; R. V. Rolando; S. H. Rosenthal; Suxiang Tong; Y. Liu
EPI_ISL_1086554, EPI_ISL_1086960, EPI_ISL_1086980, EPI_ISL_1086982, EPI_ISL_1087267, EPI_ISL_1088169	Quest Diagnostics Incorporated	Respiratory Viruses Branch, Division of Viral Diseases, Centers for Disease Control and Prevention	A. Gerasimova; A. Perez; B. Anderson; Ben L. Rambo-Martin; Clinton R. Paden; Dakota Howard; Dhwani Batra; Duncan MacCannell; F. Lacbawan; I. A. Shlyakhter; K.E. Livingston; L.E. Bernstein; M. Hua; P. Tanpaiboon; Peter W. Cook; R. M. Kagan; R. Owen; R. V. Rolando; S. H. Rosenthal; Suxiang Tong; Y. Liu
EPI_ISL_2105310	Quezon City General Hospital (PGC)	Philippine Genome Center	Alethea R. de Guzman; Anna Ong-Lim; Arianne A. Zamora; Asia Louisa U. Chong; Benedict A. Maralit; Candice Francheska B. Tambaoan; Carlo M. Lapid; Celia Carlos; Devon Ray Pacial; Edsel Maurice Salvaña; El King D. Morado; Eva Maria Cutiongco-de la Paz; Francis A. Tablizo; Irish Coleen A. Asin; Jaime C. Montoya; Jan Michael C. Yap; Jo-Hannah S. Llames; John Q. Wong; Joshua Gregor A. Dizon; Juan Antonio R. Magalang; Karol Sophia Agape R. Padilla; Kenneth M. Kim; Kris P. Punayan; Marc Edsel C. Ayes; Marc Jerrone R. Castro; Maria Rosario Singh-Vergeire and Cynthia P. Saloma; Maria Sofia L. Yangzon; Marissa Alejandria; Razel Nikka M. Hao; Rianna Patricia S. Cruz; Sheila Mae M. Araiza
EPI_ISL_2101297	RSUD Mangusada	Eijkman Institute for Molecular Biology, National Agency for Research and Innovation; Molecular Biology Laboratory, Faculty Medicine and Health Sciences, Warmadewa University	Amin Soebandrio; Edison Johar; Erly Sintya; Frilasita A Yudhaputri; Hidayat Trimarsanto; Ida Ayu Wayan Mahayani; Iskandar Adnan; Khin Saw Myint; Lidwina Priliani; Lydia V. Panggalo; Muhammad Rezki Rasyak; Safarina G Malik; Sri Masyeni; Sukma Oktavianthi; Willy Agustine
EPI_ISL_920984	Regional Virus Laboratory, Belfast Health and Social Care Trust	COVID-19 Genomics UK (COG-UK) Consortium	Alison Watt; Ciara Cox; Conall McCaughey; David Simpson; Derek Fairley; James McKenna; Mairead Connor; Susan Feeney; Tanya Curran; Zoltan Molnar
EPI_ISL_2105289, EPI_ISL_2105337, EPI_ISL_2105418, EPI_ISL_2105461, EPI_ISL_2156351	Research Institute for Tropical Medicine, Inc. (RITM)	Philippine Genome Center	Alethea R. de Guzman; Anna Ong-Lim; Arianne A. Zamora; Asia Louisa U. Chong; Benedict A. Maralit; Candice Francheska B. Tambaoan; Carlo M. Lapid; Celia Carlos; Devon Ray Pacial; Edsel Maurice Salvaña; El King D. Morado; Elcid Aaron R. Pangilinan; Eva Maria Cutiongco-de la Paz; Francis A. Tablizo; Irish Coleen A. Asin; Jaime C. Montoya; Jan Michael C. Yap; Jo-Hannah S. Llames; John Q. Wong; Joshua Gregor A. Dizon; Juan Antonio R. Magalang; Karol Sophia Agape R. Padilla; Kenneth M. Kim; Kris P. Punayan; Marc Edsel C. Ayes; Marc Jerrone R. Castro; Maria Rosario Singh-Vergeire and Cynthia P. Saloma; Maria Sofia L. Yangzon; Marissa Alejandria; Razel Nikka M. Hao; Rianna Patricia S. Cruz; Sheila Mae M. Araiza
EPI_ISL_1046774, EPI_ISL_1046782, EPI_ISL_1425749, EPI_ISL_1427816, EPI_ISL_1927843, EPI_ISL_2131797	SARS-CoV-2 testing team, National Institute of Infectious Diseases	Pathogen Genomics Center, National Institute of Infectious Diseases	; Chikako Shimokawa; Daisuke Kobayashi; Eunsil Park; Hazuka Y Furihata; Ken Miyazawa; Kentaro Itokawa; Kento Fukano; Makoto Kuroda; Masanori Hashino; Masumichi Saito; Minoru Nagi; Naomi Nojiri; Nozomu Hanaoka; Rina Tanaka; Sana Uchikoba; Takanobu Kato; Takashi Sakudoh; Tsuguto Fujimoto; Tsuyoshi Sekizuka; Yasutaka Hoshino; Yasuyuki Morishima; Yoshihiro Kaku
EPI_ISL_1445177, EPI_ISL_1966112	SAUDE COLETIVA CAPAO BONITO	Instituto Butantan / Mendelics	Antonio Jorge Martins; Bianca Cechetto Carlos. Mendelics: Bibiana Santos; Bibiana Santos; Claudia Renata dos Santos Barros; Cíntia Bittar; David Schlesinger; David Schlesinger. Hemocentro Ribeirão Preto: Simone Kashima; Debora Botequio Moretti; Dimas Tadeu Covas; Elaine Cristina Marqueze; Elaine Vieira dos Santos; Elisangela Chicaroni Mattos; Erika Freitas; Evandra Strazza Rodrigues; Felipe Allan da Silva da Costa; Flavia Aburjaile; Fábio Sossai Possebon; Guilherme Campos; Guilherme Targino Valente; Heidge Fukumasu. USP-Botucatu: Rejane Maria Tommasini Grotto; Helena Lage Ferreira; Instituto Butantan: Dimas Tadeu Covas; Jardelina de Souza Todao Bernardino; Jayme A. Souza-Neto; Jessika Cristina Chagas Lesbon; Jorge A. Petroli Marchesi; José Salvatore Leister Patané; João Paulo Kitajima; João Pessoa Araújo Jr.; Leila Sabrina Ullmann; Loyze Paola Oliveira de Lima; Luiz Aurelio de Campos Crispin. Centro de Genômica Funcional da ESALQ: Luiz Lehmann Coutinho; Luiz Carlos Junior de Alcantara; Lívia Sacchetto; Maisa C. Pereira Parra; Maria Carolina Elias; Marta Giovanetti; Marília Moraes; Maurício Lacerda Nogueira. Prefeitura de Sao Paulo: Melissa Palmieri.; Patricia Akemi Assato; Paula Rahal; Paulo Inacio da Costa; Rafael dos Santos Bezerra; Raquel de Lello Rocha Campos Cassano. NGS Soluções Genômicas: Pilar

			Drummond Sampaio Corrêa Mariani. FZEA-USP Pirassununga: Mirele Daiana Poleti; Raul Machado Neto; Ricardo Augusto Brassaloti; Ricardo Haddad; Rodrigo Tocantins Calado.; Rodrigo Tocantins Calado. FAMERP-SJRP: Cecília Artico Banho; Sandra Coccuzzo Sampaio; Simone Kashima; Svetoslav Nanev Slavov; Vagner Fonseca; Vincent Louis Viala
EPI_ISL_1445168, EPI_ISL_1445242, EPI_ISL_1445244, EPI_ISL_1966073, EPI_ISL_1966194, EPI_ISL_1966488	SECAO CENTRO DE DIAGNOSTICO SECEDI	Instituto Butantan / Mendelics	Antonio Jorge Martins; Bianca Cechetto Carlos. Mendelics: Bibiana Santos; Bibiana Santos; Claudia Renata dos Santos Barros; Cíntia Bittar; David Schlesinger; David Schlesinger. Hemocentro Ribeirão Preto: Simone Kashima; Debora Botequio Moretti; Dimas Tadeu Covas; Elaine Cristina Marqueze; Elaine Vieira dos Santos; Elisangela Chicaroni Mattos; Erika Freitas; Evandra Strazza Rodrigues; Felipe Allan da Silva da Costa; Flavia Aburjaile; Fábio Sossai Possebon; Guilherme Campos; Guilherme Targino Valente; Heidge Fukumasu. USP-Botucatu: Rejane Maria Tommasini Grotto; Helena Lage Ferreira; Instituto Butantan: Dimas Tadeu Covas; Jardelina de Souza Todao Bernardino; Jayme A. Souza-Neto; Jessika Cristina Chagas Lesbon; Jorge A. Petroli Marchesi; José Salvatore Leister Patané; João Paulo Kitajima; João Pessoa Araújo Jr.; Leila Sabrina Ullmann; Loyze Paola Oliveira de Lima; Luiz Aurelio de Campos Crispin. Centro de Genômica Funcional da ESALQ: Luiz Lehmann Coutinho; Luiz Carlos Junior de Alcantara; Lívia Sacchetto; Maisa C. Pereira Parra; Maria Carolina Elias; Marta Giovanetti; Marília Moraes; Maurício Lacerda Nogueira. Prefeitura de Sao Paulo: Melissa Palmieri.; Patricia Akemi Assato; Paula Rahal; Paulo Inacio da Costa; Rafael dos Santos Bezerra; Raquel de Lello Rocha Campos Cassano. NGS Soluções Genômicas: Pilar Drummond Sampaio Corrêa Mariani. FZEA-USP Pirassununga: Mirele Daiana Poleti; Raul Machado Neto; Ricardo Augusto Brassaloti; Ricardo Haddad; Rodrigo Tocantins Calado.; Rodrigo Tocantins Calado. FAMERP-SJRP: Cecília Artico Banho; Sandra Coccuzzo Sampaio; Simone Kashima; Svetoslav Nanev Slavov; Vagner Fonseca; Vincent Louis Viala
EPI_ISL_2344674	SECRETARIA MUNICIPAL DE SAUDE DE CORDEIROPOLIS	Instituto Butantan / FZEA-USP-Pirassununga	Antonio Jorge Martins; Claudia Renata dos Santos Barros; David Schlesinger; Debora Botequio Moretti; Dimas Tadeu Covas; Elaine Cristina Marqueze; Elaine Vieira Santos; Evandra Strazza Rodrigues; Heidge Fukumasu; Jayme Augusto de Souza-Neto; José Salvatore Leister Patané; Luiz Alcantara; Luiz Lehmann Coutinho; Maria Carolina Elias; Maurício Lacerda Nogueira; Rafael dos Santos Bezerra; Raul Machado Neto; Rejane Maria Tommasini Grotto; Ricardo Haddad; Sandra Coccuzzo Sampaio Vessoni; Simone Kashima; Svetoslav Nanev Slavov; Vincent Louis Viala
EPI_ISL_1469683, EPI_ISL_1469687	SECRETARIA MUNICIPAL DE SAUDE DE TAQUARA	Epiclin	Ana Paula Muterle; Carolina Comerlato; Eliana Márcia Da Ros Wendland; Fernando Hayashi Sant'Anna; Janira Prichula; Juliana Comerlato
EPI_ISL_1469740	SECRETARIA MUNICIPAL DE SAUDE DE TRES COROAS	Epiclin	Ana Paula Muterle; Carolina Comerlato; Eliana Márcia Da Ros Wendland; Fernando Hayashi Sant'Anna; Janira Prichula; Juliana Comerlato
EPI_ISL_1445134, EPI_ISL_1966178	SECRETARIA MUNICIPAL DE SAUDE SOROCABA	Instituto Butantan / Mendelics	Antonio Jorge Martins; Bianca Cechetto Carlos. Mendelics: Bibiana Santos; Bibiana Santos; Claudia Renata dos Santos Barros; Cíntia Bittar; David Schlesinger; David Schlesinger. Hemocentro Ribeirão Preto: Simone Kashima; Debora Botequio Moretti; Dimas Tadeu Covas; Elaine Cristina Marqueze; Elaine Vieira dos Santos; Elisangela Chicaroni Mattos; Erika Freitas; Evandra Strazza Rodrigues; Felipe Allan da Silva da Costa; Flavia Aburjaile; Fábio Sossai Possebon; Guilherme Campos; Guilherme Targino Valente; Heidge Fukumasu. USP-Botucatu: Rejane Maria Tommasini Grotto; Helena Lage Ferreira; Instituto Butantan: Dimas Tadeu Covas; Jardelina de Souza Todao Bernardino; Jayme A. Souza-Neto; Jessika Cristina Chagas Lesbon; Jorge A. Petroli Marchesi; José Salvatore Leister Patané; João Paulo Kitajima; João Pessoa Araújo Jr.; Leila Sabrina Ullmann; Loyze Paola Oliveira de Lima; Luiz Aurelio de Campos Crispin. Centro de Genômica Funcional da ESALQ: Luiz Lehmann Coutinho; Luiz Carlos Junior de Alcantara; Lívia Sacchetto; Maisa C. Pereira Parra; Maria Carolina Elias; Marta Giovanetti; Marília Moraes; Maurício Lacerda Nogueira. Prefeitura de Sao Paulo: Melissa Palmieri.; Patricia Akemi Assato; Paula Rahal; Paulo Inacio da Costa; Rafael dos Santos Bezerra; Raquel de Lello Rocha Campos Cassano. NGS Soluções Genômicas: Pilar Drummond Sampaio Corrêa Mariani. FZEA-USP Pirassununga: Mirele Daiana Poleti; Raul Machado Neto; Ricardo Augusto Brassaloti; Ricardo Haddad; Rodrigo Tocantins Calado.; Rodrigo Tocantins Calado. FAMERP-SJRP: Cecília Artico Banho; Sandra Coccuzzo Sampaio; Simone Kashima; Svetoslav Nanev Slavov; Vagner Fonseca; Vincent Louis Viala
EPI_ISL_2346082	SERRANA	Instituto Butantan / Mendelics	Antonio Jorge Martins; Claudia Renata dos Santos Barros; David Schlesinger; Debora Botequio Moretti; Dimas Tadeu Covas; Elaine Cristina Marqueze; Elaine Vieira Santos; Evandra Strazza Rodrigues; Heidge Fukumasu; Jayme Augusto de Souza-Neto; José Salvatore Leister Patané; Luiz Alcantara; Luiz Lehmann Coutinho; Maria Carolina Elias; Maurício Lacerda Nogueira; Rafael dos Santos Bezerra; Raul Machado Neto; Rejane Maria Tommasini Grotto; Ricardo Haddad; Sandra Coccuzzo Sampaio Vessoni; Simone Kashima; Svetoslav Nanev Slavov; Vincent Louis Viala
EPI_ISL_1795398, EPI_ISL_2345454	SMS SECRETARIA MUNICIPAL DE SAUDE DE BOITUVA	Instituto Butantan / ESALQ-Piracicaba	Antonio Jorge Martins; Bianca Cechetto Carlos. Mendelics: Bibiana Santos; Claudia Renata dos Santos Barros; David Schlesinger; David Schlesinger. Hemocentro Ribeirão Preto: Simone Kashima; Debora Botequio Moretti; Debora Botequio Moretti. Centro de Genômica Funcional da ESALQ: Luiz Lehmann Coutinho; Dimas Tadeu Covas; Elaine Cristina Marqueze; Elaine Vieira Santos; Elaine Vieira dos Santos; Elisangela Chicaroni Mattos; Erika Freitas; Evandra Strazza Rodrigues; Felipe Allan da Silva da Costa; Flavia Aburjaile; Guilherme Targino Valente; Heidge Fukumasu; Heidge Fukumasu. USP-Botucatu: Rejane Maria Tommasini Grotto; Instituto Butantan: Alexander Roberto Precioso; Jayme A. Souza-Neto; Jayme Augusto de Souza-Neto; Jessika Cristina Chagas Lesbon; José Salvatore Leister Patané; João Paulo Kitajima; Luiz Alcantara; Luiz Carlos Junior de Alcantara; Luiz Lehmann Coutinho; Maria Carolina Elias; Marta Giovanetti; Maurício Lacerda Nogueira; Patricia Akemi Assato; Rafael dos Santos Bezerra; Raquel de Lello Rocha Campos Cassano. NGS Soluções Genômicas: Pilar Drummond Sampaio Corrêa Mariani. FZEA-USP Pirassununga: Mirele Daiana Poleti; Raul Machado Neto; Rejane Maria Tommasini Grotto; Ricardo Augusto Brassaloti; Ricardo Haddad; Rodrigo Tocantins Calado.; Sandra Coccuzzo Sampaio; Sandra Coccuzzo Sampaio Vessoni; Simone Kashima; Svetoslav Nanev Slavov; Vagner Fonseca; Vincent Louis Viala
EPI_ISL_1966261	SMS SECRETARIA MUNICIPAL DE SAUDE DE BOITUVA	Instituto Butantan / Mendelics	Antonio Jorge Martins; Bianca Cechetto Carlos. Mendelics: Bibiana Santos; Claudia Renata dos Santos Barros; Cíntia Bittar; David Schlesinger. Hemocentro Ribeirão Preto: Simone Kashima; Debora Botequio Moretti; Elaine Cristina Marqueze; Elaine Vieira dos Santos; Elisangela Chicaroni Mattos; Erika Freitas; Evandra Strazza Rodrigues; Felipe Allan da Silva da Costa; Flavia Aburjaile; Fábio Sossai Possebon; Guilherme Campos; Guilherme Targino Valente; Heidge Fukumasu. USP-Botucatu: Rejane Maria Tommasini Grotto; Helena Lage Ferreira; Instituto Butantan: Dimas Tadeu Covas; Jardelina de Souza Todao Bernardino; Jayme A. Souza-Neto; Jessika Cristina Chagas Lesbon; Jorge A. Petroli Marchesi; José Salvatore Leister Patané; João Paulo Kitajima; João Pessoa Araújo Jr.; Leila Sabrina Ullmann; Loyze Paola Oliveira de Lima; Luiz Aurelio de Campos Crispin. Centro de Genômica Funcional da ESALQ: Luiz Lehmann Coutinho; Luiz Carlos Junior de Alcantara; Lívia Sacchetto; Maisa C. Pereira Parra; Maria Carolina Elias; Marta Giovanetti; Marília Moraes; Maurício Lacerda Nogueira. Prefeitura de Sao Paulo: Melissa Palmieri.; Patricia Akemi Assato; Paula Rahal; Paulo Inacio da Costa; Rafael dos Santos Bezerra; Raquel de Lello Rocha Campos Cassano. NGS Soluções Genômicas: Pilar Drummond Sampaio Corrêa Mariani. FZEA-USP Pirassununga: Mirele Daiana Poleti; Raul Machado Neto; Ricardo Augusto Brassaloti; Ricardo Haddad; Rodrigo Tocantins Calado. FAMERP-SJRP: Cecília Artico Banho; Sandra Coccuzzo Sampaio; Svetoslav Nanev Slavov; Vagner Fonseca; Vincent Louis Viala
EPI_ISL_547575	SVO Jundiaí	Instituto Adolfo Lutz, Interdiciplinary Procedures Center, Strategic Laboratory	Claudia Regina Gonçalves; Claudio Tavares Sacchi; Erica Valessa Ramos Gomes; Karoline Rodrigues Campos
EPI_ISL_1854406	SYNLAB	Instituto Nacional de Saude (INSA)	Borges et al
EPI_ISL_2105340, EPI_ISL_2105394	Safeguard DNA Diagnostic, Inc.	Philippine Genome Center	Alethea R. de Guzman; Anna Ong-Lim; Arianne A. Zamora; Asia Louisa U. Chong; Benedict A. Maralit; Candice Francheska B. Tambaoan; Carlo M. Lapid; Celia Carlos; Devon Ray Pacial; Edsel Maurice Salvaña; El King D. Morado; Eva Maria Cutiongco-de la Paz; Francis A. Tablizo; Irish Coleen A. Asin; Jaime C. Montoya; Jan Michael C. Yap; Jo-Hannah S. Llames; John Q. Wong; Joshua Gregor A. Dizon; Juan Antonio R. Magalang; Karol Sophia Agape R. Padilla; Kenneth M. Kim; Kris P. Punayan; Marc Edsel C. Ayes; Marc Jerrone R. Castro; Maria Rosario Singh-Vergeire and Cynthia P. Saloma; Maria Sofia L. Yangzon; Marissa Alejandria; Razel Nikka M. Hao; Rianna Patricia S. Cruz; Sheila Mae M. Araiza
EPI_ISL_1426592, EPI_ISL_1426593, EPI_ISL_1427064	Sakai City Institute of Public Health	Pathogen Genomics Center, National Institute of Infectious Diseases	Kentaro Itokawa; Makoto Kuroda; Masanori Hashino; Rina Tanaka; Tsuyoshi Sekizuka
EPI_ISL_2350041, EPI_ISL_2350066, EPI_ISL_2603719, EPI_ISL_2603730	Salud Digna	Instituto Nacional de Medicina Genomica	Abraham Campos-Romero; Cedro-Tanda A; Escobar-Arrazola; Gonzalez-Barrera D; Herrera-Montalvo LA.; Hidalgo-Miranda A; Luna-Ruiz Marco; M; Mendoza-Vargas A; Moreno-Camacho José Luis; Munguia-Garza P; Ramirez-Vega O; Rangel-DeLeon D; Reyes-Grajeda JP; Rodriguez-Gallegos Jorge
EPI_ISL_2105287	San Antonio City of Ilagan Hospital	Philippine Genome Center	Alethea R. de Guzman; Anna Ong-Lim; Arianne A. Zamora; Asia Louisa U. Chong; Benedict A. Maralit; Candice Francheska B. Tambaoan; Carlo M. Lapid; Celia Carlos; Devon Ray Pacial; Edsel Maurice Salvaña; El King D. Morado; Eva Maria Cutiongco-de la Paz; Francis A. Tablizo; Irish Coleen A. Asin; Jaime C. Montoya; Jan Michael C. Yap; Jo-Hannah S. Llames; John Q. Wong; Joshua Gregor A. Dizon; Juan Antonio R. Magalang; Karol Sophia Agape R. Padilla; Kenneth M. Kim; Kris P. Punayan; Marc Edsel C. Ayes; Marc Jerrone R. Castro; Maria Rosario Singh-Vergeire and Cynthia P. Saloma; Maria Sofia L. Yangzon; Marissa Alejandria; Razel Nikka M. Hao; Rianna Patricia S. Cruz; Sheila Mae M. Araiza
EPI_ISL_750177	Sanatorio Americano	Institut Pasteur de Montevideo	Ana Carolina Mendonça; Andrés Lizasoain; Camila Simoes; Cecilia Alonso; Cecilia Salazar; Daiana Mir; Fernando López-Tort; Fernando Motta; Gonzalo Bello; Ighor Arantes; Ignacio Ferrés; Jose Sotelo; Leticia Maya; Letícia Garay Martins; Luciana Appolinario; Lucía Spangenberg; Mailen Arleo; Mariana Brandes; Marilda Mendonça Siqueira; Marilda Tereza Mar da Rosa; María José Benitez-Galeano; Martín Graña; Matías Castells; Matías Victoria; Matías Salvo; Natalia Rego; Natalia Reyes; Pablo Smircich; Paola Cristina Resende; Rodney Colina; Tamara Fernandez-Calero; Tania Possi; Tatiana Schäffer Gregianini; Verónica Noya; Yasser Vega
EPI_ISL_583492	Santa Casa Anna Cintra	Instituto Adolfo Lutz, Interdiciplinary Procedures Center, Strategic Laboratory	Claudia Regina Gonçalves; Claudio Tavares Sacchi; Erica Valessa Ramos Gomes; Karoline Rodrigues Campos
EPI_ISL_1468454	Santa Casa de Andradina	Instituto Adolfo Lutz, Interdiciplinary Procedures Center, Strategic Laboratory	Caio Vinicius Dias Lopes; Claudia Regina Gonçalves; Claudio Tavares Sacchi; Erica Valessa Ramos Gomes; Karoline Rodrigues Campos
EPI_ISL_1493592	Santa Casa de Guaira	Instituto Adolfo Lutz, Interdiciplinary Procedures Center, Strategic Laboratory	Caio Vinicius Dias Lopes; Claudia Regina Gonçalves; Claudio Tavares Sacchi; Erica Valessa Ramos Gomes; Karoline Rodrigues Campos
EPI_ISL_603024, EPI_ISL_603038	Santa Casa de Misericordia de Araçatuba	Instituto Adolfo Lutz, Interdiciplinary Procedures Center, Strategic Laboratory	Claudia Regina Gonçalves; Claudio Tavares Sacchi; Erica Valessa Ramos Gomes; Karoline Rodrigues Campos
EPI_ISL_693212	Santa Casa de Misericordia de Braganca Paulista	Instituto Adolfo Lutz, Interdiciplinary Procedures Center, Strategic Laboratory	Claudia Regina Gonçalves; Claudio Tavares Sacchi; Erica Valessa Ramos Gomes; Karoline Rodrigues Campos
EPI_ISL_1468453	Santa Casa de Misericordia de Pereira Barreto	Instituto Adolfo Lutz, Interdiciplinary Procedures Center, Strategic Laboratory	Caio Vinicius Dias Lopes; Claudia Regina Gonçalves; Claudio Tavares Sacchi; Erica Valessa Ramos Gomes; Karoline Rodrigues Campos
EPI_ISL_524469	Santa Casa de Misericordia de Sao Paulo	Instituto Adolfo Lutz, Interdiciplinary Procedures Center, Strategic Laboratory	Claudia Regina Gonçalves; Claudio Tavares Sacchi; Erica Valessa Ramos Gomes
EPI_ISL_693198	Santa Casa de Misericordia de Sao Paulo - Hospital Central	Instituto Adolfo Lutz, Interdiciplinary Procedures Center, Strategic Laboratory	Claudia Regina Gonçalves; Claudio Tavares Sacchi; Erica Valessa Ramos Gomes; Karoline Rodrigues Campos
EPI_ISL_693211	Santa Casa de Misericordia e Maternidade	Instituto Adolfo Lutz, Interdiciplinary Procedures Center, Strategic Laboratory	Claudia Regina Gonçalves; Claudio Tavares Sacchi; Erica Valessa Ramos Gomes; Karoline Rodrigues Campos
EPI_ISL_547579	Santa Casa de Misericórdia de Araçatuba	Instituto Adolfo Lutz, Interdiciplinary Procedures Center,	Claudia Regina Gonçalves; Claudio Tavares Sacchi; Erica Valessa Ramos Gomes; Karoline Rodrigues Campos

		Strategic Laboratory	
EPI_ISL_524464, EPI_ISL_1121322	Santa Casa de Santa Isabel	Instituto Adolfo Lutz, Interdiciplinary Procedures Center, Strategic Laboratory	Caio Vinicius Dias Lopes; Claudia Regina Gonçalves; Claudio Tavares Sacchi; Erica Valessa Ramos Gomes; Karoline Rodrigues Campos
EPI_ISL_1468466	Santa Casa de Sao Carlos	Instituto Adolfo Lutz, Interdiciplinary Procedures Center, Strategic Laboratory	Caio Vinicius Dias Lopes; Claudia Regina Gonçalves; Claudio Tavares Sacchi; Erica Valessa Ramos Gomes; Karoline Rodrigues Campos
EPI_ISL_1533692	Santa Casa de Sao Paulo	Instituto Adolfo Lutz, Interdiciplinary Procedures Center, Strategic Laboratory	Caio Vinicius Dias Lopes; Claudia Regina Gonçalves; Claudio Tavares Sacchi; Erica Valessa Ramos Gomes; Karoline Rodrigues Campos; Leonardo Jose Tadeu de Araujo
EPI_ISL_693238, EPI_ISL_693239	Secao Centro de Diagnostico Secedi	Instituto Adolfo Lutz, Interdiciplinary Procedures Center, Strategic Laboratory	Claudia Regina Gonçalves; Claudio Tavares Sacchi; Erica Valessa Ramos Gomes; Karoline Rodrigues Campos
EPI_ISL_735405	Secretaria Minucipal de Saude de Birigui	Instituto Adolfo Lutz, Interdiciplinary Procedures Center, Strategic Laboratory	Claudia Regina Gonçalves; Claudio Tavares Sacchi; Erica Valessa Ramos Gomes; Karoline Rodrigues Campos
EPI_ISL_2919013, EPI_ISL_2919014, EPI_ISL_2928510	Secretaria Municipal da Saúde-Porto Alegre	Laboratório de Microbiologia Molecular - Universidade FEEVALE	Alana Witt Hansen; Fernando Rosado Spilki; Flávio Silveira; Fágner Henrique Heldt; Juliana Schons Gulart; Juliana Schons Gularte; Juliane Deise Fleck; Mariana Soares da Silva; Matheus Nunes Weber; Meriane Demoliner; Michele Filippi.; Micheli Filippi.; Paula Rodrigues de Almeida
EPI_ISL_882658	Secretaria Municipal de Saude	Instituto Adolfo Lutz, Interdiciplinary Procedures Center, Strategic Laboratory	Claudia Regina Gonçalves; Claudio Tavares Sacchi; Erica Valessa Ramos Gomes; Karoline Rodrigues Campos
EPI_ISL_708530	Secretaria Municipal de Saude de Fernandópolis	Instituto Adolfo Lutz, Interdiciplinary Procedures Center, Strategic Laboratory	Carlos Henrique Camargo; Claudia Regina Gonçalves; Claudio Tavares Sacchi; Erica Valessa Ramos Gomes; Fernanda Modesto Tolentino Binhardi; Janaina Other Martins Montanha; Karoline Rodrigues Campos; Marcia Maria Costa Nunes Soares; Maricelia Navarro Pinheiro Flores
EPI_ISL_574583	Secretaria Municipal de Saude de Jandira	Instituto Adolfo Lutz, Interdiciplinary Procedures Center, Strategic Laboratory	Claudia Regina Gonçalves; Claudio Tavares Sacchi; Erica Valessa Ramos Gomes; Karoline Rodrigues Campos
EPI_ISL_574597	Secretaria Municipal de Saude de Jarinu	Instituto Adolfo Lutz, Interdiciplinary Procedures Center, Strategic Laboratory	Claudia Regina Gonçalves; Claudio Tavares Sacchi; Erica Valessa Ramos Gomes; Karoline Rodrigues Campos
EPI_ISL_1040823	Secretaria Municipal de Saude de Piracaia	Instituto Adolfo Lutz, Interdiciplinary Procedures Center, Strategic Laboratory	Claudia Regina Gonçalves; Claudio Tavares Sacchi; Erica Valessa Ramos Gomes; Karoline Rodrigues Campos
EPI_ISL_833164	Secretaria Municipal de Saude de Santa Barbara d'oeste	Instituto Adolfo Lutz, Interdiciplinary Procedures Center, Strategic Laboratory	Claudia Regina Gonçalves; Claudio Tavares Sacchi; Erica Valessa Ramos Gomes; Karoline Rodrigues Campos
EPI_ISL_547576, EPI_ISL_603035	Secretaria Municipal de Saúde	Instituto Adolfo Lutz, Interdiciplinary Procedures Center, Strategic Laboratory	Claudia Regina Gonçalves; Claudio Tavares Sacchi; Erica Valessa Ramos Gomes; Karoline Rodrigues Campos
EPI_ISL_693221	Secretaria Municipal de Saúde de Birigui	Instituto Adolfo Lutz, Interdiciplinary Procedures Center, Strategic Laboratory	Claudia Regina Gonçalves; Claudio Tavares Sacchi; Erica Valessa Ramos Gomes; Karoline Rodrigues Campos
EPI_ISL_693215	Secretaria Municipal de Saúde de Iracemapolis	Instituto Adolfo Lutz, Interdiciplinary Procedures Center, Strategic Laboratory	Claudia Regina Gonçalves; Claudio Tavares Sacchi; Erica Valessa Ramos Gomes; Karoline Rodrigues Campos
EPI_ISL_1469588, EPI_ISL_1469647	Secretaria Municipal de Saúde de Taquara	Epiclin	Ana Paula Muterle; Carolina Comerlato; Eliana Márcia Da Ros Wendland; Fernando Hayashi Sant'Anna; Janira Prichula; Juliana Comerlato
EPI_ISL_1469803	Secretaria Municipal de Saúde de Três Coroas	Epiclin	Ana Paula Muterle; Carolina Comerlato; Eliana Márcia Da Ros Wendland; Fernando Hayashi Sant'Anna; Janira Prichula; Juliana Comerlato
EPI_ISL_693228	Secretaria Municipal de Sorocaba	Instituto Adolfo Lutz, Interdiciplinary Procedures Center, Strategic Laboratory	Claudia Regina Gonçalves; Claudio Tavares Sacchi; Erica Valessa Ramos Gomes; Karoline Rodrigues Campos
EPI_ISL_471542	Secretaria de Saude de Mogi das Cruzes	Instituto Adolfo Lutz, Interdiciplinary Procedures Center, Strategic Laboratory	Claudia Regina Gonçalves; Claudio Tavares Sacchi; Erica Valessa Ramos Gomes
EPI_ISL_583502	Serv de Vig Sanitaria Epidemio e CTRL de Zoonoses Guaruja	Instituto Adolfo Lutz, Interdiciplinary Procedures Center, Strategic Laboratory	Claudia Regina Gonçalves; Claudio Tavares Sacchi; Erica Valessa Ramos Gomes; Karoline Rodrigues Campos
EPI_ISL_2135301, EPI_ISL_2135303	Servicio Virosis Respiratorias-Departamento Virología-INEI	Instituto Nacional Enfermedades Infecciosas C.G.Malbran	Avaro M.; Baumeister E.; Benedetti E.; Campos J.; Cisterna D.; Dattero ME; Lorenzo F.; Molina V.; Perandones C.; Poklepovich T.; Pontoriero A.; Russo M.; Tuduri E.
EPI_ISL_574598	Servico de Verificacao de Obito SVO	Instituto Adolfo Lutz, Interdiciplinary Procedures Center, Strategic Laboratory	Claudia Regina Gonçalves; Claudio Tavares Sacchi; Erica Valessa Ramos Gomes; Karoline Rodrigues Campos
EPI_ISL_1165070	Siti Khodijah Hospital	Institute of Tropical Disease, Universitas Airlangga	Aldise M Nastri; Gatot Soegiarto; Jezzy R Dewantari; Kazufumi Shimizu; Krisnoadi Rahardjo; Laksmi Wulandari; Maria I Lusida; Muhammad Hamdan; Resti Yudhawati; Rima R Prasetya; Soetjipto; Yasuko Mori
EPI_ISL_1154442	Sonic - Labor Dr. von Froreich GmbH	Robert Koch Institute	
EPI_ISL_490026, EPI_ISL_593687, EPI_ISL_593698, EPI_ISL_593711	South Eastern Area Laboratory Services (SEALS)	NSW Health Pathology - Institute of Clinical Pathology and Medical Research; Westmead Hospital; University of Sydney	CIDM-PH et al.
EPI_ISL_2105232	South Super	Philippine Genome	Alethea R. de Guzman; Anna Ong-Lim; Arianne A. Zamora; Asia Louisa U. Chong; Benedict A. Maralit; Candice Francheska B. Tambaoan; Carlo M. Lapid; Celia Carlos; Devon Ray Pacial; Edsel Maurice Salvaña; El King D. Morado; Eva Maria Cutiongco-de la Paz; Francis A. Tablizo; Irish Coleen A. Asin; Jaime C. Montoya; Jan Michael C. Yap; Jo-

	Highway Molecular Diagnostic Laboratory	Center	Hannah S. Llames; John Q. Wong; Joshua Gregor A. Dizon; Juan Antonio R. Magalang; Karol Sophia Agape R. Padilla; Kenneth M. Kim; Kris P. Punayan; Marc Edsel C. Ayes; Marc Jerrone R. Castro; Maria Rosario Singh-Vergeire and Cynthia P. Saloma; Maria Sofia L. Yangzon; Marissa Alejandria; Razel Nikka M. Hao; Rianna Patricia S. Cruz; Sheila Mae M. Araiza
EPI_ISL_2105264, EPI_ISL_2105266, EPI_ISL_2105272	Southern Philippines Medical Center (SPMC)	Philippine Genome Center	Alethea R. de Guzman; Anna Ong-Lim; Arianne A. Zamora; Asia Louisa U. Chong; Benedict A. Maralit; Candice Francheska B. Tambaoan; Carlo M. Lapid; Celia Carlos; Devon Ray Pacial; Edsel Maurice Salvaña; El King D. Morado; Eva Maria Cutiongco-de la Paz; Francis A. Tablizo; Irish Coleen A. Asin; Jaime C. Montoya; Jan Michael C. Yap; Jo-Hannah S. Llames; John Q. Wong; Joshua Gregor A. Dizon; Juan Antonio R. Magalang; Karol Sophia Agape R. Padilla; Kenneth M. Kim; Kris P. Punayan; Marc Edsel C. Ayes; Marc Jerrone R. Castro; Maria Rosario Singh-Vergeire and Cynthia P. Saloma; Maria Sofia L. Yangzon; Marissa Alejandria; Razel Nikka M. Hao; Rianna Patricia S. Cruz; Sheila Mae M. Araiza
EPI_ISL_2105293, EPI_ISL_2105294, EPI_ISL_2105300, EPI_ISL_2105301, EPI_ISL_2105302, EPI_ISL_2105303, EPI_ISL_2105305, EPI_ISL_2105308, EPI_ISL_2105309, EPI_ISL_2105314, EPI_ISL_2105316, EPI_ISL_2105319, EPI_ISL_2105321, EPI_ISL_2105324, EPI_ISL_2105330, EPI_ISL_2105334, EPI_ISL_2105336, EPI_ISL_2105338, EPI_ISL_2105342, EPI_ISL_2105343, EPI_ISL_2105344, EPI_ISL_2105345, EPI_ISL_2105347, EPI_ISL_2105348, EPI_ISL_2105349, EPI_ISL_2105350, EPI_ISL_2105351, EPI_ISL_2105354, EPI_ISL_2105355, EPI_ISL_2105357, EPI_ISL_2105358, EPI_ISL_2105362, EPI_ISL_2105368, EPI_ISL_2105372, EPI_ISL_2105374, EPI_ISL_2105376, EPI_ISL_2105384, EPI_ISL_2105385, EPI_ISL_2105386, EPI_ISL_2105387, EPI_ISL_2105389, EPI_ISL_2105425			
see above	Southern Philippines Medical Center (SPMC-SNL-MBL)	Philippine Genome Center	Alethea R. de Guzman; Anna Ong-Lim; Arianne A. Zamora; Asia Louisa U. Chong; Benedict A. Maralit; Candice Francheska B. Tambaoan; Carlo M. Lapid; Celia Carlos; Devon Ray Pacial; Edsel Maurice Salvaña; El King D. Morado; Eva Maria Cutiongco-de la Paz; Francis A. Tablizo; Irish Coleen A. Asin; Jaime C. Montoya; Jan Michael C. Yap; Jo-Hannah S. Llames; John Q. Wong; Joshua Gregor A. Dizon; Juan Antonio R. Magalang; Karol Sophia Agape R. Padilla; Kenneth M. Kim; Kris P. Punayan; Marc Edsel C. Ayes; Marc Jerrone R. Castro; Maria Rosario Singh-Vergeire and Cynthia P. Saloma; Maria Sofia L. Yangzon; Marissa Alejandria; Razel Nikka M. Hao; Rianna Patricia S. Cruz; Sheila Mae M. Araiza
EPI_ISL_1364498	Stanford Health Care	Stanford University School of Medicine, Clinical Virology Laboratory	ChunHong Huang; Daniel Solis; Fumiko Yamamoto; James Zehnder; Malaya K. Sahoo; Mamdouh Sibai; Michelle Verghese; and Benjamin A. Pinsky
EPI_ISL_1760559, EPI_ISL_2280076	TXDSHS	TXDSHS	Anita Pokharel; Bonnie Oh; Chun Wang; Grace Kubin; Jenny Zhang; Karen Bobier; Lorraine Rodriguez; Maliha Rahman; Mayela Pedrueza; Myong Koag; Rachel Lee; Rashmi Tuladhar
EPI_ISL_1660606, EPI_ISL_1660607, EPI_ISL_1660610, EPI_ISL_1660613, EPI_ISL_1660614, EPI_ISL_1760108	Texas Department of State Health Services (TXDSHS)	Texas Department of State Health Services (TXDSHS)	Anita Pokharel; Bonnie Oh; Chun Wang; Grace Kubin; Jenny Zhang; Lorraine Rodriguez; Maliha Rahman; Mayela Pedrueza; Myong Koag; Rachel Lee; Rashmi Tuladhar
EPI_ISL_672205	The Ashley Laboratory, Stanford University	Chan-Zuckerberg Biohub	CZB Cliahub Consortium
EPI_ISL_2105280, EPI_ISL_2105546, EPI_ISL_2188275	The Lord's Grace Medical and Industrial Clinic	Philippine Genome Center	Alethea R. de Guzman; Anna Ong-Lim; Arianne A. Zamora; Asia Louisa U. Chong; Benedict A. Maralit; Candice Francheska B. Tambaoan; Carlo M. Lapid; Celia Carlos; Devon Ray Pacial; Edsel Maurice Salvaña; El King D. Morado; Elcid Aaron R. Pangilinan; Eva Maria Cutiongco-de la Paz; Francis A. Tablizo; Irish Coleen A. Asin; Jaime C. Montoya; Jan Michael C. Yap; Jo-Hannah S. Llames; John Q. Wong; Joshua Gregor A. Dizon; Juan Antonio R. Magalang; Karol Sophia Agape R. Padilla; Kenneth M. Kim; Kris P. Punayan; Marc Edsel C. Ayes; Marc Jerrone R. Castro; Maria Rosario Singh-Vergeire and Cynthia P. Saloma; Maria Sofia L. Yangzon; Marissa Alejandria; Razel Nikka M. Hao; Rianna Patricia S. Cruz; Sheila Mae M. Araiza
EPI_ISL_2105292, EPI_ISL_2105335, EPI_ISL_2105369, EPI_ISL_2105411	The Medical City -Â Ortigas	Philippine Genome Center	Alethea R. de Guzman; Anna Ong-Lim; Arianne A. Zamora; Asia Louisa U. Chong; Benedict A. Maralit; Candice Francheska B. Tambaoan; Carlo M. Lapid; Celia Carlos; Devon Ray Pacial; Edsel Maurice Salvaña; El King D. Morado; Eva Maria Cutiongco-de la Paz; Francis A. Tablizo; Irish Coleen A. Asin; Jaime C. Montoya; Jan Michael C. Yap; Jo-Hannah S. Llames; John Q. Wong; Joshua Gregor A. Dizon; Juan Antonio R. Magalang; Karol Sophia Agape R. Padilla; Kenneth M. Kim; Kris P. Punayan; Marc Edsel C. Ayes; Marc Jerrone R. Castro; Maria Rosario Singh-Vergeire and Cynthia P. Saloma; Maria Sofia L. Yangzon; Marissa Alejandria; Razel Nikka M. Hao; Rianna Patricia S. Cruz; Sheila Mae M. Araiza
EPI_ISL_3912447	UAPS LUIS COSTA	Analytical Competence Molecular Epidemiology Lab/ACME, Oswaldo Cruz Foundation, Ceara (FIOCRUZ CE)	Cleber Furtado Aksenen; Fabio Miyajima; Fernando Braga Stehling; Francisco Eder de Moura Lopes; Jamille Maria Mendes Bezerra; Joaquim Cesar do Nascimento Sousa Junior; Pedro Miguel Carneiro Jeronimo; Suzana Porto Almeida & Lucas Delerino on behalf of COVID-19 FIOCRUZ Genomic Network; Thais Ferreira de Oliveira; Thais de Oliveira Costa; Ticiane Cavalcante de Souza; Veridiana Pessoa Miyajima
EPI_ISL_3912450	UAPS PEDRO CELESTINO	Analytical Competence Molecular Epidemiology Lab/ACME, Oswaldo Cruz Foundation, Ceara (FIOCRUZ CE)	Cleber Furtado Aksenen; Fabio Miyajima; Fernando Braga Stehling; Francisco Eder de Moura Lopes; Jamille Maria Mendes Bezerra; Joaquim Cesar do Nascimento Sousa Junior; Pedro Miguel Carneiro Jeronimo; Suzana Porto Almeida & Lucas Delerino on behalf of COVID-19 FIOCRUZ Genomic Network; Thais Ferreira de Oliveira; Thais de Oliveira Costa; Ticiane Cavalcante de Souza; Veridiana Pessoa Miyajima
EPI_ISL_735419	UBS Alvarenga	Instituto Adolfo Lutz, Interdiciplinary Procedures Center, Strategic Laboratory	Claudia Regina Gonçalves; Claudio Tavares Sacchi; Erica Valessa Ramos Gomes; Karoline Rodrigues Campos
EPI_ISL_837053	UBS Darcy Alves e Robalinho	Instituto Adolfo Lutz, Interdiciplinary Procedures Center, Strategic Laboratory	Claudia Regina Gonçalves; Claudio Tavares Sacchi; Erica Valessa Ramos Gomes; Karoline Rodrigues Campos
EPI_ISL_735422	UBS Dematchi	Instituto Adolfo Lutz, Interdiciplinary Procedures Center, Strategic Laboratory	Claudia Regina Gonçalves; Claudio Tavares Sacchi; Erica Valessa Ramos Gomes; Karoline Rodrigues Campos
EPI_ISL_1966553	UBS II DE TANABI MILTON MARTINS PERCHES	Instituto Butantan / FZEA-USP (Pirassununga)	Antonio Jorge Martins; Bianca Cechetto Carlos. Mendelics: Bibiana Santos; Claudia Renata dos Santos Barros; Cíntia Bittar; David Schlesinger. Hemocentro Ribeirão Preto: Simone Kashima; Debora Botequio Moretti; Elaine Cristina Marqueze; Elaine Vieira dos Santos; Elisangela Chicaroni Mattos; Erika Freitas; Evandra Strazza Rodrigues; Felipe Allan da Silva da Costa; Flavia Aburjaile; Fábio Sossai Possebon; Guilherme Campos; Guilherme Targino Valente; Heidge Fukumasu. USP-Botucatu: Rejane Maria Tommasini Grotto; Helena Lage Ferreira; Instituto Butantan: Dimas Tadeu Covas; Jardelina de Souza Todao Bernardino; Jayme A. Souza-Neto; Jessika Cristina Chagas Lesbon; Jorge A. Petroli Marchesi; José Salvatore Leister Patané; João Paulo Kitajima; João Pessoa Araújo Jr.; Leila Sabrina Ullmann; Loyze Paola Oliveira de Lima; Luiz Aurelio de Campos Crispin. Centro de Genômica Funcional da ESALQ: Luiz Lehmann Coutinho; Luiz Carlos Junior de Alcantara; Lívia Sacchetto; Maisa C. Pereira Parra; Maria Carolina Elias; Marta Giovanetti; Marília Moraes; Maurício Lacerda Nogueira. Prefeitura de Sao Paulo: Melissa Palmieri.; Patricia Akemi Assato; Paula Rahal; Paulo Inacio da Costa; Rafael dos Santos Bezerra; Raquel de Lello Rocha Campos Cassano. NGS Soluções Genômicas: Pilar Drummond Sampaio Corrêa Mariani. FZEA-USP Pirassununga: Mirele Daiana Poleti; Raul Machado Neto; Ricardo Augusto Brassaloti; Ricardo Haddad; Rodrigo Tocantins Calado. FAMERP-SJRP: Cecília Artico Banho; Sandra Coccuzzo Sampaio; Svetoslav Nanev Slavov; Vagner Fonseca; Vincent Louis Viala
EPI_ISL_837054	UBS Jose Sabino Ferreira	Instituto Adolfo Lutz, Interdiciplinary Procedures Center, Strategic Laboratory	Claudia Regina Gonçalves; Claudio Tavares Sacchi; Erica Valessa Ramos Gomes; Karoline Rodrigues Campos
EPI_ISL_735421	UBS Sta Terezinha	Instituto Adolfo Lutz, Interdiciplinary Procedures Center, Strategic Laboratory	Claudia Regina Gonçalves; Claudio Tavares Sacchi; Erica Valessa Ramos Gomes; Karoline Rodrigues Campos
EPI_ISL_471648	UBS e Pronto Socorro Jd. Jacira	Instituto Adolfo Lutz, Interdiciplinary Procedures Center, Strategic Laboratory	Claudia Regina Gonçalves; Claudio Tavares Sacchi; Erica Valessa Ramos Gomes
EPI_ISL_2758653, EPI_ISL_2758655, EPI_ISL_2758659, EPI_ISL_2758660, EPI_ISL_2758663, EPI_ISL_2758664, EPI_ISL_2758665, EPI_ISL_2758666, EPI_ISL_2758680, EPI_ISL_2758689, EPI_ISL_2758690, EPI_ISL_2758691, EPI_ISL_2758697, EPI_ISL_2758721, EPI_ISL_2758751, EPI_ISL_2758753, EPI_ISL_2758755, EPI_ISL_2758766, EPI_ISL_2758777, EPI_ISL_2758779, EPI_ISL_2758783, EPI_ISL_2758784, EPI_ISL_2758785, EPI_ISL_2758788, EPI_ISL_2758792			
see above	UEL	IPEC Guarapuava	NAPI-Genômica (Novos Arranjo de Pesquisa e Inovação em Genômica): Ademar Dantas da Cunha Júnior Adriano Ferrasa Adriano Mondini Aldo Przybysz Alessandra Lourenço Cecchini Armani Alex Sandro Jorge Alexandra Ivo de Medeiros Alexandre Maller Aline Cristina Batista Rodrigues Johann Ana Lucia Ferreira Ana Marisa Fusco Almeida Anderson Joel Martino Andrade André Luís Laforga Vanzela Andrea Duarte Doetzer Andrea Name Colado Simao Andressa Pereira de Souza Anelisa Ramão Angelica Beate Winter Boldt Anna Hermínia Castro Gomes de Amorim Anna Silvia Penteado Setti da Rocha Antonio Camilo da Silva Filho Antonio Stabelini Neto Arthur Hirata Bertachi Barbara Mendes Paz Chao Betty Cristiane Kuhn Bruno Ambrozio Galindo Bruno Ribeiro Cruz Camilla Reginatto De Pierri Carla Fredrichsen Moya Araujo Carla Fredrichsen Moya Araujo Carlos Alberto Oliveira de Biagi Junior Carlos Augusto Nassar Carlos Eduardo Buss Carlos Gilberto Carlotti Junior Carlos Henrique Schneider Carolina Panis Carolina Weigert Galvão Caroline de Jesus Coelho Donha Caroline Guisantes de Salvo Toni Caryna Eurich Mazur Catiuscie Cabreira da Silva Tortorella Celso F. D. Doliveira Cesar Luiz Boguszewski Christiane Pienna Soares Chung Man Chin Claudia Moro Cleverson Busso Cristiane Cominetti Daiane Priscila Simão-Silva Dalila Luciola Zanette Daniel de Paula Daniel de Paula Daniel Rech Daniela Fiori Gradia Daniela Pretti da Cunha Tirapelli Daniela Viganó Zanoti Jeronymo Daniele Ukan Danielle Malheiros Ferreira Danielle Venturini Deborah Catharine de Assis Leite Deivid Calebe de Souza Dennis Armando Bertolini Edenir Inez Pamero Edna Maria Vissoci Reiche Edson Roberto Arpini Miguel Eduardo José de Almeida Araújo Eliana Carolina Vespero Eliandro Reis Tavares Elza Kimura Grimshaw Emanuel Maltempi de Souza Emanuele Cristina Gustani Buss Émerson Carraro Emiliana Cristina Melo ENILze Maria de Souza Fonseca Ribeiro Enilze Maria de Souza Fonseca Ribeiro Erika Izumi Erika Seki Kioshima Cotica Evani Marques Pereira Fabio Negretti Fábio Rodrigues Ferreira Seiva Felipe Dunin dos Santos Felipe Tuon Fernanda Andreia Rosa Fernanda Cestaro Prado Cortez Fernanda Ivanski Fernanda Maris Peria Flavia Regina Oliveira de Barros Franciele Aní Caovilla Follador Franciele Mara Lucca Zanardo Bohm Francinete Ramos Campos Fulviana Silva Nishiyama GABRIEL RIBEIRO CORDEIRO Gabriela Datsch Bennemann Gisele Santos de Oliveira Glaucio Valdameri Glauco Akelinghton Freire Vitiello Glauco Vieira Miranda Glaura Scantamburlo ALves Fernandes Guilherme Ferreira Silveira Gustavo Bianchini Porfírio Gustavo Lenci Marques Hélito Volpato Hildebrando Masshiro Nagai Huei Diana Lee Ilce Mara de Syllos Cólus Iris Rabinovich Israel Gomy Jackson Kawakami Jacques Duílio Brancher Jaime Luís Lopes Rocha Jaqueline Carvalho de Oliveira Jean Henrique da Silva Rodrigues Jean Leandro dos Santos Jeane Eliete Laguila Visentainer João Paulo Bianchi Ximenez Joaquim Manoel da Silva Jociani Ascari Joel Donazzolo Jorge Luis Maria Ruiz Jose Knopfholz José Luis da Conceição Silva José Sebastião dos Santos Joseane Carla Schabarum Juliana Cheleski Wiggers Juliana Mara Serpeloni Juliana Morini Küpper Cardoso Perseguini Karen Brajão de Oliveira Karin Braun Prado Karine Aparecida de Lima Katiany Rizzieri Caleffi Ferracioli Katiuscia de Oliveira Francisco Gabriel Kelvinson Fernandes Viana Larissa Beatriz Cossalter Larissa Danielle Bahls Pinto Laurival Antonio Vilas Boas Léia Carolina Lucio Líbero Mezzadri Neto Ligia Carla Faccin Galhardi Lirane Elize Defante Ferreto Luciana Furlaneto Maia Luciana Oliveira de Fariña Luciana Reis Azevedo Alanis Luciane Regina Cavalli Lucy Megumi Yamauchi Lioni Luis Paulo Gomes Mascarenhas Luis Paulo Gomes Mascarenhas Luís Paulo Mascarenhas Lupe Furtado Alle Lyvia Regina Biagi Silva Bertachi Mara Antonia Ramos Costa Mara L. Cordeiro Marcela Maria Birolim Marcelo Ricardo Vicari Marcia Edilaine Lopes Consolaro Marcia Holsbach Beltrame Marcia Regina Eches Perugini Marcos Abdo Arbex Marcos Pileggi MARCOS TADEU GRZELCZAK Marcus Peikriszwili Tartaruga Maria Angelica Ehara Watanabe Maria Antonia Ramos Costa Maria Claudia Gross Maria José Soares Mendes Giannini Maria Leandra Terencio Maria Lúcia Bonfleur Maria Luiza Guimarães de Oliveira Maria Luiza Petzl-Erler Mariana Abe Vicente Cavagnari Marina Kimiko Kadowaki Marise Fonseca dos Santos Marla Karine Amarante Maurício Turkiewicz Mauro Antonio Alves Castro Michel Rodrigo Zambrano Passarini Michele Potrich Michelle Orane Schemberger Milena Massumi Kozonoe Mônica Degraf Cavallin Monica Tereza Suldofski Mucio Luiz de Assis Cirino Nadia Graciele Krohn Najeh Maissar Khalil Nédia de Castilhos Ghisi Neide Tomimura Costa Neiva Leite Neyva Maria Lopes Romeiro Patrícia Amâncio da Rosa Patricia Dayane Carvalho Schaker Patricia Oehlmeyer Nassar Patrícia Savio de Araújo-Souza Patricia Silva Lucio Paulo Henrique Couto Souza Paulo Roberto Donadio Percy Nohama Quirino Alves de Lima Neto Rafael Deminice Rafael dos Santos Bezerra Raquel Alves dos Santos Renan Manozzo Galante Renata Ernlund Freitas de Macedo Rita de Cássia Garcia Simão Roberta Losi Guembarovski Roberto H. Herai Roberto Rosati Rodrigo Ferreira Rodrigo Rodrigues Matiello Rogério Neri Shinsato Rogério Pincela Mateus Rosane Aparecida Ribeiro Rosilene Fressatti Cardoso Rosilene Fressatti Cardoso Sandra Mara Guse Scós Venske Selene Elifio Esposito Sérgio Ossamu Ioshii Silvana Giuliatti Silvia Mara de Souza Halick Silvio Henrique Maia de Almeida Simone Neumann Wendt Spencer Luiz Marques Payão Stefan Wolanski Negrão Stephane Janaina de Moura Escobar Sueli Fumie Yamada Ogatta SUELI PERCIO QUINAIA Taciane Finatto Tatiana Mayumi Veiga Iriyoda Tayza Katelline Danilau Ostroski Tony Alexander Hild Valeria Valente Vanessa Nascimento Kozak Vanessa Santos Sotomaior Victor Breno Pedrosa Victoria Zeghbi Cochenski Borba Vivian Rotuno Moure Valdameri Wander Rogerio Pavanelli Weber Cláudio Francisco Nunes da Silva Willian Augusto de Melo Yohandra Reyes Torres
EPI_ISL_941550, EPI_ISL_941552	ULS Guarda	Instituto Nacional de Saude (INSA)	Borges et al
EPI_ISL_732179, EPI_ISL_732180, EPI_ISL_732181, EPI_ISL_732183, EPI_ISL_732184	ULS Guarda	Instituto Nacional de Saude (INSA) and Instituto Gulbenkian de Ciencia (IGC)	Borges et al

EPI_ISL_941583	ULS Litoral Alentejano	Instituto Nacional de Saude (INSA)	Borges et al
EPI_ISL_941896	ULSNE - Braganca	Instituto Nacional de Saude (INSA) and Instituto Gulbenkian de Ciencia (IGC)	Borges et al
EPI_ISL_1469719, EPI_ISL_1469733	UNIDADE DE PRONTO ATENDIMENTO DE SAPUCAIA DO SUL UPA	Epiclin	Ana Paula Muterle; Carolina Comerlato; Eliana Márcia Da Ros Wendland; Fernando Hayashi Sant'Anna; Janira Prichula; Juliana Comerlato
EPI_ISL_1469720, EPI_ISL_1469722	UNIDADE SANITARIA DE IGREJINHA	Epiclin	Ana Paula Muterle; Carolina Comerlato; Eliana Márcia Da Ros Wendland; Fernando Hayashi Sant'Anna; Janira Prichula; Juliana Comerlato
EPI_ISL_2209934	UNIDADE SENTINELA COVID19	Instituto Butantan	Antonio Jorge Martins; Claudia Renata dos Santos Barros; David Schlesinger; Debora Botequio Moretti; Dimas Tadeu Covas; Elaine Cristina Marqueze; Elaine Vieira Santos; Evandra Strazza Rodrigues; Heidge Fukumasu; Jayme Augusto de Souza-Neto; José Salvatore Leister Patané; Luiz Alcantara; Luiz Lehmann Coutinho; Maria Carolina Elias; Maurício Lacerda Nogueira; Rafael dos Santos Bezerra; Raul Machado Neto; Rejane Maria Tommasini Grotto; Ricardo Haddad; Sandra Coccuzzo Sampaio Vessoni; Simone Kashima; Svetoslav Nanev Slavov; Vincent Louis Viala.
EPI_ISL_1966752	UNIDADE SENTINELA COVID19	Instituto Butantan / Mendelics	Antonio Jorge Martins; Bianca Cechetto Carlos. Mendelics: Bibiana Santos; Claudia Renata dos Santos Barros; Cíntia Bittar; David Schlesinger. Hemocentro Ribeirão Preto: Simone Kashima; Debora Botequio Moretti; Elaine Cristina Marqueze; Elaine Vieira dos Santos; Elisangela Chicaroni Mattos; Erika Freitas; Evandra Strazza Rodrigues; Felipe Allan da Silva da Costa; Flavia Aburjaile; Fábio Sossai Possebon; Guilherme Campos; Guilherme Targino Valente; Heidge Fukumasu. USP-Botucatu: Rejane Maria Tommasini Grotto; Helena Lage Ferreira; Instituto Butantan: Dimas Tadeu Covas; Jardelina de Souza Todao Bernardino; Jayme A. Souza-Neto; Jessika Cristina Chagas Lesbon; Jorge A. Petroli Marchesi; José Salvatore Leister Patané; João Paulo Kitajima; João Pessoa Araújo Jr.; Leila Sabrina Ullmann; Loyze Paola Oliveira de Lima; Luiz Aurelio de Campos Crispin. Centro de Genômica Funcional da ESALQ: Luiz Lehmann Coutinho; Luiz Carlos Junior de Alcantara; Lívia Sacchetto; Maisa C. Pereira Parra; Maria Carolina Elias; Marta Giovanetti; Marília Moraes; Maurício Lacerda Nogueira. Prefeitura de Sao Paulo: Melissa Palmieri.; Patricia Akemi Assato; Paula Rahal; Paulo Inacio da Costa; Rafael dos Santos Bezerra; Raquel de Lello Rocha Campos Cassano. NGS Soluções Genômicas: Pilar Drummond Sampaio Corrêa Mariani. FZEA-USP Pirassununga: Mirele Daiana Poleti; Raul Machado Neto; Ricardo Augusto Brassaloti; Ricardo Haddad; Rodrigo Tocantins Calado. FAMERP-SJRP: Cecília Artico Banho; Sandra Coccuzzo Sampaio; Svetoslav Nanev Slavov; Vagner Fonseca; Vincent Louis Viala
EPI_ISL_2344658	UNIDADE SENTINELA COVID19	Instituto Butantan / UNESP-Botucatu	Antonio Jorge Martins; Claudia Renata dos Santos Barros; David Schlesinger; Debora Botequio Moretti; Dimas Tadeu Covas; Elaine Cristina Marqueze; Elaine Vieira Santos; Evandra Strazza Rodrigues; Heidge Fukumasu; Jayme Augusto de Souza-Neto; José Salvatore Leister Patané; Luiz Alcantara; Luiz Lehmann Coutinho; Maria Carolina Elias; Maurício Lacerda Nogueira; Rafael dos Santos Bezerra; Raul Machado Neto; Rejane Maria Tommasini Grotto; Ricardo Haddad; Sandra Coccuzzo Sampaio Vessoni; Simone Kashima; Svetoslav Nanev Slavov; Vincent Louis Viala
EPI_ISL_2105325	UP Health Services (PGC)	Philippine Genome Center	Alethea R. de Guzman; Anna Ong-Lim; Arianne A. Zamora; Asia Louisa U. Chong; Benedict A. Maralit; Candice Francheska B. Tambaoan; Carlo M. Lapid; Celia Carlos; Devon Ray Pacial; Edsel Maurice Salvaña; El King D. Morado; Eva Maria Cutiongco-de la Paz; Francis A. Tablizo; Irish Coleen A. Asin; Jaime C. Montoya; Jan Michael C. Yap; Jo-Hannah S. Llames; John Q. Wong; Joshua Gregor A. Dizon; Juan Antonio R. Magalang; Karol Sophia Agape R. Padilla; Kenneth M. Kim; Kris P. Punayan; Marc Edsel C. Ayes; Marc Jerrone R. Castro; Maria Rosario Singh-Vergeire and Cynthia P. Saloma; Maria Sofia L. Yangzon; Marissa Alejandria; Razel Nikka M. Hao; Rianna Patricia S. Cruz; Sheila Mae M. Araiza
EPI_ISL_523983	UPA Campo Limpo	Instituto Adolfo Lutz, Interdiciplinary Procedures Center, Strategic Laboratory	Claudia Regina Gonçalves; Claudio Tavares Sacchi; Erica Valessa Ramos Gomes
EPI_ISL_2346035	UPA DR FRANCO DA ROCHA	Instituto Butantan	Antonio Jorge Martins; Claudia Renata dos Santos Barros; David Schlesinger; Debora Botequio Moretti; Dimas Tadeu Covas; Elaine Cristina Marqueze; Elaine Vieira Santos; Evandra Strazza Rodrigues; Heidge Fukumasu; Jayme Augusto de Souza-Neto; José Salvatore Leister Patané; Luiz Alcantara; Luiz Lehmann Coutinho; Maria Carolina Elias; Maurício Lacerda Nogueira; Rafael dos Santos Bezerra; Raul Machado Neto; Rejane Maria Tommasini Grotto; Ricardo Haddad; Sandra Coccuzzo Sampaio Vessoni; Simone Kashima; Svetoslav Nanev Slavov; Vincent Louis Viala
EPI_ISL_1123372	UPA I Santa Isabel	Instituto Adolfo Lutz, Interdiciplinary Procedures Center, Strategic Laboratory	Caio Vinicius Dias Lopes; Claudia Regina Gonçalves; Claudio Tavares Sacchi; Erica Valessa Ramos Gomes; Karoline Rodrigues Campos
EPI_ISL_534311	UPA III 26 de Agosto	Instituto Adolfo Lutz, Interdiciplinary Procedures Center, Strategic Laboratory	Claudia Regina Gonçalves; Claudio Tavares Sacchi; Erica Valessa Ramos Gomes
EPI_ISL_583496	UPA Jandira	Instituto Adolfo Lutz, Interdiciplinary Procedures Center, Strategic Laboratory	Claudia Regina Gonçalves; Claudio Tavares Sacchi; Erica Valessa Ramos Gomes; Karoline Rodrigues Campos
EPI_ISL_693237, EPI_ISL_693245	UPA Santa Isabel	Instituto Adolfo Lutz, Interdiciplinary Procedures Center, Strategic Laboratory	Claudia Regina Gonçalves; Claudio Tavares Sacchi; Erica Valessa Ramos Gomes; Karoline Rodrigues Campos
EPI_ISL_523975, EPI_ISL_523980	UPA Tito Lopes	Instituto Adolfo Lutz, Interdiciplinary Procedures Center, Strategic Laboratory	Claudia Regina Gonçalves; Claudio Tavares Sacchi; Erica Valessa Ramos Gomes
EPI_ISL_468316	UPA Vila Assis	Instituto Adolfo Lutz, Interdiciplinary Procedures Center, Strategic Laboratory	Claudia Regina Gonçalves; Claudio Tavares Sacchi; Erica Valessa Ramos Gomes
EPI_ISL_861682	UPA Vila Santa Catarina	Instituto Adolfo Lutz, Interdiciplinary Procedures Center, Strategic Laboratory	Claudia Regina Gonçalves; Claudio Tavares Sacchi; Erica Valessa Ramos Gomes; Karoline Rodrigues Campos
EPI_ISL_861656	UPA de Jandira	Instituto Adolfo Lutz, Interdiciplinary Procedures Center, Strategic Laboratory	Claudia Regina Gonçalves; Claudio Tavares Sacchi; Erica Valessa Ramos Gomes; Karoline Rodrigues Campos
EPI_ISL_1445201, EPI_ISL_1966234	USAFA TUDE BASTOS	Instituto Butantan / Mendelics	Antonio Jorge Martins; Bianca Cechetto Carlos. Mendelics: Bibiana Santos; Bibiana Santos; Claudia Renata dos Santos Barros; Cíntia Bittar; David Schlesinger; David Schlesinger. Hemocentro Ribeirão Preto: Simone Kashima; Debora Botequio Moretti; Dimas Tadeu Covas; Elaine Cristina Marqueze; Elaine Vieira dos Santos; Elisangela Chicaroni Mattos; Erika Freitas; Evandra Strazza Rodrigues; Felipe Allan da Silva da Costa; Flavia Aburjaile; Fábio Sossai Possebon; Guilherme Campos; Guilherme Targino Valente; Heidge Fukumasu. USP-Botucatu: Rejane Maria Tommasini Grotto; Helena Lage Ferreira; Instituto Butantan: Dimas Tadeu Covas; Jardelina de Souza Todao Bernardino; Jayme A. Souza-Neto; Jessika Cristina Chagas Lesbon; Jorge A. Petroli Marchesi; José Salvatore Leister Patané; João Paulo Kitajima; João Pessoa Araújo Jr.; Leila Sabrina Ullmann; Loyze Paola Oliveira de Lima; Luiz Aurelio de Campos Crispin. Centro de Genômica Funcional da ESALQ: Luiz Lehmann Coutinho; Luiz Carlos Junior de Alcantara; Lívia Sacchetto; Maisa C. Pereira Parra; Maria Carolina Elias; Marta Giovanetti; Marília Moraes; Maurício Lacerda Nogueira. Prefeitura de Sao Paulo: Melissa Palmieri.; Patricia Akemi Assato; Paula Rahal; Paulo Inacio da Costa; Rafael dos Santos Bezerra; Raquel de Lello Rocha Campos Cassano. NGS Soluções Genômicas: Pilar Drummond Sampaio Corrêa Mariani. FZEA-USP Pirassununga: Mirele Daiana Poleti; Raul Machado Neto; Ricardo Augusto Brassaloti; Ricardo Haddad; Rodrigo Tocantins Calado.; Rodrigo Tocantins Calado. FAMERP-SJRP: Cecília Artico Banho; Sandra Coccuzzo Sampaio; Simone Kashima; Svetoslav Nanev Slavov; Vagner Fonseca; Vincent Louis Viala
EPI_ISL_1324137, EPI_ISL_1324140	UW Virology Lab	UW Virology Lab	Alexander Greninger; Hong Xie; Keith R Jerome; Lasata Shrestha; Margaret Mills; Meei-Li Huang; Michelle Lin; Noah Baker; Pavitra Roychoudhury; Saraswathi Sathees; Sean Ellis; Shah Mohamed Bakhash
EPI_ISL_734865	UZ Leuven, National Reference Laboratory for Coronaviruses, Laboratory Medicine, Leuven, Belgium	KU Leuven, Rega Institute, Clinical and Epidemiological Virology	Bert Vanmechelen; Joan Marti-Carerras; Piet Maes; Tony Wawina-Bokalanga
EPI_ISL_693225	Ubs Vila Rosa - Olímpia Gomes De Almeida	Instituto Adolfo Lutz, Interdiciplinary Procedures Center, Strategic Laboratory	Claudia Regina Gonçalves; Claudio Tavares Sacchi; Erica Valessa Ramos Gomes; Karoline Rodrigues Campos
EPI_ISL_735397	Unidade Respiratória Nova Hortolandia	Instituto Adolfo Lutz, Interdiciplinary Procedures Center, Strategic Laboratory	Claudia Regina Gonçalves; Claudio Tavares Sacchi; Erica Valessa Ramos Gomes; Karoline Rodrigues Campos
EPI_ISL_1469576	Unidade Sanitária de Igrejinha	Epiclin	Ana Paula Muterle; Carolina Comerlato; Eliana Márcia Da Ros Wendland; Fernando Hayashi Sant'Anna; Janira Prichula; Juliana Comerlato
EPI_ISL_1469664, EPI_ISL_1469714	Unidade de Atendimento DST AIDS TB e Han	Epiclin	Ana Paula Muterle; Carolina Comerlato; Eliana Márcia Da Ros Wendland; Fernando Hayashi Sant'Anna; Janira Prichula; Juliana Comerlato
EPI_ISL_574590	Unidade de Printo Atendimento UPA I Santa Isabel	Instituto Adolfo Lutz, Interdiciplinary Procedures Center, Strategic Laboratory	Claudia Regina Gonçalves; Claudio Tavares Sacchi; Erica Valessa Ramos Gomes; Karoline Rodrigues Campos

EPI_ISL_735409	Unidade de Pronto Atendimento Carlos Lourenco	Instituto Adolfo Lutz, Interdiciplinary Procedures Center, Strategic Laboratory	Claudia Regina Gonçalves; Claudio Tavares Sacchi; Erica Valessa Ramos Gomes; Karoline Rodrigues Campos
EPI_ISL_693214	Unidade de Pronto Atendimento Central de Caraguatatuba	Instituto Adolfo Lutz, Interdiciplinary Procedures Center, Strategic Laboratory	Claudia Regina Gonçalves; Claudio Tavares Sacchi; Erica Valessa Ramos Gomes; Karoline Rodrigues Campos
EPI_ISL_882665	Unidade de Pronto Atendimento Dra Zilda Arns	Instituto Adolfo Lutz, Interdiciplinary Procedures Center, Strategic Laboratory	Claudia Regina Gonçalves; Claudio Tavares Sacchi; Erica Valessa Ramos Gomes; Karoline Rodrigues Campos
EPI_ISL_693226	Unidade de Pronto Atendimento Sao José	Instituto Adolfo Lutz, Interdiciplinary Procedures Center, Strategic Laboratory	Claudia Regina Gonçalves; Claudio Tavares Sacchi; Erica Valessa Ramos Gomes; Karoline Rodrigues Campos
EPI_ISL_735406	Unidade de Pronto Atendimento UPA I Sta Isabel	Instituto Adolfo Lutz, Interdiciplinary Procedures Center, Strategic Laboratory	Claudia Regina Gonçalves; Claudio Tavares Sacchi; Erica Valessa Ramos Gomes; Karoline Rodrigues Campos
EPI_ISL_735417	Unidade de Pronto Atendimento de Agenor de Campos	Instituto Adolfo Lutz, Interdiciplinary Procedures Center, Strategic Laboratory	Claudia Regina Gonçalves; Claudio Tavares Sacchi; Erica Valessa Ramos Gomes; Karoline Rodrigues Campos
EPI_ISL_1469580	Unidade de Pronto Atendimento de Sapucaia do Sul	Epiclin	Ana Paula Muterle; Carolina Comerlato; Eliana Márcia Da Ros Wendland; Fernando Hayashi Sant'Anna; Janira Prichula; Juliana Comerlato
EPI_ISL_693216, EPI_ISL_693217	Unidade de Vigilância Epiudemiológica de Araras	Instituto Adolfo Lutz, Interdiciplinary Procedures Center, Strategic Laboratory	Claudia Regina Gonçalves; Claudio Tavares Sacchi; Erica Valessa Ramos Gomes; Karoline Rodrigues Campos
EPI_ISL_2491722	Universidade Federal do Sul da Bahia (UFSB)	Laboratory of Respiratory Viruses and Measles, Oswaldo Cruz Institute, FIOCRUZ	Alice Sampaio Rocha; Ana Carolina Mendonca; Anna Carolina Paixao; Elisa Cavalcante Pereira; Felicidade Pereira; Fernando Motta; Luciana Appolinario; Marilda Siqueira on behalf of the Fiocruz COVID-19 Genomic Surveillance Network; Paola Resende; Renata Serrano Lopes; Taina Venas; Thiago Mafra
EPI_ISL_873097	University of Michigan Clinical Microbiology Laboratory	Lauring Lab, University of Michigan, Department of Microbiology and Immunology	Valesano
EPI_ISL_2105391	University of the Philippines National Institutes of Health (UP NIH)	Philippine Genome Center	Alethea R. de Guzman; Anna Ong-Lim; Arianne A. Zamora; Asia Louisa U. Chong; Benedict A. Maralit; Candice Francheska B. Tambaoan; Carlo M. Lapid; Celia Carlos; Devon Ray Pacial; Edsel Maurice Salvaña; El King D. Morado; Eva Maria Cutiongco-de la Paz; Francis A. Tablizo; Irish Coleen A. Asin; Jaime C. Montoya; Jan Michael C. Yap; Jo-Hannah S. Llames; John Q. Wong; Joshua Gregor A. Dizon; Juan Antonio R. Magalang; Karol Sophia Agape R. Padilla; Kenneth M. Kim; Kris P. Punayan; Marc Edsel C. Ayes; Marc Jerrone R. Castro; Maria Rosario Singh-Vergeire and Cynthia P. Saloma; Maria Sofia L. Yangzon; Marissa Alejandria; Razel Nikka M. Hao; Rianna Patricia S. Cruz; Sheila Mae M. Araiza
EPI_ISL_1286592, EPI_ISL_1286611	Universitätsklinikum Heidelberg	Robert Koch Institute	
EPI_ISL_693234	Upa Vereador Jose Da Rocha Goncalves	Instituto Adolfo Lutz, Interdiciplinary Procedures Center, Strategic Laboratory	Claudia Regina Gonçalves; Claudio Tavares Sacchi; Erica Valessa Ramos Gomes; Karoline Rodrigues Campos
EPI_ISL_2284160	VA Connecticut Healthcare System	Yale Center for Genomic Analysis	Brooke Sullivan; Curt Scharfe; Irina Tikhonova; Kaya Bilguvar; Shrikant Mane
EPI_ISL_1469573	VIGILANCIA EM SAUDE NH	Epiclin	Ana Paula Muterle; Carolina Comerlato; Eliana Márcia Da Ros Wendland; Fernando Hayashi Sant'Anna; Janira Prichula; Juliana Comerlato
EPI_ISL_1445229, EPI_ISL_1445232, EPI_ISL_1445235, EPI_ISL_1445249, EPI_ISL_1445251, EPI_ISL_1445270, EPI_ISL_1966059, EPI_ISL_1966062, EPI_ISL_1966067, EPI_ISL_1966091, EPI_ISL_2170898			
see above	VIGILANCIA EPIDEMIOLOGICA	Instituto Butantan / Mendelics	Antonio Jorge Martins; Bianca Cechetto Carlos. Mendelics: Bibiana Santos; Bibiana Santos; Claudia Renata dos Santos Barros; Cíntia Bittar; David Schlesinger; David Schlesinger. Hemocentro Ribeirão Preto: Simone Kashima; Debora Botequio Moretti; Dimas Tadeu Covas; Elaine Cristina Marqueze; Elaine Vieira dos Santos; Elisangela Chicaroni Mattos; Erika Freitas; Evandra Strazza Rodrigues; Felipe Allan da Silva da Costa; Flavia Aburjaile; Fábio Sossai Possebon; Guilherme Campos; Guilherme Targino Valente; Heidge Fukumasu. USP-Botucatu: Rejane Maria Tommasini Grotto; Helena Lage Ferreira; Instituto Butantan: Dimas Tadeu Covas; Jardelina de Souza Todao Bernardino; Jayme A. Souza-Neto; Jessika Cristina Chagas Lesbon; Jorge A. Petroli Marchesi; José Salvatore Leister Patané; João Paulo Kitajima; João Pessoa Araújo Jr.; Leila Sabrina Ullmann; Loyze Paola Oliveira de Lima; Luiz Aurelio de Campos Crispin. Centro de Genômica Funcional da ESALQ: Luiz Lehmann Coutinho; Luiz Carlos Junior de Alcantara; Lívia Sacchetto; Maisa C. Pereira Parra; Maria Carolina Elias; Marta Giovanetti; Marília Moraes; Maurício Lacerda Nogueira. Prefeitura de Sao Paulo: Melissa Palmieri.; Patricia Akemi Assato; Paula Rahal; Paulo Inacio da Costa; Rafael dos Santos Bezerra; Raquel de Lello Rocha Campos Cassano. NGS Soluções Genômicas: Pilar Drummond Sampaio Corrêa Mariani. FZEA-USP Pirassununga: Mirele Daiana Poleti; Raul Machado Neto; Ricardo Augusto Brassaloti; Ricardo Haddad; Rodrigo Tocantins Calado.; Rodrigo Tocantins Calado. FAMERP-SJRP: Cecília Artico Banho; Sandra Coccuzzo Sampaio; Simone Kashima; Svetoslav Nanev Slavov; Vagner Fonseca; Vincent Louis Viala
EPI_ISL_2345734	VIGILANCIA EPIDEMIOLOGICA JARDINOPOLIS SP	Instituto Butantan / Mendelics	Antonio Jorge Martins; Claudia Renata dos Santos Barros; David Schlesinger; Debora Botequio Moretti; Dimas Tadeu Covas; Elaine Cristina Marqueze; Elaine Vieira Santos; Evandra Strazza Rodrigues; Heidge Fukumasu; Jayme Augusto de Souza-Neto; José Salvatore Leister Patané; Luiz Alcantara; Luiz Lehmann Coutinho; Maria Carolina Elias; Maurício Lacerda Nogueira; Rafael dos Santos Bezerra; Raul Machado Neto; Rejane Maria Tommasini Grotto; Ricardo Haddad; Sandra Coccuzzo Sampaio Vessoni; Simone Kashima; Svetoslav Nanev Slavov; Vincent Louis Viala
EPI_ISL_2105341	Valenzuela Hope Molecular Laboratory	Philippine Genome Center	Alethea R. de Guzman; Anna Ong-Lim; Arianne A. Zamora; Asia Louisa U. Chong; Benedict A. Maralit; Candice Francheska B. Tambaoan; Carlo M. Lapid; Celia Carlos; Devon Ray Pacial; Edsel Maurice Salvaña; El King D. Morado; Eva Maria Cutiongco-de la Paz; Francis A. Tablizo; Irish Coleen A. Asin; Jaime C. Montoya; Jan Michael C. Yap; Jo-Hannah S. Llames; John Q. Wong; Joshua Gregor A. Dizon; Juan Antonio R. Magalang; Karol Sophia Agape R. Padilla; Kenneth M. Kim; Kris P. Punayan; Marc Edsel C. Ayes; Marc Jerrone R. Castro; Maria Rosario Singh-Vergeire and Cynthia P. Saloma; Maria Sofia L. Yangzon; Marissa Alejandria; Razel Nikka M. Hao; Rianna Patricia S. Cruz; Sheila Mae M. Araiza
EPI_ISL_468313, EPI_ISL_468319, EPI_ISL_603033	Vigilancia Epidemiologica de São Bernardo do Campo	Instituto Adolfo Lutz, Interdiciplinary Procedures Center, Strategic Laboratory	Claudia Regina Gonçalves; Claudio Tavares Sacchi; Erica Valessa Ramos Gomes; Karoline Rodrigues Campos
EPI_ISL_515542	Vigilância Epidemiológica de Leme	Instituto Adolfo Lutz, Interdiciplinary Procedures Center, Strategic Laboratory	Claudia Regina Gonçalves; Claudio Tavares Sacchi; Erica Valessa Ramos Gomes
EPI_ISL_603023	Vigilância em Saúde Visa Sul	Instituto Adolfo Lutz, Interdiciplinary Procedures Center, Strategic Laboratory	Claudia Regina Gonçalves; Claudio Tavares Sacchi; Erica Valessa Ramos Gomes; Karoline Rodrigues Campos
EPI_ISL_547573	Vigilância em Saúde de Cajamar	Instituto Adolfo Lutz, Interdiciplinary Procedures Center, Strategic Laboratory	Claudia Regina Gonçalves; Claudio Tavares Sacchi; Erica Valessa Ramos Gomes; Karoline Rodrigues Campos
EPI_ISL_1469696	Vigilância em Saúde de Sapucaia do Sul	Epiclin	Ana Paula Muterle; Carolina Comerlato; Eliana Márcia Da Ros Wendland; Fernando Hayashi Sant'Anna; Janira Prichula; Juliana Comerlato
EPI_ISL_1004317	Viollier AG	Department of Biosystems Science and Engineering, ETH Zürich	Andrea Patrignani; Andreia Cabral de Gouvea; Catharine Aquino; Chaoran Chen; Christiane Beckmann; Christoph Noppen; David Dreifuss; Doris Popovic; Griffin White; Ivan Topolsky; Jay Tracy; Katharina Jahn; Lara Fuhrmann; Laura Neff; Lennart Opitz; Maria Domenica Moccia; Maurice Redondo; Niko Beerenwinkel; Noemie Santamaria de Souza; Olivier Kobel; Philipp Jablonski; Ralph Schlapbach; Sarah Nadeau; Simon Grüter; Sophie Seidel; Tanja Stadler; Timothy Sykes
EPI_ISL_478249	Virology Department, Royal Infirmary of Edinburgh, NHS Lothian / School of Biological Sciences, University of Edinburgh / Institute of Genetics and Molecular	COVID-19 Genomics UK (COG-UK) Consortium	Balcaza C; Colquhoun R; Dewar R; Gallagher M; Hill V; Jackson B; McCrone JT; McHugh M; O'Toole Á; Rambaut A; Rooke S; Scher E; Templeton K; Williams TC; Yu X

	Medicine, University of Edinburgh		
EPI_ISL_1968604, EPI_ISL_2273882, EPI_ISL_2273938, EPI_ISL_2283150, EPI_ISL_2283162, EPI_ISL_2283229, EPI_ISL_2402835, EPI_ISL_2402881, EPI_ISL_2450673, EPI_ISL_2450676			
see above	Weill Cornell Medicine	New York Genome Center	Andre Corvelo; Arryn Craney; Chris Mason; Dayna M. Oschwald; Hanna Rennert; Lars F Westblade; Margaret Elizabeth Ross; Melissa Cushing; Michael Zody; Olivier Elemento; Priya Velu; Samantha Fennessey; Tom Maniatis
EPI_ISL_461606, EPI_ISL_461678, EPI_ISL_473651, EPI_ISL_478094, EPI_ISL_493851, EPI_ISL_493868	West of Scotland Specialist Virology Centre, NHSGGC / MRC-University of Glasgow Centre for Virus Research	COVID-19 Genomics UK (COG-UK) Consortium	Alasdair MacLean; Alice Broos; Ana da Silva Filipe; Antonia Ho; Daniel Mair; David L Robertson; Elihu Aranday-Cortes; Emma Thomson; James Shepherd; Jenna Nichols; Joseph Hughes; Kathy Li; Kathy Smollett; Kirstyn Brunker; Kyriaki Nomikou; Lily Tong; Marc Niebel; Natasha Jesudason; Natasha Johnson; Patawee Asamaphan; Rajiv Shah; Richard Orton; Rory Gunson; Sarah McDonald; Sreenu Vattipally; Stephen Carmichael; Yasmin Parr
EPI_ISL_803346	Wisconsin State Laboratory of Hygiene Communicable Disease Division	Wisconsin State Laboratory of Hygiene Communicable Disease Division	Abigail C. Shockey; Kelsey R. Florek
EPI_ISL_486429	unknown	Clinical Laboratory, Hospital Israelita Albert Einstein	Amgarten, D.; C.L. and Pinho; F.G.; Guedes; J.R.; Malta, F.; Mangueira; R.A.; R.L.; Santana; de Menezes
EPI_ISL_458140, EPI_ISL_458141, EPI_ISL_458146, EPI_ISL_458147, EPI_ISL_524783, EPI_ISL_524785, EPI_ISL_524786, EPI_ISL_524787, EPI_ISL_848562, EPI_ISL_848563, EPI_ISL_848565, EPI_ISL_848566, EPI_ISL_848571, EPI_ISL_848582, EPI_ISL_848583, EPI_ISL_848585, EPI_ISL_848587, EPI_ISL_848588, EPI_ISL_848589, EPI_ISL_848590, EPI_ISL_848592, EPI_ISL_848593, EPI_ISL_848595, EPI_ISL_848611, EPI_ISL_848615, EPI_ISL_848617, EPI_ISL_848618, EPI_ISL_848619, EPI_ISL_848620, EPI_ISL_848621, EPI_ISL_848622, EPI_ISL_848623, EPI_ISL_848624, EPI_ISL_848628, EPI_ISL_918518			
see above	Evandro Chagas Institute	Evandro Chagas Institute	A.M.; Barbagelata; E.C.; E.M.A.; Ferreira; G.M.R; H.R; J.A.; Junior; K.C.; L.C.; L.S.; M.C.; Martins; P.S.; Pinheiro; Resque; Santos; Silva; Sousa; Sousa Junior; Viana; W.D.C.; da Silva

We gratefully acknowledge the following Authors from the Originating laboratories responsible for obtaining the specimens, as well as the Submitting laboratories where the genome data were generated and shared via GISAID, on which this research is based.

All Submitters of data may be contacted directly via www.gisaid.org

Authors are sorted alphabetically.

Accession ID	Originating Laboratory	Submitting Laboratory	Authors
EPI_ISL_4060359	"OK Public Health Laboratory, Oklahoma State DOH"	Centers for Disease Control and Prevention Division of Viral Diseases, Pathogen Discovery	Alex Burgin; Ben Rambo-Martin; Clinton Paden; Dakota Howard; Dave Wentworth; Dhwani Batra; Jasmine Padilla; Justin Lee; Krista Queen; Kristen Knipe; Kristine Lacek; Mark Burroughs; Matthew Schmerer; Meghan Bentz; Mili Sheth; Peter Cook; Sam Shepard; Sarah Nobles; Suxiang Tong; Vivien Dugan; Yvette Unoarumhi
EPI_ISL_3813952	"PA Department of Health, Bureau of Laboratories"	Centers for Disease Control and Prevention Division of Viral Diseases, Pathogen Discovery	Alex Burgin; Ben Rambo-Martin; Clinton Paden; Dakota Howard; Dave Wentworth; Dhwani Batra; Jasmine Padilla; Justin Lee; Krista Queen; Kristen Knipe; Kristine Lacek; Mark Burroughs; Matthew Schmerer; Meghan Bentz; Mili Sheth; Peter Cook; Sam Shepard; Sarah Nobles; Suxiang Tong; Vivien Dugan; Yvette Unoarumhi
EPI_ISL_3062381	A.S.L. ROMA 2 - SISP	Department of General Diagnostics; Department of Virology; Istituto Zooprofilattico Sperimentale del Lazio e della Toscana (IZSLT)	Alessia Franco; Antonella Cersini; Antonio Battisti.; Elena L. Diaconu; Fabiola Feltrin; Giuseppe Manna; Patricia Alba; Raffaela Conti; Teresa Scicluna; Virginia Carfora
EPI_ISL_3638943	AREA DE SALUD ACOSTA	Inciensa, Instituto Costarricense de Investigación y Enseñanza en Nutrición y Salud	Adriana Godínez; Claudio Soto-Garita; Estela Cordero; Francisco Duarte; Hebleen Porras; José Luis Vargas; Mariela Gutiérrez & Joselyn Prado; Melany Calderón
EPI_ISL_3638752	AREA DE SALUD ALAJUELITA	Inciensa, Instituto Costarricense de Investigación y Enseñanza en Nutrición y Salud	Adriana Godínez; Claudio Soto-Garita; Estela Cordero; Francisco Duarte; Hebleen Porras; José Luis Vargas; Mariela Gutiérrez & Joselyn Prado; Melany Calderón
EPI_ISL_3638775, EPI_ISL_3948510	AREA DE SALUD ASERRI	Inciensa, Instituto Costarricense de Investigación y Enseñanza en Nutrición y Salud	Adriana Godínez; Claudio Soto-Garita; Estela Cordero; Francisco Duarte; Hebleen Porras; José Luis Vargas; Mariela Gutiérrez & Joselyn Prado; Melany Calderón
EPI_ISL_3639056	AREA DE SALUD BAGACES	Inciensa, Instituto Costarricense de Investigación y Enseñanza en Nutrición y Salud	Adriana Godínez; Claudio Soto-Garita; Estela Cordero; Francisco Duarte; Hebleen Porras; Joselyn Prado & Adriana Bermúdez; José Luis Vargas; Mariela Gutiérrez; Melany Calderón
EPI_ISL_3298361, EPI_ISL_3948509	AREA DE SALUD CARTAGO	Inciensa, Instituto Costarricense de Investigación y Enseñanza en Nutrición y Salud	Adriana Godínez; Claudio Soto-Garita; Estela Cordero; Francisco Duarte; Hebleen Porras; Joselyn Prado & Monserrat Segura; Joselyn Prado & Moserrat Segura; José Luis Vargas; Mariela Gutiérrez; Melany Calderón
EPI_ISL_3948523	AREA DE SALUD CAÑAS	Inciensa, Instituto Costarricense de Investigación y Enseñanza en Nutrición y Salud	Adriana Godínez; Claudio Soto-Garita; Estela Cordero; Francisco Duarte; Hebleen Porras; Joselyn Prado & Adriana Bermúdez; José Luis Vargas; Mariela Gutiérrez; Melany Calderón
EPI_ISL_3638917	AREA DE SALUD GOICOECHEA 2 - CLINICA DR. JIMENEZ NUÑEZ	Inciensa, Instituto Costarricense de Investigación y Enseñanza en Nutrición y Salud	Adriana Godínez; Claudio Soto-Garita; Estela Cordero; Francisco Duarte; Hebleen Porras; Joselyn Prado & Juan Carlos Cartes; José Luis Vargas; Mariela Gutiérrez; Melany Calderón
EPI_ISL_3638970	AREA DE SALUD GOLFITO	Inciensa, Instituto Costarricense de Investigación y Enseñanza en Nutrición y Salud	Adriana Godínez; Claudio Soto-Garita; Estela Cordero; Francisco Duarte; Hebleen Porras; Joselyn Prado & Eduardo Blanco; José Luis Vargas; Mariela Gutiérrez; Melany Calderón
EPI_ISL_3639111	AREA DE SALUD GUATUSO	Inciensa, Instituto Costarricense de Investigación y Enseñanza en Nutrición y Salud	Adriana Godínez; Claudio Soto-Garita; Estela Cordero; Francisco Duarte; Hebleen Porras; Joselyn Prado & Francisco Chacón; José Luis Vargas; Mariela Gutiérrez; Melany Calderón
EPI_ISL_3948491	AREA DE SALUD HATILLO - CLINICA DR. SOLON NUÑEZ	Inciensa, Instituto Costarricense de Investigación y Enseñanza en Nutrición y Salud	Adriana Godínez; Claudio Soto-Garita; Estela Cordero; Francisco Duarte; Hebleen Porras; Joselyn Prado & Roberto Brilla; José Luis Vargas; Mariela Gutiérrez; Melany Calderón
EPI_ISL_3948531	AREA DE SALUD HEREDIA-VIRILLA	Inciensa, Instituto Costarricense de Investigación y Enseñanza en Nutrición y Salud	Adriana Godínez; Claudio Soto-Garita; Estela Cordero; Francisco Duarte; Hebleen Porras; José Luis Vargas; Mariela Gutiérrez & Joselyn Prado; Melany Calderón
EPI_ISL_3639114	AREA DE SALUD LA UNION	Inciensa, Instituto Costarricense de Investigación y Enseñanza en Nutrición y Salud	Adriana Godínez; Claudio Soto-Garita; Estela Cordero; Francisco Duarte; Hebleen Porras; Joselyn Prado & Mónica Charpentier; José Luis Vargas; Mariela Gutiérrez; Melany Calderón
EPI_ISL_3464549, EPI_ISL_3948486	AREA DE SALUD MATA REDONDA-HOSPITAL - CLINICA DR. MORENO CAÑAS	Inciensa, Instituto Costarricense de Investigación y Enseñanza en Nutrición y Salud	Adriana Godínez; Claudio Soto-Garita; Estela Cordero; Francisco Duarte; Hebleen Porras; Joselyn Prado & Ricardo González; Joselyn Prado & Ricardo González Cascante; José Luis Vargas; Mariela Gutiérrez; Melany Calderón
EPI_ISL_3638959	AREA DE SALUD MONTES DE ORO	Inciensa, Instituto Costarricense de Investigación y Enseñanza en Nutrición y Salud	Adriana Godínez; Claudio Soto-Garita; Estela Cordero; Francisco Duarte; Hebleen Porras; Joselyn Prado & María José Gómez; José Luis Vargas; Mariela Gutiérrez; Melany Calderón
EPI_ISL_3639026, EPI_ISL_3948496, EPI_ISL_3948506	AREA DE SALUD OREAMUNO-PACAYAS-TIERRA BLANCA	Inciensa, Instituto Costarricense de Investigación y Enseñanza en Nutrición y Salud	Adriana Godínez; Claudio Soto-Garita; Estela Cordero; Francisco Duarte; Hebleen Porras; Joselyn Prado & Carolina Loría; José Luis Vargas; Mariela Gutiérrez; Melany Calderón
EPI_ISL_3638947	AREA DE SALUD PAQUERA	Inciensa, Instituto Costarricense de Investigación y Enseñanza en Nutrición y Salud	Adriana Godínez; Claudio Soto-Garita; Estela Cordero; Francisco Duarte; Hebleen Porras; Joselyn Prado & Ronald Núñez; José Luis Vargas; Mariela Gutiérrez; Melany Calderón
EPI_ISL_3639075	AREA DE SALUD PAVAS (COOPESALUD)	Inciensa, Instituto Costarricense de Investigación y Enseñanza en Nutrición y Salud	Adriana Godínez; Claudio Soto-Garita; Estela Cordero; Francisco Duarte; Hebleen Porras; José Luis Vargas; Mariela Gutiérrez & Joselyn Prado; Melany Calderón
EPI_ISL_3639166	AREA DE SALUD SANTA ANA (COOPESANA)	Inciensa, Instituto Costarricense de Investigación y Enseñanza en Nutrición y Salud	Adriana Godínez; Claudio Soto-Garita; Estela Cordero; Francisco Duarte; Hebleen Porras; José Luis Vargas; Mariela Gutiérrez & Joselyn Prado; Melany Calderón
EPI_ISL_3638874, EPI_ISL_3948483	AREA DE SALUD SIQUIRRES	Inciensa, Instituto Costarricense de Investigación y Enseñanza en Nutrición y Salud	Adriana Godínez; Claudio Soto-Garita; Estela Cordero; Francisco Duarte; Hebleen Porras; José Luis Vargas; Mariela Gutiérrez & Joselyn Prado; Melany Calderón
EPI_ISL_3464548	AREA DE SALUD TURRIALBA-JIMENEZ	Inciensa, Instituto Costarricense de Investigación y Enseñanza en Nutrición y Salud	Adriana Godínez; Claudio Soto-Garita; Estela Cordero; Francisco Duarte; Hebleen Porras; Joselyn Prado & Mónica Charpentier Artavia; José Luis Vargas; Mariela Gutiérrez; Melany Calderón
EPI_ISL_3126848	ASST FBF Sacco	Laboratory of Clinical Microbiology, Virology and Bioemergencies, ASST Fatebenefratelli Sacco - Sacco Hospital	Valeria Micheli
EPI_ISL_3905332, EPI_ISL_3905334, EPI_ISL_3905349, EPI_ISL_3905355, EPI_ISL_3905359	AUSTRAL-omics, UACh	AUSTRAL-omics, UACh	Andrea Silva; Carolina Encina; Cristian Molina; Daniela Plaza; Luis Guzmán; Suany Quesada
EPI_ISL_2965605, EPI_ISL_2965612, EPI_ISL_2965614, EPI_ISL_2965618, EPI_ISL_2965622, EPI_ISL_2965629, EPI_ISL_3047420, EPI_ISL_3047481, EPI_ISL_3120265, EPI_ISL_3241830, EPI_ISL_3254986, EPI_ISL_3254988			
see above	AZDelta	AZ Delta Medical Laboratories in Roeselare, Belgium	Dieter De Smet; Frederik Van Hoecke; Geert Martens; Merijn Vanhee; on behalf of AZ Delta COVID-19 Genomics core (member of Genomic surveillance of SARS-CoV-2 in Belgium network)
EPI_ISL_3218448, EPI_ISL_3219960, EPI_ISL_3220023, EPI_ISL_3220055, EPI_ISL_3220252, EPI_ISL_3220296, EPI_ISL_3305499, EPI_ISL_3321321, EPI_ISL_3321430, EPI_ISL_3321538, EPI_ISL_3321571, EPI_ISL_3321615, EPI_ISL_3321698, EPI_ISL_3321960, EPI_ISL_3321977, EPI_ISL_3322059, EPI_ISL_3322142, EPI_ISL_3322214, EPI_ISL_3322410, EPI_ISL_3322447, EPI_ISL_3322542, EPI_ISL_3322669, EPI_ISL_3322823, EPI_ISL_3322950, EPI_ISL_3323038, EPI_ISL_3323175, EPI_ISL_3323222, EPI_ISL_3323407, EPI_ISL_3323495, EPI_ISL_3323715, EPI_ISL_3323716, EPI_ISL_3323724, EPI_ISL_3323743, EPI_ISL_3323817, EPI_ISL_3323927, EPI_ISL_3324046, EPI_ISL_3324127, EPI_ISL_3324215, EPI_ISL_3324410, EPI_ISL_3324544, EPI_ISL_3324821, EPI_ISL_3324885, EPI_ISL_3325057, EPI_ISL_3325227,			

EPI_ISL_3430033, EPI_ISL_3430185, EPI_ISL_3430191, EPI_ISL_3430233, EPI_ISL_3430365, EPI_ISL_3430438, EPI_ISL_3430545, EPI_ISL_3430701, EPI_ISL_3430703, EPI_ISL_3812948, EPI_ISL_3813212, EPI_ISL_3813278, EPI_ISL_3813308, EPI_ISL_3813456, EPI_ISL_3813472, EPI_ISL_3814408, EPI_ISL_3815183, EPI_ISL_3815189, EPI_ISL_3815200, EPI_ISL_3815203, EPI_ISL_3815484, EPI_ISL_3816025, EPI_ISL_3816060, EPI_ISL_3816564, EPI_ISL_3816690, EPI_ISL_3816702, EPI_ISL_3816733, EPI_ISL_3818302, EPI_ISL_3818609, EPI_ISL_3818636, EPI_ISL_3818660, EPI_ISL_3818819, EPI_ISL_3818994, EPI_ISL_3819082, EPI_ISL_3819561, EPI_ISL_3819995, EPI_ISL_3821296, EPI_ISL_3821558, EPI_ISL_3821669, EPI_ISL_3822499, EPI_ISL_3822715, EPI_ISL_3822867, EPI_ISL_3842668, EPI_ISL_3842790, EPI_ISL_3843072, EPI_ISL_3843074, EPI_ISL_3843076, EPI_ISL_3843079, EPI_ISL_3843081, EPI_ISL_3843083, EPI_ISL_3843088, EPI_ISL_3844428, EPI_ISL_3844584, EPI_ISL_3845171, EPI_ISL_3845238, EPI_ISL_3845370, EPI_ISL_3845396, EPI_ISL_3845852, EPI_ISL_3846130, EPI_ISL_3846340, EPI_ISL_3846638, EPI_ISL_3847406, EPI_ISL_3847734, EPI_ISL_3848009, EPI_ISL_3848251, EPI_ISL_3849901, EPI_ISL_3849909, EPI_ISL_3851079, EPI_ISL_3851199, EPI_ISL_3851863, EPI_ISL_3852806, EPI_ISL_3853010, EPI_ISL_3853618, EPI_ISL_3853911, EPI_ISL_3855192, EPI_ISL_3855436, EPI_ISL_3856611, EPI_ISL_3856616, EPI_ISL_3856672, EPI_ISL_3858368, EPI_ISL_3858383, EPI_ISL_3859123, EPI_ISL_3859582, EPI_ISL_3861401, EPI_ISL_3862132, EPI_ISL_3862573, EPI_ISL_3864154, EPI_ISL_4057062, EPI_ISL_4059095, EPI_ISL_4059273			
see above	Aegis Sciences Corporation	Centers for Disease Control and Prevention Division of Viral Diseases, Pathogen Discovery	Adrian Paskey; Alec Vest; Benjamin Rambo-Martin; Christopher Gulvick; Clinton Paden; Clinton R. Paden; Cyndi Clark; Dakota Howard; Darlene Wagner; Dhwani Batra; Dillon Nall; Duncan MacCannell; Ethan Sanders; Holly Houdeshell; Jason Caravas; Kara Moser; Matthew Hardison; Matthew Schmerer; Ola Kvalvaag; Patrick Campbell; Peter Cook; Peter W. Cook; Rob Case; Scott Sammons; Shatavia Morrison; Shaun Westlund; Vikramsinha Ghorpade; Yvette Unoarumhi
EPI_ISL_3388728	Alverno Labs	RIPHL at Rush University Medical Center	Edith Perez; Felix Araujo Perez; Kevin Kunstman; Laura Furtado; Marieta Hyde; Max Kolton; Melissa Mace; Stefan Green
EPI_ISL_3089191, EPI_ISL_3089196, EPI_ISL_3089199, EPI_ISL_3089200, EPI_ISL_3089201, EPI_ISL_3089202, EPI_ISL_3089203, EPI_ISL_3090065, EPI_ISL_3216770, EPI_ISL_3217106, EPI_ISL_3217130, EPI_ISL_3217144, EPI_ISL_3262733, EPI_ISL_3262749, EPI_ISL_3262760, EPI_ISL_3319874			
see above	Arizona State Public Health Laboratory	Arizona State Public Health Laboratory	Jessica Escobar; Katherine Fullerton; Linda Getsinger; Matthew Contursi; Nobuko Fukushima; Stacy White; Trung Huynh; Victor Waddell
EPI_ISL_3260474, EPI_ISL_3493438	Arizona State University	Arizona State University	Ajeet Bains; Efrem S. Lim; Joshua LaBaer; LaRinda A. Holland; Matthew F. Smith; Nathaniel Johnson; Nicholas J. Mellor; Peter T. Skidmore; Rabia Maqsood; Vel Murugan
EPI_ISL_3553476	Arkansas Children's Hospital	Center for Global Health, University of New Mexico Health Sciences Center	Ashton Ingold; Bobby Boyanton; Darrell Dinwiddie; Daryl Domman; Joshua L. Kennedy; Kurt Schwalm; Valerie Morley
EPI_ISL_3099255	Arkansas Public Health Laboratory, Arkansas Department of Health	University of Minnesota Genomics Center	Corbin Dirkx; Daryl M. Gohl; Jaquelyn Kuriger-Laber; John Garbe
EPI_ISL_3307684, EPI_ISL_3797192	Austrian Agency for Health and Food Safety (AGES)	Bergthaler laboratory, CeMM Research Center for Molecular Medicine of the Austrian Academy of Sciences	Andreas Bergthaler; Anna Schedl; Bekir Erguner; Benedikt Agerer; Christoph Bock; Fabian Amman; Jan Laine; Lukas Endler; Maelle Le Moing; Martin Senekowitsch; Matthew Thornton; Michael Schuster; Petr Triska; Thomas Penz
EPI_ISL_3912132	BANCO DE OLHOS DO CEARA	Analytical Competence Molecular Epidemiology Lab/ACME, Oswaldo Cruz Foundation, Ceara (FIOCRUZ CE)	Cleber Furtado Aksenen; Fabio Miyajima; Fernando Braga Stehling; Francisco Eder de Moura Lopes; Jamille Maria Mendes Bezerra; Joaquim Cesar do Nascimento Sousa Junior; Pedro Miguel Carneiro Jeronimo; Suzana Porto Almeida & Lucas Delerino on behalf of COVID-19 FIOCRUZ Genomic Network; Thais Ferreira de Oliveira; Thais de Oliveira Costa; Ticiane Cavalcante de Souza; Veridiana Pessoa Miyajima
EPI_ISL_3579326	BIOFAST	Instituto Butantan	Antonio Jorge Martins; Claudia Renata dos Santos Barros; David Schlesinger; Debora Botequio Moretti; Dimas Tadeu Covas; Elaine Cristina Marqueze; Elaine Vieira Santos; Evandra Strazza Rodrigues; Heidge Fukumasu; Jayme Augusto de Souza-Neto; José Salvatore Leister Patané; Luiz Alcantara; Luiz Lehmann Coutinho; Maria Carolina Elias; Maurício Lacerda Nogueira; Rafael dos Santos Bezerra; Raul Machado Neto; Rejane Maria Tommasini Grotto; Ricardo Haddad; Sandra Coccuzzo Sampaio Vessoni; Simone Kashima; Svetoslav Nanev Slavov; Vincent Louis Viala
EPI_ISL_3835033	BOSTON HEART DIAGNOSTICS CORP	Wadsworth Center, New York State Department of Health	Alexis Russell; Catharine Prussing; Daryl M. Lamson; Erasmus Schneider; Erica Lasek-Nesselquist; John Kelly; Jonathan Plitnick; Kirsten St. George; Matthew Shudt; Melissa A Leisner; Navjot Singh
EPI_ISL_3087039, EPI_ISL_3087088, EPI_ISL_3214808	Baylor Scott & White-Temple	Baylor Scott & White-Temple	Ari Rao; Kimberly Walker; Linden Morales; Marcus Volz; Shelby Johnson
EPI_ISL_3980440, EPI_ISL_3981426	Biomédicos de Mérida	Biomédicos de Mérida	CANCHE-PECH JOSE REYES; CAUICH-VERA KRISTIAN; CEBALLOS-LOPEZ ADRIAN; CHIN-HAU LETICIA; GARCIA-GONZALEZ IGRID; LOPEZ-NOVELO MARIA E; MENDOZA-OLIVEROS TAHALI; SAURI-MEDINA EMILY
EPI_ISL_3084623, EPI_ISL_3084663, EPI_ISL_3084906, EPI_ISL_3155849, EPI_ISL_3407759, EPI_ISL_3407760, EPI_ISL_3408337, EPI_ISL_3431226			
see above	Broad Institute Clinical Research Sequencing Platform	Infectious Disease Program, Broad Institute of Harvard and MIT	Adams, G.; B.L.; B.W.; Bauer, M.; Birren; Blumenstiel, B.; Brown, C.; Carter, A.; Chaluvadi, S.; D.J.; DeFelice, M.; DeRuff, K.; Dodge, S.; Gabriel, S.; Gallagher, G.; Gladden-Young, A.; Granger, B.; J.E.; K.J.; Lagerborg, K.; Larkin, K.; Lee, M.; Lemieux; Lennon, N.; Loreth, C.; Madoff, L.; McGovern, S.; Meldrim, J.; Normandin, E.; P.C.; Park; Pearlman, L.; Reilly, S.; Rudy, M.; Sabeti; Siddle; Smole, S.; Tomkins-Tinch, C.; Vicente, G.; and MacInnis
EPI_ISL_3723154	CDPH VBL	California Department of Public Health	Emily Smith on behalf of CDPH-COVIDNet and UCI Genome Sciences Center/GHTF
EPI_ISL_3536165	CENTRO DE ATENCAO ESPECIALIZADA	Analytical Competence Molecular Epidemiology Lab/ACME, Oswaldo Cruz Foundation, Ceara (FIOCRUZ CE)	Cleber Furtado Aksenen; Fabio Miyajima; Fernando Braga Stehling; Francisco Eder de Moura Lopes; Jamille Maria Mendes Bezerra; Joaquim César do Nascimento Sousa Junior; Pedro Miguel Carneiro Jeronimo; Suzana Porto Almeida e Lucas Delerino; Thais Ferreira de Oliveira; Thais de Oliveira Costa; Ticiane Cavalcante de Souza; Veridiana Pessoa Miyajima
EPI_ISL_3536353, EPI_ISL_3912134, EPI_ISL_3912135, EPI_ISL_3912136, EPI_ISL_3912137	CENTRO DE ATENDIMENTO PARA ENFRENTAMENTO AO COVID 19	Analytical Competence Molecular Epidemiology Lab/ACME, Oswaldo Cruz Foundation, Ceara (FIOCRUZ CE)	Cleber Furtado Aksenen; Fabio Miyajima; Fernando Braga Stehling; Francisco Eder de Moura Lopes; Jamille Maria Mendes Bezerra; Joaquim Cesar do Nascimento Sousa Junior; Joaquim César do Nascimento Sousa Junior; Pedro Miguel Carneiro Jeronimo; Suzana Porto Almeida & Lucas Delerino on behalf of COVID-19 FIOCRUZ Genomic Network; Suzana Porto Almeida e Lucas Delerino; Thais Ferreira de Oliveira; Thais de Oliveira Costa; Ticiane Cavalcante de Souza; Veridiana Pessoa Miyajima
EPI_ISL_3696640, EPI_ISL_3696644, EPI_ISL_3696647, EPI_ISL_3696659, EPI_ISL_3696668, EPI_ISL_3696752, EPI_ISL_3696769, EPI_ISL_3696788, EPI_ISL_3696808			
see above	CENTRO DE ENFRENTAMENTO COVID	Instituto Butantan	Antonio Jorge Martins; Claudia Renata dos Santos Barros; David Schlesinger; Debora Botequio Moretti; Dimas Tadeu Covas; Elaine Cristina Marqueze; Elaine Vieira Santos; Evandra Strazza Rodrigues; Heidge Fukumasu; Jayme Augusto de Souza-Neto; José Salvatore Leister Patané; Luiz Alcantara; Luiz Lehmann Coutinho; Maria Carolina Elias; Maurício Lacerda Nogueira; Rafael dos Santos Bezerra; Raul Machado Neto; Rejane Maria Tommasini Grotto; Ricardo Haddad; Sandra Coccuzzo Sampaio Vessoni; Simone Kashima; Svetoslav Nanev Slavov; Vincent Louis Viala
EPI_ISL_3922299	CENTRO DE SAUDE II DR GABRIEL MESQUITA VARGEM GDE DO SUL	Instituto Butantan	Antonio Jorge Martins; Claudia Renata dos Santos Barros; David Schlesinger; Debora Botequio Moretti; Dimas Tadeu Covas; Elaine Cristina Marqueze; Elaine Vieira Santos; Evandra Strazza Rodrigues; Heidge Fukumasu; Jayme Augusto de Souza-Neto; José Salvatore Leister Patané; Luiz Alcantara; Luiz Lehmann Coutinho; Maria Carolina Elias; Maurício Lacerda Nogueira; Rafael dos Santos Bezerra; Raul Machado Neto; Rejane Maria Tommasini Grotto; Ricardo Haddad; Sandra Coccuzzo Sampaio Vessoni; Simone Kashima; Svetoslav Nanev Slavov; Vincent Louis Viala
EPI_ISL_3131368	CERBALLIANCE MIDI-PYRENEES	CNR Virus des Infections Respiratoires - France SUD	Antonin Bal; Bruno Lina; Gregory Destras; Gwendolyne Burfin; Hadrien Regue; Laurence Josset; Martine Valette; Quentin Semanas
EPI_ISL_3269615, EPI_ISL_3269616, EPI_ISL_3269617, EPI_ISL_3269618, EPI_ISL_3398100, EPI_ISL_3547308, EPI_ISL_3547309, EPI_ISL_3547311			
see above	CH de l'Ouest Guyanais Frank Joly	Institut Pasteur de la Guyane	Anne Lavergne; Antoine Enfissi; Arielle Salmier; Dominique Rousset
EPI_ISL_3190118, EPI_ISL_3190121, EPI_ISL_3269602, EPI_ISL_3269603, EPI_ISL_3269604, EPI_ISL_3269605, EPI_ISL_3269612, EPI_ISL_3269613, EPI_ISL_3277483, EPI_ISL_3370958, EPI_ISL_3370970, EPI_ISL_3370973, EPI_ISL_3398115, EPI_ISL_3398116, EPI_ISL_3501660			
see above	CHC Andrée Rosemon	Institut Pasteur de la Guyane	Anne Lavergne; Antoine Enfissi; Arielle Salmier; Dominique Rousset
EPI_ISL_3758167, EPI_ISL_3758168, EPI_ISL_3758169, EPI_ISL_3758171, EPI_ISL_3758178, EPI_ISL_3758179, EPI_ISL_3758181, EPI_ISL_3758184, EPI_ISL_3758185, EPI_ISL_3758188, EPI_ISL_3758189, EPI_ISL_3758191, EPI_ISL_3758192, EPI_ISL_3758194, EPI_ISL_3825455, EPI_ISL_3825456, EPI_ISL_3825458, EPI_ISL_3825460, EPI_ISL_3825462, EPI_ISL_3825469, EPI_ISL_4061622, EPI_ISL_4061624, EPI_ISL_4061634, EPI_ISL_4061641, EPI_ISL_4061644, EPI_ISL_4061645			
see above	CHECK-UP - Medicina e Diagnóstico - Matriz	HLAGYN - Laboratorio de Imunologia de Transplantes de Goias	Elaize Maria Gomes de Paula; Fernando Antonio Vinhal dos Santos; Frederico Rodrigues Vinhal; Gladstone Rodrigues da Cunha Filho; Kamila Oliveira Reis De Freitas; Lucas Carlos Gomes Pereira; Nubia Silva Araújo; Sabrina Sara Moreira Duarte
EPI_ISL_3804696, EPI_ISL_3838197, EPI_ISL_3866870, EPI_ISL_3866877	CHU NIMES	CHU NIMES	Agathe Boudet; Marie-Josée Carles; Sophie Bravo; Stephan Robin
EPI_ISL_3234582, EPI_ISL_3471631	CHU Purpan - Laboratoire de Virologie - Institut Fédératif de Biologie	CHU Purpan - Laboratoire de Virologie - Institut Fédératif de Biologie	Bulach T.; Donnadieu C.; Izopet J.; Latour J.; Milhes M.; Nicot F.; Ranger N.; Salin G.; Tremeaux P.
EPI_ISL_3131351	CHU TOULOUSE	CNR Virus des Infections Respiratoires - France SUD	Antonin Bal; Bruno Lina; Gregory Destras; Gwendolyne Burfin; Hadrien Regue; Laurence Josset; Martine Valette; Quentin Semanas
EPI_ISL_3267802, EPI_ISL_3267825	CHUV	Laboratory of genomics and metagenomics	Claire Bertelli; Damien Jacot; Gilbert Greub; Sébastien Aeby; Trestan Pillonel
EPI_ISL_3387077	CLILAB	Microbiology Department	Aida Gonzalez-Diaz; Carmen Ardanuy; Jordi Camara; Jordi Niubó; Laura Calatayud; M Angeles Domínguez; Sara Marti; Yolanda Hernandez
EPI_ISL_3129963, EPI_ISL_3129965, EPI_ISL_3190132, EPI_ISL_3190133, EPI_ISL_3269608, EPI_ISL_3277486, EPI_ISL_3277487, EPI_ISL_3277488, EPI_ISL_3277489, EPI_ISL_3277492, EPI_ISL_3277493, EPI_ISL_3277494, EPI_ISL_3277496, EPI_ISL_3370962, EPI_ISL_3398104, EPI_ISL_3398107, EPI_ISL_3547310, EPI_ISL_3547312			
see above	CNR Institut Pasteur de la Guyane	Institut Pasteur de la Guyane	Anne Lavergne; Antoine Enfissi; Arielle Salmier; Dominique Rousset
EPI_ISL_3131720	CNR Virus des Infections Respiratoires - France SUD	CNR Virus des Infections Respiratoires - France SUD	Antonin Bal; Bruno Lina; Gregory Destras; Gwendolyne Burfin; Hadrien Regue; Laurence Josset; Martine Valette; Quentin Semanas
EPI_ISL_3570212, EPI_ISL_3570218	Center for Laboratory Medicine	Center for Laboratory Medicine	Yannick Gerth
EPI_ISL_3505620, EPI_ISL_3505630	Center for Research on Health in Latin America	Institute of Microbiology, Universidad San Francisco de Quito	Belén Prado-Vivar; Bernardo Gutiérrez; Erika Muñoz; Fernanda Zurita; Gabriel Trueba; Jaime Costales; Juan José Guadalupe; Michelle Grunauer; Monica Becerra-Wong; Patricio Rojas-Silva; Paúl Cárdenas; Sully Márquez; Verónica Barragán

EPI_ISL_3539226, EPI_ISL_3539227, EPI_ISL_3539228, EPI_ISL_3802961	Central Laboratory of Public Health of Bahia State (LACEN/BA)	Laboratory of Respiratory Viruses and Measles, Oswaldo Cruz Institute, FIOCRUZ	Agatha Soares; Alice Sampaio Rocha; Ana Carolina Mendonca; Anna Carolina Paixao; Elisa Cavalcante Pereira; Felicidade Pereira; Fernando Motta; Ighor Arantes; Luciana Appolinario; Marilda Siqueira on behalf of the Fiocruz COVID-19 Genomic Surveillance Network; Paola Resende; Renata Serrano Lopes; Taina Venas
EPI_ISL_3462621, EPI_ISL_3462696, EPI_ISL_3462697, EPI_ISL_3462698, EPI_ISL_3462699, EPI_ISL_3462700, EPI_ISL_3462701, EPI_ISL_3462702, EPI_ISL_3462703, EPI_ISL_3462704, EPI_ISL_3462705, EPI_ISL_3462706, EPI_ISL_3462707, EPI_ISL_3462708, EPI_ISL_3462709, EPI_ISL_3462710, EPI_ISL_3462711, EPI_ISL_3462712, EPI_ISL_3462713, EPI_ISL_3462714, EPI_ISL_3462715, EPI_ISL_3462716, EPI_ISL_3462717, EPI_ISL_3462718, EPI_ISL_3462719, EPI_ISL_3462720, EPI_ISL_3462721, EPI_ISL_3462722, EPI_ISL_3462723, EPI_ISL_3462724, EPI_ISL_3462725, EPI_ISL_3462726, EPI_ISL_3462729, EPI_ISL_3462730, EPI_ISL_3462731, EPI_ISL_3464734, EPI_ISL_4003116, EPI_ISL_4003120, EPI_ISL_4003121, EPI_ISL_4003122, EPI_ISL_4003123, EPI_ISL_4003125, EPI_ISL_4003126, EPI_ISL_4003127, EPI_ISL_4003129, EPI_ISL_4003132, EPI_ISL_4003135, EPI_ISL_4003137, EPI_ISL_4003141, EPI_ISL_4003142, EPI_ISL_4003143, EPI_ISL_4003144, EPI_ISL_4003145, EPI_ISL_4003146, EPI_ISL_4003147, EPI_ISL_4003148, EPI_ISL_4003151, EPI_ISL_4003152, EPI_ISL_4003153, EPI_ISL_4003155, EPI_ISL_4003156			
see above	Central Laboratory, Bureau of Public Health (BOG) and Academic Hospital Paramaribo	Erasmus Medical Center	Bas B Oude Munnink; Cherise Beek; Consuella Partowidjojo; Dion Gajadin; Ed PF IJzerman; Emmanuelle Munger; Gary Gummels; Ingrid SK Krishnadath; Lycke Woittiez; Marion PG Koopmans; Mireille Van de Veer; Phyllis Pinas; Princes Wongsowidjojo; Radjesh Ori; Ranisha Doerbalie; Rohma Banwari; Soeradj Harkisoen; Stephen Vreden; Tilotmadebie Ramlal; Verne Nanhoe
EPI_ISL_3635659, EPI_ISL_3635660, EPI_ISL_3635661, EPI_ISL_3635662, EPI_ISL_3635663, EPI_ISL_3635664, EPI_ISL_3635665, EPI_ISL_3635666, EPI_ISL_3635667, EPI_ISL_3635668, EPI_ISL_3635669, EPI_ISL_3635670, EPI_ISL_3635671, EPI_ISL_3635672, EPI_ISL_3635673, EPI_ISL_3635674, EPI_ISL_3944572, EPI_ISL_3944574, EPI_ISL_3944577, EPI_ISL_3944578, EPI_ISL_3944579, EPI_ISL_3944580, EPI_ISL_3944581, EPI_ISL_3944582, EPI_ISL_3944583, EPI_ISL_3944585, EPI_ISL_3944586, EPI_ISL_3944587, EPI_ISL_3944588, EPI_ISL_3944589, EPI_ISL_3944590, EPI_ISL_3944591, EPI_ISL_3944594, EPI_ISL_3944595, EPI_ISL_3944596, EPI_ISL_3944597, EPI_ISL_3944598, EPI_ISL_3944599, EPI_ISL_3944600, EPI_ISL_3944601, EPI_ISL_3944602, EPI_ISL_3944603, EPI_ISL_3944604, EPI_ISL_3944605, EPI_ISL_3944606, EPI_ISL_3944607, EPI_ISL_3944608, EPI_ISL_3944609, EPI_ISL_3944610, EPI_ISL_3944611, EPI_ISL_3944612, EPI_ISL_3944613, EPI_ISL_3944614, EPI_ISL_3944616, EPI_ISL_3944617, EPI_ISL_3944618, EPI_ISL_3944619, EPI_ISL_3944620, EPI_ISL_3944621, EPI_ISL_3944622, EPI_ISL_3944623, EPI_ISL_3944624, EPI_ISL_3944625, EPI_ISL_3944627			
see above	Central Public Health Laboratory - LACEN - Bahia, Salvador, Brazil	Central Public Health Laboratory - LACEN -Bahia, Salvador, Brazil	Arabela Leal; Felicidade Pereira; Gabriela Menezes; Jaqueline Gomes; Lenisa Dandara; Luciana Oliveira; Luiz Alcantara; Marcela Gómez; Marta Giovanetti; Stephane Tosta; Vagner Fonseca; Vanessa Nardy
EPI_ISL_3129967, EPI_ISL_3129969, EPI_ISL_3129970	Centre Hospitalier de l'Ouest Guyanais	Institut Pasteur de la Guyane	Anne Lavergne; Antoine Enfissi; Arielle Salmier; Dominique Rousset
EPI_ISL_3805729	Centro de Investigacion Biomedica del Noreste (CIBIN)	Unidad de Genomica Avanzada	; Alejandra Garcia-Gasca; Alejandra Hernandez-Teran; Alejandro Sanchez-Flores; Alfredo Herrera-Estrella; Alicia Ocaña-Mondragon; Andreu Comas-Garcia; Angel Gustavo Salas-Lais; Antonio Loza Roman; Bernardo Martinez-Miguel; Blanca Taboada; Brenda Irasema Maldonado-Meza; Bruno Gomez-Gil; Carla Ivon Herrera-Najera; Carlos F. Arias; Celia Boukadida; Celida Duque Molina; Celida Martinez- Rodriguez; Clara Esperanza Santacruz-Tinoco; Concepcion Grajales-Muñiz; Consorcio Mexicano de Vigilancia Genomica (CoViGen-Mex). Authors (in alphabetical order): Julio Elias Alvarado-Yaah; Cristobal Chaidez-Quiroz; Daniel Fregoso-Rueda; Daniel Lira Morales; Eduardo Becerril-Vargas; Fernando Fontove-Herrera; Fidencio Mejia-Nepomuceno; Francisco Pulido; Gloria Elena Espinosa-Ayala; Gloria Maria Molina-Salinas; Gloria Vazquez; Hector Esteban Paz-Juarez; Hector Montoya-Fuentes; Helen Haydee Fernanda Ramirez-Plascencia; Irvin Gonzalez-Lopez; Jean Pierre Gonzalez; Jesus Hernandez; Joel Armando Vazquez-Perez.; Jorge Salas-Hernandez; Jose Antonio Enciso-Moreno; Jose Arturo Martinez-Orozco; Jose Esteban Muñoz-Medina; Jose de Jesus Nuñez-Contreras; Juan Bautista Chale-Dzul; Julissa Enciso-Ibarra; Luis Alberto Ochoa-Carrera; Margarita Matias-Florentino; Maria Guadalupe Santiago-Mauricio; Maria Guadalupe de Jesus Mireles-Rivera; Mario Mujica-Sanchez; Marissa Perez-Garcia; Nelly Selem-Mojica; Pavel Isa; Ricardo Ciria Merce; Ricardo Grande; Rosa Maria Gutierrez Rios; Santiago avila-Rios; Selene Zarate; Susana Lopez; Veronica Mata-Haro; Victor Eduardo Garcia-Arias; Victor Hugo Borja-Aburto
EPI_ISL_3557185, EPI_ISL_3557225	Centro de Investigación Biomédica de Occidente (CIBO)	Centro de Investigación en Enfermedades Infecciosas (CIENI), Instituto Nacional de Enfermedades Respiratorias (INER)	Alejandra García-Gasca; Alejandra Hernández-Terán; Alejandro Sánchez-Flores; Alfredo Herrera-Estrella; Alicia Ocaña-Mondragón; Andreu Comas-García; Angel Gustavo Salas-Lais; Antonio Loza Román; Bernardo Martínez-Miguel; Blanca Taboada; Brenda Irasema Maldonado-Meza; Bruno Gómez-Gil; Carla Ivón Herrera-Najera; Carlos F. Arias; Celia Boukadida; Clara Esperanza Santacruz-Tinoco; Concepción Grajales-Muñiz; Consorcio Mexicano de Vigilancia Genómica (CoViGen-Mex). Authors (in alphabetical order): Julio Elias Alvarado-Yaah; Cristóbal Cháidez-Quiróz; Célida Duque Molina; Célida Martínez- Rodríguez; Daniel Fregoso-Rueda; Daniel Lira Morales; Eduardo Becerril-Vargas; Fernando Fontove-Herrera; Fidencio Mejía-Nepomuceno; Francisco Pulido; Gloria Elena Espinosa-Ayala; Gloria María Molina-Salinas; Gloria Vazquez; Hector Esteban Paz-Juárez; Hector Montoya-Fuentes; Helen Haydee Fernanda Ramírez-Plascencia; Irvin González-López; Jean Pierre González; Jesús Hernández; Joel Armando Vázquez-Pérez.; Jorge Salas-Hernández; José Antonio Enciso-Moreno; José Arturo Martínez-Orozco; José Esteban Muñoz-Medina; José de Jesús Nuñez-Contreras; Juan Bautista Chale-Dzul; Julissa Enciso-Ibarra; Kathia Elizabeth Tapia-Diaz; Luis Alberto Ochoa-Carrera; Margarita Matías-Florentino; Mario Mújica-Sánchez; Marissa Perez-Garcia; María Guadalupe Santiago-Mauricio; María Guadalupe de Jesús Mireles-Rivera; Nelly Sélem-Mojica; Pavel Isa; Ricardo Ciria Merce; Ricardo Grande; Rosa María Gutiérrez Rios; Santiago Ávila-Ríos; Selene Zárate; Susana Lopez; Verónica Mata-Haro; Victor Eduardo García-Arias; Víctor Hugo Borja-Aburto
EPI_ISL_3347554	Centro de Investigación Biomédica de Occidente (CIBO)	Instituto de Biotecnología de la UNAM	; Alejandra García-Gasca; Alejandra Hernández-Terán; Alejandro Sánchez-Flores; Alfredo Herrera-Estrella; Alicia Ocaña-Mondragón; Andreu Comas-García; Angel Gustavo Salas-Lais; Antonio Loza Román; Bernardo Martínez-Miguel; Blanca Taboada; Brenda Irasema Maldonado-Meza; Bruno Gómez-Gil; Carla Ivón Herrera-Najera; Carlos F. Arias; Celia Boukadida; Clara Esperanza Santacruz-Tinoco; Concepción Grajales-Muñiz; Consorcio Mexicano de Vigilancia Genómica (CoViGen-Mex). Authors (in alphabetical order): Julio Elias Alvarado-Yaah; Cristóbal Cháidez-Quiróz; Célida Duque Molina; Célida Martínez- Rodríguez; Daniel Fregoso-Rueda; Daniel Lira Morales; Eduardo Becerril-Vargas; Fernando Fontove-Herrera; Fidencio Mejía-Nepomuceno; Francisco Pulido; Gloria Elena Espinosa-Ayala; Gloria María Molina-Salinas; Gloria Vazquez; Hector Esteban Paz-Juárez; Hector Montoya-Fuentes; Helen Haydee Fernanda Ramírez-Plascencia; Irvin González-López; Jean Pierre González; Jesús Hernández; Joel Armando Vázquez-Pérez).; Jorge Salas-Hernández; José Antonio Enciso-Moreno; José Arturo Martínez-Orozco; José Esteban Muñoz-Medina; José de Jesús Nuñez-Contreras; Juan Bautista Chale-Dzul; Julissa Enciso-Ibarra; Kathia Elizabeth Tapia-Diaz; Luis Alberto Ochoa-Carrera; Margarita Matías-Florentino; Mario Mújica-Sánchez; Marissa Perez-Garcia; María Guadalupe Santiago-Mauricio; María Guadalupe de Jesús Mireles-Rivera; Nelly Sélem-Mojica; Pavel Isa; Ricardo Ciria Merce; Ricardo Grande; Rosa María Gutiérrez Rios; Santiago Ávila-Ríos; Selene Zárate; Susana Lopez; Verónica Mata-Haro; Victor Eduardo García-Arias; Víctor Hugo Borja-Aburto
EPI_ISL_3556954	Centro de Investigación Biomédica del Noreste (CIBIN)	Centro de Investigación en Enfermedades Infecciosas (CIENI), Instituto Nacional de Enfermedades Respiratorias (INER)	Alejandra García-Gasca; Alejandra Hernández-Terán; Alejandro Sánchez-Flores; Alfredo Herrera-Estrella; Alicia Ocaña-Mondragón; Andreu Comas-García; Angel Gustavo Salas-Lais; Antonio Loza Román; Bernardo Martínez-Miguel; Blanca Taboada; Brenda Irasema Maldonado-Meza; Bruno Gómez-Gil; Carla Ivón Herrera-Najera; Carlos F. Arias; Celia Boukadida; Clara Esperanza Santacruz-Tinoco; Concepción Grajales-Muñiz; Consorcio Mexicano de Vigilancia Genómica (CoViGen-Mex). Authors (in alphabetical order): Julio Elias Alvarado-Yaah; Cristóbal Cháidez-Quiróz; Célida Duque Molina; Célida Martínez- Rodríguez; Daniel Fregoso-Rueda; Daniel Lira Morales; Eduardo Becerril-Vargas; Fernando Fontove-Herrera; Fidencio Mejía-Nepomuceno; Francisco Pulido; Gloria Elena Espinosa-Ayala; Gloria María Molina-Salinas; Gloria Vazquez; Hector Esteban Paz-Juárez; Hector Montoya-Fuentes; Helen Haydee Fernanda Ramírez-Plascencia; Irvin González-López; Jean Pierre González; Jesús Hernández; Joel Armando Vázquez-Pérez.; Jorge Salas-Hernández; José Antonio Enciso-Moreno; José Arturo Martínez-Orozco; José Esteban Muñoz-Medina; José de Jesús Nuñez-Contreras; Juan Bautista Chale-Dzul; Julissa Enciso-Ibarra; Kathia Elizabeth Tapia-Diaz; Luis Alberto Ochoa-Carrera; Margarita Matías-Florentino; Mario Mújica-Sánchez; Marissa Perez-Garcia; María Guadalupe Santiago-Mauricio; María Guadalupe de Jesús Mireles-Rivera; Nelly Sélem-Mojica; Pavel Isa; Ricardo Ciria Merce; Ricardo Grande; Rosa María Gutiérrez Rios; Santiago Ávila-Ríos; Selene Zárate; Susana Lopez; Verónica Mata-Haro; Victor Eduardo García-Arias; Víctor Hugo Borja-Aburto
EPI_ISL_3347532	Centro de Investigación Biomédica del Noreste (CIBIN)	Instituto de Biotecnología de la UNAM	; Alejandra García-Gasca; Alejandra Hernández-Terán; Alejandro Sánchez-Flores; Alfredo Herrera-Estrella; Alicia Ocaña-Mondragón; Andreu Comas-García; Angel Gustavo Salas-Lais; Antonio Loza Román; Bernardo Martínez-Miguel; Blanca Taboada; Brenda Irasema Maldonado-Meza; Bruno Gómez-Gil; Carla Ivón Herrera-Najera; Carlos F. Arias; Celia Boukadida; Clara Esperanza Santacruz-Tinoco; Concepción Grajales-Muñiz; Consorcio Mexicano de Vigilancia Genómica (CoViGen-Mex). Authors (in alphabetical order): Julio Elias Alvarado-Yaah; Cristóbal Cháidez-Quiróz; Célida Duque Molina; Célida Martínez- Rodríguez; Daniel Fregoso-Rueda; Daniel Lira Morales; Eduardo Becerril-Vargas; Fernando Fontove-Herrera; Fidencio Mejía-Nepomuceno; Francisco Pulido; Gloria Elena Espinosa-Ayala; Gloria María Molina-Salinas; Gloria Vazquez; Hector Esteban Paz-Juárez; Hector Montoya-Fuentes; Helen Haydee Fernanda Ramírez-Plascencia; Irvin González-López; Jean Pierre González; Jesús Hernández; Joel Armando Vázquez-Pérez).; Jorge Salas-Hernández; José Antonio Enciso-Moreno; José Arturo Martínez-Orozco; José Esteban Muñoz-Medina; José de Jesús Nuñez-Contreras; Juan Bautista Chale-Dzul; Julissa Enciso-Ibarra; Kathia Elizabeth Tapia-Diaz; Luis Alberto Ochoa-Carrera; Margarita Matías-Florentino; Mario Mújica-Sánchez; Marissa Perez-Garcia; María Guadalupe Santiago-Mauricio; María Guadalupe de Jesús Mireles-Rivera; Nelly Sélem-Mojica; Pavel Isa; Ricardo Ciria Merce; Ricardo Grande; Rosa María Gutiérrez Rios; Santiago Ávila-Ríos; Selene Zárate; Susana Lopez; Verónica Mata-Haro; Victor Eduardo García-Arias; Víctor Hugo Borja-Aburto
EPI_ISL_3983576	City of Milwaukee Health Department Laboratory	City of Milwaukee Health Department Laboratory	Amy Bauer; Manjeet Khubbar; Nandu Balakrishnan; Samantha Scott; Sanjib Bhattacharyya
EPI_ISL_3076828, EPI_ISL_3076837	Clinical Microbiology, Infection Prevention and Control	Section for Molecular Diagnostics	Björn Hallström; Jonas Björkman
EPI_ISL_3092377	Cotugno	TIGEM	Antonio Grimaldi Patrizia Annunziata Francesco Panariello Biancamaria Pierri Claudia Tiberio Teresa Giuliano Valentina Bouche Chiara Colantuono Maria Concetta Cuomo Denise Di Concilio Lucio Di Filippo Anna Manfredi Marcello Salvi Antonio Limone Luigi Atripaldi Pellegrino Cerino Andrea Ballabio Davide Cacchiarelli
EPI_ISL_3920461	DC Public Health Lab/ Dept. of Forensic Sciences	DC Public Health Lab/ Dept. of Forensic Sciences	Brittany Hamilton; Connie Maza; Elizabeth Zelaya; Janis Doss; Jocelyn Hauser; Monica Mann; Nathan Bruns; Sarah Scott; Scott Nguyen
EPI_ISL_3841265, EPI_ISL_3841266, EPI_ISL_3841267, EPI_ISL_3841268, EPI_ISL_3841269, EPI_ISL_3841279, EPI_ISL_3841300, EPI_ISL_3841301, EPI_ISL_3841302, EPI_ISL_3841303, EPI_ISL_3841304, EPI_ISL_3841309, EPI_ISL_3841310			
see above	DNAGYN	LGBio (Laboratorio de Genetica & Biodiversidade)	Alex Honda Bernardes; Amanda Alves de Melo; Aparecido Divino da Cruz; Cintia Pelegrineti Targueta de Azevedo Brito; Daniela de Melo e Silva; Elisangela de Paula Silveira Lacerda; Juliana Santana de Curcio; Luiz Augusto Pereira; Marc Alexandre Duarte Gigonzac; Mariana Pires de Campos Telles; Ramilla dos Santos Braga; Renata de Oliveira Dias; Rhewter Nunes; Thais Cidália Vieira Gigonzac; Thais Guimarães Castro; Thays Millena Alves Pedroso
EPI_ISL_3692711	DPHL	Delaware Public Health Lab	Rebecca Savage
EPI_ISL_3644513, EPI_ISL_4093606, EPI_ISL_4093614	Department of Virology and Immunology, University of Helsinki and Helsinki University Hospital, Huslab Finland	Department of Virology, Faculty of Medicine, University of Helsinki, Helsinki, Finland	Essi Korhonen; Hanna Jarva; Hanna Liimatainen; Hannimari Kallio-Kokko; Harri Kangas; Hussein Alburkat; Jenni Virtanen; Maija Lappalainen; Maija Suvanto; Olli Vapalahti; Pekka Ellonen; Phuoc Truong; Ravi Kant; Sari Hannula; Satu Kurkela; Teemu Smura
EPI_ISL_3160431, EPI_ISL_3404737	Dianalabs SA	Genesupport	Geraldine Jost; Katia Jaton; Nadia Liassine; Tanguy ARAUD
EPI_ISL_3372586, EPI_ISL_3372600, EPI_ISL_3372604	Dirección regional de salud del Callao (DIRESA-CALLAO)	Centro de Investigaciones Tecnológicas, Biomédicas y Medioambientales (CITBM)	B; Huaman; J. Alarcon; M. Cuellar; M. Ramirez; M. Sovero
EPI_ISL_3369223, EPI_ISL_3369226, EPI_ISL_3369229	Division of Emerging Infectious Diseases, Bureau of Infectious Diseases Diagnosis Control, Korea Disease Control and Prevention Agency	Division of Emerging Infectious Diseases, Bureau of Infectious Diseases Diagnosis Control, Korea Disease Control and Prevention Agency	Ae Kyung Park; Chae Young Lee; Eun-Jin Kim; Heui Man Kim; Il-Hwan Kim; Jeong-Ah Kim
EPI_ISL_4076118, EPI_ISL_4076448, EPI_ISL_4077052	Dutch COVID-19 response team	National Institute for Public Health and the Environment (RIVM)	Adam Meijer; AnneMarie van den Brandt; Annelies Kroneman; Bas van der Veer; Chantal Reusken; Dennis Schmitz; Dirk Eggink; Euníce Then; Florian Zwagemaker; Harry Vennema; Ivo van Walle; Jeroen Cremer; Karim Hajji; Kim Freriks; Lisa Wijsman; Lynn Aarts; Ryanne Jaarsma; Sanne Bos; Sharon van den Brink; Stijn van Rossum; on behalf of the national COVID-19 response team
EPI_ISL_3459623	EL CAMINO HOSPITAL	Santa Clara County Public Health Laboratory	Santa Clara County Public Health Department
EPI_ISL_3692162, EPI_ISL_3692329, EPI_ISL_3937038, EPI_ISL_3937241	EXCITE Lab	Andersen lab at Scripps Research	Abigail Schnapper; Angela Scioscia; Art Mendoza; Cathy Woerle; Chip Schooley; David Pride; Helena Tubb; Jacquelyn Berumen; Liam McGinnis; Natasha Martin Cheryl Anderson; Omid Bakhtar; SEARCH Alliance San Diego with Aaron Harding; Sawyer Farmer; Sharon Reed; Tommy Valles + SEARCH
EPI_ISL_4054617	Emory Molecular Diagnostics Laboratory, Emory Healthcare	Piantadosi Lab, Emory Department of Pathology	Ahmed Babiker; Anne Piantadosi; Ludy Registre Carmola
EPI_ISL_3902824, EPI_ISL_3903286, EPI_ISL_3903287, EPI_ISL_3903289, EPI_ISL_3920890, EPI_ISL_3920891	Florida Bureau of Public Health Laboratories	Florida Bureau of Public Health Laboratories	Jason Blanton; Jiaqi Li; Namratha Tarigopula; Sarah Schmedes

EPI_ISL_3064782, EPI_ISL_3064893, EPI_ISL_3064897, EPI_ISL_3064963, EPI_ISL_3208534, EPI_ISL_3209081, EPI_ISL_3209621, EPI_ISL_3210043, EPI_ISL_3211689, EPI_ISL_3211976, EPI_ISL_3212343, EPI_ISL_3301008, EPI_ISL_3301513, EPI_ISL_3301592, EPI_ISL_3302738, EPI_ISL_3303032, EPI_ISL_3303035, EPI_ISL_3303516, EPI_ISL_3348010, EPI_ISL_3349409, EPI_ISL_3349460, EPI_ISL_3350552, EPI_ISL_3397129, EPI_ISL_3457436, EPI_ISL_3457484, EPI_ISL_3520910, EPI_ISL_3521421, EPI_ISL_3522426, EPI_ISL_3523079, EPI_ISL_3608006, EPI_ISL_3608193, EPI_ISL_3608291, EPI_ISL_3659923, EPI_ISL_3662599, EPI_ISL_3719656, EPI_ISL_4022689, EPI_ISL_4024741, EPI_ISL_4032778, EPI_ISL_4034302, EPI_ISL_4034554			
see above	Fulgent Genetics	Centers for Disease Control and Prevention Division of Viral Diseases, Pathogen Discovery	Adrian Paskey; Becky Tsai; Benafsh Sapra; Benjamin Rambo-Martin; Christopher Gulvick; Clinton Paden; Clinton R. Paden; Dakota Howard; Darlene Wagner; Dhwani Batra; Doreen Ng; Duncan MacCannell; Harry Gao; James Xie; Jason Caravas; John Gao; Joseph Fierro; Kara Moser; Matthew Schmerer; Mickey Li; Peter Cook; Peter W. Cook; Scott Sammons; Shatavia Morrison; Yan Meng; Yvette Unoarumhi
EPI_ISL_3231764, EPI_ISL_3435077, EPI_ISL_3435078	GA Department of Public Health	GA Department of Public Health	Aliyah Fields; Cynthia Dixey; Jonathan Edwards; Sharmila Talekar; Stacy Reeves; Taylor Smith; Tonia Parrott
EPI_ISL_3476495	GH A.CHENEVIER-H.MONDOR	Department of Virology, Henri Mondor University Hospital, Assistance Publique Hôpitaux de Paris, Université Paris-Est Créteil, INSERM U955	Alexandre Soulier; Christophe Rodriguez; Elisabeth Trawinski; Guillaume Gricourt; Jean-Michel Pawlotsky; Melissa N'Debi; Slim Fourati; Vanessa Demontant
EPI_ISL_3185657, EPI_ISL_3185663, EPI_ISL_3185669, EPI_ISL_3185670, EPI_ISL_3185671, EPI_ISL_3185680, EPI_ISL_3185682, EPI_ISL_3185684, EPI_ISL_3185697, EPI_ISL_3185716, EPI_ISL_3185723, EPI_ISL_3185724, EPI_ISL_3185736, EPI_ISL_3185740, EPI_ISL_3185743, EPI_ISL_3185754, EPI_ISL_3185755, EPI_ISL_3185761, EPI_ISL_3185767, EPI_ISL_3185772, EPI_ISL_3185777, EPI_ISL_3185785, EPI_ISL_3185796, EPI_ISL_3185797, EPI_ISL_3185799, EPI_ISL_3185800, EPI_ISL_3185801, EPI_ISL_3185805, EPI_ISL_3185808, EPI_ISL_3262663, EPI_ISL_3262666, EPI_ISL_3262669, EPI_ISL_3262674, EPI_ISL_3262677, EPI_ISL_3262678, EPI_ISL_3262679, EPI_ISL_3262687, EPI_ISL_3262701, EPI_ISL_3262704, EPI_ISL_3262705, EPI_ISL_3262706, EPI_ISL_3262712, EPI_ISL_3262718, EPI_ISL_3341868, EPI_ISL_3342040, EPI_ISL_3342042, EPI_ISL_3342043, EPI_ISL_3342044, EPI_ISL_3342045, EPI_ISL_3358542, EPI_ISL_3358544, EPI_ISL_3358546, EPI_ISL_3358548, EPI_ISL_3363548, EPI_ISL_3363569, EPI_ISL_3363582, EPI_ISL_3363618, EPI_ISL_3363627, EPI_ISL_3363631, EPI_ISL_3363635, EPI_ISL_3363646, EPI_ISL_3363649, EPI_ISL_3363653, EPI_ISL_3363656, EPI_ISL_3363659, EPI_ISL_3363674, EPI_ISL_3364915, EPI_ISL_3364923, EPI_ISL_3364926, EPI_ISL_3364929, EPI_ISL_3364936, EPI_ISL_3364954, EPI_ISL_3364958, EPI_ISL_3364967, EPI_ISL_3364971, EPI_ISL_3364987, EPI_ISL_3364991, EPI_ISL_3365018, EPI_ISL_3365022, EPI_ISL_3365026, EPI_ISL_3368409, EPI_ISL_3368430, EPI_ISL_3368439, EPI_ISL_3368453, EPI_ISL_3368454, EPI_ISL_3368464, EPI_ISL_3368777, EPI_ISL_3368778, EPI_ISL_3368785, EPI_ISL_3368786, EPI_ISL_3368788, EPI_ISL_3368794, EPI_ISL_3368795, EPI_ISL_3368796, EPI_ISL_3368797, EPI_ISL_3368799, EPI_ISL_3368803, EPI_ISL_3368805, EPI_ISL_3368809, EPI_ISL_3368851, EPI_ISL_3368852, EPI_ISL_3368856, EPI_ISL_3368864, EPI_ISL_3368866, EPI_ISL_3368875, EPI_ISL_3368876, EPI_ISL_3368878, EPI_ISL_3368882, EPI_ISL_3369291, EPI_ISL_3369293, EPI_ISL_3369295, EPI_ISL_3369300, EPI_ISL_3369304, EPI_ISL_3369341, EPI_ISL_3369342, EPI_ISL_3369343, EPI_ISL_3369344, EPI_ISL_3369345, EPI_ISL_3369346, EPI_ISL_3369350, EPI_ISL_3369351, EPI_ISL_3369356, EPI_ISL_3369376, EPI_ISL_3369378, EPI_ISL_3369379, EPI_ISL_3369380, EPI_ISL_3369395, EPI_ISL_3369396, EPI_ISL_3369399, EPI_ISL_3369401, EPI_ISL_3369405, EPI_ISL_3369406, EPI_ISL_3369407, EPI_ISL_3536006, EPI_ISL_3536017, EPI_ISL_3536022, EPI_ISL_3536028, EPI_ISL_3536095, EPI_ISL_3536096, EPI_ISL_3536101, EPI_ISL_3536113, EPI_ISL_3536114, EPI_ISL_3536119, EPI_ISL_3536120, EPI_ISL_3536131, EPI_ISL_3536132, EPI_ISL_3536133, EPI_ISL_3536135, EPI_ISL_3536136, EPI_ISL_3536140, EPI_ISL_3536144, EPI_ISL_3537148, EPI_ISL_3537152, EPI_ISL_3537154, EPI_ISL_3537160, EPI_ISL_3537165, EPI_ISL_3537166, EPI_ISL_3537169, EPI_ISL_3537170, EPI_ISL_3537176, EPI_ISL_3537186, EPI_ISL_3537188, EPI_ISL_3537189, EPI_ISL_3537311, EPI_ISL_3537312, EPI_ISL_3537313, EPI_ISL_3537314, EPI_ISL_3537319, EPI_ISL_3537320, EPI_ISL_3537329, EPI_ISL_3537332, EPI_ISL_3537333, EPI_ISL_3537334, EPI_ISL_3537336, EPI_ISL_3546372, EPI_ISL_3546373, EPI_ISL_3546379, EPI_ISL_3981960, EPI_ISL_3981961, EPI_ISL_3981963, EPI_ISL_3981969, EPI_ISL_3981970, EPI_ISL_3981971, EPI_ISL_3981974, EPI_ISL_3981975, EPI_ISL_3981977, EPI_ISL_3981979, EPI_ISL_3981999, EPI_ISL_3982399, EPI_ISL_3982401, EPI_ISL_3982422, EPI_ISL_3982428, EPI_ISL_3982623, EPI_ISL_3982630, EPI_ISL_3982631, EPI_ISL_3982636, EPI_ISL_3982637, EPI_ISL_3982643, EPI_ISL_3982644, EPI_ISL_3982649, EPI_ISL_3982650, EPI_ISL_3982651, EPI_ISL_3982653, EPI_ISL_3982654, EPI_ISL_3982655, EPI_ISL_3982656, EPI_ISL_3982657, EPI_ISL_3982658, EPI_ISL_3982664, EPI_ISL_3987648, EPI_ISL_3987649, EPI_ISL_3987651, EPI_ISL_3987661, EPI_ISL_3987662, EPI_ISL_3987663, EPI_ISL_3987665, EPI_ISL_3987868, EPI_ISL_3987875, EPI_ISL_3987894, EPI_ISL_3987895, EPI_ISL_3987920, EPI_ISL_3988092, EPI_ISL_3988093, EPI_ISL_3988094, EPI_ISL_3988095, EPI_ISL_3988096, EPI_ISL_3988102, EPI_ISL_3988111, EPI_ISL_3988112, EPI_ISL_3988113, EPI_ISL_3988117, EPI_ISL_3988118, EPI_ISL_3988119, EPI_ISL_3988124, EPI_ISL_3988128, EPI_ISL_3988143, EPI_ISL_3988144, EPI_ISL_3988149, EPI_ISL_3988150, EPI_ISL_3988153, EPI_ISL_3988216, EPI_ISL_3988233, EPI_ISL_3988234, EPI_ISL_3988235, EPI_ISL_3988239, EPI_ISL_3988252, EPI_ISL_3988255, EPI_ISL_3988264, EPI_ISL_3988268, EPI_ISL_3988269, EPI_ISL_3988289, EPI_ISL_3988299, EPI_ISL_4005019, EPI_ISL_4005021, EPI_ISL_4005023, EPI_ISL_4005024, EPI_ISL_4005025, EPI_ISL_4005027, EPI_ISL_4005031, EPI_ISL_4005044, EPI_ISL_4005045, EPI_ISL_4005046, EPI_ISL_4005080, EPI_ISL_4005081, EPI_ISL_4005084, EPI_ISL_4005085, EPI_ISL_4005087, EPI_ISL_4005089, EPI_ISL_4005091, EPI_ISL_4005092, EPI_ISL_4005093, EPI_ISL_4005094, EPI_ISL_4005095, EPI_ISL_4005096, EPI_ISL_4005097, EPI_ISL_4005098, EPI_ISL_4005101, EPI_ISL_4005102, EPI_ISL_4005103, EPI_ISL_4005104, EPI_ISL_4005105, EPI_ISL_4005106, EPI_ISL_4005107, EPI_ISL_4005108, EPI_ISL_4005109, EPI_ISL_4005133, EPI_ISL_4005153, EPI_ISL_4005155, EPI_ISL_4005157, EPI_ISL_4005159, EPI_ISL_4005160, EPI_ISL_4005161, EPI_ISL_4005162, EPI_ISL_4005171, EPI_ISL_4005172, EPI_ISL_4005173, EPI_ISL_4005177, EPI_ISL_4005181, EPI_ISL_4005182, EPI_ISL_4005183, EPI_ISL_4005184, EPI_ISL_4005185, EPI_ISL_4005186, EPI_ISL_4005188, EPI_ISL_4005221, EPI_ISL_4005222, EPI_ISL_4005224, EPI_ISL_4005228, EPI_ISL_4005230, EPI_ISL_4005232, EPI_ISL_4005233			
see above	Genetica Molecular and Subdepartamento de Virologia ISP Chile	Instituto de Salud Publica de Chile	Andres Castillo; Barbara Parra; Constanza Campano; Gisselle Barra; Javier Tognarelli; Jorge Fernandez; Karen Orostica; Loredana Arata; Patricia Bustos; Rodrigo Fasce; Soledad Ulloa
EPI_ISL_3624115	Gravity Diagnostics, LLC	Gravity Diagnostics, LLC	Gravity Diagnostics
EPI_ISL_3636676	H Evora	Instituto Nacional de Saude (INSA)	Borges et al
EPI_ISL_3204354	H Garcia de Orta	Instituto Nacional de Saude (INSA)	Borges et al
EPI_ISL_3536208, EPI_ISL_3536209, EPI_ISL_3536211, EPI_ISL_3536212, EPI_ISL_3912161	HEMOCE CENTRO DE HEMATOLOGIA E HEMOTERAPIA DO CEARA	Analytical Competence Molecular Epidemiology Lab/ACME, Oswaldo Cruz Foundation, Ceara (FIOCRUZ CE)	Cleber Furtado Aksenen; Fabio Miyajima; Fernando Braga Stehling; Francisco Eder de Moura Lopes; Jamille Maria Mendes Bezerra; Joaquim Cesar do Nascimento Sousa Junior; Joaquim César do Nascimento Sousa Junior; Pedro Miguel Carneiro Jeronimo; Suzana Porto Almeida & Lucas Delerino on behalf of COVID-19 FIOCRUZ Genomic Network; Suzana Porto Almeida e Lucas Delerino; Thais Ferreira de Oliveira; Thais de Oliveira Costa; Ticiane Cavalcante de Souza; Veridiana Pessoa Miyajima
EPI_ISL_3912168, EPI_ISL_3912169	HIAS HOSPITAL INFANTIL ALBERT SABIN	Analytical Competence Molecular Epidemiology Lab/ACME, Oswaldo Cruz Foundation, Ceara (FIOCRUZ CE)	Cleber Furtado Aksenen; Fabio Miyajima; Fernando Braga Stehling; Francisco Eder de Moura Lopes; Jamille Maria Mendes Bezerra; Joaquim Cesar do Nascimento Sousa Junior; Pedro Miguel Carneiro Jeronimo; Suzana Porto Almeida & Lucas Delerino on behalf of COVID-19 FIOCRUZ Genomic Network; Thais Ferreira de Oliveira; Thais de Oliveira Costa; Ticiane Cavalcante de Souza; Veridiana Pessoa Miyajima
EPI_ISL_3259358, EPI_ISL_3259359, EPI_ISL_3259363, EPI_ISL_3259364, EPI_ISL_3259365, EPI_ISL_3259366, EPI_ISL_3259368, EPI_ISL_3259371, EPI_ISL_3259376, EPI_ISL_3259377, EPI_ISL_3259378, EPI_ISL_3259379, EPI_ISL_3259380, EPI_ISL_3274748, EPI_ISL_3274749, EPI_ISL_3274753, EPI_ISL_3274755, EPI_ISL_3274757, EPI_ISL_3274762, EPI_ISL_3274763, EPI_ISL_3274767, EPI_ISL_3274769, EPI_ISL_3274770, EPI_ISL_3274774, EPI_ISL_3274777, EPI_ISL_3274783, EPI_ISL_3274784, EPI_ISL_3274786, EPI_ISL_3274788, EPI_ISL_3299430, EPI_ISL_3386132, EPI_ISL_3386137, EPI_ISL_3386141, EPI_ISL_3386149, EPI_ISL_3386152, EPI_ISL_3536439, EPI_ISL_3536441, EPI_ISL_3536443, EPI_ISL_3536446, EPI_ISL_3536452, EPI_ISL_3536453, EPI_ISL_3536458, EPI_ISL_3536459, EPI_ISL_3536466, EPI_ISL_3536470, EPI_ISL_3536472, EPI_ISL_3536474, EPI_ISL_3536478, EPI_ISL_3536479, EPI_ISL_3536480, EPI_ISL_3536482, EPI_ISL_3536485, EPI_ISL_3536489, EPI_ISL_3536490, EPI_ISL_3536494, EPI_ISL_3536496, EPI_ISL_3536498, EPI_ISL_3536507, EPI_ISL_3536508, EPI_ISL_3536511, EPI_ISL_3536516, EPI_ISL_3536518, EPI_ISL_3536520, EPI_ISL_3536521, EPI_ISL_3536534, EPI_ISL_3536537, EPI_ISL_3758060, EPI_ISL_3758065, EPI_ISL_3758074, EPI_ISL_3758080, EPI_ISL_3758086, EPI_ISL_3758087, EPI_ISL_3758092, EPI_ISL_3758093, EPI_ISL_3758097, EPI_ISL_3758100, EPI_ISL_3758109, EPI_ISL_3758111, EPI_ISL_3758127, EPI_ISL_3758128, EPI_ISL_3758130, EPI_ISL_3758133, EPI_ISL_3758135, EPI_ISL_3758141, EPI_ISL_3758142, EPI_ISL_3758145, EPI_ISL_3758146, EPI_ISL_3758149, EPI_ISL_3758154, EPI_ISL_3758156, EPI_ISL_3922206, EPI_ISL_3922210, EPI_ISL_3922212, EPI_ISL_3922213, EPI_ISL_3922215, EPI_ISL_3922223, EPI_ISL_3922229, EPI_ISL_3922230, EPI_ISL_3922232, EPI_ISL_3922236, EPI_ISL_3922238, EPI_ISL_3922250, EPI_ISL_3922254, EPI_ISL_3922256, EPI_ISL_3922257, EPI_ISL_3997118, EPI_ISL_3997120, EPI_ISL_3997123, EPI_ISL_3997129, EPI_ISL_3997135, EPI_ISL_3997144, EPI_ISL_3997153, EPI_ISL_3997160, EPI_ISL_3997163, EPI_ISL_3997164, EPI_ISL_3997172, EPI_ISL_3997180, EPI_ISL_3997183, EPI_ISL_3997185, EPI_ISL_3997190, EPI_ISL_3997195, EPI_ISL_3997209, EPI_ISL_3997210, EPI_ISL_3997211, EPI_ISL_4061629, EPI_ISL_4061663, EPI_ISL_4061664, EPI_ISL_4061667, EPI_ISL_4061670			
see above	HLAGYN - Laboratorio de Imunologia de Transplantes de Goias	HLAGYN - Laboratorio de Imunologia de Transplantes de Goias	Alessandro Leonardo Alvares Magalhaes; Erika Lopes Rocha Batista; Fernando Antonio Vinhal dos Santos; Frederico Rodrigues Vinhal; Kamila Oliveira Reis De Freitas.; Lucas Carlos Gomes Pereira; Sabrina Sara Moreira Duarte
EPI_ISL_3536229, EPI_ISL_3536235, EPI_ISL_3536244, EPI_ISL_3536250, EPI_ISL_3536260, EPI_ISL_3536261, EPI_ISL_3536262, EPI_ISL_3912170, EPI_ISL_3912171, EPI_ISL_3912172			
see above	HM HOSPITAL DE MESSEJANA DR CARLOS ALBERTO STUDART GOMES	Analytical Competence Molecular Epidemiology Lab/ACME, Oswaldo Cruz Foundation, Ceara (FIOCRUZ CE)	Cleber Furtado Aksenen; Fabio Miyajima; Fernando Braga Stehling; Francisco Eder de Moura Lopes; Jamille Maria Mendes Bezerra; Joaquim Cesar do Nascimento Sousa Junior; Joaquim César do Nascimento Sousa Junior; Pedro Miguel Carneiro Jeronimo; Suzana Porto Almeida & Lucas Delerino on behalf of COVID-19 FIOCRUZ Genomic Network; Suzana Porto Almeida e Lucas Delerino; Thais Ferreira de Oliveira; Thais de Oliveira Costa; Ticiane Cavalcante de Souza; Veridiana Pessoa Miyajima
EPI_ISL_3997818	HOSPITAL DE BELLVITGE	Microbiology Department	Aida Gonzalez-Diaz; Carmen Ardanuy; Jordi Camara; Jordi Niubó; Laura Calatayud; M Angeles Domínguez; Sara Marti; Yolanda Hernandez
EPI_ISL_3639085	HOSPITAL DR. RAFAEL A. CALDERON GUARDIA	Inciensa, Instituto Costarricense de Investigación y Enseñanza en Nutrición y Salud	Adriana Godínez; Claudio Soto-Garita; Estela Cordero; Francisco Duarte; Hebleen Porras; Joselyn Prado & Fabian Salas; José Luis Vargas; Mariela Gutiérrez; Melany Calderón
EPI_ISL_3912194, EPI_ISL_3912195	HOSPITAL E MATERNIDADE DRA ZILDA ARNS NEUMANN	Analytical Competence Molecular Epidemiology Lab/ACME, Oswaldo Cruz Foundation, Ceara (FIOCRUZ CE)	Cleber Furtado Aksenen; Fabio Miyajima; Fernando Braga Stehling; Francisco Eder de Moura Lopes; Jamille Maria Mendes Bezerra; Joaquim Cesar do Nascimento Sousa Junior; Pedro Miguel Carneiro Jeronimo; Suzana Porto Almeida & Lucas Delerino on behalf of COVID-19 FIOCRUZ Genomic Network; Thais Ferreira de Oliveira; Thais de Oliveira Costa; Ticiane Cavalcante de Souza; Veridiana Pessoa Miyajima
EPI_ISL_3536163, EPI_ISL_3536164, EPI_ISL_3536171, EPI_ISL_3536172, EPI_ISL_3536268, EPI_ISL_3536276, EPI_ISL_3536333, EPI_ISL_3536396, EPI_ISL_3912199, EPI_ISL_3912202, EPI_ISL_3912203, EPI_ISL_3912205			
see above	HOSPITAL ESTADUAL LEONARDO DA VINCI	Analytical Competence Molecular Epidemiology Lab/ACME, Oswaldo Cruz Foundation, Ceara (FIOCRUZ CE)	Cleber Furtado Aksenen; Fabio Miyajima; Fernando Braga Stehling; Francisco Eder de Moura Lopes; Jamille Maria Mendes Bezerra; Joaquim Cesar do Nascimento Sousa Junior; Joaquim César do Nascimento Sousa Junior; Pedro Miguel Carneiro Jeronimo; Suzana Porto Almeida & Lucas Delerino on behalf of COVID-19 FIOCRUZ Genomic Network; Suzana Porto Almeida e Lucas Delerino; Thais Ferreira de Oliveira; Thais de Oliveira Costa; Ticiane Cavalcante de Souza; Veridiana Pessoa Miyajima
EPI_ISL_3948522	HOSPITAL LA ANEXION	Inciensa, Instituto Costarricense de Investigación y Enseñanza en Nutrición y Salud	Adriana Godínez; Claudio Soto-Garita; Estela Cordero; Francisco Duarte; Hebleen Porras; Joselyn Prado & Adriana Bermúdez; José Luis Vargas; Mariela Gutiérrez; Melany Calderón
EPI_ISL_3639148	HOSPITAL MONSEÑOR SANABRIA	Inciensa, Instituto Costarricense de Investigación y Enseñanza en Nutrición y Salud	Adriana Godínez; Claudio Soto-Garita; Estela Cordero; Francisco Duarte; Hebleen Porras; Joselyn Prado & Andrea Moreno; José Luis Vargas; Mariela Gutiérrez; Melany Calderón
EPI_ISL_3922309	HOSPITAL MUNICIPAL DE ITAPIRA	Instituto Butantan	Antonio Jorge Martins; Claudia Renata dos Santos Barros; David Schlesinger; Debora Botequio Moretti; Dimas Tadeu Covas; Elaine Cristina Marqueze; Elaine Vieira Santos; Evandra Strazza Rodrigues; Heidge Fukumasu; Jayme Augusto de Souza-Neto; José Salvatore Leister Patané; Luiz Alcantara; Luiz Lehmann Coutinho; Maria Carolina Elias; Maurício Lacerda Nogueira; Rafael dos Santos Bezerra; Raul Machado Neto; Rejane Maria Tommasini Grotto; Ricardo Haddad; Sandra Coccuzzo Sampaio Vessoni; Simone Kashima; Svetoslav Nanev Slavov; Vincent Louis Viala
EPI_ISL_3912214, EPI_ISL_3912215	HOSPITAL MUNICIPAL NOSSA SENHORA DOS MILAGRES	Analytical Competence Molecular Epidemiology Lab/ACME, Oswaldo Cruz Foundation, Ceara (FIOCRUZ CE)	Cleber Furtado Aksenen; Fabio Miyajima; Fernando Braga Stehling; Francisco Eder de Moura Lopes; Jamille Maria Mendes Bezerra; Joaquim Cesar do Nascimento Sousa Junior; Pedro Miguel Carneiro Jeronimo; Suzana Porto Almeida & Lucas Delerino on behalf of COVID-19 FIOCRUZ Genomic Network; Thais Ferreira de Oliveira; Thais de Oliveira Costa; Ticiane Cavalcante de Souza; Veridiana Pessoa Miyajima
EPI_ISL_3761771, EPI_ISL_3761792, EPI_ISL_3761800	HOSPITAL NACIONAL DE NIÑOS	Inciensa, Instituto Costarricense de Investigación y Enseñanza en Nutrición y Salud	Cristian Pérez-Corrales & Valeria Peralta-Barquero
EPI_ISL_3912223	HOSPITAL REGIONAL DO SERTAO CENTRAL	Analytical Competence Molecular Epidemiology Lab/ACME, Oswaldo Cruz Foundation, Ceara (FIOCRUZ CE)	Cleber Furtado Aksenen; Fabio Miyajima; Fernando Braga Stehling; Francisco Eder de Moura Lopes; Jamille Maria Mendes Bezerra; Joaquim Cesar do Nascimento Sousa Junior; Pedro Miguel Carneiro Jeronimo; Suzana Porto Almeida & Lucas Delerino on behalf of COVID-19 FIOCRUZ Genomic Network; Thais Ferreira de Oliveira; Thais de Oliveira Costa; Ticiane Cavalcante de Souza; Veridiana Pessoa Miyajima
EPI_ISL_3639017	HOSPITAL SAN FRANCISCO DE ASIS	Inciensa, Instituto Costarricense de Investigación y Enseñanza en Nutrición y Salud	Adriana Godínez; Claudio Soto-Garita; Estela Cordero; Francisco Duarte; Hebleen Porras; Joselyn Prado & Adrián Fallas Mora; José Luis Vargas; Mariela Gutiérrez; Melany Calderón
EPI_ISL_3948490	HOSPITAL SAN JUAN DE DIOS	Inciensa, Instituto Costarricense de Investigación y Enseñanza en Nutrición y Salud	Adriana Godínez; Claudio Soto-Garita; Estela Cordero; Francisco Duarte; Hebleen Porras; Joselyn Prado & Juliana Mora; José Luis Vargas; Mariela Gutiérrez; Melany Calderón
EPI_ISL_3638824	HOSPITAL SAN RAFAEL DE ALAJUELA	Inciensa, Instituto Costarricense de Investigación y Enseñanza en Nutrición y Salud	Adriana Godínez; Claudio Soto-Garita; Estela Cordero; Francisco Duarte; Hebleen Porras; Joselyn Prado & Christian Sanchez; José Luis Vargas; Mariela Gutiérrez; Melany Calderón

EPI_ISL_3639143	HOSPITAL SAN VICENTE DE PAUL	Inciensa, Instituto Costarricense de Investigación y Enseñanza en Nutrición y Salud	Adriana Godínez; Claudio Soto-Garita; Estela Cordero; Francisco Duarte; Hebleen Porras; Joselyn Prado & Silvia Sáenz; José Luis Vargas; Mariela Gutiérrez; Melany Calderón
EPI_ISL_3298356, EPI_ISL_3948541	HOSPITAL SAN VITO DE COTO BRUS	Inciensa, Instituto Costarricense de Investigación y Enseñanza en Nutrición y Salud	Adriana Godínez; Claudio Soto-Garita; Estela Cordero; Francisco Duarte; Hebleen Porras; Joselyn Prado & Maria Fernanda Matamoros; José Luis Vargas; Mariela Gutiérrez; Mariela Gutiérrez & Joselyn Prado; Melany Calderón
EPI_ISL_3912228	HOSPITAL SAO JOSE DE DOENCAS INFECCIOSAS	Analytical Competence Molecular Epidemiology Lab/ACME, Oswaldo Cruz Foundation, Ceara (FIOCRUZ CE)	Cleber Furtado Aksenen; Fabio Miyajima; Fernando Braga Stehling; Francisco Eder de Moura Lopes; Jamille Maria Mendes Bezerra; Joaquim Cesar do Nascimento Sousa Junior; Pedro Miguel Carneiro Jeronimo; Suzana Porto Almeida & Lucas Delerino on behalf of COVID-19 FIOCRUZ Genomic Network; Thais Ferreira de Oliveira; Thais de Oliveira Costa; Ticiane Cavalcante de Souza; Veridiana Pessoa Miyajima
EPI_ISL_3188634	Hospital General Universitario Gregorio Marañón	Hospital General Universitario Gregorio Marañón	Cristina Rodriguez-Grande; Darío García de Viedma; Julia Suárez; Laura Pérez-Lago; Marta Herranz Martin; Patricia Muñoz; Pedro Sola Campoy; Pilar Catalán; Sergio Buenestado Serrano; Victor Manuel de la Cueva
EPI_ISL_3260112	Hospital Jaime Ferre - SAMCO Rafaela	Grupo de Genómica y Bioinformática del Instituto de Investigación de la Cadena Láctea CONICET-INTA on behalf of 'Proyecto Argentino Interinstitucional de genomica de SARS-CoV-2' (PAIS Consortium)	AF; Aliprandi D; Amadio; C; Eberhardt; Irazoqui; Isaia; JF; JM; MF; Pandolfi; Quaranta; Soratti R; V
EPI_ISL_4005953, EPI_ISL_4005957	Hospital Sharp	Microbial Genomics Laboratory	; Alejandra García-Gasca; Alejandra Hernández-Terán; Alejandro Sánchez-Flores; Alfredo Herrera-Estrella; Alicia Ocaña-Mondragón; Andreu Comas-García; Angel Gustavo Salas-Lais; Antonio Loza Román; Bernardo Martínez-Miguel; Blanca Taboada; Brenda Irasema Maldonado-Meza; Bruno Gómez-Gil; Carla Ivón Herrera-Najera; Carlos F. Arias; Celia Boukadida; Clara Esperanza Santacruz-Tinoco; Concepción Grajales-Muñiz; Consorcio Mexicano de Vigilancia Genómica (CoViGen-Mex). Authors (in alphabetical order): Julio Elias Alvarado-Yaah; Cristóbal Cháidez-Quiróz; Célida Duque Molina; Célida Martínez- Rodríguez; Daniel Fregoso-Rueda; Daniel Lira Morales; Eduardo Becerril-Vargas; Fernando Fontove-Herrera; Fidencio Mejía-Nepomuceno; Francisco Pulido; Gloria Elena Espinosa-Ayala; Gloria María Molina-Salinas; Gloria Vazquez; Hector Esteban Paz-Juárez; Hector Montoya-Fuentes; Helen Haydee Fernanda Ramírez-Plascencia; Irvin González-López; Jean Pierre González; Jesús Hernández; Joel Armando Vázquez-Pérez.; Jorge Salas-Hernández; José Antonio Enciso-Moreno; José Arturo Martínez-Orozco; José Esteban Muñoz-Medina; José de Jesús Nuñez-Contreras; Juan Bautista Chale-Dzul; Julissa Enciso-Ibarra; Luis Alberto Ochoa-Carrera; Margarita Matías-Florentino; Mario Mújica-Sánchez; Marissa Perez-Garcia; María Guadalupe Santiago-Mauricio; María Guadalupe de Jesús Mireles-Rivera; Nelly Sélem-Mojica; Pavel Isa; Ricardo Ciria Merce; Ricardo Grande; Rosa María Gutiérrez Rios; Santiago Ávila-Ríos; Selene Zárate; Susana Lopez; Verónica Mata-Haro; Victor Eduardo García-Arias; Víctor Hugo Borja-Aburto
EPI_ISL_3696641, EPI_ISL_3696682, EPI_ISL_3696726	Hospital de Campanha de Guaratinguetá	Instituto Butantan	Antonio Jorge Martins; Claudia Renata dos Santos Barros; David Schlesinger; Debora Botequio Moretti; Dimas Tadeu Covas; Elaine Cristina Marqueze; Elaine Vieira Santos; Evandra Strazza Rodrigues; Heidge Fukumasu; Jayme Augusto de Souza-Neto; José Salvatore Leister Patané; Luiz Alcantara; Luiz Lehmann Coutinho; Maria Carolina Elias; Maurício Lacerda Nogueira; Rafael dos Santos Bezerra; Raul Machado Neto; Rejane Maria Tommasini Grotto; Ricardo Haddad; Sandra Coccuzzo Sampaio Vessoni; Simone Kashima; Svetoslav Nanev Slavov; Vincent Louis Viala
EPI_ISL_3398956, EPI_ISL_4004142, EPI_ISL_4004281	Houston Health Dept.	Houston Health Dept.	Adolpho Lara; Pamela Brown; Ryker Penn; Yanlai Lai
EPI_ISL_3303826, EPI_ISL_3369475, EPI_ISL_3936902	IN State Department of Health Laboratory Services	IN State Department of Health Laboratory Services	Brian Pope; Cassandra Campion; Jamie Yeadon; Kyle Brownlee; Lixia Liu; Mark Glazier; Melissa Hindenlang
EPI_ISL_3068188, EPI_ISL_3068202, EPI_ISL_3068241, EPI_ISL_3274395, EPI_ISL_3274430, EPI_ISL_3274458, EPI_ISL_3274460, EPI_ISL_3274480, EPI_ISL_3274497, EPI_ISL_3274502, EPI_ISL_3274520, EPI_ISL_3274525, EPI_ISL_3274530, EPI_ISL_3274569, EPI_ISL_3274608, EPI_ISL_3274615			
see above	INSPI-CRN DE INFLUENZA Y OTROS VIRUS RESPIRATORIOS	NIC-INSPI	Alfredo Bruno; Daniel Ramos; Domenica de Mora.; Jimmy Garcés; Johanna Laines; Lizbeth Patiño; Manuel Gonzalez; Maria Angelica Becerra; Maritza Olmedo; Michelle Páez
EPI_ISL_3088327	INTERLAB	Omics Sciences Laboratory	Darlyn Amaya; Derly Andrade Molina; Gabriel Morey León; Juan Carlos Fernández Cadena; Rubén Armas González
EPI_ISL_3068295, EPI_ISL_3068586, EPI_ISL_3161423, EPI_ISL_3161554, EPI_ISL_3161569, EPI_ISL_3419716, EPI_ISL_3419948			
see above	IRCCS San Gallicano Dermatological Institute	IRCCS Regina Elena National Cancer Institute	Aldo Morrone; Alice Massacci; Andrea Cazzani; Eleonora Sperandio; Elisabetta Trento; Fabrizio Ensoli; Francesca De Nicola; Francesca Maione; Francesca Sivori; Frauke Goeman; Fulvia Pimpinelli; Gennaro Ciliberto; Giovanna D'agosto; Giovanni Blandino; Giulia Orlandi; Ilaria Cavallo; Ludovica Ciuffreda; Matteo Pallocca; Maurizio Fanciulli; Sabrina Strano; Sara Donzelli; Sara Petrolo
EPI_ISL_3547122, EPI_ISL_3547148	IVY3 Central Lab, Vanderbilt University Medical Center	Lauring Lab, University of Michigan, Department of Microbiology and Immunology	Gilbert
EPI_ISL_3156477, EPI_ISL_3272419, EPI_ISL_3640996, EPI_ISL_3664324	Idaho Bureau of Laboratories	Boise VA Medical Center, PALMS	Aimee Ceniseros; Cheri Lamb McFarlane; Christian Loera; Dr. Christopher Ball; Emily Bartlett; Emily Bartlette; James Razor; Matthew Burns; Ying Pei
EPI_ISL_3642917, EPI_ISL_3722427, EPI_ISL_4084693	Idaho Bureau of Laboratories	Center for Global Health, University of New Mexico Health Sciences Center	Abigail Cortez; Aimee Ceniseros; Cassidy Vanwarmerdam; Christian Loera; Christopher Ball; Darrell Dinwiddie; Daryl Domman; Josie Santos; Kurt Schwalm; Matthew Burns; Robert Voermans; Valerie Morley
EPI_ISL_3232011	Illinois Department of Public Health	Illinois Department of Public Health - Chicago Lab	Ira Heimler; Joel Price; Vineet K. Dhiman
EPI_ISL_3347020	Incyte Diagnostics Spokane	Seattle Flu Study	Amanda Adler; Barry R. Lutz; Benjamin Pelle; Brian Hiatt; Caitlin R. Wolf; Chris D. Frazar; Deborah A. Nickerson; Elisabeth Brandstetter; Erica Ryke; Geoff Melly; Helen Y. Chu; Janet A. Englund; Jay Shendure; Jover Lee; Kairsten Fay; Kirsten Lacombe; Lea M. Starita; Mark J. Rieder; Matthew Richardson; Matthew Thompson; Melissa Truong; Michael Boeckh; Michael Famulare; Misja Ilcisin; Peter D. Han; Philip Dykema; Romesh Gautom; Scott Lindquist; Thomas R. Sibley; Trevor Bedford
EPI_ISL_3299775	Infinity Biologix	Centers for Disease Control and Prevention Division of Viral Diseases, Pathogen Discovery	Adrian Paskey; Benjamin Rambo-Martin; Chirayu Goswami; Christian Bixby; Christopher Gulvick; Clinton R. Paden; Dakota Howard; Darlene Wagner; Dhwani Batra; Duncan MacCannell; Jason Caravas; Jonathan Schultz; Kara Moser; Matthew Schmerer; Peter W. Cook; Robin Grimwood; Russ Hager; Scott Sammons; Shatavia Morrison; Yihe Wang; Yvette Unoarumhi
EPI_ISL_3505610, EPI_ISL_3505611, EPI_ISL_3824606	Institute of Microbiology, Universidad San Francisco de Quito	Institute of Microbiology, Universidad San Francisco de Quito	Alexandra Gonzalez; Belén Prado-Vivar; Bernardo Gutiérrez; Betty Angulo; Erika B. Muñoz; Fernanda Zurita; Francisco Guerra; Gabriel Trueba; Hugo Vergara; Jeaninna Peña; Juan José Guadalupe; Juan Pablo Román; Liu Yuqian; Luis Fuenmayor; Luz-Angelica Castillo; Marco Viteri Yanez; Mateo Carvajal; Michelle Grunauer; Michelle Jacome; Monica Becerra-Wong; Nancy Flores Lastra; Natalia Parra; Patricio Rojas-Silva; Paúl Cárdenas; Ronny Javier Pibaque; Stephanie Espín-Arroba; Sully Márquez; Valeria Armijos; Verónica Barragán
EPI_ISL_3545781, EPI_ISL_3545787, EPI_ISL_3864599	Instituto Adolfo Lutz - Regional de Marilia	Instituto Adolfo Lutz, Interdiciplinary Procedures Center, Strategic Laboratory	Caio Vinicius Dias Lopes; Claudia Regina Gonçalves; Claudio Tavares Sacchi; Karoline Rodrigues Campos; Leonardo Tadeu de Araujo; Marlon Benedito Nascomento Santos
EPI_ISL_3545770, EPI_ISL_3545771, EPI_ISL_3545772, EPI_ISL_3545780, EPI_ISL_4081145, EPI_ISL_4081147, EPI_ISL_4081148, EPI_ISL_4081149, EPI_ISL_4081150, EPI_ISL_4081151, EPI_ISL_4081152, EPI_ISL_4081153			
see above	Instituto Adolfo Lutz - Regional de Sao Jose do Rio Preto	Instituto Adolfo Lutz, Interdiciplinary Procedures Center, Strategic Laboratory	Caio Vinicius Dias Lopes; Claudia Regina Gonçalves; Claudio Tavares Sacchi; Karoline Rodrigues Campos; Leonardo Tadeu de Araujo; Marlon Benedito Nascomento Santos; Marlon Benedito Nascomento Santos
EPI_ISL_3545774, EPI_ISL_3545775, EPI_ISL_3545785	Instituto Adolfo Lutz - Regional de Sorocaba	Instituto Adolfo Lutz, Interdiciplinary Procedures Center, Strategic Laboratory	Caio Vinicius Dias Lopes; Claudia Regina Gonçalves; Claudio Tavares Sacchi; Karoline Rodrigues Campos; Leonardo Tadeu de Araujo; Marlon Benedito Nascomento Santos
EPI_ISL_3691688, EPI_ISL_3691689, EPI_ISL_3691690, EPI_ISL_3691694, EPI_ISL_3691698, EPI_ISL_3691699, EPI_ISL_3864540, EPI_ISL_3864542, EPI_ISL_3864544, EPI_ISL_3864551, EPI_ISL_3864556, EPI_ISL_3864558, EPI_ISL_3864560, EPI_ISL_3864562, EPI_ISL_3864564, EPI_ISL_3864568, EPI_ISL_3864572, EPI_ISL_3864574, EPI_ISL_3864576, EPI_ISL_3864579, EPI_ISL_3864585, EPI_ISL_3864589, EPI_ISL_3864590, EPI_ISL_3864592, EPI_ISL_3864598, EPI_ISL_4081132, EPI_ISL_4081134, EPI_ISL_4081137, EPI_ISL_4081140, EPI_ISL_4081141, EPI_ISL_4081142, EPI_ISL_4081144			
see above	Instituto Adolfo Lutz Central	Instituto Adolfo Lutz, Interdiciplinary Procedures Center, Strategic Laboratory	Caio Vinicius Dias Lopes; Claudia Regina Gonçalves; Claudio Tavares Sacchi; Karoline Rodrigues Campos; Leonardo Tadeu de Araujo; Marlon Benedito Nascomento Santos; Marlon Benedito Nascomento Santos
EPI_ISL_3982817	Instituto Nacional de Enfermedades Respiratorias (INER)	Centro de Investigación en Enfermedades Infecciosas (CIENI), Instituto Nacional de Enfermedades Respiratorias (INER)	Alejandra García-Gasca; Alejandra Hernández-Terán; Alejandro Sánchez-Flores; Alfredo Herrera-Estrella; Alicia Ocaña-Mondragón; Andreu Comas-García; Angel Gustavo Salas-Lais; Antonio Loza Román; Bernardo Martínez-Miguel; Blanca Taboada; Brenda Irasema Maldonado-Meza; Bruno Gómez-Gil; Carla Ivón Herrera-Najera; Carlos F. Arias; Celia Boukadida; Clara Esperanza Santacruz-Tinoco; Concepción Grajales-Muñiz; Consorcio Mexicano de Vigilancia Genómica (CoViGen-Mex). Authors (in alphabetical order): Julio Elias Alvarado-Yaah; Cristóbal Cháidez-Quiróz; Célida Duque Molina; Célida Martínez-Rodríguez; Daniel Fregoso-Rueda; Daniel Lira Morales; Eduardo Becerril-Vargas; Eduardo Rivera-Martínez; Fernando Fontove-Herrera; Fidencio Mejía-Nepomuceno; Francisco Pulido; Gabriel Chavira-Trujillo; Gloria Elena Espinosa-Ayala; Gloria María Molina-Salinas; Gloria Vazquez; Hector Montoya-Fuentes; Helen Haydee Fernanda Ramírez-Plascencia; Irvin González-López; Jean Pierre González; Jesús Hernández; Joel Armando Vázquez-Pérez).; Jorge Salas-Hernández; José Antonio Enciso-Moreno; José Arturo Martínez-Orozco; José Esteban Muñoz-Medina; José de Jesús Nuñez-Contreras; Juan Bautista Chale-Dzul; Julissa Enciso-Ibarra; Kathia Elizabeth Tapia-Diaz; Luis Alberto Ochoa-Carrera; Margarita Matías-Florentino; Mario Mújica-Sánchez; Marissa Perez-Garcia; María Eugenia Jiménez-Corona; María Guadalupe Santiago-Mauricio; María Guadalupe de Jesús Mireles-Rivera; Nelly Sélem-Mojica; Pavel Isa; Ricardo Ciria Merce; Ricardo Grande; Rosa María Gutiérrez Rios; Rosario Vazquez-Larios; Santiago Ávila-Ríos; Selene Zárate; Susana Lopez; Verónica Mata-Haro; Victor Eduardo García-Arias; Víctor Hugo Borja-Aburto
EPI_ISL_3118933, EPI_ISL_3824319, EPI_ISL_3824320, EPI_ISL_3824321, EPI_ISL_3824322, EPI_ISL_3824323, EPI_ISL_3824326, EPI_ISL_3824330, EPI_ISL_3824332, EPI_ISL_3824333, EPI_ISL_3824336, EPI_ISL_3824337, EPI_ISL_3824338, EPI_ISL_3824339, EPI_ISL_3824340, EPI_ISL_3824341, EPI_ISL_3824350			
see above	Instituto de Biotecnologia - UNESP-Botucatu-SP	Instituto de Biotecnologia - UNESP-Botucatu-SP	Cecília Artico Banho; Cíntia Bittar; Fábio Sossai Possebon; Guilherme Campos; Helena Lage Ferreira; Jorge A. Petroli Marchesi; João Pessoa Araújo Jr.; Leila Sabrina Ullmann; Lívia Sacchetto; Maisa C. Pereira Parra; Marília Moraes; Maurício L. Nogueira; Paula Rahal; Paulo Inacio da Costa
EPI_ISL_3262656	Kansas Health and Environmental Lab	Kansas Health and Environmental Lab	Amanda Bradley; Jonathan Barnell; Mike Grose; and Phil Adam
EPI_ISL_3471630	LABO BIO DOC	CHU Purpan - Laboratoire de Virologie - Institut Fédératif de Biologie	Bulach T.; Donnadieu C.; Izopet J.; Latour J.; Milhes M.; Nicot F.; Ranger N.; Salin G.; Tremeaux P.
EPI_ISL_3379109	LABORATOIRE ACCOLAB SUD OUEST	CNR Virus des Infections Respiratoires - France SUD	Antonin Bal; Bruno Lina; Gregory Destras; Gwendolyne Burfin; Hadrien Regue; Laurence Josset; Martine Valette; Quentin Semanas

EPI_ISL_3131258	LABORATOIRE CBM DE MURET	CNR Virus des Infections Respiratoires - France SUD	Antonin Bal; Bruno Lina; Gregory Destras; Gwendolyne Burfin; Hadrien Regue; Laurence Josset; Martine Valette; Quentin Semanas
EPI_ISL_3841255, EPI_ISL_3841280, EPI_ISL_3841281, EPI_ISL_3841282, EPI_ISL_3841283, EPI_ISL_3841284, EPI_ISL_3841313, EPI_ISL_3841314			
see above	LACEN	LGBio (Laboratorio de Genetica & Biodiversidade)	Alex Honda Bernardes; Amanda Alves de Melo; Aparecido Divino da Cruz; Cintia Pelegrineti Targueta de Azevedo Brito; Daniela de Melo e Silva; Elisangela de Paula Silveira Lacerda; Juliana Santana de Curcio; Luiz Augusto Pereira; Marc Alexandre Duarte Gigonzac; Mariana Pires de Campos Telles; Ramilla dos Santos Braga; Renata de Oliveira Dias; Rhewter Nunes; Thais Cidália Vieira Gigonzac; Thais Guimarães Castro; Thays Millena Alves Pedroso
EPI_ISL_3447459, EPI_ISL_3447460, EPI_ISL_3447461, EPI_ISL_3447462, EPI_ISL_3447465, EPI_ISL_3447466, EPI_ISL_3447468, EPI_ISL_3447471, EPI_ISL_3447473, EPI_ISL_3447476, EPI_ISL_3447480, EPI_ISL_3447481, EPI_ISL_3447482, EPI_ISL_3447483, EPI_ISL_3447484, EPI_ISL_3447485, EPI_ISL_3447489, EPI_ISL_3447569, EPI_ISL_3447570, EPI_ISL_3447571, EPI_ISL_3447573, EPI_ISL_3447574, EPI_ISL_3447575, EPI_ISL_3447576, EPI_ISL_3447577, EPI_ISL_3447578, EPI_ISL_3447579, EPI_ISL_3447580, EPI_ISL_3447582, EPI_ISL_3447583, EPI_ISL_3447584, EPI_ISL_3447585, EPI_ISL_3447587, EPI_ISL_3447592, EPI_ISL_3447598, EPI_ISL_3703726, EPI_ISL_3703733, EPI_ISL_3703737, EPI_ISL_3703755, EPI_ISL_3703772, EPI_ISL_3703778, EPI_ISL_3703779, EPI_ISL_3703780, EPI_ISL_3835240, EPI_ISL_3835242, EPI_ISL_3835246, EPI_ISL_3835248, EPI_ISL_3835249, EPI_ISL_3835251, EPI_ISL_3835255, EPI_ISL_3835257, EPI_ISL_3835258, EPI_ISL_3835264, EPI_ISL_3835265, EPI_ISL_3835276, EPI_ISL_3835277, EPI_ISL_3835282, EPI_ISL_3835283, EPI_ISL_3835284, EPI_ISL_3835286, EPI_ISL_3835290, EPI_ISL_3835295, EPI_ISL_3835299, EPI_ISL_3835303, EPI_ISL_3835304, EPI_ISL_3835354, EPI_ISL_3835355, EPI_ISL_3835357			
see above	LACEN/PE	WallauLab on behalf of Fiocruz COVID-19 Genomic Surveillance Network	Alexandre Freitas da Silva; Cassia Docena; Constância Flávia Junqueira Ayres; Filipe Zimmer Dezordi; Gabriel Luz Wallau; Gustavo Barbosa de Lima; Lais Ceschini Machado; Lilian Caroliny Amorim Silva; Marcelo Henrique dos Santos Paiva; Matheus Filgueira Bezerra; Sinval Pinto Brandão Filho
EPI_ISL_3912232, EPI_ISL_3912233, EPI_ISL_3912234	LACEN_CENTRO DE TESTAGEM PARA VIAJANTE	Analytical Competence Molecular Epidemiology Lab/ACME, Oswaldo Cruz Foundation, Ceara (FIOCRUZ CE)	Cleber Furtado Aksenen; Fabio Miyajima; Fernando Braga Stehling; Francisco Eder de Moura Lopes; Jamille Maria Mendes Bezerra; Joaquim Cesar do Nascimento Sousa Junior; Pedro Miguel Carneiro Jeronimo; Suzana Porto Almeida & Lucas Delerino on behalf of COVID-19 FIOCRUZ Genomic Network; Thais Ferreira de Oliveira; Thais de Oliveira Costa; Ticiane Cavalcante de Souza; Veridiana Pessoa Miyajima
EPI_ISL_3047717, EPI_ISL_3047718, EPI_ISL_3047732, EPI_ISL_3047737, EPI_ISL_3047739, EPI_ISL_3047740, EPI_ISL_3047746, EPI_ISL_3134923, EPI_ISL_3134924, EPI_ISL_3134925, EPI_ISL_3134931, EPI_ISL_3134936, EPI_ISL_3134938, EPI_ISL_3134940, EPI_ISL_3134941, EPI_ISL_3134942, EPI_ISL_3134944, EPI_ISL_3134945, EPI_ISL_3134948, EPI_ISL_3134952, EPI_ISL_3134953, EPI_ISL_3134956, EPI_ISL_3134961, EPI_ISL_3134966, EPI_ISL_3134968, EPI_ISL_3134969, EPI_ISL_3134970, EPI_ISL_3134971, EPI_ISL_3134972, EPI_ISL_3134974, EPI_ISL_3134975, EPI_ISL_3134976, EPI_ISL_3149366, EPI_ISL_3246152, EPI_ISL_3246153, EPI_ISL_3246155, EPI_ISL_3246158, EPI_ISL_3246159, EPI_ISL_3246160, EPI_ISL_3246161, EPI_ISL_3246162, EPI_ISL_3246170, EPI_ISL_3246172, EPI_ISL_3246174, EPI_ISL_3246177, EPI_ISL_3246179, EPI_ISL_3246181, EPI_ISL_3246183, EPI_ISL_3246185, EPI_ISL_3246186, EPI_ISL_3246187, EPI_ISL_3246188, EPI_ISL_3246191, EPI_ISL_3246193, EPI_ISL_3246196, EPI_ISL_3385140, EPI_ISL_3385141, EPI_ISL_3385142, EPI_ISL_3385143, EPI_ISL_3385144, EPI_ISL_3385145, EPI_ISL_3385146, EPI_ISL_3385147, EPI_ISL_3385149, EPI_ISL_3385150, EPI_ISL_3385151, EPI_ISL_3385152, EPI_ISL_3385153, EPI_ISL_3385154, EPI_ISL_3505191, EPI_ISL_3505193, EPI_ISL_3505195, EPI_ISL_3505196, EPI_ISL_3505197, EPI_ISL_3505204, EPI_ISL_3505206, EPI_ISL_3804720, EPI_ISL_3804723, EPI_ISL_3804724, EPI_ISL_3804725, EPI_ISL_3804727, EPI_ISL_3804728, EPI_ISL_3804729, EPI_ISL_3804730, EPI_ISL_3804733, EPI_ISL_3804735, EPI_ISL_3804736, EPI_ISL_3804738, EPI_ISL_3804744, EPI_ISL_3941511, EPI_ISL_3941512, EPI_ISL_3941513, EPI_ISL_3941515, EPI_ISL_3941518, EPI_ISL_4081415, EPI_ISL_4081417			
see above	LATE - Laboratório de Técnicas Especiais - Hospital Israelita Albert Einstein	LATE - Laboratório de Técnicas Especiais - Hospital Israelita Albert Einstein	Alexandre Hideaki Takara; Ana Paula Moreira Salles; Anelisie da Silva Santos; Deyvid Amgarten; Erick Gustavo Dorlass; Fernanda de Mello Malta; João Renato Rebello Pinho; Marcio Anunciacao Menezes; Pedro Henrique Sebe Rodrigues; Raquel Riyuzo
EPI_ISL_3383156	LBM SEALAB DARRASSE ET ASSOCIES	CNR Virus des Infections Respiratoires - France SUD	Antonin Bal; Bruno Lina; Gregory Destras; Gwendolyne Burfin; Hadrien Regue; Laurence Josset; Martine Valette; Quentin Semanas
EPI_ISL_3206149	LBM UNIBIO VALENTIN	CNR Virus des Infections Respiratoires - France SUD	Antonin Bal; Bruno Lina; Gregory Destras; Gwendolyne Burfin; Hadrien Regue; Laurence Josset; Martine Valette; Quentin Semanas
EPI_ISL_3459386, EPI_ISL_3459387	LDSP CAQUETA	Instituto Nacional de Salud	Carlos Franco-Muñoz; Carmen Osorio; Diana Malo; Diego A. Álvarez-Díaz; Diego Andrés Prada; Gerardo Santamaría; Hector Alejandro Ruiz-Moreno; Jhonnatan Reales-González; Jorge Rivera; Juan Camilo Martinez; Julian Naizaque; Katherine Laiton-Donato; Lisseth Pardo; Magdalena Wiesner; Marcela Mercado-Reyes; Maria T. Herrera-Sepúlveda; Marta Lopez Blanco; Martha Lucia Ospina Martinez; Paola Rojas; Sergio Gomez; Sheryll Corchuelo; Ángela Alarcon Cruz
EPI_ISL_3923743	LESP Chiapas	Instituto de Diagnostico y Referencia Epidemiologicos (INDRE)	Abril Rodriguez-Maldonado; Ariadna Medina-Benitez; Claudia Wong-Arambula; Ernesto Ramirez-Gonzalez.; Gisela Barrera-Badillo; Irma Lopez-Martinez; Joaquin Quiroz-Mercado; Lucia Hernandez-Rivas; Maribel Gonzalez-Villa; Natividad Cruz-Ortiz; Sergio Rangel-Guerrero; Tatiana Nunez-Garcia; Vanessa Rivero-Arredondo
EPI_ISL_3459941, EPI_ISL_3459942	LESP Queretaro	Instituto de Diagnostico y Referencia Epidemiologicos (INDRE)	Abril Rodriguez-Maldonado; Ariadna Medina-Benitez; Claudia Wong-Arambula; Ernesto Ramirez-Gonzalez.; Gisela Barrera-Badillo; Irma Lopez-Martinez; Joaquin Quiroz-Mercado; Lucia Hernandez-Rivas; Maribel Gonzalez-Villa; Natividad Cruz-Ortiz; Sergio Rangel-Guerrero; Tatiana Nunez-Garcia; Vanessa Rivero-Arredondo
EPI_ISL_3459945, EPI_ISL_3923753, EPI_ISL_3923754	LESP Sonora	Instituto de Diagnostico y Referencia Epidemiologicos (INDRE)	Abril Rodriguez-Maldonado; Ariadna Medina-Benitez; Claudia Wong-Arambula; Ernesto Ramirez-Gonzalez.; Gisela Barrera-Badillo; Irma Lopez-Martinez; Joaquin Quiroz-Mercado; Lucia Hernandez-Rivas; Maribel Gonzalez-Villa; Natividad Cruz-Ortiz; Sergio Rangel-Guerrero; Tatiana Nunez-Garcia; Vanessa Rivero-Arredondo
EPI_ISL_3459955, EPI_ISL_3769141, EPI_ISL_3923744	LESP Tabasco	Instituto de Diagnostico y Referencia Epidemiologicos (INDRE)	Abril Rodriguez-Maldonado; Ariadna Medina-Benitez; Claudia Wong-Arambula; Ernesto Ramirez-Gonzalez.; Gisela Barrera-Badillo; Irma Lopez-Martinez; Joaquin Quiroz-Mercado; Lucia Hernandez-Rivas; Maribel Gonzalez-Villa; Natividad Cruz-Ortiz; Sergio Rangel-Guerrero; Tatiana Nunez-Garcia; Vanessa Rivero-Arredondo
EPI_ISL_3508198	LESPH	T4 OLIGO	CONNIE ALEJANDRA HERRERA-GOMEZ; HECTOR ALBERTO VILLAFUENTES-TELLEZ; LAURA SOFIA CASTILLO-ORTEGA; MELITO GARCIA-REYES; OCTAVIO GARCIA-GONZALEZ; YAJAIRA SULIM VIGUERAS-MORALES
EPI_ISL_3868205, EPI_ISL_3868313	Lab voor klinische biologie	Lab voor klinische biologie	Bruno Verhasselt; Hannelore Hamerlinck; Marija Janevska
EPI_ISL_3801471	Labo Analyses Med	National Reference Center for Viruses of Respiratory Infections, Institut Pasteur, Paris	Angela Brisebarre; Camille Capel; Christophe Malabat; Corinne Maufrais; Etienne Simon-Lorière; Frédéric Lemoine; Hub de Bioinformatique et Biostatistique; Louise Lefrançois; Marion Barbet; Maud Vanpeene; Méline Bizard; Sylvie Behillil; Sylvie Van der Werf; Vincent Enouf; Vincent Vieillefond
EPI_ISL_3239261	Labor Becker & Kollegen (Standort München)	Robert Koch Institute	
EPI_ISL_3121513	Laborarztpraxis Osnabrück	Robert Koch Institute	
EPI_ISL_3129957, EPI_ISL_3129960, EPI_ISL_3129961, EPI_ISL_3129962, EPI_ISL_3277498, EPI_ISL_3277499, EPI_ISL_3277500, EPI_ISL_3501652, EPI_ISL_3501654, EPI_ISL_3501656, EPI_ISL_3501657, EPI_ISL_3501659, EPI_ISL_3903682, EPI_ISL_3903683, EPI_ISL_3903684, EPI_ISL_3903685, EPI_ISL_3903686			
see above	Laboratoire Carage	Institut Pasteur de la Guyane	Anne Lavergne; Antoine Enfissi; Arielle Salmier; Dominique Rousset
EPI_ISL_4028969, EPI_ISL_4028985, EPI_ISL_4028988, EPI_ISL_4028992, EPI_ISL_4028999, EPI_ISL_4029003	Laboratoire National de Santé Publique – LNSP	Genomics and Proteomics Departament, Gorgas Memorial Institute For Health Studies	Alexander A Martinez; Ambar Moreno; Claudia Estrada; Claudia Gonzalez V; César Roberto Conde Pereira; Jessica Gondola; Leyda Abrego; Marlene Castillo; Melissa Gaitan; Oris Chavarria
EPI_ISL_3800194	Laboratori de Referencia de Catalunya	Laboratori de Referencia de Catalunya	Bellosillo B.; Canal M.; Hernandez JJ.; Padilla E.; Ramirez A.; Vilas A.
EPI_ISL_3539231, EPI_ISL_3539235, EPI_ISL_3539236, EPI_ISL_3539237, EPI_ISL_4080819, EPI_ISL_4080821, EPI_ISL_4080822, EPI_ISL_4080823, EPI_ISL_4080828, EPI_ISL_4080829, EPI_ISL_4080830, EPI_ISL_4080831, EPI_ISL_4080833, EPI_ISL_4080835, EPI_ISL_4080836, EPI_ISL_4080837, EPI_ISL_4080838, EPI_ISL_4080840, EPI_ISL_4080842, EPI_ISL_4080844, EPI_ISL_4080845, EPI_ISL_4080846, EPI_ISL_4080853, EPI_ISL_4080854, EPI_ISL_4080859, EPI_ISL_4080860, EPI_ISL_4080863, EPI_ISL_4080867, EPI_ISL_4080868, EPI_ISL_4080872, EPI_ISL_4080875, EPI_ISL_4080876, EPI_ISL_4080877, EPI_ISL_4080878, EPI_ISL_4080879, EPI_ISL_4080880, EPI_ISL_4080887, EPI_ISL_4080889, EPI_ISL_4080891, EPI_ISL_4080892, EPI_ISL_4080895, EPI_ISL_4081028, EPI_ISL_4081068			
see above	Laboratorio Central de Saude Publica do Estado de Goias (LACEN/GO)	Laboratory of Respiratory Viruses and Measles, Oswaldo Cruz Institute, FIOCRUZ	Agatha Cristinne Prudencio; Alice Sampaio Rocha; Ana Carolina Mendonca; Ana Flavia Mendonça; Anna Carolina Paixao; Carmen Helena Ramos; Cassiane Casanova; Elisa Cavalcante Pereira; Fernando Motta; Flavia Pereira Amorim da Silva; Ighor Leonardo Arantes Gomes; Luciana Appolinario; Luiz Augusto Pereira; Marilda Siqueira on behalf of the Fiocruz COVID-19 Genomic Surveillance Network; Paola Resende; Rafael Souza Guedes; Renata Serrano Lopes; Taina Venas; Vinicius Lemes da Silva
EPI_ISL_3539239, EPI_ISL_3539240, EPI_ISL_3802966, EPI_ISL_4061289, EPI_ISL_4061290, EPI_ISL_4061291, EPI_ISL_4061293, EPI_ISL_4061295			
see above	Laboratorio Central de Saude Publica do Estado do Para (LACEN/PA)	Laboratory of Respiratory Viruses and Measles, Oswaldo Cruz Institute, FIOCRUZ	Agatha Soares; Alice Sampaio Rocha; Ana Carolina Mendonca; Anna Carolina Paixao; Elisa Cavalcante Pereira; Fernando Motta; Ighor Arantes; Luciana Appolinario; Marilda Siqueira on behalf of the Fiocruz COVID-19 Genomic Surveillance Network; Paola Resende; Renata Serrano Lopes; Taina Venas; Valnete Andrade
EPI_ISL_3245245, EPI_ISL_3245246, EPI_ISL_3245247, EPI_ISL_3245248, EPI_ISL_3245257, EPI_ISL_3245258, EPI_ISL_3245259, EPI_ISL_3245260, EPI_ISL_3245262, EPI_ISL_3245267, EPI_ISL_3245270, EPI_ISL_3245271, EPI_ISL_3245274, EPI_ISL_3245275, EPI_ISL_3245277, EPI_ISL_3245278, EPI_ISL_3245279, EPI_ISL_3245282, EPI_ISL_3245283, EPI_ISL_3245284, EPI_ISL_3245285, EPI_ISL_3245289, EPI_ISL_3245290, EPI_ISL_3245291, EPI_ISL_3245293, EPI_ISL_3245298, EPI_ISL_3245301, EPI_ISL_3245302, EPI_ISL_3245304, EPI_ISL_3245306, EPI_ISL_3245307, EPI_ISL_3245308, EPI_ISL_3245309, EPI_ISL_3245310, EPI_ISL_3245311, EPI_ISL_3245312, EPI_ISL_3245323, EPI_ISL_3245324, EPI_ISL_3245326, EPI_ISL_3245327, EPI_ISL_3245328, EPI_ISL_3245329, EPI_ISL_3245330, EPI_ISL_3245333, EPI_ISL_3245336, EPI_ISL_3615150, EPI_ISL_3615151, EPI_ISL_3615152, EPI_ISL_3615153, EPI_ISL_3615154, EPI_ISL_3615155, EPI_ISL_3615156, EPI_ISL_3615157, EPI_ISL_3615158, EPI_ISL_3615159, EPI_ISL_3615160, EPI_ISL_3615166, EPI_ISL_3615167, EPI_ISL_3615168, EPI_ISL_3615169, EPI_ISL_3615170, EPI_ISL_3615171, EPI_ISL_3615172, EPI_ISL_3615173, EPI_ISL_3615174, EPI_ISL_3615175, EPI_ISL_3615176, EPI_ISL_3615178, EPI_ISL_3615179, EPI_ISL_3615180, EPI_ISL_3615181, EPI_ISL_3615182, EPI_ISL_3615183, EPI_ISL_3615184, EPI_ISL_3615185, EPI_ISL_3615186, EPI_ISL_3615187, EPI_ISL_3615188, EPI_ISL_3615189, EPI_ISL_3615191, EPI_ISL_3615192, EPI_ISL_3615193, EPI_ISL_3615194, EPI_ISL_3615195, EPI_ISL_3615196, EPI_ISL_3615197, EPI_ISL_3615199, EPI_ISL_3615200, EPI_ISL_3615201, EPI_ISL_3615202, EPI_ISL_3615235, EPI_ISL_3615237, EPI_ISL_3615238, EPI_ISL_3920871, EPI_ISL_3920895, EPI_ISL_3920952, EPI_ISL_3920967, EPI_ISL_3920976, EPI_ISL_3920982, EPI_ISL_3920987, EPI_ISL_3921008, EPI_ISL_3921026, EPI_ISL_3921114, EPI_ISL_3921120, EPI_ISL_3921158, EPI_ISL_3921178, EPI_ISL_3921217, EPI_ISL_3921253			
see above	Laboratorio Central Noel Nutels	Bioinformatics Laboratory / LNCC	Alessandra P Lamarca; Alexandra L Gerber; Amilcar Tanuri; Ana Paula de C Guimaraes; Ana Tereza R Vasconcelos; Andrea Cony Cavalcanti; Caio Luiz Pereira Ribeiro; Cintia Policarpo; Claudia Maria Braga de Mello; Cristiane Gomes da Silva; Douglas Terra Machado; Erica Ramos dos Santos Nascimento; Fernanda Leitao dos Santos; Flavio Dias da Silva; Gleidson da Silva de Oliveira; Leandro Magalhaes de Souza; Liliane Cavalcante; Luiz G P de Almeida; Marcio Henrique de Oliveira Garcia; Mario Sergio Ribeiro; Ricardo Jose Barbosa Salviano; Ronaldo da Silva F Jr; Silvia Carvalho
EPI_ISL_3805733, EPI_ISL_3805734	Laboratorio Central de Epidemiologia (LCE)	Unidad de Genomica Avanzada	; Alejandra Garcia-Gasca; Alejandra Hernandez-Teran; Alejandro Sanchez-Flores; Alfredo Herrera-Estrella; Alicia Ocaña-Mondragon; Andreu Comas-Garcia; Angel Gustavo Salas-Lais; Antonio Loza Roman; Bernardo Martinez-Miguel; Blanca Taboada; Brenda Irasema Maldonado-Meza; Bruno Gomez-Gil; Carla Ivon Herrera-Najera; Carlos F. Arias; Celia Boukadida; Celida Duque Molina; Celida Martinez- Rodriguez; Clara Esperanza Santacruz-Tinoco; Concepcion Grajales-Muñiz; Consorcio Mexicano de Vigilancia Genomica (CoViGen-Mex). Authors (in alphabetical order): Julio Elias Alvarado-Yaah; Cristobal Chaidez-Quiroz; Daniel Fregoso-Rueda; Daniel Lira Morales; Eduardo Becerril-Vargas; Fernando Fontove-Herrera; Fidencio Mejia-Nepomuceno; Francisco Pulido; Gloria Elena Espinosa-Ayala; Gloria Maria Molina-Salinas; Gloria Vazquez; Hector Esteban Paz-Juarez; Hector Montoya-Fuentes; Helen Haydee Fernanda Ramirez-Plascencia; Irvin Gonzalez-Lopez; Jean Pierre Gonzalez; Jesus Hernandez; Joel Armando Vazquez-Perez.; Jorge Salas-Hernandez; Jose Antonio Enciso-Moreno; Jose Arturo Martinez-Orozco; Jose Esteban Muñoz-Medina; Jose de Jesus Nuñez-Contreras; Juan Bautista Chale-Dzul; Julissa Enciso-Ibarra; Luis Alberto Ochoa-Carrera; Margarita Matias-Florentino; Maria Guadalupe Santiago-Mauricio; Maria Guadalupe de Jesus Mireles-Rivera; Mario Mujica-Sanchez; Marissa Perez-Garcia; Nelly Selem-Mojica; Pavel Isa; Ricardo Ciria Merce; Ricardo Grande; Rosa Maria Gutierrez Rios; Santiago avila-Rios; Selene Zarate; Susana Lopez; Veronica Mata-Haro; Victor Eduardo Garcia-Arias; Victor Hugo Borja-Aburto
EPI_ISL_3557087	Laboratorio Central de Epidemiología (LCE)	Centro de Investigación en Enfermedades Infecciosas (CIENI), Instituto Nacional de Enfermedades Respiratorias (INER)	Alejandra García-Gasca; Alejandra Hernández-Terán; Alejandro Sánchez-Flores; Alfredo Herrera-Estrella; Alicia Ocaña-Mondragón; Andreu Comas-García; Angel Gustavo Salas-Lais; Antonio Loza Román; Bernardo Martínez-Miguel; Blanca Taboada; Brenda Irasema Maldonado-Meza; Bruno Gómez-Gil; Carla Ivón Herrera-Najera; Carlos F. Arias; Celia Boukadida; Clara Esperanza Santacruz-Tinoco; Concepción Grajales-Muñiz; Consorcio Mexicano de Vigilancia Genómica (CoViGen-Mex). Authors (in alphabetical order): Julio Elias Alvarado-Yaah; Cristóbal Cháidez-Quiróz; Célida Duque Molina; Célida Martínez- Rodríguez; Daniel Fregoso-Rueda; Daniel Lira Morales; Eduardo Becerril-Vargas; Fernando Fontove-Herrera; Fidencio Mejía-Nepomuceno; Francisco Pulido; Gloria Elena Espinosa-Ayala; Gloria María Molina-Salinas; Gloria Vazquez; Hector Esteban Paz-Juárez; Hector Montoya-Fuentes; Helen Haydee Fernanda Ramírez-Plascencia; Irvin González-López; Jean Pierre González; Jesús Hernández; Joel Armando Vázquez-Pérez.; Jorge Salas-Hernández; José Antonio Enciso-Moreno; José Arturo Martínez-Orozco; José Esteban Muñoz-Medina; José de Jesús Nuñez-Contreras; Juan Bautista Chale-Dzul; Julissa Enciso-Ibarra; Kathia Elizabeth Tapia-Diaz; Luis Alberto Ochoa-Carrera; Margarita Matías-Florentino; Mario Mújica-Sánchez; Marissa Perez-Garcia; María Guadalupe Santiago-Mauricio; María Guadalupe de Jesús Mireles-Rivera; Nelly Sélem-Mojica; Pavel Isa; Ricardo Ciria Merce; Ricardo Grande; Rosa María Gutiérrez Rios; Santiago Ávila-Ríos; Selene Zárate; Susana Lopez; Verónica Mata-Haro; Victor Eduardo García-Arias; Víctor Hugo Borja-Aburto
EPI_ISL_3347549, EPI_ISL_3347559	Laboratorio Central de Epidemiología (LCE)	Instituto de Biotecnología de la UNAM	; Alejandra García-Gasca; Alejandra Hernández-Terán; Alejandro Sánchez-Flores; Alfredo Herrera-Estrella; Alicia Ocaña-Mondragón; Andreu Comas-García; Angel Gustavo Salas-Lais; Antonio Loza Román; Bernardo Martínez-Miguel; Blanca Taboada; Brenda Irasema Maldonado-Meza; Bruno Gómez-Gil; Carla Ivón Herrera-Najera; Carlos F. Arias; Celia Boukadida; Clara Esperanza Santacruz-Tinoco; Concepción Grajales-Muñiz; Consorcio Mexicano de Vigilancia Genómica (CoViGen-Mex). Authors (in alphabetical order): Julio Elias Alvarado-Yaah; Cristóbal Cháidez-Quiróz; Célida Duque Molina; Célida Martínez- Rodríguez; Daniel Fregoso-Rueda; Daniel Lira Morales; Eduardo Becerril-Vargas; Fernando Fontove-Herrera; Fidencio Mejía-Nepomuceno; Francisco Pulido; Gloria Elena Espinosa-Ayala; Gloria María Molina-Salinas; Gloria Vazquez; Hector Esteban Paz-Juárez; Hector Montoya-Fuentes; Helen Haydee Fernanda Ramírez-Plascencia; Irvin González-López; Jean Pierre González; Jesús Hernández; Joel Armando Vázquez-Pérez).; Jorge Salas-Hernández; José Antonio Enciso-Moreno; José Arturo Martínez-Orozco; José Esteban Muñoz-Medina; José de Jesús Nuñez-Contreras; Juan Bautista Chale-Dzul; Julissa Enciso-Ibarra; Kathia Elizabeth Tapia-Diaz; Luis Alberto Ochoa-Carrera; Margarita Matías-Florentino; Mario Mújica-Sánchez; Marissa Perez-Garcia; María Guadalupe Santiago-Mauricio; María Guadalupe de Jesús Mireles-Rivera; Nelly Sélem-Mojica; Pavel Isa; Ricardo Ciria Merce; Ricardo Grande; Rosa María Gutiérrez Rios; Santiago Ávila-Ríos; Selene Zárate; Susana Lopez; Verónica Mata-Haro; Victor Eduardo García-Arias; Víctor Hugo Borja-Aburto

EPI_ISL_3825480	Laboratorio Central de Salud Publica	Laboratorio de Biología Molecular, Instituto de Medicina Regional on behalf of 'Proyecto Argentino Interinstitucional de genomica de SARS-CoV-2' (PAIS Consortium)	Andrea Ayala; Bettina Brusés; Erica Struss; Esteban Paredes; Griselda Oria; Horacio Lucero.; Javier Mussin; Laura Formichelli; Melina Lorenzini Campos; Raúl Maximiliano Acevedo; Verónica Gómez; Victoria Femenías
EPI_ISL_3235235, EPI_ISL_3235236, EPI_ISL_3235237, EPI_ISL_4061421, EPI_ISL_4061422, EPI_ISL_4061423	Laboratorio Central de Saude Publica do Estado da Bahia (LACEN/BA)	Laboratory of Respiratory Viruses and Measles, Oswaldo Cruz Institute, FIOCRUZ	Agatha Soares; Alice Sampaio Rocha; Ana Carolina Mendonca; Anna Carolina Paixao; Elisa Cavalcante Pereira; Felicidade Pereira; Fernando Motta; Ighor Arantes; Luciana Appolinario; Marilda Siqueira on behalf of the Fiocruz COVID-19 Genomic Surveillance Network; Paola Resende; Renata Serrano Lopes; Taina Venas
EPI_ISL_3235244, EPI_ISL_3434759, EPI_ISL_3434760, EPI_ISL_3434761, EPI_ISL_3434766, EPI_ISL_3434769, EPI_ISL_3434770, EPI_ISL_3434779, EPI_ISL_3434780, EPI_ISL_3434781, EPI_ISL_3434782, EPI_ISL_3434789, EPI_ISL_3832397, EPI_ISL_4081069, EPI_ISL_4081070, EPI_ISL_4081071, EPI_ISL_4081072, EPI_ISL_4081073, EPI_ISL_4081074, EPI_ISL_4081075, EPI_ISL_4081076, EPI_ISL_4081077, EPI_ISL_4081078, EPI_ISL_4081079, EPI_ISL_4081080, EPI_ISL_4081081, EPI_ISL_4081082, EPI_ISL_4081083, EPI_ISL_4081084, EPI_ISL_4081086, EPI_ISL_4081088, EPI_ISL_4081089			
see above	Laboratorio Central de Saude Publica do Estado da Paraiba (LACEN/PB)	Laboratory of Respiratory Viruses and Measles, Oswaldo Cruz Institute, FIOCRUZ	Alice Sampaio Rocha; Ana Carolina Mendonca; Anna Carolina Paixao; Dalane Loudal Florentino Teixeira; Elisa Cavalcante Pereira; Fernando Motta; Joao Felipe Bezerra; Luciana Appolinario; Marilda Siqueira on behalf of the Fiocruz COVID-19 Genomic Surveillance Network; Paola Resende; Renata Serrano Lopes; Taina Venas
EPI_ISL_3434820, EPI_ISL_3434844, EPI_ISL_3434845, EPI_ISL_3434846, EPI_ISL_3434847, EPI_ISL_3434848, EPI_ISL_3434894, EPI_ISL_3434896, EPI_ISL_3434943, EPI_ISL_3802904, EPI_ISL_3802905, EPI_ISL_3802906, EPI_ISL_3802907, EPI_ISL_3802908, EPI_ISL_3802909, EPI_ISL_3802910, EPI_ISL_3802911, EPI_ISL_3802912, EPI_ISL_3802913, EPI_ISL_3802914, EPI_ISL_3802915, EPI_ISL_3802916, EPI_ISL_3802917, EPI_ISL_3802918, EPI_ISL_3802919, EPI_ISL_3802921, EPI_ISL_3802922, EPI_ISL_3802923, EPI_ISL_3802924, EPI_ISL_3802925, EPI_ISL_3802926, EPI_ISL_3802927, EPI_ISL_3802929, EPI_ISL_3802930, EPI_ISL_3802931, EPI_ISL_3802932, EPI_ISL_4061393, EPI_ISL_4061394, EPI_ISL_4061395, EPI_ISL_4061396, EPI_ISL_4061397, EPI_ISL_4061398, EPI_ISL_4061399, EPI_ISL_4061400, EPI_ISL_4061401, EPI_ISL_4061402, EPI_ISL_4061403, EPI_ISL_4061404, EPI_ISL_4061405, EPI_ISL_4061407, EPI_ISL_4061409, EPI_ISL_4061410, EPI_ISL_4061411, EPI_ISL_4061412, EPI_ISL_4061413, EPI_ISL_4061415, EPI_ISL_4061417, EPI_ISL_4061418, EPI_ISL_4061419, EPI_ISL_4061420			
see above	Laboratorio Central de Saude Publica do Estado de Alagoas (LACEN/AL)	Laboratory of Respiratory Viruses and Measles, Oswaldo Cruz Institute, FIOCRUZ	Agatha Soares; Alice Sampaio Rocha; Ana Carolina Mendonca; Anderson Brandao Leite; Anna Carolina Paixao; Elisa Cavalcante Pereira; Fernando Motta; Ighor Arantes; Luciana Appolinario; Marilda Siqueira on behalf of the Fiocruz COVID-19 Genomic Surveillance Network; Paola Resende; Renata Serrano Lopes; Rubens Pasa; Taina Venas
EPI_ISL_3434792, EPI_ISL_3434793, EPI_ISL_3434794, EPI_ISL_3434796, EPI_ISL_3434797, EPI_ISL_3434800, EPI_ISL_3434918, EPI_ISL_3434919, EPI_ISL_3434920, EPI_ISL_3434921, EPI_ISL_3434922, EPI_ISL_3434923, EPI_ISL_3434924, EPI_ISL_3435054, EPI_ISL_3435055, EPI_ISL_3435056, EPI_ISL_3435057, EPI_ISL_3435058, EPI_ISL_3435061, EPI_ISL_3435062, EPI_ISL_3435063, EPI_ISL_3435064, EPI_ISL_3435066, EPI_ISL_3539245, EPI_ISL_3539772, EPI_ISL_3539773, EPI_ISL_3827990, EPI_ISL_3828012, EPI_ISL_3828016			
see above	Laboratorio Central de Saude Publica do Estado de Santa Catarina (LACEN/SC)	Laboratory of Respiratory Viruses and Measles, Oswaldo Cruz Institute, FIOCRUZ	Alice Sampaio Rocha; Ana Carolina Mendonca; Anna Carolina Paixao; Darcita Buerger Rovaris; Elisa Cavalcante Pereira; Fernando Motta; Luciana Appolinario; Marilda Siqueira on behalf of the Fiocruz COVID-19 Genomic Surveillance Network; Paola Resende; Renata Serrano Lopes; Sandra Bianchini Fernandes; Taina Venas
EPI_ISL_4061449, EPI_ISL_4061450	Laboratorio Central de Saude Publica do Estado de Sergipe (LACEN/SE)	Laboratory of Respiratory Viruses and Measles, Oswaldo Cruz Institute, FIOCRUZ	Agatha Soares; Alice Sampaio Rocha; Ana Carolina Mendonca; Anna Carolina Paixao; Cliomar Alves dos Santos; Elisa Cavalcante Pereira; Fernando Motta; Ighor Arantes; Luciana Appolinario; Marilda Siqueira on behalf of the Fiocruz COVID-19 Genomic Surveillance Network; Paola Resende; Renata Serrano Lopes; Tainá Moreira Martins Venas
EPI_ISL_3539795, EPI_ISL_3539796, EPI_ISL_3539797, EPI_ISL_3539798, EPI_ISL_3539799, EPI_ISL_3539800, EPI_ISL_3539801, EPI_ISL_3539802, EPI_ISL_3539803, EPI_ISL_3539804, EPI_ISL_3539805, EPI_ISL_3539806, EPI_ISL_3539807, EPI_ISL_3539808, EPI_ISL_3539809, EPI_ISL_3539811, EPI_ISL_3539813, EPI_ISL_3539814, EPI_ISL_3539815, EPI_ISL_3539816, EPI_ISL_3539817, EPI_ISL_3539818, EPI_ISL_3539819, EPI_ISL_3539820, EPI_ISL_3539821, EPI_ISL_3539822, EPI_ISL_3539823, EPI_ISL_3539824, EPI_ISL_3539825, EPI_ISL_3539826, EPI_ISL_3539828, EPI_ISL_4081029, EPI_ISL_4081030, EPI_ISL_4081031, EPI_ISL_4081033, EPI_ISL_4081034, EPI_ISL_4081035, EPI_ISL_4081036, EPI_ISL_4081037, EPI_ISL_4081038, EPI_ISL_4081039, EPI_ISL_4081040, EPI_ISL_4081041, EPI_ISL_4081042, EPI_ISL_4081044, EPI_ISL_4081046, EPI_ISL_4081047, EPI_ISL_4081049, EPI_ISL_4081050, EPI_ISL_4081052, EPI_ISL_4081053, EPI_ISL_4081054, EPI_ISL_4081056, EPI_ISL_4081057, EPI_ISL_4081058, EPI_ISL_4081059, EPI_ISL_4081060, EPI_ISL_4081061, EPI_ISL_4081062, EPI_ISL_4081063, EPI_ISL_4081065, EPI_ISL_4081066			
see above	Laboratorio Central de Saude Publica do Estado do Amapa (LACEN/AP)	Laboratory of Respiratory Viruses and Measles, Oswaldo Cruz Institute, FIOCRUZ	Agatha Cristinne Prudencio; Alice Sampaio Rocha; Ana Carolina Mendonca; Andreia Santos Costa; Anna Carolina Paixao; Anne Caroline da Silva Soledade; Elisa Cavalcante Pereira; Fernando Motta; Ighor Leonardo Arantes Gomes; Lindomar dos Anjos Silva; Luciana Appolinario; Marcia Socorro Pereira Cavalcante; Marilda Siqueira on behalf of the Fiocruz COVID-19 Genomic Surveillance Network; Paola Resende; Renata Serrano Lopes; Taina Venas
EPI_ISL_3434721, EPI_ISL_3434729, EPI_ISL_3434730, EPI_ISL_3434732, EPI_ISL_3434733, EPI_ISL_3434734, EPI_ISL_3434735, EPI_ISL_3434736, EPI_ISL_3434737, EPI_ISL_3434738, EPI_ISL_3434739, EPI_ISL_3434740, EPI_ISL_3434741, EPI_ISL_3434744, EPI_ISL_3434745, EPI_ISL_3434747, EPI_ISL_3434748, EPI_ISL_3434951, EPI_ISL_3434989, EPI_ISL_3434990, EPI_ISL_3434992, EPI_ISL_3434993, EPI_ISL_3434994, EPI_ISL_3434999, EPI_ISL_3435000, EPI_ISL_3435001, EPI_ISL_3435002, EPI_ISL_3435003, EPI_ISL_3435004, EPI_ISL_3435006, EPI_ISL_3435007, EPI_ISL_3435009, EPI_ISL_3435010, EPI_ISL_3435012, EPI_ISL_3435017, EPI_ISL_3435018, EPI_ISL_3435020, EPI_ISL_3435021, EPI_ISL_3435022, EPI_ISL_3435023, EPI_ISL_3435026, EPI_ISL_3435027, EPI_ISL_3435028, EPI_ISL_3435033, EPI_ISL_3435034, EPI_ISL_3435036, EPI_ISL_3435040, EPI_ISL_3435041, EPI_ISL_3435068, EPI_ISL_3539933, EPI_ISL_3539934, EPI_ISL_3827993, EPI_ISL_3828001, EPI_ISL_3828009, EPI_ISL_3828014, EPI_ISL_4080765			
see above	Laboratorio Central de Saude Publica do Estado do Espirito Santo (LACEN/ES)	Laboratory of Respiratory Viruses and Measles, Oswaldo Cruz Institute, FIOCRUZ	Alice Sampaio Rocha; Ana Carolina Mendonca; Anna Carolina Paixao; Eilisa Cavalcante Pereira; Elisa Cavalcante Pereira; Fernando Motta; Luciana Appolinario; Marilda Siqueira on behalf of the Fiocruz COVID-19 Genomic Surveillance Network; Paola Resende; Renata Serrano Lopes; Rodrigo Ribeiro Rodrigues; Taina Venas
EPI_ISL_3914061, EPI_ISL_3914069, EPI_ISL_3914078, EPI_ISL_3914099, EPI_ISL_3914116, EPI_ISL_3914138, EPI_ISL_3914150, EPI_ISL_3914202, EPI_ISL_3914219, EPI_ISL_3914249, EPI_ISL_3914254, EPI_ISL_3914291, EPI_ISL_3914331, EPI_ISL_3914334, EPI_ISL_3914339			
see above	Laboratorio Central de Saude Publica do Estado do Parana (Instituto de Biologia Molecular do Paraná (LACEN-PR)	Instituto Carlos Chagas - Fiocruz	Alessandra De Melo Aguiar; Andreia Akemi Suzukawa; Andréa Rodrigues Ávila; Bruno Dallagiovanna; Dalila Zanette; Eduardo Balsanelli; Emanuel Maltempi de Souza; Fabio Passetti; Fabricio Klerynton Marchini; Fábio de Oliveira Pedrosa; Guilherme Becker; Helisson Faoro; Hellen Geremias dos Santos; Irina Nastassja Riediger; Letusa Albrecht; Lucas Blanes; Luis Gustavo Morello; Lysangela Ronalte Alves; Maria do Carmo Debur; Mauro de Medeiros Oliveira; Michelle Orane Schemberger; Paola Cristina Resende; Sheila Cristina Nardeli; Tiago Gräf; Valter Antônio de Baura
EPI_ISL_4061330, EPI_ISL_4061424	Laboratorio Central de Saude Publica do Estado do Parana (LACEN/PR)	Laboratory of Respiratory Viruses and Measles, Oswaldo Cruz Institute, FIOCRUZ	Agatha Soares; Alice Sampaio Rocha; Ana Carolina Mendonca; Anna Carolina Paixao; Elisa Cavalcante Pereira; Fernando Motta; Ighor Leonardo Arantes; Irina Riediger; Luciana Appolinario; Marilda Siqueira on behalf of the Fiocruz COVID-19 Genomic Surveillance Network; Paola Resende; Renata Serrano Lopes; Taina Venas
EPI_ISL_3235268, EPI_ISL_3235270, EPI_ISL_3235271, EPI_ISL_3539831, EPI_ISL_3539832, EPI_ISL_3539834, EPI_ISL_3539837, EPI_ISL_3539838, EPI_ISL_3539839, EPI_ISL_3832401, EPI_ISL_3832403, EPI_ISL_3832404, EPI_ISL_3832405, EPI_ISL_3832406, EPI_ISL_3832407, EPI_ISL_3832408, EPI_ISL_3832409			
see above	Laboratorio Central de Saude Publica do Estado do Rio Grande do Norte (LACEN/RN)	Laboratory of Respiratory Viruses and Measles, Oswaldo Cruz Institute, FIOCRUZ	Agatha Soares; Alice Sampaio Rocha; Ana Carolina Mendonca; Ana Paula Ferreira Costa; Anna Carolina Paixao; Antonnyo Palmielly Diogenes Lima; Aurélio de Oliveira Bento; Elisa Cavalcante Pereira; Fernando Motta; Gessika Brenna Costa Alves; Heglayne Pereira Vital da Silva; Iago de Souza Gomes; Ighor Arantes; Isabelle Cristina Clemente dos Santos; Janaina Sonale Cavalcante Nogueira de Oliveira; Jayra Juliana Paiva Alves Abrantes; Jonas José da Silva; Luciana Appolinario; Marilda Siqueira on behalf of the Fiocruz COVID-19 Genomic Surveillance Network; Paola Resende; Renata Serrano Lopes; Taina Venas; Themis Rocha de Souza; Vítor Gabriel Saldanha Fernandes
EPI_ISL_3190298, EPI_ISL_3190300, EPI_ISL_3190301, EPI_ISL_3190306, EPI_ISL_3190307, EPI_ISL_3190308, EPI_ISL_3235231, EPI_ISL_3235232, EPI_ISL_3235233, EPI_ISL_3235234, EPI_ISL_3235276, EPI_ISL_3235277, EPI_ISL_3235279, EPI_ISL_3235284, EPI_ISL_3235285, EPI_ISL_3235286, EPI_ISL_3235287, EPI_ISL_3235288, EPI_ISL_3539922, EPI_ISL_3832417, EPI_ISL_3832418, EPI_ISL_3832419, EPI_ISL_3832420, EPI_ISL_3832421, EPI_ISL_3832422, EPI_ISL_3832423, EPI_ISL_3832424, EPI_ISL_3832425, EPI_ISL_3832426, EPI_ISL_3832428, EPI_ISL_3832429, EPI_ISL_3832431, EPI_ISL_3832432, EPI_ISL_3832433, EPI_ISL_3832434, EPI_ISL_3832435, EPI_ISL_3832436, EPI_ISL_3832437, EPI_ISL_3832438			
see above	Laboratorio Central de Saude Publica do Estado do Rio Grande do Sul (LACEN-RS)	Laboratory of Respiratory Viruses and Measles, Oswaldo Cruz Institute, FIOCRUZ	Agatha Cristinne Soares; Alice Sampaio Rocha; Ana Carolina Mendonca; Anderson Brandao Leite; Anna Carolina Paixao; Elisa Cavalcante Pereira; Fernando Motta; Ighor Leonardo Arantes; Luciana Appolinario; Marilda Siqueira on behalf of the Fiocruz COVID-19 Genomic Surveillance Network; Paola Resende; Renata Serrano Lopes; Richard Salvato; Taina Venas; Tatiana Schaffer Gregianini
EPI_ISL_3235260, EPI_ISL_3235261, EPI_ISL_3235263, EPI_ISL_3235266, EPI_ISL_3434790, EPI_ISL_3434941, EPI_ISL_3539766, EPI_ISL_4061336, EPI_ISL_4080788			
see above	Laboratorio Central de Saude Publica do Estado do Rio de Janeiro (LACEN/RJ)	Laboratory of Respiratory Viruses and Measles, Oswaldo Cruz Institute, FIOCRUZ	Agatha Cristinne Prudencio Soares; Agatha Soares; Alice Sampaio Rocha; Ana Carolina Mendonca; Andrea Cony Cavalcanti; Anna Carolina Paixao; Elisa Cavalcante Pereira; Fernando Motta; Ighor Arantes; Ighor Leonardo Arantes Gomes; Luciana Appolinario; Marilda Siqueira on behalf of the Fiocruz COVID-19 Genomic Surveillance Network; Paola Resende; Renata Serrano Lopes; Taina Venas
EPI_ISL_4080800, EPI_ISL_4080801	Laboratorio Central de Saude Publica do Estado do Tocantins (LACEN/TO)	Laboratory of Respiratory Viruses and Measles, Oswaldo Cruz Institute, FIOCRUZ	Agatha Soares; Alice Sampaio Rocha; Ana Carolina Mendonca; Anna Carolina Paixao; Elisa Cavalcante Pereira; Fernando Motta; Ighor Arantes; Jucimaria Dantas Galvao; Luciana Appolinario; Marilda Siqueira on behalf of the Fiocruz COVID-19 Genomic Surveillance Network; Paola Resende; Renata Serrano Lopes; Taina Venas
EPI_ISL_4003464	Laboratorio Central, Ministerio de Salud Córdoba	Instituto de Patologia Vegetal (CIAP-INTA) on behalf of 'Proyecto Argentino Interinstitucional de genomica de SARS-CoV-2' (PAIS Consortium)	Barbas, G.; Castro, G.; Debat, HJ.; FD; Fernández; M; M.B.; Marquez, N.; Pisano; Re, V.
EPI_ISL_3527817, EPI_ISL_3527818, EPI_ISL_4051092	Laboratorio Nacional de Salud	Asociación de Salud Integral / Clínica Familiar "Luis Ángel García"	Ana S. Gonzalez-Reiche; Claudia Rangel; Danicela Mercado; Eduardo Arathon; Hilda Ruiz; Luis Aguirre; Luis Rivas; Narda Medina; Oscar Bonilla; Osmar Gamboa.
EPI_ISL_4029327	Laboratorio Nacional de Salud, Ministerio de Salud Publica y Asistencia Social	Genomics and Proteomics Department, Gorgas Memorial Institute For Health Studies	Alexander A Martinez; Ambar Moreno; Claudia Estrada; Claudia Gonzalez V; César Roberto Conde Pereira; Jessica Gondola; Leyda Abrego; Marlene Castillo; Melissa Gaitan; Oris Chavarria
EPI_ISL_3707440	Laboratorio de Genómica Microbiana, Universidad Peruana Cayetano Heredia	cov0937	Alejandra Dávila-Barclay; Diego Cuicapuza; Guillermo Salvatierra; Janet Huancachoque; Luis González; Pablo Tsukayama; Pedro E. Romero; Pool Marcos
EPI_ISL_3535744, EPI_ISL_3535754, EPI_ISL_3642589, EPI_ISL_3642591, EPI_ISL_3758927, EPI_ISL_3758932, EPI_ISL_3758949, EPI_ISL_3758953, EPI_ISL_3758955, EPI_ISL_3944414			
see above	Laboratorio de Infectologia y Virologia Molecular	Laboratory of Molecular Virology, School of Medicine, Pontificia Universidad Catolica de Chile	Ana Maria Contreras; Andres E. Munoz-Marcos; Carlos Palma; Catalina Pardo-Roa; Constanza Maldonado; Constanza Martinez-Valdevenito; Eileen Serrano; Erick Salinas; Estefany Poblete; Francisco Melo; Jennifer Angulo; Jorge Levican; Leonardo I. Almonacid; M. Belen Leyton; Marcela Ferres; Maria Jose Avendano; Rafael A. Medina; Tamara Garcia-Salum
EPI_ISL_3134280, EPI_ISL_3134281	Laboratorio de Medicina Molecular, Universidad de Magallanes	Centro Asistencial Docente y de Investigacion, Universidad de Magallanes	Carolina Pérez; Constanza Ceroni; Diego Alvarez; Inés Cid; Jacqueline Aldridge; Jorge González; Juan Ríos; Marcelo Navarrete

EPI_ISL_3761574, EPI_ISL_3761579, EPI_ISL_3761580, EPI_ISL_3761581, EPI_ISL_3761582, EPI_ISL_3761585, EPI_ISL_3761591, EPI_ISL_3761592, EPI_ISL_3761594, EPI_ISL_3761595, EPI_ISL_3761596, EPI_ISL_3761598, EPI_ISL_3761601, EPI_ISL_3761606, EPI_ISL_3761609, EPI_ISL_3761610, EPI_ISL_3761611, EPI_ISL_3761612, EPI_ISL_3761620, EPI_ISL_3761622, EPI_ISL_3761624, EPI_ISL_3761627, EPI_ISL_3761628, EPI_ISL_3761630, EPI_ISL_3761632, EPI_ISL_3761704, EPI_ISL_3761706, EPI_ISL_3761707, EPI_ISL_3761708, EPI_ISL_3761709, EPI_ISL_3761710, EPI_ISL_3761711, EPI_ISL_3761712, EPI_ISL_3761713, EPI_ISL_3761714, EPI_ISL_3761715, EPI_ISL_3761717, EPI_ISL_3761718, EPI_ISL_3761719, EPI_ISL_3761720, EPI_ISL_3761721, EPI_ISL_3761722, EPI_ISL_3761723, EPI_ISL_3761725, EPI_ISL_3761726, EPI_ISL_3761727, EPI_ISL_3761728, EPI_ISL_3761729, EPI_ISL_3761730, EPI_ISL_3761731, EPI_ISL_3761732, EPI_ISL_3761733, EPI_ISL_3761734, EPI_ISL_3761735, EPI_ISL_3761737, EPI_ISL_3761738, EPI_ISL_3761739, EPI_ISL_3761741, EPI_ISL_3761742, EPI_ISL_3761743, EPI_ISL_3761744, EPI_ISL_3761745, EPI_ISL_3761746, EPI_ISL_3761747, EPI_ISL_3761748, EPI_ISL_3761764			
see above	Laboratorio de Pesquisa em Virologia, FAMERP, SJRP	Laboratorio de Pesquisa em Virologia, FAMERP, SJRP	Beatriz de Carvalho Marques; Cecília Artico Banho; Cíntia Bittar; Fábio Sossai Possebon; Guilherme Campos; Helena Lage Ferreira; Jorge A. Petroli Marchesi; João Pessoa Araújo Jr.; Leila Sabrina Ullmann; Lívia Sacchetto; Maisa C. Pereira Parra; Marília Moraes; Maurício L. Nogueira.; Paula Rahal; Paulo Inacio da Costa
EPI_ISL_3128480, EPI_ISL_3128489, EPI_ISL_3128514	Laboratorio di Riferimento Regionale della Sicilia Occidentale per l'Emergenza COVID-19	Laboratorio di Riferimento Regionale della Sicilia Occidentale per l’Emergenza COVID-19	Carmelo Massimo Maida; Claudio Costantino; Daniela Di Naro; Fabio Tramuto; Francesco Vitale; Giorgio Graziano; Giulia Randazzo; Vincenzo Restivo; Walter Mazzucco
EPI_ISL_3112892, EPI_ISL_3112926, EPI_ISL_3112966, EPI_ISL_3326658, EPI_ISL_3327091, EPI_ISL_3327106, EPI_ISL_3327216, EPI_ISL_3327292, EPI_ISL_3327316, EPI_ISL_3327427, EPI_ISL_3327588, EPI_ISL_3327645, EPI_ISL_3327692, EPI_ISL_3327808, EPI_ISL_3327818, EPI_ISL_3327821, EPI_ISL_3328088, EPI_ISL_3328830, EPI_ISL_3328910, EPI_ISL_3329030, EPI_ISL_3329416, EPI_ISL_3329419, EPI_ISL_3329810, EPI_ISL_3330061, EPI_ISL_3330610, EPI_ISL_3430984, EPI_ISL_3431004, EPI_ISL_3509873, EPI_ISL_3511002, EPI_ISL_3511592, EPI_ISL_3511728, EPI_ISL_3512236, EPI_ISL_3512804, EPI_ISL_3512852, EPI_ISL_3513703, EPI_ISL_3514248, EPI_ISL_3514551, EPI_ISL_3514681, EPI_ISL_3514751, EPI_ISL_3515016, EPI_ISL_3515472, EPI_ISL_3516400, EPI_ISL_3516594, EPI_ISL_3517195, EPI_ISL_3517622, EPI_ISL_3517635, EPI_ISL_3517743, EPI_ISL_3517752, EPI_ISL_3517886, EPI_ISL_3517888, EPI_ISL_3517889, EPI_ISL_3517894, EPI_ISL_3518681, EPI_ISL_3518713, EPI_ISL_3518737, EPI_ISL_3518906, EPI_ISL_3518952, EPI_ISL_3519272, EPI_ISL_3519538, EPI_ISL_3519674, EPI_ISL_3519678, EPI_ISL_3519729, EPI_ISL_3519755, EPI_ISL_3520352, EPI_ISL_3606078, EPI_ISL_3606095, EPI_ISL_3606481, EPI_ISL_3607175, EPI_ISL_3607407, EPI_ISL_3677210, EPI_ISL_3677950, EPI_ISL_3678937, EPI_ISL_3679475, EPI_ISL_3680607, EPI_ISL_3682881, EPI_ISL_3685600, EPI_ISL_3685808, EPI_ISL_3686172, EPI_ISL_3686239, EPI_ISL_3686347, EPI_ISL_3686369, EPI_ISL_3686758, EPI_ISL_3687475, EPI_ISL_3687570, EPI_ISL_3688090, EPI_ISL_3740926, EPI_ISL_3740930, EPI_ISL_3740967, EPI_ISL_3744016, EPI_ISL_3744061, EPI_ISL_3744510, EPI_ISL_3746227, EPI_ISL_3746785, EPI_ISL_3748522, EPI_ISL_3749211, EPI_ISL_3749538, EPI_ISL_3749614, EPI_ISL_3750255, EPI_ISL_3750679, EPI_ISL_3750743, EPI_ISL_3751992, EPI_ISL_3752013, EPI_ISL_3752144			
see above	Laboratory Corporation of America	Centers for Disease Control and Prevention Division of Viral Diseases, Pathogen Discovery	Adrian Paskey; Amanda Douglas; Amanda Suchanek; Andrea Throop; Ayla Burns; Benjamin Rambo-Martin; Bobbi Croy; Brian Krueger; Brian Norvell; Christopher Gulvick; Christos Petropoulos; Clinton Paden; Clinton R. Paden; Craig Lukasik; Dakota Howard; Darlene Wagner; Debbie Boles; Dhwani Batra; Duncan MacCannell; Eyad Almasri; Goran Stevovic; Howard Engler; Hrushikesh Deshmukh; Jake Humphrey; Jana Schroth; Jason Caravas; Joe Voshell; John Pruitt; Jonathan Meltzer; Jonathan Williams; Kara Moser; Kimberly Wagner; Lax Iyer; Lisa Pfefferle; Lyndon Tilson; Manoj Jain; Marcia Eisenberg; Mary Ann Cristobal; Mary Cristobal; Mary Williamson; Matthew Robinson; Matthew Schmerer; Michael Levandoski; Mike Sapeta; Mindy Nye; Minoo Agarwal; Mohan Kolli; Nuthawin Charoensri; Oren Cohen; Peter Cook; Peter W. Cook; Prashant Gupta; Qian Zeng; Rama Ghatti; Scott Parker; Scott Ryan; Scott Sammons; Shatavia Morrison; Stanley Letovsky; Steven Ragan; Suresh Babu Selvaraju; Suresh Selvaraju; Susan Countryman; Susan Hicks; Suzanne Dale; Thomas Urban; Tim Kuphal; Tricia Zwiefelhofer; Vincent Drouillon; Yvette Unoarumhi
EPI_ISL_3664280	Laboratory for Molecular Diagnostics,IPHMN	Eurofins Genomics Europe Sequencing GmbH	Danijela Vujošević
EPI_ISL_3832400	Laboratory of Respiratory Viruses and Measles, Oswaldo Cruz Institute, FIOCRUZ	Laboratory of Respiratory Viruses and Measles, Oswaldo Cruz Institute, FIOCRUZ	Agatha Soares; Alice Sampaio Rocha; Ana Carolina Mendonca; Anna Carolina Paixao; Elisa Cavalcante Pereira; Fernando Motta; Ighor Arantes; Luciana Appolinario; Marilda Siqueira on behalf of the Fiocruz COVID-19 Genomic Surveillance Network; Paola Resende; Renata Serrano Lopes; Taina Venas
EPI_ISL_3460255, EPI_ISL_3460258, EPI_ISL_3461056, EPI_ISL_3461057, EPI_ISL_3461061, EPI_ISL_3461062, EPI_ISL_3461063, EPI_ISL_3461064, EPI_ISL_3461066, EPI_ISL_3461067, EPI_ISL_3461097, EPI_ISL_3461098, EPI_ISL_3461100, EPI_ISL_3461102, EPI_ISL_3461103, EPI_ISL_3461105, EPI_ISL_3461108, EPI_ISL_3492485, EPI_ISL_3492486, EPI_ISL_3492487, EPI_ISL_3492488, EPI_ISL_3492489, EPI_ISL_3492490, EPI_ISL_3492491, EPI_ISL_3492500, EPI_ISL_3492505, EPI_ISL_3492508, EPI_ISL_3492514, EPI_ISL_3756575, EPI_ISL_3806774			
see above	Laboratório de Microbiologia Molecular - Universidade FEEVALE	Molecular Microbiology Laboratory	Alana Witt Hansen; Fernando Rosado Spilki; Fágner Henrique Heldt; Juliana Schons Gulart; Juliana Schons Gularte; Juliane Deise Fleck; Mariana Soares da Silva; Matheus Nunes Weber; Meriane Demoliner; Michele Filippi.; Micheli Filippi; Paula Rodrigues de Almeida; Vyctoria Malayhka de Abreu Góes Pereira.
EPI_ISL_3529997	Lighthouse Lab in Glasgow	Wellcome Sanger Institute for the COVID-19 Genomics UK (COG-UK) Consortium	Anna Dominiczak and Alex Alderton; Carol Clugston; Cordelia Langford; David Gray; David K. Jackson; Dominic Kwiatkowski; Ewan Harrison; Harper VanSteenhouse; Ian Johnston; Jeffrey Barrett; John Sillitoe on behalf of the Wellcome Sanger Institute COVID-19 Surveillance Team; Roberto Amato; Sonia Goncalves; Yumi Kasai
EPI_ISL_3221445	Lighthouse Lab in Milton Keynes	Wellcome Sanger Institute for the COVID-19 Genomics UK (COG-UK) Consortium	Cordelia Langford; David K. Jackson; Dominic Kwiatkowski; Ewan Harrison; Ian Johnston; Jeffrey Barrett; John Sillitoe on behalf of the Wellcome Sanger Institute COVID-19 Surveillance Team; Roberto Amato; Sonia Goncalves; The Lighthouse Lab in Milton Keynes and Alex Alderton
EPI_ISL_3254285	Lincoln Diagnostics	New York City Public Health Laboratory	Jade Wang; et al.
EPI_ISL_3536368, EPI_ISL_3912236	MATERNIDADE ESCOLA ASSIS CHATEAUBRIAND	Analytical Competence Molecular Epidemiology Lab/ACME, Oswaldo Cruz Foundation, Ceara (FIOCRUZ CE)	Cleber Furtado Aksenen; Fabio Miyajima; Fernando Braga Stehling; Francisco Eder de Moura Lopes; Jamille Maria Mendes Bezerra; Joaquim Cesar do Nascimento Sousa Junior; Joaquim César do Nascimento Sousa Junior; Pedro Miguel Carneiro Jeronimo; Suzana Porto Almeida & Lucas Delerino on behalf of COVID-19 FIOCRUZ Genomic Network; Suzana Porto Almeida e Lucas Delerino; Thais Ferreira de Oliveira; Thais de Oliveira Costa; Ticiane Cavalcante de Souza; Veridiana Pessoa Miyajima
EPI_ISL_3491530, EPI_ISL_3491531	MD DOH Laboratories Administration	Centers for Disease Control and Prevention Division of Viral Diseases, Pathogen Discovery	Alex Burgin; Ben Rambo-Martin; Clinton Paden; Dakota Howard; Dave Wentworth; Dhwani Batra; Jasmine Padilla; Justin Lee; Krista Queen; Kristen Knipe; Kristine Lacek; Mark Burroughs; Matthew Schmerer; Meghan Bentz; Mili Sheth; Peter Cook; Sam Shepard; Sarah Nobles; Suxiang Tong; Vivien Dugan; Yvette Unoarumhi
EPI_ISL_3241905	MD PHL	MD PHL	Ami Patel; Eric N. Keller; Jillian Loomis; Kwang Low; Terence L. Moore; and Robert Myers
EPI_ISL_3152291, EPI_ISL_3235641	MD PHL	Maryland Department of Health Laboratories Administration	Ami Patel; Eric N. Keller; Jillian Loomis; Kwang Low; Terence L. Moore; and Robert Myers
EPI_ISL_3432935, EPI_ISL_3432936, EPI_ISL_3432937, EPI_ISL_3432938, EPI_ISL_3432939, EPI_ISL_3432940, EPI_ISL_3432941, EPI_ISL_3432942, EPI_ISL_3432943, EPI_ISL_3432944, EPI_ISL_3432945, EPI_ISL_3432946, EPI_ISL_3432947, EPI_ISL_3432948, EPI_ISL_3432949, EPI_ISL_3432950, EPI_ISL_3432951, EPI_ISL_3803222			
see above	MEPHI, Aix Marseille University	MEPHI, Aix Marseille University	Anthony LEVASSEUR
EPI_ISL_3494663	MVZ Labor Dr. Fenner und Kollegen (Standort Hamburg)	Robert Koch Institute	
EPI_ISL_3298934	Maine Health and Environmental Testing Laboratory	Tewhey Lab, The Jackson Laboratory	Barter, M.; Dewey, H.; H. and Tewhey, R.; Iosue, F.; Lynch, R.; Matluk, N.; Munger
EPI_ISL_3427182, EPI_ISL_3427279, EPI_ISL_3427338, EPI_ISL_3427340, EPI_ISL_3427581, EPI_ISL_3427603, EPI_ISL_3427658, EPI_ISL_3427913, EPI_ISL_3428594, EPI_ISL_3603040, EPI_ISL_3603806, EPI_ISL_3604040, EPI_ISL_3604274, EPI_ISL_3604495, EPI_ISL_3865103, EPI_ISL_3865218, EPI_ISL_3865230			
see above	Mako Medical	Centers for Disease Control and Prevention Division of Viral Diseases, Pathogen Discovery	Adrian Paskey; Benjamin Rambo-Martin; Christopher Gulvick; Clinton Paden; Clinton R. Paden; Dakota Howard; Darlene Wagner; Dhwani Batra; Duncan MacCannell; Jason Caravas; Kara Moser; Lauren Moon; Matthew Schmerer; Matthew Tugwell; Peter Cook; Peter W. Cook; Scott Sammons; Shatavia Morrison; Yvette Unoarumhi
EPI_ISL_3549791	Maryland Genomics, Institute for Genome Sciences, University of Maryland School of Medicine	Maryland Genomics, Institute for Genome Sciences, University of Maryland School of Medicine	Claire M; Fraser; Hazen; Holly; Humphrys; Ivette; Jacques; Jonathan; Kranthi; Lim; Lisa D; Luke J; Mike; Ott; Ravel; Roussey; Sadzewicz; Sandra; Santana-Cruz; Tallon; Tracy; Vavikolanu
EPI_ISL_3877468	Medizinisch-Diagnostisches Labor Kempten allgäulab	Robert Koch Institute	
EPI_ISL_3713461	Medizinische Laboratorien Düsseldorf	Robert Koch Institute	
EPI_ISL_3156056	Microbiology Department, Laboratori Clínic Metropolitana Nord. Hospital Universitari Germans Trias i Pujol	Can Ruti SARS-CoV-2 Sequencing Hub (HUGTiP/IrsiCaixa/IGTP)	Alba Sánchez; Alexia París; Anna Not; Antoni E Bordoy; Bonaventura Clotet; Cristina Casañ; David Panisello; Francesc Catala-Moll; Gemma Clara; Ignacio Blanco; Laia Soler; Lauro Sumoy; Marc Noguera-Julian; Maria Casadellà; Mariona Parera; Mercedes Guerrero; Montserrat Giménez; Pere-Joan Cardona; Pilar Armengol; Roger Paredes; Verónica Saludes; and Elisa Martró on behalf of the Can Ruti SARS-CoV-2 Sequencing Hub
EPI_ISL_3875815	Microbiology Department. Complexo Hospitalario Universitario de Vigo	Microbiology Department. Complexo Hospitalario Universitario de Vigo	Alfaya N; Alonso I; Alvarez M; Cabrera JJ; Carballo R; Cores O; Cortizo S; Davina C; Martinez L; Mediero G; Perez S; Potel C; Regueiro B; Rey S; Vasallo FJ; del-Campo V
EPI_ISL_3133570	Microvida	Microvida	Jaco J. Verweij; Joep J. J. M. Stohr; Suzan D. Pas
EPI_ISL_3256532, EPI_ISL_3256534, EPI_ISL_3256539, EPI_ISL_3256543, EPI_ISL_3256552, EPI_ISL_3256583, EPI_ISL_3256891, EPI_ISL_3257132, EPI_ISL_3257153, EPI_ISL_3306730, EPI_ISL_3315243, EPI_ISL_3315245, EPI_ISL_3315250, EPI_ISL_3315258, EPI_ISL_3315259, EPI_ISL_3315260, EPI_ISL_3315291, EPI_ISL_3315442, EPI_ISL_3315443, EPI_ISL_3315444, EPI_ISL_3315445, EPI_ISL_3315512, EPI_ISL_3315614, EPI_ISL_3315615, EPI_ISL_3315616, EPI_ISL_3315617, EPI_ISL_3315618, EPI_ISL_3315630, EPI_ISL_3315652, EPI_ISL_3315757, EPI_ISL_3315759, EPI_ISL_3315764, EPI_ISL_3315772, EPI_ISL_3315773, EPI_ISL_3315774, EPI_ISL_3402376, EPI_ISL_3402377, EPI_ISL_3402378, EPI_ISL_3402379, EPI_ISL_3402380, EPI_ISL_3402381, EPI_ISL_3402382, EPI_ISL_3402383, EPI_ISL_3402384, EPI_ISL_3402385, EPI_ISL_3402403, EPI_ISL_3402408, EPI_ISL_3402429, EPI_ISL_3402430, EPI_ISL_3402431, EPI_ISL_3402432, EPI_ISL_3402477, EPI_ISL_3402482, EPI_ISL_3402503, EPI_ISL_3402504, EPI_ISL_3402505, EPI_ISL_3402506, EPI_ISL_3403233, EPI_ISL_3403294, EPI_ISL_3405064, EPI_ISL_3405211, EPI_ISL_3405361, EPI_ISL_3405511, EPI_ISL_3405607, EPI_ISL_3405629, EPI_ISL_3405707, EPI_ISL_3405729, EPI_ISL_3405807, EPI_ISL_3405829, EPI_ISL_3405907, EPI_ISL_3405929, EPI_ISL_3406462, EPI_ISL_3406705, EPI_ISL_3406948, EPI_ISL_3412436, EPI_ISL_3412446, EPI_ISL_3412468, EPI_ISL_3412492, EPI_ISL_3412501, EPI_ISL_3412525, EPI_ISL_3414355, EPI_ISL_3414629, EPI_ISL_3414795, EPI_ISL_3414917, EPI_ISL_3415132, EPI_ISL_3415254, EPI_ISL_3415431, EPI_ISL_3415440, EPI_ISL_3415461, EPI_ISL_3415485, EPI_ISL_3415494, EPI_ISL_3415518, EPI_ISL_3533151, EPI_ISL_3533871, EPI_ISL_3533872, EPI_ISL_3534125			
see above	Ministry of Health Turkey	Ministry of Health Turkey	Fatma Bayrakdar; Gulay Korukluoglu; Gülay Korukluoğlu; Suleyman Yalcin; Süleyman Yalcin; Yasemin Cosgun; Yasemin Coşgun
EPI_ISL_2988441, EPI_ISL_3133154, EPI_ISL_3133159, EPI_ISL_3268226	Montana Public Health Laboratory	Montana Public Health Laboratory	Carrie Biskupiak; Deborah Gibson; Joy Ritter; Michael Dills; Michelle Mozer
EPI_ISL_3900727	NJDOH, Public Health and Environmental Laboratories	NJ PHEL	Allison Roder; Byeong Jeong; Chelsea San Filippo; Dana Woell; Jacquelyn Deverell; Lindsey Bodnar; Ryan Pachucki; Shiv K. Verma

EPI_ISL_3151733, EPI_ISL_3232172, EPI_ISL_3668028	NJDOH, Public Health and Environmental Laboratories	NJ_PHEL	Allison Roder; Byeong Jeong; Chelsea San Filippo; Dana Woell; Jacquelyn Deverell; Lindsey Bodnar; Ryan Pachucki; Shiv K. Verma
EPI_ISL_3704078, EPI_ISL_3704099, EPI_ISL_3704109, EPI_ISL_3704118, EPI_ISL_3704147, EPI_ISL_3704151, EPI_ISL_3704154, EPI_ISL_3704158, EPI_ISL_3704170, EPI_ISL_3704181, EPI_ISL_3704202, EPI_ISL_3704223, EPI_ISL_3704242, EPI_ISL_3704252, EPI_ISL_3704261, EPI_ISL_3704270, EPI_ISL_3704279, EPI_ISL_3704282, EPI_ISL_3704286, EPI_ISL_3704290, EPI_ISL_3704299, EPI_ISL_3704309, EPI_ISL_3704318, EPI_ISL_3704330, EPI_ISL_3704334, EPI_ISL_3704337, EPI_ISL_3704342, EPI_ISL_3704345, EPI_ISL_3704353, EPI_ISL_3704364, EPI_ISL_3704374, EPI_ISL_3704389, EPI_ISL_3704390, EPI_ISL_3704393, EPI_ISL_3704394, EPI_ISL_3704395, EPI_ISL_3704396, EPI_ISL_3704397, EPI_ISL_3704398, EPI_ISL_3704399, EPI_ISL_3704400, EPI_ISL_3704401, EPI_ISL_3704402, EPI_ISL_3704403, EPI_ISL_3704404, EPI_ISL_3704405, EPI_ISL_3704407, EPI_ISL_3704408, EPI_ISL_3704409, EPI_ISL_3704411, EPI_ISL_3704412, EPI_ISL_3704413, EPI_ISL_3704414, EPI_ISL_3704418, EPI_ISL_3704421, EPI_ISL_3704424, EPI_ISL_3704428, EPI_ISL_3704431, EPI_ISL_3704435, EPI_ISL_3704438, EPI_ISL_3704442, EPI_ISL_3704445, EPI_ISL_3704449, EPI_ISL_3704458, EPI_ISL_3835341, EPI_ISL_3835342, EPI_ISL_3835343, EPI_ISL_3835344, EPI_ISL_3835345, EPI_ISL_3835346, EPI_ISL_3835347, EPI_ISL_3835348, EPI_ISL_3835350			
see above	NUPIT/UFPE	WallauLab on behalf of Fiocruz COVID-19 Genomic Surveillance Network	Alexandre Freitas da Silva; Cassia Docena; Constância Flávia Junqueira Ayres; Filipe Zimmer Dezordi; Gabriel Luz Wallau; Gustavo Barbosa de Lima; Lais Ceschini Machado; Lilian Caroliny Amorim Silva; Maira Galdino da Rocha Pitta; Marcelo Henrique dos Santos Paiva; Matheus Filgueira Bezerra; Michelly Cristiny Pereira; Rômulo Pessoa e Silva; Sinval Pinto Brandão Filho
EPI_ISL_3102715, EPI_ISL_3102716	Nevada State Public Health Laboratory	Nevada State Public Health Laboratory	Andrew Gorzalski; Mark Pandori
EPI_ISL_3329246, EPI_ISL_3812700, EPI_ISL_3819196, EPI_ISL_3999557	New Mexico Department of Health Scientific Laboratory	New Mexico Department of Health Scientific Laboratory	Anastacia Griego-Fisher; D'eldra Malone; Ellie Johnson; Jennifer Benoit; Linda Salazar; Ratheesh Rajan; T. Mark Willmon
EPI_ISL_3185890	Northwestern Memorial Hospital	Northwestern University - Ozer Lab	Chad J. Achenbach; Chao Qi; Egon A. Ozer; Judd F. Hultquist; Lacy M. Simons; Lawrence J. Jennings; Michael G. Ison; Ramon Lorenzo-Redondo; Taylor J. Dean
EPI_ISL_3547380	OHSU-MM Lab	OHSU MM Lab	Yun Wu
EPI_ISL_3100170, EPI_ISL_3233062, EPI_ISL_3304459	OSPEDALE SAN SALVATORE - MEDICINA DI LABORATORIO	Istituto Zooprofilattico Sperimentale dell’Abruzzo e Molise “G. Caporale”	Ancora M; Calistri P; Cammà C; Caporale M; Curini V; Delli Compagni E; Di Domenico M; Di Lollo Valeria; Di Pasquale A; Lorusso A; Mangone I; Marcacci M; Puglia I; Rinaldi A; Savini G; Scialabba S
EPI_ISL_3538946, EPI_ISL_3657512, EPI_ISL_4020772	Ohio Department of Health Laboratory	Ohio Department of Health Laboratory	Allison Black; Brent Lee; Caitlin McDonnell; Eric Brandt; Erica Leasure; Glen McGillivary; Heather Blankenship; Holmes; Jade Mowery; Jennifer; Kelsey Florek; Keoni Omura; Kirtana Ramadugu; Quanta Brown; Stephanie Mccracken; Tyler Payne; and Tammy Bannerman
EPI_ISL_3088308	Omics Sciences Laboratory	Omics Sciences Laboratory	Darlyn Amaya; Derly Andrade Molina; Gabriel Morey León; Juan Carlos Fernández Cadena; Rubén Armas González
EPI_ISL_3067395, EPI_ISL_3568685, EPI_ISL_3592930, EPI_ISL_3760834, EPI_ISL_4004814	Oregon State Public Health Laboratory	Oregon State Public Health Laboratory	Eugene Yeboah; John Fontana and Shane Sevey; Laura Tsaknaridis; Rafia Razzaque; Vanda Makris
EPI_ISL_3013976	Osp. S.Pertini	National Institute for Infectious Diseases (INMI) L. Spallanzani I.R.C.C.S	A Di Caro; B Bartolini; CEM Gruber; E Giombini; F Messina; F Santini; G Bonfiglio; M Rueca; MR Capobianchi; O Butera
EPI_ISL_3219524, EPI_ISL_3219525, EPI_ISL_3219526, EPI_ISL_3219527, EPI_ISL_3219528, EPI_ISL_3219529, EPI_ISL_3219530, EPI_ISL_3219531, EPI_ISL_3219532, EPI_ISL_3219533, EPI_ISL_3392632, EPI_ISL_3392633, EPI_ISL_3392634, EPI_ISL_3392635, EPI_ISL_3392636, EPI_ISL_3392637, EPI_ISL_3392638, EPI_ISL_3392642, EPI_ISL_3801546, EPI_ISL_3804146, EPI_ISL_3804147, EPI_ISL_3804148, EPI_ISL_3804149, EPI_ISL_3804156, EPI_ISL_3804157, EPI_ISL_3804158			
see above	Outre mer	National Reference Center for Viruses of Respiratory Infections, Institut Pasteur, Paris	Angela Brisebarre; Camille Capel; Christophe Malabat; Corinne Maufrais; Dominique Rousset; Etienne Simon-Lorière; Frédéric Lemoine; Hub de Bioinformatique et Biostatistique; Hub de bioinformatique et Biostatistique; Louise Lefrançois; Marion Barbet; Maud Vanpeene; Méline Bizard; Sylvie Behillil; Sylvie Van der Werf; Vincent Enouf
EPI_ISL_3922300	PRONTO SOCORRO MUNICIPAL TAMBAU	Instituto Butantan	Antonio Jorge Martins; Claudia Renata dos Santos Barros; David Schlesinger; Debora Botequio Moretti; Dimas Tadeu Covas; Elaine Cristina Marqueze; Elaine Vieira Santos; Evandra Strazza Rodrigues; Heidge Fukumasu; Jayme Augusto de Souza-Neto; José Salvatore Leister Patané; Luiz Alcantara; Luiz Lehmann Coutinho; Maria Carolina Elias; Maurício Lacerda Nogueira; Rafael dos Santos Bezerra; Raul Machado Neto; Rejane Maria Tommasini Grotto; Ricardo Haddad; Sandra Coccuzzo Sampaio Vessoni; Simone Kashima; Svetoslav Nanev Slavov; Vincent Louis Viala
EPI_ISL_3010693, EPI_ISL_3010747, EPI_ISL_3188491, EPI_ISL_3331609, EPI_ISL_3394309, EPI_ISL_3569307, EPI_ISL_3778434			
see above	Pandemic Response Lab - NYC	Pandemic Response Lab, R&D	Alex Carpio; Cybill del Castillo; Dylan Law; Haiping Hao; Henry Lee; Isabel Fernandez Escapa; Jon Laurent; Melissa Hopkins; Michael Hammerling; Pradeep Bugga; Shinyoung Clair Kang; Sol Rey; William Ward
EPI_ISL_3707328	Pathology, UAB	Pathology, UAB	E.J.; Hendrickson; Leal; Lefkowitz; Moates, D.; R.C.; S.M.; Van Der Pol; W.J.
EPI_ISL_3451831	Pennsylvania Department of Health Bureau of Laboratories	Pennsylvania Department of Health Bureau of Laboratories	Dongxiang Xia
EPI_ISL_3474945, EPI_ISL_3476662, EPI_ISL_3478418	Plateau technique Biomer Metz Verriers	Department of Virology, Henri Mondor University Hospital, Assistance Publique Hôpitaux de Paris, Université Paris-Est Créteil, INSERM U955	Alexandre Soulier; Christophe Rodriguez; Elisabeth Trawinski; Guillaume Gricourt; Jean-Michel Pawlotsky; Melissa N'Debi; Slim Fourati; Vanessa Demontant
EPI_ISL_3943628	Pomona Valley Hospital Medical Center	Los Angeles County Public Health Laboratories	P. Hemarajata et al.
EPI_ISL_3303908, EPI_ISL_3303911, EPI_ISL_3303921, EPI_ISL_3303925, EPI_ISL_3395239, EPI_ISL_3395300, EPI_ISL_3395551, EPI_ISL_3395650, EPI_ISL_3395680, EPI_ISL_3395681, EPI_ISL_3395786, EPI_ISL_3396761, EPI_ISL_3396821, EPI_ISL_3431067, EPI_ISL_3431076, EPI_ISL_3456275, EPI_ISL_3456505, EPI_ISL_3456512, EPI_ISL_3460809, EPI_ISL_3460811, EPI_ISL_3460820, EPI_ISL_3460821, EPI_ISL_3460830, EPI_ISL_3460839, EPI_ISL_3460840, EPI_ISL_3823483, EPI_ISL_3823527, EPI_ISL_3823856, EPI_ISL_3824012			
see above	Quest Diagnostics Incorporated	Centers for Disease Control and Prevention Division of Viral Diseases, Pathogen Discovery	A. Gerasimova; A. Perez; Adrian Paskey; B. Anderson; Benjamin Rambo-Martin; Christopher Gulvick; Clinton Paden; Clinton R. Paden; Dakota Howard; Darlene Wagner; Dhwani Batra; Duncan MacCannell; F. Lacbawan; I. A. Shlyakhter; I. Shlyakhter; Jason Caravas; K. Livingston; K.E. Livingston; Kara Moser; L. Bernstein; L.E. Bernstein; M. Hua; Matthew Schmerer; P. Tanpaiboon; Peter Cook; Peter W. Cook; R. Kagan; R. M. Kagan; R. Owen; R. Rolando; R. V. Rolando; S. H. Rosenthal; S. Rosenthal; Sammons; Scott; Scott Sammons; Shatavia Morrison; Y. Liu; Yvette Unoarumhi
EPI_ISL_3315103	RM2/Osp. S.Pertini	National Institute for Infectious Diseases (INMI) L. Spallanzani I.R.C.C.S	A Di Caro; B Bartolini; CEM Gruber; E Giombini; F Messina; F Santini; G Bonfiglio; M Rueca; MR Capobianchi; O Butera
EPI_ISL_3446918	RM3/A.O. S.Camillo	National Institute for Infectious Diseases (INMI) L. Spallanzani I.R.C.C.S	A Di Caro; B Bartolini; CEM Gruber; E Giombini; F Messina; F Santini; G Bonfiglio; M Rueca; MR Capobianchi; O Butera
EPI_ISL_3080974, EPI_ISL_3179251, EPI_ISL_3291465, EPI_ISL_3291851, EPI_ISL_3573591, EPI_ISL_3574505, EPI_ISL_3959078, EPI_ISL_3959944			
see above	Respiratory Virus Unit, Microbiology Services Colindale, Public Health England	COVID-19 Genomics UK (COG-UK) Consortium	PHE Covid Sequencing Team
EPI_ISL_3398872, EPI_ISL_3720972	Rhode Island State Health Laboratory	Rhode Island State Health Laboratory	Ewa King; Kristin Carpenter-Azevedo; Richard C. Huard
EPI_ISL_3982687, EPI_ISL_3982721, EPI_ISL_3982725	Ribeirao Preto	Laboratorio de Biologia Molecular Hemocentro de Ribeirao Preto	Antonio Jorge Martins; Claudia Renata dos Santos Barros; David Schlesinger; Debora Botequio Moretti; Dimas Tadeu Covas; Elaine Cristina Marqueze; Elaine Vieira Santos; Evandra Strazza Rodrigues; Heidge Fukumasu; Jayme Augusto de Souza-Neto; José Salvatore Leister Patané; Luiz Alcantara; Luiz Lehmann Coutinho; Maria Carolina Elias; Maurício Lacerda Nogueira; Rafael dos Santos Bezerra; Raul Machado Neto; Rejane Maria Tommasini Grotto; Ricardo Haddad; Sandra Coccuzzo Sampaio Vessoni; Simone Kashima; Svetoslav Nanev Slavov; Vincent Louis Viala
EPI_ISL_3982690, EPI_ISL_3982707, EPI_ISL_3982714, EPI_ISL_3982720, EPI_ISL_3982724, EPI_ISL_3982727	Ribeirao Preto SP	Laboratorio de Biologia Molecular Hemocentro de Ribeirao Preto	Antonio Jorge Martins; Claudia Renata dos Santos Barros; David Schlesinger; Debora Botequio Moretti; Dimas Tadeu Covas; Elaine Cristina Marqueze; Elaine Vieira Santos; Evandra Strazza Rodrigues; Heidge Fukumasu; Jayme Augusto de Souza-Neto; José Salvatore Leister Patané; Luiz Alcantara; Luiz Lehmann Coutinho; Maria Carolina Elias; Maurício Lacerda Nogueira; Rafael dos Santos Bezerra; Raul Machado Neto; Rejane Maria Tommasini Grotto; Ricardo Haddad; Sandra Coccuzzo Sampaio Vessoni; Simone Kashima; Svetoslav Nanev Slavov; Vincent Louis Viala
EPI_ISL_3388720	Rush University Medical Center	RIPHL at Rush University Medical Center	Diane Springer; Edith Perez; Felix Araujo Perez; Joyce Houlihan; Kevin Kunstman; Laura Furtado; Marieta Hyde; Mary Hayden; Max Kolton; Stefan Green
EPI_ISL_3909190	SARS-CoV-2 Sequencing Castilla y Leon-Spain Consortium	SARS-CoV-2 Sequencing Castilla y Leon-Spain Consortium	Antonio Orduña-Domingo; Carlos Fuster Foz; Carmen Aldea-Mansilla; Carmen Gimeno Crespo; David Abad; Gabriel March Rosello; Gregoria Megías Lobón; Jose María Eiros Bouza; M. Isabel Fernandez-Natal; Marta Dominguez-Gil; Marta Hernandez; María Antonia García Castro; Mª Fe Brezmes-Valdivieso; Noelia Arenal Andrés; Silvia Rojo; Sonsoles Garcinuño Pérez
EPI_ISL_3076694, EPI_ISL_3543154, EPI_ISL_3983486	SARS-CoV-2 testing team, National Institute of Infectious Diseases	Pathogen Genomics Center, National Institute of Infectious Diseases	Hazuka Y Furihata; Hiromizu Takahashi; Kentaro Itokawa; Makoto Kuroda; Masanori Hashino; Masumichi Saito; Naomi Nojiri; Nozomu Hanaoka; Rina Tanaka; Tsuguto Fujimoto; Tsuyoshi Sekizuka
EPI_ISL_3579325	SECRETARIA MUNCIPAL DE SAUDE DE SANTA BARBARA D OESTE	Instituto Butantan	Antonio Jorge Martins; Claudia Renata dos Santos Barros; David Schlesinger; Debora Botequio Moretti; Dimas Tadeu Covas; Elaine Cristina Marqueze; Elaine Vieira Santos; Evandra Strazza Rodrigues; Heidge Fukumasu; Jayme Augusto de Souza-Neto; José Salvatore Leister Patané; Luiz Alcantara; Luiz Lehmann Coutinho; Maria Carolina Elias; Maurício Lacerda Nogueira; Rafael dos Santos Bezerra; Raul Machado Neto; Rejane Maria Tommasini Grotto; Ricardo Haddad; Sandra Coccuzzo Sampaio Vessoni; Simone Kashima; Svetoslav Nanev Slavov; Vincent Louis Viala
EPI_ISL_3912239	SECRETARIA MUNICIPAL	Analytical Competence Molecular	Cleber Furtado Aksenen; Fabio Miyajima; Fernando Braga Stehling; Francisco Eder de Moura Lopes; Jamille Maria Mendes Bezerra; Joaquim Cesar do Nascimento Sousa Junior; Pedro Miguel Carneiro Jeronimo; Suzana Porto Almeida & Lucas Delerino on behalf of COVID-19 FIOCRUZ Genomic Network; Thais Ferreira de

	DE SAUDE DE TIANGUA	Epidemiology Lab/ACME, Oswaldo Cruz Foundation, Ceara (FIOCRUZ CE)	Oliveira; Thais de Oliveira Costa; Ticiane Cavalcante de Souza; Veridiana Pessoa Miyajima
EPI_ISL_3391909	SELAS MEDILYS	Department of Virology, Henri Mondor University Hospital, Assistance Publique Hôpitaux de Paris, Université Paris-Est Créteil, INSERM U955	Alexandre Soulier; Christophe Rodriguez; Elisabeth Trawinski; Guillaume Gricourt; Jean-Michel Pawlotsky; Melissa N'Debi; Slim Fourati; Vanessa Demontant
EPI_ISL_3204579	SESARAM	Instituto Nacional de Saude (INSA)	Borges et al
EPI_ISL_3503634, EPI_ISL_3548258, EPI_ISL_3721736	Salud Digna	Instituto Nacional de Medicina Genomica	Abraham Campos-Romero; Cedro-Tanda A; Escobar-Arrazola MA; Herrera-Montalvo LA.; Hidalgo-Miranda A; Luna-Ruiz Marco; Mendoza-Vargas A; Moreno-Camacho José Luis; Munguia-Garza P; Ramirez-Vega O; Rangel-DeLeon D; Reyes-Grajeda JP; Rodriguez-Gallegos Jorge; Yair Alfaro-Mora
EPI_ISL_3912477, EPI_ISL_3912498, EPI_ISL_3912518	Salud Digna, A.C	Andersen lab at Scripps Research	Abraham Garcia Gil; Jose Luis Moreno Camacho; Marco Antonio Luna Ruiz-Esparza; Miguel A. Fernandez Rojas; SEARCH Alliance with Abraham Campos Romero
EPI_ISL_3912892, EPI_ISL_3912900, EPI_ISL_3912970, EPI_ISL_3912980	San Diego County Public Health Laboratory	Andersen lab at Scripps Research	SEARCH Alliance San Diego with Ashleigh Murphy and Brett Austin
EPI_ISL_3708859, EPI_ISL_3842031	San Diego County Public Health Laboratory	San Diego County Public Health Laboratory	Ashleigh Murphy Schafer; Brett Austin
EPI_ISL_3948575	Sanquin B.V.	Sanquin B.V.	Anton van Weert; Jalenka van Wijk; Karin van Leeuwen; Lianne Koets; Maaike Derlagen; Marco Koppelman; Nadia Keijzer
EPI_ISL_3219274, EPI_ISL_3333990, EPI_ISL_3334041	Santa Clara Valley Medical Center	Santa Clara County Public Health Laboratory	Santa Clara County Public Health Department
EPI_ISL_3261657, EPI_ISL_3757311	Scripps Medical Laboratory	Andersen lab at Scripps Research	Ellen Stefanski; Ian Mchardy; SEARCH Alliance San Diego with Michael Quigley
EPI_ISL_4083362, EPI_ISL_4083435, EPI_ISL_4083447, EPI_ISL_4083509	Servicio Virosis Respiratorias-Departamento Virología-INEI	Instituto Nacional Enfermedades Infecciosas C.G.Malbran	Avaro M.; Baumeister E.; Benedetti E.; Campos J.; Cisterna D.; Dattero ME; De Belder D.; Haim MS.; Lorenzo F.; Molina V.; Perandones C.; Poklepovich T.; Pontoriero A.; Russo M.; Sanchez Loria J.; Tuduri E.
EPI_ISL_3265888, EPI_ISL_3266010	Servicio de Microbiología Clínica (Complejo Hospitalario de Navarra, Pamplona)	Centro de Secuenciación NASERTIC	Ana Miqueleiz; Ana Navascués; Carmen Ezpeleta Baquedano
EPI_ISL_3261327, EPI_ISL_3373926, EPI_ISL_3373973, EPI_ISL_3544107, EPI_ISL_3912619, EPI_ISL_3912881	Sharp HealthCare Laboratory	Andersen lab at Scripps Research	Art Mendoza; Cathy Woerle; Jacquelyn Berumen; Liam McGinnis; Omid Bakhtar; SEARCH Alliance San Diego with Aaron Harding
EPI_ISL_3538746, EPI_ISL_3916315	Stanford Health Care	Stanford University School of Medicine, Clinical Virology Laboratory	Becky Jiang; Bernadette Troung; James Zehnder; Malaya K. Sahoo; Nathan Hammond; Selamawit Bihon; and Benjamin A. Pinsky
EPI_ISL_3925268, EPI_ISL_3925285	StarMed Healthcare	UNC Charlotte Environmental Monitoring Laboratory	Adam France; Cynthia Gibas; Erin Stiers; Jannatul Ferdous; Jessica Schlueter; Kevin Lambirth; Sam Kunkleman
EPI_ISL_3374243	State Hygienic Laboratory at the University of Iowa	State Hygienic Laboratory at the University of Iowa	Alankar Kampoowale; Anna Yakos; Davis Rieckenberg; Erik Twait; Jeff Benfer; Kris Eveland; Kristen Zanon; Mariah Knutson; Valerie Reeb; Wes Hottel
EPI_ISL_3189705	State Laboratories Division, Hawaii State Department of Health	State Laboratories Division, Hawaii State Department of Health	Ayana Garnet; Daniel Strange; Drew Kuwazaki; Edward Desmond; Pamela O'Brien; Razvan Sultana
EPI_ISL_3516374	Supera	Laboratório de Biologia Molecular - Hemocentro de Ribeirão Preto	Sao Paulo State Network for Pandemic Alert of Emerging SARS-CoV-2 variants
EPI_ISL_3157680, EPI_ISL_3157776, EPI_ISL_3157825	Swedish national genomic surveillance program of SARS-CoV-2	The Public Health Agency of Sweden	Alma Brolund; Maria Lind Karlberg; Maximilian Riess; Swedish national genomic surveillance program of SARS-CoV-2
EPI_ISL_3325819, EPI_ISL_3325849, EPI_ISL_3326334, EPI_ISL_3326700, EPI_ISL_3826189	TGen North	TGen North	Brett Van Tassel; Chris French; Darrin Lemmer; Dave Engelthaler; Hayley Yaglom; Heather Centner; Jolene Bowers
EPI_ISL_3385370, EPI_ISL_3455613	TXDSHS	TXDSHS	Anita Pokharel; Bonnie Oh; Chun Wang; Grace Kubin; Jenny Zhang; Karen Bobier; Lorraine Rodriguez; Maliha Rahman; Mayela Pedrueza; Myong Koag; Rachel Lee; Rashmi Tuladhar
EPI_ISL_3048178	Tampa General Hospital Esoteric Lab	Tampa General Hospital Esoteric Research & Development Lab	Amorce Lima; Deanna Becker; Dominic Uy; Elaine Vendrone; Jon Faughn; Jorge Mecias-Frias; Marci O'Driscoll; Suzane Silbert; Vicki Healer
EPI_ISL_3922303	UBS JARDIM FORTALEZA	Instituto Butantan	Antonio Jorge Martins; Claudia Renata dos Santos Barros; David Schlesinger; Debora Botequio Moretti; Dimas Tadeu Covas; Elaine Cristina Marqueze; Elaine Vieira Santos; Evandra Strazza Rodrigues; Heidge Fukumasu; Jayme Augusto de Souza-Neto; José Salvatore Leister Patané; Luiz Alcantara; Luiz Lehmann Coutinho; Maria Carolina Elias; Maurício Lacerda Nogueira; Rafael dos Santos Bezerra; Raul Machado Neto; Rejane Maria Tommasini Grotto; Ricardo Haddad; Sandra Coccuzzo Sampaio Vessoni; Simone Kashima; Svetoslav Nanev Slavov; Vincent Louis Viala
EPI_ISL_3922301, EPI_ISL_3922302	UBS SANTA LIDIA	Instituto Butantan	Antonio Jorge Martins; Claudia Renata dos Santos Barros; David Schlesinger; Debora Botequio Moretti; Dimas Tadeu Covas; Elaine Cristina Marqueze; Elaine Vieira Santos; Evandra Strazza Rodrigues; Heidge Fukumasu; Jayme Augusto de Souza-Neto; José Salvatore Leister Patané; Luiz Alcantara; Luiz Lehmann Coutinho; Maria Carolina Elias; Maurício Lacerda Nogueira; Rafael dos Santos Bezerra; Raul Machado Neto; Rejane Maria Tommasini Grotto; Ricardo Haddad; Sandra Coccuzzo Sampaio Vessoni; Simone Kashima; Svetoslav Nanev Slavov; Vincent Louis Viala
EPI_ISL_3370434	UC-Christus Clinical Hospital Laboratory	Laboratory of Molecular Virology, School of Medicine, Pontificia Universidad Catolica de Chile	Ana Maira Guzman; Andres E. Munoz-Marcos; Catalina Pardo-Roa; Eileen Serrano; Erick Salinas; Estefany Poblete; Francisco Melo; Jorge Levican; Leonardo I. Almonacid; Maria Jose Avendano; Maria Patricia Vega; Rafael A. Medina; Ricardo Enrique de la Barra; Tamara Garcia-Salum
EPI_ISL_3104703	UCC (Universidad Central del Caribe)	Grubaugh Lab - Yale School of Public Health	Alejandaro Vallejo Degaudenzi; Anderson Brito; Annie Watkins; Chaney Kalinich; Chantal Vogels; Elisa Contreras; Esperanza Mendoza; Isabel Ott; Jessica Rothman; Joseph Fauver; Kendall Billig; Mallery Breban; Mary Petrone; Nathan Grubaugh; Robert Paulino-Ramirez; Tara Alpert; Tobias Koch; Victor Virgilio Calderon
EPI_ISL_3370910	UCSC Genomics Institute	UCSC Genomics Institute	A. Marm Kilpatrick; Angie Hinrichs; Beth Shapiro; Bryan Thornlow; Ciara Wanket; David Haussler; Eric Beraut; Hugh Olsen; Ikenna Anigbogu; Isabel Bjork; Jakob McBroome; Jeremy Sanford; Joshua Kapp; Mark Akeson; Maximilian Haeussler; Michael Stone; Miten Jain; Molly Cassatt-Johnstone; Namrita Dhillon; Russell Corbett-Detig; Terren Chang; Yatish Turakhia
EPI_ISL_3912091	UNIDADE SENTINELA DE JUAZEIRO DO NORTE	Analytical Competence Molecular Epidemiology Lab/ACME, Oswaldo Cruz Foundation, Ceara (FIOCRUZ CE)	Cleber Furtado Aksenen; Fabio Miyajima; Fernando Braga Stehling; Francisco Eder de Moura Lopes; Jamille Maria Mendes Bezerra; Joaquim Cesar do Nascimento Sousa Junior; Pedro Miguel Carneiro Jeronimo; Suzana Porto Almeida & Lucas Delerino on behalf of COVID-19 FIOCRUZ Genomic Network; Thais Ferreira de Oliveira; Thais de Oliveira Costa; Ticiane Cavalcante de Souza; Veridiana Pessoa Miyajima
EPI_ISL_3922304	UPA SAO JOAO LAVRAS	Instituto Butantan	Antonio Jorge Martins; Claudia Renata dos Santos Barros; David Schlesinger; Debora Botequio Moretti; Dimas Tadeu Covas; Elaine Cristina Marqueze; Elaine Vieira Santos; Evandra Strazza Rodrigues; Heidge Fukumasu; Jayme Augusto de Souza-Neto; José Salvatore Leister Patané; Luiz Alcantara; Luiz Lehmann Coutinho; Maria Carolina Elias; Maurício Lacerda Nogueira; Rafael dos Santos Bezerra; Raul Machado Neto; Rejane Maria Tommasini Grotto; Ricardo Haddad; Sandra Coccuzzo Sampaio Vessoni; Simone Kashima; Svetoslav Nanev Slavov; Vincent Louis Viala
EPI_ISL_3939961	URMC LABS	URMC LABS	Andrew Cameron; Dwight Hardy; Ghinwa Dumyati; Kelly DeLary; Stepan Voljehn
EPI_ISL_3942473, EPI_ISL_4001693, EPI_ISL_4079165	US Air Force School of Aerospace Medicine	US Air Force School of Aerospace Medicine	Amanda Javorina; Anthony Fries; Carol Garrett; Clarise Starr; Cole Anderson; Deanna Muehleman; Elizabeth Macias; Fritz Castillo; Jennifer Black; Jennifer Meyer; Kelsey Lanter; Sarah Purves; William Gruner
EPI_ISL_3149580, EPI_ISL_3149665, EPI_ISL_3149692, EPI_ISL_3161179, EPI_ISL_3236592, EPI_ISL_3244868, EPI_ISL_3369571, EPI_ISL_3369588, EPI_ISL_3508018, EPI_ISL_3508031, EPI_ISL_3552266, EPI_ISL_3666624, EPI_ISL_3759154, EPI_ISL_3759507, EPI_ISL_3759541, EPI_ISL_3759553, EPI_ISL_3825874, EPI_ISL_3825976, EPI_ISL_3826062			
see above	UW Virology Lab	UW Virology Lab	Alexander Greninger; Hong Xie; Keith R Jerome; Maria Lukes; Meei-Li Huang; Nathan Breit; Patrick Mathias; Pavitra Roychoudhury; Ricardo Perez; Robert J. Livingston; Savanna S. Carmack; Sean Ellis; Shah Mohamed Bakhash; Tien V. Nguyen
EPI_ISL_3557255	Unidad de Investigación Biomédica de Zacatecas (UIBZ)	Centro de Investigación en Enfermedades Infecciosas (CIENI), Instituto Nacional de Enfermedades Respiratorias (INER)	Alejandra García-Gasca; Alejandra Hernández-Terán; Alejandro Sánchez-Flores; Alfredo Herrera-Estrella; Alicia Ocaña-Mondragón; Andreu Comas-García; Angel Gustavo Salas-Lais; Antonio Loza Román; Bernardo Martínez-Miguel; Blanca Taboada; Brenda Irasema Maldonado-Meza; Bruno Gómez-Gil; Carla Ivón Herrera-Najera; Carlos F. Arias; Celia Boukadida; Clara Esperanza Santacruz-Tinoco; Concepción Grajales-Muñiz; Consorcio Mexicano de Vigilancia Genómica (CoViGen-Mex). Authors (in alphabetical order): Julio Elias Alvarado-Yaah; Cristóbal Cháidez-Quiróz; Célida Duque Molina; Célida Martínez- Rodríguez; Daniel Fregoso-Rueda; Daniel Lira Morales; Eduardo Becerril-Vargas; Fernando Fontove-Herrera; Fidencio Mejía-Nepomuceno; Francisco Pulido; Gloria Elena Espinosa-Ayala; Gloria María Molina-Salinas; Gloria Vazquez; Hector Esteban Paz-Juárez; Hector Montoya-Fuentes; Helen Haydee Fernanda Ramírez-Plascencia; Irvin González-López; Jean Pierre González; Jesús Hernández; Joel Armando Vázquez-Pérez.; Jorge Salas-Hernández; José Antonio Enciso-Moreno; José Arturo Martínez-Orozco; José Esteban Muñoz-Medina; José de Jesús Nuñez-Contreras; Juan Bautista Chale-Dzul; Julissa Enciso-Ibarra; Kathia Elizabeth Tapia-Diaz; Luis Alberto Ochoa-Carrera; Margarita Matías-Florentino; Mario Mújica-Sánchez; Marissa Perez-Garcia; María Guadalupe Santiago-Mauricio; María Guadalupe de Jesús Mireles-Rivera; Nelly Sélem-Mojica; Pavel Isa; Ricardo Ciria Merce; Ricardo Grande; Rosa María Gutiérrez Rios; Santiago Ávila-Ríos; Selene Zárate; Susana Lopez; Verónica Mata-Haro; Victor Eduardo García-Arias; Víctor Hugo Borja-Aburto

EPI_ISL_4006331	Unidad de Investigación Médica de Yucatán (UIMY)	Instituto de Biotecnología de la UNAM	; Alejandra García-Gasca; Alejandra Hernández-Terán; Alejandro Sánchez-Flores; Alfredo Herrera-Estrella; Alicia Ocaña-Mondragón; Andreu Comas-García; Angel Gustavo Salas-Lais; Antonio Loza Román; Bernardo Martínez-Miguel; Blanca Taboada; Brenda Irasema Maldonado-Meza; Bruno Gómez-Gil; Carla Ivón Herrera-Najera; Carlos F. Arias; Celia Boukadida; Clara Esperanza Santacruz-Tinoco; Concepción Grajales-Muñiz; Consorcio Mexicano de Vigilancia Genómica (CoViGen-Mex). Authors (in alphabetical order): Julio Elias Alvarado-Yaah; Cristóbal Cháidez-Quiróz; Célida Duque Molina; Célida Martínez- Rodríguez; Daniel Fregoso-Rueda; Daniel Lira Morales; Eduardo Becerril-Vargas; Fernando Fontove-Herrera; Fidencio Mejía-Nepomuceno; Francisco Pulido; Gloria Elena Espinosa-Ayala; Gloria María Molina-Salinas; Gloria Vazquez; Hector Esteban Paz-Juárez; Hector Montoya-Fuentes; Helen Haydee Fernanda Ramírez-Plascencia; Irvin González-López; Jean Pierre González; Jesús Hernández; Joel Armando Vázquez-Pérez).; Jorge Salas-Hernández; José Antonio Enciso-Moreno; José Arturo Martínez-Orozco; José Esteban Muñoz-Medina; José de Jesús Nuñez-Contreras; Juan Bautista Chale-Dzul; Julissa Enciso-Ibarra; Kathia Elizabeth Tapia-Diaz; Luis Alberto Ochoa-Carrera; Margarita Matías-Florentino; Mario Mújica-Sánchez; Marissa Perez-Garcia; María Guadalupe Santiago-Mauricio; María Guadalupe de Jesús Mireles-Rivera; Nelly Sélem-Mojica; Pavel Isa; Ricardo Ciria Merce; Ricardo Grande; Rosa María Gutiérrez Rios; Santiago Ávila-Ríos; Selene Zárate; Susana Lopez; Verónica Mata-Haro; Victor Eduardo García-Arias; Víctor Hugo Borja-Aburto
EPI_ISL_3696701, EPI_ISL_3696711, EPI_ISL_3696758, EPI_ISL_3696767	Unidade Básica de Saúde – Dr. José Francisco de Almeida	Instituto Butantan	Antonio Jorge Martins; Claudia Renata dos Santos Barros; David Schlesinger; Debora Botequio Moretti; Dimas Tadeu Covas; Elaine Cristina Marqueze; Elaine Vieira Santos; Evandra Strazza Rodrigues; Heidge Fukumasu; Jayme Augusto de Souza-Neto; José Salvatore Leister Patané; Luiz Alcantara; Luiz Lehmann Coutinho; Maria Carolina Elias; Maurício Lacerda Nogueira; Rafael dos Santos Bezerra; Raul Machado Neto; Rejane Maria Tommasini Grotto; Ricardo Haddad; Sandra Coccuzzo Sampaio Vessoni; Simone Kashima; Svetoslav Nanev Slavov; Vincent Louis Viala
EPI_ISL_3696811	Unidade Mista de Saúde de Roseira	Instituto Butantan	Antonio Jorge Martins; Claudia Renata dos Santos Barros; David Schlesinger; Debora Botequio Moretti; Dimas Tadeu Covas; Elaine Cristina Marqueze; Elaine Vieira Santos; Evandra Strazza Rodrigues; Heidge Fukumasu; Jayme Augusto de Souza-Neto; José Salvatore Leister Patané; Luiz Alcantara; Luiz Lehmann Coutinho; Maria Carolina Elias; Maurício Lacerda Nogueira; Rafael dos Santos Bezerra; Raul Machado Neto; Rejane Maria Tommasini Grotto; Ricardo Haddad; Sandra Coccuzzo Sampaio Vessoni; Simone Kashima; Svetoslav Nanev Slavov; Vincent Louis Viala
EPI_ISL_3245351, EPI_ISL_3245353, EPI_ISL_3245361, EPI_ISL_3245377, EPI_ISL_3245382, EPI_ISL_3245388, EPI_ISL_3245391, EPI_ISL_3245393, EPI_ISL_3245402, EPI_ISL_3245403, EPI_ISL_3245404, EPI_ISL_3245405, EPI_ISL_3245407, EPI_ISL_3245413, EPI_ISL_3245414, EPI_ISL_3245415, EPI_ISL_3245418, EPI_ISL_3245420, EPI_ISL_3245421, EPI_ISL_3245423, EPI_ISL_3245425, EPI_ISL_3245437, EPI_ISL_3245439, EPI_ISL_3245449, EPI_ISL_3245452, EPI_ISL_3245453, EPI_ISL_3245456, EPI_ISL_3245459, EPI_ISL_3615203, EPI_ISL_3615204, EPI_ISL_3615205, EPI_ISL_3615206, EPI_ISL_3615207, EPI_ISL_3615208, EPI_ISL_3615210, EPI_ISL_3615211, EPI_ISL_3615212, EPI_ISL_3615213, EPI_ISL_3615214, EPI_ISL_3615215, EPI_ISL_3615217, EPI_ISL_3921467			
see above	Unidade de apoio ao diagnostico da COVID - UNADIG	Bioinformatics Laboratory / LNCC	Alessandra P Lamarca; Alexandra L Gerber; Amilcar Tanuri; Ana Paula de C Guimaraes; Ana Tereza R Vasconcelos; Andrea Cony Cavalcanti; Caio Luiz Pereira Ribeiro; Cintia Policarpo; Claudia Maria Braga de Mello; Cristiane Gomes da Silva; Douglas Terra Machado; Erica Ramos dos Santos Nascimento; Fernanda Leitao dos Santos; Flavio Dias da Silva; Gleidson da Silva de Oliveira; Leandro Magalhaes de Souza; Liliane Cavalcante; Luiz G P de Almeida; Marcio Henrique de Oliveira Garcia; Mario Sergio Ribeiro; Ricardo Jose Barbosa Salviano; Ronaldo da Silva F Jr; Silvia Carvalho
EPI_ISL_3230063	University Campus Bio-Medico of Rome (UCBM)	University Campus Bio-Medico of Rome (UCBM)	Angeletti S.; De Florio L.; Fogolari M.; Francesconi M.; Lintas C.; Riva E.; Veralli R.
EPI_ISL_3048434, EPI_ISL_3151472	University Hospitals of Geneva, Laboratory of Virology	HUG, Laboratory of Virology and the Health2030 Genome Center	Aline Mamin; Ana Rita Goncalves; Deborah Penet; Emmanouil Dermitzakis; Francisco Perez; Henri Pegeot; Ioannis Xenarios; Keith Harshman; Laurent Kaiser; Lorenzo Cerutti; Melyssa Elies; Samuel Cordey
EPI_ISL_3893656	University of Oregon COVID-19 MAP Laboratory	University of Oregon Genomics and Cell Characterizcation Core Facility (GC3F)	Ariana White; Demi Glidden; Douglas Turnbull; Jason Carriere; Jason Sydes; Jeff Bishop; Megan Criss; Peter Batzel
EPI_ISL_3231193, EPI_ISL_3231194, EPI_ISL_3231195	Università degli Studi di Perugia	Istituto Zooprofilattico Sperimentale dell'Abruzzo e Molise "G. Caporale"	Ancora M; Biagetti M; Calistri P; Camilloni B; Cammà C; Curini V; Delli Compagni E; Di Domenico M; Di Pasquale A; Giammarioli M; Lorusso A; Mangone I; Marcacci M; Mencacci A; Puglia I; Rinaldi A; Savini G; Scialabba S
EPI_ISL_3144684, EPI_ISL_3263282, EPI_ISL_3263628, EPI_ISL_3449971, EPI_ISL_3745395, EPI_ISL_3833303	Utah Public Health Laboratory	Utah Public Health Laboratory	Erin L. Young; Kelly F. Oakeson; Olinto Linares-Perdomo; Pooja Gupta
EPI_ISL_3710522	VA Palo Alto Health Care System	Genomics and Discovery, Respiratory Viruses Branch, Division of Viral Diseases, Centers for Disease Control and Prevention	Adam Retchless; Anna Kelleher; Anna Uehara; Brian Lynch; Clinton R. Paden; Dhwani Batra; Haibin Wang; Han Jia Justin Ng; Jasmine Padilla; Jing Zhang; Justin Lee; Mark Burroughs; Mili Sheth; Morgan Davis; Peter Cook; Rachel Marine; Sarah Nobles; Suxiang Tong; Tara Coalter; Yan Li; Ying Tao
EPI_ISL_3259937	VIDYMED LAUSANNE	Laboratory of genomics and metagenomics	Claire Bertelli; Damien Jacot; Gilbert Greub; Sébastien Aeby; Trestan Pillonel
EPI_ISL_3667098	Valais Hospital, Central Institute	Valais Hospital, Central Institute	Alexis Dumoulin; Deborah Penet; Emmanouil Dermitzakis; Henri Pegeot; Ioannis Xenarios; Keith Harshman; Lorenzo Cerutti; Melyssa Elies
EPI_ISL_3853848	Viollier AG	Department of Biosystems Science and Engineering, ETH Zürich	Andrea Patrizia Salzmann; Chaoran Chen; Christiane Beckmann; Christoph Noppen; Henriette Kurth; Ivan Topolsky; Kim Philipp Jablonski; Lara Fuhrmann; Louis du Plessis; Maurice Redondo; Niko Beerenwinkel; Olivier Kobel; Sarah Nadeau; Tanja Stadler
EPI_ISL_3048106, EPI_ISL_3117587, EPI_ISL_3318753, EPI_ISL_3402336, EPI_ISL_3667861, EPI_ISL_3667930, EPI_ISL_3693367, EPI_ISL_4000336			
see above	Virginia Division of Consolidated Laboratory Services	Virginia Division of Consolidated Laboratory Services	Virginia Division of Consolidated Laboratory Services
EPI_ISL_3217059, EPI_ISL_3451010, EPI_ISL_3451033, EPI_ISL_3656901, EPI_ISL_3921836, EPI_ISL_3921837, EPI_ISL_3921838, EPI_ISL_3921839, EPI_ISL_3921840, EPI_ISL_3921841, EPI_ISL_3921842, EPI_ISL_3921843			
see above	Washington State Department of Health Public Health Laboratories	Washington State Department of Health Public Health Laboratories	Avi Singh; Darren Lucas; Denny Russell; Drew MacKellar; Geoff Melly; Hannah Gray; Joenice Gonzalez; JohnAric Peterson; Philip Dykema; Rebecca Cao; Vanessa De Los Santos
EPI_ISL_3132091, EPI_ISL_3556239, EPI_ISL_4072757	Wisconsin State Laboratory of Hygiene Communicable Disease Division	Wisconsin State Laboratory of Hygiene Communicable Disease Division	Abigail C. Shockey; Alicia J. Mooney; Erika M. Hanson; Kelsey R. Florek; Richard Griesser; Sara Wagner; Tonya Danz
EPI_ISL_3236383, EPI_ISL_3841133, EPI_ISL_4006008	Yale Clinical Virology Lab	Grubaugh Lab - Yale School of Public Health	Anderson Brito; Annie Watkins; Chaney Kalinich; Chantal Vogels; Isabel Ott; Jessica Rothman; Joseph Fauver; Kendall Billig; Mallery Breban; Marie L. Landry; Mary Petrone; Nathan Grubaugh; Tara Alpert; Tobias Koch

We gratefully acknowledge the following Authors from the Originating laboratories responsible for obtaining the specimens, as well as the Submitting laboratories where the genome data were generated and shared via GISAID, on which this research is based.

All Submitters of data may be contacted directly via www.gisaid.org

Authors are sorted alphabetically.

Accession ID	Originating Laboratory	Submitting Laboratory	Authors
EPI_ISL_3128131, EPI_ISL_3128132, EPI_ISL_3128136	"AZ SPHL, Arizona Department of Health Services"	Centers for Disease Control and Prevention Division of Viral Diseases, Pathogen Discovery	Alex Burgin; Ben L. Rambo-Martin; Clinton R. Paden; Dakota Howard; Dave Wentworth; Dhwani Batra; Jasmine Padilla; Justin Lee; Krista Queen; Kristen Knipe; Kristine Lacek; Mark Burroughs; Matthew Schmerer; Meghan Bentz; Mili Sheth; Peter Cook; Sam Shepard; Sarah Nobles; Suxiang Tong; Vivien Dugan; Yvette Unoarumhi
EPI_ISL_2987104, EPI_ISL_2987111, EPI_ISL_2987114	"CDPH, Viral and Rickettsial Disease Laboratory"	Centers for Disease Control and Prevention Division of Viral Diseases, Pathogen Discovery	Alison Laufer Halpin; Ben L. Rambo-Martin; Clinton R. Paden; Dakota Howard; Darlene Wagner; Dave Wentworth; Dhwani Batra; Jasmine Padilla; Justin Lee; Katie Dillon; Krista Queen; Kristen Knipe; Kristine Lacek; Mark Burroughs; Matthew Schmerer; Mili Sheth; Peter Cook; Sam Shepard; Sarah Nobles; Shoshona Le; Suxiang Tong; Vivien Dugan; Yvette Unoarumhi
EPI_ISL_2787711	"HI Dept. of Health, State Laboratories Division"	Centers for Disease Control and Prevention Division of Viral Diseases, Pathogen Discovery	Alison Laufer Halpin; Ben L. Rambo-Martin; Clinton R. Paden; Dakota Howard; Darlene Wagner; Dave Wentworth; Dhwani Batra; Jasmine Padilla; Justin Lee; Katie Dillon; Krista Queen; Kristen Knipe; Kristine Lacek; Mark Burroughs; Matthew Schmerer; Mili Sheth; Peter Cook; Sam Shepard; Sarah Nobles; Shoshona Le; Suxiang Tong; Vivien Dugan; Yvette Unoarumhi
EPI_ISL_2690045, EPI_ISL_3355284	"NM Dept. Health, Scientific Laboratory Division "	Centers for Disease Control and Prevention Division of Viral Diseases, Pathogen Discovery	Alex Burgin; Alison Laufer Halpin; Ben L. Rambo-Martin; Ben Rambo-Martin; Clinton Paden; Clinton R. Paden; Dakota Howard; Darlene Wagner; Dave Wentworth; Dhwani Batra; Jasmine Padilla; Justin Lee; Katie Dillon; Krista Queen; Kristen Knipe; Kristine Lacek; Mark Burroughs; Matthew Schmerer; Meghan Bentz; Mili Sheth; Peter Cook; Sam Shepard; Sarah Nobles; Shoshona Le; Suxiang Tong; Vivien Dugan; Yvette Unoarumhi
EPI_ISL_3355430	"NYSDOH Wadsworth Center, Virology Lab"	Centers for Disease Control and Prevention Division of Viral Diseases, Pathogen Discovery	Alex Burgin; Ben Rambo-Martin; Clinton Paden; Dakota Howard; Dave Wentworth; Dhwani Batra; Jasmine Padilla; Justin Lee; Krista Queen; Kristen Knipe; Kristine Lacek; Mark Burroughs; Matthew Schmerer; Meghan Bentz; Mili Sheth; Peter Cook; Sam Shepard; Sarah Nobles; Suxiang Tong; Vivien Dugan; Yvette Unoarumhi
EPI_ISL_3064753, EPI_ISL_4060386	"OK Public Health Laboratory, Oklahoma State DOH"	Centers for Disease Control and Prevention Division of Viral Diseases, Pathogen Discovery	Alex Burgin; Ben L. Rambo-Martin; Ben Rambo-Martin; Clinton Paden; Clinton R. Paden; Dakota Howard; Dave Wentworth; Dhwani Batra; Jasmine Padilla; Justin Lee; Krista Queen; Kristen Knipe; Kristine Lacek; Mark Burroughs; Matthew Schmerer; Meghan Bentz; Mili Sheth; Peter Cook; Sam Shepard; Sarah Nobles; Suxiang Tong; Vivien Dugan; Yvette Unoarumhi
EPI_ISL_2494052, EPI_ISL_2494053, EPI_ISL_2494054, EPI_ISL_2494055, EPI_ISL_2494056, EPI_ISL_2494057	AFIP LESTE	Instituto Butantan	Antonio Jorge Martins; Claudia Renata dos Santos Barros; David Schlesinger; Debora Botequio Moretti; Dimas Tadeu Covas; Elaine Cristina Marqueze; Elaine Vieira Santos; Evandra Strazza Rodrigues; Heidge Fukumasu; Jayme Augusto de Souza-Neto; José Salvatore Leister Patané; Luiz Alcantara; Luiz Lehmann Coutinho; Maria Carolina Elias; Maurício Lacerda Nogueira; Rafael dos Santos Bezerra; Raul Machado Neto; Rejane Maria Tommasini Grotto; Ricardo Haddad; Sandra Coccuzzo Sampaio Vessoni; Simone Kashima; Svetoslav Nanev Slavov; Vincent Louis Viala
EPI_ISL_2493269, EPI_ISL_2494058, EPI_ISL_2494059, EPI_ISL_2494060, EPI_ISL_2494062, EPI_ISL_2494063, EPI_ISL_2494064, EPI_ISL_2494066			
see above	AFIP SUDESTE	Instituto Butantan	Antonio Jorge Martins; Claudia Renata dos Santos Barros; David Schlesinger; Debora Botequio Moretti; Dimas Tadeu Covas; Elaine Cristina Marqueze; Elaine Vieira Santos; Evandra Strazza Rodrigues; Heidge Fukumasu; Jayme Augusto de Souza-Neto; José Salvatore Leister Patané; Luiz Alcantara; Luiz Lehmann Coutinho; Maria Carolina Elias; Maurício Lacerda Nogueira; Rafael dos Santos Bezerra; Raul Machado Neto; Rejane Maria Tommasini Grotto; Ricardo Haddad; Sandra Coccuzzo Sampaio Vessoni; Simone Kashima; Svetoslav Nanev Slavov; Vincent Louis Viala
EPI_ISL_2493287, EPI_ISL_2494067, EPI_ISL_2494069, EPI_ISL_2494070, EPI_ISL_2494071, EPI_ISL_2494072, EPI_ISL_2494073, EPI_ISL_2494074, EPI_ISL_2494075, EPI_ISL_2494077, EPI_ISL_2494078, EPI_ISL_2494080, EPI_ISL_2494081, EPI_ISL_2494084, EPI_ISL_2494085, EPI_ISL_2494086, EPI_ISL_2494087, EPI_ISL_2494090, EPI_ISL_2494092, EPI_ISL_2494095, EPI_ISL_2494098, EPI_ISL_2494311, EPI_ISL_2494312, EPI_ISL_2494330			
see above	AFIP SUL	Instituto Butantan	Antonio Jorge Martins; Claudia Renata dos Santos Barros; David Schlesinger; Debora Botequio Moretti; Dimas Tadeu Covas; Elaine Cristina Marqueze; Elaine Vieira Santos; Evandra Strazza Rodrigues; Heidge Fukumasu; Jayme Augusto de Souza-Neto; José Salvatore Leister Patané; Luiz Alcantara; Luiz Lehmann Coutinho; Maria Carolina Elias; Maurício Lacerda Nogueira; Rafael dos Santos Bezerra; Raul Machado Neto; Rejane Maria Tommasini Grotto; Ricardo Haddad; Sandra Coccuzzo Sampaio Vessoni; Simone Kashima; Svetoslav Nanev Slavov; Vincent Louis Viala
EPI_ISL_3031195	ALBANY MEDICAL CENTER	Wadsworth Center, New York State Department of Health	Alexis Russell; Catharine Prussing; Daryl M. Lamson; Erasmus Schneider; Erica Lasek-Nesselquist; John Kelly; Jonathan Plitnick; Kirsten St. George; Matthew Shudt; Melissa A Leisner; Navjot Singh
EPI_ISL_3388726	ALVERNO PROFESSIONAL CLINICAL LABORATORIES LLC	RIPHL at Rush University Medical Center	Edith Perez; Felix Araujo Perez; Julia Baranski; Kevin Kunstman; Laura Furtado; Marieta Hyde; Max Kolton; Stefan Green
EPI_ISL_2462327, EPI_ISL_2494134, EPI_ISL_2494137, EPI_ISL_2494138, EPI_ISL_2494331	AMBULATORIO DE ESPECIALIDADE V E MOGI MIRIM	Instituto Butantan	Antonio Jorge Martins; Claudia Renata dos Santos Barros; David Schlesinger; Debora Botequio Moretti; Dimas Tadeu Covas; Elaine Cristina Marqueze; Elaine Vieira Santos; Evandra Strazza Rodrigues; Heidge Fukumasu; Jayme Augusto de Souza-Neto; José Salvatore Leister Patané; Luiz Alcantara; Luiz Lehmann Coutinho; Maria Carolina Elias; Maurício Lacerda Nogueira; Rafael dos Santos Bezerra; Raul Machado Neto; Rejane Maria Tommasini Grotto; Ricardo Haddad; Sandra Coccuzzo Sampaio Vessoni; Simone Kashima; Svetoslav Nanev Slavov; Vincent Louis Viala
EPI_ISL_2827997, EPI_ISL_2827998	AREA DE SALUD BAGACES	Inciensa, Instituto Costarricense de Investigación y Enseñanza en Nutrición y Salud	Adriana Godínez; Claudio Soto-Garita; Estela Cordero; Francisco Duarte; Hebleen Porras; Joselyn Prado & Karol Matamoros; José Luis Vargas; Mariela Gutiérrez; Melany Calderón
EPI_ISL_3037817	AREA DE SALUD BELEN-FLORES - CLINICA DR. JORGE VOLIO	Inciensa, Instituto Costarricense de Investigación y Enseñanza en Nutrición y Salud	Adriana Godínez; Caterina Guzmán; Claudio Soto-Garita; Estela Cordero; Francisco Duarte; Hebleen Porras; José Luis Vargas; Mariela Gutiérrez Joselyn Prado; Melany Calderón; Nazareth Ruiz & Sandra Mora Leitón
EPI_ISL_2827996, EPI_ISL_3298336	AREA DE SALUD CARTAGO	Inciensa, Instituto Costarricense de Investigación y Enseñanza en Nutrición y Salud	Adriana Godínez; Claudio Soto-Garita; Estela Cordero; Francisco Duarte; Hebleen Porras; Joselyn Prado & Monserrat Segura; José Luis Vargas; Mariela Gutiérrez; Melany Calderón
EPI_ISL_3037814	AREA DE SALUD CHACARITA - CLINICA DR. FRANCISCO QUINTANA	Inciensa, Instituto Costarricense de Investigación y Enseñanza en Nutrición y Salud	Adriana Godínez; Caterina Guzmán; Claudio Soto-Garita; Estela Cordero; Francisco Duarte; Hebleen Porras; José Luis Vargas; Mariela Gutiérrez Joselyn Prado; Melany Calderón; Nazareth Ruiz & María José Gómez
EPI_ISL_2827988, EPI_ISL_2827989, EPI_ISL_2827990	AREA DE SALUD CIUDAD QUESADA	Inciensa, Instituto Costarricense de Investigación y Enseñanza en Nutrición y Salud	Adriana Godínez; Claudio Soto-Garita; Estela Cordero; Francisco Duarte; Hebleen Porras; Joselyn Prado & Juan Carlos Villalobos; José Luis Vargas; Mariela Gutiérrez; Melany Calderón
EPI_ISL_3298363	AREA DE SALUD COBANO	Inciensa, Instituto Costarricense de Investigación y Enseñanza en Nutrición y Salud	Adriana Godínez; Claudio Soto-Garita; Estela Cordero; Francisco Duarte; Hebleen Porras; Joselyn Prado & María José Gómez; José Luis Vargas; Mariela Gutiérrez; Melany Calderón
EPI_ISL_3037821	AREA DE SALUD COTO BRUS	Inciensa, Instituto Costarricense de Investigación y Enseñanza en Nutrición y Salud	Adriana Godínez; Caterina Guzmán; Claudio Soto-Garita; Estela Cordero; Francisco Duarte; Hebleen Porras; José Luis Vargas; Mariela Gutiérrez Joselyn Prado; Melany Calderón; Nazareth Ruiz & María Fernanda Matamoros
EPI_ISL_2827999	AREA DE SALUD DESAMPARADOS 2 (COOPESALUD)	Inciensa, Instituto Costarricense de Investigación y Enseñanza en Nutrición y Salud	Adriana Godínez; Claudio Soto-Garita; Estela Cordero; Francisco Duarte; Hebleen Porras; Joselyn Prado & Alberto Quirós; José Luis Vargas; Mariela Gutiérrez; Melany Calderón
EPI_ISL_3298316	AREA DE SALUD EL GUARCO	Inciensa, Instituto Costarricense de Investigación y Enseñanza en Nutrición y Salud	Adriana Godínez; Claudio Soto-Garita; Estela Cordero; Francisco Duarte; Hebleen Porras; Joselyn Prado & Mónica Charpentier; José Luis Vargas; Mariela Gutiérrez; Melany Calderón
EPI_ISL_2828006	AREA DE SALUD ESCAZU (COOPESANA)	Inciensa, Instituto Costarricense de Investigación y Enseñanza en Nutrición y Salud	Adriana Godínez; Claudio Soto-Garita; Estela Cordero; Francisco Duarte; Hebleen Porras; Joselyn Prado & Juliana Mora; José Luis Vargas; Mariela Gutiérrez; Melany Calderón
EPI_ISL_2827985	AREA DE SALUD ESPARZA	Inciensa, Instituto Costarricense de Investigación y Enseñanza en Nutrición y Salud	Adriana Godínez; Claudio Soto-Garita; Estela Cordero; Francisco Duarte; Hebleen Porras; Joselyn Prado & María José Gómez; José Luis Vargas; Mariela Gutiérrez; Melany Calderón
EPI_ISL_2828002, EPI_ISL_3037801	AREA DE SALUD GOICOECHEA 2 - CLINICA DR. JIMENEZ NUÑEZ	Inciensa, Instituto Costarricense de Investigación y Enseñanza en Nutrición y Salud	& Nazareth Ruiz; Adriana Godínez; Caterina Guzmán; Claudio Soto-Garita; Estela Cordero; Francisco Duarte; Hebleen Porras; Joselyn Prado & Juan Carlos Cartes; José Luis Vargas; Mariela Gutiérrez; Mariela Gutiérrez Joselyn Prado; Melany Calderón
EPI_ISL_2827970	AREA DE SALUD GUATUSO	Inciensa, Instituto Costarricense de Investigación y Enseñanza en Nutrición y Salud	Adriana Godínez; Claudio Soto-Garita; Estela Cordero; Francisco Duarte; Hebleen Porras; Joselyn Prado & Francisco Chacón; José Luis Vargas; Mariela Gutiérrez; Melany Calderón
EPI_ISL_2827961	AREA DE SALUD HATILLO - CLINICA DR. SOLON NUÑEZ	Inciensa, Instituto Costarricense de Investigación y Enseñanza en Nutrición y Salud	Adriana Godínez; Claudio Soto-Garita; Estela Cordero; Francisco Duarte; Hebleen Porras; Joselyn Prado & Roberto Brilla; José Luis Vargas; Mariela Gutiérrez; Melany Calderón
EPI_ISL_3298313, EPI_ISL_3298314	AREA DE SALUD MATA REDONDA-HOSPITAL - CLINICA DR. MORENO CAÑAS	Inciensa, Instituto Costarricense de Investigación y Enseñanza en Nutrición y Salud	Adriana Godínez; Claudio Soto-Garita; Estela Cordero; Francisco Duarte; Hebleen Porras; Joselyn Prado & Ricardo González; José Luis Vargas; Mariela Gutiérrez; Melany Calderón
EPI_ISL_3037804	AREA DE SALUD OSA	Inciensa, Instituto Costarricense de	Adriana Godínez; Caterina Guzmán; Claudio Soto-Garita; Estela Cordero; Francisco Duarte; Hebleen Porras; José Luis Vargas; Mariela Gutiérrez Joselyn Prado; Melany Calderón; Nazareth Ruiz & Mariamilia Cob

		Investigación y Enseñanza en Nutrición y Salud	
EPI_ISL_3037822	AREA DE SALUD PARAISO-CERVANTES	Inciensa, Instituto Costarricense de Investigación y Enseñanza en Nutrición y Salud	Adriana Godínez; Caterina Guzmán; Claudio Soto-Garita; Estela Cordero; Francisco Duarte; Hebleen Porras; José Luis Vargas; Mariela Gutiérrez Joselyn Prado; Melany Calderón; Nazareth Ruiz & Mónica Charpentier
EPI_ISL_3037810	AREA DE SALUD SIQUIRRES	Inciensa, Instituto Costarricense de Investigación y Enseñanza en Nutrición y Salud	Adriana Godínez; Caterina Guzmán; Claudio Soto-Garita; Estela Cordero; Francisco Duarte; Hebleen Porras; José Luis Vargas; Mariela Gutiérrez Joselyn Prado; Melany Calderón; Nazareth Ruiz & Ileana Chavez Peraza
EPI_ISL_3298305	AREA DE SALUD ZAPOTE-CATEDRAL - CLINICA DR. CARLOS DURAN	Inciensa, Instituto Costarricense de Investigación y Enseñanza en Nutrición y Salud	Adriana Godínez; Claudio Soto-Garita; Estela Cordero; Francisco Duarte; Hebleen Porras; Joselyn Prado & Juliana Mora; José Luis Vargas; Mariela Gutiérrez; Melany Calderón
EPI_ISL_2695447	ARS Algarve - Laboratorio Laura Ayres	Instituto Nacional de Saude (INSA)	Borges et al
EPI_ISL_2638106, EPI_ISL_3028238	AULSS 2 Marca Trevigiana	Istituto Zooprofilattico Sperimentale delle Venezie	Adelaide Milani; Alessia Schivo; Alice Fusaro; Ambra Pastori; Annalisa Salviato; Antonia Ricci; Calogero Terregino; Edoardo Giussani; Elisa Palumbo; Erika Giorgia Quaranta; Isabella Monne; Luca Tassoni
EPI_ISL_2963463	AULSS 5 Polesana	Istituto Zooprofilattico Sperimentale delle Venezie	Adelaide Milani; Alessia Schivo; Alice Fusaro; Ambra Pastori; Annalisa Salviato; Antonia Ricci; Calogero Terregino; Edoardo Giussani; Elisa Palumbo; Erika Giorgia Quaranta; Isabella Monne; Luca Tassoni
EPI_ISL_3006782	AULSS 9 Scaligera	Istituto Zooprofilattico Sperimentale delle Venezie	Adelaide Milani; Alessia Schivo; Alice Fusaro; Ambra Pastori; Annalisa Salviato; Antonia Ricci; Calogero Terregino; Edoardo Giussani; Elisa Palumbo; Erika Giorgia Quaranta; Isabella Monne; Luca Tassoni
EPI_ISL_3039626	AULSS 9 Scaligera - Legnago	Istituto Zooprofilattico Sperimentale delle Venezie	Adelaide Milani; Alessia Schivo; Alice Fusaro; Ambra Pastori; Annalisa Salviato; Antonia Ricci; Calogero Terregino; Edoardo Giussani; Elisa Palumbo; Erika Giorgia Quaranta; Isabella Monne; Luca Tassoni
EPI_ISL_2790130	AZDelta	AZ Delta Medical Laboratories in Roeselare, Belgium	Dieter De Smet; Geert Martens; Merijn Vanhee; on behalf of AZ Delta COVID-19 Genomics core (member of Genomic surveillance of SARS-CoV-2 in Belgium network)
EPI_ISL_2370627, EPI_ISL_2370713, EPI_ISL_2422043, EPI_ISL_2422115, EPI_ISL_2422117, EPI_ISL_2442336, EPI_ISL_2489126, EPI_ISL_2489172, EPI_ISL_2489186, EPI_ISL_2489189, EPI_ISL_2489191, EPI_ISL_2489195, EPI_ISL_2489202, EPI_ISL_2489213, EPI_ISL_2489282, EPI_ISL_2489306, EPI_ISL_2489308, EPI_ISL_2489320, EPI_ISL_2489332, EPI_ISL_2489342, EPI_ISL_2489345, EPI_ISL_2489367, EPI_ISL_2489369, EPI_ISL_2489382, EPI_ISL_2489405, EPI_ISL_2489438, EPI_ISL_2489457, EPI_ISL_2489459, EPI_ISL_2489479, EPI_ISL_2489530, EPI_ISL_2489533, EPI_ISL_2489535, EPI_ISL_2489541, EPI_ISL_2489587, EPI_ISL_2489589, EPI_ISL_2489605, EPI_ISL_2489615, EPI_ISL_2489631, EPI_ISL_2489694, EPI_ISL_2489696, EPI_ISL_2489702, EPI_ISL_2489728, EPI_ISL_2489732, EPI_ISL_2489758, EPI_ISL_2489759, EPI_ISL_2489762, EPI_ISL_2489802, EPI_ISL_2527882, EPI_ISL_2527954, EPI_ISL_2527961, EPI_ISL_2527974, EPI_ISL_2528019, EPI_ISL_2528052, EPI_ISL_2528062, EPI_ISL_2528074, EPI_ISL_2528076, EPI_ISL_2528087, EPI_ISL_2528104, EPI_ISL_2528132, EPI_ISL_2528139, EPI_ISL_2528163, EPI_ISL_2528218, EPI_ISL_2528233, EPI_ISL_2528235, EPI_ISL_2528237, EPI_ISL_2528283, EPI_ISL_2528299, EPI_ISL_2528303, EPI_ISL_2528318, EPI_ISL_2528372, EPI_ISL_2528376, EPI_ISL_2528394, EPI_ISL_2528404, EPI_ISL_2528425, EPI_ISL_2528458, EPI_ISL_2528504, EPI_ISL_2528519, EPI_ISL_2528541, EPI_ISL_2528546, EPI_ISL_2528550, EPI_ISL_2528556, EPI_ISL_2528582, EPI_ISL_2528642, EPI_ISL_2528654, EPI_ISL_2528739, EPI_ISL_2528747, EPI_ISL_2528768, EPI_ISL_2528779, EPI_ISL_2528783, EPI_ISL_2528845, EPI_ISL_2528862, EPI_ISL_2528875, EPI_ISL_2528976, EPI_ISL_2528980, EPI_ISL_2528987, EPI_ISL_2529005, EPI_ISL_2530970, EPI_ISL_2531006, EPI_ISL_2531014, EPI_ISL_2531040, EPI_ISL_2531070, EPI_ISL_2534552, EPI_ISL_2686906, EPI_ISL_2686908, EPI_ISL_2686917, EPI_ISL_2686919, EPI_ISL_2686920, EPI_ISL_2686981, EPI_ISL_2686985, EPI_ISL_2686989, EPI_ISL_2686997, EPI_ISL_2687015, EPI_ISL_2687021, EPI_ISL_2687046, EPI_ISL_2687047, EPI_ISL_2687057, EPI_ISL_2687095, EPI_ISL_2687096, EPI_ISL_2687098, EPI_ISL_2687106, EPI_ISL_2687120, EPI_ISL_2687121, EPI_ISL_2687165, EPI_ISL_2687179, EPI_ISL_2687186, EPI_ISL_2687196, EPI_ISL_2687202, EPI_ISL_2687205, EPI_ISL_2687213, EPI_ISL_2687242, EPI_ISL_2687243, EPI_ISL_2687248, EPI_ISL_2687276, EPI_ISL_2687287, EPI_ISL_2687292, EPI_ISL_2687301, EPI_ISL_2687303, EPI_ISL_2687308, EPI_ISL_2687326, EPI_ISL_2687338, EPI_ISL_2687356, EPI_ISL_2687407, EPI_ISL_2687408, EPI_ISL_2687413, EPI_ISL_2687416, EPI_ISL_2687419, EPI_ISL_2687444, EPI_ISL_2687455, EPI_ISL_2687488, EPI_ISL_2687489, EPI_ISL_2687499, EPI_ISL_2687503, EPI_ISL_2687551, EPI_ISL_2687554, EPI_ISL_2687558, EPI_ISL_2687559, EPI_ISL_2709498, EPI_ISL_2709541, EPI_ISL_2709543, EPI_ISL_2784748, EPI_ISL_2784763, EPI_ISL_2784764, EPI_ISL_2784785, EPI_ISL_2784806, EPI_ISL_2784827, EPI_ISL_2784828, EPI_ISL_2784851, EPI_ISL_2784852, EPI_ISL_2784860, EPI_ISL_2784897, EPI_ISL_2784917, EPI_ISL_2784918, EPI_ISL_2784931, EPI_ISL_2784933, EPI_ISL_2784951, EPI_ISL_2784953, EPI_ISL_2784956, EPI_ISL_2784974, EPI_ISL_2784975, EPI_ISL_2784980, EPI_ISL_2784981, EPI_ISL_2784985, EPI_ISL_2784996, EPI_ISL_2784997, EPI_ISL_2785002, EPI_ISL_2785011, EPI_ISL_2785012, EPI_ISL_2785032, EPI_ISL_2785046, EPI_ISL_2785065, EPI_ISL_2785084, EPI_ISL_2785112, EPI_ISL_2785146, EPI_ISL_2785180, EPI_ISL_2785181, EPI_ISL_2785199, EPI_ISL_2785218, EPI_ISL_2785233, EPI_ISL_2785234, EPI_ISL_2785238, EPI_ISL_2785257, EPI_ISL_2785311, EPI_ISL_2785313, EPI_ISL_2785319, EPI_ISL_2785330, EPI_ISL_2785331, EPI_ISL_2785355, EPI_ISL_2785361, EPI_ISL_2785368, EPI_ISL_2785376, EPI_ISL_2785379, EPI_ISL_2785384, EPI_ISL_2785429, EPI_ISL_2785439, EPI_ISL_2785478, EPI_ISL_2785484, EPI_ISL_2785485, EPI_ISL_2785487, EPI_ISL_2785495, EPI_ISL_2785496, EPI_ISL_2832520, EPI_ISL_2832522, EPI_ISL_2832535, EPI_ISL_2875541, EPI_ISL_2875542, EPI_ISL_2875576, EPI_ISL_2875612, EPI_ISL_2875615, EPI_ISL_2875624, EPI_ISL_2875659, EPI_ISL_2875667, EPI_ISL_2875677, EPI_ISL_2875678, EPI_ISL_2875683, EPI_ISL_2875697, EPI_ISL_2875698, EPI_ISL_2875832, EPI_ISL_2875835, EPI_ISL_2875837, EPI_ISL_2875840, EPI_ISL_2875872, EPI_ISL_2875893, EPI_ISL_2875894, EPI_ISL_2877655, EPI_ISL_2877692, EPI_ISL_2877694, EPI_ISL_3217299, EPI_ISL_3217365, EPI_ISL_3217383, EPI_ISL_3217395, EPI_ISL_3217615, EPI_ISL_3217626, EPI_ISL_3217639, EPI_ISL_3217760, EPI_ISL_3217792, EPI_ISL_3217875, EPI_ISL_3218007, EPI_ISL_3218066, EPI_ISL_3218101, EPI_ISL_3218202, EPI_ISL_3218298, EPI_ISL_3218511, EPI_ISL_3218512, EPI_ISL_3218513, EPI_ISL_3218514, EPI_ISL_3218549, EPI_ISL_3218615, EPI_ISL_3218723, EPI_ISL_3220024, EPI_ISL_3220328, EPI_ISL_3220336, EPI_ISL_3220364, EPI_ISL_3220501, EPI_ISL_3220513, EPI_ISL_3220536, EPI_ISL_3220578, EPI_ISL_3220582, EPI_ISL_3220589, EPI_ISL_3220667, EPI_ISL_3220672, EPI_ISL_3220675, EPI_ISL_3220708, EPI_ISL_3220969, EPI_ISL_3220989, EPI_ISL_3221005, EPI_ISL_3221007, EPI_ISL_3221008, EPI_ISL_3221048, EPI_ISL_3305299, EPI_ISL_3305372, EPI_ISL_3305444, EPI_ISL_3305461, EPI_ISL_3321358, EPI_ISL_3321441, EPI_ISL_3321445, EPI_ISL_3321480, EPI_ISL_3321481, EPI_ISL_3323016			
see above	Aegis Sciences Corporation	Centers for Disease Control and Prevention Division of Viral Diseases, Pathogen Discovery	Adrian Paskey; Alec Vest; Benjamin Rambo-Martin; Christopher Gulvick; Clinton R. Paden; Cyndi Clark; Dakota Howard; Darlene Wagner; Dhwani Batra; Dillon Nall; Duncan MacCannell; Ethan Sanders; Holly Houdeshell; Jason Caravas; Kara Moser; Matthew Hardison; Matthew Schmerer; Ola Kvalvaag; Patrick Campbell; Peter W. Cook; Rob Case; Scott Sammons; Shatavia Morrison; Shaun Westlund; Vikramsinha Ghorpade; Yvette Unoarumhi
EPI_ISL_2445953	Affidea	Instituto Nacional de Saude (INSA)	Borges et al
EPI_ISL_2504004, EPI_ISL_2626722, EPI_ISL_2626723, EPI_ISL_2626727, EPI_ISL_2626735, EPI_ISL_2626737, EPI_ISL_2626738, EPI_ISL_2626743, EPI_ISL_2626745, EPI_ISL_2790766, EPI_ISL_2790772, EPI_ISL_2791464, EPI_ISL_2893332, EPI_ISL_2893333, EPI_ISL_2893334, EPI_ISL_2893375, EPI_ISL_2966419, EPI_ISL_2966439, EPI_ISL_2987162, EPI_ISL_3025339			
see above	Alaska State Virology Laboratory	Alaska State Virology Laboratory	Elva House; Jack Chen; Jacob Zidek; Lisa Smith; Ph.D.; Stephanie DeRonde
EPI_ISL_3388711, EPI_ISL_3388803	Alverno Labs	RIPHL at Rush University Medical Center	Edith Perez; Felix Araujo Perez; Kevin Kunstman; Laura Furtado; Marieta Hyde; Max Kolton; Melissa Mace; Stefan Green
EPI_ISL_2493286, EPI_ISL_2494039, EPI_ISL_2494040, EPI_ISL_2494041, EPI_ISL_2494042, EPI_ISL_2494043, EPI_ISL_2494044, EPI_ISL_2494045, EPI_ISL_2494046, EPI_ISL_2494047, EPI_ISL_2494048, EPI_ISL_2494049, EPI_ISL_2494050, EPI_ISL_2494051, EPI_ISL_2494329, EPI_ISL_2500985			
see above	Ambulatório IB	Instituto Butantan	Antonio Jorge Martins; Claudia Renata dos Santos Barros; David Schlesinger; Debora Botequio Moretti; Dimas Tadeu Covas; Elaine Cristina Marqueze; Elaine Vieira Santos; Evandra Strazza Rodrigues; Heidge Fukumasu; Jayme Augusto de Souza-Neto; José Salvatore Leister Patané; Luiz Alcantara; Luiz Lehmann Coutinho; Maria Carolina Elias; Maurício Lacerda Nogueira; Rafael dos Santos Bezerra; Raul Machado Neto; Rejane Maria Tommasini Grotto; Ricardo Haddad; Sandra Coccuzzo Sampaio Vessoni; Simone Kashima; Svetoslav Nanev Slavov; Vincent Louis Viala
EPI_ISL_2650135, EPI_ISL_2650136, EPI_ISL_2650137, EPI_ISL_2650141, EPI_ISL_2650142, EPI_ISL_2650148, EPI_ISL_2650151, EPI_ISL_2716405, EPI_ISL_2716406, EPI_ISL_2716407, EPI_ISL_2716411, EPI_ISL_2754301, EPI_ISL_2754305, EPI_ISL_2854198, EPI_ISL_2854199, EPI_ISL_2854200, EPI_ISL_2854201, EPI_ISL_2919071, EPI_ISL_2919072, EPI_ISL_2919073, EPI_ISL_2919076, EPI_ISL_2919078, EPI_ISL_2919079, EPI_ISL_2919081, EPI_ISL_2919082, EPI_ISL_2919083, EPI_ISL_2919084, EPI_ISL_2919093, EPI_ISL_2919097, EPI_ISL_2919101, EPI_ISL_2943052, EPI_ISL_2943060, EPI_ISL_2981919, EPI_ISL_3089183, EPI_ISL_3090069, EPI_ISL_3217104			
see above	Arizona State Public Health Laboratory	Arizona State Public Health Laboratory	Jessica Escobar; Katherine Fullerton; Linda Getsinger; Matthew Contursi; Nobuko Fukushima; Stacy White; Trung Huynh; Victor Waddell
EPI_ISL_2484113, EPI_ISL_2690351, EPI_ISL_2690380, EPI_ISL_2690385, EPI_ISL_2839100, EPI_ISL_2839101, EPI_ISL_2839102, EPI_ISL_2839103, EPI_ISL_2839104, EPI_ISL_2839105, EPI_ISL_2839106, EPI_ISL_2839107, EPI_ISL_2839108, EPI_ISL_2863926			
see above	Arizona State University	Arizona State University	Efrem S. Lim; Joshua LaBaer; Joy M. Blain; LaRinda A. Holland; Matthew F. Smith; Nicholas J. Mellor; Peter T. Skidmore; Rabia Maqsood; Valerie Harris; Vel Murugan
EPI_ISL_3504828, EPI_ISL_3553424, EPI_ISL_3553485	Arkansas Children's Hospital	Center for Global Health, University of New Mexico Health Sciences Center	Ashton Ingold; Bobby Boyanton; Darrell Dinwiddie; Daryl Domman; Joshua L. Kennedy; Kurt Schwalm; Valerie Morley
EPI_ISL_2715073, EPI_ISL_2715182, EPI_ISL_3015772, EPI_ISL_3015774	Atlas Genomics	Seattle Flu Study	Amanda Adler; Barry R. Lutz; Benjamin Pelle; Brian Hiatt; Caitlin R. Wolf; Chris D. Frazar; Deborah A. Nickerson; Elisabeth Brandstetter; Erica Ryke; Geoff Melly; Helen Y. Chu; Janet A. Englund; Jay Shendure; Jover Lee; Kairsten Fay; Kirsten Lacombe; Lea M. Starita; Mark J. Rieder; Matthew Richardson; Matthew Thompson; Melissa Truong; Michael Boeckh; Michael Famulare; Misja Ilcisin; Peter D. Han; Philip Dykema; Romesh Gautom; Scott Lindquist; Thomas R. Sibley; Trevor Bedford
EPI_ISL_2758010, EPI_ISL_2758012	Austrian Agency for Health and Food Safety (AGES)	Bergthaler laboratory, CeMM Research Center for Molecular Medicine of the Austrian Academy of Sciences	Andreas Bergthaler; Anna Schedl; Bekir Erguner; Benedikt Agerer; Christoph Bock; Fabian Amman; Jan Laine; Lukas Endler; Maelle Le Moing; Martin Senekowitsch; Matthew Thornton; Michael Schuster; Petr Triska; Thomas Penz
EPI_ISL_2493261, EPI_ISL_2493262, EPI_ISL_2494220, EPI_ISL_2494222, EPI_ISL_2494223	BIOFAST CENTRO	Instituto Butantan	Antonio Jorge Martins; Claudia Renata dos Santos Barros; David Schlesinger; Debora Botequio Moretti; Dimas Tadeu Covas; Elaine Cristina Marqueze; Elaine Vieira Santos; Evandra Strazza Rodrigues; Heidge Fukumasu; Jayme Augusto de Souza-Neto; José Salvatore Leister Patané; Luiz Alcantara; Luiz Lehmann Coutinho; Maria Carolina Elias; Maurício Lacerda Nogueira; Rafael dos Santos Bezerra; Raul Machado Neto; Rejane Maria Tommasini Grotto; Ricardo Haddad; Sandra Coccuzzo Sampaio Vessoni; Simone Kashima; Svetoslav Nanev Slavov; Vincent Louis Viala
EPI_ISL_2493248, EPI_ISL_2494227, EPI_ISL_2494228, EPI_ISL_2494229, EPI_ISL_2494231, EPI_ISL_2494232, EPI_ISL_2494233, EPI_ISL_2494234, EPI_ISL_2494235, EPI_ISL_2494236, EPI_ISL_2494237, EPI_ISL_2494238, EPI_ISL_2494239, EPI_ISL_2494240, EPI_ISL_2494241, EPI_ISL_2494242, EPI_ISL_2494243, EPI_ISL_2494244, EPI_ISL_2494246, EPI_ISL_2494248, EPI_ISL_2494249, EPI_ISL_2494250, EPI_ISL_2494251, EPI_ISL_2494253, EPI_ISL_2494255, EPI_ISL_2494256, EPI_ISL_2494257, EPI_ISL_2494258, EPI_ISL_2494259, EPI_ISL_2494261, EPI_ISL_2494262, EPI_ISL_2494263, EPI_ISL_2494264, EPI_ISL_2494265, EPI_ISL_2494301			
see above	BIOFAST LESTE	Instituto Butantan	Antonio Jorge Martins; Claudia Renata dos Santos Barros; David Schlesinger; Debora Botequio Moretti; Dimas Tadeu Covas; Elaine Cristina Marqueze; Elaine Vieira Santos; Evandra Strazza Rodrigues; Heidge Fukumasu; Jayme Augusto de Souza-Neto; José Salvatore Leister Patané; Luiz Alcantara; Luiz Lehmann Coutinho; Maria Carolina Elias; Maurício Lacerda Nogueira; Rafael dos Santos Bezerra; Raul Machado Neto; Rejane Maria Tommasini Grotto; Ricardo Haddad; Sandra Coccuzzo Sampaio Vessoni; Simone Kashima; Svetoslav Nanev Slavov; Vincent Louis Viala
EPI_ISL_2493270, EPI_ISL_2494214, EPI_ISL_2494215, EPI_ISL_2494216, EPI_ISL_2494217, EPI_ISL_2494333	BIOFAST NORTE	Instituto Butantan	Antonio Jorge Martins; Claudia Renata dos Santos Barros; David Schlesinger; Debora Botequio Moretti; Dimas Tadeu Covas; Elaine Cristina Marqueze; Elaine Vieira Santos; Evandra Strazza Rodrigues; Heidge Fukumasu; Jayme Augusto de Souza-Neto; José Salvatore Leister Patané; Luiz Alcantara; Luiz Lehmann Coutinho; Maria Carolina Elias; Maurício Lacerda Nogueira; Rafael dos Santos Bezerra; Raul Machado Neto; Rejane Maria Tommasini Grotto; Ricardo Haddad; Sandra Coccuzzo Sampaio Vessoni; Simone Kashima; Svetoslav Nanev Slavov; Vincent Louis Viala
EPI_ISL_2493260, EPI_ISL_2493267, EPI_ISL_2494201, EPI_ISL_2494202, EPI_ISL_2494203, EPI_ISL_2494204, EPI_ISL_2494205, EPI_ISL_2494206, EPI_ISL_2494207, EPI_ISL_2494209, EPI_ISL_2494211, EPI_ISL_2494212, EPI_ISL_2494313			
see above	BIOFAST SUL	Instituto Butantan	Antonio Jorge Martins; Claudia Renata dos Santos Barros; David Schlesinger; Debora Botequio Moretti; Dimas Tadeu Covas; Elaine Cristina Marqueze; Elaine Vieira Santos; Evandra Strazza Rodrigues; Heidge Fukumasu; Jayme Augusto de Souza-Neto; José Salvatore Leister Patané; Luiz Alcantara; Luiz Lehmann Coutinho; Maria Carolina Elias; Maurício Lacerda Nogueira; Rafael dos Santos Bezerra; Raul Machado Neto; Rejane Maria Tommasini Grotto; Ricardo Haddad; Sandra Coccuzzo Sampaio Vessoni; Simone Kashima; Svetoslav Nanev Slavov; Vincent Louis Viala
EPI_ISL_2933352, EPI_ISL_2933354, EPI_ISL_2933355, EPI_ISL_2933356, EPI_ISL_2933357, EPI_ISL_2933359, EPI_ISL_2933360, EPI_ISL_2933361, EPI_ISL_2933362, EPI_ISL_2933363, EPI_ISL_2933364, EPI_ISL_2933365, EPI_ISL_2933366, EPI_ISL_2933367, EPI_ISL_2933368, EPI_ISL_2933369, EPI_ISL_2933370, EPI_ISL_2933371, EPI_ISL_2933372, EPI_ISL_2933373, EPI_ISL_2933374, EPI_ISL_2933375, EPI_ISL_2933376, EPI_ISL_2933378, EPI_ISL_2933379, EPI_ISL_2933381, EPI_ISL_2933382, EPI_ISL_2933383, EPI_ISL_2933384, EPI_ISL_2933385, EPI_ISL_2933386, EPI_ISL_2933387, EPI_ISL_2933388, EPI_ISL_2933389, EPI_ISL_2933390, EPI_ISL_2933391, EPI_ISL_2933392, EPI_ISL_2933393, EPI_ISL_2933395, EPI_ISL_2933396, EPI_ISL_2933397, EPI_ISL_2933398, EPI_ISL_2933399, EPI_ISL_2933400, EPI_ISL_2933401, EPI_ISL_2933402, EPI_ISL_2933403, EPI_ISL_2933405, EPI_ISL_2933406, EPI_ISL_2933407, EPI_ISL_2933408, EPI_ISL_2933410, EPI_ISL_2933411, EPI_ISL_2933413, EPI_ISL_2933414, EPI_ISL_2933415, EPI_ISL_2933416, EPI_ISL_2933417, EPI_ISL_2933418, EPI_ISL_2933419, EPI_ISL_2933420, EPI_ISL_2933421, EPI_ISL_2933422, EPI_ISL_2933423, EPI_ISL_2933424, EPI_ISL_2933425, EPI_ISL_2933426, EPI_ISL_2933427, EPI_ISL_2933428, EPI_ISL_2933429, EPI_ISL_2933430, EPI_ISL_2933431, EPI_ISL_2933432, EPI_ISL_2933433, EPI_ISL_2933434, EPI_ISL_2933435, EPI_ISL_2933436, EPI_ISL_2933437, EPI_ISL_2933438, EPI_ISL_2933439, EPI_ISL_2933440, EPI_ISL_2933441, EPI_ISL_2933442, EPI_ISL_3130100			
see above	BIOMNIS LYON	CNR Virus des Infections Respiratoires - France SUD	Antonin Bal; Bruno Lina; Gregory Destras; Gwendolyne Burfin; Hadrien Regue; Laurence Josset; Martine Valette; Quentin Semanas
EPI_ISL_3504577, EPI_ISL_3504653	Baptist Health Medical Center	Center for Global Health, University of New Mexico Health Sciences Center	Amanda Novack; Darrell Dinwiddie; Daryl Domman; Dirk Haselow; Joshua L. Kennedy; Kurt Schwalm; Valerie Morley
EPI_ISL_2658950	Basurto University Hospital: Clinical Microbiology Laboratory	Biocruces Bizkaia	Ana de la Hoz; Estibaliz Ugalde Zarraga; José Luis Díaz de Tuesta del Arco; Mikel Gallego Rodrigo; Mikel Urrutikoetxea-Gutiérrez; Mª Carmen Nieto Toboso
EPI_ISL_2626816,	Baylor Scott & White-Temple	Baylor Scott & White-Temple	Ari Rao; Kimberly Walker; Linden Morales; Marcus Volz; Shelby Johnson

EPI_ISL_2626831, EPI_ISL_2774303, EPI_ISL_2834162, EPI_ISL_2928247, EPI_ISL_2928257			
EPI_ISL_2758372, EPI_ISL_2758373	Berkeley Medical Center	WVU and Marshall University Combined Genomics Core Facilities	James Denvir; Peter Perrotta; Peter Stoilov; Ryan Percifield; Wesley Kimble
EPI_ISL_2464480	Biogroup Bio Lam-LCD Saint-Denis	Department of Virology, Henri Mondor University Hospital, Assistance Publique Hôpitaux de Paris, Université Paris-Est Créteil, INSERM U955	Alexandre Soulier; Christophe Rodriguez; Elisabeth Trawinski; Guillaume Gricourt; Jean-Michel Pawlotsky; Melissa N'Debi; Slim Fourati; Vanessa Demontant
EPI_ISL_3145907, EPI_ISL_3145908	BioneXt Lab	Laboratoire national de sante, Microbiology, Microbial Genomics Platform	Anke Wienecke-Baldacchino; Catherine Ragimbeau; Elodie Solarino; Fatu Djabi; Jessica Tapp; Lise Pignon; Raoul Salmon; Tamir Abdelrahman; Thibault Ferrandon; Virginie Jover
EPI_ISL_2762051	Bioscientia Labor Wermsdorf	Robert Koch Institute	
EPI_ISL_2633914	Bioscientia MVZ Labor Karlsruhe GmbH	Robert Koch Institute	
EPI_ISL_3601715, EPI_ISL_3602355, EPI_ISL_3602558, EPI_ISL_3602851	British Columbia Centre For Disease Control	BCCDC Public Health Laboratory	Ana Pacagnella; Corrinne Ng; Dan Fornika; John Tyson; Kim Macdonald; Kimia Kamelian; Linda Hoang; Loretta Janz; Mel Krajden; Prystajecky Natalie; Robert Azana; Shannon Russell
EPI_ISL_2991515, EPI_ISL_2991536, EPI_ISL_2991562, EPI_ISL_2991713, EPI_ISL_2991774, EPI_ISL_2991856, EPI_ISL_2991908			
see above	Broad Institute Clinical Research Sequencing Platform	Infectious Disease Program, Broad Institute of Harvard and MIT	A.E.; Adams, G.; Anahtar, M.; B.L.; B.W.; Bauer, M.; Birren; Branda, J.; Carter, A.; Cerrato, F.; Chaluvadi, S.; Chapman; Cusick, C.; D.J.; DeRuff, K.; Flowers, K.; Gallagher, G.; Gladden-Young, A.; Gnirke, A.; Harris, J.; J.E.; K.J.; LaRocque, R.; Lagerborg, K.; Lemieux; Lin; Loreth, C.; MacInnis; Neumann, A.; Normandin, E.; P.C.; Park; Pierce, V.; Reilly, S.; Rosenberg, E.; Rudy, M.; Ryan, E.; S.B.; Sabeti; Shaw, B.; Siddle; Slater, D.; Smole, S.; Tomkins-Tinch, C.; Turbett, S.
EPI_ISL_2787323	CA-Los Angeles County Public Health Laboratory	Centers for Disease Control and Prevention Division of Viral Diseases, Pathogen Discovery	Alison Laufer Halpin; Ben L. Rambo-Martin; Clinton R. Paden; Dakota Howard; Darlene Wagner; Dave Wentworth; Dhwani Batra; Jasmine Padilla; Justin Lee; Katie Dillon; Krista Queen; Kristen Knipe; Kristine Lacek; Mark Burroughs; Matthew Schmerer; Mili Sheth; Peter Cook; Sam Shepard; Sarah Nobles; Shoshona Le; Suxiang Tong; Vivien Dugan; Yvette Unoarumhi
EPI_ISL_2891968, EPI_ISL_2892078, EPI_ISL_2892202, EPI_ISL_2892208, EPI_ISL_2892277, EPI_ISL_2892318, EPI_ISL_2922118, EPI_ISL_2923361, EPI_ISL_2923388, EPI_ISL_2923436, EPI_ISL_2923562, EPI_ISL_2923659, EPI_ISL_2923663, EPI_ISL_2923741, EPI_ISL_2923742, EPI_ISL_3244455, EPI_ISL_3244465, EPI_ISL_3244470, EPI_ISL_3244476, EPI_ISL_3244477, EPI_ISL_3244492, EPI_ISL_3244501, EPI_ISL_3244519, EPI_ISL_3669509, EPI_ISL_3669565, EPI_ISL_3669568, EPI_ISL_3669569, EPI_ISL_3669570, EPI_ISL_3669574, EPI_ISL_3669575, EPI_ISL_3669578, EPI_ISL_3669579, EPI_ISL_3669580, EPI_ISL_3669582, EPI_ISL_3669583, EPI_ISL_3669588, EPI_ISL_3669589, EPI_ISL_3669590, EPI_ISL_3669591, EPI_ISL_3669592, EPI_ISL_3669594, EPI_ISL_3669595, EPI_ISL_3669596, EPI_ISL_3669597, EPI_ISL_3671253, EPI_ISL_3671254, EPI_ISL_3671256, EPI_ISL_3671257, EPI_ISL_3671258, EPI_ISL_3671260, EPI_ISL_3671262, EPI_ISL_3671263, EPI_ISL_3671264, EPI_ISL_3671265, EPI_ISL_3671266, EPI_ISL_3671268, EPI_ISL_3671270, EPI_ISL_3671271, EPI_ISL_3671272, EPI_ISL_3671275, EPI_ISL_3671277, EPI_ISL_3671279, EPI_ISL_3671280, EPI_ISL_3672280, EPI_ISL_3695229, EPI_ISL_3695265, EPI_ISL_3695284, EPI_ISL_3695292, EPI_ISL_3695305, EPI_ISL_3695355, EPI_ISL_3695366, EPI_ISL_3695411, EPI_ISL_3695521, EPI_ISL_3695589, EPI_ISL_3695610, EPI_ISL_3695767, EPI_ISL_3695799, EPI_ISL_3695801, EPI_ISL_3717096			
see above	CDPH VBL	California Department of Public Health	CDPH-COVIDNet; Emily Smith on behalf of CDPH-COVIDNet and UCI Genome Sciences Center/GHTF; Emily Smith on behalf of CDPH-COVIDNet and UCSD EXCITE lab
EPI_ISL_2933353	CEDIBIO-UNILABS-TOUNIS	CNR Virus des Infections Respiratoires - France SUD	Antonin Bal; Bruno Lina; Gregory Destras; Gwendolyne Burfin; Hadrien Regue; Laurence Josset; Martine Valette; Quentin Semanas
EPI_ISL_2761738, EPI_ISL_2761751, EPI_ISL_2761779, EPI_ISL_2761840, EPI_ISL_2761867	CENTOGENE Frankfurt Laboratory: Niederlassung Industriepark Höchst	Robert Koch Institute	
EPI_ISL_2801364, EPI_ISL_3102261, EPI_ISL_3102274, EPI_ISL_3102278, EPI_ISL_3102288, EPI_ISL_3102305, EPI_ISL_3102495, EPI_ISL_3102496, EPI_ISL_3102497, EPI_ISL_3102498, EPI_ISL_3102499, EPI_ISL_3102501, EPI_ISL_3102502, EPI_ISL_3102503, EPI_ISL_3102504, EPI_ISL_3102507, EPI_ISL_3102524, EPI_ISL_3102525, EPI_ISL_3536352, EPI_ISL_3536354, EPI_ISL_3536355, EPI_ISL_3912138, EPI_ISL_3912139, EPI_ISL_3912140, EPI_ISL_3912141, EPI_ISL_3912142, EPI_ISL_3912143, EPI_ISL_3912144			
see above	CENTRO DE ATENDIMENTO PARA ENFRENTAMENTO AO COVID 19	Analytical Competence Molecular Epidemiology Lab/ACME, Oswaldo Cruz Foundation, Ceara (FIOCRUZ CE)	Cleber Furtado Aksenen; Cleber Furtado Aksenen e Suzana Porto Almeida; Fabio Miyajima; Fernando Braga Stehling; Francisco Eder de Moura Lopes; Jamille Maria Mendes Bezerra; Joaquim Cesar do Nascimento Sousa Junior; Joaquim César do Nascimento Sousa Junior; Pedro Miguel Carneiro Jeronimo; Suzana Porto Almeida & Lucas Delerino on behalf of COVID-19 FIOCRUZ Genomic Network; Suzana Porto Almeida e Lucas Delerino; Thais Ferreira de Oliveira; Thais de Oliveira Costa; Ticiane Cavalcante de Souza; Veridiana Pessoa Miyajima
EPI_ISL_2493790, EPI_ISL_2493791	CENTRO DE GENOMAS	Instituto Butantan	Antonio Jorge Martins; Claudia Renata dos Santos Barros; David Schlesinger; Debora Botequio Moretti; Dimas Tadeu Covas; Elaine Cristina Marqueze; Elaine Vieira Santos; Evandra Strazza Rodrigues; Heidge Fukumasu; Jayme Augusto de Souza-Neto; José Salvatore Leister Patané; Luiz Alcantara; Luiz Lehmann Coutinho; Maria Carolina Elias; Maurício Lacerda Nogueira; Rafael dos Santos Bezerra; Raul Machado Neto; Rejane Maria Tommasini Grotto; Ricardo Haddad; Sandra Coccuzzo Sampaio Vessoni; Simone Kashima; Svetoslav Nanev Slavov; Vincent Louis Viala
EPI_ISL_2493824, EPI_ISL_2493826, EPI_ISL_2494127, EPI_ISL_2494128	CENTRO DE REFERENCIA DO IDOSO DR HUMBERTO MENDES DE CARVALHO	Instituto Butantan	Antonio Jorge Martins; Claudia Renata dos Santos Barros; David Schlesinger; Debora Botequio Moretti; Dimas Tadeu Covas; Elaine Cristina Marqueze; Elaine Vieira Santos; Evandra Strazza Rodrigues; Heidge Fukumasu; Jayme Augusto de Souza-Neto; José Salvatore Leister Patané; Luiz Alcantara; Luiz Lehmann Coutinho; Maria Carolina Elias; Maurício Lacerda Nogueira; Rafael dos Santos Bezerra; Raul Machado Neto; Rejane Maria Tommasini Grotto; Ricardo Haddad; Sandra Coccuzzo Sampaio Vessoni; Simone Kashima; Svetoslav Nanev Slavov; Vincent Louis Viala
EPI_ISL_2494309	CENTRO DE REFERENCIA E TREINAMENTO DSTAIDS SAO PAULO	Instituto Butantan	Antonio Jorge Martins; Claudia Renata dos Santos Barros; David Schlesinger; Debora Botequio Moretti; Dimas Tadeu Covas; Elaine Cristina Marqueze; Elaine Vieira Santos; Evandra Strazza Rodrigues; Heidge Fukumasu; Jayme Augusto de Souza-Neto; José Salvatore Leister Patané; Luiz Alcantara; Luiz Lehmann Coutinho; Maria Carolina Elias; Maurício Lacerda Nogueira; Rafael dos Santos Bezerra; Raul Machado Neto; Rejane Maria Tommasini Grotto; Ricardo Haddad; Sandra Coccuzzo Sampaio Vessoni; Simone Kashima; Svetoslav Nanev Slavov; Vincent Louis Viala
EPI_ISL_2494179	CENTRO DE SAUDE DE ANALANDIA	Instituto Butantan	Antonio Jorge Martins; Claudia Renata dos Santos Barros; David Schlesinger; Debora Botequio Moretti; Dimas Tadeu Covas; Elaine Cristina Marqueze; Elaine Vieira Santos; Evandra Strazza Rodrigues; Heidge Fukumasu; Jayme Augusto de Souza-Neto; José Salvatore Leister Patané; Luiz Alcantara; Luiz Lehmann Coutinho; Maria Carolina Elias; Maurício Lacerda Nogueira; Rafael dos Santos Bezerra; Raul Machado Neto; Rejane Maria Tommasini Grotto; Ricardo Haddad; Sandra Coccuzzo Sampaio Vessoni; Simone Kashima; Svetoslav Nanev Slavov; Vincent Louis Viala
EPI_ISL_2493792, EPI_ISL_2493793, EPI_ISL_2493794, EPI_ISL_2493795, EPI_ISL_2493796	CENTRO DE SAUDE DE PIRAJUI	Instituto Butantan	Antonio Jorge Martins; Claudia Renata dos Santos Barros; David Schlesinger; Debora Botequio Moretti; Dimas Tadeu Covas; Elaine Cristina Marqueze; Elaine Vieira Santos; Evandra Strazza Rodrigues; Heidge Fukumasu; Jayme Augusto de Souza-Neto; José Salvatore Leister Patané; Luiz Alcantara; Luiz Lehmann Coutinho; Maria Carolina Elias; Maurício Lacerda Nogueira; Rafael dos Santos Bezerra; Raul Machado Neto; Rejane Maria Tommasini Grotto; Ricardo Haddad; Sandra Coccuzzo Sampaio Vessoni; Simone Kashima; Svetoslav Nanev Slavov; Vincent Louis Viala
EPI_ISL_3536339, EPI_ISL_3536340	CENTRO DE SAUDE DONA TEREZA HOLANDA DE OLIVEIRA	Analytical Competence Molecular Epidemiology Lab/ACME, Oswaldo Cruz Foundation, Ceara (FIOCRUZ CE)	Cleber Furtado Aksenen; Fabio Miyajima; Fernando Braga Stehling; Francisco Eder de Moura Lopes; Jamille Maria Mendes Bezerra; Joaquim César do Nascimento Sousa Junior; Pedro Miguel Carneiro Jeronimo; Suzana Porto Almeida e Lucas Delerino; Thais Ferreira de Oliveira; Thais de Oliveira Costa; Ticiane Cavalcante de Souza; Veridiana Pessoa Miyajima
EPI_ISL_2494195	CENTRO DE SAUDE II DR JOSE DE FELIPE E S PINHAL SP	Instituto Butantan	Antonio Jorge Martins; Claudia Renata dos Santos Barros; David Schlesinger; Debora Botequio Moretti; Dimas Tadeu Covas; Elaine Cristina Marqueze; Elaine Vieira Santos; Evandra Strazza Rodrigues; Heidge Fukumasu; Jayme Augusto de Souza-Neto; José Salvatore Leister Patané; Luiz Alcantara; Luiz Lehmann Coutinho; Maria Carolina Elias; Maurício Lacerda Nogueira; Rafael dos Santos Bezerra; Raul Machado Neto; Rejane Maria Tommasini Grotto; Ricardo Haddad; Sandra Coccuzzo Sampaio Vessoni; Simone Kashima; Svetoslav Nanev Slavov; Vincent Louis Viala
EPI_ISL_2494165, EPI_ISL_2494186	CENTRO DE SAUDE III DE DIVINOLANDIA	Instituto Butantan	Antonio Jorge Martins; Claudia Renata dos Santos Barros; David Schlesinger; Debora Botequio Moretti; Dimas Tadeu Covas; Elaine Cristina Marqueze; Elaine Vieira Santos; Evandra Strazza Rodrigues; Heidge Fukumasu; Jayme Augusto de Souza-Neto; José Salvatore Leister Patané; Luiz Alcantara; Luiz Lehmann Coutinho; Maria Carolina Elias; Maurício Lacerda Nogueira; Rafael dos Santos Bezerra; Raul Machado Neto; Rejane Maria Tommasini Grotto; Ricardo Haddad; Sandra Coccuzzo Sampaio Vessoni; Simone Kashima; Svetoslav Nanev Slavov; Vincent Louis Viala
EPI_ISL_2493822	CENTRO DE SAUDE III SANTA LUCIA	Instituto Butantan	Antonio Jorge Martins; Claudia Renata dos Santos Barros; David Schlesinger; Debora Botequio Moretti; Dimas Tadeu Covas; Elaine Cristina Marqueze; Elaine Vieira Santos; Evandra Strazza Rodrigues; Heidge Fukumasu; Jayme Augusto de Souza-Neto; José Salvatore Leister Patané; Luiz Alcantara; Luiz Lehmann Coutinho; Maria Carolina Elias; Maurício Lacerda Nogueira; Rafael dos Santos Bezerra; Raul Machado Neto; Rejane Maria Tommasini Grotto; Ricardo Haddad; Sandra Coccuzzo Sampaio Vessoni; Simone Kashima; Svetoslav Nanev Slavov; Vincent Louis Viala
EPI_ISL_2494109, EPI_ISL_2494115, EPI_ISL_2494123	CENTRO DE SAUDE III TABATINGA	Instituto Butantan	Antonio Jorge Martins; Claudia Renata dos Santos Barros; David Schlesinger; Debora Botequio Moretti; Dimas Tadeu Covas; Elaine Cristina Marqueze; Elaine Vieira Santos; Evandra Strazza Rodrigues; Heidge Fukumasu; Jayme Augusto de Souza-Neto; José Salvatore Leister Patané; Luiz Alcantara; Luiz Lehmann Coutinho; Maria Carolina Elias; Maurício Lacerda Nogueira; Rafael dos Santos Bezerra; Raul Machado Neto; Rejane Maria Tommasini Grotto; Ricardo Haddad; Sandra Coccuzzo Sampaio Vessoni; Simone Kashima; Svetoslav Nanev Slavov; Vincent Louis Viala
EPI_ISL_3536360, EPI_ISL_3536361	CENTRO DE SAUDE IRACEMA BRAGA SANDERS	Analytical Competence Molecular Epidemiology Lab/ACME, Oswaldo Cruz Foundation, Ceara (FIOCRUZ CE)	Cleber Furtado Aksenen; Fabio Miyajima; Fernando Braga Stehling; Francisco Eder de Moura Lopes; Jamille Maria Mendes Bezerra; Joaquim César do Nascimento Sousa Junior; Pedro Miguel Carneiro Jeronimo; Suzana Porto Almeida e Lucas Delerino; Thais Ferreira de Oliveira; Thais de Oliveira Costa; Ticiane Cavalcante de Souza; Veridiana Pessoa Miyajima
EPI_ISL_2493950, EPI_ISL_2493951	CENTRO DE SAUDE SAO ROQUE DR JOSE CARVALHO BRITO	Instituto Butantan	Antonio Jorge Martins; Claudia Renata dos Santos Barros; David Schlesinger; Debora Botequio Moretti; Dimas Tadeu Covas; Elaine Cristina Marqueze; Elaine Vieira Santos; Evandra Strazza Rodrigues; Heidge Fukumasu; Jayme Augusto de Souza-Neto; José Salvatore Leister Patané; Luiz Alcantara; Luiz Lehmann Coutinho; Maria Carolina Elias; Maurício Lacerda Nogueira; Rafael dos Santos Bezerra; Raul Machado Neto; Rejane Maria Tommasini Grotto; Ricardo Haddad; Sandra Coccuzzo Sampaio Vessoni; Simone Kashima; Svetoslav Nanev Slavov; Vincent Louis Viala
EPI_ISL_2943231	CENTRO MEDICO NAVAL SEMAR LAB BIOLOGIA MOLECULAR	CENTRO MEDICO NAVAL SEMAR LAB PATOLOGIA MOLECULAR	MEIXUEIRO CALDERON CLAUDIA. MORALES RAMIREZ OMAR. BALTIERRA URIBE SHANTAL. ZUNO CORONADO TEUTLI. GARCIA ALEGRIA IRMA.ALVAREZ JIMENEZ VIOLETA DEJANIRA. LOPEZ BUSTOS ABRAHAM. CABRERA GONZALEZ MOISES. ROMERO ROMAN EDGAR. LIEVANO ESQUINCA JAZMIN. FIGUEROA ANTONIO VICTOR. DIAZ PONCE MEDRANO JUAN ALBERTO.
EPI_ISL_2462325, EPI_ISL_2494130	CENTRO MUNICIPAL DE SAUDE RUY DE CAMARGO	Instituto Butantan	Antonio Jorge Martins; Claudia Renata dos Santos Barros; David Schlesinger; Debora Botequio Moretti; Dimas Tadeu Covas; Elaine Cristina Marqueze; Elaine Vieira Santos; Evandra Strazza Rodrigues; Heidge Fukumasu; Jayme Augusto de Souza-Neto; José Salvatore Leister Patané; Luiz Alcantara; Luiz Lehmann Coutinho; Maria Carolina Elias; Maurício Lacerda Nogueira; Rafael dos Santos Bezerra; Raul Machado Neto; Rejane Maria Tommasini Grotto; Ricardo Haddad; Sandra Coccuzzo Sampaio Vessoni; Simone Kashima; Svetoslav Nanev Slavov; Vincent Louis Viala
EPI_ISL_2494159, EPI_ISL_2494188	CENTRO SAUDE III DR JOSE TADEU MOURAO	Instituto Butantan	Antonio Jorge Martins; Claudia Renata dos Santos Barros; David Schlesinger; Debora Botequio Moretti; Dimas Tadeu Covas; Elaine Cristina Marqueze; Elaine Vieira Santos; Evandra Strazza Rodrigues; Heidge Fukumasu; Jayme Augusto de Souza-Neto; José Salvatore Leister Patané; Luiz Alcantara; Luiz Lehmann Coutinho; Maria Carolina Elias; Maurício Lacerda Nogueira; Rafael dos Santos Bezerra; Raul Machado Neto; Rejane Maria Tommasini Grotto; Ricardo Haddad; Sandra Coccuzzo Sampaio Vessoni; Simone Kashima; Svetoslav Nanev Slavov; Vincent Louis Viala
EPI_ISL_2933380	CERBALLIANCE MIDI-PYRENEES	CNR Virus des Infections Respiratoires - France SUD	Antonin Bal; Bruno Lina; Gregory Destras; Gwendolyne Burfin; Hadrien Regue; Laurence Josset; Martine Valette; Quentin Semanas
EPI_ISL_2637834	CERBALLIANCE Valmy	Department of Virology, Henri Mondor University Hospital, Assistance Publique Hôpitaux de Paris, Université Paris-Est Créteil, INSERM U955	Alexandre Soulier; Christophe Rodriguez; Elisabeth Trawinski; Guillaume Gricourt; Jean-Michel Pawlotsky; Melissa N'Debi; Slim Fourati; Vanessa Demontant
EPI_ISL_2887729	CH Valenciennes	CHU Lille - Laboratoire de Virologie	AIT YAHYA Emilie; ALIDJINOU Enagnon Kazali; BOCKET Laurence; CREPIN Michel; DEMAY Christophe; ENGELMANN Ilka; GEFFROY Sandrine; GUIGON Aurélie; LAMBERT Valérie; LAZREK Mouna; NOBILLIAUX Florian; PREVOST Brigitte; TCHANTCHOU NJOSSE YANICK; THUILLIER Caroline; TINEZ Claire
EPI_ISL_2426982, EPI_ISL_2627848, EPI_ISL_2627853, EPI_ISL_2627854, EPI_ISL_2627855, EPI_ISL_2627856, EPI_ISL_2627857, EPI_ISL_2627858, EPI_ISL_2627859, EPI_ISL_2629224, EPI_ISL_2629232, EPI_ISL_2629233, EPI_ISL_2629234, EPI_ISL_2835977			

see above	CHC Andrée Rosemon	Institut Pasteur de la Guyane	Anne Lavergne; Antoine Enfissi; Arielle Salmier; Dominique Rousset
EPI_ISL_2695339, EPI_ISL_2695345, EPI_ISL_2895275	CHEDV	Instituto Nacional de Saude (INSA)	Borges et al
EPI_ISL_3234556	CHU Purpan - Laboratoire de Virologie - Institut Fédératif de Biologie	CHU Purpan - Laboratoire de Virologie - Institut Fédératif de Biologie	Bulach T.; Donnadieu C.; Izopet J.; Latour J.; Milhes M.; Nicot F.; Ranger N.; Salin G.; Tremeaux P.
EPI_ISL_2695098	CHULN - H Santa Maria	Instituto Nacional de Saude (INSA)	Borges et al
EPI_ISL_2637260, EPI_ISL_2957044, EPI_ISL_3030542, EPI_ISL_3030557	CLILAB	Microbiology Department	Aida Gonzalez-Diaz; Carmen Ardanuy; Jordi Camara; Jordi Niubó; Laura Calatayud; M Angeles Domínguez; Miguel Fernandez-Huerta; Sara Marti
EPI_ISL_3298334	CLINICA BIBLICA	Inciensa, Instituto Costarricense de Investigación y Enseñanza en Nutrición y Salud	Adriana Godínez; Claudio Soto-Garita; Estela Cordero; Francisco Duarte; Hebleen Porras; Joselyn Prado & Karla Gutiérrez; José Luis Vargas; Mariela Gutiérrez; Melany Calderón
EPI_ISL_2426983, EPI_ISL_2426984, EPI_ISL_2627860, EPI_ISL_2627861, EPI_ISL_2627864, EPI_ISL_2627865, EPI_ISL_2627866, EPI_ISL_2629235, EPI_ISL_2629236, EPI_ISL_2629237, EPI_ISL_2835978, EPI_ISL_2835979, EPI_ISL_2835980, EPI_ISL_2835981, EPI_ISL_2835982, EPI_ISL_2835984, EPI_ISL_2983480			
see above	CNR Institut Pasteur de la Guyane	Institut Pasteur de la Guyane	Anne Lavergne; Antoine Enfissi; Arielle Salmier; Dominique Rousset
EPI_ISL_2671834	COVID LAB	Cayman Islands Forensic Science Lab	Allen Grolla; Brittany Balcewich; Jonathan Smellie; Karla Montes
EPI_ISL_2494183	CS III DR ANTONIO ANADAO SSGRAMA	Instituto Butantan	Antonio Jorge Martins; Claudia Renata dos Santos Barros; David Schlesinger; Debora Botequio Moretti; Dimas Tadeu Covas; Elaine Cristina Marqueze; Elaine Vieira Santos; Evandra Strazza Rodrigues; Heidge Fukumasu; Jayme Augusto de Souza-Neto; José Salvatore Leister Patané; Luiz Alcantara; Luiz Lehmann Coutinho; Maria Carolina Elias; Maurício Lacerda Nogueira; Rafael dos Santos Bezerra; Raul Machado Neto; Rejane Maria Tommasini Grotto; Ricardo Haddad; Sandra Coccuzzo Sampaio Vessoni; Simone Kashima; Svetoslav Nanev Slavov; Vincent Louis Viala
EPI_ISL_3912151, EPI_ISL_3912152	CSF ARACATIACU	Analytical Competence Molecular Epidemiology Lab/ACME, Oswaldo Cruz Foundation, Ceara (FIOCRUZ CE)	Cleber Furtado Aksenen; Fabio Miyajima; Fernando Braga Stehling; Francisco Eder de Moura Lopes; Jamille Maria Mendes Bezerra; Joaquim Cesar do Nascimento Sousa Junior; Pedro Miguel Carneiro Jeronimo; Suzana Porto Almeida & Lucas Delerino on behalf of COVID-19 FIOCRUZ Genomic Network; Thais Ferreira de Oliveira; Thais de Oliveira Costa; Ticiane Cavalcante de Souza; Veridiana Pessoa Miyajima
EPI_ISL_3912153	CSF COELCE	Analytical Competence Molecular Epidemiology Lab/ACME, Oswaldo Cruz Foundation, Ceara (FIOCRUZ CE)	Cleber Furtado Aksenen; Fabio Miyajima; Fernando Braga Stehling; Francisco Eder de Moura Lopes; Jamille Maria Mendes Bezerra; Joaquim Cesar do Nascimento Sousa Junior; Pedro Miguel Carneiro Jeronimo; Suzana Porto Almeida & Lucas Delerino on behalf of COVID-19 FIOCRUZ Genomic Network; Thais Ferreira de Oliveira; Thais de Oliveira Costa; Ticiane Cavalcante de Souza; Veridiana Pessoa Miyajima
EPI_ISL_3912155	CSF JUNCO	Analytical Competence Molecular Epidemiology Lab/ACME, Oswaldo Cruz Foundation, Ceara (FIOCRUZ CE)	Cleber Furtado Aksenen; Fabio Miyajima; Fernando Braga Stehling; Francisco Eder de Moura Lopes; Jamille Maria Mendes Bezerra; Joaquim Cesar do Nascimento Sousa Junior; Pedro Miguel Carneiro Jeronimo; Suzana Porto Almeida & Lucas Delerino on behalf of COVID-19 FIOCRUZ Genomic Network; Thais Ferreira de Oliveira; Thais de Oliveira Costa; Ticiane Cavalcante de Souza; Veridiana Pessoa Miyajima
EPI_ISL_3912158	CSF TERRENOS NOVOS 1	Analytical Competence Molecular Epidemiology Lab/ACME, Oswaldo Cruz Foundation, Ceara (FIOCRUZ CE)	Cleber Furtado Aksenen; Fabio Miyajima; Fernando Braga Stehling; Francisco Eder de Moura Lopes; Jamille Maria Mendes Bezerra; Joaquim Cesar do Nascimento Sousa Junior; Pedro Miguel Carneiro Jeronimo; Suzana Porto Almeida & Lucas Delerino on behalf of COVID-19 FIOCRUZ Genomic Network; Thais Ferreira de Oliveira; Thais de Oliveira Costa; Ticiane Cavalcante de Souza; Veridiana Pessoa Miyajima
EPI_ISL_2493830, EPI_ISL_2494321	CSII DR WASHINGTON LUIS M RODRIGUES DA SILVA PITANGUEIRAS	Instituto Butantan	Antonio Jorge Martins; Claudia Renata dos Santos Barros; David Schlesinger; Debora Botequio Moretti; Dimas Tadeu Covas; Elaine Cristina Marqueze; Elaine Vieira Santos; Evandra Strazza Rodrigues; Heidge Fukumasu; Jayme Augusto de Souza-Neto; José Salvatore Leister Patané; Luiz Alcantara; Luiz Lehmann Coutinho; Maria Carolina Elias; Maurício Lacerda Nogueira; Rafael dos Santos Bezerra; Raul Machado Neto; Rejane Maria Tommasini Grotto; Ricardo Haddad; Sandra Coccuzzo Sampaio Vessoni; Simone Kashima; Svetoslav Nanev Slavov; Vincent Louis Viala
EPI_ISL_2892470, EPI_ISL_2892517, EPI_ISL_3536935, EPI_ISL_4005556, EPI_ISL_4005559, EPI_ISL_4005560, EPI_ISL_4005561, EPI_ISL_4005562, EPI_ISL_4005564, EPI_ISL_4005578			
see above	California Department of Public Health	California Department of Public Health	CDPH IDLB COVIDNet et al
EPI_ISL_3134074, EPI_ISL_3134105, EPI_ISL_3134106, EPI_ISL_3134109, EPI_ISL_3134116, EPI_ISL_3134118, EPI_ISL_3134121, EPI_ISL_3134124, EPI_ISL_3134128, EPI_ISL_3134129, EPI_ISL_3134147, EPI_ISL_3134158, EPI_ISL_3134162			
see above	California Department of Public Health Valencia Branch Laboratory	California Department of Public Health	CDPH-COVIDNet; UCSF Center for Advanced Technology
EPI_ISL_2774013, EPI_ISL_3911439, EPI_ISL_3911440, EPI_ISL_3911441, EPI_ISL_3911466, EPI_ISL_3911488	Cantacuzino National Military-Medical Institute, Viral Respiratory Infections Laboratory	Cantacuzino Institute Virology	Carmen Cherciu; Luiza Ustea; Mihaela Lazar; Mihaela Oprea; Nicoleta Paraschiv; Sorin Dinu
EPI_ISL_2502507, EPI_ISL_3462615, EPI_ISL_3462616, EPI_ISL_3462617, EPI_ISL_3462618, EPI_ISL_3462619, EPI_ISL_3462620, EPI_ISL_3462627, EPI_ISL_3462628, EPI_ISL_3462629, EPI_ISL_3462630, EPI_ISL_3462631, EPI_ISL_3462632, EPI_ISL_3462633, EPI_ISL_3462634, EPI_ISL_3462635, EPI_ISL_3462636, EPI_ISL_3462637, EPI_ISL_3462638, EPI_ISL_3462639, EPI_ISL_3462640, EPI_ISL_3462641, EPI_ISL_3462642, EPI_ISL_3462643, EPI_ISL_3462644, EPI_ISL_3462645, EPI_ISL_3462646, EPI_ISL_3462647, EPI_ISL_3462648, EPI_ISL_3462649, EPI_ISL_3462650, EPI_ISL_3462651, EPI_ISL_3462652, EPI_ISL_3462653, EPI_ISL_3462654, EPI_ISL_3462655, EPI_ISL_3462656, EPI_ISL_3462657, EPI_ISL_3462658, EPI_ISL_3462659, EPI_ISL_3462660, EPI_ISL_3462661, EPI_ISL_3462662, EPI_ISL_3462663, EPI_ISL_3462664, EPI_ISL_3462665, EPI_ISL_3462666, EPI_ISL_3462667, EPI_ISL_3462668, EPI_ISL_3462669, EPI_ISL_3462670, EPI_ISL_3462671, EPI_ISL_3462672, EPI_ISL_3462673, EPI_ISL_3462674, EPI_ISL_3462675, EPI_ISL_3462676, EPI_ISL_3462677, EPI_ISL_3462678, EPI_ISL_3462679, EPI_ISL_3462680, EPI_ISL_3462681, EPI_ISL_3462682, EPI_ISL_3462683, EPI_ISL_3462684, EPI_ISL_3462685, EPI_ISL_3462686, EPI_ISL_3462687, EPI_ISL_3462688, EPI_ISL_3462689, EPI_ISL_3462690, EPI_ISL_3462691, EPI_ISL_3462692, EPI_ISL_3462693, EPI_ISL_3462694, EPI_ISL_3462695, EPI_ISL_3462727, EPI_ISL_3462728, EPI_ISL_3464729, EPI_ISL_3464730, EPI_ISL_3464731, EPI_ISL_3464732, EPI_ISL_3464733, EPI_ISL_3464735, EPI_ISL_3464736			
see above	Central Laboratory, Bureau of Public Health (BOG) and Academic Hospital Paramaribo	Erasmus Medical Center	Bas B Oude Munnink; Cherise Beek; Consuella Partowidjojo; Dion Gajadin; Ed PF IJzerman; Emmanuelle Munger; Gary Gummels; Ingrid SK Krishnadath; Lycke Woittiez; Marion PG Koopmans; Mireille Van de Veer; Phyllis Pinas; Princes Wongsowidjojo; Radjesh Ori; Ranisha Doerbalie; Rohma Banwari; Soeradj Harkisoen; Stephen Vreden; Tilotmadebie Ramlal; Verne Nanhoe
EPI_ISL_3255135, EPI_ISL_3255137, EPI_ISL_3255138, EPI_ISL_3255140, EPI_ISL_3255144, EPI_ISL_3255145, EPI_ISL_3255146, EPI_ISL_3255148, EPI_ISL_3255149, EPI_ISL_3255151, EPI_ISL_3255154, EPI_ISL_3255155, EPI_ISL_3255156, EPI_ISL_3255157, EPI_ISL_3255159, EPI_ISL_3255160, EPI_ISL_3255163, EPI_ISL_3255164, EPI_ISL_3255166, EPI_ISL_3255168, EPI_ISL_3255171, EPI_ISL_3255174, EPI_ISL_3255176, EPI_ISL_3255180, EPI_ISL_3255181, EPI_ISL_3255182, EPI_ISL_3255183, EPI_ISL_3255184, EPI_ISL_3255188, EPI_ISL_3255189, EPI_ISL_3255190, EPI_ISL_3255196, EPI_ISL_3255203, EPI_ISL_3255204, EPI_ISL_3255210, EPI_ISL_3255211, EPI_ISL_3255213, EPI_ISL_3255215, EPI_ISL_3255217, EPI_ISL_3255218, EPI_ISL_3255220, EPI_ISL_3255222, EPI_ISL_3255224, EPI_ISL_3255225, EPI_ISL_3255228, EPI_ISL_3255229, EPI_ISL_3255231, EPI_ISL_3255234, EPI_ISL_3255235, EPI_ISL_3255236, EPI_ISL_3255238, EPI_ISL_3255239, EPI_ISL_3255241, EPI_ISL_3255242, EPI_ISL_3255243, EPI_ISL_3255245, EPI_ISL_3255247, EPI_ISL_3255249, EPI_ISL_3255250, EPI_ISL_3255252, EPI_ISL_3255254, EPI_ISL_3255257, EPI_ISL_3255258, EPI_ISL_3255261, EPI_ISL_3255262, EPI_ISL_3255263, EPI_ISL_3255264, EPI_ISL_3255266, EPI_ISL_3255269, EPI_ISL_3255270, EPI_ISL_3255271, EPI_ISL_3255272, EPI_ISL_3255273, EPI_ISL_3255274, EPI_ISL_3255275, EPI_ISL_3255276, EPI_ISL_3255277, EPI_ISL_3255278, EPI_ISL_3255279, EPI_ISL_3255280, EPI_ISL_3255281			
see above	Central Public Health Laboratory - LACEN - Bahia, Salvador, Brazil	Central Public Health Laboratory - LACEN -Bahia, Salvador, Brazil	Arabela Leal; Breno Dominguez; Felicidade Pereira; Jaqueline Gomes; Luciana Oliveira; Luiz Alcantara; Marcela Gómez; Marta Giovanetti; Patrícia Cajado; Stephane Tosta; Vagner Fonseca; Vanessa Nardy
EPI_ISL_3832900	Central Washington Hospital	Seattle Flu Study	Amanda Adler; Barry R. Lutz; Benjamin Pelle; Brian Hiatt; Caitlin R. Wolf; Chris D. Frazar; Deborah A. Nickerson; Elisabeth Brandstetter; Erica Ryke; Geoff Melly; Helen Y. Chu; Janet A. Englund; Jay Shendure; Jover Lee; Kairsten Fay; Kirsten Lacombe; Lea M. Starita; Mark J. Rieder; Matthew Richardson; Matthew Thompson; Melissa Truong; Michael Boeckh; Michael Famulare; Misja Ilcisin; Peter D. Han; Philip Dykema; Romesh Gautom; Scott Lindquist; Thomas R. Sibley; Trevor Bedford
EPI_ISL_2491661	Centre Hospitalier Universitaire Clermont-Ferrand	CHU Clermont-Ferrand, service de virologie	Bisseux Maxime; Combes Patricia; Henquell Cécile; Mirand Audrey
EPI_ISL_2671574, EPI_ISL_2671575, EPI_ISL_2671576, EPI_ISL_2671582, EPI_ISL_2671585, EPI_ISL_2671586, EPI_ISL_2671703, EPI_ISL_2942410, EPI_ISL_2942411, EPI_ISL_2942418, EPI_ISL_2942426, EPI_ISL_2942428, EPI_ISL_2942434, EPI_ISL_2942438, EPI_ISL_2942440, EPI_ISL_2942460, EPI_ISL_2942732, EPI_ISL_2942737			
see above	Centro de Investigacion Biomedica de Occidente (CIBO)	Unidad de Genomica Avanzada	; Alejandra Garcia-Gasca; Alejandra Hernandez-Teran; Alejandro Sanchez-Flores; Alfredo Herrera-Estrella; Alicia Ocaña-Mondragon; Andreu Comas-Garcia; Angel Gustavo Salas-Lais; Antonio Loza Roman; Bernardo Martinez-Miguel; Blanca Taboada; Brenda Irasema Maldonado-Meza; Bruno Gomez-Gil; Carla Ivon Herrera-Najera; Carlos F. Arias; Celia Boukadida; Celida Duque Molina; Celida Martinez- Rodriguez; Clara Esperanza Santacruz-Tinoco; Concepcion Grajales-Muñiz; Consorcio Mexicano de Vigilancia Genomica (CoViGen-Mex). Authors (in alphabetical order): Julio Elias Alvarado-Yaah; Cristobal Chaidez-Quiroz; Daniel Fregoso-Rueda; Daniel Lira Morales; Eduardo Becerril-Vargas; Fernando Fontove-Herrera; Fidencio Mejia-Nepomuceno; Francisco Pulido; Gloria Elena Espinosa-Ayala; Gloria Maria Molina-Salinas; Gloria Vazquez; Hector Esteban Paz-Juarez; Hector Montoya-Fuentes; Helen Haydee Fernanda Ramirez-Plascencia; Irvin Gonzalez-Lopez; Jean Pierre Gonzalez; Jesus Hernandez; Joel Armando Vazquez-Perez.; Jorge Salas-Hernandez; Jose Antonio Enciso-Moreno; Jose Arturo Martinez-Orozco; Jose Esteban Muñoz-Medina; Jose de Jesus Nuñez-Contreras; Juan Bautista Chale-Dzul; Julissa Enciso-Ibarra; Luis Alberto Ochoa-Carrera; Margarita Matias-Florentino; Maria Guadalupe Santiago-Mauricio; Maria Guadalupe de Jesus Mireles-Rivera; Mario Mujica-Sanchez; Marissa Perez-Garcia; Nelly Selem-Mojica; Pavel Isa; Ricardo Ciria Merce; Ricardo Grande; Rosa Maria Gutierrez Rios; Santiago avila-Rios; Selene Zarate; Susana Lopez; Veronica Mata-Haro; Victor Eduardo Garcia-Arias; Victor Hugo Borja-Aburto
EPI_ISL_2671560, EPI_ISL_2942484, EPI_ISL_2942755, EPI_ISL_2942756, EPI_ISL_2942770	Centro de Investigacion Biomedica del Noreste (CIBIN)	Unidad de Genomica Avanzada	; Alejandra Garcia-Gasca; Alejandra Hernandez-Teran; Alejandro Sanchez-Flores; Alfredo Herrera-Estrella; Alicia Ocaña-Mondragon; Andreu Comas-Garcia; Angel Gustavo Salas-Lais; Antonio Loza Roman; Bernardo Martinez-Miguel; Blanca Taboada; Brenda Irasema Maldonado-Meza; Bruno Gomez-Gil; Carla Ivon Herrera-Najera; Carlos F. Arias; Celia Boukadida; Celida Duque Molina; Celida Martinez- Rodriguez; Clara Esperanza Santacruz-Tinoco; Concepcion Grajales-Muñiz; Consorcio Mexicano de Vigilancia Genomica (CoViGen-Mex). Authors (in alphabetical order): Julio Elias Alvarado-Yaah; Cristobal Chaidez-Quiroz; Daniel Fregoso-Rueda; Daniel Lira Morales; Eduardo Becerril-Vargas; Fernando Fontove-Herrera; Fidencio Mejia-Nepomuceno; Francisco Pulido; Gloria Elena Espinosa-Ayala; Gloria Maria Molina-Salinas; Gloria Vazquez; Hector Esteban Paz-Juarez; Hector Montoya-Fuentes; Helen Haydee Fernanda Ramirez-Plascencia; Irvin Gonzalez-Lopez; Jean Pierre Gonzalez; Jesus Hernandez; Joel Armando Vazquez-Perez.; Jorge Salas-Hernandez; Jose Antonio Enciso-Moreno; Jose Arturo Martinez-Orozco; Jose Esteban Muñoz-Medina; Jose de Jesus Nuñez-Contreras; Juan Bautista Chale-Dzul; Julissa Enciso-Ibarra; Luis Alberto Ochoa-Carrera; Margarita Matias-Florentino; Maria Guadalupe Santiago-Mauricio; Maria Guadalupe de Jesus Mireles-Rivera; Mario Mujica-Sanchez; Marissa Perez-Garcia; Nelly Selem-Mojica; Pavel Isa; Ricardo Ciria Merce; Ricardo Grande; Rosa Maria Gutierrez Rios; Santiago avila-Rios; Selene Zarate; Susana Lopez; Veronica Mata-Haro; Victor Eduardo Garcia-Arias; Victor Hugo Borja-Aburto
EPI_ISL_3019538, EPI_ISL_3129919	Centro de Investigación Biomédica de La Rioja - Hospital San Pedro Logroño	SeqCOVID-SPAIN consortium/IBV(CSIC)	José Manuel Azcona Gutiérrez; María Pilar Bea Escudero; María de Toro; Miriam Blasco Alberdi and SeqCOVID-SPAIN consortium
EPI_ISL_3155465, EPI_ISL_3155471, EPI_ISL_3155472, EPI_ISL_3155489, EPI_ISL_3155492, EPI_ISL_3155494, EPI_ISL_3155500			
see above	Centro de Investigación Biomédica de Occidente (CIBO)	Centro de Investigación en Enfermedades Infecciosas (CIENI), Instituto Nacional de Enfermedades Respiratorias (INER)	; Alejandra García-Gasca; Alejandra Hernández-Terán; Alejandro Sánchez-Flores; Alfredo Herrera-Estrella; Alicia Ocaña-Mondragón; Andreu Comas-García; Angel Gustavo Salas-Lais; Antonio Loza Román; Bernardo Martínez-Miguel; Blanca Taboada; Brenda Irasema Maldonado-Meza; Bruno Gómez-Gil; Carla Ivón Herrera-Najera; Carlos F. Arias; Celia Boukadida; Clara Esperanza Santacruz-Tinoco; Concepción Grajales-Muñiz; Consorcio Mexicano de Vigilancia Genómica (CoViGen-Mex). Authors (in alphabetical order): Julio Elias Alvarado-Yaah; Cristóbal Cháidez-Quiróz; Célida Duque Molina; Célida Martínez- Rodríguez; Daniel Fregoso-Rueda; Daniel Lira Morales; Eduardo Becerril-Vargas; Fernando Fontove-Herrera; Fidencio Mejía-Nepomuceno; Francisco Pulido; Gloria Elena Espinosa-Ayala; Gloria María Molina-Salinas; Gloria Vazquez; Hector Esteban Paz-Juárez; Hector Montoya-Fuentes; Helen Haydee Fernanda Ramírez-Plascencia; Irvin González-López; Jean Pierre González; Jesús Hernández; Joel Armando Vázquez-Pérez.; Jorge Salas-Hernández; José Antonio Enciso-Moreno; José Arturo Martínez-Orozco; José Esteban Muñoz-Medina; José de Jesús Nuñez-Contreras; Juan Bautista Chale-Dzul; Julissa Enciso-Ibarra; Kathia Elizabeth Tapia-Diaz; Luis Alberto Ochoa-Carrera; Margarita Matías-Florentino; Mario Mújica-Sánchez; Marissa Perez-Garcia; María Guadalupe Santiago-Mauricio; María Guadalupe de Jesús Mireles-Rivera; Nelly Sélem-Mojica; Pavel Isa; Ricardo Ciria Merce; Ricardo Grande; Rosa María Gutiérrez Rios; Santiago Ávila-Ríos; Selene Zárate; Susana Lopez; Verónica Mata-Haro; Víctor Eduardo García-Arias; Víctor Hugo Borja-Aburto
EPI_ISL_2801721, EPI_ISL_2801726, EPI_ISL_2801729, EPI_ISL_2801731, EPI_ISL_2801733, EPI_ISL_2801736, EPI_ISL_2801742, EPI_ISL_2801746, EPI_ISL_2801750			
see above	Centro de Investigación Biomédica de Occidente (CIBO)	Instituto de Biotecnología de la UNAM	; Alejandra García-Gasca; Alejandra Hernández-Terán; Alejandro Sánchez-Flores; Alfredo Herrera-Estrella; Alicia Ocaña-Mondragón; Andreu Comas-García; Angel Gustavo Salas-Lais; Antonio Loza Román; Bernardo Martínez-Miguel; Blanca Taboada; Brenda Irasema Maldonado-Meza; Bruno Gómez-Gil; Carla Ivón Herrera-Najera; Carlos F. Arias; Celia Boukadida; Clara Esperanza Santacruz-Tinoco; Concepción Grajales-Muñiz; Consorcio Mexicano de Vigilancia Genómica (CoViGen-Mex). Authors (in alphabetical order): Julio Elias Alvarado-Yaah; Cristóbal Cháidez-Quiróz; Célida Duque Molina; Célida Martínez- Rodríguez; Daniel Fregoso-Rueda; Daniel Lira Morales; Eduardo Becerril-Vargas; Fernando Fontove-Herrera; Fidencio Mejía-Nepomuceno; Francisco Pulido; Gloria Elena Espinosa-Ayala; Gloria María Molina-Salinas; Gloria Vazquez; Hector Esteban Paz-Juárez; Hector Montoya-Fuentes; Helen Haydee Fernanda Ramírez-Plascencia; Irvin González-López; Jean Pierre González; Jesús Hernández; Joel Armando Vázquez-Pérez.; Jorge Salas-Hernández; José Antonio Enciso-Moreno; José Arturo Martínez-Orozco; José Esteban Muñoz-Medina; José de Jesús Nuñez-Contreras; Juan Bautista Chale-Dzul;

			Julissa Enciso-Ibarra; Luis Alberto Ochoa-Carrera; Margarita Matías-Florentino; Mario Mújica-Sánchez; Marissa Perez-Garcia; María Guadalupe Santiago-Mauricio; María Guadalupe de Jesús Mireles-Rivera; Nelly Sélem-Mojica; Pavel Isa; Ricardo Ciria Merce; Ricardo Grande; Rosa María Gutiérrez Rios; Santiago Ávila-Ríos; Selene Zárate; Susana Lopez; Verónica Mata-Haro; Víctor Eduardo García-Arias; Víctor Hugo Borja-Aburto
EPI_ISL_2681227, EPI_ISL_2801658, EPI_ISL_2801669, EPI_ISL_2801674, EPI_ISL_2801680, EPI_ISL_2801681	Centro de Investigación Biomédica del Noreste (CIBIN)	Instituto de Biotecnología de la UNAM	; Alejandra García-Gasca; Alejandra Hernández-Terán; Alejandro Sánchez-Flores; Alfredo Herrera-Estrella; Alicia Ocaña-Mondragón; Andreu Comas-García; Angel Gustavo Salas-Lais; Antonio Loza Román; Bernardo Martínez-Miguel; Blanca Taboada; Brenda Irasema Maldonado-Meza; Bruno Gómez-Gil; Carla Ivón Herrera-Najera; Carlos F. Arias; Celia Boukadida; Clara Esperanza Santacruz-Tinoco; Concepción Grajales-Muñiz; Consorcio Mexicano de Vigilancia Genómica (CoViGen-Mex). Authors (in alphabetical order): Julio Elias Alvarado-Yaah; Cristóbal Cháidez-Quiróz; Célida Duque Molina; Célida Martínez- Rodríguez; Daniel Fregoso-Rueda; Daniel Lira Morales; Eduardo Becerril-Vargas; Fernando Fontove-Herrera; Fidencio Mejía-Nepomuceno; Francisco Pulido; Gloria Elena Espinosa-Ayala; Gloria María Molina-Salinas; Gloria Vazquez; Hector Esteban Paz-Juárez; Hector Montoya-Fuentes; Helen Haydee Fernanda Ramírez-Plascencia; Irvin González-López; Jean Pierre González; Jesús Hernández; Joel Armando Vázquez-Pérez.; Jorge Salas-Hernández; José Antonio Enciso-Moreno; José Arturo Martínez-Orozco; José Esteban Muñoz-Medina; José de Jesús Nuñez-Contreras; Juan Bautista Chale-Dzul; Julissa Enciso-Ibarra; Luis Alberto Ochoa-Carrera; Margarita Matías-Florentino; Mario Mújica-Sánchez; Marissa Perez-Garcia; María Guadalupe Santiago-Mauricio; María Guadalupe de Jesús Mireles-Rivera; Nelly Sélem-Mojica; Pavel Isa; Ricardo Ciria Merce; Ricardo Grande; Rosa María Gutiérrez Rios; Santiago Ávila-Ríos; Selene Zárate; Susana Lopez; Verónica Mata-Haro; Víctor Eduardo García-Arias; Víctor Hugo Borja-Aburto
EPI_ISL_3242687, EPI_ISL_3242691, EPI_ISL_3242693, EPI_ISL_3242696, EPI_ISL_3242700, EPI_ISL_3242706, EPI_ISL_3242707, EPI_ISL_3242708, EPI_ISL_3242709, EPI_ISL_3242710, EPI_ISL_3242712, EPI_ISL_3242714, EPI_ISL_3242716, EPI_ISL_3242718, EPI_ISL_3242726, EPI_ISL_3242743			
see above	Clinica INDISA	"Facultad de Ciencias de la Vida, UNAB"	"Claudio Meneses; Ariel Orellana"; Claudio Olmos; Daniel Leon; Dayan Sanhueza; Eduardo Castro; Gonzalo Campaña; Macarena Bastias; Paola Pidal; Ricardo Yusta; Sebastian Wolter; Susana Saez; Víctor Monreal; Waldo Diaz
EPI_ISL_2988680, EPI_ISL_3025679, EPI_ISL_3025685	Clinical Microbiology, Infection Prevention and Control	Section for Molecular Diagnostics	Björn Hallström; Jonas Björkman
EPI_ISL_2466606, EPI_ISL_2610956	Clinical Virology	Clinical Bacteriology	Adrian Egli; Alfredo Mari; Fanny Wegner; Hans Hirsch; Helena MB Seth-Smith; Julia Bielicki; Karoline Leuzinger; Manuel Battegay; Tim Roloff
EPI_ISL_2550357, EPI_ISL_2790623, EPI_ISL_2790625, EPI_ISL_2790645, EPI_ISL_2790667, EPI_ISL_2941636, EPI_ISL_3160140, EPI_ISL_3404119, EPI_ISL_3404121, EPI_ISL_3696528, EPI_ISL_3842262			
see above	Colorado Department of Public Health and Environment	Colorado Department of Public Health and Environment	Alexandria Rossheim; Diana Ir; Emily A. Travanty; Laura Bankers; Mandy Waters; Michael A. Martin; Michael Martin; Molly C. Hetherington-Rauth; Sarah Elizabeth Totten; Shannon R. Matzinger
EPI_ISL_2930338	Cotugno	TIGEM	Antonio Grimaldi Patrizia Annunziata Francesco Panariello Biancamaria Pierri Claudia Tiberio Teresa Giuliano Valentina Bouche Chiara Colantuono Maria Concetta Cuomo Denise Di Concilio Lucio Di Filippo Anna Manfredi Marcello Salvi Antonio Limone Luigi Atripaldi Pellegrino Cerino Andrea Ballabio Davide Cacchiarelli
EPI_ISL_2658982, EPI_ISL_2659116	County of San Luis Obispo Public Health Laboratory	County of San Luis Obispo Public Health Laboratory	Frances Sidhu; Katrina Erwin
EPI_ISL_3273362	Curative Labs	Curative Labs	Elias L. Salfati; Eugenia Khorosheva; George Way; J.Cesar Ignacio-Espinoza; Janet Chen; Mikhail Hanewich-Hollatz; Nabjot Sandhu; Sophia Quasem; Vladimir Slepnev; Zhiyi Xie
EPI_ISL_2941667, EPI_ISL_2941681, EPI_ISL_2941683, EPI_ISL_2941687	Curative Labs	New Mexico Department of Health Scientific Laboratory	; Anastacia Griego-Fisher; D'eldra Malone; Ellie Johnson; Jennifer Benoit; Keila Gutierrez; Linda Salazar; Ratheesh Rajan
EPI_ISL_3664275	DASA	DASA	Adriano Bonaldi; Angelica Hristov; Annelise Lopes; Bianca Cota; Cristina Oliveira; Jose Levi; Lidia Yamamoto; Paulo Pierry; Rodrigo Guarischi; Rodrigo Salazar
EPI_ISL_2612553, EPI_ISL_2612554, EPI_ISL_2612555	DOHMH Corona	New York City Public Health Laboratory	Jade Wang; et al.
EPI_ISL_2662745, EPI_ISL_2662750, EPI_ISL_2662763, EPI_ISL_2662776, EPI_ISL_3692598, EPI_ISL_3692608, EPI_ISL_3692632, EPI_ISL_3692660, EPI_ISL_3692719			
see above	DPHL	Delaware Public Health Lab	Rebecca Savage
EPI_ISL_2429493, EPI_ISL_2431024, EPI_ISL_2665775, EPI_ISL_2666225, EPI_ISL_2667603, EPI_ISL_2669007, EPI_ISL_2669330, EPI_ISL_2669458, EPI_ISL_2671258, EPI_ISL_2675490, EPI_ISL_2747517, EPI_ISL_2748057, EPI_ISL_2748372, EPI_ISL_2784618, EPI_ISL_2819380			
see above	Department of Bacteria, Parasites and Fungi, Statens Serum Institut, Copenhagen, Denmark	Statens Serum Institut Bioinformatics and Microbial Genomics	Danish Covid-19 Genome Consortium
EPI_ISL_3135153, EPI_ISL_3135891	Department of Virology and Immunology, University of Helsinki and Helsinki University Hospital, Huslab Finland	Department of Virology, Faculty of Medicine, University of Helsinki, Helsinki, Finland	Essi Korhonen; Hanna Jarva; Hanna Liimatainen; Hannimari Kallio-Kokko; Harri Kangas; Hussein Alburkat; Jenni Virtanen; Maija Lappalainen; Maija Suvanto; Olli Vapalahti; Pekka Ellonen; Phuoc Truong; Ravi Kant; Sari Hannula; Satu Kurkela; Teemu Smura
EPI_ISL_2503963	Diagnostyka	1. Academic Center for Pathomorphological and Genetic-Molecular Diagnostics ltd, Bialystok, Poland 2. National Institute of Public Health - National Institute of Hygiene, Warsaw, Poland	Anetta Sulewska; Jacek Nikliński; Janusz Dzięcioł; Joanna Kiśluk; Katarzyna Zacharczuk; Konrad Raczkowski; Magdalena Nowakowska; Małgorzata Sadkowska-Todys; Piotr Karabowicz; Piotr Majewski; Przemysław Biecek. Joanna Reszeć; Radosław Charkiewicz; Tomasz Wołkowicz
EPI_ISL_3050671, EPI_ISL_3050674, EPI_ISL_3050700, EPI_ISL_3050701, EPI_ISL_3050704, EPI_ISL_3050720, EPI_ISL_3050723, EPI_ISL_3050725, EPI_ISL_3372572, EPI_ISL_3372573, EPI_ISL_3372577, EPI_ISL_3372580, EPI_ISL_3372584			
see above	Dirección regional de salud del Callao (DIRESA-CALLAO)	Centro de Investigaciones Tecnológicas, Biomédicas y Medioambientales (CITBM)	B; Huaman; J. Alarcon; M. Cuellar; M. Ramirez; M. Sovero
EPI_ISL_2967363, EPI_ISL_2967364, EPI_ISL_2967365, EPI_ISL_2967403, EPI_ISL_2967544, EPI_ISL_2967545, EPI_ISL_3933507			
see above	Division of Emerging Infectious Diseases, Bureau of Infectious Diseases Diagnosis Control, Korea Disease Control and Prevention Agency	Division of Emerging Infectious Diseases, Bureau of Infectious Diseases Diagnosis Control, Korea Disease Control and Prevention Agency	Ae Kyung Park; Chae Young Lee; Eun-Jin Kim; Heui Man Kim; Il-Hwan Kim; Jeong-Ah Kim; Jeong-Min Kim
EPI_ISL_2405572, EPI_ISL_2405606, EPI_ISL_2406331, EPI_ISL_2475680, EPI_ISL_2475694, EPI_ISL_2475695, EPI_ISL_2475701, EPI_ISL_2610234, EPI_ISL_2610296, EPI_ISL_2610674, EPI_ISL_2610700, EPI_ISL_2610733, EPI_ISL_2610743, EPI_ISL_2672423, EPI_ISL_2672546, EPI_ISL_2672729, EPI_ISL_2673170, EPI_ISL_2673225, EPI_ISL_2787886, EPI_ISL_2787900, EPI_ISL_2787902, EPI_ISL_2787905, EPI_ISL_2787907, EPI_ISL_2862759, EPI_ISL_2862761, EPI_ISL_2862765, EPI_ISL_2862769, EPI_ISL_2981755, EPI_ISL_2981895, EPI_ISL_3136605, EPI_ISL_3136615, EPI_ISL_3137106			
see above	Dutch COVID-19 response team	National Institute for Public Health and the Environment (RIVM)	Adam Meijer; AnneMarie van den Brandt; Annelies Kroneman; Bas van der Veer; Chantal Reusken; Dennis Schmitz; Dirk Eggink; Euníce Then; Florian Zwagemaker; Harry Vennema; James Groot; Jeroen Cremer; Karim Hajji; Kim Freriks; Linda van de Nes; Lisa Wijsman; Lynn Aarts; Melissa van Tuil; Ryanne Jaarsma; Sanne Bos; Sharon van den Brink; Sjoerd Kuiling; on behalf of the national COVID-19 response team
EPI_ISL_4059813, EPI_ISL_4059814, EPI_ISL_4059815, EPI_ISL_4059816, EPI_ISL_4059817, EPI_ISL_4059818, EPI_ISL_4059819, EPI_ISL_4059820, EPI_ISL_4059821, EPI_ISL_4059822, EPI_ISL_4059823, EPI_ISL_4059824, EPI_ISL_4060090, EPI_ISL_4060091, EPI_ISL_4060093, EPI_ISL_4060094, EPI_ISL_4060095, EPI_ISL_4060103, EPI_ISL_4060106, EPI_ISL_4060107, EPI_ISL_4060109, EPI_ISL_4060110			
see above	Dynacare Manitoba	Manitoba Cadham Provincial Laboratory	David Alexander; Hilary Racher; Hongbin Li; Melissa Desaulnier; Tintu Abraham
EPI_ISL_3215320, EPI_ISL_3215323, EPI_ISL_3215815, EPI_ISL_3475847, EPI_ISL_3475851	EXCITE Lab	Andersen lab at Scripps Research	Abigail Schnapper; Angela Scioscia; Araz Majnoonian; Ashkan Hassani; Chip Schooley; David Pride; Fitri Carrissa Wijaya; Greg Humphrey; Helena Tubb; Laura Kohn; Marlene Flores; Megan Nguyen; Natasha Martin Cheryl Anderson; Patricia Gonzalez-Zuniga; Rebecca Fielding-Miller; Rodolfo Salido; Sawyer Farmer; Sharon Reed; Smruthi Karthikeyan; Tommy Valles + SEARCH; Victor Cantu; Vinton Omaleki
EPI_ISL_3718929	Emory Molecular Diagnostics Laboratory, Emory Healthcare	Piantadosi Lab, Emory Department of Pathology	Ahmed Babiker; Anne Piantadosi; Ludy Registre Carmola
EPI_ISL_3246302	Eurofins Diatherix	Hudsonalpha Genome Sequencing Center	Ada Stewart; Jane Grimwood; Jenell Webber; Jennifer Cart; John Lovell; Joshua Stough; Leslie Malone; Lori H. Handley; Melissa Williams; Stefan Brzezinski; Teresa Jones; and Jeremy Schmutz
EPI_ISL_2634510, EPI_ISL_2634515, EPI_ISL_2634522, EPI_ISL_2634526, EPI_ISL_2634534, EPI_ISL_2634536, EPI_ISL_2634551, EPI_ISL_2634563, EPI_ISL_2634567, EPI_ISL_2634585, EPI_ISL_2634605, EPI_ISL_2634610, EPI_ISL_2634612, EPI_ISL_2634619, EPI_ISL_2634652, EPI_ISL_2634667, EPI_ISL_2634669, EPI_ISL_2634687			
see above	Eurofins LifeCodexx GmbH	Robert Koch Institute	
EPI_ISL_2494267, EPI_ISL_2494269, EPI_ISL_2494271, EPI_ISL_2494272, EPI_ISL_2494302	FACULDADE DE MEDICINA ABC	Instituto Butantan	Antonio Jorge Martins; Claudia Renata dos Santos Barros; David Schlesinger; Debora Botequio Moretti; Dimas Tadeu Covas; Elaine Cristina Marqueze; Elaine Vieira Santos; Evandra Strazza Rodrigues; Heidge Fukumasu; Jayme Augusto de Souza-Neto; José Salvatore Leister Patané; Luiz Alcantara; Luiz Lehmann Coutinho; Maria Carolina Elias; Maurício Lacerda Nogueira; Rafael dos Santos Bezerra; Raul Machado Neto; Rejane Maria Tommasini Grotto; Ricardo Haddad; Sandra Coccuzzo Sampaio Vessoni; Simone Kashima; Svetoslav Nanev Slavov; Vincent Louis Viala
EPI_ISL_2840301	FL Bur. of Public Health Laboratories-Jacksonville	Centers for Disease Control and Prevention Division of Viral Diseases, Pathogen Discovery	Alison Laufer Halpin; Ben L. Rambo-Martin; Clinton R. Paden; Dakota Howard; Darlene Wagner; Dave Wentworth; Dhwani Batra; Jasmine Padilla; Justin Lee; Katie Dillon; Krista Queen; Kristen Knipe; Kristine Lacek; Mark Burroughs; Matthew Schmerer; Mili Sheth; Peter Cook; Sam Shepard; Sarah Nobles; Shoshona Le; Suxiang Tong; Vivien Dugan; Yvette Unoarumhi
EPI_ISL_2840425	FL Bureau of Public Health Laboratories-Miami	Centers for Disease Control and Prevention Division of Viral Diseases, Pathogen Discovery	Alison Laufer Halpin; Ben L. Rambo-Martin; Clinton R. Paden; Dakota Howard; Darlene Wagner; Dave Wentworth; Dhwani Batra; Jasmine Padilla; Justin Lee; Katie Dillon; Krista Queen; Kristen Knipe; Kristine Lacek; Mark Burroughs; Matthew Schmerer; Mili Sheth; Peter Cook; Sam Shepard; Sarah Nobles; Shoshona Le; Suxiang Tong; Vivien Dugan; Yvette Unoarumhi
EPI_ISL_2787209, EPI_ISL_3355380	FL Bureau of Public Health Laboratories-Tampa	Centers for Disease Control and Prevention Division of Viral Diseases, Pathogen Discovery	Alex Burgin; Alison Laufer Halpin; Ben L. Rambo-Martin; Ben Rambo-Martin; Clinton Paden; Clinton R. Paden; Dakota Howard; Darlene Wagner; Dave Wentworth; Dhwani Batra; Jasmine Padilla; Justin Lee; Katie Dillon; Krista Queen; Kristen Knipe; Kristine Lacek; Mark Burroughs; Matthew Schmerer; Meghan Bentz; Mili Sheth; Peter Cook; Sam Shepard; Sarah Nobles; Shoshona Le; Suxiang Tong; Vivien Dugan; Yvette Unoarumhi
EPI_ISL_2919148, EPI_ISL_2919158	Fakultní nemocnice Hradec Králové	Fakultní nemocnice Hradec Králové	Helena Kovarikova; Marketa Gancarcikova
EPI_ISL_3230593	Fimlab Laboratoriot Oy Tampere	Expert Microbiology, National	Carita Savolainen-Kopra; Erika Lindh; Haider al-Hello; Jani Halkilahti; Kirsi Liitsola; Niina Ikonen; Olli Vapalahti; Pekka Ellonen; Phuoc Truong; Päivi Laurila; Ravi Kant; Sari Hannula; Soile Blomqvist; Teemu Smura

		Institute for Health and Welfare	
EPI_ISL_2567245, EPI_ISL_2567246, EPI_ISL_2567247, EPI_ISL_2836332, EPI_ISL_2836414, EPI_ISL_2836488, EPI_ISL_2836489, EPI_ISL_2836498, EPI_ISL_2836505, EPI_ISL_2836511, EPI_ISL_2836551, EPI_ISL_2836643, EPI_ISL_2836736, EPI_ISL_2836738, EPI_ISL_2836800, EPI_ISL_2836841, EPI_ISL_2836844, EPI_ISL_2920458, EPI_ISL_2920461, EPI_ISL_2920464, EPI_ISL_2920466, EPI_ISL_2920491, EPI_ISL_2920531, EPI_ISL_2920544, EPI_ISL_2920550, EPI_ISL_2920560, EPI_ISL_2920573, EPI_ISL_2920575, EPI_ISL_2920590, EPI_ISL_2920611, EPI_ISL_2920613, EPI_ISL_2920620, EPI_ISL_2920623, EPI_ISL_2920624, EPI_ISL_2920625, EPI_ISL_2920627, EPI_ISL_2920630, EPI_ISL_2920676, EPI_ISL_3100753, EPI_ISL_3100765, EPI_ISL_3100776, EPI_ISL_3100829, EPI_ISL_3100840, EPI_ISL_3100847, EPI_ISL_3100889, EPI_ISL_3100902, EPI_ISL_3100952, EPI_ISL_3100959, EPI_ISL_3100969, EPI_ISL_3100971, EPI_ISL_3903285			
see above	Florida Bureau of Public Health Laboratories	Florida Bureau of Public Health Laboratories	Jason Blanton; Jiaqi Li; Namratha Tarigopula; Sarah Schmedes
EPI_ISL_2663252	Fondazione IRCCS Ca' Granda Ospedale Maggiore Policlinico	Fondazione IRCCS Ca' Granda Ospedale Maggiore Policlinico	Ferruccio Ceriotti; Sara Uceda Renteria
EPI_ISL_2371530, EPI_ISL_2371531, EPI_ISL_2371548, EPI_ISL_2441851, EPI_ISL_2441852, EPI_ISL_2441875, EPI_ISL_2441877, EPI_ISL_2441886, EPI_ISL_2441904, EPI_ISL_2441907, EPI_ISL_2441935, EPI_ISL_2479997, EPI_ISL_2479998, EPI_ISL_2480036, EPI_ISL_2480099, EPI_ISL_2480108, EPI_ISL_2480143, EPI_ISL_2525669, EPI_ISL_2525727, EPI_ISL_2525729, EPI_ISL_2527565, EPI_ISL_2527735, EPI_ISL_2527753, EPI_ISL_2527816, EPI_ISL_2598704, EPI_ISL_2598705, EPI_ISL_2598726, EPI_ISL_2598733, EPI_ISL_2598791, EPI_ISL_2599922, EPI_ISL_2686408, EPI_ISL_2686431, EPI_ISL_2686449, EPI_ISL_2686452, EPI_ISL_2686486, EPI_ISL_2686496, EPI_ISL_2686554, EPI_ISL_2686555, EPI_ISL_2686561, EPI_ISL_2686573, EPI_ISL_2686609, EPI_ISL_2686623, EPI_ISL_2686651, EPI_ISL_2686717, EPI_ISL_2686720, EPI_ISL_2686732, EPI_ISL_2785548, EPI_ISL_2785551, EPI_ISL_2785592, EPI_ISL_2785601, EPI_ISL_2785636, EPI_ISL_2785644, EPI_ISL_2785648, EPI_ISL_2785680, EPI_ISL_2785681, EPI_ISL_2785737, EPI_ISL_2785858, EPI_ISL_2785861, EPI_ISL_2785872, EPI_ISL_2785912, EPI_ISL_2785940, EPI_ISL_2873401, EPI_ISL_2873586, EPI_ISL_2873631, EPI_ISL_2873692, EPI_ISL_2873762, EPI_ISL_2873765, EPI_ISL_2873796, EPI_ISL_2873808, EPI_ISL_2928558, EPI_ISL_2928640, EPI_ISL_2928678, EPI_ISL_2928681, EPI_ISL_2929136, EPI_ISL_2929138, EPI_ISL_2929152, EPI_ISL_2929153, EPI_ISL_2929158, EPI_ISL_3016511, EPI_ISL_3016654, EPI_ISL_3065030, EPI_ISL_3208204, EPI_ISL_3208320, EPI_ISL_3208332, EPI_ISL_3208819, EPI_ISL_3299864, EPI_ISL_3299867, EPI_ISL_3299871, EPI_ISL_3299967, EPI_ISL_3300021			
see above	Fulgent Genetics	Centers for Disease Control and Prevention Division of Viral Diseases, Pathogen Discovery	Adrian Paskey; Becky Tsai; Benafsh Sapra; Benjamin Rambo-Martin; Christopher Gulvick; Clinton R. Paden; Dakota Howard; Darlene Wagner; Dhwani Batra; Doreen Ng; Duncan MacCannell; Harry Gao; James Xie; Jason Caravas; John Gao; Joseph Fierro; Kara Moser; Matthew Schmerer; Mickey Li; Peter W. Cook; Scott Sammons; Shatavia Morrison; Yan Meng; Yvette Unoarumhi
EPI_ISL_2644693	Furst Medical Laboratory	Norwegian Institute of Public Health, Department of Virology	Atiya R Ali; Debech Nadia; Engebretsen Serina Beate; Garcia Llorente Ignacio; Hilde Elshaug; Hilde Vollan; Jon Bråte; Kamilla Heddeland Instefjord; Karoline Bragstad; Kathrine Stene-Johansen; Line Victoria Moen; Marie Paulsen Madsen; Olav Hungnes; Pedersen Benedikte Nevjen; Rasmus Riis Kopperud
EPI_ISL_2645769	GA Department of Public Health	GA Department of Public Health	Aliyah Fields; Cynthia Dixey; Jonathan Edwards; Stacy Reeves; Taylor Smith; Tonia Parrott
EPI_ISL_2690029, EPI_ISL_3128097, EPI_ISL_3800440, EPI_ISL_3800483	GA Department of Public Health Laboratory	Centers for Disease Control and Prevention Division of Viral Diseases, Pathogen Discovery	Alex Burgin; Alison Laufer Halpin; Ben L. Rambo-Martin; Ben Rambo-Martin; Clinton Paden; Clinton R. Paden; Dakota Howard; Darlene Wagner; Dave Wentworth; Dhwani Batra; Jasmine Padilla; Justin Lee; Katie Dillon; Krista Queen; Kristen Knipe; Kristine Lacek; Mark Burroughs; Matthew Schmerer; Meghan Bentz; Mili Sheth; Peter Cook; Sam Shepard; Sarah Nobles; Shoshona Le; Suxiang Tong; Vivien Dugan; Yvette Unoarumhi
EPI_ISL_2689598, EPI_ISL_2754920	GA Department of Public Health Laboratory	Genomics and Discovery, Respiratory Viruses Branch, Division of Viral Diseases, Centers for Disease Control and Prevention	Adam Retchless; Anna Kelleher; Anna Uehara; Brian Lynch; Clinton R. Paden; Dhwani Batra; Haibin Wang; Han Jia Justin Ng; Jasmine Padilla; Jing Zhang; Justin Lee; Krista Queen; Mark Burroughs; Mili Sheth; Morgan Davis; Peter Cook; Rachel Marine; Sarah Nobles; Suxiang Tong; Tara Coalter; Yan Li; Ying Tao
EPI_ISL_3031058	GLENS FALLS HOSPITAL LABORATORY	Wadsworth Center, New York State Department of Health	Alexis Russell; Catharine Prussing; Daryl M. Lamson; Erasmus Schneider; Erica Lasek-Nesselquist; John Kelly; Jonathan Plitnick; Kirsten St. George; Matthew Shudt; Melissa A Leisner; Navjot Singh
EPI_ISL_2493943	GRUPO TECNICO DE VIGILANCIA SANITARIA E EPIDEMIOLOGICA	Instituto Butantan	Antonio Jorge Martins; Claudia Renata dos Santos Barros; David Schlesinger; Debora Botequio Moretti; Dimas Tadeu Covas; Elaine Cristina Marqueze; Elaine Vieira Santos; Evandra Strazza Rodrigues; Heidge Fukumasu; Jayme Augusto de Souza-Neto; José Salvatore Leister Patané; Luiz Alcantara; Luiz Lehmann Coutinho; Maria Carolina Elias; Maurício Lacerda Nogueira; Rafael dos Santos Bezerra; Raul Machado Neto; Rejane Maria Tommasini Grotto; Ricardo Haddad; Sandra Coccuzzo Sampaio Vessoni; Simone Kashima; Svetoslav Nanev Slavov; Vincent Louis Viala
EPI_ISL_2543582, EPI_ISL_2543616, EPI_ISL_2543627, EPI_ISL_2543630, EPI_ISL_2543631, EPI_ISL_2543632, EPI_ISL_2543633, EPI_ISL_2543634, EPI_ISL_2543635, EPI_ISL_2543636, EPI_ISL_2543638, EPI_ISL_2543642, EPI_ISL_2543643, EPI_ISL_2543644, EPI_ISL_2543645, EPI_ISL_2543646, EPI_ISL_2543647, EPI_ISL_2543649, EPI_ISL_2543661, EPI_ISL_2543665, EPI_ISL_2557255, EPI_ISL_2557256, EPI_ISL_2557258, EPI_ISL_2557262, EPI_ISL_2557269, EPI_ISL_2557279, EPI_ISL_2557283, EPI_ISL_2557284, EPI_ISL_2557294, EPI_ISL_2557300, EPI_ISL_2659117, EPI_ISL_2659122, EPI_ISL_2659126, EPI_ISL_2659128, EPI_ISL_2659132, EPI_ISL_2659136, EPI_ISL_2659157, EPI_ISL_2659167, EPI_ISL_2659171, EPI_ISL_2659178, EPI_ISL_2659179, EPI_ISL_2659189, EPI_ISL_2659191, EPI_ISL_2659192, EPI_ISL_2659193, EPI_ISL_2663430, EPI_ISL_2663442, EPI_ISL_2663444, EPI_ISL_2663449, EPI_ISL_2663452, EPI_ISL_2663560, EPI_ISL_2663562, EPI_ISL_2663563, EPI_ISL_2663565, EPI_ISL_2663568, EPI_ISL_2663574, EPI_ISL_2663576, EPI_ISL_2663580, EPI_ISL_2663582, EPI_ISL_2663583, EPI_ISL_2663587, EPI_ISL_2663592, EPI_ISL_2663601, EPI_ISL_2663607, EPI_ISL_2663616, EPI_ISL_2756060, EPI_ISL_2756061, EPI_ISL_2756066, EPI_ISL_2756069, EPI_ISL_2756094, EPI_ISL_2756095, EPI_ISL_2756097, EPI_ISL_2756108, EPI_ISL_2756109, EPI_ISL_2756112, EPI_ISL_2756114, EPI_ISL_2756116, EPI_ISL_2756122, EPI_ISL_2756124, EPI_ISL_2756126, EPI_ISL_2756128, EPI_ISL_2756130, EPI_ISL_2756141, EPI_ISL_2756144, EPI_ISL_2756148, EPI_ISL_2756149, EPI_ISL_2756150, EPI_ISL_2756157, EPI_ISL_2756166, EPI_ISL_2756178, EPI_ISL_2756179, EPI_ISL_2756183, EPI_ISL_2756184, EPI_ISL_2756187, EPI_ISL_2756188, EPI_ISL_2756189, EPI_ISL_2756190, EPI_ISL_2756195, EPI_ISL_2756230, EPI_ISL_2756234, EPI_ISL_2756240, EPI_ISL_2756241, EPI_ISL_2756245, EPI_ISL_2756253, EPI_ISL_2756269, EPI_ISL_2756274, EPI_ISL_2756276, EPI_ISL_2756282, EPI_ISL_2756283, EPI_ISL_2756287, EPI_ISL_2756295, EPI_ISL_2756296, EPI_ISL_2756297, EPI_ISL_2756309, EPI_ISL_2756311, EPI_ISL_2756317, EPI_ISL_2756327, EPI_ISL_2756331, EPI_ISL_2756335, EPI_ISL_2756338, EPI_ISL_2892596, EPI_ISL_2892598, EPI_ISL_2892609, EPI_ISL_2892610, EPI_ISL_2892612, EPI_ISL_2892627, EPI_ISL_2892630, EPI_ISL_2892631, EPI_ISL_2892632, EPI_ISL_2892636, EPI_ISL_2892638, EPI_ISL_2892639, EPI_ISL_2892642, EPI_ISL_2892643, EPI_ISL_2892645, EPI_ISL_2892646, EPI_ISL_2892652, EPI_ISL_2892655, EPI_ISL_2892660, EPI_ISL_2892664, EPI_ISL_2892667, EPI_ISL_2892668, EPI_ISL_2892673, EPI_ISL_2892675, EPI_ISL_2892678, EPI_ISL_2892692, EPI_ISL_2892727, EPI_ISL_2892729, EPI_ISL_2892731, EPI_ISL_2892741, EPI_ISL_2892746, EPI_ISL_2892747, EPI_ISL_2892755, EPI_ISL_2892757, EPI_ISL_2892761, EPI_ISL_2892762, EPI_ISL_2892777, EPI_ISL_2892780, EPI_ISL_2892792, EPI_ISL_2892821, EPI_ISL_2892823, EPI_ISL_2892825, EPI_ISL_2892828, EPI_ISL_2892830, EPI_ISL_2892831, EPI_ISL_2892851, EPI_ISL_2892854, EPI_ISL_2892856, EPI_ISL_2892857, EPI_ISL_2892858, EPI_ISL_2892860, EPI_ISL_2892862, EPI_ISL_2892865, EPI_ISL_2892866, EPI_ISL_2892868, EPI_ISL_2892870, EPI_ISL_2892871, EPI_ISL_2892878, EPI_ISL_2892879, EPI_ISL_2892882, EPI_ISL_2892885, EPI_ISL_2892891, EPI_ISL_2892894, EPI_ISL_2892898, EPI_ISL_2892900, EPI_ISL_2892901, EPI_ISL_2892908, EPI_ISL_2892921, EPI_ISL_2892922, EPI_ISL_2892923, EPI_ISL_2892924, EPI_ISL_2892925, EPI_ISL_2892930, EPI_ISL_2892931, EPI_ISL_2892936, EPI_ISL_2892946, EPI_ISL_2892951, EPI_ISL_2892959, EPI_ISL_2892961, EPI_ISL_2892964, EPI_ISL_2892965, EPI_ISL_2892967, EPI_ISL_2892968, EPI_ISL_2892969, EPI_ISL_2892970, EPI_ISL_2892971, EPI_ISL_2892972, EPI_ISL_2892973, EPI_ISL_2892974, EPI_ISL_2892975, EPI_ISL_2892976, EPI_ISL_2892978, EPI_ISL_2892980, EPI_ISL_2892982, EPI_ISL_2892993, EPI_ISL_2892995, EPI_ISL_2893001, EPI_ISL_2893003, EPI_ISL_2893004, EPI_ISL_2893006, EPI_ISL_2893009, EPI_ISL_2893010, EPI_ISL_2893013, EPI_ISL_2893014, EPI_ISL_2893015, EPI_ISL_2893016, EPI_ISL_2893017, EPI_ISL_2893018, EPI_ISL_2894896, EPI_ISL_2894897, EPI_ISL_2894898, EPI_ISL_2894899, EPI_ISL_2894905, EPI_ISL_2894906, EPI_ISL_2894916, EPI_ISL_2894917, EPI_ISL_2894918, EPI_ISL_2894919, EPI_ISL_2894924, EPI_ISL_2894928, EPI_ISL_2894929, EPI_ISL_2894930, EPI_ISL_2894931, EPI_ISL_2894938, EPI_ISL_2894947, EPI_ISL_2894952, EPI_ISL_2894960, EPI_ISL_2894963, EPI_ISL_2894967, EPI_ISL_2894970, EPI_ISL_2896150, EPI_ISL_2896151, EPI_ISL_2896153, EPI_ISL_2896154, EPI_ISL_2896160, EPI_ISL_2896162, EPI_ISL_2896165, EPI_ISL_2896169, EPI_ISL_2896170, EPI_ISL_2896172, EPI_ISL_2896173, EPI_ISL_2896174, EPI_ISL_2896175, EPI_ISL_2896176, EPI_ISL_2896179, EPI_ISL_2896181, EPI_ISL_2930489, EPI_ISL_2930492, EPI_ISL_2930495, EPI_ISL_2930501, EPI_ISL_2930502, EPI_ISL_2930504, EPI_ISL_2930512, EPI_ISL_2930513, EPI_ISL_2930514, EPI_ISL_2930515, EPI_ISL_2930516, EPI_ISL_2930517, EPI_ISL_2930525, EPI_ISL_2930526, EPI_ISL_2930527, EPI_ISL_2930529, EPI_ISL_2930531, EPI_ISL_2930532, EPI_ISL_2930533, EPI_ISL_2930536, EPI_ISL_2930550, EPI_ISL_2930552, EPI_ISL_3132174, EPI_ISL_3132175, EPI_ISL_3132177, EPI_ISL_3132188, EPI_ISL_3132197, EPI_ISL_3132199, EPI_ISL_3132200, EPI_ISL_3132211, EPI_ISL_3132212, EPI_ISL_3132213, EPI_ISL_3132216, EPI_ISL_3132238, EPI_ISL_3132240, EPI_ISL_3132241, EPI_ISL_3132242, EPI_ISL_3132266, EPI_ISL_3132268, EPI_ISL_3132270, EPI_ISL_3132642, EPI_ISL_3132643, EPI_ISL_3132645, EPI_ISL_3132646, EPI_ISL_3132647, EPI_ISL_3132650, EPI_ISL_3132659, EPI_ISL_3132661, EPI_ISL_3132679, EPI_ISL_3132680, EPI_ISL_3132681, EPI_ISL_3132682, EPI_ISL_3132684, EPI_ISL_3132685, EPI_ISL_3132691, EPI_ISL_3132692, EPI_ISL_3132695, EPI_ISL_3132696, EPI_ISL_3144905, EPI_ISL_3144907, EPI_ISL_3144909, EPI_ISL_3144912, EPI_ISL_3144916, EPI_ISL_3144923, EPI_ISL_3144927, EPI_ISL_3144931, EPI_ISL_3144932, EPI_ISL_3149082, EPI_ISL_3149084, EPI_ISL_3149087, EPI_ISL_3149090, EPI_ISL_3149091, EPI_ISL_3149094, EPI_ISL_3149095, EPI_ISL_3149098, EPI_ISL_3149099, EPI_ISL_3149101, EPI_ISL_3149102, EPI_ISL_3149118, EPI_ISL_3149120, EPI_ISL_3149124, EPI_ISL_3149126, EPI_ISL_3185388, EPI_ISL_3185389, EPI_ISL_3185391, EPI_ISL_3185394, EPI_ISL_3185400, EPI_ISL_3185402, EPI_ISL_3185409, EPI_ISL_3185410, EPI_ISL_3185411, EPI_ISL_3185417, EPI_ISL_3185419, EPI_ISL_3185421, EPI_ISL_3185425, EPI_ISL_3185426, EPI_ISL_3185428, EPI_ISL_3185429, EPI_ISL_3185432, EPI_ISL_3185433, EPI_ISL_3185434, EPI_ISL_3185435, EPI_ISL_3185436, EPI_ISL_3185453, EPI_ISL_3185458, EPI_ISL_3185464, EPI_ISL_3185469, EPI_ISL_3185472, EPI_ISL_3185483, EPI_ISL_3185484, EPI_ISL_3185486, EPI_ISL_3185487, EPI_ISL_3185489, EPI_ISL_3185492, EPI_ISL_3185494, EPI_ISL_3185495, EPI_ISL_3185496, EPI_ISL_3185502, EPI_ISL_3185503, EPI_ISL_3185507, EPI_ISL_3185508, EPI_ISL_3185510, EPI_ISL_3185513, EPI_ISL_3185522, EPI_ISL_3185528, EPI_ISL_3185539, EPI_ISL_3185542, EPI_ISL_3185545, EPI_ISL_3185546, EPI_ISL_3185547, EPI_ISL_3185548, EPI_ISL_3185549, EPI_ISL_3185550, EPI_ISL_3185551, EPI_ISL_3185555, EPI_ISL_3185557, EPI_ISL_3185566, EPI_ISL_3185569, EPI_ISL_3185572, EPI_ISL_3185583, EPI_ISL_3185587, EPI_ISL_3185591, EPI_ISL_3185593, EPI_ISL_3185601, EPI_ISL_3185602, EPI_ISL_3185610, EPI_ISL_3185612, EPI_ISL_3185613, EPI_ISL_3185614, EPI_ISL_3185619, EPI_ISL_3185621, EPI_ISL_3185624, EPI_ISL_3185625, EPI_ISL_3185640, EPI_ISL_3185641, EPI_ISL_3185672, EPI_ISL_3185678, EPI_ISL_3185699, EPI_ISL_3185705, EPI_ISL_3185706, EPI_ISL_3185708, EPI_ISL_3185717, EPI_ISL_3185719, EPI_ISL_3185720, EPI_ISL_3185730, EPI_ISL_3185770, EPI_ISL_3220038, EPI_ISL_3220043, EPI_ISL_3220045, EPI_ISL_3220047, EPI_ISL_3220058, EPI_ISL_3220065, EPI_ISL_3220068, EPI_ISL_3220074, EPI_ISL_3220075, EPI_ISL_3220077, EPI_ISL_3220087, EPI_ISL_3220090, EPI_ISL_3220098, EPI_ISL_3220101, EPI_ISL_3220129, EPI_ISL_3220142, EPI_ISL_3220143, EPI_ISL_3358536, EPI_ISL_3358538, EPI_ISL_3358540, EPI_ISL_3358562, EPI_ISL_3358566, EPI_ISL_3358571, EPI_ISL_3358572, EPI_ISL_3368885, EPI_ISL_3368887, EPI_ISL_3368888, EPI_ISL_3368889, EPI_ISL_3368891, EPI_ISL_3368896, EPI_ISL_3368897, EPI_ISL_3369339, EPI_ISL_3369340, EPI_ISL_3369386, EPI_ISL_3981972, EPI_ISL_3981976			
see above	Genetica Molecular and Subdepartamento de Virologia ISP Chile	Instituto de Salud Publica de Chile	Andres Castillo; Barbara Parra; Constanza Campano; Gisselle Barra; Javier Tognarelli; Jorge Fernandez; Karen Orostica; Loredana Arata; Patricia Bustos; Rodrigo Fasce; Soledad Ulloa
EPI_ISL_2621554	Genomics, Poplar Healthcare	Genomics, Poplar Healthcare	Truong, M.
EPI_ISL_4054613, EPI_ISL_4054615	Grady Memorial Hospital, Clinical Microbiology Laboratory	Piantadosi Lab, Emory Department of Pathology	Anne Piantadosi; Ludy Registre Carmola; Yun F (Wayne) Wang
EPI_ISL_2537141, EPI_ISL_2537146, EPI_ISL_2537202, EPI_ISL_2537203, EPI_ISL_2537207, EPI_ISL_2537217, EPI_ISL_2537219, EPI_ISL_2537233, EPI_ISL_2854941, EPI_ISL_2854951, EPI_ISL_2854955, EPI_ISL_2854961, EPI_ISL_2985641, EPI_ISL_2985642, EPI_ISL_2985664, EPI_ISL_2985665, EPI_ISL_2985668, EPI_ISL_2985714, EPI_ISL_2985761			
see above	Gravity Diagnostics, LLC	Gravity Diagnostics, LLC	Gravity Diagnostics
EPI_ISL_2376254, EPI_ISL_2376255, EPI_ISL_2501001	Gundersen Clinical Microbiology Laboratory	Kabara Cancer Research Institute	Craig S. Richmond; Paraic A. Kenny
EPI_ISL_2695275	H Braga	Instituto Nacional de Saude (INSA)	Borges et al
EPI_ISL_3102422, EPI_ISL_3536150, EPI_ISL_3536363	H J M A HOSPITAL JOSE MARTINIANO DE ALENCAR	Analytical Competence Molecular Epidemiology Lab/ACME, Oswaldo Cruz Foundation, Ceara (FIOCRUZ CE)	Cleber Furtado Aksenen; Fabio Miyajima; Fernando Braga Stehling; Francisco Eder de Moura Lopes; Jamille Maria Mendes Bezerra; Joaquim César do Nascimento Sousa Junior; Pedro Miguel Carneiro Jeronimo; Suzana Porto Almeida e Lucas Delerino; Thais Ferreira de Oliveira; Thais de Oliveira Costa; Ticiane Cavalcante de Souza; Veridiana Pessoa Miyajima
EPI_ISL_3102348, EPI_ISL_3102356, EPI_ISL_3102359, EPI_ISL_3102427, EPI_ISL_3536210, EPI_ISL_3536213	HEMOCE CENTRO DE HEMATOLOGIA E HEMOTERAPIA DO CEARA	Analytical Competence Molecular Epidemiology Lab/ACME, Oswaldo Cruz Foundation, Ceara (FIOCRUZ CE)	Cleber Furtado Aksenen; Fabio Miyajima; Fernando Braga Stehling; Francisco Eder de Moura Lopes; Jamille Maria Mendes Bezerra; Joaquim César do Nascimento Sousa Junior; Pedro Miguel Carneiro Jeronimo; Suzana Porto Almeida e Lucas Delerino; Thais Ferreira de Oliveira; Thais de Oliveira Costa; Ticiane Cavalcante de Souza; Veridiana Pessoa Miyajima
EPI_ISL_3912162	HEMOCE DE SOBRAL	Analytical Competence Molecular Epidemiology Lab/ACME, Oswaldo Cruz Foundation, Ceara (FIOCRUZ CE)	Cleber Furtado Aksenen; Fabio Miyajima; Fernando Braga Stehling; Francisco Eder de Moura Lopes; Jamille Maria Mendes Bezerra; Joaquim Cesar do Nascimento Sousa Junior; Pedro Miguel Carneiro Jeronimo; Suzana Porto Almeida & Lucas Delerino on behalf of COVID-19 FIOCRUZ Genomic Network; Thais Ferreira de Oliveira; Thais de Oliveira Costa; Ticiane Cavalcante de Souza; Veridiana Pessoa Miyajima
EPI_ISL_3550432, EPI_ISL_3550434, EPI_ISL_3550440	HG LAB TRUCK GmbH	Institute of Virology Department of Hygiene, Microbiology and Public Health at Innsbruck Medical University	Andre Volland; Annika Roessler; Barbara Falkensammer; David Bante; Dorothee von Laer; Heribert Stoiber; Janine Kimpel; Lisa Pipperger; Lydia Riepler; Wegene Borena
EPI_ISL_3102339, EPI_ISL_3102345, EPI_ISL_3102352, EPI_ISL_3102381, EPI_ISL_3102392, EPI_ISL_3102397, EPI_ISL_3536166, EPI_ISL_3536214			
see above	HGCC HOSPITAL GERAL DR CESAR CALS	Analytical Competence Molecular Epidemiology Lab/ACME, Oswaldo Cruz Foundation, Ceara (FIOCRUZ CE)	Cleber Furtado Aksenen; Fabio Miyajima; Fernando Braga Stehling; Francisco Eder de Moura Lopes; Jamille Maria Mendes Bezerra; Joaquim César do Nascimento Sousa Junior; Pedro Miguel Carneiro Jeronimo; Suzana Porto Almeida e Lucas Delerino; Thais Ferreira de Oliveira; Thais de Oliveira Costa; Ticiane Cavalcante de Souza; Veridiana Pessoa Miyajima
EPI_ISL_3102337, EPI_ISL_3102340, EPI_ISL_3102347, EPI_ISL_3102357, EPI_ISL_3102361, EPI_ISL_3102363, EPI_ISL_3102378, EPI_ISL_3102398, EPI_ISL_3102401, EPI_ISL_3536206, EPI_ISL_3536216, EPI_ISL_3536218, EPI_ISL_3536219, EPI_ISL_3536220, EPI_ISL_3536221			
see above	HIAS HOSPITAL INFANTIL ALBERT SABIN	Analytical Competence Molecular Epidemiology Lab/ACME, Oswaldo Cruz Foundation, Ceara (FIOCRUZ CE)	Cleber Furtado Aksenen; Fabio Miyajima; Fernando Braga Stehling; Francisco Eder de Moura Lopes; Jamille Maria Mendes Bezerra; Joaquim César do Nascimento Sousa Junior; Pedro Miguel Carneiro Jeronimo; Suzana Porto Almeida e Lucas Delerino; Thais Ferreira de Oliveira; Thais de Oliveira Costa; Ticiane Cavalcante de Souza; Veridiana Pessoa Miyajima
EPI_ISL_2497443, EPI_ISL_2497444, EPI_ISL_2497445, EPI_ISL_2497446, EPI_ISL_2497447, EPI_ISL_2497449, EPI_ISL_2497450, EPI_ISL_2497451, EPI_ISL_2497452, EPI_ISL_2497453, EPI_ISL_2497455, EPI_ISL_2497457, EPI_ISL_2497458, EPI_ISL_2497461, EPI_ISL_2497462, EPI_ISL_2497463, EPI_ISL_2497464, EPI_ISL_2497467, EPI_ISL_2497469, EPI_ISL_2497470, EPI_ISL_2617589, EPI_ISL_2617592, EPI_ISL_2617593, EPI_ISL_2617594, EPI_ISL_2617595, EPI_ISL_2617609, EPI_ISL_2617610, EPI_ISL_2617612, EPI_ISL_2617627, EPI_ISL_2680895, EPI_ISL_2680896, EPI_ISL_2680897, EPI_ISL_2680898, EPI_ISL_2680899, EPI_ISL_2680900, EPI_ISL_2680901, EPI_ISL_2680902, EPI_ISL_2680903, EPI_ISL_2680904, EPI_ISL_2680905, EPI_ISL_2680906, EPI_ISL_2680907, EPI_ISL_2680909, EPI_ISL_2680910, EPI_ISL_2680912, EPI_ISL_2680914, EPI_ISL_2680915, EPI_ISL_2921535, EPI_ISL_2921539, EPI_ISL_2921540, EPI_ISL_2921541, EPI_ISL_2921542, EPI_ISL_2921543, EPI_ISL_2921544, EPI_ISL_2921550, EPI_ISL_2921552, EPI_ISL_2921555, EPI_ISL_2921556, EPI_ISL_2921557, EPI_ISL_2921559, EPI_ISL_2921560, EPI_ISL_2921564, EPI_ISL_2921565, EPI_ISL_2921566, EPI_ISL_2921567, EPI_ISL_2921568, EPI_ISL_2921569, EPI_ISL_2921571, EPI_ISL_2921574, EPI_ISL_2921576, EPI_ISL_2921577, EPI_ISL_2921578, EPI_ISL_2921580, EPI_ISL_2921583, EPI_ISL_2921586, EPI_ISL_2921587, EPI_ISL_2921588, EPI_ISL_2921590, EPI_ISL_2921591, EPI_ISL_2921592, EPI_ISL_2921595, EPI_ISL_2921598, EPI_ISL_2921599, EPI_ISL_2921602, EPI_ISL_2921604, EPI_ISL_2921606, EPI_ISL_2921609, EPI_ISL_2928511, EPI_ISL_3254319, EPI_ISL_3254320, EPI_ISL_3254322, EPI_ISL_3254323, EPI_ISL_3254325, EPI_ISL_3254326, EPI_ISL_3254327, EPI_ISL_3254328, EPI_ISL_3254330, EPI_ISL_3254332, EPI_ISL_3254333, EPI_ISL_3254349, EPI_ISL_3254350, EPI_ISL_3254352, EPI_ISL_3254353, EPI_ISL_3254354, EPI_ISL_3254356, EPI_ISL_3254357, EPI_ISL_3254358, EPI_ISL_3254359, EPI_ISL_3254360, EPI_ISL_3254361,			

EPI_ISL_3254362, EPI_ISL_3254363, EPI_ISL_3254364, EPI_ISL_3254365, EPI_ISL_3254366, EPI_ISL_3254368, EPI_ISL_3254369, EPI_ISL_3254370, EPI_ISL_3254373, EPI_ISL_3254374, EPI_ISL_3254380, EPI_ISL_3254381, EPI_ISL_3254383, EPI_ISL_3254384, EPI_ISL_3254386, EPI_ISL_3254388, EPI_ISL_3254390, EPI_ISL_3254391, EPI_ISL_3254392, EPI_ISL_3254393, EPI_ISL_3254395, EPI_ISL_3254396, EPI_ISL_3254397, EPI_ISL_3254398, EPI_ISL_3254400, EPI_ISL_3254402, EPI_ISL_3254404, EPI_ISL_3254405, EPI_ISL_3254406, EPI_ISL_3254407, EPI_ISL_3254409, EPI_ISL_3254411, EPI_ISL_3254412, EPI_ISL_3254413, EPI_ISL_3254414, EPI_ISL_3254416, EPI_ISL_3254418, EPI_ISL_3254419, EPI_ISL_3254422, EPI_ISL_3254423, EPI_ISL_3254424, EPI_ISL_3254425, EPI_ISL_3254426, EPI_ISL_3254427, EPI_ISL_3254428, EPI_ISL_3254429, EPI_ISL_3254431, EPI_ISL_3254433, EPI_ISL_3254434, EPI_ISL_3254435, EPI_ISL_3254438, EPI_ISL_3254439, EPI_ISL_3254440, EPI_ISL_3254442, EPI_ISL_3254443, EPI_ISL_3254445, EPI_ISL_3254447, EPI_ISL_3254448, EPI_ISL_3254449, EPI_ISL_3254450, EPI_ISL_3254451, EPI_ISL_3254452, EPI_ISL_3254453, EPI_ISL_3254454, EPI_ISL_3254455, EPI_ISL_3254458, EPI_ISL_3254459, EPI_ISL_3254460, EPI_ISL_3254461, EPI_ISL_3254462, EPI_ISL_3254463, EPI_ISL_3254464, EPI_ISL_3254466, EPI_ISL_3254467, EPI_ISL_3254468, EPI_ISL_3254469, EPI_ISL_3254470, EPI_ISL_3254471, EPI_ISL_3254472, EPI_ISL_3254473, EPI_ISL_3254475, EPI_ISL_3254476, EPI_ISL_3254477, EPI_ISL_3254479, EPI_ISL_3254480, EPI_ISL_3254481, EPI_ISL_3254482, EPI_ISL_3254485, EPI_ISL_3254486, EPI_ISL_3254487, EPI_ISL_3254489, EPI_ISL_3254491, EPI_ISL_3254492, EPI_ISL_3254493, EPI_ISL_3254494, EPI_ISL_3254495, EPI_ISL_3254496, EPI_ISL_3254498, EPI_ISL_3254499, EPI_ISL_3254501, EPI_ISL_3254502, EPI_ISL_3254505, EPI_ISL_3254506, EPI_ISL_3254507, EPI_ISL_3254508, EPI_ISL_3254509, EPI_ISL_3254510, EPI_ISL_3254511, EPI_ISL_3254512, EPI_ISL_3254513, EPI_ISL_3254514, EPI_ISL_3254515, EPI_ISL_3254516, EPI_ISL_3254517, EPI_ISL_3254518, EPI_ISL_3254519, EPI_ISL_3254520, EPI_ISL_3254521, EPI_ISL_3254522, EPI_ISL_3254523, EPI_ISL_3254526, EPI_ISL_3254528, EPI_ISL_3254530, EPI_ISL_3254531, EPI_ISL_3254533, EPI_ISL_3254535, EPI_ISL_3254536, EPI_ISL_3254538, EPI_ISL_3254539, EPI_ISL_3254541, EPI_ISL_3254542, EPI_ISL_3254543, EPI_ISL_3254544, EPI_ISL_3254546, EPI_ISL_3254547, EPI_ISL_3254548, EPI_ISL_3254549, EPI_ISL_3254551, EPI_ISL_3254553, EPI_ISL_3254554, EPI_ISL_3254556, EPI_ISL_3254558, EPI_ISL_3254560, EPI_ISL_3254562, EPI_ISL_3254563, EPI_ISL_3254564, EPI_ISL_3254565, EPI_ISL_3254568, EPI_ISL_3254569, EPI_ISL_3254571, EPI_ISL_3254572, EPI_ISL_3254573, EPI_ISL_3254574, EPI_ISL_3254575, EPI_ISL_3254576, EPI_ISL_3254578, EPI_ISL_3254579, EPI_ISL_3254580, EPI_ISL_3254581, EPI_ISL_3254582, EPI_ISL_3254583, EPI_ISL_3254584, EPI_ISL_3254585, EPI_ISL_3254586, EPI_ISL_3254587, EPI_ISL_3254589, EPI_ISL_3254590, EPI_ISL_3254594, EPI_ISL_3254595, EPI_ISL_3254596, EPI_ISL_3254597, EPI_ISL_3254598, EPI_ISL_3254601, EPI_ISL_3254602, EPI_ISL_3254604, EPI_ISL_3254605, EPI_ISL_3254606, EPI_ISL_3254610, EPI_ISL_3254611, EPI_ISL_3254612, EPI_ISL_3254613, EPI_ISL_3254614, EPI_ISL_3254615, EPI_ISL_3254618, EPI_ISL_3254619, EPI_ISL_3254620, EPI_ISL_3254621, EPI_ISL_3254623, EPI_ISL_3254624, EPI_ISL_3254625, EPI_ISL_3254626, EPI_ISL_3254627, EPI_ISL_3254628, EPI_ISL_3254629, EPI_ISL_3254630, EPI_ISL_3254633, EPI_ISL_3254634, EPI_ISL_3254635, EPI_ISL_3254636, EPI_ISL_3254637, EPI_ISL_3254640, EPI_ISL_3254641, EPI_ISL_3254642, EPI_ISL_3254643, EPI_ISL_3254645, EPI_ISL_3254647, EPI_ISL_3254651, EPI_ISL_3254652, EPI_ISL_3254653, EPI_ISL_3254657, EPI_ISL_3254659, EPI_ISL_3254660, EPI_ISL_3254664, EPI_ISL_3254665, EPI_ISL_3254672, EPI_ISL_3254673, EPI_ISL_3254676, EPI_ISL_3254677, EPI_ISL_3254684, EPI_ISL_3259354, EPI_ISL_3299426, EPI_ISL_3299428, EPI_ISL_3299429, EPI_ISL_3386151, EPI_ISL_3386153, EPI_ISL_3386154, EPI_ISL_4061630, EPI_ISL_4061647, EPI_ISL_4061648, EPI_ISL_4061651, EPI_ISL_4061652, EPI_ISL_4061655, EPI_ISL_4061657, EPI_ISL_4061659, EPI_ISL_4061661			
see above	HLAGYN - Laboratorio de Imunologia de Transplantes de Goias	HLAGYN - Laboratorio de Imunologia de Transplantes de Goias	Alessandro Leonardo Alvares Magalhaes; Daniel Ferreira de Sousa; Danielle de Paiva Rezende; Erika Lopes Rocha Batista; Fernando Antonio Vinhal dos Santos; Frederico Rodrigues Vinhal; Kamila Oliveira Reis De Freitas.; Lucas Carlos Gomes Pereira; Sabrina Sara Moreira Duarte
EPI_ISL_3102408, EPI_ISL_3102419, EPI_ISL_3102426, EPI_ISL_3102430, EPI_ISL_3102431, EPI_ISL_3102432, EPI_ISL_3102434, EPI_ISL_3102436, EPI_ISL_3102440, EPI_ISL_3102444, EPI_ISL_3102448, EPI_ISL_3102453, EPI_ISL_3102457, EPI_ISL_3102461, EPI_ISL_3102462, EPI_ISL_3102464, EPI_ISL_3102469, EPI_ISL_3536205, EPI_ISL_3536222, EPI_ISL_3536223, EPI_ISL_3536224, EPI_ISL_3536225, EPI_ISL_3536227, EPI_ISL_3536228, EPI_ISL_3536230, EPI_ISL_3536232, EPI_ISL_3536233, EPI_ISL_3536236, EPI_ISL_3536237, EPI_ISL_3536238, EPI_ISL_3536239, EPI_ISL_3536240, EPI_ISL_3536241, EPI_ISL_3536242, EPI_ISL_3536243, EPI_ISL_3536245, EPI_ISL_3536247, EPI_ISL_3536248, EPI_ISL_3536249, EPI_ISL_3536251, EPI_ISL_3536252, EPI_ISL_3536254, EPI_ISL_3536257, EPI_ISL_3536258, EPI_ISL_3536259, EPI_ISL_3912173			
see above	HM HOSPITAL DE MESSEJANA DR CARLOS ALBERTO STUDART GOMES	Analytical Competence Molecular Epidemiology Lab/ACME, Oswaldo Cruz Foundation, Ceara (FIOCRUZ CE)	Cleber Furtado Aksenen; Fabio Miyajima; Fernando Braga Stehling; Francisco Eder de Moura Lopes; Jamille Maria Mendes Bezerra; Joaquim Cesar do Nascimento Sousa Junior; Joaquim César do Nascimento Sousa Junior; Pedro Miguel Carneiro Jeronimo; Suzana Porto Almeida & Lucas Delerino on behalf of COVID-19 FIOCRUZ Genomic Network; Suzana Porto Almeida e Lucas Delerino; Thais Ferreira de Oliveira; Thais de Oliveira Costa; Ticiane Cavalcante de Souza; Veridiana Pessoa Miyajima
EPI_ISL_3536177, EPI_ISL_3912188	HOSP DR THADEU DE PAULO BRITO	Analytical Competence Molecular Epidemiology Lab/ACME, Oswaldo Cruz Foundation, Ceara (FIOCRUZ CE)	Cleber Furtado Aksenen; Fabio Miyajima; Fernando Braga Stehling; Francisco Eder de Moura Lopes; Jamille Maria Mendes Bezerra; Joaquim Cesar do Nascimento Sousa Junior; Joaquim César do Nascimento Sousa Junior; Pedro Miguel Carneiro Jeronimo; Suzana Porto Almeida & Lucas Delerino on behalf of COVID-19 FIOCRUZ Genomic Network; Suzana Porto Almeida e Lucas Delerino; Thais Ferreira de Oliveira; Thais de Oliveira Costa; Ticiane Cavalcante de Souza; Veridiana Pessoa Miyajima
EPI_ISL_3536181, EPI_ISL_3536182, EPI_ISL_3536183, EPI_ISL_3912191	HOSP MUN ABELARDO GADELHA DA ROCHA	Analytical Competence Molecular Epidemiology Lab/ACME, Oswaldo Cruz Foundation, Ceara (FIOCRUZ CE)	Cleber Furtado Aksenen; Fabio Miyajima; Fernando Braga Stehling; Francisco Eder de Moura Lopes; Jamille Maria Mendes Bezerra; Joaquim Cesar do Nascimento Sousa Junior; Joaquim César do Nascimento Sousa Junior; Pedro Miguel Carneiro Jeronimo; Suzana Porto Almeida & Lucas Delerino on behalf of COVID-19 FIOCRUZ Genomic Network; Suzana Porto Almeida e Lucas Delerino; Thais Ferreira de Oliveira; Thais de Oliveira Costa; Ticiane Cavalcante de Souza; Veridiana Pessoa Miyajima
EPI_ISL_2493850, EPI_ISL_2493851, EPI_ISL_2493853, EPI_ISL_2493854, EPI_ISL_2493855, EPI_ISL_2493856, EPI_ISL_2493857, EPI_ISL_2493859, EPI_ISL_2493860, EPI_ISL_2493905, EPI_ISL_2493918, EPI_ISL_2493920, EPI_ISL_2493921, EPI_ISL_2493923, EPI_ISL_2493924, EPI_ISL_2493925, EPI_ISL_2493926, EPI_ISL_2493927, EPI_ISL_2493929, EPI_ISL_2493937, EPI_ISL_2493938, EPI_ISL_2493954			
see above	HOSP MUN DE MOGI DAS CRUZES PREF WALDEMAR COSTA FILHO	Instituto Butantan	Antonio Jorge Martins; Claudia Renata dos Santos Barros; David Schlesinger; Debora Botequio Moretti; Dimas Tadeu Covas; Elaine Cristina Marqueze; Elaine Vieira Santos; Evandra Strazza Rodrigues; Heidge Fukumasu; Jayme Augusto de Souza-Neto; José Salvatore Leister Patané; Luiz Alcantara; Luiz Lehmann Coutinho; Maria Carolina Elias; Maurício Lacerda Nogueira; Rafael dos Santos Bezerra; Raul Machado Neto; Rejane Maria Tommasini Grotto; Ricardo Haddad; Sandra Coccuzzo Sampaio Vessoni; Simone Kashima; Svetoslav Nanev Slavov; Vincent Louis Viala
EPI_ISL_2493941	HOSP MUN JOSANIAS CASTANHA BRAGA	Instituto Butantan	Antonio Jorge Martins; Claudia Renata dos Santos Barros; David Schlesinger; Debora Botequio Moretti; Dimas Tadeu Covas; Elaine Cristina Marqueze; Elaine Vieira Santos; Evandra Strazza Rodrigues; Heidge Fukumasu; Jayme Augusto de Souza-Neto; José Salvatore Leister Patané; Luiz Alcantara; Luiz Lehmann Coutinho; Maria Carolina Elias; Maurício Lacerda Nogueira; Rafael dos Santos Bezerra; Raul Machado Neto; Rejane Maria Tommasini Grotto; Ricardo Haddad; Sandra Coccuzzo Sampaio Vessoni; Simone Kashima; Svetoslav Nanev Slavov; Vincent Louis Viala
EPI_ISL_2812743	HOSPITAL CAN MISSES	HOSPITAL UNIVERSITARIO SON ESPASES	Hospital Universitario Son Espases; Servicio de Microbiología
EPI_ISL_3102285	HOSPITAL CURA DARS	Analytical Competence Molecular Epidemiology Lab/ACME, Oswaldo Cruz Foundation, Ceara (FIOCRUZ CE)	Cleber Furtado Aksenen; Fabio Miyajima; Fernando Braga Stehling; Francisco Eder de Moura Lopes; Jamille Maria Mendes Bezerra; Joaquim César do Nascimento Sousa Junior; Pedro Miguel Carneiro Jeronimo; Suzana Porto Almeida e Lucas Delerino; Thais Ferreira de Oliveira; Thais de Oliveira Costa; Ticiane Cavalcante de Souza; Veridiana Pessoa Miyajima
EPI_ISL_2493956, EPI_ISL_2493959	HOSPITAL DE CAMPANHA COVID 19 DE MAIRIPORA	Instituto Butantan	Antonio Jorge Martins; Claudia Renata dos Santos Barros; David Schlesinger; Debora Botequio Moretti; Dimas Tadeu Covas; Elaine Cristina Marqueze; Elaine Vieira Santos; Evandra Strazza Rodrigues; Heidge Fukumasu; Jayme Augusto de Souza-Neto; José Salvatore Leister Patané; Luiz Alcantara; Luiz Lehmann Coutinho; Maria Carolina Elias; Maurício Lacerda Nogueira; Rafael dos Santos Bezerra; Raul Machado Neto; Rejane Maria Tommasini Grotto; Ricardo Haddad; Sandra Coccuzzo Sampaio Vessoni; Simone Kashima; Svetoslav Nanev Slavov; Vincent Louis Viala
EPI_ISL_2493873, EPI_ISL_2493876, EPI_ISL_2493877	HOSPITAL DE CAMPANHA COVID 19 MUNICIPIO DE TAUBATE	Instituto Butantan	Antonio Jorge Martins; Claudia Renata dos Santos Barros; David Schlesinger; Debora Botequio Moretti; Dimas Tadeu Covas; Elaine Cristina Marqueze; Elaine Vieira Santos; Evandra Strazza Rodrigues; Heidge Fukumasu; Jayme Augusto de Souza-Neto; José Salvatore Leister Patané; Luiz Alcantara; Luiz Lehmann Coutinho; Maria Carolina Elias; Maurício Lacerda Nogueira; Rafael dos Santos Bezerra; Raul Machado Neto; Rejane Maria Tommasini Grotto; Ricardo Haddad; Sandra Coccuzzo Sampaio Vessoni; Simone Kashima; Svetoslav Nanev Slavov; Vincent Louis Viala
EPI_ISL_3037826	HOSPITAL DE NIÑOS DR. CARLOS SAENZ HERRERA	Inciensa, Instituto Costarricense de Investigación y Enseñanza en Nutrición y Salud	Adriana Godínez; Caterina Guzmán; Claudio Soto-Garita; Estela Cordero; Francisco Duarte; Hebleen Porras; José Luis Vargas; Mariela Gutiérrez Joselyn Prado; Melany Calderón; Nazareth Ruiz & Cristian Pérez
EPI_ISL_3459388	HOSPITAL DEPARTAMENTAL DE VILLAVICENCIO	Instituto Nacional de Salud	Carlos Franco-Muñoz; Carmen Osorio; Diana Malo; Diego A. Álvarez-Díaz; Diego Andrés Prada; Gerardo Santamaría; Hector Alejandro Ruiz-Moreno; Jhonnatan Reales-González; Jorge Rivera; Juan Camilo Martinez; Julian Naizaque; Katherine Laiton-Donato; Lisseth Pardo; Magdalena Wiesner; Marcela Mercado-Reyes; Maria T. Herrera-Sepúlveda; Marta Lopez Blanco; Martha Lucia Ospina Martinez; Paola Rojas; Sergio Gomez; Sheryll Corchuelo; Ángela Alarcon Cruz
EPI_ISL_2827976, EPI_ISL_2827977	HOSPITAL DR. ENRIQUE BALTODANO BRICEÑO	Inciensa, Instituto Costarricense de Investigación y Enseñanza en Nutrición y Salud	Adriana Godínez; Claudio Soto-Garita; Estela Cordero; Francisco Duarte; Hebleen Porras; Joselyn Prado & Adriana Bermúdez; José Luis Vargas; Mariela Gutiérrez; Melany Calderón
EPI_ISL_3464519	HOSPITAL DR. FERNANDO ESCALANTE PRADILLA	Inciensa, Instituto Costarricense de Investigación y Enseñanza en Nutrición y Salud	Adriana Godínez; Claudio Soto-Garita; Estela Cordero; Francisco Duarte; Hebleen Porras; Joselyn Prado & Yendri Ramírez Alpízar; José Luis Vargas; Mariela Gutiérrez; Melany Calderón
EPI_ISL_2827967, EPI_ISL_2828010, EPI_ISL_3037815	HOSPITAL DR. RAFAEL A. CALDERON GUARDIA	Inciensa, Instituto Costarricense de Investigación y Enseñanza en Nutrición y Salud	Adriana Godínez; Caterina Guzmán; Claudio Soto-Garita; Estela Cordero; Francisco Duarte; Hebleen Porras; Joselyn Prado & Fabian Salas; José Luis Vargas; Mariela Gutiérrez; Mariela Gutiérrez Joselyn Prado; Melany Calderón; Nazareth Ruiz & Fabián Salas
EPI_ISL_2827963, EPI_ISL_2827964, EPI_ISL_2827965	HOSPITAL DR. WILLIAM ALLEN	Inciensa, Instituto Costarricense de Investigación y Enseñanza en Nutrición y Salud	Adriana Godínez; Claudio Soto-Garita; Estela Cordero; Francisco Duarte; Hebleen Porras; José Luis Vargas; Mariela Gutiérrez & Joselyn Prado; Melany Calderón
EPI_ISL_3102412, EPI_ISL_3102472, EPI_ISL_3536263, EPI_ISL_3536264, EPI_ISL_3536265	HOSPITAL E MATERNIDADE DRA ZILDA ARNS NEUMANN	Analytical Competence Molecular Epidemiology Lab/ACME, Oswaldo Cruz Foundation, Ceara (FIOCRUZ CE)	Cleber Furtado Aksenen; Fabio Miyajima; Fernando Braga Stehling; Francisco Eder de Moura Lopes; Jamille Maria Mendes Bezerra; Joaquim César do Nascimento Sousa Junior; Pedro Miguel Carneiro Jeronimo; Suzana Porto Almeida e Lucas Delerino; Thais Ferreira de Oliveira; Thais de Oliveira Costa; Ticiane Cavalcante de Souza; Veridiana Pessoa Miyajima
EPI_ISL_2493257, EPI_ISL_2493258, EPI_ISL_2493863, EPI_ISL_2493865, EPI_ISL_2493870, EPI_ISL_2493871, EPI_ISL_2500986			
see above	HOSPITAL E MATERNIDADE NOSSA SENHORA DA AJUDA	Instituto Butantan	Antonio Jorge Martins; Claudia Renata dos Santos Barros; David Schlesinger; Debora Botequio Moretti; Dimas Tadeu Covas; Elaine Cristina Marqueze; Elaine Vieira Santos; Evandra Strazza Rodrigues; Heidge Fukumasu; Jayme Augusto de Souza-Neto; José Salvatore Leister Patané; Luiz Alcantara; Luiz Lehmann Coutinho; Maria Carolina Elias; Maurício Lacerda Nogueira; Rafael dos Santos Bezerra; Raul Machado Neto; Rejane Maria Tommasini Grotto; Ricardo Haddad; Sandra Coccuzzo Sampaio Vessoni; Simone Kashima; Svetoslav Nanev Slavov; Vincent Louis Viala
EPI_ISL_3102275, EPI_ISL_3102334, EPI_ISL_3102336, EPI_ISL_3102341, EPI_ISL_3102349, EPI_ISL_3102351, EPI_ISL_3102354, EPI_ISL_3102360, EPI_ISL_3102364, EPI_ISL_3102372, EPI_ISL_3102374, EPI_ISL_3102375, EPI_ISL_3102376, EPI_ISL_3102383, EPI_ISL_3102384, EPI_ISL_3102386, EPI_ISL_3102387, EPI_ISL_3102393, EPI_ISL_3102394, EPI_ISL_3102395, EPI_ISL_3102396, EPI_ISL_3102399, EPI_ISL_3102403, EPI_ISL_3102405, EPI_ISL_3102506, EPI_ISL_3536149, EPI_ISL_3536154, EPI_ISL_3536155, EPI_ISL_3536169, EPI_ISL_3536170, EPI_ISL_3536174, EPI_ISL_3536175, EPI_ISL_3536176, EPI_ISL_3536184, EPI_ISL_3536186, EPI_ISL_3536187, EPI_ISL_3536188, EPI_ISL_3536193, EPI_ISL_3536266, EPI_ISL_3536267, EPI_ISL_3536270, EPI_ISL_3536271, EPI_ISL_3536272, EPI_ISL_3536273, EPI_ISL_3536274, EPI_ISL_3536275, EPI_ISL_3536277, EPI_ISL_3536278, EPI_ISL_3536279, EPI_ISL_3536280, EPI_ISL_3536281, EPI_ISL_3536282, EPI_ISL_3536283, EPI_ISL_3536284, EPI_ISL_3536285, EPI_ISL_3536286, EPI_ISL_3536287, EPI_ISL_3536288, EPI_ISL_3536312, EPI_ISL_3536313, EPI_ISL_3536323, EPI_ISL_3536324, EPI_ISL_3536332, EPI_ISL_3536334, EPI_ISL_3536341, EPI_ISL_3536342, EPI_ISL_3536347, EPI_ISL_3536357, EPI_ISL_3536359, EPI_ISL_3536364, EPI_ISL_3536372, EPI_ISL_3536376, EPI_ISL_3536377, EPI_ISL_3536393, EPI_ISL_3536395, EPI_ISL_3536398, EPI_ISL_3912206			
see above	HOSPITAL ESTADUAL LEONARDO DA VINCI	Analytical Competence Molecular Epidemiology Lab/ACME, Oswaldo Cruz Foundation, Ceara (FIOCRUZ CE)	Cleber Furtado Aksenen; Fabio Miyajima; Fernando Braga Stehling; Francisco Eder de Moura Lopes; Jamille Maria Mendes Bezerra; Joaquim Cesar do Nascimento Sousa Junior; Joaquim César do Nascimento Sousa Junior; Pedro Miguel Carneiro Jeronimo; Suzana Porto Almeida & Lucas Delerino on behalf of COVID-19 FIOCRUZ Genomic Network; Suzana Porto Almeida e Lucas Delerino; Thais Ferreira de Oliveira; Thais de Oliveira Costa; Ticiane Cavalcante de Souza; Veridiana Pessoa Miyajima
EPI_ISL_2493861, EPI_ISL_2493957	HOSPITAL HELIOPOLIS UNIDADE DE GESTAO ASSISTENCIAL I SP	Instituto Butantan	Antonio Jorge Martins; Claudia Renata dos Santos Barros; David Schlesinger; Debora Botequio Moretti; Dimas Tadeu Covas; Elaine Cristina Marqueze; Elaine Vieira Santos; Evandra Strazza Rodrigues; Heidge Fukumasu; Jayme Augusto de Souza-Neto; José Salvatore Leister Patané; Luiz Alcantara; Luiz Lehmann Coutinho; Maria Carolina Elias; Maurício Lacerda Nogueira; Rafael dos Santos Bezerra; Raul Machado Neto; Rejane Maria Tommasini Grotto; Ricardo Haddad; Sandra Coccuzzo Sampaio Vessoni; Simone Kashima; Svetoslav Nanev Slavov; Vincent Louis Viala
EPI_ISL_2626156	HOSPITAL MANACOR	HOSPITAL UNIVERSITARIO SON ESPASES	Hospital Universitario Son Espases; Servicio de Microbiología
EPI_ISL_3102330	HOSPITAL MATERNIDADE AGENOR ARAUJO	Analytical Competence Molecular Epidemiology Lab/ACME, Oswaldo Cruz Foundation, Ceara (FIOCRUZ CE)	Cleber Furtado Aksenen; Fabio Miyajima; Fernando Braga Stehling; Francisco Eder de Moura Lopes; Jamille Maria Mendes Bezerra; Joaquim César do Nascimento Sousa Junior; Pedro Miguel Carneiro Jeronimo; Suzana Porto Almeida e Lucas Delerino; Thais Ferreira de Oliveira; Thais de Oliveira Costa; Ticiane Cavalcante de Souza; Veridiana Pessoa Miyajima
EPI_ISL_2827991, EPI_ISL_2827992, EPI_ISL_3298328	HOSPITAL MEXICO	Inciensa, Instituto Costarricense de Investigación y Enseñanza en Nutrición y Salud	Adriana Godínez; Claudio Soto-Garita; Estela Cordero; Francisco Duarte; Hebleen Porras; Joselyn Prado & Juan Carlos Villalobos; José Luis Vargas; Mariela Gutiérrez; Melany Calderón
EPI_ISL_2827981, EPI_ISL_2828014	HOSPITAL MONSEÑOR SANABRIA	Inciensa, Instituto Costarricense de Investigación y Enseñanza en Nutrición y Salud	Adriana Godínez; Claudio Soto-Garita; Estela Cordero; Francisco Duarte; Hebleen Porras; Joselyn Prado & María José Gómez; José Luis Vargas; Mariela Gutiérrez; Melany Calderón
EPI_ISL_2493882	HOSPITAL MUN DR GUIDO GUIDA	Instituto Butantan	Antonio Jorge Martins; Claudia Renata dos Santos Barros; David Schlesinger; Debora Botequio Moretti; Dimas Tadeu Covas; Elaine Cristina Marqueze; Elaine Vieira Santos; Evandra Strazza Rodrigues; Heidge Fukumasu; Jayme Augusto de Souza-Neto; José Salvatore Leister Patané; Luiz Alcantara; Luiz Lehmann Coutinho; Maria Carolina Elias; Maurício Lacerda Nogueira; Rafael dos Santos Bezerra; Raul Machado Neto; Rejane Maria Tommasini Grotto; Ricardo Haddad; Sandra Coccuzzo Sampaio Vessoni; Simone Kashima; Svetoslav Nanev Slavov; Vincent Louis Viala
EPI_ISL_2493955	HOSPITAL MUNICIPAL DE IBIUNA IBIUNA SP	Instituto Butantan	Antonio Jorge Martins; Claudia Renata dos Santos Barros; David Schlesinger; Debora Botequio Moretti; Dimas Tadeu Covas; Elaine Cristina Marqueze; Elaine Vieira Santos; Evandra Strazza Rodrigues; Heidge Fukumasu; Jayme Augusto de Souza-Neto; José Salvatore Leister Patané; Luiz Alcantara; Luiz Lehmann Coutinho; Maria Carolina Elias; Maurício Lacerda Nogueira; Rafael dos Santos Bezerra; Raul Machado Neto; Rejane Maria Tommasini Grotto; Ricardo Haddad; Sandra Coccuzzo Sampaio Vessoni; Simone Kashima; Svetoslav Nanev Slavov; Vincent Louis Viala

EPI_ISL_2462326, EPI_ISL_2494132, EPI_ISL_2494135, EPI_ISL_2494136, EPI_ISL_2494139, EPI_ISL_2494140	HOSPITAL MUNICIPAL DE ITAPIRA	Instituto Butantan	Antonio Jorge Martins; Claudia Renata dos Santos Barros; David Schlesinger; Debora Botequio Moretti; Dimas Tadeu Covas; Elaine Cristina Marqueze; Elaine Vieira Santos; Evandra Strazza Rodrigues; Heidge Fukumasu; Jayme Augusto de Souza-Neto; José Salvatore Leister Patané; Luiz Alcantara; Luiz Lehmann Coutinho; Maria Carolina Elias; Maurício Lacerda Nogueira; Rafael dos Santos Bezerra; Raul Machado Neto; Rejane Maria Tommasini Grotto; Ricardo Haddad; Sandra Coccuzzo Sampaio Vessoni; Simone Kashima; Svetoslav Nanev Slavov; Vincent Louis Viala
EPI_ISL_2801347	HOSPITAL MUNICIPAL DR EDUARDO DIAS	Analytical Competence Molecular Epidemiology Lab/ACME, Oswaldo Cruz Foundation, Ceara (FIOCRUZ CE)	Cleber Furtado Aksenen e Suzana Porto Almeida; Fabio Miyajima; Fernando Braga Stehling; Francisco Eder de Moura Lopes; Jamille Maria Mendes Bezerra; Joaquim César do Nascimento Sousa Junior; Pedro Miguel Carneiro Jeronimo; Thais Ferreira de Oliveira; Thais de Oliveira Costa; Ticiane Cavalcante de Souza; Veridiana Pessoa Miyajima
EPI_ISL_2462315, EPI_ISL_2493821, EPI_ISL_2493827	HOSPITAL MUNICIPAL DR MARIO GATTI CAMPINAS	Instituto Butantan	Antonio Jorge Martins; Claudia Renata dos Santos Barros; David Schlesinger; Debora Botequio Moretti; Dimas Tadeu Covas; Elaine Cristina Marqueze; Elaine Vieira Santos; Evandra Strazza Rodrigues; Heidge Fukumasu; Jayme Augusto de Souza-Neto; José Salvatore Leister Patané; Luiz Alcantara; Luiz Lehmann Coutinho; Maria Carolina Elias; Maurício Lacerda Nogueira; Rafael dos Santos Bezerra; Raul Machado Neto; Rejane Maria Tommasini Grotto; Ricardo Haddad; Sandra Coccuzzo Sampaio Vessoni; Simone Kashima; Svetoslav Nanev Slavov; Vincent Louis Viala
EPI_ISL_2494164	HOSPITAL MUNICIPAL JORGE CURY DE SANTA MARIA DA SERRA	Instituto Butantan	Antonio Jorge Martins; Claudia Renata dos Santos Barros; David Schlesinger; Debora Botequio Moretti; Dimas Tadeu Covas; Elaine Cristina Marqueze; Elaine Vieira Santos; Evandra Strazza Rodrigues; Heidge Fukumasu; Jayme Augusto de Souza-Neto; José Salvatore Leister Patané; Luiz Alcantara; Luiz Lehmann Coutinho; Maria Carolina Elias; Maurício Lacerda Nogueira; Rafael dos Santos Bezerra; Raul Machado Neto; Rejane Maria Tommasini Grotto; Ricardo Haddad; Sandra Coccuzzo Sampaio Vessoni; Simone Kashima; Svetoslav Nanev Slavov; Vincent Louis Viala
EPI_ISL_2801348, EPI_ISL_2801349, EPI_ISL_2801355, EPI_ISL_2801370, EPI_ISL_3102265, EPI_ISL_3102299, EPI_ISL_3102508, EPI_ISL_3536345, EPI_ISL_3912216, EPI_ISL_3912217, EPI_ISL_3912218, EPI_ISL_3912219, EPI_ISL_3912220			
see above	HOSPITAL MUNICIPAL NOSSA SENHORA DOS MILAGRES	Analytical Competence Molecular Epidemiology Lab/ACME, Oswaldo Cruz Foundation, Ceara (FIOCRUZ CE)	Cleber Furtado Aksenen; Cleber Furtado Aksenen e Suzana Porto Almeida; Fabio Miyajima; Fernando Braga Stehling; Francisco Eder de Moura Lopes; Jamille Maria Mendes Bezerra; Joaquim Cesar do Nascimento Sousa Junior; Joaquim César do Nascimento Sousa Junior; Pedro Miguel Carneiro Jeronimo; Suzana Porto Almeida & Lucas Delerino on behalf of COVID-19 FIOCRUZ Genomic Network; Suzana Porto Almeida e Lucas Delerino; Thais Ferreira de Oliveira; Thais de Oliveira Costa; Ticiane Cavalcante de Souza; Veridiana Pessoa Miyajima
EPI_ISL_3761773, EPI_ISL_3761782	HOSPITAL NACIONAL DE NIÑOS	Inciensa, Instituto Costarricense de Investigación y Enseñanza en Nutrición y Salud	Cristian Pérez-Corrales & Valeria Peralta-Barquero
EPI_ISL_3102505	HOSPITAL REGIONAL DE IGUATU	Analytical Competence Molecular Epidemiology Lab/ACME, Oswaldo Cruz Foundation, Ceara (FIOCRUZ CE)	Cleber Furtado Aksenen; Fabio Miyajima; Fernando Braga Stehling; Francisco Eder de Moura Lopes; Jamille Maria Mendes Bezerra; Joaquim César do Nascimento Sousa Junior; Pedro Miguel Carneiro Jeronimo; Suzana Porto Almeida e Lucas Delerino; Thais Ferreira de Oliveira; Thais de Oliveira Costa; Ticiane Cavalcante de Souza; Veridiana Pessoa Miyajima
EPI_ISL_3102304	HOSPITAL REGIONAL DO SERTAO CENTRAL	Analytical Competence Molecular Epidemiology Lab/ACME, Oswaldo Cruz Foundation, Ceara (FIOCRUZ CE)	Cleber Furtado Aksenen; Fabio Miyajima; Fernando Braga Stehling; Francisco Eder de Moura Lopes; Jamille Maria Mendes Bezerra; Joaquim César do Nascimento Sousa Junior; Pedro Miguel Carneiro Jeronimo; Suzana Porto Almeida e Lucas Delerino; Thais Ferreira de Oliveira; Thais de Oliveira Costa; Ticiane Cavalcante de Souza; Veridiana Pessoa Miyajima
EPI_ISL_3912225, EPI_ISL_3912226	HOSPITAL REGIONAL UNIMED	Analytical Competence Molecular Epidemiology Lab/ACME, Oswaldo Cruz Foundation, Ceara (FIOCRUZ CE)	Cleber Furtado Aksenen; Fabio Miyajima; Fernando Braga Stehling; Francisco Eder de Moura Lopes; Jamille Maria Mendes Bezerra; Joaquim Cesar do Nascimento Sousa Junior; Pedro Miguel Carneiro Jeronimo; Suzana Porto Almeida & Lucas Delerino on behalf of COVID-19 FIOCRUZ Genomic Network; Thais Ferreira de Oliveira; Thais de Oliveira Costa; Ticiane Cavalcante de Souza; Veridiana Pessoa Miyajima
EPI_ISL_2827973, EPI_ISL_3037808, EPI_ISL_3037811, EPI_ISL_3298308	HOSPITAL SAN JUAN DE DIOS	Inciensa, Instituto Costarricense de Investigación y Enseñanza en Nutrición y Salud	Adriana Godínez; Caterina Guzmán; Claudio Soto-Garita; Estela Cordero; Francisco Duarte; Hebleen Porras; Joselyn Prado & Juliana Mora; Joselyn Prado & Paola Sánchez; José Luis Vargas; Mariela Gutiérrez; Mariela Gutiérrez Joselyn Prado; Melany Calderón; Nazareth Ruiz & Juliana Mora
EPI_ISL_2827986	HOSPITAL SAN VICENTE DE PAUL	Inciensa, Instituto Costarricense de Investigación y Enseñanza en Nutrición y Salud	Adriana Godínez; Claudio Soto-Garita; Estela Cordero; Francisco Duarte; Hebleen Porras; Joselyn Prado & Josiany Arce; José Luis Vargas; Mariela Gutiérrez; Melany Calderón
EPI_ISL_3102428, EPI_ISL_3102449, EPI_ISL_3102451, EPI_ISL_3536153, EPI_ISL_3536289, EPI_ISL_3536290	HOSPITAL SAO JOSE DE DOENCAS INFECCIOSAS	Analytical Competence Molecular Epidemiology Lab/ACME, Oswaldo Cruz Foundation, Ceara (FIOCRUZ CE)	Cleber Furtado Aksenen; Fabio Miyajima; Fernando Braga Stehling; Francisco Eder de Moura Lopes; Jamille Maria Mendes Bezerra; Joaquim César do Nascimento Sousa Junior; Pedro Miguel Carneiro Jeronimo; Suzana Porto Almeida e Lucas Delerino; Thais Ferreira de Oliveira; Thais de Oliveira Costa; Ticiane Cavalcante de Souza; Veridiana Pessoa Miyajima
EPI_ISL_2494168, EPI_ISL_2494172	HOSPITAL SAO JOSE ITIRAPINA	Instituto Butantan	Antonio Jorge Martins; Claudia Renata dos Santos Barros; David Schlesinger; Debora Botequio Moretti; Dimas Tadeu Covas; Elaine Cristina Marqueze; Elaine Vieira Santos; Evandra Strazza Rodrigues; Heidge Fukumasu; Jayme Augusto de Souza-Neto; José Salvatore Leister Patané; Luiz Alcantara; Luiz Lehmann Coutinho; Maria Carolina Elias; Maurício Lacerda Nogueira; Rafael dos Santos Bezerra; Raul Machado Neto; Rejane Maria Tommasini Grotto; Ricardo Haddad; Sandra Coccuzzo Sampaio Vessoni; Simone Kashima; Svetoslav Nanev Slavov; Vincent Louis Viala
EPI_ISL_3536335	HOSPITAL SAO MATEUS	Analytical Competence Molecular Epidemiology Lab/ACME, Oswaldo Cruz Foundation, Ceara (FIOCRUZ CE)	Cleber Furtado Aksenen; Fabio Miyajima; Fernando Braga Stehling; Francisco Eder de Moura Lopes; Jamille Maria Mendes Bezerra; Joaquim César do Nascimento Sousa Junior; Pedro Miguel Carneiro Jeronimo; Suzana Porto Almeida e Lucas Delerino; Thais Ferreira de Oliveira; Thais de Oliveira Costa; Ticiane Cavalcante de Souza; Veridiana Pessoa Miyajima
EPI_ISL_3532488	HOSPITAL UNIVERSITARIO 12 DE OCTUBRE	HOSPITAL UNIVERSITARIO 12 DE OCTUBRE	Carmen Martín-Higuera; Esther Viedma; Irene Múñoz-Gallego; M.ª Dolores Folgueira; Mar Aguilera; Noelia Moral; Rafael Delgado; Sagrario Zurita
EPI_ISL_3102435, EPI_ISL_3102454, EPI_ISL_3536326	HOSPITAL UNIVERSITARIO WALTER CANTIDIO	Analytical Competence Molecular Epidemiology Lab/ACME, Oswaldo Cruz Foundation, Ceara (FIOCRUZ CE)	Cleber Furtado Aksenen; Fabio Miyajima; Fernando Braga Stehling; Francisco Eder de Moura Lopes; Jamille Maria Mendes Bezerra; Joaquim César do Nascimento Sousa Junior; Pedro Miguel Carneiro Jeronimo; Suzana Porto Almeida e Lucas Delerino; Thais Ferreira de Oliveira; Thais de Oliveira Costa; Ticiane Cavalcante de Souza; Veridiana Pessoa Miyajima
EPI_ISL_3235332, EPI_ISL_3235349, EPI_ISL_3235350, EPI_ISL_3235351, EPI_ISL_3235357	Hackensack Medical Center	New York Genome Center	Amy Baldwin; Andre Corvelo; Barry Kreiswirth; David Perlin; Dayna M. Oschwald; Jose Mediavilla; Kaelea Composto; Kar Chow; Liang Chen; Marcus Cunningham; Michael Zody; Samantha Fennessey; Tom Maniatis
EPI_ISL_2663225, EPI_ISL_2727868, EPI_ISL_2727869, EPI_ISL_3118528	HealthPartners Central Lab	Minnesota Department of Health, Public Health Laboratory	Alexandra Lorentz; Jacob Garfin; Matt Plumb; and Xiong Wang
EPI_ISL_3149731	Hosp. Pediatrico Dr. Avelino Castelán	Laboratorio de Biología Molecular, Instituto de Medicina Regional on behalf of 'Proyecto Argentino Interinstitucional de genomica de SARS-CoV-2' (PAIS Consortium)	Bettina Brusés; Gioia Lucia Marino; Griselda Oria; Horacio Lucero.; Javier Mussin; Julieta Ronchi; Laura Formichelli; María Andrea Gili; María Cecilia López; Melina Lorenzini Campos; Raúl Maximiliano Acevedo
EPI_ISL_2923267	Hospital	National Reference Center for Viruses of Respiratory Infections, Institut Pasteur, Paris	Angela Brisebarre; Camille Capel; Christophe Malabat; Corinne Maufrais; Etienne Simon-Lorière; FARFOUR Eric; Frédéric Lemoine; Gael Millot; Louise Lefrançois; Marion Barbet; Maud Vanpeene; Méline Bizard; Sylvie Behillil; Sylvie Van der Werf; Vincent Enouf
EPI_ISL_3092579	Hospital Alvarez Buylla	Laboratorio de Virología HUCA	Abreu F; Alvarez-Arguelles ME; Boga JA; Castelló C; Costales I; Coto E; Gómez de Oña J; Martín-Rodríguez G; Melón S; Perez-Martínez Z; Rojo S; Sandoval M
EPI_ISL_2812647, EPI_ISL_3076926	Hospital Carmen y Severo Ochoa	Laboratorio de Virología HUCA	Abreu F; Alvarez-Arguelles ME; Boga JA; Castelló C; Costales I; Coto E; Gómez de Oña J; Martín-Rodríguez G; Melón S; Perez-Martínez Z; Rojo S; Sandoval M
EPI_ISL_3147475	Hospital Center Emile Mayrisch	Laboratoire national de sante, Microbiology, Microbial Genomics Platform	Anke Wienecke-Baldacchino; Catherine Ragimbeau; Cynthia Oxacelay; Elodie Solarino; Fatu Djabi; Jessica Tapp; Lise Pignon; Raoul Salmon; Tamir Abdelrahman; Virginie Jover
EPI_ISL_3147487, EPI_ISL_3147545	Hospital Center Luxembourg	Laboratoire national de sante, Microbiology, Microbial Genomics Platform	Anke Wienecke-Baldacchino; Catherine Ragimbeau; Elodie Solarino; Fatu Djabi; Jean-Hugues Francois; Jessica Tapp; Lise Pignon; Michel Kohnen; Raoul Salmon; Tamir Abdelrahman; Virginie Jover
EPI_ISL_2402499, EPI_ISL_2484017, EPI_ISL_2484018, EPI_ISL_2601701, EPI_ISL_2601714, EPI_ISL_2675302, EPI_ISL_2782321, EPI_ISL_2782332, EPI_ISL_2782348, EPI_ISL_2782352, EPI_ISL_2782356, EPI_ISL_3167156, EPI_ISL_3505984, EPI_ISL_3505988			
see above	Hospital General Universitario Gregorio Marañón	Hospital General Universitario Gregorio Marañón	Cristina Rodriguez-Grande; Darío García de Viedma; Julia Suárez; Laura Pérez-Lago; Marta Herranz Martin; Patricia Muñoz; Pedro Sola Campoy; Pilar Catalán; Sergio Buenestado Serrano; Victor Manuel de la Cueva
EPI_ISL_3216392	Hospital General Universitario de Alicante - Instituto de Investigación Sanitaria y Biomédica de Alicante	SeqCOVID-SPAIN consortium/IBV(CSIC)	Carmen Molina Pardines and SeqCOVID-SPAIN consortium; Maripaz Ventero Martín
EPI_ISL_3033082, EPI_ISL_3033084, EPI_ISL_3033086, EPI_ISL_3260092, EPI_ISL_3260093, EPI_ISL_3260094, EPI_ISL_3260108			
see above	Hospital Jaime Ferre - SAMCO Rafaela	Grupo de Genómica y Bioinformática del Instituto de Investigación de la Cadena Láctea CONICET-INTA on behalf of	AF; Aliprandi D; Amadio; C; Eberhardt; Irazoqui; Isaia; JF; JM; MF; Pandolfi; Quaranta; Soratti R; V

		'Proyecto Argentino Interinstitucional de genomica de SARS-CoV-2' (PAIS Consortium)	
EPI_ISL_2894556, EPI_ISL_2894559, EPI_ISL_2987697, EPI_ISL_2987700, EPI_ISL_2987707	Hospital Margarita Maza de Juárez	Microbial Genomics Laboratory	; Alejandra García-Gasca; Alejandra Hernández-Terán; Alejandro Sánchez-Flores; Alfredo Herrera-Estrella; Alicia Ocaña-Mondragón; Andreu Comas-García; Angel Gustavo Salas-Lais; Antonio Loza Román; Bernardo Martínez-Miguel; Blanca Taboada; Brenda Irasema Maldonado-Meza; Bruno Gómez-Gil; Carla Ivón Herrera-Najera; Carlos F. Arias; Celia Boukadida; Clara Esperanza Santacruz-Tinoco; Concepción Grajales-Muñiz; Consorcio Mexicano de Vigilancia Genómica (CoViGen-Mex). Authors (in alphabetical order): Julio Elias Alvarado-Yaah; Cristóbal Cháidez-Quiróz; Célida Duque Molina; Célida Martínez- Rodríguez; Daniel Fregoso-Rueda; Daniel Lira Morales; Eduardo Becerril-Vargas; Fernando Fontove-Herrera; Fidencio Mejía-Nepomuceno; Francisco Pulido; Gloria Elena Espinosa-Ayala; Gloria María Molina-Salinas; Gloria Vazquez; Hector Esteban Paz-Juárez; Hector Montoya-Fuentes; Helen Haydee Fernanda Ramírez-Plascencia; Irvin González-López; Jean Pierre González; Jesús Hernández; Joel Armando Vázquez-Pérez.; Jorge Salas-Hernández; José Antonio Enciso-Moreno; José Arturo Martínez-Orozco; José Esteban Muñoz-Medina; José de Jesús Nuñez-Contreras; Juan Bautista Chale-Dzul; Julissa Enciso-Ibarra; Luis Alberto Ochoa-Carrera; Margarita Matías-Florentino; Mario Mújica-Sánchez; Marissa Perez-Garcia; María Guadalupe Santiago-Mauricio; María Guadalupe de Jesús Mireles-Rivera; Nelly Sélem-Mojica; Pavel Isa; Ricardo Ciria Merce; Ricardo Grande; Rosa María Gutiérrez Ríos; Santiago Ávila-Ríos; Selene Zárate; Susana Lopez; Verónica Mata-Haro; Victor Eduardo García-Arias; Víctor Hugo Borja-Aburto
EPI_ISL_2524776, EPI_ISL_2628167, EPI_ISL_2628170, EPI_ISL_2628171, EPI_ISL_2828226, EPI_ISL_2828227, EPI_ISL_2828233			
see above	Hospital Universitari Arnau de Vilanova	Hospital Universitari Vall d'Hebron - Vall d'Hebron Institut de Recerca	Alejandra González-Sánchez; Andrés Antón; Ariadna Rando; Carla Castillo; Cristina Andrés; Damir Garcia-Cehic; Josep Quer; Juliana Esperalba; Maria Carmen Martin; Maria Gema Codina; Maria Piñana; Tomàs Pumarola
EPI_ISL_2637324, EPI_ISL_2861766	Hospital Universitari Bellvitge	Microbiology Department	Aida Gonzalez-Diaz; Carmen Ardanuy; Jordi Camara; Jordi Niubó; Laura Calatayud; M Angeles Domínguez; Miguel Fernandez-Huerta; Sara Marti
EPI_ISL_2566592, EPI_ISL_2566593, EPI_ISL_2566594, EPI_ISL_2566595, EPI_ISL_2566596, EPI_ISL_2652058, EPI_ISL_2652059, EPI_ISL_2652064, EPI_ISL_2657359, EPI_ISL_2657366			
see above	Hospital Universitari Joan XXIII	Hospital Universitari Joan XXIII	Carla Martín; Clara Benavent; Cristina Gutiérrez; Ester Picó; Gemma Recio; Margarida Terrón; Natalia Bastón
EPI_ISL_2712852, EPI_ISL_2712870, EPI_ISL_2828111, EPI_ISL_2828116, EPI_ISL_2828151, EPI_ISL_2828171, EPI_ISL_2828215, EPI_ISL_2828217, EPI_ISL_2885302, EPI_ISL_2982331, EPI_ISL_2982387, EPI_ISL_3009983, EPI_ISL_3984105			
see above	Hospital Universitari Vall d'Hebron - Vall d'Hebron Institut de Recerca	Hospital Universitari Vall d'Hebron - Vall d'Hebron Institut de Recerca	Alejandra González-Sánchez; Andrés Antón; Ariadna Rando; Carla Castillo; Cristina Andrés; Damir Garcia-Cehic; Josep Quer; Juliana Esperalba; Karen García; Maria Carmen Martin; Maria Gema Codina; Maria Piñana; Rodrigo Vásquez; Tomàs Pumarola
EPI_ISL_2600854, EPI_ISL_2775934, EPI_ISL_2775946, EPI_ISL_2775959, EPI_ISL_2775966, EPI_ISL_2775970, EPI_ISL_2820672, EPI_ISL_2931444, EPI_ISL_2931450, EPI_ISL_3087929, EPI_ISL_3087940			
see above	Hospital Universitari i Politècnic La Fe de València	SeqCOVID-SPAIN consortium/IBV(CSIC)	Ana Gil Brusola; Eva González Barberá; José Luis López Hontangas and SeqCOVID-SPAIN consortium; María Dolores Gómez Ruiz; Salvador Giner Almaraz
EPI_ISL_2545330, EPI_ISL_2620963, EPI_ISL_2620966, EPI_ISL_2773856	Hospital Universitario Virgen de las Nieves de Granada-SAS	SeqCOVID-SPAIN consortium/IBV(CSIC)	Irene Pedrosa Corral; José M. Navarro-Marí and SeqCOVID-SPAIN consortium; Sara Sanbonmatsu Gámez
EPI_ISL_2925674, EPI_ISL_2925675, EPI_ISL_2925676, EPI_ISL_2925677	Hospital de Cabueñes	Laboratorio de Virología HUCA	Abreu F; Alvarez-Arguelles ME; Boga JA; Castelló C; Costales I; Coto E; Gómez de Oña J; Martín-Rodríguez G; Melón; Melón S; Perez-Martínez Z; Rojo S; Sandoval M
EPI_ISL_2788571	Hospital of Southern Norway - Kristiansand, Department of Medical Microbiology	Norwegian Institute of Public Health, Department of Virology	Atiya R Ali; Debech Nadia; Engebretsen Serina Beate; Garcia Llorente Ignacio; Hilde Elshaug; Hilde Vollan; Jon Bråte; Kamilla Heddeland Instefjord; Karoline Bragstad; Kathrine Stene-Johansen; Line Victoria Moen; Marie Paulsen Madsen; Olav Hungnes; Pedersen Benedikte Nevjen; Rasmus Riis Kopperud
EPI_ISL_3411959	Hospital of the University of Pennsylvania Molecular Pathology Lab	Bushman	Andrew D. Marques; Aoife M. Roche; Arupa Ganguly; Ayannah S. Fitzgerald; Brendan Kelly; Jevon Graham-Wooten; John K. Everett; Kyle Rodino; Layla A. Khatib; Mike Feldman; Ronald G. Collman and Frederic Bushan; Samantha A. Whiteside; Scott Sherrill-Mix; Shantan Reddy; Young Hwang
EPI_ISL_2597697, EPI_ISL_3023264, EPI_ISL_3023266, EPI_ISL_3023276, EPI_ISL_3023329, EPI_ISL_3023330, EPI_ISL_3023373			
see above	Hospital of the University of Pennsylvania Molecular Pathology Lab	Bushman Lab - University of Pennsylvania	Aoife M. Roche; Arupa Ganguly; Ayannah S. Fitzgerald; Brendan Kelly; Jevon Graham-Wooten; John K. Everett; Kyle Rodino; Layla A. Khatib; Mike Feldman; Ronald G. Collman and Frederic Bushan; Samantha A. Whiteside; Scott Sherrill-Mix; Shantan Reddy; Young Hwang
EPI_ISL_2616771, EPI_ISL_2968598, EPI_ISL_2968613, EPI_ISL_2968620, EPI_ISL_2968636, EPI_ISL_2968664, EPI_ISL_2968665, EPI_ISL_3398946			
see above	Houston Health Dept.	Houston Health Dept.	Adolpho Lara; Pamela Brown; Ryker Penn; Yanlai Lai
EPI_ISL_2689890, EPI_ISL_2787361, EPI_ISL_2787367, EPI_ISL_2787369, EPI_ISL_2787380, EPI_ISL_2787389, EPI_ISL_2787392, EPI_ISL_3355076			
see above	IA State Hygienic Laboratory	Centers for Disease Control and Prevention Division of Viral Diseases, Pathogen Discovery	Alex Burgin; Alison Laufer Halpin; Ben L. Rambo-Martin; Ben Rambo-Martin; Clinton Paden; Clinton R. Paden; Dakota Howard; Darlene Wagner; Dave Wentworth; Dhwani Batra; Jasmine Padilla; Justin Lee; Katie Dillon; Krista Queen; Kristen Knipe; Kristine Lacek; Mark Burroughs; Matthew Schmerer; Meghan Bentz; Mili Sheth; Peter Cook; Sam Shepard; Sarah Nobles; Shoshona Le; Suxiang Tong; Vivien Dugan; Yvette Unoarumhi
EPI_ISL_2690063, EPI_ISL_2690064, EPI_ISL_2840317, EPI_ISL_2840320, EPI_ISL_2987054, EPI_ISL_3128023, EPI_ISL_3128024, EPI_ISL_3128028, EPI_ISL_3355243, EPI_ISL_3355266, EPI_ISL_3355267, EPI_ISL_3355326			
see above	ID Bureau of Laboratories	Centers for Disease Control and Prevention Division of Viral Diseases, Pathogen Discovery	Alex Burgin; Alison Laufer Halpin; Ben L. Rambo-Martin; Ben Rambo-Martin; Clinton Paden; Clinton R. Paden; Dakota Howard; Darlene Wagner; Dave Wentworth; Dhwani Batra; Jasmine Padilla; Justin Lee; Katie Dillon; Krista Queen; Kristen Knipe; Kristine Lacek; Mark Burroughs; Matthew Schmerer; Meghan Bentz; Mili Sheth; Peter Cook; Sam Shepard; Sarah Nobles; Shoshona Le; Suxiang Tong; Vivien Dugan; Yvette Unoarumhi
EPI_ISL_2812810	IFO Regina Elena	National Institute for Infectious Diseases (INMI) L. Spallanzani I.R.C.C.S	A Di Caro; B Bartolini; CEM Gruber; E Giombini; F Messina; F Santini; G Bonfiglio; M Rueca; MR Capobianchi; O Butera
EPI_ISL_2788130, EPI_ISL_2788133, EPI_ISL_2788138, EPI_ISL_2788144, EPI_ISL_2788147, EPI_ISL_2840290	IL Dept. of Public Health Springfield Laboratory	Centers for Disease Control and Prevention Division of Viral Diseases, Pathogen Discovery	Alison Laufer Halpin; Ben L. Rambo-Martin; Clinton R. Paden; Dakota Howard; Darlene Wagner; Dave Wentworth; Dhwani Batra; Jasmine Padilla; Justin Lee; Katie Dillon; Krista Queen; Kristen Knipe; Kristine Lacek; Mark Burroughs; Matthew Schmerer; Mili Sheth; Peter Cook; Sam Shepard; Sarah Nobles; Shoshona Le; Suxiang Tong; Vivien Dugan; Yvette Unoarumhi
EPI_ISL_2544640, EPI_ISL_2544660, EPI_ISL_2557242, EPI_ISL_2725041, EPI_ISL_2860400, EPI_ISL_3030076	IN State Department of Health Laboratory Services	IN State Department of Health Laboratory Services	Brian Pope; Cassandra Campion; Jamie Yeadon; Kyle Brownlee; Lixia Liu; Mark Glazier; Melissa Hindenlang
EPI_ISL_2493987, EPI_ISL_2493994, EPI_ISL_2494001, EPI_ISL_2494327	INSIDE CRSN CACHOEIRINHA/CASA VERDE	Instituto Butantan	Antonio Jorge Martins; Claudia Renata dos Santos Barros; David Schlesinger; Debora Botequio Moretti; Dimas Tadeu Covas; Elaine Cristina Marqueze; Elaine Vieira Santos; Evandra Strazza Rodrigues; Heidge Fukumasu; Jayme Augusto de Souza-Neto; José Salvatore Leister Patané; Luiz Alcantara; Luiz Lehmann Coutinho; Maria Carolina Elias; Maurício Lacerda Nogueira; Rafael dos Santos Bezerra; Raul Machado Neto; Rejane Maria Tommasini Grotto; Ricardo Haddad; Sandra Coccuzzo Sampaio Vessoni; Simone Kashima; Svetoslav Nanev Slavov; Vincent Louis Viala
EPI_ISL_2493973, EPI_ISL_2493982, EPI_ISL_2493988, EPI_ISL_2493992, EPI_ISL_2493998, EPI_ISL_2494024, EPI_ISL_2494035			
see above	INSIDE CRSN FREGUESIA/BRASILÂNDIA	Instituto Butantan	Antonio Jorge Martins; Claudia Renata dos Santos Barros; David Schlesinger; Debora Botequio Moretti; Dimas Tadeu Covas; Elaine Cristina Marqueze; Elaine Vieira Santos; Evandra Strazza Rodrigues; Heidge Fukumasu; Jayme Augusto de Souza-Neto; José Salvatore Leister Patané; Luiz Alcantara; Luiz Lehmann Coutinho; Maria Carolina Elias; Maurício Lacerda Nogueira; Rafael dos Santos Bezerra; Raul Machado Neto; Rejane Maria Tommasini Grotto; Ricardo Haddad; Sandra Coccuzzo Sampaio Vessoni; Simone Kashima; Svetoslav Nanev Slavov; Vincent Louis Viala
EPI_ISL_2493979, EPI_ISL_2494026	INSIDE CRSN PERUS	Instituto Butantan	Antonio Jorge Martins; Claudia Renata dos Santos Barros; David Schlesinger; Debora Botequio Moretti; Dimas Tadeu Covas; Elaine Cristina Marqueze; Elaine Vieira Santos; Evandra Strazza Rodrigues; Heidge Fukumasu; Jayme Augusto de Souza-Neto; José Salvatore Leister Patané; Luiz Alcantara; Luiz Lehmann Coutinho; Maria Carolina Elias; Maurício Lacerda Nogueira; Rafael dos Santos Bezerra; Raul Machado Neto; Rejane Maria Tommasini Grotto; Ricardo Haddad; Sandra Coccuzzo Sampaio Vessoni; Simone Kashima; Svetoslav Nanev Slavov; Vincent Louis Viala
EPI_ISL_2493201, EPI_ISL_2493967, EPI_ISL_2493971, EPI_ISL_2493993, EPI_ISL_2494003, EPI_ISL_2494027	INSIDE CRSN PIRITUBA	Instituto Butantan	Antonio Jorge Martins; Claudia Renata dos Santos Barros; David Schlesinger; Debora Botequio Moretti; Dimas Tadeu Covas; Elaine Cristina Marqueze; Elaine Vieira Santos; Evandra Strazza Rodrigues; Heidge Fukumasu; Jayme Augusto de Souza-Neto; José Salvatore Leister Patané; Luiz Alcantara; Luiz Lehmann Coutinho; Maria Carolina Elias; Maurício Lacerda Nogueira; Rafael dos Santos Bezerra; Raul Machado Neto; Rejane Maria Tommasini Grotto; Ricardo Haddad; Sandra Coccuzzo Sampaio Vessoni; Simone Kashima; Svetoslav Nanev Slavov; Vincent Louis Viala
EPI_ISL_2493259, EPI_ISL_2493984, EPI_ISL_2494006, EPI_ISL_2494007	INSIDE CRSN VILA MARIA/VILA GUILHERME	Instituto Butantan	Antonio Jorge Martins; Claudia Renata dos Santos Barros; David Schlesinger; Debora Botequio Moretti; Dimas Tadeu Covas; Elaine Cristina Marqueze; Elaine Vieira Santos; Evandra Strazza Rodrigues; Heidge Fukumasu; Jayme Augusto de Souza-Neto; José Salvatore Leister Patané; Luiz Alcantara; Luiz Lehmann Coutinho; Maria Carolina Elias; Maurício Lacerda Nogueira; Rafael dos Santos Bezerra; Raul Machado Neto; Rejane Maria Tommasini Grotto; Ricardo Haddad; Sandra Coccuzzo Sampaio Vessoni; Simone Kashima; Svetoslav Nanev Slavov; Vincent Louis Viala
EPI_ISL_2493265, EPI_ISL_2493968, EPI_ISL_2494000, EPI_ISL_2494030, EPI_ISL_2494033	INSIDE CRSO BUTANTÃ	Instituto Butantan	Antonio Jorge Martins; Claudia Renata dos Santos Barros; David Schlesinger; Debora Botequio Moretti; Dimas Tadeu Covas; Elaine Cristina Marqueze; Elaine Vieira Santos; Evandra Strazza Rodrigues; Heidge Fukumasu; Jayme Augusto de Souza-Neto; José Salvatore Leister Patané; Luiz Alcantara; Luiz Lehmann Coutinho; Maria Carolina Elias; Maurício Lacerda Nogueira; Rafael dos Santos Bezerra; Raul Machado Neto; Rejane Maria Tommasini Grotto; Ricardo Haddad; Sandra Coccuzzo Sampaio Vessoni; Simone Kashima; Svetoslav Nanev Slavov; Vincent Louis Viala
EPI_ISL_2493200	INSIDE CRSO LAPA/PINHEIROS	Instituto Butantan	Antonio Jorge Martins; Claudia Renata dos Santos Barros; David Schlesinger; Debora Botequio Moretti; Dimas Tadeu Covas; Elaine Cristina Marqueze; Elaine Vieira Santos; Evandra Strazza Rodrigues; Heidge Fukumasu; Jayme Augusto de Souza-Neto; José Salvatore Leister Patané; Luiz Alcantara; Luiz Lehmann Coutinho; Maria Carolina Elias; Maurício Lacerda Nogueira; Rafael dos Santos Bezerra; Raul Machado Neto; Rejane Maria Tommasini Grotto; Ricardo Haddad; Sandra Coccuzzo Sampaio Vessoni; Simone Kashima; Svetoslav Nanev Slavov; Vincent Louis Viala
EPI_ISL_2493964, EPI_ISL_2493972, EPI_ISL_2493976, EPI_ISL_2493978, EPI_ISL_2493995, EPI_ISL_2494012, EPI_ISL_2494016, EPI_ISL_2494017, EPI_ISL_2494018, EPI_ISL_2494029, EPI_ISL_2494034, EPI_ISL_2494038			
see above	INSIDE CRSS CAPELA DO SOCORRO	Instituto Butantan	Antonio Jorge Martins; Claudia Renata dos Santos Barros; David Schlesinger; Debora Botequio Moretti; Dimas Tadeu Covas; Elaine Cristina Marqueze; Elaine Vieira Santos; Evandra Strazza Rodrigues; Heidge Fukumasu; Jayme Augusto de Souza-Neto; José Salvatore Leister Patané; Luiz Alcantara; Luiz Lehmann Coutinho; Maria Carolina Elias; Maurício Lacerda Nogueira; Rafael dos Santos Bezerra; Raul Machado Neto; Rejane Maria Tommasini Grotto; Ricardo Haddad; Sandra Coccuzzo Sampaio Vessoni; Simone Kashima; Svetoslav Nanev Slavov; Vincent Louis Viala
EPI_ISL_2493960, EPI_ISL_2493961, EPI_ISL_2493977, EPI_ISL_2493989, EPI_ISL_2493990, EPI_ISL_2494005, EPI_ISL_2494036			

see above	INSIDE CRSS PARELHEIROS	Instituto Butantan	Antonio Jorge Martins; Claudia Renata dos Santos Barros; David Schlesinger; Debora Botequio Moretti; Dimas Tadeu Covas; Elaine Cristina Marqueze; Elaine Vieira Santos; Evandra Strazza Rodrigues; Heidge Fukumasu; Jayme Augusto de Souza-Neto; José Salvatore Leister Patané; Luiz Alcantara; Luiz Lehmann Coutinho; Maria Carolina Elias; Maurício Lacerda Nogueira; Rafael dos Santos Bezerra; Raul Machado Neto; Rejane Maria Tommasini Grotto; Ricardo Haddad; Sandra Coccuzzo Sampaio Vessoni; Simone Kashima; Svetoslav Nanev Slavov; Vincent Louis Viala
EPI_ISL_2493274, EPI_ISL_2493996, EPI_ISL_2494316	INSIDE CRSSU IPIRANGA	Instituto Butantan	Antonio Jorge Martins; Claudia Renata dos Santos Barros; David Schlesinger; Debora Botequio Moretti; Dimas Tadeu Covas; Elaine Cristina Marqueze; Elaine Vieira Santos; Evandra Strazza Rodrigues; Heidge Fukumasu; Jayme Augusto de Souza-Neto; José Salvatore Leister Patané; Luiz Alcantara; Luiz Lehmann Coutinho; Maria Carolina Elias; Maurício Lacerda Nogueira; Rafael dos Santos Bezerra; Raul Machado Neto; Rejane Maria Tommasini Grotto; Ricardo Haddad; Sandra Coccuzzo Sampaio Vessoni; Simone Kashima; Svetoslav Nanev Slavov; Vincent Louis Viala
EPI_ISL_2493970	INSIDE CRSSU JABAQUARA/VILA MARIANA	Instituto Butantan	Antonio Jorge Martins; Claudia Renata dos Santos Barros; David Schlesinger; Debora Botequio Moretti; Dimas Tadeu Covas; Elaine Cristina Marqueze; Elaine Vieira Santos; Evandra Strazza Rodrigues; Heidge Fukumasu; Jayme Augusto de Souza-Neto; José Salvatore Leister Patané; Luiz Alcantara; Luiz Lehmann Coutinho; Maria Carolina Elias; Maurício Lacerda Nogueira; Rafael dos Santos Bezerra; Raul Machado Neto; Rejane Maria Tommasini Grotto; Ricardo Haddad; Sandra Coccuzzo Sampaio Vessoni; Simone Kashima; Svetoslav Nanev Slavov; Vincent Louis Viala
EPI_ISL_2494004, EPI_ISL_2494028, EPI_ISL_2494031, EPI_ISL_2494310	INSIDE CRSSU MOOCA/ARICANDUVA	Instituto Butantan	Antonio Jorge Martins; Claudia Renata dos Santos Barros; David Schlesinger; Debora Botequio Moretti; Dimas Tadeu Covas; Elaine Cristina Marqueze; Elaine Vieira Santos; Evandra Strazza Rodrigues; Heidge Fukumasu; Jayme Augusto de Souza-Neto; José Salvatore Leister Patané; Luiz Alcantara; Luiz Lehmann Coutinho; Maria Carolina Elias; Maurício Lacerda Nogueira; Rafael dos Santos Bezerra; Raul Machado Neto; Rejane Maria Tommasini Grotto; Ricardo Haddad; Sandra Coccuzzo Sampaio Vessoni; Simone Kashima; Svetoslav Nanev Slavov; Vincent Louis Viala
EPI_ISL_2493962, EPI_ISL_2493965, EPI_ISL_2493983, EPI_ISL_2493999, EPI_ISL_2494009, EPI_ISL_2494019, EPI_ISL_2494020			
see above	INSIDE CRSSU SAPOPEMBA/VILA PRUDENTE	Instituto Butantan	Antonio Jorge Martins; Claudia Renata dos Santos Barros; David Schlesinger; Debora Botequio Moretti; Dimas Tadeu Covas; Elaine Cristina Marqueze; Elaine Vieira Santos; Evandra Strazza Rodrigues; Heidge Fukumasu; Jayme Augusto de Souza-Neto; José Salvatore Leister Patané; Luiz Alcantara; Luiz Lehmann Coutinho; Maria Carolina Elias; Maurício Lacerda Nogueira; Rafael dos Santos Bezerra; Raul Machado Neto; Rejane Maria Tommasini Grotto; Ricardo Haddad; Sandra Coccuzzo Sampaio Vessoni; Simone Kashima; Svetoslav Nanev Slavov; Vincent Louis Viala
EPI_ISL_2893596, EPI_ISL_2893597, EPI_ISL_2893598, EPI_ISL_2893599, EPI_ISL_2893600, EPI_ISL_2893601, EPI_ISL_2893602, EPI_ISL_2893604, EPI_ISL_2893605, EPI_ISL_2897544, EPI_ISL_2988448, EPI_ISL_2988462, EPI_ISL_2988463, EPI_ISL_2988466, EPI_ISL_2988479, EPI_ISL_2988486, EPI_ISL_2988501, EPI_ISL_2988502, EPI_ISL_2988506, EPI_ISL_2988519, EPI_ISL_2988528, EPI_ISL_2988529, EPI_ISL_2988531, EPI_ISL_2988552, EPI_ISL_2988556, EPI_ISL_2988560, EPI_ISL_2988561, EPI_ISL_2988568, EPI_ISL_2988573, EPI_ISL_2988577, EPI_ISL_2988581, EPI_ISL_3010437, EPI_ISL_3068144, EPI_ISL_3068159, EPI_ISL_3068166, EPI_ISL_3068194, EPI_ISL_3068213, EPI_ISL_3068218, EPI_ISL_3068223, EPI_ISL_3068228, EPI_ISL_3068231, EPI_ISL_3133207, EPI_ISL_3274398, EPI_ISL_3274462, EPI_ISL_3274503, EPI_ISL_3274512, EPI_ISL_3274524, EPI_ISL_3274539, EPI_ISL_3274540, EPI_ISL_3274593			
see above	INSPI-CRN DE INFLUENZA Y OTROS VIRUS RESPIRATORIOS	NIC-INSPI	Alfredo Bruno; Daniel Ramos; Domenica de Mora.; Jimmy Garcés; Johanna Laines; Lizbeth Patiño; Manuel Gonzalez; Marcela Mejía; Maria Angelica Becerra; Maritza Olmedo; Mayra Wilca; Michelle Páez; Milton Medina
EPI_ISL_2604875, EPI_ISL_2604876, EPI_ISL_2604877, EPI_ISL_3088317, EPI_ISL_3088319	INTERLAB	Omics Sciences Laboratory	Darlyn Amaya; Derly Andrade Molina; Gabriel Morey León; Juan Carlos Fernández Cadena; Rubén Armas González
EPI_ISL_2923003, EPI_ISL_2923039, EPI_ISL_2923047, EPI_ISL_2985121, EPI_ISL_2985167, EPI_ISL_2985302, EPI_ISL_3068250			
see above	IRCCS San Gallicano Dermatological Institute	IRCCS Regina Elena National Cancer Institute	Aldo Morrone; Alice Massacci; Andrea Cazzani; Eleonora Sperandio; Elisabetta Trento; Fabrizio Ensoli; Francesca De Nicola; Frauke Goeman; Fulvia Pimpinelli; Gennaro Ciliberto; Giovanni Blandino; Giulia Orlandi; Ilaria Cavallo; Ludovica Ciuffreda; Martina Pontone; Matteo Pallocca; Maurizio Fanciulli; Sara Donzelli; Sara Petrolo; Serena Salvo; di Domenico Enea Gino
EPI_ISL_2726937, EPI_ISL_2726940, EPI_ISL_2757934, EPI_ISL_2757935, EPI_ISL_2757937	ISDH	Quantigen Biosciences	Paul J. Childress
EPI_ISL_2628209, EPI_ISL_2628300, EPI_ISL_2628305	IVIC	Laboratorio de Virología Molecular	Carmen L Loureiro; CoViVen Group; Domingo J Garzaro; Esmeralda Vizzi; Flor H Pujol; Héctor R Rangel; José Luis Zambrano; Lieska Rodríguez; Mariana Hidalgo; Pierina D´Angelo; Rossana C Jaspe; Víctor Alarcón; Yoneira Sulbaran; Zoila Moros
EPI_ISL_2789980, EPI_ISL_2801469, EPI_ISL_3010167, EPI_ISL_3281530	IVY3 Central Lab, Vanderbilt University Medical Center	Lauring Lab, University of Michigan, Department of Microbiology and Immunology	Gilbert; Valesano
EPI_ISL_3090836, EPI_ISL_3090838, EPI_ISL_3090841, EPI_ISL_3100649, EPI_ISL_3100653	Idaho Bureau of Laboratories	Boise VA Medical Center, PALMS	Aimee Ceniseros; Cheri Lamb McFarlane; Christian Loera; Dr. Christopher Ball; Emily Bartlette; James Razor; Matthew Burns; Ying Pei
EPI_ISL_3066972, EPI_ISL_3066973	Idaho Bureau of Laboratories	Center for Global Health, University of New Mexico Health Sciences Center	Christopher Ball; Darrell Dinwiddie; Daryl Domman; Kurt Schwalm; Matthew Burns; Robert Voermans; Valerie Morley
EPI_ISL_2801140, EPI_ISL_2801141, EPI_ISL_3254817	Illinois Department of Public Health	Gagnon Lab, Southern Illinois University	Keith Gagnon
EPI_ISL_2800846, EPI_ISL_2800849, EPI_ISL_2800850, EPI_ISL_2800851, EPI_ISL_2800852, EPI_ISL_2800853, EPI_ISL_2800857, EPI_ISL_2800859, EPI_ISL_2800861, EPI_ISL_2800862, EPI_ISL_2800863, EPI_ISL_2800865, EPI_ISL_3232014, EPI_ISL_3232015, EPI_ISL_3232018, EPI_ISL_3232020, EPI_ISL_3232021			
see above	Illinois Department of Public Health	Illinois Department of Public Health - Chicago Lab	Ira Heimler; Joel Price; Vineet K. Dhiman
EPI_ISL_2403009, EPI_ISL_2403012, EPI_ISL_2403053, EPI_ISL_2403055, EPI_ISL_2403074, EPI_ISL_2403083, EPI_ISL_2403148, EPI_ISL_2403158, EPI_ISL_2403165, EPI_ISL_2403168, EPI_ISL_2403173, EPI_ISL_2403175, EPI_ISL_2403176, EPI_ISL_2403177, EPI_ISL_2403179, EPI_ISL_2403180, EPI_ISL_2403183, EPI_ISL_2598169, EPI_ISL_2598176, EPI_ISL_2598186, EPI_ISL_2598202, EPI_ISL_2598206, EPI_ISL_2598207, EPI_ISL_2598244, EPI_ISL_2598245, EPI_ISL_2598246, EPI_ISL_2774312, EPI_ISL_2774316, EPI_ISL_2774320, EPI_ISL_2774330, EPI_ISL_2774331, EPI_ISL_2774332, EPI_ISL_2774333, EPI_ISL_2774336, EPI_ISL_2774339, EPI_ISL_2774346, EPI_ISL_2774353, EPI_ISL_2774365, EPI_ISL_2774367, EPI_ISL_2774368, EPI_ISL_2774370, EPI_ISL_2774371, EPI_ISL_2774380, EPI_ISL_2774382, EPI_ISL_2774383, EPI_ISL_2774384, EPI_ISL_2774385, EPI_ISL_2774389, EPI_ISL_2774391, EPI_ISL_2774392, EPI_ISL_2885392, EPI_ISL_2885410, EPI_ISL_2885459, EPI_ISL_2885461			
see above	Illinois Department of Public Health - Springfield Lab	Illinois Department of Public Health - Springfield Lab	Bryan Sim; Gordon McCall
EPI_ISL_2715220, EPI_ISL_2735971, EPI_ISL_3015619, EPI_ISL_3015628, EPI_ISL_3015629, EPI_ISL_3105046, EPI_ISL_3105047, EPI_ISL_3105050, EPI_ISL_3105051, EPI_ISL_3105052, EPI_ISL_3347000			
see above	Incyte Diagnostics Spokane	Seattle Flu Study	Amanda Adler; Barry R. Lutz; Benjamin Pelle; Brian Hiatt; Caitlin R. Wolf; Chris D. Frazar; Deborah A. Nickerson; Elisabeth Brandstetter; Erica Ryke; Geoff Melly; Helen Y. Chu; Janet A. Englund; Jay Shendure; Jover Lee; Kairsten Fay; Kirsten Lacombe; Lea M. Starita; Mark J. Rieder; Matthew Richardson; Matthew Thompson; Melissa Truong; Michael Boeckh; Michael Famulare; Misja Ilcisin; Peter D. Han; Philip Dykema; Romesh Gautom; Scott Lindquist; Thomas R. Sibley; Trevor Bedford
EPI_ISL_2660401, EPI_ISL_2660405	Infectious Disease Program, Broad Institute of Harvard and MIT	Infectious Disease Program, Broad Institute of Harvard and MIT	A.E.; Adams, G.; Anahtar, M.; B.L.; B.W.; Bauer, M.; Birren; Branda, J.; Carter, A.; Cerrato, F.; Chaluvadi, S.; Chapman; Cusick, C.; D.J.; DeRuff, K.; Flowers, K.; Gallagher, G.; Gladden-Young, A.; Gnirke, A.; Harris, J.; J.E.; K.J.; LaRocque, R.; Lagerborg, K.; Lemieux; Lin; Loreth, C.; MacInnis; Neumann, A.; Normandin, E.; P.C.; Park; Pierce, V.; Reilly, S.; Rosenberg, E.; Rudy, M.; Ryan, E.; S.B.; Sabeti; Shaw, B.; Siddle; Slater, D.; Smole, S.; Tomkins-Tinch, C.; Turbett, S.
EPI_ISL_2557441, EPI_ISL_2557445, EPI_ISL_2557465, EPI_ISL_2557518, EPI_ISL_2557527, EPI_ISL_2557538, EPI_ISL_2557541, EPI_ISL_2557574, EPI_ISL_2557601, EPI_ISL_2557614, EPI_ISL_2557626, EPI_ISL_2652607, EPI_ISL_2653014, EPI_ISL_2653015, EPI_ISL_2653035, EPI_ISL_2653056, EPI_ISL_2653059, EPI_ISL_2686857, EPI_ISL_2686883, EPI_ISL_2686893, EPI_ISL_2786681, EPI_ISL_2786699, EPI_ISL_2876051, EPI_ISL_3020027, EPI_ISL_3111088			
see above	Infinity Biologix	Centers for Disease Control and Prevention Division of Viral Diseases, Pathogen Discovery	Adrian Paskey; Benjamin Rambo-Martin; Chirayu Goswami; Christian Bixby; Christopher Gulvick; Clinton R. Paden; Dakota Howard; Darlene Wagner; Dhwani Batra; Duncan MacCannell; Jason Caravas; Jonathan Schultz; Kara Moser; Matthew Schmerer; Peter W. Cook; Robin Grimwood; Russ Hager; Scott Sammons; Shatavia Morrison; Yihe Wang; Yvette Unoarumhi
EPI_ISL_3031143	Innlandet Hospital Trust, Division Lillehammer, Department for Medical Microbiology	Norwegian Institute of Public Health, Department of Virology	Atiya R Ali; Debech Nadia; Engebretsen Serina Beate; Garcia Llorente Ignacio; Hilde Elshaug; Hilde Vollan; Jon Bråte; Kamilla Heddeland Instefjord; Karoline Bragstad; Kathrine Stene-Johansen; Line Victoria Moen; Marie Paulsen Madsen; Olav Hungnes; Pedersen Benedikte Nevjen; Rasmus Riis Kopperud
EPI_ISL_2761729, EPI_ISL_2761732, EPI_ISL_2956071, EPI_ISL_2956076	Institut für Labormedizin Mikrobiologie und Hygiene	Robert Koch Institute	
EPI_ISL_3426469, EPI_ISL_3453310, EPI_ISL_3504024	Institute of Microbiology and Immunology, Faculty of Medicine, University of Ljubljana	Institute of Microbiology and Immunology, Faculty of Medicine, University of Ljubljana	Alen Suljič; Andraž Celar; Dominika Šturm; Doroteja Vlaj; Mario Poljak; Matic Brvar; Miša Korva; Patricija Pozvek; Samo Zakotnik; Tatjana Avšič – Županc; Tomaž Mark Zorec; Špela Pleh
EPI_ISL_2492351	Institute of Microbiology, Universidad San Francisco de Quito	Institute of Microbiology, Universidad San Francisco de Quito	Belén Prado-Vivar; Bernardo Gutiérrez; Fernanda Zurita; Gabriel Trueba; Juan José Guadalupe; Lofrein Vera; Michelle Grunauer; Monica Becerra-Wong; Patricio Rojas-Silva; Paúl Cárdenas; Sully Márquez; Verónica Barragán
EPI_ISL_2919255, EPI_ISL_3316158, EPI_ISL_3316159, EPI_ISL_3316181, EPI_ISL_3316193, EPI_ISL_3316202, EPI_ISL_3316225			
see above	Instituto Adolfo Lutz - Regional de Bauru	Instituto Adolfo Lutz, Interdiciplinary Procedures Center, Strategic Laboratory	Caio Vinicius Dias Lopes; Claudia Regina Gonçalves; Claudio Tavares Sacchi; Erica Valessa Ramos Gomes; Karoline Rodrigues Campos; Leonardo Tadeu de Araujo; Marlon Benedito Nascomento Santos
EPI_ISL_2828684, EPI_ISL_2828685, EPI_ISL_2919263, EPI_ISL_3259692, EPI_ISL_3259694, EPI_ISL_3259696, EPI_ISL_3259697, EPI_ISL_3259698, EPI_ISL_3259699, EPI_ISL_3259700, EPI_ISL_3259701, EPI_ISL_3259702, EPI_ISL_3259705, EPI_ISL_3259706, EPI_ISL_3259708, EPI_ISL_3259709, EPI_ISL_3259710, EPI_ISL_3259712, EPI_ISL_3259713, EPI_ISL_3259716, EPI_ISL_3259717, EPI_ISL_3545799			
see above	Instituto Adolfo Lutz - Regional de Campinas	Instituto Adolfo Lutz, Interdiciplinary Procedures Center, Strategic Laboratory	Caio Vinicius Dias Lopes; Claudia Regina Gonçalves; Claudio Tavares Sacchi; Erica Valessa Ramos Gomes; Karoline Rodrigues Campos; Leonardo Tadeu de Araujo; Marlon Benedito Nascomento Santos
EPI_ISL_3545788, EPI_ISL_3545789, EPI_ISL_3545796, EPI_ISL_3545800, EPI_ISL_3545801, EPI_ISL_3545802, EPI_ISL_3717840, EPI_ISL_3717841, EPI_ISL_3717842, EPI_ISL_3717843, EPI_ISL_3717844, EPI_ISL_3717845, EPI_ISL_3717846, EPI_ISL_3717847, EPI_ISL_3717848, EPI_ISL_3717849, EPI_ISL_3717850, EPI_ISL_3717851, EPI_ISL_3717853, EPI_ISL_3717854, EPI_ISL_3717855, EPI_ISL_3717857, EPI_ISL_3717858, EPI_ISL_3717859, EPI_ISL_3717860, EPI_ISL_3717861, EPI_ISL_3717864, EPI_ISL_3717866, EPI_ISL_3717868, EPI_ISL_3717869, EPI_ISL_3717870, EPI_ISL_3717871, EPI_ISL_3717872, EPI_ISL_3717873, EPI_ISL_3717874, EPI_ISL_3717875, EPI_ISL_3717887, EPI_ISL_3717889, EPI_ISL_3946613, EPI_ISL_3946614			
see above	Instituto Adolfo Lutz - Regional de Marilia	Instituto Adolfo Lutz, Interdiciplinary Procedures Center, Strategic Laboratory	Caio Vinicius Dias Lopes; Claudia Regina Gonçalves; Claudio Tavares Sacchi; Karoline Rodrigues Campos; Leonardo Tadeu de Araujo; Marlon Benedito Nascimento Santos; Marlon Benedito Nascomento Santos
EPI_ISL_2614617,	Instituto Adolfo Lutz - Regional de	Instituto Adolfo Lutz,	Caio Vinicius Dias Lopes; Claudia Regina Gonçalves; Claudio Tavares Sacchi; Erica Valessa Ramos Gomes; Karoline Rodrigues Campos; Leonardo Jose Tadeu de Araujo; Leonardo Tadeu de Araujo; Marlon Benedito Nascomento Santos

EPI_ISL_3671894	Presidente Prudente	Interdiciplinary Procedures Center, Strategic Laboratory	
EPI_ISL_3671866, EPI_ISL_3671867, EPI_ISL_3671868, EPI_ISL_3671869, EPI_ISL_3671870, EPI_ISL_3671872, EPI_ISL_3671873, EPI_ISL_3671874, EPI_ISL_3671875, EPI_ISL_3671876			
see above	Instituto Adolfo Lutz - Regional de Presidente Prudente	Instituto Adolfo Lutz, Interdiciplinary Procedures Center, Strategic Laboratory	Caio Vinicius Dias Lopes; Claudia Regina Gonçalves; Claudio Tavares Sacchi; Karoline Rodrigues Campos; Leonardo Tadeu de Araujo; Marlon Benedito Nascomento Santos
EPI_ISL_3316164, EPI_ISL_3316167, EPI_ISL_3316171, EPI_ISL_3316178, EPI_ISL_3316183, EPI_ISL_3316192, EPI_ISL_3316240, EPI_ISL_3316243, EPI_ISL_3316301, EPI_ISL_3316307, EPI_ISL_3545793			
see above	Instituto Adolfo Lutz - Regional de Ribeirao Preto	Instituto Adolfo Lutz, Interdiciplinary Procedures Center, Strategic Laboratory	Caio Vinicius Dias Lopes; Claudia Regina Gonçalves; Claudio Tavares Sacchi; Karoline Rodrigues Campos; Leonardo Tadeu de Araujo; Marlon Benedito Nascomento Santos
EPI_ISL_3671877	Instituto Adolfo Lutz - Regional de Ribeirao Preto	Instituto Adolfo Lutz, Interdiciplinary Procedures Center, Strategic Laboratory	Caio Vinicius Dias Lopes; Claudia Regina Gonçalves; Claudio Tavares Sacchi; Karoline Rodrigues Campos; Leonardo Tadeu de Araujo; Marlon Benedito Nascomento Santos
EPI_ISL_3316293, EPI_ISL_3316294, EPI_ISL_3316295, EPI_ISL_3316296, EPI_ISL_3316298, EPI_ISL_3316299	Instituto Adolfo Lutz - Regional de Rio Claro	Instituto Adolfo Lutz, Interdiciplinary Procedures Center, Strategic Laboratory	Caio Vinicius Dias Lopes; Claudia Regina Gonçalves; Claudio Tavares Sacchi; Karoline Rodrigues Campos; Leonardo Tadeu de Araujo; Marlon Benedito Nascomento Santos
EPI_ISL_2828669, EPI_ISL_2828670, EPI_ISL_2828671, EPI_ISL_2828672, EPI_ISL_2828673, EPI_ISL_2828674, EPI_ISL_2828675, EPI_ISL_2828677, EPI_ISL_2828678, EPI_ISL_2828679, EPI_ISL_2828680, EPI_ISL_2828681, EPI_ISL_2828682, EPI_ISL_3717876, EPI_ISL_3717877, EPI_ISL_3717879, EPI_ISL_3717880, EPI_ISL_3717882, EPI_ISL_3717883, EPI_ISL_3717884, EPI_ISL_3717886, EPI_ISL_3717888, EPI_ISL_3946615, EPI_ISL_4056259, EPI_ISL_4056260, EPI_ISL_4056262, EPI_ISL_4056263, EPI_ISL_4056264, EPI_ISL_4056267, EPI_ISL_4056268, EPI_ISL_4056269, EPI_ISL_4056270, EPI_ISL_4056271, EPI_ISL_4056272, EPI_ISL_4056273, EPI_ISL_4056275, EPI_ISL_4056276, EPI_ISL_4056277, EPI_ISL_4056278, EPI_ISL_4056279, EPI_ISL_4056280, EPI_ISL_4056281, EPI_ISL_4056282, EPI_ISL_4056283, EPI_ISL_4056284, EPI_ISL_4056285, EPI_ISL_4056286, EPI_ISL_4056287, EPI_ISL_4056289, EPI_ISL_4056290, EPI_ISL_4056291, EPI_ISL_4056292, EPI_ISL_4056294, EPI_ISL_4056295			
see above	Instituto Adolfo Lutz - Regional de Santo Andre	Instituto Adolfo Lutz, Interdiciplinary Procedures Center, Strategic Laboratory	Caio Vinicius Dias Lopes; Claudia Regina Gonçalves; Claudio Tavares Sacchi; Erica Valessa Ramos Gomes; Karoline Rodrigues Campos; Leonardo Tadeu de Araujo; Marlon Benedito Nascimento Santos
EPI_ISL_3671878	Instituto Adolfo Lutz - Regional de Santo Andre	Instituto Adolfo Lutz, Interdiciplinary Procedures Center, Strategic Laboratory	Caio Vinicius Dias Lopes; Claudia Regina Gonçalves; Claudio Tavares Sacchi; Karoline Rodrigues Campos; Leonardo Tadeu de Araujo; Marlon Benedito Nascomento Santos
EPI_ISL_3671879, EPI_ISL_3671880, EPI_ISL_3671881, EPI_ISL_3671882, EPI_ISL_3671883, EPI_ISL_3671884, EPI_ISL_3671886, EPI_ISL_3671887, EPI_ISL_3671888, EPI_ISL_3671890, EPI_ISL_3671891			
see above	Instituto Adolfo Lutz - Regional de Santos	Instituto Adolfo Lutz, Interdiciplinary Procedures Center, Strategic Laboratory	Caio Vinicius Dias Lopes; Claudia Regina Gonçalves; Claudio Tavares Sacchi; Karoline Rodrigues Campos; Leonardo Tadeu de Araujo; Marlon Benedito Nascomento Santos
EPI_ISL_2919265, EPI_ISL_2919266, EPI_ISL_2919267, EPI_ISL_3259720, EPI_ISL_3259721, EPI_ISL_3259722, EPI_ISL_3259723, EPI_ISL_3259724, EPI_ISL_3259725, EPI_ISL_3259726, EPI_ISL_3259728, EPI_ISL_3316244			
see above	Instituto Adolfo Lutz - Regional de Sao Jose do Rio Preto	Instituto Adolfo Lutz, Interdiciplinary Procedures Center, Strategic Laboratory	Caio Vinicius Dias Lopes; Claudia Regina Gonçalves; Claudio Tavares Sacchi; Erica Valessa Ramos Gomes; Karoline Rodrigues Campos; Leonardo Tadeu de Araujo; Marlon Benedito Nascomento Santos
EPI_ISL_3671892	Instituto Adolfo Lutz - Regional de Sao Jose do Rio Preto	Instituto Adolfo Lutz, Interdiciplinary Procedures Center, Strategic Laboratory	Caio Vinicius Dias Lopes; Claudia Regina Gonçalves; Claudio Tavares Sacchi; Karoline Rodrigues Campos; Leonardo Tadeu de Araujo; Marlon Benedito Nascomento Santos
EPI_ISL_3259678, EPI_ISL_3259680, EPI_ISL_3259682, EPI_ISL_3259683, EPI_ISL_3259685, EPI_ISL_3259686, EPI_ISL_3259688, EPI_ISL_3259690, EPI_ISL_3316161, EPI_ISL_3316165, EPI_ISL_3316184, EPI_ISL_3316201, EPI_ISL_3316237, EPI_ISL_3545790, EPI_ISL_3545791, EPI_ISL_3545797, EPI_ISL_3946602, EPI_ISL_3946603, EPI_ISL_3946605, EPI_ISL_3946607, EPI_ISL_3946611, EPI_ISL_3946612			
see above	Instituto Adolfo Lutz - Regional de Sorocaba	Instituto Adolfo Lutz, Interdiciplinary Procedures Center, Strategic Laboratory	Caio Vinicius Dias Lopes; Claudia Regina Gonçalves; Claudio Tavares Sacchi; Erica Valessa Ramos Gomes; Karoline Rodrigues Campos; Leonardo Tadeu de Araujo; Marlon Benedito Nascomento Santos
EPI_ISL_2919233, EPI_ISL_2919234, EPI_ISL_2919268, EPI_ISL_2919269, EPI_ISL_2919270, EPI_ISL_2919271, EPI_ISL_2919272, EPI_ISL_2919273, EPI_ISL_2919274, EPI_ISL_2919275, EPI_ISL_2919278, EPI_ISL_2919280, EPI_ISL_2919281, EPI_ISL_2919285, EPI_ISL_2919286, EPI_ISL_2919287, EPI_ISL_2919288, EPI_ISL_2919289, EPI_ISL_2919290, EPI_ISL_2919291, EPI_ISL_2919292, EPI_ISL_2919294, EPI_ISL_3259730, EPI_ISL_3259731, EPI_ISL_3259732, EPI_ISL_3259733, EPI_ISL_3259734, EPI_ISL_3259735, EPI_ISL_3259736, EPI_ISL_3259738, EPI_ISL_3259739, EPI_ISL_3259740, EPI_ISL_3259741, EPI_ISL_3259742, EPI_ISL_3259743, EPI_ISL_3259744, EPI_ISL_3259745, EPI_ISL_3259746, EPI_ISL_3259747, EPI_ISL_3259748, EPI_ISL_3259750, EPI_ISL_3259751, EPI_ISL_3259752, EPI_ISL_3259754, EPI_ISL_3259755, EPI_ISL_3259756, EPI_ISL_3259757, EPI_ISL_3259759, EPI_ISL_3259760, EPI_ISL_3259762, EPI_ISL_3259763, EPI_ISL_3259764, EPI_ISL_3316245, EPI_ISL_3316246, EPI_ISL_3316247, EPI_ISL_3316248, EPI_ISL_3316250, EPI_ISL_3316251, EPI_ISL_3316252, EPI_ISL_3316253, EPI_ISL_3316254, EPI_ISL_3316255, EPI_ISL_3316258, EPI_ISL_3316259, EPI_ISL_3316260, EPI_ISL_3316261, EPI_ISL_3316262, EPI_ISL_3316266, EPI_ISL_3316267, EPI_ISL_3316268, EPI_ISL_3316269, EPI_ISL_3316271, EPI_ISL_3316273, EPI_ISL_3316274, EPI_ISL_3316277, EPI_ISL_3316278, EPI_ISL_3316279, EPI_ISL_3316280, EPI_ISL_3316281, EPI_ISL_3316282, EPI_ISL_3316283, EPI_ISL_3316287, EPI_ISL_3316288, EPI_ISL_3316289, EPI_ISL_3316290, EPI_ISL_3316291, EPI_ISL_3316292, EPI_ISL_3545792, EPI_ISL_3545798, EPI_ISL_3545803, EPI_ISL_3671895, EPI_ISL_3671896, EPI_ISL_3671897, EPI_ISL_3671898, EPI_ISL_3671899, EPI_ISL_3671900, EPI_ISL_3671904, EPI_ISL_3671905, EPI_ISL_3691691, EPI_ISL_3691692, EPI_ISL_3691693, EPI_ISL_3691700, EPI_ISL_3710516, EPI_ISL_3717835, EPI_ISL_3717836, EPI_ISL_3717838			
see above	Instituto Adolfo Lutz Central	Instituto Adolfo Lutz, Interdiciplinary Procedures Center, Strategic Laboratory	Caio Vinicius Dias Lopes; Claudia Regina Gonçalves; Claudio Tavares Sacchi; Erica Valessa Ramos Gomes; Karoline Rodrigues Campos; Leonardo Tadeu de Araujo; Marlon Benedito Nascimento Santos; Marlon Benedito Nascomento Santos
EPI_ISL_3671893	Instituto Adolfo Lutz Central	Instituto Adolfo Lutz, Interdiciplinary Procedures Center, Strategic Laboratory	Caio Vinicius Dias Lopes; Claudia Regina Gonçalves; Claudio Tavares Sacchi; Karoline Rodrigues Campos; Leonardo Tadeu de Araujo; Marlon Benedito Nascomento Santos
EPI_ISL_2924058	Instituto Adolfo Lutz Central	Instituto Adolfo Lutz, Rapid Response Center, Strategic Laboratory	Caio Vinicius Dias Lopes; Claudia Regina Gonçalves; Claudio Tavares Sacchi; Erica Valessa Ramos Gomes; Karoline Rodrigues Campos; Leonardo Jose Tadeu de Araujo
EPI_ISL_2777215, EPI_ISL_2777223	Instituto Nacional de Cancerología	Centro de Investigación en Enfermedades Infecciosas (CIENI), Instituto Nacional de Enfermedades Respiratorias (INER)	Alejandra Garcia-Gasca; Alejandra Hernández-Terán; Alejandro Sanchez-Flores; Alfredo Herrera-Estrella; Alicia Ocaña-Mondragón; Andreu Comas-García; Angel Gustavo Salas-Lais; Antonio Loza Román; Bernardo Martínez-Miguel; Blanca Taboada; Brenda Irasema Maldonado-Meza; Bruno Gomez-Gil; Carla Ivón Herrera-Najera; Carlos F. Arias; Celia Boukadida; Clara Esperanza Santacruz-Tinoco; Concepción Grajales-Muñiz; Consorcio Mexicano de Vigilancia Genómica (CoViGen-Mex). Authors (in alphabetical order): Julio Elias Alvarado-Yaah; Cristóbal Cháidez-Quiróz; Célida Duque Molina; Célida Martínez-Rodríguez; Daniel Fregoso-Rueda; Daniel Lira Morales; Eduardo Becerril-Vargas; Fernando Fontove-Herrera; Fidencio Mejía-Nepomuceno; Francisco Pulido; Gloria Elena Espinosa-Ayala; Gloria María Molina-Salinas; Gloria Vazquez; Hector Esteban Paz-Juárez; Hector Montoya-Fuentes; Helen Haydee Fernanda Ramírez-Plascencia; Irvin González-López; Jean Pierre González; Joel Armando Vázquez-Pérez.; Jorge Salas-Hernández; José Antonio Enciso-Moreno; José Arturo Martínez-Orozco; José Esteban Muñoz-Medina; José de Jesús Nuñez-Contreras; Juan Bautista Chale-Dzul; Julissa Enciso-Ibarra; Kathia Elizabeth Tapia-Diaz; Luis Alberto Ochoa-Carrera; Margarita Matías-Florentino; Mario Mújica-Sánchez; Marissa Pérez-García; María Guadalupe Santiago-Mauricio; María Guadalupe de Jesús Mireles-Rivera; Nelly Sélem-Mojica; Pavel Isa; Ricardo Ciria Merce; Ricardo Grande; Rosa María Gutierrez Rios; Santiago Ávila-Ríos; Selene Zárate; Susana Lopez; Victor Eduardo Garcia-Arias; Víctor Hugo Borja-Aburto
EPI_ISL_2648116, EPI_ISL_2648117, EPI_ISL_2777212, EPI_ISL_3268374, EPI_ISL_3268376, EPI_ISL_3268377	Instituto Nacional de Enfermedades Respiratorias (INER)	Centro de Investigación en Enfermedades Infecciosas (CIENI), Instituto Nacional de Enfermedades Respiratorias (INER)	; Alejandra Garcia-Gasca; Alejandra Hernández-Terán; Alejandro Sanchez-Flores; Alfredo Herrera-Estrella; Alicia Ocaña-Mondragón; Andreu Comas-García; Angel Gustavo Salas-Lais; Antonio Loza Román; Bernardo Martínez-Miguel; Blanca Taboada; Brenda Irasema Maldonado-Meza; Bruno Gomez-Gil; Carla Ivón Herrera-Najera; Carlos F. Arias; Celia Boukadida; Clara Esperanza Santacruz-Tinoco; Concepción Grajales-Muñiz; Consorcio Mexicano de Vigilancia Genómica (CoViGen-Mex). Authors (in alphabetical order): Julio Elias Alvarado-Yaah; Cristóbal Cháidez-Quiróz; Célida Duque Molina; Célida Martínez- Rodríguez; Daniel Fregoso-Rueda; Daniel Lira Morales; Eduardo Becerril-Vargas; Fernando Fontove-Herrera; Fidencio Mejía-Nepomuceno; Francisco Pulido; Gloria Elena Espinosa-Ayala; Gloria María Molina-Salinas; Gloria Vazquez; Hector Esteban Paz-Juárez; Hector Montoya-Fuentes; Helen Haydee Fernanda Ramírez-Plascencia; Irvin González-López; Jean Pierre González; Joel Armando Vázquez-Pérez.; Jorge Salas-Hernández; José Antonio Enciso-Moreno; José Arturo Martínez-Orozco; José Esteban Muñoz-Medina; José de Jesús Nuñez-Contreras; Juan Bautista Chale-Dzul; Julissa Enciso-Ibarra; Kathia Elizabeth Tapia-Diaz; Luis Alberto Ochoa-Carrera; Margarita Matías-Florentino; Mario Mújica-Sánchez; Marissa Pérez-García; María Guadalupe Santiago-Mauricio; María Guadalupe de Jesús Mireles-Rivera; Nelly Sélem-Mojica; Pavel Isa; Ricardo Ciria Merce; Ricardo Grande; Rosa María Gutierrez Rios; Santiago Ávila-Ríos; Selene Zárate; Susana Lopez; Victor Eduardo Garcia-Arias; Víctor Hugo Borja-Aburto
EPI_ISL_2810063, EPI_ISL_2894311	Instituto Nacional de Medicina Genomica	Instituto Nacional de Medicina Genomica	Cedro-Tanda A; Cisneros- Villanueva M; Escobar-Arrazola; Gonzalez-Barrera D; Herrera-Montalvo LA.; Hidalgo-Miranda A; M; Mendoza-Vargas A; Munguia-Garza P; Ramirez-Vega O; Rangel-DeLeon D; Reyes-Grajeda JP
EPI_ISL_2843904, EPI_ISL_2843907, EPI_ISL_2843909, EPI_ISL_2843910, EPI_ISL_2843911, EPI_ISL_2843916, EPI_ISL_2843917, EPI_ISL_2843919, EPI_ISL_2843921, EPI_ISL_2843923, EPI_ISL_2843927, EPI_ISL_2843930, EPI_ISL_2843931, EPI_ISL_2843934, EPI_ISL_2843937, EPI_ISL_2843938, EPI_ISL_2843943, EPI_ISL_2843945, EPI_ISL_2843946, EPI_ISL_2843948, EPI_ISL_2843951, EPI_ISL_2843955, EPI_ISL_2843956, EPI_ISL_2843957, EPI_ISL_2843958, EPI_ISL_2843959, EPI_ISL_2843962, EPI_ISL_2843963, EPI_ISL_2843964, EPI_ISL_2843968, EPI_ISL_2843969, EPI_ISL_2843971, EPI_ISL_2843972, EPI_ISL_2843974, EPI_ISL_2843977, EPI_ISL_2843978, EPI_ISL_2843979, EPI_ISL_2843981, EPI_ISL_2843983, EPI_ISL_2843984, EPI_ISL_2843985, EPI_ISL_2843987, EPI_ISL_2843988, EPI_ISL_2843989, EPI_ISL_2843991, EPI_ISL_2843992, EPI_ISL_2843993, EPI_ISL_2843994, EPI_ISL_2843996, EPI_ISL_2843997, EPI_ISL_2843998, EPI_ISL_2843999, EPI_ISL_2844002, EPI_ISL_2844003, EPI_ISL_2844004, EPI_ISL_2844005, EPI_ISL_2844006, EPI_ISL_2844007, EPI_ISL_2844008, EPI_ISL_2844009, EPI_ISL_2844010, EPI_ISL_2844011, EPI_ISL_2844012, EPI_ISL_2844014, EPI_ISL_2844016, EPI_ISL_2844017, EPI_ISL_2844018, EPI_ISL_2844019, EPI_ISL_2844020, EPI_ISL_2844022, EPI_ISL_2844024, EPI_ISL_2844025, EPI_ISL_2844026, EPI_ISL_2844027, EPI_ISL_2844030, EPI_ISL_2844032, EPI_ISL_2844035, EPI_ISL_2844036, EPI_ISL_2844037, EPI_ISL_2844040, EPI_ISL_2844041, EPI_ISL_2844043, EPI_ISL_2844045, EPI_ISL_2844047, EPI_ISL_2844048, EPI_ISL_2844050, EPI_ISL_2844051, EPI_ISL_2844053, EPI_ISL_2844057, EPI_ISL_2844058, EPI_ISL_2844061, EPI_ISL_2844063, EPI_ISL_2844065, EPI_ISL_2844067, EPI_ISL_2844072, EPI_ISL_2844073, EPI_ISL_2844077, EPI_ISL_2844078, EPI_ISL_2844081, EPI_ISL_2844084, EPI_ISL_2844086, EPI_ISL_2844087, EPI_ISL_2844090, EPI_ISL_2844091, EPI_ISL_2844097, EPI_ISL_2844099, EPI_ISL_2844103, EPI_ISL_2844105, EPI_ISL_2844106, EPI_ISL_2844107, EPI_ISL_2844108, EPI_ISL_2844111, EPI_ISL_2844112, EPI_ISL_2844113, EPI_ISL_2844114, EPI_ISL_2844115, EPI_ISL_2844117, EPI_ISL_2844118, EPI_ISL_2844119, EPI_ISL_2844126, EPI_ISL_2844127, EPI_ISL_2844128, EPI_ISL_2844133, EPI_ISL_2844137, EPI_ISL_2844140, EPI_ISL_2844142, EPI_ISL_2844144, EPI_ISL_2844147, EPI_ISL_2844148, EPI_ISL_2844149, EPI_ISL_2844150, EPI_ISL_2844155, EPI_ISL_2844156, EPI_ISL_2844161, EPI_ISL_2844162, EPI_ISL_2844165, EPI_ISL_2844170, EPI_ISL_2844172, EPI_ISL_2844179, EPI_ISL_2844181, EPI_ISL_2844183, EPI_ISL_2844188, EPI_ISL_2844189, EPI_ISL_2844190, EPI_ISL_2844191, EPI_ISL_2844193, EPI_ISL_2844195, EPI_ISL_3030615, EPI_ISL_3118852, EPI_ISL_3118857, EPI_ISL_3118859, EPI_ISL_3118860, EPI_ISL_3118865, EPI_ISL_3118868, EPI_ISL_3118875, EPI_ISL_3118878, EPI_ISL_3118879, EPI_ISL_3118883, EPI_ISL_3118884, EPI_ISL_3118886, EPI_ISL_3118894, EPI_ISL_3118899, EPI_ISL_3118901, EPI_ISL_3118902, EPI_ISL_3118908, EPI_ISL_3118909, EPI_ISL_3118910, EPI_ISL_3118914, EPI_ISL_3118916, EPI_ISL_3118923, EPI_ISL_3118924, EPI_ISL_3118925, EPI_ISL_3118928, EPI_ISL_3118932, EPI_ISL_3118936, EPI_ISL_3118938, EPI_ISL_3118939, EPI_ISL_3118941, EPI_ISL_3118943, EPI_ISL_3118945, EPI_ISL_3244532, EPI_ISL_3244533, EPI_ISL_3244534, EPI_ISL_3244536, EPI_ISL_3244537, EPI_ISL_3244539, EPI_ISL_3244540, EPI_ISL_3244541, EPI_ISL_3244542, EPI_ISL_3244544, EPI_ISL_3244552, EPI_ISL_3244555, EPI_ISL_3244557, EPI_ISL_3244561, EPI_ISL_3244562, EPI_ISL_3244566, EPI_ISL_3244572, EPI_ISL_3244573, EPI_ISL_3244574, EPI_ISL_3244575, EPI_ISL_3244579, EPI_ISL_3244584, EPI_ISL_3244588, EPI_ISL_3244592, EPI_ISL_3244594			
see above	Instituto de Biotecnologia - UNESP-Botucatu-SP	Instituto de Biotecnologia - UNESP-Botucatu-SP	Cecília Artico Banho; Cíntia Bittar; Fábio Sossai Possebon; Guilherme Campos; Helena Lage Ferreira; Jorge A. Petroli Marchesi; João Pessoa Araújo Jr.; Leila Sabrina Ullmann; Lívia Sacchetto; Maisa C. Pereira Parra; Marília Moraes; Maurício L. Nogueira; Paula Rahal; Paulo Inacio da Costa
EPI_ISL_3236442, EPI_ISL_3236443	Instituto de Medicina Tropical & Salud Global Universidad Iberoamericana	Grubaugh Lab - Yale School of Public Health	Alejandaro Vallejo Degaudenzi; Anderson Brito; Annie Watkins; Chaney Kalinich; Chantal Vogels; Elisa Contreras; Esperanza Mendoza; Isabel Ott; Jessica Rothman; Joseph Fauver; Kendall Billig; Mallery Breban; Mary Petrone; Nathan Grubaugh; Robert Paulino-Ramirez; Tara Alpert; Tobias Koch; Victor Virgilio Calderon
EPI_ISL_3020244, EPI_ISL_3020245	Instituto de Medicina Tropical Alexander Von Humboldt, Sede Cusco	Laboratorio de Genómica Microbiana, Universidad Peruana Cayetano Heredia	Alejandra Dávila-Barclay; Diego Cuicapuza; Guillermo Salvatierra; Janet Huancachoque; Luis González; Pablo Tsukayama; Pedro E. Romero; Pool Marcos
EPI_ISL_3105054, EPI_ISL_3347068, EPI_ISL_3347084, EPI_ISL_3347092	Interpath Laboratory	Seattle Flu Study	Amanda Adler; Barry R. Lutz; Benjamin Pelle; Brian Hiatt; Caitlin R. Wolf; Chris D. Frazar; Deborah A. Nickerson; Elisabeth Brandstetter; Erica Ryke; Geoff Melly; Helen Y. Chu; Janet A. Englund; Jay Shendure; Jover Lee; Kairsten Fay; Kirsten Lacombe; Lea M. Starita; Mark J. Rieder; Matthew Richardson; Matthew Thompson; Melissa Truong; Michael Boeckh; Michael Famulare; Misja Ilcisin; Peter D. Han; Philip Dykema; Romesh Gautom; Scott Lindquist; Thomas R. Sibley; Trevor Bedford
EPI_ISL_3230430	Islab, Etelä-Savon aluelaboratorio	Expert Microbiology, National Institute for Health and Welfare	Carita Savolainen-Kopra; Erika Lindh; Haider al-Hello; Jani Halkilahti; Kirsi Liitsola; Niina Ikonen; Olli Vapalahti; Pekka Ellonen; Phuoc Truong; Päivi Laurila; Ravi Kant; Sari Hannula; Soile Blomqvist; Teemu Smura
EPI_ISL_2645182	Islab, Pohjois-Karjalan aluelaboratorio	Expert Microbiology, National Institute for Health and Welfare	Carita Savolainen-Kopra; Erika Lindh; Haider al-Hello; Jani Halkilahti; Kirsi Liitsola; Niina Ikonen; Olli Vapalahti; Pekka Ellonen; Phuoc Truong; Päivi Laurila; Ravi Kant; Sari Hannula; Soile Blomqvist; Teemu Smura
EPI_ISL_3230614,	Islab, Pohjois-Savon aluelaboratorio	Expert Microbiology, National	Carita Savolainen-Kopra; Erika Lindh; Haider al-Hello; Jani Halkilahti; Kirsi Liitsola; Niina Ikonen; Olli Vapalahti; Pekka Ellonen; Phuoc Truong; Päivi Laurila; Ravi Kant; Sari Hannula; Soile Blomqvist; Teemu Smura

EPI_ISL_3230615		Institute for Health and Welfare	
EPI_ISL_3279359	Israel Central Virology laboratory	Israel National Consortium for SARS-CoV-2 sequencing	Dana Bar-Ilan; Danit Sofer; Efrat Dahan Bucris; Efrat Glick-Saar; Ella Mendelson; Gideon Rechavi; Michal Mandelboim; Miranda Geva; Neta Zuckerman; Omer Asraf; Omri Nayshool; Oran Erster; Orna Mor
EPI_ISL_2692081, EPI_ISL_2692084	John H Stroger Jr Hospital of Cook County	RIPHL at Rush University Medical Center	Ah-Young Sung; Felix Araujo Perez; Kevin Kunstman; Laura Furtado; Marieta Hyde; Max Kolton; Sharon Welbel; Stefan Green
EPI_ISL_2690054	KS Health and Environmental Laboratories	Centers for Disease Control and Prevention Division of Viral Diseases, Pathogen Discovery	Alison Laufer Halpin; Ben L. Rambo-Martin; Clinton R. Paden; Dakota Howard; Darlene Wagner; Dave Wentworth; Dhwani Batra; Jasmine Padilla; Justin Lee; Katie Dillon; Krista Queen; Kristen Knipe; Kristine Lacek; Mark Burroughs; Matthew Schmerer; Mili Sheth; Peter Cook; Sam Shepard; Sarah Nobles; Shoshona Le; Suxiang Tong; Vivien Dugan; Yvette Unoarumhi
EPI_ISL_3561409, EPI_ISL_3561424, EPI_ISL_3561441, EPI_ISL_3561827, EPI_ISL_3825803, EPI_ISL_4102639, EPI_ISL_4102687			
see above	Kaiser Permanente NW Reginal Lab	OHSU MM Lab	Amber Halse; Jeannine Lama; Xuan Qin; Yun Wu
EPI_ISL_2443131, EPI_ISL_2455455, EPI_ISL_2558702, EPI_ISL_2833925	Kansas Health and Environmental Lab	Kansas Health and Environmental Lab	Ben Olsen; Jonathan Barnell; Katherine Wiggins; Mike Grose; and Phil Adam
EPI_ISL_2898394, EPI_ISL_2899349	Karolinska University Hospital Solna	Karolinska University Hospital	Annelie Bjerkner; Isak Sylvin; Jan Albert; Karolina Ininbergs; Lina Guerra Blomqvist; Lynda Eneh; Martin Ekman; Martina Wahlund; Robert Dyrdak; Sandra Broddesson; Tanja Normark; Tobias Allander; Valtteri Wirta; Zhibing Yun
EPI_ISL_4080592	Kentucky State Public Health Lab	Kentucky State Public Health Lab	Anjayya Tummala; Chitralipi Kattunga; Joshua Tobias; Karim George; Matthew Johnson; Rachel Zinner; Rhonda Lucas; Vaneet Arora; William Grooms
EPI_ISL_2483832	Klinisch Laboratorium ZNA	Klinisch Laboratorium ZNA	Verstrepen et al.
EPI_ISL_3130541	LABORATOIRE CERBALLIANCE PLT VILLON	CNR Virus des Infections Respiratoires - France SUD	Antonin Bal; Bruno Lina; Gregory Destras; Gwendolyne Burfin; Hadrien Regue; Laurence Josset; Martine Valette; Quentin Semanas
EPI_ISL_2494141, EPI_ISL_2494142, EPI_ISL_2494143	LABORATORIO DE FRANCA	Instituto Butantan	Antonio Jorge Martins; Claudia Renata dos Santos Barros; David Schlesinger; Debora Botequio Moretti; Dimas Tadeu Covas; Elaine Cristina Marqueze; Elaine Vieira Santos; Evandra Strazza Rodrigues; Heidge Fukumasu; Jayme Augusto de Souza-Neto; José Salvatore Leister Patané; Luiz Alcantara; Luiz Lehmann Coutinho; Maria Carolina Elias; Maurício Lacerda Nogueira; Rafael dos Santos Bezerra; Raul Machado Neto; Rejane Maria Tommasini Grotto; Ricardo Haddad; Sandra Coccuzzo Sampaio Vessoni; Simone Kashima; Svetoslav Nanev Slavov; Vincent Louis Viala
EPI_ISL_2494166, EPI_ISL_2494176, EPI_ISL_2494184	LABORATORIO MUNICIPAL DE ANALISES CLINICAS DE RIO CLARO	Instituto Butantan	Antonio Jorge Martins; Claudia Renata dos Santos Barros; David Schlesinger; Debora Botequio Moretti; Dimas Tadeu Covas; Elaine Cristina Marqueze; Elaine Vieira Santos; Evandra Strazza Rodrigues; Heidge Fukumasu; Jayme Augusto de Souza-Neto; José Salvatore Leister Patané; Luiz Alcantara; Luiz Lehmann Coutinho; Maria Carolina Elias; Maurício Lacerda Nogueira; Rafael dos Santos Bezerra; Raul Machado Neto; Rejane Maria Tommasini Grotto; Ricardo Haddad; Sandra Coccuzzo Sampaio Vessoni; Simone Kashima; Svetoslav Nanev Slavov; Vincent Louis Viala
EPI_ISL_2494163, EPI_ISL_2494182, EPI_ISL_2494200, EPI_ISL_2494338	LABORATORIO MUNICIPAL DE PIRACICABA	Instituto Butantan	Antonio Jorge Martins; Claudia Renata dos Santos Barros; David Schlesinger; Debora Botequio Moretti; Dimas Tadeu Covas; Elaine Cristina Marqueze; Elaine Vieira Santos; Evandra Strazza Rodrigues; Heidge Fukumasu; Jayme Augusto de Souza-Neto; José Salvatore Leister Patané; Luiz Alcantara; Luiz Lehmann Coutinho; Maria Carolina Elias; Maurício Lacerda Nogueira; Rafael dos Santos Bezerra; Raul Machado Neto; Rejane Maria Tommasini Grotto; Ricardo Haddad; Sandra Coccuzzo Sampaio Vessoni; Simone Kashima; Svetoslav Nanev Slavov; Vincent Louis Viala
EPI_ISL_2493881, EPI_ISL_2493884, EPI_ISL_2493885, EPI_ISL_2493886, EPI_ISL_2493887, EPI_ISL_2493906, EPI_ISL_2493908, EPI_ISL_2493948, EPI_ISL_2500987			
see above	LABORATORIO MUNICIPAL DE SUZANO	Instituto Butantan	Antonio Jorge Martins; Claudia Renata dos Santos Barros; David Schlesinger; Debora Botequio Moretti; Dimas Tadeu Covas; Elaine Cristina Marqueze; Elaine Vieira Santos; Evandra Strazza Rodrigues; Heidge Fukumasu; Jayme Augusto de Souza-Neto; José Salvatore Leister Patané; Luiz Alcantara; Luiz Lehmann Coutinho; Maria Carolina Elias; Maurício Lacerda Nogueira; Rafael dos Santos Bezerra; Raul Machado Neto; Rejane Maria Tommasini Grotto; Ricardo Haddad; Sandra Coccuzzo Sampaio Vessoni; Simone Kashima; Svetoslav Nanev Slavov; Vincent Louis Viala
EPI_ISL_2895184	LAC UCoimbra	Instituto Nacional de Saude (INSA)	Borges et al
EPI_ISL_3404852, EPI_ISL_3404856	LACEN	LGBio (Laboratorio de Genetica & Biodiversidade)	Alex Honda Bernardes; Amanda Alves de Melo; Aparecido Divino da Cruz; Cintia Pelegrineti Targueta de Azevedo Brito; Daniela de Melo e Silva; Elisangela de Paula Silveira Lacerda; Juliana Santana de Curcio; Luiz Augusto Pereira; Marc Alexandre Duarte Gigonzac; Mariana Pires de Campos Telles; Ramilla dos Santos Braga; Renata de Oliveira Dias; Rhewter Nunes; Thais Cidália Vieira Gigonzac; Thais Guimarães Castro; Thays Millena Alves Pedroso
EPI_ISL_3671907, EPI_ISL_3946592, EPI_ISL_3946593, EPI_ISL_3946594	LACEN do Distrito Federal	Instituto Adolfo Lutz, Interdiciplinary Procedures Center, Strategic Laboratory	Caio Vinicius Dias Lopes; Claudia Regina Gonçalves; Claudio Tavares Sacchi; Karoline Rodrigues Campos; Leonardo Tadeu de Araujo; Marlon Benedito Nascomento Santos
EPI_ISL_2894548, EPI_ISL_2896234, EPI_ISL_2896235, EPI_ISL_2896236, EPI_ISL_2896238, EPI_ISL_2896239, EPI_ISL_2896240, EPI_ISL_2896241			
see above	LACEN do Estado de Mato Grosso	Instituto Adolfo Lutz, Interdiciplinary Procedures Center, Strategic Laboratory	Caio Vinicius Dias Lopes; Claudia Regina Gonçalves; Claudio Tavares Sacchi; Karoline Rodrigues Campos
EPI_ISL_3545804, EPI_ISL_3545805, EPI_ISL_3691360, EPI_ISL_3691362	LACEN do Estado do Mato Grosso do Sul	Instituto Adolfo Lutz, Interdiciplinary Procedures Center, Strategic Laboratory	Caio Vinicius Dias Lopes; Claudia Regina Gonçalves; Claudio Tavares Sacchi; Karoline Rodrigues Campos; Leonardo Tadeu de Araujo; Marlon Benedito Nascomento Santos
EPI_ISL_3835195, EPI_ISL_3835196, EPI_ISL_3835197, EPI_ISL_3835198, EPI_ISL_3835200, EPI_ISL_3835201, EPI_ISL_3835202, EPI_ISL_3835204, EPI_ISL_3835205, EPI_ISL_3835209, EPI_ISL_3835210, EPI_ISL_3835211, EPI_ISL_3835212, EPI_ISL_3835213, EPI_ISL_3835215, EPI_ISL_3835216, EPI_ISL_3835217, EPI_ISL_3835218, EPI_ISL_3835219, EPI_ISL_3835220, EPI_ISL_3835222, EPI_ISL_3835224, EPI_ISL_3835225, EPI_ISL_3835229, EPI_ISL_3835230, EPI_ISL_3835231, EPI_ISL_3835233, EPI_ISL_3835234, EPI_ISL_3835235, EPI_ISL_3835237, EPI_ISL_3835238, EPI_ISL_3835239			
see above	LACEN/PB	WallauLab on behalf of Fiocruz COVID-19 Genomic Surveillance Network	Alexandre Freitas da Silva; Cassia Docena; Constância Flávia Junqueira Ayres; Filipe Zimmer Dezordi; Gabriel Luz Wallau; Gustavo Barbosa de Lima; Lais Ceschini Machado; Lilian Caroliny Amorim Silva; Maira Galdino da Rocha Pitta; Marcelo Henrique dos Santos Paiva; Matheus Filgueira Bezerra; Michelly Cristiny Pereira; Rômulo Pessoa e Silva; Sinval Pinto Brandão Filho
EPI_ISL_3046268, EPI_ISL_3046270, EPI_ISL_3046272, EPI_ISL_3046273, EPI_ISL_3046275, EPI_ISL_3046277, EPI_ISL_3046278, EPI_ISL_3046280, EPI_ISL_3046281, EPI_ISL_3046282, EPI_ISL_3046283, EPI_ISL_3046284, EPI_ISL_3046285, EPI_ISL_3046286, EPI_ISL_3046287, EPI_ISL_3046288, EPI_ISL_3046290, EPI_ISL_3046291, EPI_ISL_3046292, EPI_ISL_3046293, EPI_ISL_3046294, EPI_ISL_3046295, EPI_ISL_3046296, EPI_ISL_3046297, EPI_ISL_3046299, EPI_ISL_3046300, EPI_ISL_3046301, EPI_ISL_3046305, EPI_ISL_3046306, EPI_ISL_3046307, EPI_ISL_3046308, EPI_ISL_3046309, EPI_ISL_3046313, EPI_ISL_3046314, EPI_ISL_3046315, EPI_ISL_3046316, EPI_ISL_3046317, EPI_ISL_3046318, EPI_ISL_3046320, EPI_ISL_3046322, EPI_ISL_3046323, EPI_ISL_3046325, EPI_ISL_3046326, EPI_ISL_3046332, EPI_ISL_3046338, EPI_ISL_3046339, EPI_ISL_3046344, EPI_ISL_3046345, EPI_ISL_3134665, EPI_ISL_3134667, EPI_ISL_3134669, EPI_ISL_3134672, EPI_ISL_3134676, EPI_ISL_3134680, EPI_ISL_3134681, EPI_ISL_3134682, EPI_ISL_3134683, EPI_ISL_3134684, EPI_ISL_3134685, EPI_ISL_3134687, EPI_ISL_3134688, EPI_ISL_3134689, EPI_ISL_3134690, EPI_ISL_3134691, EPI_ISL_3134692, EPI_ISL_3134693, EPI_ISL_3134695, EPI_ISL_3134696, EPI_ISL_3134698, EPI_ISL_3134701, EPI_ISL_3134702, EPI_ISL_3134703, EPI_ISL_3134704, EPI_ISL_3134705, EPI_ISL_3134706, EPI_ISL_3134707, EPI_ISL_3134708, EPI_ISL_3134709, EPI_ISL_3134710, EPI_ISL_3134715, EPI_ISL_3134716, EPI_ISL_3134717, EPI_ISL_3134718, EPI_ISL_3134719, EPI_ISL_3134720, EPI_ISL_3134721, EPI_ISL_3134834, EPI_ISL_3134858, EPI_ISL_3447469, EPI_ISL_3447470, EPI_ISL_3447472, EPI_ISL_3447475, EPI_ISL_3447478, EPI_ISL_3447479, EPI_ISL_3447487, EPI_ISL_3447488, EPI_ISL_3447600			
see above	LACEN/PE	WallauLab on behalf of Fiocruz COVID-19 Genomic Surveillance Network	Alexandre Freitas da Silva; Cassia Docena; Constância Flávia Junqueira Ayres; Filipe Zimmer Dezordi; Gabriel Luz Wallau; Gustavo Barbosa de Lima; Lais Ceschini Machado; Lilian Caroliny Amorim Silva; Marcelo Henrique dos Santos Paiva; Matheus Filgueira Bezerra; Sinval Pinto Brandão Filho
EPI_ISL_3149318, EPI_ISL_3149319, EPI_ISL_3149320, EPI_ISL_3149321	LANIIA	LANIIA-Nayarit	; Alejandra García-Gasca; Alejandra Hernández-Terán; Alejandro Sánchez-Flores; Alfredo Herrera-Estrella; Alicia Ocaña-Mondragón; Andreu Comas-García; Angel Gustavo Salas-Lais; Antonio Loza Román; Bernardo Martínez-Miguel; Blanca Taboada; Brenda Irasema Maldonado-Meza; Bruno Gómez-Gil; Carla Ivón Herrera-Najera; Carlos Eduardo Covantes-Rosales; Carlos F. Arias; Celia Boukadida; Clara Esperanza Santacruz-Tinoco; Concepción Grajales-Muñiz; Consorcio Mexicano de Vigilancia Genómica (CoViGen-Mex). Authors (in alphabetical order): Julio Elias Alvarado-Yaah; Cristóbal Cháidez-Quiróz; Célida Duque Molina; Célida Martínez- Rodríguez; Daniel Alberto Girón Pérez; Daniel Fregoso-Rueda; Daniel Lira Morales; Eduardo Becerril-Vargas; Fernando Fontove-Herrera; Fidencio Mejía-Nepomuceno; Francisco Pulido; Gloria Elena Espinosa-Ayala; Gloria María Molina-Salinas; Gloria Vazquez; Hector Esteban Paz-Juárez; Hector Montoya-Fuentes; Helen Haydee Fernanda Ramírez-Plascencia; Irvin González-López; Jean Pierre González; Jesús Hernández; Joel Armando Vázquez-Pérez. LANNIA Nayarit: Manuel Ivan Girón Pérez; Jorge Salas-Hernández; José Antonio Enciso-Moreno; José Arturo Martínez-Orozco; José Esteban Muñoz-Medina; José de Jesús Nuñez-Contreras; Juan Bautista Chale-Dzul; Julissa Enciso-Ibarra; Luis Alberto Ochoa-Carrera; Margarita Matías-Florentino; Mario Mújica-Sánchez; Marissa Perez-Garcia; María Guadalupe Santiago-Mauricio; María Guadalupe de Jesús Mireles-Rivera; Nelly Sélem-Mojica; Pavel Isa; Ricardo Ciria Merce; Ricardo Grande; Rosa María Gutiérrez Rios; Santiago Ávila-Ríos; Selene Zárate; Susana Lopez; Ulises Mercado.; Verónica Mata-Haro; Victor Eduardo García-Arias; Víctor Hugo Borja-Aburto
EPI_ISL_3010759, EPI_ISL_3010760, EPI_ISL_3010764, EPI_ISL_3010768, EPI_ISL_3010776, EPI_ISL_3010777, EPI_ISL_3010781, EPI_ISL_3010783, EPI_ISL_3010784, EPI_ISL_3010791, EPI_ISL_3010793, EPI_ISL_3010794, EPI_ISL_3010798, EPI_ISL_3010800, EPI_ISL_3010801, EPI_ISL_3010802, EPI_ISL_3010803, EPI_ISL_3010805, EPI_ISL_3010806, EPI_ISL_3010807, EPI_ISL_3010808, EPI_ISL_3010809, EPI_ISL_3010816, EPI_ISL_3010817, EPI_ISL_3010818, EPI_ISL_3010819, EPI_ISL_3010823, EPI_ISL_3010824, EPI_ISL_3010825, EPI_ISL_3010826, EPI_ISL_3010827, EPI_ISL_3010831, EPI_ISL_3010832, EPI_ISL_3010833, EPI_ISL_3010835, EPI_ISL_3010836, EPI_ISL_3010841, EPI_ISL_3010842, EPI_ISL_3010843, EPI_ISL_3010845, EPI_ISL_3010846, EPI_ISL_3010847, EPI_ISL_3010848, EPI_ISL_3010853, EPI_ISL_3010856, EPI_ISL_3010859, EPI_ISL_3010860, EPI_ISL_3010861, EPI_ISL_3010863, EPI_ISL_3010864, EPI_ISL_3010866, EPI_ISL_3010870, EPI_ISL_3010871, EPI_ISL_3010874, EPI_ISL_3010875, EPI_ISL_3010876, EPI_ISL_3010877, EPI_ISL_3010879, EPI_ISL_3010881, EPI_ISL_3010886, EPI_ISL_3010892, EPI_ISL_3010893, EPI_ISL_3010894, EPI_ISL_3010895, EPI_ISL_3010896, EPI_ISL_3010898, EPI_ISL_3010900, EPI_ISL_3010901, EPI_ISL_3010902, EPI_ISL_3010905, EPI_ISL_3010907, EPI_ISL_3010910, EPI_ISL_3010911, EPI_ISL_3010912, EPI_ISL_3010914, EPI_ISL_3010917, EPI_ISL_3010918, EPI_ISL_3010920, EPI_ISL_3010921, EPI_ISL_3010923, EPI_ISL_3010924, EPI_ISL_3010926, EPI_ISL_3010934, EPI_ISL_3047701, EPI_ISL_3047702, EPI_ISL_3047704, EPI_ISL_3047706, EPI_ISL_3047707, EPI_ISL_3047708, EPI_ISL_3047709, EPI_ISL_3047711, EPI_ISL_3047712, EPI_ISL_3047713, EPI_ISL_3047715, EPI_ISL_3047716, EPI_ISL_3047719, EPI_ISL_3047720, EPI_ISL_3047721, EPI_ISL_3047726, EPI_ISL_3047727, EPI_ISL_3047728, EPI_ISL_3047729, EPI_ISL_3047730, EPI_ISL_3047731, EPI_ISL_3047733, EPI_ISL_3047734, EPI_ISL_3047735, EPI_ISL_3047738, EPI_ISL_3047741, EPI_ISL_3047742, EPI_ISL_3047743, EPI_ISL_3047744, EPI_ISL_3047745, EPI_ISL_3134921, EPI_ISL_3134922, EPI_ISL_3134930, EPI_ISL_3134934, EPI_ISL_3134939, EPI_ISL_3134950, EPI_ISL_3134955, EPI_ISL_3134960, EPI_ISL_3134965, EPI_ISL_3134967, EPI_ISL_3134977, EPI_ISL_3246189, EPI_ISL_3261062, EPI_ISL_3261063, EPI_ISL_3261064, EPI_ISL_3261065, EPI_ISL_3261067, EPI_ISL_3261068, EPI_ISL_3261071, EPI_ISL_3261072, EPI_ISL_3261073, EPI_ISL_3261074, EPI_ISL_3261075, EPI_ISL_3261076, EPI_ISL_3261077, EPI_ISL_3261078, EPI_ISL_3261079, EPI_ISL_3261080, EPI_ISL_3261081, EPI_ISL_3261083, EPI_ISL_3261084, EPI_ISL_3261086, EPI_ISL_3261087, EPI_ISL_3261090, EPI_ISL_3261091, EPI_ISL_3261092, EPI_ISL_3261093, EPI_ISL_3261094, EPI_ISL_3261095, EPI_ISL_3261096, EPI_ISL_3261097, EPI_ISL_3261099, EPI_ISL_3261100, EPI_ISL_3261101, EPI_ISL_3261102, EPI_ISL_3261104, EPI_ISL_3261105, EPI_ISL_3261106, EPI_ISL_3261109, EPI_ISL_3261110, EPI_ISL_3261113, EPI_ISL_3261115, EPI_ISL_3267907, EPI_ISL_3267908, EPI_ISL_3267909, EPI_ISL_3267910, EPI_ISL_3267911, EPI_ISL_3267912, EPI_ISL_3267917, EPI_ISL_3267918, EPI_ISL_3267919, EPI_ISL_3267921, EPI_ISL_3267922, EPI_ISL_3267923, EPI_ISL_3267924, EPI_ISL_3267928, EPI_ISL_3267930, EPI_ISL_3267933, EPI_ISL_3267934, EPI_ISL_3267935, EPI_ISL_3267936, EPI_ISL_3267940, EPI_ISL_3267942, EPI_ISL_3267943, EPI_ISL_3267945, EPI_ISL_3267947, EPI_ISL_3267948, EPI_ISL_3267949, EPI_ISL_3267950, EPI_ISL_3267951, EPI_ISL_3267953, EPI_ISL_3267954, EPI_ISL_3267955, EPI_ISL_3267957, EPI_ISL_3267958, EPI_ISL_3267959, EPI_ISL_3267960, EPI_ISL_3267961, EPI_ISL_3267962, EPI_ISL_3267963, EPI_ISL_3267965, EPI_ISL_3267967, EPI_ISL_3267969, EPI_ISL_3267970, EPI_ISL_3267972, EPI_ISL_3267973, EPI_ISL_3267975, EPI_ISL_3267976, EPI_ISL_3267978, EPI_ISL_3267980, EPI_ISL_3267982, EPI_ISL_3267983, EPI_ISL_3267985, EPI_ISL_3267987, EPI_ISL_3267988, EPI_ISL_3267989, EPI_ISL_3267990, EPI_ISL_3267991, EPI_ISL_3267993, EPI_ISL_3267994, EPI_ISL_3267995, EPI_ISL_3267996, EPI_ISL_3267997, EPI_ISL_3267999, EPI_ISL_3268001, EPI_ISL_3268002, EPI_ISL_3268003, EPI_ISL_3268004, EPI_ISL_3268005, EPI_ISL_3268006, EPI_ISL_3268007, EPI_ISL_3268010, EPI_ISL_3268011, EPI_ISL_3268012, EPI_ISL_3268014, EPI_ISL_3268016, EPI_ISL_3268017, EPI_ISL_3268018, EPI_ISL_3268019, EPI_ISL_3268020, EPI_ISL_3268023, EPI_ISL_3304422			
see above	LATE - Laboratório de Técnicas Especiais - Hospital Israelita Albert Einstein	LATE - Laboratório de Técnicas Especiais - Hospital Israelita Albert Einstein	Alexandre Hideaki Takara; Ana Paula Moreira Salles; Anelisie da Silva Santos; Deyvid Amgarten; Erick Gustavo Dorlass; Fernanda de Mello Malta; João Renato Rebello Pinho; Marcio Anunciacao Menezes; Pedro Henrique Sebe Rodrigues; Raquel Riyuzo
EPI_ISL_2841778, EPI_ISL_2841782, EPI_ISL_2841785, EPI_ISL_2841787	LBM ALPHABIO, Marseille	LBM ALPHABIO, Marseille	Vincent GARCIA
EPI_ISL_2827783	LDSP - CAQUETA HOSPITAL MALVINAS	Instituto Nacional de Salud-Dirección de Investigación en Salud Pública	Carlos Franco-Muñoz; Carmen Osorio; Diana Malo; Diego A. Álvarez-Díaz; Diego Andrés Prada; Gerardo Santamaría; Hector Alejandro Ruiz-Moreno; Jhonnatan Reales-González; Jorge Rivera; Juan Camilo Martinez; Julian Naizaque; Katherine Laiton-Donato; Lisseth Pardo; Magdalena Wiesner; Marcela Mercado-Reyes; Maria T. Herrera-Sepúlveda; Marta Lopez Blanco; Martha Lucia Ospina Martinez; Paola Rojas; Sergio Gomez; Sheryll Corchuelo; Ángela Alarcon Cruz
EPI_ISL_2827779	LDSP - CAQUETA HOSPITAL MARIA INMACULADA	Instituto Nacional de Salud-Dirección de Investigación en Salud Pública	Carlos Franco-Muñoz; Carmen Osorio; Diana Malo; Diego A. Álvarez-Díaz; Diego Andrés Prada; Gerardo Santamaría; Hector Alejandro Ruiz-Moreno; Jhonnatan Reales-González; Jorge Rivera; Juan Camilo Martinez; Julian Naizaque; Katherine Laiton-Donato; Lisseth Pardo; Magdalena Wiesner; Marcela Mercado-Reyes; Maria T. Herrera-Sepúlveda; Marta Lopez Blanco; Martha Lucia Ospina Martinez; Paola Rojas; Sergio Gomez; Sheryll Corchuelo; Ángela Alarcon Cruz
EPI_ISL_3007486	LDSP VILLAVICENCIO - CLINICA PRIMAVERA DE VILLAVICENCIO	Instituto Nacional de Salud-Dirección de Investigación en Salud Pública	Carlos Franco-Muñoz; Carmen Osorio; Diana Malo; Diego A. Álvarez-Díaz; Diego Andrés Prada; Gerardo Santamaría; Hector Alejandro Ruiz-Moreno; Jhonnatan Reales-González; Jorge Rivera; Juan Camilo Martinez; Julian Naizaque; Katherine Laiton-Donato; Lisseth Pardo; Magdalena Wiesner; Marcela Mercado-Reyes; Maria T. Herrera-Sepúlveda; Marta Lopez Blanco; Martha Lucia Ospina Martinez; Paola Rojas; Sergio Gomez; Sheryll Corchuelo; Ángela Alarcon Cruz
EPI_ISL_2835071,	LESP Aguascalientes	Instituto de Diagnostico y	Abril Rodriguez-Maldonado; Ariadna Medina-Benitez; Claudia Wong-Arambula; Ernesto Ramirez-Gonzalez.; Gisela Barrera-Badillo; Irma Lopez-Martinez; Joaquin Quiroz-Mercado; Lucia Hernandez-Rivas; Maribel Gonzalez-Villa; Natividad Cruz-Ortiz; Sergio Rangel-Guerrero; Tatiana Nunez-Garcia; Vanessa

EPI_ISL_3265588		Referencia Epidemiologicos (INDRE)	Rivero-Arredondo
EPI_ISL_3033578	LESP Baja California Sur	Instituto de Diagnostico y Referencia Epidemiologicos (INDRE)	Abril Rodriguez-Maldonado; Ariadna Medina-Benitez; Claudia Wong-Arambula; Ernesto Ramirez-Gonzalez.; Gisela Barrera-Badillo; Irma Lopez-Martinez; Joaquin Quiroz-Mercado; Lucia Hernandez-Rivas; Maribel Gonzalez-Villa; Natividad Cruz-Ortiz; Sergio Rangel-Guerrero; Tatiana Nunez-Garcia; Vanessa Rivero-Arredondo
EPI_ISL_3265618	LESP Campeche	Instituto de Diagnostico y Referencia Epidemiologicos (INDRE)	Abril Rodriguez-Maldonado; Ariadna Medina-Benitez; Claudia Wong-Arambula; Ernesto Ramirez-Gonzalez.; Gisela Barrera-Badillo; Irma Lopez-Martinez; Joaquin Quiroz-Mercado; Lucia Hernandez-Rivas; Maribel Gonzalez-Villa; Natividad Cruz-Ortiz; Sergio Rangel-Guerrero; Tatiana Nunez-Garcia; Vanessa Rivero-Arredondo
EPI_ISL_2859010, EPI_ISL_2859011	LESP Chiapas	Instituto de Diagnostico y Referencia Epidemiologicos (INDRE)	Abril Rodriguez-Maldonado; Ariadna Medina-Benitez; Claudia Wong-Arambula; Ernesto Ramirez-Gonzalez.; Gisela Barrera-Badillo; Irma Lopez-Martinez; Joaquin Quiroz-Mercado; Lucia Hernandez-Rivas; Maribel Gonzalez-Villa; Natividad Cruz-Ortiz; Sergio Rangel-Guerrero; Tatiana Nunez-Garcia; Vanessa Rivero-Arredondo
EPI_ISL_2859062, EPI_ISL_2937769, EPI_ISL_2937770, EPI_ISL_2937804, EPI_ISL_2937805, EPI_ISL_3033593, EPI_ISL_3033594			
see above	LESP Ciudad de Mexico	Instituto de Diagnostico y Referencia Epidemiologicos (INDRE)	Abril Rodriguez-Maldonado; Ariadna Medina-Benitez; Claudia Wong-Arambula; Ernesto Ramirez-Gonzalez.; Gisela Barrera-Badillo; Irma Lopez-Martinez; Joaquin Quiroz-Mercado; Lucia Hernandez-Rivas; Maribel Gonzalez-Villa; Natividad Cruz-Ortiz; Sergio Rangel-Guerrero; Tatiana Nunez-Garcia; Vanessa Rivero-Arredondo
EPI_ISL_2674642	LESP Durango	Instituto de Diagnostico y Referencia Epidemiologicos (INDRE)	Abril Rodriguez-Maldonado; Ariadna Medina-Benitez; Claudia Wong-Arambula; Ernesto Ramirez-Gonzalez.; Gisela Barrera-Badillo; Irma Lopez-Martinez; Joaquin Quiroz-Mercado; Lucia Hernandez-Rivas; Natividad Cruz-Ortiz; Sergio Rangel-Guerrero; Tatiana Nunez-Garcia; Vanessa Rivero-Arredondo
EPI_ISL_2674664	LESP Estado de Mexico	Instituto de Diagnostico y Referencia Epidemiologicos (INDRE)	Abril Rodriguez-Maldonado; Ariadna Medina-Benitez; Claudia Wong-Arambula; Ernesto Ramirez-Gonzalez.; Gisela Barrera-Badillo; Irma Lopez-Martinez; Joaquin Quiroz-Mercado; Lucia Hernandez-Rivas; Natividad Cruz-Ortiz; Sergio Rangel-Guerrero; Tatiana Nunez-Garcia; Vanessa Rivero-Arredondo
EPI_ISL_2674695, EPI_ISL_2779056, EPI_ISL_2779057, EPI_ISL_2779058, EPI_ISL_2779120, EPI_ISL_2779186, EPI_ISL_2779187, EPI_ISL_2779188, EPI_ISL_2779190, EPI_ISL_2779191, EPI_ISL_2779192, EPI_ISL_2800917, EPI_ISL_2800924, EPI_ISL_2858991, EPI_ISL_2858992, EPI_ISL_2858993, EPI_ISL_2937772			
see above	LESP Guanajuato	Instituto de Diagnostico y Referencia Epidemiologicos (INDRE)	Abril Rodriguez-Maldonado; Ariadna Medina-Benitez; Claudia Wong-Arambula; Ernesto Ramirez-Gonzalez.; Gisela Barrera-Badillo; Irma Lopez-Martinez; Joaquin Quiroz-Mercado; Lucia Hernandez-Rivas; Maribel Gonzalez-Villa; Natividad Cruz-Ortiz; Sergio Rangel-Guerrero; Tatiana Nunez-Garcia; Vanessa Rivero-Arredondo
EPI_ISL_2674665, EPI_ISL_2674677, EPI_ISL_2859012, EPI_ISL_3033562, EPI_ISL_3033566, EPI_ISL_3265597, EPI_ISL_3278284			
see above	LESP Hidalgo	Instituto de Diagnostico y Referencia Epidemiologicos (INDRE)	Abril Rodriguez-Maldonado; Ariadna Medina-Benitez; Claudia Wong-Arambula; Ernesto Ramirez-Gonzalez.; Gisela Barrera-Badillo; Irma Lopez-Martinez; Joaquin Quiroz-Mercado; Lucia Hernandez-Rivas; Maribel Gonzalez-Villa; Natividad Cruz-Ortiz; Sergio Rangel-Guerrero; Tatiana Nunez-Garcia; Vanessa Rivero-Arredondo
EPI_ISL_2779175, EPI_ISL_2858995, EPI_ISL_2937757, EPI_ISL_2937801, EPI_ISL_2937815, EPI_ISL_3459946, EPI_ISL_3459947			
see above	LESP Jalisco	Instituto de Diagnostico y Referencia Epidemiologicos (INDRE)	Abril Rodriguez-Maldonado; Ariadna Medina-Benitez; Claudia Wong-Arambula; Ernesto Ramirez-Gonzalez.; Gisela Barrera-Badillo; Irma Lopez-Martinez; Joaquin Quiroz-Mercado; Lucia Hernandez-Rivas; Maribel Gonzalez-Villa; Natividad Cruz-Ortiz; Sergio Rangel-Guerrero; Tatiana Nunez-Garcia; Vanessa Rivero-Arredondo
EPI_ISL_3033575, EPI_ISL_3033592	LESP Michoacan	Instituto de Diagnostico y Referencia Epidemiologicos (INDRE)	Abril Rodriguez-Maldonado; Ariadna Medina-Benitez; Claudia Wong-Arambula; Ernesto Ramirez-Gonzalez.; Gisela Barrera-Badillo; Irma Lopez-Martinez; Joaquin Quiroz-Mercado; Lucia Hernandez-Rivas; Maribel Gonzalez-Villa; Natividad Cruz-Ortiz; Sergio Rangel-Guerrero; Tatiana Nunez-Garcia; Vanessa Rivero-Arredondo
EPI_ISL_2937774, EPI_ISL_3265605	LESP Nayarit	Instituto de Diagnostico y Referencia Epidemiologicos (INDRE)	Abril Rodriguez-Maldonado; Ariadna Medina-Benitez; Claudia Wong-Arambula; Ernesto Ramirez-Gonzalez.; Gisela Barrera-Badillo; Irma Lopez-Martinez; Joaquin Quiroz-Mercado; Lucia Hernandez-Rivas; Maribel Gonzalez-Villa; Natividad Cruz-Ortiz; Sergio Rangel-Guerrero; Tatiana Nunez-Garcia; Vanessa Rivero-Arredondo
EPI_ISL_2859054	LESP Nuevo Leon	Instituto de Diagnostico y Referencia Epidemiologicos (INDRE)	Abril Rodriguez-Maldonado; Ariadna Medina-Benitez; Claudia Wong-Arambula; Ernesto Ramirez-Gonzalez.; Gisela Barrera-Badillo; Irma Lopez-Martinez; Joaquin Quiroz-Mercado; Lucia Hernandez-Rivas; Maribel Gonzalez-Villa; Natividad Cruz-Ortiz; Sergio Rangel-Guerrero; Tatiana Nunez-Garcia; Vanessa Rivero-Arredondo
EPI_ISL_2674700, EPI_ISL_3033596, EPI_ISL_3033597	LESP Puebla	Instituto de Diagnostico y Referencia Epidemiologicos (INDRE)	Abril Rodriguez-Maldonado; Ariadna Medina-Benitez; Claudia Wong-Arambula; Ernesto Ramirez-Gonzalez.; Gisela Barrera-Badillo; Irma Lopez-Martinez; Joaquin Quiroz-Mercado; Lucia Hernandez-Rivas; Maribel Gonzalez-Villa; Natividad Cruz-Ortiz; Sergio Rangel-Guerrero; Tatiana Nunez-Garcia; Vanessa Rivero-Arredondo
EPI_ISL_2545693, EPI_ISL_2545694, EPI_ISL_2937758, EPI_ISL_2937798	LESP Quintana Roo	Instituto de Diagnostico y Referencia Epidemiologicos (INDRE)	Abril Rodriguez-Maldonado; Ariadna Medina-Benitez; Claudia Wong-Arambula; Ernesto Ramirez-Gonzalez.; Gisela Barrera-Badillo; Irma Lopez-Martinez; Joaquin Quiroz-Mercado; Lucia Hernandez-Rivas; Maribel Gonzalez-Villa; Natividad Cruz-Ortiz; Sergio Rangel-Guerrero; Tatiana Nunez-Garcia; Vanessa Rivero-Arredondo
EPI_ISL_2779159, EPI_ISL_2859059, EPI_ISL_2859060, EPI_ISL_2937773	LESP San Luis Potosi	Instituto de Diagnostico y Referencia Epidemiologicos (INDRE)	Abril Rodriguez-Maldonado; Ariadna Medina-Benitez; Claudia Wong-Arambula; Ernesto Ramirez-Gonzalez.; Gisela Barrera-Badillo; Irma Lopez-Martinez; Joaquin Quiroz-Mercado; Lucia Hernandez-Rivas; Maribel Gonzalez-Villa; Natividad Cruz-Ortiz; Sergio Rangel-Guerrero; Tatiana Nunez-Garcia; Vanessa Rivero-Arredondo
EPI_ISL_2545692, EPI_ISL_2674634, EPI_ISL_2859013, EPI_ISL_2859015, EPI_ISL_3033574	LESP Sinaloa	Instituto de Diagnostico y Referencia Epidemiologicos (INDRE)	Abril Rodriguez-Maldonado; Ariadna Medina-Benitez; Claudia Wong-Arambula; Ernesto Ramirez-Gonzalez.; Gisela Barrera-Badillo; Irma Lopez-Martinez; Joaquin Quiroz-Mercado; Lucia Hernandez-Rivas; Maribel Gonzalez-Villa; Natividad Cruz-Ortiz; Sergio Rangel-Guerrero; Tatiana Nunez-Garcia; Vanessa Rivero-Arredondo
EPI_ISL_2674648, EPI_ISL_2674649, EPI_ISL_2779055, EPI_ISL_2779185, EPI_ISL_2859051, EPI_ISL_2937767, EPI_ISL_2937795, EPI_ISL_2937797, EPI_ISL_3033580, EPI_ISL_3265593, EPI_ISL_3265594, EPI_ISL_3459940			
see above	LESP Sonora	Instituto de Diagnostico y Referencia Epidemiologicos (INDRE)	Abril Rodriguez-Maldonado; Ariadna Medina-Benitez; Claudia Wong-Arambula; Ernesto Ramirez-Gonzalez.; Gisela Barrera-Badillo; Irma Lopez-Martinez; Joaquin Quiroz-Mercado; Lucia Hernandez-Rivas; Maribel Gonzalez-Villa; Natividad Cruz-Ortiz; Sergio Rangel-Guerrero; Tatiana Nunez-Garcia; Vanessa Rivero-Arredondo
EPI_ISL_2779160, EPI_ISL_2779169, EPI_ISL_2859016, EPI_ISL_2937811, EPI_ISL_2937812, EPI_ISL_2937813, EPI_ISL_3033583			
see above	LESP Tabasco	Instituto de Diagnostico y Referencia Epidemiologicos (INDRE)	Abril Rodriguez-Maldonado; Ariadna Medina-Benitez; Claudia Wong-Arambula; Ernesto Ramirez-Gonzalez.; Gisela Barrera-Badillo; Irma Lopez-Martinez; Joaquin Quiroz-Mercado; Lucia Hernandez-Rivas; Maribel Gonzalez-Villa; Natividad Cruz-Ortiz; Sergio Rangel-Guerrero; Tatiana Nunez-Garcia; Vanessa Rivero-Arredondo
EPI_ISL_2779060, EPI_ISL_2779061, EPI_ISL_2779062, EPI_ISL_2779063, EPI_ISL_2779064, EPI_ISL_2779065, EPI_ISL_2858996, EPI_ISL_2858997, EPI_ISL_2858998, EPI_ISL_2858999, EPI_ISL_2859030, EPI_ISL_2859031, EPI_ISL_2859032, EPI_ISL_2937775, EPI_ISL_2937776, EPI_ISL_2937777, EPI_ISL_2937828, EPI_ISL_2937829, EPI_ISL_2937830, EPI_ISL_3033465, EPI_ISL_3033466, EPI_ISL_3033469, EPI_ISL_3265606			
see above	LESP Tamaulipas	Instituto de Diagnostico y Referencia Epidemiologicos (INDRE)	Abril Rodriguez-Maldonado; Ariadna Medina-Benitez; Claudia Wong-Arambula; Ernesto Ramirez-Gonzalez.; Gisela Barrera-Badillo; Irma Lopez-Martinez; Joaquin Quiroz-Mercado; Lucia Hernandez-Rivas; Maribel Gonzalez-Villa; Natividad Cruz-Ortiz; Sergio Rangel-Guerrero; Tatiana Nunez-Garcia; Vanessa Rivero-Arredondo
EPI_ISL_3265602	LESP Veracruz	Instituto de Diagnostico y Referencia Epidemiologicos (INDRE)	Abril Rodriguez-Maldonado; Ariadna Medina-Benitez; Claudia Wong-Arambula; Ernesto Ramirez-Gonzalez.; Gisela Barrera-Badillo; Irma Lopez-Martinez; Joaquin Quiroz-Mercado; Lucia Hernandez-Rivas; Maribel Gonzalez-Villa; Natividad Cruz-Ortiz; Sergio Rangel-Guerrero; Tatiana Nunez-Garcia; Vanessa Rivero-Arredondo
EPI_ISL_2545649	LHUB-ULB	Labo Klinische Biologie, UZA	Basil Britto Xavier; Christine Lammens; Herman Goossens; Jasmine Coppens; Marie Le Mercier; Veerle Matheeussen
EPI_ISL_2873838	LUM Laboratorio Unico Metropolitano - AUSL BOLOGNA	U.O. Microbiologia, Laboratorio Unico Centro Servizi - AUSL della Romagna	Giorgio Dirani
EPI_ISL_2695394	Lab La Salete Robles - VN Famalicao	Instituto Nacional de Saude (INSA)	Borges et al
EPI_ISL_2861479, EPI_ISL_3868312	Lab voor klinische biologie	Lab voor klinische biologie	Bruno Verhasselt; Hannelore Hamerlinck; Marija Janevska
EPI_ISL_2725223	Lab. Microbiologia e Virologia Cotugno A.O. dei Colli - Istituto Zooprofilattico Sperimentale del Mezzogiorno	Telethon Institute of Genetics and Medicine (TIGEM)	Antonio Grimaldi Patrizia Annunziata Francesco Panariello Biancamaria Pierri Claudia Tiberio Teresa Giuliano Valentina Bouche Chiara Colantuono Maria Concetta Cuomo Denise Di Concilio Lucio Di Filippo Anna Manfredi Marcello Salvi Antonio Limone Luigi Atripaldi Pellegrino Cerino Andrea Ballabio Davide Cacchiarelli
EPI_ISL_2650016	LabPLUS	Institute of Environmental Science and Research (ESR)	Anja Werno; Antje van der Linden; Arlo Upton; Chris Mansell; David Hammer; Dragana Drinkovic; Erasmus Smit; Gary McAuliffe; Hana Sofia Andersson; Hermes Perez; James Ussher; Jill Sherwood; Jing Wang; Joep de Ligt; Josh Freeman; Julia Howard; Juliet Elvy; Lauren Jelly; Mary DeAlmeida; Matt Blakiston; Matt Storey; Matthew Rogers; Max Bloomfield; Michael Addidle; Michelle Balm; Muhammad Faisal; Nikki Freed; Olin Silander; Olivia Stroeven; Rachel Boyle; Sally Roberts; SallyAnn Harbison; Sarah Jefferies; Sharmini Muttaiyah; Susan Morpeth; Susan Taylor; Timothy Blackmore; Vani Sathyendran; Veronica Playle; Virginia Hope; Xiaoyun Ren
EPI_ISL_2535296, EPI_ISL_2790872, EPI_ISL_2790971, EPI_ISL_2790978, EPI_ISL_2835744, EPI_ISL_2835851, EPI_ISL_2835852, EPI_ISL_2835853, EPI_ISL_2835854, EPI_ISL_2835856, EPI_ISL_2923134, EPI_ISL_2923135, EPI_ISL_2923176, EPI_ISL_2923179, EPI_ISL_2923181, EPI_ISL_2958763, EPI_ISL_3058210, EPI_ISL_3245972			
see above	Labo Analyses Med	National Reference Center for Viruses of Respiratory Infections, Institut Pasteur, Paris	Amaury Vaysse; Angela Brisebarre; BéNéDicte Roquebert; Camille Capel; Christophe Malabat; Corinne Maufrais; Damien Mornico; Emmanuelle Permal; Etienne Simon-Lorière; Eve Haguenoer; Frédéric Lemoine; Gael Millot; Hub Bioinformatique Biostatistiques; Hub de bioinformatique et biostatistique; Louise Lefrançois; Marion Barbet; Maud Vanpeene; Méline Bizard; OphéLie Said-Delattre; POTIRON Grégoire; Sabine Trombert; Sabine Trombert; Sylvie Behillil; Sylvie Van der Werf; TROMBERT Sabine; Victoire Baillet; Vincent Enouf
EPI_ISL_2504046, EPI_ISL_3113202	Labor Berlin Charité Vivantes GmbH / Institut für Virologie	Charité Universitätsmedizin Berlin, Institut für Virologie/Labor Berlin	Barbara Mühlemann; Christian Drosten; Christine Stephan; Peter Menzel; Rolf Schwarzer; Terry Jones; Victor M Corman
EPI_ISL_2689539	Labor Dr. Fenner und Kollegen	Heinrich Pette Institute, Leibniz	Adam Grundhoff; Alexis Robitaille; Johannes Knobloch; Martin Aepfelbacher; Nicole Fischer; Thomas Günther

		Institute for Experimental Virology	
EPI_ISL_2878457	Laboratoire Ana-L	Department of Virology, Henri Mondor University Hospital, Assistance Publique Hôpitaux de Paris, Université Paris-Est Créteil, INSERM U955	Alexandre Soulier; Christophe Rodriguez; Elisabeth Trawinski; Guillaume Gricourt; Jean-Michel Pawlotsky; Melissa N'Debi; Slim Fourati; Vanessa Demontant
EPI_ISL_2426981, EPI_ISL_2627849, EPI_ISL_2629225, EPI_ISL_2629226, EPI_ISL_2629228, EPI_ISL_2629229, EPI_ISL_2835986, EPI_ISL_2835987, EPI_ISL_2835988, EPI_ISL_2835989, EPI_ISL_2835990			
see above	Laboratoire Carage	Institut Pasteur de la Guyane	Anne Lavergne; Antoine Enfissi; Arielle Salmier; Dominique Rousset
EPI_ISL_3458779, EPI_ISL_3458806, EPI_ISL_3458809, EPI_ISL_3458826, EPI_ISL_3459046, EPI_ISL_3459085	Laboratoire de santé publique du Québec	Laboratoire de santé publique du Québec	Guillaume Bourque; Ioannis Ragoussis; Jesse Shapiro; Mark Lathrop and Michel Roger on behalf of the CoVSeQ research group; Sandrine Moreira
EPI_ISL_3906483	Laboratori de Referència de Catalunya	SeqCOVID-SPAIN consortium/IBV(CSIC)	Aida Esperanza Ramírez Marinero; Alba Vilas Basil; Eduardo Padilla León; Mireia Canal Aranda and SeqCOVID-SPAIN consortium
EPI_ISL_2983182, EPI_ISL_2983185, EPI_ISL_2983186, EPI_ISL_2983231, EPI_ISL_2983233, EPI_ISL_2983234, EPI_ISL_3190351, EPI_ISL_3190352, EPI_ISL_3190354, EPI_ISL_3190355			
see above	Laboratorio Central de Saude Publica do Estado de Goias (LACEN/GO)	Laboratory of Respiratory Viruses and Measles, Oswaldo Cruz Institute, FIOCRUZ	Agatha Cristinne Prudencio; Alice Sampaio Rocha; Ana Carolina Mendonca; Ana Flavia Mendonça; Anna Carolina Paixao; Carmen Helena Ramos; Cassiane Casanova; Elisa Cavalcante Pereira; Fernando Motta; Flavia Pereira Amorim da Silva; Ighor Leonardo Arantes Gomes; Luciana Appolinario; Luiz Augusto Pereira; Marilda Siqueira on behalf of the Fiocruz COVID-19 Genomic Surveillance Network; Paola Resende; Rafael Souza Guedes; Renata Serrano Lopes; Taina Venas; Vinicius Lemes da Silva
EPI_ISL_3539750, EPI_ISL_3539751, EPI_ISL_3539752, EPI_ISL_3539753, EPI_ISL_3539755, EPI_ISL_3539756, EPI_ISL_3539757, EPI_ISL_3539758, EPI_ISL_3539759, EPI_ISL_3539760, EPI_ISL_3539761, EPI_ISL_3539762, EPI_ISL_3539763, EPI_ISL_3539764, EPI_ISL_3539765			
see above	Laboratorio Central de Saude Publica do Estado de Minas Gerais (LACEN/MG)	Laboratory of Respiratory Viruses and Measles, Oswaldo Cruz Institute, FIOCRUZ	Alice Sampaio Rocha; Ana Carolina Mendonca; Anna Carolina Paixao; Elisa Cavalcante Pereira; Fernando Motta; Luciana Appolinario; Marilda Siqueira on behalf of the Fiocruz COVID-19 Genomic Surveillance Network; Paola Resende; Renata Serrano Lopes; Rubens Pasa; Taina Venas
EPI_ISL_2645434, EPI_ISL_2645435, EPI_ISL_2731453, EPI_ISL_2731454, EPI_ISL_2731455, EPI_ISL_2731456, EPI_ISL_2863578, EPI_ISL_2863580, EPI_ISL_2863582, EPI_ISL_2863706, EPI_ISL_2863707, EPI_ISL_2863709, EPI_ISL_2863711, EPI_ISL_2863801, EPI_ISL_2863802, EPI_ISL_2863803, EPI_ISL_2863804, EPI_ISL_2863805, EPI_ISL_2863806, EPI_ISL_2863807, EPI_ISL_2863808, EPI_ISL_2863809, EPI_ISL_2863810, EPI_ISL_2863811, EPI_ISL_2863812, EPI_ISL_2863813, EPI_ISL_2863814, EPI_ISL_2863815, EPI_ISL_2863816, EPI_ISL_2863817, EPI_ISL_2863819, EPI_ISL_2863820, EPI_ISL_2863821, EPI_ISL_2863822, EPI_ISL_2863823, EPI_ISL_2863824, EPI_ISL_2863825, EPI_ISL_2863826, EPI_ISL_2863827, EPI_ISL_2863828, EPI_ISL_2863829, EPI_ISL_2863830, EPI_ISL_2863831, EPI_ISL_2863832, EPI_ISL_2863833, EPI_ISL_2863834, EPI_ISL_2863835, EPI_ISL_2863837, EPI_ISL_2863838, EPI_ISL_2863839, EPI_ISL_2863840, EPI_ISL_2863841, EPI_ISL_2863842, EPI_ISL_2863843, EPI_ISL_2863844, EPI_ISL_2863845, EPI_ISL_2863846, EPI_ISL_2863847, EPI_ISL_2863848, EPI_ISL_2863849, EPI_ISL_2863850, EPI_ISL_2863851, EPI_ISL_2863852, EPI_ISL_2863853, EPI_ISL_2863854, EPI_ISL_2863894, EPI_ISL_4080766, EPI_ISL_4080769, EPI_ISL_4080770			
see above	Laboratorio Central de Saude Publica do Estado do Para (LACEN/PA)	Laboratory of Respiratory Viruses and Measles, Oswaldo Cruz Institute, FIOCRUZ	Agatha Cristinne Prudencio; Agatha Cristinne Prudencio Soares; Agatha Soares; Alice Sampaio Rocha; Ana Carolina Mendonca; Anna Carolina Paixao; Elisa Cavalcante Pereira; Fernando Motta; Ighor Arantes; Ighor Leonardo Arantes Gomes; Luciana Appolinario; Marilda Siqueira on behalf of the Fiocruz COVID-19 Genomic Surveillance Network; Paola Resende; Renata Serrano Lopes; Taina Venas; Valnete Andrade
EPI_ISL_2535078, EPI_ISL_2535087, EPI_ISL_2535088, EPI_ISL_2535108, EPI_ISL_2535109, EPI_ISL_2535110, EPI_ISL_2691315, EPI_ISL_2691316, EPI_ISL_2691317, EPI_ISL_2691318, EPI_ISL_2691319, EPI_ISL_2691321, EPI_ISL_2691322, EPI_ISL_2691323, EPI_ISL_2691324, EPI_ISL_2691325, EPI_ISL_2691326, EPI_ISL_2691327, EPI_ISL_2691328, EPI_ISL_2691330, EPI_ISL_2691331, EPI_ISL_2691332, EPI_ISL_2691333, EPI_ISL_2691334, EPI_ISL_2691336, EPI_ISL_2691337, EPI_ISL_2691338, EPI_ISL_2691339, EPI_ISL_2691340, EPI_ISL_2691341, EPI_ISL_2691343, EPI_ISL_2691344, EPI_ISL_2691345, EPI_ISL_2691347, EPI_ISL_2691348, EPI_ISL_2691349, EPI_ISL_2691350, EPI_ISL_2691351, EPI_ISL_2691352, EPI_ISL_2691357, EPI_ISL_2691359, EPI_ISL_2691360, EPI_ISL_2691362, EPI_ISL_2691364, EPI_ISL_2691366, EPI_ISL_2691367, EPI_ISL_2691368, EPI_ISL_2691369, EPI_ISL_2691370, EPI_ISL_2691371, EPI_ISL_2691372, EPI_ISL_2691373, EPI_ISL_2691374, EPI_ISL_2691375, EPI_ISL_2691376, EPI_ISL_2691377, EPI_ISL_2691378, EPI_ISL_2691380, EPI_ISL_2691381, EPI_ISL_2691383, EPI_ISL_2691384, EPI_ISL_2691385, EPI_ISL_2691387, EPI_ISL_2691388, EPI_ISL_2691389, EPI_ISL_2691390, EPI_ISL_2691391, EPI_ISL_2691392, EPI_ISL_2691393, EPI_ISL_2691394, EPI_ISL_2691395, EPI_ISL_2691396, EPI_ISL_2691397, EPI_ISL_2691398, EPI_ISL_2691399, EPI_ISL_2691400, EPI_ISL_2691401, EPI_ISL_2691402, EPI_ISL_2691403, EPI_ISL_2691404, EPI_ISL_2691409, EPI_ISL_2691410, EPI_ISL_2691411, EPI_ISL_2691412, EPI_ISL_2691414, EPI_ISL_2691415, EPI_ISL_2691416, EPI_ISL_2691417, EPI_ISL_2691419, EPI_ISL_2691420, EPI_ISL_2691424, EPI_ISL_2691425, EPI_ISL_2691426, EPI_ISL_2691427, EPI_ISL_2691428, EPI_ISL_2691430, EPI_ISL_2691431, EPI_ISL_2691432, EPI_ISL_2691433, EPI_ISL_2691434, EPI_ISL_2691435, EPI_ISL_2691436, EPI_ISL_2691437, EPI_ISL_2691438, EPI_ISL_2691439, EPI_ISL_2691441, EPI_ISL_2691442, EPI_ISL_2691443, EPI_ISL_2691444, EPI_ISL_2691445, EPI_ISL_2691446, EPI_ISL_2691447, EPI_ISL_2691448, EPI_ISL_2691449, EPI_ISL_2691451, EPI_ISL_2691453, EPI_ISL_2691454, EPI_ISL_2691455, EPI_ISL_2691456, EPI_ISL_2691457, EPI_ISL_2691458, EPI_ISL_2691459, EPI_ISL_2691460, EPI_ISL_2691461, EPI_ISL_2691462, EPI_ISL_2691463, EPI_ISL_2691464, EPI_ISL_2691465, EPI_ISL_2691466, EPI_ISL_2691467, EPI_ISL_2691468, EPI_ISL_2691469, EPI_ISL_2691470, EPI_ISL_2691471, EPI_ISL_2691472, EPI_ISL_2691473, EPI_ISL_2691474, EPI_ISL_2691476, EPI_ISL_2691477, EPI_ISL_2691478, EPI_ISL_2691479, EPI_ISL_2691480, EPI_ISL_2691481, EPI_ISL_2691482, EPI_ISL_2691483, EPI_ISL_2691484, EPI_ISL_2691485, EPI_ISL_2691486, EPI_ISL_2691487, EPI_ISL_2691488, EPI_ISL_2691489, EPI_ISL_2691490, EPI_ISL_2691491, EPI_ISL_2691492, EPI_ISL_2691493, EPI_ISL_2691494, EPI_ISL_2691495, EPI_ISL_2691496, EPI_ISL_2691497, EPI_ISL_2691499, EPI_ISL_2691500, EPI_ISL_2691501, EPI_ISL_2691502, EPI_ISL_2691503, EPI_ISL_2691504, EPI_ISL_2691505, EPI_ISL_2691506, EPI_ISL_2691507, EPI_ISL_2691508, EPI_ISL_2691509, EPI_ISL_2691510, EPI_ISL_2691511, EPI_ISL_2691512, EPI_ISL_2691513, EPI_ISL_2691515, EPI_ISL_2691517, EPI_ISL_2691518, EPI_ISL_2691519, EPI_ISL_2691520, EPI_ISL_2691521, EPI_ISL_2691522, EPI_ISL_2691525, EPI_ISL_2691527, EPI_ISL_2691528, EPI_ISL_2691529, EPI_ISL_2691530, EPI_ISL_2691531, EPI_ISL_2691533, EPI_ISL_2691534, EPI_ISL_2691535, EPI_ISL_2691537, EPI_ISL_2691538, EPI_ISL_2691539, EPI_ISL_2691540, EPI_ISL_2691541, EPI_ISL_2691542, EPI_ISL_2691543, EPI_ISL_2691545, EPI_ISL_2691546, EPI_ISL_2691547, EPI_ISL_2691548, EPI_ISL_2691549, EPI_ISL_2691550, EPI_ISL_2691551, EPI_ISL_2691552, EPI_ISL_2691553, EPI_ISL_2691554, EPI_ISL_2691555, EPI_ISL_2691556, EPI_ISL_2691557, EPI_ISL_2691558, EPI_ISL_2691560, EPI_ISL_2691561, EPI_ISL_2691563, EPI_ISL_2691564, EPI_ISL_2691565, EPI_ISL_2691566, EPI_ISL_2691567, EPI_ISL_2691568, EPI_ISL_2836977, EPI_ISL_2836978, EPI_ISL_2836979, EPI_ISL_2836981, EPI_ISL_2836982, EPI_ISL_2836983, EPI_ISL_2836984, EPI_ISL_2836985, EPI_ISL_2836988, EPI_ISL_2836989, EPI_ISL_2836990, EPI_ISL_2836992, EPI_ISL_2836996, EPI_ISL_2837001, EPI_ISL_2837002, EPI_ISL_2837004, EPI_ISL_2837005, EPI_ISL_2837006, EPI_ISL_2837007, EPI_ISL_2837008, EPI_ISL_2837009, EPI_ISL_2837010, EPI_ISL_2837011, EPI_ISL_2837012, EPI_ISL_2837013, EPI_ISL_2837014, EPI_ISL_2837015, EPI_ISL_2837017, EPI_ISL_2837019, EPI_ISL_2837020, EPI_ISL_2837021, EPI_ISL_2837022, EPI_ISL_2837026, EPI_ISL_2837027, EPI_ISL_2837028, EPI_ISL_2837029, EPI_ISL_2837030, EPI_ISL_2837031, EPI_ISL_2837032, EPI_ISL_2837033, EPI_ISL_2837034, EPI_ISL_2837035, EPI_ISL_2837036, EPI_ISL_2837037, EPI_ISL_2837038, EPI_ISL_2837040, EPI_ISL_2837043, EPI_ISL_2837045, EPI_ISL_2837046, EPI_ISL_2837048, EPI_ISL_2837049, EPI_ISL_2837050, EPI_ISL_2837051, EPI_ISL_2837052, EPI_ISL_2837053, EPI_ISL_2837054, EPI_ISL_2837055, EPI_ISL_2837056, EPI_ISL_2837060, EPI_ISL_2837063, EPI_ISL_2837064, EPI_ISL_2837065, EPI_ISL_2837066, EPI_ISL_2837067, EPI_ISL_2837068, EPI_ISL_2837069, EPI_ISL_2837071, EPI_ISL_2837072, EPI_ISL_2837074, EPI_ISL_2837075, EPI_ISL_2837076, EPI_ISL_2837077, EPI_ISL_2837078, EPI_ISL_2837079, EPI_ISL_2837080, EPI_ISL_2837081, EPI_ISL_2837082, EPI_ISL_2837083, EPI_ISL_2837084, EPI_ISL_2837087, EPI_ISL_2837088, EPI_ISL_2837089, EPI_ISL_2837091, EPI_ISL_2837094, EPI_ISL_2837095, EPI_ISL_2837098, EPI_ISL_2837099, EPI_ISL_2837100, EPI_ISL_2837101, EPI_ISL_2837102, EPI_ISL_2837103, EPI_ISL_2837105, EPI_ISL_2837106, EPI_ISL_2837107, EPI_ISL_2837108, EPI_ISL_2837109, EPI_ISL_2837110, EPI_ISL_2837111, EPI_ISL_2837113, EPI_ISL_2837114, EPI_ISL_2837115, EPI_ISL_2837118, EPI_ISL_2837119, EPI_ISL_2837120, EPI_ISL_2837122, EPI_ISL_2837123, EPI_ISL_2837124, EPI_ISL_2837125, EPI_ISL_2837126, EPI_ISL_2837130, EPI_ISL_2837132, EPI_ISL_2837133, EPI_ISL_2837134, EPI_ISL_2837135, EPI_ISL_2837136, EPI_ISL_2837137, EPI_ISL_2837138, EPI_ISL_2837139, EPI_ISL_2837140, EPI_ISL_2837141, EPI_ISL_2837142, EPI_ISL_2837143, EPI_ISL_2837144, EPI_ISL_2837146, EPI_ISL_2837147, EPI_ISL_2837148, EPI_ISL_2837149, EPI_ISL_2837150, EPI_ISL_2837151, EPI_ISL_2837152, EPI_ISL_2837153, EPI_ISL_2837154, EPI_ISL_2837155, EPI_ISL_2837156, EPI_ISL_2837157, EPI_ISL_2837159, EPI_ISL_2837160, EPI_ISL_2837162, EPI_ISL_2837163, EPI_ISL_2837165, EPI_ISL_2837167, EPI_ISL_2837168, EPI_ISL_2837169, EPI_ISL_2837170, EPI_ISL_2837171, EPI_ISL_2837172, EPI_ISL_2837179, EPI_ISL_2837180, EPI_ISL_2837181, EPI_ISL_2837182, EPI_ISL_2837183, EPI_ISL_2837184, EPI_ISL_2837186, EPI_ISL_2837187, EPI_ISL_2837189, EPI_ISL_2837190, EPI_ISL_2837191, EPI_ISL_2837192, EPI_ISL_2837193, EPI_ISL_2837194, EPI_ISL_2837195, EPI_ISL_2837196, EPI_ISL_2837197, EPI_ISL_2837198, EPI_ISL_2837199, EPI_ISL_2837200, EPI_ISL_2837202, EPI_ISL_2837203, EPI_ISL_2837204, EPI_ISL_2837205, EPI_ISL_2837206, EPI_ISL_2837207, EPI_ISL_3245102, EPI_ISL_3245105, EPI_ISL_3245106, EPI_ISL_3245108, EPI_ISL_3245112, EPI_ISL_3245113, EPI_ISL_3245119, EPI_ISL_3245122, EPI_ISL_3245123, EPI_ISL_3245124, EPI_ISL_3245129, EPI_ISL_3245130, EPI_ISL_3245131, EPI_ISL_3245134, EPI_ISL_3245136, EPI_ISL_3245137, EPI_ISL_3245138, EPI_ISL_3245139, EPI_ISL_3245141, EPI_ISL_3245142, EPI_ISL_3245144, EPI_ISL_3245145, EPI_ISL_3245146, EPI_ISL_3245147, EPI_ISL_3245149, EPI_ISL_3245151, EPI_ISL_3245152, EPI_ISL_3245153, EPI_ISL_3245155, EPI_ISL_3245156, EPI_ISL_3245157, EPI_ISL_3245160, EPI_ISL_3245161, EPI_ISL_3245162, EPI_ISL_3245164, EPI_ISL_3245165, EPI_ISL_3245167, EPI_ISL_3245168, EPI_ISL_3245171, EPI_ISL_3245172, EPI_ISL_3245175, EPI_ISL_3245176, EPI_ISL_3245177, EPI_ISL_3245179, EPI_ISL_3245180, EPI_ISL_3245183, EPI_ISL_3245184, EPI_ISL_3245185, EPI_ISL_3245189, EPI_ISL_3245191, EPI_ISL_3245193, EPI_ISL_3245194, EPI_ISL_3245197, EPI_ISL_3245198, EPI_ISL_3245200, EPI_ISL_3245201, EPI_ISL_3245203, EPI_ISL_3245204, EPI_ISL_3245206, EPI_ISL_3245207, EPI_ISL_3245209, EPI_ISL_3245210, EPI_ISL_3245211, EPI_ISL_3245212, EPI_ISL_3245213, EPI_ISL_3245214, EPI_ISL_3245216, EPI_ISL_3245217, EPI_ISL_3245218, EPI_ISL_3245220, EPI_ISL_3245221, EPI_ISL_3245222, EPI_ISL_3245223, EPI_ISL_3245224, EPI_ISL_3245230, EPI_ISL_3245232, EPI_ISL_3245234, EPI_ISL_3245235, EPI_ISL_3245237, EPI_ISL_3245239, EPI_ISL_3245300, EPI_ISL_3245340			
see above	Laboratorio Central Noel Nutels	Bioinformatics Laboratory / LNCC	Alessandra P Lamarca; Alexandra L Gerber; Amilcar Tanuri; Ana Paula de C Guimaraes; Ana Tereza R Vasconcelos; Andrea Cony Cavalcanti; Caio Luiz Pereira Ribeiro; Cassia Alves; Cintia Policarpo; Claudia Maria Braga de Mello; Cristiane Gomes da Silva; Diana Mariani; Douglas Terra Machado; Erica Ramos dos Santos Nascimento; Fernanda Leitao dos Santos; Flavio Dias da Silva; Gleidson da Silva de Oliveira; Leandro Magalhaes de Souza; Liliane Cavalcante; Luiz G P de Almeida; Marcio Henrique de Oliveira Garcia; Mario Sergio Ribeiro; Ricardo Jose Barbosa Salviano; Ronaldo da Silva F Jr; Silvia Carvalho
EPI_ISL_2942513, EPI_ISL_2942520, EPI_ISL_2942527, EPI_ISL_2942547, EPI_ISL_2942569, EPI_ISL_2942573, EPI_ISL_2942581, EPI_ISL_2942606, EPI_ISL_2942837, EPI_ISL_2942893, EPI_ISL_2942903, EPI_ISL_2942904, EPI_ISL_2942908, EPI_ISL_2942924			
see above	Laboratorio Central de Epidemiologia (LCE)	Unidad de Genomica Avanzada	; Alejandra Garcia-Gasca; Alejandra Hernandez-Teran; Alejandro Sanchez-Flores; Alfredo Herrera-Estrella; Alicia Ocaña-Mondragon; Andreu Comas-Garcia; Angel Gustavo Salas-Lais; Antonio Loza Roman; Bernardo Martinez-Miguel; Blanca Taboada; Brenda Irasema Maldonado-Meza; Bruno Gomez-Gil; Carla Ivon Herrera-Najera; Carlos F. Arias; Celia Boukadida; Celida Duque Molina; Celida Martinez- Rodriguez; Clara Esperanza Santacruz-Tinoco; Concepcion Grajales-Muñiz; Consorcio Mexicano de Vigilancia Genomica (CoViGen-Mex). Authors (in alphabetical order): Julio Elias Alvarado-Yaah; Cristobal Chaidez-Quiroz; Daniel Fregoso-Rueda; Daniel Lira Morales; Eduardo Becerril-Vargas; Fernando Fontove-Herrera; Fidencio Mejia-Nepomuceno; Francisco Pulido; Gloria Elena Espinosa-Ayala; Gloria Maria Molina-Salinas; Gloria Vazquez; Hector Esteban Paz-Juarez; Hector Montoya-Fuentes; Helen Haydee Fernanda Ramirez-Plascencia; Irvin Gonzalez-Lopez; Jean Pierre Gonzalez; Jesus Hernandez; Joel Armando Vazquez-Perez.; Jorge Salas-Hernandez; Jose Antonio Enciso-Moreno; Jose Arturo Martinez-Orozco; Jose Esteban Muñoz-Medina; Jose de Jesus Nuñez-Contreras; Juan Bautista Chale-Dzul; Julissa Enciso-Ibarra; Luis Alberto Ochoa-Carrera; Margarita Matias-Florentino; Maria Guadalupe Santiago-Mauricio; Maria Guadalupe de Jesus Mireles-Rivera; Mario Mujica-Sanchez; Marissa Perez-Garcia; Nelly Selem-Mojica; Pavel Isa; Ricardo Ciria Merce; Ricardo Grande; Rosa Maria Gutierrez Rios; Santiago avila-Rios; Selene Zarate; Susana Lopez; Veronica Mata-Haro; Victor Eduardo Garcia-Arias; Victor Hugo Borja-Aburto
EPI_ISL_3155578, EPI_ISL_3155611, EPI_ISL_3155623, EPI_ISL_3155625	Laboratorio Central de Epidemiología (LCE)	Centro de Investigación en Enfermedades Infecciosas (CIENI), Instituto Nacional de Enfermedades Respiratorias (INER)	; Alejandra García-Gasca; Alejandra Hernández-Terán; Alejandro Sánchez-Flores; Alfredo Herrera-Estrella; Alicia Ocaña-Mondragón; Andreu Comas-García; Angel Gustavo Salas-Lais; Antonio Loza Román; Bernardo Martínez-Miguel; Blanca Taboada; Brenda Irasema Maldonado-Meza; Bruno Gómez-Gil; Carla Ivón Herrera-Najera; Carlos F. Arias; Celia Boukadida; Clara Esperanza Santacruz-Tinoco; Concepción Grajales-Muñiz; Consorcio Mexicano de Vigilancia Genómica (CoViGen-Mex). Authors (in alphabetical order): Julio Elias Alvarado-Yaah; Cristóbal Cháidez-Quiróz; Célida Duque Molina; Célida Martínez- Rodríguez; Daniel Fregoso-Rueda; Daniel Lira Morales; Eduardo Becerril-Vargas; Fernando Fontove-Herrera; Fidencio Mejía-Nepomuceno; Francisco Pulido; Gloria Elena Espinosa-Ayala; Gloria María Molina-Salinas; Gloria Vazquez; Hector Esteban Paz-Juárez; Hector Montoya-Fuentes; Helen Haydee Fernanda Ramírez-Plascencia; Irvin González-López; Jean Pierre González; Jesús Hernández; Joel Armando Vázquez-Pérez.; Jorge Salas-Hernández; José Antonio Enciso-Moreno; José Arturo Martínez-Orozco; José Esteban Muñoz-Medina; José de Jesús Nuñez-Contreras; Juan Bautista Chale-Dzul; Julissa Enciso-Ibarra; Kathia Elizabeth Tapia-Diaz; Luis Alberto Ochoa-Carrera; Margarita Matías-Florentino; Mario Mújica-Sánchez; Marissa Perez-Garcia; María Guadalupe Santiago-Mauricio; María Guadalupe de Jesús Mireles-Rivera; Nelly Sélem-Mojica; Pavel Isa; Ricardo Ciria Merce; Ricardo Grande; Rosa María Gutiérrez Rios; Santiago Ávila-Ríos; Selene Zárate; Susana Lopez; Verónica Mata-Haro; Victor Eduardo García-Arias; Víctor Hugo Borja-Aburto
EPI_ISL_2681076, EPI_ISL_2681112, EPI_ISL_2681158, EPI_ISL_2681163, EPI_ISL_2681179, EPI_ISL_2681182, EPI_ISL_2681225, EPI_ISL_2801768, EPI_ISL_2801769, EPI_ISL_2801796, EPI_ISL_2801834, EPI_ISL_2801846, EPI_ISL_2801860, EPI_ISL_2801867, EPI_ISL_2801883, EPI_ISL_2801884			
see above	Laboratorio Central de Epidemiología (LCE)	Instituto de Biotecnología de la UNAM	; Alejandra García-Gasca; Alejandra Hernández-Terán; Alejandro Sánchez-Flores; Alfredo Herrera-Estrella; Alicia Ocaña-Mondragón; Andreu Comas-García; Angel Gustavo Salas-Lais; Antonio Loza Román; Bernardo Martínez-Miguel; Blanca Taboada; Brenda Irasema Maldonado-Meza; Bruno Gómez-Gil; Carla Ivón Herrera-Najera; Carlos F. Arias; Celia Boukadida; Clara Esperanza Santacruz-Tinoco; Concepción Grajales-Muñiz; Consorcio Mexicano de Vigilancia Genómica (CoViGen-Mex). Authors (in alphabetical order): Julio Elias Alvarado-Yaah; Cristóbal Cháidez-Quiróz; Célida Duque Molina; Célida Martínez- Rodríguez; Daniel Fregoso-Rueda; Daniel Lira Morales; Eduardo Becerril-Vargas; Fernando Fontove-Herrera; Fidencio Mejía-Nepomuceno; Francisco Pulido; Gloria Elena Espinosa-Ayala; Gloria María Molina-Salinas; Gloria Vazquez; Hector Esteban Paz-Juárez; Hector Montoya-Fuentes; Helen Haydee Fernanda Ramírez-Plascencia; Irvin González-López; Jean Pierre González; Jesús Hernández; Joel Armando Vázquez-Pérez.; Jorge Salas-Hernández; José Antonio Enciso-Moreno; José Arturo Martínez-Orozco; José Esteban Muñoz-Medina; José de Jesús Nuñez-Contreras; Juan Bautista Chale-Dzul; Julissa Enciso-Ibarra; Luis Alberto Ochoa-Carrera; Margarita Matías-Florentino; Mario Mújica-Sánchez; Marissa Perez-Garcia; María Guadalupe Santiago-Mauricio; María Guadalupe de Jesús Mireles-Rivera; Nelly Sélem-Mojica; Pavel Isa; Ricardo Ciria Merce; Ricardo Grande; Rosa María Gutiérrez Ríos; Santiago Ávila-Ríos; Selene Zárate; Susana Lopez; Verónica Mata-Haro; Victor Eduardo García-Arias; Víctor Hugo Borja-Aburto
EPI_ISL_3434751, EPI_ISL_3434752, EPI_ISL_3434755, EPI_ISL_3434758	Laboratorio Central de Saude Publica do Estado Maranhao (LACEN-MA)	Laboratory of Respiratory Viruses and Measles, Oswaldo Cruz Institute, FIOCRUZ	Alice Sampaio Rocha; Ana Carolina Mendonca; Anna Carolina Paixao; Elisa Cavalcante Pereira; Fernando Motta; Lidio Gonçalves Lima Neto; Luciana Appolinario; Marilda Siqueira on behalf of the Fiocruz COVID-19 Genomic Surveillance Network; Paola Resende; Renata Serrano Lopes; Taina Venas
EPI_ISL_2645408, EPI_ISL_2645409, EPI_ISL_2645410, EPI_ISL_2863589, EPI_ISL_2863591, EPI_ISL_2863593, EPI_ISL_2863695, EPI_ISL_2863714, EPI_ISL_2863716, EPI_ISL_2983374, EPI_ISL_2983375, EPI_ISL_2983376, EPI_ISL_3045424, EPI_ISL_3045425, EPI_ISL_3190169, EPI_ISL_3190170, EPI_ISL_3190171			
see above	Laboratorio Central de Saude Publica do Estado da Bahia (LACEN/BA)	Laboratory of Respiratory Viruses and Measles, Oswaldo Cruz Institute, FIOCRUZ	Alice Sampaio Rocha; Ana Carolina Mendonca; Anna Carolina Paixao; Elisa Cavalcante Pereira; Felicidade Pereira; Fernando Motta; Luciana Appolinario; Marilda Siqueira on behalf of the Fiocruz COVID-19 Genomic Surveillance Network; Paola Resende; Renata Serrano Lopes; Taina Venas
EPI_ISL_3190174, EPI_ISL_3190175, EPI_ISL_3190176, EPI_ISL_3190178, EPI_ISL_3190179, EPI_ISL_3190180, EPI_ISL_3190181, EPI_ISL_3190182, EPI_ISL_3190183, EPI_ISL_3190184, EPI_ISL_3190185, EPI_ISL_3190186, EPI_ISL_3190187, EPI_ISL_3190188, EPI_ISL_3235238, EPI_ISL_3235239, EPI_ISL_3235240, EPI_ISL_3235241, EPI_ISL_3235242, EPI_ISL_3235243, EPI_ISL_3235245, EPI_ISL_3235246, EPI_ISL_3235247, EPI_ISL_3235248, EPI_ISL_3235249, EPI_ISL_3235250, EPI_ISL_3235251, EPI_ISL_3235252, EPI_ISL_3235253, EPI_ISL_3235254, EPI_ISL_3235255, EPI_ISL_3434726, EPI_ISL_3434762, EPI_ISL_3434763, EPI_ISL_3434764, EPI_ISL_3434765, EPI_ISL_3434767, EPI_ISL_3434768, EPI_ISL_3434772, EPI_ISL_3434773, EPI_ISL_3434774, EPI_ISL_3434775, EPI_ISL_3434776, EPI_ISL_3434777, EPI_ISL_3434778, EPI_ISL_3434783, EPI_ISL_3434784, EPI_ISL_3434785, EPI_ISL_3434786, EPI_ISL_3434787, EPI_ISL_3434788, EPI_ISL_3434802, EPI_ISL_3434905, EPI_ISL_3539936			
see above	Laboratorio Central de Saude Publica do Estado da Paraiba (LACEN/PB)	Laboratory of Respiratory Viruses and Measles, Oswaldo Cruz Institute, FIOCRUZ	Alice Sampaio Rocha; Ana Carolina Mendonca; Anna Carolina Paixao; Dalane Loudal Florentino Teixeira; Elisa Cavalcante Pereira; Fernando Motta; Joao Felipe Bezerra; Luciana Appolinario; Marilda Siqueira on behalf of the Fiocruz COVID-19 Genomic Surveillance Network; Paola Resende; Renata Serrano Lopes; Taina Venas
EPI_ISL_2645386, EPI_ISL_2645400, EPI_ISL_2645401, EPI_ISL_2645403, EPI_ISL_2645404, EPI_ISL_2983064, EPI_ISL_2983067, EPI_ISL_2983068, EPI_ISL_2983069, EPI_ISL_2983070, EPI_ISL_2983071, EPI_ISL_2983072, EPI_ISL_2983073, EPI_ISL_2983074, EPI_ISL_2983075, EPI_ISL_2983076, EPI_ISL_2983077, EPI_ISL_2983078, EPI_ISL_2983079, EPI_ISL_2983080, EPI_ISL_2983081, EPI_ISL_2983082, EPI_ISL_2983083, EPI_ISL_2983084, EPI_ISL_2983085, EPI_ISL_2983338, EPI_ISL_2983478, EPI_ISL_3190227, EPI_ISL_3190228, EPI_ISL_3190229, EPI_ISL_3190230, EPI_ISL_3190231, EPI_ISL_3190232, EPI_ISL_3190233, EPI_ISL_3190234, EPI_ISL_3190235, EPI_ISL_3190236, EPI_ISL_3190310, EPI_ISL_3190312, EPI_ISL_3190313, EPI_ISL_3190314, EPI_ISL_3190315, EPI_ISL_3190316, EPI_ISL_3190317, EPI_ISL_3190318, EPI_ISL_3190319, EPI_ISL_3190320, EPI_ISL_3190321, EPI_ISL_3190322, EPI_ISL_3190323, EPI_ISL_3190325, EPI_ISL_3190327, EPI_ISL_3190328, EPI_ISL_3190329, EPI_ISL_3190330, EPI_ISL_3190331, EPI_ISL_3190332, EPI_ISL_3190335, EPI_ISL_3190339, EPI_ISL_3190340, EPI_ISL_3190341, EPI_ISL_3190342, EPI_ISL_3190343, EPI_ISL_3190344, EPI_ISL_3190345, EPI_ISL_3190346, EPI_ISL_3190347, EPI_ISL_3190348, EPI_ISL_3190349, EPI_ISL_3434825, EPI_ISL_3434826, EPI_ISL_3434827, EPI_ISL_3434828, EPI_ISL_3434829, EPI_ISL_3434830, EPI_ISL_3434831, EPI_ISL_3434832, EPI_ISL_3434833, EPI_ISL_3434834, EPI_ISL_3434835, EPI_ISL_3434836, EPI_ISL_3434837, EPI_ISL_3434838, EPI_ISL_3434839, EPI_ISL_3434840, EPI_ISL_3434841, EPI_ISL_3434842, EPI_ISL_3434843, EPI_ISL_3434849, EPI_ISL_3434850, EPI_ISL_3434851, EPI_ISL_3434852, EPI_ISL_3434853, EPI_ISL_3434854, EPI_ISL_3434855, EPI_ISL_3434856, EPI_ISL_3434857, EPI_ISL_3434858, EPI_ISL_3434859, EPI_ISL_3434860, EPI_ISL_3434861, EPI_ISL_3434862, EPI_ISL_3434863, EPI_ISL_3434864, EPI_ISL_3434865, EPI_ISL_3434866, EPI_ISL_3434867, EPI_ISL_3434868, EPI_ISL_3434869, EPI_ISL_3434870, EPI_ISL_3434871, EPI_ISL_3434872, EPI_ISL_3434873, EPI_ISL_3434874, EPI_ISL_3434875, EPI_ISL_3434876, EPI_ISL_3434877, EPI_ISL_3434878, EPI_ISL_3434879, EPI_ISL_3434880, EPI_ISL_3434881, EPI_ISL_3434882, EPI_ISL_3434883, EPI_ISL_3434884, EPI_ISL_3434885, EPI_ISL_3434886, EPI_ISL_3434887, EPI_ISL_3434888, EPI_ISL_3434889, EPI_ISL_3434890, EPI_ISL_3434891, EPI_ISL_3434892, EPI_ISL_3434893, EPI_ISL_3434895, EPI_ISL_3434897, EPI_ISL_3434939, EPI_ISL_3434942, EPI_ISL_3539925, EPI_ISL_3539926, EPI_ISL_3539927, EPI_ISL_3539928			
see above	Laboratorio Central de Saude Publica do Estado de Alagoas (LACEN/AL)	Laboratory of Respiratory Viruses and Measles, Oswaldo Cruz	Agatha Cristinne Prudencio; Agatha Soares; Alice Sampaio Rocha; Ana Carolina Mendonca; Anderson Brandao Leite; Anna Carolina Paixao; Elisa Cavalcante Pereira; Fernando Motta; Ighor Arantes; Ighor Leonardo Arantes Gomes; Luciana Appolinario; Marilda Siqueira on behalf of the Fiocruz COVID-19 Genomic Surveillance Network; Paola Resende; Renata Serrano Lopes; Taina Venas

		Institute, FIOCRUZ	
EPI_ISL_2863647, EPI_ISL_2863648, EPI_ISL_2863651, EPI_ISL_2863653, EPI_ISL_2863655, EPI_ISL_2863656, EPI_ISL_2863658, EPI_ISL_2863660, EPI_ISL_2863662, EPI_ISL_2863664, EPI_ISL_2863666, EPI_ISL_2863712, EPI_ISL_2983218, EPI_ISL_2983339, EPI_ISL_2983340, EPI_ISL_2983342, EPI_ISL_2983343, EPI_ISL_2983344, EPI_ISL_2983345, EPI_ISL_2983346, EPI_ISL_2983347, EPI_ISL_2983348, EPI_ISL_2983349, EPI_ISL_2983350, EPI_ISL_2983351, EPI_ISL_2983352, EPI_ISL_2983353, EPI_ISL_2983354, EPI_ISL_2983355, EPI_ISL_2983356, EPI_ISL_2983357, EPI_ISL_2983359, EPI_ISL_2983360, EPI_ISL_2983361, EPI_ISL_2983362, EPI_ISL_2983363, EPI_ISL_2983364, EPI_ISL_2983365, EPI_ISL_2983366, EPI_ISL_2983367, EPI_ISL_2983368, EPI_ISL_2983369, EPI_ISL_2983370, EPI_ISL_2983371, EPI_ISL_2983372			
see above	Laboratorio Central de Saude Publica do Estado de Santa Catarina (LACEN-SC)	Laboratory of Respiratory Viruses and Measles, Oswaldo Cruz Institute, FIOCRUZ	Agatha Cristinne Prudencio; Alice Sampaio Rocha; Ana Carolina Mendonca; Anna Carolina Paixao; Darcita Buerger Rovaris; Elisa Cavalcante Pereira; Fernando Motta; Ighor Leonardo Arantes Gomes; Luciana Appolinario; Marilda Siqueira on behalf of the Fiocruz COVID-19 Genomic Surveillance Network; Paola Resende; Renata Serrano Lopes; Sandra Bianchini Fernandes; Taina Venas
EPI_ISL_2983383, EPI_ISL_2983384, EPI_ISL_2983385, EPI_ISL_2983387, EPI_ISL_2983388, EPI_ISL_2983389, EPI_ISL_2983391, EPI_ISL_2983392, EPI_ISL_2983394, EPI_ISL_2983395, EPI_ISL_2983397, EPI_ISL_2983398, EPI_ISL_2983400, EPI_ISL_2983402, EPI_ISL_2983403, EPI_ISL_2983404, EPI_ISL_2983405, EPI_ISL_2983406, EPI_ISL_2983407, EPI_ISL_2983409, EPI_ISL_2983410, EPI_ISL_2983411, EPI_ISL_2983412, EPI_ISL_2983414, EPI_ISL_2983415, EPI_ISL_3190167, EPI_ISL_3190219, EPI_ISL_3190221, EPI_ISL_3190222, EPI_ISL_3190223, EPI_ISL_3190224, EPI_ISL_3190225, EPI_ISL_3235294, EPI_ISL_3235296, EPI_ISL_3235297, EPI_ISL_3235298, EPI_ISL_3235300, EPI_ISL_3235301, EPI_ISL_3235303, EPI_ISL_3434908, EPI_ISL_3434910, EPI_ISL_3434911, EPI_ISL_3434912, EPI_ISL_3434913, EPI_ISL_3434914, EPI_ISL_3434915, EPI_ISL_3434925, EPI_ISL_3434926, EPI_ISL_3434927, EPI_ISL_3434928, EPI_ISL_3434929, EPI_ISL_3434930, EPI_ISL_3434931, EPI_ISL_3434932, EPI_ISL_3434933, EPI_ISL_3434934, EPI_ISL_3434935, EPI_ISL_3434936, EPI_ISL_3434937, EPI_ISL_3434940, EPI_ISL_3539246, EPI_ISL_3539247, EPI_ISL_3539248, EPI_ISL_3539249, EPI_ISL_3539770, EPI_ISL_3539771			
see above	Laboratorio Central de Saude Publica do Estado de Santa Catarina (LACEN/SC)	Laboratory of Respiratory Viruses and Measles, Oswaldo Cruz Institute, FIOCRUZ	Alice Sampaio Rocha; Ana Carolina Mendonca; Anna Carolina Paixao; Darcita Buerger Rovaris; Elisa Cavalcante Pereira; Fernando Motta; Luciana Appolinario; Marilda Siqueira on behalf of the Fiocruz COVID-19 Genomic Surveillance Network; Paola Resende; Renata Serrano Lopes; Sandra Bianchini Fernandes; Taina Venas
EPI_ISL_2863573, EPI_ISL_2863575, EPI_ISL_2863621, EPI_ISL_2863623, EPI_ISL_2863626, EPI_ISL_2863630, EPI_ISL_2863637, EPI_ISL_2863639, EPI_ISL_2863641, EPI_ISL_2863643, EPI_ISL_2863645, EPI_ISL_2863719, EPI_ISL_3803004, EPI_ISL_3803006, EPI_ISL_3803007, EPI_ISL_3803008, EPI_ISL_3803009, EPI_ISL_3803010, EPI_ISL_3803012, EPI_ISL_3803014, EPI_ISL_3803015, EPI_ISL_4061451, EPI_ISL_4061452, EPI_ISL_4061453			
see above	Laboratorio Central de Saude Publica do Estado de Sergipe (LACEN/SE)	Laboratory of Respiratory Viruses and Measles, Oswaldo Cruz Institute, FIOCRUZ	Agatha Soares; Alice Sampaio Rocha; Ana Carolina Mendonca; Anna Carolina Paixao; Cliomar Alves dos Santos; Elisa Cavalcante Pereira; Fernando Motta; Ighor Arantes; Luciana Appolinario; Marilda Siqueira on behalf of the Fiocruz COVID-19 Genomic Surveillance Network; Paola Resende; Renata Serrano Lopes; Tainá Moreira Martins Venas
EPI_ISL_3434898, EPI_ISL_3434899, EPI_ISL_3434900, EPI_ISL_3434901, EPI_ISL_3434902, EPI_ISL_3434903, EPI_ISL_3434904, EPI_ISL_3434956, EPI_ISL_3434957, EPI_ISL_3434958, EPI_ISL_3434959, EPI_ISL_3434960, EPI_ISL_3434961, EPI_ISL_3434962, EPI_ISL_3434964, EPI_ISL_3434965, EPI_ISL_3434966, EPI_ISL_3434967, EPI_ISL_3434968, EPI_ISL_3434969, EPI_ISL_3434970, EPI_ISL_3434971, EPI_ISL_3434972, EPI_ISL_3434973, EPI_ISL_3434974, EPI_ISL_3434975, EPI_ISL_3434976, EPI_ISL_3434977, EPI_ISL_3434978, EPI_ISL_3434979, EPI_ISL_3434980, EPI_ISL_3434981, EPI_ISL_3435067, EPI_ISL_3539742, EPI_ISL_3539743, EPI_ISL_3539744, EPI_ISL_3539745, EPI_ISL_3539746, EPI_ISL_3539747, EPI_ISL_3539748, EPI_ISL_3539931			
see above	Laboratorio Central de Saude Publica do Estado do Amapa (LACEN/AP)	Laboratory of Respiratory Viruses and Measles, Oswaldo Cruz Institute, FIOCRUZ	Agatha Cristinne Prudencio; Alice Sampaio Rocha; Ana Carolina Mendonca; Andreia Santos Costa; Anna Carolina Paixao; Anne Caroline da Silva Soledade; Elisa Cavalcante Pereira; Fernando Motta; Ighor Leonardo Arantes Gomes; Lindomar dos Anjos Silva; Luciana Appolinario; Marcia Socorro Pereira Cavalcante; Marilda Siqueira on behalf of the Fiocruz COVID-19 Genomic Surveillance Network; Paola Resende; Renata Serrano Lopes; Taina Venas
EPI_ISL_2375477, EPI_ISL_3045370, EPI_ISL_3045463, EPI_ISL_3045466, EPI_ISL_3045467, EPI_ISL_3045556, EPI_ISL_3190244, EPI_ISL_3190245, EPI_ISL_3190246, EPI_ISL_3190247, EPI_ISL_3190248, EPI_ISL_3190249, EPI_ISL_3190250, EPI_ISL_3190251, EPI_ISL_3190252, EPI_ISL_3190253, EPI_ISL_3434746, EPI_ISL_3434987, EPI_ISL_3434988, EPI_ISL_3434995, EPI_ISL_3434996, EPI_ISL_3434997, EPI_ISL_3434998, EPI_ISL_3435008, EPI_ISL_3435011, EPI_ISL_3435013, EPI_ISL_3435014, EPI_ISL_3435015, EPI_ISL_3435016, EPI_ISL_3435019, EPI_ISL_3435024, EPI_ISL_3435025, EPI_ISL_3435029, EPI_ISL_3435030, EPI_ISL_3435031, EPI_ISL_3435035, EPI_ISL_3435037, EPI_ISL_3435038, EPI_ISL_3435039, EPI_ISL_3827985, EPI_ISL_3827994, EPI_ISL_3827997, EPI_ISL_3828000, EPI_ISL_3828003, EPI_ISL_3828004, EPI_ISL_3828006, EPI_ISL_3828008, EPI_ISL_3828010, EPI_ISL_3828011			
see above	Laboratorio Central de Saude Publica do Estado do Espirito Santo (LACEN/ES)	Laboratory of Respiratory Viruses and Measles, Oswaldo Cruz Institute, FIOCRUZ	Alice Sampaio Rocha; Ana Carolina Mendonca; Anna Carolina Paixao; Eilisa Cavalcante Pereira; Elisa Cavalcante Pereira; Fernando Motta; Luciana Appolinario; Marilda Siqueira on behalf of the Fiocruz COVID-19 Genomic Surveillance Network; Paola Resende; Renata Serrano Lopes; Rodrigo Ribeiro Rodrigues; Taina Venas
EPI_ISL_3190261	Laboratorio Central de Saude Publica do Estado do Maranhao (LACEN-MA)	Laboratory of Respiratory Viruses and Measles, Oswaldo Cruz Institute, FIOCRUZ	Agatha Soares; Alice Sampaio Rocha; Ana Carolina Mendonca; Anna Carolina Paixao; Elisa Cavalcante Pereira; Fernando Motta; Ighor Arantes; Lidio Gonçalves Lima Neto; Luciana Appolinario; Marilda Siqueira on behalf of the Fiocruz COVID-19 Genomic Surveillance Network; Paola Resende; Renata Serrano Lopes; Tainá Venas
EPI_ISL_3913651, EPI_ISL_3913656, EPI_ISL_3913660, EPI_ISL_3913664, EPI_ISL_3913669, EPI_ISL_3913676, EPI_ISL_3913681, EPI_ISL_3913685, EPI_ISL_3913690, EPI_ISL_3913694, EPI_ISL_3913698, EPI_ISL_3913703, EPI_ISL_3913707, EPI_ISL_3913712, EPI_ISL_3913715, EPI_ISL_3913720, EPI_ISL_3913724, EPI_ISL_3913729, EPI_ISL_3913733, EPI_ISL_3913738, EPI_ISL_3913741, EPI_ISL_3913746, EPI_ISL_3913751, EPI_ISL_3913755, EPI_ISL_3913759, EPI_ISL_3913764, EPI_ISL_3913772, EPI_ISL_3913775, EPI_ISL_3913780, EPI_ISL_3913784, EPI_ISL_3913792, EPI_ISL_3913797, EPI_ISL_3913801, EPI_ISL_3913809, EPI_ISL_3913818, EPI_ISL_3913823, EPI_ISL_3913827, EPI_ISL_3913832, EPI_ISL_3913841, EPI_ISL_3913845, EPI_ISL_3913849, EPI_ISL_3913854, EPI_ISL_3913862, EPI_ISL_3913874, EPI_ISL_3913879, EPI_ISL_3913882, EPI_ISL_3913891, EPI_ISL_3913896, EPI_ISL_3913900, EPI_ISL_3913905, EPI_ISL_3913909, EPI_ISL_3913913, EPI_ISL_3913917, EPI_ISL_3913921, EPI_ISL_3913930, EPI_ISL_3913934, EPI_ISL_3913938, EPI_ISL_3913943, EPI_ISL_3913947, EPI_ISL_3913955, EPI_ISL_3913960, EPI_ISL_3913964, EPI_ISL_3913969, EPI_ISL_3913977, EPI_ISL_3913981, EPI_ISL_3913985, EPI_ISL_3913989, EPI_ISL_3913994, EPI_ISL_3914002, EPI_ISL_3914007, EPI_ISL_3914010, EPI_ISL_3914014, EPI_ISL_3914019, EPI_ISL_3914023, EPI_ISL_3914027, EPI_ISL_3914032, EPI_ISL_3914040, EPI_ISL_3914045, EPI_ISL_3914053, EPI_ISL_3914064, EPI_ISL_3914073, EPI_ISL_3914082, EPI_ISL_3914090, EPI_ISL_3914095, EPI_ISL_3914103, EPI_ISL_3914107, EPI_ISL_3914111, EPI_ISL_3914119, EPI_ISL_3914124, EPI_ISL_3914129, EPI_ISL_3914134, EPI_ISL_3914142, EPI_ISL_3914147, EPI_ISL_3914155, EPI_ISL_3914158, EPI_ISL_3914166, EPI_ISL_3914171, EPI_ISL_3914175, EPI_ISL_3914193, EPI_ISL_3914198, EPI_ISL_3914206, EPI_ISL_3914223, EPI_ISL_3914236, EPI_ISL_3914240, EPI_ISL_3914244, EPI_ISL_3914258, EPI_ISL_3914271, EPI_ISL_3914279, EPI_ISL_3914287, EPI_ISL_3914296, EPI_ISL_3914300, EPI_ISL_3914304, EPI_ISL_3914308, EPI_ISL_3914317, EPI_ISL_3914326, EPI_ISL_3914342, EPI_ISL_3914347, EPI_ISL_3914351, EPI_ISL_3914355, EPI_ISL_3914359, EPI_ISL_3914363, EPI_ISL_3914368, EPI_ISL_3914372, EPI_ISL_3914381, EPI_ISL_3914384, EPI_ISL_3914389, EPI_ISL_3914394, EPI_ISL_3914397, EPI_ISL_3914402, EPI_ISL_3914407, EPI_ISL_3914415, EPI_ISL_3914418, EPI_ISL_3914423, EPI_ISL_3914427, EPI_ISL_3914430, EPI_ISL_3914434			
see above	Laboratorio Central de Saude Publica do Estado do Parana (Instituto de Biologia Molecular do Paraná (LACEN-PR)	Instituto Carlos Chagas - Fiocruz	Alessandra De Melo Aguiar; Andreia Akemi Suzukawa; Andréa Rodrigues Ávila; Bruno Dallagiovanna; Dalila Zanette; Eduardo Balsanelli; Emanuel Maltempi de Souza; Fabio Passetti; Fabricio Klerynton Marchini; Fábio de Oliveira Pedrosa; Guilherme Becker; Helisson Faoro; Hellen Geremias dos Santos; Irina Nastassja Riediger; Letusa Albrecht; Lucas Blanes; Luis Gustavo Morello; Lysangela Ronalte Alves; Maria do Carmo Debur; Mauro de Medeiros Oliveira; Michelle Orane Schemberger; Paola Cristina Resende; Sheila Cristina Nardeli; Tiago Gräf; Valter Antônio de Baura
EPI_ISL_2645445, EPI_ISL_2645446, EPI_ISL_2645448, EPI_ISL_2645449, EPI_ISL_2645450, EPI_ISL_2645451, EPI_ISL_2645452, EPI_ISL_2645453, EPI_ISL_2645454, EPI_ISL_2645455, EPI_ISL_2645456, EPI_ISL_2645457, EPI_ISL_2645459, EPI_ISL_2645460, EPI_ISL_2645462, EPI_ISL_2645464, EPI_ISL_2645467, EPI_ISL_2645468, EPI_ISL_2645469, EPI_ISL_2645473, EPI_ISL_2645475, EPI_ISL_2645476, EPI_ISL_2645477, EPI_ISL_2645478, EPI_ISL_2645479, EPI_ISL_2645481, EPI_ISL_2645483, EPI_ISL_2645486, EPI_ISL_2645493, EPI_ISL_2645496, EPI_ISL_2645502, EPI_ISL_2983426, EPI_ISL_2983427, EPI_ISL_2983428, EPI_ISL_2983430, EPI_ISL_2983431, EPI_ISL_2983433, EPI_ISL_2983434, EPI_ISL_2983435, EPI_ISL_2983438, EPI_ISL_2983439, EPI_ISL_2983440, EPI_ISL_2983441, EPI_ISL_2983442, EPI_ISL_2983443, EPI_ISL_2983444, EPI_ISL_2983445, EPI_ISL_2983446, EPI_ISL_2983449, EPI_ISL_2983451, EPI_ISL_3190190, EPI_ISL_3190191, EPI_ISL_3190192, EPI_ISL_3190266, EPI_ISL_3190267, EPI_ISL_3190268, EPI_ISL_3190360, EPI_ISL_3190361, EPI_ISL_3190362, EPI_ISL_3190363, EPI_ISL_3190364, EPI_ISL_3190365, EPI_ISL_3190366, EPI_ISL_3190367, EPI_ISL_3190369, EPI_ISL_3190371, EPI_ISL_3190372, EPI_ISL_3190373, EPI_ISL_3190374, EPI_ISL_3190375, EPI_ISL_3190376, EPI_ISL_3190377, EPI_ISL_3190378, EPI_ISL_3190379, EPI_ISL_3190380, EPI_ISL_3190381, EPI_ISL_3190382, EPI_ISL_3190383, EPI_ISL_3190384, EPI_ISL_3190385, EPI_ISL_3190387, EPI_ISL_3190388, EPI_ISL_3190389, EPI_ISL_3190390, EPI_ISL_3190391, EPI_ISL_3435048			
see above	Laboratorio Central de Saude Publica do Estado do Parana (LACEN/PR)	Laboratory of Respiratory Viruses and Measles, Oswaldo Cruz Institute, FIOCRUZ	Agatha Soares; Alice Sampaio Rocha; Ana Carolina Mendonca; Anna Carolina Paixao; Elisa Cavalcante Pereira; Fernando Motta; Ighor Arantes; Ighor Leonardo Arantes; Irina Riediger; Luciana Appolinario; Marilda Siqueira on behalf of the Fiocruz COVID-19 Genomic Surveillance Network; Paola Resende; Renata Serrano Lopes; Taina Venas
EPI_ISL_3190392, EPI_ISL_3190393, EPI_ISL_3190394, EPI_ISL_3190395, EPI_ISL_3190396, EPI_ISL_3190397, EPI_ISL_3190398, EPI_ISL_3190399, EPI_ISL_3190400, EPI_ISL_3190401, EPI_ISL_3190404, EPI_ISL_3235269, EPI_ISL_3235272, EPI_ISL_3235273, EPI_ISL_3235274, EPI_ISL_3235275			
see above	Laboratorio Central de Saude Publica do Estado do Rio Grande do Norte (LACEN/RN)	Laboratory of Respiratory Viruses and Measles, Oswaldo Cruz Institute, FIOCRUZ	Agatha Soares; Alice Sampaio Rocha; Ana Carolina Mendonca; Ana Paula Ferreira Costa; Anna Carolina Paixao; Antonnyo Palmielly Diogenes Lima; Aurélio de Oliveira Bento; Elisa Cavalcante Pereira; Fernando Motta; Gessika Brenna Costa Alves; Heglayne Pereira Vital da Silva; Iago de Souza Gomes; Ighor Arantes; Isabelle Cristina Clemente dos Santos; Janaina Sonale Cavalcante Nogueira de Oliveira; Jayra Juliana Paiva Alves Abrantes; Jonas José da Silva; Luciana Appolinario; Marilda Siqueira on behalf of the Fiocruz COVID-19 Genomic Surveillance Network; Paola Resende; Renata Serrano Lopes; Taina Venas; Themis Rocha de Souza; Vítor Gabriel Saldanha Fernandes
EPI_ISL_2603441, EPI_ISL_2603529, EPI_ISL_2603530, EPI_ISL_2603531, EPI_ISL_2603532, EPI_ISL_2603533, EPI_ISL_2603534, EPI_ISL_2603535, EPI_ISL_2982762, EPI_ISL_2982763, EPI_ISL_2982764, EPI_ISL_2982765, EPI_ISL_2982766, EPI_ISL_2982767, EPI_ISL_2982768, EPI_ISL_2982775, EPI_ISL_2982776, EPI_ISL_2982777, EPI_ISL_2982781, EPI_ISL_2982784, EPI_ISL_2982788, EPI_ISL_2982792, EPI_ISL_2982795, EPI_ISL_2982796, EPI_ISL_2982797, EPI_ISL_2982798, EPI_ISL_2982800, EPI_ISL_2982801, EPI_ISL_2982802, EPI_ISL_2982803, EPI_ISL_2982807, EPI_ISL_2982808, EPI_ISL_2982811, EPI_ISL_2982813, EPI_ISL_2982814, EPI_ISL_2982815, EPI_ISL_2982816, EPI_ISL_2982818, EPI_ISL_2982819, EPI_ISL_2982820, EPI_ISL_2982821, EPI_ISL_2982824, EPI_ISL_2982826, EPI_ISL_2982827, EPI_ISL_2982828, EPI_ISL_2982829, EPI_ISL_2982830, EPI_ISL_3190303, EPI_ISL_3190305, EPI_ISL_3190406, EPI_ISL_3190407, EPI_ISL_3235278, EPI_ISL_3235280, EPI_ISL_3235281, EPI_ISL_3235282, EPI_ISL_3235283, EPI_ISL_3235289, EPI_ISL_3235290, EPI_ISL_3434907, EPI_ISL_3539241, EPI_ISL_3539242, EPI_ISL_3539243, EPI_ISL_3539244, EPI_ISL_3539767, EPI_ISL_3539768, EPI_ISL_3539769, EPI_ISL_3539840, EPI_ISL_3539841, EPI_ISL_3539842, EPI_ISL_3539843, EPI_ISL_3539845, EPI_ISL_3539846, EPI_ISL_3539847, EPI_ISL_3539848, EPI_ISL_3539849, EPI_ISL_3539850, EPI_ISL_3539851, EPI_ISL_3539852, EPI_ISL_3539853, EPI_ISL_3539855, EPI_ISL_3539856, EPI_ISL_3539858, EPI_ISL_3539859, EPI_ISL_3539861, EPI_ISL_3539863, EPI_ISL_3539864, EPI_ISL_3539865, EPI_ISL_3539867, EPI_ISL_3539868, EPI_ISL_3539869, EPI_ISL_3539870, EPI_ISL_3539871, EPI_ISL_3539872, EPI_ISL_3539873, EPI_ISL_3539874, EPI_ISL_3539875, EPI_ISL_3539876, EPI_ISL_3539877, EPI_ISL_3539878, EPI_ISL_3539880, EPI_ISL_3539881, EPI_ISL_3539882, EPI_ISL_3539883, EPI_ISL_3539884, EPI_ISL_3539885, EPI_ISL_3539886, EPI_ISL_3539887, EPI_ISL_3539888, EPI_ISL_3539891, EPI_ISL_3539892, EPI_ISL_3539893, EPI_ISL_3539894, EPI_ISL_3539895, EPI_ISL_3539896, EPI_ISL_3539897, EPI_ISL_3539898, EPI_ISL_3539901, EPI_ISL_3539902, EPI_ISL_3539903, EPI_ISL_3539904, EPI_ISL_3539905, EPI_ISL_3539938, EPI_ISL_3539939, EPI_ISL_3539940, EPI_ISL_3539941, EPI_ISL_3539943, EPI_ISL_3539944, EPI_ISL_3539945, EPI_ISL_3539946, EPI_ISL_3539947, EPI_ISL_3539948, EPI_ISL_3539949, EPI_ISL_3539950, EPI_ISL_3539951, EPI_ISL_3539952, EPI_ISL_3539953, EPI_ISL_3539954, EPI_ISL_3539956, EPI_ISL_3539957, EPI_ISL_3539958, EPI_ISL_3539961, EPI_ISL_3539962, EPI_ISL_3539963, EPI_ISL_3539964, EPI_ISL_3539965, EPI_ISL_3539966, EPI_ISL_3539967, EPI_ISL_3539968, EPI_ISL_3539970, EPI_ISL_3539971, EPI_ISL_3539972, EPI_ISL_3539973, EPI_ISL_3539974, EPI_ISL_3539975, EPI_ISL_3539976, EPI_ISL_3539977, EPI_ISL_3539978, EPI_ISL_3539979, EPI_ISL_3539980, EPI_ISL_3539985, EPI_ISL_3539986, EPI_ISL_3539988, EPI_ISL_3539989, EPI_ISL_3539990, EPI_ISL_3539991, EPI_ISL_3539992, EPI_ISL_3539993, EPI_ISL_3539994, EPI_ISL_3539995, EPI_ISL_3539996, EPI_ISL_3539997, EPI_ISL_3832410, EPI_ISL_3832411, EPI_ISL_3832412, EPI_ISL_3832413, EPI_ISL_3832414, EPI_ISL_3832415, EPI_ISL_3832416, EPI_ISL_4061391			
see above	Laboratorio Central de Saude Publica do Estado do Rio Grande do Sul (LACEN-RS)	Laboratory of Respiratory Viruses and Measles, Oswaldo Cruz Institute, FIOCRUZ	Agatha Cristinne Soares; Alice Sampaio Rocha; Ana Carolina Mendonca; Anderson Brandao Leite; Anna Carolina Paixao; Elisa Cavalcante Pereira; Fernando Motta; Ighor Leonardo Arantes; Luciana Appolinario; Marilda Siqueira on behalf of the Fiocruz COVID-19 Genomic Surveillance Network; Paola Resende; Renata Serrano Lopes; Richard Salvato; Taina Venas; Tatiana Schaffer Gregianini
EPI_ISL_2863584, EPI_ISL_2863585, EPI_ISL_2863586, EPI_ISL_2863587, EPI_ISL_2863588, EPI_ISL_2863602, EPI_ISL_2863604, EPI_ISL_2863667, EPI_ISL_2863669, EPI_ISL_2863671, EPI_ISL_2863673, EPI_ISL_2863675, EPI_ISL_2863677, EPI_ISL_2863679, EPI_ISL_2863680, EPI_ISL_2863698, EPI_ISL_2863700, EPI_ISL_2863702, EPI_ISL_2863704, EPI_ISL_2863717, EPI_ISL_2863721, EPI_ISL_2863723, EPI_ISL_2863727, EPI_ISL_2982742, EPI_ISL_2982744, EPI_ISL_2982745, EPI_ISL_2982746, EPI_ISL_2982747, EPI_ISL_2982748, EPI_ISL_2982749, EPI_ISL_2982751, EPI_ISL_2982752, EPI_ISL_2982754, EPI_ISL_2982756, EPI_ISL_2983214, EPI_ISL_2983215, EPI_ISL_2983216			
see above	Laboratorio Central de Saude Publica do Estado do Rio de Janeiro (LACEN-RJ)	Laboratory of Respiratory Viruses and Measles, Oswaldo Cruz Institute, FIOCRUZ	Agatha Cristinne Prudencio; Alice Sampaio Rocha; Ana Carolina Mendonca; Andrea Cony Cavalcanti; Anna Carolina Paixao; Elisa Cavalcante Pereira; Fernando Motta; Ighor Leonardo Arantes Gomes; Luciana Appolinario; Marilda Siqueira on behalf of the Fiocruz COVID-19 Genomic Surveillance Network; Paola Resende; Renata Serrano Lopes; Taina Venas
EPI_ISL_3434906	Laboratorio Central de Saude Publica do Estado do Rio de Janeiro (LACEN/RJ)	Laboratory of Respiratory Viruses and Measles, Oswaldo Cruz Institute, FIOCRUZ	Agatha Cristinne Prudencio Soares; Alice Sampaio Rocha; Ana Carolina Mendonca; Andrea Cony Cavalcanti; Anna Carolina Paixao; Elisa Cavalcante Pereira; Fernando Motta; Ighor Leonardo Arantes Gomes; Luciana Appolinario; Marilda Siqueira on behalf of the Fiocruz COVID-19 Genomic Surveillance Network; Paola Resende; Renata Serrano Lopes; Taina Venas
EPI_ISL_3235304, EPI_ISL_3235305, EPI_ISL_3235306, EPI_ISL_3235307, EPI_ISL_3235308, EPI_ISL_3235313	Laboratorio Central de Saude Publica do Estado do Tocantins (LACEN/TO)	Laboratory of Respiratory Viruses and Measles, Oswaldo Cruz Institute, FIOCRUZ	Agatha Soares; Alice Sampaio Rocha; Ana Carolina Mendonca; Anna Carolina Paixao; Elisa Cavalcante Pereira; Fernando Motta; Ighor Arantes; Jucimaria Dantas Galvao; Luciana Appolinario; Marilda Siqueira on behalf of the Fiocruz COVID-19 Genomic Surveillance Network; Paola Resende; Renata Serrano Lopes; Taina Venas
EPI_ISL_3033064, EPI_ISL_3033065, EPI_ISL_3033068, EPI_ISL_3033071, EPI_ISL_3033072, EPI_ISL_3033074, EPI_ISL_3033078, EPI_ISL_3033079, EPI_ISL_3260096, EPI_ISL_3260097, EPI_ISL_3260098, EPI_ISL_3260099, EPI_ISL_3260100, EPI_ISL_3260104, EPI_ISL_3260105			
see above	Laboratorio Central de la Ciudad de Santa Fe	Grupo de Genómica y Bioinformática del Instituto de Investigación de la Cadena Láctea CONICET-INTA on behalf of 'Proyecto Argentino Interinstitucional de genomica de SARS-CoV-2' (PAIS Consortium)	AF; Amadio; C; Eberhardt; G; Irazoqui; JM; MF; Mugna; Ojeda; Pastor; Rompato; V
EPI_ISL_2940274, EPI_ISL_2940275, EPI_ISL_2940285, EPI_ISL_2940290	Laboratorio Central, Ministerio de Salud Córdoba	Instituto de Patologia Vegetal (CIAP-INTA) on behalf of 'Proyecto Argentino Interinstitucional de genomica de SARS-CoV-2' (PAIS Consortium)	Barbas, G.; Castro, G.; Debat, HJ.; FD; Fernández; M; M.B.; Marquez, N.; Pisano; Re, V.
EPI_ISL_2662459	Laboratorio HUB -Azienda Ospedaliero Universitaria - AOU – Cagliari	Laboratorio SPOKE Biologia Molecolare -Azienda Ospedaliero Universitaria - AOU – Cagliari	Alessandra Scano; Ferdinando Coghe; Germano Orrù; Miriam Loddo; Riccardo Cappai; Sara Fais; Valentina Medda
EPI_ISL_3527820, EPI_ISL_3527822, EPI_ISL_3835365,	Laboratorio Nacional de Salud	Asociación de Salud Integral / Clínica Familiar "Luis Ángel García"	Ana S. Gonzalez-Reiche; Claudia Rangel; Danicela Mercado; Eduardo Arathon; Hilda Ruiz; Luis Aguirre; Luis Rivas; Narda Medina; Oscar Bonilla; Osmar Gamboa.

EPI_ISL_3835378, EPI_ISL_3835379, EPI_ISL_3835385			
EPI_ISL_3275241, EPI_ISL_3275242, EPI_ISL_4029319	Laboratorio Nacional de Salud, Ministerio de Salud Publica y Asistencia Social	Genomics and Proteomics Departament, Gorgas Memorial Institute For Health Studies	Alexander A Martinez; Ambar Moreno; Claudia Estrada; Claudia Gonzalez V; César Roberto Conde Pereira; Jessica Gondola; Leyda Abrego; Marlene Castillo; Melissa Gaitan; Oris Chavarria
EPI_ISL_2777453, EPI_ISL_3050307, EPI_ISL_4030314, EPI_ISL_4030315, EPI_ISL_4030316, EPI_ISL_4030330, EPI_ISL_4030332, EPI_ISL_4030338, EPI_ISL_4030344, EPI_ISL_4030345, EPI_ISL_4030356, EPI_ISL_4030359, EPI_ISL_4030365			
see above	Laboratorio de Ecologia de Doencas Transmissiveis na Amazonia, Instituto Leonidas e Maria Deane - Fiocruz Amazonia	Laboratorio de Ecologia de Doencas Transmissiveis na Amazonia, Instituto Leonidas e Maria Deane - Fiocruz Amazonia	Alex Martins; André Corado; Debora Duarte; Fabiola Mendonça da Silva Chui; Felipe Naveca; Fernanda Nascimento; Fernando Fonseca de Almeida Val; George Silva; Gisely Cardoso de Melo; Karina Pessoa; Luciana Gonçalves; Marcus Vinicius Guimarães de Lacerda; Maria Júlia Brandão; Maria Paula Gomes Mourão; Mariana Xavier Simão; Matilde Mejía; Michele Jesus; Valdinete Nascimento; Vanderson de Souza Sampaio; Victor Souza; Ágatha Costa
EPI_ISL_3133693, EPI_ISL_3370436, EPI_ISL_3370440, EPI_ISL_3370456, EPI_ISL_3642594	Laboratorio de Infectologia y Virologia Molecular	Laboratory of Molecular Virology, School of Medicine, Pontificia Universidad Catolica de Chile	Alejandro Bhrun; Ana Maria Contreras; Andres E. Munoz-Marcos; Carlos Palma; Catalina Pardo-Roa; Constanza Maldonado; Constanza Martinez-Valdevenito; Eileen Serrano; Erick Salinas; Estefany Poblete; Francisco Melo; Jennifer Angulo; Jorge Levican; Leonardo I. Almonacid; M. Belen Leyton; Magdalena Vera; Marcela Ferres; Maria Jose Avendano; Rafael A. Medina; Tamara Garcia-Salum
EPI_ISL_2854154, EPI_ISL_3134255	Laboratorio de Medicina Molecular, Universidad de Magallanes	Centro Asistencial Docente y de Investigacion, Universidad de Magallanes	Carolina Pérez; Constanza Ceroni; Diego Alvarez; Inés Cid; Jacqueline Aldridge; Jorge González; Juan Ríos; Marcelo Navarrete; Roberto Uribe-Paredes
EPI_ISL_3048979, EPI_ISL_3048980, EPI_ISL_3048981, EPI_ISL_3048982, EPI_ISL_3048983, EPI_ISL_3048984, EPI_ISL_3048985, EPI_ISL_3048986, EPI_ISL_3048987, EPI_ISL_3048988, EPI_ISL_3048989, EPI_ISL_3048990, EPI_ISL_3048991, EPI_ISL_3048992, EPI_ISL_3048993, EPI_ISL_3048994, EPI_ISL_3048995, EPI_ISL_3048996, EPI_ISL_3048997, EPI_ISL_3048998, EPI_ISL_3049000, EPI_ISL_3049001, EPI_ISL_3049002, EPI_ISL_3049003, EPI_ISL_3049004, EPI_ISL_3049005, EPI_ISL_3049007, EPI_ISL_3049008, EPI_ISL_3049010, EPI_ISL_3049012, EPI_ISL_3049016, EPI_ISL_3049017, EPI_ISL_3049018, EPI_ISL_3049019, EPI_ISL_3049020, EPI_ISL_3049023, EPI_ISL_3049025, EPI_ISL_3049027, EPI_ISL_3049029, EPI_ISL_3055529, EPI_ISL_3055530, EPI_ISL_3055532, EPI_ISL_3761573, EPI_ISL_3761586, EPI_ISL_3761587, EPI_ISL_3761588, EPI_ISL_3761589, EPI_ISL_3761597, EPI_ISL_3761599, EPI_ISL_3761605, EPI_ISL_3761617, EPI_ISL_3761637, EPI_ISL_3761638, EPI_ISL_3761639, EPI_ISL_3761640, EPI_ISL_3761641, EPI_ISL_3761642, EPI_ISL_3761643, EPI_ISL_3761644, EPI_ISL_3761647, EPI_ISL_3761648, EPI_ISL_3761649, EPI_ISL_3761650, EPI_ISL_3761651, EPI_ISL_3761652, EPI_ISL_3761653, EPI_ISL_3761654, EPI_ISL_3761655, EPI_ISL_3761656, EPI_ISL_3761657, EPI_ISL_3761659, EPI_ISL_3761661, EPI_ISL_3761662, EPI_ISL_3761663, EPI_ISL_3761664, EPI_ISL_3761665, EPI_ISL_3761666, EPI_ISL_3761667, EPI_ISL_3761668, EPI_ISL_3761669, EPI_ISL_3761670, EPI_ISL_3761671, EPI_ISL_3761672, EPI_ISL_3761673, EPI_ISL_3761675, EPI_ISL_3761676, EPI_ISL_3761677, EPI_ISL_3761678, EPI_ISL_3761679, EPI_ISL_3761680, EPI_ISL_3761681, EPI_ISL_3761684, EPI_ISL_3761685, EPI_ISL_3761686, EPI_ISL_3761687, EPI_ISL_3761688, EPI_ISL_3761689, EPI_ISL_3761690, EPI_ISL_3761693, EPI_ISL_3761694, EPI_ISL_3761695, EPI_ISL_3761696, EPI_ISL_3761697, EPI_ISL_3761699, EPI_ISL_3761754, EPI_ISL_3761755, EPI_ISL_3761756, EPI_ISL_3761757, EPI_ISL_3761758, EPI_ISL_3761759, EPI_ISL_3761761, EPI_ISL_3761767			
see above	Laboratorio de Pesquisa em Virologia, FAMERP, SJRP	Laboratorio de Pesquisa em Virologia, FAMERP, SJRP	Beatriz de Carvalho Marques; Cecília Artico Banho; Cíntia Bittar; Fábio Sossai Possebon; Guilherme Campos; Helena Lage Ferreira; Jorge A. Petroli Marchesi; João Pessoa Araújo Jr.; Leila Sabrina Ullmann; Lívia Sacchetto; Maisa C. Pereira Parra; Marília Moraes; Maurício L. Nogueira.; Paula Rahal; Paulo Inacio da Costa
EPI_ISL_3672424, EPI_ISL_3672425, EPI_ISL_3672427, EPI_ISL_3672429, EPI_ISL_3672430, EPI_ISL_3672431, EPI_ISL_3672432, EPI_ISL_3672433, EPI_ISL_3672435, EPI_ISL_3672436, EPI_ISL_3672439, EPI_ISL_3826386, EPI_ISL_3826387, EPI_ISL_3826390, EPI_ISL_3826391, EPI_ISL_3826416, EPI_ISL_3826418, EPI_ISL_3826419, EPI_ISL_3826421, EPI_ISL_3826422, EPI_ISL_3826423, EPI_ISL_3826432, EPI_ISL_3826435, EPI_ISL_3826438, EPI_ISL_3826439, EPI_ISL_3826443, EPI_ISL_3826444, EPI_ISL_3991565, EPI_ISL_4052705, EPI_ISL_4052707, EPI_ISL_4052708, EPI_ISL_4052709, EPI_ISL_4052710, EPI_ISL_4052727, EPI_ISL_4052731, EPI_ISL_4080441, EPI_ISL_4080442, EPI_ISL_4080443, EPI_ISL_4080444, EPI_ISL_4080445, EPI_ISL_4080446, EPI_ISL_4080447, EPI_ISL_4080448, EPI_ISL_4080450, EPI_ISL_4080456, EPI_ISL_4080457, EPI_ISL_4080458, EPI_ISL_4080459, EPI_ISL_4080460, EPI_ISL_4080461, EPI_ISL_4080462, EPI_ISL_4080464, EPI_ISL_4080466, EPI_ISL_4080467, EPI_ISL_4080468, EPI_ISL_4080469, EPI_ISL_4080470, EPI_ISL_4080472, EPI_ISL_4080473, EPI_ISL_4080476, EPI_ISL_4080477, EPI_ISL_4080478, EPI_ISL_4080479, EPI_ISL_4080482, EPI_ISL_4080485, EPI_ISL_4080486, EPI_ISL_4080487, EPI_ISL_4080488, EPI_ISL_4080491, EPI_ISL_4080492, EPI_ISL_4080493, EPI_ISL_4080495, EPI_ISL_4080502, EPI_ISL_4080503, EPI_ISL_4080506, EPI_ISL_4080511, EPI_ISL_4080512, EPI_ISL_4080514, EPI_ISL_4080515, EPI_ISL_4080516, EPI_ISL_4080518, EPI_ISL_4080521, EPI_ISL_4080522, EPI_ISL_4080523, EPI_ISL_4080524, EPI_ISL_4080525, EPI_ISL_4080526, EPI_ISL_4080528, EPI_ISL_4080529, EPI_ISL_4080530, EPI_ISL_4080532, EPI_ISL_4080536, EPI_ISL_4080538, EPI_ISL_4080539, EPI_ISL_4080540, EPI_ISL_4080541, EPI_ISL_4080542, EPI_ISL_4080543, EPI_ISL_4080544, EPI_ISL_4080545, EPI_ISL_4080550, EPI_ISL_4080552, EPI_ISL_4080554, EPI_ISL_4080555			
see above	Laboratorio de Referencia Nacional de Virus Respiratorios. Centro Nacional de Salud Publica. Instituto Nacional de Salud Peru.	Laboratorio de Referencia Nacional de Virus Respiratorios. Centro Nacional de Salud Publica. Instituto Nacional de Salud Peru.	Carlos Padilla Rojas; Henri Bailon Calderon; Iris Silva Molina; Joseph Huayra Niquen; Lely Solari Zerpa; Luis Barcena Flores; Marco Galarza Perez; Nancy Rojas Serrano; Nieves Sevilla Castañeda; Omar Caceres Rey; Orson Mestanza Millones; Princesa Medrano Alhuay; Priscila Lope Pari; Sandra Morales Ruiz; Sara Gordillo Vilchez; Steve Acedo Lazo; Veronica Hurtado Vela; Victor Jimenez Vasquez; Wendy Lizarraga Olivares
EPI_ISL_2608291, EPI_ISL_2812650, EPI_ISL_2812707, EPI_ISL_2812709, EPI_ISL_3076938, EPI_ISL_3076940	Laboratorio de Virología HUCA	Laboratorio de Virología HUCA	Abreu F; Alvarez-Arguelles ME; Boga JA; Castelló C; Costales I; Coto E; Gómez de Oña J; Martín-Rodríguez G; Melón S; Perez-Martínez Z; Rojo S; Sandoval M
EPI_ISL_2544205, EPI_ISL_2708965, EPI_ISL_2708968, EPI_ISL_2918882	Laboratorio di Microbiologia	Laboratorio di Microbiologia	Martinetti Lucchini Gladys; Valeria Spina
EPI_ISL_2765565	Laboratorio di Riferimento Regionale della Sicilia Occidentale per l'Emergenza COVID-19	Laboratorio di Riferimento Regionale della Sicilia Occidentale per l'Emergenza COVID-19	Carmelo Massimo Maida; Claudio Costantino; Daniela Di Naro; Fabio Tramuto; Francesco Vitale; Giorgio Graziano; Giulia Randazzo; Vincenzo Restivo; Walter Mazzucco
EPI_ISL_2680918, EPI_ISL_2680925, EPI_ISL_2987688, EPI_ISL_2987689, EPI_ISL_2987691, EPI_ISL_2987692	Laboratorios Delia Barraza	Laboratorio nacional-LANIIA-CIAD	; Alejandra García-Gasca; Alejandra Hernández-Terán; Alejandro Sánchez-Flores; Alfredo Herrera-Estrella; Alicia Ocaña-Mondragón; Andreu Comas-García; Angel Gustavo Salas-Lais; Antonio Loza Román; Bernardo Martínez-Miguel; Blanca Taboada; Brenda Irasema Maldonado-Meza; Bruno Gómez-Gil; Carla Ivón Herrera-Najera; Carlos F. Arias; Celia Boukadida; Clara Esperanza Santacruz-Tinoco; Claudia Soto Félix; Concepción Grajales-Muñiz; Consorcio Mexicano de Vigilancia Genómica (CoViGen-Mex). Authors (in alphabetical order): Julio Elias Alvarado-Yaah; Cristóbal Cháidez-Quiróz; Célida Duque Molina; Célida Martínez- Rodríguez; Daniel Fregoso-Rueda; Daniel Lira Morales; Eduardo Becerril-Vargas; Fernando Fontove-Herrera; Fidencio Mejía-Nepomuceno; Francisco Pulido; Gloria Elena Espinosa-Ayala; Gloria María Molina-Salinas; Gloria Vazquez; Hector Esteban Paz-Juárez; Hector Montoya-Fuentes; Helen Haydee Fernanda Ramírez-Plascencia; Irvin González-López; Jean Pierre González; Jesús Hernández; Joel Armando Vázquez-Pérez. Laboratorios Delia Barraza: María Delia Barraza Sámano; Jorge Salas-Hernández; José Antonio Enciso-Moreno; José Arturo Martínez-Orozco; José Esteban Muñoz-Medina; José de Jesús Nuñez-Contreras; Juan Bautista Chale-Dzul; Julissa Enciso-Ibarra; Luis Alberto Ochoa-Carrera; Margarita Matías-Florentino; Mario Mújica-Sánchez; Marissa Perez-Garcia; María Guadalupe Santiago-Mauricio; María Guadalupe de Jesús Mireles-Rivera; Melissa García Angulo.; Nelly Sélem-Mojica; Pavel Isa; Ricardo Ciria Merce; Ricardo Grande; Rosa María Gutiérrez Rios; Santiago Ávila-Ríos; Selene Zárate; Susana Lopez; Verónica Mata-Haro; Victor Eduardo García-Arias; Víctor Hugo Borja-Aburto
EPI_ISL_2399380, EPI_ISL_2399399, EPI_ISL_2399403, EPI_ISL_2480207, EPI_ISL_2480218, EPI_ISL_2480220, EPI_ISL_2480232, EPI_ISL_2480233, EPI_ISL_2480272, EPI_ISL_2480312, EPI_ISL_2480368, EPI_ISL_2480378, EPI_ISL_2480380, EPI_ISL_2480382, EPI_ISL_2480402, EPI_ISL_2480404, EPI_ISL_2480418, EPI_ISL_2480509, EPI_ISL_2480522, EPI_ISL_2480565, EPI_ISL_2480818, EPI_ISL_2480858, EPI_ISL_2480859, EPI_ISL_2480860, EPI_ISL_2480861, EPI_ISL_2480922, EPI_ISL_2480923, EPI_ISL_2480924, EPI_ISL_2480944, EPI_ISL_2480976, EPI_ISL_2481145, EPI_ISL_2481220, EPI_ISL_2481221, EPI_ISL_2481224, EPI_ISL_2481225, EPI_ISL_2481252, EPI_ISL_2481293, EPI_ISL_2481301, EPI_ISL_2481445, EPI_ISL_2481448, EPI_ISL_2481449, EPI_ISL_2481472, EPI_ISL_2481485, EPI_ISL_2481554, EPI_ISL_2481565, EPI_ISL_2481580, EPI_ISL_2481612, EPI_ISL_2481647, EPI_ISL_2481651, EPI_ISL_2481652, EPI_ISL_2481658, EPI_ISL_2481698, EPI_ISL_2481706, EPI_ISL_2481710, EPI_ISL_2481736, EPI_ISL_2481748, EPI_ISL_2481761, EPI_ISL_2481764, EPI_ISL_2481838, EPI_ISL_2481847, EPI_ISL_2481856, EPI_ISL_2481879, EPI_ISL_2481939, EPI_ISL_2481961, EPI_ISL_2481962, EPI_ISL_2481964, EPI_ISL_2481965, EPI_ISL_2481967, EPI_ISL_2481968, EPI_ISL_2481969, EPI_ISL_2481970, EPI_ISL_2482020, EPI_ISL_2482027, EPI_ISL_2482037, EPI_ISL_2482038, EPI_ISL_2482039, EPI_ISL_2482040, EPI_ISL_2482045, EPI_ISL_2482048, EPI_ISL_2482050, EPI_ISL_2482055, EPI_ISL_2482057, EPI_ISL_2482078, EPI_ISL_2482118, EPI_ISL_2482127, EPI_ISL_2482130, EPI_ISL_2482138, EPI_ISL_2482175, EPI_ISL_2482185, EPI_ISL_2482192, EPI_ISL_2482215, EPI_ISL_2482216, EPI_ISL_2482222, EPI_ISL_2482252, EPI_ISL_2482253, EPI_ISL_2482255, EPI_ISL_2611122, EPI_ISL_2611124, EPI_ISL_2611135, EPI_ISL_2611136, EPI_ISL_2611147, EPI_ISL_2611151, EPI_ISL_2611183, EPI_ISL_2611187, EPI_ISL_2611190, EPI_ISL_2611194, EPI_ISL_2611220, EPI_ISL_2611229, EPI_ISL_2611232, EPI_ISL_2611233, EPI_ISL_2611238, EPI_ISL_2611239, EPI_ISL_2611240, EPI_ISL_2611242, EPI_ISL_2611281, EPI_ISL_2611301, EPI_ISL_2611304, EPI_ISL_2611313, EPI_ISL_2611321, EPI_ISL_2611324, EPI_ISL_2611332, EPI_ISL_2611334, EPI_ISL_2611344, EPI_ISL_2611349, EPI_ISL_2611364, EPI_ISL_2611372, EPI_ISL_2611384, EPI_ISL_2611392, EPI_ISL_2611397, EPI_ISL_2611398, EPI_ISL_2611418, EPI_ISL_2611428, EPI_ISL_2611447, EPI_ISL_2611482, EPI_ISL_2611488, EPI_ISL_2611512, EPI_ISL_2611516, EPI_ISL_2611539, EPI_ISL_2611552, EPI_ISL_2611555, EPI_ISL_2611558, EPI_ISL_2611561, EPI_ISL_2611592, EPI_ISL_2611593, EPI_ISL_2611607, EPI_ISL_2611626, EPI_ISL_2611627, EPI_ISL_2611631, EPI_ISL_2611645, EPI_ISL_2611646, EPI_ISL_2611656, EPI_ISL_2611767, EPI_ISL_2611812, EPI_ISL_2611823, EPI_ISL_2872170, EPI_ISL_2872173, EPI_ISL_2872187, EPI_ISL_2872208, EPI_ISL_2872299, EPI_ISL_2872305, EPI_ISL_2872334, EPI_ISL_2872430, EPI_ISL_2872473, EPI_ISL_2872474, EPI_ISL_2872620, EPI_ISL_2872677, EPI_ISL_2872710, EPI_ISL_2872735, EPI_ISL_2872736, EPI_ISL_2872785, EPI_ISL_2872809, EPI_ISL_2872810, EPI_ISL_2872831, EPI_ISL_2872841, EPI_ISL_2872842, EPI_ISL_2872844, EPI_ISL_2872873, EPI_ISL_2872874, EPI_ISL_2872881, EPI_ISL_2872927, EPI_ISL_2872939, EPI_ISL_2872941, EPI_ISL_2872997, EPI_ISL_2873078, EPI_ISL_2873088, EPI_ISL_2873122, EPI_ISL_2873124, EPI_ISL_2873165, EPI_ISL_2873167, EPI_ISL_2873189, EPI_ISL_2873190, EPI_ISL_2873196, EPI_ISL_2873199, EPI_ISL_2873200, EPI_ISL_2873201, EPI_ISL_2873221, EPI_ISL_2873222, EPI_ISL_2873227, EPI_ISL_2873232, EPI_ISL_2873239, EPI_ISL_2873244, EPI_ISL_2873254, EPI_ISL_2873255, EPI_ISL_2873279, EPI_ISL_2873282, EPI_ISL_2873284, EPI_ISL_2873320, EPI_ISL_2926208, EPI_ISL_2926213, EPI_ISL_2926255, EPI_ISL_2926257, EPI_ISL_2926272, EPI_ISL_2926339, EPI_ISL_2926360, EPI_ISL_2926369, EPI_ISL_2926376, EPI_ISL_2926396, EPI_ISL_2926410, EPI_ISL_2926415, EPI_ISL_2926429, EPI_ISL_2926451, EPI_ISL_2926500, EPI_ISL_2926514, EPI_ISL_2926523, EPI_ISL_2926530, EPI_ISL_2926531, EPI_ISL_2926532, EPI_ISL_2926537, EPI_ISL_2926676, EPI_ISL_2926745, EPI_ISL_2926778, EPI_ISL_2926847, EPI_ISL_2926903, EPI_ISL_2926963, EPI_ISL_2926966, EPI_ISL_2926978, EPI_ISL_2927001, EPI_ISL_2927084, EPI_ISL_2927114, EPI_ISL_2927115, EPI_ISL_2927132, EPI_ISL_2927159, EPI_ISL_2927167, EPI_ISL_2927240, EPI_ISL_2927242, EPI_ISL_2927265, EPI_ISL_2927307, EPI_ISL_2927312, EPI_ISL_2927320, EPI_ISL_2927321, EPI_ISL_2927327, EPI_ISL_2927332, EPI_ISL_2927340, EPI_ISL_2927360, EPI_ISL_2927367, EPI_ISL_2927392, EPI_ISL_2927393, EPI_ISL_2927402, EPI_ISL_2927486, EPI_ISL_2927489, EPI_ISL_2927548, EPI_ISL_2927588, EPI_ISL_2927626, EPI_ISL_2927646, EPI_ISL_2927676, EPI_ISL_2927691, EPI_ISL_2927694, EPI_ISL_2927695, EPI_ISL_2927715, EPI_ISL_3016915, EPI_ISL_3016930, EPI_ISL_3016967, EPI_ISL_3016975, EPI_ISL_3017014, EPI_ISL_3017017, EPI_ISL_3017019, EPI_ISL_3017026, EPI_ISL_3017174, EPI_ISL_3017176, EPI_ISL_3017221, EPI_ISL_3017242, EPI_ISL_3017279, EPI_ISL_3017327, EPI_ISL_3017348, EPI_ISL_3017349, EPI_ISL_3017519, EPI_ISL_3017520, EPI_ISL_3017521, EPI_ISL_3017548, EPI_ISL_3017579, EPI_ISL_3017588, EPI_ISL_3017589, EPI_ISL_3017598, EPI_ISL_3017614, EPI_ISL_3017615, EPI_ISL_3017654, EPI_ISL_3017665, EPI_ISL_3017697, EPI_ISL_3017717, EPI_ISL_3017719, EPI_ISL_3017731, EPI_ISL_3017743, EPI_ISL_3017744, EPI_ISL_3017776, EPI_ISL_3017801, EPI_ISL_3017802, EPI_ISL_3017812, EPI_ISL_3017833, EPI_ISL_3017855, EPI_ISL_3017887, EPI_ISL_3017905, EPI_ISL_3017911, EPI_ISL_3017950, EPI_ISL_3017969, EPI_ISL_3017992, EPI_ISL_3017994, EPI_ISL_3018005, EPI_ISL_3018006, EPI_ISL_3018095, EPI_ISL_3018106, EPI_ISL_3018151, EPI_ISL_3020069, EPI_ISL_3111330, EPI_ISL_3111339, EPI_ISL_3111358, EPI_ISL_3111457, EPI_ISL_3111471, EPI_ISL_3111489, EPI_ISL_3111491, EPI_ISL_3111492, EPI_ISL_3111576, EPI_ISL_3111677, EPI_ISL_3111779, EPI_ISL_3111811, EPI_ISL_3111814, EPI_ISL_3111848, EPI_ISL_3112180, EPI_ISL_3112302, EPI_ISL_3112304, EPI_ISL_3112416, EPI_ISL_3112459, EPI_ISL_3112478, EPI_ISL_3112540, EPI_ISL_3112569, EPI_ISL_3112652, EPI_ISL_3112764, EPI_ISL_3112995, EPI_ISL_3113038, EPI_ISL_3113219, EPI_ISL_3113280, EPI_ISL_3113287, EPI_ISL_3113321, EPI_ISL_3113359, EPI_ISL_3113497, EPI_ISL_3113502, EPI_ISL_3113622, EPI_ISL_3325548, EPI_ISL_3325577, EPI_ISL_3325732, EPI_ISL_3325734, EPI_ISL_3326343, EPI_ISL_3326376, EPI_ISL_3326546, EPI_ISL_3326562, EPI_ISL_3326572, EPI_ISL_3326649, EPI_ISL_3326911, EPI_ISL_3328025, EPI_ISL_3328026, EPI_ISL_3328443, EPI_ISL_3328455, EPI_ISL_3328467, EPI_ISL_3328481, EPI_ISL_3328507, EPI_ISL_3328514, EPI_ISL_3328532, EPI_ISL_3328657, EPI_ISL_3328756, EPI_ISL_3330637, EPI_ISL_3330657, EPI_ISL_3330704			
see above	Laboratory Corporation of America	Centers for Disease Control and Prevention Division of Viral Diseases, Pathogen Discovery	; Adrian Paskey; Amanda Douglas; Amanda Suchanek; Andrea Throop; Ayla Burns; Benjamin Rambo-Martin; Bobbi Croy; Brian Krueger; Brian Norvell; Christopher Gulvick; Christos Petropoulos; Clinton R. Paden; Craig Lukasik; Dakota Howard; Darlene Wagner; Debbie Boles; Dhwani Batra; Duncan MacCannell; Eyad Almasri; Goran Stevovic; Howard Engler; Hrushikesh Deshmukh; Jake Humphrey; Jana Schroth; Jason Caravas; Joe Voshell; John Pruitt; Jonathan Meltzer; Jonathan Williams; Kara Moser; Kimberly Wagner; Lax Iyer; Lisa Pfefferle; Lyndon Tilson; Manoj Jain; Marcia Eisenberg; Mary Ann Cristobal; Mary Williamson; Matthew Robinson; Matthew Schmerer; Michael Levandoski; Mike Sapeta; Mindy Nye; Minoo Agarwal; Mohan Kolli; Nuthawin Charoensri; Oren Cohen; Peter W. Cook; Prashant Gupta; Qian Zeng; Rama Ghatti; Scott Parker; Scott Ryan; Scott Sammons; Shatavia Morrison; Stanley Letovsky; Steven Ragan; Suresh Babu Selvaraju; Susan Countryman; Susan Hicks; Suzanne Dale; Thomas Urban; Tim Kuphal; Tricia Zwiefelhofer; Vincent Drouillon; Yvette Unoarumhi
EPI_ISL_2811913	Laboratory Medicine, University of Washington	University of Washington/Fred Hutch	Castor; J.L.
EPI_ISL_2557405, EPI_ISL_2557407, EPI_ISL_2557409, EPI_ISL_2557410, EPI_ISL_2645506, EPI_ISL_2863605, EPI_ISL_2863607, EPI_ISL_2863609, EPI_ISL_2863611, EPI_ISL_2863613, EPI_ISL_2863614, EPI_ISL_2863616, EPI_ISL_2863689, EPI_ISL_2863691, EPI_ISL_2863693, EPI_ISL_2863797, EPI_ISL_2863798, EPI_ISL_2863799, EPI_ISL_2863800, EPI_ISL_2863897, EPI_ISL_2982717, EPI_ISL_2982718, EPI_ISL_2982719, EPI_ISL_2982720, EPI_ISL_2982721, EPI_ISL_2982722, EPI_ISL_2982724, EPI_ISL_2982725, EPI_ISL_2982726, EPI_ISL_2982729, EPI_ISL_2982730, EPI_ISL_2982731, EPI_ISL_2982732, EPI_ISL_2982733, EPI_ISL_2982736, EPI_ISL_2982737, EPI_ISL_2982738, EPI_ISL_2982740, EPI_ISL_2982865, EPI_ISL_2982866, EPI_ISL_2983187, EPI_ISL_2983236, EPI_ISL_2983237, EPI_ISL_2983379, EPI_ISL_2983380, EPI_ISL_2983381, EPI_ISL_2983382, EPI_ISL_3045374, EPI_ISL_3045375, EPI_ISL_3045376, EPI_ISL_3045377, EPI_ISL_3045382, EPI_ISL_3045383, EPI_ISL_3045423, EPI_ISL_3045470, EPI_ISL_3045472, EPI_ISL_3045473, EPI_ISL_3045474, EPI_ISL_3045475, EPI_ISL_3045476, EPI_ISL_3045477, EPI_ISL_3045478, EPI_ISL_3045479, EPI_ISL_3045480, EPI_ISL_3045481, EPI_ISL_3045482, EPI_ISL_3045485, EPI_ISL_3045488, EPI_ISL_3045489, EPI_ISL_3045490, EPI_ISL_3045491, EPI_ISL_3045492, EPI_ISL_3045494, EPI_ISL_3045495, EPI_ISL_3045496, EPI_ISL_3045497, EPI_ISL_3045498, EPI_ISL_3045499, EPI_ISL_3045500, EPI_ISL_3045501, EPI_ISL_3045502, EPI_ISL_3045503, EPI_ISL_3045505, EPI_ISL_3045506, EPI_ISL_3045507, EPI_ISL_3045511, EPI_ISL_3045512, EPI_ISL_3045513, EPI_ISL_3045515, EPI_ISL_3045518, EPI_ISL_3045519, EPI_ISL_3045521, EPI_ISL_3045522, EPI_ISL_3045523, EPI_ISL_3045524, EPI_ISL_3045525, EPI_ISL_3045526, EPI_ISL_3045527, EPI_ISL_3045528, EPI_ISL_3045529, EPI_ISL_3045531, EPI_ISL_3045532, EPI_ISL_3045533, EPI_ISL_3045537, EPI_ISL_3045538, EPI_ISL_3045539, EPI_ISL_3045540, EPI_ISL_3045544, EPI_ISL_3045545, EPI_ISL_3045546, EPI_ISL_3045547, EPI_ISL_3045548, EPI_ISL_3045549, EPI_ISL_3045551, EPI_ISL_3045552, EPI_ISL_3045553, EPI_ISL_3190168, EPI_ISL_3435049, EPI_ISL_3435050, EPI_ISL_3435051, EPI_ISL_3435052, EPI_ISL_3539998, EPI_ISL_3539999			
see above	Laboratory of Respiratory Viruses and Measles, Oswaldo Cruz Institute, FIOCRUZ	Laboratory of Respiratory Viruses and Measles, Oswaldo Cruz Institute, FIOCRUZ	Agatha Cristinne Prudencio; Agatha Cristinne Prudencio Soares; Alice Sampaio Rocha; Ana Carolina Mendonca; Anna Carolina Paixao; Elisa Cavalcante Pereira; Fernando Motta; Ighor Leonardo Arantes Gomes; Luciana Appolinario; Marilda Siqueira on behalf of the Fiocruz COVID-19 Genomic Surveillance Network; Paola Resende; Renata Serrano Lopes; Taina Venas
EPI_ISL_3025606, EPI_ISL_3025615, EPI_ISL_3025620, EPI_ISL_3025626, EPI_ISL_3461216, EPI_ISL_3461217, EPI_ISL_3461239, EPI_ISL_3461240, EPI_ISL_3461243, EPI_ISL_3461285, EPI_ISL_3461328			
see above	Laboratory, Gravity Diagnostics	Laboratory, Gravity Diagnostics	Diagnostics, G.
EPI_ISL_2777293, EPI_ISL_2777512, EPI_ISL_2777564, EPI_ISL_2777616, EPI_ISL_2777631, EPI_ISL_2777651, EPI_ISL_2777658, EPI_ISL_2777669, EPI_ISL_2777690, EPI_ISL_2777692, EPI_ISL_2777806, EPI_ISL_2777863, EPI_ISL_2777896, EPI_ISL_2777897, EPI_ISL_2777898, EPI_ISL_3050298, EPI_ISL_3050302, EPI_ISL_3050303, EPI_ISL_3050308, EPI_ISL_3050309, EPI_ISL_3050312, EPI_ISL_3050315, EPI_ISL_3050319, EPI_ISL_3050322, EPI_ISL_3050332, EPI_ISL_3050337, EPI_ISL_3050344, EPI_ISL_3050354, EPI_ISL_3050363, EPI_ISL_3050367, EPI_ISL_3050375, EPI_ISL_3050376, EPI_ISL_3050384, EPI_ISL_3050387, EPI_ISL_3050391, EPI_ISL_3050394, EPI_ISL_3050401, EPI_ISL_3050403, EPI_ISL_3050406, EPI_ISL_3050407, EPI_ISL_3050415, EPI_ISL_3050431, EPI_ISL_3050434, EPI_ISL_3050436, EPI_ISL_3050464, EPI_ISL_3050470, EPI_ISL_3050471, EPI_ISL_3050490, EPI_ISL_3050501, EPI_ISL_3050502, EPI_ISL_3050503, EPI_ISL_3050506, EPI_ISL_3050519, EPI_ISL_3050529, EPI_ISL_3050538, EPI_ISL_3050551, EPI_ISL_3050558, EPI_ISL_3050565, EPI_ISL_3050566, EPI_ISL_3050570, EPI_ISL_3050593, EPI_ISL_3050605, EPI_ISL_3050624, EPI_ISL_3050627, EPI_ISL_3050636			
see above	Laboratório Central de Saúde Pública do Amazonas - LACEN-AM	Laboratorio de Ecologia de Doencas Transmissiveis na Amazonia, Instituto Leonidas e Maria Deane - Fiocruz Amazonia	André Corado; Debora Duarte; Felipe Naveca; Fernanda Nascimento; George Silva; Karina Pessoa; Luciana Gonçalves; Maria Júlia Brandão; Matilde Mejía; Michele Jesus; Valdinete Nascimento; Victor Souza; Ágatha Costa
EPI_ISL_3426438, EPI_ISL_3584949, EPI_ISL_3585952, EPI_ISL_3587490, EPI_ISL_3602957, EPI_ISL_3602958, EPI_ISL_3602960, EPI_ISL_3602964, EPI_ISL_3610353, EPI_ISL_3610354, EPI_ISL_3610355, EPI_ISL_3610356, EPI_ISL_3610358, EPI_ISL_3610359			
see above	Laboratório Central de Saúde Pública do Estado do Tocantins (LACEN/TO)	Bioinformatics and Biotechnology Laboratory (Labinftec/Federal	Bergmann Morais Ribeiro; Chácaras 69/72; Fabrício Souza Campos; Fernando Lucas Melo; Gurupi/TO Ueric José Borges de Souza; Jucimária Dantas Galvão; Laboratório de Bioinformática e Biotecnologia; Lote 7; Raíssa Nunes dos Santos; Rua Badejós; Ueric José Borges de Souza; Zona Rural | 77.402-970| UFT Campus de Gurupi

		University of Tocantins, Campus of Gurupi)	
EPI_ISL_2835216, EPI_ISL_2835220, EPI_ISL_2835221, EPI_ISL_2835222, EPI_ISL_2835223, EPI_ISL_2983455, EPI_ISL_2983457, EPI_ISL_2983459, EPI_ISL_2983460, EPI_ISL_2983462, EPI_ISL_2983463, EPI_ISL_2983464, EPI_ISL_2983466, EPI_ISL_2983467, EPI_ISL_2983468, EPI_ISL_3539775, EPI_ISL_3539776, EPI_ISL_3539777, EPI_ISL_3539778, EPI_ISL_3539779, EPI_ISL_3539780, EPI_ISL_3539781, EPI_ISL_3539782, EPI_ISL_3539783, EPI_ISL_3539784, EPI_ISL_3539785, EPI_ISL_3539786, EPI_ISL_3539787, EPI_ISL_3539789, EPI_ISL_3539790			
see above	Laboratório de Biotecnologia Aplicada (LBA) - Laboratório de Biologia Molecular - Hospital das Clínicas, Faculdade de Medicina de Botucatu, Departamento de Bioprocessos e Biotecnologia - Faculdade de Ciências Agronômicas, UNESP – Botucatu/SP	Laboratory of Respiratory Viruses and Measles, Oswaldo Cruz Institute, FIOCRUZ	Alice Sampaio Rocha; Ana Carolina Mendonca; Anna Carolina Paixao; Elisa Cavalcante Pereira; Felipe Allan da Silva Costa; Fernando Motta; Jayme Augusto de Souza Neto; Leonardo Nazario de Moraes; Luciana Appolinario; Marilda Siqueira on behalf of the Fiocruz COVID-19 Genomic Surveillance Network; Paola Resende; Patricia Akemi Assato; Rejane Maria Tommasini; Renata Serrano Lopes; Taina Venas
EPI_ISL_3456068, EPI_ISL_3456069, EPI_ISL_3873632, EPI_ISL_3873661, EPI_ISL_3873663, EPI_ISL_3873665, EPI_ISL_3873666			
see above	Laboratório de Microbiologia Molecular - Universidade FEEVALE	Molecular Microbiology Laboratory	Alana Witt Hansen; Fernando Rosado Spilki; Fágner Henrique Heldt; Juliana Schons Gulart; Juliana Schons Gularte; Juliane Deise Fleck; Mariana Soares da Silva; Matheus Nunes Weber; Meriane Demoliner; Michele Filippi.; Micheli Filippi; Paula Rodrigues de Almeida; Vyctoria Malayhka de Abreu Góes Pereira.
EPI_ISL_2557412, EPI_ISL_2557413	Labortorio Central de Saude Publica do Estado de Santa Catarina (LACEN/SC)	Laboratory of Respiratory Viruses and Measles, Oswaldo Cruz Institute, FIOCRUZ	Alice Sampaio Rocha; Ana Carolina Mendonca; Anna Carolina Paixao; Darcita Buerger Rovaris; Elisa Cavalcante Pereira; Fernando Motta; Luciana Appolinario; Marilda Siqueira on behalf of the Fiocruz COVID-19 Genomic Surveillance Network; Paola Resende; Renata Serrano Lopes; Sandra Bianchini Fernandes; Taina Venas
EPI_ISL_2645505, EPI_ISL_2863891, EPI_ISL_3045378, EPI_ISL_3045379, EPI_ISL_3045380, EPI_ISL_3190193, EPI_ISL_3190194, EPI_ISL_3190195, EPI_ISL_3190196, EPI_ISL_3190197, EPI_ISL_3190198, EPI_ISL_3190199, EPI_ISL_3190200, EPI_ISL_3190203, EPI_ISL_3190204, EPI_ISL_3190205, EPI_ISL_3190207, EPI_ISL_3190209, EPI_ISL_3190210, EPI_ISL_3190214, EPI_ISL_3190216, EPI_ISL_3190296			
see above	Labortorio Central de Saude Publica do Estado do Rio de Janeiro (LACEN/RJ)	Laboratory of Respiratory Viruses and Measles, Oswaldo Cruz Institute, FIOCRUZ	Agatha Cristinne Prudencio Soares; Alice Sampaio Rocha; Ana Carolina Mendonca; Andrea Cony Cavalcanti; Anna Carolina Paixao; Elisa Cavalcante Pereira; Fernando Motta; Ighor Leonardo Arantes Gomes; Luciana Appolinario; Marilda Siqueira on behalf of the Fiocruz COVID-19 Genomic Surveillance Network; Paola Resende; Renata Serrano Lopes; Taina Venas
EPI_ISL_2645507, EPI_ISL_2645512, EPI_ISL_2645513, EPI_ISL_2863600, EPI_ISL_2983418, EPI_ISL_2983419, EPI_ISL_2983420, EPI_ISL_2983422, EPI_ISL_2983423			
see above	Labortorio Central de Saude Publica do Estado do Tocantins (LACEN/TO)	Laboratory of Respiratory Viruses and Measles, Oswaldo Cruz Institute, FIOCRUZ	Alice Sampaio Rocha; Ana Carolina Mendonca; Anna Carolina Paixao; Elisa Cavalcante Pereira; Fernando Motta; Jucimaria Dantas Galvao; Luciana Appolinario; Marilda Siqueira on behalf of the Fiocruz COVID-19 Genomic Surveillance Network; Paola Resende; Renata Serrano Lopes; Taina Venas
EPI_ISL_2613809, EPI_ISL_2613810, EPI_ISL_2613811, EPI_ISL_2613812, EPI_ISL_2613813, EPI_ISL_2613814, EPI_ISL_2613815			
see above	Lboratorio Central de Saude Publica do Estado do Parana (LACEN/PR)	Laboratory of Respiratory Viruses and Measles, Oswaldo Cruz Institute, FIOCRUZ	Alice Sampaio Rocha; Ana Carolina Mendonca; Anna Carolina Paixao; Elisa Cavalcante Pereira; Fernando Motta; Irina Riediger; Luciana Appolinario; Marilda Siqueira on behalf of the Fiocruz COVID-19 Genomic Surveillance Network; Paola Resende; Renata Serrano Lopes; Taina Venas
EPI_ISL_2392333, EPI_ISL_2395953, EPI_ISL_2437005, EPI_ISL_2446442	Lighthouse Lab in Milton Keynes	Wellcome Sanger Institute for the COVID-19 Genomics UK (COG-UK) Consortium	Cordelia Langford; David K. Jackson; Dominic Kwiatkowski; Ewan Harrison; Ian Johnston; Jeffrey Barrett; John Sillitoe on behalf of the Wellcome Sanger Institute COVID-19 Surveillance Team; Roberto Amato; Sonia Goncalves; The Lighthouse Lab in Milton Keynes and Alex Alderton
EPI_ISL_2390202	Limbach - MVZ Labor Ravensburg Labor Dr. Gärtner	Robert Koch Institute	
EPI_ISL_2842581, EPI_ISL_2842582, EPI_ISL_2842583, EPI_ISL_2842587, EPI_ISL_2842594	Lurie Children's Hospital of Chicago	Northwestern University - Ozer Lab	Egon A. Ozer; Judd F. Hultquist; Lacy M. Simons; Larry K. Kociolek; Michael G. Ison; Ramon Lorenzo-Redondo; Taylor J. Dean; William J. Muller; Xiaotian; Zheng
EPI_ISL_3102429, EPI_ISL_3102455	MATERNIDADE ESCOLA ASSIS CHATEAUBRIAND	Analytical Competence Molecular Epidemiology Lab/ACME, Oswaldo Cruz Foundation, Ceara (FIOCRUZ CE)	Cleber Furtado Aksenen; Fabio Miyajima; Fernando Braga Stehling; Francisco Eder de Moura Lopes; Jamille Maria Mendes Bezerra; Joaquim César do Nascimento Sousa Junior; Pedro Miguel Carneiro Jeronimo; Suzana Porto Almeida e Lucas Delerino; Thais Ferreira de Oliveira; Thais de Oliveira Costa; Ticiane Cavalcante de Souza; Veridiana Pessoa Miyajima
EPI_ISL_2598083, EPI_ISL_2709816, EPI_ISL_2790050, EPI_ISL_2861207, EPI_ISL_2862024, EPI_ISL_2955557, EPI_ISL_2955566, EPI_ISL_2955569, EPI_ISL_2980037, EPI_ISL_3020114, EPI_ISL_3045923, EPI_ISL_3099901			
see above	MD PHL	MD PHL	Maryland Department of Health Laboratories Administration
EPI_ISL_2957399	MDI Limbach Berlin GmbH; MVZ Labor Berlin	Robert Koch Institute	
EPI_ISL_3230761	MEDILAB	CHU Poitiers	Agnes BEBY-DEFAUX; Birama N'DIAYE; Caroline MICHAUD; Magali GARCIA; Manon PRAT; Maxime PICHON; Nicolas LEVEQUE; Valentin BON-BARET
EPI_ISL_2659675, EPI_ISL_2659754	MEPHI, Aix Marseille University	MEPHI, Aix Marseille University	Anthony LEVASSEUR
EPI_ISL_2626996	MONTEFIORE MEDICAL CENTER LABORATORIES	Wadsworth Center, New York State Department of Health	Alexis Russell; Catharine Prussing; Daryl M. Lamson; Erasmus Schneider; Erica Lasek-Nesselquist; John Kelly; Jonathan Plitnick; Kirsten St. George; Matthew Shudt; Melissa A Leisner; Navjot Singh
EPI_ISL_2787554	MS Public Health Laboratory	Centers for Disease Control and Prevention Division of Viral Diseases, Pathogen Discovery	Alison Laufer Halpin; Ben L. Rambo-Martin; Clinton R. Paden; Dakota Howard; Darlene Wagner; Dave Wentworth; Dhwani Batra; Jasmine Padilla; Justin Lee; Katie Dillon; Krista Queen; Kristen Knipe; Kristine Lacek; Mark Burroughs; Matthew Schmerer; Mili Sheth; Peter Cook; Sam Shepard; Sarah Nobles; Shoshona Le; Suxiang Tong; Vivien Dugan; Yvette Unoarumhi
EPI_ISL_2646337, EPI_ISL_2646340, EPI_ISL_2858795, EPI_ISL_2858801	MS Public Health Laboratory	University of Mississippi Medical Center, Molecular and Genomics Core Facility	Ashley C. Johnson; D. Ashley Robinson; Ithiel J. Frame; Krishna K. Ayyalasomayajula; Michael R. Garrett; Wenjie Wu
EPI_ISL_2690127	MT Public Health Laboratory	Centers for Disease Control and Prevention Division of Viral Diseases, Pathogen Discovery	Alison Laufer Halpin; Ben L. Rambo-Martin; Clinton R. Paden; Dakota Howard; Darlene Wagner; Dave Wentworth; Dhwani Batra; Jasmine Padilla; Justin Lee; Katie Dillon; Krista Queen; Kristen Knipe; Kristine Lacek; Mark Burroughs; Matthew Schmerer; Mili Sheth; Peter Cook; Sam Shepard; Sarah Nobles; Shoshona Le; Suxiang Tong; Vivien Dugan; Yvette Unoarumhi
EPI_ISL_2472447, EPI_ISL_2472482, EPI_ISL_2632831, EPI_ISL_2635874, EPI_ISL_2635888, EPI_ISL_2762510	MVZ Labor Dr. Limbach & Kollegen GbR	Robert Koch Institute	
EPI_ISL_2647377, EPI_ISL_2647466, EPI_ISL_2647483, EPI_ISL_2647487, EPI_ISL_2647528, EPI_ISL_2647529, EPI_ISL_2647588, EPI_ISL_2647651, EPI_ISL_2647655, EPI_ISL_2647694, EPI_ISL_2647714, EPI_ISL_2647716, EPI_ISL_2647845, EPI_ISL_2647854, EPI_ISL_2647866, EPI_ISL_2647869, EPI_ISL_2647889, EPI_ISL_2647890, EPI_ISL_2647936, EPI_ISL_2647956, EPI_ISL_2786002, EPI_ISL_2786004, EPI_ISL_2786086, EPI_ISL_2786093, EPI_ISL_2786115, EPI_ISL_2786129, EPI_ISL_2786191, EPI_ISL_2786251, EPI_ISL_2786283, EPI_ISL_2786329, EPI_ISL_2786442, EPI_ISL_2786460, EPI_ISL_2786470, EPI_ISL_2786508, EPI_ISL_2786527, EPI_ISL_2786580, EPI_ISL_2875242, EPI_ISL_2875281, EPI_ISL_2875282, EPI_ISL_2875290, EPI_ISL_2875351, EPI_ISL_2875368, EPI_ISL_2875397, EPI_ISL_2875402, EPI_ISL_2875438, EPI_ISL_2875477, EPI_ISL_2875483, EPI_ISL_2875503, EPI_ISL_3065124, EPI_ISL_3065150, EPI_ISL_3065172, EPI_ISL_3065282, EPI_ISL_3065479, EPI_ISL_3065499, EPI_ISL_3110617, EPI_ISL_3110717, EPI_ISL_3110748, EPI_ISL_3110749, EPI_ISL_3110831, EPI_ISL_3110860, EPI_ISL_3110893, EPI_ISL_3110919			
see above	Mako Medical	Centers for Disease Control and Prevention Division of Viral Diseases, Pathogen Discovery	Adrian Paskey; Benjamin Rambo-Martin; Christopher Gulvick; Clinton R. Paden; Dakota Howard; Darlene Wagner; Dhwani Batra; Duncan MacCannell; Jason Caravas; Kara Moser; Lauren Moon; Matthew Schmerer; Matthew Tugwell; Peter W. Cook; Scott Sammons; Shatavia Morrison; Yvette Unoarumhi
EPI_ISL_3009153, EPI_ISL_3009168, EPI_ISL_3435356, EPI_ISL_3435357, EPI_ISL_3435359	Maryland Genomics, Institute for Genome Sciences, University of Maryland School of Medicine	Maryland Genomics, Institute for Genome Sciences, University of Maryland School of Medicine	Claire M; Fraser; Hazen; Holly; Humphrys; Ivette; Jacques; Jonathan; Kranthi; Lim; Lisa D; Luke J; Mike; Ott; Ravel; Roussey; Sadzewicz; Sandra; Santana-Cruz; Tallon; Tracy; Vavikolanu
EPI_ISL_2550770	Maryland Public Health Laboratory (MD PHL)	Maryland Public Health Laboratory (MD PHL)	Maryland Department of Health Laboratories Administration
EPI_ISL_2484936, EPI_ISL_2688808, EPI_ISL_2688819, EPI_ISL_2920258, EPI_ISL_2920266, EPI_ISL_2920271	Massachusetts State Public Health Laboratory	Massachusetts State Public Health Laboratory	Andrew Lang; Glen Gallagher; Sandra Smole; Timelia Fink
EPI_ISL_2958947	Mayo Clinic & Mayo Clinic Laboratories	Minnesota Department of Health, Public Health Laboratory	Alexandra Lorentz; Jacob Garfin; Matt Plumb; and Xiong Wang
EPI_ISL_2544257	Medica	Institute of Medical Virology	Alexandra Trkola; Annette Audigé; Cyril Shah; Gabriela Ziltener; Guido Bloemberg; Jon Huder; Jürg Böni; Kevin Steiner; Maria Grünberg; Maryam Zaheri; Michael Huber; Riccarda Capaul; Stefan Schmutz; Verena Kufner
EPI_ISL_2636815	Medizinisch-Diagnostisches Labor Kempten allgäulab	Robert Koch Institute	
EPI_ISL_2433970, EPI_ISL_2433982	Microbiologia CATLAB	Can Ruti SARS-CoV-2 Sequencing Hub (HUGTiP/IrsiCaixa/IGTP)	Alba Sánchez; Anna Not; Antoni E Bordoy; Bonaventura Clotet; Cristina Casañ; Cristina Esteban; Francesc Catala-Moll; Gemma Clara; Ignacio Blanco; Marc Noguera-Julian; Maria Casadellà; Mariona Parera; Mercedes Guerrero; Montserrat Giménez; Pere-Joan Cardona; Pilar Armengol; Roger Paredes; Verónica Saludes; and Elisa Martró on behalf of the Can Ruti SARS-CoV-2 Sequencing Hub.

EPI_ISL_2448064, EPI_ISL_2650271, EPI_ISL_2650293, EPI_ISL_2650328, EPI_ISL_2832110, EPI_ISL_2832172	Microbiologia CATLAB	Can Ruti SARS-CoV-2 Sequencing Hub (HUGTiP/IrsiCaixa/IGTP)	Alba Snchez; Alba Sánchez; Alexia París; Anna Not; Antoni E Bordoy; Bonaventura Clotet; Cristina Casa; Cristina Casañ; Cristina Esteban; David Panisello; Francesc Catala-Moll; Gemma Clara; Ignacio Blanco; Laia Soler; Lauro Sumoy; Marc Noguera-Julian; Maria Casadell; Maria Casadellà; Mariona Parera; Mercedes Guerrero; Montserrat Gimnez; Montserrat Giménez; Pere-Joan Cardona; Pilar Armengol; Roger Paredes; Vernica Saludes; Verónica Saludes; and Elisa Martr on behalf of the Can Ruti SARS-CoV-2 Sequencing Hub.; and Elisa Martró on behalf of the Can Ruti SARS-CoV-2 Sequencing Hub
EPI_ISL_2448054, EPI_ISL_2448086, EPI_ISL_2671448, EPI_ISL_2812471, EPI_ISL_2812485, EPI_ISL_2832166, EPI_ISL_2832188, EPI_ISL_2897003, EPI_ISL_2897006, EPI_ISL_2926161, EPI_ISL_2926172, EPI_ISL_3027975, EPI_ISL_3163990, EPI_ISL_3320201, EPI_ISL_3320217, EPI_ISL_3455752			
see above	Microbiology Department, Laboratori Clínic Metropolitana Nord. Hospital Universitari Germans Trias i Pujol	Can Ruti SARS-CoV-2 Sequencing Hub (HUGTiP/IrsiCaixa/IGTP)	Alba Snchez; Alba Sánchez; Alexia París; Anna Not; Antoni E Bordoy; Bonaventura Clotet; Cristina Casa; Cristina Casañ; Cristina Esteban; David Panisello; Francesc Catala-Moll; Gemma Clara; Ignacio Blanco; Laia Soler; Lauro Sumoy; Marc Noguera-Julian; Maria Casadell; Maria Casadellà; Mariona Parera; Mercedes Guerrero; Montserrat Gimnez; Montserrat Giménez; Pere-Joan Cardona; Pilar Armengol; Roger Paredes; Vernica Saludes; Verónica Saludes; and Elisa Martr on behalf of the Can Ruti SARS-CoV-2 Sequencing Hub.; and Elisa Martró on behalf of the Can Ruti SARS-CoV-2 Sequencing Hub
EPI_ISL_2756396, EPI_ISL_2774104	Microbiology Department, University Hospital Donostia	Microbiology Department, University Hospital Donostia	Cilla G.; Gomez M; Marimon JM; Martin-Peñaranda T; Montes M; Piñeiro L; Sorarrain A
EPI_ISL_2501367, EPI_ISL_2501368, EPI_ISL_2861303, EPI_ISL_2967893	Microbiology Department. Complexo Hospitalario Universitario de Vigo	Microbiology Department. Complexo Hospitalario Universitario de Vigo	Alfaya N; Alonso I; Alvarez M; Cabrera JJ; Carballo R; Cores O; Cortizo S; Davina C; Martinez L; Mediero G; Perez S; Potel C; Regueiro B; Rey S; Vasallo FJ; del-Campo V
EPI_ISL_3026893	Microbiology Division, SC DHEC	Microbiology Division, SC DHEC	Freeman; J.S.
EPI_ISL_2936682, EPI_ISL_2958605, EPI_ISL_2984697, EPI_ISL_2984741, EPI_ISL_2987761, EPI_ISL_2987789, EPI_ISL_2987790, EPI_ISL_2987793, EPI_ISL_2987795, EPI_ISL_2987796, EPI_ISL_2987810, EPI_ISL_2987811, EPI_ISL_2987812, EPI_ISL_2987813, EPI_ISL_2987815, EPI_ISL_2987816, EPI_ISL_2987817, EPI_ISL_2987818, EPI_ISL_2987819, EPI_ISL_2987923, EPI_ISL_2987973, EPI_ISL_2988015, EPI_ISL_2988020, EPI_ISL_3032541, EPI_ISL_3032559, EPI_ISL_3032569, EPI_ISL_3032574, EPI_ISL_3032580, EPI_ISL_3032586, EPI_ISL_3032587, EPI_ISL_3032633, EPI_ISL_3032636, EPI_ISL_3032638, EPI_ISL_3032644, EPI_ISL_3032654, EPI_ISL_3032706, EPI_ISL_3032817, EPI_ISL_3063340, EPI_ISL_3063345, EPI_ISL_3063349, EPI_ISL_3063351, EPI_ISL_3063361, EPI_ISL_3063365, EPI_ISL_3066700, EPI_ISL_3066701, EPI_ISL_3066703, EPI_ISL_3066705, EPI_ISL_3066706, EPI_ISL_3066707, EPI_ISL_3066709, EPI_ISL_3066710, EPI_ISL_3066726, EPI_ISL_3066727, EPI_ISL_3066729, EPI_ISL_3066730, EPI_ISL_3066744, EPI_ISL_3066745, EPI_ISL_3404496, EPI_ISL_3404498, EPI_ISL_3404499, EPI_ISL_3404541, EPI_ISL_3906776, EPI_ISL_3906902, EPI_ISL_3907002, EPI_ISL_3907003			
see above	Ministry of Health Turkey	Ministry of Health Turkey	Fatma Bayrakdar; Gulay Korukluoglu; Gülay Korukluoğlu; Suleyman Yalcin; Süleyman Yalcin; Yasemin Cosgun; Yasemin Coşgun
EPI_ISL_2425383, EPI_ISL_2501152, EPI_ISL_2565864, EPI_ISL_2658515, EPI_ISL_2727839, EPI_ISL_2727855, EPI_ISL_2958944, EPI_ISL_3099434, EPI_ISL_3099435, EPI_ISL_3099436			
see above	Minnesota Department of Health, Public Health Laboratory	Minnesota Department of Health, Public Health Laboratory	Alexandra Lorentz; Jacob Garfin; Matt Plumb; and Xiong Wang
EPI_ISL_2635085, EPI_ISL_2753648, EPI_ISL_3011018	Missouri State Public Health Laboratory	Missouri State Public Health Laboratory	Ashley New; Joshua Barry; Matthew Sinn
EPI_ISL_3142934, EPI_ISL_3142935	Molecular & Genomic Pathology Laboratory, Thomas Jefferson University Hospital	Molecular & Genomic Pathology Laboratory, Thomas Jefferson University Hospital	Nitika Badjatia; Run Jin; Shannon Mahoney; Zi-xuan Wang
EPI_ISL_2692159, EPI_ISL_2692168, EPI_ISL_2710337, EPI_ISL_2731437, EPI_ISL_2941725, EPI_ISL_2941763, EPI_ISL_2943174, EPI_ISL_2943199, EPI_ISL_2943208, EPI_ISL_2943219, EPI_ISL_2943220, EPI_ISL_2982696, EPI_ISL_2982701, EPI_ISL_2982709			
see above	Montana Public Health Laboratory	Montana Public Health Laboratory	Carrie Biskupiak; Deborah Gibson; Joy Ritter; Michael Dills; Michelle Mozer
EPI_ISL_4060426	NC State Laboratory of Public Health	Centers for Disease Control and Prevention Division of Viral Diseases, Pathogen Discovery	Alex Burgin; Ben Rambo-Martin; Clinton Paden; Dakota Howard; Dave Wentworth; Dhwani Batra; Jasmine Padilla; Justin Lee; Krista Queen; Kristen Knipe; Kristine Lacek; Mark Burroughs; Matthew Schmerer; Meghan Bentz; Mili Sheth; Peter Cook; Sam Shepard; Sarah Nobles; Suxiang Tong; Vivien Dugan; Yvette Unoarumhi
EPI_ISL_3242077	ND Dept. of Health Laboratory Services-Microbiology	Centers for Disease Control and Prevention Division of Viral Diseases, Pathogen Discovery	Alex Burgin; Ben L. Rambo-Martin; Clinton R. Paden; Dakota Howard; Dave Wentworth; Dhwani Batra; Jasmine Padilla; Justin Lee; Krista Queen; Kristen Knipe; Kristine Lacek; Mark Burroughs; Matthew Schmerer; Meghan Bentz; Mili Sheth; Peter Cook; Sam Shepard; Sarah Nobles; Suxiang Tong; Vivien Dugan; Yvette Unoarumhi
EPI_ISL_2689917, EPI_ISL_2689927, EPI_ISL_2689930, EPI_ISL_2987007	NE Public Health Laboratory	Centers for Disease Control and Prevention Division of Viral Diseases, Pathogen Discovery	Alison Laufer Halpin; Ben L. Rambo-Martin; Clinton R. Paden; Dakota Howard; Darlene Wagner; Dave Wentworth; Dhwani Batra; Jasmine Padilla; Justin Lee; Katie Dillon; Krista Queen; Kristen Knipe; Kristine Lacek; Mark Burroughs; Matthew Schmerer; Mili Sheth; Peter Cook; Sam Shepard; Sarah Nobles; Shoshona Le; Suxiang Tong; Vivien Dugan; Yvette Unoarumhi
EPI_ISL_2689938, EPI_ISL_2787857, EPI_ISL_2787928, EPI_ISL_3800472	NJ Public Health and Environmental Laboratories	Centers for Disease Control and Prevention Division of Viral Diseases, Pathogen Discovery	Alex Burgin; Alison Laufer Halpin; Ben L. Rambo-Martin; Ben Rambo-Martin; Clinton Paden; Clinton R. Paden; Dakota Howard; Darlene Wagner; Dave Wentworth; Dhwani Batra; Jasmine Padilla; Justin Lee; Katie Dillon; Krista Queen; Kristen Knipe; Kristine Lacek; Mark Burroughs; Matthew Schmerer; Meghan Bentz; Mili Sheth; Peter Cook; Sam Shepard; Sarah Nobles; Shoshona Le; Suxiang Tong; Vivien Dugan; Yvette Unoarumhi
EPI_ISL_2443124, EPI_ISL_2557117, EPI_ISL_2557131, EPI_ISL_2557134, EPI_ISL_2557148, EPI_ISL_2557157, EPI_ISL_2649830, EPI_ISL_2649831, EPI_ISL_2842649, EPI_ISL_2842660, EPI_ISL_2842684, EPI_ISL_3023754, EPI_ISL_3129992			
see above	NJDOH, Public Health and Environmental Laboratories	NJ_PHEL	Allison Roder; Byeong Jeong; Chelsea San Filippo; Dana Woell; Jacquelyn Deverell; Lindsey Bodnar; Ryan Pachucki; Shiv K. Verma
EPI_ISL_2627155, EPI_ISL_2627177, EPI_ISL_2627179, EPI_ISL_3031060, EPI_ISL_3031066, EPI_ISL_3031067	NORTHWELL HEALTH LABORATORIES	Wadsworth Center, New York State Department of Health	Alexis Russell; Catharine Prussing; Daryl M. Lamson; Erasmus Schneider; Erica Lasek-Nesselquist; John Kelly; Jonathan Plitnick; Kirsten St. George; Matthew Shudt; Melissa A Leisner; Navjot Singh
EPI_ISL_2821280, EPI_ISL_2821281, EPI_ISL_2821282, EPI_ISL_2821283, EPI_ISL_2821284, EPI_ISL_3134752, EPI_ISL_3134754, EPI_ISL_3134755, EPI_ISL_3134759, EPI_ISL_3134760, EPI_ISL_3134763, EPI_ISL_3134765, EPI_ISL_3134768, EPI_ISL_3134769, EPI_ISL_3134770, EPI_ISL_3134771, EPI_ISL_3134772, EPI_ISL_3134774, EPI_ISL_3134775, EPI_ISL_3134776, EPI_ISL_3134778, EPI_ISL_3134779, EPI_ISL_3134780, EPI_ISL_3134781, EPI_ISL_3134783, EPI_ISL_3134784, EPI_ISL_3134785, EPI_ISL_3134786, EPI_ISL_3134789, EPI_ISL_3134790, EPI_ISL_3134792, EPI_ISL_3134793, EPI_ISL_3134794, EPI_ISL_3134795, EPI_ISL_3134796, EPI_ISL_3134797, EPI_ISL_3134798, EPI_ISL_3134799, EPI_ISL_3134800, EPI_ISL_3134801, EPI_ISL_3134803, EPI_ISL_3134804, EPI_ISL_3134805, EPI_ISL_3134806, EPI_ISL_3134808, EPI_ISL_3134813, EPI_ISL_3447458, EPI_ISL_3447490, EPI_ISL_3447491, EPI_ISL_3447492, EPI_ISL_3447493, EPI_ISL_3447494, EPI_ISL_3447495, EPI_ISL_3447496, EPI_ISL_3447498, EPI_ISL_3447500, EPI_ISL_3447501, EPI_ISL_3447502, EPI_ISL_3447503, EPI_ISL_3447504, EPI_ISL_3447505, EPI_ISL_3447506, EPI_ISL_3447507, EPI_ISL_3447508, EPI_ISL_3447510, EPI_ISL_3447511, EPI_ISL_3447512, EPI_ISL_3447513, EPI_ISL_3447514, EPI_ISL_3447515, EPI_ISL_3447516, EPI_ISL_3447517, EPI_ISL_3447518, EPI_ISL_3447519, EPI_ISL_3447521, EPI_ISL_3447522, EPI_ISL_3447524, EPI_ISL_3447525, EPI_ISL_3447526, EPI_ISL_3447527, EPI_ISL_3447528, EPI_ISL_3447529, EPI_ISL_3447530, EPI_ISL_3447531, EPI_ISL_3447533, EPI_ISL_3447534, EPI_ISL_3447535, EPI_ISL_3447536, EPI_ISL_3447537, EPI_ISL_3447538, EPI_ISL_3447539, EPI_ISL_3447540, EPI_ISL_3447541, EPI_ISL_3447542, EPI_ISL_3447543, EPI_ISL_3447545, EPI_ISL_3447546, EPI_ISL_3447547, EPI_ISL_3447548, EPI_ISL_3447549, EPI_ISL_3447550, EPI_ISL_3447551, EPI_ISL_3447552, EPI_ISL_3447553, EPI_ISL_3447554, EPI_ISL_3447556, EPI_ISL_3447557, EPI_ISL_3447558, EPI_ISL_3447559, EPI_ISL_3447560, EPI_ISL_3447561, EPI_ISL_3447562, EPI_ISL_3447563, EPI_ISL_3447564, EPI_ISL_3447565, EPI_ISL_3447566, EPI_ISL_3447567, EPI_ISL_3447568, EPI_ISL_3447594, EPI_ISL_3447595, EPI_ISL_3447596, EPI_ISL_3447597, EPI_ISL_3703782, EPI_ISL_3703783, EPI_ISL_3703784, EPI_ISL_3703785, EPI_ISL_3703787, EPI_ISL_3703788, EPI_ISL_3703789, EPI_ISL_3703792, EPI_ISL_3703794, EPI_ISL_3703796, EPI_ISL_3703797, EPI_ISL_3703798, EPI_ISL_3703799, EPI_ISL_3703801, EPI_ISL_3703813, EPI_ISL_3703816, EPI_ISL_3703823, EPI_ISL_3703827, EPI_ISL_3703831, EPI_ISL_3703834, EPI_ISL_3703846, EPI_ISL_3703849, EPI_ISL_3703853, EPI_ISL_3703868, EPI_ISL_3703876, EPI_ISL_3703886, EPI_ISL_3703905, EPI_ISL_3703909, EPI_ISL_3703912, EPI_ISL_3703917, EPI_ISL_3703928, EPI_ISL_3703938, EPI_ISL_3703948, EPI_ISL_3703958, EPI_ISL_3703962, EPI_ISL_3703969, EPI_ISL_3703972, EPI_ISL_3703980, EPI_ISL_3703991, EPI_ISL_3704002, EPI_ISL_3704012, EPI_ISL_3704016, EPI_ISL_3704024, EPI_ISL_3704035, EPI_ISL_3704043, EPI_ISL_3704066, EPI_ISL_3704070, EPI_ISL_3704074			
see above	NUPIT/UFPE	WallauLab on behalf of Fiocruz COVID-19 Genomic Surveillance Network	Alexandre Freitas da Silva; Antonio Mauro Rezende; Cassia Docena; Constância Flávia Junqueira Ayres; Cássia Docena; Duschinka Ribeiro Duarte Guedes; Elisama Helvecio; Filipe Zimmer Dezordi; Gabriel Luz Wallau; Gustavo Barbosa de Lima; Lais Ceschini Machado; Larissa Krokovsky; Laís Ceschini Machado; Lilian Caroliny Amorim Silva; Maira Galdino da Rocha Pitta; Marcelo Henrique dos Santos Paiva; Matheus Filgueira Bezerra; Michelly Cristiny Pereira; Rômulo Pessoa e Silva; Sinval Pinto Brandão Filho
EPI_ISL_2663535, EPI_ISL_2753941	NYU Langone Health	Departments of Pathology and Medicine, New York University School of Medicine	Adriana Heguy; Christian Marier; Dacia Dimartino; Emily Guzman; Gael Westby; Guiqing Wang; Paolo Cotzia; Paul Zappile; Peter Meyn; Sitharam Ramaswami; Yutong Zhang
EPI_ISL_3063113	National Institute of Public Health	National Institute of Public Health	Alexander Nagy; Dusan Trnka; Helena Jirincova; Jaromira Vecerova; Timotej Suri
EPI_ISL_2621962, EPI_ISL_2621963	National Public Health Laboratory, National Centre for Infectious Diseases	National Public Health Laboratory, National Centre for Infectious Diseases	Lin Cui; Raymond Tzer Pin Lin; Royce Ang; Tze Minn Mak; Zhenyang Zhou
EPI_ISL_2308536, EPI_ISL_2382430, EPI_ISL_2466750, EPI_ISL_2597931, EPI_ISL_2689123	Nebraska Public Health Laboratory	NPHL COVID-19 Response Team	NPHL COVID-19 Response Team
EPI_ISL_2612507	Nevada State Public Health Laboratory	Nevada State Public Health Laboratory	Andrew Gorzalski; Mark Pandori
EPI_ISL_2652076, EPI_ISL_2941647, EPI_ISL_3086158	New Mexico Department of Health Scientific Laboratory	New Mexico Department of Health Scientific Laboratory	; Anastacia Griego-Fisher; D'eldra Malone; Ellie Johnson; Jennifer Benoit; Keila Gutierrez; Linda Salazar; Ratheesh Rajan
EPI_ISL_3230464	NordLab Kajaani	Expert Microbiology, National Institute for Health and Welfare	Carita Savolainen-Kopra; Erika Lindh; Haider al-Hello; Jani Halkilahti; Kirsi Liitsola; Niina Ikonen; Olli Vapalahti; Pekka Ellonen; Phuoc Truong; Päivi Laurila; Ravi Kant; Sari Hannula; Soile Blomqvist; Teemu Smura
EPI_ISL_2736189, EPI_ISL_2736201	Northwest Laboratory	Seattle Flu Study	Amanda Adler; Barry R. Lutz; Benjamin Pelle; Brian Hiatt; Caitlin R. Wolf; Chris D. Frazar; Deborah A. Nickerson; Elisabeth Brandstetter; Erica Ryke; Geoff Melly; Helen Y. Chu; Janet A. Englund; Jay Shendure; Jover Lee; Kairsten Fay; Kirsten Lacombe; Lea M. Starita; Mark J. Rieder; Matthew Richardson; Matthew Thompson; Melissa Truong; Michael Boeckh; Michael Famulare; Misja Ilcisin; Peter D. Han; Philip Dykema; Romesh Gautom; Scott Lindquist; Thomas R. Sibley; Trevor Bedford
EPI_ISL_2373049, EPI_ISL_2842596, EPI_ISL_2842597, EPI_ISL_2842605, EPI_ISL_2842608, EPI_ISL_2842612, EPI_ISL_2842613			
see above	Northwestern Memorial Hospital	Northwestern University - Ozer Lab	Chad J. Achenbach; Chao Qi; Egon A. Ozer; Judd F. Hultquist; Lacy M. Simons; Lawrence J. Jennings; Michael G. Ison; Ramon Lorenzo-Redondo; Taylor J. Dean
EPI_ISL_3388710	Northwestern Memorial Hospital	RIPHL at Rush University Medical Center	Amber Kimble; Chao Qi; Edith Perez; Felix Araujo Perez; Kevin Kunstman; Laura Furtado; Marieta Hyde; Max Kolton; Stefan Green
EPI_ISL_2838856, EPI_ISL_2838857, EPI_ISL_2838858, EPI_ISL_2838859, EPI_ISL_2838860, EPI_ISL_2838861, EPI_ISL_2838862, EPI_ISL_2838863, EPI_ISL_2838864, EPI_ISL_2838865, EPI_ISL_2838866, EPI_ISL_2838867, EPI_ISL_2838869, EPI_ISL_2838870, EPI_ISL_2838871, EPI_ISL_2838872, EPI_ISL_2838873, EPI_ISL_2838874, EPI_ISL_2838875, EPI_ISL_2838876, EPI_ISL_2838877, EPI_ISL_2838878, EPI_ISL_2838879, EPI_ISL_2838880, EPI_ISL_2838881, EPI_ISL_2838882, EPI_ISL_2838883			
see above	OHSU Lab Services Molecular Microbiology	Oregon SARS-CoV-2 Genome	'[UsersoroakBox SyncPHI_SARS-COV-2_Sample_Sheet_infoGISAID_Submission_Infometa_master_2021.xlsx]release20_final'!D:AC; 26; FALSE); VLOOKUP(C466; VLOOKUP(C468; VLOOKUP(C474; VLOOKUP(C477; VLOOKUP(C483; VLOOKUP(C488; VLOOKUP(C502; VLOOKUP(C503; VLOOKUP(C504;

	Lab	Sequencing Center	VLOOKUP(C505; VLOOKUP(C508; VLOOKUP(C510; VLOOKUP(C516; VLOOKUP(C517; VLOOKUP(C518; VLOOKUP(C519; VLOOKUP(C521; VLOOKUP(C522; VLOOKUP(C525; VLOOKUP(C526; VLOOKUP(C528; VLOOKUP(C529; VLOOKUP(C530; VLOOKUP(C533; VLOOKUP(C534; VLOOKUP(C539; VLOOKUP(C547
EPI_ISL_2689979, EPI_ISL_2689981, EPI_ISL_2689982, EPI_ISL_2689984, EPI_ISL_2787421, EPI_ISL_2787436, EPI_ISL_2787447, EPI_ISL_2787450, EPI_ISL_2787453, EPI_ISL_2787459, EPI_ISL_2840415, EPI_ISL_2987015, EPI_ISL_3355222			
see above	OR State PHL-Virology/Immunology Section	Centers for Disease Control and Prevention Division of Viral Diseases, Pathogen Discovery	Alex Burgin; Alison Laufer Halpin; Ben L. Rambo-Martin; Ben Rambo-Martin; Clinton Paden; Clinton R. Paden; Dakota Howard; Darlene Wagner; Dave Wentworth; Dhwani Batra; Jasmine Padilla; Justin Lee; Katie Dillon; Krista Queen; Kristen Knipe; Kristine Lacek; Mark Burroughs; Matthew Schmerer; Meghan Bentz; Mili Sheth; Peter Cook; Sam Shepard; Sarah Nobles; Shoshona Le; Suxiang Tong; Vivien Dugan; Yvette Unoarumhi
EPI_ISL_2966615	Ochsner Health	Bioinfoexperts, LLC	Amy Feehan; David J. Nolan; Judy Crabtree; Julia-Garcia-Diaz; Lucio Miele; Rebecca Rose; Susanna L. Lamers; Tessa LaFleur
EPI_ISL_2621529	Omega Diagnostics at Mounes	Omega Diagnostics at Mounes	Ashleigh McGrail; Cynthia Corley; Latira Haynes-Jacob; MD; Vivek Khare
EPI_ISL_2423535, EPI_ISL_2604871	Omics Sciences Laboratory	Omics Sciences Laboratory	Darlyn Amaya; Derly Andrade Molina; Gabriel Morey León; Juan Carlos Fernández Cadena; Rubén Armas González
EPI_ISL_2928154, EPI_ISL_2928156, EPI_ISL_2928158, EPI_ISL_2928161, EPI_ISL_2928167, EPI_ISL_2928169, EPI_ISL_2928172, EPI_ISL_2928173, EPI_ISL_2928176, EPI_ISL_2928183, EPI_ISL_2928193, EPI_ISL_2928195, EPI_ISL_2928196, EPI_ISL_3010309, EPI_ISL_3099977			
see above	Orange County Public Health Laboratory	Orange County Public Health Laboratory	Angelica Torres; Julia Wolfe
EPI_ISL_2424249, EPI_ISL_2424250, EPI_ISL_2473664, EPI_ISL_2473665, EPI_ISL_2473666, EPI_ISL_2473667, EPI_ISL_2557922, EPI_ISL_2557923, EPI_ISL_2557926, EPI_ISL_2604198, EPI_ISL_2604205, EPI_ISL_2604209, EPI_ISL_2612540, EPI_ISL_2612541, EPI_ISL_2612545, EPI_ISL_2631151, EPI_ISL_2631155, EPI_ISL_2631187, EPI_ISL_2727464, EPI_ISL_2727476, EPI_ISL_2727487, EPI_ISL_2790735, EPI_ISL_2790737, EPI_ISL_2790739, EPI_ISL_2790740, EPI_ISL_2790741, EPI_ISL_2790742, EPI_ISL_2790744, EPI_ISL_2790745, EPI_ISL_2790746, EPI_ISL_2893422, EPI_ISL_2893423, EPI_ISL_2893424, EPI_ISL_2893425, EPI_ISL_2893426, EPI_ISL_2893427, EPI_ISL_2893428, EPI_ISL_2893429, EPI_ISL_2928905, EPI_ISL_2933949, EPI_ISL_2955407, EPI_ISL_3010589, EPI_ISL_3010594, EPI_ISL_3048118, EPI_ISL_3048120, EPI_ISL_3067393, EPI_ISL_3090726			
see above	Oregon State Public Health Laboratory	Oregon State Public Health Laboratory	Eugene Yeboah; John Fontana and Shane Sevey; Laura Tsaknaridis; Rafia Razzaque; Vanda Makris
EPI_ISL_2925501	Ospedale di Genzano	National Institute for Infectious Diseases (INMI) L. Spallanzani I.R.C.C.S	A Di Caro; B Bartolini; CEM Gruber; E Giombini; F Messina; F Santini; G Bonfiglio; M Rueca; MR Capobianchi; O Butera
EPI_ISL_3219518, EPI_ISL_3219519, EPI_ISL_3219520, EPI_ISL_3219521, EPI_ISL_3219522, EPI_ISL_3219523	Outre mer	National Reference Center for Viruses of Respiratory Infections, Institut Pasteur, Paris	Angela Brisebarre; Camille Capel; Christophe Malabat; Corinne Maufrais; Dominique Rousset; Etienne Simon-Lorière; Frédéric Lemoine; Hub de Bioinformatique et Biostatistique; Louise Lefrançois; Marion Barbet; Maud Vanpeene; Méline Bizard; Sylvie Behillil; Sylvie Van der Werf; Vincent Enouf
EPI_ISL_3105045, EPI_ISL_3105048, EPI_ISL_3105049	Overlake Hospital	Seattle Flu Study	Amanda Adler; Barry R. Lutz; Benjamin Pelle; Brian Hiatt; Caitlin R. Wolf; Chris D. Frazar; Deborah A. Nickerson; Elisabeth Brandstetter; Erica Ryke; Geoff Melly; Helen Y. Chu; Janet A. Englund; Jay Shendure; Jover Lee; Kairsten Fay; Kirsten Lacombe; Lea M. Starita; Mark J. Rieder; Matthew Richardson; Matthew Thompson; Melissa Truong; Michael Boeckh; Michael Famulare; Misja Ilcisin; Peter D. Han; Philip Dykema; Romesh Gautom; Scott Lindquist; Thomas R. Sibley; Trevor Bedford
EPI_ISL_2903092	Oxford Viromics, NDM, University of Oxford; Oxford University Hospitals; Basingstoke and North Hampshire Hospital	COVID-19 Genomics UK (COG-UK) Consortium	Alex Mobbs; Amy Trebes; Anita Justice; Catrin Moore; Christophe Fraser; David Bonsall; David Buck; Emma Wise; George Macintyre; Jessica Lynch; John Todd; Mariateresa de Cesare; Matilde Mori; Monique Andersson; Nathan Moore; Nick Cortes; Robert Shaw; Stephen Kidd; Tanya Golubchik; Timothy Peto
EPI_ISL_2493913	POLICLINICA COVID 19 ITAPETININGA	Instituto Butantan	Antonio Jorge Martins; Claudia Renata dos Santos Barros; David Schlesinger; Debora Botequio Moretti; Dimas Tadeu Covas; Elaine Cristina Marqueze; Elaine Vieira Santos; Evandra Strazza Rodrigues; Heidge Fukumasu; Jayme Augusto de Souza-Neto; José Salvatore Leister Patané; Luiz Alcantara; Luiz Lehmann Coutinho; Maria Carolina Elias; Maurício Lacerda Nogueira; Rafael dos Santos Bezerra; Raul Machado Neto; Rejane Maria Tommasini Grotto; Ricardo Haddad; Sandra Coccuzzo Sampaio Vessoni; Simone Kashima; Svetoslav Nanev Slavov; Vincent Louis Viala
EPI_ISL_3536291	POLO BASE DO TRILHO	Analytical Competence Molecular Epidemiology Lab/ACME, Oswaldo Cruz Foundation, Ceara (FIOCRUZ CE)	Cleber Furtado Aksenen; Fabio Miyajima; Fernando Braga Stehling; Francisco Eder de Moura Lopes; Jamille Maria Mendes Bezerra; Joaquim César do Nascimento Sousa Junior; Pedro Miguel Carneiro Jeronimo; Suzana Porto Almeida e Lucas Delerino; Thais Ferreira de Oliveira; Thais de Oliveira Costa; Ticiane Cavalcante de Souza; Veridiana Pessoa Miyajima
EPI_ISL_2493883, EPI_ISL_2493896, EPI_ISL_2493902, EPI_ISL_2493904, EPI_ISL_2493911, EPI_ISL_2493915, EPI_ISL_2493932, EPI_ISL_2493935, EPI_ISL_2493936, EPI_ISL_2494325, EPI_ISL_2494326			
see above	PRONTO ATENDIMENTO DE VARGEM GRANDE PAULISTA	Instituto Butantan	Antonio Jorge Martins; Claudia Renata dos Santos Barros; David Schlesinger; Debora Botequio Moretti; Dimas Tadeu Covas; Elaine Cristina Marqueze; Elaine Vieira Santos; Evandra Strazza Rodrigues; Heidge Fukumasu; Jayme Augusto de Souza-Neto; José Salvatore Leister Patané; Luiz Alcantara; Luiz Lehmann Coutinho; Maria Carolina Elias; Maurício Lacerda Nogueira; Rafael dos Santos Bezerra; Raul Machado Neto; Rejane Maria Tommasini Grotto; Ricardo Haddad; Sandra Coccuzzo Sampaio Vessoni; Simone Kashima; Svetoslav Nanev Slavov; Vincent Louis Viala
EPI_ISL_2493846	PS E AMBULATORIO ENGENHEIRO CARDOSO	Instituto Butantan	Antonio Jorge Martins; Claudia Renata dos Santos Barros; David Schlesinger; Debora Botequio Moretti; Dimas Tadeu Covas; Elaine Cristina Marqueze; Elaine Vieira Santos; Evandra Strazza Rodrigues; Heidge Fukumasu; Jayme Augusto de Souza-Neto; José Salvatore Leister Patané; Luiz Alcantara; Luiz Lehmann Coutinho; Maria Carolina Elias; Maurício Lacerda Nogueira; Rafael dos Santos Bezerra; Raul Machado Neto; Rejane Maria Tommasini Grotto; Ricardo Haddad; Sandra Coccuzzo Sampaio Vessoni; Simone Kashima; Svetoslav Nanev Slavov; Vincent Louis Viala
EPI_ISL_2493900	PSF DR ANTONIO PIRES DE ALMEIDA PORTO FELIZ	Instituto Butantan	Antonio Jorge Martins; Claudia Renata dos Santos Barros; David Schlesinger; Debora Botequio Moretti; Dimas Tadeu Covas; Elaine Cristina Marqueze; Elaine Vieira Santos; Evandra Strazza Rodrigues; Heidge Fukumasu; Jayme Augusto de Souza-Neto; José Salvatore Leister Patané; Luiz Alcantara; Luiz Lehmann Coutinho; Maria Carolina Elias; Maurício Lacerda Nogueira; Rafael dos Santos Bezerra; Raul Machado Neto; Rejane Maria Tommasini Grotto; Ricardo Haddad; Sandra Coccuzzo Sampaio Vessoni; Simone Kashima; Svetoslav Nanev Slavov; Vincent Louis Viala
EPI_ISL_2349631, EPI_ISL_2349675, EPI_ISL_2446041, EPI_ISL_2446051, EPI_ISL_2446088, EPI_ISL_2446091, EPI_ISL_2446094, EPI_ISL_2631339, EPI_ISL_2631340, EPI_ISL_2631349, EPI_ISL_2631350, EPI_ISL_2631354, EPI_ISL_2631359, EPI_ISL_2631360, EPI_ISL_2631366, EPI_ISL_2631386, EPI_ISL_2631397, EPI_ISL_2631407, EPI_ISL_2634916, EPI_ISL_2634943, EPI_ISL_2827487, EPI_ISL_2827488, EPI_ISL_2827489, EPI_ISL_2827500, EPI_ISL_2923915, EPI_ISL_2923945, EPI_ISL_3010646, EPI_ISL_3010649, EPI_ISL_3010653, EPI_ISL_3010670, EPI_ISL_3010678, EPI_ISL_3010754, EPI_ISL_3010757			
see above	Pandemic Response Lab - NYC	Pandemic Response Lab, R&D	Cybill del Castillo; Dylan Law; Haiping Hao; Henry Lee; Isabel Fernandez Escapa; Jon Laurent; Katharine Nelson; Melissa Hopkins; Michael Hammerling; Pradeep Bugga; Shinyoung Clair Kang; Sol Rey; William Ward
EPI_ISL_2832117, EPI_ISL_2832124, EPI_ISL_2832127, EPI_ISL_2897033, EPI_ISL_2897056	Parc Taulí Hospital Universitari	Can Ruti SARS-CoV-2 Sequencing Hub (HUGTiP/IrsiCaixa/IGTP)	Alba Sánchez; Alexia París; Anna Not; Antoni E Bordoy; Bonaventura Clotet; Cristina Casañ; David Panisello; Francesc Catala-Moll; Gemma Clara; Ignacio Blanco; Laia Soler; Lauro Sumoy; Marc Noguera-Julian; Maria Casadellà; Mariona Parera; Mercedes Guerrero; Montserrat Giménez; Pere-Joan Cardona; Pilar Armengol; Roger Paredes; Verónica Saludes; and Elisa Martró on behalf of the Can Ruti SARS-CoV-2 Sequencing Hub
EPI_ISL_3050138, EPI_ISL_3050162, EPI_ISL_3398772	Pathology, UAB	Pathology, UAB	E.J.; Hendrickson; Hernandez, J.; Leal; Lefkowitz; Moates, D.; R.C.; S.M.
EPI_ISL_3127929	Philadelphia Department of Public Health	Centers for Disease Control and Prevention Division of Viral Diseases, Pathogen Discovery	Alex Burgin; Ben L. Rambo-Martin; Clinton R. Paden; Dakota Howard; Dave Wentworth; Dhwani Batra; Jasmine Padilla; Justin Lee; Krista Queen; Kristen Knipe; Kristine Lacek; Mark Burroughs; Matthew Schmerer; Meghan Bentz; Mili Sheth; Peter Cook; Sam Shepard; Sarah Nobles; Suxiang Tong; Vivien Dugan; Yvette Unoarumhi
EPI_ISL_2861583	Platform BIS UZA/UAntwerpen	Labo Klinische Biologie, UZA	Basil Britto Xavier; Christine Lammens; Herman Goossens; Ines Verbesselt; Jasmine Coppens; Kathleen Holemans; Marie Le Mercier; Veerle Matheeussen
EPI_ISL_3120103	Policlinico San Donato	Laboratory of Clinical Microbiology, Virology and Bioemergencies, ASST Fatebenefratelli Sacco - Sacco Hospital	Valeria Micheli
EPI_ISL_2925488	Policlinico Tor Vergata	National Institute for Infectious Diseases (INMI) L. Spallanzani I.R.C.C.S	A Di Caro; B Bartolini; CEM Gruber; E Giombini; F Messina; F Santini; G Bonfiglio; M Rueca; MR Capobianchi; O Butera
EPI_ISL_3276769	Providence Saint John's Health Center	Los Angeles County Public Health Laboratories	P. Hemarajata et al.
EPI_ISL_2699447, EPI_ISL_2709231	Public Health Laboratory, Minnesota Department of Health	University of Minnesota Genomics Center	Corbin Dirkx; Daryl M. Gohl; Jaquelyn Kuriger-Laber; John Garbe; and Sean Wang
EPI_ISL_3132189	Puerto Rico Department of Health	Centers for Disease Control and Prevention, Dengue Branch	Betzabel Flores; Gabriela Paz-Bailey; Gilberto A. Santiago; Glenda Gonzalez; Jorge L. Munoz-Jordan; Keyla Charriez
EPI_ISL_2615917, EPI_ISL_2615920	QLabs	WVU and Marshall University Combined Genomics Core Facilities	James Denvir; Peter Perrotta; Peter Stoilov; Ryan Percifield; Wesley Kimble
EPI_ISL_2451428, EPI_ISL_2451463, EPI_ISL_2451473, EPI_ISL_2451509, EPI_ISL_2451518, EPI_ISL_2451524, EPI_ISL_2451538, EPI_ISL_2451549, EPI_ISL_2451555, EPI_ISL_2451559, EPI_ISL_2451565, EPI_ISL_2451566, EPI_ISL_2463008, EPI_ISL_2463013, EPI_ISL_2463014, EPI_ISL_2463015, EPI_ISL_2463017, EPI_ISL_2488822, EPI_ISL_2488823, EPI_ISL_2488824, EPI_ISL_2488825, EPI_ISL_2488827, EPI_ISL_2488829, EPI_ISL_2488830, EPI_ISL_2488831, EPI_ISL_2488832, EPI_ISL_2488833, EPI_ISL_2488834, EPI_ISL_2488835, EPI_ISL_2488837, EPI_ISL_2488840, EPI_ISL_2488841, EPI_ISL_2488844, EPI_ISL_2488850, EPI_ISL_2488851, EPI_ISL_2488853, EPI_ISL_2488854, EPI_ISL_2488855, EPI_ISL_2488856, EPI_ISL_2488857, EPI_ISL_2488860, EPI_ISL_2534519, EPI_ISL_2534521, EPI_ISL_2534522, EPI_ISL_2534524, EPI_ISL_2534525, EPI_ISL_2534526, EPI_ISL_2534527, EPI_ISL_2534528, EPI_ISL_2534529, EPI_ISL_2534530, EPI_ISL_2534531, EPI_ISL_2534532, EPI_ISL_2534553, EPI_ISL_2534554, EPI_ISL_2534555, EPI_ISL_2534556, EPI_ISL_2534557, EPI_ISL_2534558, EPI_ISL_2534560, EPI_ISL_2534565, EPI_ISL_2649948, EPI_ISL_2649950, EPI_ISL_2649951, EPI_ISL_2649952, EPI_ISL_2649953, EPI_ISL_2649955, EPI_ISL_2649956, EPI_ISL_2649957, EPI_ISL_2649958, EPI_ISL_2649959, EPI_ISL_2649960, EPI_ISL_2649962, EPI_ISL_2649963, EPI_ISL_2649964, EPI_ISL_2649966, EPI_ISL_2649967, EPI_ISL_2649968, EPI_ISL_2649969, EPI_ISL_2649979, EPI_ISL_2649980, EPI_ISL_2649981, EPI_ISL_2649982, EPI_ISL_2649983, EPI_ISL_2649984, EPI_ISL_2649987, EPI_ISL_2686360, EPI_ISL_2686361, EPI_ISL_2686362, EPI_ISL_2686364, EPI_ISL_2686365, EPI_ISL_2686366, EPI_ISL_2686367, EPI_ISL_2686370, EPI_ISL_2686373, EPI_ISL_2686374, EPI_ISL_2686375, EPI_ISL_2686376, EPI_ISL_2686377, EPI_ISL_2686380, EPI_ISL_2686381, EPI_ISL_2686386, EPI_ISL_2686387, EPI_ISL_2686388, EPI_ISL_2872074, EPI_ISL_2876105, EPI_ISL_2876106, EPI_ISL_2876107, EPI_ISL_2876109, EPI_ISL_2876110, EPI_ISL_2876113, EPI_ISL_2876114, EPI_ISL_2876115, EPI_ISL_2876116, EPI_ISL_2876119, EPI_ISL_2876120, EPI_ISL_2876121, EPI_ISL_2876122, EPI_ISL_2876123, EPI_ISL_2876124, EPI_ISL_2876125, EPI_ISL_2876126, EPI_ISL_2876127, EPI_ISL_2876128, EPI_ISL_2876129, EPI_ISL_2876132, EPI_ISL_2876134, EPI_ISL_2876135, EPI_ISL_2876137, EPI_ISL_2876142, EPI_ISL_2876145, EPI_ISL_2876146, EPI_ISL_2876148, EPI_ISL_2876149, EPI_ISL_2876153, EPI_ISL_2876154, EPI_ISL_2876155, EPI_ISL_2876160, EPI_ISL_2876162, EPI_ISL_2876163, EPI_ISL_2876166, EPI_ISL_2876167, EPI_ISL_2876616, EPI_ISL_2876617, EPI_ISL_2876619, EPI_ISL_2876620, EPI_ISL_2876621, EPI_ISL_2876624, EPI_ISL_2876626, EPI_ISL_2876628, EPI_ISL_2876629, EPI_ISL_2876630, EPI_ISL_2876631, EPI_ISL_2876632, EPI_ISL_2876633, EPI_ISL_2876636, EPI_ISL_2876638, EPI_ISL_2876639, EPI_ISL_2876640, EPI_ISL_2876644, EPI_ISL_2876645, EPI_ISL_2876646, EPI_ISL_2876647, EPI_ISL_2876649, EPI_ISL_2876650, EPI_ISL_2876651, EPI_ISL_2876652, EPI_ISL_2876653, EPI_ISL_2876654, EPI_ISL_2876656, EPI_ISL_2876660, EPI_ISL_2876661, EPI_ISL_2876662, EPI_ISL_2876663, EPI_ISL_2876665, EPI_ISL_2876666, EPI_ISL_2876667, EPI_ISL_2876668, EPI_ISL_2876672, EPI_ISL_2876673, EPI_ISL_2876675, EPI_ISL_2876676, EPI_ISL_2876677, EPI_ISL_2876734, EPI_ISL_2876735, EPI_ISL_2876737, EPI_ISL_2876741, EPI_ISL_2876742, EPI_ISL_2876743, EPI_ISL_2876744, EPI_ISL_2876745, EPI_ISL_2876746, EPI_ISL_2876748, EPI_ISL_2876749, EPI_ISL_2876750, EPI_ISL_2876752, EPI_ISL_2876753, EPI_ISL_2876755, EPI_ISL_2876756, EPI_ISL_2876757, EPI_ISL_2876758, EPI_ISL_2876760, EPI_ISL_2876762, EPI_ISL_2876763, EPI_ISL_2876764, EPI_ISL_2876765, EPI_ISL_2876766, EPI_ISL_2876768, EPI_ISL_2876770, EPI_ISL_2876771, EPI_ISL_2876772, EPI_ISL_2876774, EPI_ISL_2876775, EPI_ISL_2876776, EPI_ISL_2876777, EPI_ISL_2876778, EPI_ISL_2876781, EPI_ISL_2876782, EPI_ISL_2876783, EPI_ISL_2876785, EPI_ISL_2876788, EPI_ISL_2876789, EPI_ISL_2876791, EPI_ISL_2876793, EPI_ISL_2876794, EPI_ISL_2876795, EPI_ISL_2876798, EPI_ISL_2876799, EPI_ISL_2876800, EPI_ISL_2876801, EPI_ISL_2876803, EPI_ISL_2876804, EPI_ISL_2876806, EPI_ISL_2876807, EPI_ISL_2876809, EPI_ISL_2876810, EPI_ISL_2876813, EPI_ISL_2876814, EPI_ISL_2876818, EPI_ISL_2876824, EPI_ISL_2876825, EPI_ISL_2876827, EPI_ISL_2876830, EPI_ISL_2876831, EPI_ISL_2876832, EPI_ISL_2876834, EPI_ISL_2876837, EPI_ISL_2876838, EPI_ISL_2876839, EPI_ISL_2876840, EPI_ISL_2876841, EPI_ISL_2876842, EPI_ISL_2876843, EPI_ISL_2876844, EPI_ISL_2876845, EPI_ISL_2876846, EPI_ISL_2876849, EPI_ISL_2876852, EPI_ISL_2876853, EPI_ISL_2876854, EPI_ISL_2876855, EPI_ISL_2876858, EPI_ISL_2876859, EPI_ISL_2876860, EPI_ISL_2876861, EPI_ISL_2876862, EPI_ISL_2876866, EPI_ISL_2876868, EPI_ISL_2876869, EPI_ISL_2876870, EPI_ISL_2876874, EPI_ISL_2876875, EPI_ISL_2876877, EPI_ISL_2876879, EPI_ISL_2876881, EPI_ISL_2876882, EPI_ISL_2876884, EPI_ISL_2876885, EPI_ISL_2876886, EPI_ISL_2876888, EPI_ISL_2876892, EPI_ISL_2876893, EPI_ISL_2876896, EPI_ISL_2876897, EPI_ISL_2876898, EPI_ISL_2876899, EPI_ISL_2876901, EPI_ISL_2876902, EPI_ISL_2876904, EPI_ISL_2876906, EPI_ISL_2876908, EPI_ISL_2876909, EPI_ISL_2876914, EPI_ISL_2876916, EPI_ISL_2876917, EPI_ISL_2876918, EPI_ISL_2876919, EPI_ISL_2876920, EPI_ISL_2876922, EPI_ISL_2876924, EPI_ISL_2876925, EPI_ISL_2876926, EPI_ISL_2876927, EPI_ISL_2876928, EPI_ISL_2876930, EPI_ISL_2876933, EPI_ISL_2876934, EPI_ISL_3085829, EPI_ISL_3085830, EPI_ISL_3085831, EPI_ISL_3085836, EPI_ISL_3085840, EPI_ISL_3085841, EPI_ISL_3085843, EPI_ISL_3085848, EPI_ISL_3085849, EPI_ISL_3085850, EPI_ISL_3085851, EPI_ISL_3085852, EPI_ISL_3085854, EPI_ISL_3085855, EPI_ISL_3085856, EPI_ISL_3085858, EPI_ISL_3085862, EPI_ISL_3085864, EPI_ISL_3085866, EPI_ISL_3303840, EPI_ISL_3303843, EPI_ISL_3303845, EPI_ISL_3303847, EPI_ISL_3303850, EPI_ISL_3303852, EPI_ISL_3303853, EPI_ISL_3303855, EPI_ISL_3303856, EPI_ISL_3303857, EPI_ISL_3303859, EPI_ISL_3303860, EPI_ISL_3303865, EPI_ISL_3303867, EPI_ISL_3303868, EPI_ISL_3303872, EPI_ISL_3303875, EPI_ISL_3303876, EPI_ISL_3303879, EPI_ISL_3303881, EPI_ISL_3303884, EPI_ISL_3303886, EPI_ISL_3303887, EPI_ISL_3303890, EPI_ISL_3303891, EPI_ISL_3303893, EPI_ISL_3303897, EPI_ISL_3303898, EPI_ISL_3303900, EPI_ISL_3303901, EPI_ISL_3303904, EPI_ISL_3303906, EPI_ISL_3303909, EPI_ISL_3303912, EPI_ISL_3303916, EPI_ISL_3303917, EPI_ISL_3303918, EPI_ISL_3304999, EPI_ISL_3305007, EPI_ISL_3305011, EPI_ISL_3305013, EPI_ISL_3305015, EPI_ISL_3305027, EPI_ISL_3305033, EPI_ISL_3305039, EPI_ISL_3305040, EPI_ISL_3305045, EPI_ISL_3305056, EPI_ISL_3305065, EPI_ISL_3305070, EPI_ISL_3305072, EPI_ISL_3305078, EPI_ISL_3305080, EPI_ISL_3305093, EPI_ISL_3305098, EPI_ISL_3305099, EPI_ISL_3305100, EPI_ISL_3305105, EPI_ISL_3305111, EPI_ISL_3305122, EPI_ISL_3305124, EPI_ISL_3305130, EPI_ISL_3305133, EPI_ISL_3305135, EPI_ISL_3305140, EPI_ISL_3305143, EPI_ISL_3305148, EPI_ISL_3305154, EPI_ISL_3305155, EPI_ISL_3305156, EPI_ISL_3305165, EPI_ISL_3305170, EPI_ISL_3305171, EPI_ISL_3305183			
see above	Quest Diagnostics Incorporated	Centers for Disease Control and	A. Gerasimova; A. Perez; Adrian Paskey; B. Anderson; Benjamin Rambo-Martin; Christopher Gulvick; Clinton R. Paden; Dakota Howard; Darlene Wagner; Dhwani Batra; Duncan MacCannell; F. Lacbawan; I. A. Shlyakhter; Jason Caravas; K.E. Livingston; Kara Moser; L.E. Bernstein; M. Hua; Matthew

		Prevention Division of Viral Diseases, Pathogen Discovery	Schmerer; P. Tanpaiboon; Peter W. Cook; R. M. Kagan; R. Owen; R. V. Rolando; S. H. Rosenthal; Scott Sammons; Shatavia Morrison; Y. Liu; Yvette Unoarumhi
EPI_ISL_2645357, EPI_ISL_2787984, EPI_ISL_2787987, EPI_ISL_2840374, EPI_ISL_3355373	RI State Health Laboratories	Centers for Disease Control and Prevention Division of Viral Diseases, Pathogen Discovery	Alex Burgin; Alison Laufer Halpin; Ben L. Rambo-Martin; Ben Rambo-Martin; Clinton Paden; Clinton R. Paden; Dakota Howard; Darlene Wagner; Dave Wentworth; Dhwani Batra; Jasmine Padilla; Justin Lee; Katie Dillon; Krista Queen; Kristen Knipe; Kristine Lacek; Mark Burroughs; Matthew Schmerer; Meghan Bentz; Mili Sheth; Peter Cook; Sam Shepard; Sarah Nobles; Shoshona Le; Suxiang Tong; Vivien Dugan; Yvette Unoarumhi
EPI_ISL_3091694	Respiratory Viruses Branch, Centers for Disease Control and Prevention	Respiratory Viruses Branch, Centers for Disease Control and Prevention	Anderson, B.; Batra, D.; Bernstein, L.; C.R.; Caravas, J.; Cook; Gerasimova, A.; Gulvick, C.; Howard, D.; Hua, M.; I.A.; Kagan; Lacbawan, F.; Liu, Y.; Livingston, K.; MacCannell, D.; Morrison, S.; Moser, K.; Owen, R.; P.W.; Paden; Paskey, A.; Perez, A.; R.M.; R.V.; Rambo-Martin, B.; Rolando; Rosenthal; S.H.; Sammons, S.; Schmerer, M.; Shlyakhter; Tanpaiboon, P.; Unoarumhi, Y.; Wagner, D.
EPI_ISL_3982730	Ribeirao Preto	Ribeirao Preto	Antonio Jorge Martins; Claudia Renata dos Santos Barros; David Schlesinger; Debora Botequio Moretti; Dimas Tadeu Covas; Elaine Cristina Marqueze; Elaine Vieira Santos; Evandra Strazza Rodrigues; Heidge Fukumasu; Jayme Augusto de Souza-Neto; José Salvatore Leister Patané; Luiz Alcantara; Luiz Lehmann Coutinho; Maria Carolina Elias; Maurício Lacerda Nogueira; Rafael dos Santos Bezerra; Raul Machado Neto; Rejane Maria Tommasini Grotto; Ricardo Haddad; Sandra Coccuzzo Sampaio Vessoni; Simone Kashima; Svetoslav Nanev Slavov; Vincent Louis Viala
EPI_ISL_3982693, EPI_ISL_3982697, EPI_ISL_3982701, EPI_ISL_3982711	Ribeirao Preto SP	Laboratorio de Biologia Molecular Hemocentro de Ribeirao Preto	Antonio Jorge Martins; Claudia Renata dos Santos Barros; David Schlesinger; Debora Botequio Moretti; Dimas Tadeu Covas; Elaine Cristina Marqueze; Elaine Vieira Santos; Evandra Strazza Rodrigues; Heidge Fukumasu; Jayme Augusto de Souza-Neto; José Salvatore Leister Patané; Luiz Alcantara; Luiz Lehmann Coutinho; Maria Carolina Elias; Maurício Lacerda Nogueira; Rafael dos Santos Bezerra; Raul Machado Neto; Rejane Maria Tommasini Grotto; Ricardo Haddad; Sandra Coccuzzo Sampaio Vessoni; Simone Kashima; Svetoslav Nanev Slavov; Vincent Louis Viala
EPI_ISL_2691942, EPI_ISL_2931090, EPI_ISL_2931092, EPI_ISL_2931093, EPI_ISL_2931094, EPI_ISL_2931100, EPI_ISL_2931102, EPI_ISL_3388714, EPI_ISL_3388715, EPI_ISL_3388808			
see above	Rush University Medical Center	RIPHL at Rush University Medical Center	Diane Springer; Edith Perez; Felix Araujo Perez; Joyce Houlihan; Kevin Kunstman; Laura Furtado; Marieta Hyde; Mary Hayden; Max Kolton; Stefan Green
EPI_ISL_2925707	S. Camillo	National Institute for Infectious Diseases (INMI) L. Spallanzani I.R.C.C.S	A Di Caro; B Bartolini; CEM Gruber; E Giombini; F Messina; F Santini; G Bonfiglio; M Rueca; MR Capobianchi; O Butera
EPI_ISL_2840624, EPI_ISL_2840626, EPI_ISL_2840628	S.C. Laboratorio Analisi, ASL 3 Liguria	U.O. Igiene, Ospedale Policlinico San Martino	Bruzzone Bianca; Caligiuri Patrizia; De Pace Vanessa; Domnich Alexander; Icardi Giancarlo on behalf of SARS-CoV-2 ITALIAN RESEARCH ENTERPRISE-(SCIRE) Collaborative Group; Orsi Andrea; Ricucci Valentina; Spitaleri Antonino
EPI_ISL_2493909	SANTA CASA SALTO DE PIRAPORA	Instituto Butantan	Antonio Jorge Martins; Claudia Renata dos Santos Barros; David Schlesinger; Debora Botequio Moretti; Dimas Tadeu Covas; Elaine Cristina Marqueze; Elaine Vieira Santos; Evandra Strazza Rodrigues; Heidge Fukumasu; Jayme Augusto de Souza-Neto; José Salvatore Leister Patané; Luiz Alcantara; Luiz Lehmann Coutinho; Maria Carolina Elias; Maurício Lacerda Nogueira; Rafael dos Santos Bezerra; Raul Machado Neto; Rejane Maria Tommasini Grotto; Ricardo Haddad; Sandra Coccuzzo Sampaio Vessoni; Simone Kashima; Svetoslav Nanev Slavov; Vincent Louis Viala
EPI_ISL_2494169, EPI_ISL_2494177, EPI_ISL_2494189, EPI_ISL_2494196	SANTA CASA SANTA RITA DO PASSA QUATRO	Instituto Butantan	Antonio Jorge Martins; Claudia Renata dos Santos Barros; David Schlesinger; Debora Botequio Moretti; Dimas Tadeu Covas; Elaine Cristina Marqueze; Elaine Vieira Santos; Evandra Strazza Rodrigues; Heidge Fukumasu; Jayme Augusto de Souza-Neto; José Salvatore Leister Patané; Luiz Alcantara; Luiz Lehmann Coutinho; Maria Carolina Elias; Maurício Lacerda Nogueira; Rafael dos Santos Bezerra; Raul Machado Neto; Rejane Maria Tommasini Grotto; Ricardo Haddad; Sandra Coccuzzo Sampaio Vessoni; Simone Kashima; Svetoslav Nanev Slavov; Vincent Louis Viala
EPI_ISL_2957883, EPI_ISL_2958106, EPI_ISL_2986110, EPI_ISL_2986642, EPI_ISL_2986643, EPI_ISL_2986644, EPI_ISL_3587525			
see above	SARS-CoV-2 Sequencing Castilla y Leon-Spain Consortium	SARS-CoV-2 Sequencing Castilla y Leon-Spain Consortium	Antonio Orduña-Domingo; Carlos Fuster Foz; Carmen Aldea-Mansilla; Carmen Gimeno Crespo; David Abad; Gabriel March Rosello; Gabriel March Rossello; Gregoria Megías Lobón; Jose María Eiros Bouza; Laura Sánchez de Prada; M. Isabel Fernandez-Natal; Marta Dominguez-Gil; Marta Hernandez; María Antonia García Castro; Mª Fe Brezmes-Valdivieso; Noelia Arenal Andrés; Silvia Rojo; Sonsoles Garcinuño Pérez
EPI_ISL_2621771, EPI_ISL_2621772, EPI_ISL_2828304, EPI_ISL_2930565	SARS-CoV-2 testing team, National Institute of Infectious Diseases	Pathogen Genomics Center, National Institute of Infectious Diseases	Hazuka Y Furihata; Hiromizu Takahashi; Kentaro Itokawa; Makoto Kuroda; Masanori Hashino; Masumichi Saito; Naomi Nojiri; Nozomu Hanaoka; Rina Tanaka; Sana Uchikoba; Tsuguto Fujimoto; Tsuyoshi Sekizuka
EPI_ISL_2828387, EPI_ISL_2828388	SC (UCO) Igiene e Sanità Pubblica, ASUGI, Trieste	ARGO Laboratorio Genomica ed Epigenomica	Barbone F; Breda C; Busetti M; D'Agaro P; Dal Monego S; Degasperi M; Fontana F; Licastro D; Marcello A; Piscianz E; Segat L
EPI_ISL_2828404	SC (UCO) Igiene e Sanità Pubblica, ASUGI, Trieste and SC Microbiologia e Virologia, ASFO, Pordenone	ARGO Laboratorio Genomica ed Epigenomica	Barbone F; Breda C; D'Agaro P; Dal Monego S; De Rosa R; Degasperi M; Licastro D; Marcello A; Segat L
EPI_ISL_2494100	SECAO CENTRO DE DIAGNOSTICO SECEDI	Instituto Butantan	Antonio Jorge Martins; Claudia Renata dos Santos Barros; David Schlesinger; Debora Botequio Moretti; Dimas Tadeu Covas; Elaine Cristina Marqueze; Elaine Vieira Santos; Evandra Strazza Rodrigues; Heidge Fukumasu; Jayme Augusto de Souza-Neto; José Salvatore Leister Patané; Luiz Alcantara; Luiz Lehmann Coutinho; Maria Carolina Elias; Maurício Lacerda Nogueira; Rafael dos Santos Bezerra; Raul Machado Neto; Rejane Maria Tommasini Grotto; Ricardo Haddad; Sandra Coccuzzo Sampaio Vessoni; Simone Kashima; Svetoslav Nanev Slavov; Vincent Louis Viala
EPI_ISL_2494161, EPI_ISL_2494170, EPI_ISL_2494178	SECRETARIA DE SAUDE DE SANTA GERTRUDES	Instituto Butantan	Antonio Jorge Martins; Claudia Renata dos Santos Barros; David Schlesinger; Debora Botequio Moretti; Dimas Tadeu Covas; Elaine Cristina Marqueze; Elaine Vieira Santos; Evandra Strazza Rodrigues; Heidge Fukumasu; Jayme Augusto de Souza-Neto; José Salvatore Leister Patané; Luiz Alcantara; Luiz Lehmann Coutinho; Maria Carolina Elias; Maurício Lacerda Nogueira; Rafael dos Santos Bezerra; Raul Machado Neto; Rejane Maria Tommasini Grotto; Ricardo Haddad; Sandra Coccuzzo Sampaio Vessoni; Simone Kashima; Svetoslav Nanev Slavov; Vincent Louis Viala
EPI_ISL_2494167, EPI_ISL_2494190	SECRETARIA DE SAUDE DE SAO PEDRO	Instituto Butantan	Antonio Jorge Martins; Claudia Renata dos Santos Barros; David Schlesinger; Debora Botequio Moretti; Dimas Tadeu Covas; Elaine Cristina Marqueze; Elaine Vieira Santos; Evandra Strazza Rodrigues; Heidge Fukumasu; Jayme Augusto de Souza-Neto; José Salvatore Leister Patané; Luiz Alcantara; Luiz Lehmann Coutinho; Maria Carolina Elias; Maurício Lacerda Nogueira; Rafael dos Santos Bezerra; Raul Machado Neto; Rejane Maria Tommasini Grotto; Ricardo Haddad; Sandra Coccuzzo Sampaio Vessoni; Simone Kashima; Svetoslav Nanev Slavov; Vincent Louis Viala
EPI_ISL_2493903	SECRETARIA MUNICIPAL DA SAUDE DE ITARARE	Instituto Butantan	Antonio Jorge Martins; Claudia Renata dos Santos Barros; David Schlesinger; Debora Botequio Moretti; Dimas Tadeu Covas; Elaine Cristina Marqueze; Elaine Vieira Santos; Evandra Strazza Rodrigues; Heidge Fukumasu; Jayme Augusto de Souza-Neto; José Salvatore Leister Patané; Luiz Alcantara; Luiz Lehmann Coutinho; Maria Carolina Elias; Maurício Lacerda Nogueira; Rafael dos Santos Bezerra; Raul Machado Neto; Rejane Maria Tommasini Grotto; Ricardo Haddad; Sandra Coccuzzo Sampaio Vessoni; Simone Kashima; Svetoslav Nanev Slavov; Vincent Louis Viala
EPI_ISL_2493275, EPI_ISL_2493276, EPI_ISL_2494110	SECRETARIA MUNICIPAL DE SAUDE	Instituto Butantan	Antonio Jorge Martins; Claudia Renata dos Santos Barros; David Schlesinger; Debora Botequio Moretti; Dimas Tadeu Covas; Elaine Cristina Marqueze; Elaine Vieira Santos; Evandra Strazza Rodrigues; Heidge Fukumasu; Jayme Augusto de Souza-Neto; José Salvatore Leister Patané; Luiz Alcantara; Luiz Lehmann Coutinho; Maria Carolina Elias; Maurício Lacerda Nogueira; Rafael dos Santos Bezerra; Raul Machado Neto; Rejane Maria Tommasini Grotto; Ricardo Haddad; Sandra Coccuzzo Sampaio Vessoni; Simone Kashima; Svetoslav Nanev Slavov; Vincent Louis Viala
EPI_ISL_2494185	SECRETARIA MUNICIPAL DE SAUDE DE CORDEIROPOLIS	Instituto Butantan	Antonio Jorge Martins; Claudia Renata dos Santos Barros; David Schlesinger; Debora Botequio Moretti; Dimas Tadeu Covas; Elaine Cristina Marqueze; Elaine Vieira Santos; Evandra Strazza Rodrigues; Heidge Fukumasu; Jayme Augusto de Souza-Neto; José Salvatore Leister Patané; Luiz Alcantara; Luiz Lehmann Coutinho; Maria Carolina Elias; Maurício Lacerda Nogueira; Rafael dos Santos Bezerra; Raul Machado Neto; Rejane Maria Tommasini Grotto; Ricardo Haddad; Sandra Coccuzzo Sampaio Vessoni; Simone Kashima; Svetoslav Nanev Slavov; Vincent Louis Viala
EPI_ISL_2493825	SECRETARIA MUNICIPAL DE SAUDE DE MORUNGABA	Instituto Butantan	Antonio Jorge Martins; Claudia Renata dos Santos Barros; David Schlesinger; Debora Botequio Moretti; Dimas Tadeu Covas; Elaine Cristina Marqueze; Elaine Vieira Santos; Evandra Strazza Rodrigues; Heidge Fukumasu; Jayme Augusto de Souza-Neto; José Salvatore Leister Patané; Luiz Alcantara; Luiz Lehmann Coutinho; Maria Carolina Elias; Maurício Lacerda Nogueira; Rafael dos Santos Bezerra; Raul Machado Neto; Rejane Maria Tommasini Grotto; Ricardo Haddad; Sandra Coccuzzo Sampaio Vessoni; Simone Kashima; Svetoslav Nanev Slavov; Vincent Louis Viala
EPI_ISL_2493802, EPI_ISL_2494306, EPI_ISL_2494307, EPI_ISL_2494308	SECRETARIA MUNICIPAL DE SAUDE DE PEDERNEIRAS	Instituto Butantan	Antonio Jorge Martins; Claudia Renata dos Santos Barros; David Schlesinger; Debora Botequio Moretti; Dimas Tadeu Covas; Elaine Cristina Marqueze; Elaine Vieira Santos; Evandra Strazza Rodrigues; Heidge Fukumasu; Jayme Augusto de Souza-Neto; José Salvatore Leister Patané; Luiz Alcantara; Luiz Lehmann Coutinho; Maria Carolina Elias; Maurício Lacerda Nogueira; Rafael dos Santos Bezerra; Raul Machado Neto; Rejane Maria Tommasini Grotto; Ricardo Haddad; Sandra Coccuzzo Sampaio Vessoni; Simone Kashima; Svetoslav Nanev Slavov; Vincent Louis Viala
EPI_ISL_2462329, EPI_ISL_2494146	SECRETARIA MUNICIPAL DE SAUDE DE PRES BERNARDES SP	Instituto Butantan	Antonio Jorge Martins; Claudia Renata dos Santos Barros; David Schlesinger; Debora Botequio Moretti; Dimas Tadeu Covas; Elaine Cristina Marqueze; Elaine Vieira Santos; Evandra Strazza Rodrigues; Heidge Fukumasu; Jayme Augusto de Souza-Neto; José Salvatore Leister Patané; Luiz Alcantara; Luiz Lehmann Coutinho; Maria Carolina Elias; Maurício Lacerda Nogueira; Rafael dos Santos Bezerra; Raul Machado Neto; Rejane Maria Tommasini Grotto; Ricardo Haddad; Sandra Coccuzzo Sampaio Vessoni; Simone Kashima; Svetoslav Nanev Slavov; Vincent Louis Viala
EPI_ISL_2801350, EPI_ISL_2801359, EPI_ISL_2801366, EPI_ISL_3102500, EPI_ISL_3536383, EPI_ISL_3536384, EPI_ISL_3536386, EPI_ISL_3536387, EPI_ISL_3536388, EPI_ISL_3536389, EPI_ISL_3536390, EPI_ISL_3536391, EPI_ISL_3912241, EPI_ISL_3912244, EPI_ISL_3912245, EPI_ISL_3912387			
see above	SECRETARIA MUNICIPAL DE SAUDE DE TIANGUA	Analytical Competence Molecular Epidemiology Lab/ACME, Oswaldo Cruz Foundation, Ceara (FIOCRUZ CE)	Cleber Furtado Aksenen; Cleber Furtado Aksenen e Suzana Porto Almeida; Fabio Miyajima; Fernando Braga Stehling; Francisco Eder de Moura Lopes; Jamille Mendes Bezerra; Joaquim Cesar do Nascimento Sousa Junior; Joaquim César do Nascimento Sousa Junior; Pedro Miguel Carneiro Jeronimo; Suzana Porto Almeida & Lucas Delerino on behalf of COVID-19 FIOCRUZ Genomic Network; Suzana Porto Almeida e Lucas Delerino; Thais Ferreira de Oliveira; Thais de Oliveira Costa; Ticiane Cavalcante de Souza; Veridiana Pessoa Miyajima
EPI_ISL_2895352, EPI_ISL_2895353, EPI_ISL_2895354	SEEBMO	Instituto Nacional de Saude (INSA)	Borges et al
EPI_ISL_2795758	SELAS MEDILYS	Department of Virology, Henri Mondor University Hospital, Assistance Publique Hôpitaux de Paris, Université Paris-Est Créteil, INSERM U955	Alexandre Soulier; Christophe Rodriguez; Elisabeth Trawinski; Guillaume Gricourt; Jean-Michel Pawlotsky; Melissa N'Debi; Slim Fourati; Vanessa Demontant
EPI_ISL_2493832, EPI_ISL_2493833, EPI_ISL_2493837, EPI_ISL_2493838, EPI_ISL_2493839, EPI_ISL_2493840, EPI_ISL_2493842, EPI_ISL_2493890, EPI_ISL_2493892, EPI_ISL_2493894, EPI_ISL_2494294, EPI_ISL_2494322			
see above	SERV DE VIG SANITARIA EPIDEMIO E CTRL DE ZOONOZES GUARUJA	Instituto Butantan	Antonio Jorge Martins; Claudia Renata dos Santos Barros; David Schlesinger; Debora Botequio Moretti; Dimas Tadeu Covas; Elaine Cristina Marqueze; Elaine Vieira Santos; Evandra Strazza Rodrigues; Heidge Fukumasu; Jayme Augusto de Souza-Neto; José Salvatore Leister Patané; Luiz Alcantara; Luiz Lehmann Coutinho; Maria Carolina Elias; Maurício Lacerda Nogueira; Rafael dos Santos Bezerra; Raul Machado Neto; Rejane Maria Tommasini Grotto; Ricardo Haddad; Sandra Coccuzzo Sampaio Vessoni; Simone Kashima; Svetoslav Nanev Slavov; Vincent Louis Viala
EPI_ISL_2895384, EPI_ISL_2895389	SESARAM	Instituto Nacional de Saude (INSA)	Borges et al
EPI_ISL_2462317, EPI_ISL_2493872, EPI_ISL_2493874, EPI_ISL_2493942	SMS ARUJA	Instituto Butantan	Antonio Jorge Martins; Claudia Renata dos Santos Barros; David Schlesinger; Debora Botequio Moretti; Dimas Tadeu Covas; Elaine Cristina Marqueze; Elaine Vieira Santos; Evandra Strazza Rodrigues; Heidge Fukumasu; Jayme Augusto de Souza-Neto; José Salvatore Leister Patané; Luiz Alcantara; Luiz Lehmann Coutinho; Maria Carolina Elias; Maurício Lacerda Nogueira; Rafael dos Santos Bezerra; Raul Machado Neto; Rejane Maria Tommasini Grotto; Ricardo Haddad; Sandra Coccuzzo Sampaio Vessoni; Simone Kashima; Svetoslav Nanev Slavov; Vincent Louis Viala
EPI_ISL_3536156, EPI_ISL_3536157, EPI_ISL_3536158, EPI_ISL_3536159, EPI_ISL_3536161	SOCIEDADE HOSPITALAR PE DIONISIO	Analytical Competence Molecular Epidemiology Lab/ACME, Oswaldo Cruz Foundation, Ceara (FIOCRUZ CE)	Cleber Furtado Aksenen; Fabio Miyajima; Fernando Braga Stehling; Francisco Eder de Moura Lopes; Jamille Mendes Bezerra; Joaquim César do Nascimento Sousa Junior; Pedro Miguel Carneiro Jeronimo; Suzana Porto Almeida e Lucas Delerino; Thais Ferreira de Oliveira; Thais de Oliveira Costa; Ticiane Cavalcante de Souza; Veridiana Pessoa Miyajima
EPI_ISL_2627152, EPI_ISL_2868528	STONY BROOK UNIVERSITY HOSPITAL	Wadsworth Center, New York State Department of Health	Alexis Russell; Catharine Prussing; Daryl M. Lamson; Erasmus Schneider; Erica Lasek-Nesselquist; John Kelly; Jonathan Plitnick; Kirsten St. George; Matthew Shudt; Melissa A Leisner; Navjot Singh

EPI_ISL_2868260, EPI_ISL_2868261	SUNY UPSTATE MEDICAL UNIVERSITY	Wadsworth Center, New York State Department of Health	Alexis Russell; Catharine Prussing; Daryl M. Lamson; Erasmus Schneider; Erica Lasek-Nesselquist; John Kelly; Jonathan Plitnick; Kirsten St. George; Matthew Shudt; Melissa A Leisner; Navjot Singh
EPI_ISL_2933358	SYNLAB GASCOGNE	CNR Virus des Infections Respiratoires - France SUD	Antonin Bal; Bruno Lina; Gregory Destras; Gwendolyne Burfin; Hadrien Regue; Laurence Josset; Martine Valette; Quentin Semanas
EPI_ISL_3044006	SYNLAB Labor München Zentrum LMZ	Robert Koch Institute	
EPI_ISL_2472365	SYNLAB MVZ Berlin	Robert Koch Institute	
EPI_ISL_2389554, EPI_ISL_2389772, EPI_ISL_2633059, EPI_ISL_2635424, EPI_ISL_2844917	SYNLAB MVZ Heidelberg	Robert Koch Institute	
EPI_ISL_2635200, EPI_ISL_2759897	SYNLAB MVZ Leverkusen	Robert Koch Institute	
EPI_ISL_2469537, EPI_ISL_2633330	SYNLAB MVZ Trier	Robert Koch Institute	
EPI_ISL_2469483, EPI_ISL_2472105	SYNLAB MVZ Weiden	Robert Koch Institute	
EPI_ISL_2603678, EPI_ISL_2603701, EPI_ISL_2603702, EPI_ISL_2603710, EPI_ISL_2603733, EPI_ISL_2603770, EPI_ISL_2603771, EPI_ISL_2617007, EPI_ISL_2617014, EPI_ISL_2692580, EPI_ISL_2692655, EPI_ISL_2692656, EPI_ISL_2692762, EPI_ISL_2692792, EPI_ISL_2692797, EPI_ISL_2810033, EPI_ISL_2810080, EPI_ISL_2810086, EPI_ISL_2810096, EPI_ISL_2810103, EPI_ISL_2810169, EPI_ISL_2810256, EPI_ISL_2810270, EPI_ISL_2894265, EPI_ISL_2894378, EPI_ISL_2894410, EPI_ISL_2978363, EPI_ISL_2978442, EPI_ISL_2978444, EPI_ISL_2978449, EPI_ISL_2978489, EPI_ISL_2978499, EPI_ISL_2978517, EPI_ISL_3067926, EPI_ISL_3067935, EPI_ISL_3067936, EPI_ISL_3067937, EPI_ISL_3721739			
see above	Salud Digna	Instituto Nacional de Medicina Genomica	Abraham Campos-Romero; Cedro-Tanda A; Escobar-Arrazola; Escobar-Arrazola MA; Gonzalez-Barrera D; Herrera-Montalvo LA.; Hidalgo-Miranda A; Luna-Ruiz Marco; M; Mendoza-Vargas A; Moreno-Camacho José Luis; Munguia-Garza P; Ramirez-Vega O; Rangel-DeLeon D; Reyes-Grajeda JP; Rodriguez-Gallegos Jorge; Yair Alfaro-Mora
EPI_ISL_3373977, EPI_ISL_3373979, EPI_ISL_3373982, EPI_ISL_3373994, EPI_ISL_3374014, EPI_ISL_3374016, EPI_ISL_3374019, EPI_ISL_3374020, EPI_ISL_3374023, EPI_ISL_3374024, EPI_ISL_3374025, EPI_ISL_3374026, EPI_ISL_3374030, EPI_ISL_3374031, EPI_ISL_3374036, EPI_ISL_3374038, EPI_ISL_3374039, EPI_ISL_3912490, EPI_ISL_3912512			
see above	Salud Digna, A.C	Andersen lab at Scripps Research	Abraham Garcia Gil; Jose Luis Moreno Camacho; Marco Antonio Luna Ruiz-Esparza; Miguel A. Fernandez Rojas; SEARCH Alliance with Abraham Campos Romero
EPI_ISL_3374049, EPI_ISL_3374052, EPI_ISL_3374055, EPI_ISL_3374057, EPI_ISL_3374060, EPI_ISL_3374068, EPI_ISL_3374069, EPI_ISL_3374071, EPI_ISL_3374113, EPI_ISL_3374114, EPI_ISL_3374121			
see above	San Diego County Public Health Laboratory	Andersen lab at Scripps Research	Brett Austin; Jovan Shephard; SEARCH Alliance San Diego with Ashleigh Murphy
EPI_ISL_2840862, EPI_ISL_2840896, EPI_ISL_2841534, EPI_ISL_2841536, EPI_ISL_2841614, EPI_ISL_2842703, EPI_ISL_2842704, EPI_ISL_2842705, EPI_ISL_2873837, EPI_ISL_3275048, EPI_ISL_3346596, EPI_ISL_3346597			
see above	San Diego County Public Health Laboratory	San Diego County Public Health Laboratory	Ashleigh Murphy Schafer; Brett Austin; San Diego County Public Health Laboratory
EPI_ISL_2650574, EPI_ISL_2691314, EPI_ISL_2934069, EPI_ISL_2967940	Santa Clara Valley Medical Center	Santa Clara County Public Health Laboratory	Santa Clara County Public Health Department
EPI_ISL_2627004	Saratoga Hospital Laboratory	Wadsworth Center, New York State Department of Health	Alexis Russell; Catharine Prussing; Daryl M. Lamson; Erasmus Schneider; Erica Lasek-Nesselquist; John Kelly; Jonathan Plitnick; Kirsten St. George; Matthew Shudt; Melissa A Leisner; Navjot Singh
EPI_ISL_2735928, EPI_ISL_2735931	Seattle Flu Study	Seattle Flu Study	Amanda Adler; Barry R. Lutz; Benjamin Pelle; Caitlin R. Wolf; Chris D. Frazar; Deborah A. Nickerson; Elisabeth Brandstetter; Erica Ryke; Helen Y. Chu; Janet A. Englund; Jay Shendure; Jeff Duchin; Jover Lee; Kairsten Fay; Karen Cowgill; Kirsten Lacombe; Lea M. Starita; Mark J. Rieder; Matthew Richardson; Matthew Thompson; Melissa Truong; Michael Boeckh; Michael Famulare; Misja Ilcisin; Peter D. Han; Stephanie Schrag; Thomas R. Sibley; Trevor Bedford
EPI_ISL_2680930	Servicio Diagnóstico Clínico	Laboratorio nacional-LANIIA-CIAD	; Alejandra García-Gasca; Alejandra Hernández-Terán; Alejandro Sánchez-Flores; Alfredo Herrera-Estrella; Alicia Ocaña-Mondragón; Andreu Comas-García; Angel Gustavo Salas-Lais; Antonio Loza Román; Bernardo Martínez-Miguel; Blanca Taboada; Brenda Irasema Maldonado-Meza; Bruno Gómez-Gil; Carla Ivón Herrera-Najera; Carlos F. Arias; Celia Boukadida; Clara Esperanza Santacruz-Tinoco; Concepción Grajales-Muñiz; Consorcio Mexicano de Vigilancia Genómica (CoViGen-Mex). Authors (in alphabetical order): Julio Elias Alvarado-Yaah; Cristóbal Cháidez-Quiróz; Célida Duque Molina; Célida Martínez- Rodríguez; Daniel Fregoso-Rueda; Daniel Lira Morales; Eduardo Becerril-Vargas; Fernando Fontove-Herrera; Fidencio Mejía-Nepomuceno; Francisco Pulido; Gloria Elena Espinosa-Ayala; Gloria María Molina-Salinas; Gloria Vazquez; Hector Esteban Paz-Juárez; Hector Montoya-Fuentes; Helen Haydee Fernanda Ramírez-Plascencia; Irvin González-López; Jean Pierre González; Jesús Hernández; Joel Armando Vázquez-Pérez.; Jorge Salas-Hernández; José Antonio Enciso-Moreno; José Arturo Martínez-Orozco; José Esteban Muñoz-Medina; José de Jesús Nuñez-Contreras; Juan Bautista Chale-Dzul; Julissa Enciso-Ibarra; Luis Alberto Ochoa-Carrera; Margarita Matías-Florentino; Mario Mújica-Sánchez; Marissa Perez-Garcia; María Guadalupe Santiago-Mauricio; María Guadalupe de Jesús Mireles-Rivera; Nelly Sélem-Mojica; Pavel Isa; Ricardo Ciria Merce; Ricardo Grande; Rosa María Gutiérrez Rios; Santiago Ávila-Ríos; Selene Zárate; Susana Lopez; Verónica Mata-Haro; Victor Eduardo García-Arias; Víctor Hugo Borja-Aburto
EPI_ISL_4082378, EPI_ISL_4082415, EPI_ISL_4082433, EPI_ISL_4082434, EPI_ISL_4082437, EPI_ISL_4082445, EPI_ISL_4082479, EPI_ISL_4082507, EPI_ISL_4082509, EPI_ISL_4082510, EPI_ISL_4082521, EPI_ISL_4082522, EPI_ISL_4082524, EPI_ISL_4082530, EPI_ISL_4082536, EPI_ISL_4082544, EPI_ISL_4082545, EPI_ISL_4082572, EPI_ISL_4082576, EPI_ISL_4082594, EPI_ISL_4082602, EPI_ISL_4082609, EPI_ISL_4082611, EPI_ISL_4082629, EPI_ISL_4082634, EPI_ISL_4082638, EPI_ISL_4082652, EPI_ISL_4082664, EPI_ISL_4082665, EPI_ISL_4082713, EPI_ISL_4082715, EPI_ISL_4082717, EPI_ISL_4082718, EPI_ISL_4082722, EPI_ISL_4082728, EPI_ISL_4082747, EPI_ISL_4082777, EPI_ISL_4082778, EPI_ISL_4082805, EPI_ISL_4082810, EPI_ISL_4082833, EPI_ISL_4082834, EPI_ISL_4082837, EPI_ISL_4082841, EPI_ISL_4082843, EPI_ISL_4082845, EPI_ISL_4082847, EPI_ISL_4082848, EPI_ISL_4082849, EPI_ISL_4082852, EPI_ISL_4082878, EPI_ISL_4082887, EPI_ISL_4082893, EPI_ISL_4082909, EPI_ISL_4082915, EPI_ISL_4082922, EPI_ISL_4082932, EPI_ISL_4082941, EPI_ISL_4082964, EPI_ISL_4082991, EPI_ISL_4082997, EPI_ISL_4082998, EPI_ISL_4083001, EPI_ISL_4083004, EPI_ISL_4083008, EPI_ISL_4083009, EPI_ISL_4083010, EPI_ISL_4083018, EPI_ISL_4083019, EPI_ISL_4083021, EPI_ISL_4083022, EPI_ISL_4083023, EPI_ISL_4083024, EPI_ISL_4083026, EPI_ISL_4083028, EPI_ISL_4083030, EPI_ISL_4083031, EPI_ISL_4083033, EPI_ISL_4083034, EPI_ISL_4083035, EPI_ISL_4083037, EPI_ISL_4083046, EPI_ISL_4083063, EPI_ISL_4083074, EPI_ISL_4083084, EPI_ISL_4083097, EPI_ISL_4083100, EPI_ISL_4083101, EPI_ISL_4083117, EPI_ISL_4083138, EPI_ISL_4083152, EPI_ISL_4083162, EPI_ISL_4083170, EPI_ISL_4083171, EPI_ISL_4083178, EPI_ISL_4083224, EPI_ISL_4083226, EPI_ISL_4083237, EPI_ISL_4083244, EPI_ISL_4083248, EPI_ISL_4083249, EPI_ISL_4083250, EPI_ISL_4083255, EPI_ISL_4083257, EPI_ISL_4083264, EPI_ISL_4083266, EPI_ISL_4083268, EPI_ISL_4083269, EPI_ISL_4083285, EPI_ISL_4083296, EPI_ISL_4083298, EPI_ISL_4083317, EPI_ISL_4083335, EPI_ISL_4083358, EPI_ISL_4083360, EPI_ISL_4083396, EPI_ISL_4083426, EPI_ISL_4083429, EPI_ISL_4083432, EPI_ISL_4083434			
see above	Servicio Virosis Respiratorias-Departamento Virología-INEI	Instituto Nacional Enfermedades Infecciosas C.G.Malbran	Avaro M.; Baumeister E.; Benedetti E.; Campos J.; Cisterna D.; Dattero ME; De Belder D.; Haim MS.; Lorenzo F.; Molina V.; Perandones C.; Poklepovich T.; Pontoriero A.; Russo M.; Sanchez Loria J.; Tuduri E.
EPI_ISL_3149734, EPI_ISL_3388829	Servicio de Inmunología del Hospital Julio Perrando	Laboratorio de Biología Molecular, Instituto de Medicina Regional on behalf of 'Proyecto Argentino Interinstitucional de genomica de SARS-CoV-2' (PAIS Consortium)	Bettina Brusés; Florencia Vallejos Schulze; Griselda Oria; Horacio Lucero.; Javier Mussin; Laura Formichelli; María Delia Foussal; Melina Lorenzini Campos; Raúl Maximiliano Acevedo
EPI_ISL_2558044, EPI_ISL_2758565, EPI_ISL_2758594, EPI_ISL_3219110, EPI_ISL_3219120	Servicio de Microbiologia Hospital Ramon y Cajal	Servicio de Microbiologia Hospital Ramon y Cajal	Galan JC; Martinez L. Abreu M; Ponce M; y Gonzalez-Alba JM
EPI_ISL_2516600, EPI_ISL_2516673, EPI_ISL_2516674, EPI_ISL_2516693, EPI_ISL_2516694, EPI_ISL_2516695, EPI_ISL_2516705, EPI_ISL_2516706, EPI_ISL_2516707, EPI_ISL_2516708, EPI_ISL_2516716, EPI_ISL_2516725, EPI_ISL_2516752, EPI_ISL_2516755, EPI_ISL_2516859, EPI_ISL_2934624			
see above	Servicio de Microbiología Clínica (Complejo Hospitalario de Navarra, Pamplona)	Centro de Secuenciación NASERTIC	Ana Miqueleiz; Ana Navascués; Carmen Ezpeleta Baquedano
EPI_ISL_2658202, EPI_ISL_2658207, EPI_ISL_2658212, EPI_ISL_2658215, EPI_ISL_2658235, EPI_ISL_2658236, EPI_ISL_2658243, EPI_ISL_3060990			
see above	Servicio de Microbiología. Consorcio Hospital General Universitario de Valencia	SeqCOVID-SPAIN consortium/IBV(CSIC)	Begoña Fuster Escrivá; Carme Salvador García; Concepción Gimeno Cardona and SeqCOVID-SPAIN consortium; María Dolores Ocete; Rafael Medina González
EPI_ISL_2600911, EPI_ISL_2600917, EPI_ISL_2931406	Servicio de Microbiología. Hospital Clínico Universitario de Valencia	SeqCOVID-SPAIN consortium/IBV(CSIC)	David Navarro Ortega; Eliseo Albert Vicent; Ignacio Torres and SeqCOVID-SPAIN consortium
EPI_ISL_3008587	Servicio de Microbiología. Hospital General Universitario de Castellón	SeqCOVID-SPAIN consortium/IBV(CSIC)	María Dolores Tirado Balaguer and SeqCOVID-SPAIN consortium; Rosario Moreno Muñoz
EPI_ISL_3063787, EPI_ISL_3907299	Servicio de Microbiología. Hospital Universitario Doctor Peset	SeqCOVID-SPAIN consortium/IBV(CSIC)	José Miguel Nogueira Coito and SeqCOVID-SPAIN consortium; Juan Alberola Enguídanos; Juan José Camarena Miñana; Rosa González Pellicer
EPI_ISL_2790433	Servizo de Microbioloxía. Complexo Hospitalario de Santiago de Compostela	Servizo de Microbioloxía. Complexo Hospitalario de Santiago de Compostela	Amparo Coira; Antonio Aguilera; Carlos García_Riestra; Daniel Navarro; Gema Barbeito; Iria Roca; José Llovo; Laura Millán; Manuela Hernández; María Luisa Pérez_del_Molino; Mercedes Treviño; Rocío trastoy; Teresa Lopez_Valiño; Xosé Costa
EPI_ISL_3024497	Shamir Medical Center (Asaf Harofe)	Shamir Medical Center (Asaf Harofe)	Abu Hamad Ramzia; Adina Bar Chaim; Anna Vishnevsky; Chen Weiner; Nir Rainy; Patricia Benveniste-Lekovitz; Reut Sorek Abramovich; Yevgeni Yegorov
EPI_ISL_2712542, EPI_ISL_2920435, EPI_ISL_3373825	Sharp HealthCare Laboratory	Andersen lab at Scripps Research	Art Mendoza; Cathy Woerle; Jacquelyn Berumen; Liam McGinnis; Omid Bakhtar; SEARCH Alliance San Diego with Aaron Harding
EPI_ISL_3254818, EPI_ISL_3254819, EPI_ISL_3552166	Simple Laboratories	Gagnon Lab, Southern Illinois University	Keith Gagnon
EPI_ISL_2484889	Southern Nevada Public Health Laboratory	Southern Nevada Public Health Laboratory	Michael Picker
EPI_ISL_2918821	Spital Limmattal	Institute of Medical Virology	Alexandra Trkola; Annette Audigé; Catharine Aquino; Cyril Shah; Daniel Ehrsam; Gabriela Ziltener; Guido Bloemberg; Hubert Rehrauer; Isabel Stürmer; Joel Wirz; Jon Huder; Jürg Böni; Kevin Steiner; Maria Grünberg; Maryam Zaheri; Michael Huber; Riccarda Capaul; Stefan Schmutz; Verena Kufner; Weihong Qi
EPI_ISL_3050186, EPI_ISL_3050210,	Stanford Health Care	Stanford University School of Medicine, Clinical Virology	Becky Jiang; Bernadette Troung; Daniel Solis; Hannah Wang; James Zehnder; Malaya K. Sahoo; Mamdouh Sibai; Nathan Hammond; Selamawit Bihon; and Benjamin A. Pinsky

EPI_ISL_4072051, EPI_ISL_4072052		Laboratory	
EPI_ISL_3419023	StarMed Healthcare	UNC Charlotte Environmental Monitoring Laboratory	Adam France; Cynthia Gibas; Erin Stiers; Jannatul Ferdous; Jessica Schlueter; Kevin Lambirth; Sam Kunkleman
EPI_ISL_2724749, EPI_ISL_2724788, EPI_ISL_2724799, EPI_ISL_2724825, EPI_ISL_2724828, EPI_ISL_2724830, EPI_ISL_2724833, EPI_ISL_2724834, EPI_ISL_2724842, EPI_ISL_2724844, EPI_ISL_2724849, EPI_ISL_2724873, EPI_ISL_2724880, EPI_ISL_2724900, EPI_ISL_2724909, EPI_ISL_3033098, EPI_ISL_3033104, EPI_ISL_3033105, EPI_ISL_3033106, EPI_ISL_3033120, EPI_ISL_3033163, EPI_ISL_3033187, EPI_ISL_3033188, EPI_ISL_3033266, EPI_ISL_3307957			
see above	State Laboratories Division, Hawaii State Department of Health	State Laboratories Division, Hawaii State Department of Health	Ayana Garnet; Daniel Strange; Drew Kuwazaki; Edward Desmond; Pamela O'Brien; Razvan Sultana
EPI_ISL_2800567, EPI_ISL_2800569, EPI_ISL_2800572, EPI_ISL_2800574, EPI_ISL_2800575, EPI_ISL_2800576, EPI_ISL_2800580, EPI_ISL_2800590, EPI_ISL_2800591, EPI_ISL_2800592, EPI_ISL_3049994, EPI_ISL_3050004, EPI_ISL_3050008, EPI_ISL_3050050, EPI_ISL_3050131			
see above	State Testing Facility	Altius Institute for Biomedical Research	Alex Isner; Alex Nguyen; Amanda Gale; Audra Johnson; Clem Green; Daniel Bates; Eric Thorland; Jacob Rodriguez; Jean Robinston; Jemma Nelson; Jessica Kunder; John Stamatoyannopoulos; Joshua Richards; Julia Wald; Kneshay Harper; Lauren Mitchell; Mark Frerker; Matt Hartman; Michael Buckley; Muhammad Halimun; Rebecca Bruders; Sadie Patraw; Sofia Olsson; Tobias Ragoczy
EPI_ISL_2581162, EPI_ISL_2581166, EPI_ISL_2581180, EPI_ISL_2618952, EPI_ISL_2618969, EPI_ISL_2619010, EPI_ISL_2619021, EPI_ISL_2619038, EPI_ISL_2792714, EPI_ISL_2792769, EPI_ISL_2792804, EPI_ISL_2792874, EPI_ISL_2792929, EPI_ISL_2792931, EPI_ISL_2792935, EPI_ISL_2793103, EPI_ISL_2793165, EPI_ISL_2888088, EPI_ISL_2888091, EPI_ISL_2888115, EPI_ISL_2980940, EPI_ISL_2980941, EPI_ISL_3157564, EPI_ISL_3795575, EPI_ISL_3998556, EPI_ISL_3998558, EPI_ISL_3998560, EPI_ISL_3998561, EPI_ISL_3998562, EPI_ISL_3998563, EPI_ISL_3998569, EPI_ISL_3998570, EPI_ISL_3998572, EPI_ISL_3998578, EPI_ISL_3998581, EPI_ISL_3998584, EPI_ISL_3998869, EPI_ISL_3998892			
see above	Swedish national genomic surveillance program of SARS-CoV-2	The Public Health Agency of Sweden	Alma Brolund; Maria Lind Karlberg; Maximilian Riess; Swedish national genomic surveillance program of SARS-CoV-2
EPI_ISL_2769146	Swiss Analysis AG	Institute of Medical Virology	Alexandra Trkola; Annette Audigé; Cyril Shah; Gabriela Ziltener; Guido Bloemberg; Jon Huder; Jürg Böni; Kevin Steiner; Maria Grünberg; Maryam Zaheri; Michael Huber; Riccarda Capaul; Stefan Schmutz; Verena Kufner
EPI_ISL_3030108	Synlab Suisse SA	Clinical Bacteriology	Adrian Egli; Alfredo Mari; Fanny Wegner; Hans Hirsch; Helena MB Seth-Smith; Julia Bielicki; Karoline Leuzinger; Manuel Battegay; Tim Roloff
EPI_ISL_2626957, EPI_ISL_2626963, EPI_ISL_2868602, EPI_ISL_2868604, EPI_ISL_2868605, EPI_ISL_2868607	TEMPUS LABS INC	Wadsworth Center, New York State Department of Health	Alexis Russell; Catharine Prussing; Daryl M. Lamson; Erasmus Schneider; Erica Lasek-Nesselquist; John Kelly; Jonathan Plitnick; Kirsten St. George; Matthew Shudt; Melissa A Leisner; Navjot Singh
EPI_ISL_2769191, EPI_ISL_2769204, EPI_ISL_2769277, EPI_ISL_3088803, EPI_ISL_3325976, EPI_ISL_3325992, EPI_ISL_3326351, EPI_ISL_3326437, EPI_ISL_3326706, EPI_ISL_3460398, EPI_ISL_3460423, EPI_ISL_3460429, EPI_ISL_3460463, EPI_ISL_3460472, EPI_ISL_3460490, EPI_ISL_3460511, EPI_ISL_3460515, EPI_ISL_3460516, EPI_ISL_3460532, EPI_ISL_3460614, EPI_ISL_3661970, EPI_ISL_3826274			
see above	TGen North	TGen North	"Jolene Bowers; Brett Van Tassel; Chris French; Darrin Lemmer; Dave Engelthaler; Dave Engelthaler"; Hayley Yaglom; Heather Centner; Jolene Bowers
EPI_ISL_3242037	TX-Houston Health Department Laboratory	Centers for Disease Control and Prevention Division of Viral Diseases, Pathogen Discovery	Alex Burgin; Ben L. Rambo-Martin; Clinton R. Paden; Dakota Howard; Dave Wentworth; Dhwani Batra; Jasmine Padilla; Justin Lee; Krista Queen; Kristen Knipe; Kristine Lacek; Mark Burroughs; Matthew Schmerer; Meghan Bentz; Mili Sheth; Peter Cook; Sam Shepard; Sarah Nobles; Suxiang Tong; Vivien Dugan; Yvette Unoarumhi
EPI_ISL_3267887	TXDSHS	TXDSHS	Anita Pokharel; Bonnie Oh; Chun Wang; Grace Kubin; Jenny Zhang; Karen Bobier; Lorraine Rodriguez; Maliha Rahman; Mayela Pedrueza; Myong Koag; Rachel Lee; Rashmi Tuladhar
EPI_ISL_2790535, EPI_ISL_2800904, EPI_ISL_2800905, EPI_ISL_2833809, EPI_ISL_2966844, EPI_ISL_2966850	Tampa General Hospital Esoteric Lab	Tampa General Hospital Esoteric Research & Development Lab	Amorce Lima; Deanna Becker; Dominic Uy; Elaine Vendrone; Jon Faughn; Jorge Mecias-Frias; Suzane Silbert; Vicki Healer
EPI_ISL_3925995, EPI_ISL_3926065, EPI_ISL_3930809	The National University Hospital of Iceland	deCODE genetics	Agnar Helgason; Alma Moller; Arna B Agustsdottir; Arnaldur Gylfason; Asgeir Sigurdsson; Aslaug Jonasdottir; Berglind Eiriksdottir; Bjarni Thorbjornsson; Brynjar O Jensson; Daniel F Gudbjartsson; Droplaug N Magnusdottir; Elisabet E Gardarsdottir; Emil A Thorarensen; Gardar Sveinbjornsson; Gisli Masson; Gudmundur Georgsson; Gudmundur L Norddahl; Gudrun Sigmundsdottir; Hakon Jonsson; Hannes Eggertsson; Hilma Holm; Ingileif Jonsdottir; Jona Saemundsdottir; Kamilla S Josefsdottir; Kari Stefansson; Karl G Kristinsson; Kjartan R Gudmundsson; Kristin E Sveinsdottir; Kristjan E Hjorleifsson; Louise le Roux; Maney Sveinsdottir; Olafia S Gretarsdottir; Olafur T Magnusson; Pall Melsted; Patrick Sulem; Run Fridriksdottir; Solvi Rognvaldsson; Thora R Gunnarsdottir; Thordur Kristjansson; Thorolfur Gudnason; Unnur Thorsteinsdottir
EPI_ISL_2762132	Thüringer Landesamtes für Verbraucherschutz	Robert Koch Institute	
EPI_ISL_2774572	Torrance Memorial Medical Center	Los Angeles County Public Health Laboratories	P. Hemarajata et al.
EPI_ISL_3152417	TriCore Reference Laboratories	Center for Global Health, University of New Mexico Health Sciences Center	Cecilia Thompson; Darrell Dinwiddie; Daryl Domman; Karissa Culbreath; Kendra Pesko; Kurt Schwalm; Valerie Morley
EPI_ISL_3049350	Tulare County Public Health Laboratory	Tulare County Public Health Laboratory	Denise L Lopez; Joshua Kootstra
EPI_ISL_2840625	U.O. Igiene, Ospedale Policlinico San Martino IRCCS Genova	U.O. Igiene, Ospedale Policlinico San Martino	Bruzzone Bianca; Caligiuri Patrizia; De Pace Vanessa; Domnich Alexander; Icardi Giancarlo on behalf of SARS-CoV-2 ITALIAN RESEARCH ENTERPRISE-(SCIRE) Collaborative Group; Orsi Andrea; Ricucci Valentina
EPI_ISL_3266323	U.O. Microbiologia Laboratorio Unico Centro Servizi - AUSL della Romagna	U.O. Microbiologia, Laboratorio Unico Centro Servizi - AUSL della Romagna	Giorgio Dirani
EPI_ISL_3912250	UAPS FERNANDO FACANHA	Analytical Competence Molecular Epidemiology Lab/ACME, Oswaldo Cruz Foundation, Ceara (FIOCRUZ CE)	Cleber Furtado Aksenen; Fabio Miyajima; Fernando Braga Stehling; Francisco Eder de Moura Lopes; Jamille Maria Mendes Bezerra; Joaquim Cesar do Nascimento Sousa Junior; Pedro Miguel Carneiro Jeronimo; Suzana Porto Almeida & Lucas Delerino on behalf of COVID-19 FIOCRUZ Genomic Network; Thais Ferreira de Oliveira; Thais de Oliveira Costa; Ticiane Cavalcante de Souza; Veridiana Pessoa Miyajima
EPI_ISL_3912251	UAPS FLORESTA	Analytical Competence Molecular Epidemiology Lab/ACME, Oswaldo Cruz Foundation, Ceara (FIOCRUZ CE)	Cleber Furtado Aksenen; Fabio Miyajima; Fernando Braga Stehling; Francisco Eder de Moura Lopes; Jamille Maria Mendes Bezerra; Joaquim Cesar do Nascimento Sousa Junior; Pedro Miguel Carneiro Jeronimo; Suzana Porto Almeida & Lucas Delerino on behalf of COVID-19 FIOCRUZ Genomic Network; Thais Ferreira de Oliveira; Thais de Oliveira Costa; Ticiane Cavalcante de Souza; Veridiana Pessoa Miyajima
EPI_ISL_3912264	UBASF AMADOR	Analytical Competence Molecular Epidemiology Lab/ACME, Oswaldo Cruz Foundation, Ceara (FIOCRUZ CE)	Cleber Furtado Aksenen; Fabio Miyajima; Fernando Braga Stehling; Francisco Eder de Moura Lopes; Jamille Maria Mendes Bezerra; Joaquim Cesar do Nascimento Sousa Junior; Pedro Miguel Carneiro Jeronimo; Suzana Porto Almeida & Lucas Delerino on behalf of COVID-19 FIOCRUZ Genomic Network; Thais Ferreira de Oliveira; Thais de Oliveira Costa; Ticiane Cavalcante de Souza; Veridiana Pessoa Miyajima
EPI_ISL_3912265	UBASF DE GUARIBAS	Analytical Competence Molecular Epidemiology Lab/ACME, Oswaldo Cruz Foundation, Ceara (FIOCRUZ CE)	Cleber Furtado Aksenen; Fabio Miyajima; Fernando Braga Stehling; Francisco Eder de Moura Lopes; Jamille Maria Mendes Bezerra; Joaquim Cesar do Nascimento Sousa Junior; Pedro Miguel Carneiro Jeronimo; Suzana Porto Almeida & Lucas Delerino on behalf of COVID-19 FIOCRUZ Genomic Network; Thais Ferreira de Oliveira; Thais de Oliveira Costa; Ticiane Cavalcante de Souza; Veridiana Pessoa Miyajima
EPI_ISL_3912268	UBS ACUDE DO MATO	Analytical Competence Molecular Epidemiology Lab/ACME, Oswaldo Cruz Foundation, Ceara (FIOCRUZ CE)	Cleber Furtado Aksenen; Fabio Miyajima; Fernando Braga Stehling; Francisco Eder de Moura Lopes; Jamille Maria Mendes Bezerra; Joaquim Cesar do Nascimento Sousa Junior; Pedro Miguel Carneiro Jeronimo; Suzana Porto Almeida & Lucas Delerino on behalf of COVID-19 FIOCRUZ Genomic Network; Thais Ferreira de Oliveira; Thais de Oliveira Costa; Ticiane Cavalcante de Souza; Veridiana Pessoa Miyajima
EPI_ISL_3912270, EPI_ISL_3912272, EPI_ISL_3912273, EPI_ISL_3912275, EPI_ISL_3912276, EPI_ISL_3912277	UBS CAIC II	Analytical Competence Molecular Epidemiology Lab/ACME, Oswaldo Cruz Foundation, Ceara (FIOCRUZ CE)	Cleber Furtado Aksenen; Fabio Miyajima; Fernando Braga Stehling; Francisco Eder de Moura Lopes; Jamille Maria Mendes Bezerra; Joaquim Cesar do Nascimento Sousa Junior; Pedro Miguel Carneiro Jeronimo; Suzana Porto Almeida & Lucas Delerino on behalf of COVID-19 FIOCRUZ Genomic Network; Thais Ferreira de Oliveira; Thais de Oliveira Costa; Ticiane Cavalcante de Souza; Veridiana Pessoa Miyajima
EPI_ISL_2494144	UBS CELESTINO CARNIELO	Instituto Butantan	Antonio Jorge Martins; Claudia Renata dos Santos Barros; David Schlesinger; Debora Botequio Moretti; Dimas Tadeu Covas; Elaine Cristina Marqueze; Elaine Vieira Santos; Evandra Strazza Rodrigues; Heidge Fukumasu; Jayme Augusto de Souza-Neto; José Salvatore Leister Patané; Luiz Alcantara; Luiz Lehmann Coutinho; Maria Carolina Elias; Maurício Lacerda Nogueira; Rafael dos Santos Bezerra; Raul Machado Neto; Rejane Maria Tommasini Grotto; Ricardo Haddad; Sandra Coccuzzo Sampaio Vessoni; Simone Kashima; Svetoslav Nanev Slavov; Vincent Louis Viala
EPI_ISL_3912278	UBS CENTRO	Analytical Competence Molecular Epidemiology Lab/ACME, Oswaldo Cruz Foundation, Ceara (FIOCRUZ CE)	Cleber Furtado Aksenen; Fabio Miyajima; Fernando Braga Stehling; Francisco Eder de Moura Lopes; Jamille Maria Mendes Bezerra; Joaquim Cesar do Nascimento Sousa Junior; Pedro Miguel Carneiro Jeronimo; Suzana Porto Almeida & Lucas Delerino on behalf of COVID-19 FIOCRUZ Genomic Network; Thais Ferreira de Oliveira; Thais de Oliveira Costa; Ticiane Cavalcante de Souza; Veridiana Pessoa Miyajima
EPI_ISL_2493866, EPI_ISL_2493868, EPI_ISL_2493869	UBS DE JAMBEIRO JOCELINA MEIRELES	Instituto Butantan	Antonio Jorge Martins; Claudia Renata dos Santos Barros; David Schlesinger; Debora Botequio Moretti; Dimas Tadeu Covas; Elaine Cristina Marqueze; Elaine Vieira Santos; Evandra Strazza Rodrigues; Heidge Fukumasu; Jayme Augusto de Souza-Neto; José Salvatore Leister Patané; Luiz Alcantara; Luiz Lehmann Coutinho; Maria Carolina Elias; Maurício Lacerda Nogueira; Rafael dos Santos Bezerra; Raul Machado Neto; Rejane Maria Tommasini Grotto; Ricardo Haddad; Sandra Coccuzzo Sampaio Vessoni; Simone Kashima; Svetoslav Nanev Slavov; Vincent Louis Viala
EPI_ISL_3536374	UBS DE MUFUMBAL	Analytical Competence Molecular Epidemiology Lab/ACME, Oswaldo Cruz Foundation, Ceara (FIOCRUZ CE)	Cleber Furtado Aksenen; Fabio Miyajima; Fernando Braga Stehling; Francisco Eder de Moura Lopes; Jamille Maria Mendes Bezerra; Joaquim César do Nascimento Sousa Junior; Pedro Miguel Carneiro Jeronimo; Suzana Porto Almeida e Lucas Delerino; Thais Ferreira de Oliveira; Thais de Oliveira Costa; Ticiane Cavalcante de Souza; Veridiana Pessoa Miyajima
EPI_ISL_3912280, EPI_ISL_3912281, EPI_ISL_3912282, EPI_ISL_3912283, EPI_ISL_3912284, EPI_ISL_3912285, EPI_ISL_3912287			
see above	UBS DO BAIRRO SAO JOSE	Analytical Competence Molecular Epidemiology Lab/ACME, Oswaldo Cruz Foundation, Ceara (FIOCRUZ	Cleber Furtado Aksenen; Fabio Miyajima; Fernando Braga Stehling; Francisco Eder de Moura Lopes; Jamille Maria Mendes Bezerra; Joaquim Cesar do Nascimento Sousa Junior; Pedro Miguel Carneiro Jeronimo; Suzana Porto Almeida & Lucas Delerino on behalf of COVID-19 FIOCRUZ Genomic Network; Thais Ferreira de Oliveira; Thais de Oliveira Costa; Ticiane Cavalcante de Souza; Veridiana Pessoa Miyajima

		CE)	
EPI_ISL_3536375	UBS DO CAMPO LINDO	Analytical Competence Molecular Epidemiology Lab/ACME, Oswaldo Cruz Foundation, Ceara (FIOCRUZ CE)	Cleber Furtado Aksenen; Fabio Miyajima; Fernando Braga Stehling; Francisco Eder de Moura Lopes; Jamille Maria Mendes Bezerra; Joaquim César do Nascimento Sousa Junior; Pedro Miguel Carneiro Jeronimo; Suzana Porto Almeida e Lucas Delerino; Thais Ferreira de Oliveira; Thais de Oliveira Costa; Ticiane Cavalcante de Souza; Veridiana Pessoa Miyajima
EPI_ISL_2494114, EPI_ISL_2494118, EPI_ISL_2494119, EPI_ISL_2494120, EPI_ISL_2494121, EPI_ISL_2494122, EPI_ISL_2494126, EPI_ISL_2494129			
see above	UBS DR ALFREDO DANTAS DE SOUZA UMUARAMA	Instituto Butantan	Antonio Jorge Martins; Claudia Renata dos Santos Barros; David Schlesinger; Debora Botequio Moretti; Dimas Tadeu Covas; Elaine Cristina Marqueze; Elaine Vieira Santos; Evandra Strazza Rodrigues; Heidge Fukumasu; Jayme Augusto de Souza-Neto; José Salvatore Leister Patané; Luiz Alcantara; Luiz Lehmann Coutinho; Maria Carolina Elias; Maurício Lacerda Nogueira; Rafael dos Santos Bezerra; Raul Machado Neto; Rejane Maria Tommasini Grotto; Ricardo Haddad; Sandra Coccuzzo Sampaio Vessoni; Simone Kashima; Svetoslav Nanev Slavov; Vincent Louis Viala
EPI_ISL_2494320	UBS JARDIM CAIUBY	Instituto Butantan	Antonio Jorge Martins; Claudia Renata dos Santos Barros; David Schlesinger; Debora Botequio Moretti; Dimas Tadeu Covas; Elaine Cristina Marqueze; Elaine Vieira Santos; Evandra Strazza Rodrigues; Heidge Fukumasu; Jayme Augusto de Souza-Neto; José Salvatore Leister Patané; Luiz Alcantara; Luiz Lehmann Coutinho; Maria Carolina Elias; Maurício Lacerda Nogueira; Rafael dos Santos Bezerra; Raul Machado Neto; Rejane Maria Tommasini Grotto; Ricardo Haddad; Sandra Coccuzzo Sampaio Vessoni; Simone Kashima; Svetoslav Nanev Slavov; Vincent Louis Viala
EPI_ISL_2493849	UBS JARDIM ODETE	Instituto Butantan	Antonio Jorge Martins; Claudia Renata dos Santos Barros; David Schlesinger; Debora Botequio Moretti; Dimas Tadeu Covas; Elaine Cristina Marqueze; Elaine Vieira Santos; Evandra Strazza Rodrigues; Heidge Fukumasu; Jayme Augusto de Souza-Neto; José Salvatore Leister Patané; Luiz Alcantara; Luiz Lehmann Coutinho; Maria Carolina Elias; Maurício Lacerda Nogueira; Rafael dos Santos Bezerra; Raul Machado Neto; Rejane Maria Tommasini Grotto; Ricardo Haddad; Sandra Coccuzzo Sampaio Vessoni; Simone Kashima; Svetoslav Nanev Slavov; Vincent Louis Viala
EPI_ISL_3912288	UBS MARATOAN	Analytical Competence Molecular Epidemiology Lab/ACME, Oswaldo Cruz Foundation, Ceara (FIOCRUZ CE)	Cleber Furtado Aksenen; Fabio Miyajima; Fernando Braga Stehling; Francisco Eder de Moura Lopes; Jamille Maria Mendes Bezerra; Joaquim Cesar do Nascimento Sousa Junior; Pedro Miguel Carneiro Jeronimo; Suzana Porto Almeida & Lucas Delerino on behalf of COVID-19 FIOCRUZ Genomic Network; Thais Ferreira de Oliveira; Thais de Oliveira Costa; Ticiane Cavalcante de Souza; Veridiana Pessoa Miyajima
EPI_ISL_3419663, EPI_ISL_3419683, EPI_ISL_3419684, EPI_ISL_3419690	UC Davis Genome Center and Healthy Davis Together	UC Davis Genome Center	UC Davis
EPI_ISL_3067001, EPI_ISL_3067009, EPI_ISL_3067011, EPI_ISL_3067012, EPI_ISL_3067050, EPI_ISL_3370432, EPI_ISL_3370433, EPI_ISL_3370457			
see above	UC-Christus Clinical Hospital Laboratory	Laboratory of Molecular Virology, School of Medicine, Pontificia Universidad Catolica de Chile	Ana Maira Guzman; Andres E. Munoz-Marcos; Catalina Pardo-Roa; Eileen Serrano; Erick Salinas; Estefany Poblete; Francisco Melo; Jorge Levican; Leonardo I. Almonacid; Maria Jose Avendano; Maria Patricia Vega; Rafael A. Medina; Ricardo Enrique de la Barra; Tamara Garcia-Salum
EPI_ISL_2860319, EPI_ISL_2860326	UCC	Grubaugh Lab - Yale School of Public Health	Anderson Brito; Andrea Firpo; Annie Watkins; Chaney Kalinich; Chantal Vogels; David Rivera Aponte; Ernesto Vazquez; Fabiola Cruz-Lopez; Fabiola Fontanet; Isabel Ott; Jessica Rothman; Joseph Fauver; Karolane Gonzalez; Kendall Billig; Mallery Breban; Mary Petrone; Natalie Machargo; Nathan Grubaugh; Patricia Serrano; Tania Mitwalli; Tara Alpert; Tobias Koch; Vianca Aponte
EPI_ISL_2960091, EPI_ISL_2960100, EPI_ISL_2960102, EPI_ISL_3025377, EPI_ISL_3025378, EPI_ISL_3025382, EPI_ISL_3025383, EPI_ISL_3025385, EPI_ISL_3025388, EPI_ISL_3104709, EPI_ISL_3236525			
see above	UCC (Universidad Central del Caribe)	Grubaugh Lab - Yale School of Public Health	Alejandaro Vallejo Degaudenzi; Anderson Brito; Andrea Firpo; Annie Watkins; Chaney Kalinich; Chantal Vogels; David Rivera Aponte; Elisa Contreras; Ernesto Vazquez; Esperanza Mendoza; Fabiola Cruz-Lopez; Fabiola Fontanet; Isabel Ott; Jessica Rothman; Joseph Fauver; Karolane Gonzalez; Kendall Billig; Mallery Breban; Mary Petrone; Natalie Machargo; Nathan Grubaugh; Patricia Serrano; Robert Paulino-Ramirez; Tania Mitwalli; Tara Alpert; Tobias Koch; Vianca Aponte; Victor Virgilio Calderon
EPI_ISL_3506323, EPI_ISL_3710096	UCLA Clinical Micro Lab	Los Angeles County Public Health Laboratories	P. Hemarajata et al.
EPI_ISL_2547293, EPI_ISL_2547300, EPI_ISL_2547301, EPI_ISL_2547302, EPI_ISL_2547313, EPI_ISL_2547319, EPI_ISL_2547320, EPI_ISL_2547321, EPI_ISL_2661538, EPI_ISL_2661544, EPI_ISL_2661545, EPI_ISL_2661546, EPI_ISL_2661547, EPI_ISL_2661548, EPI_ISL_2661549, EPI_ISL_3462085, EPI_ISL_3462086, EPI_ISL_3462088, EPI_ISL_3462089, EPI_ISL_3462090			
see above	UHTL, University Hospitals	UHTL, University Hospitals	Alouani, D.; Sadri, N.; Song, X.
EPI_ISL_2695325, EPI_ISL_2695326	ULSM - Matosinhos	Instituto Nacional de Saude (INSA)	Borges et al
EPI_ISL_3061561	UNC-CH COVID Surveillance Lab	Jeremy Wang	Amir Barzin; Amy James Loftis; Corbin Jones; Jeremy Wang; Melissa Miller; Susan Fiscus
EPI_ISL_3066764	UNIDAD DE DIAGNOSTICO HEMATO ONCOLOGICA	Instituto Nacional de Salud	Carlos Franco-Muñoz; Carmen Osorio; Diana Malo; Diego A. Álvarez-Díaz; Diego Andrés Prada; Gerardo Santamaría; Hector Alejandro Ruiz-Moreno; Jhonnatan Reales-González; Jorge Rivera; Juan Camilo Martinez; Julian Naizaque; Katherine Laiton-Donato; Lisseth Pardo; Magdalena Wiesner; Marcela Mercado-Reyes; Maria T.Herrera-Sepúlveda; Marta Lopez Blanco; Martha Lucia Ospina Martinez; Paola Rojas; Sergio Gomez; Sheryll Corchuelo; Ángela Alarcon Cruz
EPI_ISL_3385787	UNIDAD DE DIAGNOSTICO HEMATO ONCOLOGICA	Instituto Nacional de Salud-Dirección de Investigación en Salud Pública	Carlos Franco-Muñoz; Carmen Osorio; Diana Malo; Diego A. Álvarez-Díaz; Diego Andrés Prada; Gerardo Santamaría; Hector Alejandro Ruiz-Moreno; Jhonnatan Reales-González; Jorge Rivera; Juan Camilo Martinez; Julian Naizaque; Katherine Laiton-Donato; Lisseth Pardo; Magdalena Wiesner; Marcela Mercado-Reyes; Maria T. Herrera-Sepúlveda; Marta Lopez Blanco; Martha Lucia Ospina Martinez; Paola Rojas; Sergio Gomez; Sheryll Corchuelo; Ángela Alarcon Cruz
EPI_ISL_3536379	UNIDADE BASICA DE SAUDE ALTO BRILHANTE	Analytical Competence Molecular Epidemiology Lab/ACME, Oswaldo Cruz Foundation, Ceara (FIOCRUZ CE)	Cleber Furtado Aksenen; Fabio Miyajima; Fernando Braga Stehling; Francisco Eder de Moura Lopes; Jamille Maria Mendes Bezerra; Joaquim César do Nascimento Sousa Junior; Pedro Miguel Carneiro Jeronimo; Suzana Porto Almeida e Lucas Delerino; Thais Ferreira de Oliveira; Thais de Oliveira Costa; Ticiane Cavalcante de Souza; Veridiana Pessoa Miyajima
EPI_ISL_3536380	UNIDADE BASICA DE SAUDE COLIBRIS	Analytical Competence Molecular Epidemiology Lab/ACME, Oswaldo Cruz Foundation, Ceara (FIOCRUZ CE)	Cleber Furtado Aksenen; Fabio Miyajima; Fernando Braga Stehling; Francisco Eder de Moura Lopes; Jamille Maria Mendes Bezerra; Joaquim César do Nascimento Sousa Junior; Pedro Miguel Carneiro Jeronimo; Suzana Porto Almeida e Lucas Delerino; Thais Ferreira de Oliveira; Thais de Oliveira Costa; Ticiane Cavalcante de Souza; Veridiana Pessoa Miyajima
EPI_ISL_3912293	UNIDADE BASICA DE SAUDE DA GIRILANDIA	Analytical Competence Molecular Epidemiology Lab/ACME, Oswaldo Cruz Foundation, Ceara (FIOCRUZ CE)	Cleber Furtado Aksenen; Fabio Miyajima; Fernando Braga Stehling; Francisco Eder de Moura Lopes; Jamille Maria Mendes Bezerra; Joaquim Cesar do Nascimento Sousa Junior; Pedro Miguel Carneiro Jeronimo; Suzana Porto Almeida & Lucas Delerino on behalf of COVID-19 FIOCRUZ Genomic Network; Thais Ferreira de Oliveira; Thais de Oliveira Costa; Ticiane Cavalcante de Souza; Veridiana Pessoa Miyajima
EPI_ISL_3912294, EPI_ISL_3912295	UNIDADE BASICA DE SAUDE DA SEDE	Analytical Competence Molecular Epidemiology Lab/ACME, Oswaldo Cruz Foundation, Ceara (FIOCRUZ CE)	Cleber Furtado Aksenen; Fabio Miyajima; Fernando Braga Stehling; Francisco Eder de Moura Lopes; Jamille Maria Mendes Bezerra; Joaquim Cesar do Nascimento Sousa Junior; Pedro Miguel Carneiro Jeronimo; Suzana Porto Almeida & Lucas Delerino on behalf of COVID-19 FIOCRUZ Genomic Network; Thais Ferreira de Oliveira; Thais de Oliveira Costa; Ticiane Cavalcante de Souza; Veridiana Pessoa Miyajima
EPI_ISL_3912296	UNIDADE BASICA DE SAUDE DE BOA AGUA	Analytical Competence Molecular Epidemiology Lab/ACME, Oswaldo Cruz Foundation, Ceara (FIOCRUZ CE)	Cleber Furtado Aksenen; Fabio Miyajima; Fernando Braga Stehling; Francisco Eder de Moura Lopes; Jamille Maria Mendes Bezerra; Joaquim Cesar do Nascimento Sousa Junior; Pedro Miguel Carneiro Jeronimo; Suzana Porto Almeida & Lucas Delerino on behalf of COVID-19 FIOCRUZ Genomic Network; Thais Ferreira de Oliveira; Thais de Oliveira Costa; Ticiane Cavalcante de Souza; Veridiana Pessoa Miyajima
EPI_ISL_3912297	UNIDADE BASICA DE SAUDE DE ROLDAO	Analytical Competence Molecular Epidemiology Lab/ACME, Oswaldo Cruz Foundation, Ceara (FIOCRUZ CE)	Cleber Furtado Aksenen; Fabio Miyajima; Fernando Braga Stehling; Francisco Eder de Moura Lopes; Jamille Maria Mendes Bezerra; Joaquim Cesar do Nascimento Sousa Junior; Pedro Miguel Carneiro Jeronimo; Suzana Porto Almeida & Lucas Delerino on behalf of COVID-19 FIOCRUZ Genomic Network; Thais Ferreira de Oliveira; Thais de Oliveira Costa; Ticiane Cavalcante de Souza; Veridiana Pessoa Miyajima
EPI_ISL_3536344	UNIDADE BASICA DE SAUDE DE SAO VICENTE	Analytical Competence Molecular Epidemiology Lab/ACME, Oswaldo Cruz Foundation, Ceara (FIOCRUZ CE)	Cleber Furtado Aksenen; Fabio Miyajima; Fernando Braga Stehling; Francisco Eder de Moura Lopes; Jamille Maria Mendes Bezerra; Joaquim César do Nascimento Sousa Junior; Pedro Miguel Carneiro Jeronimo; Suzana Porto Almeida e Lucas Delerino; Thais Ferreira de Oliveira; Thais de Oliveira Costa; Ticiane Cavalcante de Souza; Veridiana Pessoa Miyajima
EPI_ISL_3912298	UNIDADE BASICA DE SAUDE DIVINO ESPIRITO SANTO	Analytical Competence Molecular Epidemiology Lab/ACME, Oswaldo Cruz Foundation, Ceara (FIOCRUZ CE)	Cleber Furtado Aksenen; Fabio Miyajima; Fernando Braga Stehling; Francisco Eder de Moura Lopes; Jamille Maria Mendes Bezerra; Joaquim Cesar do Nascimento Sousa Junior; Pedro Miguel Carneiro Jeronimo; Suzana Porto Almeida & Lucas Delerino on behalf of COVID-19 FIOCRUZ Genomic Network; Thais Ferreira de Oliveira; Thais de Oliveira Costa; Ticiane Cavalcante de Souza; Veridiana Pessoa Miyajima
EPI_ISL_3912299, EPI_ISL_3912300	UNIDADE BASICA DE SAUDE DO CAMPO DE AVIACAO	Analytical Competence Molecular Epidemiology Lab/ACME, Oswaldo Cruz Foundation, Ceara (FIOCRUZ CE)	Cleber Furtado Aksenen; Fabio Miyajima; Fernando Braga Stehling; Francisco Eder de Moura Lopes; Jamille Maria Mendes Bezerra; Joaquim Cesar do Nascimento Sousa Junior; Pedro Miguel Carneiro Jeronimo; Suzana Porto Almeida & Lucas Delerino on behalf of COVID-19 FIOCRUZ Genomic Network; Thais Ferreira de Oliveira; Thais de Oliveira Costa; Ticiane Cavalcante de Souza; Veridiana Pessoa Miyajima
EPI_ISL_3912301	UNIDADE BASICA DE SAUDE DO SAO FRANCISCO	Analytical Competence Molecular Epidemiology Lab/ACME, Oswaldo Cruz Foundation, Ceara (FIOCRUZ CE)	Cleber Furtado Aksenen; Fabio Miyajima; Fernando Braga Stehling; Francisco Eder de Moura Lopes; Jamille Maria Mendes Bezerra; Joaquim Cesar do Nascimento Sousa Junior; Pedro Miguel Carneiro Jeronimo; Suzana Porto Almeida & Lucas Delerino on behalf of COVID-19 FIOCRUZ Genomic Network; Thais Ferreira de Oliveira; Thais de Oliveira Costa; Ticiane Cavalcante de Souza; Veridiana Pessoa Miyajima
EPI_ISL_2494175	UNIDADE BASICA DE SAUDE DR MATHEUS GABRIEL BONASSA	Instituto Butantan	Antonio Jorge Martins; Claudia Renata dos Santos Barros; David Schlesinger; Debora Botequio Moretti; Dimas Tadeu Covas; Elaine Cristina Marqueze; Elaine Vieira Santos; Evandra Strazza Rodrigues; Heidge Fukumasu; Jayme Augusto de Souza-Neto; José Salvatore Leister Patané; Luiz Alcantara; Luiz Lehmann Coutinho; Maria Carolina Elias; Maurício Lacerda Nogueira; Rafael dos Santos Bezerra; Raul Machado Neto; Rejane Maria Tommasini Grotto; Ricardo Haddad; Sandra Coccuzzo Sampaio Vessoni; Simone Kashima; Svetoslav Nanev Slavov; Vincent Louis Viala
EPI_ISL_3536329	UNIDADE BASICA DE SAUDE FAZENDINHA	Analytical Competence Molecular Epidemiology Lab/ACME, Oswaldo Cruz Foundation, Ceara (FIOCRUZ CE)	Cleber Furtado Aksenen; Fabio Miyajima; Fernando Braga Stehling; Francisco Eder de Moura Lopes; Jamille Maria Mendes Bezerra; Joaquim César do Nascimento Sousa Junior; Pedro Miguel Carneiro Jeronimo; Suzana Porto Almeida e Lucas Delerino; Thais Ferreira de Oliveira; Thais de Oliveira Costa; Ticiane Cavalcante de Souza; Veridiana Pessoa Miyajima
EPI_ISL_3536178, EPI_ISL_3536179, EPI_ISL_3536180	UNIDADE BASICA DE SAUDE HORACIO FONTENELE DE SAMPAIO	Analytical Competence Molecular Epidemiology Lab/ACME, Oswaldo Cruz Foundation, Ceara (FIOCRUZ CE)	Cleber Furtado Aksenen; Fabio Miyajima; Fernando Braga Stehling; Francisco Eder de Moura Lopes; Jamille Maria Mendes Bezerra; Joaquim César do Nascimento Sousa Junior; Pedro Miguel Carneiro Jeronimo; Suzana Porto Almeida e Lucas Delerino; Thais Ferreira de Oliveira; Thais de Oliveira Costa; Ticiane Cavalcante de Souza; Veridiana Pessoa Miyajima
EPI_ISL_3536330	UNIDADE BASICA DE SAUDE PICOS	Analytical Competence Molecular Epidemiology Lab/ACME, Oswaldo Cruz Foundation, Ceara (FIOCRUZ CE)	Cleber Furtado Aksenen; Fabio Miyajima; Fernando Braga Stehling; Francisco Eder de Moura Lopes; Jamille Maria Mendes Bezerra; Joaquim César do Nascimento Sousa Junior; Pedro Miguel Carneiro Jeronimo; Suzana Porto Almeida e Lucas Delerino; Thais Ferreira de Oliveira; Thais de Oliveira Costa; Ticiane Cavalcante de Souza; Veridiana Pessoa Miyajima

EPI_ISL_3536381	UNIDADE BASICA DE SAUDE SANTA TEREZA	Analytical Competence Molecular Epidemiology Lab/ACME, Oswaldo Cruz Foundation, Ceara (FIOCRUZ CE)	Cleber Furtado Aksenen; Fabio Miyajima; Fernando Braga Stehling; Francisco Eder de Moura Lopes; Jamille Maria Mendes Bezerra; Joaquim César do Nascimento Sousa Junior; Pedro Miguel Carneiro Jeronimo; Suzana Porto Almeida e Lucas Delerino; Thais Ferreira de Oliveira; Thais de Oliveira Costa; Ticiane Cavalcante de Souza; Veridiana Pessoa Miyajima
EPI_ISL_3536382	UNIDADE BASICA DE SAUDE SANTANA	Analytical Competence Molecular Epidemiology Lab/ACME, Oswaldo Cruz Foundation, Ceara (FIOCRUZ CE)	Cleber Furtado Aksenen; Fabio Miyajima; Fernando Braga Stehling; Francisco Eder de Moura Lopes; Jamille Maria Mendes Bezerra; Joaquim César do Nascimento Sousa Junior; Pedro Miguel Carneiro Jeronimo; Suzana Porto Almeida e Lucas Delerino; Thais Ferreira de Oliveira; Thais de Oliveira Costa; Ticiane Cavalcante de Souza; Veridiana Pessoa Miyajima
EPI_ISL_3536365, EPI_ISL_3912303	UNIDADE BASICA DE SAUDE SEDE I	Analytical Competence Molecular Epidemiology Lab/ACME, Oswaldo Cruz Foundation, Ceara (FIOCRUZ CE)	Cleber Furtado Aksenen; Fabio Miyajima; Fernando Braga Stehling; Francisco Eder de Moura Lopes; Jamille Maria Mendes Bezerra; Joaquim Cesar do Nascimento Sousa Junior; Joaquim César do Nascimento Sousa Junior; Pedro Miguel Carneiro Jeronimo; Suzana Porto Almeida & Lucas Delerino on behalf of COVID-19 FIOCRUZ Genomic Network; Suzana Porto Almeida e Lucas Delerino; Thais Ferreira de Oliveira; Thais de Oliveira Costa; Ticiane Cavalcante de Souza; Veridiana Pessoa Miyajima
EPI_ISL_3536338	UNIDADE BASICA DE SAUDE VALMIR XAVIER	Analytical Competence Molecular Epidemiology Lab/ACME, Oswaldo Cruz Foundation, Ceara (FIOCRUZ CE)	Cleber Furtado Aksenen; Fabio Miyajima; Fernando Braga Stehling; Francisco Eder de Moura Lopes; Jamille Maria Mendes Bezerra; Joaquim César do Nascimento Sousa Junior; Pedro Miguel Carneiro Jeronimo; Suzana Porto Almeida e Lucas Delerino; Thais Ferreira de Oliveira; Thais de Oliveira Costa; Ticiane Cavalcante de Souza; Veridiana Pessoa Miyajima
EPI_ISL_3536148	UNIDADE BASICA DE SAUDE VILA COBAL	Analytical Competence Molecular Epidemiology Lab/ACME, Oswaldo Cruz Foundation, Ceara (FIOCRUZ CE)	Cleber Furtado Aksenen; Fabio Miyajima; Fernando Braga Stehling; Francisco Eder de Moura Lopes; Jamille Maria Mendes Bezerra; Joaquim César do Nascimento Sousa Junior; Pedro Miguel Carneiro Jeronimo; Suzana Porto Almeida e Lucas Delerino; Thais Ferreira de Oliveira; Thais de Oliveira Costa; Ticiane Cavalcante de Souza; Veridiana Pessoa Miyajima
EPI_ISL_3912307, EPI_ISL_3912308, EPI_ISL_3912309	UNIDADE DE PRONTO ATENDIMENTO ARACATI UPA ARACATI	Analytical Competence Molecular Epidemiology Lab/ACME, Oswaldo Cruz Foundation, Ceara (FIOCRUZ CE)	Cleber Furtado Aksenen; Fabio Miyajima; Fernando Braga Stehling; Francisco Eder de Moura Lopes; Jamille Maria Mendes Bezerra; Joaquim Cesar do Nascimento Sousa Junior; Pedro Miguel Carneiro Jeronimo; Suzana Porto Almeida & Lucas Delerino on behalf of COVID-19 FIOCRUZ Genomic Network; Thais Ferreira de Oliveira; Thais de Oliveira Costa; Ticiane Cavalcante de Souza; Veridiana Pessoa Miyajima
EPI_ISL_3912311	UNIDADE DE PRONTO ATENDIMENTO DA JUREMA	Analytical Competence Molecular Epidemiology Lab/ACME, Oswaldo Cruz Foundation, Ceara (FIOCRUZ CE)	Cleber Furtado Aksenen; Fabio Miyajima; Fernando Braga Stehling; Francisco Eder de Moura Lopes; Jamille Maria Mendes Bezerra; Joaquim Cesar do Nascimento Sousa Junior; Pedro Miguel Carneiro Jeronimo; Suzana Porto Almeida & Lucas Delerino on behalf of COVID-19 FIOCRUZ Genomic Network; Thais Ferreira de Oliveira; Thais de Oliveira Costa; Ticiane Cavalcante de Souza; Veridiana Pessoa Miyajima
EPI_ISL_2462319, EPI_ISL_2494102, EPI_ISL_2494104, EPI_ISL_2494105, EPI_ISL_2494106, EPI_ISL_2494334	UNIDADE DE PRONTO ATENDIMENTO DE AGENOR DE CAMPOS	Instituto Butantan	Antonio Jorge Martins; Claudia Renata dos Santos Barros; David Schlesinger; Debora Botequio Moretti; Dimas Tadeu Covas; Elaine Cristina Marqueze; Elaine Vieira Santos; Evandra Strazza Rodrigues; Heidge Fukumasu; Jayme Augusto de Souza-Neto; José Salvatore Leister Patané; Luiz Alcantara; Luiz Lehmann Coutinho; Maria Carolina Elias; Maurício Lacerda Nogueira; Rafael dos Santos Bezerra; Raul Machado Neto; Rejane Maria Tommasini Grotto; Ricardo Haddad; Sandra Coccuzzo Sampaio Vessoni; Simone Kashima; Svetoslav Nanev Slavov; Vincent Louis Viala
EPI_ISL_3912314, EPI_ISL_3912315, EPI_ISL_3912316, EPI_ISL_3912317	UNIDADE DE PRONTO ATENDIMENTO DE EUSEBIO	Analytical Competence Molecular Epidemiology Lab/ACME, Oswaldo Cruz Foundation, Ceara (FIOCRUZ CE)	Cleber Furtado Aksenen; Fabio Miyajima; Fernando Braga Stehling; Francisco Eder de Moura Lopes; Jamille Maria Mendes Bezerra; Joaquim Cesar do Nascimento Sousa Junior; Pedro Miguel Carneiro Jeronimo; Suzana Porto Almeida & Lucas Delerino on behalf of COVID-19 FIOCRUZ Genomic Network; Thais Ferreira de Oliveira; Thais de Oliveira Costa; Ticiane Cavalcante de Souza; Veridiana Pessoa Miyajima
EPI_ISL_2493835	UNIDADE DE PRONTO ATENDIMENTO DE PITANGUEIRAS	Instituto Butantan	Antonio Jorge Martins; Claudia Renata dos Santos Barros; David Schlesinger; Debora Botequio Moretti; Dimas Tadeu Covas; Elaine Cristina Marqueze; Elaine Vieira Santos; Evandra Strazza Rodrigues; Heidge Fukumasu; Jayme Augusto de Souza-Neto; José Salvatore Leister Patané; Luiz Alcantara; Luiz Lehmann Coutinho; Maria Carolina Elias; Maurício Lacerda Nogueira; Rafael dos Santos Bezerra; Raul Machado Neto; Rejane Maria Tommasini Grotto; Ricardo Haddad; Sandra Coccuzzo Sampaio Vessoni; Simone Kashima; Svetoslav Nanev Slavov; Vincent Louis Viala
EPI_ISL_2493844	UNIDADE DE SAUDE DA FAMILIA JARDIM AMERICA ITAQUAQUECETUBA	Instituto Butantan	Antonio Jorge Martins; Claudia Renata dos Santos Barros; David Schlesinger; Debora Botequio Moretti; Dimas Tadeu Covas; Elaine Cristina Marqueze; Elaine Vieira Santos; Evandra Strazza Rodrigues; Heidge Fukumasu; Jayme Augusto de Souza-Neto; José Salvatore Leister Patané; Luiz Alcantara; Luiz Lehmann Coutinho; Maria Carolina Elias; Maurício Lacerda Nogueira; Rafael dos Santos Bezerra; Raul Machado Neto; Rejane Maria Tommasini Grotto; Ricardo Haddad; Sandra Coccuzzo Sampaio Vessoni; Simone Kashima; Svetoslav Nanev Slavov; Vincent Louis Viala
EPI_ISL_2494181	UNIDADE DE VIGILANCIA EPIDEMIOLOGICA	Instituto Butantan	Antonio Jorge Martins; Claudia Renata dos Santos Barros; David Schlesinger; Debora Botequio Moretti; Dimas Tadeu Covas; Elaine Cristina Marqueze; Elaine Vieira Santos; Evandra Strazza Rodrigues; Heidge Fukumasu; Jayme Augusto de Souza-Neto; José Salvatore Leister Patané; Luiz Alcantara; Luiz Lehmann Coutinho; Maria Carolina Elias; Maurício Lacerda Nogueira; Rafael dos Santos Bezerra; Raul Machado Neto; Rejane Maria Tommasini Grotto; Ricardo Haddad; Sandra Coccuzzo Sampaio Vessoni; Simone Kashima; Svetoslav Nanev Slavov; Vincent Louis Viala
EPI_ISL_2493947	UNIDADE MISTA ARACOIABA DA SERRA	Instituto Butantan	Antonio Jorge Martins; Claudia Renata dos Santos Barros; David Schlesinger; Debora Botequio Moretti; Dimas Tadeu Covas; Elaine Cristina Marqueze; Elaine Vieira Santos; Evandra Strazza Rodrigues; Heidge Fukumasu; Jayme Augusto de Souza-Neto; José Salvatore Leister Patané; Luiz Alcantara; Luiz Lehmann Coutinho; Maria Carolina Elias; Maurício Lacerda Nogueira; Rafael dos Santos Bezerra; Raul Machado Neto; Rejane Maria Tommasini Grotto; Ricardo Haddad; Sandra Coccuzzo Sampaio Vessoni; Simone Kashima; Svetoslav Nanev Slavov; Vincent Louis Viala
EPI_ISL_3912320	UNIDADE PRONTO ATENDIMENTO AUTRAN NUNES	Analytical Competence Molecular Epidemiology Lab/ACME, Oswaldo Cruz Foundation, Ceara (FIOCRUZ CE)	Cleber Furtado Aksenen; Fabio Miyajima; Fernando Braga Stehling; Francisco Eder de Moura Lopes; Jamille Maria Mendes Bezerra; Joaquim Cesar do Nascimento Sousa Junior; Pedro Miguel Carneiro Jeronimo; Suzana Porto Almeida & Lucas Delerino on behalf of COVID-19 FIOCRUZ Genomic Network; Thais Ferreira de Oliveira; Thais de Oliveira Costa; Ticiane Cavalcante de Souza; Veridiana Pessoa Miyajima
EPI_ISL_3102331	UNIDADE PRONTO ATENDIMENTO PRAIA DO FUTURO	Analytical Competence Molecular Epidemiology Lab/ACME, Oswaldo Cruz Foundation, Ceara (FIOCRUZ CE)	Cleber Furtado Aksenen; Fabio Miyajima; Fernando Braga Stehling; Francisco Eder de Moura Lopes; Jamille Maria Mendes Bezerra; Joaquim César do Nascimento Sousa Junior; Pedro Miguel Carneiro Jeronimo; Suzana Porto Almeida e Lucas Delerino; Thais Ferreira de Oliveira; Thais de Oliveira Costa; Ticiane Cavalcante de Souza; Veridiana Pessoa Miyajima
EPI_ISL_3102298, EPI_ISL_3536194, EPI_ISL_3536195, EPI_ISL_3536196, EPI_ISL_3536197, EPI_ISL_3536198, EPI_ISL_3536201, EPI_ISL_3912087, EPI_ISL_3912088, EPI_ISL_3912094, EPI_ISL_3912337, EPI_ISL_3912338, EPI_ISL_3912339, EPI_ISL_3912341, EPI_ISL_3912342, EPI_ISL_3912343, EPI_ISL_3912344, EPI_ISL_3912345, EPI_ISL_3912348, EPI_ISL_3912355, EPI_ISL_3912358, EPI_ISL_3912359, EPI_ISL_3912362, EPI_ISL_3912363, EPI_ISL_3912364, EPI_ISL_3912365, EPI_ISL_3912366, EPI_ISL_3912367, EPI_ISL_3912369, EPI_ISL_3912370, EPI_ISL_3912371, EPI_ISL_3912372, EPI_ISL_3912373, EPI_ISL_3912377, EPI_ISL_3912378, EPI_ISL_3912379			
see above	UNIDADE REFERENCIA COVID 19	Analytical Competence Molecular Epidemiology Lab/ACME, Oswaldo Cruz Foundation, Ceara (FIOCRUZ CE)	Cleber Furtado Aksenen; Fabio Miyajima; Fernando Braga Stehling; Francisco Eder de Moura Lopes; Jamille Maria Mendes Bezerra; Joaquim Cesar do Nascimento Sousa Junior; Pedro Miguel Carneiro Jeronimo; Suzana Porto Almeida & Lucas Delerino on behalf of COVID-19 FIOCRUZ Genomic Network; Suzana Porto Almeida e Lucas Delerino; Thais Ferreira de Oliveira; Thais de Oliveira Costa; Ticiane Cavalcante de Souza; Veridiana Pessoa Miyajima
EPI_ISL_2493897, EPI_ISL_2493912	UNIDADE SENTINELA COVID19	Instituto Butantan	Antonio Jorge Martins; Claudia Renata dos Santos Barros; David Schlesinger; Debora Botequio Moretti; Dimas Tadeu Covas; Elaine Cristina Marqueze; Elaine Vieira Santos; Evandra Strazza Rodrigues; Heidge Fukumasu; Jayme Augusto de Souza-Neto; José Salvatore Leister Patané; Luiz Alcantara; Luiz Lehmann Coutinho; Maria Carolina Elias; Maurício Lacerda Nogueira; Rafael dos Santos Bezerra; Raul Machado Neto; Rejane Maria Tommasini Grotto; Ricardo Haddad; Sandra Coccuzzo Sampaio Vessoni; Simone Kashima; Svetoslav Nanev Slavov; Vincent Louis Viala
EPI_ISL_2493907	UNIDADE SENTINELA VILA APARECIDA	Instituto Butantan	Antonio Jorge Martins; Claudia Renata dos Santos Barros; David Schlesinger; Debora Botequio Moretti; Dimas Tadeu Covas; Elaine Cristina Marqueze; Elaine Vieira Santos; Evandra Strazza Rodrigues; Heidge Fukumasu; Jayme Augusto de Souza-Neto; José Salvatore Leister Patané; Luiz Alcantara; Luiz Lehmann Coutinho; Maria Carolina Elias; Maurício Lacerda Nogueira; Rafael dos Santos Bezerra; Raul Machado Neto; Rejane Maria Tommasini Grotto; Ricardo Haddad; Sandra Coccuzzo Sampaio Vessoni; Simone Kashima; Svetoslav Nanev Slavov; Vincent Louis Viala
EPI_ISL_2628717, EPI_ISL_2628825, EPI_ISL_2628962, EPI_ISL_2645941, EPI_ISL_2645995, EPI_ISL_2646099, EPI_ISL_2796397			
see above	UNILABS	Instituto Nacional de Saude (INSA)	Borges et al
EPI_ISL_2796048	UNILABS	Instituto Nacional de Saude (INSA) and Instituto Gulbenkian de Ciencia (IGC)	Borges et al
EPI_ISL_2820961	UOC Microbiologia e Virologia, Azienda Ospedaliera Universitaria Senese, Siena, Italy	Dipartimento di Biotecnologie Mediche	Claudia Gandolfo; David Pinzauti; Francesco Santoro; Gabriele Anichini; Gianni Gori Savellini; Gianni Pozzi; Maria Grazia Cusi
EPI_ISL_2493816, EPI_ISL_2493848, EPI_ISL_2493852, EPI_ISL_2493858	UPA SADAKO SEDOGUTI	Instituto Butantan	Antonio Jorge Martins; Claudia Renata dos Santos Barros; David Schlesinger; Debora Botequio Moretti; Dimas Tadeu Covas; Elaine Cristina Marqueze; Elaine Vieira Santos; Evandra Strazza Rodrigues; Heidge Fukumasu; Jayme Augusto de Souza-Neto; José Salvatore Leister Patané; Luiz Alcantara; Luiz Lehmann Coutinho; Maria Carolina Elias; Maurício Lacerda Nogueira; Rafael dos Santos Bezerra; Raul Machado Neto; Rejane Maria Tommasini Grotto; Ricardo Haddad; Sandra Coccuzzo Sampaio Vessoni; Simone Kashima; Svetoslav Nanev Slavov; Vincent Louis Viala
EPI_ISL_2920237	URMC LABS	URMC LABS	Andrew Cameron; Dwight Hardy; Kelly DeLary; Stepan Voljehn
EPI_ISL_2868325, EPI_ISL_2868527, EPI_ISL_3031239	URMC LABS	Wadsworth Center, New York State Department of Health	Alexis Russell; Catharine Prussing; Daryl M. Lamson; Erasmus Schneider; Erica Lasek-Nesselquist; John Kelly; Jonathan Plitnick; Kirsten St. George; Matthew Shudt; Melissa A Leisner; Navjot Singh
EPI_ISL_3048152, EPI_ISL_3048160	US Air Force School of Aerospace Medicine	US Air Force School of Aerospace Medicine	Amanda Javorina; Anthony Fries; Carol Garrett; Clarise Starr; Elizabeth Macias; Jennifer Meyer; Sarah Purves; William Gruner
EPI_ISL_2493879	USAFA MARACANA	Instituto Butantan	Antonio Jorge Martins; Claudia Renata dos Santos Barros; David Schlesinger; Debora Botequio Moretti; Dimas Tadeu Covas; Elaine Cristina Marqueze; Elaine Vieira Santos; Evandra Strazza Rodrigues; Heidge Fukumasu; Jayme Augusto de Souza-Neto; José Salvatore Leister Patané; Luiz Alcantara; Luiz Lehmann Coutinho; Maria Carolina Elias; Maurício Lacerda Nogueira; Rafael dos Santos Bezerra; Raul Machado Neto; Rejane Maria Tommasini Grotto; Ricardo Haddad; Sandra Coccuzzo Sampaio Vessoni; Simone Kashima; Svetoslav Nanev Slavov; Vincent Louis Viala
EPI_ISL_2493895	USAFA MIRIM	Instituto Butantan	Antonio Jorge Martins; Claudia Renata dos Santos Barros; David Schlesinger; Debora Botequio Moretti; Dimas Tadeu Covas; Elaine Cristina Marqueze; Elaine Vieira Santos; Evandra Strazza Rodrigues; Heidge Fukumasu; Jayme Augusto de Souza-Neto; José Salvatore Leister Patané; Luiz Alcantara; Luiz Lehmann Coutinho; Maria Carolina Elias; Maurício Lacerda Nogueira; Rafael dos Santos Bezerra; Raul Machado Neto; Rejane Maria Tommasini Grotto; Ricardo Haddad; Sandra Coccuzzo Sampaio Vessoni; Simone Kashima; Svetoslav Nanev Slavov; Vincent Louis Viala
EPI_ISL_2493831	USAFA SAO JORGE	Instituto Butantan	Antonio Jorge Martins; Claudia Renata dos Santos Barros; David Schlesinger; Debora Botequio Moretti; Dimas Tadeu Covas; Elaine Cristina Marqueze; Elaine Vieira Santos; Evandra Strazza Rodrigues; Heidge Fukumasu; Jayme Augusto de Souza-Neto; José Salvatore Leister Patané; Luiz Alcantara; Luiz Lehmann Coutinho; Maria Carolina Elias; Maurício Lacerda Nogueira; Rafael dos Santos Bezerra; Raul Machado Neto; Rejane Maria Tommasini Grotto; Ricardo Haddad; Sandra Coccuzzo Sampaio Vessoni; Simone Kashima; Svetoslav Nanev Slavov; Vincent Louis Viala
EPI_ISL_2493801	USF JARDIM AMERICA FIGUEIRA	Instituto Butantan	Antonio Jorge Martins; Claudia Renata dos Santos Barros; David Schlesinger; Debora Botequio Moretti; Dimas Tadeu Covas; Elaine Cristina Marqueze; Elaine Vieira Santos; Evandra Strazza Rodrigues; Heidge Fukumasu; Jayme Augusto de Souza-Neto; José Salvatore Leister Patané; Luiz Alcantara; Luiz Lehmann Coutinho; Maria Carolina Elias; Maurício Lacerda Nogueira; Rafael dos Santos Bezerra; Raul Machado Neto; Rejane Maria Tommasini Grotto; Ricardo Haddad; Sandra Coccuzzo Sampaio Vessoni; Simone Kashima; Svetoslav Nanev Slavov; Vincent Louis Viala
EPI_ISL_2493811, EPI_ISL_2493814, EPI_ISL_2493817	USF JARDIM CAMANDUCAIA	Instituto Butantan	Antonio Jorge Martins; Claudia Renata dos Santos Barros; David Schlesinger; Debora Botequio Moretti; Dimas Tadeu Covas; Elaine Cristina Marqueze; Elaine Vieira Santos; Evandra Strazza Rodrigues; Heidge Fukumasu; Jayme Augusto de Souza-Neto; José Salvatore Leister Patané; Luiz Alcantara; Luiz Lehmann Coutinho; Maria Carolina Elias; Maurício Lacerda Nogueira; Rafael dos Santos Bezerra; Raul Machado Neto; Rejane Maria Tommasini Grotto; Ricardo Haddad; Sandra Coccuzzo Sampaio Vessoni; Simone Kashima; Svetoslav Nanev Slavov; Vincent Louis Viala
EPI_ISL_2493799, EPI_ISL_2493810,	USF JARDIM DAS AVES MOREIRINHA	Instituto Butantan	Antonio Jorge Martins; Claudia Renata dos Santos Barros; David Schlesinger; Debora Botequio Moretti; Dimas Tadeu Covas; Elaine Cristina Marqueze; Elaine Vieira Santos; Evandra Strazza Rodrigues; Heidge Fukumasu; Jayme Augusto de Souza-Neto; José Salvatore Leister Patané; Luiz Alcantara; Luiz Lehmann Coutinho; Maria Carolina Elias; Maurício Lacerda Nogueira; Rafael dos Santos Bezerra; Raul Machado Neto; Rejane Maria Tommasini Grotto; Ricardo Haddad; Sandra Coccuzzo Sampaio Vessoni; Simone Kashima; Svetoslav Nanev Slavov; Vincent Louis Viala

EPI_ISL_2493812, EPI_ISL_2493813			
EPI_ISL_2493819	USF JARDIM JOSELY	Instituto Butantan	Antonio Jorge Martins; Claudia Renata dos Santos Barros; David Schlesinger; Debora Botequio Moretti; Dimas Tadeu Covas; Elaine Cristina Marqueze; Elaine Vieira Santos; Evandra Strazza Rodrigues; Heidge Fukumasu; Jayme Augusto de Souza-Neto; José Salvatore Leister Patané; Luiz Alcantara; Luiz Lehmann Coutinho; Maria Carolina Elias; Maurício Lacerda Nogueira; Rafael dos Santos Bezerra; Raul Machado Neto; Rejane Maria Tommasini Grotto; Ricardo Haddad; Sandra Coccuzzo Sampaio Vessoni; Simone Kashima; Svetoslav Nanev Slavov; Vincent Louis Viala
EPI_ISL_2493798, EPI_ISL_2493809	USF JARDIM SAO DIMAS	Instituto Butantan	Antonio Jorge Martins; Claudia Renata dos Santos Barros; David Schlesinger; Debora Botequio Moretti; Dimas Tadeu Covas; Elaine Cristina Marqueze; Elaine Vieira Santos; Evandra Strazza Rodrigues; Heidge Fukumasu; Jayme Augusto de Souza-Neto; José Salvatore Leister Patané; Luiz Alcantara; Luiz Lehmann Coutinho; Maria Carolina Elias; Maurício Lacerda Nogueira; Rafael dos Santos Bezerra; Raul Machado Neto; Rejane Maria Tommasini Grotto; Ricardo Haddad; Sandra Coccuzzo Sampaio Vessoni; Simone Kashima; Svetoslav Nanev Slavov; Vincent Louis Viala
EPI_ISL_2493815	USF JARDIM SILVESTRE II	Instituto Butantan	Antonio Jorge Martins; Claudia Renata dos Santos Barros; David Schlesinger; Debora Botequio Moretti; Dimas Tadeu Covas; Elaine Cristina Marqueze; Elaine Vieira Santos; Evandra Strazza Rodrigues; Heidge Fukumasu; Jayme Augusto de Souza-Neto; José Salvatore Leister Patané; Luiz Alcantara; Luiz Lehmann Coutinho; Maria Carolina Elias; Maurício Lacerda Nogueira; Rafael dos Santos Bezerra; Raul Machado Neto; Rejane Maria Tommasini Grotto; Ricardo Haddad; Sandra Coccuzzo Sampaio Vessoni; Simone Kashima; Svetoslav Nanev Slavov; Vincent Louis Viala
EPI_ISL_2493818	USF SANTA MARIA DO AMPARO	Instituto Butantan	Antonio Jorge Martins; Claudia Renata dos Santos Barros; David Schlesinger; Debora Botequio Moretti; Dimas Tadeu Covas; Elaine Cristina Marqueze; Elaine Vieira Santos; Evandra Strazza Rodrigues; Heidge Fukumasu; Jayme Augusto de Souza-Neto; José Salvatore Leister Patané; Luiz Alcantara; Luiz Lehmann Coutinho; Maria Carolina Elias; Maurício Lacerda Nogueira; Rafael dos Santos Bezerra; Raul Machado Neto; Rejane Maria Tommasini Grotto; Ricardo Haddad; Sandra Coccuzzo Sampaio Vessoni; Simone Kashima; Svetoslav Nanev Slavov; Vincent Louis Viala
EPI_ISL_2494145	USF SERROTE REGISTRO	Instituto Butantan	Antonio Jorge Martins; Claudia Renata dos Santos Barros; David Schlesinger; Debora Botequio Moretti; Dimas Tadeu Covas; Elaine Cristina Marqueze; Elaine Vieira Santos; Evandra Strazza Rodrigues; Heidge Fukumasu; Jayme Augusto de Souza-Neto; José Salvatore Leister Patané; Luiz Alcantara; Luiz Lehmann Coutinho; Maria Carolina Elias; Maurício Lacerda Nogueira; Rafael dos Santos Bezerra; Raul Machado Neto; Rejane Maria Tommasini Grotto; Ricardo Haddad; Sandra Coccuzzo Sampaio Vessoni; Simone Kashima; Svetoslav Nanev Slavov; Vincent Louis Viala
EPI_ISL_2493800	USF TRES PONTES	Instituto Butantan	Antonio Jorge Martins; Claudia Renata dos Santos Barros; David Schlesinger; Debora Botequio Moretti; Dimas Tadeu Covas; Elaine Cristina Marqueze; Elaine Vieira Santos; Evandra Strazza Rodrigues; Heidge Fukumasu; Jayme Augusto de Souza-Neto; José Salvatore Leister Patané; Luiz Alcantara; Luiz Lehmann Coutinho; Maria Carolina Elias; Maurício Lacerda Nogueira; Rafael dos Santos Bezerra; Raul Machado Neto; Rejane Maria Tommasini Grotto; Ricardo Haddad; Sandra Coccuzzo Sampaio Vessoni; Simone Kashima; Svetoslav Nanev Slavov; Vincent Louis Viala
EPI_ISL_2493914	USF YEN WANN SHENG	Instituto Butantan	Antonio Jorge Martins; Claudia Renata dos Santos Barros; David Schlesinger; Debora Botequio Moretti; Dimas Tadeu Covas; Elaine Cristina Marqueze; Elaine Vieira Santos; Evandra Strazza Rodrigues; Heidge Fukumasu; Jayme Augusto de Souza-Neto; José Salvatore Leister Patané; Luiz Alcantara; Luiz Lehmann Coutinho; Maria Carolina Elias; Maurício Lacerda Nogueira; Rafael dos Santos Bezerra; Raul Machado Neto; Rejane Maria Tommasini Grotto; Ricardo Haddad; Sandra Coccuzzo Sampaio Vessoni; Simone Kashima; Svetoslav Nanev Slavov; Vincent Louis Viala
EPI_ISL_2432602, EPI_ISL_2432619, EPI_ISL_2497213, EPI_ISL_2497214, EPI_ISL_2497215, EPI_ISL_2497216, EPI_ISL_2497221, EPI_ISL_2497232, EPI_ISL_2497247, EPI_ISL_2497263, EPI_ISL_2497268, EPI_ISL_2497287, EPI_ISL_2497292, EPI_ISL_2497299, EPI_ISL_2497303, EPI_ISL_2497310, EPI_ISL_2497325, EPI_ISL_2497338, EPI_ISL_2497356, EPI_ISL_2497365, EPI_ISL_2497383, EPI_ISL_2497392, EPI_ISL_2497396, EPI_ISL_2497932, EPI_ISL_2497933, EPI_ISL_2497934, EPI_ISL_2497935, EPI_ISL_2497936, EPI_ISL_2497937, EPI_ISL_2497938, EPI_ISL_2497939, EPI_ISL_2497940, EPI_ISL_2501473, EPI_ISL_2501477, EPI_ISL_2501481, EPI_ISL_2501482, EPI_ISL_2501483, EPI_ISL_2501487, EPI_ISL_2501489, EPI_ISL_2501490, EPI_ISL_2501491, EPI_ISL_2501492, EPI_ISL_2501493, EPI_ISL_2501494, EPI_ISL_2545020, EPI_ISL_2545075, EPI_ISL_2545107, EPI_ISL_2545119, EPI_ISL_2545123, EPI_ISL_2545153, EPI_ISL_2545154, EPI_ISL_2545155, EPI_ISL_2545157, EPI_ISL_2545159, EPI_ISL_2545160, EPI_ISL_2545161, EPI_ISL_2545162, EPI_ISL_2545163, EPI_ISL_2545165, EPI_ISL_2545166, EPI_ISL_2545167, EPI_ISL_2545168, EPI_ISL_2545169, EPI_ISL_2545170, EPI_ISL_2545171, EPI_ISL_2545172, EPI_ISL_2545173, EPI_ISL_2545174, EPI_ISL_2545175, EPI_ISL_2545176, EPI_ISL_2545177, EPI_ISL_2549343, EPI_ISL_2549346, EPI_ISL_2549354, EPI_ISL_2549382, EPI_ISL_2549399, EPI_ISL_2549432, EPI_ISL_2549447, EPI_ISL_2549458, EPI_ISL_2549471, EPI_ISL_2549478, EPI_ISL_2549495, EPI_ISL_2549496, EPI_ISL_2549504, EPI_ISL_2549519, EPI_ISL_2549520, EPI_ISL_2549523, EPI_ISL_2549532, EPI_ISL_2549540, EPI_ISL_2549543, EPI_ISL_2549548, EPI_ISL_2549553, EPI_ISL_2549555, EPI_ISL_2549556, EPI_ISL_2549557, EPI_ISL_2549558, EPI_ISL_2549559, EPI_ISL_2617628, EPI_ISL_2617970, EPI_ISL_2617975, EPI_ISL_2617987, EPI_ISL_2617992, EPI_ISL_2618002, EPI_ISL_2618005, EPI_ISL_2618023, EPI_ISL_2618038, EPI_ISL_2618049, EPI_ISL_2618053, EPI_ISL_2618056, EPI_ISL_2618088, EPI_ISL_2693116, EPI_ISL_2693129, EPI_ISL_2693180, EPI_ISL_2693253, EPI_ISL_2693260, EPI_ISL_2693278, EPI_ISL_2693299, EPI_ISL_2693684, EPI_ISL_2693746, EPI_ISL_2693781, EPI_ISL_2693801, EPI_ISL_2693813, EPI_ISL_2716479, EPI_ISL_2716488, EPI_ISL_2716734, EPI_ISL_2802896, EPI_ISL_2802923, EPI_ISL_2802937, EPI_ISL_2802944, EPI_ISL_2802955, EPI_ISL_2802959, EPI_ISL_2802990, EPI_ISL_2802995, EPI_ISL_2802998, EPI_ISL_2803005, EPI_ISL_2803309, EPI_ISL_2864531, EPI_ISL_2864533, EPI_ISL_2864534, EPI_ISL_2864537, EPI_ISL_2895012, EPI_ISL_2937933, EPI_ISL_2967614, EPI_ISL_2967625, EPI_ISL_2967658, EPI_ISL_2967691, EPI_ISL_2967692, EPI_ISL_2967707, EPI_ISL_2967725, EPI_ISL_3025927, EPI_ISL_3025931, EPI_ISL_3025955, EPI_ISL_3025960, EPI_ISL_3032167, EPI_ISL_3063560, EPI_ISL_3063563, EPI_ISL_3063607, EPI_ISL_3063609, EPI_ISL_3063657, EPI_ISL_3063684			
see above	UW Virology Lab	UW Virology Lab	Alexander Greninger; Hong Xie; Keith R Jerome; Lasata Shrestha; Maria Lukes; Meei-Li Huang; Michelle Lin; Nathan Breit; Noah R. Baker; Patrick Mathias; Pavitra Roychoudhury; Ricardo Perez; Robert J. Livingston; Sean Ellis; Shah Mohamed Bakhash; Tien V. Nguyen
EPI_ISL_2942330	Unidad de Investigacion Medica de Yucatan (UIMY)	Unidad de Genomica Avanzada	; Alejandra Garcia-Gasca; Alejandra Hernandez-Teran; Alejandro Sanchez-Flores; Alfredo Herrera-Estrella; Alicia Ocaña-Mondragon; Andreu Comas-Garcia; Angel Gustavo Salas-Lais; Antonio Loza Roman; Bernardo Martinez-Miguel; Blanca Taboada; Brenda Irasema Maldonado-Meza; Bruno Gomez-Gil; Carla Ivon Herrera-Najera; Carlos F. Arias; Celia Boukadida; Celida Duque Molina; Celida Martinez- Rodriguez; Clara Esperanza Santacruz-Tinoco; Concepcion Grajales-Muñiz; Consorcio Mexicano de Vigilancia Genomica (CoViGen-Mex). Authors (in alphabetical order): Julio Elias Alvarado-Yaah; Cristobal Chaidez-Quiroz; Daniel Fregoso-Rueda; Daniel Lira Morales; Eduardo Becerril-Vargas; Fernando Fontove-Herrera; Fidencio Mejia-Nepomuceno; Francisco Pulido; Gloria Elena Espinosa-Ayala; Gloria Maria Molina-Salinas; Gloria Vazquez; Hector Esteban Paz-Juarez; Hector Montoya-Fuentes; Helen Haydee Fernanda Ramirez-Plascencia; Irvin Gonzalez-Lopez; Jean Pierre Gonzalez; Jesus Hernandez; Joel Armando Vazquez-Perez.; Jorge Salas-Hernandez; Jose Antonio Enciso-Moreno; Jose Arturo Martinez-Orozco; Jose Esteban Muñoz-Medina; Jose de Jesus Nuñez-Contreras; Juan Bautista Chale-Dzul; Julissa Enciso-Ibarra; Luis Alberto Ochoa-Carrera; Margarita Matias-Florentino; Maria Guadalupe Santiago-Mauricio; Maria Guadalupe de Jesus Mireles-Rivera; Mario Mujica-Sanchez; Marissa Perez-Garcia; Nelly Selem-Mojica; Pavel Isa; Ricardo Ciria Merce; Ricardo Grande; Rosa Maria Gutierrez Rios; Santiago avila-Rios; Selene Zarate; Susana Lopez; Veronica Mata-Haro; Victor Eduardo Garcia-Arias; Victor Hugo Borja-Aburto
EPI_ISL_2681209	Unidad de Investigación Biomédica de Zacatecas (UIBZ)	Instituto de Biotecnología de la UNAM	; Alejandra García-Gasca; Alejandra Hernández-Terán; Alejandro Sánchez-Flores; Alfredo Herrera-Estrella; Alicia Ocaña-Mondragón; Andreu Comas-García; Angel Gustavo Salas-Lais; Antonio Loza Román; Bernardo Martínez-Miguel; Blanca Taboada; Brenda Irasema Maldonado-Meza; Bruno Gómez-Gil; Carla Ivón Herrera-Najera; Carlos F. Arias; Celia Boukadida; Clara Esperanza Santacruz-Tinoco; Concepción Grajales-Muñiz; Consorcio Mexicano de Vigilancia Genómica (CoViGen-Mex). Authors (in alphabetical order): Julio Elias Alvarado-Yaah; Cristóbal Cháidez-Quiróz; Célida Duque Molina; Célida Martínez- Rodríguez; Daniel Fregoso-Rueda; Daniel Lira Morales; Eduardo Becerril-Vargas; Fernando Fontove-Herrera; Fidencio Mejía-Nepomuceno; Francisco Pulido; Gloria Elena Espinosa-Ayala; Gloria María Molina-Salinas; Gloria Vazquez; Hector Esteban Paz-Juárez; Hector Montoya-Fuentes; Helen Haydee Fernanda Ramírez-Plascencia; Irvin González-López; Jean Pierre González; Jesús Hernández; Joel Armando Vázquez-Pérez.; Jorge Salas-Hernández; José Antonio Enciso-Moreno; José Arturo Martínez-Orozco; José Esteban Muñoz-Medina; José de Jesús Nuñez-Contreras; Juan Bautista Chale-Dzul; Julissa Enciso-Ibarra; Luis Alberto Ochoa-Carrera; Margarita Matías-Florentino; Mario Mújica-Sánchez; Marissa Perez-Garcia; María Guadalupe Santiago-Mauricio; María Guadalupe de Jesús Mireles-Rivera; Nelly Sélem-Mojica; Pavel Isa; Ricardo Ciria Merce; Ricardo Grande; Rosa María Gutiérrez Rios; Santiago Ávila-Ríos; Selene Zárate; Susana Lopez; Verónica Mata-Haro; Victor Eduardo García-Arias; Víctor Hugo Borja-Aburto
EPI_ISL_2681055, EPI_ISL_2681090, EPI_ISL_2681141, EPI_ISL_2681244, EPI_ISL_2801588, EPI_ISL_2801617, EPI_ISL_2801642, EPI_ISL_2801656, EPI_ISL_2801657			
see above	Unidad de Investigación Médica de Yucatán (UIMY)	Instituto de Biotecnología de la UNAM	; Alejandra García-Gasca; Alejandra Hernández-Terán; Alejandro Sánchez-Flores; Alfredo Herrera-Estrella; Alicia Ocaña-Mondragón; Andreu Comas-García; Angel Gustavo Salas-Lais; Antonio Loza Román; Bernardo Martínez-Miguel; Blanca Taboada; Brenda Irasema Maldonado-Meza; Bruno Gómez-Gil; Carla Ivón Herrera-Najera; Carlos F. Arias; Celia Boukadida; Clara Esperanza Santacruz-Tinoco; Concepción Grajales-Muñiz; Consorcio Mexicano de Vigilancia Genómica (CoViGen-Mex). Authors (in alphabetical order): Julio Elias Alvarado-Yaah; Cristóbal Cháidez-Quiróz; Célida Duque Molina; Célida Martínez- Rodríguez; Daniel Fregoso-Rueda; Daniel Lira Morales; Eduardo Becerril-Vargas; Fernando Fontove-Herrera; Fidencio Mejía-Nepomuceno; Francisco Pulido; Gloria Elena Espinosa-Ayala; Gloria María Molina-Salinas; Gloria Vazquez; Hector Esteban Paz-Juárez; Hector Montoya-Fuentes; Helen Haydee Fernanda Ramírez-Plascencia; Irvin González-López; Jean Pierre González; Jesús Hernández; Joel Armando Vázquez-Pérez.; Jorge Salas-Hernández; José Antonio Enciso-Moreno; José Arturo Martínez-Orozco; José Esteban Muñoz-Medina; José de Jesús Nuñez-Contreras; Juan Bautista Chale-Dzul; Julissa Enciso-Ibarra; Luis Alberto Ochoa-Carrera; Margarita Matías-Florentino; Mario Mújica-Sánchez; Marissa Perez-Garcia; María Guadalupe Santiago-Mauricio; María Guadalupe de Jesús Mireles-Rivera; Nelly Sélem-Mojica; Pavel Isa; Ricardo Ciria Merce; Ricardo Grande; Rosa María Gutiérrez Rios; Santiago Ávila-Ríos; Selene Zárate; Susana Lopez; Verónica Mata-Haro; Victor Eduardo García-Arias; Víctor Hugo Borja-Aburto
EPI_ISL_2691569, EPI_ISL_2691570, EPI_ISL_2691571, EPI_ISL_2691572, EPI_ISL_2691574, EPI_ISL_2691575, EPI_ISL_2691576, EPI_ISL_2691577, EPI_ISL_2691578, EPI_ISL_2691579, EPI_ISL_2691581, EPI_ISL_2691582, EPI_ISL_2691583, EPI_ISL_2691584, EPI_ISL_2691586, EPI_ISL_2691587, EPI_ISL_2691590, EPI_ISL_2691591, EPI_ISL_2691592, EPI_ISL_2691593, EPI_ISL_2691594, EPI_ISL_2691595, EPI_ISL_2691596, EPI_ISL_2691598, EPI_ISL_2691599, EPI_ISL_2691600, EPI_ISL_2691601, EPI_ISL_2691602, EPI_ISL_2691603, EPI_ISL_2691604, EPI_ISL_2691605, EPI_ISL_2691606, EPI_ISL_2691607, EPI_ISL_2691608, EPI_ISL_2691609, EPI_ISL_2691610, EPI_ISL_2691611, EPI_ISL_2691612, EPI_ISL_2691613, EPI_ISL_2691614, EPI_ISL_2691615, EPI_ISL_2691616, EPI_ISL_2691617, EPI_ISL_2691618, EPI_ISL_2691619, EPI_ISL_2691620, EPI_ISL_2691621, EPI_ISL_2691622, EPI_ISL_2691628, EPI_ISL_2691629, EPI_ISL_2691630, EPI_ISL_2691631, EPI_ISL_2691633, EPI_ISL_2691635, EPI_ISL_2691636, EPI_ISL_2691638, EPI_ISL_2691639, EPI_ISL_2691640, EPI_ISL_2691642, EPI_ISL_2691643, EPI_ISL_2691644, EPI_ISL_2691647, EPI_ISL_2691648, EPI_ISL_2691649, EPI_ISL_2691650, EPI_ISL_2691651, EPI_ISL_2691652, EPI_ISL_2691653, EPI_ISL_2691655, EPI_ISL_2691657, EPI_ISL_2691658, EPI_ISL_2691659, EPI_ISL_2691660, EPI_ISL_2691661, EPI_ISL_2691662, EPI_ISL_2691664, EPI_ISL_2691665, EPI_ISL_2691666, EPI_ISL_2691667, EPI_ISL_2691668, EPI_ISL_2691669, EPI_ISL_2691670, EPI_ISL_2691671, EPI_ISL_2691672, EPI_ISL_2691673, EPI_ISL_2691674, EPI_ISL_2691677, EPI_ISL_2691678, EPI_ISL_2691679, EPI_ISL_2691680, EPI_ISL_2691681, EPI_ISL_2691682, EPI_ISL_2691683, EPI_ISL_2691684, EPI_ISL_2837208, EPI_ISL_2837209, EPI_ISL_2837210, EPI_ISL_2837211, EPI_ISL_2837213, EPI_ISL_2837214, EPI_ISL_2837215, EPI_ISL_2837216, EPI_ISL_2837217, EPI_ISL_2837218, EPI_ISL_2837219, EPI_ISL_2837221, EPI_ISL_2837222, EPI_ISL_2837223, EPI_ISL_2837224, EPI_ISL_2837225, EPI_ISL_2837226, EPI_ISL_2837227, EPI_ISL_2837228, EPI_ISL_2837229, EPI_ISL_2837231, EPI_ISL_2837232, EPI_ISL_2837233, EPI_ISL_2837234, EPI_ISL_2837235, EPI_ISL_2837236, EPI_ISL_2837238, EPI_ISL_2837239, EPI_ISL_2837240, EPI_ISL_2837241, EPI_ISL_2837242, EPI_ISL_2837244, EPI_ISL_2837245, EPI_ISL_2837249, EPI_ISL_2837250, EPI_ISL_2837252, EPI_ISL_2837253, EPI_ISL_2837254, EPI_ISL_2837255, EPI_ISL_2837256, EPI_ISL_2837257, EPI_ISL_2837258, EPI_ISL_2837259, EPI_ISL_2837260, EPI_ISL_2837261, EPI_ISL_2837262, EPI_ISL_2837264, EPI_ISL_2837265, EPI_ISL_2837267, EPI_ISL_2837268, EPI_ISL_2837269, EPI_ISL_2837270, EPI_ISL_2837272, EPI_ISL_2837273, EPI_ISL_2837274, EPI_ISL_2837275, EPI_ISL_2837276, EPI_ISL_2837277, EPI_ISL_2837279, EPI_ISL_2837280, EPI_ISL_2837282, EPI_ISL_2837283, EPI_ISL_2837284, EPI_ISL_2837285, EPI_ISL_2837287, EPI_ISL_2837288, EPI_ISL_2837289, EPI_ISL_2837290, EPI_ISL_2837291, EPI_ISL_2837292, EPI_ISL_2837295, EPI_ISL_2837298, EPI_ISL_2837300, EPI_ISL_2837301, EPI_ISL_2837302, EPI_ISL_2837303, EPI_ISL_2837304, EPI_ISL_2837305, EPI_ISL_2837306, EPI_ISL_2837307, EPI_ISL_2837308, EPI_ISL_2837309, EPI_ISL_2837310, EPI_ISL_2837312, EPI_ISL_2837313, EPI_ISL_2837314, EPI_ISL_3072149, EPI_ISL_3072151, EPI_ISL_3072152, EPI_ISL_3072153, EPI_ISL_3072155, EPI_ISL_3072158, EPI_ISL_3072159, EPI_ISL_3072160, EPI_ISL_3072161, EPI_ISL_3072163, EPI_ISL_3072166, EPI_ISL_3072167, EPI_ISL_3072168, EPI_ISL_3072170, EPI_ISL_3072171, EPI_ISL_3072172, EPI_ISL_3072173, EPI_ISL_3072174, EPI_ISL_3072177, EPI_ISL_3072178, EPI_ISL_3072179, EPI_ISL_3072180, EPI_ISL_3072181, EPI_ISL_3072185, EPI_ISL_3072186, EPI_ISL_3072187, EPI_ISL_3072188, EPI_ISL_3072189, EPI_ISL_3072191, EPI_ISL_3072192, EPI_ISL_3072193, EPI_ISL_3072194, EPI_ISL_3072196, EPI_ISL_3072198, EPI_ISL_3072199, EPI_ISL_3072200, EPI_ISL_3072202, EPI_ISL_3072203, EPI_ISL_3072204, EPI_ISL_3072205, EPI_ISL_3072206, EPI_ISL_3072207, EPI_ISL_3072208, EPI_ISL_3072209, EPI_ISL_3072210, EPI_ISL_3072214, EPI_ISL_3072221, EPI_ISL_3072223, EPI_ISL_3072224, EPI_ISL_3072225, EPI_ISL_3072226, EPI_ISL_3072227, EPI_ISL_3072228, EPI_ISL_3072229, EPI_ISL_3072230, EPI_ISL_3072232, EPI_ISL_3072233, EPI_ISL_3072234, EPI_ISL_3072235, EPI_ISL_3072236, EPI_ISL_3072237, EPI_ISL_3072238, EPI_ISL_3072239, EPI_ISL_3072240, EPI_ISL_3072241, EPI_ISL_3072242, EPI_ISL_3072244, EPI_ISL_3072245, EPI_ISL_3072246, EPI_ISL_3072247, EPI_ISL_3072248, EPI_ISL_3072249, EPI_ISL_3072251, EPI_ISL_3072253, EPI_ISL_3072254, EPI_ISL_3072255, EPI_ISL_3072257, EPI_ISL_3072258, EPI_ISL_3072261, EPI_ISL_3072263, EPI_ISL_3072265, EPI_ISL_3072266, EPI_ISL_3072267, EPI_ISL_3072268, EPI_ISL_3072270, EPI_ISL_3072271, EPI_ISL_3072272, EPI_ISL_3072273, EPI_ISL_3072274, EPI_ISL_3072276, EPI_ISL_3072277, EPI_ISL_3072278, EPI_ISL_3072281, EPI_ISL_3072282, EPI_ISL_3072283, EPI_ISL_3072285, EPI_ISL_3072286, EPI_ISL_3072288, EPI_ISL_3072289, EPI_ISL_3072290, EPI_ISL_3072292, EPI_ISL_3072294, EPI_ISL_3072297, EPI_ISL_3072299, EPI_ISL_3072300, EPI_ISL_3072302, EPI_ISL_3072306, EPI_ISL_3072308, EPI_ISL_3072309, EPI_ISL_3072311, EPI_ISL_3072312, EPI_ISL_3072313, EPI_ISL_3072314, EPI_ISL_3072315, EPI_ISL_3072316, EPI_ISL_3072317, EPI_ISL_3072318, EPI_ISL_3072319, EPI_ISL_3072320, EPI_ISL_3072322, EPI_ISL_3072323, EPI_ISL_3072324, EPI_ISL_3072327, EPI_ISL_3072329, EPI_ISL_3072331, EPI_ISL_3072333, EPI_ISL_3072335, EPI_ISL_3072337, EPI_ISL_3072341, EPI_ISL_3072343, EPI_ISL_3072347, EPI_ISL_3072359, EPI_ISL_3072366, EPI_ISL_3072370, EPI_ISL_3072374, EPI_ISL_3072378, EPI_ISL_3072394, EPI_ISL_3072396, EPI_ISL_3072399, EPI_ISL_3072401, EPI_ISL_3072403, EPI_ISL_3072409, EPI_ISL_3072411, EPI_ISL_3072415, EPI_ISL_3072417, EPI_ISL_3072423, EPI_ISL_3072425, EPI_ISL_3072427, EPI_ISL_3072431, EPI_ISL_3072434, EPI_ISL_3072436, EPI_ISL_3072438, EPI_ISL_3072440, EPI_ISL_3072444, EPI_ISL_3072448, EPI_ISL_3072450, EPI_ISL_3072452, EPI_ISL_3072454, EPI_ISL_3072456, EPI_ISL_3072458, EPI_ISL_3072462, EPI_ISL_3072463, EPI_ISL_3072465, EPI_ISL_3072467, EPI_ISL_3072469, EPI_ISL_3072475, EPI_ISL_3072477, EPI_ISL_3072479, EPI_ISL_3072481, EPI_ISL_3072483, EPI_ISL_3072485, EPI_ISL_3072487, EPI_ISL_3072491, EPI_ISL_3072494, EPI_ISL_3072497, EPI_ISL_3072501, EPI_ISL_3072507, EPI_ISL_3072509, EPI_ISL_3072513, EPI_ISL_3072519, EPI_ISL_3072521, EPI_ISL_3072525, EPI_ISL_3072531, EPI_ISL_3072535, EPI_ISL_3072543, EPI_ISL_3072547, EPI_ISL_3072551, EPI_ISL_3072555, EPI_ISL_3072559, EPI_ISL_3072563, EPI_ISL_3072565, EPI_ISL_3072571, EPI_ISL_3072575, EPI_ISL_3072577, EPI_ISL_3072579, EPI_ISL_3072581, EPI_ISL_3072584, EPI_ISL_3072586, EPI_ISL_3072590, EPI_ISL_3072592, EPI_ISL_3072595, EPI_ISL_3072597, EPI_ISL_3072598, EPI_ISL_3072604, EPI_ISL_3072606, EPI_ISL_3072612, EPI_ISL_3072614, EPI_ISL_3072622, EPI_ISL_3072626, EPI_ISL_3245362, EPI_ISL_3245383, EPI_ISL_3245395, EPI_ISL_3245396, EPI_ISL_3245409, EPI_ISL_3245411			
see above	Unidade de apoio ao diagnostico da COVID - UNADIG	Bioinformatics Laboratory / LNCC	Alessandra P Lamarca; Alexandra L Gerber; Amilcar Tanuri; Ana Paula de C Guimaraes; Ana Tereza R Vasconcelos; Andrea Cony Cavalcanti; Caio Luiz Pereira Ribeiro; Cassia Alves; Cintia Policarpo; Claudia Maria Braga de Mello; Cristiane Gomes da Silva; Diana Mariani; Douglas Terra Machado; Erica Ramos dos Santos Nascimento; Fernanda Leitao dos Santos; Flavio Dias da Silva; Gleidson da Silva de Oliveira; Leandro Magalhaes de Souza; Liliane Cavalcante; Luiz G P de Almeida; Marcio Henrique de Oliveira Garcia; Mario Sergio Ribeiro; Ricardo Jose Barbosa Salviano; Ronaldo da Silva F Jr; Silvia Carvalho
EPI_ISL_2544253	Unilabs	Institute of Medical Virology	Alexandra Trkola; Annette Audigé; Cyril Shah; Gabriela Ziltener; Guido Bloemberg; Jon Huder; Jürg Böni; Kevin Steiner; Maria Grünberg; Maryam Zaheri; Michael Huber; Riccarda Capaul; Stefan Schmutz; Verena Kufner
EPI_ISL_2840091, EPI_ISL_2840156, EPI_ISL_2897535, EPI_ISL_2899164, EPI_ISL_2899544	Unilabs	Karolinska University Hospital	Annelie Bjerkner; Isak Sylvin; Jan Albert; Karolina Ininbergs; Lina Guerra Blomqvist; Lynda Eneh; Martin Ekman; Martina Wahlund; Robert Dyrdak; Sandra Broddesson; Tanja Normark; Tobias Allander; Valtteri Wirta; Zhibing Yun
EPI_ISL_2776218	Universidad Central del Caribe (UCC)	Grubaugh Lab - Yale School of Public Health	Anderson Brito; Andrea Firpo; Annie Watkins; Chaney Kalinich; Chantal Vogels; David Rivera Aponte; Ernesto Vazquez; Fabiola Cruz-Lopez; Fabiola Fontanet; Isabel Ott; Jessica Rothman; Joseph Fauver; Kendall Billig; Mallery Breban; Mary Petrone; Natalie Machargo; Nathan Grubaugh; Patricia Serrano; Tania Mitwalli; Tara Alpert; Tobias Koch; Vianca Aponte
EPI_ISL_3477567	Universidad del Valle, LDAB-Laboratorio de Diagnostico de Agentes Biologicos	Universidad del Valle, TAO-Lab, VIREM, NEAS Network	Andres Castillo; Beatriz Parra & COVID-19 Team Univalle; Diana López-Alvarez; Nelson Rivera Franco
EPI_ISL_3076939	University Clinical Hospital of Mostar, Department of Microbiology and Molecular Diagnostics	University of Sarajevo, Veterinary Faculty, Laboratory for Molecular Diagnostic and Research Laboratory	Goletic S; Goletic T; Hodzic A.; Jazic A.; Nicevic M.; Ostojic M; Sabic E.; Softic A; Terzic I.
EPI_ISL_2405336, EPI_ISL_2681457	University Hospitals of Geneva, Laboratory of Virology	HUG, Laboratory of Virology and the Health2030 Genome Center	Ana Rita Goncalves; Deborah Penet; Emmanouil Dermitzakis; Henri Pegeot; Ioannis Xenarios; Keith Harshman; Laurent Kaiser; Lorenzo Cerutti; Melyssa Elies; Samuel Cordey
EPI_ISL_2692019, EPI_ISL_2692022, EPI_ISL_2692023, EPI_ISL_3388801	University of Chicago Medicine	RIPHL at Rush University Medical Center	Edith Perez; Felix Araujo Perez; Kevin Kunstman; Laura Furtado; Lauren Megger; Marieta Hyde; Max Kolton; Stefan Green
EPI_ISL_2692112, EPI_ISL_2692121, EPI_ISL_3388712	University of Illinois at Chicago	RIPHL at Rush University Medical Center	Edith Perez; Felix Araujo Perez; Iuliana Bentea; Julie Bentea; Kevin Kunstman; Laura Furtado; Marieta Hyde; Max Kolton; Omar Perez; Stefan Green
EPI_ISL_3537377, EPI_ISL_3537381	University of Iowa Hospitals and Clinics	State Hygienic Laboratory at the University of Iowa	Alankar Kampoowale; Anna Yakos; Erik Twait; Kris Eveland; Mariah Knutson; Valerie Reeb; Wes Hottel
EPI_ISL_2690251	University of Mississippi Medical Center, Department of Pathology	University of Mississippi Medical Center, Molecular and Genomics Core Facility	Ashley C. Johnson; D. Ashley Robinson; Ithiel J. Frame; Krishna K. Ayyalasomayajula; Michael R. Garrett; Wenjie Wu
EPI_ISL_2650508, EPI_ISL_2650510, EPI_ISL_2650512, EPI_ISL_2650515	University of Oregon COVID-19 MAP Laboratory	University of Oregon Genomics and Cell Characterization Core Facility (GC3F)	Ariana White; Douglas Turnbull; Jason Carriere; Jason Sydes; Jeff Bishop; Peter Batzel

EPI_ISL_2484682, EPI_ISL_2918786	UniversitätsSpital Zürich	Institute of Medical Virology	Alexandra Trkola; Annette Audigé; Catharine Aquino; Cyril Shah; Daniel Ehrsam; Gabriela Ziltener; Guido Bloemberg; Hubert Rehrauer; Isabel Stürmer; Joel Wirz; Jon Huder; Jürg Böni; Kevin Steiner; Maria Grünberg; Maryam Zaheri; Michael Huber; Riccarda Capaul; Stefan Schmutz; Verena Kufner; Weihong Qi
EPI_ISL_2544269	UniversitätsSpital Zürich 009	Institute of Medical Virology	Alexandra Trkola; Annette Audigé; Cyril Shah; Gabriela Ziltener; Guido Bloemberg; Jon Huder; Jürg Böni; Kevin Steiner; Maria Grünberg; Maryam Zaheri; Michael Huber; Riccarda Capaul; Stefan Schmutz; Verena Kufner
EPI_ISL_3506141	Usansolo-Galdakao University Hospital	Cruces University Hospital	Ana Belén de la Hz; Ana Gual-de-Torrella; Izaskun Alejo-Cancho; Mikel Gallego
EPI_ISL_2678949, EPI_ISL_2678954, EPI_ISL_2678965, EPI_ISL_2679234, EPI_ISL_2679344, EPI_ISL_2856304, EPI_ISL_2856315, EPI_ISL_2856371, EPI_ISL_2856376, EPI_ISL_2856512, EPI_ISL_2856556, EPI_ISL_2856563, EPI_ISL_2856580, EPI_ISL_2856711, EPI_ISL_2856721, EPI_ISL_2856739, EPI_ISL_2856811, EPI_ISL_2856814, EPI_ISL_2856853, EPI_ISL_2856854, EPI_ISL_2856888, EPI_ISL_2856909, EPI_ISL_2856931, EPI_ISL_2856966, EPI_ISL_2857044, EPI_ISL_2857077, EPI_ISL_2857164, EPI_ISL_2857232, EPI_ISL_2857257, EPI_ISL_2857336, EPI_ISL_2857347, EPI_ISL_2857582, EPI_ISL_2857585, EPI_ISL_2857608, EPI_ISL_2857704, EPI_ISL_2857706, EPI_ISL_2857746, EPI_ISL_2857747, EPI_ISL_2857767, EPI_ISL_3132731, EPI_ISL_3132736, EPI_ISL_3132778, EPI_ISL_3132958, EPI_ISL_3132965, EPI_ISL_3133017, EPI_ISL_3144682, EPI_ISL_3268526, EPI_ISL_3268532, EPI_ISL_3268537, EPI_ISL_3268559, EPI_ISL_3268601, EPI_ISL_3268609, EPI_ISL_3268615, EPI_ISL_3268701, EPI_ISL_3268786, EPI_ISL_3268835, EPI_ISL_3268861, EPI_ISL_3268907, EPI_ISL_3268916, EPI_ISL_3268945, EPI_ISL_3268952, EPI_ISL_3269897, EPI_ISL_3745387, EPI_ISL_3745388, EPI_ISL_3745390, EPI_ISL_3745392, EPI_ISL_3745393, EPI_ISL_3745397, EPI_ISL_3745398, EPI_ISL_3745400, EPI_ISL_3756245, EPI_ISL_3756246, EPI_ISL_3756247, EPI_ISL_3756249, EPI_ISL_3756250, EPI_ISL_3756251, EPI_ISL_3756252, EPI_ISL_3756253, EPI_ISL_3756254, EPI_ISL_3756255, EPI_ISL_3756256, EPI_ISL_3756257, EPI_ISL_3756258, EPI_ISL_3756259, EPI_ISL_3756260			
see above	Utah Public Health Laboratory	Utah Public Health Laboratory	Erin L. Young; Kelly F. Oakeson; Olinto Linares-Perdomo; Pooja Gupta; Tara Gallagher
EPI_ISL_3355143, EPI_ISL_3355159, EPI_ISL_3355209	VA-Division of Consolidated Laboratory Services	Centers for Disease Control and Prevention Division of Viral Diseases, Pathogen Discovery	Alex Burgin; Ben Rambo-Martin; Clinton Paden; Dakota Howard; Dave Wentworth; Dhwani Batra; Jasmine Padilla; Justin Lee; Krista Queen; Kristen Knipe; Kristine Lacek; Mark Burroughs; Matthew Schmerer; Meghan Bentz; Mili Sheth; Peter Cook; Sam Shepard; Sarah Nobles; Suxiang Tong; Vivien Dugan; Yvette Unoarumhi
EPI_ISL_2462316, EPI_ISL_2493841	VIGILANCIA EM SAUDE DE FCO MORATO	Instituto Butantan	Antonio Jorge Martins; Claudia Renata dos Santos Barros; David Schlesinger; Debora Botequio Moretti; Dimas Tadeu Covas; Elaine Cristina Marqueze; Elaine Vieira Santos; Evandra Strazza Rodrigues; Heidge Fukumasu; Jayme Augusto de Souza-Neto; José Salvatore Leister Patané; Luiz Alcantara; Luiz Lehmann Coutinho; Maria Carolina Elias; Maurício Lacerda Nogueira; Rafael dos Santos Bezerra; Raul Machado Neto; Rejane Maria Tommasini Grotto; Ricardo Haddad; Sandra Coccuzzo Sampaio Vessoni; Simone Kashima; Svetoslav Nanev Slavov; Vincent Louis Viala
EPI_ISL_2494111, EPI_ISL_2494112, EPI_ISL_2494113	VIGILANCIA EM SAUDE SAE VIGILANCIA EPIDEMIOLOGICA	Instituto Butantan	Antonio Jorge Martins; Claudia Renata dos Santos Barros; David Schlesinger; Debora Botequio Moretti; Dimas Tadeu Covas; Elaine Cristina Marqueze; Elaine Vieira Santos; Evandra Strazza Rodrigues; Heidge Fukumasu; Jayme Augusto de Souza-Neto; José Salvatore Leister Patané; Luiz Alcantara; Luiz Lehmann Coutinho; Maria Carolina Elias; Maurício Lacerda Nogueira; Rafael dos Santos Bezerra; Raul Machado Neto; Rejane Maria Tommasini Grotto; Ricardo Haddad; Sandra Coccuzzo Sampaio Vessoni; Simone Kashima; Svetoslav Nanev Slavov; Vincent Louis Viala
EPI_ISL_2493916	VIGILANCIA EPIDEMIOLOGICA DE FERRAZ DE VASCONCELOS	Instituto Butantan	Antonio Jorge Martins; Claudia Renata dos Santos Barros; David Schlesinger; Debora Botequio Moretti; Dimas Tadeu Covas; Elaine Cristina Marqueze; Elaine Vieira Santos; Evandra Strazza Rodrigues; Heidge Fukumasu; Jayme Augusto de Souza-Neto; José Salvatore Leister Patané; Luiz Alcantara; Luiz Lehmann Coutinho; Maria Carolina Elias; Maurício Lacerda Nogueira; Rafael dos Santos Bezerra; Raul Machado Neto; Rejane Maria Tommasini Grotto; Ricardo Haddad; Sandra Coccuzzo Sampaio Vessoni; Simone Kashima; Svetoslav Nanev Slavov; Vincent Louis Viala
EPI_ISL_2494162, EPI_ISL_2494173, EPI_ISL_2494336	VIGILANCIA EPIDEMIOLOGICA DE LEME	Instituto Butantan	Antonio Jorge Martins; Claudia Renata dos Santos Barros; David Schlesinger; Debora Botequio Moretti; Dimas Tadeu Covas; Elaine Cristina Marqueze; Elaine Vieira Santos; Evandra Strazza Rodrigues; Heidge Fukumasu; Jayme Augusto de Souza-Neto; José Salvatore Leister Patané; Luiz Alcantara; Luiz Lehmann Coutinho; Maria Carolina Elias; Maurício Lacerda Nogueira; Rafael dos Santos Bezerra; Raul Machado Neto; Rejane Maria Tommasini Grotto; Ricardo Haddad; Sandra Coccuzzo Sampaio Vessoni; Simone Kashima; Svetoslav Nanev Slavov; Vincent Louis Viala
EPI_ISL_2494171, EPI_ISL_2494191, EPI_ISL_2494197, EPI_ISL_2494198	VIGILANCIA EPIDEMIOLOGICA E CONTROLE DE VETORES PIRASSUNUN	Instituto Butantan	Antonio Jorge Martins; Claudia Renata dos Santos Barros; David Schlesinger; Debora Botequio Moretti; Dimas Tadeu Covas; Elaine Cristina Marqueze; Elaine Vieira Santos; Evandra Strazza Rodrigues; Heidge Fukumasu; Jayme Augusto de Souza-Neto; José Salvatore Leister Patané; Luiz Alcantara; Luiz Lehmann Coutinho; Maria Carolina Elias; Maurício Lacerda Nogueira; Rafael dos Santos Bezerra; Raul Machado Neto; Rejane Maria Tommasini Grotto; Ricardo Haddad; Sandra Coccuzzo Sampaio Vessoni; Simone Kashima; Svetoslav Nanev Slavov; Vincent Louis Viala
EPI_ISL_2493944, EPI_ISL_2493945, EPI_ISL_2493949, EPI_ISL_2493953, EPI_ISL_2493958	VIGILANCIA SANITARIA E VIG EPIDEMIOLOGICA DE ITAPEVI	Instituto Butantan	Antonio Jorge Martins; Claudia Renata dos Santos Barros; David Schlesinger; Debora Botequio Moretti; Dimas Tadeu Covas; Elaine Cristina Marqueze; Elaine Vieira Santos; Evandra Strazza Rodrigues; Heidge Fukumasu; Jayme Augusto de Souza-Neto; José Salvatore Leister Patané; Luiz Alcantara; Luiz Lehmann Coutinho; Maria Carolina Elias; Maurício Lacerda Nogueira; Rafael dos Santos Bezerra; Raul Machado Neto; Rejane Maria Tommasini Grotto; Ricardo Haddad; Sandra Coccuzzo Sampaio Vessoni; Simone Kashima; Svetoslav Nanev Slavov; Vincent Louis Viala
EPI_ISL_2544492, EPI_ISL_2662372, EPI_ISL_2662403, EPI_ISL_2662445, EPI_ISL_2662446, EPI_ISL_2724466, EPI_ISL_2938392, EPI_ISL_2938458, EPI_ISL_3007545, EPI_ISL_3007610, EPI_ISL_3007621			
see above	Viollier AG	Department of Biosystems Science and Engineering, ETH Zürich	Andrea Patrignani; Andrea Patrizia Salzmann; Andreia Cabral de Gouvea; Catharine Aquino; Chaoran Chen; Christian Beisel; Christiane Beckmann; Christoph Noppen; Daniel Ehrsam; Doris Popovic; Elodie Burcklen; Griffin White; Henriette Kurth; Ina Nissen; Isabel Stürmer; Ivan Topolsky; Jay Tracy; Kim Philipp Jablonski; Lara Fuhrmann; Laura Neff; Lennart Opitz; Maria Domenica Moccia; Maurice Redondo; Mirjam Feldkamp; Natascha Santacroce; Niko Beerenwinkel; Olivier Kobel; Ralph Schlapbach; Rebecca Denes; Sarah Nadeau; Simon Grüter; Tanja Stadler; Timothy Sykes
EPI_ISL_2420698, EPI_ISL_2420707	Viollier AG	Viollier AG	Andrea Patrizia Salzmann; Christiane Beckmann; Christoph Noppen; Henriette Kurth; Maurice Redondo; Olivier Kobel
EPI_ISL_2603346, EPI_ISL_2603359, EPI_ISL_2689853, EPI_ISL_2689854, EPI_ISL_2776029, EPI_ISL_2776030, EPI_ISL_2776043, EPI_ISL_2776050, EPI_ISL_2776054, EPI_ISL_2920793, EPI_ISL_2982646, EPI_ISL_2982657, EPI_ISL_3025879, EPI_ISL_3048097, EPI_ISL_3123430			
see above	Virginia Division of Consolidated Laboratory Services	Virginia Division of Consolidated Laboratory Services	Virginia DCLS; Virginia Division of Consolidated Laboratory Services
EPI_ISL_3230510	Vita Laboratoriot Oy	Expert Microbiology, National Institute for Health and Welfare	Carita Savolainen-Kopra; Erika Lindh; Haider al-Hello; Jani Halkilahti; Kirsi Liitsola; Niina Ikonen; Olli Vapalahti; Pekka Ellonen; Phuoc Truong; Päivi Laurila; Ravi Kant; Sari Hannula; Soile Blomqvist; Teemu Smura
EPI_ISL_2690098, EPI_ISL_2690108, EPI_ISL_2690110, EPI_ISL_2788021, EPI_ISL_2788026	WA State Department of Health	Centers for Disease Control and Prevention Division of Viral Diseases, Pathogen Discovery	Alison Laufer Halpin; Ben L. Rambo-Martin; Clinton R. Paden; Dakota Howard; Darlene Wagner; Dave Wentworth; Dhwani Batra; Jasmine Padilla; Justin Lee; Katie Dillon; Krista Queen; Kristen Knipe; Kristine Lacek; Mark Burroughs; Matthew Schmerer; Mili Sheth; Peter Cook; Sam Shepard; Sarah Nobles; Shoshona Le; Suxiang Tong; Vivien Dugan; Yvette Unoarumhi
EPI_ISL_3031272, EPI_ISL_3031273, EPI_ISL_3031274	WESTCHESTER MEDICAL CENTER	Wadsworth Center, New York State Department of Health	Alexis Russell; Catharine Prussing; Daryl M. Lamson; Erasmus Schneider; Erica Lasek-Nesselquist; John Kelly; Jonathan Plitnick; Kirsten St. George; Matthew Shudt; Melissa A Leisner; Navjot Singh
EPI_ISL_2787585	WI State Laboratory of Hygiene	Centers for Disease Control and Prevention Division of Viral Diseases, Pathogen Discovery	Alison Laufer Halpin; Ben L. Rambo-Martin; Clinton R. Paden; Dakota Howard; Darlene Wagner; Dave Wentworth; Dhwani Batra; Jasmine Padilla; Justin Lee; Katie Dillon; Krista Queen; Kristen Knipe; Kristine Lacek; Mark Burroughs; Matthew Schmerer; Mili Sheth; Peter Cook; Sam Shepard; Sarah Nobles; Shoshona Le; Suxiang Tong; Vivien Dugan; Yvette Unoarumhi
EPI_ISL_2597899	WSSE w Gdańsku	1. Tricity SARS-CoV-2 sequencing consortium: University of Gdansk, Medical University of Gdansk, Vaxican Ltd., Invicta Ltd. 2. National Institute of Public Health - National Institute of Hygiene, Warsaw, Poland	Celina Cybulska; Karolina Gackowska; Katarzyna Groth; Katarzyna Zacharczuk; Krystyna Bienkowska Szewczyk; Lukasz Rabalski; Maciej Grzybek; Maciej Kosinski; Magdalena Nowakowska; Marcin Lubocki; Małgorzata Sadkowska-Todys; Tomasz Wołkowicz
EPI_ISL_3009183	WSSE w Katowicach	1. Academic Center for Pathomorphological and Genetic-Molecular Diagnostics ltd, Bialystok, Poland 2. National Institute of Public Health - National Institute of Hygiene, Warsaw, Poland	Anetta Sulewska; Jacek Nikliński; Janusz Dzięcioł; Joanna Kiśluk; Katarzyna Zacharczuk; Konrad Raczkowski; Magdalena Nowakowska; Małgorzata Sadkowska-Todys; Piotr Karabowicz; Piotr Majewski; Przemysław Biecek. Joanna Reszeć; Radosław Charkiewicz; Tomasz Wołkowicz
EPI_ISL_2557057, EPI_ISL_2557063, EPI_ISL_2557066, EPI_ISL_2557067, EPI_ISL_2557071, EPI_ISL_2557075, EPI_ISL_2557079, EPI_ISL_2651042, EPI_ISL_2651054, EPI_ISL_2651062, EPI_ISL_2651064, EPI_ISL_2651077, EPI_ISL_2651083, EPI_ISL_2651084, EPI_ISL_2651086, EPI_ISL_2651090, EPI_ISL_2651097, EPI_ISL_2651105, EPI_ISL_2716463, EPI_ISL_2716464, EPI_ISL_2716466, EPI_ISL_2716469, EPI_ISL_2716470, EPI_ISL_2759880, EPI_ISL_2834708, EPI_ISL_3016356, EPI_ISL_3016360, EPI_ISL_3016387, EPI_ISL_3216979			
see above	Washington State Department of Health Public Health Laboratories	Washington State Department of Health Public Health Laboratories	Avi Singh; Darren Lucas; Denny Russell; Drew MacKellar; Geoff Melly; Hannah Gray; Joenice Gonzalez; JohnAric Peterson; Philip Dykema; Rebecca Cao; Vanessa De Los Santos
EPI_ISL_2774524, EPI_ISL_3235364, EPI_ISL_3235365, EPI_ISL_3246657	Weill Cornell Medicine	New York Genome Center	Amy Baldwin; Andre Corvelo; Arryn Craney; Chris Mason; Dayna M. Oschwald; Hanna Rennert; Lars F Westblade; Margaret Elizabeth Ross; Melissa Cushing; Michael Zody; Olivier Elemento; Priya Velu; Samantha Fennessey; Tom Maniatis
EPI_ISL_2876102	Wexner Medical Center	The Ohio State University College of Medicine	Koenig, S.; Seminetta, J.
EPI_ISL_2400234, EPI_ISL_2499942, EPI_ISL_2534256, EPI_ISL_2612191, EPI_ISL_2612195, EPI_ISL_2627938, EPI_ISL_2627939, EPI_ISL_2675197, EPI_ISL_2677640, EPI_ISL_2756526, EPI_ISL_2756532			
see above	Wichita State University - Molecular Diagnostics Lab	Kansas Health and Environmental Lab	Ben Olsen; Jonathan Barnell; Katherine Wiggins; Mike Grose; and Phil Adam
EPI_ISL_2833947	Wichita State University- Molecular Diagnostic Lab	Kansas Health and Environmental Lab	Ben Olsen; Jonathan Barnell; Katherine Wiggins; Mike Grose; and Phil Adam
EPI_ISL_2382562, EPI_ISL_2382563, EPI_ISL_2383017, EPI_ISL_2383018	Wichita State University- Molecular Diagonstic lab	Kansas Health and Environmental Lab	Ben Olsen; Jonathan Barnell; Katherine Wiggins; Mike Grose; and Phil Adam
EPI_ISL_2383180, EPI_ISL_2561917, EPI_ISL_2561926	Willis-Knighton Medical Center Hospital Laboratory	LSUHS Emerging Viral Threat Laboratory	Alexander Mijalis; Andrew D. Yurochko; April N. Johnson; Christopher G. Kevil; Gregory L. Ware; Jennifer L. Carroll; Jeremy P. Kamil; John A. Vanchiere; Joseph A. Bocchini; Lorie M. Atkins; Maarten Van Diest; Rona S. Scott
EPI_ISL_2558601, EPI_ISL_2558668, EPI_ISL_2558669, EPI_ISL_2727681, EPI_ISL_2727687, EPI_ISL_2727705, EPI_ISL_2727708, EPI_ISL_2727712, EPI_ISL_2727766, EPI_ISL_2727775, EPI_ISL_2727782, EPI_ISL_2727792, EPI_ISL_2727816, EPI_ISL_2727817, EPI_ISL_2727826, EPI_ISL_3131920, EPI_ISL_3131936, EPI_ISL_3131949, EPI_ISL_3131960, EPI_ISL_3234718, EPI_ISL_3234720			
see above	Wisconsin State Laboratory of Hygiene Communicable Disease Division	Wisconsin State Laboratory of Hygiene Communicable Disease Division	Abigail C. Shockey; Alicia J. Mooney; Erika M. Hanson; Kelsey R. Florek; Richard Griesser; Sara Wagner; Tonya Danz
EPI_ISL_2712919, EPI_ISL_2712921, EPI_ISL_2726819, EPI_ISL_2754064, EPI_ISL_2801550, EPI_ISL_2833845, EPI_ISL_2834976, EPI_ISL_2960293, EPI_ISL_3038893, EPI_ISL_3062475, EPI_ISL_3160600, EPI_ISL_3160630, EPI_ISL_3207429			
see above	Wyoming Public Health Laboratory	Wyoming Public Health Laboratory	Ashley Norberg; Brian Dominguez; Cari Sloma; Channing Weber; Chayse Rowley; Elliot Thomasson; Jim Mildenberger; Marley Goetz; Robert Petit; Sam Britz; Taylor Fearing; and Rob Christensen

EPI_ISL_2716232, EPI_ISL_2716245, EPI_ISL_2860312, EPI_ISL_3025425, EPI_ISL_3104693, EPI_ISL_3104694	Yale Clinical Virology Lab	Grubaugh Lab - Yale School of Public Health	Anderson Brito; Annie Watkins; Chaney Kalinich; Chantal Vogels; Isabel Ott; Jessica Rothman; Joseph Fauver; Kendall Billig; Mallery Breban; Marie L. Landry; Mary Petrone; Nathan Grubaugh; Tara Alpert; Tobias Koch

We gratefully acknowledge the following Authors from the Originating laboratories responsible for obtaining the specimens, as well as the Submitting laboratories where the genome data were generated and shared via GISAID, on which this research is based.

All Submitters of data may be contacted directly via www.gisaid.org

Authors are sorted alphabetically.

Accession ID	Originating Laboratory	Submitting Laboratory	Authors
EPI_ISL_2689871, EPI_ISL_2689873	"AK State Public Health Lab, State Health Department"	Centers for Disease Control and Prevention Division of Viral Diseases, Pathogen Discovery	Alison Laufer Halpin; Ben L. Rambo-Martin; Clinton R. Paden; Dakota Howard; Darlene Wagner; Dave Wentworth; Dhwani Batra; Jasmine Padilla; Justin Lee; Katie Dillon; Krista Queen; Kristen Knipe; Kristine Lacek; Mark Burroughs; Matthew Schmerer; Mili Sheth; Peter Cook; Sam Shepard; Sarah Nobles; Shoshona Le; Suxiang Tong; Vivien Dugan; Yvette Unoarumhi
EPI_ISL_3354922	"AR Dept. of Health-PHL, Molecular Diagnostics"	Centers for Disease Control and Prevention Division of Viral Diseases, Pathogen Discovery	Alex Burgin; Ben Rambo-Martin; Clinton Paden; Dakota Howard; Dave Wentworth; Dhwani Batra; Jasmine Padilla; Justin Lee; Krista Queen; Kristen Knipe; Kristine Lacek; Mark Burroughs; Matthew Schmerer; Meghan Bentz; Mili Sheth; Peter Cook; Sam Shepard; Sarah Nobles; Suxiang Tong; Vivien Dugan; Yvette Unoarumhi
EPI_ISL_2463003	"TX DSHS, Lab Services Section MC 1947"	Centers for Disease Control and Prevention Division of Viral Diseases, Pathogen Discovery	Alison Laufer Halpin; Ben L. Rambo-Martin; Clinton R. Paden; Dakota Howard; Darlene Wagner; Dave Wentworth; Dhwani Batra; Jasmine Padilla; Justin Lee; Katie Dillon; Krista Queen; Kristen Knipe; Kristine Lacek; Mark Burroughs; Matthew Schmerer; Mili Sheth; Peter Cook; Sam Shepard; Sarah Nobles; Shoshona Le; Suxiang Tong; Vivien Dugan; Yvette Unoarumhi
EPI_ISL_2650703	A.S.L. NOVARA - ISTITUTO S.S. TRINITA' - BORGOMANERO - S.S. LABORATORIO ANALISI CHIMICO CLINICHE -	Fondazione del Piemonte per l'Oncologia IRCCS	Antonino Sottile; Giorgio Giardina; Paola Marino; Silvia Brossa
EPI_ISL_3347194, EPI_ISL_3347195, EPI_ISL_3347196, EPI_ISL_3347197, EPI_ISL_3347198, EPI_ISL_3347199, EPI_ISL_3347200, EPI_ISL_3347201, EPI_ISL_3347202			
see above	ADILAB	Laboratorio Departamental de Salud Publica de Antioquia	Andres F. Cardona-Rios; Gloria Isabel Escobar; Idabely Betancur Ortiz; Juan P. Hernandez-Ortiz; Maria Stella López
EPI_ISL_2493256, EPI_ISL_2493285, EPI_ISL_2493739, EPI_ISL_2493740, EPI_ISL_2493741, EPI_ISL_2493742, EPI_ISL_2493744, EPI_ISL_2493745, EPI_ISL_2493749, EPI_ISL_2493751, EPI_ISL_2493753, EPI_ISL_2493754, EPI_ISL_2493755, EPI_ISL_2493756, EPI_ISL_2493758, EPI_ISL_2493759, EPI_ISL_2493761, EPI_ISL_2493762, EPI_ISL_2494293			
see above	AFIP	Instituto Butantan	Antonio Jorge Martins; Claudia Renata dos Santos Barros; David Schlesinger; Debora Botequio Moretti; Dimas Tadeu Covas; Elaine Cristina Marqueze; Elaine Vieira Santos; Evandra Strazza Rodrigues; Heidge Fukumasu; Jayme Augusto de Souza-Neto; José Salvatore Leister Patané; Luiz Alcantara; Luiz Lehmann Coutinho; Maria Carolina Elias; Maurício Lacerda Nogueira; Rafael dos Santos Bezerra; Raul Machado Neto; Rejane Maria Tommasini Grotto; Ricardo Haddad; Sandra Coccuzzo Sampaio Vessoni; Simone Kashima; Svetoslav Nanev Slavov; Vincent Louis Viala
EPI_ISL_2229280, EPI_ISL_2229281, EPI_ISL_2229282, EPI_ISL_2383983	AL Dept. of Public Health Bureau of Clinical Laboratories	Centers for Disease Control and Prevention Division of Viral Diseases, Pathogen Discovery	Alison Laufer Halpin; Ben L. Rambo-Martin; Clinton R. Paden; Dakota Howard; Darlene Wagner; Dave Wentworth; Dhwani Batra; Jasmine Padilla; Justin Lee; Katie Dillon; Krista Queen; Kristen Knipe; Kristine Lacek; Mark Burroughs; Matthew Schmerer; Mili Sheth; Peter Cook; Sam Shepard; Sarah Nobles; Shoshona Le; Suxiang Tong; Vivien Dugan; Yvette Unoarumhi
EPI_ISL_2098500, EPI_ISL_2279505, EPI_ISL_2627157	ALBANY MEDICAL CENTER	Wadsworth Center, New York State Department of Health	Alexis Russell; Catharine Prussing; Daryl M. Lamson; Erasmus Schneider; Erica Lasek-Nesselquist; John Kelly; Jonathan Plitnick; Kirsten St. George; Matthew Shudt; Melissa A Leisner; Navjot Singh
EPI_ISL_2208874	AMB MEDICO DR FRANCISCO FELIX DE MENDONCA SAUDADE	Instituto Butantan / ESALQ-Piracicaba	Antonio Jorge Martins; Claudia Renata dos Santos Barros; David Schlesinger; Debora Botequio Moretti; Dimas Tadeu Covas; Elaine Cristina Marqueze; Elaine Vieira Santos; Evandra Strazza Rodrigues; Heidge Fukumasu; Jayme Augusto de Souza-Neto; José Salvatore Leister Patané; Luiz Alcantara; Luiz Lehmann Coutinho; Maria Carolina Elias; Maurício Lacerda Nogueira; Rafael dos Santos Bezerra; Raul Machado Neto; Rejane Maria Tommasini Grotto; Ricardo Haddad; Sandra Coccuzzo Sampaio Vessoni; Simone Kashima; Svetoslav Nanev Slavov; Vincent Louis Viala
EPI_ISL_2493553	AMBULATORIO AVIACAO	Instituto Butantan	Antonio Jorge Martins; Claudia Renata dos Santos Barros; David Schlesinger; Debora Botequio Moretti; Dimas Tadeu Covas; Elaine Cristina Marqueze; Elaine Vieira Santos; Evandra Strazza Rodrigues; Heidge Fukumasu; Jayme Augusto de Souza-Neto; José Salvatore Leister Patané; Luiz Alcantara; Luiz Lehmann Coutinho; Maria Carolina Elias; Maurício Lacerda Nogueira; Rafael dos Santos Bezerra; Raul Machado Neto; Rejane Maria Tommasini Grotto; Ricardo Haddad; Sandra Coccuzzo Sampaio Vessoni; Simone Kashima; Svetoslav Nanev Slavov; Vincent Louis Viala
EPI_ISL_2493549, EPI_ISL_2493554	AMBULATORIO BOQUEIRAO	Instituto Butantan	Antonio Jorge Martins; Claudia Renata dos Santos Barros; David Schlesinger; Debora Botequio Moretti; Dimas Tadeu Covas; Elaine Cristina Marqueze; Elaine Vieira Santos; Evandra Strazza Rodrigues; Heidge Fukumasu; Jayme Augusto de Souza-Neto; José Salvatore Leister Patané; Luiz Alcantara; Luiz Lehmann Coutinho; Maria Carolina Elias; Maurício Lacerda Nogueira; Rafael dos Santos Bezerra; Raul Machado Neto; Rejane Maria Tommasini Grotto; Ricardo Haddad; Sandra Coccuzzo Sampaio Vessoni; Simone Kashima; Svetoslav Nanev Slavov; Vincent Louis Viala
EPI_ISL_2493608, EPI_ISL_2493615	AMBULATORIO COVID 19	Instituto Butantan	Antonio Jorge Martins; Claudia Renata dos Santos Barros; David Schlesinger; Debora Botequio Moretti; Dimas Tadeu Covas; Elaine Cristina Marqueze; Elaine Vieira Santos; Evandra Strazza Rodrigues; Heidge Fukumasu; Jayme Augusto de Souza-Neto; José Salvatore Leister Patané; Luiz Alcantara; Luiz Lehmann Coutinho; Maria Carolina Elias; Maurício Lacerda Nogueira; Rafael dos Santos Bezerra; Raul Machado Neto; Rejane Maria Tommasini Grotto; Ricardo Haddad; Sandra Coccuzzo Sampaio Vessoni; Simone Kashima; Svetoslav Nanev Slavov; Vincent Louis Viala
EPI_ISL_2445420, EPI_ISL_2445422, EPI_ISL_2493255, EPI_ISL_2493529, EPI_ISL_2493530, EPI_ISL_2493532, EPI_ISL_2493533, EPI_ISL_2493534, EPI_ISL_2500980			
see above	AMBULATORIO DE ESPECIALIDADE V E MOGI MIRIM	Instituto Butantan	Antonio Jorge Martins; Claudia Renata dos Santos Barros; David Schlesinger; Debora Botequio Moretti; Dimas Tadeu Covas; Elaine Cristina Marqueze; Elaine Vieira Santos; Evandra Strazza Rodrigues; Heidge Fukumasu; Jayme Augusto de Souza-Neto; José Salvatore Leister Patané; Luiz Alcantara; Luiz Lehmann Coutinho; Maria Carolina Elias; Maurício Lacerda Nogueira; Rafael dos Santos Bezerra; Raul Machado Neto; Rejane Maria Tommasini Grotto; Ricardo Haddad; Sandra Coccuzzo Sampaio Vessoni; Simone Kashima; Svetoslav Nanev Slavov; Vincent Louis Viala
EPI_ISL_2473762, EPI_ISL_2473763, EPI_ISL_2473770, EPI_ISL_2473772, EPI_ISL_2473774, EPI_ISL_2493364	AMBULATÓRIO INSTITUTO BUTANTAN	Instituto Butantan	Antonio Jorge Martins; Claudia Renata dos Santos Barros; David Schlesinger; Debora Botequio Moretti; Dimas Tadeu Covas; Elaine Cristina Marqueze; Elaine Vieira Santos; Evandra Strazza Rodrigues; Heidge Fukumasu; Jayme Augusto de Souza-Neto; José Salvatore Leister Patané; Luiz Alcantara; Luiz Lehmann Coutinho; Maria Carolina Elias; Maurício Lacerda Nogueira; Rafael dos Santos Bezerra; Raul Machado Neto; Rejane Maria Tommasini Grotto; Ricardo Haddad; Sandra Coccuzzo Sampaio Vessoni; Simone Kashima; Svetoslav Nanev Slavov; Vincent Louis Viala
EPI_ISL_2451779	AR Dept. of Health-PHL, Molecular Diagnostics	Centers for Disease Control and Prevention Division of Viral Diseases, Pathogen Discovery	Alison Laufer Halpin; Ben L. Rambo-Martin; Clinton R. Paden; Dakota Howard; Darlene Wagner; Dave Wentworth; Dhwani Batra; Jasmine Padilla; Justin Lee; Katie Dillon; Krista Queen; Kristen Knipe; Kristine Lacek; Mark Burroughs; Matthew Schmerer; Mili Sheth; Peter Cook; Sam Shepard; Sarah Nobles; Shoshona Le; Suxiang Tong; Vivien Dugan; Yvette Unoarumhi
EPI_ISL_2155825	AR Dept. of Health-PHL, Molecular Diagnostics	Pathogen Discovery, Respiratory Viruses Branch, Division of Viral Diseases, Centers for Disease Control and Prevention	Adam Retchless; Anna Kelleher; Anna Uehara; Brian Lynch; Clinton R. Paden; Dhwani Batra; Haibin Wang; Han Jia Justin Ng; Jasmine Padilla; Jing Zhang; Justin Lee; Krista Queen; Mark Burroughs; Mili Sheth; Morgan Davis; Peter Cook; Rachel Marine; Sarah Nobles; Suxiang Tong; Tara Coalter; Yan Li; Ying Tao
EPI_ISL_2502728	AREA DE SALUD CARRILLO	Inciensa, Instituto Costarricense de InvestigaciOn y Enseñanza en NutriciOn y Salud	Adriana Godínez; Claudio Soto-Garita; Estela Cordero; Francisco Duarte; Hebleen Porras; Jose Luis Vargas; Joselyn Prado & Ivanna Krize-Morún; Mariela Gutierrez; Melany CalderOn
EPI_ISL_2273001	AREA DE SALUD CORONADO	Inciensa, Instituto Costarricense de Investigación y Enseñanza en Nutrición y Salud	Adriana Godínez; Claudio Soto-Garita; Estela Cordero; Francisco Duarte; Hebleen Porras; Joselyn Prado & Mariel López; José Luis Vargas; Mariela Gutiérrez; Melany Calderón
EPI_ISL_2502725	AREA DE SALUD CURRIDABAT 2	Inciensa, Instituto Costarricense de InvestigaciOn y Enseñanza en NutriciOn y Salud	Adriana Godínez; Claudio Soto-Garita; Estela Cordero; Francisco Duarte; Hebleen Porras; Jose Luis Vargas; Joselyn Prado & Mariel LOpez; Mariela Gutierrez; Melany CalderOn
EPI_ISL_2272983	AREA DE SALUD GOICOECHEA 2 - CLINICA DR. JIMENEZ NUÑEZ	Inciensa, Instituto Costarricense de Investigación y Enseñanza en Nutrición y Salud	Adriana Godínez; Claudio Soto-Garita; Estela Cordero; Francisco Duarte; Hebleen Porras; Joselyn Prado & Juan Carlos Cartes Parra; José Luis Vargas; Mariela Gutiérrez; Melany Calderón
EPI_ISL_2502747	AREA DE SALUD PARAISO-CERVANTES	Inciensa, Instituto Costarricense de InvestigaciOn y Enseñanza en NutriciOn y Salud	Adriana Godínez; Claudio Soto-Garita; Estela Cordero; Francisco Duarte; Hebleen Porras; Jose Luis Vargas; Mariela Gutierrez & Joselyn Prado; Melany CalderOn
EPI_ISL_2273004	AREA DE SALUD SAN JUAN-SAN DIEGO-CONCEPCION 2	Inciensa, Instituto Costarricense de Investigación y Enseñanza en Nutrición y Salud	Adriana Godínez; Claudio Soto-Garita; Estela Cordero; Francisco Duarte; Hebleen Porras; Joselyn Prado & Mariel López; José Luis Vargas; Mariela Gutiérrez; Melany Calderón
EPI_ISL_2658269	AREA DE SALUD ZAPOTE-CATEDRAL - CLINICA DR. CARLOS DURAN	Inciensa, Instituto Costarricense de Investigación y Enseñanza en Nutrición y Salud	Adriana Godínez; Claudio Soto-Garita; Estela Cordero; Francisco Duarte; Hebleen Porras; Joselyn Prado & Andony Cordero-Jiménez; José Luis Vargas; Mariela Gutiérrez; Melany Calderón
EPI_ISL_1999796, EPI_ISL_1999817, EPI_ISL_1999841, EPI_ISL_1999873, EPI_ISL_2000082, EPI_ISL_2000100, EPI_ISL_2000104, EPI_ISL_2000119, EPI_ISL_2039060, EPI_ISL_2039083, EPI_ISL_2039096, EPI_ISL_2039124, EPI_ISL_2039127, EPI_ISL_2039144, EPI_ISL_2039149, EPI_ISL_2039153, EPI_ISL_2039158, EPI_ISL_2039163, EPI_ISL_2039390, EPI_ISL_2039416, EPI_ISL_2039460, EPI_ISL_2039469, EPI_ISL_2039470, EPI_ISL_2039485, EPI_ISL_2039527, EPI_ISL_2039666, EPI_ISL_2039783, EPI_ISL_2039843, EPI_ISL_2039845, EPI_ISL_2040165, EPI_ISL_2040252, EPI_ISL_2040294, EPI_ISL_2040306, EPI_ISL_2040348, EPI_ISL_2040377, EPI_ISL_2041076, EPI_ISL_2041407, EPI_ISL_2041424, EPI_ISL_2041519, EPI_ISL_2041681, EPI_ISL_2041807, EPI_ISL_2041816, EPI_ISL_2041845, EPI_ISL_2041892, EPI_ISL_2041893, EPI_ISL_2041936, EPI_ISL_2041942, EPI_ISL_2042059, EPI_ISL_2042103, EPI_ISL_2042228, EPI_ISL_2042229, EPI_ISL_2042248, EPI_ISL_2042494, EPI_ISL_2042505, EPI_ISL_2042539, EPI_ISL_2042605, EPI_ISL_2042611, EPI_ISL_2042629, EPI_ISL_2042646, EPI_ISL_2042650, EPI_ISL_2042675, EPI_ISL_2042721, EPI_ISL_2042729, EPI_ISL_2042745, EPI_ISL_2042813, EPI_ISL_2042844, EPI_ISL_2042853, EPI_ISL_2042896, EPI_ISL_2042900, EPI_ISL_2042917, EPI_ISL_2042980, EPI_ISL_2043034, EPI_ISL_2043041, EPI_ISL_2043116, EPI_ISL_2043172, EPI_ISL_2043175, EPI_ISL_2043178, EPI_ISL_2043196, EPI_ISL_2043197, EPI_ISL_2043238, EPI_ISL_2043249, EPI_ISL_2043259, EPI_ISL_2043318, EPI_ISL_2043358, EPI_ISL_2043361, EPI_ISL_2043365, EPI_ISL_2043430, EPI_ISL_2043447, EPI_ISL_2043466, EPI_ISL_2043489, EPI_ISL_2043503, EPI_ISL_2043505, EPI_ISL_2043511, EPI_ISL_2043517, EPI_ISL_2043523, EPI_ISL_2043527, EPI_ISL_2043545, EPI_ISL_2043549, EPI_ISL_2089479, EPI_ISL_2089506, EPI_ISL_2089512, EPI_ISL_2089591, EPI_ISL_2089605, EPI_ISL_2089608, EPI_ISL_2089609, EPI_ISL_2089626, EPI_ISL_2089660, EPI_ISL_2089703, EPI_ISL_2089707, EPI_ISL_2089842, EPI_ISL_2089849, EPI_ISL_2089857, EPI_ISL_2089866, EPI_ISL_2089921, EPI_ISL_2089943, EPI_ISL_2089953, EPI_ISL_2089959, EPI_ISL_2089992, EPI_ISL_2089993, EPI_ISL_2089995, EPI_ISL_2090010, EPI_ISL_2090030, EPI_ISL_2090061, EPI_ISL_2090063, EPI_ISL_2090196, EPI_ISL_2090257, EPI_ISL_2090266, EPI_ISL_2090271, EPI_ISL_2090311, EPI_ISL_2090329, EPI_ISL_2090352, EPI_ISL_2090354, EPI_ISL_2090361, EPI_ISL_2090368, EPI_ISL_2090395, EPI_ISL_2090402, EPI_ISL_2090403, EPI_ISL_2090431, EPI_ISL_2090455, EPI_ISL_2090456, EPI_ISL_2090457, EPI_ISL_2132545, EPI_ISL_2145803, EPI_ISL_2145804, EPI_ISL_2145821, EPI_ISL_2145837, EPI_ISL_2145851, EPI_ISL_2145866, EPI_ISL_2145869, EPI_ISL_2145870, EPI_ISL_2145899, EPI_ISL_2145903, EPI_ISL_2145911, EPI_ISL_2145912, EPI_ISL_2145925, EPI_ISL_2145965, EPI_ISL_2145972, EPI_ISL_2146010, EPI_ISL_2146112, EPI_ISL_2146137, EPI_ISL_2146167, EPI_ISL_2146186, EPI_ISL_2146237, EPI_ISL_2146239, EPI_ISL_2146257, EPI_ISL_2146258, EPI_ISL_2146280, EPI_ISL_2146319, EPI_ISL_2146322, EPI_ISL_2146337, EPI_ISL_2146354, EPI_ISL_2146387, EPI_ISL_2146399, EPI_ISL_2146401, EPI_ISL_2146448, EPI_ISL_2146466, EPI_ISL_2146467, EPI_ISL_2146468, EPI_ISL_2146493, EPI_ISL_2146521, EPI_ISL_2146522, EPI_ISL_2146528, EPI_ISL_2146529, EPI_ISL_2146551, EPI_ISL_2146562, EPI_ISL_2146628, EPI_ISL_2146644, EPI_ISL_2146653, EPI_ISL_2146657, EPI_ISL_2146684, EPI_ISL_2146719, EPI_ISL_2146722, EPI_ISL_2146736, EPI_ISL_2146775, EPI_ISL_2146780, EPI_ISL_2146784, EPI_ISL_2146795, EPI_ISL_2146824, EPI_ISL_2146833, EPI_ISL_2146877, EPI_ISL_2146886, EPI_ISL_2146894, EPI_ISL_2146921, EPI_ISL_2146957, EPI_ISL_2146959, EPI_ISL_2147010, EPI_ISL_2147040, EPI_ISL_2147045, EPI_ISL_2147071, EPI_ISL_2147101, EPI_ISL_2147103, EPI_ISL_2147115, EPI_ISL_2147126, EPI_ISL_2147131, EPI_ISL_2147132, EPI_ISL_2147133, EPI_ISL_2147147, EPI_ISL_2147159, EPI_ISL_2147188, EPI_ISL_2147244, EPI_ISL_2147252, EPI_ISL_2147398, EPI_ISL_2147403, EPI_ISL_2147405, EPI_ISL_2147419, EPI_ISL_2147454, EPI_ISL_2147461, EPI_ISL_2147484, EPI_ISL_2147503, EPI_ISL_2147520, EPI_ISL_2147522, EPI_ISL_2147537, EPI_ISL_2147542, EPI_ISL_2147547, EPI_ISL_2147548, EPI_ISL_2147564, EPI_ISL_2147572, EPI_ISL_2147604, EPI_ISL_2147605, EPI_ISL_2147606, EPI_ISL_2147616, EPI_ISL_2147667, EPI_ISL_2147669, EPI_ISL_2147671, EPI_ISL_2147755, EPI_ISL_2147817, EPI_ISL_2147832, EPI_ISL_2147887, EPI_ISL_2147897, EPI_ISL_2147921, EPI_ISL_2147943, EPI_ISL_2147949, EPI_ISL_2147956, EPI_ISL_2147967, EPI_ISL_2147968, EPI_ISL_2148006, EPI_ISL_2148008, EPI_ISL_2148068, EPI_ISL_2148106, EPI_ISL_2148190, EPI_ISL_2148195, EPI_ISL_2148200, EPI_ISL_2148245, EPI_ISL_2148248, EPI_ISL_2148257, EPI_ISL_2148258, EPI_ISL_2148277, EPI_ISL_2148312, EPI_ISL_2148322, EPI_ISL_2148373, EPI_ISL_2148378, EPI_ISL_2148406, EPI_ISL_2148416, EPI_ISL_2148433, EPI_ISL_2148451, EPI_ISL_2148459, EPI_ISL_2148505, EPI_ISL_2148507, EPI_ISL_2148527, EPI_ISL_2148541, EPI_ISL_2148576, EPI_ISL_2148632, EPI_ISL_2148635, EPI_ISL_2148668, EPI_ISL_2148710, EPI_ISL_2148728, EPI_ISL_2148763, EPI_ISL_2148766, EPI_ISL_2148767, EPI_ISL_2148768, EPI_ISL_2148778, EPI_ISL_2148782, EPI_ISL_2148788, EPI_ISL_2148798, EPI_ISL_2148813, EPI_ISL_2148814, EPI_ISL_2148817, EPI_ISL_2148853, EPI_ISL_2148901, EPI_ISL_2148902, EPI_ISL_2148905, EPI_ISL_2148910, EPI_ISL_2148913, EPI_ISL_2148914, EPI_ISL_2148925, EPI_ISL_2148939, EPI_ISL_2148947, EPI_ISL_2148948, EPI_ISL_2149145, EPI_ISL_2149216, EPI_ISL_2149217, EPI_ISL_2149302, EPI_ISL_2149311, EPI_ISL_2149318, EPI_ISL_2149340, EPI_ISL_2149356, EPI_ISL_2149385, EPI_ISL_2149391, EPI_ISL_2149418, EPI_ISL_2149448, EPI_ISL_2149499, EPI_ISL_2149541, EPI_ISL_2149542, EPI_ISL_2149543, EPI_ISL_2149549, EPI_ISL_2149565, EPI_ISL_2149589, EPI_ISL_2149594, EPI_ISL_2149632, EPI_ISL_2149655, EPI_ISL_2149656, EPI_ISL_2149697, EPI_ISL_2149707, EPI_ISL_2149728, EPI_ISL_2149745, EPI_ISL_2149753, EPI_ISL_2149775, EPI_ISL_2149795, EPI_ISL_2149829, EPI_ISL_2149861, EPI_ISL_2149919, EPI_ISL_2149922, EPI_ISL_2149926, EPI_ISL_2150079, EPI_ISL_2150082, EPI_ISL_2150099, EPI_ISL_2150117, EPI_ISL_2150139, EPI_ISL_2150140, EPI_ISL_2150151, EPI_ISL_2150191, EPI_ISL_2150192, EPI_ISL_2150218, EPI_ISL_2150222, EPI_ISL_2150315, EPI_ISL_2150325, EPI_ISL_2150330, EPI_ISL_2150361, EPI_ISL_2150384, EPI_ISL_2150433, EPI_ISL_2150465, EPI_ISL_2150466, EPI_ISL_2150494, EPI_ISL_2150501, EPI_ISL_2150507, EPI_ISL_2150535, EPI_ISL_2150539, EPI_ISL_2150542, EPI_ISL_2150554, EPI_ISL_2150567, EPI_ISL_2150685, EPI_ISL_2150686, EPI_ISL_2150786, EPI_ISL_2150805,			

EPI_ISL_2150852, EPI_ISL_2150854, EPI_ISL_2150906, EPI_ISL_2150907, EPI_ISL_2150937, EPI_ISL_2150960, EPI_ISL_2150964, EPI_ISL_2158851, EPI_ISL_2158874, EPI_ISL_2158883, EPI_ISL_2158911, EPI_ISL_2158919, EPI_ISL_2158936, EPI_ISL_2158973, EPI_ISL_2158982, EPI_ISL_2158985, EPI_ISL_2159125, EPI_ISL_2159128, EPI_ISL_2159135, EPI_ISL_2159158, EPI_ISL_2159172, EPI_ISL_2159215, EPI_ISL_2159220, EPI_ISL_2159250, EPI_ISL_2159252, EPI_ISL_2159263, EPI_ISL_2159307, EPI_ISL_2159329, EPI_ISL_2159366, EPI_ISL_2159367, EPI_ISL_2159430, EPI_ISL_2159431, EPI_ISL_2159463, EPI_ISL_2180537, EPI_ISL_2180582, EPI_ISL_2180586, EPI_ISL_2180588, EPI_ISL_2180656, EPI_ISL_2180666, EPI_ISL_2180671, EPI_ISL_2180720, EPI_ISL_2180723, EPI_ISL_2180732, EPI_ISL_2180746, EPI_ISL_2180749, EPI_ISL_2180751, EPI_ISL_2180799, EPI_ISL_2180818, EPI_ISL_2180832, EPI_ISL_2180865, EPI_ISL_2180880, EPI_ISL_2180910, EPI_ISL_2180948, EPI_ISL_2181054, EPI_ISL_2181101, EPI_ISL_2181107, EPI_ISL_2181134, EPI_ISL_2181174, EPI_ISL_2181175, EPI_ISL_2181196, EPI_ISL_2181225, EPI_ISL_2181234, EPI_ISL_2181251, EPI_ISL_2181278, EPI_ISL_2181279, EPI_ISL_2181292, EPI_ISL_2181338, EPI_ISL_2181349, EPI_ISL_2181372, EPI_ISL_2181375, EPI_ISL_2181376, EPI_ISL_2181387, EPI_ISL_2181388, EPI_ISL_2181403, EPI_ISL_2181434, EPI_ISL_2181446, EPI_ISL_2181554, EPI_ISL_2181556, EPI_ISL_2181560, EPI_ISL_2181578, EPI_ISL_2181604, EPI_ISL_2181610, EPI_ISL_2181626, EPI_ISL_2181637, EPI_ISL_2181644, EPI_ISL_2187363, EPI_ISL_2187377, EPI_ISL_2187389, EPI_ISL_2187403, EPI_ISL_2187408, EPI_ISL_2187409, EPI_ISL_2187432, EPI_ISL_2187442, EPI_ISL_2187486, EPI_ISL_2187490, EPI_ISL_2187505, EPI_ISL_2187513, EPI_ISL_2187574, EPI_ISL_2187609, EPI_ISL_2187614, EPI_ISL_2202991, EPI_ISL_2203094, EPI_ISL_2203219, EPI_ISL_2203221, EPI_ISL_2203229, EPI_ISL_2204817, EPI_ISL_2204857, EPI_ISL_2241648, EPI_ISL_2241669, EPI_ISL_2241671, EPI_ISL_2241678, EPI_ISL_2280329, EPI_ISL_2280351, EPI_ISL_2280354, EPI_ISL_2280356, EPI_ISL_2280357, EPI_ISL_2280404, EPI_ISL_2280407, EPI_ISL_2280413, EPI_ISL_2280414, EPI_ISL_2280426, EPI_ISL_2280442, EPI_ISL_2280467, EPI_ISL_2280504, EPI_ISL_2280505, EPI_ISL_2280513, EPI_ISL_2280521, EPI_ISL_2280530, EPI_ISL_2280547, EPI_ISL_2280553, EPI_ISL_2280554, EPI_ISL_2280564, EPI_ISL_2280579, EPI_ISL_2280601, EPI_ISL_2280604, EPI_ISL_2280652, EPI_ISL_2280653, EPI_ISL_2280654, EPI_ISL_2280655, EPI_ISL_2280680, EPI_ISL_2280681, EPI_ISL_2280736, EPI_ISL_2280753, EPI_ISL_2280760, EPI_ISL_2280761, EPI_ISL_2280762, EPI_ISL_2280829, EPI_ISL_2280847, EPI_ISL_2280854, EPI_ISL_2280933, EPI_ISL_2280934, EPI_ISL_2280948, EPI_ISL_2280963, EPI_ISL_2280974, EPI_ISL_2280987, EPI_ISL_2281044, EPI_ISL_2281063, EPI_ISL_2281079, EPI_ISL_2281100, EPI_ISL_2281105, EPI_ISL_2281112, EPI_ISL_2281126, EPI_ISL_2281148, EPI_ISL_2281208, EPI_ISL_2283819, EPI_ISL_2283822, EPI_ISL_2283828, EPI_ISL_2283846, EPI_ISL_2283848, EPI_ISL_2283854, EPI_ISL_2283856, EPI_ISL_2283861, EPI_ISL_2283863, EPI_ISL_2283867, EPI_ISL_2283869, EPI_ISL_2283876, EPI_ISL_2283878, EPI_ISL_2321252, EPI_ISL_2321267, EPI_ISL_2321301, EPI_ISL_2321338, EPI_ISL_2321358, EPI_ISL_2367654, EPI_ISL_2367658, EPI_ISL_2367673, EPI_ISL_2368210, EPI_ISL_2368238, EPI_ISL_2368259, EPI_ISL_2368285, EPI_ISL_2368286, EPI_ISL_2368299, EPI_ISL_2368336, EPI_ISL_2368340, EPI_ISL_2368381, EPI_ISL_2368385, EPI_ISL_2368386, EPI_ISL_2368471, EPI_ISL_2368478, EPI_ISL_2368542, EPI_ISL_2368559, EPI_ISL_2368582, EPI_ISL_2368622, EPI_ISL_2368657, EPI_ISL_2368681, EPI_ISL_2368698, EPI_ISL_2368786, EPI_ISL_2368800, EPI_ISL_2368838, EPI_ISL_2368927, EPI_ISL_2368969, EPI_ISL_2369960, EPI_ISL_2370031, EPI_ISL_2370088, EPI_ISL_2370188, EPI_ISL_2370336, EPI_ISL_2370538, EPI_ISL_2370622, EPI_ISL_2372306, EPI_ISL_2372310, EPI_ISL_2372312, EPI_ISL_2372322, EPI_ISL_2372347, EPI_ISL_2372372, EPI_ISL_2372384, EPI_ISL_2372386, EPI_ISL_2372435, EPI_ISL_2372445, EPI_ISL_2372452, EPI_ISL_2421834, EPI_ISL_2421849, EPI_ISL_2421869, EPI_ISL_2421876, EPI_ISL_2421882, EPI_ISL_2421885, EPI_ISL_2421886, EPI_ISL_2421896, EPI_ISL_2421908, EPI_ISL_2421937, EPI_ISL_2421943, EPI_ISL_2421946, EPI_ISL_2421998, EPI_ISL_2422002, EPI_ISL_2422055, EPI_ISL_2422093, EPI_ISL_2422111, EPI_ISL_2442324, EPI_ISL_2489829, EPI_ISL_2489837, EPI_ISL_2489841, EPI_ISL_2489843, EPI_ISL_2489846, EPI_ISL_2489875, EPI_ISL_2489884, EPI_ISL_2489887, EPI_ISL_2600264, EPI_ISL_2600265, EPI_ISL_2600290			
see above	Aegis Sciences Corporation	Centers for Disease Control and Prevention Division of Viral Diseases, Pathogen Discovery	Adrian Paskey; Alec Vest; Benjamin Rambo-Martin; Christopher Gulvick; Clinton R. Paden; Cyndi Clark; Dakota Howard; Darlene Wagner; Dhwani Batra; Dillon Nall; Duncan MacCannell; Ethan Sanders; Holly Houdeshell; Jason Caravas; Kara Moser; Matthew Hardison; Matthew Schmerer; Ola Kvalvaag; Patrick Campbell; Peter W. Cook; Rob Case; Scott Sammons; Shatavia Morrison; Shaun Westlund; Vikramsinha Ghorpade; Yvette Unoarumhi
EPI_ISL_2249137	Affidea	Instituto Nacional de Saude (INSA) and Centro de Investigaçao em Biodiversidade e Recursos Geneticos (CIBIO), Universidade do Porto	Borges et al
EPI_ISL_2136442, EPI_ISL_2154069, EPI_ISL_2187648, EPI_ISL_2272531, EPI_ISL_2272538, EPI_ISL_2308647, EPI_ISL_2376008, EPI_ISL_2376069, EPI_ISL_2376089, EPI_ISL_2376098, EPI_ISL_2422100, EPI_ISL_2422101, EPI_ISL_2455425			
see above	Alaska State Virology Laboratory	Alaska State Virology Laboratory	Elva House; Jack Chen; Jacob Zidek; Lisa Smith; Ph.D.; Stephanie DeRonde
EPI_ISL_3243025, EPI_ISL_3243026, EPI_ISL_3243028, EPI_ISL_3243029, EPI_ISL_3243030, EPI_ISL_3243032, EPI_ISL_3243033			
see above	Applied DNA Sciences Inc	Applied DNA Sciences Inc	Huan Dong; Lucas Liu; Spencer William; Yuhua Sun
EPI_ISL_2227487, EPI_ISL_2227488, EPI_ISL_2227489, EPI_ISL_2325174	Arizona State Public Health Laboratory	Arizona State Public Health Laboratory	Jessica Escobar; Katherine Fullerton; Linda Getsinger; Nobuko Fukushima; Stacy White; Trung Huynh; Victor Waddell
EPI_ISL_2007175, EPI_ISL_2007176, EPI_ISL_2007178, EPI_ISL_2015335, EPI_ISL_2015344, EPI_ISL_2153660, EPI_ISL_2153661, EPI_ISL_2153663, EPI_ISL_2153664, EPI_ISL_2250017, EPI_ISL_2250018, EPI_ISL_2250021, EPI_ISL_2250036, EPI_ISL_2250047, EPI_ISL_2250060, EPI_ISL_2250082, EPI_ISL_2250100, EPI_ISL_2250102, EPI_ISL_2250123, EPI_ISL_2250130, EPI_ISL_2250132, EPI_ISL_2250147			
see above	Arizona State University	Arizona State University	Efrem S. Lim; Joshua LaBaer; Joy M. Blain; LaRinda A. Holland; Matthew F. Smith; Nicholas J. Mellor; Peter T. Skidmore; Rabia Maqsood; Valerie Harris; Vel Murugan
EPI_ISL_2484741, EPI_ISL_2484742, EPI_ISL_2931027, EPI_ISL_2931039, EPI_ISL_2931040	Arkansas Children's Hospital	Center for Global Health, University of New Mexico Health Sciences Center	Amanda Novack; Darrell Dinwiddie; Daryl Domman; Dirk Haselow; Joshua L. Kennedy; Kurt Schwalm; Valerie Morley
EPI_ISL_2484740, EPI_ISL_2931024, EPI_ISL_2939042, EPI_ISL_2939077, EPI_ISL_2939081, EPI_ISL_2939084	Arkansas Department of Health	Center for Global Health, University of New Mexico Health Sciences Center	Amanda Novack; Atul Kothari; Darrell Dinwiddie; Daryl Domman; Dirk Haselow; Joshua L. Kennedy; Kurt Schwalm; Valerie Morley
EPI_ISL_2692305, EPI_ISL_2692447, EPI_ISL_2715034	Atlas Genomics	Seattle Flu Study	Amanda Adler; Barry R. Lutz; Benjamin Pelle; Brian Hiatt; Caitlin R. Wolf; Chris D. Frazar; Deborah A. Nickerson; Elisabeth Brandstetter; Erica Ryke; Geoff Melly; Helen Y. Chu; Janet A. Englund; Jay Shendure; Jover Lee; Kairsten Fay; Kirsten Lacombe; Lea M. Starita; Mark J. Rieder; Matthew Richardson; Matthew Thompson; Melissa Truong; Michael Boeckh; Michael Famulare; Misja Ilcisin; Peter D. Han; Philip Dykema; Romesh Gautom; Scott Lindquist; Thomas R. Sibley; Trevor Bedford
EPI_ISL_2485062, EPI_ISL_2485063, EPI_ISL_2485158	Austrian Agency for Health and Food Safety (AGES)	Bergthaler laboratory, CeMM Research Center for Molecular Medicine of the Austrian Academy of Sciences	Andreas Bergthaler; Anna Schedl; Bekir Erguner; Benedikt Agerer; Christoph Bock; Fabian Amman; Jan Laine; Lukas Endler; Maelle Le Moing; Martin Senekowitsch; Michael Schuster; Petr Triska; Thomas Penz
EPI_ISL_2249139	Avelab	Instituto Nacional de Saude (INSA) and Centro de Investigaçao em Biodiversidade e Recursos Geneticos (CIBIO), Universidade do Porto	Borges et al
EPI_ISL_1840731, EPI_ISL_1840732, EPI_ISL_1840750, EPI_ISL_1840758, EPI_ISL_1840770, EPI_ISL_1840781, EPI_ISL_2080258, EPI_ISL_2080274, EPI_ISL_2080282, EPI_ISL_2080294, EPI_ISL_2080295, EPI_ISL_2080297, EPI_ISL_2318906			
see above	Azienda Sanitaria dell'Alto Adige Laboratorio Aziendale di Microbiologia e Virologia	Istituto di Genomica Applicata	Davide Scaglione; Eleonora Paparelli; Elisa Masi; Elisabetta Giacobazzi; Elisabetta Pagani; Gabriele Magris; Irena Jurman; Irene Bianconi; Michele Morgante; Stefanie Wieser; Vera Vendramin
EPI_ISL_2444173, EPI_ISL_2444202	BADY BASSITT	Instituto Butantan / FAMERP	Antonio Jorge Martins; Claudia Renata dos Santos Barros; David Schlesinger; Debora Botequio Moretti; Dimas Tadeu Covas; Elaine Cristina Marqueze; Elaine Vieira Santos; Evandra Strazza Rodrigues; Heidge Fukumasu; Jayme Augusto de Souza-Neto; José Salvatore Leister Patané; Luiz Alcantara; Luiz Lehmann Coutinho; Maria Carolina Elias; Maurício Lacerda Nogueira; Rafael dos Santos Bezerra; Raul Machado Neto; Rejane Maria Tommasini Grotto; Ricardo Haddad; Sandra Coccuzzo Sampaio Vessoni; Simone Kashima; Svetoslav Nanev Slavov; Vincent Louis Viala
EPI_ISL_2444212	BARRETOS	Instituto Butantan / FAMERP	Antonio Jorge Martins; Claudia Renata dos Santos Barros; David Schlesinger; Debora Botequio Moretti; Dimas Tadeu Covas; Elaine Cristina Marqueze; Elaine Vieira Santos; Evandra Strazza Rodrigues; Heidge Fukumasu; Jayme Augusto de Souza-Neto; José Salvatore Leister Patané; Luiz Alcantara; Luiz Lehmann Coutinho; Maria Carolina Elias; Maurício Lacerda Nogueira; Rafael dos Santos Bezerra; Raul Machado Neto; Rejane Maria Tommasini Grotto; Ricardo Haddad; Sandra Coccuzzo Sampaio Vessoni; Simone Kashima; Svetoslav Nanev Slavov; Vincent Louis Viala
EPI_ISL_2493199, EPI_ISL_2493763, EPI_ISL_2493765, EPI_ISL_2493768, EPI_ISL_2493769, EPI_ISL_2493771, EPI_ISL_2493772, EPI_ISL_2493773, EPI_ISL_2493774, EPI_ISL_2493776, EPI_ISL_2493777, EPI_ISL_2493778, EPI_ISL_2493780, EPI_ISL_2493782, EPI_ISL_2493784, EPI_ISL_2493786, EPI_ISL_2493787, EPI_ISL_2493788, EPI_ISL_2494314, EPI_ISL_2494315, EPI_ISL_2494319, EPI_ISL_2500974			
see above	BIOFAST	Instituto Butantan	Antonio Jorge Martins; Claudia Renata dos Santos Barros; David Schlesinger; Debora Botequio Moretti; Dimas Tadeu Covas; Elaine Cristina Marqueze; Elaine Vieira Santos; Evandra Strazza Rodrigues; Heidge Fukumasu; Jayme Augusto de Souza-Neto; José Salvatore Leister Patané; Luiz Alcantara; Luiz Lehmann Coutinho; Maria Carolina Elias; Maurício Lacerda Nogueira; Rafael dos Santos Bezerra; Raul Machado Neto; Rejane Maria Tommasini Grotto; Ricardo Haddad; Sandra Coccuzzo Sampaio Vessoni; Simone Kashima; Svetoslav Nanev Slavov; Vincent Louis Viala
EPI_ISL_2445085	BIOFAST CENTRO SANTA CECÍLIA	Instituto Butantan	Antonio Jorge Martins; Claudia Renata dos Santos Barros; David Schlesinger; Debora Botequio Moretti; Dimas Tadeu Covas; Elaine Cristina Marqueze; Elaine Vieira Santos; Evandra Strazza Rodrigues; Heidge Fukumasu; Jayme Augusto de Souza-Neto; José Salvatore Leister Patané; Luiz Alcantara; Luiz Lehmann Coutinho; Maria Carolina Elias; Maurício Lacerda Nogueira; Rafael dos Santos Bezerra; Raul Machado Neto; Rejane Maria Tommasini Grotto; Ricardo Haddad; Sandra Coccuzzo Sampaio Vessoni; Simone Kashima; Svetoslav Nanev Slavov; Vincent Louis Viala
EPI_ISL_2445086	BIOFAST CENTRO SÉ	Instituto Butantan	Antonio Jorge Martins; Claudia Renata dos Santos Barros; David Schlesinger; Debora Botequio Moretti; Dimas Tadeu Covas; Elaine Cristina Marqueze; Elaine Vieira Santos; Evandra Strazza Rodrigues; Heidge Fukumasu; Jayme Augusto de Souza-Neto; José Salvatore Leister Patané; Luiz Alcantara; Luiz Lehmann Coutinho; Maria Carolina Elias; Maurício Lacerda Nogueira; Rafael dos Santos Bezerra; Raul Machado Neto; Rejane Maria Tommasini Grotto; Ricardo Haddad; Sandra Coccuzzo Sampaio Vessoni; Simone Kashima; Svetoslav Nanev Slavov; Vincent Louis Viala
EPI_ISL_2445094	BIOFAST LESTE GUAIANASES	Instituto Butantan	Antonio Jorge Martins; Claudia Renata dos Santos Barros; David Schlesinger; Debora Botequio Moretti; Dimas Tadeu Covas; Elaine Cristina Marqueze; Elaine Vieira Santos; Evandra Strazza Rodrigues; Heidge Fukumasu; Jayme Augusto de Souza-Neto; José Salvatore Leister Patané; Luiz Alcantara; Luiz Lehmann Coutinho; Maria Carolina Elias; Maurício Lacerda Nogueira; Rafael dos Santos Bezerra; Raul Machado Neto; Rejane Maria Tommasini Grotto; Ricardo Haddad; Sandra Coccuzzo Sampaio Vessoni; Simone Kashima; Svetoslav Nanev Slavov; Vincent Louis Viala
EPI_ISL_2445099, EPI_ISL_2445102	BIOFAST LESTE ITAIM PAULISTA	Instituto Butantan	Antonio Jorge Martins; Claudia Renata dos Santos Barros; David Schlesinger; Debora Botequio Moretti; Dimas Tadeu Covas; Elaine Cristina Marqueze; Elaine Vieira Santos; Evandra Strazza Rodrigues; Heidge Fukumasu; Jayme Augusto de Souza-Neto; José Salvatore Leister Patané; Luiz Alcantara; Luiz Lehmann Coutinho; Maria Carolina Elias; Maurício Lacerda Nogueira; Rafael dos Santos Bezerra; Raul Machado Neto; Rejane Maria Tommasini Grotto; Ricardo Haddad; Sandra Coccuzzo Sampaio Vessoni; Simone Kashima; Svetoslav Nanev Slavov; Vincent Louis Viala
EPI_ISL_2445088, EPI_ISL_2445090, EPI_ISL_2445092	BIOFAST LESTE ITAQUERA	Instituto Butantan	Antonio Jorge Martins; Claudia Renata dos Santos Barros; David Schlesinger; Debora Botequio Moretti; Dimas Tadeu Covas; Elaine Cristina Marqueze; Elaine Vieira Santos; Evandra Strazza Rodrigues; Heidge Fukumasu; Jayme Augusto de Souza-Neto; José Salvatore Leister Patané; Luiz Alcantara; Luiz Lehmann Coutinho; Maria Carolina Elias; Maurício Lacerda Nogueira; Rafael dos Santos Bezerra; Raul Machado Neto; Rejane Maria Tommasini Grotto; Ricardo Haddad; Sandra Coccuzzo Sampaio Vessoni; Simone Kashima; Svetoslav Nanev Slavov; Vincent Louis Viala
EPI_ISL_2445097, EPI_ISL_2445098	BIOFAST LESTE SÃO MIGUEL PAULISTA	Instituto Butantan	Antonio Jorge Martins; Claudia Renata dos Santos Barros; David Schlesinger; Debora Botequio Moretti; Dimas Tadeu Covas; Elaine Cristina Marqueze; Elaine Vieira Santos; Evandra Strazza Rodrigues; Heidge Fukumasu; Jayme Augusto de Souza-Neto; José Salvatore Leister Patané; Luiz Alcantara; Luiz Lehmann Coutinho; Maria Carolina Elias; Maurício Lacerda Nogueira; Rafael dos Santos Bezerra; Raul Machado Neto; Rejane Maria Tommasini Grotto; Ricardo Haddad; Sandra Coccuzzo Sampaio Vessoni; Simone Kashima; Svetoslav Nanev Slavov; Vincent Louis Viala
EPI_ISL_2445103	BIOFAST LESTE TIRADENTES	Instituto Butantan	Antonio Jorge Martins; Claudia Renata dos Santos Barros; David Schlesinger; Debora Botequio Moretti; Dimas Tadeu Covas; Elaine Cristina Marqueze; Elaine Vieira Santos; Evandra Strazza Rodrigues; Heidge Fukumasu; Jayme Augusto de Souza-Neto; José Salvatore Leister Patané; Luiz Alcantara; Luiz Lehmann Coutinho; Maria Carolina Elias; Maurício Lacerda Nogueira; Rafael dos Santos Bezerra; Raul Machado Neto; Rejane Maria Tommasini Grotto; Ricardo Haddad; Sandra Coccuzzo Sampaio Vessoni; Simone Kashima; Svetoslav Nanev Slavov; Vincent Louis Viala
EPI_ISL_2445105, EPI_ISL_2445107, EPI_ISL_2473811	BIOFAST NORTE SANTANA/JAÇANA/TUCURUVI	Instituto Butantan	Antonio Jorge Martins; Claudia Renata dos Santos Barros; David Schlesinger; Debora Botequio Moretti; Dimas Tadeu Covas; Elaine Cristina Marqueze; Elaine Vieira Santos; Evandra Strazza Rodrigues; Heidge Fukumasu; Jayme Augusto de Souza-Neto; José Salvatore Leister Patané; Luiz Alcantara; Luiz Lehmann Coutinho; Maria Carolina Elias; Maurício Lacerda Nogueira; Rafael dos Santos Bezerra; Raul Machado Neto; Rejane Maria Tommasini Grotto; Ricardo Haddad; Sandra Coccuzzo Sampaio Vessoni; Simone Kashima; Svetoslav Nanev Slavov; Vincent Louis Viala
EPI_ISL_2445108, EPI_ISL_2445109, EPI_ISL_2445112	BIOFAST SUL SANTO AMARO/CIDADE ADEMAR	Instituto Butantan	Antonio Jorge Martins; Claudia Renata dos Santos Barros; David Schlesinger; Debora Botequio Moretti; Dimas Tadeu Covas; Elaine Cristina Marqueze; Elaine Vieira Santos; Evandra Strazza Rodrigues; Heidge Fukumasu; Jayme Augusto de Souza-Neto; José Salvatore Leister Patané; Luiz Alcantara; Luiz Lehmann Coutinho; Maria Carolina Elias; Maurício Lacerda Nogueira; Rafael dos Santos Bezerra; Raul Machado Neto; Rejane Maria Tommasini Grotto; Ricardo Haddad; Sandra Coccuzzo Sampaio Vessoni; Simone Kashima; Svetoslav Nanev Slavov; Vincent Louis Viala
EPI_ISL_2373844	BIOMNIS LYON	CNR Virus des Infections Respiratoires - France SUD	Antonin Bal; Bruno Lina; Gregory Destras; Gwendolyne Burfin; Hadrien Regue; Laurence Josset; Martine Valette; Quentin Semanas
EPI_ISL_2421233	BOSTON HEART DIAGNOSTICS CORP	Wadsworth Center, New York State Department of Health	Alexis Russell; Catharine Prussing; Daryl M. Lamson; Erasmus Schneider; Erica Lasek-Nesselquist; John Kelly; Jonathan Plitnick; Kirsten St. George; Matthew Shudt; Melissa A Leisner; Navjot Singh
EPI_ISL_2484739	Baptist Health Medical Center	Center for Global Health, University of New Mexico Health Sciences Center	Amanda Novack; Darrell Dinwiddie; Daryl Domman; Dirk Haselow; Joshua L. Kennedy; Kurt Schwalm; Valerie Morley
EPI_ISL_2262754, EPI_ISL_2262815	Bayerisches Landesamt für Gesundheit und Lebensmittelsicherheit (LGL)	Robert Koch Institute	
EPI_ISL_2083061,	Baylor Scott & White-Temple	Baylor Scott & White-Temple	Ari Rao; Kimberly Walker; Linden Morales; Marcus Volz; Shelby Hendrickson; Shelby Johnson

EPI_ISL_2083080, EPI_ISL_2193240, EPI_ISL_2193265, EPI_ISL_2443472, EPI_ISL_2443507			
EPI_ISL_2204446	Berkeley Medical Center	WVU and Marshall University Combined Genomics Core Facilities	James Denvir; Peter Perrotta; Peter Stoilov; Ryan Percifield; Wesley Kimble
EPI_ISL_2105670, EPI_ISL_2105671	Biolab Diagnostic Laboratories	Biolab Diagnostic Laboratories	Ahmad Tibi; Amid Abdelnour; Badia Saddedin; Eiad Atwa; Issa Abu-Dayyeh; Lama Hussein; Shayma Ali
EPI_ISL_2698103, EPI_ISL_2698104	Biology, UFLA, Universidade Federal de Lavras	Biology, UFLA	Barcante, J.; Cherem, J.; Fernandes, G.; Luciano, P.; Melo, D.; Pylro, V.
EPI_ISL_1917098, EPI_ISL_1917111, EPI_ISL_1917129, EPI_ISL_2401290, EPI_ISL_2401348, EPI_ISL_2401349, EPI_ISL_2401558, EPI_ISL_3145466, EPI_ISL_3145467, EPI_ISL_3145468, EPI_ISL_3145469, EPI_ISL_3145470, EPI_ISL_3145471, EPI_ISL_3145473, EPI_ISL_3145474			
see above	BioneXt Lab	Laboratoire national de sante, Microbiology, Microbial Genomics Platform	Anke Wienecke-Baldacchino; Catherine Ragimbeau; Elodie Solarino; Fatu Djabi; Jessica Tapp; Lise Pignon; Raoul Salmon; Tamir Abdelrahman; Thibault Ferrandon; Virginie Jover
EPI_ISL_3494467, EPI_ISL_3494483	Boston University CTL	Boston University/National Emerging Infectious Disease Laboratories	Catherine Klapperich; Jacquelyn Turcinovic; John H. Connor; Lena Landeverde; Lynn Doucette-Stamm
EPI_ISL_3484463, EPI_ISL_3484465, EPI_ISL_3485505, EPI_ISL_3485949, EPI_ISL_3486879	British Columbia Centre For Disease Control	BCCDC Public Health Laboratory	Ana Pacagnella; Corrinne Ng; Dan Fornika; John Tyson; Kim Macdonald; Kimia Kamelian; Linda Hoang; Loretta Janz; Mel Krajden; Prystajecky Natalie; Robert Azana; Shannon Russell
EPI_ISL_2192739, EPI_ISL_2192747, EPI_ISL_2192775, EPI_ISL_2192804, EPI_ISL_2192833, EPI_ISL_2192842, EPI_ISL_2192857, EPI_ISL_2192880, EPI_ISL_2192882, EPI_ISL_2192928, EPI_ISL_2192931, EPI_ISL_2192947, EPI_ISL_2193017, EPI_ISL_2193033, EPI_ISL_2193040, EPI_ISL_2193055, EPI_ISL_2193060, EPI_ISL_2193067, EPI_ISL_2193107, EPI_ISL_2193152, EPI_ISL_2193179			
see above	Broad Institute Clinical Research Sequencing Platform	Infectious Disease Program, Broad Institute of Harvard and MIT	Adams, G.; B.L.; B.W.; Bauer, M.; Birren; Blumenstiel, B.; Brown, C.; Carter, A.; Chaluvadi, S.; D.J.; DeFelice, M.; DeRuff, K.; Dodge, S.; Gabriel, S.; Gallagher, G.; Gladden-Young, A.; Granger, B.; J.E.; K.J.; Lagerborg, K.; Larkin, K.; Lee, M.; Lemieux; Lennon, N.; Loreth, C.; Madoff, L.; McGovern, S.; Meldrim, J.; Normandin, E.; P.C.; Park; Pearlman, L.; Reilly, S.; Rudy, M.; Sabeti; Siddle; Smole, S.; Tomkins-Tinch, C.; Vicente, G.; and MacInnis
EPI_ISL_2341513, EPI_ISL_2496898, EPI_ISL_2496899, EPI_ISL_2496900, EPI_ISL_2496901, EPI_ISL_2496902, EPI_ISL_2496905, EPI_ISL_2496906, EPI_ISL_2496907, EPI_ISL_2496909, EPI_ISL_2496913, EPI_ISL_2497648, EPI_ISL_2497649, EPI_ISL_2497650, EPI_ISL_2497651, EPI_ISL_2497652, EPI_ISL_2497655, EPI_ISL_2497665, EPI_ISL_2497668, EPI_ISL_2500044, EPI_ISL_2500102, EPI_ISL_2500120, EPI_ISL_2500149, EPI_ISL_2500164, EPI_ISL_2500170, EPI_ISL_2500186, EPI_ISL_2500210			
see above	CA DPH Viral and Rickettsial Disease Laboratory	Chan-Zuckerberg Biohub	CZB Cliahub Consortium
EPI_ISL_2105135, EPI_ISL_2248730, EPI_ISL_2383987	CA-Los Angeles County Public Health Laboratory	Centers for Disease Control and Prevention Division of Viral Diseases, Pathogen Discovery	Alison Laufer Halpin; Ben L. Rambo-Martin; Clinton R. Paden; Dakota Howard; Darlene Wagner; Dave Wentworth; Dhwani Batra; Jasmine Padilla; Justin Lee; Katie Dillon; Krista Queen; Kristen Knipe; Kristine Lacek; Mark Burroughs; Matthew Schmerer; Mili Sheth; Peter Cook; Sam Shepard; Sarah Nobles; Shoshona Le; Suxiang Tong; Vivien Dugan; Yvette Unoarumhi
EPI_ISL_2445514, EPI_ISL_2473723, EPI_ISL_2473788, EPI_ISL_2473818	CADIP VILA REGINA FERNANDOPOLIS	Instituto Butantan	Antonio Jorge Martins; Claudia Renata dos Santos Barros; David Schlesinger; Debora Botequio Moretti; Dimas Tadeu Covas; Elaine Cristina Marqueze; Elaine Vieira Santos; Evandra Strazza Rodrigues; Heidge Fukumasu; Jayme Augusto de Souza-Neto; José Salvatore Leister Patané; Luiz Alcantara; Luiz Lehmann Coutinho; Maria Carolina Elias; Maurício Lacerda Nogueira; Rafael dos Santos Bezerra; Raul Machado Neto; Rejane Maria Tommasini Grotto; Ricardo Haddad; Sandra Coccuzzo Sampaio Vessoni; Simone Kashima; Svetoslav Nanev Slavov; Vincent Louis Viala
EPI_ISL_2825980, EPI_ISL_2825981, EPI_ISL_2826003, EPI_ISL_2826004, EPI_ISL_2882291, EPI_ISL_2890943, EPI_ISL_2891723, EPI_ISL_2891724, EPI_ISL_2891725, EPI_ISL_2891748, EPI_ISL_2891769, EPI_ISL_2891824, EPI_ISL_2891826, EPI_ISL_2891829, EPI_ISL_2891837, EPI_ISL_2891839, EPI_ISL_2891846, EPI_ISL_2891855, EPI_ISL_2891886, EPI_ISL_2891920, EPI_ISL_2891930, EPI_ISL_2891939, EPI_ISL_2891951, EPI_ISL_2891986, EPI_ISL_2892018, EPI_ISL_2892127, EPI_ISL_2892222, EPI_ISL_2892223, EPI_ISL_2892226, EPI_ISL_2892228, EPI_ISL_2892233, EPI_ISL_2892261, EPI_ISL_2892323, EPI_ISL_2892337, EPI_ISL_2892339, EPI_ISL_2892343, EPI_ISL_2892357, EPI_ISL_2892359, EPI_ISL_2921703, EPI_ISL_2921765, EPI_ISL_2921766, EPI_ISL_2921767, EPI_ISL_2921827, EPI_ISL_2921828, EPI_ISL_2921837, EPI_ISL_2921838, EPI_ISL_2921839, EPI_ISL_2921840, EPI_ISL_2921841, EPI_ISL_2921862, EPI_ISL_2921887, EPI_ISL_2921892, EPI_ISL_2921915, EPI_ISL_2921947, EPI_ISL_2921948, EPI_ISL_2921975, EPI_ISL_2921977, EPI_ISL_2921978, EPI_ISL_2921979, EPI_ISL_2921980, EPI_ISL_2922040, EPI_ISL_2922063, EPI_ISL_2922138, EPI_ISL_2922139, EPI_ISL_2922154, EPI_ISL_2922177, EPI_ISL_2922178, EPI_ISL_2922205, EPI_ISL_2922206, EPI_ISL_2922208, EPI_ISL_2922279, EPI_ISL_2923616, EPI_ISL_2976970, EPI_ISL_2976984, EPI_ISL_2976988, EPI_ISL_2977007, EPI_ISL_2977020, EPI_ISL_2977025, EPI_ISL_2977046, EPI_ISL_2977053, EPI_ISL_2977067, EPI_ISL_2977068, EPI_ISL_2977075, EPI_ISL_2977076, EPI_ISL_2977095, EPI_ISL_2977096, EPI_ISL_2977108, EPI_ISL_2977148, EPI_ISL_2977166, EPI_ISL_2977170, EPI_ISL_2977173, EPI_ISL_2977198, EPI_ISL_2977205, EPI_ISL_2977229, EPI_ISL_2977236, EPI_ISL_2977267, EPI_ISL_2977271, EPI_ISL_2977281, EPI_ISL_2977288, EPI_ISL_2977299, EPI_ISL_2977307, EPI_ISL_2977313, EPI_ISL_2977315, EPI_ISL_2977319, EPI_ISL_2977322, EPI_ISL_2977326, EPI_ISL_2977339, EPI_ISL_2977346, EPI_ISL_2977356, EPI_ISL_2977361, EPI_ISL_2977372, EPI_ISL_2977378, EPI_ISL_2977385, EPI_ISL_2977391, EPI_ISL_2977393, EPI_ISL_2978154, EPI_ISL_3243752, EPI_ISL_3243757, EPI_ISL_3243767, EPI_ISL_3243785, EPI_ISL_3243810, EPI_ISL_3243836, EPI_ISL_3243837, EPI_ISL_3243847, EPI_ISL_3243855, EPI_ISL_3243857, EPI_ISL_3243875, EPI_ISL_3243878, EPI_ISL_3243879, EPI_ISL_3243902, EPI_ISL_3243909, EPI_ISL_3243912, EPI_ISL_3243914, EPI_ISL_3243921, EPI_ISL_3243941, EPI_ISL_3243956, EPI_ISL_3243960, EPI_ISL_3244010, EPI_ISL_3244011, EPI_ISL_3244013, EPI_ISL_3244022, EPI_ISL_3244026, EPI_ISL_3244053, EPI_ISL_3244061, EPI_ISL_3244065, EPI_ISL_3244066, EPI_ISL_3244081, EPI_ISL_3244106, EPI_ISL_3244113, EPI_ISL_3244126, EPI_ISL_3244145, EPI_ISL_3244147, EPI_ISL_3244149, EPI_ISL_3244160, EPI_ISL_3244171, EPI_ISL_3244173, EPI_ISL_3244178, EPI_ISL_3244180, EPI_ISL_3244193, EPI_ISL_3244201, EPI_ISL_3244222, EPI_ISL_3244231, EPI_ISL_3244237, EPI_ISL_3244246, EPI_ISL_3244266, EPI_ISL_3244277, EPI_ISL_3244288, EPI_ISL_3244296, EPI_ISL_3244300, EPI_ISL_3244311, EPI_ISL_3244320, EPI_ISL_3244363, EPI_ISL_3244375, EPI_ISL_3244408, EPI_ISL_3244439, EPI_ISL_3244448			
see above	CDPH VBL	California Department of Public Health	CDPH-COVIDNet; Emily Smith on behalf of CDPH-COVIDNet and UCSD EXCITE lab; UCLA Technology Center for Genomics & Bioinformatics; UCSD EXCITE lab
EPI_ISL_2097252	CENTERS LAB NJ, LLC (DBA MEDLABS DIAGNOSTICS)	Wadsworth Center, New York State Department of Health	Alexis Russell; Catharine Prussing; Daryl M. Lamson; Erasmus Schneider; Erica Lasek-Nesselquist; John Kelly; Jonathan Plitnick; Kirsten St. George; Matthew Shudt; Melissa A Leisner; Navjot Singh
EPI_ISL_2344961, EPI_ISL_2344963, EPI_ISL_2344967, EPI_ISL_2344974, EPI_ISL_2344981, EPI_ISL_2344984, EPI_ISL_2344986, EPI_ISL_2344991, EPI_ISL_2344992, EPI_ISL_2344993, EPI_ISL_2344994, EPI_ISL_2344996, EPI_ISL_2344998			
see above	CENTRO DE APOIO EPIDEMIOLOGICO	Instituto Butantan / ESALQ-Piracicaba	Antonio Jorge Martins; Claudia Renata dos Santos Barros; David Schlesinger; Debora Botequio Moretti; Dimas Tadeu Covas; Elaine Cristina Marqueze; Elaine Vieira Santos; Evandra Strazza Rodrigues; Heidge Fukumasu; Jayme Augusto de Souza-Neto; José Salvatore Leister Patané; Luiz Alcantara; Luiz Lehmann Coutinho; Maria Carolina Elias; Maurício Lacerda Nogueira; Rafael dos Santos Bezerra; Raul Machado Neto; Rejane Maria Tommasini Grotto; Ricardo Haddad; Sandra Coccuzzo Sampaio Vessoni; Simone Kashima; Svetoslav Nanev Slavov; Vincent Louis Viala
EPI_ISL_2209086, EPI_ISL_2209721, EPI_ISL_2345689, EPI_ISL_2345735, EPI_ISL_2345779, EPI_ISL_2345830, EPI_ISL_2345832, EPI_ISL_2345878			
see above	CENTRO DE APOIO EPIDEMIOLOGICO	Instituto Butantan / Mendelics	Antonio Jorge Martins; Claudia Renata dos Santos Barros; David Schlesinger; Debora Botequio Moretti; Dimas Tadeu Covas; Elaine Cristina Marqueze; Elaine Vieira Santos; Evandra Strazza Rodrigues; Heidge Fukumasu; Jayme Augusto de Souza-Neto; José Salvatore Leister Patané; Luiz Alcantara; Luiz Lehmann Coutinho; Maria Carolina Elias; Maurício Lacerda Nogueira; Rafael dos Santos Bezerra; Raul Machado Neto; Rejane Maria Tommasini Grotto; Ricardo Haddad; Sandra Coccuzzo Sampaio Vessoni; Simone Kashima; Svetoslav Nanev Slavov; Vincent Louis Viala
EPI_ISL_3102309, EPI_ISL_3102329, EPI_ISL_3102511, EPI_ISL_3102512, EPI_ISL_3102515	CENTRO DE ATENDIMENTO PARA ENFRENTAMENTO A COVID 19	Analytical Competence Molecular Epidemiology Lab/ACME, Oswaldo Cruz Foundation, Ceara (FIOCRUZ CE)	Cleber Furtado Aksenen; Fabio Miyajima; Fernando Braga Stehling; Francisco Eder de Moura Lopes; Jamille Maria Mendes Bezerra; Joaquim César do Nascimento Sousa Junior; Pedro Miguel Carneiro Jeronimo; Suzana Porto Almeida e Lucas Delerino; Thais Ferreira de Oliveira; Thais de Oliveira Costa; Ticiane Cavalcante de Souza; Veridiana Pessoa Miyajima
EPI_ISL_2801337, EPI_ISL_2801341, EPI_ISL_2801343, EPI_ISL_2801344, EPI_ISL_2801346, EPI_ISL_2801363, EPI_ISL_3102270, EPI_ISL_3102277, EPI_ISL_3102519			
see above	CENTRO DE ATENDIMENTO PARA ENFRENTAMENTO AO COVID 19	Analytical Competence Molecular Epidemiology Lab/ACME, Oswaldo Cruz Foundation, Ceara (FIOCRUZ CE)	Cleber Furtado Aksenen; Cleber Furtado Aksenen e Suzana Porto Almeida; Fabio Miyajima; Fernando Braga Stehling; Francisco Eder de Moura Lopes; Jamille Maria Mendes Bezerra; Joaquim César do Nascimento Sousa Junior; Pedro Miguel Carneiro Jeronimo; Suzana Porto Almeida e Lucas Delerino; Thais Ferreira de Oliveira; Thais de Oliveira Costa; Ticiane Cavalcante de Souza; Veridiana Pessoa Miyajima
EPI_ISL_2493548	CENTRO DE ESPECIALIDADES DE AGUAI AGUAI	Instituto Butantan	Antonio Jorge Martins; Claudia Renata dos Santos Barros; David Schlesinger; Debora Botequio Moretti; Dimas Tadeu Covas; Elaine Cristina Marqueze; Elaine Vieira Santos; Evandra Strazza Rodrigues; Heidge Fukumasu; Jayme Augusto de Souza-Neto; José Salvatore Leister Patané; Luiz Alcantara; Luiz Lehmann Coutinho; Maria Carolina Elias; Maurício Lacerda Nogueira; Rafael dos Santos Bezerra; Raul Machado Neto; Rejane Maria Tommasini Grotto; Ricardo Haddad; Sandra Coccuzzo Sampaio Vessoni; Simone Kashima; Svetoslav Nanev Slavov; Vincent Louis Viala
EPI_ISL_2445551	CENTRO DE ESPECIALIDADES DE PRIMAVERA	Instituto Butantan	Antonio Jorge Martins; Claudia Renata dos Santos Barros; David Schlesinger; Debora Botequio Moretti; Dimas Tadeu Covas; Elaine Cristina Marqueze; Elaine Vieira Santos; Evandra Strazza Rodrigues; Heidge Fukumasu; Jayme Augusto de Souza-Neto; José Salvatore Leister Patané; Luiz Alcantara; Luiz Lehmann Coutinho; Maria Carolina Elias; Maurício Lacerda Nogueira; Rafael dos Santos Bezerra; Raul Machado Neto; Rejane Maria Tommasini Grotto; Ricardo Haddad; Sandra Coccuzzo Sampaio Vessoni; Simone Kashima; Svetoslav Nanev Slavov; Vincent Louis Viala
EPI_ISL_2210219	CENTRO DE ESPECIALIDADES DE PRIMAVERA	Instituto Butantan / Mendelics	Antonio Jorge Martins; Claudia Renata dos Santos Barros; David Schlesinger; Debora Botequio Moretti; Dimas Tadeu Covas; Elaine Cristina Marqueze; Elaine Vieira Santos; Evandra Strazza Rodrigues; Heidge Fukumasu; Jayme Augusto de Souza-Neto; José Salvatore Leister Patané; Luiz Alcantara; Luiz Lehmann Coutinho; Maria Carolina Elias; Maurício Lacerda Nogueira; Rafael dos Santos Bezerra; Raul Machado Neto; Rejane Maria Tommasini Grotto; Ricardo Haddad; Sandra Coccuzzo Sampaio Vessoni; Simone Kashima; Svetoslav Nanev Slavov; Vincent Louis Viala
EPI_ISL_2445539	CENTRO DE ESPECIALIDADES MARIAS DA GLORIA	Instituto Butantan	Antonio Jorge Martins; Claudia Renata dos Santos Barros; David Schlesinger; Debora Botequio Moretti; Dimas Tadeu Covas; Elaine Cristina Marqueze; Elaine Vieira Santos; Evandra Strazza Rodrigues; Heidge Fukumasu; Jayme Augusto de Souza-Neto; José Salvatore Leister Patané; Luiz Alcantara; Luiz Lehmann Coutinho; Maria Carolina Elias; Maurício Lacerda Nogueira; Rafael dos Santos Bezerra; Raul Machado Neto; Rejane Maria Tommasini Grotto; Ricardo Haddad; Sandra Coccuzzo Sampaio Vessoni; Simone Kashima; Svetoslav Nanev Slavov; Vincent Louis Viala
EPI_ISL_2493375	CENTRO DE ESPECIALIDDS MEDICAS IRMA LEOPOLDINA PIRASSUNUNGA	Instituto Butantan	Antonio Jorge Martins; Claudia Renata dos Santos Barros; David Schlesinger; Debora Botequio Moretti; Dimas Tadeu Covas; Elaine Cristina Marqueze; Elaine Vieira Santos; Evandra Strazza Rodrigues; Heidge Fukumasu; Jayme Augusto de Souza-Neto; José Salvatore Leister Patané; Luiz Alcantara; Luiz Lehmann Coutinho; Maria Carolina Elias; Maurício Lacerda Nogueira; Rafael dos Santos Bezerra; Raul Machado Neto; Rejane Maria Tommasini Grotto; Ricardo Haddad; Sandra Coccuzzo Sampaio Vessoni; Simone Kashima; Svetoslav Nanev Slavov; Vincent Louis Viala
EPI_ISL_2493436, EPI_ISL_2493437, EPI_ISL_2493438	CENTRO DE GENOMAS SUL	Instituto Butantan	Antonio Jorge Martins; Claudia Renata dos Santos Barros; David Schlesinger; Debora Botequio Moretti; Dimas Tadeu Covas; Elaine Cristina Marqueze; Elaine Vieira Santos; Evandra Strazza Rodrigues; Heidge Fukumasu; Jayme Augusto de Souza-Neto; José Salvatore Leister Patané; Luiz Alcantara; Luiz Lehmann Coutinho; Maria Carolina Elias; Maurício Lacerda Nogueira; Rafael dos Santos Bezerra; Raul Machado Neto; Rejane Maria Tommasini Grotto; Ricardo Haddad; Sandra Coccuzzo Sampaio Vessoni; Simone Kashima; Svetoslav Nanev Slavov; Vincent Louis Viala
EPI_ISL_2345815, EPI_ISL_2345860, EPI_ISL_2345865	CENTRO DE REFERENCIA DO IDOSO DR HUMBERTO MENDES DE CARVALHO	Instituto Butantan / Mendelics	Antonio Jorge Martins; Claudia Renata dos Santos Barros; David Schlesinger; Debora Botequio Moretti; Dimas Tadeu Covas; Elaine Cristina Marqueze; Elaine Vieira Santos; Evandra Strazza Rodrigues; Heidge Fukumasu; Jayme Augusto de Souza-Neto; José Salvatore Leister Patané; Luiz Alcantara; Luiz Lehmann Coutinho; Maria Carolina Elias; Maurício Lacerda Nogueira; Rafael dos Santos Bezerra; Raul Machado Neto; Rejane Maria Tommasini Grotto; Ricardo Haddad; Sandra Coccuzzo Sampaio Vessoni; Simone Kashima; Svetoslav Nanev Slavov; Vincent Louis Viala
EPI_ISL_2493394	CENTRO DE SAUDE	Instituto Butantan	Antonio Jorge Martins; Claudia Renata dos Santos Barros; David Schlesinger; Debora Botequio Moretti; Dimas Tadeu Covas; Elaine Cristina Marqueze; Elaine Vieira Santos; Evandra Strazza Rodrigues; Heidge Fukumasu; Jayme Augusto de Souza-Neto; José Salvatore Leister Patané; Luiz Alcantara; Luiz Lehmann Coutinho; Maria Carolina Elias; Maurício Lacerda Nogueira; Rafael dos Santos Bezerra; Raul Machado Neto; Rejane Maria Tommasini Grotto; Ricardo Haddad; Sandra Coccuzzo Sampaio Vessoni; Simone Kashima; Svetoslav Nanev Slavov; Vincent Louis Viala
EPI_ISL_2344852	CENTRO DE SAUDE DE ANALANDIA	Instituto Butantan / ESALQ-Piracicaba	Antonio Jorge Martins; Claudia Renata dos Santos Barros; David Schlesinger; Debora Botequio Moretti; Dimas Tadeu Covas; Elaine Cristina Marqueze; Elaine Vieira Santos; Evandra Strazza Rodrigues; Heidge Fukumasu; Jayme Augusto de Souza-Neto; José Salvatore Leister Patané; Luiz Alcantara; Luiz Lehmann Coutinho; Maria Carolina Elias; Maurício Lacerda Nogueira; Rafael dos Santos Bezerra; Raul Machado Neto; Rejane Maria Tommasini Grotto; Ricardo Haddad; Sandra Coccuzzo Sampaio Vessoni; Simone Kashima; Svetoslav Nanev Slavov; Vincent Louis Viala
EPI_ISL_2345222	CENTRO DE SAUDE DE AVAI	Instituto Butantan / ESALQ-Piracicaba	Antonio Jorge Martins; Claudia Renata dos Santos Barros; David Schlesinger; Debora Botequio Moretti; Dimas Tadeu Covas; Elaine Cristina Marqueze; Elaine Vieira Santos; Evandra Strazza Rodrigues; Heidge Fukumasu; Jayme Augusto de Souza-Neto; José Salvatore Leister Patané; Luiz Alcantara; Luiz Lehmann Coutinho; Maria Carolina Elias; Maurício Lacerda Nogueira; Rafael dos Santos Bezerra; Raul Machado Neto; Rejane Maria Tommasini Grotto; Ricardo Haddad; Sandra Coccuzzo Sampaio Vessoni; Simone Kashima; Svetoslav Nanev Slavov; Vincent Louis Viala
EPI_ISL_1967212	CENTRO DE SAUDE DE BARBOSA	Instituto Butantan / Mendelics	Antonio Jorge Martins; Bianca Cechetto Carlos. Mendelics: Bibiana Santos; Claudia Renata dos Santos Barros; Cíntia Bittar; David Schlesinger. Hemocentro Ribeirão Preto: Simone Kashima; Debora Botequio Moretti; Elaine Cristina Marqueze; Elaine Vieira dos Santos; Elisangela Chicaroni Mattos; Erika Freitas; Evandra Strazza Rodrigues; Felipe Allan da Silva da Costa; Flavia Aburjaile; Fábio Sossai Possebon; Guilherme Campos; Guilherme Targino Valente; Heidge Fukumasu. USP-Botucatu: Rejane Maria Tommasini Grotto; Helena Lage Ferreira; Instituto Butantan: Dimas Tadeu Covas; Jardelina de Souza Todao Bernardino; Jayme A. Souza-Neto; Jessika Cristina Chagas Lesbon; Jorge A. Petroli Marchesi; José Salvatore Leister Patané; João Paulo Kitajima; João Pessoa Araújo Jr.; Leila Sabrina Ullmann; Loyze Paola Oliveira de Lima; Luiz Aurelio de Campos Crispin. Centro de Genômica Funcional da ESALQ: Luiz Lehmann Coutinho; Luiz Carlos Junior de Alcantara; Lívia Sacchetto; Maisa C. Pereira Parra; Maria Carolina Elias; Marta Giovanetti; Marília Moraes; Maurício Lacerda Nogueira. Prefeitura de Sao Paulo: Melissa Palmieri.; Patricia Akemi Assato; Paula Rahal; Paulo Inacio da Costa; Rafael dos Santos Bezerra; Raquel de Lello Rocha Campos Cassano. NGS Soluções Genômicas: Pilar Drummond Sampaio Corrêa Mariani. FZEA-USP Pirassununga: Mirele Daiana Poleti; Raul Machado Neto; Ricardo Augusto Brassaloti; Ricardo Haddad; Rodrigo Tocantins Calado. FAMERP-SJRP: Cecília Artico Banho; Sandra Coccuzzo Sampaio; Svetoslav Nanev Slavov; Vagner Fonseca; Vincent Louis Viala

EPI_ISL_2473820	CENTRO DE SAUDE DE CANITAR	Instituto Butantan	Antonio Jorge Martins; Claudia Renata dos Santos Barros; David Schlesinger; Debora Botequio Moretti; Dimas Tadeu Covas; Elaine Cristina Marqueze; Elaine Vieira Santos; Evandra Strazza Rodrigues; Heidge Fukumasu; Jayme Augusto de Souza-Neto; José Salvatore Leister Patané; Luiz Alcantara; Luiz Lehmann Coutinho; Maria Carolina Elias; Maurício Lacerda Nogueira; Rafael dos Santos Bezerra; Raul Machado Neto; Rejane Maria Tommasini Grotto; Ricardo Haddad; Sandra Coccuzzo Sampaio Vessoni; Simone Kashima; Svetoslav Nanev Slavov; Vincent Louis Viala
EPI_ISL_2445197	CENTRO DE SAUDE DE CRUZALIA	Instituto Butantan	Antonio Jorge Martins; Claudia Renata dos Santos Barros; David Schlesinger; Debora Botequio Moretti; Dimas Tadeu Covas; Elaine Cristina Marqueze; Elaine Vieira Santos; Evandra Strazza Rodrigues; Heidge Fukumasu; Jayme Augusto de Souza-Neto; José Salvatore Leister Patané; Luiz Alcantara; Luiz Lehmann Coutinho; Maria Carolina Elias; Maurício Lacerda Nogueira; Rafael dos Santos Bezerra; Raul Machado Neto; Rejane Maria Tommasini Grotto; Ricardo Haddad; Sandra Coccuzzo Sampaio Vessoni; Simone Kashima; Svetoslav Nanev Slavov; Vincent Louis Viala
EPI_ISL_2445153	CENTRO DE SAUDE DE FLORIDA PAULISTA	Instituto Butantan	Antonio Jorge Martins; Claudia Renata dos Santos Barros; David Schlesinger; Debora Botequio Moretti; Dimas Tadeu Covas; Elaine Cristina Marqueze; Elaine Vieira Santos; Evandra Strazza Rodrigues; Heidge Fukumasu; Jayme Augusto de Souza-Neto; José Salvatore Leister Patané; Luiz Alcantara; Luiz Lehmann Coutinho; Maria Carolina Elias; Maurício Lacerda Nogueira; Rafael dos Santos Bezerra; Raul Machado Neto; Rejane Maria Tommasini Grotto; Ricardo Haddad; Sandra Coccuzzo Sampaio Vessoni; Simone Kashima; Svetoslav Nanev Slavov; Vincent Louis Viala
EPI_ISL_2473713	CENTRO DE SAUDE DE IBIRAREMA	Instituto Butantan	Antonio Jorge Martins; Claudia Renata dos Santos Barros; David Schlesinger; Debora Botequio Moretti; Dimas Tadeu Covas; Elaine Cristina Marqueze; Elaine Vieira Santos; Evandra Strazza Rodrigues; Heidge Fukumasu; Jayme Augusto de Souza-Neto; José Salvatore Leister Patané; Luiz Alcantara; Luiz Lehmann Coutinho; Maria Carolina Elias; Maurício Lacerda Nogueira; Rafael dos Santos Bezerra; Raul Machado Neto; Rejane Maria Tommasini Grotto; Ricardo Haddad; Sandra Coccuzzo Sampaio Vessoni; Simone Kashima; Svetoslav Nanev Slavov; Vincent Louis Viala
EPI_ISL_2445428, EPI_ISL_2445429, EPI_ISL_2445430, EPI_ISL_2445431, EPI_ISL_2445433, EPI_ISL_2445434, EPI_ISL_2493586			
see above	CENTRO DE SAUDE DE LUCELIA	Instituto Butantan	Antonio Jorge Martins; Claudia Renata dos Santos Barros; David Schlesinger; Debora Botequio Moretti; Dimas Tadeu Covas; Elaine Cristina Marqueze; Elaine Vieira Santos; Evandra Strazza Rodrigues; Heidge Fukumasu; Jayme Augusto de Souza-Neto; José Salvatore Leister Patané; Luiz Alcantara; Luiz Lehmann Coutinho; Maria Carolina Elias; Maurício Lacerda Nogueira; Rafael dos Santos Bezerra; Raul Machado Neto; Rejane Maria Tommasini Grotto; Ricardo Haddad; Sandra Coccuzzo Sampaio Vessoni; Simone Kashima; Svetoslav Nanev Slavov; Vincent Louis Viala
EPI_ISL_2209593, EPI_ISL_2345657, EPI_ISL_2345702, EPI_ISL_2345753	CENTRO DE SAUDE DE MARACAI	Instituto Butantan / Mendelics	Antonio Jorge Martins; Claudia Renata dos Santos Barros; David Schlesinger; Debora Botequio Moretti; Dimas Tadeu Covas; Elaine Cristina Marqueze; Elaine Vieira Santos; Evandra Strazza Rodrigues; Heidge Fukumasu; Jayme Augusto de Souza-Neto; José Salvatore Leister Patané; Luiz Alcantara; Luiz Lehmann Coutinho; Maria Carolina Elias; Maurício Lacerda Nogueira; Rafael dos Santos Bezerra; Raul Machado Neto; Rejane Maria Tommasini Grotto; Ricardo Haddad; Sandra Coccuzzo Sampaio Vessoni; Simone Kashima; Svetoslav Nanev Slavov; Vincent Louis Viala
EPI_ISL_2345236	CENTRO DE SAUDE DE OCAUCU	Instituto Butantan / ESALQ-Piracicaba	Antonio Jorge Martins; Claudia Renata dos Santos Barros; David Schlesinger; Debora Botequio Moretti; Dimas Tadeu Covas; Elaine Cristina Marqueze; Elaine Vieira Santos; Evandra Strazza Rodrigues; Heidge Fukumasu; Jayme Augusto de Souza-Neto; José Salvatore Leister Patané; Luiz Alcantara; Luiz Lehmann Coutinho; Maria Carolina Elias; Maurício Lacerda Nogueira; Rafael dos Santos Bezerra; Raul Machado Neto; Rejane Maria Tommasini Grotto; Ricardo Haddad; Sandra Coccuzzo Sampaio Vessoni; Simone Kashima; Svetoslav Nanev Slavov; Vincent Louis Viala
EPI_ISL_2345192, EPI_ISL_2345201	CENTRO DE SAUDE DE OSVALDO CRUZ	Instituto Butantan / ESALQ-Piracicaba	Antonio Jorge Martins; Claudia Renata dos Santos Barros; David Schlesinger; Debora Botequio Moretti; Dimas Tadeu Covas; Elaine Cristina Marqueze; Elaine Vieira Santos; Evandra Strazza Rodrigues; Heidge Fukumasu; Jayme Augusto de Souza-Neto; José Salvatore Leister Patané; Luiz Alcantara; Luiz Lehmann Coutinho; Maria Carolina Elias; Maurício Lacerda Nogueira; Rafael dos Santos Bezerra; Raul Machado Neto; Rejane Maria Tommasini Grotto; Ricardo Haddad; Sandra Coccuzzo Sampaio Vessoni; Simone Kashima; Svetoslav Nanev Slavov; Vincent Louis Viala
EPI_ISL_2473744, EPI_ISL_2473745	CENTRO DE SAUDE DE PIRAJUI	Instituto Butantan	Antonio Jorge Martins; Claudia Renata dos Santos Barros; David Schlesinger; Debora Botequio Moretti; Dimas Tadeu Covas; Elaine Cristina Marqueze; Elaine Vieira Santos; Evandra Strazza Rodrigues; Heidge Fukumasu; Jayme Augusto de Souza-Neto; José Salvatore Leister Patané; Luiz Alcantara; Luiz Lehmann Coutinho; Maria Carolina Elias; Maurício Lacerda Nogueira; Rafael dos Santos Bezerra; Raul Machado Neto; Rejane Maria Tommasini Grotto; Ricardo Haddad; Sandra Coccuzzo Sampaio Vessoni; Simone Kashima; Svetoslav Nanev Slavov; Vincent Louis Viala
EPI_ISL_2445193, EPI_ISL_2445194	CENTRO DE SAUDE DE RIBEIRAO DO SUL	Instituto Butantan	Antonio Jorge Martins; Claudia Renata dos Santos Barros; David Schlesinger; Debora Botequio Moretti; Dimas Tadeu Covas; Elaine Cristina Marqueze; Elaine Vieira Santos; Evandra Strazza Rodrigues; Heidge Fukumasu; Jayme Augusto de Souza-Neto; José Salvatore Leister Patané; Luiz Alcantara; Luiz Lehmann Coutinho; Maria Carolina Elias; Maurício Lacerda Nogueira; Rafael dos Santos Bezerra; Raul Machado Neto; Rejane Maria Tommasini Grotto; Ricardo Haddad; Sandra Coccuzzo Sampaio Vessoni; Simone Kashima; Svetoslav Nanev Slavov; Vincent Louis Viala
EPI_ISL_2209288, EPI_ISL_2345730, EPI_ISL_2345775, EPI_ISL_2362246	CENTRO DE SAUDE DR JOAO JOAQUIM FERREIRA CRISTAIS PAULISTA	Instituto Butantan / Mendelics	Antonio Jorge Martins; Claudia Renata dos Santos Barros; David Schlesinger; Debora Botequio Moretti; Dimas Tadeu Covas; Elaine Cristina Marqueze; Elaine Vieira Santos; Evandra Strazza Rodrigues; Heidge Fukumasu; Jayme Augusto de Souza-Neto; José Salvatore Leister Patané; Luiz Alcantara; Luiz Lehmann Coutinho; Maria Carolina Elias; Maurício Lacerda Nogueira; Rafael dos Santos Bezerra; Raul Machado Neto; Rejane Maria Tommasini Grotto; Ricardo Haddad; Sandra Coccuzzo Sampaio Vessoni; Simone Kashima; Svetoslav Nanev Slavov; Vincent Louis Viala
EPI_ISL_2493354	CENTRO DE SAUDE DR JOSE AGOSTINHO NOGUEIRA PAULICEIA	Instituto Butantan	Antonio Jorge Martins; Claudia Renata dos Santos Barros; David Schlesinger; Debora Botequio Moretti; Dimas Tadeu Covas; Elaine Cristina Marqueze; Elaine Vieira Santos; Evandra Strazza Rodrigues; Heidge Fukumasu; Jayme Augusto de Souza-Neto; José Salvatore Leister Patané; Luiz Alcantara; Luiz Lehmann Coutinho; Maria Carolina Elias; Maurício Lacerda Nogueira; Rafael dos Santos Bezerra; Raul Machado Neto; Rejane Maria Tommasini Grotto; Ricardo Haddad; Sandra Coccuzzo Sampaio Vessoni; Simone Kashima; Svetoslav Nanev Slavov; Vincent Louis Viala
EPI_ISL_2493377, EPI_ISL_2493378, EPI_ISL_2494150	CENTRO DE SAUDE DR MARIO DIAS DE AGUIAR CAPIVARI	Instituto Butantan	Antonio Jorge Martins; Claudia Renata dos Santos Barros; David Schlesinger; Debora Botequio Moretti; Dimas Tadeu Covas; Elaine Cristina Marqueze; Elaine Vieira Santos; Evandra Strazza Rodrigues; Heidge Fukumasu; Jayme Augusto de Souza-Neto; José Salvatore Leister Patané; Luiz Alcantara; Luiz Lehmann Coutinho; Maria Carolina Elias; Maurício Lacerda Nogueira; Rafael dos Santos Bezerra; Raul Machado Neto; Rejane Maria Tommasini Grotto; Ricardo Haddad; Sandra Coccuzzo Sampaio Vessoni; Simone Kashima; Svetoslav Nanev Slavov; Vincent Louis Viala
EPI_ISL_2445441, EPI_ISL_2445442, EPI_ISL_2445445, EPI_ISL_2445450, EPI_ISL_2445453	CENTRO DE SAUDE DR NELSON DA CUNHA BASTOS	Instituto Butantan	Antonio Jorge Martins; Claudia Renata dos Santos Barros; David Schlesinger; Debora Botequio Moretti; Dimas Tadeu Covas; Elaine Cristina Marqueze; Elaine Vieira Santos; Evandra Strazza Rodrigues; Heidge Fukumasu; Jayme Augusto de Souza-Neto; José Salvatore Leister Patané; Luiz Alcantara; Luiz Lehmann Coutinho; Maria Carolina Elias; Maurício Lacerda Nogueira; Rafael dos Santos Bezerra; Raul Machado Neto; Rejane Maria Tommasini Grotto; Ricardo Haddad; Sandra Coccuzzo Sampaio Vessoni; Simone Kashima; Svetoslav Nanev Slavov; Vincent Louis Viala
EPI_ISL_2344832, EPI_ISL_2344873, EPI_ISL_2344904, EPI_ISL_2344905, EPI_ISL_2344907	CENTRO DE SAUDE DR RENATO DE CARVALHO RIBEIRO ANGATUBA	Instituto Butantan / ESALQ-Piracicaba	Antonio Jorge Martins; Claudia Renata dos Santos Barros; David Schlesinger; Debora Botequio Moretti; Dimas Tadeu Covas; Elaine Cristina Marqueze; Elaine Vieira Santos; Evandra Strazza Rodrigues; Heidge Fukumasu; Jayme Augusto de Souza-Neto; José Salvatore Leister Patané; Luiz Alcantara; Luiz Lehmann Coutinho; Maria Carolina Elias; Maurício Lacerda Nogueira; Rafael dos Santos Bezerra; Raul Machado Neto; Rejane Maria Tommasini Grotto; Ricardo Haddad; Sandra Coccuzzo Sampaio Vessoni; Simone Kashima; Svetoslav Nanev Slavov; Vincent Louis Viala
EPI_ISL_2473787	CENTRO DE SAUDE II CARDOSO	Instituto Butantan	Antonio Jorge Martins; Claudia Renata dos Santos Barros; David Schlesinger; Debora Botequio Moretti; Dimas Tadeu Covas; Elaine Cristina Marqueze; Elaine Vieira Santos; Evandra Strazza Rodrigues; Heidge Fukumasu; Jayme Augusto de Souza-Neto; José Salvatore Leister Patané; Luiz Alcantara; Luiz Lehmann Coutinho; Maria Carolina Elias; Maurício Lacerda Nogueira; Rafael dos Santos Bezerra; Raul Machado Neto; Rejane Maria Tommasini Grotto; Ricardo Haddad; Sandra Coccuzzo Sampaio Vessoni; Simone Kashima; Svetoslav Nanev Slavov; Vincent Louis Viala
EPI_ISL_2445529, EPI_ISL_2445531, EPI_ISL_2445533	CENTRO DE SAUDE II DE POTIRENDABA CESARINO BENFATTI	Instituto Butantan	Antonio Jorge Martins; Claudia Renata dos Santos Barros; David Schlesinger; Debora Botequio Moretti; Dimas Tadeu Covas; Elaine Cristina Marqueze; Elaine Vieira Santos; Evandra Strazza Rodrigues; Heidge Fukumasu; Jayme Augusto de Souza-Neto; José Salvatore Leister Patané; Luiz Alcantara; Luiz Lehmann Coutinho; Maria Carolina Elias; Maurício Lacerda Nogueira; Rafael dos Santos Bezerra; Raul Machado Neto; Rejane Maria Tommasini Grotto; Ricardo Haddad; Sandra Coccuzzo Sampaio Vessoni; Simone Kashima; Svetoslav Nanev Slavov; Vincent Louis Viala
EPI_ISL_2493643	CENTRO DE SAUDE II DR GABRIEL MESQUITA VARGEM GDE DO SUL	Instituto Butantan	Antonio Jorge Martins; Claudia Renata dos Santos Barros; David Schlesinger; Debora Botequio Moretti; Dimas Tadeu Covas; Elaine Cristina Marqueze; Elaine Vieira Santos; Evandra Strazza Rodrigues; Heidge Fukumasu; Jayme Augusto de Souza-Neto; José Salvatore Leister Patané; Luiz Alcantara; Luiz Lehmann Coutinho; Maria Carolina Elias; Maurício Lacerda Nogueira; Rafael dos Santos Bezerra; Raul Machado Neto; Rejane Maria Tommasini Grotto; Ricardo Haddad; Sandra Coccuzzo Sampaio Vessoni; Simone Kashima; Svetoslav Nanev Slavov; Vincent Louis Viala
EPI_ISL_2345650, EPI_ISL_2345695, EPI_ISL_2362262	CENTRO DE SAUDE II DR GABRIEL MESQUITA VARGEM GDE DO SUL	Instituto Butantan / Mendelics	Antonio Jorge Martins; Claudia Renata dos Santos Barros; David Schlesinger; Debora Botequio Moretti; Dimas Tadeu Covas; Elaine Cristina Marqueze; Elaine Vieira Santos; Evandra Strazza Rodrigues; Heidge Fukumasu; Jayme Augusto de Souza-Neto; José Salvatore Leister Patané; Luiz Alcantara; Luiz Lehmann Coutinho; Maria Carolina Elias; Maurício Lacerda Nogueira; Rafael dos Santos Bezerra; Raul Machado Neto; Rejane Maria Tommasini Grotto; Ricardo Haddad; Sandra Coccuzzo Sampaio Vessoni; Simone Kashima; Svetoslav Nanev Slavov; Vincent Louis Viala
EPI_ISL_2494160	CENTRO DE SAUDE II DR JOSE DE FELIPE E S PINHAL SP	Instituto Butantan	Antonio Jorge Martins; Claudia Renata dos Santos Barros; David Schlesinger; Debora Botequio Moretti; Dimas Tadeu Covas; Elaine Cristina Marqueze; Elaine Vieira Santos; Evandra Strazza Rodrigues; Heidge Fukumasu; Jayme Augusto de Souza-Neto; José Salvatore Leister Patané; Luiz Alcantara; Luiz Lehmann Coutinho; Maria Carolina Elias; Maurício Lacerda Nogueira; Rafael dos Santos Bezerra; Raul Machado Neto; Rejane Maria Tommasini Grotto; Ricardo Haddad; Sandra Coccuzzo Sampaio Vessoni; Simone Kashima; Svetoslav Nanev Slavov; Vincent Louis Viala
EPI_ISL_2445317, EPI_ISL_2493537, EPI_ISL_2493540, EPI_ISL_2493542, EPI_ISL_2493571, EPI_ISL_2493572, EPI_ISL_2493574, EPI_ISL_2493575, EPI_ISL_2493576, EPI_ISL_2493578, EPI_ISL_2493580, EPI_ISL_2493582, EPI_ISL_2493583, EPI_ISL_2493675, EPI_ISL_2493676, EPI_ISL_2493680, EPI_ISL_2493682, EPI_ISL_2493683			
see above	CENTRO DE SAUDE II IBITINGA	Instituto Butantan	Antonio Jorge Martins; Claudia Renata dos Santos Barros; David Schlesinger; Debora Botequio Moretti; Dimas Tadeu Covas; Elaine Cristina Marqueze; Elaine Vieira Santos; Evandra Strazza Rodrigues; Heidge Fukumasu; Jayme Augusto de Souza-Neto; José Salvatore Leister Patané; Luiz Alcantara; Luiz Lehmann Coutinho; Maria Carolina Elias; Maurício Lacerda Nogueira; Rafael dos Santos Bezerra; Raul Machado Neto; Rejane Maria Tommasini Grotto; Ricardo Haddad; Sandra Coccuzzo Sampaio Vessoni; Simone Kashima; Svetoslav Nanev Slavov; Vincent Louis Viala
EPI_ISL_2209353, EPI_ISL_2209368, EPI_ISL_2209527, EPI_ISL_2345644, EPI_ISL_2345649, EPI_ISL_2345724, EPI_ISL_2345743, EPI_ISL_2345747, EPI_ISL_2345766, EPI_ISL_2345791, EPI_ISL_2345861			
see above	CENTRO DE SAUDE II IBITINGA	Instituto Butantan / Mendelics	Antonio Jorge Martins; Claudia Renata dos Santos Barros; David Schlesinger; Debora Botequio Moretti; Dimas Tadeu Covas; Elaine Cristina Marqueze; Elaine Vieira Santos; Evandra Strazza Rodrigues; Heidge Fukumasu; Jayme Augusto de Souza-Neto; José Salvatore Leister Patané; Luiz Alcantara; Luiz Lehmann Coutinho; Maria Carolina Elias; Maurício Lacerda Nogueira; Rafael dos Santos Bezerra; Raul Machado Neto; Rejane Maria Tommasini Grotto; Ricardo Haddad; Sandra Coccuzzo Sampaio Vessoni; Simone Kashima; Svetoslav Nanev Slavov; Vincent Louis Viala
EPI_ISL_2493336, EPI_ISL_2493337, EPI_ISL_2493338, EPI_ISL_2493339, EPI_ISL_2493344, EPI_ISL_2493347	CENTRO DE SAUDE II JUNQUEIROPOLIS	Instituto Butantan	Antonio Jorge Martins; Claudia Renata dos Santos Barros; David Schlesinger; Debora Botequio Moretti; Dimas Tadeu Covas; Elaine Cristina Marqueze; Elaine Vieira Santos; Evandra Strazza Rodrigues; Heidge Fukumasu; Jayme Augusto de Souza-Neto; José Salvatore Leister Patané; Luiz Alcantara; Luiz Lehmann Coutinho; Maria Carolina Elias; Maurício Lacerda Nogueira; Rafael dos Santos Bezerra; Raul Machado Neto; Rejane Maria Tommasini Grotto; Ricardo Haddad; Sandra Coccuzzo Sampaio Vessoni; Simone Kashima; Svetoslav Nanev Slavov; Vincent Louis Viala
EPI_ISL_2445603	CENTRO DE SAUDE II SAO MIGUEL ARCANJO	Instituto Butantan	Antonio Jorge Martins; Claudia Renata dos Santos Barros; David Schlesinger; Debora Botequio Moretti; Dimas Tadeu Covas; Elaine Cristina Marqueze; Elaine Vieira Santos; Evandra Strazza Rodrigues; Heidge Fukumasu; Jayme Augusto de Souza-Neto; José Salvatore Leister Patané; Luiz Alcantara; Luiz Lehmann Coutinho; Maria Carolina Elias; Maurício Lacerda Nogueira; Rafael dos Santos Bezerra; Raul Machado Neto; Rejane Maria Tommasini Grotto; Ricardo Haddad; Sandra Coccuzzo Sampaio Vessoni; Simone Kashima; Svetoslav Nanev Slavov; Vincent Louis Viala
EPI_ISL_2493263	CENTRO DE SAUDE III AFFONSO LUZZI SANTA CRUZ DAS PALMEIRAS	Instituto Butantan	Antonio Jorge Martins; Claudia Renata dos Santos Barros; David Schlesinger; Debora Botequio Moretti; Dimas Tadeu Covas; Elaine Cristina Marqueze; Elaine Vieira Santos; Evandra Strazza Rodrigues; Heidge Fukumasu; Jayme Augusto de Souza-Neto; José Salvatore Leister Patané; Luiz Alcantara; Luiz Lehmann Coutinho; Maria Carolina Elias; Maurício Lacerda Nogueira; Rafael dos Santos Bezerra; Raul Machado Neto; Rejane Maria Tommasini Grotto; Ricardo Haddad; Sandra Coccuzzo Sampaio Vessoni; Simone Kashima; Svetoslav Nanev Slavov; Vincent Louis Viala
EPI_ISL_2209307, EPI_ISL_2209312, EPI_ISL_2345792, EPI_ISL_2362260	CENTRO DE SAUDE III AFFONSO LUZZI SANTA CRUZ DAS PALMEIRAS	Instituto Butantan / Mendelics	Antonio Jorge Martins; Claudia Renata dos Santos Barros; David Schlesinger; Debora Botequio Moretti; Dimas Tadeu Covas; Elaine Cristina Marqueze; Elaine Vieira Santos; Evandra Strazza Rodrigues; Heidge Fukumasu; Jayme Augusto de Souza-Neto; José Salvatore Leister Patané; Luiz Alcantara; Luiz Lehmann Coutinho; Maria Carolina Elias; Maurício Lacerda Nogueira; Rafael dos Santos Bezerra; Raul Machado Neto; Rejane Maria Tommasini Grotto; Ricardo Haddad; Sandra Coccuzzo Sampaio Vessoni; Simone Kashima; Svetoslav Nanev Slavov; Vincent Louis Viala
EPI_ISL_2345195	CENTRO DE SAUDE III ALTO ALEGRE	Instituto Butantan / ESALQ-Piracicaba	Antonio Jorge Martins; Claudia Renata dos Santos Barros; David Schlesinger; Debora Botequio Moretti; Dimas Tadeu Covas; Elaine Cristina Marqueze; Elaine Vieira Santos; Evandra Strazza Rodrigues; Heidge Fukumasu; Jayme Augusto de Souza-Neto; José Salvatore Leister Patané; Luiz Alcantara; Luiz Lehmann Coutinho; Maria Carolina Elias; Maurício Lacerda Nogueira; Rafael dos Santos Bezerra; Raul Machado Neto; Rejane Maria Tommasini Grotto; Ricardo Haddad; Sandra Coccuzzo Sampaio Vessoni; Simone Kashima; Svetoslav Nanev Slavov; Vincent Louis Viala
EPI_ISL_2493539, EPI_ISL_2493544, EPI_ISL_2493547, EPI_ISL_2493570	CENTRO DE SAUDE III BORBOREMA	Instituto Butantan	Antonio Jorge Martins; Claudia Renata dos Santos Barros; David Schlesinger; Debora Botequio Moretti; Dimas Tadeu Covas; Elaine Cristina Marqueze; Elaine Vieira Santos; Evandra Strazza Rodrigues; Heidge Fukumasu; Jayme Augusto de Souza-Neto; José Salvatore Leister Patané; Luiz Alcantara; Luiz Lehmann Coutinho; Maria Carolina Elias; Maurício Lacerda Nogueira; Rafael dos Santos Bezerra; Raul Machado Neto; Rejane Maria Tommasini Grotto; Ricardo Haddad; Sandra Coccuzzo Sampaio Vessoni; Simone Kashima; Svetoslav Nanev Slavov; Vincent Louis Viala
EPI_ISL_2494317, EPI_ISL_2494332	CENTRO DE SAUDE III DE AGUAS DE SAO PEDRO	Instituto Butantan	Antonio Jorge Martins; Claudia Renata dos Santos Barros; David Schlesinger; Debora Botequio Moretti; Dimas Tadeu Covas; Elaine Cristina Marqueze; Elaine Vieira Santos; Evandra Strazza Rodrigues; Heidge Fukumasu; Jayme Augusto de Souza-Neto; José Salvatore Leister Patané; Luiz Alcantara; Luiz Lehmann Coutinho; Maria Carolina Elias; Maurício Lacerda Nogueira; Rafael dos Santos Bezerra; Raul Machado Neto; Rejane Maria Tommasini Grotto; Ricardo Haddad; Sandra Coccuzzo Sampaio Vessoni; Simone Kashima; Svetoslav Nanev Slavov; Vincent Louis Viala
EPI_ISL_2345188	CENTRO DE SAUDE III DE BOCAINA	Instituto Butantan / ESALQ-Piracicaba	Antonio Jorge Martins; Claudia Renata dos Santos Barros; David Schlesinger; Debora Botequio Moretti; Dimas Tadeu Covas; Elaine Cristina Marqueze; Elaine Vieira Santos; Evandra Strazza Rodrigues; Heidge Fukumasu; Jayme Augusto de Souza-Neto; José Salvatore Leister Patané; Luiz Alcantara; Luiz Lehmann Coutinho; Maria Carolina Elias; Maurício Lacerda Nogueira; Rafael dos Santos Bezerra; Raul Machado Neto; Rejane Maria Tommasini Grotto; Ricardo Haddad; Sandra Coccuzzo Sampaio Vessoni; Simone Kashima; Svetoslav Nanev Slavov; Vincent Louis Viala
EPI_ISL_2493669	CENTRO DE SAUDE III DOBRADA	Instituto Butantan	Antonio Jorge Martins; Claudia Renata dos Santos Barros; David Schlesinger; Debora Botequio Moretti; Dimas Tadeu Covas; Elaine Cristina Marqueze; Elaine Vieira Santos; Evandra Strazza Rodrigues; Heidge Fukumasu; Jayme Augusto de Souza-Neto; José Salvatore Leister Patané; Luiz Alcantara; Luiz Lehmann Coutinho; Maria Carolina Elias; Maurício Lacerda Nogueira; Rafael dos Santos Bezerra; Raul Machado Neto; Rejane Maria Tommasini Grotto; Ricardo Haddad; Sandra Coccuzzo Sampaio Vessoni; Simone Kashima; Svetoslav Nanev Slavov; Vincent Louis Viala
EPI_ISL_2209702	CENTRO DE SAUDE III NELCIDIO DA SILVEIRA BASTOS	Instituto Butantan / Mendelics	Antonio Jorge Martins; Claudia Renata dos Santos Barros; David Schlesinger; Debora Botequio Moretti; Dimas Tadeu Covas; Elaine Cristina Marqueze; Elaine Vieira Santos; Evandra Strazza Rodrigues; Heidge Fukumasu; Jayme Augusto de Souza-Neto; José Salvatore Leister Patané; Luiz Alcantara; Luiz Lehmann Coutinho; Maria Carolina Elias; Maurício Lacerda Nogueira; Rafael dos Santos Bezerra; Raul Machado Neto; Rejane Maria Tommasini Grotto; Ricardo Haddad; Sandra Coccuzzo Sampaio Vessoni; Simone Kashima; Svetoslav Nanev Slavov; Vincent Louis Viala
EPI_ISL_2208938, EPI_ISL_2209968, EPI_ISL_2210119, EPI_ISL_2345697, EPI_ISL_2345842	CENTRO DE SAUDE III SALES OLIVEIRA	Instituto Butantan / Mendelics	Antonio Jorge Martins; Claudia Renata dos Santos Barros; David Schlesinger; Debora Botequio Moretti; Dimas Tadeu Covas; Elaine Cristina Marqueze; Elaine Vieira Santos; Evandra Strazza Rodrigues; Heidge Fukumasu; Jayme Augusto de Souza-Neto; José Salvatore Leister Patané; Luiz Alcantara; Luiz Lehmann Coutinho; Maria Carolina Elias; Maurício Lacerda Nogueira; Rafael dos Santos Bezerra; Raul Machado Neto; Rejane Maria Tommasini Grotto; Ricardo Haddad; Sandra Coccuzzo Sampaio Vessoni; Simone Kashima; Svetoslav Nanev Slavov; Vincent Louis Viala
EPI_ISL_2493677	CENTRO DE SAUDE III TABATINGA	Instituto Butantan	Antonio Jorge Martins; Claudia Renata dos Santos Barros; David Schlesinger; Debora Botequio Moretti; Dimas Tadeu Covas; Elaine Cristina Marqueze; Elaine Vieira Santos; Evandra Strazza Rodrigues; Heidge Fukumasu; Jayme Augusto de Souza-Neto; José Salvatore Leister Patané; Luiz Alcantara; Luiz Lehmann

			Coutinho; Maria Carolina Elias; Maurício Lacerda Nogueira; Rafael dos Santos Bezerra; Raul Machado Neto; Rejane Maria Tommasini Grotto; Ricardo Haddad; Sandra Coccuzzo Sampaio Vessoni; Simone Kashima; Svetoslav Nanev Slavov; Vincent Louis Viala
EPI_ISL_2445360, EPI_ISL_2445365, EPI_ISL_2445448, EPI_ISL_2445451, EPI_ISL_2445452	CENTRO INTEGRADO DE SAUDE	Instituto Butantan	Antonio Jorge Martins; Claudia Renata dos Santos Barros; David Schlesinger; Debora Botequio Moretti; Dimas Tadeu Covas; Elaine Cristina Marqueze; Elaine Vieira Santos; Evandra Strazza Rodrigues; Heidge Fukumasu; Jayme Augusto de Souza-Neto; José Salvatore Leister Patané; Luiz Alcantara; Luiz Lehmann Coutinho; Maria Carolina Elias; Maurício Lacerda Nogueira; Rafael dos Santos Bezerra; Raul Machado Neto; Rejane Maria Tommasini Grotto; Ricardo Haddad; Sandra Coccuzzo Sampaio Vessoni; Simone Kashima; Svetoslav Nanev Slavov; Vincent Louis Viala
EPI_ISL_2493292, EPI_ISL_2493300	CENTRO MEDICO PMESP	Instituto Butantan	Antonio Jorge Martins; Claudia Renata dos Santos Barros; David Schlesinger; Debora Botequio Moretti; Dimas Tadeu Covas; Elaine Cristina Marqueze; Elaine Vieira Santos; Evandra Strazza Rodrigues; Heidge Fukumasu; Jayme Augusto de Souza-Neto; José Salvatore Leister Patané; Luiz Alcantara; Luiz Lehmann Coutinho; Maria Carolina Elias; Maurício Lacerda Nogueira; Rafael dos Santos Bezerra; Raul Machado Neto; Rejane Maria Tommasini Grotto; Ricardo Haddad; Sandra Coccuzzo Sampaio Vessoni; Simone Kashima; Svetoslav Nanev Slavov; Vincent Louis Viala
EPI_ISL_2345129	CENTRO MEDICO PMESP	Instituto Butantan / ESALQ-Piracicaba	Antonio Jorge Martins; Claudia Renata dos Santos Barros; David Schlesinger; Debora Botequio Moretti; Dimas Tadeu Covas; Elaine Cristina Marqueze; Elaine Vieira Santos; Evandra Strazza Rodrigues; Heidge Fukumasu; Jayme Augusto de Souza-Neto; José Salvatore Leister Patané; Luiz Alcantara; Luiz Lehmann Coutinho; Maria Carolina Elias; Maurício Lacerda Nogueira; Rafael dos Santos Bezerra; Raul Machado Neto; Rejane Maria Tommasini Grotto; Ricardo Haddad; Sandra Coccuzzo Sampaio Vessoni; Simone Kashima; Svetoslav Nanev Slavov; Vincent Louis Viala
EPI_ISL_2493597, EPI_ISL_2493600, EPI_ISL_2493606, EPI_ISL_2493625	CENTRO MEDICO SOCIAL COMUNITARIO JANUARIO TEODORO DE SOUZA	Instituto Butantan	Antonio Jorge Martins; Claudia Renata dos Santos Barros; David Schlesinger; Debora Botequio Moretti; Dimas Tadeu Covas; Elaine Cristina Marqueze; Elaine Vieira Santos; Evandra Strazza Rodrigues; Heidge Fukumasu; Jayme Augusto de Souza-Neto; José Salvatore Leister Patané; Luiz Alcantara; Luiz Lehmann Coutinho; Maria Carolina Elias; Maurício Lacerda Nogueira; Rafael dos Santos Bezerra; Raul Machado Neto; Rejane Maria Tommasini Grotto; Ricardo Haddad; Sandra Coccuzzo Sampaio Vessoni; Simone Kashima; Svetoslav Nanev Slavov; Vincent Louis Viala
EPI_ISL_2493433, EPI_ISL_2493445	CENTRO SAUDE III DR JOSE TADEU MOURAO	Instituto Butantan	Antonio Jorge Martins; Claudia Renata dos Santos Barros; David Schlesinger; Debora Botequio Moretti; Dimas Tadeu Covas; Elaine Cristina Marqueze; Elaine Vieira Santos; Evandra Strazza Rodrigues; Heidge Fukumasu; Jayme Augusto de Souza-Neto; José Salvatore Leister Patané; Luiz Alcantara; Luiz Lehmann Coutinho; Maria Carolina Elias; Maurício Lacerda Nogueira; Rafael dos Santos Bezerra; Raul Machado Neto; Rejane Maria Tommasini Grotto; Ricardo Haddad; Sandra Coccuzzo Sampaio Vessoni; Simone Kashima; Svetoslav Nanev Slavov; Vincent Louis Viala
EPI_ISL_2614611, EPI_ISL_2614612	CETESB - Companhia Ambiental do Estado de Sao Paulo	Instituto Adolfo Lutz, Interdiciplinary Procedures Center, Strategic Laboratory	Caio Vinicius Dias Lopes; Claudia Regina Gonçalves; Claudio Tavares Sacchi; Erica Valessa Ramos Gomes; Karoline Rodrigues Campos; Leonardo Jose Tadeu de Araujo; Luisa Zanolli Moreno; Maria Ines Zanoli Sato; Mikaela R. F. Barbosa
EPI_ISL_2015908	CH DE SAINTONGE	CNR Virus des Infections Respiratoires - France SUD	Antonin Bal; Bruno Lina; Gregory Destras; Gwendolyne Burfin; Hadrien Regue; Laurence Josset; Martine Valette; Quentin Semanas
EPI_ISL_2627846	CHC Andrée Rosemon	Institut Pasteur de la Guyane	Anne Lavergne; Antoine Enfissi; Arielle Salmier; Dominique Rousset
EPI_ISL_2248789, EPI_ISL_2248792	CHEDV	Instituto Nacional de Saude (INSA)	Borges et al
EPI_ISL_2004290, EPI_ISL_2248827, EPI_ISL_2362105, EPI_ISL_2362114	CHLC	Instituto Nacional de Saude (INSA)	Borges et al
EPI_ISL_2510151	CHUGA-IBP-laboratoire de Virologie	IBP-laboratoire de virologie	Alban Caporossi; Anne Signori -Schmuck; Anne-Karen Faure; Aurélie Truffot; Benjamin Nemoz; Hugo Jardin; Julien Andréani; Julien Lupo; Léa Ponderand; Pascal Poignard; Patrice Morand; Raphaële Germi; Sylvie Larrat
EPI_ISL_1941821	CHUV	Laboratory of genomics and metagenomics	Claire Bertelli; Damien Jacot; Gilbert Greub; Sebastien Aeby; Trestan Pillonel
EPI_ISL_2533931	CIAD-Hermosillo	Immunology Laboratory	; Alejandra García-Gasca; Alejandra Hernández-Terán; Alejandro Sánchez-Flores; Alfredo Herrera-Estrella; Alicia Ocaña-Mondragón; Andreu Comas-García; Angel Gustavo Salas-Lais; Antonio Loza Román; Bernardo Martínez-Miguel; Blanca Taboada; Brenda Irasema Maldonado-Meza; Bruno Gómez-Gil; Carla Ivón Herrera-Najera; Carlos F. Arias; Celia Boukadida; Clara Esperanza Santacruz-Tinoco; Concepción Grajales-Muñiz; Consorcio Mexicano de Vigilancia Genómica (CoViGen-Mex). Authors (in alphabetical order): Julio Elias Alvarado-Yaah; Cristóbal Cháidez-Quiróz; Célida Duque Molina; Célida Martínez-Rodríguez; Daniel Fregoso-Rueda; Daniel Lira Morales; Eduardo Becerril-Vargas; Fernando Fontove-Herrera; Fidencio Mejía-Nepomuceno; Francisco Pulido; Gloria Elena Espinosa-Ayala; Gloria María Molina-Salinas; Gloria Vazquez; Hector Esteban Paz-Juárez; Hector Montoya-Fuentes; Helen Haydee Fernanda Ramírez-Plascencia; Irvin González-López; Jean Pierre González; Jesús Hernández; Joel Armando Vázquez-Pérez.; Jorge Salas-Hernández; José Antonio Enciso-Moreno; José Arturo Martínez-Orozco; José Esteban Muñoz-Medina; José de Jesús Nuñez-Contreras; Juan Bautista Chale-Dzul; Julissa Enciso-Ibarra; Luis Alberto Ochoa-Carrera; Margarita Matías-Florentino; Mario Mújica-Sánchez; Marissa Perez-Garcia; María Guadalupe Santiago-Mauricio; María Guadalupe de Jesús Mireles-Rivera; Nelly Sélem-Mojica; Pavel Isa; Ricardo Ciria Merce; Ricardo Grande; Rosa María Gutiérrez Rios; Santiago Ávila-Ríos; Selene Zárate; Susana Lopez; Verónica Mata-Haro; Víctor Eduardo García-Arias; Víctor Hugo Borja-Aburto
EPI_ISL_2017661, EPI_ISL_2017665, EPI_ISL_2017668, EPI_ISL_2351958, EPI_ISL_2351959, EPI_ISL_2351960, EPI_ISL_2351964, EPI_ISL_2351968, EPI_ISL_2351969, EPI_ISL_2351970, EPI_ISL_2351978, EPI_ISL_2516497, EPI_ISL_2516531			
see above	CLILAB	Microbiology Department	Aida Gonzalez-Diaz; Carmen Ardanuy; Jordi Camara; Jordi Niubó; Laura Calatayud; M Angeles Domínguez; Miguel Fernandez-Huerta; Sara Marti
EPI_ISL_3355515	CLINICA PUTUMAYO	Corporacion CorpoGen-Universidad de los Andes-Universidad Central	Christian Romero; Jorge Duitama; Juan Manuel Anzola; Laura González; Maryam Chaib De Mares; María Mercedes Zambrano; Nelly Díaz; Patricia Del Portillo; Silvia Restrepo
EPI_ISL_2362548	CNR Institut Pasteur de la Guyane	Institut Pasteur de la Guyane	Anne Lavergne; Antoine Enfissi; Arielle Salmier; Dominique Rousset
EPI_ISL_2373756	CNR Virus des Infections Respiratoires - France SUD	CNR Virus des Infections Respiratoires - France SUD	Antonin Bal; Bruno Lina; Gregory Destras; Gwendolyne Burfin; Hadrien Regue; Laurence Josset; Martine Valette; Quentin Semanas
EPI_ISL_2493384, EPI_ISL_2493385, EPI_ISL_2493446, EPI_ISL_2494153	COORDENADORIA MUNICIPAL DE SAUDE DE IRACEMAPOLIS	Instituto Butantan	Antonio Jorge Martins; Claudia Renata dos Santos Barros; David Schlesinger; Debora Botequio Moretti; Dimas Tadeu Covas; Elaine Cristina Marqueze; Elaine Vieira Santos; Evandra Strazza Rodrigues; Heidge Fukumasu; Jayme Augusto de Souza-Neto; José Salvatore Leister Patané; Luiz Alcantara; Luiz Lehmann Coutinho; Maria Carolina Elias; Maurício Lacerda Nogueira; Rafael dos Santos Bezerra; Raul Machado Neto; Rejane Maria Tommasini Grotto; Ricardo Haddad; Sandra Coccuzzo Sampaio Vessoni; Simone Kashima; Svetoslav Nanev Slavov; Vincent Louis Viala
EPI_ISL_2345111	COVID 19 CENTRO DE COMBATE AO CORONAVIRUS CCC JANDIRA	Instituto Butantan / ESALQ-Piracicaba	Antonio Jorge Martins; Claudia Renata dos Santos Barros; David Schlesinger; Debora Botequio Moretti; Dimas Tadeu Covas; Elaine Cristina Marqueze; Elaine Vieira Santos; Evandra Strazza Rodrigues; Heidge Fukumasu; Jayme Augusto de Souza-Neto; José Salvatore Leister Patané; Luiz Alcantara; Luiz Lehmann Coutinho; Maria Carolina Elias; Maurício Lacerda Nogueira; Rafael dos Santos Bezerra; Raul Machado Neto; Rejane Maria Tommasini Grotto; Ricardo Haddad; Sandra Coccuzzo Sampaio Vessoni; Simone Kashima; Svetoslav Nanev Slavov; Vincent Louis Viala
EPI_ISL_2378754	CS DE APARECIDA DOESTE	Instituto Butantan	Antonio Jorge Martins; Claudia Renata dos Santos Barros; David Schlesinger; Debora Botequio Moretti; Dimas Tadeu Covas; Elaine Cristina Marqueze; Elaine Vieira Santos; Evandra Strazza Rodrigues; Heidge Fukumasu; Jayme Augusto de Souza-Neto; José Salvatore Leister Patané; Luiz Alcantara; Luiz Lehmann Coutinho; Maria Carolina Elias; Maurício Lacerda Nogueira; Rafael dos Santos Bezerra; Raul Machado Neto; Rejane Maria Tommasini Grotto; Ricardo Haddad; Sandra Coccuzzo Sampaio Vessoni; Simone Kashima; Svetoslav Nanev Slavov; Vincent Louis Viala
EPI_ISL_2445209, EPI_ISL_2445210, EPI_ISL_2473720	CS DE BALSAMO	Instituto Butantan	Antonio Jorge Martins; Claudia Renata dos Santos Barros; David Schlesinger; Debora Botequio Moretti; Dimas Tadeu Covas; Elaine Cristina Marqueze; Elaine Vieira Santos; Evandra Strazza Rodrigues; Heidge Fukumasu; Jayme Augusto de Souza-Neto; José Salvatore Leister Patané; Luiz Alcantara; Luiz Lehmann Coutinho; Maria Carolina Elias; Maurício Lacerda Nogueira; Rafael dos Santos Bezerra; Raul Machado Neto; Rejane Maria Tommasini Grotto; Ricardo Haddad; Sandra Coccuzzo Sampaio Vessoni; Simone Kashima; Svetoslav Nanev Slavov; Vincent Louis Viala
EPI_ISL_2344863	CS DE BALSAMO	Instituto Butantan / ESALQ-Piracicaba	Antonio Jorge Martins; Claudia Renata dos Santos Barros; David Schlesinger; Debora Botequio Moretti; Dimas Tadeu Covas; Elaine Cristina Marqueze; Elaine Vieira Santos; Evandra Strazza Rodrigues; Heidge Fukumasu; Jayme Augusto de Souza-Neto; José Salvatore Leister Patané; Luiz Alcantara; Luiz Lehmann Coutinho; Maria Carolina Elias; Maurício Lacerda Nogueira; Rafael dos Santos Bezerra; Raul Machado Neto; Rejane Maria Tommasini Grotto; Ricardo Haddad; Sandra Coccuzzo Sampaio Vessoni; Simone Kashima; Svetoslav Nanev Slavov; Vincent Louis Viala
EPI_ISL_2344823, EPI_ISL_2344824, EPI_ISL_2344857, EPI_ISL_2344860	CS DE CATIGUA	Instituto Butantan / ESALQ-Piracicaba	Antonio Jorge Martins; Claudia Renata dos Santos Barros; David Schlesinger; Debora Botequio Moretti; Dimas Tadeu Covas; Elaine Cristina Marqueze; Elaine Vieira Santos; Evandra Strazza Rodrigues; Heidge Fukumasu; Jayme Augusto de Souza-Neto; José Salvatore Leister Patané; Luiz Alcantara; Luiz Lehmann Coutinho; Maria Carolina Elias; Maurício Lacerda Nogueira; Rafael dos Santos Bezerra; Raul Machado Neto; Rejane Maria Tommasini Grotto; Ricardo Haddad; Sandra Coccuzzo Sampaio Vessoni; Simone Kashima; Svetoslav Nanev Slavov; Vincent Louis Viala
EPI_ISL_2445212, EPI_ISL_2494297	CS DE COSMORAMA	Instituto Butantan	Antonio Jorge Martins; Claudia Renata dos Santos Barros; David Schlesinger; Debora Botequio Moretti; Dimas Tadeu Covas; Elaine Cristina Marqueze; Elaine Vieira Santos; Evandra Strazza Rodrigues; Heidge Fukumasu; Jayme Augusto de Souza-Neto; José Salvatore Leister Patané; Luiz Alcantara; Luiz Lehmann Coutinho; Maria Carolina Elias; Maurício Lacerda Nogueira; Rafael dos Santos Bezerra; Raul Machado Neto; Rejane Maria Tommasini Grotto; Ricardo Haddad; Sandra Coccuzzo Sampaio Vessoni; Simone Kashima; Svetoslav Nanev Slavov; Vincent Louis Viala
EPI_ISL_2344871, EPI_ISL_2344874	CS DE COSMORAMA	Instituto Butantan / ESALQ-Piracicaba	Antonio Jorge Martins; Claudia Renata dos Santos Barros; David Schlesinger; Debora Botequio Moretti; Dimas Tadeu Covas; Elaine Cristina Marqueze; Elaine Vieira Santos; Evandra Strazza Rodrigues; Heidge Fukumasu; Jayme Augusto de Souza-Neto; José Salvatore Leister Patané; Luiz Alcantara; Luiz Lehmann Coutinho; Maria Carolina Elias; Maurício Lacerda Nogueira; Rafael dos Santos Bezerra; Raul Machado Neto; Rejane Maria Tommasini Grotto; Ricardo Haddad; Sandra Coccuzzo Sampaio Vessoni; Simone Kashima; Svetoslav Nanev Slavov; Vincent Louis Viala
EPI_ISL_2344882	CS DE MARAPOAMA	Instituto Butantan / ESALQ-Piracicaba	Antonio Jorge Martins; Claudia Renata dos Santos Barros; David Schlesinger; Debora Botequio Moretti; Dimas Tadeu Covas; Elaine Cristina Marqueze; Elaine Vieira Santos; Evandra Strazza Rodrigues; Heidge Fukumasu; Jayme Augusto de Souza-Neto; José Salvatore Leister Patané; Luiz Alcantara; Luiz Lehmann Coutinho; Maria Carolina Elias; Maurício Lacerda Nogueira; Rafael dos Santos Bezerra; Raul Machado Neto; Rejane Maria Tommasini Grotto; Ricardo Haddad; Sandra Coccuzzo Sampaio Vessoni; Simone Kashima; Svetoslav Nanev Slavov; Vincent Louis Viala
EPI_ISL_2493424	CS DE MONTE APRAZIVEL	Instituto Butantan	Antonio Jorge Martins; Claudia Renata dos Santos Barros; David Schlesinger; Debora Botequio Moretti; Dimas Tadeu Covas; Elaine Cristina Marqueze; Elaine Vieira Santos; Evandra Strazza Rodrigues; Heidge Fukumasu; Jayme Augusto de Souza-Neto; José Salvatore Leister Patané; Luiz Alcantara; Luiz Lehmann Coutinho; Maria Carolina Elias; Maurício Lacerda Nogueira; Rafael dos Santos Bezerra; Raul Machado Neto; Rejane Maria Tommasini Grotto; Ricardo Haddad; Sandra Coccuzzo Sampaio Vessoni; Simone Kashima; Svetoslav Nanev Slavov; Vincent Louis Viala
EPI_ISL_2493405, EPI_ISL_2493406, EPI_ISL_2493407, EPI_ISL_2493412, EPI_ISL_2493414, EPI_ISL_2493416	CS DE NEVES PAULISTA	Instituto Butantan	Antonio Jorge Martins; Claudia Renata dos Santos Barros; David Schlesinger; Debora Botequio Moretti; Dimas Tadeu Covas; Elaine Cristina Marqueze; Elaine Vieira Santos; Evandra Strazza Rodrigues; Heidge Fukumasu; Jayme Augusto de Souza-Neto; José Salvatore Leister Patané; Luiz Alcantara; Luiz Lehmann Coutinho; Maria Carolina Elias; Maurício Lacerda Nogueira; Rafael dos Santos Bezerra; Raul Machado Neto; Rejane Maria Tommasini Grotto; Ricardo Haddad; Sandra Coccuzzo Sampaio Vessoni; Simone Kashima; Svetoslav Nanev Slavov; Vincent Louis Viala
EPI_ISL_2445213, EPI_ISL_2445214, EPI_ISL_2445215, EPI_ISL_2445217, EPI_ISL_2445218, EPI_ISL_2473721, EPI_ISL_2473722, EPI_ISL_2493661, EPI_ISL_2493662			
see above	CS DE NOVO HORIZONTE	Instituto Butantan	Antonio Jorge Martins; Claudia Renata dos Santos Barros; David Schlesinger; Debora Botequio Moretti; Dimas Tadeu Covas; Elaine Cristina Marqueze; Elaine Vieira Santos; Evandra Strazza Rodrigues; Heidge Fukumasu; Jayme Augusto de Souza-Neto; José Salvatore Leister Patané; Luiz Alcantara; Luiz Lehmann Coutinho; Maria Carolina Elias; Maurício Lacerda Nogueira; Rafael dos Santos Bezerra; Raul Machado Neto; Rejane Maria Tommasini Grotto; Ricardo Haddad; Sandra Coccuzzo Sampaio Vessoni; Simone Kashima; Svetoslav Nanev Slavov; Vincent Louis Viala
EPI_ISL_2344872, EPI_ISL_2344875, EPI_ISL_2344887, EPI_ISL_2344889	CS DE ONDA VERDE	Instituto Butantan / ESALQ-Piracicaba	Antonio Jorge Martins; Claudia Renata dos Santos Barros; David Schlesinger; Debora Botequio Moretti; Dimas Tadeu Covas; Elaine Cristina Marqueze; Elaine Vieira Santos; Evandra Strazza Rodrigues; Heidge Fukumasu; Jayme Augusto de Souza-Neto; José Salvatore Leister Patané; Luiz Alcantara; Luiz Lehmann Coutinho; Maria Carolina Elias; Maurício Lacerda Nogueira; Rafael dos Santos Bezerra; Raul Machado Neto; Rejane Maria Tommasini Grotto; Ricardo Haddad; Sandra Coccuzzo Sampaio Vessoni; Simone Kashima; Svetoslav Nanev Slavov; Vincent Louis Viala
EPI_ISL_2493411, EPI_ISL_2493419, EPI_ISL_2493429	CS DE ORINDIUVA	Instituto Butantan	Antonio Jorge Martins; Claudia Renata dos Santos Barros; David Schlesinger; Debora Botequio Moretti; Dimas Tadeu Covas; Elaine Cristina Marqueze; Elaine Vieira Santos; Evandra Strazza Rodrigues; Heidge Fukumasu; Jayme Augusto de Souza-Neto; José Salvatore Leister Patané; Luiz Alcantara; Luiz Lehmann Coutinho; Maria Carolina Elias; Maurício Lacerda Nogueira; Rafael dos Santos Bezerra; Raul Machado Neto; Rejane Maria Tommasini Grotto; Ricardo Haddad; Sandra Coccuzzo Sampaio Vessoni; Simone Kashima; Svetoslav Nanev Slavov; Vincent Louis Viala
EPI_ISL_2493410, EPI_ISL_2493413, EPI_ISL_2493415, EPI_ISL_2493417, EPI_ISL_2493420	CS DE PALMARES PAULISTA	Instituto Butantan	Antonio Jorge Martins; Claudia Renata dos Santos Barros; David Schlesinger; Debora Botequio Moretti; Dimas Tadeu Covas; Elaine Cristina Marqueze; Elaine Vieira Santos; Evandra Strazza Rodrigues; Heidge Fukumasu; Jayme Augusto de Souza-Neto; José Salvatore Leister Patané; Luiz Alcantara; Luiz Lehmann Coutinho; Maria Carolina Elias; Maurício Lacerda Nogueira; Rafael dos Santos Bezerra; Raul Machado Neto; Rejane Maria Tommasini Grotto; Ricardo Haddad; Sandra Coccuzzo Sampaio Vessoni; Simone Kashima; Svetoslav Nanev Slavov; Vincent Louis Viala
EPI_ISL_2209164, EPI_ISL_2344859	CS DE PALMARES PAULISTA	Instituto Butantan / ESALQ-Piracicaba	Antonio Jorge Martins; Claudia Renata dos Santos Barros; David Schlesinger; Debora Botequio Moretti; Dimas Tadeu Covas; Elaine Cristina Marqueze; Elaine Vieira Santos; Evandra Strazza Rodrigues; Heidge Fukumasu; Jayme Augusto de Souza-Neto; José Salvatore Leister Patané; Luiz Alcantara; Luiz Lehmann Coutinho; Maria Carolina Elias; Maurício Lacerda Nogueira; Rafael dos Santos Bezerra; Raul Machado Neto; Rejane Maria Tommasini Grotto; Ricardo Haddad; Sandra Coccuzzo Sampaio Vessoni; Simone Kashima; Svetoslav Nanev Slavov; Vincent Louis Viala
EPI_ISL_2445211	CS DE PLANALTO	Instituto Butantan	Antonio Jorge Martins; Claudia Renata dos Santos Barros; David Schlesinger; Debora Botequio Moretti; Dimas Tadeu Covas; Elaine Cristina Marqueze; Elaine Vieira Santos; Evandra Strazza Rodrigues; Heidge Fukumasu; Jayme Augusto de Souza-Neto; José Salvatore Leister Patané; Luiz Alcantara; Luiz Lehmann

			Coutinho; Maria Carolina Elias; Maurício Lacerda Nogueira; Rafael dos Santos Bezerra; Raul Machado Neto; Rejane Maria Tommasini Grotto; Ricardo Haddad; Sandra Coccuzzo Sampaio Vessoni; Simone Kashima; Svetoslav Nanev Slavov; Vincent Louis Viala
EPI_ISL_2493362	CS DE ROSANA	Instituto Butantan	Antonio Jorge Martins; Claudia Renata dos Santos Barros; David Schlesinger; Debora Botequio Moretti; Dimas Tadeu Covas; Elaine Cristina Marqueze; Elaine Vieira Santos; Evandra Strazza Rodrigues; Heidge Fukumasu; Jayme Augusto de Souza-Neto; José Salvatore Leister Patané; Luiz Alcantara; Luiz Lehmann Coutinho; Maria Carolina Elias; Maurício Lacerda Nogueira; Rafael dos Santos Bezerra; Raul Machado Neto; Rejane Maria Tommasini Grotto; Ricardo Haddad; Sandra Coccuzzo Sampaio Vessoni; Simone Kashima; Svetoslav Nanev Slavov; Vincent Louis Viala
EPI_ISL_2493400	CS DE SANTA ALBERTINA	Instituto Butantan	Antonio Jorge Martins; Claudia Renata dos Santos Barros; David Schlesinger; Debora Botequio Moretti; Dimas Tadeu Covas; Elaine Cristina Marqueze; Elaine Vieira Santos; Evandra Strazza Rodrigues; Heidge Fukumasu; Jayme Augusto de Souza-Neto; José Salvatore Leister Patané; Luiz Alcantara; Luiz Lehmann Coutinho; Maria Carolina Elias; Maurício Lacerda Nogueira; Rafael dos Santos Bezerra; Raul Machado Neto; Rejane Maria Tommasini Grotto; Ricardo Haddad; Sandra Coccuzzo Sampaio Vessoni; Simone Kashima; Svetoslav Nanev Slavov; Vincent Louis Viala
EPI_ISL_2344854, EPI_ISL_2344855, EPI_ISL_2344856	CS DE SANTA ALBERTINA	Instituto Butantan / ESALQ-Piracicaba	Antonio Jorge Martins; Claudia Renata dos Santos Barros; David Schlesinger; Debora Botequio Moretti; Dimas Tadeu Covas; Elaine Cristina Marqueze; Elaine Vieira Santos; Evandra Strazza Rodrigues; Heidge Fukumasu; Jayme Augusto de Souza-Neto; José Salvatore Leister Patané; Luiz Alcantara; Luiz Lehmann Coutinho; Maria Carolina Elias; Maurício Lacerda Nogueira; Rafael dos Santos Bezerra; Raul Machado Neto; Rejane Maria Tommasini Grotto; Ricardo Haddad; Sandra Coccuzzo Sampaio Vessoni; Simone Kashima; Svetoslav Nanev Slavov; Vincent Louis Viala
EPI_ISL_2493421, EPI_ISL_2493422	CS DE SANTA RITA DOESTE	Instituto Butantan	Antonio Jorge Martins; Claudia Renata dos Santos Barros; David Schlesinger; Debora Botequio Moretti; Dimas Tadeu Covas; Elaine Cristina Marqueze; Elaine Vieira Santos; Evandra Strazza Rodrigues; Heidge Fukumasu; Jayme Augusto de Souza-Neto; José Salvatore Leister Patané; Luiz Alcantara; Luiz Lehmann Coutinho; Maria Carolina Elias; Maurício Lacerda Nogueira; Rafael dos Santos Bezerra; Raul Machado Neto; Rejane Maria Tommasini Grotto; Ricardo Haddad; Sandra Coccuzzo Sampaio Vessoni; Simone Kashima; Svetoslav Nanev Slavov; Vincent Louis Viala
EPI_ISL_2493430, EPI_ISL_2493431	CS DE VALENTIM GENTIL	Instituto Butantan	Antonio Jorge Martins; Claudia Renata dos Santos Barros; David Schlesinger; Debora Botequio Moretti; Dimas Tadeu Covas; Elaine Cristina Marqueze; Elaine Vieira Santos; Evandra Strazza Rodrigues; Heidge Fukumasu; Jayme Augusto de Souza-Neto; José Salvatore Leister Patané; Luiz Alcantara; Luiz Lehmann Coutinho; Maria Carolina Elias; Maurício Lacerda Nogueira; Rafael dos Santos Bezerra; Raul Machado Neto; Rejane Maria Tommasini Grotto; Ricardo Haddad; Sandra Coccuzzo Sampaio Vessoni; Simone Kashima; Svetoslav Nanev Slavov; Vincent Louis Viala
EPI_ISL_2493299	CS II DE IGARAPAVA	Instituto Butantan	Antonio Jorge Martins; Claudia Renata dos Santos Barros; David Schlesinger; Debora Botequio Moretti; Dimas Tadeu Covas; Elaine Cristina Marqueze; Elaine Vieira Santos; Evandra Strazza Rodrigues; Heidge Fukumasu; Jayme Augusto de Souza-Neto; José Salvatore Leister Patané; Luiz Alcantara; Luiz Lehmann Coutinho; Maria Carolina Elias; Maurício Lacerda Nogueira; Rafael dos Santos Bezerra; Raul Machado Neto; Rejane Maria Tommasini Grotto; Ricardo Haddad; Sandra Coccuzzo Sampaio Vessoni; Simone Kashima; Svetoslav Nanev Slavov; Vincent Louis Viala
EPI_ISL_2445399, EPI_ISL_2445401	CS II DR ANTONIO VICOSO MOREIRA DE REZENDE SUMARE	Instituto Butantan	Antonio Jorge Martins; Claudia Renata dos Santos Barros; David Schlesinger; Debora Botequio Moretti; Dimas Tadeu Covas; Elaine Cristina Marqueze; Elaine Vieira Santos; Evandra Strazza Rodrigues; Heidge Fukumasu; Jayme Augusto de Souza-Neto; José Salvatore Leister Patané; Luiz Alcantara; Luiz Lehmann Coutinho; Maria Carolina Elias; Maurício Lacerda Nogueira; Rafael dos Santos Bezerra; Raul Machado Neto; Rejane Maria Tommasini Grotto; Ricardo Haddad; Sandra Coccuzzo Sampaio Vessoni; Simone Kashima; Svetoslav Nanev Slavov; Vincent Louis Viala
EPI_ISL_2445455, EPI_ISL_2445458, EPI_ISL_2445459, EPI_ISL_2445461, EPI_ISL_2445462	CS II DR JAHYR DE PAULA RIBEIRO GUARA	Instituto Butantan	Antonio Jorge Martins; Claudia Renata dos Santos Barros; David Schlesinger; Debora Botequio Moretti; Dimas Tadeu Covas; Elaine Cristina Marqueze; Elaine Vieira Santos; Evandra Strazza Rodrigues; Heidge Fukumasu; Jayme Augusto de Souza-Neto; José Salvatore Leister Patané; Luiz Alcantara; Luiz Lehmann Coutinho; Maria Carolina Elias; Maurício Lacerda Nogueira; Rafael dos Santos Bezerra; Raul Machado Neto; Rejane Maria Tommasini Grotto; Ricardo Haddad; Sandra Coccuzzo Sampaio Vessoni; Simone Kashima; Svetoslav Nanev Slavov; Vincent Louis Viala
EPI_ISL_2345262	CS II DR JAHYR DE PAULA RIBEIRO GUARA	Instituto Butantan / ESALQ-Piracicaba	Antonio Jorge Martins; Claudia Renata dos Santos Barros; David Schlesinger; Debora Botequio Moretti; Dimas Tadeu Covas; Elaine Cristina Marqueze; Elaine Vieira Santos; Evandra Strazza Rodrigues; Heidge Fukumasu; Jayme Augusto de Souza-Neto; José Salvatore Leister Patané; Luiz Alcantara; Luiz Lehmann Coutinho; Maria Carolina Elias; Maurício Lacerda Nogueira; Rafael dos Santos Bezerra; Raul Machado Neto; Rejane Maria Tommasini Grotto; Ricardo Haddad; Sandra Coccuzzo Sampaio Vessoni; Simone Kashima; Svetoslav Nanev Slavov; Vincent Louis Viala
EPI_ISL_2445406	CS II DR MIGUEL VITALIANO ORLANDIA	Instituto Butantan	Antonio Jorge Martins; Claudia Renata dos Santos Barros; David Schlesinger; Debora Botequio Moretti; Dimas Tadeu Covas; Elaine Cristina Marqueze; Elaine Vieira Santos; Evandra Strazza Rodrigues; Heidge Fukumasu; Jayme Augusto de Souza-Neto; José Salvatore Leister Patané; Luiz Alcantara; Luiz Lehmann Coutinho; Maria Carolina Elias; Maurício Lacerda Nogueira; Rafael dos Santos Bezerra; Raul Machado Neto; Rejane Maria Tommasini Grotto; Ricardo Haddad; Sandra Coccuzzo Sampaio Vessoni; Simone Kashima; Svetoslav Nanev Slavov; Vincent Louis Viala
EPI_ISL_2345078, EPI_ISL_2345194, EPI_ISL_2345198, EPI_ISL_2345200	CS II DR MIGUEL VITALIANO ORLANDIA	Instituto Butantan / ESALQ-Piracicaba	Antonio Jorge Martins; Claudia Renata dos Santos Barros; David Schlesinger; Debora Botequio Moretti; Dimas Tadeu Covas; Elaine Cristina Marqueze; Elaine Vieira Santos; Evandra Strazza Rodrigues; Heidge Fukumasu; Jayme Augusto de Souza-Neto; José Salvatore Leister Patané; Luiz Alcantara; Luiz Lehmann Coutinho; Maria Carolina Elias; Maurício Lacerda Nogueira; Rafael dos Santos Bezerra; Raul Machado Neto; Rejane Maria Tommasini Grotto; Ricardo Haddad; Sandra Coccuzzo Sampaio Vessoni; Simone Kashima; Svetoslav Nanev Slavov; Vincent Louis Viala
EPI_ISL_2209850	CS II DR SEBASTIAO RIBEIRO DO VALLE	Instituto Butantan / Mendelics	Antonio Jorge Martins; Claudia Renata dos Santos Barros; David Schlesinger; Debora Botequio Moretti; Dimas Tadeu Covas; Elaine Cristina Marqueze; Elaine Vieira Santos; Evandra Strazza Rodrigues; Heidge Fukumasu; Jayme Augusto de Souza-Neto; José Salvatore Leister Patané; Luiz Alcantara; Luiz Lehmann Coutinho; Maria Carolina Elias; Maurício Lacerda Nogueira; Rafael dos Santos Bezerra; Raul Machado Neto; Rejane Maria Tommasini Grotto; Ricardo Haddad; Sandra Coccuzzo Sampaio Vessoni; Simone Kashima; Svetoslav Nanev Slavov; Vincent Louis Viala
EPI_ISL_2493573	CS III DR ANTONIO ANADAO SSGRAMA	Instituto Butantan	Antonio Jorge Martins; Claudia Renata dos Santos Barros; David Schlesinger; Debora Botequio Moretti; Dimas Tadeu Covas; Elaine Cristina Marqueze; Elaine Vieira Santos; Evandra Strazza Rodrigues; Heidge Fukumasu; Jayme Augusto de Souza-Neto; José Salvatore Leister Patané; Luiz Alcantara; Luiz Lehmann Coutinho; Maria Carolina Elias; Maurício Lacerda Nogueira; Rafael dos Santos Bezerra; Raul Machado Neto; Rejane Maria Tommasini Grotto; Ricardo Haddad; Sandra Coccuzzo Sampaio Vessoni; Simone Kashima; Svetoslav Nanev Slavov; Vincent Louis Viala
EPI_ISL_2345273, EPI_ISL_2345274, EPI_ISL_2345278	CS IOLANDA FLEURY DE CAMPOS GUEDES NUPORANGA	Instituto Butantan / ESALQ-Piracicaba	Antonio Jorge Martins; Claudia Renata dos Santos Barros; David Schlesinger; Debora Botequio Moretti; Dimas Tadeu Covas; Elaine Cristina Marqueze; Elaine Vieira Santos; Evandra Strazza Rodrigues; Heidge Fukumasu; Jayme Augusto de Souza-Neto; José Salvatore Leister Patané; Luiz Alcantara; Luiz Lehmann Coutinho; Maria Carolina Elias; Maurício Lacerda Nogueira; Rafael dos Santos Bezerra; Raul Machado Neto; Rejane Maria Tommasini Grotto; Ricardo Haddad; Sandra Coccuzzo Sampaio Vessoni; Simone Kashima; Svetoslav Nanev Slavov; Vincent Louis Viala
EPI_ISL_2344831	CSA COMPLEXO DE SAUDE ANHANGUERA LTDA	Instituto Butantan	Antonio Jorge Martins; Claudia Renata dos Santos Barros; David Schlesinger; Debora Botequio Moretti; Dimas Tadeu Covas; Elaine Cristina Marqueze; Elaine Vieira Santos; Evandra Strazza Rodrigues; Heidge Fukumasu; Jayme Augusto de Souza-Neto; José Salvatore Leister Patané; Luiz Alcantara; Luiz Lehmann Coutinho; Maria Carolina Elias; Maurício Lacerda Nogueira; Rafael dos Santos Bezerra; Raul Machado Neto; Rejane Maria Tommasini Grotto; Ricardo Haddad; Sandra Coccuzzo Sampaio Vessoni; Simone Kashima; Svetoslav Nanev Slavov; Vincent Louis Viala
EPI_ISL_2344906	CSA COMPLEXO DE SAUDE ANHANGUERA TUCURUVI	Instituto Butantan	Antonio Jorge Martins; Claudia Renata dos Santos Barros; David Schlesinger; Debora Botequio Moretti; Dimas Tadeu Covas; Elaine Cristina Marqueze; Elaine Vieira Santos; Evandra Strazza Rodrigues; Heidge Fukumasu; Jayme Augusto de Souza-Neto; José Salvatore Leister Patané; Luiz Alcantara; Luiz Lehmann Coutinho; Maria Carolina Elias; Maurício Lacerda Nogueira; Rafael dos Santos Bezerra; Raul Machado Neto; Rejane Maria Tommasini Grotto; Ricardo Haddad; Sandra Coccuzzo Sampaio Vessoni; Simone Kashima; Svetoslav Nanev Slavov; Vincent Louis Viala
EPI_ISL_2493301	CSII DR JOSE FERREIRA TELLES	Instituto Butantan	Antonio Jorge Martins; Claudia Renata dos Santos Barros; David Schlesinger; Debora Botequio Moretti; Dimas Tadeu Covas; Elaine Cristina Marqueze; Elaine Vieira Santos; Evandra Strazza Rodrigues; Heidge Fukumasu; Jayme Augusto de Souza-Neto; José Salvatore Leister Patané; Luiz Alcantara; Luiz Lehmann Coutinho; Maria Carolina Elias; Maurício Lacerda Nogueira; Rafael dos Santos Bezerra; Raul Machado Neto; Rejane Maria Tommasini Grotto; Ricardo Haddad; Sandra Coccuzzo Sampaio Vessoni; Simone Kashima; Svetoslav Nanev Slavov; Vincent Louis Viala
EPI_ISL_2345171, EPI_ISL_2345246	CSII DR JOSE FERREIRA TELLES	Instituto Butantan / ESALQ-Piracicaba	Antonio Jorge Martins; Claudia Renata dos Santos Barros; David Schlesinger; Debora Botequio Moretti; Dimas Tadeu Covas; Elaine Cristina Marqueze; Elaine Vieira Santos; Evandra Strazza Rodrigues; Heidge Fukumasu; Jayme Augusto de Souza-Neto; José Salvatore Leister Patané; Luiz Alcantara; Luiz Lehmann Coutinho; Maria Carolina Elias; Maurício Lacerda Nogueira; Rafael dos Santos Bezerra; Raul Machado Neto; Rejane Maria Tommasini Grotto; Ricardo Haddad; Sandra Coccuzzo Sampaio Vessoni; Simone Kashima; Svetoslav Nanev Slavov; Vincent Louis Viala
EPI_ISL_2493622	CSII DR WASHINGTON LUIS M RODRIGUES DA SILVA PITANGUEIRAS	Instituto Butantan	Antonio Jorge Martins; Claudia Renata dos Santos Barros; David Schlesinger; Debora Botequio Moretti; Dimas Tadeu Covas; Elaine Cristina Marqueze; Elaine Vieira Santos; Evandra Strazza Rodrigues; Heidge Fukumasu; Jayme Augusto de Souza-Neto; José Salvatore Leister Patané; Luiz Alcantara; Luiz Lehmann Coutinho; Maria Carolina Elias; Maurício Lacerda Nogueira; Rafael dos Santos Bezerra; Raul Machado Neto; Rejane Maria Tommasini Grotto; Ricardo Haddad; Sandra Coccuzzo Sampaio Vessoni; Simone Kashima; Svetoslav Nanev Slavov; Vincent Louis Viala
EPI_ISL_2208992, EPI_ISL_2345680, EPI_ISL_2345824, EPI_ISL_2345868, EPI_ISL_2362268	CSII DR WASHINGTON LUIS M RODRIGUES DA SILVA PITANGUEIRAS	Instituto Butantan / Mendelics	Antonio Jorge Martins; Claudia Renata dos Santos Barros; David Schlesinger; Debora Botequio Moretti; Dimas Tadeu Covas; Elaine Cristina Marqueze; Elaine Vieira Santos; Evandra Strazza Rodrigues; Heidge Fukumasu; Jayme Augusto de Souza-Neto; José Salvatore Leister Patané; Luiz Alcantara; Luiz Lehmann Coutinho; Maria Carolina Elias; Maurício Lacerda Nogueira; Rafael dos Santos Bezerra; Raul Machado Neto; Rejane Maria Tommasini Grotto; Ricardo Haddad; Sandra Coccuzzo Sampaio Vessoni; Simone Kashima; Svetoslav Nanev Slavov; Vincent Louis Viala
EPI_ISL_2105892	CSL, Maryland Department of Health	CSL, Maryland Department of Health	Keller, E.; Loomis, J.; Low, K.
EPI_ISL_2537469, EPI_ISL_2892480, EPI_ISL_3536926, EPI_ISL_3536928, EPI_ISL_3536930, EPI_ISL_3536933, EPI_ISL_4005512, EPI_ISL_4005520, EPI_ISL_4005522, EPI_ISL_4005534, EPI_ISL_4005535, EPI_ISL_4005537, EPI_ISL_4005543, EPI_ISL_4005549, EPI_ISL_4005551			
see above	California Department of Public Health	California Department of Public Health	CDPH IDLB COVIDNet et al
EPI_ISL_3134058, EPI_ISL_3134076, EPI_ISL_3134087	California Department of Public Health Valencia Branch Laboratory	California Department of Public Health	CDPH-COVIDNet; UCSF Center for Advanced Technology
EPI_ISL_2778847, EPI_ISL_2778870, EPI_ISL_2778895, EPI_ISL_2778935	California Department of Public Health Valencia Branch Laboratory (CDPH VBL)	California Department of Public Health	CDPH-COVIDNet; UCLA Technology Center for Genomics & Bioinformatics
EPI_ISL_3144947	Center for Virology	Center for Virology	Jeremy V. Camp; Monika Redlberger-Fritz; Stephan W. Aberle
EPI_ISL_2296515, EPI_ISL_2296516	Centracare Laboratory Services	Minnesota Department of Health, Public Health Laboratory	Alexandra Lorentz; Jacob Garfin; Matt Plumb; and Xiong Wang
EPI_ISL_2502476, EPI_ISL_2502477, EPI_ISL_2502478, EPI_ISL_2502479, EPI_ISL_2502480, EPI_ISL_2502481, EPI_ISL_2502482, EPI_ISL_2502483, EPI_ISL_2502484, EPI_ISL_2502485, EPI_ISL_2502486, EPI_ISL_2502487, EPI_ISL_2502488, EPI_ISL_2502489, EPI_ISL_2502490, EPI_ISL_2502491, EPI_ISL_2502492, EPI_ISL_2502493, EPI_ISL_2502494, EPI_ISL_2502495, EPI_ISL_2502496, EPI_ISL_2502497, EPI_ISL_2502498, EPI_ISL_2502499, EPI_ISL_2502500, EPI_ISL_2502501, EPI_ISL_2502502, EPI_ISL_2502503, EPI_ISL_2502504, EPI_ISL_2502505, EPI_ISL_2502506, EPI_ISL_2502508, EPI_ISL_3462589, EPI_ISL_3462622, EPI_ISL_3462623, EPI_ISL_3462624, EPI_ISL_3462625, EPI_ISL_3462626			
see above	Central Laboratory, Bureau of Public Health (BOG) and Academic Hospital Paramaribo	Erasmus Medical Center	Bas B Oude Munnink; Cherise Beek; Consuella Partowidjojo; Dion Gajadin; Ed PF IJzerman; Emmanuelle Munger; Gary Gummels; Ingrid SK Krishnadath; Lycke Woittiez; Marion PG Koopmans; Mireille Van de Veer; Phyllis Pinas; Princes Wongsowidjojo; Radjesh Ori; Ranisha Doerbalie; Rohma Banwari; Soeradj Harkisoen; Stephen Vreden; Tilotmadebie Ramlal; Verne Nanhoe
EPI_ISL_3255060, EPI_ISL_3255062, EPI_ISL_3255065, EPI_ISL_3255066, EPI_ISL_3255069, EPI_ISL_3255071, EPI_ISL_3255072, EPI_ISL_3255073, EPI_ISL_3255076, EPI_ISL_3255080, EPI_ISL_3255083, EPI_ISL_3255086, EPI_ISL_3255087, EPI_ISL_3255088, EPI_ISL_3255089, EPI_ISL_3255090, EPI_ISL_3255091, EPI_ISL_3255092, EPI_ISL_3255093, EPI_ISL_3255094, EPI_ISL_3255095, EPI_ISL_3255096, EPI_ISL_3255097, EPI_ISL_3255099, EPI_ISL_3255100, EPI_ISL_3255102, EPI_ISL_3255103, EPI_ISL_3255104, EPI_ISL_3255106, EPI_ISL_3255107, EPI_ISL_3255108, EPI_ISL_3255109, EPI_ISL_3255110, EPI_ISL_3255111, EPI_ISL_3255112, EPI_ISL_3255113, EPI_ISL_3255114, EPI_ISL_3255115, EPI_ISL_3255119, EPI_ISL_3255120, EPI_ISL_3255121, EPI_ISL_3255123, EPI_ISL_3255125, EPI_ISL_3255126, EPI_ISL_3255127, EPI_ISL_3255128, EPI_ISL_3255129, EPI_ISL_3255130, EPI_ISL_3255131, EPI_ISL_3255133, EPI_ISL_3255136, EPI_ISL_3255139, EPI_ISL_3255143, EPI_ISL_3255150, EPI_ISL_3255153, EPI_ISL_3255161, EPI_ISL_3255165, EPI_ISL_3255172, EPI_ISL_3255173, EPI_ISL_3255177, EPI_ISL_3255178, EPI_ISL_3255191, EPI_ISL_3255194, EPI_ISL_3255201, EPI_ISL_3255207, EPI_ISL_3255219, EPI_ISL_3255223, EPI_ISL_3255233			
see above	Central Public Health Laboratory - LACEN -Bahia, Salvador, Brazil	Central Public Health Laboratory - LACEN -Bahia, Salvador, Brazil	Arabela Leal; Breno Dominguez; Felicidade Pereira; Jaqueline Gomes; Luciana Oliveira; Luiz Alcantara; Marcela Gómez; Marta Giovanetti; Patrícia Cajado; Stephane Tosta; Vagner Fonseca; Vanessa Nardy
EPI_ISL_1853299, EPI_ISL_2002663, EPI_ISL_2002664, EPI_ISL_2134734	Centre Hospitalier Universitaire Clermont-Ferrand	CHU Clermont-Ferrand, service de virologie	Bisseux Maxime; Combes Patricia; Henquell Cécile; Mirand Audrey
EPI_ISL_2671553	Centro de Investigacion Biomedica de Occidente (CIBO)	Unidad de Genomica Avanzada	; Alejandra Garcia-Gasca; Alejandra Hernandez-Teran; Alejandro Sanchez-Flores; Alfredo Herrera-Estrella; Alicia Ocaña-Mondragon; Andreu Comas-Garcia; Angel Gustavo Salas-Lais; Antonio Loza Roman; Bernardo Martinez-Miguel; Blanca Taboada; Brenda Irasema Maldonado-Meza; Bruno Gomez-Gil; Carla Ivon Herrera-Najera; Carlos F. Arias; Celia Boukadida; Celida Duque Molina; Celida Martinez- Rodriguez; Clara Esperanza Santacruz-Tinoco; Concepcion Grajales-Muñiz; Consorcio Mexicano de Vigilancia Genomica (CoViGen-Mex). Authors (in alphabetical order): Julio Elias Alvarado-Yaah; Cristobal Chaidez-Quiroz; Daniel Fregoso-Rueda; Daniel Lira Morales; Eduardo Becerril-Vargas; Fernando Fontove-Herrera; Fidencio Mejia-Nepomuceno; Francisco Pulido; Gloria Elena Espinosa-Ayala; Gloria Maria Molina-Salinas; Gloria Vazquez; Hector Esteban Paz-Juarez; Hector Montoya-Fuentes; Helen Haydee Fernanda Ramirez-Plascencia; Irvin Gonzalez-Lopez; Jean Pierre Gonzalez; Jesus Hernandez; Joel Armando Vazquez-Perez.; Jorge Salas-Hernandez; Jose Antonio Enciso-Moreno; Jose Arturo Martinez-Orozco; Jose Esteban Muñoz-Medina; Jose de Jesus Nuñez-Contreras; Juan Bautista Chale-Dzul; Julissa Enciso-Ibarra; Luis Alberto Ochoa-Carrera; Margarita Matias-Florentino; Maria Guadalupe Santiago-Mauricio; Maria Guadalupe de Jesus Mireles-Rivera; Mario Mujica-Sanchez; Marissa Perez-Garcia; Nelly Selem-Mojica; Pavel Isa; Ricardo Ciria Merce; Ricardo Grande; Rosa Maria Gutierrez Rios; Santiago avila-Rios; Selene Zarate; Susana Lopez; Veronica Mata-Haro; Victor Eduardo Garcia-Arias; Victor Hugo Borja-Aburto
EPI_ISL_2671543, EPI_ISL_2671619, EPI_ISL_2671624, EPI_ISL_2671716, EPI_ISL_2671719	Centro de Investigacion Biomedica del Noreste (CIBIN)	Unidad de Genomica Avanzada	; Alejandra Garcia-Gasca; Alejandra Hernandez-Teran; Alejandro Sanchez-Flores; Alfredo Herrera-Estrella; Alicia Ocaña-Mondragon; Andreu Comas-Garcia; Angel Gustavo Salas-Lais; Antonio Loza Roman; Bernardo Martinez-Miguel; Blanca Taboada; Brenda Irasema Maldonado-Meza; Bruno Gomez-Gil; Carla Ivon Herrera-Najera; Carlos F. Arias; Celia Boukadida; Celida Duque Molina; Celida Martinez- Rodriguez; Clara Esperanza Santacruz-Tinoco; Concepcion Grajales-Muñiz; Consorcio Mexicano de Vigilancia Genomica (CoViGen-Mex). Authors (in alphabetical order): Julio Elias Alvarado-Yaah; Cristobal Chaidez-Quiroz; Daniel Fregoso-Rueda; Daniel Lira Morales; Eduardo Becerril-Vargas; Fernando Fontove-Herrera; Fidencio Mejia-Nepomuceno; Francisco Pulido; Gloria Elena Espinosa-Ayala; Gloria Maria Molina-Salinas; Gloria Vazquez; Hector Esteban Paz-Juarez; Hector Montoya-Fuentes; Helen Haydee Fernanda Ramirez-Plascencia; Irvin Gonzalez-Lopez; Jean Pierre Gonzalez; Jesus Hernandez; Joel Armando Vazquez-Perez.; Jorge Salas-Hernandez; Jose Antonio Enciso-Moreno; Jose Arturo Martinez-Orozco; Jose Esteban Muñoz-Medina; Jose de Jesus Nuñez-Contreras; Juan Bautista Chale-Dzul; Julissa Enciso-Ibarra; Luis Alberto Ochoa-Carrera; Margarita Matias-Florentino; Maria Guadalupe Santiago-Mauricio; Maria Guadalupe de Jesus Mireles-Rivera; Mario Mujica-Sanchez; Marissa Perez-Garcia; Nelly Selem-Mojica; Pavel Isa; Ricardo Ciria Merce; Ricardo Grande; Rosa Maria Gutierrez Rios; Santiago avila-Rios; Selene Zarate; Susana Lopez; Veronica Mata-Haro; Victor Eduardo Garcia-Arias; Victor Hugo Borja-Aburto
EPI_ISL_2490382,	Centro de Investigación Biomédica de	Centro de Investigación en	; Alejandra Garcia-Gasca; Alejandra Hernández-Terán; Alejandro Sanchez-Flores; Alfredo Herrera-Estrella; Alicia Ocaña-Mondragón; Andreu Comas-García; Angel Gustavo Salas-Lais; Antonio Loza Román; Bernardo Martínez-Miguel; Blanca Taboada; Brenda Irasema Maldonado-Meza; Bruno Gomez-Gil; Carla

EPI_ISL_2490383, EPI_ISL_2490384	Occidente (CIBO)	Enfermedades Infecciosas (CIENI), Instituto Nacional de Enfermedades Respiratorias (INER)	Ivón Herrera-Najera; Carlos F. Arias; Celia Boukadida; Clara Esperanza Santacruz-Tinoco; Concepción Grajales-Muñiz; Consorcio Mexicano de Vigilancia Genómica (CoViGen-Mex). Authors (in alphabetical order): Julio Elias Alvarado-Yaah; Cristóbal Cháidez-Quiróz; Célida Duque Molina; Célida Martínez-Rodríguez; Daniel Fregoso-Rueda; Daniel Lira Morales; Eduardo Becerril-Vargas; Fernando Fontove-Herrera; Fidencio Mejía-Nepomuceno; Francisco Pulido; Gloria Elena Espinosa-Ayala; Gloria María Molina-Salinas; Gloria Vazquez; Hector Esteban Paz-Juárez; Hector Montoya-Fuentes; Helen Haydee Fernanda Ramírez-Plascencia; Irvin González-López; Jean Pierre González; Joel Armando Vázquez-Pérez.; Jorge Salas-Hernández; José Antonio Enciso-Moreno; José Arturo Martínez-Orozco; José Esteban Muñoz-Medina; José de Jesús Nuñez-Contreras; Juan Bautista Chale-Dzul; Julissa Enciso-Ibarra; Luis Alberto Ochoa-Carrera; Margarita Matías-Florentino; Mario Mújica-Sánchez; Marissa Perez-Garcia; María Guadalupe Santiago-Mauricio; María Guadalupe de Jesús Mireles-Rivera; Nelly Sélem-Mojica; Pavel Isa; Ricardo Ciria Merce; Ricardo Grande; Rosa María Gutierrez Rios; Santiago Ávila-Ríos; Selene Zárate; Susana Lopez; Victor Eduardo Garcia-Arias; Víctor Hugo Borja-Aburto
EPI_ISL_2402171	Centro de Investigación Biomédica de Occidente (CIBO)	Unidad de Genomica Avanzada	Alejandro Sanchez-Flores; Alfredo Herrera-Estrella; Alicia Ocana-Mondragon; Angel Gustavo Salas-Lais; Bernardo Martinez-Miguel; Blanca Taboada; Brenda Irasema Maldonado-Meza; Carla Ivon Herrera-Najera; Carlos F. Arias; Celia Boukadida; Clara Esperanza Santacruz-Tinoco; Concepcion Grajales-Muniz; Consorcio Mexicano de Vigilancia Genomica (CoViGen-Mex). Authors (in alphabetical order): Julio Elias Alvarado-Yaah; Fernando Fontove-Herrera; Francisco Pulido; Gloria Elena Espinoza-Ayala; Gloria Maria Molina-Salinas; Gloria Vazquez; Hector Esteban Paz-Juarez; Hector Montoya-Fuentes; Helen Haydee Fernanda Ramirez-Plascencia; Jorge Ivan Salinal-Nevarez; Jose Antonio Enciso-Moreno; Jose Esteban Munoz-Medina; Jose de Jesus Nunez-Contreras; Juan Bautista Chale-Dzul; Luis Alberto Ochoa-Carrera; Margarita Matias-Florentino; Maria Guadalupe Santiago-Mauricio; Maria Guadalupe de Jesus Mireles-Rivera; Nelly Selem-Mojica; Pavel Isa; Ricardo Grande; Santiago Avila-Rios; Victor Hugo Borja-Aburto
EPI_ISL_2490359, EPI_ISL_2490360, EPI_ISL_2490361, EPI_ISL_2490364, EPI_ISL_2490365, EPI_ISL_2490366	Centro de Investigación Biomédica del Noreste (CIBIN)	Centro de Investigación en Enfermedades Infecciosas (CIENI), Instituto Nacional de Enfermedades Respiratorias (INER)	; Alejandra Garcia-Gasca; Alejandra Hernández-Terán; Alejandro Sanchez-Flores; Alfredo Herrera-Estrella; Alicia Ocaña-Mondragón; Andreu Comas-García; Angel Gustavo Salas-Lais; Antonio Loza Román; Bernardo Martínez-Miguel; Blanca Taboada; Brenda Irasema Maldonado-Meza; Bruno Gomez-Gil; Carla Ivón Herrera-Najera; Carlos F. Arias; Celia Boukadida; Clara Esperanza Santacruz-Tinoco; Concepción Grajales-Muñiz; Consorcio Mexicano de Vigilancia Genómica (CoViGen-Mex). Authors (in alphabetical order): Julio Elias Alvarado-Yaah; Cristóbal Cháidez-Quiróz; Célida Duque Molina; Célida Martínez-Rodríguez; Daniel Fregoso-Rueda; Daniel Lira Morales; Eduardo Becerril-Vargas; Fernando Fontove-Herrera; Fidencio Mejía-Nepomuceno; Francisco Pulido; Gloria Elena Espinosa-Ayala; Gloria María Molina-Salinas; Gloria Vazquez; Hector Esteban Paz-Juárez; Hector Montoya-Fuentes; Helen Haydee Fernanda Ramírez-Plascencia; Irvin González-López; Jean Pierre González; Joel Armando Vázquez-Pérez.; Jorge Salas-Hernández; José Antonio Enciso-Moreno; José Arturo Martínez-Orozco; José Esteban Muñoz-Medina; José de Jesús Nuñez-Contreras; Juan Bautista Chale-Dzul; Julissa Enciso-Ibarra; Luis Alberto Ochoa-Carrera; Margarita Matías-Florentino; Mario Mújica-Sánchez; Marissa Perez-Garcia; María Guadalupe Santiago-Mauricio; María Guadalupe de Jesús Mireles-Rivera; Nelly Sélem-Mojica; Pavel Isa; Ricardo Ciria Merce; Ricardo Grande; Rosa María Gutierrez Rios; Santiago Ávila-Ríos; Selene Zárate; Susana Lopez; Victor Eduardo Garcia-Arias; Víctor Hugo Borja-Aburto
EPI_ISL_2401953, EPI_ISL_2401979, EPI_ISL_2401991	Centro de Investigación Biomédica del Noreste (CIBIN)	Unidad de Genomica Avanzada	Alejandro Sanchez-Flores; Alfredo Herrera-Estrella; Alicia Ocana-Mondragon; Angel Gustavo Salas-Lais; Bernardo Martinez-Miguel; Blanca Taboada; Brenda Irasema Maldonado-Meza; Carla Ivon Herrera-Najera; Carlos F. Arias; Celia Boukadida; Clara Esperanza Santacruz-Tinoco; Concepcion Grajales-Muniz; Consorcio Mexicano de Vigilancia Genomica (CoViGen-Mex). Authors (in alphabetical order): Julio Elias Alvarado-Yaah; Fernando Fontove-Herrera; Francisco Pulido; Gloria Elena Espinoza-Ayala; Gloria Maria Molina-Salinas; Gloria Vazquez; Hector Esteban Paz-Juarez; Hector Montoya-Fuentes; Helen Haydee Fernanda Ramirez-Plascencia; Jorge Ivan Salinal-Nevarez; Jose Antonio Enciso-Moreno; Jose Esteban Munoz-Medina; Jose de Jesus Nunez-Contreras; Juan Bautista Chale-Dzul; Luis Alberto Ochoa-Carrera; Margarita Matias-Florentino; Maria Guadalupe Santiago-Mauricio; Maria Guadalupe de Jesus Mireles-Rivera; Nelly Selem-Mojica; Pavel Isa; Ricardo Grande; Santiago Avila-Rios; Victor Hugo Borja-Aburto
EPI_ISL_2004300, EPI_ISL_2004301	Cintramedica	Instituto Nacional de Saude (INSA)	Borges et al
EPI_ISL_2258223, EPI_ISL_2258229, EPI_ISL_2258231, EPI_ISL_2258240, EPI_ISL_2258241, EPI_ISL_2258252, EPI_ISL_2273859, EPI_ISL_2617566, EPI_ISL_3074103			
see above	City of Milwaukee Health Department Laboratory	City of Milwaukee Health Department Laboratory	Amy Bauer; Jennifer Lentz; Manjeet Khubbar; Samantha Scott; Sanjib Bhattacharyya
EPI_ISL_2467143, EPI_ISL_2467152, EPI_ISL_2467174, EPI_ISL_2467217	Clinica INDISA	"Facultad de Ciencias de la Vida, UNAB"	"Claudio Meneses; Ariel Orellana"; Claudio Olmos; Daniel Leon; Dayan Sanhueza; Eduardo Castro; Gonzalo Campaña; Macarena Bastias; Paola Pidal; Ricardo Yusta; Sebastian Wolter; Susana Saez; Víctor Monreal; Waldo Diaz
EPI_ISL_2098734, EPI_ISL_2380856	Clinical Molecular Microbiology Laboratory, UNC Hospitals	Jeremy Wang	Alexander Rubinsteyn; Brianna de la Houssaye; Colleen Rice; Corbin Jones; Jason Smedberg; Jeremy Wang; Jose Luis Torres Castillo; Melissa Miller; Robert Hagan; Shawn Hawken
EPI_ISL_1805786	Clinical Reference Laboratory	Kansas Health and Environmental Lab	Ben Olsen; Jonathan Barnell; Mike Grose; and Phil Adam
EPI_ISL_2408426, EPI_ISL_2466571	Clinical Virology	Clinical Bacteriology	Adrian Egli; Alfredo Mari; Fanny Wegner; Hans Hirsch; Helena MB Seth-Smith; Julia Bielicki; Karoline Leuzinger; Manuel Battegay; Tim Roloff
EPI_ISL_2309507, EPI_ISL_2309567, EPI_ISL_2309879, EPI_ISL_2309958, EPI_ISL_2309972, EPI_ISL_2310745, EPI_ISL_2549913, EPI_ISL_2549925, EPI_ISL_2549993, EPI_ISL_2550187, EPI_ISL_2550261, EPI_ISL_2550275, EPI_ISL_2550311, EPI_ISL_2987419, EPI_ISL_3160136, EPI_ISL_3160137, EPI_ISL_3583885, EPI_ISL_3842256, EPI_ISL_3842258			
see above	Colorado Department of Public Health and Environment	Colorado Department of Public Health and Environment	Alexandria Rossheim; Diana Ir; Emily A. Travanty; Laura Bankers; Mandy Waters; Michael Martin; Molly C. Hetherington-Rauth; Sarah Elizabeth Totten; Shannon R. Matzinger
EPI_ISL_2285125	Complejo Hospitalario Universitario de Pontevedra	Microbiology Department. Complexo Hospitalario Universitario de Vigo	Campello M; Perez S; Regueiro B; Trigo M
EPI_ISL_2496914	Contra Costa County Public Health Lab	Chan-Zuckerberg Biohub	CZB Cliahub Consortium
EPI_ISL_3663778, EPI_ISL_3663779, EPI_ISL_3663781, EPI_ISL_3663784, EPI_ISL_3663785, EPI_ISL_3663786, EPI_ISL_3663789, EPI_ISL_3663791, EPI_ISL_3663792, EPI_ISL_3663793, EPI_ISL_3663795, EPI_ISL_3663796, EPI_ISL_3663797, EPI_ISL_3663798, EPI_ISL_3663799, EPI_ISL_3663800, EPI_ISL_3663801, EPI_ISL_3663802, EPI_ISL_3663804, EPI_ISL_3663805, EPI_ISL_3663806, EPI_ISL_3663808, EPI_ISL_3663809, EPI_ISL_3663810, EPI_ISL_3663811, EPI_ISL_3663812, EPI_ISL_3663813, EPI_ISL_3663816, EPI_ISL_3663822, EPI_ISL_3663823, EPI_ISL_3663824, EPI_ISL_3663826, EPI_ISL_3663828, EPI_ISL_3663829, EPI_ISL_3663830, EPI_ISL_3663832, EPI_ISL_3663833, EPI_ISL_3663835, EPI_ISL_3663836, EPI_ISL_3663837, EPI_ISL_3663839, EPI_ISL_3663840, EPI_ISL_3663841, EPI_ISL_3663845, EPI_ISL_3663846, EPI_ISL_3663848, EPI_ISL_3663850, EPI_ISL_3663851, EPI_ISL_3663852, EPI_ISL_3663853, EPI_ISL_3663854, EPI_ISL_3663859, EPI_ISL_3663861, EPI_ISL_3663862, EPI_ISL_3663863, EPI_ISL_3663865, EPI_ISL_3663866, EPI_ISL_3663867, EPI_ISL_3663870, EPI_ISL_3663871, EPI_ISL_3663872, EPI_ISL_3663874, EPI_ISL_3663878, EPI_ISL_3663879, EPI_ISL_3663880, EPI_ISL_3663881, EPI_ISL_3663882, EPI_ISL_3663883, EPI_ISL_3663886, EPI_ISL_3663888, EPI_ISL_3663889, EPI_ISL_3663891, EPI_ISL_3663893, EPI_ISL_3663894, EPI_ISL_3663900, EPI_ISL_3663906, EPI_ISL_3663907, EPI_ISL_3663910, EPI_ISL_3663911, EPI_ISL_3663912, EPI_ISL_3663913, EPI_ISL_3663916, EPI_ISL_3663917, EPI_ISL_3663918, EPI_ISL_3663920, EPI_ISL_3663922, EPI_ISL_3663923, EPI_ISL_3663924, EPI_ISL_3663926, EPI_ISL_3663927, EPI_ISL_3663928, EPI_ISL_3663930, EPI_ISL_3663931, EPI_ISL_3663932, EPI_ISL_3663934, EPI_ISL_3663935, EPI_ISL_3663937, EPI_ISL_3663939, EPI_ISL_3663941, EPI_ISL_3663942, EPI_ISL_3663943, EPI_ISL_3663944, EPI_ISL_3663947, EPI_ISL_3663950, EPI_ISL_3663951, EPI_ISL_3663952, EPI_ISL_3663953, EPI_ISL_3663954, EPI_ISL_3663955, EPI_ISL_3663956, EPI_ISL_3663957, EPI_ISL_3663958, EPI_ISL_3663959, EPI_ISL_3663962, EPI_ISL_3663964, EPI_ISL_3663965, EPI_ISL_3663966, EPI_ISL_3663967, EPI_ISL_3663968, EPI_ISL_3663969, EPI_ISL_3663971, EPI_ISL_3663975, EPI_ISL_3663977, EPI_ISL_3663978, EPI_ISL_3663980, EPI_ISL_3663981, EPI_ISL_3663982, EPI_ISL_3663983, EPI_ISL_3663984, EPI_ISL_3663985, EPI_ISL_3663986, EPI_ISL_3663989, EPI_ISL_3663991, EPI_ISL_3663992, EPI_ISL_3663995, EPI_ISL_3663996, EPI_ISL_3663997, EPI_ISL_3663998, EPI_ISL_3664001, EPI_ISL_3664002, EPI_ISL_3664003, EPI_ISL_3664004, EPI_ISL_3664005, EPI_ISL_3664006, EPI_ISL_3664007, EPI_ISL_3664009, EPI_ISL_3664010, EPI_ISL_3664012, EPI_ISL_3664014, EPI_ISL_3664016, EPI_ISL_3664017, EPI_ISL_3664018, EPI_ISL_3664020, EPI_ISL_3664021, EPI_ISL_3664025, EPI_ISL_3664026, EPI_ISL_3664027, EPI_ISL_3664028, EPI_ISL_3664029, EPI_ISL_3664030, EPI_ISL_3664032, EPI_ISL_3664033, EPI_ISL_3664034, EPI_ISL_3664036, EPI_ISL_3664037, EPI_ISL_3664038, EPI_ISL_3664043, EPI_ISL_3664045, EPI_ISL_3664046, EPI_ISL_3664048, EPI_ISL_3664049, EPI_ISL_3664050, EPI_ISL_3664051, EPI_ISL_3664053, EPI_ISL_3664054, EPI_ISL_3664055, EPI_ISL_3664057, EPI_ISL_3664058, EPI_ISL_3664059, EPI_ISL_3664062, EPI_ISL_3664063, EPI_ISL_3664065, EPI_ISL_3664067, EPI_ISL_3664068, EPI_ISL_3664070, EPI_ISL_3664073, EPI_ISL_3664077, EPI_ISL_3664078, EPI_ISL_3664079, EPI_ISL_3664080, EPI_ISL_3664084, EPI_ISL_3664086, EPI_ISL_3664087, EPI_ISL_3664088, EPI_ISL_3664089, EPI_ISL_3664093, EPI_ISL_3664094, EPI_ISL_3664095, EPI_ISL_3664096, EPI_ISL_3664098, EPI_ISL_3664101, EPI_ISL_3664106, EPI_ISL_3664107, EPI_ISL_3664108, EPI_ISL_3664109, EPI_ISL_3664110, EPI_ISL_3664111, EPI_ISL_3664113, EPI_ISL_3664114, EPI_ISL_3664124, EPI_ISL_3664125, EPI_ISL_3664127, EPI_ISL_3664130, EPI_ISL_3664132, EPI_ISL_3664133, EPI_ISL_3664134, EPI_ISL_3664135, EPI_ISL_3664136, EPI_ISL_3664138, EPI_ISL_3664139, EPI_ISL_3664140, EPI_ISL_3664141, EPI_ISL_3664142, EPI_ISL_3664143, EPI_ISL_3664144, EPI_ISL_3664148, EPI_ISL_3664149, EPI_ISL_3664150, EPI_ISL_3664151, EPI_ISL_3664154, EPI_ISL_3664155, EPI_ISL_3664158, EPI_ISL_3664159, EPI_ISL_3664161, EPI_ISL_3664162, EPI_ISL_3664164, EPI_ISL_3664165, EPI_ISL_3664170, EPI_ISL_3664173, EPI_ISL_3664177, EPI_ISL_3664178, EPI_ISL_3664180, EPI_ISL_3664181, EPI_ISL_3664182, EPI_ISL_3664183, EPI_ISL_3664187, EPI_ISL_3664188, EPI_ISL_3664190, EPI_ISL_3664192, EPI_ISL_3664193, EPI_ISL_3664194, EPI_ISL_3664195, EPI_ISL_3664196, EPI_ISL_3664199, EPI_ISL_3664200, EPI_ISL_3664202, EPI_ISL_3664203, EPI_ISL_3664204, EPI_ISL_3664205, EPI_ISL_3664208, EPI_ISL_3664210, EPI_ISL_3664211, EPI_ISL_3664212, EPI_ISL_3664213, EPI_ISL_3664214, EPI_ISL_3664215, EPI_ISL_3664216, EPI_ISL_3664217, EPI_ISL_3664218, EPI_ISL_3664221, EPI_ISL_3664222, EPI_ISL_3664223, EPI_ISL_3664224, EPI_ISL_3664225, EPI_ISL_3664226, EPI_ISL_3664227, EPI_ISL_3664228, EPI_ISL_3664229, EPI_ISL_3664230, EPI_ISL_3664231, EPI_ISL_3664232, EPI_ISL_3664234, EPI_ISL_3664235, EPI_ISL_3664236, EPI_ISL_3664237, EPI_ISL_3664238, EPI_ISL_3664239, EPI_ISL_3664244, EPI_ISL_3664245, EPI_ISL_3664246, EPI_ISL_3664249, EPI_ISL_3664251, EPI_ISL_3664252, EPI_ISL_3664253, EPI_ISL_3664256, EPI_ISL_3664257, EPI_ISL_3664258, EPI_ISL_3664259, EPI_ISL_3664261, EPI_ISL_3664263, EPI_ISL_3664264, EPI_ISL_3664267, EPI_ISL_3664268, EPI_ISL_3664269, EPI_ISL_3664271, EPI_ISL_3664273			
see above	DASA	DASA	Adriano Bonaldi; Angelica Hristov; Annelise Lopes; Bianca Cota; Cristina Oliveira; Jose Levi; Lidia Yamamoto; Paulo Pierry; Rodrigo Guarischi; Rodrigo Salazar
EPI_ISL_2229246, EPI_ISL_2451775	DC Public Health Lab/ Dept. of Forensic Sciences	Centers for Disease Control and Prevention Division of Viral Diseases, Pathogen Discovery	Alison Laufer Halpin; Ben L. Rambo-Martin; Clinton R. Paden; Dakota Howard; Darlene Wagner; Dave Wentworth; Dhwani Batra; Jasmine Padilla; Justin Lee; Katie Dillon; Krista Queen; Kristen Knipe; Kristine Lacek; Mark Burroughs; Matthew Schmerer; Mili Sheth; Peter Cook; Sam Shepard; Sarah Nobles; Shoshona Le; Suxiang Tong; Vivien Dugan; Yvette Unoarumhi
EPI_ISL_2228976, EPI_ISL_2452011, EPI_ISL_2840444	DE Public Health Laboratory	Centers for Disease Control and Prevention Division of Viral Diseases, Pathogen Discovery	Alison Laufer Halpin; Ben L. Rambo-Martin; Clinton R. Paden; Dakota Howard; Darlene Wagner; Dave Wentworth; Dhwani Batra; Jasmine Padilla; Justin Lee; Katie Dillon; Krista Queen; Kristen Knipe; Kristine Lacek; Mark Burroughs; Matthew Schmerer; Mili Sheth; Peter Cook; Sam Shepard; Sarah Nobles; Shoshona Le; Suxiang Tong; Vivien Dugan; Yvette Unoarumhi
EPI_ISL_2493631, EPI_ISL_2493632, EPI_ISL_2493634, EPI_ISL_2493636, EPI_ISL_2493638, EPI_ISL_2493639, EPI_ISL_2493641, EPI_ISL_2493642			
see above	DEPARTAMENTO DE SAUDE MUNICIPAL SOCORRO SP	Instituto Butantan	Antonio Jorge Martins; Claudia Renata dos Santos Barros; David Schlesinger; Debora Botequio Moretti; Dimas Tadeu Covas; Elaine Cristina Marqueze; Elaine Vieira Santos; Evandra Strazza Rodrigues; Heidge Fukumasu; Jayme Augusto de Souza-Neto; José Salvatore Leister Patané; Luiz Alcantara; Luiz Lehmann Coutinho; Maria Carolina Elias; Maurício Lacerda Nogueira; Rafael dos Santos Bezerra; Raul Machado Neto; Rejane Maria Tommasini Grotto; Ricardo Haddad; Sandra Coccuzzo Sampaio Vessoni; Simone Kashima; Svetoslav Nanev Slavov; Vincent Louis Viala
EPI_ISL_2345240, EPI_ISL_2345241, EPI_ISL_2345243, EPI_ISL_2345244, EPI_ISL_2345249, EPI_ISL_2345252, EPI_ISL_2345254, EPI_ISL_2345255, EPI_ISL_2345257, EPI_ISL_2345258, EPI_ISL_2345259, EPI_ISL_2345260			
see above	DEPARTAMENTO DE SAUDE MUNICIPAL SOCORRO SP	Instituto Butantan / ESALQ-Piracicaba	Antonio Jorge Martins; Claudia Renata dos Santos Barros; David Schlesinger; Debora Botequio Moretti; Dimas Tadeu Covas; Elaine Cristina Marqueze; Elaine Vieira Santos; Evandra Strazza Rodrigues; Heidge Fukumasu; Jayme Augusto de Souza-Neto; José Salvatore Leister Patané; Luiz Alcantara; Luiz Lehmann Coutinho; Maria Carolina Elias; Maurício Lacerda Nogueira; Rafael dos Santos Bezerra; Raul Machado Neto; Rejane Maria Tommasini Grotto; Ricardo Haddad; Sandra Coccuzzo Sampaio Vessoni; Simone Kashima; Svetoslav Nanev Slavov; Vincent Louis Viala
EPI_ISL_2209045, EPI_ISL_2209370, EPI_ISL_2209739, EPI_ISL_2345679, EPI_ISL_2345683, EPI_ISL_2345732, EPI_ISL_2345776, EPI_ISL_2345799, EPI_ISL_2345827, EPI_ISL_2345871			
see above	DEPARTAMENTO DE SAUDE MUNICIPAL SOCORRO SP	Instituto Butantan / Mendelics	Antonio Jorge Martins; Claudia Renata dos Santos Barros; David Schlesinger; Debora Botequio Moretti; Dimas Tadeu Covas; Elaine Cristina Marqueze; Elaine Vieira Santos; Evandra Strazza Rodrigues; Heidge Fukumasu; Jayme Augusto de Souza-Neto; José Salvatore Leister Patané; Luiz Alcantara; Luiz Lehmann Coutinho; Maria Carolina Elias; Maurício Lacerda Nogueira; Rafael dos Santos Bezerra; Raul Machado Neto; Rejane Maria Tommasini Grotto; Ricardo Haddad; Sandra Coccuzzo Sampaio Vessoni; Simone Kashima; Svetoslav Nanev Slavov; Vincent Louis Viala
EPI_ISL_2445177, EPI_ISL_2445178, EPI_ISL_2445180, EPI_ISL_2445182, EPI_ISL_2445184, EPI_ISL_2445186	DIRETORIA MUNICIPAL DE SAUDE	Instituto Butantan	Antonio Jorge Martins; Claudia Renata dos Santos Barros; David Schlesinger; Debora Botequio Moretti; Dimas Tadeu Covas; Elaine Cristina Marqueze; Elaine Vieira Santos; Evandra Strazza Rodrigues; Heidge Fukumasu; Jayme Augusto de Souza-Neto; José Salvatore Leister Patané; Luiz Alcantara; Luiz Lehmann Coutinho; Maria Carolina Elias; Maurício Lacerda Nogueira; Rafael dos Santos Bezerra; Raul Machado Neto; Rejane Maria Tommasini Grotto; Ricardo Haddad; Sandra Coccuzzo Sampaio Vessoni; Simone Kashima; Svetoslav Nanev Slavov; Vincent Louis Viala
EPI_ISL_2493374	DIRETORIA MUNICIPAL DE SAUDE DE ENGENHEIRO COELHO	Instituto Butantan	Antonio Jorge Martins; Claudia Renata dos Santos Barros; David Schlesinger; Debora Botequio Moretti; Dimas Tadeu Covas; Elaine Cristina Marqueze; Elaine Vieira Santos; Evandra Strazza Rodrigues; Heidge Fukumasu; Jayme Augusto de Souza-Neto; José Salvatore Leister Patané; Luiz Alcantara; Luiz Lehmann Coutinho; Maria Carolina Elias; Maurício Lacerda Nogueira; Rafael dos Santos Bezerra; Raul Machado Neto; Rejane Maria Tommasini Grotto; Ricardo Haddad; Sandra Coccuzzo Sampaio Vessoni; Simone Kashima; Svetoslav Nanev Slavov; Vincent Louis Viala
EPI_ISL_2322910	DOHMH PHL	New York City Public Health Laboratory	Jade Wang; et al.
EPI_ISL_2383713, EPI_ISL_2383852, EPI_ISL_2383864, EPI_ISL_2383877	DPHL	Delaware Public Health Lab	Rebecca Savage
EPI_ISL_2429555, EPI_ISL_2430305, EPI_ISL_2430615, EPI_ISL_2431115, EPI_ISL_2431486, EPI_ISL_2431537	Department of Bacteria, Parasites and Fungi, Statens Serum Institut, Copenhagen, Denmark	Statens Serum Institut Bioinformatics and Microbial Genomics	Danish Covid-19 Genome Consortium
EPI_ISL_2099850, EPI_ISL_2099851	Department of Public Health Bucharest	National Institute of Infectious Diseases-Prof. Dr. Matei Bals Molecular Diagnostics Laboratory	Andreea Tudor; Corina Casangiu; Dan Otelea; Leontina Banica; Marius Surleac; Ovidiu Vlaicu; Simona Paraschiv
EPI_ISL_2608834	Department of Virology and	Department of Virology, Faculty	Essi Korhonen; Hanna Jarva; Hanna Liimatainen; Hannimari Kallio-Kokko; Harri Kangas; Hussein Alburkat; Jenni Virtanen; Maija Lappalainen; Maija Suvanto; Olli Vapalahti; Pekka Ellonen; Phuoc Truong; Ravi Kant; Sari Hannula; Satu Kurkela; Teemu Smura

	Immunology, University of Helsinki and Helsinki University Hospital, Huslab Finland	of Medicine, University of Helsinki, Helsinki, Finland	
EPI_ISL_2403852, EPI_ISL_2403858, EPI_ISL_2403859	Dianalabs SA	Genesupport	Tanguy ARAUD
EPI_ISL_3578151, EPI_ISL_3578168, EPI_ISL_3578189	Dr. Risch Ostschweiz AG	Microbiology, Dr. Risch	Dominique Fabien Hilti; Faina Wehrli; Lorenz Risch; Martin Risch; Nadia Wohlwend; Sinem Kas; Thomas Bodmer
EPI_ISL_1962255, EPI_ISL_1962278, EPI_ISL_1962279, EPI_ISL_2093856, EPI_ISL_2093859, EPI_ISL_2093861, EPI_ISL_2093862, EPI_ISL_2093863, EPI_ISL_2093870, EPI_ISL_2093881, EPI_ISL_2093883, EPI_ISL_2093885, EPI_ISL_2217848, EPI_ISL_2219019, EPI_ISL_2219022, EPI_ISL_2219024, EPI_ISL_2219035, EPI_ISL_2219050, EPI_ISL_2219052, EPI_ISL_2219059, EPI_ISL_2219068, EPI_ISL_2219102, EPI_ISL_2303365, EPI_ISL_2303375, EPI_ISL_2303390, EPI_ISL_2303391, EPI_ISL_2405523, EPI_ISL_2405534, EPI_ISL_2405886, EPI_ISL_2405922, EPI_ISL_2406150, EPI_ISL_2406247, EPI_ISL_2406269, EPI_ISL_2406366, EPI_ISL_2406457, EPI_ISL_2475681, EPI_ISL_2475682, EPI_ISL_2475689, EPI_ISL_2475705, EPI_ISL_2475708, EPI_ISL_2673227, EPI_ISL_2673236			
see above	Dutch COVID-19 response team	National Institute for Public Health and the Environment (RIVM)	Adam Meijer; AnneMarie van den Brandt; Annelies Kroneman; Bas van der Veer; Chantal Reusken; Dennis Schmitz; Dirk Eggink; Euníce Then; Florian Zwagemaker; Harry Vennema; James Groot; Jeroen Cremer; Karim Hajji; Kim Freriks; Linda van de Nes; Lisa Wijsman; Lynn Aarts; Melissa van Tuil; Ryanne Jaarsma; Sanne Bos; Sharon van den Brink; Sjoerd Kuiling; on behalf of the national COVID-19 response team
EPI_ISL_4060100, EPI_ISL_4060104, EPI_ISL_4060105	Dynacare Manitoba	Manitoba Cadham Provincial Laboratory	David Alexander; Hilary Racher; Hongbin Li; Melissa Desaulnier; Tintu Abraham
EPI_ISL_2344869	E S F DR EDSON GIANETTO JD CARLOS CASSETARI	Instituto Butantan / ESALQ-Piracicaba	Antonio Jorge Martins; Claudia Renata dos Santos Barros; David Schlesinger; Debora Botequio Moretti; Dimas Tadeu Covas; Elaine Cristina Marqueze; Elaine Vieira Santos; Evandra Strazza Rodrigues; Heidge Fukumasu; Jayme Augusto de Souza-Neto; José Salvatore Leister Patané; Luiz Alcantara; Luiz Lehmann Coutinho; Maria Carolina Elias; Maurício Lacerda Nogueira; Rafael dos Santos Bezerra; Raul Machado Neto; Rejane Maria Tommasini Grotto; Ricardo Haddad; Sandra Coccuzzo Sampaio Vessoni; Simone Kashima; Svetoslav Nanev Slavov; Vincent Louis Viala
EPI_ISL_2344877	E S F WALDO DSTEFANO JD PRIMAVERA JOSE BONIFACIO	Instituto Butantan / ESALQ-Piracicaba	Antonio Jorge Martins; Claudia Renata dos Santos Barros; David Schlesinger; Debora Botequio Moretti; Dimas Tadeu Covas; Elaine Cristina Marqueze; Elaine Vieira Santos; Evandra Strazza Rodrigues; Heidge Fukumasu; Jayme Augusto de Souza-Neto; José Salvatore Leister Patané; Luiz Alcantara; Luiz Lehmann Coutinho; Maria Carolina Elias; Maurício Lacerda Nogueira; Rafael dos Santos Bezerra; Raul Machado Neto; Rejane Maria Tommasini Grotto; Ricardo Haddad; Sandra Coccuzzo Sampaio Vessoni; Simone Kashima; Svetoslav Nanev Slavov; Vincent Louis Viala
EPI_ISL_1941841	EHNV	Laboratory of genomics and metagenomics	Claire Bertelli; Damien Jacot; Gilbert Greub; Sebastien Aeby; Trestan Pillonel
EPI_ISL_2493401, EPI_ISL_2493408	EMERGENCIA RESPIRATORIA DE NOVA GRANADA	Instituto Butantan	Antonio Jorge Martins; Claudia Renata dos Santos Barros; David Schlesinger; Debora Botequio Moretti; Dimas Tadeu Covas; Elaine Cristina Marqueze; Elaine Vieira Santos; Evandra Strazza Rodrigues; Heidge Fukumasu; Jayme Augusto de Souza-Neto; José Salvatore Leister Patané; Luiz Alcantara; Luiz Lehmann Coutinho; Maria Carolina Elias; Maurício Lacerda Nogueira; Rafael dos Santos Bezerra; Raul Machado Neto; Rejane Maria Tommasini Grotto; Ricardo Haddad; Sandra Coccuzzo Sampaio Vessoni; Simone Kashima; Svetoslav Nanev Slavov; Vincent Louis Viala
EPI_ISL_2344834, EPI_ISL_2344835, EPI_ISL_2344845, EPI_ISL_2344861	EMERGENCIA RESPIRATORIA DE NOVA GRANADA	Instituto Butantan / ESALQ-Piracicaba	Antonio Jorge Martins; Claudia Renata dos Santos Barros; David Schlesinger; Debora Botequio Moretti; Dimas Tadeu Covas; Elaine Cristina Marqueze; Elaine Vieira Santos; Evandra Strazza Rodrigues; Heidge Fukumasu; Jayme Augusto de Souza-Neto; José Salvatore Leister Patané; Luiz Alcantara; Luiz Lehmann Coutinho; Maria Carolina Elias; Maurício Lacerda Nogueira; Rafael dos Santos Bezerra; Raul Machado Neto; Rejane Maria Tommasini Grotto; Ricardo Haddad; Sandra Coccuzzo Sampaio Vessoni; Simone Kashima; Svetoslav Nanev Slavov; Vincent Louis Viala
EPI_ISL_2345128	ESF COHAB IV	Instituto Butantan / ESALQ-Piracicaba	Antonio Jorge Martins; Claudia Renata dos Santos Barros; David Schlesinger; Debora Botequio Moretti; Dimas Tadeu Covas; Elaine Cristina Marqueze; Elaine Vieira Santos; Evandra Strazza Rodrigues; Heidge Fukumasu; Jayme Augusto de Souza-Neto; José Salvatore Leister Patané; Luiz Alcantara; Luiz Lehmann Coutinho; Maria Carolina Elias; Maurício Lacerda Nogueira; Rafael dos Santos Bezerra; Raul Machado Neto; Rejane Maria Tommasini Grotto; Ricardo Haddad; Sandra Coccuzzo Sampaio Vessoni; Simone Kashima; Svetoslav Nanev Slavov; Vincent Louis Viala
EPI_ISL_2445535, EPI_ISL_2473790	ESF I E II	Instituto Butantan	Antonio Jorge Martins; Claudia Renata dos Santos Barros; David Schlesinger; Debora Botequio Moretti; Dimas Tadeu Covas; Elaine Cristina Marqueze; Elaine Vieira Santos; Evandra Strazza Rodrigues; Heidge Fukumasu; Jayme Augusto de Souza-Neto; José Salvatore Leister Patané; Luiz Alcantara; Luiz Lehmann Coutinho; Maria Carolina Elias; Maurício Lacerda Nogueira; Rafael dos Santos Bezerra; Raul Machado Neto; Rejane Maria Tommasini Grotto; Ricardo Haddad; Sandra Coccuzzo Sampaio Vessoni; Simone Kashima; Svetoslav Nanev Slavov; Vincent Louis Viala
EPI_ISL_2445515	ESF NOVA TANABI II	Instituto Butantan	Antonio Jorge Martins; Claudia Renata dos Santos Barros; David Schlesinger; Debora Botequio Moretti; Dimas Tadeu Covas; Elaine Cristina Marqueze; Elaine Vieira Santos; Evandra Strazza Rodrigues; Heidge Fukumasu; Jayme Augusto de Souza-Neto; José Salvatore Leister Patané; Luiz Alcantara; Luiz Lehmann Coutinho; Maria Carolina Elias; Maurício Lacerda Nogueira; Rafael dos Santos Bezerra; Raul Machado Neto; Rejane Maria Tommasini Grotto; Ricardo Haddad; Sandra Coccuzzo Sampaio Vessoni; Simone Kashima; Svetoslav Nanev Slavov; Vincent Louis Viala
EPI_ISL_2445157, EPI_ISL_2473706	ESF PARQUE COLINAS	Instituto Butantan	Antonio Jorge Martins; Claudia Renata dos Santos Barros; David Schlesinger; Debora Botequio Moretti; Dimas Tadeu Covas; Elaine Cristina Marqueze; Elaine Vieira Santos; Evandra Strazza Rodrigues; Heidge Fukumasu; Jayme Augusto de Souza-Neto; José Salvatore Leister Patané; Luiz Alcantara; Luiz Lehmann Coutinho; Maria Carolina Elias; Maurício Lacerda Nogueira; Rafael dos Santos Bezerra; Raul Machado Neto; Rejane Maria Tommasini Grotto; Ricardo Haddad; Sandra Coccuzzo Sampaio Vessoni; Simone Kashima; Svetoslav Nanev Slavov; Vincent Louis Viala
EPI_ISL_2445191, EPI_ISL_2445192, EPI_ISL_2473712	ESF PQ UNIVERSITARIO DE ASSIS	Instituto Butantan	Antonio Jorge Martins; Claudia Renata dos Santos Barros; David Schlesinger; Debora Botequio Moretti; Dimas Tadeu Covas; Elaine Cristina Marqueze; Elaine Vieira Santos; Evandra Strazza Rodrigues; Heidge Fukumasu; Jayme Augusto de Souza-Neto; José Salvatore Leister Patané; Luiz Alcantara; Luiz Lehmann Coutinho; Maria Carolina Elias; Maurício Lacerda Nogueira; Rafael dos Santos Bezerra; Raul Machado Neto; Rejane Maria Tommasini Grotto; Ricardo Haddad; Sandra Coccuzzo Sampaio Vessoni; Simone Kashima; Svetoslav Nanev Slavov; Vincent Louis Viala
EPI_ISL_2344870	ESF RURAL DR JOSE CICERO FONTES XAVIER	Instituto Butantan / ESALQ-Piracicaba	Antonio Jorge Martins; Claudia Renata dos Santos Barros; David Schlesinger; Debora Botequio Moretti; Dimas Tadeu Covas; Elaine Cristina Marqueze; Elaine Vieira Santos; Evandra Strazza Rodrigues; Heidge Fukumasu; Jayme Augusto de Souza-Neto; José Salvatore Leister Patané; Luiz Alcantara; Luiz Lehmann Coutinho; Maria Carolina Elias; Maurício Lacerda Nogueira; Rafael dos Santos Bezerra; Raul Machado Neto; Rejane Maria Tommasini Grotto; Ricardo Haddad; Sandra Coccuzzo Sampaio Vessoni; Simone Kashima; Svetoslav Nanev Slavov; Vincent Louis Viala
EPI_ISL_2445169, EPI_ISL_2445176	ESF VILA GLORIA I	Instituto Butantan	Antonio Jorge Martins; Claudia Renata dos Santos Barros; David Schlesinger; Debora Botequio Moretti; Dimas Tadeu Covas; Elaine Cristina Marqueze; Elaine Vieira Santos; Evandra Strazza Rodrigues; Heidge Fukumasu; Jayme Augusto de Souza-Neto; José Salvatore Leister Patané; Luiz Alcantara; Luiz Lehmann Coutinho; Maria Carolina Elias; Maurício Lacerda Nogueira; Rafael dos Santos Bezerra; Raul Machado Neto; Rejane Maria Tommasini Grotto; Ricardo Haddad; Sandra Coccuzzo Sampaio Vessoni; Simone Kashima; Svetoslav Nanev Slavov; Vincent Louis Viala
EPI_ISL_2445171, EPI_ISL_2473816	ESF VILA PROGRESSO	Instituto Butantan	Antonio Jorge Martins; Claudia Renata dos Santos Barros; David Schlesinger; Debora Botequio Moretti; Dimas Tadeu Covas; Elaine Cristina Marqueze; Elaine Vieira Santos; Evandra Strazza Rodrigues; Heidge Fukumasu; Jayme Augusto de Souza-Neto; José Salvatore Leister Patané; Luiz Alcantara; Luiz Lehmann Coutinho; Maria Carolina Elias; Maurício Lacerda Nogueira; Rafael dos Santos Bezerra; Raul Machado Neto; Rejane Maria Tommasini Grotto; Ricardo Haddad; Sandra Coccuzzo Sampaio Vessoni; Simone Kashima; Svetoslav Nanev Slavov; Vincent Louis Viala
EPI_ISL_2445158, EPI_ISL_2445160	ESF VITORIA	Instituto Butantan	Antonio Jorge Martins; Claudia Renata dos Santos Barros; David Schlesinger; Debora Botequio Moretti; Dimas Tadeu Covas; Elaine Cristina Marqueze; Elaine Vieira Santos; Evandra Strazza Rodrigues; Heidge Fukumasu; Jayme Augusto de Souza-Neto; José Salvatore Leister Patané; Luiz Alcantara; Luiz Lehmann Coutinho; Maria Carolina Elias; Maurício Lacerda Nogueira; Rafael dos Santos Bezerra; Raul Machado Neto; Rejane Maria Tommasini Grotto; Ricardo Haddad; Sandra Coccuzzo Sampaio Vessoni; Simone Kashima; Svetoslav Nanev Slavov; Vincent Louis Viala
EPI_ISL_3215305, EPI_ISL_3215593, EPI_ISL_3215602, EPI_ISL_3215627, EPI_ISL_3215650, EPI_ISL_3215668, EPI_ISL_3215669, EPI_ISL_3215674, EPI_ISL_3215691, EPI_ISL_3215692, EPI_ISL_3215700, EPI_ISL_3215715, EPI_ISL_3215725, EPI_ISL_3215743, EPI_ISL_3215746			
see above	EXCITE Lab	Andersen lab at Scripps Research	Abigail Schnapper; Angela Scioscia; Cheryl Anderson; Chip Schooley; David Pride; Greg Humphrey; Helena Tubb; Natasha Martin; Natasha Martin Cheryl Anderson; Sawyer Farmer; Sharon Reed; Smruthi Karthikeyan; Tommy Valles + SEARCH; Tommy Valles + SEARCH
EPI_ISL_2346154, EPI_ISL_2346155, EPI_ISL_3758745	Emory Molecular Diagnostics Laboratory, Emory Healthcare	Piantadosi Lab, Emory Department of Pathology	Ahmed Babiker; Anne Piantadosi; Dara Khosravi
EPI_ISL_2316877, EPI_ISL_2316950, EPI_ISL_2316952, EPI_ISL_2316955, EPI_ISL_2317019, EPI_ISL_2317031, EPI_ISL_2471625, EPI_ISL_2471628, EPI_ISL_2471639, EPI_ISL_2471642, EPI_ISL_2471656			
see above	Eurofins LifeCodexx GmbH	Robert Koch Institute	
EPI_ISL_2445114, EPI_ISL_2493708, EPI_ISL_2493709, EPI_ISL_2493710, EPI_ISL_2493712	FACULDADE DE MEDICINA ABC	Instituto Butantan	Antonio Jorge Martins; Claudia Renata dos Santos Barros; David Schlesinger; Debora Botequio Moretti; Dimas Tadeu Covas; Elaine Cristina Marqueze; Elaine Vieira Santos; Evandra Strazza Rodrigues; Heidge Fukumasu; Jayme Augusto de Souza-Neto; José Salvatore Leister Patané; Luiz Alcantara; Luiz Lehmann Coutinho; Maria Carolina Elias; Maurício Lacerda Nogueira; Rafael dos Santos Bezerra; Raul Machado Neto; Rejane Maria Tommasini Grotto; Ricardo Haddad; Sandra Coccuzzo Sampaio Vessoni; Simone Kashima; Svetoslav Nanev Slavov; Vincent Louis Viala
EPI_ISL_3355485	FUNDACIÓN HOSPITAL SAN PEDRO	Corporacion CorpoGen-Universidad de los Andes-Universidad Central	Christian Romero; Jorge Duitama; Juan Manuel Anzola; Laura González; Maryam Chaib De Mares; María Mercedes Zambrano; Nelly Díaz; Patricia Del Portillo; Silvia Restrepo
EPI_ISL_2008736, EPI_ISL_2277740, EPI_ISL_2277956, EPI_ISL_2277957, EPI_ISL_2277962, EPI_ISL_2277963, EPI_ISL_2277965, EPI_ISL_2277968, EPI_ISL_2277969, EPI_ISL_2277971, EPI_ISL_2277973, EPI_ISL_2277974, EPI_ISL_2277975, EPI_ISL_2277976, EPI_ISL_2442745, EPI_ISL_2442785, EPI_ISL_2442795, EPI_ISL_2442809, EPI_ISL_2442831, EPI_ISL_2442833, EPI_ISL_2442946, EPI_ISL_2442947, EPI_ISL_2442965, EPI_ISL_2567092, EPI_ISL_2567093, EPI_ISL_2567099, EPI_ISL_2567147, EPI_ISL_2567164, EPI_ISL_2567165, EPI_ISL_2567178, EPI_ISL_2836028, EPI_ISL_2836031, EPI_ISL_2836105, EPI_ISL_2836330, EPI_ISL_2836449, EPI_ISL_2836676			
see above	Florida Bureau of Public Health Laboratories	Florida Bureau of Public Health Laboratories	Jason Blanton; Sarah Schmedes
EPI_ISL_2620708, EPI_ISL_2620954	Fondazione IRCCS Ca' Granda Ospedale Maggiore Policlinico	Fondazione IRCCS Ca' Granda Ospedale Maggiore Policlinico	Ferruccio Ceriotti; Sara Uceda Renteria
EPI_ISL_1839261, EPI_ISL_1839596, EPI_ISL_1839893, EPI_ISL_1839980, EPI_ISL_1839997, EPI_ISL_1840063, EPI_ISL_1840076, EPI_ISL_1840092, EPI_ISL_1840093, EPI_ISL_1840116, EPI_ISL_1992053, EPI_ISL_1992082, EPI_ISL_1992088, EPI_ISL_1992152, EPI_ISL_1992174, EPI_ISL_1992201, EPI_ISL_1992202, EPI_ISL_1992203, EPI_ISL_1992206, EPI_ISL_1992212, EPI_ISL_1992287, EPI_ISL_1992317, EPI_ISL_1992345, EPI_ISL_1992348, EPI_ISL_1992448, EPI_ISL_1992449, EPI_ISL_1992457, EPI_ISL_1992542, EPI_ISL_1992587, EPI_ISL_1992643, EPI_ISL_1992800, EPI_ISL_1992805, EPI_ISL_1992813, EPI_ISL_1992855, EPI_ISL_1992892, EPI_ISL_1992921, EPI_ISL_1992981, EPI_ISL_1992984, EPI_ISL_1992985, EPI_ISL_1993040, EPI_ISL_1993067, EPI_ISL_1993076, EPI_ISL_1993170, EPI_ISL_1993173, EPI_ISL_1993176, EPI_ISL_1993177, EPI_ISL_1993183, EPI_ISL_1993190, EPI_ISL_1993195, EPI_ISL_1993217, EPI_ISL_1993249, EPI_ISL_1993252, EPI_ISL_1993255, EPI_ISL_1993270, EPI_ISL_1993357, EPI_ISL_2043693, EPI_ISL_2043701, EPI_ISL_2043712, EPI_ISL_2043777, EPI_ISL_2043865, EPI_ISL_2043880, EPI_ISL_2043953, EPI_ISL_2043969, EPI_ISL_2043976, EPI_ISL_2043977, EPI_ISL_2043988, EPI_ISL_2133941, EPI_ISL_2133988, EPI_ISL_2134052, EPI_ISL_2134084, EPI_ISL_2134148, EPI_ISL_2134151, EPI_ISL_2134177, EPI_ISL_2134189, EPI_ISL_2134191, EPI_ISL_2134192, EPI_ISL_2134194, EPI_ISL_2134225, EPI_ISL_2134245, EPI_ISL_2134312, EPI_ISL_2134321, EPI_ISL_2134332, EPI_ISL_2134337, EPI_ISL_2134345, EPI_ISL_2134433, EPI_ISL_2134434, EPI_ISL_2134442, EPI_ISL_2134464, EPI_ISL_2134465, EPI_ISL_2134466, EPI_ISL_2134471, EPI_ISL_2134499, EPI_ISL_2134502, EPI_ISL_2134514, EPI_ISL_2160765, EPI_ISL_2160778, EPI_ISL_2160829, EPI_ISL_2160837, EPI_ISL_2160845, EPI_ISL_2160903, EPI_ISL_2160904, EPI_ISL_2160909, EPI_ISL_2160935, EPI_ISL_2160988, EPI_ISL_2161001, EPI_ISL_2161024, EPI_ISL_2179864, EPI_ISL_2179894, EPI_ISL_2179900, EPI_ISL_2269221, EPI_ISL_2269251, EPI_ISL_2269256, EPI_ISL_2269277, EPI_ISL_2269314, EPI_ISL_2269360, EPI_ISL_2269368, EPI_ISL_2269371, EPI_ISL_2269436, EPI_ISL_2269502, EPI_ISL_2269503, EPI_ISL_2269509, EPI_ISL_2269525, EPI_ISL_2280263, EPI_ISL_2368135, EPI_ISL_2371518, EPI_ISL_2371573, EPI_ISL_2371575, EPI_ISL_2371576, EPI_ISL_2371578, EPI_ISL_2371579, EPI_ISL_2371580, EPI_ISL_2371584, EPI_ISL_2371585, EPI_ISL_2371587, EPI_ISL_2371639, EPI_ISL_2441831, EPI_ISL_2480073, EPI_ISL_2525557, EPI_ISL_2525633, EPI_ISL_2526057			
see above	Fulgent Genetics	Centers for Disease Control and Prevention Division of Viral Diseases, Pathogen Discovery	Adrian Paskey; Becky Tsai; Benafsh Sapra; Benjamin Rambo-Martin; Christopher Gulvick; Clinton R. Paden; Dakota Howard; Darlene Wagner; Dhwani Batra; Doreen Ng; Duncan MacCannell; Harry Gao; James Xie; Jason Caravas; John Gao; Joseph Fierro; Kara Moser; Matthew Schmerer; Mickey Li; Peter W. Cook; Scott Sammons; Shatavia Morrison; Yan Meng; Yvette Unoarumhi
EPI_ISL_2550708	GA Department of Public Health	GA Department of Public Health	Aliyah Fields; Cynthia Dixey; Jonathan Edwards; Stacy Reeves; Taylor Smith; Tonia Parrott
EPI_ISL_2229306	GA Department of Public Health Laboratory	Centers for Disease Control and Prevention Division of Viral Diseases, Pathogen Discovery	Alison Laufer Halpin; Ben L. Rambo-Martin; Clinton R. Paden; Dakota Howard; Darlene Wagner; Dave Wentworth; Dhwani Batra; Jasmine Padilla; Justin Lee; Katie Dillon; Krista Queen; Kristen Knipe; Kristine Lacek; Mark Burroughs; Matthew Schmerer; Mili Sheth; Peter Cook; Sam Shepard; Sarah Nobles; Shoshona Le; Suxiang Tong; Vivien Dugan; Yvette Unoarumhi
EPI_ISL_2311749, EPI_ISL_2422457	GA Department of Public Health Laboratory	Genomics and Discovery, Respiratory Viruses Branch, Division of Viral Diseases, Centers for Disease Control and Prevention	Adam Retchless; Anna Kelleher; Anna Uehara; Brian Lynch; Clinton R. Paden; Dhwani Batra; Haibin Wang; Han Jia Justin Ng; Jasmine Padilla; Jing Zhang; Justin Lee; Krista Queen; Mark Burroughs; Mili Sheth; Morgan Davis; Peter Cook; Rachel Marine; Sarah Nobles; Suxiang Tong; Tara Coalter; Yan Li; Ying Tao
EPI_ISL_2228873	GH A.CHENEVIER-H.MONDOR	Department of Virology, Henri Mondor University Hospital, Assistance Publique Hôpitaux de Paris, Université Paris-Est Créteil, INSERM U955	Alexandre Soulier; Christophe Rodriguez; Elisabeth Trawinski; Guillaume Gricourt; Jean-Michel Pawlotsky; Melissa N'Debi; Slim Fourati; Vanessa Demontant

EPI_ISL_2421160, EPI_ISL_3031056	GLENS FALLS HOSPITAL LABORATORY	Wadsworth Center, New York State Department of Health	Alexis Russell; Catharine Prussing; Daryl M. Lamson; Erasmus Schneider; Erica Lasek-Nesselquist; John Kelly; Jonathan Plitnick; Kirsten St. George; Matthew Shudt; Melissa A Leisner; Navjot Singh
EPI_ISL_2345663, EPI_ISL_2345701, EPI_ISL_2345709, EPI_ISL_2345756	GRIPARIO FALCAO	Instituto Butantan / Mendelics	Antonio Jorge Martins; Claudia Renata dos Santos Barros; David Schlesinger; Debora Botequio Moretti; Dimas Tadeu Covas; Elaine Cristina Marqueze; Elaine Vieira Santos; Evandra Strazza Rodrigues; Heidge Fukumasu; Jayme Augusto de Souza-Neto; José Salvatore Leister Patané; Luiz Alcantara; Luiz Lehmann Coutinho; Maria Carolina Elias; Maurício Lacerda Nogueira; Rafael dos Santos Bezerra; Raul Machado Neto; Rejane Maria Tommasini Grotto; Ricardo Haddad; Sandra Coccuzzo Sampaio Vessoni; Simone Kashima; Svetoslav Nanev Slavov; Vincent Louis Viala
EPI_ISL_2228704, EPI_ISL_2333688	GROUPE HOSPITALIER SUD ILE DE FRANCE	Department of Virology, Henri Mondor University Hospital, Assistance Publique Hôpitaux de Paris, Université Paris-Est Créteil, INSERM U955	Alexandre Soulier; Christophe Rodriguez; Elisabeth Trawinski; Guillaume Gricourt; Jean-Michel Pawlotsky; Melissa N'Debi; Slim Fourati; Vanessa Demontant
EPI_ISL_2421228	Garnet Health Medical Center Laboratory	Wadsworth Center, New York State Department of Health	Alexis Russell; Catharine Prussing; Daryl M. Lamson; Erasmus Schneider; Erica Lasek-Nesselquist; John Kelly; Jonathan Plitnick; Kirsten St. George; Matthew Shudt; Melissa A Leisner; Navjot Singh
EPI_ISL_3491798, EPI_ISL_3491799, EPI_ISL_3491800, EPI_ISL_3491803, EPI_ISL_3491805, EPI_ISL_3491810, EPI_ISL_3491820, EPI_ISL_3491822, EPI_ISL_3491824, EPI_ISL_3491825, EPI_ISL_3491835			
see above	Gencore - Universidad de los Andes	Gencore - Universidad de los Andes	Catherine Jaller; Cristian Barrera; David González; Luisa Sacristan; Marcela Guevara; Silvia Restrepo
EPI_ISL_2009545, EPI_ISL_2009556, EPI_ISL_2009558, EPI_ISL_2009559, EPI_ISL_2391013, EPI_ISL_2391030, EPI_ISL_2391049, EPI_ISL_2391068, EPI_ISL_2391086, EPI_ISL_2391087, EPI_ISL_2391093, EPI_ISL_2391099, EPI_ISL_2391100, EPI_ISL_2391124, EPI_ISL_2391125, EPI_ISL_2391133, EPI_ISL_2391153, EPI_ISL_2391157, EPI_ISL_2391173, EPI_ISL_2391180, EPI_ISL_2391185, EPI_ISL_2391187, EPI_ISL_2391190, EPI_ISL_2391191, EPI_ISL_2391196, EPI_ISL_2391208, EPI_ISL_2391214, EPI_ISL_2391217, EPI_ISL_2391238, EPI_ISL_2391241, EPI_ISL_2391248, EPI_ISL_2391250, EPI_ISL_2391252, EPI_ISL_2391253, EPI_ISL_2391254, EPI_ISL_2391255, EPI_ISL_2391259, EPI_ISL_2391260, EPI_ISL_2391261, EPI_ISL_2391263, EPI_ISL_2391266, EPI_ISL_2391268, EPI_ISL_2391270, EPI_ISL_2391293, EPI_ISL_2391296, EPI_ISL_2391297, EPI_ISL_2391299, EPI_ISL_2391303, EPI_ISL_2391306, EPI_ISL_2391310, EPI_ISL_2391319, EPI_ISL_2391322, EPI_ISL_2391323, EPI_ISL_2391326, EPI_ISL_2391327, EPI_ISL_2391336, EPI_ISL_2391342, EPI_ISL_2391345, EPI_ISL_2391347, EPI_ISL_2391348, EPI_ISL_2391352, EPI_ISL_2391356, EPI_ISL_2391393, EPI_ISL_2391407, EPI_ISL_2391408, EPI_ISL_2391415, EPI_ISL_2391416, EPI_ISL_2391419, EPI_ISL_2391420, EPI_ISL_2391437, EPI_ISL_2391439, EPI_ISL_2391442, EPI_ISL_2391446, EPI_ISL_2391448, EPI_ISL_2391449, EPI_ISL_2391470, EPI_ISL_2391471, EPI_ISL_2391475, EPI_ISL_2391476, EPI_ISL_2508551, EPI_ISL_2508613, EPI_ISL_2508616, EPI_ISL_2509122, EPI_ISL_2509130, EPI_ISL_2509131, EPI_ISL_2509132, EPI_ISL_2509133, EPI_ISL_2509134, EPI_ISL_2509135, EPI_ISL_2509136, EPI_ISL_2509138, EPI_ISL_2509139, EPI_ISL_2509141, EPI_ISL_2509145, EPI_ISL_2509155, EPI_ISL_2509156, EPI_ISL_2509166, EPI_ISL_2509167, EPI_ISL_2509282, EPI_ISL_2509288, EPI_ISL_2509300, EPI_ISL_2509302, EPI_ISL_2509308, EPI_ISL_2509324, EPI_ISL_2543449, EPI_ISL_2543452, EPI_ISL_2543454, EPI_ISL_2543457, EPI_ISL_2543458, EPI_ISL_2543461, EPI_ISL_2543464, EPI_ISL_2543465, EPI_ISL_2543469, EPI_ISL_2543470, EPI_ISL_2543471, EPI_ISL_2543482, EPI_ISL_2543483, EPI_ISL_2543484, EPI_ISL_2543489, EPI_ISL_2543492, EPI_ISL_2543494, EPI_ISL_2543496, EPI_ISL_2543497, EPI_ISL_2543499, EPI_ISL_2543500, EPI_ISL_2543509, EPI_ISL_2543510, EPI_ISL_2543515, EPI_ISL_2543542, EPI_ISL_2543543, EPI_ISL_2543548, EPI_ISL_2543549, EPI_ISL_2543551, EPI_ISL_2543617, EPI_ISL_2543620, EPI_ISL_2557264, EPI_ISL_2557268, EPI_ISL_2566533, EPI_ISL_2597267, EPI_ISL_2597268, EPI_ISL_2597271, EPI_ISL_2597272, EPI_ISL_2597273, EPI_ISL_2597274, EPI_ISL_2597278, EPI_ISL_2597279, EPI_ISL_2597283, EPI_ISL_2892726			
see above	Genetica Molecular and Subdepartamento de Virologia ISP Chile	Instituto de Salud Publica de Chile	Andres Castillo; Barbara Parra; Constanza Campano; Gisselle Barra; Javier Tognarelli; Jorge Fernandez; Karen Orostica; Loredana Arata; Patricia Bustos; Rodrigo Fasce; Soledad Ulloa
EPI_ISL_2248888, EPI_ISL_2340184, EPI_ISL_2340221, EPI_ISL_2340225	Germano de Sousa	Instituto Nacional de Saude (INSA)	Borges et al
EPI_ISL_2340344	Germano de Sousa	Instituto Nacional de Saude (INSA) and BioSystems & Integrative Sciences Institute (BioISI) Genomics Unit, FCUL	Borges et al
EPI_ISL_2360225	Gesundheitsdirektion Zürich	Institute of Medical Virology	Alexandra Trkola; Annette Audigé; Cyril Shah; Gabriela Ziltener; Guido Bloemberg; Jon Huder; Jürg Böni; Kevin Steiner; Maria Grünberg; Maryam Zaheri; Michael Huber; Riccarda Capaul; Stefan Schmutz; Verena Kufner
EPI_ISL_2535843, EPI_ISL_2535845, EPI_ISL_2535863, EPI_ISL_2535873, EPI_ISL_2535878, EPI_ISL_2535904, EPI_ISL_2535977, EPI_ISL_2536023, EPI_ISL_2536025, EPI_ISL_2536036, EPI_ISL_2537097, EPI_ISL_2537099, EPI_ISL_2537108, EPI_ISL_2537109, EPI_ISL_2601808, EPI_ISL_2601821, EPI_ISL_2601869, EPI_ISL_2601908, EPI_ISL_2601911, EPI_ISL_2602027, EPI_ISL_2602034, EPI_ISL_2602055, EPI_ISL_2602060, EPI_ISL_2602076, EPI_ISL_2602079, EPI_ISL_2602080, EPI_ISL_2602099, EPI_ISL_2602149, EPI_ISL_2602248, EPI_ISL_2602278, EPI_ISL_2602309, EPI_ISL_2602381, EPI_ISL_2613840, EPI_ISL_2613860, EPI_ISL_2613863, EPI_ISL_2613873, EPI_ISL_2613877, EPI_ISL_2613885, EPI_ISL_2613889, EPI_ISL_2613892, EPI_ISL_2613908, EPI_ISL_2614004, EPI_ISL_2614011, EPI_ISL_2614012, EPI_ISL_2614024, EPI_ISL_2614027, EPI_ISL_2614039, EPI_ISL_2614055, EPI_ISL_2854839, EPI_ISL_2854858, EPI_ISL_2854860, EPI_ISL_2854917			
see above	Gravity Diagnostics, LLC	Gravity Diagnostics, LLC	Gravity Diagnostics
EPI_ISL_1745232, EPI_ISL_1960121, EPI_ISL_2157237, EPI_ISL_2157239, EPI_ISL_2157248, EPI_ISL_2376238, EPI_ISL_2376239, EPI_ISL_2376243, EPI_ISL_2376247, EPI_ISL_2376249, EPI_ISL_2500991			
see above	Gundersen Clinical Microbiology Laboratory	Kabara Cancer Research Institute	Craig S. Richmond; Paraic A. Kenny
EPI_ISL_3102213	HGCC HOSPITAL GERAL DR CESAR CALS	Analytical Competence Molecular Epidemiology Lab/ACME, Oswaldo Cruz Foundation, Ceara (FIOCRUZ CE)	Cleber Furtado Aksenen; Fabio Miyajima; Fernando Braga Stehling; Francisco Eder de Moura Lopes; Jamille Maria Mendes Bezerra; Joaquim César do Nascimento Sousa Junior; Pedro Miguel Carneiro Jeronimo; Suzana Porto Almeida e Lucas Delerino; Thais Ferreira de Oliveira; Thais de Oliveira Costa; Ticiane Cavalcante de Souza; Veridiana Pessoa Miyajima
EPI_ISL_1941834	HIB	Laboratory of genomics and metagenomics	Claire Bertelli; Damien Jacot; Gilbert Greub; Sebastien Aeby; Trestan Pillonel
EPI_ISL_2017370, EPI_ISL_2017372, EPI_ISL_2017384, EPI_ISL_2017385, EPI_ISL_2017386, EPI_ISL_2017387, EPI_ISL_2017389, EPI_ISL_2017390, EPI_ISL_2017432, EPI_ISL_2017434, EPI_ISL_2017435, EPI_ISL_2017437, EPI_ISL_2017438, EPI_ISL_2017439, EPI_ISL_2187985, EPI_ISL_2187987, EPI_ISL_2187988, EPI_ISL_2187990, EPI_ISL_2187991, EPI_ISL_2187992, EPI_ISL_2187994, EPI_ISL_2187995, EPI_ISL_2187997, EPI_ISL_2187999, EPI_ISL_2188001, EPI_ISL_2188002, EPI_ISL_2188004, EPI_ISL_2188005, EPI_ISL_2188012, EPI_ISL_2188013, EPI_ISL_2188014, EPI_ISL_2188015, EPI_ISL_2188016, EPI_ISL_2188017, EPI_ISL_2188018, EPI_ISL_2188019, EPI_ISL_2188020, EPI_ISL_2188021, EPI_ISL_2188023, EPI_ISL_2188024, EPI_ISL_2188025, EPI_ISL_2188027, EPI_ISL_2222900, EPI_ISL_2222902, EPI_ISL_2222905, EPI_ISL_2222911, EPI_ISL_2222916, EPI_ISL_2222917, EPI_ISL_2222918, EPI_ISL_2227564, EPI_ISL_2227565, EPI_ISL_2348604, EPI_ISL_2348605, EPI_ISL_2348606, EPI_ISL_2348607, EPI_ISL_2348608, EPI_ISL_2348609, EPI_ISL_2348610, EPI_ISL_2348611, EPI_ISL_2348612, EPI_ISL_2348614, EPI_ISL_2348615, EPI_ISL_2466448, EPI_ISL_2466449, EPI_ISL_2466450, EPI_ISL_2466451, EPI_ISL_2466452, EPI_ISL_2466454, EPI_ISL_2466455, EPI_ISL_2466456, EPI_ISL_2466457, EPI_ISL_2466458, EPI_ISL_2466459, EPI_ISL_2466460, EPI_ISL_2466461, EPI_ISL_2466462, EPI_ISL_2466464, EPI_ISL_2466465, EPI_ISL_2497437, EPI_ISL_2497438, EPI_ISL_2497439, EPI_ISL_2497441, EPI_ISL_2497442, EPI_ISL_2497465, EPI_ISL_2617584, EPI_ISL_2617585, EPI_ISL_2617586, EPI_ISL_3254316, EPI_ISL_3254321, EPI_ISL_3254465, EPI_ISL_4061646			
see above	HLAGYN - Laboratorio de Imunologia de Transplantes de Goias	HLAGYN - Laboratorio de Imunologia de Transplantes de Goias	Alessandro Leonardo Alvares Magalhaes; Daniel Ferreira de Sousa; Danielle de Paiva Rezende; Erika Lopes Rocha Batista; Fernando Antonio Vinhal dos Santos; Frederico Rodrigues Vinhal; Kamila Oliveira Reis De Freitas.; Lucas Carlos Gomes Pereira; Paola Cristina Resende Silva; Sabrina Sara Moreira Duarte
EPI_ISL_2209313, EPI_ISL_2344914, EPI_ISL_2344948	HOSP E MATERNIDADE MUNICIPAL N SRA MONTE SERRAT	Instituto Butantan / ESALQ-Piracicaba	Antonio Jorge Martins; Claudia Renata dos Santos Barros; David Schlesinger; Debora Botequio Moretti; Dimas Tadeu Covas; Elaine Cristina Marqueze; Elaine Vieira Santos; Evandra Strazza Rodrigues; Heidge Fukumasu; Jayme Augusto de Souza-Neto; José Salvatore Leister Patané; Luiz Alcantara; Luiz Lehmann Coutinho; Maria Carolina Elias; Maurício Lacerda Nogueira; Rafael dos Santos Bezerra; Raul Machado Neto; Rejane Maria Tommasini Grotto; Ricardo Haddad; Sandra Coccuzzo Sampaio Vessoni; Simone Kashima; Svetoslav Nanev Slavov; Vincent Louis Viala
EPI_ISL_2345764, EPI_ISL_2362254	HOSP E MATERNIDADE MUNICIPAL N SRA MONTE SERRAT	Instituto Butantan / Mendelics	Antonio Jorge Martins; Claudia Renata dos Santos Barros; David Schlesinger; Debora Botequio Moretti; Dimas Tadeu Covas; Elaine Cristina Marqueze; Elaine Vieira Santos; Evandra Strazza Rodrigues; Heidge Fukumasu; Jayme Augusto de Souza-Neto; José Salvatore Leister Patané; Luiz Alcantara; Luiz Lehmann Coutinho; Maria Carolina Elias; Maurício Lacerda Nogueira; Rafael dos Santos Bezerra; Raul Machado Neto; Rejane Maria Tommasini Grotto; Ricardo Haddad; Sandra Coccuzzo Sampaio Vessoni; Simone Kashima; Svetoslav Nanev Slavov; Vincent Louis Viala
EPI_ISL_2473728, EPI_ISL_2473729, EPI_ISL_2473749, EPI_ISL_2473758, EPI_ISL_2473759	HOSP GERAL DE SAO PAULO	Instituto Butantan	Antonio Jorge Martins; Claudia Renata dos Santos Barros; David Schlesinger; Debora Botequio Moretti; Dimas Tadeu Covas; Elaine Cristina Marqueze; Elaine Vieira Santos; Evandra Strazza Rodrigues; Heidge Fukumasu; Jayme Augusto de Souza-Neto; José Salvatore Leister Patané; Luiz Alcantara; Luiz Lehmann Coutinho; Maria Carolina Elias; Maurício Lacerda Nogueira; Rafael dos Santos Bezerra; Raul Machado Neto; Rejane Maria Tommasini Grotto; Ricardo Haddad; Sandra Coccuzzo Sampaio Vessoni; Simone Kashima; Svetoslav Nanev Slavov; Vincent Louis Viala
EPI_ISL_2345110	HOSP MAT 8 DE MAIO	Instituto Butantan / ESALQ-Piracicaba	Antonio Jorge Martins; Claudia Renata dos Santos Barros; David Schlesinger; Debora Botequio Moretti; Dimas Tadeu Covas; Elaine Cristina Marqueze; Elaine Vieira Santos; Evandra Strazza Rodrigues; Heidge Fukumasu; Jayme Augusto de Souza-Neto; José Salvatore Leister Patané; Luiz Alcantara; Luiz Lehmann Coutinho; Maria Carolina Elias; Maurício Lacerda Nogueira; Rafael dos Santos Bezerra; Raul Machado Neto; Rejane Maria Tommasini Grotto; Ricardo Haddad; Sandra Coccuzzo Sampaio Vessoni; Simone Kashima; Svetoslav Nanev Slavov; Vincent Louis Viala
EPI_ISL_2209024, EPI_ISL_2345061, EPI_ISL_2345064, EPI_ISL_2345074, EPI_ISL_2345075	HOSP MUN CIDADE TIRADENTES CARMEN PRUDENTE	Instituto Butantan / ESALQ-Piracicaba	Antonio Jorge Martins; Claudia Renata dos Santos Barros; David Schlesinger; Debora Botequio Moretti; Dimas Tadeu Covas; Elaine Cristina Marqueze; Elaine Vieira Santos; Evandra Strazza Rodrigues; Heidge Fukumasu; Jayme Augusto de Souza-Neto; José Salvatore Leister Patané; Luiz Alcantara; Luiz Lehmann Coutinho; Maria Carolina Elias; Maurício Lacerda Nogueira; Rafael dos Santos Bezerra; Raul Machado Neto; Rejane Maria Tommasini Grotto; Ricardo Haddad; Sandra Coccuzzo Sampaio Vessoni; Simone Kashima; Svetoslav Nanev Slavov; Vincent Louis Viala
EPI_ISL_2209383, EPI_ISL_2345043, EPI_ISL_2345067, EPI_ISL_2345068, EPI_ISL_2345071, EPI_ISL_2345072, EPI_ISL_2345073			
see above	HOSP MUN DE MOGI DAS CRUZES PREF WALDEMAR COSTA FILHO	Instituto Butantan / ESALQ-Piracicaba	Antonio Jorge Martins; Claudia Renata dos Santos Barros; David Schlesinger; Debora Botequio Moretti; Dimas Tadeu Covas; Elaine Cristina Marqueze; Elaine Vieira Santos; Evandra Strazza Rodrigues; Heidge Fukumasu; Jayme Augusto de Souza-Neto; José Salvatore Leister Patané; Luiz Alcantara; Luiz Lehmann Coutinho; Maria Carolina Elias; Maurício Lacerda Nogueira; Rafael dos Santos Bezerra; Raul Machado Neto; Rejane Maria Tommasini Grotto; Ricardo Haddad; Sandra Coccuzzo Sampaio Vessoni; Simone Kashima; Svetoslav Nanev Slavov; Vincent Louis Viala
EPI_ISL_2345076, EPI_ISL_2345081, EPI_ISL_2345104, EPI_ISL_2345108	HOSP MUN JOSANIAS CASTANHA BRAGA	Instituto Butantan / ESALQ-Piracicaba	Antonio Jorge Martins; Claudia Renata dos Santos Barros; David Schlesinger; Debora Botequio Moretti; Dimas Tadeu Covas; Elaine Cristina Marqueze; Elaine Vieira Santos; Evandra Strazza Rodrigues; Heidge Fukumasu; Jayme Augusto de Souza-Neto; José Salvatore Leister Patané; Luiz Alcantara; Luiz Lehmann Coutinho; Maria Carolina Elias; Maurício Lacerda Nogueira; Rafael dos Santos Bezerra; Raul Machado Neto; Rejane Maria Tommasini Grotto; Ricardo Haddad; Sandra Coccuzzo Sampaio Vessoni; Simone Kashima; Svetoslav Nanev Slavov; Vincent Louis Viala
EPI_ISL_3102518	HOSPITAL MATERNIDADE DR LUIS DE GONZAGA DA FONSECA MOTA	Analytical Competence Molecular Epidemiology Lab/ACME, Oswaldo Cruz Foundation, Ceara (FIOCRUZ CE)	Cleber Furtado Aksenen; Fabio Miyajima; Fernando Braga Stehling; Francisco Eder de Moura Lopes; Jamille Maria Mendes Bezerra; Joaquim César do Nascimento Sousa Junior; Pedro Miguel Carneiro Jeronimo; Suzana Porto Almeida e Lucas Delerino; Thais Ferreira de Oliveira; Thais de Oliveira Costa; Ticiane Cavalcante de Souza; Veridiana Pessoa Miyajima
EPI_ISL_2100346	HOSPITAL CAN MISSES	HOSPITAL UNIVERSITARIO SON ESPASES	Antonio Oliver; Carla López-Causapé; Pablo Fraile-Ribot; SeqCovid
EPI_ISL_3102255	HOSPITAL CURA DARS	Analytical Competence Molecular Epidemiology Lab/ACME, Oswaldo Cruz Foundation, Ceara (FIOCRUZ CE)	Cleber Furtado Aksenen; Fabio Miyajima; Fernando Braga Stehling; Francisco Eder de Moura Lopes; Jamille Maria Mendes Bezerra; Joaquim César do Nascimento Sousa Junior; Pedro Miguel Carneiro Jeronimo; Suzana Porto Almeida e Lucas Delerino; Thais Ferreira de Oliveira; Thais de Oliveira Costa; Ticiane Cavalcante de Souza; Veridiana Pessoa Miyajima
EPI_ISL_2345044, EPI_ISL_2345045, EPI_ISL_2345066	HOSPITAL DE CAMPANHA COVID 19 CAIEIRAS	Instituto Butantan / ESALQ-Piracicaba	Antonio Jorge Martins; Claudia Renata dos Santos Barros; David Schlesinger; Debora Botequio Moretti; Dimas Tadeu Covas; Elaine Cristina Marqueze; Elaine Vieira Santos; Evandra Strazza Rodrigues; Heidge Fukumasu; Jayme Augusto de Souza-Neto; José Salvatore Leister Patané; Luiz Alcantara; Luiz Lehmann Coutinho; Maria Carolina Elias; Maurício Lacerda Nogueira; Rafael dos Santos Bezerra; Raul Machado Neto; Rejane Maria Tommasini Grotto; Ricardo Haddad; Sandra Coccuzzo Sampaio Vessoni; Simone Kashima; Svetoslav Nanev Slavov; Vincent Louis Viala
EPI_ISL_2445571, EPI_ISL_2445572, EPI_ISL_2445577, EPI_ISL_2445579, EPI_ISL_2445583, EPI_ISL_2445588, EPI_ISL_2445591, EPI_ISL_2473800			
see above	HOSPITAL DE CAMPANHA COVID 19 MUNICIPIO DE TAUBATE	Instituto Butantan	Antonio Jorge Martins; Claudia Renata dos Santos Barros; David Schlesinger; Debora Botequio Moretti; Dimas Tadeu Covas; Elaine Cristina Marqueze; Elaine Vieira Santos; Evandra Strazza Rodrigues; Heidge Fukumasu; Jayme Augusto de Souza-Neto; José Salvatore Leister Patané; Luiz Alcantara; Luiz Lehmann Coutinho; Maria Carolina Elias; Maurício Lacerda Nogueira; Rafael dos Santos Bezerra; Raul Machado Neto; Rejane Maria Tommasini Grotto; Ricardo Haddad; Sandra Coccuzzo Sampaio Vessoni; Simone Kashima; Svetoslav Nanev Slavov; Vincent Louis Viala
EPI_ISL_2344955, EPI_ISL_2344956, EPI_ISL_2344957, EPI_ISL_2344973, EPI_ISL_2344975, EPI_ISL_2344977, EPI_ISL_2344978, EPI_ISL_2344980, EPI_ISL_2344997, EPI_ISL_2345005, EPI_ISL_2345008, EPI_ISL_2345011, EPI_ISL_2345018, EPI_ISL_2345021			

see above	HOSPITAL DE CAMPANHA COVID 19 MUNICIPIO DE TAUBATE	Instituto Butantan / ESALQ-Piracicaba	Antonio Jorge Martins; Claudia Renata dos Santos Barros; David Schlesinger; Debora Botequio Moretti; Dimas Tadeu Covas; Elaine Cristina Marqueze; Elaine Vieira Santos; Evandra Strazza Rodrigues; Heidge Fukumasu; Jayme Augusto de Souza-Neto; José Salvatore Leister Patané; Luiz Alcantara; Luiz Lehmann Coutinho; Maria Carolina Elias; Maurício Lacerda Nogueira; Rafael dos Santos Bezerra; Raul Machado Neto; Rejane Maria Tommasini Grotto; Ricardo Haddad; Sandra Coccuzzo Sampaio Vessoni; Simone Kashima; Svetoslav Nanev Slavov; Vincent Louis Viala
EPI_ISL_2209101, EPI_ISL_2209363, EPI_ISL_2209883, EPI_ISL_2210101, EPI_ISL_2210236, EPI_ISL_2345722, EPI_ISL_2345731, EPI_ISL_2345819, EPI_ISL_2345826, EPI_ISL_2345870			
see above	HOSPITAL DE CAMPANHA COVID 19 MUNICIPIO DE TAUBATE	Instituto Butantan / Mendelics	Antonio Jorge Martins; Claudia Renata dos Santos Barros; David Schlesinger; Debora Botequio Moretti; Dimas Tadeu Covas; Elaine Cristina Marqueze; Elaine Vieira Santos; Evandra Strazza Rodrigues; Heidge Fukumasu; Jayme Augusto de Souza-Neto; José Salvatore Leister Patané; Luiz Alcantara; Luiz Lehmann Coutinho; Maria Carolina Elias; Maurício Lacerda Nogueira; Rafael dos Santos Bezerra; Raul Machado Neto; Rejane Maria Tommasini Grotto; Ricardo Haddad; Sandra Coccuzzo Sampaio Vessoni; Simone Kashima; Svetoslav Nanev Slavov; Vincent Louis Viala
EPI_ISL_2344848, EPI_ISL_2344849	HOSPITAL DE MISERICORDIA ALTINOPOLIS	Instituto Butantan / ESALQ-Piracicaba	Antonio Jorge Martins; Claudia Renata dos Santos Barros; David Schlesinger; Debora Botequio Moretti; Dimas Tadeu Covas; Elaine Cristina Marqueze; Elaine Vieira Santos; Evandra Strazza Rodrigues; Heidge Fukumasu; Jayme Augusto de Souza-Neto; José Salvatore Leister Patané; Luiz Alcantara; Luiz Lehmann Coutinho; Maria Carolina Elias; Maurício Lacerda Nogueira; Rafael dos Santos Bezerra; Raul Machado Neto; Rejane Maria Tommasini Grotto; Ricardo Haddad; Sandra Coccuzzo Sampaio Vessoni; Simone Kashima; Svetoslav Nanev Slavov; Vincent Louis Viala
EPI_ISL_2493305, EPI_ISL_2493306, EPI_ISL_2493307, EPI_ISL_2493308, EPI_ISL_2493309, EPI_ISL_2493312, EPI_ISL_2493316, EPI_ISL_2493318, EPI_ISL_2493320, EPI_ISL_2493321, EPI_ISL_2493322, EPI_ISL_2493323, EPI_ISL_2493324, EPI_ISL_2493325, EPI_ISL_2493330, EPI_ISL_2493331, EPI_ISL_2493334			
see above	HOSPITAL DO RIM E HIPERTENSAO	Instituto Butantan	Antonio Jorge Martins; Claudia Renata dos Santos Barros; David Schlesinger; Debora Botequio Moretti; Dimas Tadeu Covas; Elaine Cristina Marqueze; Elaine Vieira Santos; Evandra Strazza Rodrigues; Heidge Fukumasu; Jayme Augusto de Souza-Neto; José Salvatore Leister Patané; Luiz Alcantara; Luiz Lehmann Coutinho; Maria Carolina Elias; Maurício Lacerda Nogueira; Rafael dos Santos Bezerra; Raul Machado Neto; Rejane Maria Tommasini Grotto; Ricardo Haddad; Sandra Coccuzzo Sampaio Vessoni; Simone Kashima; Svetoslav Nanev Slavov; Vincent Louis Viala
EPI_ISL_2345131	HOSPITAL DR OSIRIS FLORINDO COELHO FERRAZ DE VASCONCELOS	Instituto Butantan / ESALQ-Piracicaba	Antonio Jorge Martins; Claudia Renata dos Santos Barros; David Schlesinger; Debora Botequio Moretti; Dimas Tadeu Covas; Elaine Cristina Marqueze; Elaine Vieira Santos; Evandra Strazza Rodrigues; Heidge Fukumasu; Jayme Augusto de Souza-Neto; José Salvatore Leister Patané; Luiz Alcantara; Luiz Lehmann Coutinho; Maria Carolina Elias; Maurício Lacerda Nogueira; Rafael dos Santos Bezerra; Raul Machado Neto; Rejane Maria Tommasini Grotto; Ricardo Haddad; Sandra Coccuzzo Sampaio Vessoni; Simone Kashima; Svetoslav Nanev Slavov; Vincent Louis Viala
EPI_ISL_2493370	HOSPITAL E MATERNIDADE MADRE VANNINI	Instituto Butantan	Antonio Jorge Martins; Claudia Renata dos Santos Barros; David Schlesinger; Debora Botequio Moretti; Dimas Tadeu Covas; Elaine Cristina Marqueze; Elaine Vieira Santos; Evandra Strazza Rodrigues; Heidge Fukumasu; Jayme Augusto de Souza-Neto; José Salvatore Leister Patané; Luiz Alcantara; Luiz Lehmann Coutinho; Maria Carolina Elias; Maurício Lacerda Nogueira; Rafael dos Santos Bezerra; Raul Machado Neto; Rejane Maria Tommasini Grotto; Ricardo Haddad; Sandra Coccuzzo Sampaio Vessoni; Simone Kashima; Svetoslav Nanev Slavov; Vincent Louis Viala
EPI_ISL_2344982	HOSPITAL E MATERNIDADE NOSSA SENHORA DA AJUDA	Instituto Butantan / ESALQ-Piracicaba	Antonio Jorge Martins; Claudia Renata dos Santos Barros; David Schlesinger; Debora Botequio Moretti; Dimas Tadeu Covas; Elaine Cristina Marqueze; Elaine Vieira Santos; Evandra Strazza Rodrigues; Heidge Fukumasu; Jayme Augusto de Souza-Neto; José Salvatore Leister Patané; Luiz Alcantara; Luiz Lehmann Coutinho; Maria Carolina Elias; Maurício Lacerda Nogueira; Rafael dos Santos Bezerra; Raul Machado Neto; Rejane Maria Tommasini Grotto; Ricardo Haddad; Sandra Coccuzzo Sampaio Vessoni; Simone Kashima; Svetoslav Nanev Slavov; Vincent Louis Viala
EPI_ISL_2209292	HOSPITAL E MATERNIDADE NOSSA SENHORA DA AJUDA	Instituto Butantan / Mendelics	Antonio Jorge Martins; Claudia Renata dos Santos Barros; David Schlesinger; Debora Botequio Moretti; Dimas Tadeu Covas; Elaine Cristina Marqueze; Elaine Vieira Santos; Evandra Strazza Rodrigues; Heidge Fukumasu; Jayme Augusto de Souza-Neto; José Salvatore Leister Patané; Luiz Alcantara; Luiz Lehmann Coutinho; Maria Carolina Elias; Maurício Lacerda Nogueira; Rafael dos Santos Bezerra; Raul Machado Neto; Rejane Maria Tommasini Grotto; Ricardo Haddad; Sandra Coccuzzo Sampaio Vessoni; Simone Kashima; Svetoslav Nanev Slavov; Vincent Louis Viala
EPI_ISL_2209269, EPI_ISL_2209315, EPI_ISL_2344899, EPI_ISL_2344911, EPI_ISL_2344944, EPI_ISL_2344945, EPI_ISL_2344946			
see above	HOSPITAL E PRONTO SOCORRO PORTINARI	Instituto Butantan	Antonio Jorge Martins; Claudia Renata dos Santos Barros; David Schlesinger; Debora Botequio Moretti; Dimas Tadeu Covas; Elaine Cristina Marqueze; Elaine Vieira Santos; Evandra Strazza Rodrigues; Heidge Fukumasu; Jayme Augusto de Souza-Neto; José Salvatore Leister Patané; Luiz Alcantara; Luiz Lehmann Coutinho; Maria Carolina Elias; Maurício Lacerda Nogueira; Rafael dos Santos Bezerra; Raul Machado Neto; Rejane Maria Tommasini Grotto; Ricardo Haddad; Sandra Coccuzzo Sampaio Vessoni; Simone Kashima; Svetoslav Nanev Slavov; Vincent Louis Viala; Vincent Louis Viala.
EPI_ISL_1967299	HOSPITAL E PRONTO SOCORRO PORTINARI	Instituto Butantan / Mendelics	Antonio Jorge Martins; Bianca Cechetto Carlos. Mendelics: Bibiana Santos; Claudia Renata dos Santos Barros; Cíntia Bittar; David Schlesinger. Hemocentro Ribeirão Preto: Simone Kashima; Debora Botequio Moretti; Elaine Cristina Marqueze; Elaine Vieira dos Santos; Elisangela Chicaroni Mattos; Erika Freitas; Evandra Strazza Rodrigues; Felipe Allan da Silva da Costa; Flavia Aburjaile; Fábio Sossai Possebon; Guilherme Campos; Guilherme Targino Valente; Heidge Fukumasu. USP-Botucatu: Rejane Maria Tommasini Grotto; Helena Lage Ferreira; Instituto Butantan: Dimas Tadeu Covas; Jardelina de Souza Todao Bernardino; Jayme A. Souza-Neto; Jessika Cristina Chagas Lesbon; Jorge A. Petroli Marchesi; José Salvatore Leister Patané; João Paulo Kitajima; João Pessoa Araújo Jr.; Leila Sabrina Ullmann; Loyze Paola Oliveira de Lima; Luiz Aurelio de Campos Crispin. Centro de Genômica Funcional da ESALQ: Luiz Lehmann Coutinho; Luiz Carlos Junior de Alcantara; Lívia Sacchetto; Maisa C. Pereira Parra; Maria Carolina Elias; Marta Giovanetti; Marília Moraes; Maurício Lacerda Nogueira. Prefeitura de Sao Paulo: Melissa Palmieri.; Patricia Akemi Assato; Paula Rahal; Paulo Inacio da Costa; Rafael dos Santos Bezerra; Raquel de Lello Rocha Campos Cassano. NGS Soluções Genômicas: Pilar Drummond Sampaio Corrêa Mariani. FZEA-USP Pirassununga: Mirele Daiana Poleti; Raul Machado Neto; Ricardo Augusto Brassaloti; Ricardo Haddad; Rodrigo Tocantins Calado. FAMERP-SJRP: Cecília Artico Banho; Sandra Coccuzzo Sampaio; Svetoslav Nanev Slavov; Vagner Fonseca; Vincent Louis Viala
EPI_ISL_2345029	HOSPITAL ESCOLA DA UNIVERSIDADE DE TAUBATE	Instituto Butantan / ESALQ-Piracicaba	Antonio Jorge Martins; Claudia Renata dos Santos Barros; David Schlesinger; Debora Botequio Moretti; Dimas Tadeu Covas; Elaine Cristina Marqueze; Elaine Vieira Santos; Evandra Strazza Rodrigues; Heidge Fukumasu; Jayme Augusto de Souza-Neto; José Salvatore Leister Patané; Luiz Alcantara; Luiz Lehmann Coutinho; Maria Carolina Elias; Maurício Lacerda Nogueira; Rafael dos Santos Bezerra; Raul Machado Neto; Rejane Maria Tommasini Grotto; Ricardo Haddad; Sandra Coccuzzo Sampaio Vessoni; Simone Kashima; Svetoslav Nanev Slavov; Vincent Louis Viala
EPI_ISL_3102276, EPI_ISL_3102289, EPI_ISL_3102290, EPI_ISL_3102317	HOSPITAL ESTADUAL LEONARDO DA VINCI	Analytical Competence Molecular Epidemiology Lab/ACME, Oswaldo Cruz Foundation, Ceara (FIOCRUZ CE)	Cleber Furtado Aksenen; Fabio Miyajima; Fernando Braga Stehling; Francisco Eder de Moura Lopes; Jamille Maria Mendes Bezerra; Joaquim César do Nascimento Sousa Junior; Pedro Miguel Carneiro Jeronimo; Suzana Porto Almeida e Lucas Delerino; Thais Ferreira de Oliveira; Thais de Oliveira Costa; Ticiane Cavalcante de Souza; Veridiana Pessoa Miyajima
EPI_ISL_3260869	HOSPITAL GENERAL UNIVERSITARIO DE ALBACETE	Instituto de Salud Carlos III	A. Monzón; CARIDAD; F. Casas; I. Jiménez; I. SAINZ DE BARANDA CAMINO; M. Camarero; P. Zaballos; S. Cuesta; S. Iglesias-Caballero; S. Pozo; S. Varona; Sandonís; V. Vázquez-Morón
EPI_ISL_3260824, EPI_ISL_3260876	HOSPITAL GENERAL UNIVERSITARIO DE GUADALAJARA	Instituto de Salud Carlos III	A. Monzón; ALEJANDRO; F. Casas; I. Jiménez; I.GONZALEZ PRAETORIUS; Iglesias-Caballero; M. Sandonís; P. Zaballos; S. Camarero; S. Cuesta; S. Iglesias-Caballero; S. Pozo; S. Varona; V. Camarero; V. Vázquez-Morón; Vázquez-Morón
EPI_ISL_3260840	HOSPITAL GENERAL UNIVERSITARIO SANTA LUCIA	Instituto de Salud Carlos III	A. Monzón; ANA; F. Casas; I. Jiménez; I.BLAZQUEZ ABELLAN; M. Sandonís; P. Zaballos; S. Cuesta; S. Iglesias-Caballero; S. Pozo; S. Varona; V. Camarero; Vázquez-Morón
EPI_ISL_2345143	HOSPITAL GERAL JESUS TEIXEIRA DA COSTA GUAIANASES SAO PAULO	Instituto Butantan / ESALQ-Piracicaba	Antonio Jorge Martins; Claudia Renata dos Santos Barros; David Schlesinger; Debora Botequio Moretti; Dimas Tadeu Covas; Elaine Cristina Marqueze; Elaine Vieira Santos; Evandra Strazza Rodrigues; Heidge Fukumasu; Jayme Augusto de Souza-Neto; José Salvatore Leister Patané; Luiz Alcantara; Luiz Lehmann Coutinho; Maria Carolina Elias; Maurício Lacerda Nogueira; Rafael dos Santos Bezerra; Raul Machado Neto; Rejane Maria Tommasini Grotto; Ricardo Haddad; Sandra Coccuzzo Sampaio Vessoni; Simone Kashima; Svetoslav Nanev Slavov; Vincent Louis Viala
EPI_ISL_3102287, EPI_ISL_3102320	HOSPITAL MATERNIDADE SANTA RITA	Analytical Competence Molecular Epidemiology Lab/ACME, Oswaldo Cruz Foundation, Ceara (FIOCRUZ CE)	Cleber Furtado Aksenen; Fabio Miyajima; Fernando Braga Stehling; Francisco Eder de Moura Lopes; Jamille Maria Mendes Bezerra; Joaquim César do Nascimento Sousa Junior; Pedro Miguel Carneiro Jeronimo; Suzana Porto Almeida e Lucas Delerino; Thais Ferreira de Oliveira; Thais de Oliveira Costa; Ticiane Cavalcante de Souza; Veridiana Pessoa Miyajima
EPI_ISL_2493687, EPI_ISL_2493689	HOSPITAL MUNICIPAL DE IBIUNA IBIUNA SP	Instituto Butantan	Antonio Jorge Martins; Claudia Renata dos Santos Barros; David Schlesinger; Debora Botequio Moretti; Dimas Tadeu Covas; Elaine Cristina Marqueze; Elaine Vieira Santos; Evandra Strazza Rodrigues; Heidge Fukumasu; Jayme Augusto de Souza-Neto; José Salvatore Leister Patané; Luiz Alcantara; Luiz Lehmann Coutinho; Maria Carolina Elias; Maurício Lacerda Nogueira; Rafael dos Santos Bezerra; Raul Machado Neto; Rejane Maria Tommasini Grotto; Ricardo Haddad; Sandra Coccuzzo Sampaio Vessoni; Simone Kashima; Svetoslav Nanev Slavov; Vincent Louis Viala
EPI_ISL_2208890	HOSPITAL MUNICIPAL DE IBIUNA IBIUNA SP	Instituto Butantan / Mendelics	Antonio Jorge Martins; Claudia Renata dos Santos Barros; David Schlesinger; Debora Botequio Moretti; Dimas Tadeu Covas; Elaine Cristina Marqueze; Elaine Vieira Santos; Evandra Strazza Rodrigues; Heidge Fukumasu; Jayme Augusto de Souza-Neto; José Salvatore Leister Patané; Luiz Alcantara; Luiz Lehmann Coutinho; Maria Carolina Elias; Maurício Lacerda Nogueira; Rafael dos Santos Bezerra; Raul Machado Neto; Rejane Maria Tommasini Grotto; Ricardo Haddad; Sandra Coccuzzo Sampaio Vessoni; Simone Kashima; Svetoslav Nanev Slavov; Vincent Louis Viala
EPI_ISL_2493253, EPI_ISL_2493254, EPI_ISL_2493592, EPI_ISL_2493593, EPI_ISL_2493609, EPI_ISL_2493650, EPI_ISL_2493651			
see above	HOSPITAL MUNICIPAL DE ILHABELA GOV MARIO COVAS JR	Instituto Butantan	Antonio Jorge Martins; Claudia Renata dos Santos Barros; David Schlesinger; Debora Botequio Moretti; Dimas Tadeu Covas; Elaine Cristina Marqueze; Elaine Vieira Santos; Evandra Strazza Rodrigues; Heidge Fukumasu; Jayme Augusto de Souza-Neto; José Salvatore Leister Patané; Luiz Alcantara; Luiz Lehmann Coutinho; Maria Carolina Elias; Maurício Lacerda Nogueira; Rafael dos Santos Bezerra; Raul Machado Neto; Rejane Maria Tommasini Grotto; Ricardo Haddad; Sandra Coccuzzo Sampaio Vessoni; Simone Kashima; Svetoslav Nanev Slavov; Vincent Louis Viala
EPI_ISL_2493628	HOSPITAL MUNICIPAL DE ITAPIRA	Instituto Butantan	Antonio Jorge Martins; Claudia Renata dos Santos Barros; David Schlesinger; Debora Botequio Moretti; Dimas Tadeu Covas; Elaine Cristina Marqueze; Elaine Vieira Santos; Evandra Strazza Rodrigues; Heidge Fukumasu; Jayme Augusto de Souza-Neto; José Salvatore Leister Patané; Luiz Alcantara; Luiz Lehmann Coutinho; Maria Carolina Elias; Maurício Lacerda Nogueira; Rafael dos Santos Bezerra; Raul Machado Neto; Rejane Maria Tommasini Grotto; Ricardo Haddad; Sandra Coccuzzo Sampaio Vessoni; Simone Kashima; Svetoslav Nanev Slavov; Vincent Louis Viala
EPI_ISL_2209603, EPI_ISL_2345774	HOSPITAL MUNICIPAL DE ITAPIRA	Instituto Butantan / Mendelics	Antonio Jorge Martins; Claudia Renata dos Santos Barros; David Schlesinger; Debora Botequio Moretti; Dimas Tadeu Covas; Elaine Cristina Marqueze; Elaine Vieira Santos; Evandra Strazza Rodrigues; Heidge Fukumasu; Jayme Augusto de Souza-Neto; José Salvatore Leister Patané; Luiz Alcantara; Luiz Lehmann Coutinho; Maria Carolina Elias; Maurício Lacerda Nogueira; Rafael dos Santos Bezerra; Raul Machado Neto; Rejane Maria Tommasini Grotto; Ricardo Haddad; Sandra Coccuzzo Sampaio Vessoni; Simone Kashima; Svetoslav Nanev Slavov; Vincent Louis Viala
EPI_ISL_2445394, EPI_ISL_2445397, EPI_ISL_2445400, EPI_ISL_2445403, EPI_ISL_2445408, EPI_ISL_2445410, EPI_ISL_2445414, EPI_ISL_2493519, EPI_ISL_2493520, EPI_ISL_2493522			
see above	HOSPITAL MUNICIPAL DR MARIO GATTI CAMPINAS	Instituto Butantan	Antonio Jorge Martins; Claudia Renata dos Santos Barros; David Schlesinger; Debora Botequio Moretti; Dimas Tadeu Covas; Elaine Cristina Marqueze; Elaine Vieira Santos; Evandra Strazza Rodrigues; Heidge Fukumasu; Jayme Augusto de Souza-Neto; José Salvatore Leister Patané; Luiz Alcantara; Luiz Lehmann Coutinho; Maria Carolina Elias; Maurício Lacerda Nogueira; Rafael dos Santos Bezerra; Raul Machado Neto; Rejane Maria Tommasini Grotto; Ricardo Haddad; Sandra Coccuzzo Sampaio Vessoni; Simone Kashima; Svetoslav Nanev Slavov; Vincent Louis Viala
EPI_ISL_2445506, EPI_ISL_2445507, EPI_ISL_2445509, EPI_ISL_2493392, EPI_ISL_2493523, EPI_ISL_2493524	HOSPITAL MUNICIPAL DR TABAJARA RAMOS	Instituto Butantan	Antonio Jorge Martins; Claudia Renata dos Santos Barros; David Schlesinger; Debora Botequio Moretti; Dimas Tadeu Covas; Elaine Cristina Marqueze; Elaine Vieira Santos; Evandra Strazza Rodrigues; Heidge Fukumasu; Jayme Augusto de Souza-Neto; José Salvatore Leister Patané; Luiz Alcantara; Luiz Lehmann Coutinho; Maria Carolina Elias; Maurício Lacerda Nogueira; Rafael dos Santos Bezerra; Raul Machado Neto; Rejane Maria Tommasini Grotto; Ricardo Haddad; Sandra Coccuzzo Sampaio Vessoni; Simone Kashima; Svetoslav Nanev Slavov; Vincent Louis Viala
EPI_ISL_2362259	HOSPITAL MUNICIPAL DR WALDEMAR TEBALDI	Instituto Butantan / Mendelics	Antonio Jorge Martins; Claudia Renata dos Santos Barros; David Schlesinger; Debora Botequio Moretti; Dimas Tadeu Covas; Elaine Cristina Marqueze; Elaine Vieira Santos; Evandra Strazza Rodrigues; Heidge Fukumasu; Jayme Augusto de Souza-Neto; José Salvatore Leister Patané; Luiz Alcantara; Luiz Lehmann Coutinho; Maria Carolina Elias; Maurício Lacerda Nogueira; Rafael dos Santos Bezerra; Raul Machado Neto; Rejane Maria Tommasini Grotto; Ricardo Haddad; Sandra Coccuzzo Sampaio Vessoni; Simone Kashima; Svetoslav Nanev Slavov; Vincent Louis Viala
EPI_ISL_2493441	HOSPITAL MUNICIPAL JORGE CURY DE SANTA MARIA DA SERRA	Instituto Butantan	Antonio Jorge Martins; Claudia Renata dos Santos Barros; David Schlesinger; Debora Botequio Moretti; Dimas Tadeu Covas; Elaine Cristina Marqueze; Elaine Vieira Santos; Evandra Strazza Rodrigues; Heidge Fukumasu; Jayme Augusto de Souza-Neto; José Salvatore Leister Patané; Luiz Alcantara; Luiz Lehmann Coutinho; Maria Carolina Elias; Maurício Lacerda Nogueira; Rafael dos Santos Bezerra; Raul Machado Neto; Rejane Maria Tommasini Grotto; Ricardo Haddad; Sandra Coccuzzo Sampaio Vessoni; Simone Kashima; Svetoslav Nanev Slavov; Vincent Louis Viala
EPI_ISL_2493688	HOSPITAL MUNICIPAL LEONARDUS VAN MELLIS	Instituto Butantan	Antonio Jorge Martins; Claudia Renata dos Santos Barros; David Schlesinger; Debora Botequio Moretti; Dimas Tadeu Covas; Elaine Cristina Marqueze; Elaine Vieira Santos; Evandra Strazza Rodrigues; Heidge Fukumasu; Jayme Augusto de Souza-Neto; José Salvatore Leister Patané; Luiz Alcantara; Luiz Lehmann Coutinho; Maria Carolina Elias; Maurício Lacerda Nogueira; Rafael dos Santos Bezerra; Raul Machado Neto; Rejane Maria Tommasini Grotto; Ricardo Haddad; Sandra Coccuzzo Sampaio Vessoni; Simone Kashima; Svetoslav Nanev Slavov; Vincent Louis Viala
EPI_ISL_2801335, EPI_ISL_2801336, EPI_ISL_2801345, EPI_ISL_2801352, EPI_ISL_2801358, EPI_ISL_2801361, EPI_ISL_2801362, EPI_ISL_2801368, EPI_ISL_3102296, EPI_ISL_3102517			
see above	HOSPITAL MUNICIPAL NOSSA SENHORA DOS MILAGRES	Analytical Competence Molecular Epidemiology Lab/ACME, Oswaldo Cruz Foundation, Ceara (FIOCRUZ CE)	Cleber Furtado Aksenen; Cleber Furtado Aksenen e Suzana Porto Almeida; Fabio Miyajima; Fernando Braga Stehling; Francisco Eder de Moura Lopes; Jamille Maria Mendes Bezerra; Joaquim César do Nascimento Sousa Junior; Pedro Miguel Carneiro Jeronimo; Suzana Porto Almeida e Lucas Delerino; Thais Ferreira de Oliveira; Thais de Oliveira Costa; Ticiane Cavalcante de Souza; Veridiana Pessoa Miyajima
EPI_ISL_2445230	HOSPITAL MUNICIPAL REYNALDO GUERRA CAJATI	Instituto Butantan	Antonio Jorge Martins; Claudia Renata dos Santos Barros; David Schlesinger; Debora Botequio Moretti; Dimas Tadeu Covas; Elaine Cristina Marqueze; Elaine Vieira Santos; Evandra Strazza Rodrigues; Heidge Fukumasu; Jayme Augusto de Souza-Neto; José Salvatore Leister Patané; Luiz Alcantara; Luiz Lehmann Coutinho; Maria Carolina Elias; Maurício Lacerda Nogueira; Rafael dos Santos Bezerra; Raul Machado Neto; Rejane Maria Tommasini Grotto; Ricardo Haddad; Sandra Coccuzzo Sampaio Vessoni; Simone Kashima; Svetoslav Nanev Slavov; Vincent Louis Viala
EPI_ISL_2344960, EPI_ISL_2344969	HOSPITAL MUNICIPAL REYNALDO GUERRA CAJATI	Instituto Butantan / ESALQ-Piracicaba	Antonio Jorge Martins; Claudia Renata dos Santos Barros; David Schlesinger; Debora Botequio Moretti; Dimas Tadeu Covas; Elaine Cristina Marqueze; Elaine Vieira Santos; Evandra Strazza Rodrigues; Heidge Fukumasu; Jayme Augusto de Souza-Neto; José Salvatore Leister Patané; Luiz Alcantara; Luiz Lehmann Coutinho; Maria Carolina Elias; Maurício Lacerda Nogueira; Rafael dos Santos Bezerra; Raul Machado Neto; Rejane Maria Tommasini Grotto; Ricardo Haddad; Sandra Coccuzzo Sampaio Vessoni; Simone Kashima; Svetoslav Nanev Slavov; Vincent Louis Viala
EPI_ISL_2493296	HOSPITAL MUNICIPAL SANTO ANTONIO	Instituto Butantan	Antonio Jorge Martins; Claudia Renata dos Santos Barros; David Schlesinger; Debora Botequio Moretti; Dimas Tadeu Covas; Elaine Cristina Marqueze; Elaine Vieira Santos; Evandra Strazza Rodrigues; Heidge Fukumasu; Jayme Augusto de Souza-Neto; José Salvatore Leister Patané; Luiz Alcantara; Luiz Lehmann Coutinho; Maria Carolina Elias; Maurício Lacerda Nogueira; Rafael dos Santos Bezerra; Raul Machado Neto; Rejane Maria Tommasini Grotto; Ricardo Haddad; Sandra Coccuzzo Sampaio Vessoni; Simone Kashima; Svetoslav Nanev Slavov; Vincent Louis Viala
EPI_ISL_2345130	HOSPITAL NOSSA SENHORA APARECIDA	Instituto Butantan / ESALQ-Piracicaba	Antonio Jorge Martins; Claudia Renata dos Santos Barros; David Schlesinger; Debora Botequio Moretti; Dimas Tadeu Covas; Elaine Cristina Marqueze; Elaine Vieira Santos; Evandra Strazza Rodrigues; Heidge Fukumasu; Jayme Augusto de Souza-Neto; José Salvatore Leister Patané; Luiz Alcantara; Luiz Lehmann Coutinho; Maria Carolina Elias; Maurício Lacerda Nogueira; Rafael dos Santos Bezerra; Raul Machado Neto; Rejane Maria Tommasini Grotto; Ricardo Haddad; Sandra Coccuzzo Sampaio Vessoni; Simone Kashima; Svetoslav Nanev Slavov; Vincent Louis Viala
EPI_ISL_2801367, EPI_ISL_3102319, EPI_ISL_3102513	HOSPITAL OTOCLINICA	Analytical Competence Molecular Epidemiology Lab/ACME, Oswaldo Cruz Foundation, Ceara (FIOCRUZ CE)	Cleber Furtado Aksenen; Cleber Furtado Aksenen e Suzana Porto Almeida; Fabio Miyajima; Fernando Braga Stehling; Francisco Eder de Moura Lopes; Jamille Maria Mendes Bezerra; Joaquim César do Nascimento Sousa Junior; Pedro Miguel Carneiro Jeronimo; Suzana Porto Almeida e Lucas Delerino; Thais Ferreira de Oliveira; Thais de Oliveira Costa; Ticiane Cavalcante de Souza; Veridiana Pessoa Miyajima
EPI_ISL_2445550	HOSPITAL REGIONAL DE ITAPETININGA	Instituto Butantan	Antonio Jorge Martins; Claudia Renata dos Santos Barros; David Schlesinger; Debora Botequio Moretti; Dimas Tadeu Covas; Elaine Cristina Marqueze; Elaine Vieira Santos; Evandra Strazza Rodrigues; Heidge Fukumasu; Jayme Augusto de Souza-Neto; José Salvatore Leister Patané; Luiz Alcantara; Luiz Lehmann Coutinho; Maria Carolina Elias; Maurício Lacerda Nogueira; Rafael dos Santos Bezerra; Raul Machado Neto; Rejane Maria Tommasini Grotto; Ricardo Haddad; Sandra Coccuzzo Sampaio Vessoni; Simone Kashima; Svetoslav Nanev Slavov; Vincent Louis Viala
EPI_ISL_2801334	HOSPITAL REGIONAL DO CARIRI	Analytical Competence Molecular Epidemiology	Cleber Furtado Aksenen e Suzana Porto Almeida; Fabio Miyajima; Fernando Braga Stehling; Francisco Eder de Moura Lopes; Jamille Maria Mendes Bezerra; Joaquim César do Nascimento Sousa Junior; Pedro Miguel Carneiro Jeronimo; Thais Ferreira de Oliveira; Thais de Oliveira Costa; Ticiane Cavalcante de Souza; Veridiana Pessoa Miyajima

		Lab/ACME, Oswaldo Cruz Foundation, Ceara (FIOCRUZ CE)	
EPI_ISL_3102267	HOSPITAL REGIONAL NORTE	Analytical Competence Molecular Epidemiology Lab/ACME, Oswaldo Cruz Foundation, Ceara (FIOCRUZ CE)	Cleber Furtado Aksenen; Fabio Miyajima; Fernando Braga Stehling; Francisco Eder de Moura Lopes; Jamille Maria Mendes Bezerra; Joaquim César do Nascimento Sousa Junior; Pedro Miguel Carneiro Jeronimo; Suzana Porto Almeida e Lucas Delerino; Thais Ferreira de Oliveira; Thais de Oliveira Costa; Ticiane Cavalcante de Souza; Veridiana Pessoa Miyajima
EPI_ISL_2658278	HOSPITAL SAN FRANCISCO DE ASIS	Inciensa, Instituto Costarricense de Investigación y Enseñanza en Nutrición y Salud	Adriana Godínez; Claudio Soto-Garita; Estela Cordero; Francisco Duarte; Hebleen Porras; Joselyn Prado & Juan Carlos Villalobos-Ugalde; José Luis Vargas; Mariela Gutiérrez; Melany Calderón
EPI_ISL_2502760, EPI_ISL_2502762	HOSPITAL SAN JUAN DE DIOS	Inciensa, Instituto Costarricense de InvestigaciOn y Enseñanza en NutriciOn y Salud	Adriana Godínez; Claudio Soto-Garita; Estela Cordero; Francisco Duarte; Hebleen Porras; Jose Luis Vargas; Joselyn Prado & Marco Cháves-Otárola; Mariela Gutierrez; Melany CalderOn
EPI_ISL_2502719	HOSPITAL SAN VICENTE DE PAUL	Inciensa, Instituto Costarricense de InvestigaciOn y Enseñanza en NutriciOn y Salud	Adriana Godínez; Claudio Soto-Garita; Estela Cordero; Francisco Duarte; Hebleen Porras; Jose Luis Vargas; Joselyn Prado & Silvia Saenz-Garcia; Mariela Gutierrez; Melany CalderOn
EPI_ISL_2445220	HOSPITAL SANTO ANTONIO DE JUQUIA JUQUIA	Instituto Butantan	Antonio Jorge Martins; Claudia Renata dos Santos Barros; David Schlesinger; Debora Botequio Moretti; Dimas Tadeu Covas; Elaine Cristina Marqueze; Elaine Vieira Santos; Evandra Strazza Rodrigues; Heidge Fukumasu; Jayme Augusto de Souza-Neto; José Salvatore Leister Patané; Luiz Alcantara; Luiz Lehmann Coutinho; Maria Carolina Elias; Maurício Lacerda Nogueira; Rafael dos Santos Bezerra; Raul Machado Neto; Rejane Maria Tommasini Grotto; Ricardo Haddad; Sandra Coccuzzo Sampaio Vessoni; Simone Kashima; Svetoslav Nanev Slavov; Vincent Louis Viala
EPI_ISL_2445242	HOSPITAL SAO FRANCISCO SC LTDA	Instituto Butantan	Antonio Jorge Martins; Claudia Renata dos Santos Barros; David Schlesinger; Debora Botequio Moretti; Dimas Tadeu Covas; Elaine Cristina Marqueze; Elaine Vieira Santos; Evandra Strazza Rodrigues; Heidge Fukumasu; Jayme Augusto de Souza-Neto; José Salvatore Leister Patané; Luiz Alcantara; Luiz Lehmann Coutinho; Maria Carolina Elias; Maurício Lacerda Nogueira; Rafael dos Santos Bezerra; Raul Machado Neto; Rejane Maria Tommasini Grotto; Ricardo Haddad; Sandra Coccuzzo Sampaio Vessoni; Simone Kashima; Svetoslav Nanev Slavov; Vincent Louis Viala
EPI_ISL_2801369	HOSPITAL SAO MATEUS	Analytical Competence Molecular Epidemiology Lab/ACME, Oswaldo Cruz Foundation, Ceara (FIOCRUZ CE)	Cleber Furtado Aksenen e Suzana Porto Almeida; Fabio Miyajima; Fernando Braga Stehling; Francisco Eder de Moura Lopes; Jamille Maria Mendes Bezerra; Joaquim César do Nascimento Sousa Junior; Pedro Miguel Carneiro Jeronimo; Thais Ferreira de Oliveira; Thais de Oliveira Costa; Ticiane Cavalcante de Souza; Veridiana Pessoa Miyajima
EPI_ISL_3502489, EPI_ISL_3502727, EPI_ISL_3502728, EPI_ISL_3502729, EPI_ISL_3502741, EPI_ISL_3502744, EPI_ISL_3502746, EPI_ISL_3502750, EPI_ISL_3502754, EPI_ISL_3502760, EPI_ISL_3532472			
see above	HOSPITAL UNIVERSITARIO 12 DE OCTUBRE	HOSPITAL UNIVERSITARIO 12 DE OCTUBRE	Carmen Martín-Higuera; Esther Viedma; Irene Múñoz-Gallego; M.ª Dolores Folgueira; Mar Aguilera; Noelia Moral; Rafael Delgado; Sagrario Zurita
EPI_ISL_2100300, EPI_ISL_2100328, EPI_ISL_2100340, EPI_ISL_2100342, EPI_ISL_2134884, EPI_ISL_2134934, EPI_ISL_2241439, EPI_ISL_2241449, EPI_ISL_2241457			
see above	HOSPITAL UNIVERSITARIO SON ESPASES	HOSPITAL UNIVERSITARIO SON ESPASES	Antonio Oliver; Carla López-Causapé; Pablo Fraile-Ribot; SeqCovid
EPI_ISL_2241452	HOSPITAL UNIVERSITARIO SON LLATZER	HOSPITAL UNIVERSITARIO SON ESPASES	Antonio Oliver; Carla López-Causapé; Pablo Fraile-Ribot; SeqCovid
EPI_ISL_2738508, EPI_ISL_2738515, EPI_ISL_2738517, EPI_ISL_3235352, EPI_ISL_3235354, EPI_ISL_3235356, EPI_ISL_3235358			
see above	Hackensack Medical Center	New York Genome Center	Amy Baldwin; Andre Corvelo; Barry Kreiswirth; David Perlin; Dayna M. Oschwald; Jose Mediavilla; Kaelea Composto; Kar Chow; Liang Chen; Marcus Cunningham; Michael Zody; Samantha Fennessey; Tom Maniatis
EPI_ISL_2006656	Health Services Laboratories	Wellcome Sanger Institute for the COVID-19 Genomics UK (COG-UK) Consortium	Cordelia Langford; David K. Jackson; Dominic Kwiatkowski; Ewan Harrison; Health Services Laboratories and Alex Alderton; Ian Johnston; Jeffrey Barrett; John Sillitoe on behalf of the Wellcome Sanger Institute COVID-19 Surveillance Team; Roberto Amato; Sonia Goncalves
EPI_ISL_2727870	HealthPartners Central Lab	Minnesota Department of Health, Public Health Laboratory	Alexandra Lorentz; Jacob Garfin; Matt Plumb; and Xiong Wang
EPI_ISL_1990624, EPI_ISL_1990644, EPI_ISL_1990668, EPI_ISL_1990737, EPI_ISL_1990757, EPI_ISL_1990804, EPI_ISL_1990814, EPI_ISL_1990827, EPI_ISL_1991374, EPI_ISL_1991493, EPI_ISL_1991524, EPI_ISL_1991580, EPI_ISL_1991601, EPI_ISL_1991667, EPI_ISL_1991677, EPI_ISL_2009737, EPI_ISL_2009745, EPI_ISL_2009777, EPI_ISL_2009807, EPI_ISL_2009875, EPI_ISL_2009880, EPI_ISL_2009889, EPI_ISL_2009898, EPI_ISL_2009900, EPI_ISL_2009921, EPI_ISL_2009932, EPI_ISL_2009951, EPI_ISL_2010001, EPI_ISL_2010013, EPI_ISL_2010031, EPI_ISL_2010037, EPI_ISL_2010084, EPI_ISL_2010096, EPI_ISL_2010122, EPI_ISL_2010132, EPI_ISL_2010149, EPI_ISL_2010185, EPI_ISL_2010274, EPI_ISL_2010282, EPI_ISL_2010312, EPI_ISL_2010351, EPI_ISL_2010395, EPI_ISL_2010400, EPI_ISL_2010420, EPI_ISL_2010422, EPI_ISL_2010428, EPI_ISL_2010442, EPI_ISL_2010462, EPI_ISL_2010485, EPI_ISL_2010515, EPI_ISL_2010521, EPI_ISL_2010534, EPI_ISL_2010580, EPI_ISL_2010622, EPI_ISL_2010671, EPI_ISL_2010741, EPI_ISL_2010749, EPI_ISL_2010807, EPI_ISL_2035280, EPI_ISL_2097347, EPI_ISL_2097388, EPI_ISL_2097390, EPI_ISL_2097397, EPI_ISL_2097399, EPI_ISL_2097425, EPI_ISL_2097433, EPI_ISL_2097435, EPI_ISL_2097448, EPI_ISL_2097462, EPI_ISL_2097463, EPI_ISL_2097466, EPI_ISL_2097467, EPI_ISL_2097498, EPI_ISL_2097573, EPI_ISL_2097593, EPI_ISL_2097594, EPI_ISL_2097635, EPI_ISL_2097639, EPI_ISL_2097654, EPI_ISL_2097659, EPI_ISL_2097668, EPI_ISL_2097747, EPI_ISL_2097753, EPI_ISL_2097762, EPI_ISL_2097850, EPI_ISL_2097857, EPI_ISL_2097862, EPI_ISL_2097874, EPI_ISL_2097895, EPI_ISL_2097898, EPI_ISL_2097911, EPI_ISL_2097973, EPI_ISL_2098075, EPI_ISL_2098086, EPI_ISL_2098097, EPI_ISL_2098108, EPI_ISL_2098165, EPI_ISL_2098205, EPI_ISL_2098243, EPI_ISL_2098255, EPI_ISL_2132610, EPI_ISL_2144059, EPI_ISL_2144084, EPI_ISL_2144100, EPI_ISL_2144116, EPI_ISL_2144121, EPI_ISL_2144126, EPI_ISL_2144140, EPI_ISL_2144194, EPI_ISL_2144205, EPI_ISL_2144211, EPI_ISL_2144233, EPI_ISL_2144276, EPI_ISL_2144299, EPI_ISL_2144346, EPI_ISL_2144372, EPI_ISL_2144376, EPI_ISL_2144391, EPI_ISL_2144393, EPI_ISL_2144396, EPI_ISL_2144401, EPI_ISL_2144404, EPI_ISL_2144417, EPI_ISL_2144451, EPI_ISL_2144540, EPI_ISL_2144545, EPI_ISL_2144568, EPI_ISL_2144598, EPI_ISL_2144605, EPI_ISL_2144635, EPI_ISL_2144708, EPI_ISL_2144724, EPI_ISL_2144742, EPI_ISL_2144759, EPI_ISL_2144774, EPI_ISL_2144934, EPI_ISL_2144959, EPI_ISL_2144982, EPI_ISL_2144998, EPI_ISL_2145002, EPI_ISL_2145018, EPI_ISL_2145029, EPI_ISL_2145031, EPI_ISL_2145061, EPI_ISL_2145144, EPI_ISL_2145185, EPI_ISL_2145201, EPI_ISL_2145236, EPI_ISL_2145320, EPI_ISL_2145352, EPI_ISL_2145372, EPI_ISL_2145384, EPI_ISL_2145556, EPI_ISL_2145761, EPI_ISL_2159478, EPI_ISL_2159479, EPI_ISL_2159485, EPI_ISL_2159490, EPI_ISL_2159492, EPI_ISL_2159497, EPI_ISL_2159508, EPI_ISL_2159518, EPI_ISL_2159535, EPI_ISL_2159538, EPI_ISL_2159557, EPI_ISL_2159571, EPI_ISL_2159590, EPI_ISL_2159635, EPI_ISL_2159736, EPI_ISL_2159778, EPI_ISL_2159786, EPI_ISL_2159824, EPI_ISL_2159831, EPI_ISL_2159865, EPI_ISL_2159889, EPI_ISL_2159906, EPI_ISL_2159940, EPI_ISL_2159943, EPI_ISL_2159950, EPI_ISL_2159977, EPI_ISL_2159997, EPI_ISL_2160037, EPI_ISL_2160048, EPI_ISL_2160052, EPI_ISL_2160054, EPI_ISL_2160093, EPI_ISL_2160102, EPI_ISL_2160106, EPI_ISL_2160147, EPI_ISL_2160163, EPI_ISL_2160181, EPI_ISL_2160182, EPI_ISL_2160206, EPI_ISL_2160212, EPI_ISL_2160218, EPI_ISL_2160235, EPI_ISL_2160241, EPI_ISL_2160255, EPI_ISL_2160271, EPI_ISL_2160279, EPI_ISL_2160312, EPI_ISL_2160351, EPI_ISL_2160367, EPI_ISL_2160388, EPI_ISL_2160394, EPI_ISL_2160408, EPI_ISL_2160418, EPI_ISL_2160434, EPI_ISL_2160493, EPI_ISL_2160524, EPI_ISL_2160526, EPI_ISL_2160527, EPI_ISL_2160537, EPI_ISL_2160545, EPI_ISL_2160617, EPI_ISL_2160634, EPI_ISL_2160645, EPI_ISL_2160683, EPI_ISL_2160722, EPI_ISL_2187320, EPI_ISL_2187339, EPI_ISL_2187623, EPI_ISL_2187632, EPI_ISL_2187633, EPI_ISL_2187638, EPI_ISL_2247374, EPI_ISL_2247512, EPI_ISL_2247521, EPI_ISL_2247539, EPI_ISL_2247561, EPI_ISL_2247588, EPI_ISL_2247628, EPI_ISL_2247631, EPI_ISL_2247657, EPI_ISL_2247692, EPI_ISL_2247714, EPI_ISL_2247715, EPI_ISL_2247807, EPI_ISL_2247820, EPI_ISL_2247834, EPI_ISL_2247850, EPI_ISL_2247864, EPI_ISL_2247872, EPI_ISL_2247874, EPI_ISL_2247941, EPI_ISL_2248015, EPI_ISL_2248057, EPI_ISL_2248069, EPI_ISL_2248070, EPI_ISL_2248072, EPI_ISL_2248089, EPI_ISL_2248148, EPI_ISL_2248151, EPI_ISL_2248156, EPI_ISL_2248232, EPI_ISL_2248233, EPI_ISL_2248293, EPI_ISL_2248335, EPI_ISL_2248355, EPI_ISL_2248368, EPI_ISL_2248384, EPI_ISL_2248409, EPI_ISL_2248420, EPI_ISL_2248432, EPI_ISL_2248434, EPI_ISL_2268352, EPI_ISL_2269627, EPI_ISL_2269635, EPI_ISL_2269637, EPI_ISL_2269649, EPI_ISL_2269669, EPI_ISL_2269670, EPI_ISL_2269709, EPI_ISL_2269750, EPI_ISL_2269768, EPI_ISL_2269773, EPI_ISL_2269813, EPI_ISL_2269839, EPI_ISL_2269844, EPI_ISL_2269873, EPI_ISL_2269888, EPI_ISL_2269947, EPI_ISL_2269976, EPI_ISL_2269982, EPI_ISL_2269986, EPI_ISL_2269988, EPI_ISL_2270014, EPI_ISL_2270047, EPI_ISL_2270105, EPI_ISL_2270111, EPI_ISL_2270172, EPI_ISL_2270199, EPI_ISL_2270203, EPI_ISL_2270224, EPI_ISL_2270231, EPI_ISL_2270241, EPI_ISL_2270257, EPI_ISL_2270302, EPI_ISL_2270309, EPI_ISL_2270315, EPI_ISL_2270316, EPI_ISL_2270317, EPI_ISL_2270333, EPI_ISL_2270360, EPI_ISL_2270387, EPI_ISL_2270388, EPI_ISL_2270412, EPI_ISL_2270445, EPI_ISL_2270456, EPI_ISL_2270461, EPI_ISL_2270474, EPI_ISL_2270479, EPI_ISL_2270499, EPI_ISL_2270508, EPI_ISL_2270519, EPI_ISL_2270529, EPI_ISL_2270534, EPI_ISL_2270536, EPI_ISL_2270584, EPI_ISL_2270595, EPI_ISL_2270597, EPI_ISL_2270598, EPI_ISL_2270603, EPI_ISL_2270628, EPI_ISL_2270655, EPI_ISL_2270692, EPI_ISL_2270703, EPI_ISL_2270706, EPI_ISL_2270714, EPI_ISL_2270719, EPI_ISL_2270739, EPI_ISL_2270761, EPI_ISL_2270779, EPI_ISL_2270783, EPI_ISL_2270792, EPI_ISL_2270800, EPI_ISL_2270830, EPI_ISL_2270833, EPI_ISL_2280178, EPI_ISL_2280209, EPI_ISL_2280219, EPI_ISL_2319996, EPI_ISL_2320016, EPI_ISL_2320018, EPI_ISL_2320033, EPI_ISL_2320046, EPI_ISL_2320054, EPI_ISL_2320100, EPI_ISL_2320126, EPI_ISL_2320167, EPI_ISL_2320174, EPI_ISL_2320199, EPI_ISL_2320218, EPI_ISL_2320223, EPI_ISL_2320274, EPI_ISL_2320278, EPI_ISL_2320303, EPI_ISL_2320330, EPI_ISL_2320378, EPI_ISL_2320387, EPI_ISL_2320394, EPI_ISL_2320427, EPI_ISL_2320454, EPI_ISL_2320455, EPI_ISL_2320479, EPI_ISL_2320506, EPI_ISL_2320513, EPI_ISL_2320540, EPI_ISL_2320541, EPI_ISL_2320548, EPI_ISL_2320583, EPI_ISL_2320584, EPI_ISL_2320595, EPI_ISL_2320600, EPI_ISL_2320687, EPI_ISL_2320691, EPI_ISL_2320694, EPI_ISL_2320702, EPI_ISL_2320709, EPI_ISL_2320722, EPI_ISL_2320731, EPI_ISL_2320743, EPI_ISL_2320752, EPI_ISL_2320772, EPI_ISL_2320784, EPI_ISL_2320792, EPI_ISL_2320799, EPI_ISL_2320851, EPI_ISL_2320857, EPI_ISL_2320862, EPI_ISL_2320877, EPI_ISL_2320882, EPI_ISL_2320905, EPI_ISL_2320929, EPI_ISL_2320939, EPI_ISL_2320958, EPI_ISL_2320974, EPI_ISL_2320993, EPI_ISL_2321015, EPI_ISL_2321016, EPI_ISL_2321033, EPI_ISL_2321062, EPI_ISL_2321082, EPI_ISL_2321110, EPI_ISL_2321114, EPI_ISL_2321128, EPI_ISL_2367609, EPI_ISL_2367611, EPI_ISL_2367614, EPI_ISL_2367617, EPI_ISL_2367638, EPI_ISL_2440073, EPI_ISL_2440075, EPI_ISL_2440091, EPI_ISL_2440106, EPI_ISL_2440132, EPI_ISL_2440167, EPI_ISL_2440176, EPI_ISL_2440187, EPI_ISL_2440195, EPI_ISL_2440203, EPI_ISL_2440227, EPI_ISL_2440236, EPI_ISL_2440242, EPI_ISL_2440245, EPI_ISL_2440265, EPI_ISL_2440284, EPI_ISL_2440309, EPI_ISL_2440344, EPI_ISL_2440350, EPI_ISL_2440352, EPI_ISL_2440358, EPI_ISL_2440367, EPI_ISL_2440382, EPI_ISL_2440384, EPI_ISL_2440395, EPI_ISL_2440399, EPI_ISL_2440406, EPI_ISL_2440412, EPI_ISL_2440429, EPI_ISL_2440461, EPI_ISL_2440466, EPI_ISL_2440467, EPI_ISL_2440483, EPI_ISL_2440516, EPI_ISL_2440523, EPI_ISL_2440526, EPI_ISL_2451495, EPI_ISL_2451500, EPI_ISL_2598855, EPI_ISL_2598856, EPI_ISL_2598860, EPI_ISL_2598880, EPI_ISL_2598886, EPI_ISL_2598907, EPI_ISL_2598912, EPI_ISL_2598914, EPI_ISL_2598936, EPI_ISL_2598960, EPI_ISL_2598964, EPI_ISL_2598968, EPI_ISL_2598975, EPI_ISL_2598977, EPI_ISL_2598988, EPI_ISL_2598997, EPI_ISL_2599002, EPI_ISL_2599019, EPI_ISL_2599023, EPI_ISL_2599035, EPI_ISL_2599039, EPI_ISL_2599057, EPI_ISL_2599060, EPI_ISL_2599077, EPI_ISL_2599098, EPI_ISL_2599100, EPI_ISL_2599120, EPI_ISL_2599123, EPI_ISL_2599239, EPI_ISL_2599240, EPI_ISL_2599260, EPI_ISL_2599280, EPI_ISL_2599289, EPI_ISL_2599298, EPI_ISL_2599304, EPI_ISL_2599320, EPI_ISL_2599342, EPI_ISL_2599345, EPI_ISL_2599347, EPI_ISL_2599402, EPI_ISL_2599416, EPI_ISL_2599417, EPI_ISL_2599431, EPI_ISL_2599441, EPI_ISL_2599450, EPI_ISL_2599452, EPI_ISL_2599496, EPI_ISL_2599516, EPI_ISL_2599538, EPI_ISL_2599549, EPI_ISL_2599563, EPI_ISL_2599611, EPI_ISL_2599623, EPI_ISL_2649916, EPI_ISL_2649917, EPI_ISL_2649926, EPI_ISL_2649936			
see above	Helix/Illumina	Centers for Disease Control and Prevention Division of Viral Diseases, Pathogen Discovery	Adrian Paskey; Alexandre Bolze; Ary Ascencio; Benjamin Rambo-Martin; Brad Sickler; Charlotte Rivera-Garcia; Christine Tran; Christopher Gulvick; Clinton R. Paden; Dakota Howard; Darlene Wagner; David Becker; Dhwani Batra; Duncan MacCannell; Efren Sandoval; Eileen de Feo; Elizabeth Cirulli; Eric Allen; Geraint Levan; James Lu; Jan Antico; Jason Caravas; Jason Nguyen; Jimmy Ramirez; Jingtao Liu; Kara Moser; Kelly Schiabor Barrett; Kim Gietzen; Magnus Isaksson; Marc Laurent; Matthew Schmerer; Matthew Tolentino; Nicole L. Washington; Peter W. Cook; Phil Febbo; Ryan Cho; Scott Sammons; Shannon Wickline; Shatavia Morrison; Sherry Wang; Simon White; Tyler Cassens; William Lee; Yvette Unoarumhi
EPI_ISL_2029808, EPI_ISL_2029930, EPI_ISL_2030070, EPI_ISL_2188427, EPI_ISL_2279645, EPI_ISL_2374380, EPI_ISL_2462941			
see above	Hospital	National Reference Center for Viruses of Respiratory Infections, Institut Pasteur, Paris	Alain Delepine; Amaury Vaysse; Angela Brisebarre; Axelle Paquin; Camille Capel; Christophe Malabat; Corinne Maufrais; Emmanuelle Permal; Eric Farfour; Etienne Simon-Lorière; Frédéric Lemoine; Louise Lefrançois; Marion Barbet; Maud Vanpeene; Méline Bizard; Nabil Gastli; Pascale Martres; StéPhanie Marque Juillet; Sylvie Behillil; Sylvie Van der Werf; Vincent Enouf
EPI_ISL_2812290	Hospital Cangas y Severo Ochoa	Laboratorio de Virología HUCA	Abreu F; Alvarez-Arguelles ME; Boga JA; Castelló C; Costales I; Coto E; Gómez de Oña J; Martín-Rodríguez G; Melón S; Perez-Martínez Z; Rojo S; Sandoval M
EPI_ISL_2812439	Hospital Carmen y Severo Ochoa	Laboratorio de Virología HUCA	Abreu F; Alvarez-Arguelles ME; Boga JA; Castelló C; Costales I; Coto E; Gómez de Oña J; Martín-Rodríguez G; Melón S; Perez-Martínez Z; Rojo S; Sandoval M
EPI_ISL_2439984	Hospital Francesc de Borja (Gandia)	SeqCOVID-SPAIN consortium/IBV(CSIC)	Inmaculada Vidal Catalá; Isabel García Nava and SeqCOVID-SPAIN consortium; Mª Rosa Navarro Cots; Nieves Orta Mira
EPI_ISL_1914327, EPI_ISL_2080923, EPI_ISL_2178828, EPI_ISL_2304183, EPI_ISL_2304185, EPI_ISL_2304186, EPI_ISL_3505909, EPI_ISL_3505911, EPI_ISL_3505912, EPI_ISL_3505913, EPI_ISL_3505915, EPI_ISL_3505917, EPI_ISL_3505921, EPI_ISL_3505923, EPI_ISL_3505927, EPI_ISL_3505932, EPI_ISL_3505935, EPI_ISL_3505940, EPI_ISL_3505943, EPI_ISL_3505948, EPI_ISL_3505949, EPI_ISL_3505957, EPI_ISL_3505961, EPI_ISL_3505963, EPI_ISL_3505967			
see above	Hospital General Universitario Gregorio Marañón	Hospital General Universitario Gregorio Marañón	Cristina Rodriguez-Grande; Darío García de Viedma; Julia Suárez; Laura Pérez-Lago; Marta Herranz Martin; Patricia Muñoz; Pedro Sola Campoy; Pilar Catalán; Sergio Buenestado Serrano; Victor Manuel de la Cueva; Victor de la Cueva
EPI_ISL_2271588	Hospital General Universitario de Alicante - Instituto de Investigación Sanitaria y Biomédica de Alicante	SeqCOVID-SPAIN consortium/IBV(CSIC)	Carmen Molina Pardines and SeqCOVID-SPAIN consortium; Maripaz Ventero Martín
EPI_ISL_2614581	Hospital Geral do Grajau	Instituto Adolfo Lutz, Interdiciplinary Procedures Center, Strategic Laboratory	Caio Vinicius Dias Lopes; Claudia Regina Gonçalves; Claudio Tavares Sacchi; Erica Valessa Ramos Gomes; Karoline Rodrigues Campos; Leonardo Jose Tadeu de Araujo
EPI_ISL_3033046, EPI_ISL_3033047, EPI_ISL_3033048, EPI_ISL_3033050, EPI_ISL_3033051, EPI_ISL_3260091	Hospital Jaime Ferre - SAMCO Rafaela	Grupo de Genómica y Bioinformática del Instituto de Investigación de la Cadena Láctea CONICET-INTA on behalf of 'Proyecto Argentino Interinstitucional de genomica de SARS-CoV-2' (PAIS Consortium)	AF; Aliprandi D; Amadio; C; Eberhardt; Irazoqui; Isaia; JF; JM; MF; Pandolfi; Quaranta; Soratti R; V
EPI_ISL_2365419, EPI_ISL_2662822	Hospital San Agustín	Laboratorio de Virología HUCA	Abreu F; Alvarez-Arguelles ME; Boga JA; Castelló C; Costales I; Coto E; Gómez de Oña J; Martín-Rodríguez G; Melón S; Perez-Martínez Z; Rojo S; Sandoval M
EPI_ISL_3230021	Hospital Señor del Milagro	Instituto de Patologia Vegetal (CIAP-INTA) on behalf of 'Proyecto Argentino Interinstitucional de genomica de SARS-CoV-2' (PAIS	Debat, HJ.; FD; Fernanda Aguero; Fernández; Lavaque Esteban; Marquez, N.; Pamela Cajal; Raskovsky Viviana; Veronica Lesser

		Consortium)	
EPI_ISL_2284955, EPI_ISL_2284956, EPI_ISL_2284957, EPI_ISL_2284958, EPI_ISL_2284968, EPI_ISL_2284969, EPI_ISL_2284974, EPI_ISL_2284975, EPI_ISL_2391949, EPI_ISL_2391950			
see above	Hospital Universitari Arnau de Vilanova	Hospital Universitari Vall d'Hebron - Vall d'Hebron Institut de Recerca	Alejandra González-Sánchez; Andrés Antón; Ariadna Rando; Carla Castillo; Cristina Andrés; Damir Garcia-Cehic; Josep Quer; Juliana Esperalba; Maria Carmen Martin; Maria Gema Codina; Maria Piñana; Tomàs Pumarola
EPI_ISL_2017673, EPI_ISL_2351935, EPI_ISL_2516404, EPI_ISL_2516527	Hospital Universitari Bellvitge	Microbiology Department	Aida Gonzalez-Diaz; Carmen Ardanuy; Jordi Camara; Jordi Niubó; Laura Calatayud; M Angeles Domínguez; Miguel Fernandez-Huerta; Sara Marti
EPI_ISL_2652049, EPI_ISL_2652054	Hospital Universitari Joan XXIII	Hospital Universitari Joan XXIII	Carla Martín; Clara Benavent; Cristina Gutiérrez; Ester Picó; Gemma Recio; Margarida Terrón; Natalia Bastón
EPI_ISL_1908893, EPI_ISL_2047850, EPI_ISL_2284977, EPI_ISL_2391939	Hospital Universitari Joan XXIII de Tarragona	Hospital Universitari Vall d'Hebron - Vall d'Hebron Institut de Recerca	Alejandra González-Sánchez; Andrés Antón; Ariadna Rando; Carla Castillo; Cristina Andrés; Damir Garcia-Cehic; Josep Quer; Juliana Esperalba; Maria Carmen Martin; Maria Gema Codina; Maria Piñana; Tomàs Pumarola
EPI_ISL_2047711, EPI_ISL_2047712, EPI_ISL_2047721, EPI_ISL_2162247, EPI_ISL_2162253, EPI_ISL_2200155	Hospital Universitari Vall d'Hebron - Vall d'Hebron Institut de Recerca	Hospital Universitari Vall d'Hebron - Vall d'Hebron Institut de Recerca	Alejandra González-Sánchez; Andrés Antón; Ariadna Rando; Carla Castillo; Cristina Andrés; Damir Garcia-Cehic; Josep Quer; Juliana Esperalba; Maria Carmen Martin; Maria Gema Codina; Maria Piñana; Tomàs Pumarola
EPI_ISL_2323396	Hospital Universitari i Politècnic La Fe de València	SeqCOVID-SPAIN consortium/IBV(CSIC)	Ana Gil Brusola; Eva González Barberá; José Luis López Hontangas and SeqCOVID-SPAIN consortium; María Dolores Gómez Ruiz; Salvador Giner Almaraz
EPI_ISL_2465946	Hospital Universitario Virgen de las Nieves de Granada-SAS	SeqCOVID-SPAIN consortium/IBV(CSIC)	Irene Pedrosa Corral; José M. Navarro-Marí and SeqCOVID-SPAIN consortium; Sara Sanbonmatsu Gámez
EPI_ISL_2238703, EPI_ISL_2238704	Hospital de Cabueñes	Laboratorio de Virología HUCA	Abreu F; Alvarez-Arguelles ME; Boga JA; Castelló C; Costales I; Coto E; Gómez de Oña J; Martín-Rodríguez G; Melón S; Perez-Martínez Z; Rojo S; Sandoval M
EPI_ISL_2238720	Hospital de San Agustin	Laboratorio de Virología HUCA	Abreu F; Alvarez-Arguelles ME; Boga JA; Castelló C; Costales I; Coto E; Gómez de Oña J; Martín-Rodríguez G; Melón S; Perez-Martínez Z; Rojo S; Sandoval M
EPI_ISL_2407652	Hospital de San Agustín	Laboratorio de Virología HUCA	Abreu F; Alvarez-Arguelles ME; Boga JA; Castelló C; Costales I; Coto E; Gómez de Oña J; Martín-Rodríguez G; Melón S; Perez-Martínez Z; Rojo S; Sandoval M
EPI_ISL_3022946, EPI_ISL_3022992, EPI_ISL_3023015, EPI_ISL_3023037	Hospital of the University of Pennsylvania Molecular Pathology Lab	Bushman Lab - University of Pennsylvania	Aoife M. Roche; Arupa Ganguly; Ayannah S. Fitzgerald; Brendan Kelly; Jevon Graham-Wooten; John K. Everett; Kyle Rodino; Layla A. Khatib; Mike Feldman; Ronald G. Collman and Frederic Bushan; Samantha A. Whiteside; Scott Sherrill-Mix; Shantan Reddy; Young Hwang
EPI_ISL_1961020	Houston Health Dept.	Houston Health Dept.	Adolpho Lara; Pamela Brown; Ryker Penn; Yanlai Lai
EPI_ISL_2224488, EPI_ISL_2224693, EPI_ISL_2224718, EPI_ISL_2225125, EPI_ISL_2225228, EPI_ISL_2225308, EPI_ISL_2225349, EPI_ISL_2225382, EPI_ISL_2225387, EPI_ISL_2225419, EPI_ISL_2225425			
see above	Houston Methodist Hospital	Houston Methodist Hospital	Ilya J. Finkelstein; James J. Davis; Jessica Cambric; Jimmy Gollihar; Kristina Reppond; Layne Pruitt; Madison N. Shyer; Marcus Nguyen; Matthew Ojeda Saavedra; Paul A. Christensen; Prasanti Yerramilli; Randall J. Olsen; Robert Olson; Ryan Gadd; S. Wesley Long; Sishir Subedi; and James M. Musser
EPI_ISL_2497657, EPI_ISL_2497660, EPI_ISL_2497663, EPI_ISL_2497664, EPI_ISL_2500025, EPI_ISL_2500061, EPI_ISL_2658738			
see above	Humboldt County Public Health Laboratory	Chan-Zuckerberg Biohub	CZB Cliahub Consortium
EPI_ISL_2445617, EPI_ISL_2445620, EPI_ISL_2445630, EPI_ISL_2680474, EPI_ISL_2680483	Humboldt County Public Health Laboratory	Humboldt County Public Health Laboratory	Jeremy Corrigan
EPI_ISL_1937747	IA State Hygienic Laboratory	Centers for Disease Control and Prevention Division of Viral Diseases, Pathogen Discovery	Alison Laufer Halpin; Ben L. Rambo-Martin; Clinton R. Paden; Dakota Howard; Darlene Wagner; Dave Wentworth; Dhwani Batra; Jasmine Padilla; Justin Lee; Katie Dillon; Krista Queen; Kristen Knipe; Kristine Lacek; Mark Burroughs; Matthew Schmerer; Mili Sheth; Peter Cook; Sam Shepard; Sarah Nobles; Shoshona Le; Suxiang Tong; Vivien Dugan; Yvette Unoarumhi
EPI_ISL_2452079, EPI_ISL_2690066, EPI_ISL_2690070, EPI_ISL_2690072, EPI_ISL_2690082, EPI_ISL_2690083	ID Bureau of Laboratories	Centers for Disease Control and Prevention Division of Viral Diseases, Pathogen Discovery	Alison Laufer Halpin; Ben L. Rambo-Martin; Clinton R. Paden; Dakota Howard; Darlene Wagner; Dave Wentworth; Dhwani Batra; Jasmine Padilla; Justin Lee; Katie Dillon; Krista Queen; Kristen Knipe; Kristine Lacek; Mark Burroughs; Matthew Schmerer; Mili Sheth; Peter Cook; Sam Shepard; Sarah Nobles; Shoshona Le; Suxiang Tong; Vivien Dugan; Yvette Unoarumhi
EPI_ISL_2444813, EPI_ISL_2444815, EPI_ISL_2444819, EPI_ISL_2444820, EPI_ISL_2444821, EPI_ISL_2444822, EPI_ISL_2444823, EPI_ISL_2444824, EPI_ISL_2444827, EPI_ISL_2444828, EPI_ISL_2444829, EPI_ISL_2444830			
see above	IICS-UNA	IICS-UNA	Adriana Valenzuela; Alejandra Rojas; Chyntia Diaz; Eva Nara; Fatima Cardozo; Florencia del Puerto; Joel Ortiz; Jonas Fernandez; Laura Franco; Laura Mendoza; Leticia Rojas; Magaly Martinez; Maria Eugenia Galeano.
EPI_ISL_2229216, EPI_ISL_2229221, EPI_ISL_2229223, EPI_ISL_2229226, EPI_ISL_2229237, EPI_ISL_2229238, EPI_ISL_2229239, EPI_ISL_2229241, EPI_ISL_2248732, EPI_ISL_2451787, EPI_ISL_2451791, EPI_ISL_2451792, EPI_ISL_2451798, EPI_ISL_2451803, EPI_ISL_2451804, EPI_ISL_2451808, EPI_ISL_2451809, EPI_ISL_2451811, EPI_ISL_2463002			
see above	IL Dept. of Public Health Springfield Laboratory	Centers for Disease Control and Prevention Division of Viral Diseases, Pathogen Discovery	Alison Laufer Halpin; Ben L. Rambo-Martin; Clinton R. Paden; Dakota Howard; Darlene Wagner; Dave Wentworth; Dhwani Batra; Jasmine Padilla; Justin Lee; Katie Dillon; Krista Queen; Kristen Knipe; Kristine Lacek; Mark Burroughs; Matthew Schmerer; Mili Sheth; Peter Cook; Sam Shepard; Sarah Nobles; Shoshona Le; Suxiang Tong; Vivien Dugan; Yvette Unoarumhi
EPI_ISL_2463004, EPI_ISL_2689991	IN State Department of Health Laboratory Services	Centers for Disease Control and Prevention Division of Viral Diseases, Pathogen Discovery	Alison Laufer Halpin; Ben L. Rambo-Martin; Clinton R. Paden; Dakota Howard; Darlene Wagner; Dave Wentworth; Dhwani Batra; Jasmine Padilla; Justin Lee; Katie Dillon; Krista Queen; Kristen Knipe; Kristine Lacek; Mark Burroughs; Matthew Schmerer; Mili Sheth; Peter Cook; Sam Shepard; Sarah Nobles; Shoshona Le; Suxiang Tong; Vivien Dugan; Yvette Unoarumhi
EPI_ISL_1994879, EPI_ISL_1994906, EPI_ISL_1994908, EPI_ISL_1994909, EPI_ISL_1994911, EPI_ISL_1994915, EPI_ISL_1994920, EPI_ISL_2097176, EPI_ISL_2097192, EPI_ISL_2097206, EPI_ISL_2097213, EPI_ISL_2242547, EPI_ISL_2304496, EPI_ISL_2361369, EPI_ISL_2361371, EPI_ISL_2361378, EPI_ISL_2361384, EPI_ISL_2361389, EPI_ISL_2361413, EPI_ISL_2361434, EPI_ISL_2361449, EPI_ISL_2420155, EPI_ISL_2420158, EPI_ISL_2420163, EPI_ISL_2420170, EPI_ISL_2420208, EPI_ISL_2420213, EPI_ISL_2420216, EPI_ISL_2439149, EPI_ISL_2439150, EPI_ISL_2439166, EPI_ISL_2439178, EPI_ISL_2439181, EPI_ISL_2768054			
see above	IN State Department of Health Laboratory Services	IN State Department of Health Laboratory Services	Brian Pope; Cassandra Campion; Jamie Yeadon; Kyle Brownlee; Lixia Liu; Mark Glazier; Melissa Hindenlang
EPI_ISL_2319321, EPI_ISL_2319349, EPI_ISL_2319369, EPI_ISL_2319370, EPI_ISL_2319372, EPI_ISL_2319373	INDRE	Instituto Nacional de Medicina Genomica	Cedro-Tanda A; Garcia-Cardenas FJ; Gisela Barrera-Badillo; Gonzalez-Barrera D; Gonzalez-Woge MA; Herrera-Montalvo LA.; Hidalgo-Miranda A; Irma Lopez-Martinez; Jose Ernesto Ramirez González; Mendoza-Vargas A; Munguia-Garza P; Rangel-DeLeon D; Reyes-Grajeda JP
EPI_ISL_2628297, EPI_ISL_2628307	INHRR	Laboratorio de Virología Molecular	Carmen L Loureiro; CoViVen Group; Domingo J Garzaro; Esmeralda Vizzi; Flor H Pujol; Héctor R Rangel; José Luis Zambrano; Lieska Rodríguez; Mariana Hidalgo; Pierina D´Angelo; Rossana C Jaspe; Víctor Alarcón; Yoneira Sulbaran; Zoila Moros
EPI_ISL_2493969, EPI_ISL_2494037	INSIDE CRSN FREGUESIA/BRASILÂNDIA	Instituto Butantan	Antonio Jorge Martins; Claudia Renata dos Santos Barros; David Schlesinger; Debora Botequio Moretti; Dimas Tadeu Covas; Elaine Cristina Marqueze; Elaine Vieira Santos; Evandra Strazza Rodrigues; Heidge Fukumasu; Jayme Augusto de Souza-Neto; José Salvatore Leister Patané; Luiz Alcantara; Luiz Lehmann Coutinho; Maria Carolina Elias; Maurício Lacerda Nogueira; Rafael dos Santos Bezerra; Raul Machado Neto; Rejane Maria Tommasini Grotto; Ricardo Haddad; Sandra Coccuzzo Sampaio Vessoni; Simone Kashima; Svetoslav Nanev Slavov; Vincent Louis Viala
EPI_ISL_2493974	INSIDE CRSN PERUS	Instituto Butantan	Antonio Jorge Martins; Claudia Renata dos Santos Barros; David Schlesinger; Debora Botequio Moretti; Dimas Tadeu Covas; Elaine Cristina Marqueze; Elaine Vieira Santos; Evandra Strazza Rodrigues; Heidge Fukumasu; Jayme Augusto de Souza-Neto; José Salvatore Leister Patané; Luiz Alcantara; Luiz Lehmann Coutinho; Maria Carolina Elias; Maurício Lacerda Nogueira; Rafael dos Santos Bezerra; Raul Machado Neto; Rejane Maria Tommasini Grotto; Ricardo Haddad; Sandra Coccuzzo Sampaio Vessoni; Simone Kashima; Svetoslav Nanev Slavov; Vincent Louis Viala
EPI_ISL_2493966	INSIDE CRSN VILA MARIA/VILA GUILHERME	Instituto Butantan	Antonio Jorge Martins; Claudia Renata dos Santos Barros; David Schlesinger; Debora Botequio Moretti; Dimas Tadeu Covas; Elaine Cristina Marqueze; Elaine Vieira Santos; Evandra Strazza Rodrigues; Heidge Fukumasu; Jayme Augusto de Souza-Neto; José Salvatore Leister Patané; Luiz Alcantara; Luiz Lehmann Coutinho; Maria Carolina Elias; Maurício Lacerda Nogueira; Rafael dos Santos Bezerra; Raul Machado Neto; Rejane Maria Tommasini Grotto; Ricardo Haddad; Sandra Coccuzzo Sampaio Vessoni; Simone Kashima; Svetoslav Nanev Slavov; Vincent Louis Viala
EPI_ISL_2493991	INSIDE CRSSU MOOCA/ARICANDUVA	Instituto Butantan	Antonio Jorge Martins; Claudia Renata dos Santos Barros; David Schlesinger; Debora Botequio Moretti; Dimas Tadeu Covas; Elaine Cristina Marqueze; Elaine Vieira Santos; Evandra Strazza Rodrigues; Heidge Fukumasu; Jayme Augusto de Souza-Neto; José Salvatore Leister Patané; Luiz Alcantara; Luiz Lehmann Coutinho; Maria Carolina Elias; Maurício Lacerda Nogueira; Rafael dos Santos Bezerra; Raul Machado Neto; Rejane Maria Tommasini Grotto; Ricardo Haddad; Sandra Coccuzzo Sampaio Vessoni; Simone Kashima; Svetoslav Nanev Slavov; Vincent Louis Viala
EPI_ISL_2494013, EPI_ISL_2494021	INSIDE CRSSU SAPOPEMBA/VILA PRUDENTE	Instituto Butantan	Antonio Jorge Martins; Claudia Renata dos Santos Barros; David Schlesinger; Debora Botequio Moretti; Dimas Tadeu Covas; Elaine Cristina Marqueze; Elaine Vieira Santos; Evandra Strazza Rodrigues; Heidge Fukumasu; Jayme Augusto de Souza-Neto; José Salvatore Leister Patané; Luiz Alcantara; Luiz Lehmann Coutinho; Maria Carolina Elias; Maurício Lacerda Nogueira; Rafael dos Santos Bezerra; Raul Machado Neto; Rejane Maria Tommasini Grotto; Ricardo Haddad; Sandra Coccuzzo Sampaio Vessoni; Simone Kashima; Svetoslav Nanev Slavov; Vincent Louis Viala
EPI_ISL_2493247, EPI_ISL_2493713, EPI_ISL_2493714, EPI_ISL_2493715, EPI_ISL_2493716, EPI_ISL_2493718, EPI_ISL_2493719, EPI_ISL_2493720, EPI_ISL_2493721, EPI_ISL_2493724, EPI_ISL_2493725, EPI_ISL_2493726, EPI_ISL_2493727, EPI_ISL_2493728, EPI_ISL_2493729, EPI_ISL_2493732, EPI_ISL_2493733, EPI_ISL_2493735, EPI_ISL_2493736, EPI_ISL_2493737, EPI_ISL_2493738, EPI_ISL_2494318, EPI_ISL_2500972, EPI_ISL_2500973			
see above	INSIDE DIAGNÓSTICOS	Instituto Butantan	Antonio Jorge Martins; Claudia Renata dos Santos Barros; David Schlesinger; Debora Botequio Moretti; Dimas Tadeu Covas; Elaine Cristina Marqueze; Elaine Vieira Santos; Evandra Strazza Rodrigues; Heidge Fukumasu; Jayme Augusto de Souza-Neto; José Salvatore Leister Patané; Luiz Alcantara; Luiz Lehmann Coutinho; Maria Carolina Elias; Maurício Lacerda Nogueira; Rafael dos Santos Bezerra; Raul Machado Neto; Rejane Maria Tommasini Grotto; Ricardo Haddad; Sandra Coccuzzo Sampaio Vessoni; Simone Kashima; Svetoslav Nanev Slavov; Vincent Louis Viala
EPI_ISL_2493704, EPI_ISL_2493705, EPI_ISL_2493707	INSIDE DIAGNÓSTICOS NORTE BRASILÂNDIA/FREGUESIA	Instituto Butantan	Antonio Jorge Martins; Claudia Renata dos Santos Barros; David Schlesinger; Debora Botequio Moretti; Dimas Tadeu Covas; Elaine Cristina Marqueze; Elaine Vieira Santos; Evandra Strazza Rodrigues; Heidge Fukumasu; Jayme Augusto de Souza-Neto; José Salvatore Leister Patané; Luiz Alcantara; Luiz Lehmann Coutinho; Maria Carolina Elias; Maurício Lacerda Nogueira; Rafael dos Santos Bezerra; Raul Machado Neto; Rejane Maria Tommasini Grotto; Ricardo Haddad; Sandra Coccuzzo Sampaio Vessoni; Simone Kashima; Svetoslav Nanev Slavov; Vincent Louis Viala
EPI_ISL_2445050, EPI_ISL_2445081	INSIDE DIAGNÓSTICOS NORTE BRASILÂNDIA/FREGUESIA DO Ó	Instituto Butantan	Antonio Jorge Martins; Claudia Renata dos Santos Barros; David Schlesinger; Debora Botequio Moretti; Dimas Tadeu Covas; Elaine Cristina Marqueze; Elaine Vieira Santos; Evandra Strazza Rodrigues; Heidge Fukumasu; Jayme Augusto de Souza-Neto; José Salvatore Leister Patané; Luiz Alcantara; Luiz Lehmann Coutinho; Maria Carolina Elias; Maurício Lacerda Nogueira; Rafael dos Santos Bezerra; Raul Machado Neto; Rejane Maria Tommasini Grotto; Ricardo Haddad; Sandra Coccuzzo Sampaio Vessoni; Simone Kashima; Svetoslav Nanev Slavov; Vincent Louis Viala

EPI_ISL_2445059, EPI_ISL_2445065, EPI_ISL_2445070, EPI_ISL_2493706	INSIDE DIAGNÓSTICOS NORTE CACHOEIRINHA/CASA VERDE	Instituto Butantan	Antonio Jorge Martins; Claudia Renata dos Santos Barros; David Schlesinger; Debora Botequio Moretti; Dimas Tadeu Covas; Elaine Cristina Marqueze; Elaine Vieira Santos; Evandra Strazza Rodrigues; Heidge Fukumasu; Jayme Augusto de Souza-Neto; José Salvatore Leister Patané; Luiz Alcantara; Luiz Lehmann Coutinho; Maria Carolina Elias; Maurício Lacerda Nogueira; Rafael dos Santos Bezerra; Raul Machado Neto; Rejane Maria Tommasini Grotto; Ricardo Haddad; Sandra Coccuzzo Sampaio Vessoni; Simone Kashima; Svetoslav Nanev Slavov; Vincent Louis Viala
EPI_ISL_2445062, EPI_ISL_2445071	INSIDE DIAGNÓSTICOS NORTE PERUS	Instituto Butantan	Antonio Jorge Martins; Claudia Renata dos Santos Barros; David Schlesinger; Debora Botequio Moretti; Dimas Tadeu Covas; Elaine Cristina Marqueze; Elaine Vieira Santos; Evandra Strazza Rodrigues; Heidge Fukumasu; Jayme Augusto de Souza-Neto; José Salvatore Leister Patané; Luiz Alcantara; Luiz Lehmann Coutinho; Maria Carolina Elias; Maurício Lacerda Nogueira; Rafael dos Santos Bezerra; Raul Machado Neto; Rejane Maria Tommasini Grotto; Ricardo Haddad; Sandra Coccuzzo Sampaio Vessoni; Simone Kashima; Svetoslav Nanev Slavov; Vincent Louis Viala
EPI_ISL_2445058	INSIDE DIAGNÓSTICOS NORTE PIRITUBA	Instituto Butantan	Antonio Jorge Martins; Claudia Renata dos Santos Barros; David Schlesinger; Debora Botequio Moretti; Dimas Tadeu Covas; Elaine Cristina Marqueze; Elaine Vieira Santos; Evandra Strazza Rodrigues; Heidge Fukumasu; Jayme Augusto de Souza-Neto; José Salvatore Leister Patané; Luiz Alcantara; Luiz Lehmann Coutinho; Maria Carolina Elias; Maurício Lacerda Nogueira; Rafael dos Santos Bezerra; Raul Machado Neto; Rejane Maria Tommasini Grotto; Ricardo Haddad; Sandra Coccuzzo Sampaio Vessoni; Simone Kashima; Svetoslav Nanev Slavov; Vincent Louis Viala
EPI_ISL_2445047, EPI_ISL_2445060	INSIDE DIAGNÓSTICOS NORTE VILA MARIA/VILA GUILHERME	Instituto Butantan	Antonio Jorge Martins; Claudia Renata dos Santos Barros; David Schlesinger; Debora Botequio Moretti; Dimas Tadeu Covas; Elaine Cristina Marqueze; Elaine Vieira Santos; Evandra Strazza Rodrigues; Heidge Fukumasu; Jayme Augusto de Souza-Neto; José Salvatore Leister Patané; Luiz Alcantara; Luiz Lehmann Coutinho; Maria Carolina Elias; Maurício Lacerda Nogueira; Rafael dos Santos Bezerra; Raul Machado Neto; Rejane Maria Tommasini Grotto; Ricardo Haddad; Sandra Coccuzzo Sampaio Vessoni; Simone Kashima; Svetoslav Nanev Slavov; Vincent Louis Viala
EPI_ISL_2445074	INSIDE DIAGNÓSTICOS OESTE BUTANTÃ	Instituto Butantan	Antonio Jorge Martins; Claudia Renata dos Santos Barros; David Schlesinger; Debora Botequio Moretti; Dimas Tadeu Covas; Elaine Cristina Marqueze; Elaine Vieira Santos; Evandra Strazza Rodrigues; Heidge Fukumasu; Jayme Augusto de Souza-Neto; José Salvatore Leister Patané; Luiz Alcantara; Luiz Lehmann Coutinho; Maria Carolina Elias; Maurício Lacerda Nogueira; Rafael dos Santos Bezerra; Raul Machado Neto; Rejane Maria Tommasini Grotto; Ricardo Haddad; Sandra Coccuzzo Sampaio Vessoni; Simone Kashima; Svetoslav Nanev Slavov; Vincent Louis Viala
EPI_ISL_2445073	INSIDE DIAGNÓSTICOS OESTE LAPA/PINHEIROS	Instituto Butantan	Antonio Jorge Martins; Claudia Renata dos Santos Barros; David Schlesinger; Debora Botequio Moretti; Dimas Tadeu Covas; Elaine Cristina Marqueze; Elaine Vieira Santos; Evandra Strazza Rodrigues; Heidge Fukumasu; Jayme Augusto de Souza-Neto; José Salvatore Leister Patané; Luiz Alcantara; Luiz Lehmann Coutinho; Maria Carolina Elias; Maurício Lacerda Nogueira; Rafael dos Santos Bezerra; Raul Machado Neto; Rejane Maria Tommasini Grotto; Ricardo Haddad; Sandra Coccuzzo Sampaio Vessoni; Simone Kashima; Svetoslav Nanev Slavov; Vincent Louis Viala
EPI_ISL_2445067, EPI_ISL_2445072, EPI_ISL_2445076, EPI_ISL_2445084	INSIDE DIAGNÓSTICOS SUDESTE IPIRANGA	Instituto Butantan	Antonio Jorge Martins; Claudia Renata dos Santos Barros; David Schlesinger; Debora Botequio Moretti; Dimas Tadeu Covas; Elaine Cristina Marqueze; Elaine Vieira Santos; Evandra Strazza Rodrigues; Heidge Fukumasu; Jayme Augusto de Souza-Neto; José Salvatore Leister Patané; Luiz Alcantara; Luiz Lehmann Coutinho; Maria Carolina Elias; Maurício Lacerda Nogueira; Rafael dos Santos Bezerra; Raul Machado Neto; Rejane Maria Tommasini Grotto; Ricardo Haddad; Sandra Coccuzzo Sampaio Vessoni; Simone Kashima; Svetoslav Nanev Slavov; Vincent Louis Viala
EPI_ISL_2445056	INSIDE DIAGNÓSTICOS SUDESTE MOOCA/ARICANDUVA	Instituto Butantan	Antonio Jorge Martins; Claudia Renata dos Santos Barros; David Schlesinger; Debora Botequio Moretti; Dimas Tadeu Covas; Elaine Cristina Marqueze; Elaine Vieira Santos; Evandra Strazza Rodrigues; Heidge Fukumasu; Jayme Augusto de Souza-Neto; José Salvatore Leister Patané; Luiz Alcantara; Luiz Lehmann Coutinho; Maria Carolina Elias; Maurício Lacerda Nogueira; Rafael dos Santos Bezerra; Raul Machado Neto; Rejane Maria Tommasini Grotto; Ricardo Haddad; Sandra Coccuzzo Sampaio Vessoni; Simone Kashima; Svetoslav Nanev Slavov; Vincent Louis Viala
EPI_ISL_2445052, EPI_ISL_2473815	INSIDE DIAGNÓSTICOS SUDESTE VILA MARIANA/JABAQUARA	Instituto Butantan	Antonio Jorge Martins; Claudia Renata dos Santos Barros; David Schlesinger; Debora Botequio Moretti; Dimas Tadeu Covas; Elaine Cristina Marqueze; Elaine Vieira Santos; Evandra Strazza Rodrigues; Heidge Fukumasu; Jayme Augusto de Souza-Neto; José Salvatore Leister Patané; Luiz Alcantara; Luiz Lehmann Coutinho; Maria Carolina Elias; Maurício Lacerda Nogueira; Rafael dos Santos Bezerra; Raul Machado Neto; Rejane Maria Tommasini Grotto; Ricardo Haddad; Sandra Coccuzzo Sampaio Vessoni; Simone Kashima; Svetoslav Nanev Slavov; Vincent Louis Viala
EPI_ISL_2445054, EPI_ISL_2445057, EPI_ISL_2445080	INSIDE DIAGNÓSTICOS SUL CAPELA DO SOCORRO	Instituto Butantan	Antonio Jorge Martins; Claudia Renata dos Santos Barros; David Schlesinger; Debora Botequio Moretti; Dimas Tadeu Covas; Elaine Cristina Marqueze; Elaine Vieira Santos; Evandra Strazza Rodrigues; Heidge Fukumasu; Jayme Augusto de Souza-Neto; José Salvatore Leister Patané; Luiz Alcantara; Luiz Lehmann Coutinho; Maria Carolina Elias; Maurício Lacerda Nogueira; Rafael dos Santos Bezerra; Raul Machado Neto; Rejane Maria Tommasini Grotto; Ricardo Haddad; Sandra Coccuzzo Sampaio Vessoni; Simone Kashima; Svetoslav Nanev Slavov; Vincent Louis Viala
EPI_ISL_2445051, EPI_ISL_2445075	INSIDE DIAGNÓSTICOS SUL PARELHEIROS	Instituto Butantan	Antonio Jorge Martins; Claudia Renata dos Santos Barros; David Schlesinger; Debora Botequio Moretti; Dimas Tadeu Covas; Elaine Cristina Marqueze; Elaine Vieira Santos; Evandra Strazza Rodrigues; Heidge Fukumasu; Jayme Augusto de Souza-Neto; José Salvatore Leister Patané; Luiz Alcantara; Luiz Lehmann Coutinho; Maria Carolina Elias; Maurício Lacerda Nogueira; Rafael dos Santos Bezerra; Raul Machado Neto; Rejane Maria Tommasini Grotto; Ricardo Haddad; Sandra Coccuzzo Sampaio Vessoni; Simone Kashima; Svetoslav Nanev Slavov; Vincent Louis Viala
EPI_ISL_2445046	INSIDE LABORATÓRIOS NORTE PIRITUBA	Instituto Butantan	Antonio Jorge Martins; Claudia Renata dos Santos Barros; David Schlesinger; Debora Botequio Moretti; Dimas Tadeu Covas; Elaine Cristina Marqueze; Elaine Vieira Santos; Evandra Strazza Rodrigues; Heidge Fukumasu; Jayme Augusto de Souza-Neto; José Salvatore Leister Patané; Luiz Alcantara; Luiz Lehmann Coutinho; Maria Carolina Elias; Maurício Lacerda Nogueira; Rafael dos Santos Bezerra; Raul Machado Neto; Rejane Maria Tommasini Grotto; Ricardo Haddad; Sandra Coccuzzo Sampaio Vessoni; Simone Kashima; Svetoslav Nanev Slavov; Vincent Louis Viala
EPI_ISL_2458114, EPI_ISL_2458115, EPI_ISL_2488728, EPI_ISL_2757630	INSPI-CRN DE INFLUENZA Y OTROS VIRUS RESPIRATORIOS	NIC-INSPI	Alfredo Bruno; Domenica de Mora.; Jimmy Garcés; Johanna Laines; Lizbeth Patiño; Manuel Gonzalez; Maritza Olmedo; Michelle Páez
EPI_ISL_2444184	IPIGUA	Instituto Butantan / FAMERP	Antonio Jorge Martins; Claudia Renata dos Santos Barros; David Schlesinger; Debora Botequio Moretti; Dimas Tadeu Covas; Elaine Cristina Marqueze; Elaine Vieira Santos; Evandra Strazza Rodrigues; Heidge Fukumasu; Jayme Augusto de Souza-Neto; José Salvatore Leister Patané; Luiz Alcantara; Luiz Lehmann Coutinho; Maria Carolina Elias; Maurício Lacerda Nogueira; Rafael dos Santos Bezerra; Raul Machado Neto; Rejane Maria Tommasini Grotto; Ricardo Haddad; Sandra Coccuzzo Sampaio Vessoni; Simone Kashima; Svetoslav Nanev Slavov; Vincent Louis Viala
EPI_ISL_2612491, EPI_ISL_2693359, EPI_ISL_2693365, EPI_ISL_2693367, EPI_ISL_2712986	ISDH	Quantigen Biosciences	Paul J. Childress
EPI_ISL_2628306	IVIC	Laboratorio de Virología Molecular	Carmen L Loureiro; CoViVen Group; Domingo J Garzaro; Esmeralda Vizzi; Flor H Pujol; Héctor R Rangel; José Luis Zambrano; Lieska Rodríguez; Mariana Hidalgo; Pierina D´Angelo; Rossana C Jaspe; Víctor Alarcón; Yoneira Sulbaran; Zoila Moros
EPI_ISL_2427311, EPI_ISL_2500373, EPI_ISL_2500407, EPI_ISL_2500420, EPI_ISL_2598272, EPI_ISL_2598284, EPI_ISL_2789975, EPI_ISL_2789977			
see above	IVY3 Central Lab, Vanderbilt University Medical Center	Lauring Lab, University of Michigan, Department of Microbiology and Immunology	Gilbert; Valesano
EPI_ISL_2786785, EPI_ISL_2786786	Ibaraki Prefectural Institute of Public Health	Ibaraki Prefectural Institute of Public Health	Keiko Goto; Kentaro Itokawa; Makoto Kuroda; Masanori Hashino; Rina Tanaka; Tsuyoshi Sekizuka
EPI_ISL_2598130, EPI_ISL_2834650, EPI_ISL_2834656	Idaho Bureau of Laboratories	Center for Global Health, University of New Mexico Health Sciences Center	Christopher Ball; Darrell Dinwiddie; Daryl Domman; Kurt Schwalm; Matthew Burns; Robert Voermans; Valerie Morley
EPI_ISL_2295874, EPI_ISL_2295877, EPI_ISL_2295878, EPI_ISL_2295879, EPI_ISL_2295882, EPI_ISL_2295917, EPI_ISL_2801135, EPI_ISL_2801136, EPI_ISL_2801137, EPI_ISL_2801138, EPI_ISL_2801139, EPI_ISL_2801142, EPI_ISL_2960186, EPI_ISL_3670779			
see above	Illinois Department of Public Health	Gagnon Lab, Southern Illinois University	Keith Gagnon
EPI_ISL_1965031, EPI_ISL_1965071, EPI_ISL_1965077, EPI_ISL_1965081, EPI_ISL_1965085, EPI_ISL_1965090, EPI_ISL_1965100, EPI_ISL_1965116, EPI_ISL_2140025, EPI_ISL_2140026, EPI_ISL_2140027, EPI_ISL_2140028, EPI_ISL_2140030, EPI_ISL_2140031, EPI_ISL_2300283, EPI_ISL_2300286, EPI_ISL_2300328, EPI_ISL_2300353, EPI_ISL_2300387, EPI_ISL_2300396, EPI_ISL_2300414, EPI_ISL_2300519, EPI_ISL_2300525, EPI_ISL_2300531, EPI_ISL_2300536, EPI_ISL_2300582, EPI_ISL_2300631, EPI_ISL_2467401, EPI_ISL_2467406, EPI_ISL_2467414, EPI_ISL_2467450, EPI_ISL_2467452, EPI_ISL_2467453, EPI_ISL_2467455, EPI_ISL_2467459, EPI_ISL_2467461, EPI_ISL_2467467, EPI_ISL_2467472, EPI_ISL_2467474, EPI_ISL_2467483, EPI_ISL_2467486, EPI_ISL_2467488, EPI_ISL_2467502, EPI_ISL_2467508, EPI_ISL_2800858, EPI_ISL_2800860, EPI_ISL_2800866			
see above	Illinois Department of Public Health	Illinois Department of Public Health - Chicago Lab	Ira Heimler; Joel Price; Vineet K. Dhiman
EPI_ISL_1825800, EPI_ISL_1825807, EPI_ISL_1825837, EPI_ISL_1825842, EPI_ISL_1825844, EPI_ISL_1825852, EPI_ISL_1825865, EPI_ISL_1938505, EPI_ISL_1938507, EPI_ISL_1938517, EPI_ISL_1938532, EPI_ISL_1938551, EPI_ISL_1938557, EPI_ISL_1938567, EPI_ISL_1938571, EPI_ISL_1938575, EPI_ISL_1938577, EPI_ISL_1938578, EPI_ISL_1938581, EPI_ISL_1938582, EPI_ISL_1938584, EPI_ISL_1938585, EPI_ISL_1938591, EPI_ISL_1938594, EPI_ISL_1938600, EPI_ISL_1938605, EPI_ISL_1938606, EPI_ISL_1938607, EPI_ISL_1938611, EPI_ISL_1938613, EPI_ISL_1938621, EPI_ISL_1938631, EPI_ISL_1938634, EPI_ISL_1938635, EPI_ISL_1938636, EPI_ISL_1938639, EPI_ISL_1938640, EPI_ISL_1938641, EPI_ISL_1938650, EPI_ISL_1938654, EPI_ISL_1938656, EPI_ISL_1938662, EPI_ISL_1938664, EPI_ISL_1938665, EPI_ISL_2000780, EPI_ISL_2000787, EPI_ISL_2000795, EPI_ISL_2000796, EPI_ISL_2000799, EPI_ISL_2000800, EPI_ISL_2000803, EPI_ISL_2000827, EPI_ISL_2000828, EPI_ISL_2000830, EPI_ISL_2000831, EPI_ISL_2000841, EPI_ISL_2000847, EPI_ISL_2000851, EPI_ISL_2086492, EPI_ISL_2086496, EPI_ISL_2086499, EPI_ISL_2086500, EPI_ISL_2086507, EPI_ISL_2086517, EPI_ISL_2086543, EPI_ISL_2086560, EPI_ISL_2086561, EPI_ISL_2086562, EPI_ISL_2135039, EPI_ISL_2135040, EPI_ISL_2135041, EPI_ISL_2135044, EPI_ISL_2135047, EPI_ISL_2135048, EPI_ISL_2135051, EPI_ISL_2135052, EPI_ISL_2135058, EPI_ISL_2135064, EPI_ISL_2135069, EPI_ISL_2135089, EPI_ISL_2135103, EPI_ISL_2135106, EPI_ISL_2135110, EPI_ISL_2135113, EPI_ISL_2135114, EPI_ISL_2135119, EPI_ISL_2135122, EPI_ISL_2135123, EPI_ISL_2135131, EPI_ISL_2203492, EPI_ISL_2203498, EPI_ISL_2203505, EPI_ISL_2203506, EPI_ISL_2203519, EPI_ISL_2203521, EPI_ISL_2203524, EPI_ISL_2203525, EPI_ISL_2203531, EPI_ISL_2203533, EPI_ISL_2203537, EPI_ISL_2203542, EPI_ISL_2203553, EPI_ISL_2203555, EPI_ISL_2203558, EPI_ISL_2203559, EPI_ISL_2203562, EPI_ISL_2203564, EPI_ISL_2203565, EPI_ISL_2203567, EPI_ISL_2203573, EPI_ISL_2203577, EPI_ISL_2203580, EPI_ISL_2203594, EPI_ISL_2203596, EPI_ISL_2203619, EPI_ISL_2203626, EPI_ISL_2203627, EPI_ISL_2203630, EPI_ISL_2203632, EPI_ISL_2203635, EPI_ISL_2203639, EPI_ISL_2203653, EPI_ISL_2203663, EPI_ISL_2203671, EPI_ISL_2203689, EPI_ISL_2203692, EPI_ISL_2203696, EPI_ISL_2203700, EPI_ISL_2203726, EPI_ISL_2203736, EPI_ISL_2203744, EPI_ISL_2203755, EPI_ISL_2203763, EPI_ISL_2339343, EPI_ISL_2339346, EPI_ISL_2339352, EPI_ISL_2339354, EPI_ISL_2339355, EPI_ISL_2339357, EPI_ISL_2339362, EPI_ISL_2339367, EPI_ISL_2339374, EPI_ISL_2339378, EPI_ISL_2339379, EPI_ISL_2339387, EPI_ISL_2339389, EPI_ISL_2339392, EPI_ISL_2339393, EPI_ISL_2339394, EPI_ISL_2339396, EPI_ISL_2339397, EPI_ISL_2339399, EPI_ISL_2339400, EPI_ISL_2339401, EPI_ISL_2339405, EPI_ISL_2339410, EPI_ISL_2339412, EPI_ISL_2339415, EPI_ISL_2339416, EPI_ISL_2339419, EPI_ISL_2339426, EPI_ISL_2339432, EPI_ISL_2339433, EPI_ISL_2339434, EPI_ISL_2339435, EPI_ISL_2403013, EPI_ISL_2403015, EPI_ISL_2403016, EPI_ISL_2403017, EPI_ISL_2403021, EPI_ISL_2403026, EPI_ISL_2403027, EPI_ISL_2403031, EPI_ISL_2403034, EPI_ISL_2403036, EPI_ISL_2403037, EPI_ISL_2403043, EPI_ISL_2403044, EPI_ISL_2403058, EPI_ISL_2403072, EPI_ISL_2403073, EPI_ISL_2403088, EPI_ISL_2403090, EPI_ISL_2403091, EPI_ISL_2403092, EPI_ISL_2403097, EPI_ISL_2403098, EPI_ISL_2403101, EPI_ISL_2403103, EPI_ISL_2403104, EPI_ISL_2403106, EPI_ISL_2403107, EPI_ISL_2403127, EPI_ISL_2403131, EPI_ISL_2403132, EPI_ISL_2403138, EPI_ISL_2403155, EPI_ISL_2598209			
see above	Illinois Department of Public Health - Springfield Lab	Illinois Department of Public Health - Springfield Lab	Bryan Sim; Gordon McCall
EPI_ISL_2987712	Imágenes Diagnósticas de Mazatlán	Microbial Genomics Laboratory	; Alejandra García-Gasca; Alejandra Hernández-Terán; Alejandro Sánchez-Flores; Alfredo Herrera-Estrella; Alicia Ocaña-Mondragón; Andreu Comas-García; Angel Gustavo Salas-Lais; Antonio Loza Román; Bernardo Martínez-Miguel; Blanca Taboada; Brenda Irasema Maldonado-Meza; Bruno Gómez-Gil; Carla Ivón Herrera-Najera; Carlos F. Arias; Celia Boukadida; Clara Esperanza Santacruz-Tinoco; Concepción Grajales-Muñiz; Consorcio Mexicano de Vigilancia Genómica (CoViGen-Mex). Authors (in alphabetical order): Julio Elias Alvarado-Yaah; Cristóbal Cháidez-Quiróz; Célida Duque Molina; Célida Martínez-Rodríguez; Daniel Fregoso-Rueda; Daniel Lira Morales; Eduardo Becerril-Vargas; Fernando Fontove-Herrera; Fidencio Mejía-Nepomuceno; Francisco Pulido; Gloria Elena Espinosa-Ayala; Gloria María Molina-Salinas; Gloria Vazquez; Hector Esteban Paz-Juárez; Hector Montoya-Fuentes; Helen Haydee Fernanda Ramírez-Plascencia; Irvin González-López; Jean Pierre González; Jesús Hernández; Joel Armando Vázquez-Pérez.; Jorge Salas-Hernández; José Antonio Enciso-Moreno; José Arturo Martínez-Orozco; José Esteban Muñoz-Medina; José de Jesús Nuñez-Contreras; Juan Bautista Chale-Dzul; Julissa Enciso-Ibarra; Luis Alberto Ochoa-Carrera; Margarita Matías-Florentino; Mario Mújica-Sánchez; Marissa Perez-Garcia; María Guadalupe Santiago-Mauricio; María Guadalupe de Jesús Mireles-Rivera; Nelly Sélem-Mojica; Pavel Isa; Ricardo Ciria Merce; Ricardo Grande; Rosa María Gutiérrez Rios; Santiago Ávila-Ríos; Selene Zárate; Susana Lopez; Verónica Mata-Haro; Víctor Eduardo García-Arias; Víctor Hugo Borja-Aburto
EPI_ISL_2230897	InDRE	Instituto Nacional de Medicina Genomica	Cedro-Tanda A; Garcia-Cardenas FJ; Gisela Barrera-Badillo; Gonzalez-Barrera D; Gonzalez-Woge MA; Herrera-Montalvo LA.; Hidalgo-Miranda A; Irma Lopez-Martinez; Jose Ernesto Ramirez González; Mendoza-Vargas A; Munguia-Garza P; Rangel-DeLeon D; Reyes-Grajeda JP
EPI_ISL_2011564, EPI_ISL_2011567, EPI_ISL_2011623, EPI_ISL_2011633, EPI_ISL_2136745, EPI_ISL_2490282, EPI_ISL_2490285, EPI_ISL_2536388, EPI_ISL_2536390, EPI_ISL_2536392, EPI_ISL_2536418, EPI_ISL_2536424, EPI_ISL_2536428, EPI_ISL_2536437, EPI_ISL_2536438, EPI_ISL_2536459, EPI_ISL_2536466, EPI_ISL_2536469, EPI_ISL_2692422, EPI_ISL_2692433, EPI_ISL_2714927, EPI_ISL_2714937, EPI_ISL_2714941, EPI_ISL_2714943			
see above	Incyte Diagnostics Spokane	Seattle Flu Study	Amanda Adler; Barry R. Lutz; Benjamin Pelle; Brian Hiatt; Caitlin R. Wolf; Chris D. Frazar; Deborah A. Nickerson; Elisabeth Brandstetter; Erica Ryke; Geoff Melly; Helen Y. Chu; Janet A. Englund; Jay Shendure; Jover Lee; Kairsten Fay; Kirsten Lacombe; Lea M. Starita; Mark J. Rieder; Matthew Richardson; Matthew Thompson; Melissa Truong; Michael Boeckh; Michael Famulare; Misja Ilcisin; Peter D. Han; Philip Dykema; Romesh Gautom; Scott Lindquist; Thomas R. Sibley; Trevor Bedford
EPI_ISL_2304418, EPI_ISL_2304419, EPI_ISL_2304448, EPI_ISL_2304449, EPI_ISL_2304451, EPI_ISL_2304452, EPI_ISL_2304454			
see above	Indiana Animal Disease Diagnostic Laboratory	Carpi Laboratory - Purdue University	Abebe A Fola; G Kenitra Hendrix; Giovanna Carpi; Ilinca I Ciubotariu; Jack Dorman; Jobin J Kattoor; Lev Gorenstein; Nicole M Perry; Rebecca P Wilkes
EPI_ISL_2444235	Indiana State Department of Health	Quantigen Biosciences	Paul J. Chidress
EPI_ISL_2660339, EPI_ISL_2660351, EPI_ISL_2660355, EPI_ISL_2660363	Infectious Disease Program, Broad Institute of Harvard and MIT	Infectious Disease Program, Broad Institute of Harvard and MIT	A.E.; Adams, G.; Anahtar, M.; B.L.; B.W.; Bauer, M.; Birren; Branda, J.; Carter, A.; Cerrato, F.; Chaluvadi, S.; Chapman; Cusick, C.; D.J.; DeRuff, K.; Flowers, K.; Gallagher, G.; Gladden-Young, A.; Gnirke, A.; Harris, J.; J.E.; K.J.; LaRocque, R.; Lagerborg, K.; Lemieux; Lin; Loreth, C.; MacInnis; Neumann, A.; Normandin, E.; P.C.; Park; Pierce, V.; Reilly, S.; Rosenberg, E.; Rudy, M.; Ryan, E.; S.B.; Sabeti; Shaw, B.; Siddle; Slater, D.; Smole, S.; Tomkins-Tinch, C.; Turbett, S.
EPI_ISL_2087169, EPI_ISL_2087180, EPI_ISL_2087204, EPI_ISL_2087239, EPI_ISL_2087245, EPI_ISL_2087284, EPI_ISL_2087351, EPI_ISL_2087374, EPI_ISL_2087463, EPI_ISL_2087567, EPI_ISL_2087621, EPI_ISL_2087640, EPI_ISL_2087772, EPI_ISL_2087783, EPI_ISL_2087803, EPI_ISL_2087827, EPI_ISL_2087900, EPI_ISL_2087935, EPI_ISL_2088221, EPI_ISL_2088241, EPI_ISL_2088250, EPI_ISL_2088376, EPI_ISL_2088412, EPI_ISL_2088418, EPI_ISL_2088456, EPI_ISL_2088514, EPI_ISL_2088564, EPI_ISL_2088619, EPI_ISL_2088651, EPI_ISL_2088742, EPI_ISL_2088832, EPI_ISL_2088858, EPI_ISL_2088951, EPI_ISL_2089330, EPI_ISL_2089416, EPI_ISL_2132374, EPI_ISL_2186443, EPI_ISL_2186561, EPI_ISL_2186568, EPI_ISL_2186620, EPI_ISL_2186815, EPI_ISL_2186862, EPI_ISL_2187001, EPI_ISL_2187222, EPI_ISL_2187223, EPI_ISL_2282269, EPI_ISL_2282561, EPI_ISL_2282601, EPI_ISL_2282639, EPI_ISL_2282640, EPI_ISL_2282655, EPI_ISL_2282714, EPI_ISL_2282787, EPI_ISL_2282899, EPI_ISL_2323487, EPI_ISL_2323648, EPI_ISL_2323673, EPI_ISL_2323677, EPI_ISL_2323678, EPI_ISL_2323698, EPI_ISL_2323701, EPI_ISL_2323705, EPI_ISL_2323758, EPI_ISL_2323812, EPI_ISL_2323904, EPI_ISL_2323958, EPI_ISL_2324039, EPI_ISL_2324062, EPI_ISL_2324078, EPI_ISL_2324094, EPI_ISL_2324110, EPI_ISL_2375552, EPI_ISL_2375555, EPI_ISL_2375684, EPI_ISL_2652549, EPI_ISL_2652797, EPI_ISL_2652818, EPI_ISL_2652884, EPI_ISL_2652896, EPI_ISL_2652938, EPI_ISL_2652968			

see above	Infinity Biologix	Centers for Disease Control and Prevention Division of Viral Diseases, Pathogen Discovery	Adrian Paskey; Benjamin Rambo-Martin; Chirayu Goswami; Christian Bixby; Christopher Gulvick; Clinton R. Paden; Dakota Howard; Darlene Wagner; Dhwani Batra; Duncan MacCannell; Jason Caravas; Jonathan Schultz; Kara Moser; Matthew Schmerer; Peter W. Cook; Robin Grimwood; Russ Hager; Scott Sammons; Shatavia Morrison; Yihe Wang; Yvette Unoarumhi
EPI_ISL_2828662, EPI_ISL_2828663, EPI_ISL_2828664, EPI_ISL_2828665, EPI_ISL_2828666, EPI_ISL_2828667	Instituo Adolfo Lutz - Reginal de Ribeirao Preto	Instituto Adolfo Lutz, Interdiciplinary Procedures Center, Strategic Laboratory	Caio Vinicius Dias Lopes; Claudia Regina Gonçalves; Claudio Tavares Sacchi; Erica Valessa Ramos Gomes; Karoline Rodrigues Campos
EPI_ISL_2691151, EPI_ISL_2691152, EPI_ISL_2691153, EPI_ISL_2691154, EPI_ISL_2691157, EPI_ISL_2691158, EPI_ISL_2691160, EPI_ISL_2691161, EPI_ISL_2691162, EPI_ISL_2691163, EPI_ISL_2691164, EPI_ISL_2691165, EPI_ISL_2691166, EPI_ISL_2691167, EPI_ISL_2691168, EPI_ISL_2691169, EPI_ISL_2828654, EPI_ISL_2828655, EPI_ISL_2828657, EPI_ISL_2828658, EPI_ISL_2828660			
see above	Instituo Adolfo Lutz Central	Instituto Adolfo Lutz, Interdiciplinary Procedures Center, Strategic Laboratory	Caio Vinicius Dias Lopes; Claudia Regina Gonçalves; Claudio Tavares Sacchi; Erica Valessa Ramos Gomes; Karoline Rodrigues Campos; Leonardo Jose Tadeu de Araujo
EPI_ISL_1739244, EPI_ISL_2361461	Institute of Microbiology, Universidad San Francisco de Quito	Institute of Microbiology, Universidad San Francisco de Quito	Belén Prado-Vivar; Bernardo Gutiérrez; Ernesto Pazos; Fernada Zurita; Gabriel Trueba; Gabriela Zambrano; Juan José Guadalupe; Michelle Grunauer; Monica Becerra-Wong; Patricio Rojas-Silva; Paúl Cárdenas; Sully Márquez; Verónica Barragán
EPI_ISL_2691171, EPI_ISL_2691172, EPI_ISL_2691173, EPI_ISL_2691176, EPI_ISL_2691177, EPI_ISL_2691178, EPI_ISL_2691180, EPI_ISL_2691181, EPI_ISL_2691182, EPI_ISL_2691183, EPI_ISL_2691184, EPI_ISL_2691185, EPI_ISL_2691186, EPI_ISL_2691187, EPI_ISL_2691188, EPI_ISL_2691189, EPI_ISL_2691190, EPI_ISL_2691191, EPI_ISL_2691192, EPI_ISL_2691193, EPI_ISL_2691195, EPI_ISL_2691196, EPI_ISL_2691197, EPI_ISL_2691198, EPI_ISL_2691199, EPI_ISL_2691200, EPI_ISL_2691201, EPI_ISL_2691202, EPI_ISL_2691203, EPI_ISL_2828647, EPI_ISL_2828648, EPI_ISL_2828649, EPI_ISL_2828651, EPI_ISL_2828652, EPI_ISL_2828653			
see above	Instituto Adolfo Lutz - Reginal de Sao José do Rio Preto	Instituto Adolfo Lutz, Interdiciplinary Procedures Center, Strategic Laboratory	Caio Vinicius Dias Lopes; Claudia Regina Gonçalves; Claudio Tavares Sacchi; Erica Valessa Ramos Gomes; Karoline Rodrigues Campos; Leonardo Jose Tadeu de Araujo
EPI_ISL_2691110, EPI_ISL_2691116, EPI_ISL_2691118, EPI_ISL_2919240, EPI_ISL_2919241, EPI_ISL_2919243, EPI_ISL_2919244, EPI_ISL_2919248, EPI_ISL_2919250, EPI_ISL_2919252, EPI_ISL_2919254			
see above	Instituto Adolfo Lutz - Regional de Bauru	Instituto Adolfo Lutz, Interdiciplinary Procedures Center, Strategic Laboratory	Caio Vinicius Dias Lopes; Claudia Regina Gonçalves; Claudio Tavares Sacchi; Erica Valessa Ramos Gomes; Karoline Rodrigues Campos; Leonardo Jose Tadeu de Araujo
EPI_ISL_2828686, EPI_ISL_2828691, EPI_ISL_2828692, EPI_ISL_2828693, EPI_ISL_2828694, EPI_ISL_2828695, EPI_ISL_3316200, EPI_ISL_3316207, EPI_ISL_3316216			
see above	Instituto Adolfo Lutz - Regional de Campinas	Instituto Adolfo Lutz, Interdiciplinary Procedures Center, Strategic Laboratory	Caio Vinicius Dias Lopes; Claudia Regina Gonçalves; Claudio Tavares Sacchi; Erica Valessa Ramos Gomes; Karoline Rodrigues Campos; Leonardo Tadeu de Araujo; Marlon Benedito Nascomento Santos
EPI_ISL_2614627, EPI_ISL_2614628, EPI_ISL_2691089, EPI_ISL_2691090, EPI_ISL_2691091, EPI_ISL_2691092, EPI_ISL_2691093, EPI_ISL_2691094, EPI_ISL_2691095			
see above	Instituto Adolfo Lutz - Regional de Marilia	Instituto Adolfo Lutz, Interdiciplinary Procedures Center, Strategic Laboratory	Caio Vinicius Dias Lopes; Claudia Regina Gonçalves; Claudio Tavares Sacchi; Erica Valessa Ramos Gomes; Karoline Rodrigues Campos; Leonardo Jose Tadeu de Araujo
EPI_ISL_2614618, EPI_ISL_2614619, EPI_ISL_2614620, EPI_ISL_2614621, EPI_ISL_2614622, EPI_ISL_2614623, EPI_ISL_2614624, EPI_ISL_2614625, EPI_ISL_3316154, EPI_ISL_3316156, EPI_ISL_3316174, EPI_ISL_3316228, EPI_ISL_3316234, EPI_ISL_3316235			
see above	Instituto Adolfo Lutz - Regional de Presidente Prudente	Instituto Adolfo Lutz, Interdiciplinary Procedures Center, Strategic Laboratory	Caio Vinicius Dias Lopes; Claudia Regina Gonçalves; Claudio Tavares Sacchi; Erica Valessa Ramos Gomes; Karoline Rodrigues Campos; Leonardo Jose Tadeu de Araujo; Leonardo Tadeu de Araujo; Marlon Benedito Nascomento Santos
EPI_ISL_2691123, EPI_ISL_2691124, EPI_ISL_2691125	Instituto Adolfo Lutz - Regional de Ribeirao Preto	Instituto Adolfo Lutz, Interdiciplinary Procedures Center, Strategic Laboratory	Caio Vinicius Dias Lopes; Claudia Regina Gonçalves; Claudio Tavares Sacchi; Erica Valessa Ramos Gomes; Karoline Rodrigues Campos; Leonardo Jose Tadeu de Araujo
EPI_ISL_2828697, EPI_ISL_2828698, EPI_ISL_2828700, EPI_ISL_2828701, EPI_ISL_2828703	Instituto Adolfo Lutz - Regional de Santos	Instituto Adolfo Lutz, Interdiciplinary Procedures Center, Strategic Laboratory	Caio Vinicius Dias Lopes; Claudia Regina Gonçalves; Claudio Tavares Sacchi; Erica Valessa Ramos Gomes; Karoline Rodrigues Campos
EPI_ISL_2919264, EPI_ISL_2958834, EPI_ISL_2958835, EPI_ISL_2958836, EPI_ISL_2958837, EPI_ISL_2958838, EPI_ISL_2958839			
see above	Instituto Adolfo Lutz - Regional de Sao Jose do Rio Preto	Instituto Adolfo Lutz, Interdiciplinary Procedures Center, Strategic Laboratory	Caio Vinicius Dias Lopes; Claudia Regina Gonçalves; Claudio Tavares Sacchi; Erica Valessa Ramos Gomes; Karoline Rodrigues Campos
EPI_ISL_2958844, EPI_ISL_2958845, EPI_ISL_2958846	Instituto Adolfo Lutz - Regional de Sorocaba	Instituto Adolfo Lutz, Interdiciplinary Procedures Center, Strategic Laboratory	Caio Vinicius Dias Lopes; Claudia Regina Gonçalves; Claudio Tavares Sacchi; Erica Valessa Ramos Gomes; Karoline Rodrigues Campos
EPI_ISL_2691126, EPI_ISL_2691127, EPI_ISL_2691128, EPI_ISL_2691129, EPI_ISL_2691130, EPI_ISL_2691131, EPI_ISL_2691132, EPI_ISL_2691134, EPI_ISL_2691135, EPI_ISL_2691136, EPI_ISL_2691139, EPI_ISL_2691140, EPI_ISL_2691141, EPI_ISL_2691142, EPI_ISL_2691143, EPI_ISL_2756427, EPI_ISL_2756428, EPI_ISL_2756429, EPI_ISL_2756430, EPI_ISL_2756431, EPI_ISL_2958848, EPI_ISL_2958849, EPI_ISL_2958850, EPI_ISL_2958851, EPI_ISL_2958852, EPI_ISL_2958853			
see above	Instituto Adolfo Lutz Central	Instituto Adolfo Lutz, Interdiciplinary Procedures Center, Strategic Laboratory	Caio Vinicius Dias Lopes; Claudia Regina Gonçalves; Claudio Tavares Sacchi; Erica Valessa Ramos Gomes; Karoline Rodrigues Campos; Leonardo Jose Tadeu de Araujo
EPI_ISL_2241317, EPI_ISL_2241318, EPI_ISL_2241319, EPI_ISL_2241322, EPI_ISL_2241323, EPI_ISL_2241324, EPI_ISL_2241325, EPI_ISL_2241326, EPI_ISL_4104677, EPI_ISL_4104681, EPI_ISL_4104691, EPI_ISL_4104695, EPI_ISL_4104704, EPI_ISL_4104705, EPI_ISL_4104709, EPI_ISL_4104715, EPI_ISL_4104716, EPI_ISL_4104719, EPI_ISL_4104725, EPI_ISL_4104727, EPI_ISL_4104734			
see above	Instituto Butantan	Instituto Butantan	Antonio Jorge Martins; Claudia Renata dos Santos Barros; David Schlesinger; Debora Botequio Moretti; Dimas Tadeu Covas; Elaine Cristina Marqueze; Elaine Vieira Santos; Evandra Strazza Rodrigues; Heidge Fukumasu; Jayme Augusto de Souza-Neto; José Salvatore Leister Patané; Luiz Alcantara; Luiz Lehmann Coutinho; Maria Carolina Elias; Maurício Lacerda Nogueira; Rafael dos Santos Bezerra; Raul Machado Neto; Rejane Maria Tommasini Grotto; Ricardo Haddad; Sandra Coccuzzo Sampaio Vessoni; Simone Kashima; Svetoslav Nanev Slavov; Vincent Louis Viala
EPI_ISL_2348761, EPI_ISL_2348790	Instituto Nacional De Investigación En Salud Pública-Crn De Influenza Y Otros Virus Respiratorios	NIC-Instituto Nacional de Investigación en Salud Pública	Alfredo Bruno; Domenica de Mora.; Jimmy Garcés; Johanna Laines; Lizbeth Patiño; Manuel Gonzalez; Maritza Olmedo; Michelle Páez
EPI_ISL_2346467, EPI_ISL_2490588	Instituto Nacional de Enfermedades Respiratorias (INER)	Centro de Investigación en Enfermedades Infecciosas (CIENI), Instituto Nacional de Enfermedades Respiratorias (INER)	; Alejandra Garcia-Gasca; Alejandra Hernández-Terán; Alejandro Sanchez-Flores; Alfredo Herrera-Estrella; Alicia Ocaña-Mondragón; Andreu Comas-García; Angel Gustavo Salas-Lais; Antonio Loza Román; Bernardo Martínez-Miguel; Blanca Taboada; Brenda Irasema Maldonado-Meza; Bruno Gomez-Gil; Carla Ivón Herrera-Najera; Carlos F. Arias; Celia Boukadida; Clara Esperanza Santacruz-Tinoco; Concepción Grajales-Muñiz; Consorcio Mexicano de Vigilancia Genómica (CoViGen-Mex). Authors (in alphabetical order): Julio Elias Alvarado-Yaah; Cristóbal Cháidez-Quiróz; Célida Duque Molina; Célida Martínez-Rodríguez; Daniel Fregoso-Rueda; Daniel Lira Morales; Eduardo Becerril-Vargas; Fernando Fontove-Herrera; Fidencio Mejía-Nepomuceno; Francisco Pulido; Gloria Elena Espinosa-Ayala; Gloria María Molina-Salinas; Gloria Vazquez; Hector Esteban Paz-Juárez; Hector Montoya-Fuentes; Helen Haydee Fernanda Ramírez-Plascencia; Irvin González-López; Jean Pierre González; Joel Armando Vázquez-Pérez; Joel Armando Vázquez-Pérez.; Jorge Salas-Hernández; José Antonio Enciso-Moreno; José Arturo Martínez-Orozco; José Esteban Muñoz-Medina; José de Jesús Nuñez-Contreras; Juan Bautista Chale-Dzul; Julissa Enciso-Ibarra; Luis Alberto Ochoa-Carrera; Margarita Matías-Florentino; Mario Mújica-Sánchez; Marissa Perez-Garcia; María Guadalupe Santiago-Mauricio; María Guadalupe de Jesús Mireles-Rivera; Nelly Sélem-Mojica; Pavel Isa; Ricardo Ciria Merce; Ricardo Grande; Rosa María Gutierrez Rios; Santiago Ávila-Ríos; Selene Zárate; Susana Lopez; Victor Eduardo Garcia-Arias; Víctor Hugo Borja-Aburto
EPI_ISL_2646131	Instituto Nacional de Medicina Genomica	Centro de Investigación en Enfermedades Infecciosas (CIENI), Instituto Nacional de Enfermedades Respiratorias (INER)	Avila-Rios S; Boukadida C; Cedro-Tanda A; Herrera-Montalvo LA; Hidalgo-Miranda A; Matias-Florentino M; Mendoza-Vargas A; Perez-Garcia M; Reyes-Grajeda JP; Reyes-Teran G
EPI_ISL_2295417, EPI_ISL_2295418, EPI_ISL_2295419, EPI_ISL_2295420, EPI_ISL_2295423, EPI_ISL_2295425, EPI_ISL_2295427, EPI_ISL_2295428, EPI_ISL_2295432, EPI_ISL_2295434, EPI_ISL_2295435, EPI_ISL_2295436, EPI_ISL_2295437, EPI_ISL_2295438, EPI_ISL_2295439, EPI_ISL_2295440, EPI_ISL_2295442, EPI_ISL_2295444, EPI_ISL_2295447, EPI_ISL_2295449, EPI_ISL_2295450, EPI_ISL_2295451, EPI_ISL_2378643, EPI_ISL_2378646, EPI_ISL_2378649, EPI_ISL_2378650, EPI_ISL_2378653, EPI_ISL_2378656, EPI_ISL_2378659, EPI_ISL_2378661, EPI_ISL_2378664, EPI_ISL_2378665, EPI_ISL_2378666, EPI_ISL_2378667, EPI_ISL_2378668, EPI_ISL_2378669, EPI_ISL_2378672, EPI_ISL_2378673, EPI_ISL_2378674, EPI_ISL_2378676, EPI_ISL_2378678, EPI_ISL_2378679, EPI_ISL_2378680, EPI_ISL_2378681, EPI_ISL_2378684, EPI_ISL_2378686, EPI_ISL_2378688, EPI_ISL_2378689, EPI_ISL_2378690, EPI_ISL_2378691, EPI_ISL_2378692, EPI_ISL_2378693, EPI_ISL_2378694, EPI_ISL_3118820, EPI_ISL_3118828, EPI_ISL_3118833, EPI_ISL_3118834, EPI_ISL_3118835, EPI_ISL_3118840, EPI_ISL_3118842, EPI_ISL_3118846			
see above	Instituto de Biotecnologia - UNESP-Botucatu-SP	Instituto de Biotecnologia - UNESP-Botucatu-SP	Cecília Artico Banho; Cíntia Bittar; Fábio Sossai Possebon; Guilherme Campos; Helena Lage Ferreira; Jorge A. Petroli Marchesi; João Pessoa Araújo Jr.; Leila Sabrina Ullmann; Lívia Sacchetto; Maisa C. Pereira Parra; Marília Moraes; Maurício L. Nogueira; Paula Rahal; Paulo Inacio da Costa
EPI_ISL_3104701, EPI_ISL_3104702, EPI_ISL_3104706, EPI_ISL_3104707, EPI_ISL_3104708, EPI_ISL_3236435	Instituto de Medicina Tropical & Salud Global Universidad Iberoamericana	Grubaugh Lab - Yale School of Public Health	Alejandaro Vallejo Degaudenzi; Anderson Brito; Andrea Firpo; Annie Watkins; Chaney Kalinich; Chantal Vogels; David Rivera Aponte; Elisa Contreras; Ernesto Vazquez; Esperanza Mendoza; Fabiola Cruz-Lopez; Fabiola Fontanet; Isabel Ott; Jessica Rothman; Joseph Fauver; Karolane Gonzalez; Kendall Billig; Mallery Breban; Mary Petrone; Natalie Machargo; Nathan Grubaugh; Patricia Serrano; Robert Paulino-Ramirez; Tania Mitwalli; Tara Alpert; Tobias Koch; Vianca Aponte; Victor Virgilio Calderon
EPI_ISL_2536526, EPI_ISL_2536536, EPI_ISL_2715007	Interpath Laboratory	Seattle Flu Study	Amanda Adler; Barry R. Lutz; Benjamin Pelle; Brian Hiatt; Caitlin R. Wolf; Chris D. Frazar; Deborah A. Nickerson; Elisabeth Brandstetter; Erica Ryke; Geoff Melly; Helen Y. Chu; Janet A. Englund; Jay Shendure; Jover Lee; Kairsten Fay; Kirsten Lacombe; Lea M. Starita; Mark J. Rieder; Matthew Richardson; Matthew Thompson; Melissa Truong; Michael Boeckh; Michael Famulare; Misja Ilcisin; Peter D. Han; Philip Dykema; Romesh Gautom; Scott Lindquist; Thomas R. Sibley; Trevor Bedford
EPI_ISL_2691146	Intituto Adolfo Lutz - Regional de Sorocaba	Instituto Adolfo Lutz, Interdiciplinary Procedures Center, Strategic Laboratory	Caio Vinicius Dias Lopes; Claudia Regina Gonçalves; Claudio Tavares Sacchi; Erica Valessa Ramos Gomes; Karoline Rodrigues Campos; Leonardo Jose Tadeu de Araujo
EPI_ISL_2636150	Israel Central Virology laboratory	Israel National Consortium for SARS-CoV-2 sequencing	Dana Bar-Ilan; Efrat Dahan Bucris; Efrat Glick-Saar; Ella Mendelson; Gideon Rechavi; Michal Mandelboim; Miranda Geva; Neta Zuckerman; Omri Nayshool; Oran Erster; Orna Mor
EPI_ISL_2194355, EPI_ISL_2194401, EPI_ISL_2194436,	Istituto Zooprofilattico Sperimentale del Mezzogiorno	TIGEM	Antonio Grimaldi Patrizia Annunziata Francesco Panariello Biancamaria Pierri Claudia Tiberio Teresa Giuliano Valentina Bouche Chiara Colantuono Maria Concetta Cuomo Denise Di Concilio Lucio Di Filippo Anna Manfredi Marcello Salvi Antonio Limone Luigi Atripaldi Pellegrino Cerino Andrea Ballabio Davide Cacchiarelli

EPI_ISL_2282039, EPI_ISL_2282090, EPI_ISL_2282125			
EPI_ISL_2615918, EPI_ISL_2615919	J.W. Ruby Memorial Hospital	WVU and Marshall University Combined Genomics Core Facilities	James Denvir; Peter Perrotta; Peter Stoilov; Ryan Percifield; Wesley Kimble
EPI_ISL_2444186	JOSE BONIFACIO	Instituto Butantan / FAMERP	Antonio Jorge Martins; Claudia Renata dos Santos Barros; David Schlesinger; Debora Botequio Moretti; Dimas Tadeu Covas; Elaine Cristina Marqueze; Elaine Vieira Santos; Evandra Strazza Rodrigues; Heidge Fukumasu; Jayme Augusto de Souza-Neto; José Salvatore Leister Patané; Luiz Alcantara; Luiz Lehmann Coutinho; Maria Carolina Elias; Maurício Lacerda Nogueira; Rafael dos Santos Bezerra; Raul Machado Neto; Rejane Maria Tommasini Grotto; Ricardo Haddad; Sandra Coccuzzo Sampaio Vessoni; Simone Kashima; Svetoslav Nanev Slavov; Vincent Louis Viala
EPI_ISL_2322377	Jessa	Jessa	Berden et al. on behalf of the Jessa_cmdLab
EPI_ISL_2248906, EPI_ISL_2248907, EPI_ISL_2248910	Joaquim Chaves Saude	Instituto Nacional de Saude (INSA)	Borges et al
EPI_ISL_2476376, EPI_ISL_2476380, EPI_ISL_2476384, EPI_ISL_2476386, EPI_ISL_2692054, EPI_ISL_2692057, EPI_ISL_2692060, EPI_ISL_2692063, EPI_ISL_2692064, EPI_ISL_2692066, EPI_ISL_2692068, EPI_ISL_2692070, EPI_ISL_2692072, EPI_ISL_2692074, EPI_ISL_2692076			
see above	John H Stroger Jr Hospital of Cook County	RIPHL at Rush University Medical Center	Ah-Young Sung; Felix Araujo Perez; Kevin Kunstman; Laura Furtado; Marieta Hyde; Max Kolton; Sharon Welbel; Stefan Green
EPI_ISL_2331499, EPI_ISL_2331546	Johns Hopkins Hospital Department of Pathology	Johns Hopkins Hospital Department of Pathology	Adannaya Amadi; C. Paul Morris; Chun Huai Luo; Heba H. Mostafa; Matthew Schwartz; Nicholas Gallagher
EPI_ISL_2384201, EPI_ISL_2645292, EPI_ISL_2645297	KS Health and Environmental Laboratories	Centers for Disease Control and Prevention Division of Viral Diseases, Pathogen Discovery	Alison Laufer Halpin; Ben L. Rambo-Martin; Clinton R. Paden; Dakota Howard; Darlene Wagner; Dave Wentworth; Dhwani Batra; Jasmine Padilla; Justin Lee; Katie Dillon; Krista Queen; Kristen Knipe; Kristine Lacek; Mark Burroughs; Matthew Schmerer; Mili Sheth; Peter Cook; Sam Shepard; Sarah Nobles; Shoshona Le; Suxiang Tong; Vivien Dugan; Yvette Unoarumhi
EPI_ISL_2425155, EPI_ISL_2726430, EPI_ISL_2726467, EPI_ISL_2726473, EPI_ISL_2832723, EPI_ISL_2833071, EPI_ISL_2833419, EPI_ISL_2833426			
see above	KU Leuven, Rega Institute, Clinical and Epidemiological Virology	KU Leuven, Rega Institute, Clinical and Epidemiological Virology	Bert Vanmechelen; Joan Marti-Carerras; Piet Maes; Tony Wawina-Bokalanga
EPI_ISL_2383979	KY State Public Health Lab	Centers for Disease Control and Prevention Division of Viral Diseases, Pathogen Discovery	Alison Laufer Halpin; Ben L. Rambo-Martin; Clinton R. Paden; Dakota Howard; Darlene Wagner; Dave Wentworth; Dhwani Batra; Jasmine Padilla; Justin Lee; Katie Dillon; Krista Queen; Kristen Knipe; Kristine Lacek; Mark Burroughs; Matthew Schmerer; Mili Sheth; Peter Cook; Sam Shepard; Sarah Nobles; Shoshona Le; Suxiang Tong; Vivien Dugan; Yvette Unoarumhi
EPI_ISL_3561093, EPI_ISL_3561231, EPI_ISL_3561270, EPI_ISL_3561295, EPI_ISL_3561318, EPI_ISL_3561509, EPI_ISL_3561554			
see above	Kaiser Permanente NW Reginal Lab	OHSU MM Lab	Yun Wu
EPI_ISL_3505123, EPI_ISL_3505124, EPI_ISL_3505125, EPI_ISL_3505126, EPI_ISL_3505127, EPI_ISL_3505128, EPI_ISL_3505129, EPI_ISL_3505130			
see above	Kaiser Permanente NW Reginal Lab	OHSU-MM Lab	Yun Wu
EPI_ISL_2934066, EPI_ISL_2934068	Kaiser Regional	Santa Clara County Public Health Laboratory	Santa Clara County Public Health Department
EPI_ISL_1805462, EPI_ISL_1964227, EPI_ISL_2037393, EPI_ISL_2092832, EPI_ISL_2092834, EPI_ISL_2101374, EPI_ISL_2156041, EPI_ISL_2230472, EPI_ISL_2271719, EPI_ISL_2289071, EPI_ISL_2289072, EPI_ISL_2289073, EPI_ISL_2289074			
see above	Kansas Health and Environmental Lab	Kansas Health and Environmental Lab	Ben Olsen; Jonathan Barnell; Katherine Wiggins; Mike Grose; and Phil Adam
EPI_ISL_1964231	Kansas Health and Environmental Lab	Wichita State University - Molecular Diagnostics Lab	Ben Olsen; Jonathan Barnell; Mike Grose; and Phil Adam
EPI_ISL_2898414	Karolinska University Hospital Solna	Karolinska University Hospital	Annelie Bjerkner; Isak Sylvin; Jan Albert; Karolina Ininbergs; Lina Guerra Blomqvist; Lynda Eneh; Martin Ekman; Martina Wahlund; Robert Dyrdak; Sandra Broddesson; Tanja Normark; Tobias Allander; Valtteri Wirta; Zhibing Yun
EPI_ISL_3737713, EPI_ISL_3737714, EPI_ISL_3737715, EPI_ISL_3742793, EPI_ISL_3758007, EPI_ISL_4080577, EPI_ISL_4080578, EPI_ISL_4080579, EPI_ISL_4080580, EPI_ISL_4080726			
see above	Kentucky State Public Health Lab	Kentucky State Public Health Lab	Anjayya Tummala; Chitralipi Kattunga; Joshua Tobias; Karim George; Matthew Johnson; Rachel Zinner; Rhonda Lucas; Vaneet Arora; William Grooms
EPI_ISL_3354895	LA Office of Public Health Laboratories	Centers for Disease Control and Prevention Division of Viral Diseases, Pathogen Discovery	Alex Burgin; Ben Rambo-Martin; Clinton Paden; Dakota Howard; Dave Wentworth; Dhwani Batra; Jasmine Padilla; Justin Lee; Krista Queen; Kristen Knipe; Kristine Lacek; Mark Burroughs; Matthew Schmerer; Meghan Bentz; Mili Sheth; Peter Cook; Sam Shepard; Sarah Nobles; Suxiang Tong; Vivien Dugan; Yvette Unoarumhi
EPI_ISL_2482673	LABORATOIRE CREAVALLEE	CNR Virus des Infections Respiratoires - France SUD	Antonin Bal; Bruno Lina; Gregory Destras; Gwendolyne Burfin; Hadrien Regue; Laurence Josset; Martine Valette; Quentin Semanas
EPI_ISL_2445495, EPI_ISL_2445497, EPI_ISL_2473783, EPI_ISL_2493646, EPI_ISL_2493647, EPI_ISL_2493648	LABORATORIO DE FRANCA	Instituto Butantan	Antonio Jorge Martins; Claudia Renata dos Santos Barros; David Schlesinger; Debora Botequio Moretti; Dimas Tadeu Covas; Elaine Cristina Marqueze; Elaine Vieira Santos; Evandra Strazza Rodrigues; Heidge Fukumasu; Jayme Augusto de Souza-Neto; José Salvatore Leister Patané; Luiz Alcantara; Luiz Lehmann Coutinho; Maria Carolina Elias; Maurício Lacerda Nogueira; Rafael dos Santos Bezerra; Raul Machado Neto; Rejane Maria Tommasini Grotto; Ricardo Haddad; Sandra Coccuzzo Sampaio Vessoni; Simone Kashima; Svetoslav Nanev Slavov; Vincent Louis Viala
EPI_ISL_2445561, EPI_ISL_2445562	LABORATORIO DR PAULO EMILIO DALESSANDRO PINDAMONHANGABA	Instituto Butantan	Antonio Jorge Martins; Claudia Renata dos Santos Barros; David Schlesinger; Debora Botequio Moretti; Dimas Tadeu Covas; Elaine Cristina Marqueze; Elaine Vieira Santos; Evandra Strazza Rodrigues; Heidge Fukumasu; Jayme Augusto de Souza-Neto; José Salvatore Leister Patané; Luiz Alcantara; Luiz Lehmann Coutinho; Maria Carolina Elias; Maurício Lacerda Nogueira; Rafael dos Santos Bezerra; Raul Machado Neto; Rejane Maria Tommasini Grotto; Ricardo Haddad; Sandra Coccuzzo Sampaio Vessoni; Simone Kashima; Svetoslav Nanev Slavov; Vincent Louis Viala
EPI_ISL_2651229	LABORATORIO HEMATOLOGICO	Universidad Nacional de Colombia - Laboratorio Genómico One Health	Andres F. Cardona-Rios; Carlos Franco-Muñoz; Carolina Muñoz-Arango; Celeny Ortiz; Daniel O. Maldonado-Perez; Diego A. Álvarez-Díaz; Hector Alejandro Ruiz-Moreno; Idabely Betancur Ortiz; Jorge E. Osorio; Juan P. Hernandez-Ortiz; Karl A Ciuoderis; Katherine Laiton-Donato; Laura Silvana Perez; Lina M. Hurtado; Marcela Mercado-Reyes; Maria Angélica Maya; Maria Stella López; Rita Almanza Payares; Sandra Ines Cano; Simón Villegas Velásquez
EPI_ISL_2345141, EPI_ISL_2345142, EPI_ISL_2345149, EPI_ISL_2345150, EPI_ISL_2345152	LABORATORIO LOCAL DE ITAPECERICA DA SERRA	Instituto Butantan / ESALQ-Piracicaba	Antonio Jorge Martins; Claudia Renata dos Santos Barros; David Schlesinger; Debora Botequio Moretti; Dimas Tadeu Covas; Elaine Cristina Marqueze; Elaine Vieira Santos; Evandra Strazza Rodrigues; Heidge Fukumasu; Jayme Augusto de Souza-Neto; José Salvatore Leister Patané; Luiz Alcantara; Luiz Lehmann Coutinho; Maria Carolina Elias; Maurício Lacerda Nogueira; Rafael dos Santos Bezerra; Raul Machado Neto; Rejane Maria Tommasini Grotto; Ricardo Haddad; Sandra Coccuzzo Sampaio Vessoni; Simone Kashima; Svetoslav Nanev Slavov; Vincent Louis Viala
EPI_ISL_2493684, EPI_ISL_2493703	LABORATORIO MUNICIPAL DE ANALISES CLINICAS DE ITANHAEM	Instituto Butantan	Antonio Jorge Martins; Claudia Renata dos Santos Barros; David Schlesinger; Debora Botequio Moretti; Dimas Tadeu Covas; Elaine Cristina Marqueze; Elaine Vieira Santos; Evandra Strazza Rodrigues; Heidge Fukumasu; Jayme Augusto de Souza-Neto; José Salvatore Leister Patané; Luiz Alcantara; Luiz Lehmann Coutinho; Maria Carolina Elias; Maurício Lacerda Nogueira; Rafael dos Santos Bezerra; Raul Machado Neto; Rejane Maria Tommasini Grotto; Ricardo Haddad; Sandra Coccuzzo Sampaio Vessoni; Simone Kashima; Svetoslav Nanev Slavov; Vincent Louis Viala
EPI_ISL_2493368, EPI_ISL_2493379, EPI_ISL_2493380, EPI_ISL_2493381, EPI_ISL_2493397, EPI_ISL_2493398, EPI_ISL_2493403, EPI_ISL_2493440, EPI_ISL_2494151			
see above	LABORATORIO MUNICIPAL DE ANALISES CLINICAS DE RIO CLARO	Instituto Butantan	Antonio Jorge Martins; Claudia Renata dos Santos Barros; David Schlesinger; Debora Botequio Moretti; Dimas Tadeu Covas; Elaine Cristina Marqueze; Elaine Vieira Santos; Evandra Strazza Rodrigues; Heidge Fukumasu; Jayme Augusto de Souza-Neto; José Salvatore Leister Patané; Luiz Alcantara; Luiz Lehmann Coutinho; Maria Carolina Elias; Maurício Lacerda Nogueira; Rafael dos Santos Bezerra; Raul Machado Neto; Rejane Maria Tommasini Grotto; Ricardo Haddad; Sandra Coccuzzo Sampaio Vessoni; Simone Kashima; Svetoslav Nanev Slavov; Vincent Louis Viala
EPI_ISL_2493449, EPI_ISL_2493451, EPI_ISL_2493453, EPI_ISL_2493454, EPI_ISL_2493649, EPI_ISL_2493653, EPI_ISL_2494154, EPI_ISL_2494157			
see above	LABORATORIO MUNICIPAL DE PIRACICABA	Instituto Butantan	Antonio Jorge Martins; Claudia Renata dos Santos Barros; David Schlesinger; Debora Botequio Moretti; Dimas Tadeu Covas; Elaine Cristina Marqueze; Elaine Vieira Santos; Evandra Strazza Rodrigues; Heidge Fukumasu; Jayme Augusto de Souza-Neto; José Salvatore Leister Patané; Luiz Alcantara; Luiz Lehmann Coutinho; Maria Carolina Elias; Maurício Lacerda Nogueira; Rafael dos Santos Bezerra; Raul Machado Neto; Rejane Maria Tommasini Grotto; Ricardo Haddad; Sandra Coccuzzo Sampaio Vessoni; Simone Kashima; Svetoslav Nanev Slavov; Vincent Louis Viala
EPI_ISL_2344838, EPI_ISL_2344864, EPI_ISL_2344917, EPI_ISL_2344971, EPI_ISL_2344972, EPI_ISL_2344979, EPI_ISL_2344983, EPI_ISL_2344988, EPI_ISL_2345034, EPI_ISL_2345118, EPI_ISL_2345169, EPI_ISL_2345170, EPI_ISL_2345186, EPI_ISL_2345248, EPI_ISL_2345250, EPI_ISL_2345251, EPI_ISL_2345271, EPI_ISL_2345288, EPI_ISL_2345289, EPI_ISL_2345290, EPI_ISL_2345292, EPI_ISL_2345293, EPI_ISL_2345297, EPI_ISL_2345298			
see above	LABORATORIO MUNICIPAL DE PIRACICABA	Instituto Butantan / ESALQ-Piracicaba	Antonio Jorge Martins; Claudia Renata dos Santos Barros; David Schlesinger; Debora Botequio Moretti; Dimas Tadeu Covas; Elaine Cristina Marqueze; Elaine Vieira Santos; Evandra Strazza Rodrigues; Heidge Fukumasu; Jayme Augusto de Souza-Neto; José Salvatore Leister Patané; Luiz Alcantara; Luiz Lehmann Coutinho; Maria Carolina Elias; Maurício Lacerda Nogueira; Rafael dos Santos Bezerra; Raul Machado Neto; Rejane Maria Tommasini Grotto; Ricardo Haddad; Sandra Coccuzzo Sampaio Vessoni; Simone Kashima; Svetoslav Nanev Slavov; Vincent Louis Viala
EPI_ISL_2210259, EPI_ISL_2345741	LABORATORIO MUNICIPAL DE SUZANO	Instituto Butantan / Mendelics	Antonio Jorge Martins; Claudia Renata dos Santos Barros; David Schlesinger; Debora Botequio Moretti; Dimas Tadeu Covas; Elaine Cristina Marqueze; Elaine Vieira Santos; Evandra Strazza Rodrigues; Heidge Fukumasu; Jayme Augusto de Souza-Neto; José Salvatore Leister Patané; Luiz Alcantara; Luiz Lehmann Coutinho; Maria Carolina Elias; Maurício Lacerda Nogueira; Rafael dos Santos Bezerra; Raul Machado Neto; Rejane Maria Tommasini Grotto; Ricardo Haddad; Sandra Coccuzzo Sampaio Vessoni; Simone Kashima; Svetoslav Nanev Slavov; Vincent Louis Viala
EPI_ISL_2488814, EPI_ISL_2488815, EPI_ISL_2488816, EPI_ISL_2488817, EPI_ISL_2488818, EPI_ISL_2488819	LACEN - Laboratório Central de Saúde Pública do Para	Evandro Chagas Institute	A.M.; Barbagelata; E.C.; E.M.A.; Ferreira; J.A.; Junior; K.C.; L.C.; L.S.; M.C.; P.S.; Pinheiro; Santos; Silva; Sousa; Sousa Junior; W.D.C.; da Silva
EPI_ISL_3316308, EPI_ISL_3316309, EPI_ISL_3316310, EPI_ISL_3316311, EPI_ISL_3316312, EPI_ISL_3316313, EPI_ISL_3316314, EPI_ISL_3316315, EPI_ISL_3316316, EPI_ISL_3671906			
see above	LACEN do Distrito Federal	Instituto Adolfo Lutz, Interdiciplinary Procedures Center, Strategic Laboratory	Caio Vinicius Dias Lopes; Claudia Regina Gonçalves; Claudio Tavares Sacchi; Karoline Rodrigues Campos; Leonardo Tadeu de Araujo; Marlon Benedito Nascomento Santos
EPI_ISL_2691206, EPI_ISL_2691207, EPI_ISL_2691208, EPI_ISL_2691209, EPI_ISL_2691210, EPI_ISL_2691211, EPI_ISL_2691212, EPI_ISL_2691213, EPI_ISL_2691216, EPI_ISL_2691217, EPI_ISL_2691218			
see above	LACEN do Estado de Goias	Instituto Adolfo Lutz, Interdiciplinary Procedures Center, Strategic Laboratory	Caio Vinicius Dias Lopes; Claudia Regina Gonçalves; Claudio Tavares Sacchi; Erica Valessa Ramos Gomes; Karoline Rodrigues Campos; Leonardo Jose Tadeu de Araujo
EPI_ISL_3316160, EPI_ISL_3316166, EPI_ISL_3316176, EPI_ISL_3316177, EPI_ISL_3316180, EPI_ISL_3316182, EPI_ISL_3316185, EPI_ISL_3316188, EPI_ISL_3316190, EPI_ISL_3316203, EPI_ISL_3316211, EPI_ISL_3316212, EPI_ISL_3316217, EPI_ISL_3316219, EPI_ISL_3316223, EPI_ISL_3316226, EPI_ISL_3316229, EPI_ISL_3316230, EPI_ISL_3316231, EPI_ISL_3316233, EPI_ISL_3316241			

see above	LACEN do Estado de Mato Grosso	Instituto Adolfo Lutz, Interdiciplinary Procedures Center, Strategic Laboratory	Caio Vinicius Dias Lopes; Claudia Regina Gonçalves; Claudio Tavares Sacchi; Karoline Rodrigues Campos; Leonardo Tadeu de Araujo; Marlon Benedito Nascomento Santos
EPI_ISL_2919205, EPI_ISL_2919207, EPI_ISL_2919209, EPI_ISL_2919210, EPI_ISL_2919211, EPI_ISL_2919212, EPI_ISL_2919213, EPI_ISL_2919214, EPI_ISL_2919215, EPI_ISL_2919218, EPI_ISL_2919226			
see above	LACEN do Estado de Tocantins	Instituto Adolfo Lutz, Interdiciplinary Procedures Center, Strategic Laboratory	Caio Vinicius Dias Lopes; Claudia Regina Gonçalves; Claudio Tavares Sacchi; Erica Valessa Ramos Gomes; Karoline Rodrigues Campos
EPI_ISL_3545806, EPI_ISL_3545808, EPI_ISL_3545809, EPI_ISL_3545810, EPI_ISL_3545811, EPI_ISL_3545812, EPI_ISL_3545813, EPI_ISL_3545814, EPI_ISL_3691363, EPI_ISL_3691364, EPI_ISL_3691365, EPI_ISL_3691367, EPI_ISL_3691368, EPI_ISL_3691369, EPI_ISL_3691371, EPI_ISL_3691372, EPI_ISL_3691373, EPI_ISL_3691374, EPI_ISL_3691375, EPI_ISL_3691376, EPI_ISL_3691377, EPI_ISL_3691378, EPI_ISL_3691379, EPI_ISL_3691380, EPI_ISL_3691381			
see above	LACEN do Estado do Mato Grosso do Sul	Instituto Adolfo Lutz, Interdiciplinary Procedures Center, Strategic Laboratory	Caio Vinicius Dias Lopes; Claudia Regina Gonçalves; Claudio Tavares Sacchi; Karoline Rodrigues Campos; Leonardo Tadeu de Araujo; Marlon Benedito Nascomento Santos
EPI_ISL_3691685, EPI_ISL_3691686, EPI_ISL_3691687	LACEN do Estado do Piaui	Instituto Adolfo Lutz, Interdiciplinary Procedures Center, Strategic Laboratory	Caio Vinicius Dias Lopes; Claudia Regina Gonçalves; Claudio Tavares Sacchi; Karoline Rodrigues Campos; Leonardo Tadeu de Araujo; Marlon Benedito Nascomento Santos
EPI_ISL_2958858, EPI_ISL_2958859, EPI_ISL_2958860, EPI_ISL_2958861, EPI_ISL_2958862, EPI_ISL_2958863, EPI_ISL_2958864, EPI_ISL_2958866, EPI_ISL_2958867, EPI_ISL_2958868, EPI_ISL_2958869, EPI_ISL_2958870, EPI_ISL_2958871, EPI_ISL_2958873, EPI_ISL_2958874, EPI_ISL_2958875, EPI_ISL_2958876, EPI_ISL_2958877, EPI_ISL_2958878, EPI_ISL_2958879			
see above	LACEN do Mato Grosso do Sul	Instituto Adolfo Lutz, Interdiciplinary Procedures Center, Strategic Laboratory	Caio Vinicius Dias Lopes; Claudia Regina Gonçalves; Claudio Tavares Sacchi; Erica Valessa Ramos Gomes; Karoline Rodrigues Campos
EPI_ISL_3835228	LACEN/PB	WallauLab on behalf of Fiocruz COVID-19 Genomic Surveillance Network	Alexandre Freitas da Silva; Cassia Docena; Constância Flávia Junqueira Ayres; Filipe Zimmer Dezordi; Gabriel Luz Wallau; Gustavo Barbosa de Lima; Lais Ceschini Machado; Lilian Caroliny Amorim Silva; Marcelo Henrique dos Santos Paiva; Matheus Filgueira Bezerra; Sinval Pinto Brandão Filho
EPI_ISL_2821274, EPI_ISL_2821275, EPI_ISL_3134550, EPI_ISL_3134551, EPI_ISL_3134552, EPI_ISL_3134554, EPI_ISL_3134555, EPI_ISL_3134556, EPI_ISL_3134557, EPI_ISL_3134558, EPI_ISL_3134559, EPI_ISL_3134560, EPI_ISL_3134561, EPI_ISL_3134562, EPI_ISL_3134563, EPI_ISL_3134564, EPI_ISL_3134565, EPI_ISL_3134566, EPI_ISL_3134567, EPI_ISL_3134568, EPI_ISL_3134569, EPI_ISL_3134570, EPI_ISL_3134571, EPI_ISL_3134572, EPI_ISL_3134573, EPI_ISL_3134574, EPI_ISL_3134575, EPI_ISL_3134576, EPI_ISL_3134577, EPI_ISL_3134578, EPI_ISL_3134580, EPI_ISL_3134581, EPI_ISL_3134582, EPI_ISL_3134583, EPI_ISL_3134584, EPI_ISL_3134585, EPI_ISL_3134586, EPI_ISL_3134587, EPI_ISL_3134588, EPI_ISL_3134590, EPI_ISL_3134591, EPI_ISL_3134592, EPI_ISL_3134593, EPI_ISL_3134594, EPI_ISL_3134596, EPI_ISL_3134597, EPI_ISL_3134598, EPI_ISL_3134599, EPI_ISL_3134600, EPI_ISL_3134601, EPI_ISL_3134602, EPI_ISL_3134603, EPI_ISL_3134604, EPI_ISL_3134605, EPI_ISL_3134606, EPI_ISL_3134607, EPI_ISL_3134608, EPI_ISL_3134610, EPI_ISL_3134611, EPI_ISL_3134612, EPI_ISL_3134613, EPI_ISL_3134614, EPI_ISL_3134615, EPI_ISL_3134616, EPI_ISL_3134617, EPI_ISL_3134618, EPI_ISL_3134619, EPI_ISL_3134620, EPI_ISL_3134621, EPI_ISL_3134622, EPI_ISL_3134623, EPI_ISL_3134624, EPI_ISL_3134625, EPI_ISL_3134626, EPI_ISL_3134627, EPI_ISL_3134628, EPI_ISL_3134629, EPI_ISL_3134630, EPI_ISL_3134632, EPI_ISL_3134633, EPI_ISL_3134634, EPI_ISL_3134635, EPI_ISL_3134637, EPI_ISL_3134638, EPI_ISL_3134639, EPI_ISL_3134640, EPI_ISL_3134641, EPI_ISL_3134643, EPI_ISL_3134645, EPI_ISL_3134646, EPI_ISL_3134647, EPI_ISL_3134648, EPI_ISL_3134649, EPI_ISL_3134650, EPI_ISL_3134652, EPI_ISL_3134653, EPI_ISL_3134654, EPI_ISL_3134655, EPI_ISL_3134656, EPI_ISL_3134657, EPI_ISL_3134658, EPI_ISL_3134659, EPI_ISL_3134660, EPI_ISL_3134661, EPI_ISL_3134662, EPI_ISL_3134663, EPI_ISL_3134836, EPI_ISL_3134837, EPI_ISL_3134838, EPI_ISL_3134840, EPI_ISL_3134841, EPI_ISL_3134842, EPI_ISL_3134845, EPI_ISL_3134846, EPI_ISL_3134848, EPI_ISL_3134849, EPI_ISL_3134850, EPI_ISL_3134851, EPI_ISL_3134852, EPI_ISL_3134856, EPI_ISL_3134857, EPI_ISL_3134859, EPI_ISL_3152237, EPI_ISL_3447589, EPI_ISL_3447590, EPI_ISL_3447591, EPI_ISL_3703158, EPI_ISL_3703169, EPI_ISL_3703170, EPI_ISL_3703171, EPI_ISL_3703172, EPI_ISL_3703173, EPI_ISL_3703174, EPI_ISL_3703175, EPI_ISL_3703176, EPI_ISL_3703177, EPI_ISL_3703178, EPI_ISL_3703179, EPI_ISL_3703180, EPI_ISL_3703181, EPI_ISL_3703182, EPI_ISL_3703183, EPI_ISL_3703184, EPI_ISL_3703185, EPI_ISL_3703188, EPI_ISL_3703197, EPI_ISL_3703207, EPI_ISL_3703216, EPI_ISL_3703226, EPI_ISL_3703236, EPI_ISL_3703246, EPI_ISL_3703255, EPI_ISL_3703265, EPI_ISL_3703275, EPI_ISL_3703285, EPI_ISL_3703295, EPI_ISL_3703306, EPI_ISL_3703316, EPI_ISL_3703334, EPI_ISL_3703344, EPI_ISL_3703353, EPI_ISL_3703362, EPI_ISL_3703370, EPI_ISL_3703374, EPI_ISL_3703385, EPI_ISL_3703396, EPI_ISL_3703399, EPI_ISL_3703403, EPI_ISL_3703407, EPI_ISL_3703417, EPI_ISL_3703421, EPI_ISL_3703424, EPI_ISL_3703428, EPI_ISL_3703432, EPI_ISL_3703436, EPI_ISL_3703451, EPI_ISL_3703461, EPI_ISL_3703471, EPI_ISL_3703475, EPI_ISL_3703479, EPI_ISL_3703490, EPI_ISL_3703498, EPI_ISL_3703508, EPI_ISL_3703511, EPI_ISL_3703515, EPI_ISL_3703519, EPI_ISL_3703523, EPI_ISL_3703527, EPI_ISL_3703540, EPI_ISL_3703544, EPI_ISL_3703550, EPI_ISL_3703558, EPI_ISL_3703569, EPI_ISL_3703572, EPI_ISL_3703575, EPI_ISL_3703580, EPI_ISL_3703584, EPI_ISL_3703588, EPI_ISL_3703600, EPI_ISL_3703603, EPI_ISL_3703607, EPI_ISL_3703612, EPI_ISL_3703621, EPI_ISL_3703635, EPI_ISL_3703638, EPI_ISL_3703642, EPI_ISL_3703646, EPI_ISL_3703650, EPI_ISL_3703654, EPI_ISL_3703657, EPI_ISL_3703661, EPI_ISL_3703665			
see above	LACEN/PE	WallauLab on behalf of Fiocruz COVID-19 Genomic Surveillance Network	Alexandre Freitas da Silva; Antonio Mauro Rezende; Cassia Docena; Constância Flávia Junqueira Ayres; Cássia Docena; Duschinka Ribeiro Duarte Guedes; Elisama Helvecio; Filipe Zimmer Dezordi; Gabriel Luz Wallau; Gustavo Barbosa de Lima; Lais Ceschini Machado; Larissa Krokovsky; Laís Ceschini Machado; Lilian Caroliny Amorim Silva; Marcelo Henrique dos Santos Paiva; Matheus Filgueira Bezerra; Sinval Pinto Brandão Filho
EPI_ISL_3010762, EPI_ISL_3010765, EPI_ISL_3010770, EPI_ISL_3010771, EPI_ISL_3010773, EPI_ISL_3010774, EPI_ISL_3010778, EPI_ISL_3010779, EPI_ISL_3010780, EPI_ISL_3010785, EPI_ISL_3010787, EPI_ISL_3010788, EPI_ISL_3010789, EPI_ISL_3010790, EPI_ISL_3010792, EPI_ISL_3010795, EPI_ISL_3010796, EPI_ISL_3010797, EPI_ISL_3010812, EPI_ISL_3010813, EPI_ISL_3010815, EPI_ISL_3010821, EPI_ISL_3010822, EPI_ISL_3010829, EPI_ISL_3010834, EPI_ISL_3010838, EPI_ISL_3010844, EPI_ISL_3010850, EPI_ISL_3010855, EPI_ISL_3010857, EPI_ISL_3010858, EPI_ISL_3010872, EPI_ISL_3010873, EPI_ISL_3010878, EPI_ISL_3010880, EPI_ISL_3010882, EPI_ISL_3010883, EPI_ISL_3010884, EPI_ISL_3010887, EPI_ISL_3010908, EPI_ISL_3010916, EPI_ISL_3010919, EPI_ISL_3010927, EPI_ISL_3010929, EPI_ISL_3010930, EPI_ISL_3261103			
see above	LATE - Laboratório de Técnicas Especiais - Hospital Israelita Albert Einstein	LATE - Laboratório de Técnicas Especiais - Hospital Israelita Albert Einstein	Alexandre Hideaki Takara; Ana Paula Moreira Salles; Anelisie da Silva Santos; Deyvid Amgarten; Erick Gustavo Dorlass; Fernanda de Mello Malta; João Renato Rebello Pinho; Marcio Anunciacao Menezes; Pedro Henrique Sebe Rodrigues; Raquel Riyuzo
EPI_ISL_2483064	LBM CBM Le Chesnay	Eurofins Biomnis	Véronique Jacomo; Xavier Naudot
EPI_ISL_2373664	LBM UNIBIO VALENTIN	CNR Virus des Infections Respiratoires - France SUD	Antonin Bal; Bruno Lina; Gregory Destras; Gwendolyne Burfin; Hadrien Regue; Laurence Josset; Martine Valette; Quentin Semanas
EPI_ISL_2296086, EPI_ISL_2476439	LESP Baja California	Instituto de Diagnostico y Referencia Epidemiologicos (INDRE)	Abril Rodriguez-Maldonado; Ariadna Medina-Benitez; Claudia Wong-Arambula; Ernesto Ramirez-Gonzalez.; Gisela Barrera-Badillo; Irma Lopez-Martinez; Joaquin Quiroz-Mercado; Lucia Hernandez-Rivas; Natividad Cruz-Ortiz; Sergio Rangel-Guerrero; Tatiana Nunez-Garcia; Vanessa Rivero-Arredondo
EPI_ISL_2296085	LESP Chiapas	Instituto de Diagnostico y Referencia Epidemiologicos (INDRE)	Abril Rodriguez-Maldonado; Ariadna Medina-Benitez; Claudia Wong-Arambula; Ernesto Ramirez-Gonzalez.; Gisela Barrera-Badillo; Irma Lopez-Martinez; Joaquin Quiroz-Mercado; Lucia Hernandez-Rivas; Natividad Cruz-Ortiz; Sergio Rangel-Guerrero; Tatiana Nunez-Garcia; Vanessa Rivero-Arredondo
EPI_ISL_2105694, EPI_ISL_2105716, EPI_ISL_2105717, EPI_ISL_2105718, EPI_ISL_2296060, EPI_ISL_2296074, EPI_ISL_2296075, EPI_ISL_2296078, EPI_ISL_2476409, EPI_ISL_2476410, EPI_ISL_2476411, EPI_ISL_2476412, EPI_ISL_2476427, EPI_ISL_2476428, EPI_ISL_2476429, EPI_ISL_2545695, EPI_ISL_2545696, EPI_ISL_2545697			
see above	LESP Ciudad de Mexico	Instituto de Diagnostico y Referencia Epidemiologicos (INDRE)	Abril Rodriguez-Maldonado; Ariadna Medina-Benitez; Claudia Wong-Arambula; Ernesto Ramirez-Gonzalez.; Gisela Barrera-Badillo; Irma Lopez-Martinez; Joaquin Quiroz-Mercado; Lucia Hernandez-Rivas; Natividad Cruz-Ortiz; Sergio Rangel-Guerrero; Tatiana Nunez-Garcia; Vanessa Rivero-Arredondo
EPI_ISL_2476438	LESP Colima	Instituto de Diagnostico y Referencia Epidemiologicos (INDRE)	Abril Rodriguez-Maldonado; Ariadna Medina-Benitez; Claudia Wong-Arambula; Ernesto Ramirez-Gonzalez.; Gisela Barrera-Badillo; Irma Lopez-Martinez; Joaquin Quiroz-Mercado; Lucia Hernandez-Rivas; Natividad Cruz-Ortiz; Sergio Rangel-Guerrero; Tatiana Nunez-Garcia; Vanessa Rivero-Arredondo
EPI_ISL_2296073, EPI_ISL_2545698	LESP Estado de Mexico	Instituto de Diagnostico y Referencia Epidemiologicos (INDRE)	Abril Rodriguez-Maldonado; Ariadna Medina-Benitez; Claudia Wong-Arambula; Ernesto Ramirez-Gonzalez.; Gisela Barrera-Badillo; Irma Lopez-Martinez; Joaquin Quiroz-Mercado; Lucia Hernandez-Rivas; Natividad Cruz-Ortiz; Sergio Rangel-Guerrero; Tatiana Nunez-Garcia; Vanessa Rivero-Arredondo
EPI_ISL_2105686, EPI_ISL_2105687, EPI_ISL_2296093, EPI_ISL_2476415, EPI_ISL_2476418, EPI_ISL_2476419, EPI_ISL_2476420, EPI_ISL_2476421, EPI_ISL_2476422, EPI_ISL_2476424, EPI_ISL_2476426, EPI_ISL_2674656			
see above	LESP Guanajuato	Instituto de Diagnostico y Referencia Epidemiologicos (INDRE)	Abril Rodriguez-Maldonado; Ariadna Medina-Benitez; Claudia Wong-Arambula; Ernesto Ramirez-Gonzalez.; Gisela Barrera-Badillo; Irma Lopez-Martinez; Joaquin Quiroz-Mercado; Lucia Hernandez-Rivas; Natividad Cruz-Ortiz; Sergio Rangel-Guerrero; Tatiana Nunez-Garcia; Vanessa Rivero-Arredondo
EPI_ISL_2105715, EPI_ISL_2296070	LESP Jalisco	Instituto de Diagnostico y Referencia Epidemiologicos (INDRE)	Abril Rodriguez-Maldonado; Ariadna Medina-Benitez; Claudia Wong-Arambula; Ernesto Ramirez-Gonzalez.; Gisela Barrera-Badillo; Irma Lopez-Martinez; Joaquin Quiroz-Mercado; Lucia Hernandez-Rivas; Natividad Cruz-Ortiz; Sergio Rangel-Guerrero; Tatiana Nunez-Garcia; Vanessa Rivero-Arredondo
EPI_ISL_2296094, EPI_ISL_2476440	LESP Morelos	Instituto de Diagnostico y Referencia Epidemiologicos (INDRE)	Abril Rodriguez-Maldonado; Ariadna Medina-Benitez; Claudia Wong-Arambula; Ernesto Ramirez-Gonzalez.; Gisela Barrera-Badillo; Irma Lopez-Martinez; Joaquin Quiroz-Mercado; Lucia Hernandez-Rivas; Natividad Cruz-Ortiz; Sergio Rangel-Guerrero; Tatiana Nunez-Garcia; Vanessa Rivero-Arredondo
EPI_ISL_2296068	LESP Nuevo Leon	Instituto de Diagnostico y Referencia Epidemiologicos (INDRE)	Abril Rodriguez-Maldonado; Ariadna Medina-Benitez; Claudia Wong-Arambula; Ernesto Ramirez-Gonzalez.; Gisela Barrera-Badillo; Irma Lopez-Martinez; Joaquin Quiroz-Mercado; Lucia Hernandez-Rivas; Natividad Cruz-Ortiz; Sergio Rangel-Guerrero; Tatiana Nunez-Garcia; Vanessa Rivero-Arredondo
EPI_ISL_2476430	LESP Oaxaca	Instituto de Diagnostico y Referencia Epidemiologicos (INDRE)	Abril Rodriguez-Maldonado; Ariadna Medina-Benitez; Claudia Wong-Arambula; Ernesto Ramirez-Gonzalez.; Gisela Barrera-Badillo; Irma Lopez-Martinez; Joaquin Quiroz-Mercado; Lucia Hernandez-Rivas; Natividad Cruz-Ortiz; Sergio Rangel-Guerrero; Tatiana Nunez-Garcia; Vanessa Rivero-Arredondo
EPI_ISL_2545682	LESP Queretaro	Instituto de Diagnostico y Referencia Epidemiologicos (INDRE)	Abril Rodriguez-Maldonado; Ariadna Medina-Benitez; Claudia Wong-Arambula; Ernesto Ramirez-Gonzalez.; Gisela Barrera-Badillo; Irma Lopez-Martinez; Joaquin Quiroz-Mercado; Lucia Hernandez-Rivas; Natividad Cruz-Ortiz; Sergio Rangel-Guerrero; Tatiana Nunez-Garcia; Vanessa Rivero-Arredondo
EPI_ISL_2296087	LESP Quintana Roo	Instituto de Diagnostico y Referencia Epidemiologicos (INDRE)	Abril Rodriguez-Maldonado; Ariadna Medina-Benitez; Claudia Wong-Arambula; Ernesto Ramirez-Gonzalez.; Gisela Barrera-Badillo; Irma Lopez-Martinez; Joaquin Quiroz-Mercado; Lucia Hernandez-Rivas; Natividad Cruz-Ortiz; Sergio Rangel-Guerrero; Tatiana Nunez-Garcia; Vanessa Rivero-Arredondo
EPI_ISL_2476437	LESP Sinaloa	Instituto de Diagnostico y Referencia Epidemiologicos (INDRE)	Abril Rodriguez-Maldonado; Ariadna Medina-Benitez; Claudia Wong-Arambula; Ernesto Ramirez-Gonzalez.; Gisela Barrera-Badillo; Irma Lopez-Martinez; Joaquin Quiroz-Mercado; Lucia Hernandez-Rivas; Natividad Cruz-Ortiz; Sergio Rangel-Guerrero; Tatiana Nunez-Garcia; Vanessa Rivero-Arredondo
EPI_ISL_2296065, EPI_ISL_2476416, EPI_ISL_2476425, EPI_ISL_2476431, EPI_ISL_2476432, EPI_ISL_2476433, EPI_ISL_2476434, EPI_ISL_2476435, EPI_ISL_2476436, EPI_ISL_2545680, EPI_ISL_2545681, EPI_ISL_2545690, EPI_ISL_2545691			
see above	LESP Tamaulipas	Instituto de Diagnostico y Referencia Epidemiologicos (INDRE)	Abril Rodriguez-Maldonado; Ariadna Medina-Benitez; Claudia Wong-Arambula; Ernesto Ramirez-Gonzalez.; Gisela Barrera-Badillo; Irma Lopez-Martinez; Joaquin Quiroz-Mercado; Lucia Hernandez-Rivas; Natividad Cruz-Ortiz; Sergio Rangel-Guerrero; Tatiana Nunez-Garcia; Vanessa Rivero-Arredondo
EPI_ISL_2476414, EPI_ISL_2545684, EPI_ISL_2545685, EPI_ISL_2545686, EPI_ISL_2545687, EPI_ISL_2545688	LESP Yucatan	Instituto de Diagnostico y Referencia Epidemiologicos (INDRE)	Abril Rodriguez-Maldonado; Ariadna Medina-Benitez; Claudia Wong-Arambula; Ernesto Ramirez-Gonzalez.; Gisela Barrera-Badillo; Irma Lopez-Martinez; Joaquin Quiroz-Mercado; Lucia Hernandez-Rivas; Natividad Cruz-Ortiz; Sergio Rangel-Guerrero; Tatiana Nunez-Garcia; Vanessa Rivero-Arredondo

EPI_ISL_2476417	LESP Zacatecas	Instituto de Diagnostico y Referencia Epidemiologicos (INDRE)	Abril Rodriguez-Maldonado; Ariadna Medina-Benitez; Claudia Wong-Arambula; Ernesto Ramirez-Gonzalez.; Gisela Barrera-Badillo; Irma Lopez-Martinez; Joaquin Quiroz-Mercado; Lucia Hernandez-Rivas; Natividad Cruz-Ortiz; Sergio Rangel-Guerrero; Tatiana Nunez-Garcia; Vanessa Rivero-Arredondo
EPI_ISL_2029739	Labo Analyses Med	National Reference Center for Viruses of Respiratory Infections, Institut Pasteur, Paris	Angela Brisebarre; Arditi Cocco; Camille Capel; Christophe Malabat; Corinne Maufrais; Etienne Simon-Lorière; Frédéric Lemoine; Louise Lefrançois; Marion Barbet; Maud Vanpeene; Méline Bizard; Pierre Lechat; Sylvie Behillil; Sylvie Van der Werf; Vincent Enouf
EPI_ISL_2678400	Labor Becker & Kollegen (Standort München)	Robert Koch Institute	
EPI_ISL_2384910, EPI_ISL_2384917	Labor Dr. Fenner und Kollegen	Heinrich Pette Institute, Leibniz Institute for Experimental Virology	Adam Grundhoff; Alexis Robitaille; Johannes Knobloch; Martin Aepfelbacher; Nicole Fischer; Thomas Günther
EPI_ISL_2129701, EPI_ISL_2389341	Labor Dr. Heidrich & Kollegen MVZ GmbH Hamburg	Robert Koch Institute	
EPI_ISL_2129083	Labor Monchengladbach MVZ Dr. Stein + Kollegen GbR	Robert Koch Institute	
EPI_ISL_2125029	Labor Prof. Dr. G. Enders MVZ GbR	Robert Koch Institute	
EPI_ISL_2316147	Labor für medizinische Mikrobiologie Bakteriologie Mykologie Virologie & Infektionsserologie - Labor Mölbis	Robert Koch Institute	
EPI_ISL_2122501	Laborarztpraxis Osnabruck	Robert Koch Institute	
EPI_ISL_2227905, EPI_ISL_2362537, EPI_ISL_2362553, EPI_ISL_2426980	Laboratoire Carage	Institut Pasteur de la Guyane	A. Enfissi; A. Salmier; Anne Lavergne; Antoine Enfissi; Arielle Salmier; Dominique Rousset
EPI_ISL_2492500, EPI_ISL_2492502, EPI_ISL_2492503, EPI_ISL_2492506, EPI_ISL_2492507, EPI_ISL_2492509, EPI_ISL_2492510, EPI_ISL_2492512, EPI_ISL_2492513, EPI_ISL_2492515, EPI_ISL_2492517, EPI_ISL_2492518, EPI_ISL_2492519, EPI_ISL_2492520, EPI_ISL_2492521, EPI_ISL_2492522, EPI_ISL_2492523, EPI_ISL_2492524, EPI_ISL_2492525, EPI_ISL_2492526, EPI_ISL_2492527, EPI_ISL_2492531, EPI_ISL_2492533, EPI_ISL_2492534, EPI_ISL_2492536, EPI_ISL_2492538, EPI_ISL_2492539, EPI_ISL_2492540, EPI_ISL_2492541, EPI_ISL_2492543, EPI_ISL_2492544, EPI_ISL_2492546			
see above	Laboratoire National de Santé Publique – LNSP (HAITI - LNSP)	Laboratory of Respiratory Viruses and Measles, Oswaldo Cruz Institute, FIOCRUZ	Alice Sampaio Rocha; Ana Carolina Mendonca; Anna Carolina Paixao; Elisa Cavalcante Pereira; Fernando Motta; Ito Journel; Jaques Boncy; Luciana Appolinario; Marilda Siqueira on behalf of the Fiocruz COVID-19 Genomic Surveillance Network; Paola Resende; Patrick Delly; Renata Serrano Lopes; Taina Venas
EPI_ISL_3458621	Laboratoire de santé publique du Québec	Laboratoire de santé publique du Québec	Guillaume Bourque; Ioannis Ragoussis; Jesse Shapiro; Mark Lathrop and Michel Roger on behalf of the CoVSeQ research group; Sandrine Moreira
EPI_ISL_2401051, EPI_ISL_2401055, EPI_ISL_2401076, EPI_ISL_2401143, EPI_ISL_2401159, EPI_ISL_2401180, EPI_ISL_2401181, EPI_ISL_2401183			
see above	Laboratoire national de sante, Microbiology, Virology	Laboratoire national de sante, Microbiology, Microbial Genomics Platform	Anke Wienecke-Baldacchino; Catherine Ragimbeau; Fatu Djabi; Jessica Tapp; Lise Pignon; Raoul Salmon; Tamir Abdelrahman; Trung Nguyen Nguyen
EPI_ISL_2400867	Laboratoires Reunis	Laboratoire national de sante, Microbiology, Microbial Genomics Platform	Anke Wienecke-Baldacchino; Bernard Weber; Catherine Ragimbeau; Fatu Djabi; Jessica Tapp; Lise Pignon; Raoul Salmon; Tamir Abdelrahman
EPI_ISL_2400675, EPI_ISL_2400677, EPI_ISL_2400715, EPI_ISL_2400731, EPI_ISL_2400775, EPI_ISL_3148148, EPI_ISL_3148149			
see above	Laboratoires d'analyses medicales - Ketterthill	Laboratoire national de sante, Microbiology, Microbial Genomics Platform	Anke Wienecke-Baldacchino; Caroline Scheiber; Catherine Ragimbeau; Elodie Solarino; Fatu Djabi; Jessica Tapp; Lise Pignon; Raoul Salmon; Serge Vedy; Tamir Abdelrahman; Virginie Jover
EPI_ISL_2497876, EPI_ISL_2497877, EPI_ISL_2626433	Laboratori de Referencia de Catalunya	Laboratori de Referencia de Catalunya	Bellosillo B.; Canal M.; Hernandez JJ.; Padilla E.; Ramirez A.; Vilas A.
EPI_ISL_2983179, EPI_ISL_3190350	Laboratorio Central de Saude Publica do Estado de Goias (LACEN/GO)	Laboratory of Respiratory Viruses and Measles, Oswaldo Cruz Institute, FIOCRUZ	Agatha Cristinne Prudencio; Alice Sampaio Rocha; Ana Carolina Mendonca; Ana Flavia Mendonça; Anna Carolina Paixao; Carmen Helena Ramos; Cassiane Casanova; Elisa Cavalcante Pereira; Fernando Motta; Flavia Pereira Amorim da Silva; Ighor Leonardo Arantes Gomes; Luciana Appolinario; Luiz Augusto Pereira; Marilda Siqueira on behalf of the Fiocruz COVID-19 Genomic Surveillance Network; Paola Resende; Rafael Souza Guedes; Renata Serrano Lopes; Taina Venas; Vinicius Lemes da Silva
EPI_ISL_2196304, EPI_ISL_2196305, EPI_ISL_2443561, EPI_ISL_2443562, EPI_ISL_2645421, EPI_ISL_2645422, EPI_ISL_2645424, EPI_ISL_2645425, EPI_ISL_2645426, EPI_ISL_2645428, EPI_ISL_2645429, EPI_ISL_2645430, EPI_ISL_2645431, EPI_ISL_2645432, EPI_ISL_2645433, EPI_ISL_2645437, EPI_ISL_2645438, EPI_ISL_2645439, EPI_ISL_4080767			
see above	Laboratorio Central de Saude Publica do Estado do Para (LACEN/PA)	Laboratory of Respiratory Viruses and Measles, Oswaldo Cruz Institute, FIOCRUZ	Agatha Soares; Alice Sampaio Rocha; Ana Carolina Mendonca; Anna Carolina Paixao; Elisa Cavalcante Pereira; Fernando Motta; Ighor Arantes; Luciana Appolinario; Marilda Siqueira on behalf of the Fiocruz COVID-19 Genomic Surveillance Network; Paola Resende; Renata Serrano Lopes; Taina Venas; Valnete Andrade
EPI_ISL_1923562, EPI_ISL_2047600, EPI_ISL_2156063, EPI_ISL_2156081, EPI_ISL_2340131	Laboratorio Aziendale di Microbiologia e Virologia, Azienda Sanitaria dell'Alto Adige	Laboratorio Aziendale di Microbiologia e Virologia, Azienda Sanitaria dell'Alto Adige	Anne Picard; Bartolomeo Mobilio Rodriguez; Chiara Cantaloni; Claudia Volpato; Elisa Masi; Elisabetta Giacobazzi; Elisabetta Pagani; Irene Bianconi; Stefanie Wieser
EPI_ISL_2101403, EPI_ISL_2101404, EPI_ISL_2101405, EPI_ISL_2101406, EPI_ISL_2101409, EPI_ISL_2101411, EPI_ISL_2101412, EPI_ISL_2101413, EPI_ISL_2101414, EPI_ISL_2101415, EPI_ISL_2101416, EPI_ISL_2101417, EPI_ISL_2101418, EPI_ISL_2101419, EPI_ISL_2101420, EPI_ISL_2101421, EPI_ISL_2101422, EPI_ISL_2101423, EPI_ISL_2101425, EPI_ISL_2101426, EPI_ISL_2101434, EPI_ISL_2101435, EPI_ISL_2101436, EPI_ISL_2101437, EPI_ISL_2101438, EPI_ISL_2101439, EPI_ISL_2101440, EPI_ISL_2101441, EPI_ISL_2101442, EPI_ISL_2101445, EPI_ISL_2101446, EPI_ISL_2101447, EPI_ISL_2101448, EPI_ISL_2101449, EPI_ISL_2101451, EPI_ISL_2101452, EPI_ISL_2101453, EPI_ISL_2101464, EPI_ISL_2101466, EPI_ISL_2101467, EPI_ISL_2101478, EPI_ISL_2101479, EPI_ISL_2101480, EPI_ISL_2101481, EPI_ISL_2101482, EPI_ISL_2101489, EPI_ISL_2101490, EPI_ISL_2101491, EPI_ISL_2101492, EPI_ISL_2101493, EPI_ISL_2101500, EPI_ISL_2101501, EPI_ISL_2101502, EPI_ISL_2101503, EPI_ISL_2101504, EPI_ISL_2101507, EPI_ISL_2101514, EPI_ISL_2101515, EPI_ISL_2101516, EPI_ISL_2101517, EPI_ISL_2101518, EPI_ISL_2101519, EPI_ISL_2101528, EPI_ISL_2101529, EPI_ISL_2101530, EPI_ISL_2101531, EPI_ISL_2101532, EPI_ISL_2101533, EPI_ISL_2101534, EPI_ISL_2101535, EPI_ISL_2101536, EPI_ISL_2101537, EPI_ISL_2101538, EPI_ISL_2101539, EPI_ISL_2101540, EPI_ISL_2101544, EPI_ISL_2101545, EPI_ISL_2101548, EPI_ISL_2101563, EPI_ISL_2101564, EPI_ISL_2101565, EPI_ISL_2101573, EPI_ISL_2101574, EPI_ISL_2101575, EPI_ISL_2101576, EPI_ISL_2101577, EPI_ISL_2101578, EPI_ISL_2101579, EPI_ISL_2101580, EPI_ISL_2101581, EPI_ISL_2101582, EPI_ISL_2101584, EPI_ISL_2101585, EPI_ISL_2101586, EPI_ISL_2101590, EPI_ISL_2101592, EPI_ISL_2101594, EPI_ISL_2101596, EPI_ISL_2101597, EPI_ISL_2101598, EPI_ISL_2101599, EPI_ISL_2101600, EPI_ISL_2101601, EPI_ISL_2101603, EPI_ISL_2101604, EPI_ISL_2101605, EPI_ISL_2101606, EPI_ISL_2101608, EPI_ISL_2101609, EPI_ISL_2101610, EPI_ISL_2101611, EPI_ISL_2101613, EPI_ISL_2101615, EPI_ISL_2101616, EPI_ISL_2101617, EPI_ISL_2101618, EPI_ISL_2101619, EPI_ISL_2101620, EPI_ISL_2101622, EPI_ISL_2101623, EPI_ISL_2101624, EPI_ISL_2101625, EPI_ISL_2101626, EPI_ISL_2101627, EPI_ISL_2101628, EPI_ISL_2101629, EPI_ISL_2101630, EPI_ISL_2101631, EPI_ISL_2101632, EPI_ISL_2101633, EPI_ISL_2101634, EPI_ISL_2101635, EPI_ISL_2101636, EPI_ISL_2101637, EPI_ISL_2101672, EPI_ISL_2101674, EPI_ISL_2101675, EPI_ISL_2101690, EPI_ISL_2101691, EPI_ISL_2101697, EPI_ISL_2101698, EPI_ISL_2101699, EPI_ISL_2101700, EPI_ISL_2101701, EPI_ISL_2101702, EPI_ISL_2101703, EPI_ISL_2101704, EPI_ISL_2101705, EPI_ISL_2101706, EPI_ISL_2101707, EPI_ISL_2101708, EPI_ISL_2101709, EPI_ISL_2101710, EPI_ISL_2101711, EPI_ISL_2101712, EPI_ISL_2101713, EPI_ISL_2101716, EPI_ISL_2101717, EPI_ISL_2101718, EPI_ISL_2101719, EPI_ISL_2101725, EPI_ISL_2101730, EPI_ISL_2101732, EPI_ISL_2101733, EPI_ISL_2101736, EPI_ISL_2101737, EPI_ISL_2101738, EPI_ISL_2101745, EPI_ISL_2385432, EPI_ISL_2385433, EPI_ISL_2385434, EPI_ISL_2385435, EPI_ISL_2385436, EPI_ISL_2385437, EPI_ISL_2385438, EPI_ISL_2385440, EPI_ISL_2385441, EPI_ISL_2385442, EPI_ISL_2385443, EPI_ISL_2385444, EPI_ISL_2385445, EPI_ISL_2385446, EPI_ISL_2385447, EPI_ISL_2385448, EPI_ISL_2385449, EPI_ISL_2385450, EPI_ISL_2385451, EPI_ISL_2385452, EPI_ISL_2385453, EPI_ISL_2385454, EPI_ISL_2385455, EPI_ISL_2385456, EPI_ISL_2385457, EPI_ISL_2385458, EPI_ISL_2385459, EPI_ISL_2385460, EPI_ISL_2385461, EPI_ISL_2385462, EPI_ISL_2385463, EPI_ISL_2385464, EPI_ISL_2385465, EPI_ISL_2385466, EPI_ISL_2385467, EPI_ISL_2385468, EPI_ISL_2385469, EPI_ISL_2385470, EPI_ISL_2385471, EPI_ISL_2385472, EPI_ISL_2385474, EPI_ISL_2385475, EPI_ISL_2385476, EPI_ISL_2385477, EPI_ISL_2385478, EPI_ISL_2385480, EPI_ISL_2385482, EPI_ISL_2385483, EPI_ISL_2385484, EPI_ISL_2385485, EPI_ISL_2385486, EPI_ISL_2385487, EPI_ISL_2385488, EPI_ISL_2385489, EPI_ISL_2385490, EPI_ISL_2385491, EPI_ISL_2385492, EPI_ISL_2385493, EPI_ISL_2385496, EPI_ISL_2385498, EPI_ISL_2385499, EPI_ISL_2385500, EPI_ISL_2385501, EPI_ISL_2385504, EPI_ISL_2385505, EPI_ISL_2385506, EPI_ISL_2385507, EPI_ISL_2385508, EPI_ISL_2385510, EPI_ISL_2385511, EPI_ISL_2385512, EPI_ISL_2385513, EPI_ISL_2385514, EPI_ISL_2385515, EPI_ISL_2385516, EPI_ISL_2385517, EPI_ISL_2385518, EPI_ISL_2385519, EPI_ISL_2385521, EPI_ISL_2385522, EPI_ISL_2385523, EPI_ISL_2385527, EPI_ISL_2385528, EPI_ISL_2385530, EPI_ISL_2385531, EPI_ISL_2385532, EPI_ISL_2385533, EPI_ISL_2385534, EPI_ISL_2385535, EPI_ISL_2385536, EPI_ISL_2385537, EPI_ISL_2385538, EPI_ISL_2385539, EPI_ISL_2385541, EPI_ISL_2385542, EPI_ISL_2385543, EPI_ISL_2385544, EPI_ISL_2385545, EPI_ISL_2385546, EPI_ISL_2385547, EPI_ISL_2385548, EPI_ISL_2385549, EPI_ISL_2385550, EPI_ISL_2385551, EPI_ISL_2385552, EPI_ISL_2385553, EPI_ISL_2385554, EPI_ISL_2385558, EPI_ISL_2385559, EPI_ISL_2385561, EPI_ISL_2385562, EPI_ISL_2385563, EPI_ISL_2385567, EPI_ISL_2385568, EPI_ISL_2385570, EPI_ISL_2385571, EPI_ISL_2385572, EPI_ISL_2385573, EPI_ISL_2385574, EPI_ISL_2385575, EPI_ISL_2385576, EPI_ISL_2385577, EPI_ISL_2385578, EPI_ISL_2385579, EPI_ISL_2385580, EPI_ISL_2385581, EPI_ISL_2385583, EPI_ISL_2385584, EPI_ISL_2385586, EPI_ISL_2385587, EPI_ISL_2385589, EPI_ISL_2385590, EPI_ISL_2385592, EPI_ISL_2385593, EPI_ISL_2385594, EPI_ISL_2385595, EPI_ISL_2385596, EPI_ISL_2385597, EPI_ISL_2385598, EPI_ISL_2385599, EPI_ISL_2385600, EPI_ISL_2385601, EPI_ISL_2385602, EPI_ISL_2385603, EPI_ISL_2385604, EPI_ISL_2385605, EPI_ISL_2385606, EPI_ISL_2385607, EPI_ISL_2385608, EPI_ISL_2385609, EPI_ISL_2385610, EPI_ISL_2385611, EPI_ISL_2385612, EPI_ISL_2385613, EPI_ISL_2385616, EPI_ISL_2385620, EPI_ISL_2385624, EPI_ISL_2385625, EPI_ISL_2385627, EPI_ISL_2385629, EPI_ISL_2385630, EPI_ISL_2385631, EPI_ISL_2385632, EPI_ISL_2385633, EPI_ISL_2385634, EPI_ISL_2385636, EPI_ISL_2385637, EPI_ISL_2385639, EPI_ISL_2385641, EPI_ISL_2385642, EPI_ISL_2385644, EPI_ISL_2385647, EPI_ISL_2385648, EPI_ISL_2385649, EPI_ISL_2385651, EPI_ISL_2385652, EPI_ISL_2385654, EPI_ISL_2385655, EPI_ISL_2385656, EPI_ISL_2385657, EPI_ISL_2385658, EPI_ISL_2385659, EPI_ISL_2385660, EPI_ISL_2385661, EPI_ISL_2385662, EPI_ISL_2385663, EPI_ISL_2385664, EPI_ISL_2385665, EPI_ISL_2385666, EPI_ISL_2385667, EPI_ISL_2385668, EPI_ISL_2534862, EPI_ISL_2534863, EPI_ISL_2534865, EPI_ISL_2534866, EPI_ISL_2534868, EPI_ISL_2534869, EPI_ISL_2534870, EPI_ISL_2534871, EPI_ISL_2534872, EPI_ISL_2534873, EPI_ISL_2534874, EPI_ISL_2534875, EPI_ISL_2534876, EPI_ISL_2534877, EPI_ISL_2534878, EPI_ISL_2534879, EPI_ISL_2534880, EPI_ISL_2534883, EPI_ISL_2534884, EPI_ISL_2534885, EPI_ISL_2534886, EPI_ISL_2534887, EPI_ISL_2534888, EPI_ISL_2534889, EPI_ISL_2534890, EPI_ISL_2534891, EPI_ISL_2534892, EPI_ISL_2534893, EPI_ISL_2534894, EPI_ISL_2534896, EPI_ISL_2534897, EPI_ISL_2534898, EPI_ISL_2534899, EPI_ISL_2534900, EPI_ISL_2534901, EPI_ISL_2534902, EPI_ISL_2534903, EPI_ISL_2534904, EPI_ISL_2534905, EPI_ISL_2534906, EPI_ISL_2534907, EPI_ISL_2534908, EPI_ISL_2534909, EPI_ISL_2534910, EPI_ISL_2534911, EPI_ISL_2534912, EPI_ISL_2534913, EPI_ISL_2534914, EPI_ISL_2534915, EPI_ISL_2534916, EPI_ISL_2534917, EPI_ISL_2534918, EPI_ISL_2534919, EPI_ISL_2534920, EPI_ISL_2534921, EPI_ISL_2534922, EPI_ISL_2534925, EPI_ISL_2534927, EPI_ISL_2534928, EPI_ISL_2534929, EPI_ISL_2534930, EPI_ISL_2534931, EPI_ISL_2534933, EPI_ISL_2534934, EPI_ISL_2534935, EPI_ISL_2534936, EPI_ISL_2534938, EPI_ISL_2534939, EPI_ISL_2534941, EPI_ISL_2534942, EPI_ISL_2534944, EPI_ISL_2534946, EPI_ISL_2534947, EPI_ISL_2534948, EPI_ISL_2534949, EPI_ISL_2534950, EPI_ISL_2534951, EPI_ISL_2534952, EPI_ISL_2534955, EPI_ISL_2534956, EPI_ISL_2534957, EPI_ISL_2534959, EPI_ISL_2534960, EPI_ISL_2534961, EPI_ISL_2534962, EPI_ISL_2534963, EPI_ISL_2534964, EPI_ISL_2534965, EPI_ISL_2534966, EPI_ISL_2534967, EPI_ISL_2534968, EPI_ISL_2534971, EPI_ISL_2534973, EPI_ISL_2534974, EPI_ISL_2534975, EPI_ISL_2534976, EPI_ISL_2534977, EPI_ISL_2534978, EPI_ISL_2534979, EPI_ISL_2534980, EPI_ISL_2534981, EPI_ISL_2534982, EPI_ISL_2534983, EPI_ISL_2534984, EPI_ISL_2534985, EPI_ISL_2534986, EPI_ISL_2534987, EPI_ISL_2534988, EPI_ISL_2534989, EPI_ISL_2534990, EPI_ISL_2534991, EPI_ISL_2534992, EPI_ISL_2534993, EPI_ISL_2534994, EPI_ISL_2534996, EPI_ISL_2534997, EPI_ISL_2534999, EPI_ISL_2535000, EPI_ISL_2535001, EPI_ISL_2535002, EPI_ISL_2535003, EPI_ISL_2535004, EPI_ISL_2535005, EPI_ISL_2535006, EPI_ISL_2535007, EPI_ISL_2535009, EPI_ISL_2535010, EPI_ISL_2535011, EPI_ISL_2535012, EPI_ISL_2535014, EPI_ISL_2535015, EPI_ISL_2535017, EPI_ISL_2535021, EPI_ISL_2535022, EPI_ISL_2535023, EPI_ISL_2535024, EPI_ISL_2535025, EPI_ISL_2535027, EPI_ISL_2535028, EPI_ISL_2535029, EPI_ISL_2535030, EPI_ISL_2535031, EPI_ISL_2535032, EPI_ISL_2535033, EPI_ISL_2535034, EPI_ISL_2535035, EPI_ISL_2535036, EPI_ISL_2535039, EPI_ISL_2535040, EPI_ISL_2535041, EPI_ISL_2535042, EPI_ISL_2535043, EPI_ISL_2535044, EPI_ISL_2535045, EPI_ISL_2535047, EPI_ISL_2535048, EPI_ISL_2535049, EPI_ISL_2535050, EPI_ISL_2535051, EPI_ISL_2535052, EPI_ISL_2535053, EPI_ISL_2535054, EPI_ISL_2535055, EPI_ISL_2535056, EPI_ISL_2535058, EPI_ISL_2535059, EPI_ISL_2535060, EPI_ISL_2535061, EPI_ISL_2535062, EPI_ISL_2535063, EPI_ISL_2535064, EPI_ISL_2535065, EPI_ISL_2535066, EPI_ISL_2535067, EPI_ISL_2535068, EPI_ISL_2535069, EPI_ISL_2535070, EPI_ISL_2535072, EPI_ISL_2535073, EPI_ISL_2535074, EPI_ISL_2535075, EPI_ISL_2535076, EPI_ISL_2535077, EPI_ISL_2535080, EPI_ISL_2535081, EPI_ISL_2535082, EPI_ISL_2535083, EPI_ISL_2535084, EPI_ISL_2535085, EPI_ISL_2535089, EPI_ISL_2535090, EPI_ISL_2535091, EPI_ISL_2535092, EPI_ISL_2535093, EPI_ISL_2535094, EPI_ISL_2535095, EPI_ISL_2535096, EPI_ISL_2535097, EPI_ISL_2535098, EPI_ISL_2535099, EPI_ISL_2535100, EPI_ISL_2535102, EPI_ISL_2535106, EPI_ISL_2691353, EPI_ISL_2691354, EPI_ISL_2691355, EPI_ISL_2691356			
see above	Laboratorio Central Noel Nutels	Bioinformatics Laboratory / LNCC	Alessandra P Lamarca; Alexandra L Gerber; Amilcar Tanuri; Ana Paula de C Guimaraes; Ana Tereza R Vasconcelos; Andrea Cony Cavalcanti; Caio Luiz Pereira Ribeiro; Cassia Alves; Cintia Policarpo; Claudia Maria Braga de Mello; Cristiane Gomes da Silva; Diana Mariani; Douglas Terra Machado; Flavio Dias da Silva; Gleidson da Silva de Oliveira; Leandro Magalhaes de Souza; Liliane Cavalcante; Luiz G P de Almeida; Marcio Henrique de Oliveira Garcia; Mario Sergio Ribeiro; Ronaldo da Silva F Jr; Silvia Carvalho
EPI_ISL_2671535, EPI_ISL_2671637, EPI_ISL_2671645	Laboratorio Central de Epidemiologia (LCE)	Unidad de Genomica Avanzada	; Alejandra Garcia-Gasca; Alejandra Hernandez-Teran; Alejandro Sanchez-Flores; Alfredo Herrera-Estrella; Alicia Ocaña-Mondragon; Andreu Comas-Garcia; Angel Gustavo Salas-Lais; Antonio Loza Roman; Bernardo Martinez-Miguel; Blanca Taboada; Brenda Irasema Maldonado-Meza; Bruno Gomez-Gil; Carla Ivon Herrera-Najera; Carlos F. Arias; Celia Boukadida; Celida Duque Molina; Celida Martinez- Rodriguez; Clara Esperanza Santacruz-Tinoco; Concepcion Grajales-Muñiz; Consorcio Mexicano de Vigilancia Genomica (CoViGen-Mex). Authors (in alphabetical order): Julio Elias Alvarado-Yaah; Cristobal Chaidez-Quiroz; Daniel Fregoso-Rueda; Daniel Lira Morales; Eduardo Becerril-Vargas; Fernando Fontove-Herrera; Fidencio Mejia-Nepomuceno; Francisco Pulido; Gloria Elena Espinosa-Ayala; Gloria Maria Molina-Salinas; Gloria Vazquez; Hector Esteban Paz-Juarez; Hector Montoya-Fuentes; Helen Haydee Fernanda Ramirez-Plascencia; Irvin Gonzalez-Lopez; Jean Pierre Gonzalez; Jesus Hernandez; Joel Armando Vazquez-Perez.; Jorge Salas-Hernandez; Jose Antonio Enciso-Moreno; Jose Arturo Martinez-Orozco; Jose Esteban Muñoz-Medina; Jose de Jesus Nuñez-Contreras; Juan Bautista Chale-Dzul; Julissa Enciso-Ibarra; Luis Alberto Ochoa-Carrera; Margarita Matias-Florentino; Maria Guadalupe Santiago-Mauricio; Maria Guadalupe de Jesus Mireles-Rivera; Mario Mujica-Sanchez; Marissa Perez-Garcia; Nelly Selem-Mojica; Pavel Isa; Ricardo Ciria Merce; Ricardo Grande; Rosa Maria Gutierrez Rios; Santiago avila-Rios; Selene Zarate; Susana Lopez; Veronica Mata-Haro; Victor Eduardo Garcia-Arias; Victor Hugo Borja-Aburto
EPI_ISL_2490388, EPI_ISL_2490391	Laboratorio Central de Epidemiología (LCE)	Centro de Investigación en Enfermedades Infecciosas (CIENI), Instituto Nacional de Enfermedades Respiratorias (INER)	; Alejandra Garcia-Gasca; Alejandra Hernández-Terán; Alejandro Sanchez-Flores; Alfredo Herrera-Estrella; Alicia Ocaña-Mondragón; Andreu Comas-Garcia; Angel Gustavo Salas-Lais; Antonio Loza Román; Bernardo Martínez-Miguel; Blanca Taboada; Brenda Irasema Maldonado-Meza; Bruno Gomez-Gil; Carla Ivón Herrera-Najera; Carlos F. Arias; Celia Boukadida; Clara Esperanza Santacruz-Tinoco; Concepción Grajales-Muñiz; Consorcio Mexicano de Vigilancia Genómica (CoViGen-Mex). Authors (in alphabetical order): Julio Elias Alvarado-Yaah; Cristóbal Cháidez-Quiróz; Célida Duque Molina; Célida Martínez-Rodríguez; Daniel Fregoso-Rueda; Daniel Lira Morales; Eduardo Becerril-Vargas; Fernando Fontove-Herrera; Fidencio Mejía-Nepomuceno; Francisco Pulido; Gloria Elena Espinosa-Ayala; Gloria María Molina-Salinas; Gloria Vazquez; Hector Esteban Paz-Juárez; Hector Montoya-Fuentes; Helen Haydee Fernanda Ramírez-Plascencia; Irvin González-López; Jean Pierre González; Joel Armando Vázquez-Pérez.; Jorge Salas-Hernández; José Antonio Enciso-Moreno; José Arturo Martínez-Orozco; José Esteban Muñoz-Medina; José de Jesús Nuñez-Contreras; Juan Bautista Chale-Dzul; Julissa Enciso-Ibarra; Luis Alberto Ochoa-Carrera; Margarita Matías-Florentino; Mario Mújica-Sánchez; Marissa Perez-Garcia; María Guadalupe Santiago-Mauricio; María Guadalupe de Jesús Mireles-Rivera; Nelly Sélem-Mojica; Pavel Isa; Ricardo Ciria Merce; Ricardo Grande; Rosa María Gutierrez Rios; Santiago Ávila-Ríos; Selene Zárate; Susana Lopez; Victor Eduardo Garcia-Arias; Víctor Hugo Borja-Aburto
EPI_ISL_2391526,	Laboratorio Central de Epidemiología	Instituto de Biotecnología de la	; Alejandra Garcia-Gasca; Alejandra García-Gasca; Alejandra Hernández-Terán; Alejandro Sanchez-Flores; Alejandro Sánchez-Flores; Alfredo Herrera-Estrella; Alicia Ocaña-Mondragón; Andreu Comas-García; Angel Gustavo Salas-Lais; Antonio Loza Román; Bernardo Martínez-Miguel; Blanca Taboada; Brenda

EPI_ISL_2391549, EPI_ISL_2391609, EPI_ISL_2391610, EPI_ISL_2681081	(LCE)	UNAM	Irasema Maldonado-Meza; Bruno Gomez-Gil; Bruno Gómez-Gil; Carla Ivón Herrera-Najera; Carlos F. Arias; Celia Boukadida; Clara Esperanza Santacruz-Tinoco; Concepción Grajales-Muñiz; Consorcio Mexicano de Vigilancia Genómica (CoViGen-Mex). Authors (in alphabetical order): Julio Elias Alvarado-Yaah; Cristóbal Cháidez-Quiróz; Célida Duque Molina; Célida Martínez- Rodríguez; Daniel Fregoso-Rueda; Daniel Lira Morales; Eduardo Becerril-Vargas; Fernando Fontove-Herrera; Fidencio Mejía-Nepomuceno; Francisco Pulido; Gloria Elena Espinosa-Ayala; Gloria María Molina-Salinas; Gloria Vazquez; Hector Esteban Paz-Juárez; Hector Montoya-Fuentes; Helen Haydee Fernanda Ramírez-Plascencia; Irvin González-López; Jean Pierre González; Jesús Hernández; Joel Armando Vázquez-Pérez.; Jorge Salas-Hernández; José Antonio Enciso-Moreno; José Arturo Martínez-Orozco; José Esteban Muñoz-Medina; José de Jesús Nuñez-Contreras; Juan Bautista Chale-Dzul; Julissa Enciso-Ibarra; Luis Alberto Ochoa-Carrera; Margarita Matías-Florentino; Mario Mújica-Sánchez; Marissa Perez-Garcia; María Guadalupe Santiago-Mauricio; María Guadalupe de Jesús Mireles-Rivera; Nelly Sélem-Mojica; Pavel Isa; Ricardo Ciria Merce; Ricardo Grande; Rosa María Gutierrez Rios; Rosa María Gutiérrez Rios; Santiago Ávila-Ríos; Selene Zárate; Susana Lopez; Verónica Mata-Haro; Victor Eduardo Garcia-Arias; Victor Eduardo García-Arias; Víctor Hugo Borja-Aburto
EPI_ISL_2402216	Laboratorio Central de Epidemiología (LCE)	Unidad de Genomica Avanzada	Alejandro Sanchez-Flores; Alfredo Herrera-Estrella; Alicia Ocana-Mondragon; Angel Gustavo Salas-Lais; Bernardo Martinez-Miguel; Blanca Taboada; Brenda Irasema Maldonado-Meza; Carla Ivon Herrera-Najera; Carlos F. Arias; Celia Boukadida; Clara Esperanza Santacruz-Tinoco; Concepcion Grajales-Muniz; Consorcio Mexicano de Vigilancia Genomica (CoViGen-Mex). Authors (in alphabetical order): Julio Elias Alvarado-Yaah; Fernando Fontove-Herrera; Francisco Pulido; Gloria Elena Espinoza-Ayala; Gloria Maria Molina-Salinas; Gloria Vazquez; Hector Esteban Paz-Juarez; Hector Montoya-Fuentes; Helen Haydee Fernanda Ramirez-Plascencia; Jorge Ivan Salinal-Nevarez; Jose Antonio Enciso-Moreno; Jose Esteban Munoz-Medina; Jose de Jesus Nunez-Contreras; Juan Bautista Chale-Dzul; Luis Alberto Ochoa-Carrera; Margarita Matias-Florentino; Maria Guadalupe Santiago-Mauricio; Maria Guadalupe de Jesus Mireles-Rivera; Nelly Selem-Mojica; Pavel Isa; Ricardo Grande; Santiago Avila-Rios; Victor Hugo Borja-Aburto
EPI_ISL_3149735, EPI_ISL_3149736, EPI_ISL_3388827	Laboratorio Central de Salud Publica	Laboratorio de Biología Molecular, Instituto de Medicina Regional on behalf of 'Proyecto Argentino Interinstitucional de genomica de SARS-CoV-2' (PAIS Consortium)	Andrea Ayala; Bettina Brusés; Erica Struss; Esteban Paredes; Griselda Oria; Horacio Lucero.; Javier Mussin; Laura Formichelli; Melina Lorenzini Campos; Raúl Maximiliano Acevedo; Verónica Gómez; Victoria Femenías
EPI_ISL_2196253, EPI_ISL_2196260, EPI_ISL_2196261, EPI_ISL_2274072, EPI_ISL_2274073, EPI_ISL_2274074, EPI_ISL_2274075, EPI_ISL_2466262, EPI_ISL_2466263, EPI_ISL_2466264, EPI_ISL_2557337, EPI_ISL_2557338, EPI_ISL_2557339			
see above	Laboratorio Central de Saude Publica do Estado da Bahia (LACEN/BA)	Laboratory of Respiratory Viruses and Measles, Oswaldo Cruz Institute, FIOCRUZ	Alice Sampaio Rocha; Ana Carolina Mendonca; Anderson Brandao Leite; Anna Carolina Paixao; Elisa Cavalcante Pereira; Felicidade Pereira; Fernando Motta; Luciana Appolinario; Marilda Siqueira on behalf of the Fiocruz COVID-19 Genomic Surveillance Network; Paola Resende; Renata Serrano Lopes; Taina Venas
EPI_ISL_2536323, EPI_ISL_2536338, EPI_ISL_2536344, EPI_ISL_3061882, EPI_ISL_3061889, EPI_ISL_3061890	Laboratorio Central de Saude Publica do Estado da Paraiba (LACEN-PB)	Laboratory of Respiratory Viruses and Measles, Oswaldo Cruz Institute, FIOCRUZ	Alice Sampaio Rocha; Ana Carolina Mendonca; Anna Carolina Paixao; Dalane Loudal Florentino Teixeira; Elisa Cavalcante Pereira; Fernando Motta; Joao Felipe Bezerra; Luciana Appolinario; Marilda Siqueira on behalf of the Fiocruz COVID-19 Genomic Surveillance Network; Paola Resende; Renata Serrano Lopes; Taina Venas
EPI_ISL_3190189	Laboratorio Central de Saude Publica do Estado da Paraiba (LACEN/PB)	Laboratory of Respiratory Viruses and Measles, Oswaldo Cruz Institute, FIOCRUZ	Alice Sampaio Rocha; Ana Carolina Mendonca; Anna Carolina Paixao; Dalane Loudal Florentino Teixeira; Elisa Cavalcante Pereira; Fernando Motta; Joao Felipe Bezerra; Luciana Appolinario; Marilda Siqueira on behalf of the Fiocruz COVID-19 Genomic Surveillance Network; Paola Resende; Renata Serrano Lopes; Taina Venas
EPI_ISL_2274069, EPI_ISL_2274071, EPI_ISL_2466231, EPI_ISL_2466232, EPI_ISL_2466233, EPI_ISL_2466235, EPI_ISL_2466236, EPI_ISL_2466237, EPI_ISL_2466238, EPI_ISL_2466239, EPI_ISL_2466240, EPI_ISL_2466241, EPI_ISL_2466242, EPI_ISL_2466243, EPI_ISL_2466244, EPI_ISL_2466245, EPI_ISL_2466246, EPI_ISL_2466247, EPI_ISL_2466248, EPI_ISL_2466249, EPI_ISL_2466250, EPI_ISL_2466251, EPI_ISL_2466252, EPI_ISL_2466253, EPI_ISL_2466254, EPI_ISL_2466255, EPI_ISL_2466256, EPI_ISL_2466257, EPI_ISL_2466258, EPI_ISL_2466259, EPI_ISL_2557310, EPI_ISL_2557311, EPI_ISL_2557312, EPI_ISL_2557313, EPI_ISL_2557314, EPI_ISL_2557315, EPI_ISL_2557316, EPI_ISL_2557317, EPI_ISL_2557318, EPI_ISL_2557319, EPI_ISL_2557320, EPI_ISL_2557321, EPI_ISL_2557322, EPI_ISL_2557323, EPI_ISL_2557324, EPI_ISL_2557325, EPI_ISL_2557326, EPI_ISL_2557327, EPI_ISL_2557328, EPI_ISL_2557329, EPI_ISL_2557330, EPI_ISL_2557331, EPI_ISL_2557332, EPI_ISL_2557333, EPI_ISL_2557334, EPI_ISL_2557335, EPI_ISL_2557336, EPI_ISL_2645384, EPI_ISL_2645388, EPI_ISL_2645389, EPI_ISL_2645390, EPI_ISL_2645391, EPI_ISL_2645392, EPI_ISL_2645394, EPI_ISL_2645395, EPI_ISL_2645396, EPI_ISL_2645402, EPI_ISL_2645406, EPI_ISL_2645407			
see above	Laboratorio Central de Saude Publica do Estado de Alagoas (LACEN/AL)	Laboratory of Respiratory Viruses and Measles, Oswaldo Cruz Institute, FIOCRUZ	Alice Sampaio Rocha; Ana Carolina Mendonca; Anderson Brandao Leite; Anna Carolina Paixao; Elisa Cavalcante Pereira; Fernando Motta; Luciana Appolinario; Marilda Siqueira on behalf of the Fiocruz COVID-19 Genomic Surveillance Network; Paola Resende; Renata Serrano Lopes; Taina Venas
EPI_ISL_3235302, EPI_ISL_3832396, EPI_ISL_3832439, EPI_ISL_3832440, EPI_ISL_3832441, EPI_ISL_3832442, EPI_ISL_3832443, EPI_ISL_4061434, EPI_ISL_4061435, EPI_ISL_4061436			
see above	Laboratorio Central de Saude Publica do Estado de Santa Catarina (LACEN/SC)	Laboratory of Respiratory Viruses and Measles, Oswaldo Cruz Institute, FIOCRUZ	Alice Sampaio Rocha; Ana Carolina Mendonca; Anna Carolina Paixao; Darcita Buerger Rovaris; Elisa Cavalcante Pereira; Fernando Motta; Luciana Appolinario; Marilda Siqueira on behalf of the Fiocruz COVID-19 Genomic Surveillance Network; Paola Resende; Renata Serrano Lopes; Sandra Bianchini Fernandes; Taina Venas
EPI_ISL_2863618, EPI_ISL_2863619, EPI_ISL_2863625, EPI_ISL_2863628, EPI_ISL_2863635	Laboratorio Central de Saude Publica do Estado de Sergipe (LACEN/SE)	Laboratory of Respiratory Viruses and Measles, Oswaldo Cruz Institute, FIOCRUZ	Alice Sampaio Rocha; Ana Carolina Mendonca; Anna Carolina Paixao; Cliomar Alves dos Santos; Elisa Cavalcante Pereira; Fernando Motta; Luciana Appolinario; Marilda Siqueira on behalf of the Fiocruz COVID-19 Genomic Surveillance Network; Paola Resende; Renata Serrano Lopes; Tainá Moreira Martins Venas
EPI_ISL_2196262, EPI_ISL_3045432, EPI_ISL_3045433, EPI_ISL_3045448, EPI_ISL_3045462, EPI_ISL_3190238, EPI_ISL_3190239, EPI_ISL_3190241, EPI_ISL_3190242, EPI_ISL_3190243			
see above	Laboratorio Central de Saude Publica do Estado do Espirito Santo (LACEN/ES)	Laboratory of Respiratory Viruses and Measles, Oswaldo Cruz Institute, FIOCRUZ	Alice Sampaio Rocha; Ana Carolina Mendonca; Anna Carolina Paixao; Eilisa Cavalcante Pereira; Elisa Cavalcante Pereira; Fernando Motta; Luciana Appolinario; Marilda Siqueira on behalf of the Fiocruz COVID-19 Genomic Surveillance Network; Paola Resende; Renata Serrano Lopes; Rodrigo Ribeiro Rodrigues; Taina Venas
EPI_ISL_2983189, EPI_ISL_2983190, EPI_ISL_2983191, EPI_ISL_2983193, EPI_ISL_2983195, EPI_ISL_2983241, EPI_ISL_2983243, EPI_ISL_2983250, EPI_ISL_2983251, EPI_ISL_2983253, EPI_ISL_2983254, EPI_ISL_2983268, EPI_ISL_2983269, EPI_ISL_2983270, EPI_ISL_2983272, EPI_ISL_2983273, EPI_ISL_2983274, EPI_ISL_2983275, EPI_ISL_2983276, EPI_ISL_2983279, EPI_ISL_2983282, EPI_ISL_2983283, EPI_ISL_2983284, EPI_ISL_2983285, EPI_ISL_2983286, EPI_ISL_2983287, EPI_ISL_2983288, EPI_ISL_2983289, EPI_ISL_2983291, EPI_ISL_2983292, EPI_ISL_2983293, EPI_ISL_2983294, EPI_ISL_2983295, EPI_ISL_2983296, EPI_ISL_2983297, EPI_ISL_2983298, EPI_ISL_2983300, EPI_ISL_2983301, EPI_ISL_2983303, EPI_ISL_2983304, EPI_ISL_2983325, EPI_ISL_2983326, EPI_ISL_2983327, EPI_ISL_2983328, EPI_ISL_2983333, EPI_ISL_2983334, EPI_ISL_2983336, EPI_ISL_3190255, EPI_ISL_3190257, EPI_ISL_3190258			
see above	Laboratorio Central de Saude Publica do Estado do Maranhao (LACEN-MA)	Laboratory of Respiratory Viruses and Measles, Oswaldo Cruz Institute, FIOCRUZ	Agatha Cristinne Prudencio; Agatha Soares; Alice Sampaio Rocha; Ana Carolina Mendonca; Anna Carolina Paixao; Elisa Cavalcante Pereira; Fernando Motta; Ighor Arantes; Ighor Leonardo Arantes Gomes; Lidio Gonçalves Lima Neto; Luciana Appolinario; Marilda Siqueira on behalf of the Fiocruz COVID-19 Genomic Surveillance Network; Paola Resende; Renata Serrano Lopes; Taina Moreira Venas; Tainá Venas
EPI_ISL_2536354	Laboratorio Central de Saude Publica do Estado do Para (LACEN/PA)	Laboratory of Respiratory Viruses and Measles, Oswaldo Cruz Institute, FIOCRUZ	Alice Sampaio Rocha; Ana Carolina Mendonca; Anna Carolina Paixao; Elisa Cavalcante Pereira; Fernando Motta; Luciana Appolinario; Marilda Siqueira on behalf of the Fiocruz COVID-19 Genomic Surveillance Network; Paola Resende; Renata Serrano Lopes; Taina Venas; Valnete Andrade
EPI_ISL_2645458, EPI_ISL_2645461, EPI_ISL_2645463, EPI_ISL_2645465, EPI_ISL_2645466, EPI_ISL_2645470, EPI_ISL_2645471, EPI_ISL_2645472, EPI_ISL_2645474, EPI_ISL_2645482, EPI_ISL_2645484, EPI_ISL_2645485, EPI_ISL_2645487, EPI_ISL_2645491, EPI_ISL_2645492, EPI_ISL_2645494, EPI_ISL_2645495, EPI_ISL_2645497, EPI_ISL_2645498, EPI_ISL_2645499, EPI_ISL_2645500, EPI_ISL_2645501, EPI_ISL_2645503, EPI_ISL_2645504, EPI_ISL_2983447, EPI_ISL_2983448, EPI_ISL_2983450, EPI_ISL_4061299, EPI_ISL_4061300, EPI_ISL_4061301, EPI_ISL_4061302, EPI_ISL_4061305, EPI_ISL_4061306, EPI_ISL_4061308, EPI_ISL_4061309, EPI_ISL_4061312, EPI_ISL_4061313, EPI_ISL_4061314, EPI_ISL_4061315, EPI_ISL_4061317, EPI_ISL_4061318, EPI_ISL_4061320, EPI_ISL_4061321, EPI_ISL_4061322, EPI_ISL_4061323, EPI_ISL_4061324, EPI_ISL_4061325, EPI_ISL_4061326			
see above	Laboratorio Central de Saude Publica do Estado do Parana (LACEN/PR)	Laboratory of Respiratory Viruses and Measles, Oswaldo Cruz Institute, FIOCRUZ	Agatha Soares; Alice Sampaio Rocha; Ana Carolina Mendonca; Anna Carolina Paixao; Elisa Cavalcante Pereira; Fernando Motta; Ighor Leonardo Arantes; Irina Riediger; Luciana Appolinario; Marilda Siqueira on behalf of the Fiocruz COVID-19 Genomic Surveillance Network; Paola Resende; Renata Serrano Lopes; Taina Venas
EPI_ISL_2603432, EPI_ISL_2603433, EPI_ISL_2603436, EPI_ISL_2603442, EPI_ISL_2603443, EPI_ISL_2603444, EPI_ISL_2603445, EPI_ISL_2603446, EPI_ISL_2603447, EPI_ISL_2603448, EPI_ISL_2603450, EPI_ISL_2603536, EPI_ISL_2603537, EPI_ISL_2603538, EPI_ISL_2603539, EPI_ISL_2603540, EPI_ISL_2603541, EPI_ISL_2603542, EPI_ISL_2603547, EPI_ISL_2603548, EPI_ISL_2603550, EPI_ISL_2603551, EPI_ISL_2603552, EPI_ISL_2603553, EPI_ISL_2603554, EPI_ISL_2603555, EPI_ISL_2603556, EPI_ISL_2603559, EPI_ISL_2603560, EPI_ISL_2603562, EPI_ISL_2603563, EPI_ISL_2603564, EPI_ISL_2603565, EPI_ISL_2603566, EPI_ISL_2603567, EPI_ISL_2603568, EPI_ISL_2603569, EPI_ISL_2603571, EPI_ISL_2603572, EPI_ISL_2603573, EPI_ISL_2603574, EPI_ISL_2603575, EPI_ISL_2603576, EPI_ISL_2603577, EPI_ISL_2603578, EPI_ISL_2603579, EPI_ISL_2603581, EPI_ISL_2603583, EPI_ISL_2603586, EPI_ISL_2603587, EPI_ISL_2603588, EPI_ISL_2603589, EPI_ISL_2603590, EPI_ISL_2982757, EPI_ISL_2982758, EPI_ISL_2982759, EPI_ISL_2982760, EPI_ISL_2982761, EPI_ISL_2982770, EPI_ISL_2982771, EPI_ISL_2982772, EPI_ISL_2982773, EPI_ISL_2982774, EPI_ISL_2982778, EPI_ISL_2982782, EPI_ISL_2982783, EPI_ISL_2982785, EPI_ISL_2982787, EPI_ISL_2982789, EPI_ISL_2982790, EPI_ISL_2982791, EPI_ISL_2982793			
see above	Laboratorio Central de Saude Publica do Estado do Rio Grande do Sul (LACEN-RS)	Laboratory of Respiratory Viruses and Measles, Oswaldo Cruz Institute, FIOCRUZ	Alice Sampaio Rocha; Ana Carolina Mendonca; Anderson Brandao Leite; Anna Carolina Paixao; Elisa Cavalcante Pereira; Fernando Motta; Luciana Appolinario; Marilda Siqueira on behalf of the Fiocruz COVID-19 Genomic Surveillance Network; Paola Resende; Renata Serrano Lopes; Taina Venas
EPI_ISL_3048803, EPI_ISL_3048805, EPI_ISL_3048807, EPI_ISL_3048808	Laboratorio Central de Saude Publica do Estado do Rio Grande do Sul (LACEN-RS)	Laboratório de Biologia Molecular da Universidade Federal de Ciências da Saúde de Porto Alegre	Adriana Seixas; Ana B. G. Veiga; Ana Paula Muterle Varela; Fabiana Quoos Mayer; Fernando Hayashi Sant'Anna; Janira Prichula; Letícia Garay Martins; Richard Steiner Salvato; Tatiana Schäffer Gregianini
EPI_ISL_2983213	Laboratorio Central de Saude Publica do Estado do Rio de Janeiro (LACEN-RJ)	Laboratory of Respiratory Viruses and Measles, Oswaldo Cruz Institute, FIOCRUZ	Agatha Cristinne Prudencio; Alice Sampaio Rocha; Ana Carolina Mendonca; Andrea Cony Cavalcanti; Anna Carolina Paixao; Elisa Cavalcante Pereira; Fernando Motta; Ighor Leonardo Arantes Gomes; Luciana Appolinario; Marilda Siqueira on behalf of the Fiocruz COVID-19 Genomic Surveillance Network; Paola Resende; Renata Serrano Lopes; Taina Venas
EPI_ISL_3033037, EPI_ISL_3033040, EPI_ISL_3033041, EPI_ISL_3033044, EPI_ISL_3033052, EPI_ISL_3033053, EPI_ISL_3033054, EPI_ISL_3033055, EPI_ISL_3033057, EPI_ISL_3033060, EPI_ISL_3033062, EPI_ISL_3033063			
see above	Laboratorio Central de la Ciudad de Santa Fe	Grupo de Genómica y Bioinformática del Instituto de Investigación de la Cadena Láctea CONICET-INTA on behalf of 'Proyecto Argentino Interinstitucional de genomica de SARS-CoV-2' (PAIS Consortium)	AF; Amadio; C; Eberhardt; G; Irazoqui; JM; MF; Mugna; Ojeda; Pastor; Rompato; V
EPI_ISL_2271688, EPI_ISL_2271690, EPI_ISL_2271708	Laboratorio Central, Ministerio de Salud Cordoba	Instituto de Patologia Vegetal (CIAP-INTA) on behalf of 'Proyecto Argentino Interinstitucional de genomica de SARS-CoV-2' (PAIS Consortium)	Barbas, G.; Castro, G.; Debat, HJ.; FD; Fernandez; M; M.B.; Marquez, N.; Pisano; Re, V.
EPI_ISL_3333567	Laboratorio Clínico Gamma	MELISA Institute Genomics and Proteomics SpA	Cristian Vargas; Elard Koch; Francisco Alvarez; Guillermo Nourdin; Mauricio Hernandez
EPI_ISL_3045409, EPI_ISL_3045415, EPI_ISL_3045418, EPI_ISL_3045419,	Laboratorio Nacional de Salud Pública Dr. Defilló - LNSPDD	Laboratory of Respiratory Viruses and Measles, Oswaldo Cruz Institute, FIOCRUZ	Alice Sampaio Rocha; Ana Carolina Mendonca; Anna Carolina Paixao; Elisa Cavalcante Pereira; Fernando Motta; Grey Benoit Vasquez; Isaac Miguel Sanchez; Ivonne Imbert; Lucia de la Cruz; Luciana Appolinario; Marilda Siqueira on behalf of the Fiocruz COVID-19 Genomic Surveillance Network; Nurys de Castro; Paola Resende; Renata Serrano Lopes; Ronald Skewes; Taina Venas

EPI_ISL_3045420			
EPI_ISL_3275219	Laboratorio Nacional de Salud, Ministerio de Salud Publica y Asistencia Social	Genomics and Proteomics Departament, Gorgas Memorial Institute For Health Studies	Alexander A Martinez; Ambar Moreno; Claudia Estrada; Claudia Gonzalez V; César Roberto Conde Pereira; Jessica Gondola; Leyda Abrego; Marlene Castillo; Oris Chavarria
EPI_ISL_3307458	Laboratorio SYNLAB Colombia	Corporación para Investigaciones Biológicas-CIB	Jeanneth Mosquera Rendon; Jenny Santiago Cuesta; Marcela Mercado Reyes; Uriel A. Hurtado Paez
EPI_ISL_2777494, EPI_ISL_4030325, EPI_ISL_4030327, EPI_ISL_4030335, EPI_ISL_4030337, EPI_ISL_4030339, EPI_ISL_4030346, EPI_ISL_4030349, EPI_ISL_4030355, EPI_ISL_4030358, EPI_ISL_4030361, EPI_ISL_4030364			
see above	Laboratorio de Ecologia de Doencas Transmissiveis na Amazonia, Instituto Leonidas e Maria Deane - Fiocruz Amazonia	Laboratorio de Ecologia de Doencas Transmissiveis na Amazonia, Instituto Leonidas e Maria Deane - Fiocruz Amazonia	Alex Martins; André Corado; Debora Duarte; Fabiola Mendonça da Silva Chui; Felipe Naveca; Fernanda Nascimento; Fernando Fonseca de Almeida Val; George Silva; Gisely Cardoso de Melo; Karina Pessoa; Luciana Gonçalves; Marcus Vinicius Guimarães de Lacerda; Maria Júlia Brandão; Maria Paula Gomes Mourão; Mariana Xavier Simão; Matilde Mejía; Michele Jesus; Valdinete Nascimento; Vanderson de Souza Sampaio; Victor Souza; Ágatha Costa
EPI_ISL_2728553	Laboratorio de Infectologia y Virologia Molecular	Laboratory of Molecular Virology, School of Medicine, Pontificia Universidad Catolica de Chile	Alejandro Bhrun; Ana Maria Contreras; Andres E. Munoz-Marcos; Carlos Palma; Catalina Pardo-Roa; Constanza Maldonado; Constanza Martinez-Valdevenito; Eileen Serrano; Erick Salinas; Estefany Poblete; Francisco Melo; Jennifer Angulo; Jorge Levican; Leonardo I. Almonacid; M. Belen Leyton; Magdalena Vera; Marcela Ferres; Maria Jose Avendano; Rafael A. Medina; Tamara Garcia-Salum
EPI_ISL_2854140	Laboratorio de Medicina Molecular, Universidad de Magallanes	Centro Asistencial Docente y de Investigacion, Universidad de Magallanes	Constanza Ceroni; Diego Alvarez; Jacqueline Aldridge; Jorge González; Marcelo Navarrete; Roberto Uribe-Paredes
EPI_ISL_2008913, EPI_ISL_2008915, EPI_ISL_2008923, EPI_ISL_2008927, EPI_ISL_2008931, EPI_ISL_3048959, EPI_ISL_3048960, EPI_ISL_3048961, EPI_ISL_3048962, EPI_ISL_3048964, EPI_ISL_3048966, EPI_ISL_3048968, EPI_ISL_3048969, EPI_ISL_3048970, EPI_ISL_3048971, EPI_ISL_3048972, EPI_ISL_3048973, EPI_ISL_3048974, EPI_ISL_3048976, EPI_ISL_3048977, EPI_ISL_3048978, EPI_ISL_3761613, EPI_ISL_3761616, EPI_ISL_3761636, EPI_ISL_3761645, EPI_ISL_3761646, EPI_ISL_3761749, EPI_ISL_3761752, EPI_ISL_3761763			
see above	Laboratorio de Pesquisa em Virologia, FAMERP, SJRP	Laboratorio de Pesquisa em Virologia, FAMERP, SJRP	Beatriz de Carvalho Marques; Cecília Artico Banho; Cíntia Bittar; Fábio Sossai Possebon; Guilherme Campos; Helena Lage Ferreira; Jorge A. Petroli Marchesi; João Pessoa Araújo Jr.; Leila Sabrina Ullmann; Lívia Sacchetto; Maisa C. Pereira Parra; Marília Moraes; Maurício L. Nogueira.; Paula Rahal; Paulo Inacio da Costa
EPI_ISL_3401433, EPI_ISL_3401453, EPI_ISL_3401455, EPI_ISL_3401458, EPI_ISL_3401459, EPI_ISL_3401463, EPI_ISL_3401464, EPI_ISL_3672413, EPI_ISL_3672414, EPI_ISL_3672415, EPI_ISL_3672418, EPI_ISL_3826392, EPI_ISL_3826393, EPI_ISL_3826394, EPI_ISL_3826395, EPI_ISL_3826396, EPI_ISL_3826397, EPI_ISL_3826398, EPI_ISL_3826399, EPI_ISL_3826400, EPI_ISL_3826401, EPI_ISL_3826403, EPI_ISL_3826404, EPI_ISL_3826407, EPI_ISL_3826409, EPI_ISL_3826410, EPI_ISL_3826411, EPI_ISL_3826413, EPI_ISL_4052702, EPI_ISL_4052714, EPI_ISL_4052715, EPI_ISL_4052717, EPI_ISL_4052719, EPI_ISL_4052720, EPI_ISL_4052721, EPI_ISL_4052722, EPI_ISL_4052724, EPI_ISL_4052725, EPI_ISL_4052726, EPI_ISL_4052728, EPI_ISL_4052729			
see above	Laboratorio de Referencia Nacional de Virus Respiratorios. Centro Nacional de Salud Publica. Instituto Nacional de Salud Peru.	Laboratorio de Referencia Nacional de Virus Respiratorios. Centro Nacional de Salud Publica. Instituto Nacional de Salud Peru.	Carlos Padilla Rojas; Henri Bailon Calderon; Iris Silva Molina; Joseph Huayra Niquen; Lely Solari Zerpa; Luis Barcena Flores; Marco Galarza Perez; Nancy Rojas Serrano; Nieves Sevilla Castañeda; Omar Caceres Rey; Orson Mestanza Millones; Princesa Medrano Alhuay; Priscila Lope Pari; Sandra Morales Ruiz; Sara Gordillo Vilchez; Steve Acedo Lazo; Veronica Hurtado Vela; Victor Jimenez Vasquez; Wendy Lizarraga Olivares
EPI_ISL_3023587, EPI_ISL_3023588, EPI_ISL_3023599, EPI_ISL_3375938, EPI_ISL_3376007, EPI_ISL_3376013, EPI_ISL_3376020, EPI_ISL_3376021, EPI_ISL_3376023, EPI_ISL_3376031, EPI_ISL_3376382, EPI_ISL_3376383			
see above	Laboratorio de Referencial Nacional de Virus Respiratorios	Laboratorio de Referencial Nacional de Virus Respiratorios	Carlos Padilla Rojas; Henri Bailon Calderon; Iris Silva Molina; Joseph Huayra Niquen; Lely Solari Zerpa; Luis Barcena Flores; Marco Galarza Perez; Nancy Rojas Serrano; Omar Caceres Rey; Orson Mestanza Millones; Priscila Lope Pari; Sandra Morales Ruiz; Steve Acedo Lazo; Veronica Hurtado Vela
EPI_ISL_1926315, EPI_ISL_1926316, EPI_ISL_1926589, EPI_ISL_1926612, EPI_ISL_1926667, EPI_ISL_1926675, EPI_ISL_1926678, EPI_ISL_1926685, EPI_ISL_1926690, EPI_ISL_1926829, EPI_ISL_1926835, EPI_ISL_1926839, EPI_ISL_1926989, EPI_ISL_1927003, EPI_ISL_1927009, EPI_ISL_1927018, EPI_ISL_1927055, EPI_ISL_1927072, EPI_ISL_1927085, EPI_ISL_1927142, EPI_ISL_1927259, EPI_ISL_1927307, EPI_ISL_1928731, EPI_ISL_1928733, EPI_ISL_1928741, EPI_ISL_1928742, EPI_ISL_1928787, EPI_ISL_1928803, EPI_ISL_1928809, EPI_ISL_1928811, EPI_ISL_1928814, EPI_ISL_1928818, EPI_ISL_1928828, EPI_ISL_1928879, EPI_ISL_1928897, EPI_ISL_1928907, EPI_ISL_1928908, EPI_ISL_1928909, EPI_ISL_1928928, EPI_ISL_1928929, EPI_ISL_1928980, EPI_ISL_1928986, EPI_ISL_1928987, EPI_ISL_1929039, EPI_ISL_1929111, EPI_ISL_1929137, EPI_ISL_1929161, EPI_ISL_1929198, EPI_ISL_1929201, EPI_ISL_1929323, EPI_ISL_1929370, EPI_ISL_1929553, EPI_ISL_1929984, EPI_ISL_1930106, EPI_ISL_1930112, EPI_ISL_1930276, EPI_ISL_1930412, EPI_ISL_1930439, EPI_ISL_1930531, EPI_ISL_1930579, EPI_ISL_2044024, EPI_ISL_2044065, EPI_ISL_2044267, EPI_ISL_2044278, EPI_ISL_2044295, EPI_ISL_2044456, EPI_ISL_2044471, EPI_ISL_2044472, EPI_ISL_2044487, EPI_ISL_2044489, EPI_ISL_2044496, EPI_ISL_2044499, EPI_ISL_2044501, EPI_ISL_2044505, EPI_ISL_2044555, EPI_ISL_2044602, EPI_ISL_2044671, EPI_ISL_2044683, EPI_ISL_2044693, EPI_ISL_2044697, EPI_ISL_2044698, EPI_ISL_2044699, EPI_ISL_2044720, EPI_ISL_2044853, EPI_ISL_2045023, EPI_ISL_2045127, EPI_ISL_2045128, EPI_ISL_2045132, EPI_ISL_2045145, EPI_ISL_2045155, EPI_ISL_2045159, EPI_ISL_2045183, EPI_ISL_2045202, EPI_ISL_2045204, EPI_ISL_2045215, EPI_ISL_2045231, EPI_ISL_2045239, EPI_ISL_2045247, EPI_ISL_2045250, EPI_ISL_2045257, EPI_ISL_2045275, EPI_ISL_2045311, EPI_ISL_2045318, EPI_ISL_2045323, EPI_ISL_2045324, EPI_ISL_2045334, EPI_ISL_2045404, EPI_ISL_2045410, EPI_ISL_2045412, EPI_ISL_2045416, EPI_ISL_2045458, EPI_ISL_2045470, EPI_ISL_2045588, EPI_ISL_2045763, EPI_ISL_2045771, EPI_ISL_2045779, EPI_ISL_2045784, EPI_ISL_2045858, EPI_ISL_2045872, EPI_ISL_2046300, EPI_ISL_2046385, EPI_ISL_2046456, EPI_ISL_2046488, EPI_ISL_2046524, EPI_ISL_2046569, EPI_ISL_2046623, EPI_ISL_2046739, EPI_ISL_2046796, EPI_ISL_2182100, EPI_ISL_2182182, EPI_ISL_2182211, EPI_ISL_2182217, EPI_ISL_2182231, EPI_ISL_2182239, EPI_ISL_2182313, EPI_ISL_2182319, EPI_ISL_2182333, EPI_ISL_2182341, EPI_ISL_2182349, EPI_ISL_2182399, EPI_ISL_2182437, EPI_ISL_2182452, EPI_ISL_2182463, EPI_ISL_2182535, EPI_ISL_2182628, EPI_ISL_2182658, EPI_ISL_2182666, EPI_ISL_2182725, EPI_ISL_2182728, EPI_ISL_2182755, EPI_ISL_2182761, EPI_ISL_2182805, EPI_ISL_2182853, EPI_ISL_2182883, EPI_ISL_2182976, EPI_ISL_2182990, EPI_ISL_2183097, EPI_ISL_2183177, EPI_ISL_2183380, EPI_ISL_2183411, EPI_ISL_2183480, EPI_ISL_2183486, EPI_ISL_2183491, EPI_ISL_2183509, EPI_ISL_2183512, EPI_ISL_2183537, EPI_ISL_2183540, EPI_ISL_2183561, EPI_ISL_2183603, EPI_ISL_2183617, EPI_ISL_2183623, EPI_ISL_2183626, EPI_ISL_2183634, EPI_ISL_2183639, EPI_ISL_2183645, EPI_ISL_2183656, EPI_ISL_2183664, EPI_ISL_2183757, EPI_ISL_2183778, EPI_ISL_2183781, EPI_ISL_2183798, EPI_ISL_2183816, EPI_ISL_2183821, EPI_ISL_2183826, EPI_ISL_2183840, EPI_ISL_2183847, EPI_ISL_2183855, EPI_ISL_2183860, EPI_ISL_2183862, EPI_ISL_2183868, EPI_ISL_2183870, EPI_ISL_2183883, EPI_ISL_2183884, EPI_ISL_2183898, EPI_ISL_2183902, EPI_ISL_2183912, EPI_ISL_2183918, EPI_ISL_2183987, EPI_ISL_2183988, EPI_ISL_2184005, EPI_ISL_2184013, EPI_ISL_2184016, EPI_ISL_2184032, EPI_ISL_2184033, EPI_ISL_2184056, EPI_ISL_2184174, EPI_ISL_2184190, EPI_ISL_2184195, EPI_ISL_2184257, EPI_ISL_2184325, EPI_ISL_2184348, EPI_ISL_2184440, EPI_ISL_2184550, EPI_ISL_2184551, EPI_ISL_2184559, EPI_ISL_2184563, EPI_ISL_2184588, EPI_ISL_2184596, EPI_ISL_2184612, EPI_ISL_2184620, EPI_ISL_2184639, EPI_ISL_2184712, EPI_ISL_2184725, EPI_ISL_2184726, EPI_ISL_2184730, EPI_ISL_2184731, EPI_ISL_2184732, EPI_ISL_2184736, EPI_ISL_2184753, EPI_ISL_2184766, EPI_ISL_2184770, EPI_ISL_2184775, EPI_ISL_2184788, EPI_ISL_2184811, EPI_ISL_2184820, EPI_ISL_2184833, EPI_ISL_2184915, EPI_ISL_2185069, EPI_ISL_2185076, EPI_ISL_2185084, EPI_ISL_2185086, EPI_ISL_2185097, EPI_ISL_2185134, EPI_ISL_2185161, EPI_ISL_2185470, EPI_ISL_2185479, EPI_ISL_2185483, EPI_ISL_2304837, EPI_ISL_2304918, EPI_ISL_2304923, EPI_ISL_2304925, EPI_ISL_2304926, EPI_ISL_2304959, EPI_ISL_2304961, EPI_ISL_2304963, EPI_ISL_2305051, EPI_ISL_2305057, EPI_ISL_2305060, EPI_ISL_2305063, EPI_ISL_2305074, EPI_ISL_2305095, EPI_ISL_2305191, EPI_ISL_2305257, EPI_ISL_2305293, EPI_ISL_2305336, EPI_ISL_2305339, EPI_ISL_2305682, EPI_ISL_2305684, EPI_ISL_2305729, EPI_ISL_2305733, EPI_ISL_2305735, EPI_ISL_2305738, EPI_ISL_2305741, EPI_ISL_2305745, EPI_ISL_2305766, EPI_ISL_2305790, EPI_ISL_2305791, EPI_ISL_2305805, EPI_ISL_2305810, EPI_ISL_2305814, EPI_ISL_2305818, EPI_ISL_2305834, EPI_ISL_2305842, EPI_ISL_2305860, EPI_ISL_2305904, EPI_ISL_2305905, EPI_ISL_2305906, EPI_ISL_2305974, EPI_ISL_2306026, EPI_ISL_2306028, EPI_ISL_2306030, EPI_ISL_2306032, EPI_ISL_2306051, EPI_ISL_2306054, EPI_ISL_2306057, EPI_ISL_2306059, EPI_ISL_2306071, EPI_ISL_2306082, EPI_ISL_2306120, EPI_ISL_2306131, EPI_ISL_2306186, EPI_ISL_2306193, EPI_ISL_2306218, EPI_ISL_2306276, EPI_ISL_2306286, EPI_ISL_2306329, EPI_ISL_2306335, EPI_ISL_2306336, EPI_ISL_2306414, EPI_ISL_2306417, EPI_ISL_2306422, EPI_ISL_2306426, EPI_ISL_2306429, EPI_ISL_2306432, EPI_ISL_2306433, EPI_ISL_2306435, EPI_ISL_2306439, EPI_ISL_2306442, EPI_ISL_2306455, EPI_ISL_2306461, EPI_ISL_2306468, EPI_ISL_2306471, EPI_ISL_2306475, EPI_ISL_2306478, EPI_ISL_2306481, EPI_ISL_2306489, EPI_ISL_2306504, EPI_ISL_2306540, EPI_ISL_2306550, EPI_ISL_2306552, EPI_ISL_2306555, EPI_ISL_2306557, EPI_ISL_2306564, EPI_ISL_2306565, EPI_ISL_2306566, EPI_ISL_2306574, EPI_ISL_2306599, EPI_ISL_2306603, EPI_ISL_2306616, EPI_ISL_2306621, EPI_ISL_2306623, EPI_ISL_2306634, EPI_ISL_2306640, EPI_ISL_2306671, EPI_ISL_2306681, EPI_ISL_2306718, EPI_ISL_2306727, EPI_ISL_2306729, EPI_ISL_2306743, EPI_ISL_2306748, EPI_ISL_2306759, EPI_ISL_2306761, EPI_ISL_2306762, EPI_ISL_2306777, EPI_ISL_2306811, EPI_ISL_2306849, EPI_ISL_2306885, EPI_ISL_2306917, EPI_ISL_2306919, EPI_ISL_2306930, EPI_ISL_2306942, EPI_ISL_2306976, EPI_ISL_2306979, EPI_ISL_2306988, EPI_ISL_2306993, EPI_ISL_2306997, EPI_ISL_2307053, EPI_ISL_2324462, EPI_ISL_2324468, EPI_ISL_2324469, EPI_ISL_2324474, EPI_ISL_2324481, EPI_ISL_2324487, EPI_ISL_2324493, EPI_ISL_2324495, EPI_ISL_2324519, EPI_ISL_2324530, EPI_ISL_2324536, EPI_ISL_2324538, EPI_ISL_2324544, EPI_ISL_2324565, EPI_ISL_2324570, EPI_ISL_2324577, EPI_ISL_2324580, EPI_ISL_2324588, EPI_ISL_2397857, EPI_ISL_2397866, EPI_ISL_2397903, EPI_ISL_2397944, EPI_ISL_2397955, EPI_ISL_2397956, EPI_ISL_2397957, EPI_ISL_2397998, EPI_ISL_2398020, EPI_ISL_2398042, EPI_ISL_2398055, EPI_ISL_2398076, EPI_ISL_2398079, EPI_ISL_2398083, EPI_ISL_2398097, EPI_ISL_2398111, EPI_ISL_2398114, EPI_ISL_2398115, EPI_ISL_2398153, EPI_ISL_2398158, EPI_ISL_2398166, EPI_ISL_2398168, EPI_ISL_2398177, EPI_ISL_2398192, EPI_ISL_2398200, EPI_ISL_2398208, EPI_ISL_2398220, EPI_ISL_2398222, EPI_ISL_2398225, EPI_ISL_2398235, EPI_ISL_2398244, EPI_ISL_2398245, EPI_ISL_2398247, EPI_ISL_2398248, EPI_ISL_2398252, EPI_ISL_2398255, EPI_ISL_2398256, EPI_ISL_2398258, EPI_ISL_2398274, EPI_ISL_2398276, EPI_ISL_2398281, EPI_ISL_2398318, EPI_ISL_2398319, EPI_ISL_2398324, EPI_ISL_2398325, EPI_ISL_2398334, EPI_ISL_2398338, EPI_ISL_2398343, EPI_ISL_2398351, EPI_ISL_2398352, EPI_ISL_2398355, EPI_ISL_2398364, EPI_ISL_2398372, EPI_ISL_2398373, EPI_ISL_2398376, EPI_ISL_2398386, EPI_ISL_2398401, EPI_ISL_2398451, EPI_ISL_2398458, EPI_ISL_2398459, EPI_ISL_2398476, EPI_ISL_2398479, EPI_ISL_2398481, EPI_ISL_2398482, EPI_ISL_2398483, EPI_ISL_2398491, EPI_ISL_2398502, EPI_ISL_2398503, EPI_ISL_2398508, EPI_ISL_2398513, EPI_ISL_2398520, EPI_ISL_2398526, EPI_ISL_2398530, EPI_ISL_2398531, EPI_ISL_2398567, EPI_ISL_2398632, EPI_ISL_2398765, EPI_ISL_2398769, EPI_ISL_2398801, EPI_ISL_2398803, EPI_ISL_2398804, EPI_ISL_2398842, EPI_ISL_2398845, EPI_ISL_2398884, EPI_ISL_2398902, EPI_ISL_2398917, EPI_ISL_2398922, EPI_ISL_2398928, EPI_ISL_2398935, EPI_ISL_2398936, EPI_ISL_2398941, EPI_ISL_2398948, EPI_ISL_2398957, EPI_ISL_2398969, EPI_ISL_2398979, EPI_ISL_2398981, EPI_ISL_2399009, EPI_ISL_2399041, EPI_ISL_2399050, EPI_ISL_2399052, EPI_ISL_2399065, EPI_ISL_2399149, EPI_ISL_2399155, EPI_ISL_2399166, EPI_ISL_2399193, EPI_ISL_2399204, EPI_ISL_2399233, EPI_ISL_2399234, EPI_ISL_2399254, EPI_ISL_2399255, EPI_ISL_2399265, EPI_ISL_2399267, EPI_ISL_2399268, EPI_ISL_2399272, EPI_ISL_2399290, EPI_ISL_2399302, EPI_ISL_2399336, EPI_ISL_2399338, EPI_ISL_2399351, EPI_ISL_2399355, EPI_ISL_2399367, EPI_ISL_2480199, EPI_ISL_2480638, EPI_ISL_2480639, EPI_ISL_2480646, EPI_ISL_2480702, EPI_ISL_2480790, EPI_ISL_2480791, EPI_ISL_2480987, EPI_ISL_2481019, EPI_ISL_2481039, EPI_ISL_2481044, EPI_ISL_2481058, EPI_ISL_2481084, EPI_ISL_2481110, EPI_ISL_2481130, EPI_ISL_2481143, EPI_ISL_2481144, EPI_ISL_2481514, EPI_ISL_2481521, EPI_ISL_2481597, EPI_ISL_2481875, EPI_ISL_2611267, EPI_ISL_2611268, EPI_ISL_2611271, EPI_ISL_2611272, EPI_ISL_2611280			
see above	Laboratory Corporation of America	Centers for Disease Control and Prevention Division of Viral Diseases, Pathogen Discovery	Adrian Paskey; Amanda Douglas; Amanda Suchanek; Andrea Throop; Ayla Burns; Benjamin Rambo-Martin; Bobbi Croy; Brian Krueger; Brian Norvell; Christopher Gulvick; Christos Petropoulos; Clinton R. Paden; Craig Lukasik; Dakota Howard; Darlene Wagner; Debbie Boles; Dhwani Batra; Duncan MacCannell; Eyad Almasri; Goran Stevovic; Howard Engler; Hrushikesh Deshmukh; Jake Humphrey; Jana Schroth; Jason Caravas; Joe Voshell; John Pruitt; Jonathan Meltzer; Jonathan Williams; Kara Moser; Kimberly Wagner; Lax Iyer; Lisa Pfefferle; Lyndon Tilson; Manoj Jain; Marcia Eisenberg; Mary Ann Cristobal; Mary Williamson; Matthew Robinson; Matthew Schmerer; Michael Levandoski; Mike Sapeta; Mindy Nye; Minoo Agarwal; Mohan Kolli; Nuthawin Charoensri; Oren Cohen; Peter W. Cook; Prashant Gupta; Qian Zeng; Rama Ghatti; Scott Parker; Scott Ryan; Scott Sammons; Shatavia Morrison; Stanley Letovsky; Steven Ragan; Suresh Babu Selvaraju; Susan Countryman; Susan Hicks; Suzanne Dale; Thomas Urban; Tim Kuphal; Tricia Zwiefelhofer; Vincent Drouillon; Yvette Unoarumhi
EPI_ISL_2835679	Laboratory Medicine, University of Washington	Laboratory Medicine, University of Washington	Castor; J.L.
EPI_ISL_2157545, EPI_ISL_2196251, EPI_ISL_2443601, EPI_ISL_2443605, EPI_ISL_2443606, EPI_ISL_2443607, EPI_ISL_2443610, EPI_ISL_2443611, EPI_ISL_2443613, EPI_ISL_2443634, EPI_ISL_2443659, EPI_ISL_2443660, EPI_ISL_2443661, EPI_ISL_2443662, EPI_ISL_2557403, EPI_ISL_3045381, EPI_ISL_3045471, EPI_ISL_3434727, EPI_ISL_3434728, EPI_ISL_3539937			
see above	Laboratory of Respiratory Viruses and Measles, Oswaldo Cruz Institute, FIOCRUZ	Laboratory of Respiratory Viruses and Measles, Oswaldo Cruz Institute, FIOCRUZ	Agatha Cristinne Prudencio; Agatha Cristinne Prudencio Soares; Alice Sampaio Rocha; Ana Carolina Mendonca; Andrea Cony Cavalcanti; Anna Carolina Paixao; Elisa Cavalcante Pereira; Fernando Motta; Ighor Leonardo Arantes Gomes; Luciana Appolinario; Marilda Siqueira on behalf of the Fiocruz COVID-19 Genomic Surveillance Network; Paola Resende; Renata Serrano Lopes; Taina Venas
EPI_ISL_2696345, EPI_ISL_2696352, EPI_ISL_2696373, EPI_ISL_2696378, EPI_ISL_2696394, EPI_ISL_2696396, EPI_ISL_2696397, EPI_ISL_2696417, EPI_ISL_2696466, EPI_ISL_2696555, EPI_ISL_2696567, EPI_ISL_2696612, EPI_ISL_2696651, EPI_ISL_2696654, EPI_ISL_2696754, EPI_ISL_2696783, EPI_ISL_2696815, EPI_ISL_2696877, EPI_ISL_3025502, EPI_ISL_3025522, EPI_ISL_3025524, EPI_ISL_3025584			
see above	Laboratory, Gravity Diagnostics	Laboratory, Gravity Diagnostics	Diagnostics, G.
EPI_ISL_2157542, EPI_ISL_2157543, EPI_ISL_2157544	Laboratório Central de Saude Publica do Estado da Bahia (LACEN/BA)	Laboratory of Respiratory Viruses and Measles, Oswaldo Cruz Institute, FIOCRUZ	Alice Sampaio Rocha; Ana Carolina Mendonca; Anna Carolina Paixao; Elisa Cavalcante Pereira; Felicidade Pereira; Fernando Motta; Luciana Appolinario; Marilda Siqueira on behalf of the Fiocruz COVID-19 Genomic Surveillance Network; Paola Resende; Renata Serrano Lopes; Taina Venas
EPI_ISL_2777242, EPI_ISL_2777243, EPI_ISL_2777244, EPI_ISL_2777245, EPI_ISL_2777246, EPI_ISL_2777280, EPI_ISL_2777281, EPI_ISL_2777282, EPI_ISL_2777283, EPI_ISL_2777284, EPI_ISL_2777285, EPI_ISL_2777292, EPI_ISL_2777295, EPI_ISL_2777297, EPI_ISL_2777299, EPI_ISL_2777313, EPI_ISL_2777318, EPI_ISL_2777334, EPI_ISL_2777345, EPI_ISL_2777346, EPI_ISL_2777347, EPI_ISL_2777348, EPI_ISL_2777511, EPI_ISL_2777513, EPI_ISL_2777515, EPI_ISL_2777517, EPI_ISL_2777524, EPI_ISL_2777527, EPI_ISL_2777530, EPI_ISL_2777532, EPI_ISL_2777545, EPI_ISL_2777546, EPI_ISL_2777553, EPI_ISL_2777556, EPI_ISL_2777559, EPI_ISL_2777561, EPI_ISL_2777562, EPI_ISL_2777570, EPI_ISL_2777573, EPI_ISL_2777575, EPI_ISL_2777584, EPI_ISL_2777585, EPI_ISL_2777586, EPI_ISL_2777591, EPI_ISL_2777593, EPI_ISL_2777600, EPI_ISL_2777601, EPI_ISL_2777603, EPI_ISL_2777604, EPI_ISL_2777605, EPI_ISL_2777606, EPI_ISL_2777612, EPI_ISL_2777622, EPI_ISL_2777624, EPI_ISL_2777627, EPI_ISL_2777628, EPI_ISL_2777630, EPI_ISL_2777632, EPI_ISL_2777634, EPI_ISL_2777635, EPI_ISL_2777636, EPI_ISL_2777637, EPI_ISL_2777639, EPI_ISL_2777641, EPI_ISL_2777642, EPI_ISL_2777643, EPI_ISL_2777645, EPI_ISL_2777654, EPI_ISL_2777659, EPI_ISL_2777675, EPI_ISL_2777683, EPI_ISL_2777686, EPI_ISL_2777687, EPI_ISL_2777693, EPI_ISL_2777702, EPI_ISL_2777703, EPI_ISL_2777711, EPI_ISL_2777757, EPI_ISL_2777764, EPI_ISL_2777765, EPI_ISL_2777833, EPI_ISL_2777834, EPI_ISL_2777837, EPI_ISL_2777839, EPI_ISL_2777844, EPI_ISL_2777845, EPI_ISL_2777849, EPI_ISL_2777850, EPI_ISL_2777875, EPI_ISL_2777876, EPI_ISL_2777877, EPI_ISL_2777878, EPI_ISL_2777879, EPI_ISL_2777880, EPI_ISL_2777881, EPI_ISL_2777882, EPI_ISL_2777883, EPI_ISL_2777884, EPI_ISL_2777885, EPI_ISL_2777886, EPI_ISL_2777888, EPI_ISL_2777892, EPI_ISL_2777900, EPI_ISL_2777903, EPI_ISL_2777911, EPI_ISL_2777913, EPI_ISL_2777915, EPI_ISL_2777916, EPI_ISL_2777917, EPI_ISL_2777919, EPI_ISL_2777922, EPI_ISL_2777923, EPI_ISL_2777932, EPI_ISL_2777934, EPI_ISL_2777935, EPI_ISL_2777936, EPI_ISL_2777938, EPI_ISL_2777939, EPI_ISL_2777940, EPI_ISL_2777950, EPI_ISL_2777952, EPI_ISL_2777954, EPI_ISL_2777955, EPI_ISL_2777962, EPI_ISL_2777963, EPI_ISL_2777964, EPI_ISL_2777965, EPI_ISL_2777980, EPI_ISL_2777989, EPI_ISL_2777990, EPI_ISL_2777994, EPI_ISL_2778006, EPI_ISL_2778007			
see above	Laboratório Central de Saúde Pública do Amazonas - LACEN-AM	Laboratorio de Ecologia de Doencas Transmissiveis na Amazonia, Instituto Leonidas e Maria Deane - Fiocruz Amazonia	André Corado; Debora Duarte; Felipe Naveca; Fernanda Nascimento; George Silva; Karina Pessoa; Luciana Gonçalves; Maria Júlia Brandão; Matilde Mejía; Michele Jesus; Valdinete Nascimento; Victor Souza; Ágatha Costa
EPI_ISL_2466190, EPI_ISL_2466191, EPI_ISL_2466192, EPI_ISL_2466193, EPI_ISL_2466194, EPI_ISL_2466195, EPI_ISL_2466196, EPI_ISL_2466197, EPI_ISL_2466198, EPI_ISL_2466199			
see above	Laboratório de Biologia Molecular de Doenças Infecciosas e do Câncer (LADIC - UFRN)	Laboratory of Respiratory Viruses and Measles, Oswaldo Cruz Institute, FIOCRUZ	Alice Sampaio Rocha; Ana Carolina Mendonca; Anna Carolina Paixao; Elisa Cavalcante Pereira; Fernando Motta; Josélio Araújo; Luciana Appolinario; Marilda Siqueira on behalf of the Fiocruz COVID-19 Genomic Surveillance Network; Paola Resende; Renata Serrano Lopes; Taina Venas
EPI_ISL_2731508, EPI_ISL_2731509, EPI_ISL_2731513, EPI_ISL_2731514, EPI_ISL_2731516, EPI_ISL_2731517, EPI_ISL_2731519, EPI_ISL_2731520, EPI_ISL_2731521, EPI_ISL_2731524, EPI_ISL_2731526, EPI_ISL_2731527, EPI_ISL_2731528, EPI_ISL_2731529, EPI_ISL_2731530, EPI_ISL_2731531, EPI_ISL_2731532, EPI_ISL_2731533, EPI_ISL_2731536, EPI_ISL_2731538, EPI_ISL_2731540, EPI_ISL_2731542, EPI_ISL_2731544, EPI_ISL_2731545, EPI_ISL_2731546, EPI_ISL_2731548, EPI_ISL_2731549, EPI_ISL_2731551, EPI_ISL_2731553, EPI_ISL_2731554, EPI_ISL_2731555, EPI_ISL_2731556, EPI_ISL_2731557, EPI_ISL_2731558, EPI_ISL_2731559, EPI_ISL_2731567, EPI_ISL_2731568, EPI_ISL_2731569, EPI_ISL_2731575, EPI_ISL_2731578, EPI_ISL_2731580, EPI_ISL_2731581, EPI_ISL_2731582, EPI_ISL_2731584, EPI_ISL_2731585, EPI_ISL_2731586, EPI_ISL_2731588, EPI_ISL_2731589, EPI_ISL_2731590, EPI_ISL_2731591, EPI_ISL_2731593, EPI_ISL_2731595, EPI_ISL_2731598, EPI_ISL_2731599, EPI_ISL_2731600, EPI_ISL_2731601, EPI_ISL_2731602, EPI_ISL_2731603, EPI_ISL_2731604, EPI_ISL_2731610, EPI_ISL_2731611, EPI_ISL_2731612, EPI_ISL_2731613, EPI_ISL_2731614, EPI_ISL_2731615, EPI_ISL_2731616, EPI_ISL_2731617, EPI_ISL_2731618, EPI_ISL_2731620, EPI_ISL_2731621, EPI_ISL_2731622, EPI_ISL_2731632, EPI_ISL_2731634, EPI_ISL_2731640, EPI_ISL_2731642, EPI_ISL_2731643, EPI_ISL_2731644, EPI_ISL_2731645, EPI_ISL_2731646, EPI_ISL_2731649, EPI_ISL_2731650, EPI_ISL_2731651, EPI_ISL_2731654, EPI_ISL_2731655, EPI_ISL_2731656, EPI_ISL_2731660, EPI_ISL_2731663, EPI_ISL_2731664, EPI_ISL_2731665, EPI_ISL_2731667, EPI_ISL_2731668, EPI_ISL_2731669, EPI_ISL_2731671, EPI_ISL_2731672, EPI_ISL_2731673, EPI_ISL_2731675, EPI_ISL_2731676, EPI_ISL_2731677, EPI_ISL_2790137, EPI_ISL_2790139, EPI_ISL_2790140, EPI_ISL_2790144, EPI_ISL_2790145, EPI_ISL_2790146, EPI_ISL_2790147, EPI_ISL_2790148, EPI_ISL_2790149, EPI_ISL_2790150, EPI_ISL_2790151, EPI_ISL_2790152, EPI_ISL_2790153, EPI_ISL_2790154, EPI_ISL_2790155, EPI_ISL_2790156, EPI_ISL_2790157, EPI_ISL_2790158, EPI_ISL_2790159, EPI_ISL_2790165, EPI_ISL_2790166, EPI_ISL_2790168, EPI_ISL_2790169, EPI_ISL_2790171, EPI_ISL_2790172, EPI_ISL_2790173, EPI_ISL_2790174, EPI_ISL_2835107, EPI_ISL_2835108, EPI_ISL_2835110, EPI_ISL_2835111, EPI_ISL_2835112, EPI_ISL_2835113, EPI_ISL_2835116, EPI_ISL_2835118, EPI_ISL_2835119, EPI_ISL_2835120, EPI_ISL_2835124, EPI_ISL_2835125, EPI_ISL_2835126, EPI_ISL_2835129, EPI_ISL_2835130, EPI_ISL_2835132, EPI_ISL_2835135, EPI_ISL_2835141, EPI_ISL_2835145, EPI_ISL_2835146, EPI_ISL_2835147, EPI_ISL_2835151, EPI_ISL_2835152, EPI_ISL_2835153, EPI_ISL_2835154, EPI_ISL_2835155, EPI_ISL_2835156, EPI_ISL_2835157, EPI_ISL_2835159, EPI_ISL_2835160, EPI_ISL_2835161, EPI_ISL_2835162, EPI_ISL_2835163, EPI_ISL_2835164, EPI_ISL_2835165, EPI_ISL_2835167, EPI_ISL_2835171, EPI_ISL_2835172, EPI_ISL_2835174, EPI_ISL_2835175, EPI_ISL_2835176, EPI_ISL_2835177, EPI_ISL_2835178, EPI_ISL_2835179, EPI_ISL_2835180, EPI_ISL_2835181, EPI_ISL_2835182, EPI_ISL_2835183, EPI_ISL_2835184, EPI_ISL_2835188, EPI_ISL_2835189, EPI_ISL_2835191, EPI_ISL_2835192, EPI_ISL_2835193, EPI_ISL_2835194, EPI_ISL_2835196, EPI_ISL_2835198, EPI_ISL_2835202, EPI_ISL_2835203, EPI_ISL_2835207, EPI_ISL_2835208, EPI_ISL_2835214, EPI_ISL_2983471			
see above	Laboratório de Biotecnologia Aplicada (LBA) - Laboratório de Biologia Molecular - Hospital das Clínicas, Faculdade de	Laboratory of Respiratory Viruses and Measles, Oswaldo Cruz Institute, FIOCRUZ	Alice Sampaio Rocha; Ana Carolina Mendonca; Anna Carolina Paixao; Elisa Cavalcante Pereira; Felipe Allan da Silva Costa; Fernando Motta; Jayme Augusto de Souza Neto; Leonardo Nazario de Moraes; Luciana Appolinario; Marilda Siqueira on behalf of the Fiocruz COVID-19 Genomic Surveillance Network; Paola Resende; Patricia Akemi Assato; Rejane Maria Tommasini; Renata Serrano Lopes; Taina Venas

	Medicina de Botucatu, Departamento de Bioprocessos e Biotecnologia - Faculdade de Ciências Agronômicas, UNESP – Botucatu/SP		
EPI_ISL_2375845, EPI_ISL_2382535, EPI_ISL_2382536, EPI_ISL_3873650, EPI_ISL_3873656, EPI_ISL_3873658, EPI_ISL_3873660, EPI_ISL_4051953, EPI_ISL_4051955			
see above	Laboratório de Microbiologia Molecular - Universidade FEEVALE	Molecular Microbiology Laboratory	Alana Witt Hansen; Fernando Rosado Spilki; Flávio Silveira; Fágner Henrique Heldt; Juliana Schons Gularte; Juliane Deise Fleck; Mariana Soares da Silva; Matheus Nunes Weber; Meriane Demoliner; Micheli Filippi; Micheli Filippi.; Paula Rodrigues de Almeida; Vyctoria Malayhka de Abreu Góes Pereira.
EPI_ISL_2629833, EPI_ISL_2629834, EPI_ISL_2629835, EPI_ISL_2629838	Laboratório de Virologia Molecular - Universidade Federal do Rio de Janeiro	Laboratório de Virologia Molecular - Universidade Federal do Rio de Janeiro	; Alice Laschuk Herlinger; Amilcar Tanuri; André Felipe Andrade dos Santos; Carolina Moreira Voloch; Cássia Cristina Alves Gonçalves; Diana Mariani; Débora Souza Faffe; Filipe Romero Rebello Moreira; Francine Bittencourt Schiffler; Isabela de Carvalho Leitão; Marcelo Calado de Paula Tôrres; Matheus Augusto Calvano Cosentino; Mirela D’arc; Orlando da Costa Ferreira Junior; Rafael Mello Galliez; Raíssa Mirella dos Santos Cunha da Costa; Renato Santana de Aguiar; Terezinha Marta Pereira Pinto Castineiras; Thamiris dos Santos Miranda; Átila Duque Rossi
EPI_ISL_2112830	Labormedizin Darmstadt	Robert Koch Institute	
EPI_ISL_2443529, EPI_ISL_2443675, EPI_ISL_2443676, EPI_ISL_2443678, EPI_ISL_2443679, EPI_ISL_2443680, EPI_ISL_2443681, EPI_ISL_2443682, EPI_ISL_2443683, EPI_ISL_2443708, EPI_ISL_2443710, EPI_ISL_2466260, EPI_ISL_2536227, EPI_ISL_2536229, EPI_ISL_2536230, EPI_ISL_2536231, EPI_ISL_2536232, EPI_ISL_2536233, EPI_ISL_2536234, EPI_ISL_2536235, EPI_ISL_2536236, EPI_ISL_2536237, EPI_ISL_2536238, EPI_ISL_2536239, EPI_ISL_2536240, EPI_ISL_2536241, EPI_ISL_2536242, EPI_ISL_2536243, EPI_ISL_2536244, EPI_ISL_2536245, EPI_ISL_2536246, EPI_ISL_2536247, EPI_ISL_2536248, EPI_ISL_2536249, EPI_ISL_2536250, EPI_ISL_2536251, EPI_ISL_2536252, EPI_ISL_2536253, EPI_ISL_2536254, EPI_ISL_2536255, EPI_ISL_2536256, EPI_ISL_2536257, EPI_ISL_2536258, EPI_ISL_2536259, EPI_ISL_2536260, EPI_ISL_2536261, EPI_ISL_2536263, EPI_ISL_2557414, EPI_ISL_2557416, EPI_ISL_2557417, EPI_ISL_2557418, EPI_ISL_2557419, EPI_ISL_2557420, EPI_ISL_2557421, EPI_ISL_3061899			
see above	Labortorio Central de Saude Publica do Estado de Santa Catarina (LACEN/SC)	Laboratory of Respiratory Viruses and Measles, Oswaldo Cruz Institute, FIOCRUZ	Alice Sampaio Rocha; Ana Carolina Mendonca; Anna Carolina Paixao; Darcita Buerger Rovaris; Elisa Cavalcante Pereira; Fernando Motta; Luciana Appolinario; Marilda Siqueira on behalf of the Fiocruz COVID-19 Genomic Surveillance Network; Paola Resende; Renata Serrano Lopes; Sandra Bianchini Fernandes; Taina Venas
EPI_ISL_2196333, EPI_ISL_2196334, EPI_ISL_2274098, EPI_ISL_2443639, EPI_ISL_2443640, EPI_ISL_2443641, EPI_ISL_2443642, EPI_ISL_2443643, EPI_ISL_2443644, EPI_ISL_2443645, EPI_ISL_2443646, EPI_ISL_2443647, EPI_ISL_2443648, EPI_ISL_2443649, EPI_ISL_2443650, EPI_ISL_2443651, EPI_ISL_2443652, EPI_ISL_2443653, EPI_ISL_2443654, EPI_ISL_2443655, EPI_ISL_2443656, EPI_ISL_2443657, EPI_ISL_2443658, EPI_ISL_2466230, EPI_ISL_2536275, EPI_ISL_2536276, EPI_ISL_2536277, EPI_ISL_2536278, EPI_ISL_2536279, EPI_ISL_2536281, EPI_ISL_2536282, EPI_ISL_2536283, EPI_ISL_2536284, EPI_ISL_2536285, EPI_ISL_2536286, EPI_ISL_2536287, EPI_ISL_2536288, EPI_ISL_2536289, EPI_ISL_2536290, EPI_ISL_2536291, EPI_ISL_2536292, EPI_ISL_2536293, EPI_ISL_2536294, EPI_ISL_2536295, EPI_ISL_2536296, EPI_ISL_2536297, EPI_ISL_2536298, EPI_ISL_2536299, EPI_ISL_2536300, EPI_ISL_2536301, EPI_ISL_2536302, EPI_ISL_2557376, EPI_ISL_2557377, EPI_ISL_2557378, EPI_ISL_2557379, EPI_ISL_2557380, EPI_ISL_2557381, EPI_ISL_2557382, EPI_ISL_2557383, EPI_ISL_2557384, EPI_ISL_2557404, EPI_ISL_2557406, EPI_ISL_3061894, EPI_ISL_3061895, EPI_ISL_3061897			
see above	Labortorio Central de Saude Publica do Estado do Rio de Janeiro (LACEN/RJ)	Laboratory of Respiratory Viruses and Measles, Oswaldo Cruz Institute, FIOCRUZ	Alice Sampaio Rocha; Ana Carolina Mendonca; Andrea Cony Cavalcanti; Anna Carolina Paixao; Elisa Cavalcante Pereira; Fernando Motta; Luciana Appolinario; Marilda Siqueira on behalf of the Fiocruz COVID-19 Genomic Surveillance Network; Paola Resende; Renata Serrano Lopes; Taina Venas
EPI_ISL_2645510	Labortorio Central de Saude Publica do Estado do Tocantins (LACEN/TO)	Laboratory of Respiratory Viruses and Measles, Oswaldo Cruz Institute, FIOCRUZ	Alice Sampaio Rocha; Ana Carolina Mendonca; Anna Carolina Paixao; Elisa Cavalcante Pereira; Fernando Motta; Jucimaria Dantas Galvao; Luciana Appolinario; Marilda Siqueira on behalf of the Fiocruz COVID-19 Genomic Surveillance Network; Paola Resende; Renata Serrano Lopes; Taina Venas
EPI_ISL_2443528, EPI_ISL_2443543, EPI_ISL_2443544, EPI_ISL_2443547, EPI_ISL_2443548, EPI_ISL_2443549, EPI_ISL_2443550, EPI_ISL_2443554, EPI_ISL_2443573, EPI_ISL_2443574, EPI_ISL_2443575, EPI_ISL_2443576, EPI_ISL_2557359, EPI_ISL_2557361, EPI_ISL_2557363, EPI_ISL_2557364, EPI_ISL_2557365, EPI_ISL_2557366, EPI_ISL_2557367, EPI_ISL_2557368, EPI_ISL_2557369, EPI_ISL_2557370, EPI_ISL_2557371, EPI_ISL_2557372, EPI_ISL_2557373, EPI_ISL_2557375, EPI_ISL_2603430, EPI_ISL_2603439, EPI_ISL_2603454, EPI_ISL_2603455, EPI_ISL_2603460, EPI_ISL_2603461, EPI_ISL_2603464, EPI_ISL_2603466, EPI_ISL_2603470, EPI_ISL_2645440, EPI_ISL_2645441, EPI_ISL_2645442, EPI_ISL_2645443, EPI_ISL_2863682, EPI_ISL_2863684, EPI_ISL_2863685, EPI_ISL_2863687, EPI_ISL_2863725, EPI_ISL_2863795, EPI_ISL_2863855, EPI_ISL_2863856, EPI_ISL_2863860, EPI_ISL_2863861, EPI_ISL_2863862, EPI_ISL_2863863, EPI_ISL_2863864, EPI_ISL_2863865, EPI_ISL_2863866, EPI_ISL_2863867, EPI_ISL_2863868, EPI_ISL_2863869, EPI_ISL_2863870, EPI_ISL_2863871, EPI_ISL_2863872, EPI_ISL_2863873, EPI_ISL_2863874, EPI_ISL_2863875, EPI_ISL_2863876, EPI_ISL_2863877, EPI_ISL_2863878, EPI_ISL_2863880, EPI_ISL_2863881, EPI_ISL_2863882, EPI_ISL_2863883, EPI_ISL_2863884, EPI_ISL_2863885, EPI_ISL_2863886, EPI_ISL_2863887, EPI_ISL_2863888, EPI_ISL_2863889, EPI_ISL_2863895, EPI_ISL_2863896			
see above	Lboratorio Central de Saude Publica do Estado do Parana (LACEN/PR)	Laboratory of Respiratory Viruses and Measles, Oswaldo Cruz Institute, FIOCRUZ	Agatha Cristinne Prudencio; Agatha Cristinne Prudencio Soares; Alice Sampaio Rocha; Ana Carolina Mendonca; Anna Carolina Paixao; Elisa Cavalcante Pereira; Fernando Motta; Ighor Leonardo Arantes Gomes; Irina Riediger; Luciana Appolinario; Marilda Siqueira on behalf of the Fiocruz COVID-19 Genomic Surveillance Network; Paola Resende; Renata Serrano Lopes; Taina Venas
EPI_ISL_2692136, EPI_ISL_2692138, EPI_ISL_2692139, EPI_ISL_2692140, EPI_ISL_2692141	Lifescan	RIPHL at Rush University Medical Center	Chicago Department of Public Health; Felix Araujo Perez; Kevin Kunstman; Laura Furtado; Marieta Hyde; Max Kolton; Stefan Green
EPI_ISL_2117614, EPI_ISL_2117967, EPI_ISL_2118029	Lighthouse Lab in Alderley Park	Wellcome Sanger Institute for the COVID-19 Genomics UK (COG-UK) Consortium	Cordelia Langford; David K. Jackson; Dominic Kwiatkowski; Ewan Harrison; Ian Johnston; Jacquelyn Wynn; Jeffrey Barrett; John Sillitoe on behalf of the Wellcome Sanger Institute COVID-19 Surveillance Team; Mairead Hyland; Roberto Amato; Sonia Goncalves; The Lighthouse Lab in Alderley Park and Alex Alderton
EPI_ISL_1740541, EPI_ISL_1740689	Lighthouse Lab in Cambridge	Wellcome Sanger Institute for the COVID-19 Genomics UK (COG-UK) Consortium	Cordelia Langford; David K. Jackson; Dominic Kwiatkowski; Ewan Harrison; Ian Johnston; Jeffrey Barrett; John Sillitoe on behalf of the Wellcome Sanger Institute COVID-19 Surveillance Team; Rob Howes; Roberto Amato; Sonia Goncalves; The Lighthouse Lab in Cambridge and Alex Alderton
EPI_ISL_1921451, EPI_ISL_1921677, EPI_ISL_2004797, EPI_ISL_2021062, EPI_ISL_2021666, EPI_ISL_2022037, EPI_ISL_2117733, EPI_ISL_2117840, EPI_ISL_2118938, EPI_ISL_2119812, EPI_ISL_2172155, EPI_ISL_2172194, EPI_ISL_2316530			
see above	Lighthouse Lab in Milton Keynes	Wellcome Sanger Institute for the COVID-19 Genomics UK (COG-UK) Consortium	Cordelia Langford; David K. Jackson; Dominic Kwiatkowski; Ewan Harrison; Ian Johnston; Jeffrey Barrett; John Sillitoe on behalf of the Wellcome Sanger Institute COVID-19 Surveillance Team; Roberto Amato; Sonia Goncalves; The Lighthouse Lab in Milton Keynes and Alex Alderton
EPI_ISL_2266523	Limbach - MVZ Humangenetik Ulm	Robert Koch Institute	
EPI_ISL_2373062, EPI_ISL_2373069, EPI_ISL_2373076, EPI_ISL_2373094, EPI_ISL_2373100, EPI_ISL_2373103, EPI_ISL_2373104, EPI_ISL_2373107, EPI_ISL_2842585, EPI_ISL_2842589			
see above	Lurie Children's Hospital of Chicago	Northwestern University - Ozer Lab	Egon A. Ozer; Judd F. Hultquist; Lacy M. Simons; Larry K. Kociolek; Michael G. Ison; Ramon Lorenzo-Redondo; Taylor J. Dean; William J. Muller; Xiaotian; Zheng
EPI_ISL_2249573, EPI_ISL_2249576	M Health Fairview	Minnesota Department of Health, Public Health Laboratory	Alexandra Lorentz; Jacob Garfin; Matt Plumb; and Xiong Wang
EPI_ISL_2451813	MA State Public Health Laboratory	Centers for Disease Control and Prevention Division of Viral Diseases, Pathogen Discovery	Alison Laufer Halpin; Ben L. Rambo-Martin; Clinton R. Paden; Dakota Howard; Darlene Wagner; Dave Wentworth; Dhwani Batra; Jasmine Padilla; Justin Lee; Katie Dillon; Krista Queen; Kristen Knipe; Kristine Lacek; Mark Burroughs; Matthew Schmerer; Mili Sheth; Peter Cook; Sam Shepard; Sarah Nobles; Shoshona Le; Suxiang Tong; Vivien Dugan; Yvette Unoarumhi
EPI_ISL_2801332, EPI_ISL_2801333	MATERNIDADE ESCOLA ASSIS CHATEAUBRIAND	Analytical Competence Molecular Epidemiology Lab/ACME, Oswaldo Cruz Foundation, Ceara (FIOCRUZ CE)	Cleber Furtado Aksenen e Suzana Porto Almeida; Fabio Miyajima; Fernando Braga Stehling; Francisco Eder de Moura Lopes; Jamille Maria Mendes Bezerra; Joaquim César do Nascimento Sousa Junior; Pedro Miguel Carneiro Jeronimo; Thais Ferreira de Oliveira; Thais de Oliveira Costa; Ticiane Cavalcante de Souza; Veridiana Pessoa Miyajima
EPI_ISL_1811581, EPI_ISL_2227429	MD PHL	MD PHL	Maryland Department of Health Laboratories Administration
EPI_ISL_2383931	ME Health and Environmental Testing Laboratory	Centers for Disease Control and Prevention Division of Viral Diseases, Pathogen Discovery	Alison Laufer Halpin; Ben L. Rambo-Martin; Clinton R. Paden; Dakota Howard; Darlene Wagner; Dave Wentworth; Dhwani Batra; Jasmine Padilla; Justin Lee; Katie Dillon; Krista Queen; Kristen Knipe; Kristine Lacek; Mark Burroughs; Matthew Schmerer; Mili Sheth; Peter Cook; Sam Shepard; Sarah Nobles; Shoshona Le; Suxiang Tong; Vivien Dugan; Yvette Unoarumhi
EPI_ISL_2453794, EPI_ISL_2453796, EPI_ISL_2453806, EPI_ISL_2453816	MEPHI, Aix Marseille University	MEPHI, Aix Marseille University	Anthony LEVASSEUR
EPI_ISL_2451618	MI - Michigan Department of Health and Human Services - Bureau of Laboratories	Centers for Disease Control and Prevention Division of Viral Diseases, Pathogen Discovery	Alison Laufer Halpin; Ben L. Rambo-Martin; Clinton R. Paden; Dakota Howard; Darlene Wagner; Dave Wentworth; Dhwani Batra; Jasmine Padilla; Justin Lee; Katie Dillon; Krista Queen; Kristen Knipe; Kristine Lacek; Mark Burroughs; Matthew Schmerer; Mili Sheth; Peter Cook; Sam Shepard; Sarah Nobles; Shoshona Le; Suxiang Tong; Vivien Dugan; Yvette Unoarumhi
EPI_ISL_2229261, EPI_ISL_3354883	MO State Public Health Laboratory	Centers for Disease Control and Prevention Division of Viral Diseases, Pathogen Discovery	Alex Burgin; Alison Laufer Halpin; Ben L. Rambo-Martin; Ben Rambo-Martin; Clinton Paden; Clinton R. Paden; Dakota Howard; Darlene Wagner; Dave Wentworth; Dhwani Batra; Jasmine Padilla; Justin Lee; Katie Dillon; Krista Queen; Kristen Knipe; Kristine Lacek; Mark Burroughs; Matthew Schmerer; Meghan Bentz; Mili Sheth; Peter Cook; Sam Shepard; Sarah Nobles; Shoshona Le; Suxiang Tong; Vivien Dugan; Yvette Unoarumhi
EPI_ISL_2444203, EPI_ISL_2444210	MONTE APRAZIVEL	Instituto Butantan / FAMERP	Antonio Jorge Martins; Claudia Renata dos Santos Barros; David Schlesinger; Debora Botequio Moretti; Dimas Tadeu Covas; Elaine Cristina Marqueze; Elaine Vieira Santos; Evandra Strazza Rodrigues; Heidge Fukumasu; Jayme Augusto de Souza-Neto; José Salvatore Leister Patané; Luiz Alcantara; Luiz Lehmann Coutinho; Maria Carolina Elias; Maurício Lacerda Nogueira; Rafael dos Santos Bezerra; Raul Machado Neto; Rejane Maria Tommasini Grotto; Ricardo Haddad; Sandra Coccuzzo Sampaio Vessoni; Simone Kashima; Svetoslav Nanev Slavov; Vincent Louis Viala
EPI_ISL_2274608	MONTEFIORE MEDICAL CENTER LABORATORIES	Wadsworth Center, New York State Department of Health	Alexis Russell; Catharine Prussing; Daryl M. Lamson; Erasmus Schneider; Erica Lasek-Nesselquist; John Kelly; Jonathan Plitnick; Kirsten St. George; Matthew Shudt; Melissa A Leisner; Navjot Singh
EPI_ISL_2451640	MT Public Health Laboratory	Centers for Disease Control and Prevention Division of Viral Diseases, Pathogen Discovery	Alison Laufer Halpin; Ben L. Rambo-Martin; Clinton R. Paden; Dakota Howard; Darlene Wagner; Dave Wentworth; Dhwani Batra; Jasmine Padilla; Justin Lee; Katie Dillon; Krista Queen; Kristen Knipe; Kristine Lacek; Mark Burroughs; Matthew Schmerer; Mili Sheth; Peter Cook; Sam Shepard; Sarah Nobles; Shoshona Le; Suxiang Tong; Vivien Dugan; Yvette Unoarumhi
EPI_ISL_2111276	MVZ Labor Dr. Fenner und Kollegen (Standort Hamburg)	Robert Koch Institute	
EPI_ISL_1849414, EPI_ISL_1849483	MVZ Labor Krone GbR	Robert Koch Institute	
EPI_ISL_2314745	MVZ für Laboratoriumsmedizin und Mikrobiologie Koblenz-Mittelrhein (Labor Koblenz)	Robert Koch Institute	
EPI_ISL_2101010, EPI_ISL_2101014, EPI_ISL_2501190	Maine HETL	Health and Environmental Testing Laboratory	Barter, M.; Dewey, H.; Grieser, H.; H. and Tewhey, R.; Lynch, R.; Martha, J.; Matluk, N.; Meak, S.; Munger
EPI_ISL_1826331, EPI_ISL_1826332, EPI_ISL_2245512, EPI_ISL_2245527, EPI_ISL_2245568, EPI_ISL_2245596, EPI_ISL_2802401, EPI_ISL_2802402, EPI_ISL_2802436, EPI_ISL_2802525, EPI_ISL_2802526, EPI_ISL_2802527, EPI_ISL_2802528, EPI_ISL_2802529, EPI_ISL_2802530, EPI_ISL_2802531, EPI_ISL_2802532, EPI_ISL_2802691, EPI_ISL_3298815, EPI_ISL_3298842, EPI_ISL_3298847, EPI_ISL_3298856,			

EPI_ISL_3298857, EPI_ISL_3298874, EPI_ISL_3298878, EPI_ISL_3298879, EPI_ISL_3298890, EPI_ISL_4083775, EPI_ISL_4083810			
see above	Maine Health and Environmental Testing Laboratory	Tewhey Lab, The Jackson Laboratory	Barter, M.; Dewey, H.; H. and Tewhey, R.; Iosue, F.; Lynch, R.; Matluk, N.; Munger
EPI_ISL_2285048, EPI_ISL_2307182, EPI_ISL_2307204, EPI_ISL_2307272, EPI_ISL_2307289, EPI_ISL_2441051, EPI_ISL_2450966, EPI_ISL_2450988, EPI_ISL_2451021, EPI_ISL_2451027, EPI_ISL_2451029, EPI_ISL_2451088, EPI_ISL_2451212, EPI_ISL_2488897, EPI_ISL_2488952, EPI_ISL_2488979, EPI_ISL_2489013, EPI_ISL_2489040, EPI_ISL_2489042, EPI_ISL_2525127, EPI_ISL_2525324, EPI_ISL_2525355, EPI_ISL_2646386, EPI_ISL_2646418, EPI_ISL_2646444, EPI_ISL_2646447, EPI_ISL_2646459, EPI_ISL_2646463, EPI_ISL_2646464, EPI_ISL_2646472, EPI_ISL_2646474, EPI_ISL_2646505, EPI_ISL_2646507, EPI_ISL_2646516, EPI_ISL_2646523, EPI_ISL_2646573, EPI_ISL_2646578, EPI_ISL_2646619, EPI_ISL_2646685, EPI_ISL_2646692, EPI_ISL_2646724, EPI_ISL_2646732, EPI_ISL_2646801, EPI_ISL_2646872, EPI_ISL_2646888, EPI_ISL_2646959, EPI_ISL_2647100, EPI_ISL_2647303, EPI_ISL_2647314, EPI_ISL_2647321, EPI_ISL_2647323, EPI_ISL_2647325, EPI_ISL_2647376, EPI_ISL_2647434, EPI_ISL_2647439, EPI_ISL_2647451, EPI_ISL_2647917, EPI_ISL_2647965, EPI_ISL_2786548			
see above	Mako Medical	Centers for Disease Control and Prevention Division of Viral Diseases, Pathogen Discovery	Adrian Paskey; Benjamin Rambo-Martin; Christopher Gulvick; Clinton R. Paden; Dakota Howard; Darlene Wagner; Dhwani Batra; Duncan MacCannell; Jason Caravas; Kara Moser; Lauren Moon; Matthew Schmerer; Matthew Tugwell; Peter W. Cook; Scott Sammons; Shatavia Morrison; Yvette Unoarumhi
EPI_ISL_2501831, EPI_ISL_2598490, EPI_ISL_3011863	Maryland Genomics, Institute for Genome Sciences, University of Maryland School of Medicine	Maryland Genomics, Institute for Genome Sciences, University of Maryland School of Medicine	Claire M; Fraser; Hazen; Holly; Humphrys; Ivette; Jacques; Jonathan; Kranthi; Lim; Lisa D; Luke J; Mike; Ott; Ravel; Roussey; Sadzewicz; Sandra; Santana-Cruz; Tallon; Tracy; Vavikolanu
EPI_ISL_2550760	Maryland Public Health Laboratory (MD PHL)	Maryland Public Health Laboratory (MD PHL)	Maryland Department of Health Laboratories Administration
EPI_ISL_2016473, EPI_ISL_2016481, EPI_ISL_2016483, EPI_ISL_2362372	Massachusetts General Hospital	Infectious Disease Program, Broad Institute of Harvard and MIT	A.E.; Adams, G.; Anahtar, M.; B.L.; B.W.; Bauer, M.; Birren; Branda, J.; Carter, A.; Cerrato, F.; Chaluvadi, S.; Chapman; Cusick, C.; D.J.; DeRuff, K.; E. and Sabeti; Flowers, K.; Gallagher, G.; Gladden-Young, A.; Gnirke, A.; Harris, J.; J.E.; K.J.; LaRocque, R.; Lagerborg, K.; Lemieux; Lin; Loreth, C.; MacInnis; Neumann, A.; Normandin, E.; P.C.; Park; Pierce, V.; Reilly, S.; Rosenberg; Rudy, M.; Ryan, E.; S.B.; Shaw, B.; Siddle; Slater, D.; Smole, S.; Tomkins-Tinch, C.; Turbett, S.
EPI_ISL_2008341, EPI_ISL_2008344, EPI_ISL_2229517, EPI_ISL_2229808, EPI_ISL_2400245, EPI_ISL_2483777, EPI_ISL_2483781, EPI_ISL_2483790, EPI_ISL_2483799, EPI_ISL_2483801, EPI_ISL_2483803, EPI_ISL_2484937			
see above	Massachusetts State Public Health Laboratory	Massachusetts State Public Health Laboratory	Andrew Lang; Glen Gallagher; Sandra Smole; Timelia Fink
EPI_ISL_2136412	Mayo Clinic & Mayo Clinic Laboratories	Minnesota Department of Health, Public Health Laboratory	Alexandra Lorentz; Jacob Garfin; Matt Plumb; and Xiong Wang
EPI_ISL_2389368	Med. Labor Prof. Schenk Dr. Ansorge & Kollegen	Robert Koch Institute	
EPI_ISL_1999698, EPI_ISL_1999734, EPI_ISL_1999766, EPI_ISL_1999797, EPI_ISL_2433981, EPI_ISL_2434027, EPI_ISL_2434039			
see above	Microbiologia CATLAB	Can Ruti SARS-CoV-2 Sequencing Hub (HUGTiP/IrsiCaixa/IGTP)	Alba Sánchez; Anna Not; Antoni E Bordoy; Bonaventura Clotet; Cristina Casañ; Cristina Esteban; Francesc Catala-Moll; Gemma Clara; Ignacio Blanco; Marc Noguera-Julian; Maria Casadellà; Mariona Parera; Mercedes Guerrero; Montserrat Giménez; Pere-Joan Cardona; Pilar Armengol; Roger Paredes; Verónica Saludes; and Elisa Martró on behalf of the Can Ruti SARS-CoV-2 Sequencing Hub.
EPI_ISL_1892991, EPI_ISL_2000122, EPI_ISL_2136876, EPI_ISL_2136877, EPI_ISL_2136900, EPI_ISL_2136912, EPI_ISL_2136924, EPI_ISL_2136947			
see above	Microbiologia CATLAB	Can Ruti SARS-CoV-2 Sequencing Hub (HUGTiP/IrsiCaixa/IGTP)	Alba Sánchez; Anna Not; Antoni E Bordoy; Bonaventura Clotet; Cristina Casañ; Cristina Esteban; Francesc Catala-Moll; Gemma Clara; Ignacio Blanco; Marc Noguera-Julian; Maria Casadellà; Mariona Parera; Mercedes Guerrero; Montserrat Giménez; Pere-Joan Cardona; Pilar Armengol; Roger Paredes; Verónica Saludes; and Elisa Martró on behalf of the Can Ruti SARS-CoV-2 Sequencing Hub.
EPI_ISL_3138668	Microbiology Department - University Hospital Brussel	Microbiology Department - University Hospital Brussel	Thomas Demuyser
EPI_ISL_2140625, EPI_ISL_2372690, EPI_ISL_2448032	Microbiology Department, Laboratori Clínic Metropolitana Nord. Hospital Universitari Germans Trias i Pujol	Can Ruti SARS-CoV-2 Sequencing Hub (HUGTiP/IrsiCaixa/IGTP)	Alba Snchez; Alba Sánchez; Anna Not; Antoni E Bordoy; Bonaventura Clotet; Cristina Casa; Cristina Casañ; Cristina Esteban; Francesc Catala-Moll; Gemma Clara; Ignacio Blanco; Marc Noguera-Julian; Maria Casadell; Maria Casadellà; Mariona Parera; Mercedes Guerrero; Montserrat Gimnez; Montserrat Giménez; Pere-Joan Cardona; Pilar Armengol; Roger Pared; Roger Paredes; Vernica Saludes; Verónica Saludes; and Elisa Martr on behalf of the Can Ruti SARS-CoV-2 Sequencing Hub.; and Elisa Martró on behalf of the Can Ruti SARS-CoV-2 Sequencing Hub.
EPI_ISL_1831601, EPI_ISL_1831662, EPI_ISL_1892968, EPI_ISL_1893046, EPI_ISL_2380035, EPI_ISL_2380037, EPI_ISL_2380046			
see above	Microbiology Department, Laboratori Clínic Metropolitana Nord. Hospital Universitari Germans Trias i Pujol.	Can Ruti SARS-CoV-2 Sequencing Hub (HUGTiP/IrsiCaixa/IGTP)	Alba Sánchez; Anna Not; Antoni E Bordoy; Bonaventura Clotet; Cristina Casañ; Cristina Esteban; Francesc Catala-Moll; Gemma Clara; Ignacio Blanco; Marc Noguera-Julian; Maria Casadellà; Mariona Parera; Mercedes Guerrero; Montserrat Giménez; Pere-Joan Cardona; Pilar Armengol; Roger Pared; Roger Paredes; Verónica Saludes; and Elisa Martró on behalf of the Can Ruti SARS-CoV-2 Sequencing Hub.
EPI_ISL_2036092, EPI_ISL_2325183, EPI_ISL_2502699	Microbiology Department, University Hospital Donostia	Microbiology Department, University Hospital Donostia	Cilla G.; Gomez M; Grandioso D; Marimon JM; Martin-Peñaranda T; Montes M; Piñeiro L; Sorarrain A
EPI_ISL_2086155, EPI_ISL_2086156, EPI_ISL_2086157, EPI_ISL_2086158, EPI_ISL_2086159, EPI_ISL_2086163, EPI_ISL_2086164, EPI_ISL_2086165, EPI_ISL_2086171, EPI_ISL_2086172, EPI_ISL_2086173, EPI_ISL_2967793, EPI_ISL_2967838			
see above	Microbiology Department. Complexo Hospitalario Universitario de Vigo	Microbiology Department. Complexo Hospitalario Universitario de Vigo	Alfaya N; Alonso I; Alvarez M; Cabrera JJ; Carballo R; Cores O; Cortizo S; Davina C; Martinez L; Mediero G; Perez S; Potel C; Regueiro B; Rey S; Vasallo FJ; del-Campo V
EPI_ISL_2312976	Microbiology Division, SC DHEC	Microbiology Division, SC DHEC	Flores, H.; Freeman, J.
EPI_ISL_2029100, EPI_ISL_2029101, EPI_ISL_2259193	Microvida	Microvida	Jaco Verweij; Joep Stohr; Joep Stöhr; Suzan D. Pas
EPI_ISL_2406491	Middlemore Hospital	Institute of Environmental Science and Research (ESR)	Anja Werno; Antje van der Linden; Arlo Upton; Chris Mansell; David Hammer; Dragana Drinkovic; Erasmus Smit; Gary McAuliffe; Hana Sofia Andersson; Hermes Perez; James Ussher; Jill Sherwood; Jing Wang; Joep de Ligt; Josh Freeman; Julia Howard; Juliet Elvy; Lauren Jelly; Mary DeAlmeida; Matt Blakiston; Matt Storey; Matthew Rogers; Max Bloomfield; Michael Addidle; Michelle Balm; Muhammad Faisal; Nikki Freed; Olin Silander; Olivia Stroeven; Rachel Boyle; Sally Roberts; SallyAnn Harbison; Sarah Jefferies; Sharmini Muttaiyah; Susan Morpeth; Susan Taylor; Timothy Blackmore; Vani Sathyendran; Veronica Playle; Virginia Hope; Xiaoyun Ren
EPI_ISL_2092803, EPI_ISL_2136384, EPI_ISL_2136395, EPI_ISL_2136397, EPI_ISL_2136426, EPI_ISL_2178660, EPI_ISL_2244913, EPI_ISL_2249519, EPI_ISL_2249537, EPI_ISL_2249545, EPI_ISL_2249547, EPI_ISL_2249548, EPI_ISL_2325100, EPI_ISL_2332698, EPI_ISL_2403691, EPI_ISL_2425394, EPI_ISL_2658542			
see above	Minnesota Department of Health, Public Health Laboratory	Minnesota Department of Health, Public Health Laboratory	Alexandra Lorentz; Jacob Garfin; Matt Plumb; and Xiong Wang
EPI_ISL_2035571, EPI_ISL_2035591, EPI_ISL_2158679, EPI_ISL_2158682, EPI_ISL_2158684, EPI_ISL_2158690, EPI_ISL_2304242, EPI_ISL_2304250, EPI_ISL_2304260, EPI_ISL_2501926			
see above	Missouri State Public Health Laboratory	Missouri State Public Health Laboratory	Ashley New; Joshua Barry; Matthew Sinn
EPI_ISL_2105943, EPI_ISL_2105944, EPI_ISL_2105945, EPI_ISL_2105949, EPI_ISL_2105956, EPI_ISL_2107889	Molecular Diagnostics Pathology Department Mater Dei Hospital Malta	Molecular Diagnostics Pathology Department Mater Dei Hospital Malta	C Cilia; G Zahra; L Grech; R Borg
EPI_ISL_2646995, EPI_ISL_2941746, EPI_ISL_2941753	Montana Public Health Laboratory	Montana Public Health Laboratory	Carrie Biskupiak; Deborah Gibson; Joy Ritter; Michael Dills; Michelle Mozer
EPI_ISL_3355029	ND Dept. of Health Laboratory Services-Microbiology	Centers for Disease Control and Prevention Division of Viral Diseases, Pathogen Discovery	Alex Burgin; Ben Rambo-Martin; Clinton Paden; Dakota Howard; Dave Wentworth; Dhwani Batra; Jasmine Padilla; Justin Lee; Krista Queen; Kristen Knipe; Kristine Lacek; Mark Burroughs; Matthew Schmerer; Meghan Bentz; Mili Sheth; Peter Cook; Sam Shepard; Sarah Nobles; Suxiang Tong; Vivien Dugan; Yvette Unoarumhi
EPI_ISL_2384008, EPI_ISL_3354969	NH Dept. of Health and Human Services Public Health Labs	Centers for Disease Control and Prevention Division of Viral Diseases, Pathogen Discovery	Alex Burgin; Alison Laufer Halpin; Ben L. Rambo-Martin; Ben Rambo-Martin; Clinton Paden; Clinton R. Paden; Dakota Howard; Darlene Wagner; Dave Wentworth; Dhwani Batra; Jasmine Padilla; Justin Lee; Katie Dillon; Krista Queen; Kristen Knipe; Kristine Lacek; Mark Burroughs; Matthew Schmerer; Meghan Bentz; Mili Sheth; Peter Cook; Sam Shepard; Sarah Nobles; Shoshona Le; Suxiang Tong; Vivien Dugan; Yvette Unoarumhi
EPI_ISL_1969075, EPI_ISL_2185595, EPI_ISL_2281856, EPI_ISL_2557099	NJDOH, Public Health and Environmental Laboratories	NJ_PHEL	Allison Roder; Byeong Jeong; Chelsea San Filippo; Dana Woell; Jacquelyn Deverell; Lindsey Bodnar; Matthew Scarnati; Ryan Pachucki; Shiv K. Verma
EPI_ISL_2097278, EPI_ISL_2097289	NORTHWELL HEALTH LABORATORIES	Wadsworth Center, New York State Department of Health	Alexis Russell; Catharine Prussing; Daryl M. Lamson; Erasmus Schneider; Erica Lasek-Nesselquist; John Kelly; Jonathan Plitnick; Kirsten St. George; Matthew Shudt; Melissa A Leisner; Navjot Singh
EPI_ISL_2345165, EPI_ISL_2345174, EPI_ISL_2345176, EPI_ISL_2345177	NUCLEO DE ESPECIALIDADES EM SAUDE	Instituto Butantan / ESALQ-Piracicaba	Antonio Jorge Martins; Claudia Renata dos Santos Barros; David Schlesinger; Debora Botequio Moretti; Dimas Tadeu Covas; Elaine Cristina Marqueze; Elaine Vieira Santos; Evandra Strazza Rodrigues; Heidge Fukumasu; Jayme Augusto de Souza-Neto; José Salvatore Leister Patané; Luiz Alcantara; Luiz Lehmann Coutinho; Maria Carolina Elias; Maurício Lacerda Nogueira; Rafael dos Santos Bezerra; Raul Machado Neto; Rejane Maria Tommasini Grotto; Ricardo Haddad; Sandra Coccuzzo Sampaio Vessoni; Simone Kashima; Svetoslav Nanev Slavov; Vincent Louis Viala
EPI_ISL_2345800	NUCLEO DE SAUDE MARY DOTA DE BAURU	Instituto Butantan / Mendelics	Antonio Jorge Martins; Claudia Renata dos Santos Barros; David Schlesinger; Debora Botequio Moretti; Dimas Tadeu Covas; Elaine Cristina Marqueze; Elaine Vieira Santos; Evandra Strazza Rodrigues; Heidge Fukumasu; Jayme Augusto de Souza-Neto; José Salvatore Leister Patané; Luiz Alcantara; Luiz Lehmann Coutinho; Maria Carolina Elias; Maurício Lacerda Nogueira; Rafael dos Santos Bezerra; Raul Machado Neto; Rejane Maria Tommasini Grotto; Ricardo Haddad; Sandra Coccuzzo Sampaio Vessoni; Simone Kashima; Svetoslav Nanev Slavov; Vincent Louis Viala
EPI_ISL_2445138, EPI_ISL_2473751	NUCLEO DE SAUDE VILA FALCAO DE BAURU	Instituto Butantan	Antonio Jorge Martins; Claudia Renata dos Santos Barros; David Schlesinger; Debora Botequio Moretti; Dimas Tadeu Covas; Elaine Cristina Marqueze; Elaine Vieira Santos; Evandra Strazza Rodrigues; Heidge Fukumasu; Jayme Augusto de Souza-Neto; José Salvatore Leister Patané; Luiz Alcantara; Luiz Lehmann Coutinho; Maria Carolina Elias; Maurício Lacerda Nogueira; Rafael dos Santos Bezerra; Raul Machado Neto; Rejane Maria Tommasini Grotto; Ricardo Haddad; Sandra Coccuzzo Sampaio Vessoni; Simone Kashima; Svetoslav Nanev Slavov; Vincent Louis Viala
EPI_ISL_2345215, EPI_ISL_2345216,	NUCLEO DE SAUDE VILA FALCAO DE BAURU	Instituto Butantan / ESALQ-Piracicaba	Antonio Jorge Martins; Claudia Renata dos Santos Barros; David Schlesinger; Debora Botequio Moretti; Dimas Tadeu Covas; Elaine Cristina Marqueze; Elaine Vieira Santos; Evandra Strazza Rodrigues; Heidge Fukumasu; Jayme Augusto de Souza-Neto; José Salvatore Leister Patané; Luiz Alcantara; Luiz Lehmann Coutinho; Maria Carolina Elias; Maurício Lacerda Nogueira; Rafael dos Santos Bezerra; Raul Machado Neto; Rejane Maria Tommasini Grotto; Ricardo Haddad; Sandra Coccuzzo Sampaio Vessoni; Simone Kashima; Svetoslav Nanev Slavov; Vincent Louis Viala

EPI_ISL_2345217, EPI_ISL_2345218, EPI_ISL_2345242, EPI_ISL_2345263			
EPI_ISL_2345084, EPI_ISL_2345091, EPI_ISL_2345132, EPI_ISL_2345145, EPI_ISL_2345146, EPI_ISL_2345147, EPI_ISL_2345148, EPI_ISL_2345164			
see above	NUCLEO SAUDE III N HABITACIONAL PRESIDENTE GEISEL BAURU	Instituto Butantan / ESALQ-Piracicaba	Antonio Jorge Martins; Claudia Renata dos Santos Barros; David Schlesinger; Debora Botequio Moretti; Dimas Tadeu Covas; Elaine Cristina Marqueze; Elaine Vieira Santos; Evandra Strazza Rodrigues; Heidge Fukumasu; Jayme Augusto de Souza-Neto; José Salvatore Leister Patané; Luiz Alcantara; Luiz Lehmann Coutinho; Maria Carolina Elias; Maurício Lacerda Nogueira; Rafael dos Santos Bezerra; Raul Machado Neto; Rejane Maria Tommasini Grotto; Ricardo Haddad; Sandra Coccuzzo Sampaio Vessoni; Simone Kashima; Svetoslav Nanev Slavov; Vincent Louis Viala
EPI_ISL_2451588	NV-Southern Nevada Public Health Laboratory	Centers for Disease Control and Prevention Division of Viral Diseases, Pathogen Discovery	Alison Laufer Halpin; Ben L. Rambo-Martin; Clinton R. Paden; Dakota Howard; Darlene Wagner; Dave Wentworth; Dhwani Batra; Jasmine Padilla; Justin Lee; Katie Dillon; Krista Queen; Kristen Knipe; Kristine Lacek; Mark Burroughs; Matthew Schmerer; Mili Sheth; Peter Cook; Sam Shepard; Sarah Nobles; Shoshona Le; Suxiang Tong; Vivien Dugan; Yvette Unoarumhi
EPI_ISL_2098603, EPI_ISL_2098618, EPI_ISL_2274698	NYC Pandemic Response Lab	Wadsworth Center, New York State Department of Health	Alexis Russell; Catharine Prussing; Daryl M. Lamson; Erasmus Schneider; Erica Lasek-Nesselquist; John Kelly; Jonathan Plitnick; Kirsten St. George; Matthew Shudt; Melissa A Leisner; Navjot Singh
EPI_ISL_2018244	NYSDOH Wadsworth Center, Virology Lab	Centers for Disease Control and Prevention Division of Viral Diseases, Pathogen Discovery	Alison Laufer Halpin; Ben L. Rambo-Martin; Clinton R. Paden; Dakota Howard; Darlene Wagner; Dave Wentworth; Dhwani Batra; Jasmine Padilla; Justin Lee; Katie Dillon; Krista Queen; Kristen Knipe; Kristine Lacek; Mark Burroughs; Matthew Schmerer; Mili Sheth; Peter Cook; Sam Shepard; Sarah Nobles; Shoshona Le; Suxiang Tong; Vivien Dugan; Yvette Unoarumhi
EPI_ISL_2161718, EPI_ISL_2161747, EPI_ISL_2161775, EPI_ISL_2427404, EPI_ISL_2427406, EPI_ISL_2427408, EPI_ISL_2427410, EPI_ISL_2427432, EPI_ISL_2427464, EPI_ISL_2427468			
see above	NYU Langone Health	Departments of Pathology and Medicine, New York University School of Medicine	Adriana Heguy; Christian Marier; Dacia Dimartino; Emily Guzman; Gael Westby; Guiqing Wang; Paolo Cotzia; Paul Zappile; Peter Meyn; Sitharam Ramaswami; Yutong Zhang
EPI_ISL_2023408, EPI_ISL_2023409, EPI_ISL_2233081, EPI_ISL_2400270, EPI_ISL_2400274, EPI_ISL_2689668	Nebraska Public Health Laboratory	NPHL COVID-19 Response Team	NPHL COVID-19 Response Team
EPI_ISL_2277638	Nevada State Public Health Laboratory	Nevada State Public Health Laboratory	Andrew Gorzalski; Mark Pandori
EPI_ISL_1970970, EPI_ISL_2322768, EPI_ISL_2322770, EPI_ISL_2322789, EPI_ISL_2322792, EPI_ISL_2322848, EPI_ISL_2381909, EPI_ISL_2381943, EPI_ISL_2652062			
see above	New Mexico Department of Health Scientific Laboratory	New Mexico Department of Health Scientific Laboratory	Anastacia Griego-Fisher; D'eldra Malone; Ellie Johnson; Jennifer Benoit; Keila Gutierrez; Linda Salazar; Mark Willmon; Ratheesh Rajan
EPI_ISL_1920010	Northwell Health Laboratories	Wadsworth Center, New York State Department of Health	Alexis Russell; Catharine Prussing; Daryl M. Lamson; Erasmus Schneider; Erica Lasek-Nesselquist; John Kelly; Jonathan Plitnick; Kirsten St. George; Matthew Shudt; Melissa A Leisner; Navjot Singh
EPI_ISL_2490159, EPI_ISL_2490251, EPI_ISL_2692356, EPI_ISL_2692368	Northwest Laboratory	Seattle Flu Study	Amanda Adler; Barry R. Lutz; Benjamin Pelle; Brian Hiatt; Caitlin R. Wolf; Chris D. Frazar; Deborah A. Nickerson; Elisabeth Brandstetter; Erica Ryke; Geoff Melly; Helen Y. Chu; Janet A. Englund; Jay Shendure; Jover Lee; Kairsten Fay; Kirsten Lacombe; Lea M. Starita; Mark J. Rieder; Matthew Richardson; Matthew Thompson; Melissa Truong; Michael Boeckh; Michael Famulare; Misja Ilcisin; Peter D. Han; Philip Dykema; Romesh Gautom; Scott Lindquist; Thomas R. Sibley; Trevor Bedford
EPI_ISL_2009333, EPI_ISL_2009339, EPI_ISL_2009347, EPI_ISL_2009351, EPI_ISL_2009352, EPI_ISL_2372956, EPI_ISL_2372972, EPI_ISL_2372974, EPI_ISL_2372978, EPI_ISL_2372984, EPI_ISL_2372992, EPI_ISL_2372993, EPI_ISL_2373002, EPI_ISL_2373008, EPI_ISL_2373009, EPI_ISL_2373019, EPI_ISL_2373023, EPI_ISL_2373031, EPI_ISL_2373032, EPI_ISL_2373040, EPI_ISL_2373042			
see above	Northwestern Memorial Hospital	Northwestern University - Ozer Lab	Chad J. Achenbach; Chao Qi; Egon A. Ozer; Judd F. Hultquist; Lacy M. Simons; Lawrence J. Jennings; Michael G. Ison; Ramon Lorenzo-Redondo; Taylor J. Dean
EPI_ISL_2692034, EPI_ISL_2692045, EPI_ISL_2692046, EPI_ISL_3388716, EPI_ISL_3388717, EPI_ISL_3388718, EPI_ISL_3388722, EPI_ISL_3388802			
see above	Northwestern Memorial Hospital	RIPHL at Rush University Medical Center	Amber Kimble; Chao Qi; Edith Perez; Felix Araujo Perez; Kevin Kunstman; Laura Furtado; Marieta Hyde; Max Kolton; Stefan Green
EPI_ISL_2227690, EPI_ISL_2227691, EPI_ISL_2227692, EPI_ISL_2227693, EPI_ISL_2378934, EPI_ISL_2378977, EPI_ISL_2378981, EPI_ISL_2462076, EPI_ISL_2462090, EPI_ISL_2462091, EPI_ISL_2462105, EPI_ISL_2462117, EPI_ISL_2462120, EPI_ISL_2462135, EPI_ISL_2462136, EPI_ISL_2462137, EPI_ISL_2462138, EPI_ISL_2462140, EPI_ISL_2462147, EPI_ISL_2462153, EPI_ISL_2462173, EPI_ISL_2462179, EPI_ISL_2462182, EPI_ISL_2462184, EPI_ISL_2462185, EPI_ISL_2462187, EPI_ISL_2462198, EPI_ISL_2462202, EPI_ISL_2462221, EPI_ISL_2462223, EPI_ISL_2462230, EPI_ISL_2462231, EPI_ISL_2462232, EPI_ISL_2462241, EPI_ISL_2462253, EPI_ISL_2462263, EPI_ISL_2462270, EPI_ISL_2462272, EPI_ISL_2462280, EPI_ISL_2462281, EPI_ISL_2462285, EPI_ISL_2462294, EPI_ISL_2462297, EPI_ISL_2462302, EPI_ISL_2462304, EPI_ISL_2462305, EPI_ISL_2462308, EPI_ISL_2663619, EPI_ISL_2663623, EPI_ISL_2663642, EPI_ISL_2663643, EPI_ISL_2663648, EPI_ISL_2663651, EPI_ISL_2663652, EPI_ISL_2663669, EPI_ISL_2663671, EPI_ISL_2663672, EPI_ISL_2663679, EPI_ISL_2663687, EPI_ISL_2663703, EPI_ISL_2663706, EPI_ISL_2663707, EPI_ISL_2663716, EPI_ISL_2663722, EPI_ISL_2663723, EPI_ISL_2663736, EPI_ISL_2663738, EPI_ISL_2663740, EPI_ISL_2663741, EPI_ISL_2663752, EPI_ISL_2663755, EPI_ISL_2663764, EPI_ISL_2663765, EPI_ISL_2838825, EPI_ISL_2838826, EPI_ISL_2838827, EPI_ISL_2838828, EPI_ISL_2838829, EPI_ISL_2838830, EPI_ISL_2838831, EPI_ISL_2838832, EPI_ISL_2838833, EPI_ISL_2838834, EPI_ISL_2838835, EPI_ISL_2838836, EPI_ISL_2838837, EPI_ISL_2838838, EPI_ISL_2838839, EPI_ISL_2838840, EPI_ISL_2838841, EPI_ISL_2838842, EPI_ISL_2838843, EPI_ISL_2838844, EPI_ISL_2838845, EPI_ISL_2838846, EPI_ISL_2838847, EPI_ISL_2838848, EPI_ISL_2838849, EPI_ISL_2838850, EPI_ISL_2838851, EPI_ISL_2838852, EPI_ISL_2838853, EPI_ISL_2838854, EPI_ISL_2838855			
see above	OHSU Lab Services Molecular Microbiology Lab	Oregon SARS-CoV-2 Genome Sequencing Center	'[UsersoroakBox SyncPHI_SARS-COV-2_Sample_Sheet_infoGISAID_Submission_Infometa_master_2021.xlsx]release20_final'!D:AC; 26; Alec J. Hirsch; Andrew C. Adey; Benjamin N. Bimber; Brendan L. O'Connell; Brian J. O'Roak; Cierra LaBlanc; Daniel N. Streblow; Donna Hansel; FALSE); Guang Fan; Kayla Carter; Ruth V. Nichols; Sally Grindstaff; Sonia Acharya; VLOOKUP(C378; VLOOKUP(C379; VLOOKUP(C389; VLOOKUP(C390; VLOOKUP(C391; VLOOKUP(C395; VLOOKUP(C396; VLOOKUP(C397; VLOOKUP(C398; VLOOKUP(C410; VLOOKUP(C412; VLOOKUP(C413; VLOOKUP(C416; VLOOKUP(C418; VLOOKUP(C419; VLOOKUP(C421; VLOOKUP(C422; VLOOKUP(C423; VLOOKUP(C424; VLOOKUP(C427; VLOOKUP(C428; VLOOKUP(C429; VLOOKUP(C430; VLOOKUP(C433; VLOOKUP(C434; VLOOKUP(C438; VLOOKUP(C440; VLOOKUP(C441; VLOOKUP(C454; VLOOKUP(C459; VLOOKUP(C463; William B. Messer; Xuan Qin
EPI_ISL_2467884	OK Public Health Laboratory, Oklahoma State DOH	Genomics and Discovery, Respiratory Viruses Branch, Division of Viral Diseases, Centers for Disease Control and Prevention	Adam Retchless; Anna Kelleher; Anna Uehara; Brian Lynch; Clinton R. Paden; Dhwani Batra; Haibin Wang; Han Jia Justin Ng; Jasmine Padilla; Jing Zhang; Justin Lee; Krista Queen; Mark Burroughs; Mili Sheth; Morgan Davis; Peter Cook; Rachel Marine; Sarah Nobles; Suxiang Tong; Tara Coalter; Yan Li; Ying Tao
EPI_ISL_2384211, EPI_ISL_2384217, EPI_ISL_2384220, EPI_ISL_2399429, EPI_ISL_2451980, EPI_ISL_2451989, EPI_ISL_2451994, EPI_ISL_2689978, EPI_ISL_2689988, EPI_ISL_3354886, EPI_ISL_3354890, EPI_ISL_3354911, EPI_ISL_3354936			
see above	OR State PHL-Virology/Immunology Section	Centers for Disease Control and Prevention Division of Viral Diseases, Pathogen Discovery	Alex Burgin; Alison Laufer Halpin; Ben L. Rambo-Martin; Ben Rambo-Martin; Clinton Paden; Clinton R. Paden; Dakota Howard; Darlene Wagner; Dave Wentworth; Dhwani Batra; Jasmine Padilla; Justin Lee; Katie Dillon; Krista Queen; Kristen Knipe; Kristine Lacek; Mark Burroughs; Matthew Schmerer; Meghan Bentz; Mili Sheth; Peter Cook; Sam Shepard; Sarah Nobles; Shoshona Le; Suxiang Tong; Vivien Dugan; Yvette Unoarumhi
EPI_ISL_2753965, EPI_ISL_2755100, EPI_ISL_2768100, EPI_ISL_2858702, EPI_ISL_2858703	Ohio Department of Health Laboratory	Ohio Department of Health Laboratory	Allison Black; Brent Lee; Caitlin McDonnell; Eric Brandt; Erica Leasure; Glen McGillivary; Heather Blankenship; Holmes; Jade Mowery; Jennifer; Kelsey Florek; Keoni Omura; Kirtana Ramadugu; Quanta Brown; Stephanie Mccracken; Tyler Payne; and Tammy Bannerman
EPI_ISL_2566359, EPI_ISL_2566360, EPI_ISL_2566361	Operation Infectious Diseases, Naval Health Research Center	Operation Infectious Diseases, Naval Health Research Center	A.E.; Balansay-Ames; C.A.; Fierro, M.; Iniguez-Stevens, E.; Kriner, P.; M.S.; Moser, K.; Myers; Pan; Phippard; R.S.; R.W.; Underwood
EPI_ISL_2500059, EPI_ISL_2500104	Orange County Public Health Lab	Chan-Zuckerberg Biohub	CZB Cliahub Consortium
EPI_ISL_1897467, EPI_ISL_1967717, EPI_ISL_1967718, EPI_ISL_2023470, EPI_ISL_2023471, EPI_ISL_2023472, EPI_ISL_2023477, EPI_ISL_2081513, EPI_ISL_2081522, EPI_ISL_2086677, EPI_ISL_2086696, EPI_ISL_2138891, EPI_ISL_2138892, EPI_ISL_2139551, EPI_ISL_2139560, EPI_ISL_2139565, EPI_ISL_2203066, EPI_ISL_2203067, EPI_ISL_2203070, EPI_ISL_2203071, EPI_ISL_2203095, EPI_ISL_2250168, EPI_ISL_2339321, EPI_ISL_2339322, EPI_ISL_2339323, EPI_ISL_2339324, EPI_ISL_2339325, EPI_ISL_2339326, EPI_ISL_2339327, EPI_ISL_2339335, EPI_ISL_2346112, EPI_ISL_2346113, EPI_ISL_2346120, EPI_ISL_2348927, EPI_ISL_2348931, EPI_ISL_2348933, EPI_ISL_2348949, EPI_ISL_2348953, EPI_ISL_2404739, EPI_ISL_2404748, EPI_ISL_2424247, EPI_ISL_2424248, EPI_ISL_2424255, EPI_ISL_2473661, EPI_ISL_2473662, EPI_ISL_2473663, EPI_ISL_2473671, EPI_ISL_2473672, EPI_ISL_2557921, EPI_ISL_2727477			
see above	Oregon State Public Health Laboratory	Oregon State Public Health Laboratory	Eugene Yeboah; John Fontana and Shane Sevey; Laura Tsaknaridis; Rafia Razzaque; Vanda Makris
EPI_ISL_2295791	Oregon State University TRACE Project	OSU Center for Genome Research and Biocomputing	Oregon State University TRACE Project
EPI_ISL_2293349, EPI_ISL_2293357, EPI_ISL_2293358, EPI_ISL_2293359, EPI_ISL_2293360, EPI_ISL_2293361, EPI_ISL_2293362, EPI_ISL_2293363, EPI_ISL_2293364, EPI_ISL_2293365, EPI_ISL_2293367, EPI_ISL_2293368, EPI_ISL_2293369, EPI_ISL_2293370, EPI_ISL_2293371, EPI_ISL_2293373, EPI_ISL_2293374, EPI_ISL_2293375, EPI_ISL_2293376, EPI_ISL_2293377, EPI_ISL_2293378, EPI_ISL_2293379, EPI_ISL_2293380, EPI_ISL_2293381, EPI_ISL_2293382			
see above	Outre mer	National Reference Center for Viruses of Respiratory Infections, Institut Pasteur, Paris	Angela Brisebarre; Camille Capel; Christophe Malabat; Corinne Maufrais; Dominique Rousset; Etienne Simon-Lorière; Frédéric Lemoine; Louise Lefrançois; Marion Barbet; Maud Vanpeene; Méline Bizard; Pierre Lechat; Sylvie Behillil; Sylvie Van der Werf; Vincent Enouf
EPI_ISL_2445407, EPI_ISL_2445439	PA CAPELA	Instituto Butantan	Antonio Jorge Martins; Claudia Renata dos Santos Barros; David Schlesinger; Debora Botequio Moretti; Dimas Tadeu Covas; Elaine Cristina Marqueze; Elaine Vieira Santos; Evandra Strazza Rodrigues; Heidge Fukumasu; Jayme Augusto de Souza-Neto; José Salvatore Leister Patané; Luiz Alcantara; Luiz Lehmann Coutinho; Maria Carolina Elias; Maurício Lacerda Nogueira; Rafael dos Santos Bezerra; Raul Machado Neto; Rejane Maria Tommasini Grotto; Ricardo Haddad; Sandra Coccuzzo Sampaio Vessoni; Simone Kashima; Svetoslav Nanev Slavov; Vincent Louis Viala
EPI_ISL_2493489, EPI_ISL_2493490, EPI_ISL_2493501, EPI_ISL_2493504	PA MATAO	Instituto Butantan	Antonio Jorge Martins; Claudia Renata dos Santos Barros; David Schlesinger; Debora Botequio Moretti; Dimas Tadeu Covas; Elaine Cristina Marqueze; Elaine Vieira Santos; Evandra Strazza Rodrigues; Heidge Fukumasu; Jayme Augusto de Souza-Neto; José Salvatore Leister Patané; Luiz Alcantara; Luiz Lehmann Coutinho; Maria Carolina Elias; Maurício Lacerda Nogueira; Rafael dos Santos Bezerra; Raul Machado Neto; Rejane Maria Tommasini Grotto; Ricardo Haddad; Sandra Coccuzzo Sampaio Vessoni; Simone Kashima; Svetoslav Nanev Slavov; Vincent Louis Viala
EPI_ISL_2493356, EPI_ISL_2493357, EPI_ISL_2493358, EPI_ISL_2493359	PAS JOAO ANTONIO DO NASCIMENTO	Instituto Butantan	Antonio Jorge Martins; Claudia Renata dos Santos Barros; David Schlesinger; Debora Botequio Moretti; Dimas Tadeu Covas; Elaine Cristina Marqueze; Elaine Vieira Santos; Evandra Strazza Rodrigues; Heidge Fukumasu; Jayme Augusto de Souza-Neto; José Salvatore Leister Patané; Luiz Alcantara; Luiz Lehmann Coutinho; Maria Carolina Elias; Maurício Lacerda Nogueira; Rafael dos Santos Bezerra; Raul Machado Neto; Rejane Maria Tommasini Grotto; Ricardo Haddad; Sandra Coccuzzo Sampaio Vessoni; Simone Kashima; Svetoslav Nanev Slavov; Vincent Louis Viala
EPI_ISL_2345675	PAS JOAO ANTONIO DO NASCIMENTO	Instituto Butantan / Mendelics	Antonio Jorge Martins; Claudia Renata dos Santos Barros; David Schlesinger; Debora Botequio Moretti; Dimas Tadeu Covas; Elaine Cristina Marqueze; Elaine Vieira Santos; Evandra Strazza Rodrigues; Heidge Fukumasu; Jayme Augusto de Souza-Neto; José Salvatore Leister Patané; Luiz Alcantara; Luiz Lehmann Coutinho; Maria Carolina Elias; Maurício Lacerda Nogueira; Rafael dos Santos Bezerra; Raul Machado Neto; Rejane Maria Tommasini Grotto; Ricardo Haddad; Sandra Coccuzzo Sampaio Vessoni; Simone Kashima; Svetoslav Nanev Slavov; Vincent Louis Viala
EPI_ISL_2500981	PAS JULIANIA DE HERCULANDIA	Instituto Butantan	Antonio Jorge Martins; Claudia Renata dos Santos Barros; David Schlesinger; Debora Botequio Moretti; Dimas Tadeu Covas; Elaine Cristina Marqueze; Elaine Vieira Santos; Evandra Strazza Rodrigues; Heidge Fukumasu; Jayme Augusto de Souza-Neto; José Salvatore Leister Patané; Luiz Alcantara; Luiz Lehmann

			Coutinho; Maria Carolina Elias; Maurício Lacerda Nogueira; Rafael dos Santos Bezerra; Raul Machado Neto; Rejane Maria Tommasini Grotto; Ricardo Haddad; Sandra Coccuzzo Sampaio Vessoni; Simone Kashima; Svetoslav Nanev Slavov; Vincent Louis Viala
EPI_ISL_2208911	PENITENCIARIA DE PRESIDENTE PRUDENTE	Instituto Butantan / Mendelics	Antonio Jorge Martins; Claudia Renata dos Santos Barros; David Schlesinger; Debora Botequio Moretti; Dimas Tadeu Covas; Elaine Cristina Marqueze; Elaine Vieira Santos; Evandra Strazza Rodrigues; Heidge Fukumasu; Jayme Augusto de Souza-Neto; José Salvatore Leister Patané; Luiz Alcantara; Luiz Lehmann Coutinho; Maria Carolina Elias; Maurício Lacerda Nogueira; Rafael dos Santos Bezerra; Raul Machado Neto; Rejane Maria Tommasini Grotto; Ricardo Haddad; Sandra Coccuzzo Sampaio Vessoni; Simone Kashima; Svetoslav Nanev Slavov; Vincent Louis Viala
EPI_ISL_2445596, EPI_ISL_2493667, EPI_ISL_2493673	POLICLINICA COVID 19 ITAPETININGA	Instituto Butantan	Antonio Jorge Martins; Claudia Renata dos Santos Barros; David Schlesinger; Debora Botequio Moretti; Dimas Tadeu Covas; Elaine Cristina Marqueze; Elaine Vieira Santos; Evandra Strazza Rodrigues; Heidge Fukumasu; Jayme Augusto de Souza-Neto; José Salvatore Leister Patané; Luiz Alcantara; Luiz Lehmann Coutinho; Maria Carolina Elias; Maurício Lacerda Nogueira; Rafael dos Santos Bezerra; Raul Machado Neto; Rejane Maria Tommasini Grotto; Ricardo Haddad; Sandra Coccuzzo Sampaio Vessoni; Simone Kashima; Svetoslav Nanev Slavov; Vincent Louis Viala
EPI_ISL_2344927, EPI_ISL_2344941	POLICLINICA COVID 19 ITAPETININGA	Instituto Butantan / ESALQ-Piracicaba	Antonio Jorge Martins; Claudia Renata dos Santos Barros; David Schlesinger; Debora Botequio Moretti; Dimas Tadeu Covas; Elaine Cristina Marqueze; Elaine Vieira Santos; Evandra Strazza Rodrigues; Heidge Fukumasu; Jayme Augusto de Souza-Neto; José Salvatore Leister Patané; Luiz Alcantara; Luiz Lehmann Coutinho; Maria Carolina Elias; Maurício Lacerda Nogueira; Rafael dos Santos Bezerra; Raul Machado Neto; Rejane Maria Tommasini Grotto; Ricardo Haddad; Sandra Coccuzzo Sampaio Vessoni; Simone Kashima; Svetoslav Nanev Slavov; Vincent Louis Viala
EPI_ISL_2345809, EPI_ISL_2345814, EPI_ISL_2345859	POLICLINICA COVID 19 ITAPETININGA	Instituto Butantan / Mendelics	Antonio Jorge Martins; Claudia Renata dos Santos Barros; David Schlesinger; Debora Botequio Moretti; Dimas Tadeu Covas; Elaine Cristina Marqueze; Elaine Vieira Santos; Evandra Strazza Rodrigues; Heidge Fukumasu; Jayme Augusto de Souza-Neto; José Salvatore Leister Patané; Luiz Alcantara; Luiz Lehmann Coutinho; Maria Carolina Elias; Maurício Lacerda Nogueira; Rafael dos Santos Bezerra; Raul Machado Neto; Rejane Maria Tommasini Grotto; Ricardo Haddad; Sandra Coccuzzo Sampaio Vessoni; Simone Kashima; Svetoslav Nanev Slavov; Vincent Louis Viala
EPI_ISL_2493198, EPI_ISL_2493246, EPI_ISL_2493468, EPI_ISL_2493471, EPI_ISL_2493478, EPI_ISL_2493481, EPI_ISL_2493493, EPI_ISL_2493499, EPI_ISL_2493502, EPI_ISL_2493503			
see above	POLICLINICA HORTOLANDIA	Instituto Butantan	Antonio Jorge Martins; Claudia Renata dos Santos Barros; David Schlesinger; Debora Botequio Moretti; Dimas Tadeu Covas; Elaine Cristina Marqueze; Elaine Vieira Santos; Evandra Strazza Rodrigues; Heidge Fukumasu; Jayme Augusto de Souza-Neto; José Salvatore Leister Patané; Luiz Alcantara; Luiz Lehmann Coutinho; Maria Carolina Elias; Maurício Lacerda Nogueira; Rafael dos Santos Bezerra; Raul Machado Neto; Rejane Maria Tommasini Grotto; Ricardo Haddad; Sandra Coccuzzo Sampaio Vessoni; Simone Kashima; Svetoslav Nanev Slavov; Vincent Louis Viala
EPI_ISL_2345265	POLICLINICA HORTOLANDIA	Instituto Butantan / ESALQ-Piracicaba	Antonio Jorge Martins; Claudia Renata dos Santos Barros; David Schlesinger; Debora Botequio Moretti; Dimas Tadeu Covas; Elaine Cristina Marqueze; Elaine Vieira Santos; Evandra Strazza Rodrigues; Heidge Fukumasu; Jayme Augusto de Souza-Neto; José Salvatore Leister Patané; Luiz Alcantara; Luiz Lehmann Coutinho; Maria Carolina Elias; Maurício Lacerda Nogueira; Rafael dos Santos Bezerra; Raul Machado Neto; Rejane Maria Tommasini Grotto; Ricardo Haddad; Sandra Coccuzzo Sampaio Vessoni; Simone Kashima; Svetoslav Nanev Slavov; Vincent Louis Viala
EPI_ISL_2445574, EPI_ISL_2445576	POSTO DE SAUDE BENVIRA E S F 4	Instituto Butantan	Antonio Jorge Martins; Claudia Renata dos Santos Barros; David Schlesinger; Debora Botequio Moretti; Dimas Tadeu Covas; Elaine Cristina Marqueze; Elaine Vieira Santos; Evandra Strazza Rodrigues; Heidge Fukumasu; Jayme Augusto de Souza-Neto; José Salvatore Leister Patané; Luiz Alcantara; Luiz Lehmann Coutinho; Maria Carolina Elias; Maurício Lacerda Nogueira; Rafael dos Santos Bezerra; Raul Machado Neto; Rejane Maria Tommasini Grotto; Ricardo Haddad; Sandra Coccuzzo Sampaio Vessoni; Simone Kashima; Svetoslav Nanev Slavov; Vincent Louis Viala
EPI_ISL_3102280	POSTO DE SAUDE MARIA DE LOURDES MAGALHAES MAIA	Analytical Competence Molecular Epidemiology Lab/ACME, Oswaldo Cruz Foundation, Ceara (FIOCRUZ CE)	Cleber Furtado Aksenen; Fabio Miyajima; Fernando Braga Stehling; Francisco Eder de Moura Lopes; Jamille Maria Mendes Bezerra; Joaquim César do Nascimento Sousa Junior; Pedro Miguel Carneiro Jeronimo; Suzana Porto Almeida e Lucas Delerino; Thais Ferreira de Oliveira; Thais de Oliveira Costa; Ticiane Cavalcante de Souza; Veridiana Pessoa Miyajima
EPI_ISL_2493393	POSTO MEDICO CIDADE NOVA	Instituto Butantan	Antonio Jorge Martins; Claudia Renata dos Santos Barros; David Schlesinger; Debora Botequio Moretti; Dimas Tadeu Covas; Elaine Cristina Marqueze; Elaine Vieira Santos; Evandra Strazza Rodrigues; Heidge Fukumasu; Jayme Augusto de Souza-Neto; José Salvatore Leister Patané; Luiz Alcantara; Luiz Lehmann Coutinho; Maria Carolina Elias; Maurício Lacerda Nogueira; Rafael dos Santos Bezerra; Raul Machado Neto; Rejane Maria Tommasini Grotto; Ricardo Haddad; Sandra Coccuzzo Sampaio Vessoni; Simone Kashima; Svetoslav Nanev Slavov; Vincent Louis Viala
EPI_ISL_2493589, EPI_ISL_2493590, EPI_ISL_2493629	POSTO MEDICO NATAL DIEGUES DE ESTIVA GERBI	Instituto Butantan	Antonio Jorge Martins; Claudia Renata dos Santos Barros; David Schlesinger; Debora Botequio Moretti; Dimas Tadeu Covas; Elaine Cristina Marqueze; Elaine Vieira Santos; Evandra Strazza Rodrigues; Heidge Fukumasu; Jayme Augusto de Souza-Neto; José Salvatore Leister Patané; Luiz Alcantara; Luiz Lehmann Coutinho; Maria Carolina Elias; Maurício Lacerda Nogueira; Rafael dos Santos Bezerra; Raul Machado Neto; Rejane Maria Tommasini Grotto; Ricardo Haddad; Sandra Coccuzzo Sampaio Vessoni; Simone Kashima; Svetoslav Nanev Slavov; Vincent Louis Viala
EPI_ISL_3354889	PR Public Health Lab	Centers for Disease Control and Prevention Division of Viral Diseases, Pathogen Discovery	Alex Burgin; Ben Rambo-Martin; Clinton Paden; Dakota Howard; Dave Wentworth; Dhwani Batra; Jasmine Padilla; Justin Lee; Krista Queen; Kristen Knipe; Kristine Lacek; Mark Burroughs; Matthew Schmerer; Meghan Bentz; Mili Sheth; Peter Cook; Sam Shepard; Sarah Nobles; Suxiang Tong; Vivien Dugan; Yvette Unoarumhi
EPI_ISL_2493277, EPI_ISL_2493355, EPI_ISL_2493694, EPI_ISL_2493695	PROGRAMA SAUDE DA FAMILIA I IEPE	Instituto Butantan	Antonio Jorge Martins; Claudia Renata dos Santos Barros; David Schlesinger; Debora Botequio Moretti; Dimas Tadeu Covas; Elaine Cristina Marqueze; Elaine Vieira Santos; Evandra Strazza Rodrigues; Heidge Fukumasu; Jayme Augusto de Souza-Neto; José Salvatore Leister Patané; Luiz Alcantara; Luiz Lehmann Coutinho; Maria Carolina Elias; Maurício Lacerda Nogueira; Rafael dos Santos Bezerra; Raul Machado Neto; Rejane Maria Tommasini Grotto; Ricardo Haddad; Sandra Coccuzzo Sampaio Vessoni; Simone Kashima; Svetoslav Nanev Slavov; Vincent Louis Viala
EPI_ISL_2445241, EPI_ISL_2445244, EPI_ISL_2445252, EPI_ISL_2445369	PRONTO ATENDIMENTO DE VARGEM GRANDE PAULISTA	Instituto Butantan	Antonio Jorge Martins; Claudia Renata dos Santos Barros; David Schlesinger; Debora Botequio Moretti; Dimas Tadeu Covas; Elaine Cristina Marqueze; Elaine Vieira Santos; Evandra Strazza Rodrigues; Heidge Fukumasu; Jayme Augusto de Souza-Neto; José Salvatore Leister Patané; Luiz Alcantara; Luiz Lehmann Coutinho; Maria Carolina Elias; Maurício Lacerda Nogueira; Rafael dos Santos Bezerra; Raul Machado Neto; Rejane Maria Tommasini Grotto; Ricardo Haddad; Sandra Coccuzzo Sampaio Vessoni; Simone Kashima; Svetoslav Nanev Slavov; Vincent Louis Viala
EPI_ISL_2345714	PRONTO ATENDIMENTO MUNICIPAL	Instituto Butantan / Mendelics	Antonio Jorge Martins; Claudia Renata dos Santos Barros; David Schlesinger; Debora Botequio Moretti; Dimas Tadeu Covas; Elaine Cristina Marqueze; Elaine Vieira Santos; Evandra Strazza Rodrigues; Heidge Fukumasu; Jayme Augusto de Souza-Neto; José Salvatore Leister Patané; Luiz Alcantara; Luiz Lehmann Coutinho; Maria Carolina Elias; Maurício Lacerda Nogueira; Rafael dos Santos Bezerra; Raul Machado Neto; Rejane Maria Tommasini Grotto; Ricardo Haddad; Sandra Coccuzzo Sampaio Vessoni; Simone Kashima; Svetoslav Nanev Slavov; Vincent Louis Viala
EPI_ISL_2493470	PRONTO ATENDIMENTO SAO JOSE	Instituto Butantan	Antonio Jorge Martins; Claudia Renata dos Santos Barros; David Schlesinger; Debora Botequio Moretti; Dimas Tadeu Covas; Elaine Cristina Marqueze; Elaine Vieira Santos; Evandra Strazza Rodrigues; Heidge Fukumasu; Jayme Augusto de Souza-Neto; José Salvatore Leister Patané; Luiz Alcantara; Luiz Lehmann Coutinho; Maria Carolina Elias; Maurício Lacerda Nogueira; Rafael dos Santos Bezerra; Raul Machado Neto; Rejane Maria Tommasini Grotto; Ricardo Haddad; Sandra Coccuzzo Sampaio Vessoni; Simone Kashima; Svetoslav Nanev Slavov; Vincent Louis Viala
EPI_ISL_2493552, EPI_ISL_2493556, EPI_ISL_2493564	PRONTO SOCORRO DE AGENOR DE CAMPOS MONGAGUA	Instituto Butantan	Antonio Jorge Martins; Claudia Renata dos Santos Barros; David Schlesinger; Debora Botequio Moretti; Dimas Tadeu Covas; Elaine Cristina Marqueze; Elaine Vieira Santos; Evandra Strazza Rodrigues; Heidge Fukumasu; Jayme Augusto de Souza-Neto; José Salvatore Leister Patané; Luiz Alcantara; Luiz Lehmann Coutinho; Maria Carolina Elias; Maurício Lacerda Nogueira; Rafael dos Santos Bezerra; Raul Machado Neto; Rejane Maria Tommasini Grotto; Ricardo Haddad; Sandra Coccuzzo Sampaio Vessoni; Simone Kashima; Svetoslav Nanev Slavov; Vincent Louis Viala
EPI_ISL_2493594, EPI_ISL_2493595, EPI_ISL_2493613, EPI_ISL_2493618, EPI_ISL_2493624, EPI_ISL_2493626	PRONTO SOCORRO MUNICIPAL DE CRAVINHOS	Instituto Butantan	Antonio Jorge Martins; Claudia Renata dos Santos Barros; David Schlesinger; Debora Botequio Moretti; Dimas Tadeu Covas; Elaine Cristina Marqueze; Elaine Vieira Santos; Evandra Strazza Rodrigues; Heidge Fukumasu; Jayme Augusto de Souza-Neto; José Salvatore Leister Patané; Luiz Alcantara; Luiz Lehmann Coutinho; Maria Carolina Elias; Maurício Lacerda Nogueira; Rafael dos Santos Bezerra; Raul Machado Neto; Rejane Maria Tommasini Grotto; Ricardo Haddad; Sandra Coccuzzo Sampaio Vessoni; Simone Kashima; Svetoslav Nanev Slavov; Vincent Louis Viala
EPI_ISL_2345199	PRONTO SOCORRO MUNICIPAL DE SEVERINIA	Instituto Butantan / ESALQ-Piracicaba	Antonio Jorge Martins; Claudia Renata dos Santos Barros; David Schlesinger; Debora Botequio Moretti; Dimas Tadeu Covas; Elaine Cristina Marqueze; Elaine Vieira Santos; Evandra Strazza Rodrigues; Heidge Fukumasu; Jayme Augusto de Souza-Neto; José Salvatore Leister Patané; Luiz Alcantara; Luiz Lehmann Coutinho; Maria Carolina Elias; Maurício Lacerda Nogueira; Rafael dos Santos Bezerra; Raul Machado Neto; Rejane Maria Tommasini Grotto; Ricardo Haddad; Sandra Coccuzzo Sampaio Vessoni; Simone Kashima; Svetoslav Nanev Slavov; Vincent Louis Viala
EPI_ISL_2493527	PRONTO SOCORRO MUNICIPAL TAMBAU	Instituto Butantan	Antonio Jorge Martins; Claudia Renata dos Santos Barros; David Schlesinger; Debora Botequio Moretti; Dimas Tadeu Covas; Elaine Cristina Marqueze; Elaine Vieira Santos; Evandra Strazza Rodrigues; Heidge Fukumasu; Jayme Augusto de Souza-Neto; José Salvatore Leister Patané; Luiz Alcantara; Luiz Lehmann Coutinho; Maria Carolina Elias; Maurício Lacerda Nogueira; Rafael dos Santos Bezerra; Raul Machado Neto; Rejane Maria Tommasini Grotto; Ricardo Haddad; Sandra Coccuzzo Sampaio Vessoni; Simone Kashima; Svetoslav Nanev Slavov; Vincent Louis Viala
EPI_ISL_2345729	PRONTO SOCORRO MUNICIPAL TAMBAU	Instituto Butantan / Mendelics	Antonio Jorge Martins; Claudia Renata dos Santos Barros; David Schlesinger; Debora Botequio Moretti; Dimas Tadeu Covas; Elaine Cristina Marqueze; Elaine Vieira Santos; Evandra Strazza Rodrigues; Heidge Fukumasu; Jayme Augusto de Souza-Neto; José Salvatore Leister Patané; Luiz Alcantara; Luiz Lehmann Coutinho; Maria Carolina Elias; Maurício Lacerda Nogueira; Rafael dos Santos Bezerra; Raul Machado Neto; Rejane Maria Tommasini Grotto; Ricardo Haddad; Sandra Coccuzzo Sampaio Vessoni; Simone Kashima; Svetoslav Nanev Slavov; Vincent Louis Viala
EPI_ISL_2209716	PSF DO PARQUE DOS PINHEIROS	Instituto Butantan / Mendelics	Antonio Jorge Martins; Claudia Renata dos Santos Barros; David Schlesinger; Debora Botequio Moretti; Dimas Tadeu Covas; Elaine Cristina Marqueze; Elaine Vieira Santos; Evandra Strazza Rodrigues; Heidge Fukumasu; Jayme Augusto de Souza-Neto; José Salvatore Leister Patané; Luiz Alcantara; Luiz Lehmann Coutinho; Maria Carolina Elias; Maurício Lacerda Nogueira; Rafael dos Santos Bezerra; Raul Machado Neto; Rejane Maria Tommasini Grotto; Ricardo Haddad; Sandra Coccuzzo Sampaio Vessoni; Simone Kashima; Svetoslav Nanev Slavov; Vincent Louis Viala
EPI_ISL_2344910	PSF MARIA JOSE SALTO DE PIRAPORA	Instituto Butantan / ESALQ-Piracicaba	Antonio Jorge Martins; Claudia Renata dos Santos Barros; David Schlesinger; Debora Botequio Moretti; Dimas Tadeu Covas; Elaine Cristina Marqueze; Elaine Vieira Santos; Evandra Strazza Rodrigues; Heidge Fukumasu; Jayme Augusto de Souza-Neto; José Salvatore Leister Patané; Luiz Alcantara; Luiz Lehmann Coutinho; Maria Carolina Elias; Maurício Lacerda Nogueira; Rafael dos Santos Bezerra; Raul Machado Neto; Rejane Maria Tommasini Grotto; Ricardo Haddad; Sandra Coccuzzo Sampaio Vessoni; Simone Kashima; Svetoslav Nanev Slavov; Vincent Louis Viala
EPI_ISL_2345672	PSF MARIA JOSE SALTO DE PIRAPORA	Instituto Butantan / Mendelics	Antonio Jorge Martins; Claudia Renata dos Santos Barros; David Schlesinger; Debora Botequio Moretti; Dimas Tadeu Covas; Elaine Cristina Marqueze; Elaine Vieira Santos; Evandra Strazza Rodrigues; Heidge Fukumasu; Jayme Augusto de Souza-Neto; José Salvatore Leister Patané; Luiz Alcantara; Luiz Lehmann Coutinho; Maria Carolina Elias; Maurício Lacerda Nogueira; Rafael dos Santos Bezerra; Raul Machado Neto; Rejane Maria Tommasini Grotto; Ricardo Haddad; Sandra Coccuzzo Sampaio Vessoni; Simone Kashima; Svetoslav Nanev Slavov; Vincent Louis Viala
EPI_ISL_2344918	PSF PALMITALZINHO APIAI	Instituto Butantan / ESALQ-Piracicaba	Antonio Jorge Martins; Claudia Renata dos Santos Barros; David Schlesinger; Debora Botequio Moretti; Dimas Tadeu Covas; Elaine Cristina Marqueze; Elaine Vieira Santos; Evandra Strazza Rodrigues; Heidge Fukumasu; Jayme Augusto de Souza-Neto; José Salvatore Leister Patané; Luiz Alcantara; Luiz Lehmann Coutinho; Maria Carolina Elias; Maurício Lacerda Nogueira; Rafael dos Santos Bezerra; Raul Machado Neto; Rejane Maria Tommasini Grotto; Ricardo Haddad; Sandra Coccuzzo Sampaio Vessoni; Simone Kashima; Svetoslav Nanev Slavov; Vincent Louis Viala
EPI_ISL_2493640, EPI_ISL_2493644, EPI_ISL_2493645	PSF VIRGINIO BASSO	Instituto Butantan	Antonio Jorge Martins; Claudia Renata dos Santos Barros; David Schlesinger; Debora Botequio Moretti; Dimas Tadeu Covas; Elaine Cristina Marqueze; Elaine Vieira Santos; Evandra Strazza Rodrigues; Heidge Fukumasu; Jayme Augusto de Souza-Neto; José Salvatore Leister Patané; Luiz Alcantara; Luiz Lehmann Coutinho; Maria Carolina Elias; Maurício Lacerda Nogueira; Rafael dos Santos Bezerra; Raul Machado Neto; Rejane Maria Tommasini Grotto; Ricardo Haddad; Sandra Coccuzzo Sampaio Vessoni; Simone Kashima; Svetoslav Nanev Slavov; Vincent Louis Viala
EPI_ISL_1717818, EPI_ISL_1717859, EPI_ISL_1717950, EPI_ISL_1828239, EPI_ISL_1828491, EPI_ISL_1828520, EPI_ISL_1828569, EPI_ISL_1828614, EPI_ISL_1828615, EPI_ISL_1828683, EPI_ISL_2036997, EPI_ISL_2037071, EPI_ISL_2037072, EPI_ISL_2037091, EPI_ISL_2037109, EPI_ISL_2037153, EPI_ISL_2037165, EPI_ISL_2037181, EPI_ISL_2037216, EPI_ISL_2037300, EPI_ISL_2037338, EPI_ISL_2037375, EPI_ISL_2106316, EPI_ISL_2106360, EPI_ISL_2106365, EPI_ISL_2106383, EPI_ISL_2106488, EPI_ISL_2106568, EPI_ISL_2235006, EPI_ISL_2235042, EPI_ISL_2235090, EPI_ISL_2235158, EPI_ISL_2235168, EPI_ISL_2235191, EPI_ISL_2235194, EPI_ISL_2235205, EPI_ISL_2235215			
see above	Pandemic Response Lab - NYC	Pandemic Response Lab, R&D	Cybill del Castillo; Dylan Law; Haiping Hao; Henry Lee; Jon Laurent; Katharine Nelson; Melissa Hopkins; Michael Hammerling; Pradeep Bugga; Shinyoung Clair Kang; Sol Rey; William Ward
EPI_ISL_3194067	Pathogen Genomics Center, National Institute of Infectious Diseases	Pathogen Genomics Center, National Institute of Infectious Diseases	Kentaro Itokawa; Makoto Kuroda; Masanori Hashino; Rina Tanaka; Tsuyoshi Sekizuka
EPI_ISL_2993757	Pathology, UAB	Pathology, UAB	E.J.; Hendrickson; Hernandez, J.; Leal; Lefkowitz; Moates, D.; R.C.; S.M.
EPI_ISL_2451606	Philadelphia Department of Public Health	Centers for Disease Control and Prevention Division of Viral Diseases, Pathogen Discovery	Alison Laufer Halpin; Ben L. Rambo-Martin; Clinton R. Paden; Dakota Howard; Darlene Wagner; Dave Wentworth; Dhwani Batra; Jasmine Padilla; Justin Lee; Katie Dillon; Krista Queen; Kristen Knipe; Kristine Lacek; Mark Burroughs; Matthew Schmerer; Mili Sheth; Peter Cook; Sam Shepard; Sarah Nobles; Shoshona Le; Suxiang Tong; Vivien Dugan; Yvette Unoarumhi
EPI_ISL_2663311	Plataforma de Vigilancia Molecular (PVM) - FIOCRUZ/BA	Plataforma de Vigilancia Molecular (PVM) - FIOCRUZ/BA	Bruno Bezerril Andrade; Camila I. de Oliveira on behalf of the Fiocruz COVID-19 Genomic Surveillance Network.; Clarissa Araújo Gurgel; Leonardo Paiva Farias; Marina Cucco; Ricardo Khouri; Tiago Graf
EPI_ISL_2131313, EPI_ISL_2272771, EPI_ISL_2362593	Platform BIS UZA/UAntwerpen	Labo Klinische Biologie, UZA	Basil Britto Xavier; Christine Lammens; Herman Goossens; Jasmine Coppens; Marie Le Mercier; Veerle Matheeussen
EPI_ISL_2391780, EPI_ISL_2405204	Pomona Valley Hospital Medical Center	Los Angeles County Public Health Laboratories	P. Hemarajata et al.
EPI_ISL_2759149	Population Medicine and Diagnostic Sciences, Cornell University	Population Medicine and Diagnostic Sciences, Cornell University	Anderson; B.D.; Caserta; Cronk; D.G.; Diel; L.C.; Laverack, M.; Mitchell; P.K.; Plocharczyk, E.; R.R.; Venugopalan, R.
EPI_ISL_4052654	Precision Genomics	MUSC Molecular Pathology Laboratory	Ana Olivo; Ana Vallari; Barbara Harris; Dariusz Pytel; Frederick S. Nolte; Gavin Cloherty; Jaclyn Dunne; Jeremy Stuart; Jim Madory; Julie W. Hirschhorn; Kristen Maurer; Mary Rodgers; Scott Curry; Todd Meyer; W. Bailey Glen Jr
EPI_ISL_2345907, EPI_ISL_2345911, EPI_ISL_2345915, EPI_ISL_2345916, EPI_ISL_2345917, EPI_ISL_2345920, EPI_ISL_2345923, EPI_ISL_2345926, EPI_ISL_2345927, EPI_ISL_2345928, EPI_ISL_2345932, EPI_ISL_2345933, EPI_ISL_2345934, EPI_ISL_2345935, EPI_ISL_2345937, EPI_ISL_2345938, EPI_ISL_2346094, EPI_ISL_2346095, EPI_ISL_2346096, EPI_ISL_2346097			
see above	Prefeirtura de SP	Instituto Butantan	Antonio Jorge Martins; Claudia Renata dos Santos Barros; David Schlesinger; Debora Botequio Moretti; Dimas Tadeu Covas; Elaine Cristina Marqueze; Elaine Vieira Santos; Evandra Strazza Rodrigues; Heidge Fukumasu; Jayme Augusto de Souza-Neto; José Salvatore Leister Patané; Luiz Alcantara; Luiz Lehmann Coutinho; Maria Carolina Elias; Maurício Lacerda Nogueira; Rafael dos Santos Bezerra; Raul Machado Neto; Rejane Maria Tommasini Grotto; Ricardo Haddad; Sandra Coccuzzo Sampaio Vessoni; Simone Kashima; Svetoslav Nanev Slavov; Vincent Louis Viala

EPI_ISL_2232606	Premier Medical Lab	Minnesota Department of Health, Public Health Laboratory	Alexandra Lorentz; Jacob Garfin; Matt Plumb; and Xiong Wang
EPI_ISL_2551453	Providence Regional Medical Center Everett	Providence St. Joseph Health Molecular Genomics Laboratory	Alexa K Dowdell; Brian D Piening; Carlo B Bifulco; Fred L Robinson; Mary Campbell; Rogan Rattray
EPI_ISL_2294568, EPI_ISL_2697716, EPI_ISL_2697779, EPI_ISL_2697800, EPI_ISL_2697856, EPI_ISL_2697864, EPI_ISL_2698216, EPI_ISL_2698232, EPI_ISL_2698234, EPI_ISL_2698247, EPI_ISL_2698317, EPI_ISL_2698363, EPI_ISL_2698387, EPI_ISL_2698423, EPI_ISL_2698429, EPI_ISL_2698546, EPI_ISL_2698728, EPI_ISL_2699183, EPI_ISL_2699253, EPI_ISL_2699399, EPI_ISL_2699437, EPI_ISL_2709021, EPI_ISL_2709031, EPI_ISL_2709048			
see above	Public Health Laboratory, Minnesota Department of Health	University of Minnesota Genomics Center	Corbin Dirkx; Daryl M. Gohl; Jaquelyn Kuriger-Laber; John Garbe; and Sean Wang
EPI_ISL_2196082, EPI_ISL_2196150	QLabs	WVU and Marshall University Combined Genomics Core Facilities	James Denvir; Peter Perrotta; Peter Stoilov; Ryan Percifield; Wesley Kimble
EPI_ISL_1942187, EPI_ISL_1989981, EPI_ISL_1989982, EPI_ISL_1989986, EPI_ISL_1989988, EPI_ISL_1989990, EPI_ISL_2034720, EPI_ISL_2034724, EPI_ISL_2034758, EPI_ISL_2034799, EPI_ISL_2034815, EPI_ISL_2034829, EPI_ISL_2035303, EPI_ISL_2035307, EPI_ISL_2035309, EPI_ISL_2035311, EPI_ISL_2035312, EPI_ISL_2035315, EPI_ISL_2035316, EPI_ISL_2035317, EPI_ISL_2035318, EPI_ISL_2035320, EPI_ISL_2035321, EPI_ISL_2035322, EPI_ISL_2035323, EPI_ISL_2035329, EPI_ISL_2035330, EPI_ISL_2035331, EPI_ISL_2035335, EPI_ISL_2035336, EPI_ISL_2035337, EPI_ISL_2035338, EPI_ISL_2035340, EPI_ISL_2035343, EPI_ISL_2035346, EPI_ISL_2035348, EPI_ISL_2035349, EPI_ISL_2035350, EPI_ISL_2035351, EPI_ISL_2035352, EPI_ISL_2035356, EPI_ISL_2035357, EPI_ISL_2035360, EPI_ISL_2035363, EPI_ISL_2035364, EPI_ISL_2132576, EPI_ISL_2132581, EPI_ISL_2132583, EPI_ISL_2132586, EPI_ISL_2132619, EPI_ISL_2132620, EPI_ISL_2132621, EPI_ISL_2132623, EPI_ISL_2132624, EPI_ISL_2132625, EPI_ISL_2132626, EPI_ISL_2132627, EPI_ISL_2132631, EPI_ISL_2140733, EPI_ISL_2140734, EPI_ISL_2140735, EPI_ISL_2140737, EPI_ISL_2140738, EPI_ISL_2140739, EPI_ISL_2140742, EPI_ISL_2140743, EPI_ISL_2140744, EPI_ISL_2140745, EPI_ISL_2140746, EPI_ISL_2140747, EPI_ISL_2140749, EPI_ISL_2140750, EPI_ISL_2140751, EPI_ISL_2140752, EPI_ISL_2140753, EPI_ISL_2140757, EPI_ISL_2140760, EPI_ISL_2140766, EPI_ISL_2140767, EPI_ISL_2140769, EPI_ISL_2140774, EPI_ISL_2140775, EPI_ISL_2140777, EPI_ISL_2140778, EPI_ISL_2140779, EPI_ISL_2140780, EPI_ISL_2140782, EPI_ISL_2140785, EPI_ISL_2140786, EPI_ISL_2140787, EPI_ISL_2140788, EPI_ISL_2140789, EPI_ISL_2140790, EPI_ISL_2140791, EPI_ISL_2140792, EPI_ISL_2140797, EPI_ISL_2187231, EPI_ISL_2187233, EPI_ISL_2187235, EPI_ISL_2187236, EPI_ISL_2187237, EPI_ISL_2187238, EPI_ISL_2187240, EPI_ISL_2187243, EPI_ISL_2187244, EPI_ISL_2187245, EPI_ISL_2187246, EPI_ISL_2187247, EPI_ISL_2187249, EPI_ISL_2187250, EPI_ISL_2187251, EPI_ISL_2187252, EPI_ISL_2187253, EPI_ISL_2187254, EPI_ISL_2187255, EPI_ISL_2187257, EPI_ISL_2187258, EPI_ISL_2187259, EPI_ISL_2187260, EPI_ISL_2187261, EPI_ISL_2187262, EPI_ISL_2187263, EPI_ISL_2187265, EPI_ISL_2187267, EPI_ISL_2187268, EPI_ISL_2187269, EPI_ISL_2187270, EPI_ISL_2187273, EPI_ISL_2187275, EPI_ISL_2187278, EPI_ISL_2187279, EPI_ISL_2187280, EPI_ISL_2187281, EPI_ISL_2187282, EPI_ISL_2187284, EPI_ISL_2187285, EPI_ISL_2187288, EPI_ISL_2187289, EPI_ISL_2187290, EPI_ISL_2187291, EPI_ISL_2187293, EPI_ISL_2187296, EPI_ISL_2187299, EPI_ISL_2187300, EPI_ISL_2187301, EPI_ISL_2187303, EPI_ISL_2187305, EPI_ISL_2187306, EPI_ISL_2187309, EPI_ISL_2187312, EPI_ISL_2187313, EPI_ISL_2187314, EPI_ISL_2241627, EPI_ISL_2241629, EPI_ISL_2241630, EPI_ISL_2241633, EPI_ISL_2241635, EPI_ISL_2241636, EPI_ISL_2241637, EPI_ISL_2241638, EPI_ISL_2241640, EPI_ISL_2268144, EPI_ISL_2268146, EPI_ISL_2268148, EPI_ISL_2268152, EPI_ISL_2268159, EPI_ISL_2268161, EPI_ISL_2268164, EPI_ISL_2268166, EPI_ISL_2268169, EPI_ISL_2268171, EPI_ISL_2268174, EPI_ISL_2268178, EPI_ISL_2268181, EPI_ISL_2268184, EPI_ISL_2268186, EPI_ISL_2268189, EPI_ISL_2268271, EPI_ISL_2268275, EPI_ISL_2268278, EPI_ISL_2268282, EPI_ISL_2268287, EPI_ISL_2268297, EPI_ISL_2268299, EPI_ISL_2268302, EPI_ISL_2268304, EPI_ISL_2268307, EPI_ISL_2268311, EPI_ISL_2268314, EPI_ISL_2268321, EPI_ISL_2268323, EPI_ISL_2268325, EPI_ISL_2268328, EPI_ISL_2268331, EPI_ISL_2268335, EPI_ISL_2268366, EPI_ISL_2268367, EPI_ISL_2268368, EPI_ISL_2268369, EPI_ISL_2268370, EPI_ISL_2268374, EPI_ISL_2268375, EPI_ISL_2268379, EPI_ISL_2268380, EPI_ISL_2268381, EPI_ISL_2268382, EPI_ISL_2268383, EPI_ISL_2268384, EPI_ISL_2268387, EPI_ISL_2268388, EPI_ISL_2268390, EPI_ISL_2268391, EPI_ISL_2268392, EPI_ISL_2268393, EPI_ISL_2280082, EPI_ISL_2280084, EPI_ISL_2280085, EPI_ISL_2280086, EPI_ISL_2280088, EPI_ISL_2280090, EPI_ISL_2280091, EPI_ISL_2280092, EPI_ISL_2280093, EPI_ISL_2280094, EPI_ISL_2280095, EPI_ISL_2280096, EPI_ISL_2280097, EPI_ISL_2280099, EPI_ISL_2280100, EPI_ISL_2280101, EPI_ISL_2280102, EPI_ISL_2280103, EPI_ISL_2280104, EPI_ISL_2280106, EPI_ISL_2280108, EPI_ISL_2280110, EPI_ISL_2280111, EPI_ISL_2280112, EPI_ISL_2280113, EPI_ISL_2280117, EPI_ISL_2280118, EPI_ISL_2280120, EPI_ISL_2280121, EPI_ISL_2280123, EPI_ISL_2280124, EPI_ISL_2280125, EPI_ISL_2280127, EPI_ISL_2280128, EPI_ISL_2280129, EPI_ISL_2280130, EPI_ISL_2280131, EPI_ISL_2280132, EPI_ISL_2280133, EPI_ISL_2280134, EPI_ISL_2280135, EPI_ISL_2280136, EPI_ISL_2280137, EPI_ISL_2280139, EPI_ISL_2280143, EPI_ISL_2280144, EPI_ISL_2280145, EPI_ISL_2280146, EPI_ISL_2280152, EPI_ISL_2280153, EPI_ISL_2280155, EPI_ISL_2280157, EPI_ISL_2280158, EPI_ISL_2280159, EPI_ISL_2280160, EPI_ISL_2280161, EPI_ISL_2280163, EPI_ISL_2280167, EPI_ISL_2280168, EPI_ISL_2280169, EPI_ISL_2280171, EPI_ISL_2280172, EPI_ISL_2280173, EPI_ISL_2280174, EPI_ISL_2307143, EPI_ISL_2307146, EPI_ISL_2307149, EPI_ISL_2307150, EPI_ISL_2307152, EPI_ISL_2307153, EPI_ISL_2307154, EPI_ISL_2307155, EPI_ISL_2307156, EPI_ISL_2307157, EPI_ISL_2307158, EPI_ISL_2307160, EPI_ISL_2307161, EPI_ISL_2307162, EPI_ISL_2307163, EPI_ISL_2307164, EPI_ISL_2371691, EPI_ISL_2371692, EPI_ISL_2371693, EPI_ISL_2371694, EPI_ISL_2371697, EPI_ISL_2371698, EPI_ISL_2371700, EPI_ISL_2371702, EPI_ISL_2371703, EPI_ISL_2371706, EPI_ISL_2371707, EPI_ISL_2371708, EPI_ISL_2371709, EPI_ISL_2371710, EPI_ISL_2371711, EPI_ISL_2371714, EPI_ISL_2371715, EPI_ISL_2371717, EPI_ISL_2371719, EPI_ISL_2371720, EPI_ISL_2371722, EPI_ISL_2371723, EPI_ISL_2371724, EPI_ISL_2371725, EPI_ISL_2371726, EPI_ISL_2371727, EPI_ISL_2371729, EPI_ISL_2371730, EPI_ISL_2371732, EPI_ISL_2371734, EPI_ISL_2371735, EPI_ISL_2372410, EPI_ISL_2372411, EPI_ISL_2372412, EPI_ISL_2372413, EPI_ISL_2372414, EPI_ISL_2372415, EPI_ISL_2372416, EPI_ISL_2372417, EPI_ISL_2372418, EPI_ISL_2372419, EPI_ISL_2372420, EPI_ISL_2372421, EPI_ISL_2372422, EPI_ISL_2372423, EPI_ISL_2372900, EPI_ISL_2372902, EPI_ISL_2372903, EPI_ISL_2372904, EPI_ISL_2372905, EPI_ISL_2372906, EPI_ISL_2372908, EPI_ISL_2372909, EPI_ISL_2372911, EPI_ISL_2372912, EPI_ISL_2372914, EPI_ISL_2372915, EPI_ISL_2372916, EPI_ISL_2372919, EPI_ISL_2372920, EPI_ISL_2372921, EPI_ISL_2372922, EPI_ISL_2372923, EPI_ISL_2372924, EPI_ISL_2372927, EPI_ISL_2372928, EPI_ISL_2372929, EPI_ISL_2372930, EPI_ISL_2372932, EPI_ISL_2372933, EPI_ISL_2372934, EPI_ISL_2372936, EPI_ISL_2372937, EPI_ISL_2372938, EPI_ISL_2372939, EPI_ISL_2372940, EPI_ISL_2372942, EPI_ISL_2372943, EPI_ISL_2372945, EPI_ISL_2372946, EPI_ISL_2372948, EPI_ISL_2399463, EPI_ISL_2399464, EPI_ISL_2399467, EPI_ISL_2399468, EPI_ISL_2399469, EPI_ISL_2399471, EPI_ISL_2399472, EPI_ISL_2399473, EPI_ISL_2399474, EPI_ISL_2399475, EPI_ISL_2399476, EPI_ISL_2399477, EPI_ISL_2399480, EPI_ISL_2399481, EPI_ISL_2399482, EPI_ISL_2399483, EPI_ISL_2399485, EPI_ISL_2399487, EPI_ISL_2399488, EPI_ISL_2399490, EPI_ISL_2399491, EPI_ISL_2399493, EPI_ISL_2399495, EPI_ISL_2399496, EPI_ISL_2399499, EPI_ISL_2399500, EPI_ISL_2399503, EPI_ISL_2399504, EPI_ISL_2399505, EPI_ISL_2399507, EPI_ISL_2399509, EPI_ISL_2399510, EPI_ISL_2399511, EPI_ISL_2439774, EPI_ISL_2451404, EPI_ISL_2451405, EPI_ISL_2451408, EPI_ISL_2451410, EPI_ISL_2451411, EPI_ISL_2451413, EPI_ISL_2451415, EPI_ISL_2451417, EPI_ISL_2451418, EPI_ISL_2451419, EPI_ISL_2451420, EPI_ISL_2451422, EPI_ISL_2451424, EPI_ISL_2451425, EPI_ISL_2451427, EPI_ISL_2451429, EPI_ISL_2451430, EPI_ISL_2451431, EPI_ISL_2451432, EPI_ISL_2451434, EPI_ISL_2451435, EPI_ISL_2451436, EPI_ISL_2451437, EPI_ISL_2451438, EPI_ISL_2451439, EPI_ISL_2451440, EPI_ISL_2451443, EPI_ISL_2451445, EPI_ISL_2451446, EPI_ISL_2451447, EPI_ISL_2451448, EPI_ISL_2451449, EPI_ISL_2451451, EPI_ISL_2451452, EPI_ISL_2451453, EPI_ISL_2451454, EPI_ISL_2451455, EPI_ISL_2451456, EPI_ISL_2451457, EPI_ISL_2451458, EPI_ISL_2451459, EPI_ISL_2451460, EPI_ISL_2451461, EPI_ISL_2451462, EPI_ISL_2451468, EPI_ISL_2451469, EPI_ISL_2451471, EPI_ISL_2451472, EPI_ISL_2451474, EPI_ISL_2451475, EPI_ISL_2451477, EPI_ISL_2451479, EPI_ISL_2451480, EPI_ISL_2451481, EPI_ISL_2451484, EPI_ISL_2451485, EPI_ISL_2451486, EPI_ISL_2451488, EPI_ISL_2451489, EPI_ISL_2451507, EPI_ISL_2451508, EPI_ISL_2451510, EPI_ISL_2451512, EPI_ISL_2451513, EPI_ISL_2451514, EPI_ISL_2451515, EPI_ISL_2451516, EPI_ISL_2451519, EPI_ISL_2451521, EPI_ISL_2451522, EPI_ISL_2451523, EPI_ISL_2451526, EPI_ISL_2451528, EPI_ISL_2451531, EPI_ISL_2451532, EPI_ISL_2451539, EPI_ISL_2451545, EPI_ISL_2451547, EPI_ISL_2451548, EPI_ISL_2451553, EPI_ISL_2451554, EPI_ISL_2451556, EPI_ISL_2451560, EPI_ISL_2451561, EPI_ISL_2451562			
see above	Quest Diagnostics Incorporated	Centers for Disease Control and Prevention Division of Viral Diseases, Pathogen Discovery	A. Gerasimova; A. Perez; Adrian Paskey; B. Anderson; Benjamin Rambo-Martin; Christopher Gulvick; Clinton R. Paden; Dakota Howard; Darlene Wagner; Dhwani Batra; Duncan MacCannell; F. Lacbawan; I. A. Shlyakhter; Jason Caravas; K.E. Livingston; Kara Moser; L.E. Bernstein; M. Hua; Matthew Schmerer; P. Tanpaiboon; Peter W. Cook; R. M. Kagan; R. Owen; R. V. Rolando; S. H. Rosenthal; Scott Sammons; Shatavia Morrison; Y. Liu; Yvette Unoarumhi
EPI_ISL_2105113, EPI_ISL_2105129, EPI_ISL_2105133, EPI_ISL_2105144, EPI_ISL_2384137, EPI_ISL_2384147, EPI_ISL_2384148, EPI_ISL_2384149, EPI_ISL_2452233, EPI_ISL_2452241, EPI_ISL_2452242, EPI_ISL_2452256, EPI_ISL_2452268			
see above	RI State Health Laboratories	Centers for Disease Control and Prevention Division of Viral Diseases, Pathogen Discovery	Alison Laufer Halpin; Ben L. Rambo-Martin; Clinton R. Paden; Dakota Howard; Darlene Wagner; Dave Wentworth; Dhwani Batra; Jasmine Padilla; Justin Lee; Katie Dillon; Krista Queen; Kristen Knipe; Kristine Lacek; Mark Burroughs; Matthew Schmerer; Mili Sheth; Peter Cook; Sam Shepard; Sarah Nobles; Shoshona Le; Suxiang Tong; Vivien Dugan; Yvette Unoarumhi
EPI_ISL_2004447	Randox Laboratories	Wellcome Sanger Institute for the COVID-19 Genomics UK (COG-UK) Consortium	Cordelia Langford; David K. Jackson; Dominic Kwiatkowski; Ewan Harrison; Ian Johnston; Jeffrey Barrett; John Sillitoe on behalf of the Wellcome Sanger Institute COVID-19 Surveillance Team; Randox Laboratories and Alex Alderton; Roberto Amato; Sonia Goncalves
EPI_ISL_2319845, EPI_ISL_2319865, EPI_ISL_2319885, EPI_ISL_2319895, EPI_ISL_2319910, EPI_ISL_2319911, EPI_ISL_2319915, EPI_ISL_2319919, EPI_ISL_2319935, EPI_ISL_2319943, EPI_ISL_2319946, EPI_ISL_2319948, EPI_ISL_2319956, EPI_ISL_2319957, EPI_ISL_2319959, EPI_ISL_2319960, EPI_ISL_2319963, EPI_ISL_2319964, EPI_ISL_2319965, EPI_ISL_2319967, EPI_ISL_2319969, EPI_ISL_2319975, EPI_ISL_2319976, EPI_ISL_2319977, EPI_ISL_2474032, EPI_ISL_2474062, EPI_ISL_2474066, EPI_ISL_2474069, EPI_ISL_2474070, EPI_ISL_2474072, EPI_ISL_2474074, EPI_ISL_2474076, EPI_ISL_2474077, EPI_ISL_2474078, EPI_ISL_2474083, EPI_ISL_2474096, EPI_ISL_2474106, EPI_ISL_2474126, EPI_ISL_2474136, EPI_ISL_2474151, EPI_ISL_2474172, EPI_ISL_2474173, EPI_ISL_2474176, EPI_ISL_2474177, EPI_ISL_2662183, EPI_ISL_2662190, EPI_ISL_2662191, EPI_ISL_2662195, EPI_ISL_2662197, EPI_ISL_2662202, EPI_ISL_2662204, EPI_ISL_2662205, EPI_ISL_2662221, EPI_ISL_2662226, EPI_ISL_2662234, EPI_ISL_2662241, EPI_ISL_2662253, EPI_ISL_2662255, EPI_ISL_2662262, EPI_ISL_2662265, EPI_ISL_2662266, EPI_ISL_2662267, EPI_ISL_2662271, EPI_ISL_2662273, EPI_ISL_2662280, EPI_ISL_2662282, EPI_ISL_2662283, EPI_ISL_2662284			
see above	Reditus Laboratories	Reditus Laboratories	Alexa Eichelberger; Cassy Philips; Joshua J. Geltz; M.S.; Ph.D.; Rex Dyer; Robert M. Sgambelluri
EPI_ISL_3776073, EPI_ISL_3777139, EPI_ISL_3777148	Respiratory Virus Unit, Microbiology Services Colindale, Public Health England	COVID-19 Genomics UK (COG-UK) Consortium	PHE Covid Sequencing Team
EPI_ISL_2106026, EPI_ISL_2106030, EPI_ISL_2106049, EPI_ISL_2106100, EPI_ISL_2106103, EPI_ISL_2253127, EPI_ISL_2253128, EPI_ISL_2253129, EPI_ISL_2253130, EPI_ISL_2253131, EPI_ISL_2253132, EPI_ISL_2253133, EPI_ISL_2253135, EPI_ISL_2253136, EPI_ISL_2253139, EPI_ISL_2253141, EPI_ISL_2253142, EPI_ISL_2253144, EPI_ISL_2253145, EPI_ISL_2253147, EPI_ISL_2253151, EPI_ISL_2253154, EPI_ISL_2253156, EPI_ISL_2253158, EPI_ISL_2253159, EPI_ISL_2253161, EPI_ISL_2253162, EPI_ISL_2254274, EPI_ISL_2254320, EPI_ISL_2254331, EPI_ISL_2254351, EPI_ISL_2254358, EPI_ISL_2254370, EPI_ISL_2254375, EPI_ISL_2254377, EPI_ISL_2254457, EPI_ISL_2254461, EPI_ISL_2254467, EPI_ISL_2254484			
see above	Respiratory Viruses Branch, Centers for Disease Control and Prevention	Respiratory Viruses Branch, Centers for Disease Control and Prevention	Anderson, B.; Baker; Bakhash; Batra, D.; Bernstein, L.; Breit, N.; C.R.; Campbell, P.; Caravas, J.; Case, R.; Clark, C.; Cook; Ellis, S.; Gerasimova, A.; Ghorpade, V.; Greninger, A.; Gulvick, C.; Hardison, M.; Houdeshell, H.; Howard, D.; Hua, M.; Huang; I.A.; Jerome; K.R.; Kagan; Kvalvaag, O.; Lacbawan, F.; Lin, M.; Liu, Y.; Livingston; Livingston, K.; M.-L.; M.S.; MacCannell, D.; Mathias, P.; Morrison, S.; Moser, K.; N.R.; Nall, D.; Nguyen; Owen, R.; P.W.; Paden; Paskey, A.; Perez, A.; Perez, R.; R.J.; R.M.; R.V.; Rambo-Martin, B.; Rolando; Rosenthal; Roychoudhury; S.H.; S.M.; Sammons, S.; Sanders, E.; Schmerer, M.; Shlyakhter; Shrestha, L.; T.V.; Tanpaiboon, P.; Unoarumhi, Y.; Vest, A.; Wagner, D.; Westlund, S.; Xie, H.
EPI_ISL_2016490, EPI_ISL_2016491, EPI_ISL_2016504, EPI_ISL_2016515, EPI_ISL_2016516, EPI_ISL_2016523, EPI_ISL_2016554, EPI_ISL_2016556, EPI_ISL_2016581, EPI_ISL_2016597, EPI_ISL_2133808, EPI_ISL_2133814, EPI_ISL_2133879, EPI_ISL_2133886, EPI_ISL_2133888, EPI_ISL_2133897, EPI_ISL_2133911, EPI_ISL_2133926, EPI_ISL_2232415, EPI_ISL_2232434, EPI_ISL_2232435, EPI_ISL_2232439, EPI_ISL_2232443, EPI_ISL_2232444, EPI_ISL_2232445, EPI_ISL_2232451, EPI_ISL_2232530, EPI_ISL_2232532, EPI_ISL_2232537, EPI_ISL_2232538, EPI_ISL_2362382, EPI_ISL_2362390, EPI_ISL_2362397, EPI_ISL_2362403, EPI_ISL_2362406, EPI_ISL_2362416, EPI_ISL_2362417, EPI_ISL_2362440			
see above	Rhode Island Department of Health	Infectious Disease Program, Broad Institute of Harvard and MIT	Adams, G.; Azevedo, K.; B.L.; B.W.; Bauer, M.; Birren; Carter, A.; Chaluvadi, S.; D.J.; DeRuff, K.; Gladden-Young, A.; Huard, R.; J.E.; K.J.; King, E.; Lagerborg, K.; Lemieux; Loreth, C.; Miller, A.; Normandin, E.; P.C.; Park; Pearlman, L.; Reilly, S.; Rudy, M.; Sabeti; Siddle; Tomkins-Tinch, C.; and MacInnis
EPI_ISL_2604798, EPI_ISL_2604813, EPI_ISL_2604861	Rhode Island State Health Laboratories	Rhode Island State Health Laboratories	Ewa King; Kristin Carpenter-Azevedo; Richard C. Huard
EPI_ISL_2425504, EPI_ISL_2425519, EPI_ISL_2425522, EPI_ISL_2426847, EPI_ISL_2426848, EPI_ISL_2426849, EPI_ISL_2725578			
see above	Rhode Island State Health Laboratory	Rhode Island State Health Laboratory	Ewa King; Kristin Carpenter-Azevedo; Richard C. Huard
EPI_ISL_2691916, EPI_ISL_2691926, EPI_ISL_2691936, EPI_ISL_2691937, EPI_ISL_2691939, EPI_ISL_2691940, EPI_ISL_2691944, EPI_ISL_2691948, EPI_ISL_2691952			
see above	Rush University Medical Center	RIPHL at Rush University Medical Center	Diane Springer; Felix Araujo Perez; Joyce Houlihan; Kevin Kunstman; Laura Furtado; Marieta Hyde; Mary Hayden; Max Kolton; Stefan Green
EPI_ISL_2445155, EPI_ISL_2445156, EPI_ISL_2445159, EPI_ISL_2445161, EPI_ISL_2445162, EPI_ISL_2445163, EPI_ISL_2445166, EPI_ISL_2445170, EPI_ISL_2445174, EPI_ISL_2445409, EPI_ISL_2445411, EPI_ISL_2445413, EPI_ISL_2473708, EPI_ISL_2473709			
see above	SAE CTA SERV ATEND ESPEC TESTAGEM E ACONSELHAMENTO	Instituto Butantan	Antonio Jorge Martins; Claudia Renata dos Santos Barros; David Schlesinger; Debora Botequio Moretti; Dimas Tadeu Covas; Elaine Cristina Marqueze; Elaine Vieira Santos; Evandra Strazza Rodrigues; Heidge Fukumasu; Jayme Augusto de Souza-Neto; José Salvatore Leister Patané; Luiz Alcantara; Luiz Lehmann Coutinho; Maria Carolina Elias; Maurício Lacerda Nogueira; Rafael dos Santos Bezerra; Raul Machado Neto; Rejane Maria Tommasini Grotto; Ricardo Haddad; Sandra Coccuzzo Sampaio Vessoni; Simone Kashima; Svetoslav Nanev Slavov; Vincent Louis Viala
EPI_ISL_2344802, EPI_ISL_2344803, EPI_ISL_2344804, EPI_ISL_2344805, EPI_ISL_2344810, EPI_ISL_2344811, EPI_ISL_2344812, EPI_ISL_2344813, EPI_ISL_2344815, EPI_ISL_2344816, EPI_ISL_2344817, EPI_ISL_2344819			
see above	SAE CTA SERV ATEND ESPEC TESTAGEM E ACONSELHAMENTO	Instituto Butantan / ESALQ-Piracicaba	Antonio Jorge Martins; Claudia Renata dos Santos Barros; David Schlesinger; Debora Botequio Moretti; Dimas Tadeu Covas; Elaine Cristina Marqueze; Elaine Vieira Santos; Evandra Strazza Rodrigues; Heidge Fukumasu; Jayme Augusto de Souza-Neto; José Salvatore Leister Patané; Luiz Alcantara; Luiz Lehmann Coutinho; Maria Carolina Elias; Maurício Lacerda Nogueira; Rafael dos Santos Bezerra; Raul Machado Neto; Rejane Maria Tommasini Grotto; Ricardo Haddad; Sandra Coccuzzo Sampaio Vessoni; Simone Kashima; Svetoslav Nanev Slavov; Vincent Louis Viala
EPI_ISL_2445139, EPI_ISL_2445140, EPI_ISL_2445141, EPI_ISL_2473755	SANTA CASA DE DOIS CORREGOS	Instituto Butantan	Antonio Jorge Martins; Claudia Renata dos Santos Barros; David Schlesinger; Debora Botequio Moretti; Dimas Tadeu Covas; Elaine Cristina Marqueze; Elaine Vieira Santos; Evandra Strazza Rodrigues; Heidge Fukumasu; Jayme Augusto de Souza-Neto; José Salvatore Leister Patané; Luiz Alcantara; Luiz Lehmann Coutinho; Maria Carolina Elias; Maurício Lacerda Nogueira; Rafael dos Santos Bezerra; Raul Machado Neto; Rejane Maria Tommasini Grotto; Ricardo Haddad; Sandra Coccuzzo Sampaio Vessoni; Simone Kashima; Svetoslav Nanev Slavov; Vincent Louis Viala
EPI_ISL_2345037	SANTA CASA DE GUARAREMA	Instituto Butantan	Antonio Jorge Martins; Claudia Renata dos Santos Barros; David Schlesinger; Debora Botequio Moretti; Dimas Tadeu Covas; Elaine Cristina Marqueze; Elaine Vieira Santos; Evandra Strazza Rodrigues; Heidge Fukumasu; Jayme Augusto de Souza-Neto; José Salvatore Leister Patané; Luiz Alcantara; Luiz Lehmann Coutinho; Maria Carolina Elias; Maurício Lacerda Nogueira; Rafael dos Santos Bezerra; Raul Machado Neto; Rejane Maria Tommasini Grotto; Ricardo Haddad; Sandra Coccuzzo Sampaio Vessoni; Simone Kashima; Svetoslav Nanev Slavov; Vincent Louis Viala
EPI_ISL_2345115, EPI_ISL_2345121, EPI_ISL_2345124	SANTA CASA DE GUARAREMA	Instituto Butantan / ESALQ-Piracicaba	Antonio Jorge Martins; Claudia Renata dos Santos Barros; David Schlesinger; Debora Botequio Moretti; Dimas Tadeu Covas; Elaine Cristina Marqueze; Elaine Vieira Santos; Evandra Strazza Rodrigues; Heidge Fukumasu; Jayme Augusto de Souza-Neto; José Salvatore Leister Patané; Luiz Alcantara; Luiz Lehmann Coutinho; Maria Carolina Elias; Maurício Lacerda Nogueira; Rafael dos Santos Bezerra; Raul Machado Neto; Rejane Maria Tommasini Grotto; Ricardo Haddad; Sandra Coccuzzo Sampaio Vessoni; Simone Kashima; Svetoslav Nanev Slavov; Vincent Louis Viala
EPI_ISL_2170997	SANTA CASA DE GUARAREMA	Instituto Butantan / Mendelics	Antonio Jorge Martins; Bianca Cechetto Carlos. Mendelics: Bibiana Santos; Claudia Renata dos Santos Barros; Cíntia Bittar; David Schlesinger. Hemocentro Ribeirão Preto: Simone Kashima; Debora Botequio Moretti; Elaine Cristina Marqueze; Elaine Vieira dos Santos; Elisangela Chicaroni Mattos; Erika Freitas; Evandra Strazza Rodrigues; Felipe Allan da Silva da Costa; Flavia Aburjaile; Fábio Sossai Possebon; Guilherme Campos; Guilherme Targino Valente; Heidge Fukumasu. USP-Botucatu: Rejane Maria Tommasini Grotto; Helena Lage Ferreira; Instituto Butantan: Dimas Tadeu Covas; Jardelina de Souza Todao Bernardino; Jayme A. Souza-Neto; Jessika Cristina Chagas Lesbon; Jorge A. Petroli Marchesi; José Salvatore Leister Patané; João Paulo Kitajima; João Pessoa Araújo Jr.; Leila Sabrina Ullmann; Loyze Paola Oliveira de Lima; Luiz Aurelio de Campos Crispin. Centro de Genômica Funcional da ESALQ: Luiz Lehmann Coutinho; Luiz Carlos Junior de Alcantara; Lívia Sacchetto; Maisa C. Pereira Parra; Maria Carolina Elias; Marta Giovanetti; Marília Moraes; Maurício Lacerda Nogueira. Prefeitura de Sao Paulo: Melissa Palmieri.; Patricia Akemi Assato; Paula Rahal; Paulo Inacio da Costa; Rafael dos Santos Bezerra; Raquel de Lello Rocha Campos Cassano. NGS Soluções Genômicas: Pilar Drummond Sampaio Corrêa Mariani. FZEA-USP Pirassununga: Mirele Daiana Poleti; Raul Machado Neto; Ricardo Augusto Brassaloti; Ricardo Haddad; Rodrigo Tocantins Calado. FAMERP-SJRP: Cecília Artico Banho; Sandra Coccuzzo Sampaio; Svetoslav Nanev Slavov; Vagner Fonseca; Vincent Louis Viala
EPI_ISL_2344865, EPI_ISL_2344876, EPI_ISL_2344878, EPI_ISL_2344880,	SANTA CASA DE JOSE BONIFACIO	Instituto Butantan / ESALQ-Piracicaba	Antonio Jorge Martins; Claudia Renata dos Santos Barros; David Schlesinger; Debora Botequio Moretti; Dimas Tadeu Covas; Elaine Cristina Marqueze; Elaine Vieira Santos; Evandra Strazza Rodrigues; Heidge Fukumasu; Jayme Augusto de Souza-Neto; José Salvatore Leister Patané; Luiz Alcantara; Luiz Lehmann Coutinho; Maria Carolina Elias; Maurício Lacerda Nogueira; Rafael dos Santos Bezerra; Raul Machado Neto; Rejane Maria Tommasini Grotto; Ricardo Haddad; Sandra Coccuzzo Sampaio Vessoni; Simone Kashima; Svetoslav Nanev Slavov; Vincent Louis Viala

EPI_ISL_2344883			
EPI_ISL_2493681	SANTA CASA DE MISERICORDIA DE TIETE	Instituto Butantan	Antonio Jorge Martins; Claudia Renata dos Santos Barros; David Schlesinger; Debora Botequio Moretti; Dimas Tadeu Covas; Elaine Cristina Marqueze; Elaine Vieira Santos; Evandra Strazza Rodrigues; Heidge Fukumasu; Jayme Augusto de Souza-Neto; José Salvatore Leister Patané; Luiz Alcantara; Luiz Lehmann Coutinho; Maria Carolina Elias; Maurício Lacerda Nogueira; Rafael dos Santos Bezerra; Raul Machado Neto; Rejane Maria Tommasini Grotto; Ricardo Haddad; Sandra Coccuzzo Sampaio Vessoni; Simone Kashima; Svetoslav Nanev Slavov; Vincent Louis Viala
EPI_ISL_2209872, EPI_ISL_2210133	SANTA CASA DE MISERICORDIA DE UBATUBA	Instituto Butantan / Mendelics	Antonio Jorge Martins; Claudia Renata dos Santos Barros; David Schlesinger; Debora Botequio Moretti; Dimas Tadeu Covas; Elaine Cristina Marqueze; Elaine Vieira Santos; Evandra Strazza Rodrigues; Heidge Fukumasu; Jayme Augusto de Souza-Neto; José Salvatore Leister Patané; Luiz Alcantara; Luiz Lehmann Coutinho; Maria Carolina Elias; Maurício Lacerda Nogueira; Rafael dos Santos Bezerra; Raul Machado Neto; Rejane Maria Tommasini Grotto; Ricardo Haddad; Sandra Coccuzzo Sampaio Vessoni; Simone Kashima; Svetoslav Nanev Slavov; Vincent Louis Viala
EPI_ISL_2209108, EPI_ISL_2345004, EPI_ISL_2345007, EPI_ISL_2345009, EPI_ISL_2345010, EPI_ISL_2345012	SANTA CASA DE MISERICORDIA SAO JOSE	Instituto Butantan / ESALQ-Piracicaba	Antonio Jorge Martins; Claudia Renata dos Santos Barros; David Schlesinger; Debora Botequio Moretti; Dimas Tadeu Covas; Elaine Cristina Marqueze; Elaine Vieira Santos; Evandra Strazza Rodrigues; Heidge Fukumasu; Jayme Augusto de Souza-Neto; José Salvatore Leister Patané; Luiz Alcantara; Luiz Lehmann Coutinho; Maria Carolina Elias; Maurício Lacerda Nogueira; Rafael dos Santos Bezerra; Raul Machado Neto; Rejane Maria Tommasini Grotto; Ricardo Haddad; Sandra Coccuzzo Sampaio Vessoni; Simone Kashima; Svetoslav Nanev Slavov; Vincent Louis Viala
EPI_ISL_2209888, EPI_ISL_2210250, EPI_ISL_2345682, EPI_ISL_2345727, EPI_ISL_2345739, EPI_ISL_2345783, EPI_ISL_2362269			
see above	SANTA CASA DE MISERICORDIA SAO JOSE	Instituto Butantan / Mendelics	Antonio Jorge Martins; Claudia Renata dos Santos Barros; David Schlesinger; Debora Botequio Moretti; Dimas Tadeu Covas; Elaine Cristina Marqueze; Elaine Vieira Santos; Evandra Strazza Rodrigues; Heidge Fukumasu; Jayme Augusto de Souza-Neto; José Salvatore Leister Patané; Luiz Alcantara; Luiz Lehmann Coutinho; Maria Carolina Elias; Maurício Lacerda Nogueira; Rafael dos Santos Bezerra; Raul Machado Neto; Rejane Maria Tommasini Grotto; Ricardo Haddad; Sandra Coccuzzo Sampaio Vessoni; Simone Kashima; Svetoslav Nanev Slavov; Vincent Louis Viala
EPI_ISL_2344891, EPI_ISL_2344892, EPI_ISL_2344894	SANTA CASA DE PORTO FELIZ	Instituto Butantan / ESALQ-Piracicaba	Antonio Jorge Martins; Claudia Renata dos Santos Barros; David Schlesinger; Debora Botequio Moretti; Dimas Tadeu Covas; Elaine Cristina Marqueze; Elaine Vieira Santos; Evandra Strazza Rodrigues; Heidge Fukumasu; Jayme Augusto de Souza-Neto; José Salvatore Leister Patané; Luiz Alcantara; Luiz Lehmann Coutinho; Maria Carolina Elias; Maurício Lacerda Nogueira; Rafael dos Santos Bezerra; Raul Machado Neto; Rejane Maria Tommasini Grotto; Ricardo Haddad; Sandra Coccuzzo Sampaio Vessoni; Simone Kashima; Svetoslav Nanev Slavov; Vincent Louis Viala
EPI_ISL_2444154, EPI_ISL_2444157, EPI_ISL_2444159, EPI_ISL_2444160, EPI_ISL_2444161, EPI_ISL_2444163, EPI_ISL_2444164, EPI_ISL_2444166, EPI_ISL_2444167, EPI_ISL_2444168, EPI_ISL_2444169, EPI_ISL_2444171, EPI_ISL_2444174, EPI_ISL_2444176, EPI_ISL_2444178, EPI_ISL_2444179, EPI_ISL_2444180, EPI_ISL_2444182, EPI_ISL_2444185, EPI_ISL_2444187, EPI_ISL_2444188, EPI_ISL_2444190, EPI_ISL_2444193, EPI_ISL_2444196, EPI_ISL_2444197, EPI_ISL_2444198, EPI_ISL_2444200, EPI_ISL_2444201, EPI_ISL_2444204, EPI_ISL_2444205, EPI_ISL_2444206, EPI_ISL_2444207, EPI_ISL_2444209, EPI_ISL_2444211, EPI_ISL_2444213, EPI_ISL_2444214, EPI_ISL_2444215, EPI_ISL_2444216, EPI_ISL_2444218, EPI_ISL_2444219, EPI_ISL_2444220, EPI_ISL_2444221, EPI_ISL_2444222, EPI_ISL_2455396, EPI_ISL_2455397			
see above	SAO JOSE DO RIO PRETO	Instituto Butantan / FAMERP	Antonio Jorge Martins; Claudia Renata dos Santos Barros; David Schlesinger; Debora Botequio Moretti; Dimas Tadeu Covas; Elaine Cristina Marqueze; Elaine Vieira Santos; Evandra Strazza Rodrigues; Heidge Fukumasu; Jayme Augusto de Souza-Neto; José Salvatore Leister Patané; Luiz Alcantara; Luiz Lehmann Coutinho; Maria Carolina Elias; Maurício Lacerda Nogueira; Rafael dos Santos Bezerra; Raul Machado Neto; Rejane Maria Tommasini Grotto; Ricardo Haddad; Sandra Coccuzzo Sampaio Vessoni; Simone Kashima; Svetoslav Nanev Slavov; Vincent Louis Viala
EPI_ISL_2098626	SARATOGA HOSPITAL LABORATORY	Wadsworth Center, New York State Department of Health	Alexis Russell; Catharine Prussing; Daryl M. Lamson; Erasmus Schneider; Erica Lasek-Nesselquist; John Kelly; Jonathan Plitnick; Kirsten St. George; Matthew Shudt; Melissa A Leisner; Navjot Singh
EPI_ISL_2791991, EPI_ISL_2986088	SARS-CoV-2 Sequencing Castilla y Leon-Spain Consortium	SARS-CoV-2 Sequencing Castilla y Leon-Spain Consortium	Antonio Orduña-Domingo; Carlos Fuster Foz; Carmen Aldea-Mansilla; Carmen Gimeno Crespo; David Abad; Gabriel March Rossello; Gregoria Megías Lobón; Jose María Eiros Bouza; Laura Sánchez de Prada; M. Isabel Fernandez-Natal; Marta Dominguez-Gil; Marta Hernandez; María Antonia García Castro; Mª Fe Brezmes-Valdivieso; Noelia Arenal Andrés; Silvia Rojo; Sonsoles Garcinuño Pérez
EPI_ISL_2131666, EPI_ISL_2336130, EPI_ISL_2336133, EPI_ISL_2337801, EPI_ISL_2447611, EPI_ISL_2447612	SARS-CoV-2 testing team, National Institute of Infectious Diseases	Pathogen Genomics Center, National Institute of Infectious Diseases	Hazuka Y Furihata; Kentaro Itokawa; Makoto Kuroda; Masanori Hashino; Masumichi Saito; Naomi Nojiri; Nozomu Hanaoka; Rina Tanaka; Sana Uchikoba; Tsuguto Fujimoto; Tsuyoshi Sekizuka
EPI_ISL_2018303	SC Dept of Health and Env. Control-Bureau of Laboratories	Centers for Disease Control and Prevention Division of Viral Diseases, Pathogen Discovery	Alison Laufer Halpin; Ben L. Rambo-Martin; Clinton R. Paden; Dakota Howard; Darlene Wagner; Dave Wentworth; Dhwani Batra; Jasmine Padilla; Justin Lee; Katie Dillon; Krista Queen; Kristen Knipe; Kristine Lacek; Mark Burroughs; Matthew Schmerer; Mili Sheth; Peter Cook; Sam Shepard; Sarah Nobles; Shoshona Le; Suxiang Tong; Vivien Dugan; Yvette Unoarumhi
EPI_ISL_3355017	SD Public Health Laboratory	Centers for Disease Control and Prevention Division of Viral Diseases, Pathogen Discovery	Alex Burgin; Ben Rambo-Martin; Clinton Paden; Dakota Howard; Dave Wentworth; Dhwani Batra; Jasmine Padilla; Justin Lee; Krista Queen; Kristen Knipe; Kristine Lacek; Mark Burroughs; Matthew Schmerer; Meghan Bentz; Mili Sheth; Peter Cook; Sam Shepard; Sarah Nobles; Suxiang Tong; Vivien Dugan; Yvette Unoarumhi
EPI_ISL_2345050, EPI_ISL_2345051	SECAO CENTRO DE DIAGNOSTICO SECEDI	Instituto Butantan / ESALQ-Piracicaba	Antonio Jorge Martins; Claudia Renata dos Santos Barros; David Schlesinger; Debora Botequio Moretti; Dimas Tadeu Covas; Elaine Cristina Marqueze; Elaine Vieira Santos; Evandra Strazza Rodrigues; Heidge Fukumasu; Jayme Augusto de Souza-Neto; José Salvatore Leister Patané; Luiz Alcantara; Luiz Lehmann Coutinho; Maria Carolina Elias; Maurício Lacerda Nogueira; Rafael dos Santos Bezerra; Raul Machado Neto; Rejane Maria Tommasini Grotto; Ricardo Haddad; Sandra Coccuzzo Sampaio Vessoni; Simone Kashima; Svetoslav Nanev Slavov; Vincent Louis Viala
EPI_ISL_2345667, EPI_ISL_2345854	SECAO CENTRO DE DIAGNOSTICO SECEDI	Instituto Butantan / Mendelics	Antonio Jorge Martins; Claudia Renata dos Santos Barros; David Schlesinger; Debora Botequio Moretti; Dimas Tadeu Covas; Elaine Cristina Marqueze; Elaine Vieira Santos; Evandra Strazza Rodrigues; Heidge Fukumasu; Jayme Augusto de Souza-Neto; José Salvatore Leister Patané; Luiz Alcantara; Luiz Lehmann Coutinho; Maria Carolina Elias; Maurício Lacerda Nogueira; Rafael dos Santos Bezerra; Raul Machado Neto; Rejane Maria Tommasini Grotto; Ricardo Haddad; Sandra Coccuzzo Sampaio Vessoni; Simone Kashima; Svetoslav Nanev Slavov; Vincent Louis Viala
EPI_ISL_2493621, EPI_ISL_2493627	SECRETARIA DE SAUDE	Instituto Butantan	Antonio Jorge Martins; Claudia Renata dos Santos Barros; David Schlesinger; Debora Botequio Moretti; Dimas Tadeu Covas; Elaine Cristina Marqueze; Elaine Vieira Santos; Evandra Strazza Rodrigues; Heidge Fukumasu; Jayme Augusto de Souza-Neto; José Salvatore Leister Patané; Luiz Alcantara; Luiz Lehmann Coutinho; Maria Carolina Elias; Maurício Lacerda Nogueira; Rafael dos Santos Bezerra; Raul Machado Neto; Rejane Maria Tommasini Grotto; Ricardo Haddad; Sandra Coccuzzo Sampaio Vessoni; Simone Kashima; Svetoslav Nanev Slavov; Vincent Louis Viala
EPI_ISL_2445464, EPI_ISL_2445468, EPI_ISL_2445470, EPI_ISL_2445474, EPI_ISL_2445475, EPI_ISL_2445477	SECRETARIA DE SAUDE DE LOUVEIRA	Instituto Butantan	Antonio Jorge Martins; Claudia Renata dos Santos Barros; David Schlesinger; Debora Botequio Moretti; Dimas Tadeu Covas; Elaine Cristina Marqueze; Elaine Vieira Santos; Evandra Strazza Rodrigues; Heidge Fukumasu; Jayme Augusto de Souza-Neto; José Salvatore Leister Patané; Luiz Alcantara; Luiz Lehmann Coutinho; Maria Carolina Elias; Maurício Lacerda Nogueira; Rafael dos Santos Bezerra; Raul Machado Neto; Rejane Maria Tommasini Grotto; Ricardo Haddad; Sandra Coccuzzo Sampaio Vessoni; Simone Kashima; Svetoslav Nanev Slavov; Vincent Louis Viala
EPI_ISL_2494148	SECRETARIA DE SAUDE DE RAFARD	Instituto Butantan	Antonio Jorge Martins; Claudia Renata dos Santos Barros; David Schlesinger; Debora Botequio Moretti; Dimas Tadeu Covas; Elaine Cristina Marqueze; Elaine Vieira Santos; Evandra Strazza Rodrigues; Heidge Fukumasu; Jayme Augusto de Souza-Neto; José Salvatore Leister Patané; Luiz Alcantara; Luiz Lehmann Coutinho; Maria Carolina Elias; Maurício Lacerda Nogueira; Rafael dos Santos Bezerra; Raul Machado Neto; Rejane Maria Tommasini Grotto; Ricardo Haddad; Sandra Coccuzzo Sampaio Vessoni; Simone Kashima; Svetoslav Nanev Slavov; Vincent Louis Viala
EPI_ISL_2493389, EPI_ISL_2493390, EPI_ISL_2493447, EPI_ISL_2494156	SECRETARIA DE SAUDE DE SANTA GERTRUDES	Instituto Butantan	Antonio Jorge Martins; Claudia Renata dos Santos Barros; David Schlesinger; Debora Botequio Moretti; Dimas Tadeu Covas; Elaine Cristina Marqueze; Elaine Vieira Santos; Evandra Strazza Rodrigues; Heidge Fukumasu; Jayme Augusto de Souza-Neto; José Salvatore Leister Patané; Luiz Alcantara; Luiz Lehmann Coutinho; Maria Carolina Elias; Maurício Lacerda Nogueira; Rafael dos Santos Bezerra; Raul Machado Neto; Rejane Maria Tommasini Grotto; Ricardo Haddad; Sandra Coccuzzo Sampaio Vessoni; Simone Kashima; Svetoslav Nanev Slavov; Vincent Louis Viala
EPI_ISL_2494155	SECRETARIA DE SAUDE DE SAO PEDRO	Instituto Butantan	Antonio Jorge Martins; Claudia Renata dos Santos Barros; David Schlesinger; Debora Botequio Moretti; Dimas Tadeu Covas; Elaine Cristina Marqueze; Elaine Vieira Santos; Evandra Strazza Rodrigues; Heidge Fukumasu; Jayme Augusto de Souza-Neto; José Salvatore Leister Patané; Luiz Alcantara; Luiz Lehmann Coutinho; Maria Carolina Elias; Maurício Lacerda Nogueira; Rafael dos Santos Bezerra; Raul Machado Neto; Rejane Maria Tommasini Grotto; Ricardo Haddad; Sandra Coccuzzo Sampaio Vessoni; Simone Kashima; Svetoslav Nanev Slavov; Vincent Louis Viala
EPI_ISL_2209329, EPI_ISL_2209516, EPI_ISL_2209905, EPI_ISL_2210305, EPI_ISL_2345703, EPI_ISL_2345802	SECRETARIA DE SAUDE PUBLICA DE PRAIA GRANDE	Instituto Butantan / Mendelics	Antonio Jorge Martins; Claudia Renata dos Santos Barros; David Schlesinger; Debora Botequio Moretti; Dimas Tadeu Covas; Elaine Cristina Marqueze; Elaine Vieira Santos; Evandra Strazza Rodrigues; Heidge Fukumasu; Jayme Augusto de Souza-Neto; José Salvatore Leister Patané; Luiz Alcantara; Luiz Lehmann Coutinho; Maria Carolina Elias; Maurício Lacerda Nogueira; Rafael dos Santos Bezerra; Raul Machado Neto; Rejane Maria Tommasini Grotto; Ricardo Haddad; Sandra Coccuzzo Sampaio Vessoni; Simone Kashima; Svetoslav Nanev Slavov; Vincent Louis Viala
EPI_ISL_2493598, EPI_ISL_2493601, EPI_ISL_2493603, EPI_ISL_2493612	SECRETARIA MUNICIPAL DA SAUDE DE GUARIBA	Instituto Butantan	Antonio Jorge Martins; Claudia Renata dos Santos Barros; David Schlesinger; Debora Botequio Moretti; Dimas Tadeu Covas; Elaine Cristina Marqueze; Elaine Vieira Santos; Evandra Strazza Rodrigues; Heidge Fukumasu; Jayme Augusto de Souza-Neto; José Salvatore Leister Patané; Luiz Alcantara; Luiz Lehmann Coutinho; Maria Carolina Elias; Maurício Lacerda Nogueira; Rafael dos Santos Bezerra; Raul Machado Neto; Rejane Maria Tommasini Grotto; Ricardo Haddad; Sandra Coccuzzo Sampaio Vessoni; Simone Kashima; Svetoslav Nanev Slavov; Vincent Louis Viala
EPI_ISL_2344896	SECRETARIA MUNICIPAL DA SAUDE DE ITAPEVA SP	Instituto Butantan / ESALQ-Piracicaba	Antonio Jorge Martins; Claudia Renata dos Santos Barros; David Schlesinger; Debora Botequio Moretti; Dimas Tadeu Covas; Elaine Cristina Marqueze; Elaine Vieira Santos; Evandra Strazza Rodrigues; Heidge Fukumasu; Jayme Augusto de Souza-Neto; José Salvatore Leister Patané; Luiz Alcantara; Luiz Lehmann Coutinho; Maria Carolina Elias; Maurício Lacerda Nogueira; Rafael dos Santos Bezerra; Raul Machado Neto; Rejane Maria Tommasini Grotto; Ricardo Haddad; Sandra Coccuzzo Sampaio Vessoni; Simone Kashima; Svetoslav Nanev Slavov; Vincent Louis Viala
EPI_ISL_2445599	SECRETARIA MUNICIPAL DA SAUDE DE ITARARE	Instituto Butantan	Antonio Jorge Martins; Claudia Renata dos Santos Barros; David Schlesinger; Debora Botequio Moretti; Dimas Tadeu Covas; Elaine Cristina Marqueze; Elaine Vieira Santos; Evandra Strazza Rodrigues; Heidge Fukumasu; Jayme Augusto de Souza-Neto; José Salvatore Leister Patané; Luiz Alcantara; Luiz Lehmann Coutinho; Maria Carolina Elias; Maurício Lacerda Nogueira; Rafael dos Santos Bezerra; Raul Machado Neto; Rejane Maria Tommasini Grotto; Ricardo Haddad; Sandra Coccuzzo Sampaio Vessoni; Simone Kashima; Svetoslav Nanev Slavov; Vincent Louis Viala
EPI_ISL_2344903, EPI_ISL_2344912	SECRETARIA MUNICIPAL DA SAUDE DE ITARARE	Instituto Butantan / ESALQ-Piracicaba	Antonio Jorge Martins; Claudia Renata dos Santos Barros; David Schlesinger; Debora Botequio Moretti; Dimas Tadeu Covas; Elaine Cristina Marqueze; Elaine Vieira Santos; Evandra Strazza Rodrigues; Heidge Fukumasu; Jayme Augusto de Souza-Neto; José Salvatore Leister Patané; Luiz Alcantara; Luiz Lehmann Coutinho; Maria Carolina Elias; Maurício Lacerda Nogueira; Rafael dos Santos Bezerra; Raul Machado Neto; Rejane Maria Tommasini Grotto; Ricardo Haddad; Sandra Coccuzzo Sampaio Vessoni; Simone Kashima; Svetoslav Nanev Slavov; Vincent Louis Viala
EPI_ISL_2345855	SECRETARIA MUNICIPAL DA SAUDE DE ITARARE	Instituto Butantan / Mendelics	Antonio Jorge Martins; Claudia Renata dos Santos Barros; David Schlesinger; Debora Botequio Moretti; Dimas Tadeu Covas; Elaine Cristina Marqueze; Elaine Vieira Santos; Evandra Strazza Rodrigues; Heidge Fukumasu; Jayme Augusto de Souza-Neto; José Salvatore Leister Patané; Luiz Alcantara; Luiz Lehmann Coutinho; Maria Carolina Elias; Maurício Lacerda Nogueira; Rafael dos Santos Bezerra; Raul Machado Neto; Rejane Maria Tommasini Grotto; Ricardo Haddad; Sandra Coccuzzo Sampaio Vessoni; Simone Kashima; Svetoslav Nanev Slavov; Vincent Louis Viala
EPI_ISL_2345875	SECRETARIA MUNICIPAL DA SAUDE DE JOANOPOLIS	Instituto Butantan / Mendelics	Antonio Jorge Martins; Claudia Renata dos Santos Barros; David Schlesinger; Debora Botequio Moretti; Dimas Tadeu Covas; Elaine Cristina Marqueze; Elaine Vieira Santos; Evandra Strazza Rodrigues; Heidge Fukumasu; Jayme Augusto de Souza-Neto; José Salvatore Leister Patané; Luiz Alcantara; Luiz Lehmann Coutinho; Maria Carolina Elias; Maurício Lacerda Nogueira; Rafael dos Santos Bezerra; Raul Machado Neto; Rejane Maria Tommasini Grotto; Ricardo Haddad; Sandra Coccuzzo Sampaio Vessoni; Simone Kashima; Svetoslav Nanev Slavov; Vincent Louis Viala
EPI_ISL_2493513, EPI_ISL_2493577, EPI_ISL_2493579, EPI_ISL_2493581, EPI_ISL_2493630, EPI_ISL_2493633, EPI_ISL_2493635, EPI_ISL_2493637			
see above	SECRETARIA MUNICIPAL DE SAUDE	Instituto Butantan	Antonio Jorge Martins; Claudia Renata dos Santos Barros; David Schlesinger; Debora Botequio Moretti; Dimas Tadeu Covas; Elaine Cristina Marqueze; Elaine Vieira Santos; Evandra Strazza Rodrigues; Heidge Fukumasu; Jayme Augusto de Souza-Neto; José Salvatore Leister Patané; Luiz Alcantara; Luiz Lehmann Coutinho; Maria Carolina Elias; Maurício Lacerda Nogueira; Rafael dos Santos Bezerra; Raul Machado Neto; Rejane Maria Tommasini Grotto; Ricardo Haddad; Sandra Coccuzzo Sampaio Vessoni; Simone Kashima; Svetoslav Nanev Slavov; Vincent Louis Viala
EPI_ISL_2493367, EPI_ISL_2493396, EPI_ISL_2493427, EPI_ISL_2493428, EPI_ISL_2493452, EPI_ISL_2494158	SECRETARIA MUNICIPAL DE SAUDE DE CORDEIROPOLIS	Instituto Butantan	Antonio Jorge Martins; Claudia Renata dos Santos Barros; David Schlesinger; Debora Botequio Moretti; Dimas Tadeu Covas; Elaine Cristina Marqueze; Elaine Vieira Santos; Evandra Strazza Rodrigues; Heidge Fukumasu; Jayme Augusto de Souza-Neto; José Salvatore Leister Patané; Luiz Alcantara; Luiz Lehmann Coutinho; Maria Carolina Elias; Maurício Lacerda Nogueira; Rafael dos Santos Bezerra; Raul Machado Neto; Rejane Maria Tommasini Grotto; Ricardo Haddad; Sandra Coccuzzo Sampaio Vessoni; Simone Kashima; Svetoslav Nanev Slavov; Vincent Louis Viala
EPI_ISL_2344858	SECRETARIA MUNICIPAL DE SAUDE DE CORDEIROPOLIS	Instituto Butantan / ESALQ-Piracicaba	Antonio Jorge Martins; Claudia Renata dos Santos Barros; David Schlesinger; Debora Botequio Moretti; Dimas Tadeu Covas; Elaine Cristina Marqueze; Elaine Vieira Santos; Evandra Strazza Rodrigues; Heidge Fukumasu; Jayme Augusto de Souza-Neto; José Salvatore Leister Patané; Luiz Alcantara; Luiz Lehmann Coutinho; Maria Carolina Elias; Maurício Lacerda Nogueira; Rafael dos Santos Bezerra; Raul Machado Neto; Rejane Maria Tommasini Grotto; Ricardo Haddad; Sandra Coccuzzo Sampaio Vessoni; Simone Kashima; Svetoslav Nanev Slavov; Vincent Louis Viala
EPI_ISL_2345116, EPI_ISL_2345117, EPI_ISL_2345125, EPI_ISL_2345237, EPI_ISL_2345238	SECRETARIA MUNICIPAL DE SAUDE DE JARINU	Instituto Butantan / ESALQ-Piracicaba	Antonio Jorge Martins; Claudia Renata dos Santos Barros; David Schlesinger; Debora Botequio Moretti; Dimas Tadeu Covas; Elaine Cristina Marqueze; Elaine Vieira Santos; Evandra Strazza Rodrigues; Heidge Fukumasu; Jayme Augusto de Souza-Neto; José Salvatore Leister Patané; Luiz Alcantara; Luiz Lehmann Coutinho; Maria Carolina Elias; Maurício Lacerda Nogueira; Rafael dos Santos Bezerra; Raul Machado Neto; Rejane Maria Tommasini Grotto; Ricardo Haddad; Sandra Coccuzzo Sampaio Vessoni; Simone Kashima; Svetoslav Nanev Slavov; Vincent Louis Viala
EPI_ISL_2801338, EPI_ISL_2801339, EPI_ISL_2801340, EPI_ISL_2801351, EPI_ISL_2801353, EPI_ISL_2801354, EPI_ISL_2801356			

see above	SECRETARIA MUNICIPAL DE SAUDE DE TIANGUA	Analytical Competence Molecular Epidemiology Lab/ACME, Oswaldo Cruz Foundation, Ceara (FIOCRUZ CE)	Cleber Furtado Aksenen e Suzana Porto Almeida; Fabio Miyajima; Fernando Braga Stehling; Francisco Eder de Moura Lopes; Jamille Maria Mendes Bezerra; Joaquim César do Nascimento Sousa Junior; Pedro Miguel Carneiro Jeronimo; Thais Ferreira de Oliveira; Thais de Oliveira Costa; Ticiane Cavalcante de Souza; Veridiana Pessoa Miyajima
EPI_ISL_2445386	SECRETARIA MUNICIPAL DE SAUDE DE VALPARAISO SP	Instituto Butantan	Antonio Jorge Martins; Claudia Renata dos Santos Barros; David Schlesinger; Debora Botequio Moretti; Dimas Tadeu Covas; Elaine Cristina Marqueze; Elaine Vieira Santos; Evandra Strazza Rodrigues; Heidge Fukumasu; Jayme Augusto de Souza-Neto; José Salvatore Leister Patané; Luiz Alcantara; Luiz Lehmann Coutinho; Maria Carolina Elias; Maurício Lacerda Nogueira; Rafael dos Santos Bezerra; Raul Machado Neto; Rejane Maria Tommasini Grotto; Ricardo Haddad; Sandra Coccuzzo Sampaio Vessoni; Simone Kashima; Svetoslav Nanev Slavov; Vincent Louis Viala
EPI_ISL_3102520	SECRETARIA MUNICIPAL DE SAUDE DE VARZEA ALEGRE	Analytical Competence Molecular Epidemiology Lab/ACME, Oswaldo Cruz Foundation, Ceara (FIOCRUZ CE)	Cleber Furtado Aksenen; Fabio Miyajima; Fernando Braga Stehling; Francisco Eder de Moura Lopes; Jamille Maria Mendes Bezerra; Joaquim César do Nascimento Sousa Junior; Pedro Miguel Carneiro Jeronimo; Suzana Porto Almeida e Lucas Delerino; Thais Ferreira de Oliveira; Thais de Oliveira Costa; Ticiane Cavalcante de Souza; Veridiana Pessoa Miyajima
EPI_ISL_2493679	SECRETARIA MUNICIPAL DE SAUDE PORTO FERREIRA	Instituto Butantan	Antonio Jorge Martins; Claudia Renata dos Santos Barros; David Schlesinger; Debora Botequio Moretti; Dimas Tadeu Covas; Elaine Cristina Marqueze; Elaine Vieira Santos; Evandra Strazza Rodrigues; Heidge Fukumasu; Jayme Augusto de Souza-Neto; José Salvatore Leister Patané; Luiz Alcantara; Luiz Lehmann Coutinho; Maria Carolina Elias; Maurício Lacerda Nogueira; Rafael dos Santos Bezerra; Raul Machado Neto; Rejane Maria Tommasini Grotto; Ricardo Haddad; Sandra Coccuzzo Sampaio Vessoni; Simone Kashima; Svetoslav Nanev Slavov; Vincent Louis Viala
EPI_ISL_2493654, EPI_ISL_2493657, EPI_ISL_2493660, EPI_ISL_2493668, EPI_ISL_2493693, EPI_ISL_2493698, EPI_ISL_2500979, EPI_ISL_2500983			
see above	SECRETARIA MUNICIPAL DE SAUDE SOROCABA	Instituto Butantan	Antonio Jorge Martins; Claudia Renata dos Santos Barros; David Schlesinger; Debora Botequio Moretti; Dimas Tadeu Covas; Elaine Cristina Marqueze; Elaine Vieira Santos; Evandra Strazza Rodrigues; Heidge Fukumasu; Jayme Augusto de Souza-Neto; José Salvatore Leister Patané; Luiz Alcantara; Luiz Lehmann Coutinho; Maria Carolina Elias; Maurício Lacerda Nogueira; Rafael dos Santos Bezerra; Raul Machado Neto; Rejane Maria Tommasini Grotto; Ricardo Haddad; Sandra Coccuzzo Sampaio Vessoni; Simone Kashima; Svetoslav Nanev Slavov; Vincent Louis Viala
EPI_ISL_2344897, EPI_ISL_2344919, EPI_ISL_2344921, EPI_ISL_2344922, EPI_ISL_2344923, EPI_ISL_2344928, EPI_ISL_2344929, EPI_ISL_2344930, EPI_ISL_2344931, EPI_ISL_2344933, EPI_ISL_2344934, EPI_ISL_2344935, EPI_ISL_2344937, EPI_ISL_2344938, EPI_ISL_2344939			
see above	SECRETARIA MUNICIPAL DE SAUDE SOROCABA	Instituto Butantan / ESALQ-Piracicaba	Antonio Jorge Martins; Claudia Renata dos Santos Barros; David Schlesinger; Debora Botequio Moretti; Dimas Tadeu Covas; Elaine Cristina Marqueze; Elaine Vieira Santos; Evandra Strazza Rodrigues; Heidge Fukumasu; Jayme Augusto de Souza-Neto; José Salvatore Leister Patané; Luiz Alcantara; Luiz Lehmann Coutinho; Maria Carolina Elias; Maurício Lacerda Nogueira; Rafael dos Santos Bezerra; Raul Machado Neto; Rejane Maria Tommasini Grotto; Ricardo Haddad; Sandra Coccuzzo Sampaio Vessoni; Simone Kashima; Svetoslav Nanev Slavov; Vincent Louis Viala
EPI_ISL_2209664, EPI_ISL_2345655, EPI_ISL_2345664, EPI_ISL_2345700, EPI_ISL_2345805, EPI_ISL_2345848, EPI_ISL_2345852			
see above	SECRETARIA MUNICIPAL DE SAUDE SOROCABA	Instituto Butantan / Mendelics	Antonio Jorge Martins; Claudia Renata dos Santos Barros; David Schlesinger; Debora Botequio Moretti; Dimas Tadeu Covas; Elaine Cristina Marqueze; Elaine Vieira Santos; Evandra Strazza Rodrigues; Heidge Fukumasu; Jayme Augusto de Souza-Neto; José Salvatore Leister Patané; Luiz Alcantara; Luiz Lehmann Coutinho; Maria Carolina Elias; Maurício Lacerda Nogueira; Rafael dos Santos Bezerra; Raul Machado Neto; Rejane Maria Tommasini Grotto; Ricardo Haddad; Sandra Coccuzzo Sampaio Vessoni; Simone Kashima; Svetoslav Nanev Slavov; Vincent Louis Viala
EPI_ISL_2493562, EPI_ISL_2493565, EPI_ISL_2493567, EPI_ISL_2493568, EPI_ISL_2493569	SERV DE VIG SANITARIA EPIDEMIO E CTRL DE ZOONOZES GUARUJA	Instituto Butantan	Antonio Jorge Martins; Claudia Renata dos Santos Barros; David Schlesinger; Debora Botequio Moretti; Dimas Tadeu Covas; Elaine Cristina Marqueze; Elaine Vieira Santos; Evandra Strazza Rodrigues; Heidge Fukumasu; Jayme Augusto de Souza-Neto; José Salvatore Leister Patané; Luiz Alcantara; Luiz Lehmann Coutinho; Maria Carolina Elias; Maurício Lacerda Nogueira; Rafael dos Santos Bezerra; Raul Machado Neto; Rejane Maria Tommasini Grotto; Ricardo Haddad; Sandra Coccuzzo Sampaio Vessoni; Simone Kashima; Svetoslav Nanev Slavov; Vincent Louis Viala
EPI_ISL_2209341, EPI_ISL_2345810	SERV DE VIG SANITARIA EPIDEMIO E CTRL DE ZOONOZES GUARUJA	Instituto Butantan / Mendelics	Antonio Jorge Martins; Claudia Renata dos Santos Barros; David Schlesinger; Debora Botequio Moretti; Dimas Tadeu Covas; Elaine Cristina Marqueze; Elaine Vieira Santos; Evandra Strazza Rodrigues; Heidge Fukumasu; Jayme Augusto de Souza-Neto; José Salvatore Leister Patané; Luiz Alcantara; Luiz Lehmann Coutinho; Maria Carolina Elias; Maurício Lacerda Nogueira; Rafael dos Santos Bezerra; Raul Machado Neto; Rejane Maria Tommasini Grotto; Ricardo Haddad; Sandra Coccuzzo Sampaio Vessoni; Simone Kashima; Svetoslav Nanev Slavov; Vincent Louis Viala
EPI_ISL_2344940	SMS IPERO	Instituto Butantan / ESALQ-Piracicaba	Antonio Jorge Martins; Claudia Renata dos Santos Barros; David Schlesinger; Debora Botequio Moretti; Dimas Tadeu Covas; Elaine Cristina Marqueze; Elaine Vieira Santos; Evandra Strazza Rodrigues; Heidge Fukumasu; Jayme Augusto de Souza-Neto; José Salvatore Leister Patané; Luiz Alcantara; Luiz Lehmann Coutinho; Maria Carolina Elias; Maurício Lacerda Nogueira; Rafael dos Santos Bezerra; Raul Machado Neto; Rejane Maria Tommasini Grotto; Ricardo Haddad; Sandra Coccuzzo Sampaio Vessoni; Simone Kashima; Svetoslav Nanev Slavov; Vincent Louis Viala
EPI_ISL_2209419	SMS IPERO	Instituto Butantan / Mendelics	Antonio Jorge Martins; Claudia Renata dos Santos Barros; David Schlesinger; Debora Botequio Moretti; Dimas Tadeu Covas; Elaine Cristina Marqueze; Elaine Vieira Santos; Evandra Strazza Rodrigues; Heidge Fukumasu; Jayme Augusto de Souza-Neto; José Salvatore Leister Patané; Luiz Alcantara; Luiz Lehmann Coutinho; Maria Carolina Elias; Maurício Lacerda Nogueira; Rafael dos Santos Bezerra; Raul Machado Neto; Rejane Maria Tommasini Grotto; Ricardo Haddad; Sandra Coccuzzo Sampaio Vessoni; Simone Kashima; Svetoslav Nanev Slavov; Vincent Louis Viala
EPI_ISL_2344888, EPI_ISL_2344913	SMS SECRETARIA MUNICIPAL DE SAUDE DE BOITUVA	Instituto Butantan / ESALQ-Piracicaba	Antonio Jorge Martins; Claudia Renata dos Santos Barros; David Schlesinger; Debora Botequio Moretti; Dimas Tadeu Covas; Elaine Cristina Marqueze; Elaine Vieira Santos; Evandra Strazza Rodrigues; Heidge Fukumasu; Jayme Augusto de Souza-Neto; José Salvatore Leister Patané; Luiz Alcantara; Luiz Lehmann Coutinho; Maria Carolina Elias; Maurício Lacerda Nogueira; Rafael dos Santos Bezerra; Raul Machado Neto; Rejane Maria Tommasini Grotto; Ricardo Haddad; Sandra Coccuzzo Sampaio Vessoni; Simone Kashima; Svetoslav Nanev Slavov; Vincent Louis Viala
EPI_ISL_2210223, EPI_ISL_2345713, EPI_ISL_2345718	SMS SECRETARIA MUNICIPAL DE SAUDE DE BOITUVA	Instituto Butantan / Mendelics	Antonio Jorge Martins; Claudia Renata dos Santos Barros; David Schlesinger; Debora Botequio Moretti; Dimas Tadeu Covas; Elaine Cristina Marqueze; Elaine Vieira Santos; Evandra Strazza Rodrigues; Heidge Fukumasu; Jayme Augusto de Souza-Neto; José Salvatore Leister Patané; Luiz Alcantara; Luiz Lehmann Coutinho; Maria Carolina Elias; Maurício Lacerda Nogueira; Rafael dos Santos Bezerra; Raul Machado Neto; Rejane Maria Tommasini Grotto; Ricardo Haddad; Sandra Coccuzzo Sampaio Vessoni; Simone Kashima; Svetoslav Nanev Slavov; Vincent Louis Viala
EPI_ISL_2097297, EPI_ISL_2274580, EPI_ISL_2421052, EPI_ISL_2422317	STONY BROOK UNIVERSITY HOSPITAL	Wadsworth Center, New York State Department of Health	Alexis Russell; Catharine Prussing; Daryl M. Lamson; Erasmus Schneider; Erica Lasek-Nesselquist; John Kelly; Jonathan Plitnick; Kirsten St. George; Matthew Shudt; Melissa A Leisner; Navjot Singh
EPI_ISL_2098138	SUNY UPSTATE MEDICAL UNIVERSITY	Wadsworth Center, New York State Department of Health	Alexis Russell; Catharine Prussing; Daryl M. Lamson; Erasmus Schneider; Erica Lasek-Nesselquist; John Kelly; Jonathan Plitnick; Kirsten St. George; Matthew Shudt; Melissa A Leisner; Navjot Singh
EPI_ISL_2004328	SYNLAB	Instituto Nacional de Saude (INSA)	Borges et al
EPI_ISL_1848074	SYNLAB Jena Oncoscreen	Robert Koch Institute	
EPI_ISL_2105731, EPI_ISL_2105794, EPI_ISL_2105847, EPI_ISL_2105848, EPI_ISL_2105857, EPI_ISL_2230900, EPI_ISL_2230903, EPI_ISL_2319198, EPI_ISL_2319216, EPI_ISL_2319233, EPI_ISL_2319236, EPI_ISL_2319286, EPI_ISL_2349882, EPI_ISL_2349884, EPI_ISL_2350133, EPI_ISL_2444402, EPI_ISL_2444506, EPI_ISL_2444518, EPI_ISL_2444545			
see above	Salud Digna	Instituto Nacional de Medicina Genomica	Abraham Campos-Romero; Cedro-Tanda A; Escobar-Arrazola; Gonzalez-Barrera D; Herrera-Montalvo LA.; Hidalgo-Miranda A; Luna-Ruiz Marco; M; Mendoza-Vargas A; Moreno-Camacho José Luis; Munguia-Garza P; Ramirez-Vega O; Rangel-DeLeon D; Reyes-Grajeda JP; Rodriguez-Gallegos Jorge
EPI_ISL_2231178, EPI_ISL_2329537, EPI_ISL_2329595, EPI_ISL_2329601, EPI_ISL_2329608, EPI_ISL_2422993, EPI_ISL_2423003, EPI_ISL_2423011, EPI_ISL_2423013, EPI_ISL_2423017, EPI_ISL_2628598, EPI_ISL_2628614, EPI_ISL_2628627, EPI_ISL_2628630, EPI_ISL_2628677, EPI_ISL_2628786, EPI_ISL_2628844, EPI_ISL_2628904			
see above	San Diego County Public Health Laboratory	Andersen lab at Scripps Research	Brett Austin; Jovan Shephard; SEARCH Alliance San Diego with Ashleigh Murphy
EPI_ISL_2840867, EPI_ISL_2840895	San Diego County Public Health Laboratory	San Diego County Public Health Laboratory	Ashleigh Murphy Schafer; Brett Austin
EPI_ISL_1970975, EPI_ISL_1970978, EPI_ISL_2287924	San Juan Regional Medical Center	New Mexico Department of Health Scientific Laboratory	Anastacia Griego-Fisher; D'eldra Malone; Ellie Johnson; Jennifer Benoit; Linda Salazar; Mark Willmon; Ratheesh Rajan
EPI_ISL_2631213, EPI_ISL_2659047, EPI_ISL_2659075, EPI_ISL_2659111, EPI_ISL_2659114	Santa Clara Valley Medical Center	Santa Clara County Public Health Laboratory	Santa Clara County Public Health Department
EPI_ISL_1969513, EPI_ISL_1969518, EPI_ISL_1969532, EPI_ISL_3373746, EPI_ISL_3543807	Scripps Medical Laboratory	Andersen lab at Scripps Research	Ellen Stefanski; Ian Mchardy; SEARCH Alliance San Diego with Michael Quigley
EPI_ISL_2179729, EPI_ISL_2179731, EPI_ISL_2179732, EPI_ISL_2179752, EPI_ISL_2179753, EPI_ISL_2179754, EPI_ISL_2179758, EPI_ISL_2179794, EPI_ISL_2179796, EPI_ISL_2179797, EPI_ISL_2179802			
see above	Servicio Microbiología Hospital La Paz	Servicio Microbiología Hospital La Paz	Elie Dahdouh; Fernando Lázaro; Jesús Mingorance Cruz; Rubén Cáceres
EPI_ISL_2136127, EPI_ISL_2136130, EPI_ISL_2136132, EPI_ISL_2140045, EPI_ISL_2140060, EPI_ISL_2140078, EPI_ISL_2140084, EPI_ISL_2140086, EPI_ISL_2140093, EPI_ISL_2140106, EPI_ISL_2140135, EPI_ISL_2140143, EPI_ISL_2140144, EPI_ISL_2158741, EPI_ISL_2158831, EPI_ISL_2158843, EPI_ISL_4082187, EPI_ISL_4082200, EPI_ISL_4082202, EPI_ISL_4082206, EPI_ISL_4082207, EPI_ISL_4082215, EPI_ISL_4082216, EPI_ISL_4082219, EPI_ISL_4082221, EPI_ISL_4082246, EPI_ISL_4082286, EPI_ISL_4082295, EPI_ISL_4082298, EPI_ISL_4082310, EPI_ISL_4082330, EPI_ISL_4082338, EPI_ISL_4082344, EPI_ISL_4082354, EPI_ISL_4082356, EPI_ISL_4082358, EPI_ISL_4082372, EPI_ISL_4082405, EPI_ISL_4082426, EPI_ISL_4082459, EPI_ISL_4082489, EPI_ISL_4082493, EPI_ISL_4082525, EPI_ISL_4082526, EPI_ISL_4082527, EPI_ISL_4082539, EPI_ISL_4082624, EPI_ISL_4082648, EPI_ISL_4082653, EPI_ISL_4082661, EPI_ISL_4082721, EPI_ISL_4082766, EPI_ISL_4083068, EPI_ISL_4083069			
see above	Servicio Virosis Respiratorias-Departamento Virología-INEI	Instituto Nacional Enfermedades Infecciosas C.G.Malbran	Avaro M.; Baumeister E.; Benedetti E.; Campos J.; Cisterna D.; Dattero ME; De Belder D.; Haim MS.; Lorenzo F.; Molina V.; Perandones C.; Poklepovich T.; Pontoriero A.; Russo M.; Sanchez Loria J.; Tuduri E.
EPI_ISL_2104452, EPI_ISL_2426641, EPI_ISL_2426651, EPI_ISL_2558027	Servicio de Microbiologia Hospital Ramon y Cajal	Servicio de Microbiologia Hospital Ramon y Cajal	Galan JC; Martinez L. Abreu M; Ponce M; y Gonzalez-Alba JM
EPI_ISL_1922315, EPI_ISL_1922316, EPI_ISL_1922318, EPI_ISL_1922321, EPI_ISL_1922394, EPI_ISL_1922397, EPI_ISL_1922479, EPI_ISL_2133001, EPI_ISL_2133002, EPI_ISL_2510576, EPI_ISL_2510579, EPI_ISL_2510590, EPI_ISL_2510591, EPI_ISL_2510593			
see above	Servicio de Microbiología Clínica (Complejo Hospitalario de Navarra, Pamplona)	Centro de Secuenciación NASERTIC	Ana Miqueleiz; Ana Navascués; Carmen Ezpeleta Baquedano
EPI_ISL_2187013	Servicio de Microbiología, Hospital General Universitario de Ciudad Real	Servicio de Microbiología, Hospital General Universitario de Ciudad Real	Jose Martinez Alarcon; Soledad Illescas
EPI_ISL_2545376, EPI_ISL_2545401, EPI_ISL_2545405	Servicio de Microbiología, Laboratori Clínic Metropolitana Nord. Hospital Universitari Germans Trias i Pujol. Institut d'Investigació en Ciències de la Salut Germans Trias i Pujol (IGTP)	SeqCOVID-SPAIN consortium/IBV(CSIC)	Adrián Antuori; Anabel Fernández; Anna Not; Antoni E. Bordoy; Cristina Casañ and SeqCOVID-SPAIN consortium; Elisa Martró; Nona Romaní; Verónica Saludes
EPI_ISL_2271613, EPI_ISL_2362312,	Servicio de Microbiología. Consorcio Hospital General Universitario de	SeqCOVID-SPAIN consortium/IBV(CSIC)	Begoña Fuster Escrivá; Carme Salvador García; Concepción Gimeno Cardona and SeqCOVID-SPAIN consortium; María Dolores Ocete; Rafael Medina González

EPI_ISL_2658196, EPI_ISL_2658198	Valencia		
EPI_ISL_2016629	Servicio de Microbiología. Hospital Clínico Universitario de Valencia	SeqCOVID-SPAIN consortium/IBV(CSIC)	David Navarro Ortega; Eliseo Albert Vicent; Ignacio Torres and SeqCOVID-SPAIN consortium
EPI_ISL_2627507	Servicio de Microbiología. Hospital Universitario Doctor Peset	SeqCOVID-SPAIN consortium/IBV(CSIC)	José Miguel Nogueira Coito and SeqCOVID-SPAIN consortium; Juan Alberola Enguídanos; Juan José Camarena Miñana; Rosa González Pellicer
EPI_ISL_2162847	Servizo de Microbioloxía. Complexo Hospitalario de Santiago de Compostela	Servizo de Microbioloxía. Complexo Hospitalario de Santiago de Compostela	Amparo Coira; Antonio Aguilera; Carlos García_Riestra; Daniel Navarro; Gema Barbeito; Iria Roca; José Llovo; Laura Millán; Manuela Hernández; María Luisa Pérez_del_Molino; Mercedes Treviño; Rocío trastoy; Teresa Lopez_Valiño; Xosé Costa
EPI_ISL_2231176, EPI_ISL_2231177, EPI_ISL_2231179, EPI_ISL_2231180, EPI_ISL_2231181, EPI_ISL_2231182, EPI_ISL_2231184, EPI_ISL_2231185, EPI_ISL_2329450, EPI_ISL_2329452, EPI_ISL_2329453, EPI_ISL_2329459, EPI_ISL_2329462, EPI_ISL_2329463, EPI_ISL_2422816, EPI_ISL_2422833			
see above	Sharp HealthCare Laboratory	Andersen lab at Scripps Research	Art Mendoza; Cathy Woerle; Jacquelyn Berumen; Liam McGinnis; Omid Bakhtar; SEARCH Alliance San Diego with Aaron Harding
EPI_ISL_2296014, EPI_ISL_2296019, EPI_ISL_2296024, EPI_ISL_2296034, EPI_ISL_2296044, EPI_ISL_2801143, EPI_ISL_2801144, EPI_ISL_2801145, EPI_ISL_2801146, EPI_ISL_2801147, EPI_ISL_2801148, EPI_ISL_2801149, EPI_ISL_2801150, EPI_ISL_2801151			
see above	Simple Laboratories	Gagnon Lab, Southern Illinois University	Keith Gagnon
EPI_ISL_2230739, EPI_ISL_2484886	Southern Nevada Public Health Laboratory	Southern Nevada Public Health Laboratory	Michael Picker
EPI_ISL_2102287, EPI_ISL_2510856, EPI_ISL_2510888, EPI_ISL_2510973, EPI_ISL_2510976	Spital Limmattal	Institute of Medical Virology	Alexandra Trkola; Annette AudigŽ; Catharine Aquino; Cyril Shah; Daniel Ehrsam; Gabriela Ziltener; Guido Bloemberg; Hubert Rehrauer; Isabel StŸrmer; Joel Wirz; Jon Huder; JŸrg Bšni; Kevin Steiner; Maria GrŸnberg; Maryam Zaheri; Michael Huber; Riccarda Capaul; Stefan Schmutz; Verena Kufner; Weihong Qi
EPI_ISL_2136954, EPI_ISL_2136986, EPI_ISL_2136995, EPI_ISL_2449879	Spital MŠnnedorf AG	Institute of Medical Virology	Alexandra Trkola; Annette AudigŽ; Catharine Aquino; Cyril Shah; Daniel Ehrsam; Gabriela Ziltener; Guido Bloemberg; Hubert Rehrauer; Isabel StŸrmer; Joel Wirz; Jon Huder; JŸrg Bšni; Kevin Steiner; Maria GrŸnberg; Maryam Zaheri; Michael Huber; Riccarda Capaul; Stefan Schmutz; Verena Kufner; Weihong Qi
EPI_ISL_3418888, EPI_ISL_3418895, EPI_ISL_3418896, EPI_ISL_3418957, EPI_ISL_3418973	StarMed Healthcare	UNC Charlotte Environmental Monitoring Laboratory	Adam France; Cynthia Gibas; Erin Stiers; Jannatul Ferdous; Jessica Schlueter; Kevin Lambirth; Sam Kunkleman
EPI_ISL_2007997, EPI_ISL_2135446, EPI_ISL_2135447, EPI_ISL_2135474, EPI_ISL_2724535, EPI_ISL_2724590, EPI_ISL_2724624, EPI_ISL_2724635, EPI_ISL_2724646, EPI_ISL_2724669, EPI_ISL_2724720, EPI_ISL_2724730, EPI_ISL_2724739, EPI_ISL_2724807			
see above	State Laboratories Division, Hawaii State Department of Health	State Laboratories Division, Hawaii State Department of Health	Ayana Garnet; Drew Kuwazaki; Edward Desmond; Pamela O'Brien; Razvan Sultana
EPI_ISL_2378181, EPI_ISL_2378182, EPI_ISL_2378546, EPI_ISL_2378592, EPI_ISL_2472922, EPI_ISL_2472954, EPI_ISL_2473147, EPI_ISL_2473153, EPI_ISL_2473183, EPI_ISL_2473255, EPI_ISL_2473270, EPI_ISL_2473299, EPI_ISL_2473310, EPI_ISL_2473366, EPI_ISL_2473368, EPI_ISL_2473382, EPI_ISL_2473390, EPI_ISL_2473409, EPI_ISL_2473423, EPI_ISL_2473478, EPI_ISL_2473489, EPI_ISL_2473495, EPI_ISL_2473523, EPI_ISL_2473526, EPI_ISL_2473537, EPI_ISL_2473557, EPI_ISL_2473561, EPI_ISL_2473579, EPI_ISL_2473638, EPI_ISL_2800583			
see above	State Testing Facility	Altius Institute for Biomedical Research	Alex Isner; Alex Nguyen; Amanda Gale; Audra Johnson; Clem Green; Daniel Bates; Eric Thorland; Jacob Rodriguez; Jean Robinston; Jemma Nelson; Jessica Kunder; John Stamatoyannopoulos; Joshua Richards; Julia Wald; Kneshay Harper; Lauren Mitchell; Mark Frerker; Matt Hartman; Michael Buckley; Muhammad Halimun; Rebecca Bruders; Sadie Patraw; Sofia Olsson; Tobias Ragoczy
EPI_ISL_2391791	Sun Clinical Laboratory	Los Angeles County Public Health Laboratories	P. Hemarajata et al.
EPI_ISL_2692135	Swedish Covenant Hospital	RIPHL at Rush University Medical Center	Chicago Department of Public Health; Felix Araujo Perez; Kevin Kunstman; Laura Furtado; Marieta Hyde; Max Kolton; Stefan Green
EPI_ISL_2033275, EPI_ISL_2033311, EPI_ISL_2257985, EPI_ISL_2413880, EPI_ISL_2413987, EPI_ISL_2413998, EPI_ISL_2414004, EPI_ISL_2414029, EPI_ISL_2414091, EPI_ISL_2414220, EPI_ISL_2414270, EPI_ISL_2581053, EPI_ISL_2581084, EPI_ISL_2581124, EPI_ISL_2581130, EPI_ISL_2581444, EPI_ISL_2980127, EPI_ISL_3157893			
see above	Swedish national genomic surveillance program of SARS-CoV-2	The Public Health Agency of Sweden	Alma Brolund; Maria Lind Karlberg; Maximilian Riess; Swedish national genomic surveillance program of SARS-CoV-2
EPI_ISL_2421182	TEMPUS LABS INC	Wadsworth Center, New York State Department of Health	Alexis Russell; Catharine Prussing; Daryl M. Lamson; Erasmus Schneider; Erica Lasek-Nesselquist; John Kelly; Jonathan Plitnick; Kirsten St. George; Matthew Shudt; Melissa A Leisner; Navjot Singh
EPI_ISL_2281451, EPI_ISL_2281501, EPI_ISL_2423702, EPI_ISL_2424098, EPI_ISL_2491282, EPI_ISL_3088567	TGen North	TGen North	"Jolene Bowers; Ashlyn Pfeiffer; Brett Van Tassel; Chris French; Darrin Lemmer; Dave Engelthaler; Dave Engelthaler"; Hayley Yaglom; Heather Centner; Jolene Bowers; The Arizona COVID Genomics Union (ACGU); The Arizona COVID Genomics Union (ACGU)"
EPI_ISL_2450366	TN Division of Laboratory Services	Genomics and Discovery, Respiratory Viruses Branch, Division of Viral Diseases, Centers for Disease Control and Prevention	Adam Retchless; Anna Kelleher; Anna Uehara; Brian Lynch; Clinton R. Paden; Dhwani Batra; Haibin Wang; Han Jia Justin Ng; Jasmine Padilla; Jing Zhang; Justin Lee; Krista Queen; Mark Burroughs; Mili Sheth; Morgan Davis; Peter Cook; Rachel Marine; Sarah Nobles; Suxiang Tong; Tara Coalter; Yan Li; Ying Tao
EPI_ISL_2451912	TX DSHS, Lab Services Section MC 1947	Centers for Disease Control and Prevention Division of Viral Diseases, Pathogen Discovery	Alison Laufer Halpin; Ben L. Rambo-Martin; Clinton R. Paden; Dakota Howard; Darlene Wagner; Dave Wentworth; Dhwani Batra; Jasmine Padilla; Justin Lee; Katie Dillon; Krista Queen; Kristen Knipe; Kristine Lacek; Mark Burroughs; Matthew Schmerer; Mili Sheth; Peter Cook; Sam Shepard; Sarah Nobles; Shoshona Le; Suxiang Tong; Vivien Dugan; Yvette Unoarumhi
EPI_ISL_2649802, EPI_ISL_2649809, EPI_ISL_2649815, EPI_ISL_2790522, EPI_ISL_2790532, EPI_ISL_2790544, EPI_ISL_2966835			
see above	Tampa General Hospital Esoteric Lab	Tampa General Hospital Esoteric Research & Development Lab	Amorce Lima; Deanna Becker; Dominic Uy; Elaine Vendrone; Jon Faughn; Jorge Mecias-Frias; Suzane Silbert; Vicki Healer
EPI_ISL_2501617	Texas Children's Hospital	Texas Children's Microbiome Center	Ila Singh; James Dunn; James Versalovic; Jennifer K. Spinler; Ruth Ann Luna
EPI_ISL_1826530, EPI_ISL_1970663, EPI_ISL_1970674, EPI_ISL_2133353, EPI_ISL_2230024, EPI_ISL_2230026, EPI_ISL_2346333, EPI_ISL_2346334, EPI_ISL_2346335, EPI_ISL_2346338			
see above	The Jackson Laboratory	The Jackson Laboratory	Adams M; Bergeron D; Kelly K; Li L; Long J; Omerza G; Renzette N
EPI_ISL_3930751	The National University Hospital of Iceland	deCODE genetics	Agnar Helgason; Alma Moller; Arna B Agustsdottir; Arnaldur Gylfason; Asgeir Sigurdsson; Aslaug Jonasdottir; Berglind Eiriksdottir; Bjarni Thorbjornsson; Brynjar O Jensson; Daniel F Gudbjartsson; Droplaug N Magnusdottir; Elisabet E Gardarsdottir; Emil A Thorarensen; Gardar Sveinbjornsson; Gisli Masson; Gudmundur Georgsson; Gudmundur L Norddahl; Gudrun Sigmundsdottir; Hakon Jonsson; Hannes Eggertsson; Hilma Holm; Ingileif Jonsdottir; Jona Saemundsdottir; Kamilla S Josefsdottir; Kari Stefansson; Karl G Kristinsson; Kjartan R Gudmundsson; Kristin E Sveinsdottir; Kristjan E Hjorleifsson; Louise le Roux; Maney Sveinsdottir; Olafia S Gretarsdottir; Olafur T Magnusson; Pall Melsted; Patrick Sulem; Run Fridriksdottir; Solvi Rognvaldsson; Thora R Gunnarsdottir; Thordur Kristjansson; Thorolfur Gudnason; Unnur Thorsteinsdottir
EPI_ISL_2834212	The Ohio State University Applied Microbiology Services Laboratory	The Ohio State University Applied Microbiology Services Laboratory	Seth A. Faith PhD
EPI_ISL_2454361	TriCore Reference Laboratories	Center for Global Health, University of New Mexico Health Sciences Center	Cecilia Thompson; Darrell Dinwiddie; Daryl Domman; Karissa Culbreath; Kendra Pesko; Kurt Schwalm; Valerie Morley
EPI_ISL_2598094, EPI_ISL_2598100	TriCore Reference Laboratories	Center for Global Health, University of New Mexico Health Sciences Center	Cecilia Thompson; Darrell Dinwiddie; Daryl Domman; Karissa Culbreath; Kendra Pesko; Kurt Schwalm; Valerie Morley
EPI_ISL_2208930	UBS ALAMBARI	Instituto Butantan / Mendelics	Antonio Jorge Martins; Claudia Renata dos Santos Barros; David Schlesinger; Debora Botequio Moretti; Dimas Tadeu Covas; Elaine Cristina Marqueze; Elaine Vieira Santos; Evandra Strazza Rodrigues; Heidge Fukumasu; Jayme Augusto de Souza-Neto; José Salvatore Leister Patané; Luiz Alcantara; Luiz Lehmann Coutinho; Maria Carolina Elias; Maurício Lacerda Nogueira; Rafael dos Santos Bezerra; Raul Machado Neto; Rejane Maria Tommasini Grotto; Ricardo Haddad; Sandra Coccuzzo Sampaio Vessoni; Simone Kashima; Svetoslav Nanev Slavov; Vincent Louis Viala
EPI_ISL_2345154, EPI_ISL_2345155, EPI_ISL_2345157	UBS BONFIM	Instituto Butantan / ESALQ-Piracicaba	Antonio Jorge Martins; Claudia Renata dos Santos Barros; David Schlesinger; Debora Botequio Moretti; Dimas Tadeu Covas; Elaine Cristina Marqueze; Elaine Vieira Santos; Evandra Strazza Rodrigues; Heidge Fukumasu; Jayme Augusto de Souza-Neto; José Salvatore Leister Patané; Luiz Alcantara; Luiz Lehmann Coutinho; Maria Carolina Elias; Maurício Lacerda Nogueira; Rafael dos Santos Bezerra; Raul Machado Neto; Rejane Maria Tommasini Grotto; Ricardo Haddad; Sandra Coccuzzo Sampaio Vessoni; Simone Kashima; Svetoslav Nanev Slavov; Vincent Louis Viala
EPI_ISL_2445173	UBS CAIC OURINHOS	Instituto Butantan	Antonio Jorge Martins; Claudia Renata dos Santos Barros; David Schlesinger; Debora Botequio Moretti; Dimas Tadeu Covas; Elaine Cristina Marqueze; Elaine Vieira Santos; Evandra Strazza Rodrigues; Heidge Fukumasu; Jayme Augusto de Souza-Neto; José Salvatore Leister Patané; Luiz Alcantara; Luiz Lehmann Coutinho; Maria Carolina Elias; Maurício Lacerda Nogueira; Rafael dos Santos Bezerra; Raul Machado Neto; Rejane Maria Tommasini Grotto; Ricardo Haddad; Sandra Coccuzzo Sampaio Vessoni; Simone Kashima; Svetoslav Nanev Slavov; Vincent Louis Viala
EPI_ISL_2445207, EPI_ISL_2473718	UBS CELESTINO CARNIELO	Instituto Butantan	Antonio Jorge Martins; Claudia Renata dos Santos Barros; David Schlesinger; Debora Botequio Moretti; Dimas Tadeu Covas; Elaine Cristina Marqueze; Elaine Vieira Santos; Evandra Strazza Rodrigues; Heidge Fukumasu; Jayme Augusto de Souza-Neto; José Salvatore Leister Patané; Luiz Alcantara; Luiz Lehmann Coutinho; Maria Carolina Elias; Maurício Lacerda Nogueira; Rafael dos Santos Bezerra; Raul Machado Neto; Rejane Maria Tommasini Grotto; Ricardo Haddad; Sandra Coccuzzo Sampaio Vessoni; Simone Kashima; Svetoslav Nanev Slavov; Vincent Louis Viala
EPI_ISL_2445208, EPI_ISL_2473719, EPI_ISL_2493655, EPI_ISL_2493656,	UBS DE PARAISO	Instituto Butantan	Antonio Jorge Martins; Claudia Renata dos Santos Barros; David Schlesinger; Debora Botequio Moretti; Dimas Tadeu Covas; Elaine Cristina Marqueze; Elaine Vieira Santos; Evandra Strazza Rodrigues; Heidge Fukumasu; Jayme Augusto de Souza-Neto; José Salvatore Leister Patané; Luiz Alcantara; Luiz Lehmann Coutinho; Maria Carolina Elias; Maurício Lacerda Nogueira; Rafael dos Santos Bezerra; Raul Machado Neto; Rejane Maria Tommasini Grotto; Ricardo Haddad; Sandra Coccuzzo Sampaio Vessoni; Simone Kashima; Svetoslav Nanev Slavov; Vincent Louis Viala

EPI_ISL_2493658			
EPI_ISL_2445528, EPI_ISL_2493418	UBS DE PEDRANOPOLIS	Instituto Butantan	Antonio Jorge Martins; Claudia Renata dos Santos Barros; David Schlesinger; Debora Botequio Moretti; Dimas Tadeu Covas; Elaine Cristina Marqueze; Elaine Vieira Santos; Evandra Strazza Rodrigues; Heidge Fukumasu; Jayme Augusto de Souza-Neto; José Salvatore Leister Patané; Luiz Alcantara; Luiz Lehmann Coutinho; Maria Carolina Elias; Maurício Lacerda Nogueira; Rafael dos Santos Bezerra; Raul Machado Neto; Rejane Maria Tommasini Grotto; Ricardo Haddad; Sandra Coccuzzo Sampaio Vessoni; Simone Kashima; Svetoslav Nanev Slavov; Vincent Louis Viala
EPI_ISL_2344847	UBS DE PEDRANOPOLIS	Instituto Butantan / ESALQ-Piracicaba	Antonio Jorge Martins; Claudia Renata dos Santos Barros; David Schlesinger; Debora Botequio Moretti; Dimas Tadeu Covas; Elaine Cristina Marqueze; Elaine Vieira Santos; Evandra Strazza Rodrigues; Heidge Fukumasu; Jayme Augusto de Souza-Neto; José Salvatore Leister Patané; Luiz Alcantara; Luiz Lehmann Coutinho; Maria Carolina Elias; Maurício Lacerda Nogueira; Rafael dos Santos Bezerra; Raul Machado Neto; Rejane Maria Tommasini Grotto; Ricardo Haddad; Sandra Coccuzzo Sampaio Vessoni; Simone Kashima; Svetoslav Nanev Slavov; Vincent Louis Viala
EPI_ISL_2344850	UBS DE SAO JOAO DAS DUAS PONTES	Instituto Butantan / ESALQ-Piracicaba	Antonio Jorge Martins; Claudia Renata dos Santos Barros; David Schlesinger; Debora Botequio Moretti; Dimas Tadeu Covas; Elaine Cristina Marqueze; Elaine Vieira Santos; Evandra Strazza Rodrigues; Heidge Fukumasu; Jayme Augusto de Souza-Neto; José Salvatore Leister Patané; Luiz Alcantara; Luiz Lehmann Coutinho; Maria Carolina Elias; Maurício Lacerda Nogueira; Rafael dos Santos Bezerra; Raul Machado Neto; Rejane Maria Tommasini Grotto; Ricardo Haddad; Sandra Coccuzzo Sampaio Vessoni; Simone Kashima; Svetoslav Nanev Slavov; Vincent Louis Viala
EPI_ISL_2445195	UBS DE SUD MENNUCCI	Instituto Butantan	Antonio Jorge Martins; Claudia Renata dos Santos Barros; David Schlesinger; Debora Botequio Moretti; Dimas Tadeu Covas; Elaine Cristina Marqueze; Elaine Vieira Santos; Evandra Strazza Rodrigues; Heidge Fukumasu; Jayme Augusto de Souza-Neto; José Salvatore Leister Patané; Luiz Alcantara; Luiz Lehmann Coutinho; Maria Carolina Elias; Maurício Lacerda Nogueira; Rafael dos Santos Bezerra; Raul Machado Neto; Rejane Maria Tommasini Grotto; Ricardo Haddad; Sandra Coccuzzo Sampaio Vessoni; Simone Kashima; Svetoslav Nanev Slavov; Vincent Louis Viala
EPI_ISL_2445206, EPI_ISL_2493402, EPI_ISL_2493404, EPI_ISL_2493409, EPI_ISL_2493423, EPI_ISL_2493425, EPI_ISL_2493426, EPI_ISL_2494337			
see above	UBS DE TABAPUA	Instituto Butantan	Antonio Jorge Martins; Claudia Renata dos Santos Barros; David Schlesinger; Debora Botequio Moretti; Dimas Tadeu Covas; Elaine Cristina Marqueze; Elaine Vieira Santos; Evandra Strazza Rodrigues; Heidge Fukumasu; Jayme Augusto de Souza-Neto; José Salvatore Leister Patané; Luiz Alcantara; Luiz Lehmann Coutinho; Maria Carolina Elias; Maurício Lacerda Nogueira; Rafael dos Santos Bezerra; Raul Machado Neto; Rejane Maria Tommasini Grotto; Ricardo Haddad; Sandra Coccuzzo Sampaio Vessoni; Simone Kashima; Svetoslav Nanev Slavov; Vincent Louis Viala
EPI_ISL_2445536, EPI_ISL_2493659, EPI_ISL_2493665, EPI_ISL_2493666	UBS DE UBARANA	Instituto Butantan	Antonio Jorge Martins; Claudia Renata dos Santos Barros; David Schlesinger; Debora Botequio Moretti; Dimas Tadeu Covas; Elaine Cristina Marqueze; Elaine Vieira Santos; Evandra Strazza Rodrigues; Heidge Fukumasu; Jayme Augusto de Souza-Neto; José Salvatore Leister Patané; Luiz Alcantara; Luiz Lehmann Coutinho; Maria Carolina Elias; Maurício Lacerda Nogueira; Rafael dos Santos Bezerra; Raul Machado Neto; Rejane Maria Tommasini Grotto; Ricardo Haddad; Sandra Coccuzzo Sampaio Vessoni; Simone Kashima; Svetoslav Nanev Slavov; Vincent Louis Viala
EPI_ISL_2345190, EPI_ISL_2345191	UBS DR ABELARDO PINHEIRO GUIMARAES SCRPARDO	Instituto Butantan / ESALQ-Piracicaba	Antonio Jorge Martins; Claudia Renata dos Santos Barros; David Schlesinger; Debora Botequio Moretti; Dimas Tadeu Covas; Elaine Cristina Marqueze; Elaine Vieira Santos; Evandra Strazza Rodrigues; Heidge Fukumasu; Jayme Augusto de Souza-Neto; José Salvatore Leister Patané; Luiz Alcantara; Luiz Lehmann Coutinho; Maria Carolina Elias; Maurício Lacerda Nogueira; Rafael dos Santos Bezerra; Raul Machado Neto; Rejane Maria Tommasini Grotto; Ricardo Haddad; Sandra Coccuzzo Sampaio Vessoni; Simone Kashima; Svetoslav Nanev Slavov; Vincent Louis Viala
EPI_ISL_2345156, EPI_ISL_2345158, EPI_ISL_2345159, EPI_ISL_2345162, EPI_ISL_2345163, EPI_ISL_2345166, EPI_ISL_2345167, EPI_ISL_2345168, EPI_ISL_2345173, EPI_ISL_2345175, EPI_ISL_2345179			
see above	UBS DR ALFREDO DANTAS DE SOUZA UMUARAMA	Instituto Butantan / ESALQ-Piracicaba	Antonio Jorge Martins; Claudia Renata dos Santos Barros; David Schlesinger; Debora Botequio Moretti; Dimas Tadeu Covas; Elaine Cristina Marqueze; Elaine Vieira Santos; Evandra Strazza Rodrigues; Heidge Fukumasu; Jayme Augusto de Souza-Neto; José Salvatore Leister Patané; Luiz Alcantara; Luiz Lehmann Coutinho; Maria Carolina Elias; Maurício Lacerda Nogueira; Rafael dos Santos Bezerra; Raul Machado Neto; Rejane Maria Tommasini Grotto; Ricardo Haddad; Sandra Coccuzzo Sampaio Vessoni; Simone Kashima; Svetoslav Nanev Slavov; Vincent Louis Viala
EPI_ISL_1967205, EPI_ISL_1967206, EPI_ISL_1967207, EPI_ISL_1967208, EPI_ISL_1967209, EPI_ISL_2345646, EPI_ISL_2345745			
see above	UBS DR ALFREDO DANTAS DE SOUZA UMUARAMA	Instituto Butantan / Mendelics	Antonio Jorge Martins; Bianca Cechetto Carlos. Mendelics: Bibiana Santos; Claudia Renata dos Santos Barros; Cíntia Bittar; David Schlesinger; David Schlesinger. Hemocentro Ribeirão Preto: Simone Kashima; Debora Botequio Moretti; Dimas Tadeu Covas; Elaine Cristina Marqueze; Elaine Vieira Santos; Elaine Vieira dos Santos; Elisangela Chicaroni Mattos; Erika Freitas; Evandra Strazza Rodrigues; Felipe Allan da Silva da Costa; Flavia Aburjaile; Fábio Sossai Possebon; Guilherme Campos; Guilherme Targino Valente; Heidge Fukumasu; Heidge Fukumasu. USP-Botucatu: Rejane Maria Tommasini Grotto; Helena Lage Ferreira; Instituto Butantan: Dimas Tadeu Covas; Jardelina de Souza Todao Bernardino; Jayme A. Souza-Neto; Jayme Augusto de Souza-Neto; Jessika Cristina Chagas Lesbon; Jorge A. Petroli Marchesi; José Salvatore Leister Patané; João Paulo Kitajima; João Pessoa Araújo Jr.; Leila Sabrina Ullmann; Loyze Paola Oliveira de Lima; Luiz Alcantara; Luiz Aurelio de Campos Crispin. Centro de Genômica Funcional da ESALQ: Luiz Lehmann Coutinho; Luiz Carlos Junior de Alcantara; Luiz Lehmann Coutinho; Lívia Sacchetto; Maisa C. Pereira Parra; Maria Carolina Elias; Marta Giovanetti; Marília Moraes; Maurício Lacerda Nogueira; Maurício Lacerda Nogueira. Prefeitura de Sao Paulo: Melissa Palmieri.; Patricia Akemi Assato; Paula Rahal; Paulo Inacio da Costa; Rafael dos Santos Bezerra; Raquel de Lello Rocha Campos Cassano. NGS Soluções Genômicas: Pilar Drummond Sampaio Corrêa Mariani. FZEA-USP Pirassununga: Mirele Daiana Poleti; Raul Machado Neto; Rejane Maria Tommasini Grotto; Ricardo Augusto Brassaloti; Ricardo Haddad; Rodrigo Tocantins Calado. FAMERP-SJRP: Cecília Artico Banho; Sandra Coccuzzo Sampaio; Sandra Coccuzzo Sampaio Vessoni; Simone Kashima; Svetoslav Nanev Slavov; Vagner Fonseca; Vincent Louis Viala
EPI_ISL_2345803	UBS DR ERNESTO GAION	Instituto Butantan / Mendelics	Antonio Jorge Martins; Claudia Renata dos Santos Barros; David Schlesinger; Debora Botequio Moretti; Dimas Tadeu Covas; Elaine Cristina Marqueze; Elaine Vieira Santos; Evandra Strazza Rodrigues; Heidge Fukumasu; Jayme Augusto de Souza-Neto; José Salvatore Leister Patané; Luiz Alcantara; Luiz Lehmann Coutinho; Maria Carolina Elias; Maurício Lacerda Nogueira; Rafael dos Santos Bezerra; Raul Machado Neto; Rejane Maria Tommasini Grotto; Ricardo Haddad; Sandra Coccuzzo Sampaio Vessoni; Simone Kashima; Svetoslav Nanev Slavov; Vincent Louis Viala
EPI_ISL_2345757	UBS DR WALDOMIRO FERREIRA NEVES SCRPARDO	Instituto Butantan / Mendelics	Antonio Jorge Martins; Claudia Renata dos Santos Barros; David Schlesinger; Debora Botequio Moretti; Dimas Tadeu Covas; Elaine Cristina Marqueze; Elaine Vieira Santos; Evandra Strazza Rodrigues; Heidge Fukumasu; Jayme Augusto de Souza-Neto; José Salvatore Leister Patané; Luiz Alcantara; Luiz Lehmann Coutinho; Maria Carolina Elias; Maurício Lacerda Nogueira; Rafael dos Santos Bezerra; Raul Machado Neto; Rejane Maria Tommasini Grotto; Ricardo Haddad; Sandra Coccuzzo Sampaio Vessoni; Simone Kashima; Svetoslav Nanev Slavov; Vincent Louis Viala
EPI_ISL_2345822, EPI_ISL_2345858	UBS II DE ALVARES MACHADO	Instituto Butantan / Mendelics	Antonio Jorge Martins; Claudia Renata dos Santos Barros; David Schlesinger; Debora Botequio Moretti; Dimas Tadeu Covas; Elaine Cristina Marqueze; Elaine Vieira Santos; Evandra Strazza Rodrigues; Heidge Fukumasu; Jayme Augusto de Souza-Neto; José Salvatore Leister Patané; Luiz Alcantara; Luiz Lehmann Coutinho; Maria Carolina Elias; Maurício Lacerda Nogueira; Rafael dos Santos Bezerra; Raul Machado Neto; Rejane Maria Tommasini Grotto; Ricardo Haddad; Sandra Coccuzzo Sampaio Vessoni; Simone Kashima; Svetoslav Nanev Slavov; Vincent Louis Viala
EPI_ISL_2493278, EPI_ISL_2493326, EPI_ISL_2493327, EPI_ISL_2493328, EPI_ISL_2493341, EPI_ISL_2493343, EPI_ISL_2493346, EPI_ISL_2493348, EPI_ISL_2493349, EPI_ISL_2493350, EPI_ISL_2493351, EPI_ISL_2493352, EPI_ISL_2493353, EPI_ISL_2500984			
see above	UBS II DE PIRAPOZINHO C SERVICO DE EACS E ESF	Instituto Butantan	Antonio Jorge Martins; Claudia Renata dos Santos Barros; David Schlesinger; Debora Botequio Moretti; Dimas Tadeu Covas; Elaine Cristina Marqueze; Elaine Vieira Santos; Evandra Strazza Rodrigues; Heidge Fukumasu; Jayme Augusto de Souza-Neto; José Salvatore Leister Patané; Luiz Alcantara; Luiz Lehmann Coutinho; Maria Carolina Elias; Maurício Lacerda Nogueira; Rafael dos Santos Bezerra; Raul Machado Neto; Rejane Maria Tommasini Grotto; Ricardo Haddad; Sandra Coccuzzo Sampaio Vessoni; Simone Kashima; Svetoslav Nanev Slavov; Vincent Louis Viala
EPI_ISL_2445482, EPI_ISL_2445484	UBS II DE REGENTE FEIJO	Instituto Butantan	Antonio Jorge Martins; Claudia Renata dos Santos Barros; David Schlesinger; Debora Botequio Moretti; Dimas Tadeu Covas; Elaine Cristina Marqueze; Elaine Vieira Santos; Evandra Strazza Rodrigues; Heidge Fukumasu; Jayme Augusto de Souza-Neto; José Salvatore Leister Patané; Luiz Alcantara; Luiz Lehmann Coutinho; Maria Carolina Elias; Maurício Lacerda Nogueira; Rafael dos Santos Bezerra; Raul Machado Neto; Rejane Maria Tommasini Grotto; Ricardo Haddad; Sandra Coccuzzo Sampaio Vessoni; Simone Kashima; Svetoslav Nanev Slavov; Vincent Louis Viala
EPI_ISL_2209618	UBS II DE REGENTE FEIJO	Instituto Butantan / Mendelics	Antonio Jorge Martins; Claudia Renata dos Santos Barros; David Schlesinger; Debora Botequio Moretti; Dimas Tadeu Covas; Elaine Cristina Marqueze; Elaine Vieira Santos; Evandra Strazza Rodrigues; Heidge Fukumasu; Jayme Augusto de Souza-Neto; José Salvatore Leister Patané; Luiz Alcantara; Luiz Lehmann Coutinho; Maria Carolina Elias; Maurício Lacerda Nogueira; Rafael dos Santos Bezerra; Raul Machado Neto; Rejane Maria Tommasini Grotto; Ricardo Haddad; Sandra Coccuzzo Sampaio Vessoni; Simone Kashima; Svetoslav Nanev Slavov; Vincent Louis Viala
EPI_ISL_2445202, EPI_ISL_2445516, EPI_ISL_2445518, EPI_ISL_2445520, EPI_ISL_2445534	UBS II DE TANABI MILTON MARTINS PERCHES	Instituto Butantan	Antonio Jorge Martins; Claudia Renata dos Santos Barros; David Schlesinger; Debora Botequio Moretti; Dimas Tadeu Covas; Elaine Cristina Marqueze; Elaine Vieira Santos; Evandra Strazza Rodrigues; Heidge Fukumasu; Jayme Augusto de Souza-Neto; José Salvatore Leister Patané; Luiz Alcantara; Luiz Lehmann Coutinho; Maria Carolina Elias; Maurício Lacerda Nogueira; Rafael dos Santos Bezerra; Raul Machado Neto; Rejane Maria Tommasini Grotto; Ricardo Haddad; Sandra Coccuzzo Sampaio Vessoni; Simone Kashima; Svetoslav Nanev Slavov; Vincent Louis Viala
EPI_ISL_2445552, EPI_ISL_2445555, EPI_ISL_2473796	UBS II DR JOSE CARLOS MACUCO JANINI	Instituto Butantan	Antonio Jorge Martins; Claudia Renata dos Santos Barros; David Schlesinger; Debora Botequio Moretti; Dimas Tadeu Covas; Elaine Cristina Marqueze; Elaine Vieira Santos; Evandra Strazza Rodrigues; Heidge Fukumasu; Jayme Augusto de Souza-Neto; José Salvatore Leister Patané; Luiz Alcantara; Luiz Lehmann Coutinho; Maria Carolina Elias; Maurício Lacerda Nogueira; Rafael dos Santos Bezerra; Raul Machado Neto; Rejane Maria Tommasini Grotto; Ricardo Haddad; Sandra Coccuzzo Sampaio Vessoni; Simone Kashima; Svetoslav Nanev Slavov; Vincent Louis Viala
EPI_ISL_2209600, EPI_ISL_2210148	UBS II DR JOSE CARLOS MACUCO JANINI	Instituto Butantan / Mendelics	Antonio Jorge Martins; Claudia Renata dos Santos Barros; David Schlesinger; Debora Botequio Moretti; Dimas Tadeu Covas; Elaine Cristina Marqueze; Elaine Vieira Santos; Evandra Strazza Rodrigues; Heidge Fukumasu; Jayme Augusto de Souza-Neto; José Salvatore Leister Patané; Luiz Alcantara; Luiz Lehmann Coutinho; Maria Carolina Elias; Maurício Lacerda Nogueira; Rafael dos Santos Bezerra; Raul Machado Neto; Rejane Maria Tommasini Grotto; Ricardo Haddad; Sandra Coccuzzo Sampaio Vessoni; Simone Kashima; Svetoslav Nanev Slavov; Vincent Louis Viala
EPI_ISL_2493531, EPI_ISL_2493545, EPI_ISL_2493550	UBS III DE PARIQUERA ACU PARIQUERA ACU	Instituto Butantan	Antonio Jorge Martins; Claudia Renata dos Santos Barros; David Schlesinger; Debora Botequio Moretti; Dimas Tadeu Covas; Elaine Cristina Marqueze; Elaine Vieira Santos; Evandra Strazza Rodrigues; Heidge Fukumasu; Jayme Augusto de Souza-Neto; José Salvatore Leister Patané; Luiz Alcantara; Luiz Lehmann Coutinho; Maria Carolina Elias; Maurício Lacerda Nogueira; Rafael dos Santos Bezerra; Raul Machado Neto; Rejane Maria Tommasini Grotto; Ricardo Haddad; Sandra Coccuzzo Sampaio Vessoni; Simone Kashima; Svetoslav Nanev Slavov; Vincent Louis Viala
EPI_ISL_2209494	UBS III DE PARIQUERA ACU PARIQUERA ACU	Instituto Butantan / Mendelics	Antonio Jorge Martins; Claudia Renata dos Santos Barros; David Schlesinger; Debora Botequio Moretti; Dimas Tadeu Covas; Elaine Cristina Marqueze; Elaine Vieira Santos; Evandra Strazza Rodrigues; Heidge Fukumasu; Jayme Augusto de Souza-Neto; José Salvatore Leister Patané; Luiz Alcantara; Luiz Lehmann Coutinho; Maria Carolina Elias; Maurício Lacerda Nogueira; Rafael dos Santos Bezerra; Raul Machado Neto; Rejane Maria Tommasini Grotto; Ricardo Haddad; Sandra Coccuzzo Sampaio Vessoni; Simone Kashima; Svetoslav Nanev Slavov; Vincent Louis Viala
EPI_ISL_2493686, EPI_ISL_2493699	UBS III DE RANCHARIA	Instituto Butantan	Antonio Jorge Martins; Claudia Renata dos Santos Barros; David Schlesinger; Debora Botequio Moretti; Dimas Tadeu Covas; Elaine Cristina Marqueze; Elaine Vieira Santos; Evandra Strazza Rodrigues; Heidge Fukumasu; Jayme Augusto de Souza-Neto; José Salvatore Leister Patané; Luiz Alcantara; Luiz Lehmann Coutinho; Maria Carolina Elias; Maurício Lacerda Nogueira; Rafael dos Santos Bezerra; Raul Machado Neto; Rejane Maria Tommasini Grotto; Ricardo Haddad; Sandra Coccuzzo Sampaio Vessoni; Simone Kashima; Svetoslav Nanev Slavov; Vincent Louis Viala
EPI_ISL_2345666, EPI_ISL_2345710	UBS III DE RANCHARIA	Instituto Butantan / Mendelics	Antonio Jorge Martins; Claudia Renata dos Santos Barros; David Schlesinger; Debora Botequio Moretti; Dimas Tadeu Covas; Elaine Cristina Marqueze; Elaine Vieira Santos; Evandra Strazza Rodrigues; Heidge Fukumasu; Jayme Augusto de Souza-Neto; José Salvatore Leister Patané; Luiz Alcantara; Luiz Lehmann Coutinho; Maria Carolina Elias; Maurício Lacerda Nogueira; Rafael dos Santos Bezerra; Raul Machado Neto; Rejane Maria Tommasini Grotto; Ricardo Haddad; Sandra Coccuzzo Sampaio Vessoni; Simone Kashima; Svetoslav Nanev Slavov; Vincent Louis Viala
EPI_ISL_2445543	UBS III DR BENITO MALZONE	Instituto Butantan	Antonio Jorge Martins; Claudia Renata dos Santos Barros; David Schlesinger; Debora Botequio Moretti; Dimas Tadeu Covas; Elaine Cristina Marqueze; Elaine Vieira Santos; Evandra Strazza Rodrigues; Heidge Fukumasu; Jayme Augusto de Souza-Neto; José Salvatore Leister Patané; Luiz Alcantara; Luiz Lehmann Coutinho; Maria Carolina Elias; Maurício Lacerda Nogueira; Rafael dos Santos Bezerra; Raul Machado Neto; Rejane Maria Tommasini Grotto; Ricardo Haddad; Sandra Coccuzzo Sampaio Vessoni; Simone Kashima; Svetoslav Nanev Slavov; Vincent Louis Viala
EPI_ISL_2345193, EPI_ISL_2345196	UBS III MURUTINGA DO SUL	Instituto Butantan / ESALQ-Piracicaba	Antonio Jorge Martins; Claudia Renata dos Santos Barros; David Schlesinger; Debora Botequio Moretti; Dimas Tadeu Covas; Elaine Cristina Marqueze; Elaine Vieira Santos; Evandra Strazza Rodrigues; Heidge Fukumasu; Jayme Augusto de Souza-Neto; José Salvatore Leister Patané; Luiz Alcantara; Luiz Lehmann Coutinho; Maria Carolina Elias; Maurício Lacerda Nogueira; Rafael dos Santos Bezerra; Raul Machado Neto; Rejane Maria Tommasini Grotto; Ricardo Haddad; Sandra Coccuzzo Sampaio Vessoni; Simone Kashima; Svetoslav Nanev Slavov; Vincent Louis Viala
EPI_ISL_2345223, EPI_ISL_2345225, EPI_ISL_2345228, EPI_ISL_2345229, EPI_ISL_2345230, EPI_ISL_2345231, EPI_ISL_2345232, EPI_ISL_2345233, EPI_ISL_2345234, EPI_ISL_2345235			
see above	UBS IV GUARARAPES	Instituto Butantan / ESALQ-Piracicaba	Antonio Jorge Martins; Claudia Renata dos Santos Barros; David Schlesinger; Debora Botequio Moretti; Dimas Tadeu Covas; Elaine Cristina Marqueze; Elaine Vieira Santos; Evandra Strazza Rodrigues; Heidge Fukumasu; Jayme Augusto de Souza-Neto; José Salvatore Leister Patané; Luiz Alcantara; Luiz Lehmann Coutinho; Maria Carolina Elias; Maurício Lacerda Nogueira; Rafael dos Santos Bezerra; Raul Machado Neto; Rejane Maria Tommasini Grotto; Ricardo Haddad; Sandra Coccuzzo Sampaio Vessoni; Simone Kashima; Svetoslav Nanev Slavov; Vincent Louis Viala
EPI_ISL_1967285, EPI_ISL_1967286	UBS JARDIM CABUCU	Instituto Butantan / Mendelics	Antonio Jorge Martins; Bianca Cechetto Carlos. Mendelics: Bibiana Santos; Claudia Renata dos Santos Barros; Cíntia Bittar; David Schlesinger. Hemocentro Ribeirão Preto: Simone Kashima; Debora Botequio Moretti; Elaine Cristina Marqueze; Elaine Vieira dos Santos; Elisangela Chicaroni Mattos; Erika Freitas; Evandra Strazza Rodrigues; Felipe Allan da Silva da Costa; Flavia Aburjaile; Fábio Sossai Possebon; Guilherme Campos; Guilherme Targino Valente; Heidge Fukumasu. USP-Botucatu: Rejane Maria Tommasini Grotto; Helena Lage Ferreira; Instituto Butantan: Dimas Tadeu Covas; Jardelina de Souza Todao Bernardino; Jayme A. Souza-Neto; Jessika Cristina Chagas Lesbon; Jorge A. Petroli Marchesi; José Salvatore Leister Patané; João Paulo Kitajima; João Pessoa Araújo Jr.; Leila Sabrina Ullmann; Loyze Paola Oliveira de Lima; Luiz Aurelio de Campos Crispin. Centro de Genômica Funcional da ESALQ: Luiz Lehmann Coutinho; Luiz Carlos Junior de Alcantara; Lívia Sacchetto; Maisa C. Pereira Parra; Maria Carolina Elias; Marta Giovanetti; Marília Moraes; Maurício Lacerda Nogueira. Prefeitura de Sao Paulo: Melissa Palmieri.; Patricia Akemi Assato; Paula Rahal; Paulo Inacio da Costa; Rafael dos Santos Bezerra; Raquel de Lello Rocha Campos Cassano. NGS Soluções Genômicas: Pilar Drummond Sampaio Corrêa Mariani. FZEA-USP Pirassununga: Mirele Daiana Poleti; Raul Machado Neto; Ricardo Augusto Brassaloti; Ricardo Haddad; Rodrigo Tocantins Calado. FAMERP-SJRP: Cecília Artico Banho; Sandra Coccuzzo Sampaio; Svetoslav Nanev Slavov; Vagner Fonseca; Vincent Louis Viala
EPI_ISL_2171000	UBS JARDIM MUNHOZ	Instituto Butantan / Mendelics	Antonio Jorge Martins; Bianca Cechetto Carlos. Mendelics: Bibiana Santos; Claudia Renata dos Santos Barros; Cíntia Bittar; David Schlesinger. Hemocentro Ribeirão Preto: Simone Kashima; Debora Botequio Moretti; Elaine Cristina Marqueze; Elaine Vieira dos Santos; Elisangela Chicaroni Mattos; Erika Freitas; Evandra Strazza Rodrigues; Felipe Allan da Silva da Costa; Flavia Aburjaile; Fábio Sossai Possebon; Guilherme Campos; Guilherme Targino Valente; Heidge Fukumasu. USP-Botucatu: Rejane Maria Tommasini Grotto; Helena Lage Ferreira; Instituto Butantan: Dimas Tadeu Covas; Jardelina de Souza Todao Bernardino; Jayme A. Souza-Neto; Jessika Cristina Chagas Lesbon; Jorge A. Petroli Marchesi; José Salvatore Leister Patané; João Paulo Kitajima; João Pessoa Araújo Jr.; Leila Sabrina Ullmann; Loyze Paola Oliveira de Lima; Luiz Aurelio de Campos Crispin. Centro de Genômica Funcional da ESALQ: Luiz Lehmann Coutinho; Luiz Carlos Junior de Alcantara; Lívia Sacchetto; Maisa C. Pereira Parra; Maria Carolina Elias; Marta Giovanetti; Marília Moraes; Maurício Lacerda Nogueira. Prefeitura de Sao Paulo: Melissa Palmieri.; Patricia Akemi Assato; Paula Rahal; Paulo Inacio da Costa; Rafael dos Santos Bezerra; Raquel de Lello Rocha Campos Cassano. NGS Soluções Genômicas: Pilar Drummond Sampaio Corrêa Mariani. FZEA-USP Pirassununga: Mirele Daiana Poleti; Raul Machado Neto; Ricardo Augusto Brassaloti; Ricardo Haddad; Rodrigo Tocantins Calado. FAMERP-SJRP: Cecília Artico Banho; Sandra Coccuzzo Sampaio; Svetoslav Nanev Slavov; Vagner Fonseca; Vincent Louis Viala
EPI_ISL_2344902	UBS JARDIM VITORINO IPERO	Instituto Butantan / ESALQ-Piracicaba	Antonio Jorge Martins; Claudia Renata dos Santos Barros; David Schlesinger; Debora Botequio Moretti; Dimas Tadeu Covas; Elaine Cristina Marqueze; Elaine Vieira Santos; Evandra Strazza Rodrigues; Heidge Fukumasu; Jayme Augusto de Souza-Neto; José Salvatore Leister Patané; Luiz Alcantara; Luiz Lehmann Coutinho; Maria Carolina Elias; Maurício Lacerda Nogueira; Rafael dos Santos Bezerra; Raul Machado Neto; Rejane Maria Tommasini Grotto; Ricardo Haddad; Sandra Coccuzzo Sampaio Vessoni; Simone Kashima; Svetoslav Nanev Slavov; Vincent Louis Viala
EPI_ISL_2344825	UBS JD SALTENSE	Instituto Butantan / ESALQ-Piracicaba	Antonio Jorge Martins; Claudia Renata dos Santos Barros; David Schlesinger; Debora Botequio Moretti; Dimas Tadeu Covas; Elaine Cristina Marqueze; Elaine Vieira Santos; Evandra Strazza Rodrigues; Heidge Fukumasu; Jayme Augusto de Souza-Neto; José Salvatore Leister Patané; Luiz Alcantara; Luiz Lehmann Coutinho; Maria Carolina Elias; Maurício Lacerda Nogueira; Rafael dos Santos Bezerra; Raul Machado Neto; Rejane Maria Tommasini Grotto; Ricardo Haddad; Sandra Coccuzzo Sampaio Vessoni; Simone Kashima; Svetoslav Nanev Slavov; Vincent Louis Viala
EPI_ISL_2493663	UBS LUIS FACHIN IPIGUA	Instituto Butantan	Antonio Jorge Martins; Claudia Renata dos Santos Barros; David Schlesinger; Debora Botequio Moretti; Dimas Tadeu Covas; Elaine Cristina Marqueze; Elaine Vieira Santos; Evandra Strazza Rodrigues; Heidge Fukumasu; Jayme Augusto de Souza-Neto; José Salvatore Leister Patané; Luiz Alcantara; Luiz Lehmann Coutinho; Maria Carolina Elias; Maurício Lacerda Nogueira; Rafael dos Santos Bezerra; Raul Machado Neto; Rejane Maria Tommasini Grotto; Ricardo Haddad; Sandra Coccuzzo Sampaio Vessoni; Simone Kashima; Svetoslav Nanev Slavov; Vincent Louis Viala
EPI_ISL_2493584	UBS MARIA IZABEL	Instituto Butantan	Antonio Jorge Martins; Claudia Renata dos Santos Barros; David Schlesinger; Debora Botequio Moretti; Dimas Tadeu Covas; Elaine Cristina Marqueze; Elaine Vieira Santos; Evandra Strazza Rodrigues; Heidge Fukumasu; Jayme Augusto de Souza-Neto; José Salvatore Leister Patané; Luiz Alcantara; Luiz Lehmann Coutinho; Maria Carolina Elias; Maurício Lacerda Nogueira; Rafael dos Santos Bezerra; Raul Machado Neto; Rejane Maria Tommasini Grotto; Ricardo Haddad; Sandra Coccuzzo Sampaio Vessoni; Simone Kashima; Svetoslav Nanev Slavov; Vincent Louis Viala
EPI_ISL_2445198, EPI_ISL_2445199, EPI_ISL_2445200, EPI_ISL_2445201, EPI_ISL_2445522, EPI_ISL_2445524, EPI_ISL_2473714, EPI_ISL_2473715			
see above	UBS PASTOR MARCELINO DEUNGARO RIO GRANDE	Instituto Butantan	Antonio Jorge Martins; Claudia Renata dos Santos Barros; David Schlesinger; Debora Botequio Moretti; Dimas Tadeu Covas; Elaine Cristina Marqueze; Elaine Vieira Santos; Evandra Strazza Rodrigues; Heidge Fukumasu; Jayme Augusto de Souza-Neto; José Salvatore Leister Patané; Luiz Alcantara; Luiz Lehmann Coutinho; Maria Carolina Elias; Maurício Lacerda Nogueira; Rafael dos Santos Bezerra; Raul Machado Neto; Rejane Maria Tommasini Grotto; Ricardo Haddad; Sandra Coccuzzo Sampaio Vessoni; Simone Kashima; Svetoslav Nanev Slavov; Vincent Louis Viala
EPI_ISL_2493293	UBS PAULO HUMBERTO DE FIGUEIREDO	Instituto Butantan	Antonio Jorge Martins; Claudia Renata dos Santos Barros; David Schlesinger; Debora Botequio Moretti; Dimas Tadeu Covas; Elaine Cristina Marqueze; Elaine Vieira Santos; Evandra Strazza Rodrigues; Heidge Fukumasu; Jayme Augusto de Souza-Neto; José Salvatore Leister Patané; Luiz Alcantara; Luiz Lehmann

			Coutinho; Maria Carolina Elias; Maurício Lacerda Nogueira; Rafael dos Santos Bezerra; Raul Machado Neto; Rejane Maria Tommasini Grotto; Ricardo Haddad; Sandra Coccuzzo Sampaio Vessoni; Simone Kashima; Svetoslav Nanev Slavov; Vincent Louis Viala
EPI_ISL_2445254	UBS SAO LOURENCO DA SERRA	Instituto Butantan	Antonio Jorge Martins; Claudia Renata dos Santos Barros; David Schlesinger; Debora Botequio Moretti; Dimas Tadeu Covas; Elaine Cristina Marqueze; Elaine Vieira Santos; Evandra Strazza Rodrigues; Heidge Fukumasu; Jayme Augusto de Souza-Neto; José Salvatore Leister Patané; Luiz Alcantara; Luiz Lehmann Coutinho; Maria Carolina Elias; Maurício Lacerda Nogueira; Rafael dos Santos Bezerra; Raul Machado Neto; Rejane Maria Tommasini Grotto; Ricardo Haddad; Sandra Coccuzzo Sampaio Vessoni; Simone Kashima; Svetoslav Nanev Slavov; Vincent Louis Viala
EPI_ISL_2345054	UBS SAO LOURENCO DA SERRA	Instituto Butantan / ESALQ-Piracicaba	Antonio Jorge Martins; Claudia Renata dos Santos Barros; David Schlesinger; Debora Botequio Moretti; Dimas Tadeu Covas; Elaine Cristina Marqueze; Elaine Vieira Santos; Evandra Strazza Rodrigues; Heidge Fukumasu; Jayme Augusto de Souza-Neto; José Salvatore Leister Patané; Luiz Alcantara; Luiz Lehmann Coutinho; Maria Carolina Elias; Maurício Lacerda Nogueira; Rafael dos Santos Bezerra; Raul Machado Neto; Rejane Maria Tommasini Grotto; Ricardo Haddad; Sandra Coccuzzo Sampaio Vessoni; Simone Kashima; Svetoslav Nanev Slavov; Vincent Louis Viala
EPI_ISL_2445188, EPI_ISL_2473710	UBS VILA RIBEIRO	Instituto Butantan	Antonio Jorge Martins; Claudia Renata dos Santos Barros; David Schlesinger; Debora Botequio Moretti; Dimas Tadeu Covas; Elaine Cristina Marqueze; Elaine Vieira Santos; Evandra Strazza Rodrigues; Heidge Fukumasu; Jayme Augusto de Souza-Neto; José Salvatore Leister Patané; Luiz Alcantara; Luiz Lehmann Coutinho; Maria Carolina Elias; Maurício Lacerda Nogueira; Rafael dos Santos Bezerra; Raul Machado Neto; Rejane Maria Tommasini Grotto; Ricardo Haddad; Sandra Coccuzzo Sampaio Vessoni; Simone Kashima; Svetoslav Nanev Slavov; Vincent Louis Viala
EPI_ISL_2345134	UBS VILA RIBEIRO	Instituto Butantan / ESALQ-Piracicaba	Antonio Jorge Martins; Claudia Renata dos Santos Barros; David Schlesinger; Debora Botequio Moretti; Dimas Tadeu Covas; Elaine Cristina Marqueze; Elaine Vieira Santos; Evandra Strazza Rodrigues; Heidge Fukumasu; Jayme Augusto de Souza-Neto; José Salvatore Leister Patané; Luiz Alcantara; Luiz Lehmann Coutinho; Maria Carolina Elias; Maurício Lacerda Nogueira; Rafael dos Santos Bezerra; Raul Machado Neto; Rejane Maria Tommasini Grotto; Ricardo Haddad; Sandra Coccuzzo Sampaio Vessoni; Simone Kashima; Svetoslav Nanev Slavov; Vincent Louis Viala
EPI_ISL_3419583	UC Davis Genome Center and Healthy Davis Together	UC Davis Genome Center	UC Davis
EPI_ISL_3321297, EPI_ISL_3321303, EPI_ISL_3535720	UC-Christus Clinical Hospital Laboratory	Laboratory of Molecular Virology, School of Medicine, Pontificia Universidad Catolica de Chile	Ana Maira Guzman; Andres E. Munoz-Marcos; Catalina Pardo-Roa; Eileen Serrano; Erick Salinas; Estefany Poblete; Francisco Melo; Jorge Levican; Leonardo I. Almonacid; Maria Jose Avendano; Maria Patricia Vega; Rafael A. Medina; Ricardo Enrique de la Barra; Tamara Garcia-Salum
EPI_ISL_3104695, EPI_ISL_3104696, EPI_ISL_3104697, EPI_ISL_3104698, EPI_ISL_3104699, EPI_ISL_3104700	UCC (Universidad Central del Caribe)	Grubaugh Lab - Yale School of Public Health	Anderson Brito; Andrea Firpo; Annie Watkins; Chaney Kalinich; Chantal Vogels; David Rivera Aponte; Ernesto Vazquez; Fabiola Cruz-Lopez; Fabiola Fontanet; Isabel Ott; Jessica Rothman; Joseph Fauver; Karolane Gonzalez; Kendall Billig; Mallery Breban; Mary Petrone; Natalie Machargo; Nathan Grubaugh; Patricia Serrano; Tania Mitwalli; Tara Alpert; Tobias Koch; Vianca Aponte
EPI_ISL_2444195	UCHOA	Instituto Butantan / FAMERP	Antonio Jorge Martins; Claudia Renata dos Santos Barros; David Schlesinger; Debora Botequio Moretti; Dimas Tadeu Covas; Elaine Cristina Marqueze; Elaine Vieira Santos; Evandra Strazza Rodrigues; Heidge Fukumasu; Jayme Augusto de Souza-Neto; José Salvatore Leister Patané; Luiz Alcantara; Luiz Lehmann Coutinho; Maria Carolina Elias; Maurício Lacerda Nogueira; Rafael dos Santos Bezerra; Raul Machado Neto; Rejane Maria Tommasini Grotto; Ricardo Haddad; Sandra Coccuzzo Sampaio Vessoni; Simone Kashima; Svetoslav Nanev Slavov; Vincent Louis Viala
EPI_ISL_2405167, EPI_ISL_2617950, EPI_ISL_2620751	UCLA Clinical Micro Lab	Los Angeles County Public Health Laboratories	P. Hemarajata et al.
EPI_ISL_2547290, EPI_ISL_2547291, EPI_ISL_2547292, EPI_ISL_2547299, EPI_ISL_2547312, EPI_ISL_2547314, EPI_ISL_2547315, EPI_ISL_2547316			
see above	UHTL, University Hospitals	UHTL, University Hospitals	Alouani, D.; Sadri, N.; Song, X.
EPI_ISL_2448598	ULSS 2 Marca Trevigiana	Istituto Zooprofilattico Sperimentale delle Venezie	Adelaide Milani; Alessia Schivo; Alice Fusaro; Ambra Pastori; Annalisa Salviato; Antonia Ricci; Calogero Terregino; Edoardo Giussani; Elisa Palumbo; Erika Giorgia Quaranta; Isabella Monne; Luca Tassoni
EPI_ISL_2448471	ULSS 5 Polesana	Istituto Zooprofilattico Sperimentale delle Venezie	Adelaide Milani; Alessia Schivo; Alice Fusaro; Ambra Pastori; Annalisa Salviato; Antonia Ricci; Calogero Terregino; Edoardo Giussani; Elisa Palumbo; Erika Giorgia Quaranta; Isabella Monne; Luca Tassoni
EPI_ISL_3409608, EPI_ISL_3410080	UMass Memorial Medical Center	Center for Microbiome Research	Doyle V. Ward; Karl J. Simon; Richard T. Ellison III
EPI_ISL_2493585, EPI_ISL_2493588	UNIDADE BASICA DE SAUDE DE TARUMA	Instituto Butantan	Antonio Jorge Martins; Claudia Renata dos Santos Barros; David Schlesinger; Debora Botequio Moretti; Dimas Tadeu Covas; Elaine Cristina Marqueze; Elaine Vieira Santos; Evandra Strazza Rodrigues; Heidge Fukumasu; Jayme Augusto de Souza-Neto; José Salvatore Leister Patané; Luiz Alcantara; Luiz Lehmann Coutinho; Maria Carolina Elias; Maurício Lacerda Nogueira; Rafael dos Santos Bezerra; Raul Machado Neto; Rejane Maria Tommasini Grotto; Ricardo Haddad; Sandra Coccuzzo Sampaio Vessoni; Simone Kashima; Svetoslav Nanev Slavov; Vincent Louis Viala
EPI_ISL_2345203, EPI_ISL_2345204	UNIDADE BASICA DE SAUDE DE TARUMA	Instituto Butantan / ESALQ-Piracicaba	Antonio Jorge Martins; Claudia Renata dos Santos Barros; David Schlesinger; Debora Botequio Moretti; Dimas Tadeu Covas; Elaine Cristina Marqueze; Elaine Vieira Santos; Evandra Strazza Rodrigues; Heidge Fukumasu; Jayme Augusto de Souza-Neto; José Salvatore Leister Patané; Luiz Alcantara; Luiz Lehmann Coutinho; Maria Carolina Elias; Maurício Lacerda Nogueira; Rafael dos Santos Bezerra; Raul Machado Neto; Rejane Maria Tommasini Grotto; Ricardo Haddad; Sandra Coccuzzo Sampaio Vessoni; Simone Kashima; Svetoslav Nanev Slavov; Vincent Louis Viala
EPI_ISL_2494152	UNIDADE BASICA DE SAUDE DR MATHEUS GABRIEL BONASSA	Instituto Butantan	Antonio Jorge Martins; Claudia Renata dos Santos Barros; David Schlesinger; Debora Botequio Moretti; Dimas Tadeu Covas; Elaine Cristina Marqueze; Elaine Vieira Santos; Evandra Strazza Rodrigues; Heidge Fukumasu; Jayme Augusto de Souza-Neto; José Salvatore Leister Patané; Luiz Alcantara; Luiz Lehmann Coutinho; Maria Carolina Elias; Maurício Lacerda Nogueira; Rafael dos Santos Bezerra; Raul Machado Neto; Rejane Maria Tommasini Grotto; Ricardo Haddad; Sandra Coccuzzo Sampaio Vessoni; Simone Kashima; Svetoslav Nanev Slavov; Vincent Louis Viala
EPI_ISL_2493196, EPI_ISL_2493197, EPI_ISL_2493311, EPI_ISL_2493314, EPI_ISL_2493319, EPI_ISL_2493457, EPI_ISL_2493458			
see above	UNIDADE BASICA DE SAUDE HORTOLANDIA PACS	Instituto Butantan	Antonio Jorge Martins; Claudia Renata dos Santos Barros; David Schlesinger; Debora Botequio Moretti; Dimas Tadeu Covas; Elaine Cristina Marqueze; Elaine Vieira Santos; Evandra Strazza Rodrigues; Heidge Fukumasu; Jayme Augusto de Souza-Neto; José Salvatore Leister Patané; Luiz Alcantara; Luiz Lehmann Coutinho; Maria Carolina Elias; Maurício Lacerda Nogueira; Rafael dos Santos Bezerra; Raul Machado Neto; Rejane Maria Tommasini Grotto; Ricardo Haddad; Sandra Coccuzzo Sampaio Vessoni; Simone Kashima; Svetoslav Nanev Slavov; Vincent Louis Viala
EPI_ISL_2209732, EPI_ISL_2210126, EPI_ISL_2345770	UNIDADE BASICA DE SAUDE II ESTRELA DO NORTE	Instituto Butantan / Mendelics	Antonio Jorge Martins; Claudia Renata dos Santos Barros; David Schlesinger; Debora Botequio Moretti; Dimas Tadeu Covas; Elaine Cristina Marqueze; Elaine Vieira Santos; Evandra Strazza Rodrigues; Heidge Fukumasu; Jayme Augusto de Souza-Neto; José Salvatore Leister Patané; Luiz Alcantara; Luiz Lehmann Coutinho; Maria Carolina Elias; Maurício Lacerda Nogueira; Rafael dos Santos Bezerra; Raul Machado Neto; Rejane Maria Tommasini Grotto; Ricardo Haddad; Sandra Coccuzzo Sampaio Vessoni; Simone Kashima; Svetoslav Nanev Slavov; Vincent Louis Viala
EPI_ISL_2210141, EPI_ISL_2345673, EPI_ISL_2345719	UNIDADE BASICA DE SAUDE JARDIM DO LAGO	Instituto Butantan / Mendelics	Antonio Jorge Martins; Claudia Renata dos Santos Barros; David Schlesinger; Debora Botequio Moretti; Dimas Tadeu Covas; Elaine Cristina Marqueze; Elaine Vieira Santos; Evandra Strazza Rodrigues; Heidge Fukumasu; Jayme Augusto de Souza-Neto; José Salvatore Leister Patané; Luiz Alcantara; Luiz Lehmann Coutinho; Maria Carolina Elias; Maurício Lacerda Nogueira; Rafael dos Santos Bezerra; Raul Machado Neto; Rejane Maria Tommasini Grotto; Ricardo Haddad; Sandra Coccuzzo Sampaio Vessoni; Simone Kashima; Svetoslav Nanev Slavov; Vincent Louis Viala
EPI_ISL_2445463, EPI_ISL_2493294, EPI_ISL_2493295	UNIDADE BASICA SAUDE NATAL ABADIO LACERDA	Instituto Butantan	Antonio Jorge Martins; Claudia Renata dos Santos Barros; David Schlesinger; Debora Botequio Moretti; Dimas Tadeu Covas; Elaine Cristina Marqueze; Elaine Vieira Santos; Evandra Strazza Rodrigues; Heidge Fukumasu; Jayme Augusto de Souza-Neto; José Salvatore Leister Patané; Luiz Alcantara; Luiz Lehmann Coutinho; Maria Carolina Elias; Maurício Lacerda Nogueira; Rafael dos Santos Bezerra; Raul Machado Neto; Rejane Maria Tommasini Grotto; Ricardo Haddad; Sandra Coccuzzo Sampaio Vessoni; Simone Kashima; Svetoslav Nanev Slavov; Vincent Louis Viala
EPI_ISL_2345789	UNIDADE BASICA SAUDE NATAL ABADIO LACERDA	Instituto Butantan / Mendelics	Antonio Jorge Martins; Claudia Renata dos Santos Barros; David Schlesinger; Debora Botequio Moretti; Dimas Tadeu Covas; Elaine Cristina Marqueze; Elaine Vieira Santos; Evandra Strazza Rodrigues; Heidge Fukumasu; Jayme Augusto de Souza-Neto; José Salvatore Leister Patané; Luiz Alcantara; Luiz Lehmann Coutinho; Maria Carolina Elias; Maurício Lacerda Nogueira; Rafael dos Santos Bezerra; Raul Machado Neto; Rejane Maria Tommasini Grotto; Ricardo Haddad; Sandra Coccuzzo Sampaio Vessoni; Simone Kashima; Svetoslav Nanev Slavov; Vincent Louis Viala
EPI_ISL_2493473, EPI_ISL_2493480, EPI_ISL_2493484, EPI_ISL_2493492	UNIDADE DE PRONTO ATENDIMENTO	Instituto Butantan	Antonio Jorge Martins; Claudia Renata dos Santos Barros; David Schlesinger; Debora Botequio Moretti; Dimas Tadeu Covas; Elaine Cristina Marqueze; Elaine Vieira Santos; Evandra Strazza Rodrigues; Heidge Fukumasu; Jayme Augusto de Souza-Neto; José Salvatore Leister Patané; Luiz Alcantara; Luiz Lehmann Coutinho; Maria Carolina Elias; Maurício Lacerda Nogueira; Rafael dos Santos Bezerra; Raul Machado Neto; Rejane Maria Tommasini Grotto; Ricardo Haddad; Sandra Coccuzzo Sampaio Vessoni; Simone Kashima; Svetoslav Nanev Slavov; Vincent Louis Viala
EPI_ISL_2344885, EPI_ISL_2344886	UNIDADE DE PRONTO ATENDIMENTO DE JALES	Instituto Butantan / ESALQ-Piracicaba	Antonio Jorge Martins; Claudia Renata dos Santos Barros; David Schlesinger; Debora Botequio Moretti; Dimas Tadeu Covas; Elaine Cristina Marqueze; Elaine Vieira Santos; Evandra Strazza Rodrigues; Heidge Fukumasu; Jayme Augusto de Souza-Neto; José Salvatore Leister Patané; Luiz Alcantara; Luiz Lehmann Coutinho; Maria Carolina Elias; Maurício Lacerda Nogueira; Rafael dos Santos Bezerra; Raul Machado Neto; Rejane Maria Tommasini Grotto; Ricardo Haddad; Sandra Coccuzzo Sampaio Vessoni; Simone Kashima; Svetoslav Nanev Slavov; Vincent Louis Viala
EPI_ISL_2345085, EPI_ISL_2345089, EPI_ISL_2345092, EPI_ISL_2345098, EPI_ISL_2345102, EPI_ISL_2345103, EPI_ISL_2345106			
see above	UNIDADE DE PRONTO ATENDIMENTO DE VARZEA PAULISTA UPA II	Instituto Butantan / ESALQ-Piracicaba	Antonio Jorge Martins; Claudia Renata dos Santos Barros; David Schlesinger; Debora Botequio Moretti; Dimas Tadeu Covas; Elaine Cristina Marqueze; Elaine Vieira Santos; Evandra Strazza Rodrigues; Heidge Fukumasu; Jayme Augusto de Souza-Neto; José Salvatore Leister Patané; Luiz Alcantara; Luiz Lehmann Coutinho; Maria Carolina Elias; Maurício Lacerda Nogueira; Rafael dos Santos Bezerra; Raul Machado Neto; Rejane Maria Tommasini Grotto; Ricardo Haddad; Sandra Coccuzzo Sampaio Vessoni; Simone Kashima; Svetoslav Nanev Slavov; Vincent Louis Viala
EPI_ISL_2345080, EPI_ISL_2345083, EPI_ISL_2345100, EPI_ISL_2345105, EPI_ISL_2345119	UNIDADE DE PRONTO ATENDIMENTO UPA DRA ANA OLIVIA BENTIVOGLIO	Instituto Butantan / ESALQ-Piracicaba	Antonio Jorge Martins; Claudia Renata dos Santos Barros; David Schlesinger; Debora Botequio Moretti; Dimas Tadeu Covas; Elaine Cristina Marqueze; Elaine Vieira Santos; Evandra Strazza Rodrigues; Heidge Fukumasu; Jayme Augusto de Souza-Neto; José Salvatore Leister Patané; Luiz Alcantara; Luiz Lehmann Coutinho; Maria Carolina Elias; Maurício Lacerda Nogueira; Rafael dos Santos Bezerra; Raul Machado Neto; Rejane Maria Tommasini Grotto; Ricardo Haddad; Sandra Coccuzzo Sampaio Vessoni; Simone Kashima; Svetoslav Nanev Slavov; Vincent Louis Viala
EPI_ISL_2345127, EPI_ISL_2345135	UNIDADE DE PRONTO ATENDIMENTO UPA I SANTA ISABEL	Instituto Butantan / ESALQ-Piracicaba	Antonio Jorge Martins; Claudia Renata dos Santos Barros; David Schlesinger; Debora Botequio Moretti; Dimas Tadeu Covas; Elaine Cristina Marqueze; Elaine Vieira Santos; Evandra Strazza Rodrigues; Heidge Fukumasu; Jayme Augusto de Souza-Neto; José Salvatore Leister Patané; Luiz Alcantara; Luiz Lehmann Coutinho; Maria Carolina Elias; Maurício Lacerda Nogueira; Rafael dos Santos Bezerra; Raul Machado Neto; Rejane Maria Tommasini Grotto; Ricardo Haddad; Sandra Coccuzzo Sampaio Vessoni; Simone Kashima; Svetoslav Nanev Slavov; Vincent Louis Viala
EPI_ISL_2494305	UNIDADE DE SAUDE DA FAMILIA NOVO HORIZONTE	Instituto Butantan	Antonio Jorge Martins; Claudia Renata dos Santos Barros; David Schlesinger; Debora Botequio Moretti; Dimas Tadeu Covas; Elaine Cristina Marqueze; Elaine Vieira Santos; Evandra Strazza Rodrigues; Heidge Fukumasu; Jayme Augusto de Souza-Neto; José Salvatore Leister Patané; Luiz Alcantara; Luiz Lehmann Coutinho; Maria Carolina Elias; Maurício Lacerda Nogueira; Rafael dos Santos Bezerra; Raul Machado Neto; Rejane Maria Tommasini Grotto; Ricardo Haddad; Sandra Coccuzzo Sampaio Vessoni; Simone Kashima; Svetoslav Nanev Slavov; Vincent Louis Viala
EPI_ISL_2345212, EPI_ISL_2345227	UNIDADE DE SAUDE DA FAMILIA NOVO HORIZONTE	Instituto Butantan / ESALQ-Piracicaba	Antonio Jorge Martins; Claudia Renata dos Santos Barros; David Schlesinger; Debora Botequio Moretti; Dimas Tadeu Covas; Elaine Cristina Marqueze; Elaine Vieira Santos; Evandra Strazza Rodrigues; Heidge Fukumasu; Jayme Augusto de Souza-Neto; José Salvatore Leister Patané; Luiz Alcantara; Luiz Lehmann Coutinho; Maria Carolina Elias; Maurício Lacerda Nogueira; Rafael dos Santos Bezerra; Raul Machado Neto; Rejane Maria Tommasini Grotto; Ricardo Haddad; Sandra Coccuzzo Sampaio Vessoni; Simone Kashima; Svetoslav Nanev Slavov; Vincent Louis Viala
EPI_ISL_2493526	UNIDADE DE SAUDE DE ITOBI ALCIBIADES PIRES	Instituto Butantan	Antonio Jorge Martins; Claudia Renata dos Santos Barros; David Schlesinger; Debora Botequio Moretti; Dimas Tadeu Covas; Elaine Cristina Marqueze; Elaine Vieira Santos; Evandra Strazza Rodrigues; Heidge Fukumasu; Jayme Augusto de Souza-Neto; José Salvatore Leister Patané; Luiz Alcantara; Luiz Lehmann Coutinho; Maria Carolina Elias; Maurício Lacerda Nogueira; Rafael dos Santos Bezerra; Raul Machado Neto; Rejane Maria Tommasini Grotto; Ricardo Haddad; Sandra Coccuzzo Sampaio Vessoni; Simone Kashima; Svetoslav Nanev Slavov; Vincent Louis Viala
EPI_ISL_2493297, EPI_ISL_2493298	UNIDADE DE SAUDE DR PHEBO DE OLIVEIRA ROGE FERREIRA	Instituto Butantan	Antonio Jorge Martins; Claudia Renata dos Santos Barros; David Schlesinger; Debora Botequio Moretti; Dimas Tadeu Covas; Elaine Cristina Marqueze; Elaine Vieira Santos; Evandra Strazza Rodrigues; Heidge Fukumasu; Jayme Augusto de Souza-Neto; José Salvatore Leister Patané; Luiz Alcantara; Luiz Lehmann Coutinho; Maria Carolina Elias; Maurício Lacerda Nogueira; Rafael dos Santos Bezerra; Raul Machado Neto; Rejane Maria Tommasini Grotto; Ricardo Haddad; Sandra Coccuzzo Sampaio Vessoni; Simone Kashima; Svetoslav Nanev Slavov; Vincent Louis Viala
EPI_ISL_2445480	UNIDADE DE VIGILANCIA EM SAUDE	Instituto Butantan	Antonio Jorge Martins; Claudia Renata dos Santos Barros; David Schlesinger; Debora Botequio Moretti; Dimas Tadeu Covas; Elaine Cristina Marqueze; Elaine Vieira Santos; Evandra Strazza Rodrigues; Heidge Fukumasu; Jayme Augusto de Souza-Neto; José Salvatore Leister Patané; Luiz Alcantara; Luiz Lehmann Coutinho; Maria Carolina Elias; Maurício Lacerda Nogueira; Rafael dos Santos Bezerra; Raul Machado Neto; Rejane Maria Tommasini Grotto; Ricardo Haddad; Sandra Coccuzzo Sampaio Vessoni; Simone Kashima; Svetoslav Nanev Slavov; Vincent Louis Viala
EPI_ISL_2345269, EPI_ISL_2345270, EPI_ISL_2345272, EPI_ISL_2345275, EPI_ISL_2345277, EPI_ISL_2345279, EPI_ISL_2345281, EPI_ISL_2345285, EPI_ISL_2345286, EPI_ISL_2345287			
see above	UNIDADE DE VIGILANCIA EM SAUDE	Instituto Butantan / ESALQ-Piracicaba	Antonio Jorge Martins; Claudia Renata dos Santos Barros; David Schlesinger; Debora Botequio Moretti; Dimas Tadeu Covas; Elaine Cristina Marqueze; Elaine Vieira Santos; Evandra Strazza Rodrigues; Heidge Fukumasu; Jayme Augusto de Souza-Neto; José Salvatore Leister Patané; Luiz Alcantara; Luiz Lehmann Coutinho; Maria Carolina Elias; Maurício Lacerda Nogueira; Rafael dos Santos Bezerra; Raul Machado Neto; Rejane Maria Tommasini Grotto; Ricardo Haddad; Sandra Coccuzzo Sampaio Vessoni; Simone Kashima; Svetoslav Nanev Slavov; Vincent Louis Viala
EPI_ISL_2445236, EPI_ISL_2445237	UNIDADE MISTA DE IGUAPE	Instituto Butantan	Antonio Jorge Martins; Claudia Renata dos Santos Barros; David Schlesinger; Debora Botequio Moretti; Dimas Tadeu Covas; Elaine Cristina Marqueze; Elaine Vieira Santos; Evandra Strazza Rodrigues; Heidge Fukumasu; Jayme Augusto de Souza-Neto; José Salvatore Leister Patané; Luiz Alcantara; Luiz Lehmann Coutinho; Maria Carolina Elias; Maurício Lacerda Nogueira; Rafael dos Santos Bezerra; Raul Machado Neto; Rejane Maria Tommasini Grotto; Ricardo Haddad; Sandra Coccuzzo Sampaio Vessoni; Simone Kashima; Svetoslav Nanev Slavov; Vincent Louis Viala
EPI_ISL_2493602, EPI_ISL_2493604, EPI_ISL_2493605, EPI_ISL_2493607, EPI_ISL_2493611, EPI_ISL_2493614, EPI_ISL_2493616, EPI_ISL_2493617, EPI_ISL_2493620, EPI_ISL_2493623			
see above	UNIDADE MISTA DE LUIZ ANTONIO	Instituto Butantan	Antonio Jorge Martins; Claudia Renata dos Santos Barros; David Schlesinger; Debora Botequio Moretti; Dimas Tadeu Covas; Elaine Cristina Marqueze; Elaine Vieira Santos; Evandra Strazza Rodrigues; Heidge Fukumasu; Jayme Augusto de Souza-Neto; José Salvatore Leister Patané; Luiz Alcantara; Luiz Lehmann Coutinho; Maria Carolina Elias; Maurício Lacerda Nogueira; Rafael dos Santos Bezerra; Raul Machado Neto; Rejane Maria Tommasini Grotto; Ricardo Haddad; Sandra Coccuzzo Sampaio Vessoni; Simone Kashima; Svetoslav Nanev Slavov; Vincent Louis Viala
EPI_ISL_3102542	UNIDADE MISTA DE SAUDE DE MISSAO VELHA	Analytical Competence Molecular Epidemiology Lab/ACME, Oswaldo Cruz Foundation, Ceara (FIOCRUZ CE)	Cleber Furtado Aksenen; Fabio Miyajima; Fernando Braga Stehling; Francisco Eder de Moura Lopes; Jamille Maria Mendes Bezerra; Joaquim César do Nascimento Sousa Junior; Pedro Miguel Carneiro Jeronimo; Suzana Porto Almeida e Lucas Delerino; Thais Ferreira de Oliveira; Thais de Oliveira Costa; Ticiane Cavalcante de Souza; Veridiana Pessoa Miyajima
EPI_ISL_2445196	UNIDADE MISTA DE SAUDE DE ORIENTE	Instituto Butantan	Antonio Jorge Martins; Claudia Renata dos Santos Barros; David Schlesinger; Debora Botequio Moretti; Dimas Tadeu Covas; Elaine Cristina Marqueze; Elaine Vieira Santos; Evandra Strazza Rodrigues; Heidge Fukumasu; Jayme Augusto de Souza-Neto; José Salvatore Leister Patané; Luiz Alcantara; Luiz Lehmann

			Coutinho; Maria Carolina Elias; Maurício Lacerda Nogueira; Rafael dos Santos Bezerra; Raul Machado Neto; Rejane Maria Tommasini Grotto; Ricardo Haddad; Sandra Coccuzzo Sampaio Vessoni; Simone Kashima; Svetoslav Nanev Slavov; Vincent Louis Viala
EPI_ISL_2493387, EPI_ISL_2493442	UNIDADE MISTA DE SAUDE MOMBUCA	Instituto Butantan	Antonio Jorge Martins; Claudia Renata dos Santos Barros; David Schlesinger; Debora Botequio Moretti; Dimas Tadeu Covas; Elaine Cristina Marqueze; Elaine Vieira Santos; Evandra Strazza Rodrigues; Heidge Fukumasu; Jayme Augusto de Souza-Neto; José Salvatore Leister Patané; Luiz Alcantara; Luiz Lehmann Coutinho; Maria Carolina Elias; Maurício Lacerda Nogueira; Rafael dos Santos Bezerra; Raul Machado Neto; Rejane Maria Tommasini Grotto; Ricardo Haddad; Sandra Coccuzzo Sampaio Vessoni; Simone Kashima; Svetoslav Nanev Slavov; Vincent Louis Viala
EPI_ISL_2445585	UNIDADE MISTA DE SAUDE ROSEIRA DR PAULO GUIMARAES CASTRO	Instituto Butantan	Antonio Jorge Martins; Claudia Renata dos Santos Barros; David Schlesinger; Debora Botequio Moretti; Dimas Tadeu Covas; Elaine Cristina Marqueze; Elaine Vieira Santos; Evandra Strazza Rodrigues; Heidge Fukumasu; Jayme Augusto de Souza-Neto; José Salvatore Leister Patané; Luiz Alcantara; Luiz Lehmann Coutinho; Maria Carolina Elias; Maurício Lacerda Nogueira; Rafael dos Santos Bezerra; Raul Machado Neto; Rejane Maria Tommasini Grotto; Ricardo Haddad; Sandra Coccuzzo Sampaio Vessoni; Simone Kashima; Svetoslav Nanev Slavov; Vincent Louis Viala
EPI_ISL_2493372	UNIDADE MISTA OSVALDO P CAMARGO ELIAS FAUSTO	Instituto Butantan	Antonio Jorge Martins; Claudia Renata dos Santos Barros; David Schlesinger; Debora Botequio Moretti; Dimas Tadeu Covas; Elaine Cristina Marqueze; Elaine Vieira Santos; Evandra Strazza Rodrigues; Heidge Fukumasu; Jayme Augusto de Souza-Neto; José Salvatore Leister Patané; Luiz Alcantara; Luiz Lehmann Coutinho; Maria Carolina Elias; Maurício Lacerda Nogueira; Rafael dos Santos Bezerra; Raul Machado Neto; Rejane Maria Tommasini Grotto; Ricardo Haddad; Sandra Coccuzzo Sampaio Vessoni; Simone Kashima; Svetoslav Nanev Slavov; Vincent Louis Viala
EPI_ISL_2801360	UNIDADE PRONTO ATENDIMENTO CONJUNTO CEARA	Analytical Competence Molecular Epidemiology Lab/ACME, Oswaldo Cruz Foundation, Ceara (FIOCRUZ CE)	Cleber Furtado Aksenen e Suzana Porto Almeida; Fabio Miyajima; Fernando Braga Stehling; Francisco Eder de Moura Lopes; Jamille Maria Mendes Bezerra; Joaquim César do Nascimento Sousa Junior; Pedro Miguel Carneiro Jeronimo; Thais Ferreira de Oliveira; Thais de Oliveira Costa; Ticiane Cavalcante de Souza; Veridiana Pessoa Miyajima
EPI_ISL_2801357	UNIDADE PRONTO ATENDIMENTO ITAPERI	Analytical Competence Molecular Epidemiology Lab/ACME, Oswaldo Cruz Foundation, Ceara (FIOCRUZ CE)	Cleber Furtado Aksenen e Suzana Porto Almeida; Fabio Miyajima; Fernando Braga Stehling; Francisco Eder de Moura Lopes; Jamille Maria Mendes Bezerra; Joaquim César do Nascimento Sousa Junior; Pedro Miguel Carneiro Jeronimo; Thais Ferreira de Oliveira; Thais de Oliveira Costa; Ticiane Cavalcante de Souza; Veridiana Pessoa Miyajima
EPI_ISL_2344916	UNIDADE PSF PINHEIROS APIAI	Instituto Butantan / ESALQ-Piracicaba	Antonio Jorge Martins; Claudia Renata dos Santos Barros; David Schlesinger; Debora Botequio Moretti; Dimas Tadeu Covas; Elaine Cristina Marqueze; Elaine Vieira Santos; Evandra Strazza Rodrigues; Heidge Fukumasu; Jayme Augusto de Souza-Neto; José Salvatore Leister Patané; Luiz Alcantara; Luiz Lehmann Coutinho; Maria Carolina Elias; Maurício Lacerda Nogueira; Rafael dos Santos Bezerra; Raul Machado Neto; Rejane Maria Tommasini Grotto; Ricardo Haddad; Sandra Coccuzzo Sampaio Vessoni; Simone Kashima; Svetoslav Nanev Slavov; Vincent Louis Viala
EPI_ISL_2445154, EPI_ISL_2445175, EPI_ISL_2445181, EPI_ISL_2445185, EPI_ISL_2473707, EPI_ISL_2473724, EPI_ISL_2473812			
see above	UNIDADE REFERENCIAL SUDOESTE	Instituto Butantan	Antonio Jorge Martins; Claudia Renata dos Santos Barros; David Schlesinger; Debora Botequio Moretti; Dimas Tadeu Covas; Elaine Cristina Marqueze; Elaine Vieira Santos; Evandra Strazza Rodrigues; Heidge Fukumasu; Jayme Augusto de Souza-Neto; José Salvatore Leister Patané; Luiz Alcantara; Luiz Lehmann Coutinho; Maria Carolina Elias; Maurício Lacerda Nogueira; Rafael dos Santos Bezerra; Raul Machado Neto; Rejane Maria Tommasini Grotto; Ricardo Haddad; Sandra Coccuzzo Sampaio Vessoni; Simone Kashima; Svetoslav Nanev Slavov; Vincent Louis Viala
EPI_ISL_2345079, EPI_ISL_2345109, EPI_ISL_2345136, EPI_ISL_2345139, EPI_ISL_2345160	UNIDADE REFERENCIAL SUDOESTE	Instituto Butantan / ESALQ-Piracicaba	Antonio Jorge Martins; Claudia Renata dos Santos Barros; David Schlesinger; Debora Botequio Moretti; Dimas Tadeu Covas; Elaine Cristina Marqueze; Elaine Vieira Santos; Evandra Strazza Rodrigues; Heidge Fukumasu; Jayme Augusto de Souza-Neto; José Salvatore Leister Patané; Luiz Alcantara; Luiz Lehmann Coutinho; Maria Carolina Elias; Maurício Lacerda Nogueira; Rafael dos Santos Bezerra; Raul Machado Neto; Rejane Maria Tommasini Grotto; Ricardo Haddad; Sandra Coccuzzo Sampaio Vessoni; Simone Kashima; Svetoslav Nanev Slavov; Vincent Louis Viala
EPI_ISL_2493249, EPI_ISL_2493250, EPI_ISL_2493461, EPI_ISL_2493474, EPI_ISL_2493477, EPI_ISL_2493483, EPI_ISL_2493507, EPI_ISL_2493511, EPI_ISL_2493515, EPI_ISL_2493517, EPI_ISL_2493518, EPI_ISL_2494292			
see above	UNIDADE RESPIRATORIA NOVA HORTOLANDIA	Instituto Butantan	Antonio Jorge Martins; Claudia Renata dos Santos Barros; David Schlesinger; Debora Botequio Moretti; Dimas Tadeu Covas; Elaine Cristina Marqueze; Elaine Vieira Santos; Evandra Strazza Rodrigues; Heidge Fukumasu; Jayme Augusto de Souza-Neto; José Salvatore Leister Patané; Luiz Alcantara; Luiz Lehmann Coutinho; Maria Carolina Elias; Maurício Lacerda Nogueira; Rafael dos Santos Bezerra; Raul Machado Neto; Rejane Maria Tommasini Grotto; Ricardo Haddad; Sandra Coccuzzo Sampaio Vessoni; Simone Kashima; Svetoslav Nanev Slavov; Vincent Louis Viala
EPI_ISL_2208922, EPI_ISL_2345042, EPI_ISL_2345047, EPI_ISL_2345049, EPI_ISL_2345052, EPI_ISL_2345053	UNIDADE RESPIRATORIA NOVA HORTOLANDIA	Instituto Butantan / ESALQ-Piracicaba	Antonio Jorge Martins; Claudia Renata dos Santos Barros; David Schlesinger; Debora Botequio Moretti; Dimas Tadeu Covas; Elaine Cristina Marqueze; Elaine Vieira Santos; Evandra Strazza Rodrigues; Heidge Fukumasu; Jayme Augusto de Souza-Neto; José Salvatore Leister Patané; Luiz Alcantara; Luiz Lehmann Coutinho; Maria Carolina Elias; Maurício Lacerda Nogueira; Rafael dos Santos Bezerra; Raul Machado Neto; Rejane Maria Tommasini Grotto; Ricardo Haddad; Sandra Coccuzzo Sampaio Vessoni; Simone Kashima; Svetoslav Nanev Slavov; Vincent Louis Viala
EPI_ISL_2208916	UNIDADE SENTINELA COVID19	Instituto Butantan / Mendelics	Antonio Jorge Martins; Claudia Renata dos Santos Barros; David Schlesinger; Debora Botequio Moretti; Dimas Tadeu Covas; Elaine Cristina Marqueze; Elaine Vieira Santos; Evandra Strazza Rodrigues; Heidge Fukumasu; Jayme Augusto de Souza-Neto; José Salvatore Leister Patané; Luiz Alcantara; Luiz Lehmann Coutinho; Maria Carolina Elias; Maurício Lacerda Nogueira; Rafael dos Santos Bezerra; Raul Machado Neto; Rejane Maria Tommasini Grotto; Ricardo Haddad; Sandra Coccuzzo Sampaio Vessoni; Simone Kashima; Svetoslav Nanev Slavov; Vincent Louis Viala
EPI_ISL_2445329, EPI_ISL_2493272, EPI_ISL_2493273, EPI_ISL_2493566	UPA DE BEBEDOURO	Instituto Butantan	Antonio Jorge Martins; Claudia Renata dos Santos Barros; David Schlesinger; Debora Botequio Moretti; Dimas Tadeu Covas; Elaine Cristina Marqueze; Elaine Vieira Santos; Evandra Strazza Rodrigues; Heidge Fukumasu; Jayme Augusto de Souza-Neto; José Salvatore Leister Patané; Luiz Alcantara; Luiz Lehmann Coutinho; Maria Carolina Elias; Maurício Lacerda Nogueira; Rafael dos Santos Bezerra; Raul Machado Neto; Rejane Maria Tommasini Grotto; Ricardo Haddad; Sandra Coccuzzo Sampaio Vessoni; Simone Kashima; Svetoslav Nanev Slavov; Vincent Louis Viala
EPI_ISL_2208944	UPA DE BEBEDOURO	Instituto Butantan / Mendelics	Antonio Jorge Martins; Claudia Renata dos Santos Barros; David Schlesinger; Debora Botequio Moretti; Dimas Tadeu Covas; Elaine Cristina Marqueze; Elaine Vieira Santos; Evandra Strazza Rodrigues; Heidge Fukumasu; Jayme Augusto de Souza-Neto; José Salvatore Leister Patané; Luiz Alcantara; Luiz Lehmann Coutinho; Maria Carolina Elias; Maurício Lacerda Nogueira; Rafael dos Santos Bezerra; Raul Machado Neto; Rejane Maria Tommasini Grotto; Ricardo Haddad; Sandra Coccuzzo Sampaio Vessoni; Simone Kashima; Svetoslav Nanev Slavov; Vincent Louis Viala
EPI_ISL_2445245, EPI_ISL_2445249, EPI_ISL_2445250, EPI_ISL_2445256, EPI_ISL_2445260, EPI_ISL_2445261, EPI_ISL_2445264			
see above	UPA DR AKIRA TADA	Instituto Butantan	Antonio Jorge Martins; Claudia Renata dos Santos Barros; David Schlesinger; Debora Botequio Moretti; Dimas Tadeu Covas; Elaine Cristina Marqueze; Elaine Vieira Santos; Evandra Strazza Rodrigues; Heidge Fukumasu; Jayme Augusto de Souza-Neto; José Salvatore Leister Patané; Luiz Alcantara; Luiz Lehmann Coutinho; Maria Carolina Elias; Maurício Lacerda Nogueira; Rafael dos Santos Bezerra; Raul Machado Neto; Rejane Maria Tommasini Grotto; Ricardo Haddad; Sandra Coccuzzo Sampaio Vessoni; Simone Kashima; Svetoslav Nanev Slavov; Vincent Louis Viala
EPI_ISL_2345056, EPI_ISL_2345057, EPI_ISL_2345058	UPA DR AKIRA TADA	Instituto Butantan / ESALQ-Piracicaba	Antonio Jorge Martins; Claudia Renata dos Santos Barros; David Schlesinger; Debora Botequio Moretti; Dimas Tadeu Covas; Elaine Cristina Marqueze; Elaine Vieira Santos; Evandra Strazza Rodrigues; Heidge Fukumasu; Jayme Augusto de Souza-Neto; José Salvatore Leister Patané; Luiz Alcantara; Luiz Lehmann Coutinho; Maria Carolina Elias; Maurício Lacerda Nogueira; Rafael dos Santos Bezerra; Raul Machado Neto; Rejane Maria Tommasini Grotto; Ricardo Haddad; Sandra Coccuzzo Sampaio Vessoni; Simone Kashima; Svetoslav Nanev Slavov; Vincent Louis Viala
EPI_ISL_2171026	UPA DR AKIRA TADA	Instituto Butantan / Mendelics	Antonio Jorge Martins; Bianca Cechetto Carlos. Mendelics: Bibiana Santos; Claudia Renata dos Santos Barros; Cíntia Bittar; David Schlesinger. Hemocentro Ribeirão Preto: Simone Kashima; Debora Botequio Moretti; Elaine Cristina Marqueze; Elaine Vieira dos Santos; Elisangela Chicaroni Mattos; Erika Freitas; Evandra Strazza Rodrigues; Felipe Allan da Silva da Costa; Flavia Aburjaile; Fábio Sossai Possebon; Guilherme Campos; Guilherme Targino Valente; Heidge Fukumasu. USP-Botucatu: Rejane Maria Tommasini Grotto; Helena Lage Ferreira; Instituto Butantan: Dimas Tadeu Covas; Jardelina de Souza Todao Bernardino; Jayme A. Souza-Neto; Jessika Cristina Chagas Lesbon; Jorge A. Petroli Marchesi; José Salvatore Leister Patané; João Paulo Kitajima; João Pessoa Araújo Jr.; Leila Sabrina Ullmann; Loyze Paola Oliveira de Lima; Luiz Aurelio de Campos Crispin. Centro de Genômica Funcional da ESALQ: Luiz Lehmann Coutinho; Luiz Carlos Junior de Alcantara; Lívia Sacchetto; Maisa C. Pereira Parra; Maria Carolina Elias; Marta Giovanetti; Marília Moraes; Maurício Lacerda Nogueira. Prefeitura de Sao Paulo: Melissa Palmieri.; Patricia Akemi Assato; Paula Rahal; Paulo Inacio da Costa; Rafael dos Santos Bezerra; Raquel de Lello Rocha Campos Cassano. NGS Soluções Genômicas: Pilar Drummond Sampaio Corrêa Mariani. FZEA-USP Pirassununga: Mirele Daiana Poleti; Raul Machado Neto; Ricardo Augusto Brassaloti; Ricardo Haddad; Rodrigo Tocantins Calado. FAMERP-SJRP: Cecília Artico Banho; Sandra Coccuzzo Sampaio; Svetoslav Nanev Slavov; Vagner Fonseca; Vincent Louis Viala
EPI_ISL_2345014	UPA DR FRANCO DA ROCHA	Instituto Butantan	Antonio Jorge Martins; Claudia Renata dos Santos Barros; David Schlesinger; Debora Botequio Moretti; Dimas Tadeu Covas; Elaine Cristina Marqueze; Elaine Vieira Santos; Evandra Strazza Rodrigues; Heidge Fukumasu; Jayme Augusto de Souza-Neto; José Salvatore Leister Patané; Luiz Alcantara; Luiz Lehmann Coutinho; Maria Carolina Elias; Maurício Lacerda Nogueira; Rafael dos Santos Bezerra; Raul Machado Neto; Rejane Maria Tommasini Grotto; Ricardo Haddad; Sandra Coccuzzo Sampaio Vessoni; Simone Kashima; Svetoslav Nanev Slavov; Vincent Louis Viala
EPI_ISL_2493510, EPI_ISL_2493514, EPI_ISL_2494303	UPA II JUNDIAI	Instituto Butantan	Antonio Jorge Martins; Claudia Renata dos Santos Barros; David Schlesinger; Debora Botequio Moretti; Dimas Tadeu Covas; Elaine Cristina Marqueze; Elaine Vieira Santos; Evandra Strazza Rodrigues; Heidge Fukumasu; Jayme Augusto de Souza-Neto; José Salvatore Leister Patané; Luiz Alcantara; Luiz Lehmann Coutinho; Maria Carolina Elias; Maurício Lacerda Nogueira; Rafael dos Santos Bezerra; Raul Machado Neto; Rejane Maria Tommasini Grotto; Ricardo Haddad; Sandra Coccuzzo Sampaio Vessoni; Simone Kashima; Svetoslav Nanev Slavov; Vincent Louis Viala
EPI_ISL_2209746	UPA MARY DOTA	Instituto Butantan / Mendelics	Antonio Jorge Martins; Claudia Renata dos Santos Barros; David Schlesinger; Debora Botequio Moretti; Dimas Tadeu Covas; Elaine Cristina Marqueze; Elaine Vieira Santos; Evandra Strazza Rodrigues; Heidge Fukumasu; Jayme Augusto de Souza-Neto; José Salvatore Leister Patané; Luiz Alcantara; Luiz Lehmann Coutinho; Maria Carolina Elias; Maurício Lacerda Nogueira; Rafael dos Santos Bezerra; Raul Machado Neto; Rejane Maria Tommasini Grotto; Ricardo Haddad; Sandra Coccuzzo Sampaio Vessoni; Simone Kashima; Svetoslav Nanev Slavov; Vincent Louis Viala
EPI_ISL_2210275	UPA UNIDADE DE PRONTO ATENDIMENTO 24 HORAS BOM JESUS	Instituto Butantan / Mendelics	Antonio Jorge Martins; Claudia Renata dos Santos Barros; David Schlesinger; Debora Botequio Moretti; Dimas Tadeu Covas; Elaine Cristina Marqueze; Elaine Vieira Santos; Evandra Strazza Rodrigues; Heidge Fukumasu; Jayme Augusto de Souza-Neto; José Salvatore Leister Patané; Luiz Alcantara; Luiz Lehmann Coutinho; Maria Carolina Elias; Maurício Lacerda Nogueira; Rafael dos Santos Bezerra; Raul Machado Neto; Rejane Maria Tommasini Grotto; Ricardo Haddad; Sandra Coccuzzo Sampaio Vessoni; Simone Kashima; Svetoslav Nanev Slavov; Vincent Louis Viala
EPI_ISL_2344999, EPI_ISL_2345000, EPI_ISL_2345001, EPI_ISL_2445275, EPI_ISL_2445300, EPI_ISL_2445302	UPA VILA SANTA CATARINA	Instituto Butantan	Antonio Jorge Martins; Claudia Renata dos Santos Barros; David Schlesinger; Debora Botequio Moretti; Dimas Tadeu Covas; Elaine Cristina Marqueze; Elaine Vieira Santos; Evandra Strazza Rodrigues; Heidge Fukumasu; Jayme Augusto de Souza-Neto; José Salvatore Leister Patané; Luiz Alcantara; Luiz Lehmann Coutinho; Maria Carolina Elias; Maurício Lacerda Nogueira; Rafael dos Santos Bezerra; Raul Machado Neto; Rejane Maria Tommasini Grotto; Ricardo Haddad; Sandra Coccuzzo Sampaio Vessoni; Simone Kashima; Svetoslav Nanev Slavov; Vincent Louis Viala
EPI_ISL_1967298	UPA VILA SANTA CATARINA	Instituto Butantan / Mendelics	Antonio Jorge Martins; Bianca Cechetto Carlos. Mendelics: Bibiana Santos; Claudia Renata dos Santos Barros; Cíntia Bittar; David Schlesinger. Hemocentro Ribeirão Preto: Simone Kashima; Debora Botequio Moretti; Elaine Cristina Marqueze; Elaine Vieira dos Santos; Elisangela Chicaroni Mattos; Erika Freitas; Evandra Strazza Rodrigues; Felipe Allan da Silva da Costa; Flavia Aburjaile; Fábio Sossai Possebon; Guilherme Campos; Guilherme Targino Valente; Heidge Fukumasu. USP-Botucatu: Rejane Maria Tommasini Grotto; Helena Lage Ferreira; Instituto Butantan: Dimas Tadeu Covas; Jardelina de Souza Todao Bernardino; Jayme A. Souza-Neto; Jessika Cristina Chagas Lesbon; Jorge A. Petroli Marchesi; José Salvatore Leister Patané; João Paulo Kitajima; João Pessoa Araújo Jr.; Leila Sabrina Ullmann; Loyze Paola Oliveira de Lima; Luiz Aurelio de Campos Crispin. Centro de Genômica Funcional da ESALQ: Luiz Lehmann Coutinho; Luiz Carlos Junior de Alcantara; Lívia Sacchetto; Maisa C. Pereira Parra; Maria Carolina Elias; Marta Giovanetti; Marília Moraes; Maurício Lacerda Nogueira. Prefeitura de Sao Paulo: Melissa Palmieri.; Patricia Akemi Assato; Paula Rahal; Paulo Inacio da Costa; Rafael dos Santos Bezerra; Raquel de Lello Rocha Campos Cassano. NGS Soluções Genômicas: Pilar Drummond Sampaio Corrêa Mariani. FZEA-USP Pirassununga: Mirele Daiana Poleti; Raul Machado Neto; Ricardo Augusto Brassaloti; Ricardo Haddad; Rodrigo Tocantins Calado. FAMERP-SJRP: Cecília Artico Banho; Sandra Coccuzzo Sampaio; Svetoslav Nanev Slavov; Vagner Fonseca; Vincent Louis Viala
EPI_ISL_2426773, EPI_ISL_2426774, EPI_ISL_2426783, EPI_ISL_2450425, EPI_ISL_2661479, EPI_ISL_2661486	US Air Force School of Aerospace Medicine	US Air Force School of Aerospace Medicine	Amanda Javorina; Anthony Fries; Carol Garrett; Clarise Starr; Cole Anderson; Elizabeth Macias; Fritz Castillo; Jennifer Meyer; Sarah Purves; William Gruner
EPI_ISL_2493546	USAFA FORTE	Instituto Butantan	Antonio Jorge Martins; Claudia Renata dos Santos Barros; David Schlesinger; Debora Botequio Moretti; Dimas Tadeu Covas; Elaine Cristina Marqueze; Elaine Vieira Santos; Evandra Strazza Rodrigues; Heidge Fukumasu; Jayme Augusto de Souza-Neto; José Salvatore Leister Patané; Luiz Alcantara; Luiz Lehmann Coutinho; Maria Carolina Elias; Maurício Lacerda Nogueira; Rafael dos Santos Bezerra; Raul Machado Neto; Rejane Maria Tommasini Grotto; Ricardo Haddad; Sandra Coccuzzo Sampaio Vessoni; Simone Kashima; Svetoslav Nanev Slavov; Vincent Louis Viala
EPI_ISL_2493536, EPI_ISL_2494304	USAFA GUILHERMINA	Instituto Butantan	Antonio Jorge Martins; Claudia Renata dos Santos Barros; David Schlesinger; Debora Botequio Moretti; Dimas Tadeu Covas; Elaine Cristina Marqueze; Elaine Vieira Santos; Evandra Strazza Rodrigues; Heidge Fukumasu; Jayme Augusto de Souza-Neto; José Salvatore Leister Patané; Luiz Alcantara; Luiz Lehmann Coutinho; Maria Carolina Elias; Maurício Lacerda Nogueira; Rafael dos Santos Bezerra; Raul Machado Neto; Rejane Maria Tommasini Grotto; Ricardo Haddad; Sandra Coccuzzo Sampaio Vessoni; Simone Kashima; Svetoslav Nanev Slavov; Vincent Louis Viala
EPI_ISL_2493555	USAFA MELVI	Instituto Butantan	Antonio Jorge Martins; Claudia Renata dos Santos Barros; David Schlesinger; Debora Botequio Moretti; Dimas Tadeu Covas; Elaine Cristina Marqueze; Elaine Vieira Santos; Evandra Strazza Rodrigues; Heidge Fukumasu; Jayme Augusto de Souza-Neto; José Salvatore Leister Patané; Luiz Alcantara; Luiz Lehmann Coutinho; Maria Carolina Elias; Maurício Lacerda Nogueira; Rafael dos Santos Bezerra; Raul Machado Neto; Rejane Maria Tommasini Grotto; Ricardo Haddad; Sandra Coccuzzo Sampaio Vessoni; Simone Kashima; Svetoslav Nanev Slavov; Vincent Louis Viala
EPI_ISL_2493557	USAFA OCIAN	Instituto Butantan	Antonio Jorge Martins; Claudia Renata dos Santos Barros; David Schlesinger; Debora Botequio Moretti; Dimas Tadeu Covas; Elaine Cristina Marqueze; Elaine Vieira Santos; Evandra Strazza Rodrigues; Heidge Fukumasu; Jayme Augusto de Souza-Neto; José Salvatore Leister Patané; Luiz Alcantara; Luiz Lehmann Coutinho; Maria Carolina Elias; Maurício Lacerda Nogueira; Rafael dos Santos Bezerra; Raul Machado Neto; Rejane Maria Tommasini Grotto; Ricardo Haddad; Sandra Coccuzzo Sampaio Vessoni; Simone Kashima; Svetoslav Nanev Slavov; Vincent Louis Viala
EPI_ISL_2493559, EPI_ISL_2493561	USAFA PRINCESA	Instituto Butantan	Antonio Jorge Martins; Claudia Renata dos Santos Barros; David Schlesinger; Debora Botequio Moretti; Dimas Tadeu Covas; Elaine Cristina Marqueze; Elaine Vieira Santos; Evandra Strazza Rodrigues; Heidge Fukumasu; Jayme Augusto de Souza-Neto; José Salvatore Leister Patané; Luiz Alcantara; Luiz Lehmann Coutinho; Maria Carolina Elias; Maurício Lacerda Nogueira; Rafael dos Santos Bezerra; Raul Machado Neto; Rejane Maria Tommasini Grotto; Ricardo Haddad; Sandra Coccuzzo Sampaio Vessoni; Simone Kashima; Svetoslav Nanev Slavov; Vincent Louis Viala
EPI_ISL_2493560, EPI_ISL_2493563	USAFA SAO JORGE	Instituto Butantan	Antonio Jorge Martins; Claudia Renata dos Santos Barros; David Schlesinger; Debora Botequio Moretti; Dimas Tadeu Covas; Elaine Cristina Marqueze; Elaine Vieira Santos; Evandra Strazza Rodrigues; Heidge Fukumasu; Jayme Augusto de Souza-Neto; José Salvatore Leister Patané; Luiz Alcantara; Luiz Lehmann Coutinho; Maria Carolina Elias; Maurício Lacerda Nogueira; Rafael dos Santos Bezerra; Raul Machado Neto; Rejane Maria Tommasini Grotto; Ricardo Haddad; Sandra Coccuzzo Sampaio Vessoni; Simone Kashima; Svetoslav Nanev Slavov; Vincent Louis Viala
EPI_ISL_2493551	USAFA TUDE BASTOS	Instituto Butantan	Antonio Jorge Martins; Claudia Renata dos Santos Barros; David Schlesinger; Debora Botequio Moretti; Dimas Tadeu Covas; Elaine Cristina Marqueze; Elaine Vieira Santos; Evandra Strazza Rodrigues; Heidge Fukumasu; Jayme Augusto de Souza-Neto; José Salvatore Leister Patané; Luiz Alcantara; Luiz Lehmann Coutinho; Maria Carolina Elias; Maurício Lacerda Nogueira; Rafael dos Santos Bezerra; Raul Machado Neto; Rejane Maria Tommasini Grotto; Ricardo Haddad; Sandra Coccuzzo Sampaio Vessoni; Simone Kashima; Svetoslav Nanev Slavov; Vincent Louis Viala

EPI_ISL_2391784	USC Clinical Lab	Los Angeles County Public Health Laboratories	P. Hemarajata et al.
EPI_ISL_2345746	USF ARCADAS	Instituto Butantan / Mendelics	Antonio Jorge Martins; Claudia Renata dos Santos Barros; David Schlesinger; Debora Botequio Moretti; Dimas Tadeu Covas; Elaine Cristina Marqueze; Elaine Vieira Santos; Evandra Strazza Rodrigues; Heidge Fukumasu; Jayme Augusto de Souza-Neto; José Salvatore Leister Patané; Luiz Alcantara; Luiz Lehmann Coutinho; Maria Carolina Elias; Maurício Lacerda Nogueira; Rafael dos Santos Bezerra; Raul Machado Neto; Rejane Maria Tommasini Grotto; Ricardo Haddad; Sandra Coccuzzo Sampaio Vessoni; Simone Kashima; Svetoslav Nanev Slavov; Vincent Louis Viala
EPI_ISL_2445456	USF II DR JOSE RODOLFO ZIMMERMANN DIAS	Instituto Butantan	Antonio Jorge Martins; Claudia Renata dos Santos Barros; David Schlesinger; Debora Botequio Moretti; Dimas Tadeu Covas; Elaine Cristina Marqueze; Elaine Vieira Santos; Evandra Strazza Rodrigues; Heidge Fukumasu; Jayme Augusto de Souza-Neto; José Salvatore Leister Patané; Luiz Alcantara; Luiz Lehmann Coutinho; Maria Carolina Elias; Maurício Lacerda Nogueira; Rafael dos Santos Bezerra; Raul Machado Neto; Rejane Maria Tommasini Grotto; Ricardo Haddad; Sandra Coccuzzo Sampaio Vessoni; Simone Kashima; Svetoslav Nanev Slavov; Vincent Louis Viala
EPI_ISL_2473711	USF ITAJUBY	Instituto Butantan	Antonio Jorge Martins; Claudia Renata dos Santos Barros; David Schlesinger; Debora Botequio Moretti; Dimas Tadeu Covas; Elaine Cristina Marqueze; Elaine Vieira Santos; Evandra Strazza Rodrigues; Heidge Fukumasu; Jayme Augusto de Souza-Neto; José Salvatore Leister Patané; Luiz Alcantara; Luiz Lehmann Coutinho; Maria Carolina Elias; Maurício Lacerda Nogueira; Rafael dos Santos Bezerra; Raul Machado Neto; Rejane Maria Tommasini Grotto; Ricardo Haddad; Sandra Coccuzzo Sampaio Vessoni; Simone Kashima; Svetoslav Nanev Slavov; Vincent Louis Viala
EPI_ISL_2445203, EPI_ISL_2445204, EPI_ISL_2445205, EPI_ISL_2445526, EPI_ISL_2473716, EPI_ISL_2473717, EPI_ISL_2473817			
see above	USF PAULISTA FERNANDOPOLIS ANTONIO PIVATO	Instituto Butantan	Antonio Jorge Martins; Claudia Renata dos Santos Barros; David Schlesinger; Debora Botequio Moretti; Dimas Tadeu Covas; Elaine Cristina Marqueze; Elaine Vieira Santos; Evandra Strazza Rodrigues; Heidge Fukumasu; Jayme Augusto de Souza-Neto; José Salvatore Leister Patané; Luiz Alcantara; Luiz Lehmann Coutinho; Maria Carolina Elias; Maurício Lacerda Nogueira; Rafael dos Santos Bezerra; Raul Machado Neto; Rejane Maria Tommasini Grotto; Ricardo Haddad; Sandra Coccuzzo Sampaio Vessoni; Simone Kashima; Svetoslav Nanev Slavov; Vincent Louis Viala
EPI_ISL_2445392, EPI_ISL_2493463, EPI_ISL_2493469, EPI_ISL_2493475, EPI_ISL_2493479	USF SALERNO	Instituto Butantan	Antonio Jorge Martins; Claudia Renata dos Santos Barros; David Schlesinger; Debora Botequio Moretti; Dimas Tadeu Covas; Elaine Cristina Marqueze; Elaine Vieira Santos; Evandra Strazza Rodrigues; Heidge Fukumasu; Jayme Augusto de Souza-Neto; José Salvatore Leister Patané; Luiz Alcantara; Luiz Lehmann Coutinho; Maria Carolina Elias; Maurício Lacerda Nogueira; Rafael dos Santos Bezerra; Raul Machado Neto; Rejane Maria Tommasini Grotto; Ricardo Haddad; Sandra Coccuzzo Sampaio Vessoni; Simone Kashima; Svetoslav Nanev Slavov; Vincent Louis Viala
EPI_ISL_2383988	UT-Unified State Labs: Public Health Utah DOH	Centers for Disease Control and Prevention Division of Viral Diseases, Pathogen Discovery	Alison Laufer Halpin; Ben L. Rambo-Martin; Clinton R. Paden; Dakota Howard; Darlene Wagner; Dave Wentworth; Dhwani Batra; Jasmine Padilla; Justin Lee; Katie Dillon; Krista Queen; Kristen Knipe; Kristine Lacek; Mark Burroughs; Matthew Schmerer; Mili Sheth; Peter Cook; Sam Shepard; Sarah Nobles; Shoshona Le; Suxiang Tong; Vivien Dugan; Yvette Unoarumhi
EPI_ISL_2002420, EPI_ISL_2002816, EPI_ISL_2002881, EPI_ISL_2031481, EPI_ISL_2031482, EPI_ISL_2031483, EPI_ISL_2031487, EPI_ISL_2031494, EPI_ISL_2037449, EPI_ISL_2037584, EPI_ISL_2037587, EPI_ISL_2037652, EPI_ISL_2037654, EPI_ISL_2037656, EPI_ISL_2037664, EPI_ISL_2037666, EPI_ISL_2037667, EPI_ISL_2037682, EPI_ISL_2037684, EPI_ISL_2082514, EPI_ISL_2082569, EPI_ISL_2082570, EPI_ISL_2082572, EPI_ISL_2082573, EPI_ISL_2082641, EPI_ISL_2082671, EPI_ISL_2082675, EPI_ISL_2082677, EPI_ISL_2082681, EPI_ISL_2082699, EPI_ISL_2086397, EPI_ISL_2086422, EPI_ISL_2104837, EPI_ISL_2104840, EPI_ISL_2104842, EPI_ISL_2104885, EPI_ISL_2104889, EPI_ISL_2104956, EPI_ISL_2104977, EPI_ISL_2104979, EPI_ISL_2105034, EPI_ISL_2105055, EPI_ISL_2161842, EPI_ISL_2194050, EPI_ISL_2194051, EPI_ISL_2194174, EPI_ISL_2233293, EPI_ISL_2272617, EPI_ISL_2272656, EPI_ISL_2273779, EPI_ISL_2273807, EPI_ISL_2299454, EPI_ISL_2332880, EPI_ISL_2332923, EPI_ISL_2332940, EPI_ISL_2332988, EPI_ISL_2332989, EPI_ISL_2333008, EPI_ISL_2333034, EPI_ISL_2333061, EPI_ISL_2340712, EPI_ISL_2340743, EPI_ISL_2340768, EPI_ISL_2340804, EPI_ISL_2426281, EPI_ISL_2426347, EPI_ISL_2426424, EPI_ISL_2426430, EPI_ISL_2426433, EPI_ISL_2426436, EPI_ISL_2426507, EPI_ISL_2426515, EPI_ISL_2426518, EPI_ISL_2432639, EPI_ISL_2432675, EPI_ISL_2432766, EPI_ISL_2432776, EPI_ISL_2432783, EPI_ISL_2432785, EPI_ISL_2432844, EPI_ISL_2432849, EPI_ISL_2432859, EPI_ISL_2432877, EPI_ISL_2432902, EPI_ISL_2432911, EPI_ISL_2502847, EPI_ISL_2502913, EPI_ISL_2502924, EPI_ISL_2502944, EPI_ISL_2503028, EPI_ISL_2503066, EPI_ISL_2503133, EPI_ISL_2544938, EPI_ISL_2544942, EPI_ISL_2544958, EPI_ISL_2544968, EPI_ISL_2618311, EPI_ISL_2618363, EPI_ISL_2618412, EPI_ISL_2618442, EPI_ISL_2618585, EPI_ISL_2618637, EPI_ISL_2618660, EPI_ISL_2618665, EPI_ISL_2618669, EPI_ISL_2618702, EPI_ISL_2618728			
see above	UW Virology Lab	UW Virology Lab	Alexander Greninger; Hong Xie; Keith R Jerome; Lasata Shrestha; Meei-Li Huang; Michelle Lin; Nathan Breit; Noah R. Baker; Patrick Mathias; Pavitra Roychoudhury; Ricardo Perez; Robert J. Livingston; Sean Ellis; Shah Mohamed Bakhash; Tien V. Nguyen
EPI_ISL_2671499, EPI_ISL_2671670, EPI_ISL_2671672, EPI_ISL_2671678	Unidad de Investigacion Medica de Yucatan (UIMY)	Unidad de Genomica Avanzada	; Alejandra Garcia-Gasca; Alejandra Hernandez-Teran; Alejandro Sanchez-Flores; Alfredo Herrera-Estrella; Alicia Ocaña-Mondragon; Andreu Comas-Garcia; Angel Gustavo Salas-Lais; Antonio Loza Roman; Bernardo Martinez-Miguel; Blanca Taboada; Brenda Irasema Maldonado-Meza; Bruno Gomez-Gil; Carla Ivon Herrera-Najera; Carlos F. Arias; Celia Boukadida; Celida Duque Molina; Celida Martinez- Rodriguez; Clara Esperanza Santacruz-Tinoco; Concepcion Grajales-Muñiz; Consorcio Mexicano de Vigilancia Genomica (CoViGen-Mex). Authors (in alphabetical order): Julio Elias Alvarado-Yaah; Cristobal Chaidez-Quiroz; Daniel Fregoso-Rueda; Daniel Lira Morales; Eduardo Becerril-Vargas; Fernando Fontove-Herrera; Fidencio Mejia-Nepomuceno; Francisco Pulido; Gloria Elena Espinosa-Ayala; Gloria Maria Molina-Salinas; Gloria Vazquez; Hector Esteban Paz-Juarez; Hector Montoya-Fuentes; Helen Haydee Fernanda Ramirez-Plascencia; Irvin Gonzalez-Lopez; Jean Pierre Gonzalez; Jesus Hernandez; Joel Armando Vazquez-Perez.; Jorge Salas-Hernandez; Jose Antonio Enciso-Moreno; Jose Arturo Martinez-Orozco; Jose Esteban Muñoz-Medina; Jose de Jesus Nuñez-Contreras; Juan Bautista Chale-Dzul; Julissa Enciso-Ibarra; Luis Alberto Ochoa-Carrera; Margarita Matias-Florentino; Maria Guadalupe Santiago-Mauricio; Maria Guadalupe de Jesus Mireles-Rivera; Mario Mujica-Sanchez; Marissa Perez-Garcia; Nelly Selem-Mojica; Pavel Isa; Ricardo Ciria Merce; Ricardo Grande; Rosa Maria Gutierrez Rios; Santiago avila-Rios; Selene Zarate; Susana Lopez; Veronica Mata-Haro; Victor Eduardo Garcia-Arias; Victor Hugo Borja-Aburto
EPI_ISL_2490385, EPI_ISL_2490386	Unidad de Investigación Biomédica de Zacatecas (UIBZ)	Centro de Investigación en Enfermedades Infecciosas (CIENI), Instituto Nacional de Enfermedades Respiratorias (INER)	; Alejandra Garcia-Gasca; Alejandra Hernández-Terán; Alejandro Sanchez-Flores; Alfredo Herrera-Estrella; Alicia Ocaña-Mondragón; Andreu Comas-García; Angel Gustavo Salas-Lais; Antonio Loza Román; Bernardo Martínez-Miguel; Blanca Taboada; Brenda Irasema Maldonado-Meza; Bruno Gomez-Gil; Carla Ivón Herrera-Najera; Carlos F. Arias; Celia Boukadida; Clara Esperanza Santacruz-Tinoco; Concepción Grajales-Muñiz; Consorcio Mexicano de Vigilancia Genómica (CoViGen-Mex). Authors (in alphabetical order): Julio Elias Alvarado-Yaah; Cristóbal Cháidez-Quiróz; Célida Duque Molina; Célida Martínez-Rodríguez; Daniel Fregoso-Rueda; Daniel Lira Morales; Eduardo Becerril-Vargas; Fernando Fontove-Herrera; Fidencio Mejía-Nepomuceno; Francisco Pulido; Gloria Elena Espinosa-Ayala; Gloria María Molina-Salinas; Gloria Vazquez; Hector Esteban Paz-Juárez; Hector Montoya-Fuentes; Helen Haydee Fernanda Ramírez-Plascencia; Irvin González-López; Jean Pierre González; Joel Armando Vázquez-Pérez.; Jorge Salas-Hernández; José Antonio Enciso-Moreno; José Arturo Martínez-Orozco; José Esteban Muñoz-Medina; José de Jesús Nuñez-Contreras; Juan Bautista Chale-Dzul; Julissa Enciso-Ibarra; Luis Alberto Ochoa-Carrera; Margarita Matías-Florentino; Mario Mújica-Sánchez; Marissa Perez-Garcia; María Guadalupe Santiago-Mauricio; María Guadalupe de Jesús Mireles-Rivera; Nelly Sélem-Mojica; Pavel Isa; Ricardo Ciria Merce; Ricardo Grande; Rosa María Gutierrez Rios; Santiago Ávila-Ríos; Selene Zárate; Susana Lopez; Victor Eduardo Garcia-Arias; Víctor Hugo Borja-Aburto
EPI_ISL_2490376, EPI_ISL_2490381	Unidad de Investigación Médica de Yucatán (UIMY)	Centro de Investigación en Enfermedades Infecciosas (CIENI), Instituto Nacional de Enfermedades Respiratorias (INER)	; Alejandra Garcia-Gasca; Alejandra Hernández-Terán; Alejandro Sánchez-Flores; Alfredo Herrera-Estrella; Alicia Ocaña-Mondragón; Andreu Comas-García; Angel Gustavo Salas-Lais; Antonio Loza Román; Bernardo Martínez-Miguel; Blanca Taboada; Brenda Irasema Maldonado-Meza; Bruno Gomez-Gil; Carla Ivón Herrera-Najera; Carlos F. Arias; Celia Boukadida; Clara Esperanza Santacruz-Tinoco; Concepción Grajales-Muñiz; Consorcio Mexicano de Vigilancia Genómica (CoViGen-Mex). Authors (in alphabetical order): Julio Elias Alvarado-Yaah; Cristóbal Cháidez-Quiróz; Célida Duque Molina; Célida Martínez-Rodríguez; Daniel Fregoso-Rueda; Daniel Lira Morales; Eduardo Becerril-Vargas; Fernando Fontove-Herrera; Fidencio Mejía-Nepomuceno; Francisco Pulido; Gloria Elena Espinosa-Ayala; Gloria María Molina-Salinas; Gloria Vazquez; Hector Esteban Paz-Juárez; Hector Montoya-Fuentes; Helen Haydee Fernanda Ramírez-Plascencia; Irvin González-López; Jean Pierre González; Joel Armando Vázquez-Pérez.; Jorge Salas-Hernández; José Antonio Enciso-Moreno; José Arturo Martínez-Orozco; José Esteban Muñoz-Medina; José de Jesús Nuñez-Contreras; Juan Bautista Chale-Dzul; Julissa Enciso-Ibarra; Luis Alberto Ochoa-Carrera; Margarita Matías-Florentino; Mario Mújica-Sánchez; Marissa Perez-Garcia; María Guadalupe Santiago-Mauricio; María Guadalupe de Jesús Mireles-Rivera; Nelly Sélem-Mojica; Pavel Isa; Ricardo Ciria Merce; Ricardo Grande; Rosa María Gutierrez Rios; Santiago Ávila-Ríos; Selene Zárate; Susana Lopez; Victor Eduardo Garcia-Arias; Víctor Hugo Borja-Aburto
EPI_ISL_2535111, EPI_ISL_2535112, EPI_ISL_2535113, EPI_ISL_2535114, EPI_ISL_2535115, EPI_ISL_2535116, EPI_ISL_2535117, EPI_ISL_2535118, EPI_ISL_2535119, EPI_ISL_2535120, EPI_ISL_2535121, EPI_ISL_2535124, EPI_ISL_2535125, EPI_ISL_2535126, EPI_ISL_2535127, EPI_ISL_2535128, EPI_ISL_2535131, EPI_ISL_2535132, EPI_ISL_2535133, EPI_ISL_2535134, EPI_ISL_2535135, EPI_ISL_2535136, EPI_ISL_2535137, EPI_ISL_2535138, EPI_ISL_2535140, EPI_ISL_2535141, EPI_ISL_2535142, EPI_ISL_2535143, EPI_ISL_2535144, EPI_ISL_2535145, EPI_ISL_2535146, EPI_ISL_2535147, EPI_ISL_2535148, EPI_ISL_2535151, EPI_ISL_2535152, EPI_ISL_2535154, EPI_ISL_2535155, EPI_ISL_2535156, EPI_ISL_2535157, EPI_ISL_2535158, EPI_ISL_2535159, EPI_ISL_2535160, EPI_ISL_2535161, EPI_ISL_2535162, EPI_ISL_2535163, EPI_ISL_2535164, EPI_ISL_2535165, EPI_ISL_2535166, EPI_ISL_2535167, EPI_ISL_2535168, EPI_ISL_2535169, EPI_ISL_2535170, EPI_ISL_2535171, EPI_ISL_2535172, EPI_ISL_2535173, EPI_ISL_2535174, EPI_ISL_2535176, EPI_ISL_2535178, EPI_ISL_2535179, EPI_ISL_2535180, EPI_ISL_2535181, EPI_ISL_2535182, EPI_ISL_2535184, EPI_ISL_2535185, EPI_ISL_2535187, EPI_ISL_2535188, EPI_ISL_2535190, EPI_ISL_2535191, EPI_ISL_2535192, EPI_ISL_2535193, EPI_ISL_2535194, EPI_ISL_2535195, EPI_ISL_2535196, EPI_ISL_2535197, EPI_ISL_2535198, EPI_ISL_2535199, EPI_ISL_2535200, EPI_ISL_2535201, EPI_ISL_2535202, EPI_ISL_2535203, EPI_ISL_2535204, EPI_ISL_2535205, EPI_ISL_2535206, EPI_ISL_2535207, EPI_ISL_2535208, EPI_ISL_2535209, EPI_ISL_2535210, EPI_ISL_2535211, EPI_ISL_2535212, EPI_ISL_2535213, EPI_ISL_2535216, EPI_ISL_2535217, EPI_ISL_2535218, EPI_ISL_2535221, EPI_ISL_2535222, EPI_ISL_2535224, EPI_ISL_2535225, EPI_ISL_2535226, EPI_ISL_2535227, EPI_ISL_2535228, EPI_ISL_2535229, EPI_ISL_2535230, EPI_ISL_2535231, EPI_ISL_2535232, EPI_ISL_2535233, EPI_ISL_2535234, EPI_ISL_2535235, EPI_ISL_2691623, EPI_ISL_2691624, EPI_ISL_2691626, EPI_ISL_2691641, EPI_ISL_2691645, EPI_ISL_2691646			
see above	Unidade de apoio ao diagnostico da COVID - UNADIG	Bioinformatics Laboratory / LNCC	Alessandra P Lamarca; Alexandra L Gerber; Amilcar Tanuri; Ana Paula de C Guimaraes; Ana Tereza R Vasconcelos; Andrea Cony Cavalcanti; Caio Luiz Pereira Ribeiro; Cassia Alves; Cintia Policarpo; Claudia Maria Braga de Mello; Cristiane Gomes da Silva; Diana Mariani; Douglas Terra Machado; Flavio Dias da Silva; Gleidson da Silva de Oliveira; Leandro Magalhaes de Souza; Liliane Cavalcante; Luiz G P de Almeida; Marcio Henrique de Oliveira Garcia; Mario Sergio Ribeiro; Ronaldo da Silva F Jr; Silvia Carvalho
EPI_ISL_2101508, EPI_ISL_2101509, EPI_ISL_2101511, EPI_ISL_2101513, EPI_ISL_2101671, EPI_ISL_2101714, EPI_ISL_2385669, EPI_ISL_2385670, EPI_ISL_2385671, EPI_ISL_2385674, EPI_ISL_2385675, EPI_ISL_2385676, EPI_ISL_2385677, EPI_ISL_2385678, EPI_ISL_2385679, EPI_ISL_2385680, EPI_ISL_2385681, EPI_ISL_2385682, EPI_ISL_2385683, EPI_ISL_2385684, EPI_ISL_2385685, EPI_ISL_2385686, EPI_ISL_2385689, EPI_ISL_2385690, EPI_ISL_2385691, EPI_ISL_2385692, EPI_ISL_2385693, EPI_ISL_2385694, EPI_ISL_2385695, EPI_ISL_2385697, EPI_ISL_2385698, EPI_ISL_2385699, EPI_ISL_2385700, EPI_ISL_2385701, EPI_ISL_2385702, EPI_ISL_2385704, EPI_ISL_2385705, EPI_ISL_2385707, EPI_ISL_2385708, EPI_ISL_2385709, EPI_ISL_2385711, EPI_ISL_2385712, EPI_ISL_2385713, EPI_ISL_2385714, EPI_ISL_2385717, EPI_ISL_2385718, EPI_ISL_2385719, EPI_ISL_2385720, EPI_ISL_2385723, EPI_ISL_2385724, EPI_ISL_2385725, EPI_ISL_2385726, EPI_ISL_2385727, EPI_ISL_2385728, EPI_ISL_2385729, EPI_ISL_2385730, EPI_ISL_2385731, EPI_ISL_2385733, EPI_ISL_2385734, EPI_ISL_2385735, EPI_ISL_2385736, EPI_ISL_2385737, EPI_ISL_2385739, EPI_ISL_2385740, EPI_ISL_2385741, EPI_ISL_2385742, EPI_ISL_2385743, EPI_ISL_2385745, EPI_ISL_2385748, EPI_ISL_2385749, EPI_ISL_2385750, EPI_ISL_2385751, EPI_ISL_2385752, EPI_ISL_2385753, EPI_ISL_2385754, EPI_ISL_2385755, EPI_ISL_2385757, EPI_ISL_2385758, EPI_ISL_2385759, EPI_ISL_2385760, EPI_ISL_2385761, EPI_ISL_2385764, EPI_ISL_2385765, EPI_ISL_2385766, EPI_ISL_2385768, EPI_ISL_2385769, EPI_ISL_2385770, EPI_ISL_2385771, EPI_ISL_2385772, EPI_ISL_2385773, EPI_ISL_2385774, EPI_ISL_2385776, EPI_ISL_2385777, EPI_ISL_2385778, EPI_ISL_2385779, EPI_ISL_2385780, EPI_ISL_2385781, EPI_ISL_2385782, EPI_ISL_2385784, EPI_ISL_2385785, EPI_ISL_2385786			
see above	Unidade de apoio ao diagnóstico da COVID – UNADIG	Bioinformatics Laboratory / LNCC	Alessandra P Lamarca; Alexandra L Gerber; Amilcar Tanuri; Ana Paula de C Guimaraes; Ana Tereza R Vasconcelos; Andrea Cony Cavalcanti; Caio Luiz Pereira Ribeiro; Cassia Alves; Cintia Policarpo; Claudia Maria Braga de Mello; Cristiane Gomes da Silva; Diana Mariani; Douglas Terra Machado; Flavio Dias da Silva; Gleidson da Silva de Oliveira; Leandro Magalhaes de Souza; Liliane Cavalcante; Luiz G P de Almeida; Marcio Henrique de Oliveira Garcia; Mario Sergio Ribeiro; Ronaldo da Silva F Jr; Silvia Carvalho
EPI_ISL_2204905	United Hospital Center	WVU and Marshall University Combined Genomics Core Facilities	James Denvir; Peter Perrotta; Peter Stoilov; Ryan Percifield; Wesley Kimble
EPI_ISL_3216907, EPI_ISL_3216908, EPI_ISL_3477562, EPI_ISL_3477566	Universidad del Valle, LDAB-Laboratorio de Diagnostico de Agentes Biologicos	Universidad del Valle, TAO-Lab, VIREM, NEAS Network	Andres Castillo; Beatriz Parra & COVID-19 Team Univalle; Diana López-Alvarez; Nelson Rivera Franco
EPI_ISL_2091058, EPI_ISL_2091059, EPI_ISL_2091061, EPI_ISL_2091062, EPI_ISL_2091063, EPI_ISL_2091064, EPI_ISL_2091078, EPI_ISL_2091079, EPI_ISL_2091080, EPI_ISL_2091108, EPI_ISL_2091109, EPI_ISL_2091111, EPI_ISL_2091112, EPI_ISL_2091113, EPI_ISL_2091114, EPI_ISL_2193388, EPI_ISL_2193389, EPI_ISL_2193390, EPI_ISL_2193391, EPI_ISL_2193393, EPI_ISL_2193401, EPI_ISL_2193402, EPI_ISL_2193403, EPI_ISL_2193404, EPI_ISL_2193405, EPI_ISL_2193406, EPI_ISL_2193407, EPI_ISL_2193411, EPI_ISL_2193414, EPI_ISL_2193417, EPI_ISL_2193435, EPI_ISL_2193437, EPI_ISL_2193438, EPI_ISL_2193439			
see above	University Hospitals Translational Laboratory (UHTL), University Hospitals	University Hospitals Translational Laboratory (UHTL), University Hospitals	Alouani, D.; Sadri, N.; Song, X.
EPI_ISL_1963270, EPI_ISL_1963272	University Hospitals of Geneva, Laboratory of Virology	HUG, Laboratory of Virology and the Health2030 Genome Center	Ana Rita Goncalves; Deborah Penet; Emmanouil Dermitzakis; Henri Pegeot; Ioannis Xenarios; Keith Harshman; Laurent Kaiser; Lorenzo Cerutti; Melyssa Elies; Samuel Cordey
EPI_ISL_2931009	University of Arkansas for Medical Sciences	Center for Global Health, University of New Mexico Health Sciences Center	Catherine Kirkpatrick; Darrell Dinwiddie; Daryl Domman; David Ussery; Jennifer Laudadio; Joshua L. Kennedy; Kurt Schwalm; Susan Harley; Uday Chalwadi; Valerie Morley
EPI_ISL_2408064	University of Bari Biomedical Sciences and Human Oncology	University of Bari Biomedical Sciences and Human Oncology	Accogli M.; Chironna M.; Loconsole D.; Sallustio A.
EPI_ISL_2476350, EPI_ISL_2476359, EPI_ISL_2476365, EPI_ISL_2476370, EPI_ISL_2691960, EPI_ISL_2691964, EPI_ISL_2691967, EPI_ISL_2691988, EPI_ISL_2691991, EPI_ISL_2692011, EPI_ISL_2931108, EPI_ISL_3388709			
see above	University of Chicago Medicine	RIPHL at Rush University Medical Center	Edith Perez; Felix Araujo Perez; Kevin Kunstman; Laura Furtado; Lauren Megger; Marieta Hyde; Max Kolton; Stefan Green
EPI_ISL_2476390, EPI_ISL_2476391, EPI_ISL_2476393, EPI_ISL_2476400, EPI_ISL_2692086, EPI_ISL_2692096, EPI_ISL_2692100, EPI_ISL_2692101, EPI_ISL_2692103, EPI_ISL_2692104, EPI_ISL_2692107, EPI_ISL_2692108, EPI_ISL_2692113, EPI_ISL_2692114, EPI_ISL_2692122, EPI_ISL_2692125, EPI_ISL_2692129, EPI_ISL_2692131, EPI_ISL_2692132			
see above	University of Illinois at Chicago	RIPHL at Rush University Medical Center	Felix Araujo Perez; Julie Bentea; Kevin Kunstman; Laura Furtado; Marieta Hyde; Max Kolton; Omar Perez; Stefan Green
EPI_ISL_2001220, EPI_ISL_2001240, EPI_ISL_2001258, EPI_ISL_2100396, EPI_ISL_2136244, EPI_ISL_2376042, EPI_ISL_2400358, EPI_ISL_2400362, EPI_ISL_2400366, EPI_ISL_2400372			
see above	University of Michigan Clinical Microbiology Laboratory	Lauring Lab, University of Michigan, Department of Microbiology and Immunology	Gilbert; Valesano
EPI_ISL_2404840, EPI_ISL_2404846	University of Oregon COVID-19 MAP Laboratory	University of Oregon Genomics and Cell Characericzation Core Facility (GC3F)	Ariana White; Douglas Turnbull; Jason Carriere; Jason Sydes; Jeff Bishop; Peter Batzel
EPI_ISL_1850441	UniversitÃ¤tsklinikum MÃ¼nster Institut fÃ¼r Virologie	Robert Koch Institute	
EPI_ISL_2086795, EPI_ISL_2086872, EPI_ISL_2136993, EPI_ISL_2449934, EPI_ISL_2510857, EPI_ISL_2510860, EPI_ISL_2510880, EPI_ISL_2510900, EPI_ISL_2510924, EPI_ISL_2510943, EPI_ISL_2510979, EPI_ISL_2510999, EPI_ISL_2511002, EPI_ISL_2511008			
see above	UniversitŠtsSpital ZŸrich	Institute of Medical Virology	Alexandra Trkola; Annette AudigŽ; Catharine Aquino; Cyril Shah; Daniel Ehrsam; Gabriela Ziltener; Guido Bloemberg; Hubert Rehrauer; Isabel StŸrmer; Joel Wirz; Jon Huder; JŸrg Bšni; Kevin Steiner; Maria GrŸnberg; Maryam Zaheri; Michael Huber; Riccarda Capaul; Stefan Schmutz; Verena Kufner; Weihong Qi
EPI_ISL_2152435,	Usansolo-Galdakao University Hospital	Cruces University Hospital	Ana Belén de la Hoz; Ana Gual-de-Torrella; Izaskun Alejo-Cancho; Mikel Gallego

EPI_ISL_2408062, EPI_ISL_2408063, EPI_ISL_2483459			
EPI_ISL_2284852, EPI_ISL_2292200, EPI_ISL_2292801, EPI_ISL_2456321, EPI_ISL_2456350, EPI_ISL_2456508, EPI_ISL_2456649, EPI_ISL_2456657, EPI_ISL_2458490, EPI_ISL_2679471, EPI_ISL_2679601, EPI_ISL_2679632, EPI_ISL_2855894, EPI_ISL_2856068, EPI_ISL_2856073, EPI_ISL_2856080, EPI_ISL_2856095, EPI_ISL_2856452, EPI_ISL_2856473, EPI_ISL_2856496, EPI_ISL_2857455, EPI_ISL_2857920, EPI_ISL_2857948, EPI_ISL_2857955, EPI_ISL_2857979, EPI_ISL_2857990, EPI_ISL_2858009, EPI_ISL_2858015, EPI_ISL_2858028, EPI_ISL_3269714			
see above	Utah Public Health Laboratory	Utah Public Health Laboratory	Erin L. Young; Kelly F. Oakeson; Olinto Linares-Perdomo; Tara Gallagher
EPI_ISL_2210083, EPI_ISL_2344951, EPI_ISL_2344958, EPI_ISL_2344959, EPI_ISL_2344962, EPI_ISL_2344964, EPI_ISL_2344968, EPI_ISL_2344970, EPI_ISL_2345039, EPI_ISL_2345040			
see above	VIGILANCIA EM SAUDE DE FCO MORATO	Instituto Butantan	Antonio Jorge Martins; Claudia Renata dos Santos Barros; David Schlesinger; Debora Botequio Moretti; Dimas Tadeu Covas; Elaine Cristina Marqueze; Elaine Vieira Santos; Evandra Strazza Rodrigues; Heidge Fukumasu; Jayme Augusto de Souza-Neto; José Salvatore Leister Patané; Luiz Alcantara; Luiz Lehmann Coutinho; Maria Carolina Elias; Maurício Lacerda Nogueira; Rafael dos Santos Bezerra; Raul Machado Neto; Rejane Maria Tommasini Grotto; Ricardo Haddad; Sandra Coccuzzo Sampaio Vessoni; Simone Kashima; Svetoslav Nanev Slavov; Vincent Louis Viala; Vincent Louis Viala.
EPI_ISL_2170994	VIGILANCIA EM SAUDE DE FCO MORATO	Instituto Butantan / Mendelics	Antonio Jorge Martins; Bianca Cechetto Carlos. Mendelics: Bibiana Santos; Claudia Renata dos Santos Barros; Cíntia Bittar; David Schlesinger. Hemocentro Ribeirão Preto: Simone Kashima; Debora Botequio Moretti; Elaine Cristina Marqueze; Elaine Vieira dos Santos; Elisangela Chicaroni Mattos; Erika Freitas; Evandra Strazza Rodrigues; Felipe Allan da Silva da Costa; Flavia Aburjaile; Fábio Sossai Possebon; Guilherme Campos; Guilherme Targino Valente; Heidge Fukumasu. USP-Botucatu: Rejane Maria Tommasini Grotto; Helena Lage Ferreira; Instituto Butantan: Dimas Tadeu Covas; Jardelina de Souza Todao Bernardino; Jayme A. Souza-Neto; Jessika Cristina Chagas Lesbon; Jorge A. Petroli Marchesi; José Salvatore Leister Patané; João Paulo Kitajima; João Pessoa Araújo Jr.; Leila Sabrina Ullmann; Loyze Paola Oliveira de Lima; Luiz Aurelio de Campos Crispin. Centro de Genômica Funcional da ESALQ: Luiz Lehmann Coutinho; Luiz Carlos Junior de Alcantara; Lívia Sacchetto; Maisa C. Pereira Parra; Maria Carolina Elias; Marta Giovanetti; Marília Moraes; Maurício Lacerda Nogueira. Prefeitura de Sao Paulo: Melissa Palmieri.; Patricia Akemi Assato; Paula Rahal; Paulo Inacio da Costa; Rafael dos Santos Bezerra; Raquel de Lello Rocha Campos Cassano. NGS Soluções Genômicas: Pilar Drummond Sampaio Corrêa Mariani. FZEA-USP Pirassununga: Mirele Daiana Poleti; Raul Machado Neto; Ricardo Augusto Brassaloti; Ricardo Haddad; Rodrigo Tocantins Calado. FAMERP-SJRP: Cecília Artico Banho; Sandra Coccuzzo Sampaio; Svetoslav Nanev Slavov; Vagner Fonseca; Vincent Louis Viala
EPI_ISL_2209304, EPI_ISL_2209309, EPI_ISL_2345651, EPI_ISL_2362250	VIGILANCIA EM SAUDE SAE VIGILANCIA EPIDEMIOLOGICA	Instituto Butantan / Mendelics	Antonio Jorge Martins; Claudia Renata dos Santos Barros; David Schlesinger; Debora Botequio Moretti; Dimas Tadeu Covas; Elaine Cristina Marqueze; Elaine Vieira Santos; Evandra Strazza Rodrigues; Heidge Fukumasu; Jayme Augusto de Souza-Neto; José Salvatore Leister Patané; Luiz Alcantara; Luiz Lehmann Coutinho; Maria Carolina Elias; Maurício Lacerda Nogueira; Rafael dos Santos Bezerra; Raul Machado Neto; Rejane Maria Tommasini Grotto; Ricardo Haddad; Sandra Coccuzzo Sampaio Vessoni; Simone Kashima; Svetoslav Nanev Slavov; Vincent Louis Viala
EPI_ISL_2493366, EPI_ISL_2493395, EPI_ISL_2493399, EPI_ISL_2493450, EPI_ISL_2494335	VIGILANCIA EPIDEMIOLOGICA DE LEME	Instituto Butantan	Antonio Jorge Martins; Claudia Renata dos Santos Barros; David Schlesinger; Debora Botequio Moretti; Dimas Tadeu Covas; Elaine Cristina Marqueze; Elaine Vieira Santos; Evandra Strazza Rodrigues; Heidge Fukumasu; Jayme Augusto de Souza-Neto; José Salvatore Leister Patané; Luiz Alcantara; Luiz Lehmann Coutinho; Maria Carolina Elias; Maurício Lacerda Nogueira; Rafael dos Santos Bezerra; Raul Machado Neto; Rejane Maria Tommasini Grotto; Ricardo Haddad; Sandra Coccuzzo Sampaio Vessoni; Simone Kashima; Svetoslav Nanev Slavov; Vincent Louis Viala
EPI_ISL_2493365, EPI_ISL_2493391, EPI_ISL_2493432, EPI_ISL_2493434, EPI_ISL_2493448, EPI_ISL_2500982	VIGILANCIA EPIDEMIOLOGICA E CONTROLE DE VETORES PIRASSUNUN	Instituto Butantan	Antonio Jorge Martins; Claudia Renata dos Santos Barros; David Schlesinger; Debora Botequio Moretti; Dimas Tadeu Covas; Elaine Cristina Marqueze; Elaine Vieira Santos; Evandra Strazza Rodrigues; Heidge Fukumasu; Jayme Augusto de Souza-Neto; José Salvatore Leister Patané; Luiz Alcantara; Luiz Lehmann Coutinho; Maria Carolina Elias; Maurício Lacerda Nogueira; Rafael dos Santos Bezerra; Raul Machado Neto; Rejane Maria Tommasini Grotto; Ricardo Haddad; Sandra Coccuzzo Sampaio Vessoni; Simone Kashima; Svetoslav Nanev Slavov; Vincent Louis Viala
EPI_ISL_2344830	VIGILANCIA EPIDEMIOLOGICA E CONTROLE DE VETORES PIRASSUNUN	Instituto Butantan / ESALQ-Piracicaba	Antonio Jorge Martins; Claudia Renata dos Santos Barros; David Schlesinger; Debora Botequio Moretti; Dimas Tadeu Covas; Elaine Cristina Marqueze; Elaine Vieira Santos; Evandra Strazza Rodrigues; Heidge Fukumasu; Jayme Augusto de Souza-Neto; José Salvatore Leister Patané; Luiz Alcantara; Luiz Lehmann Coutinho; Maria Carolina Elias; Maurício Lacerda Nogueira; Rafael dos Santos Bezerra; Raul Machado Neto; Rejane Maria Tommasini Grotto; Ricardo Haddad; Sandra Coccuzzo Sampaio Vessoni; Simone Kashima; Svetoslav Nanev Slavov; Vincent Louis Viala
EPI_ISL_2493599, EPI_ISL_2493610	VIGILANCIA EPIDEMIOLOGICA JARDINOPOLIS SP	Instituto Butantan	Antonio Jorge Martins; Claudia Renata dos Santos Barros; David Schlesinger; Debora Botequio Moretti; Dimas Tadeu Covas; Elaine Cristina Marqueze; Elaine Vieira Santos; Evandra Strazza Rodrigues; Heidge Fukumasu; Jayme Augusto de Souza-Neto; José Salvatore Leister Patané; Luiz Alcantara; Luiz Lehmann Coutinho; Maria Carolina Elias; Maurício Lacerda Nogueira; Rafael dos Santos Bezerra; Raul Machado Neto; Rejane Maria Tommasini Grotto; Ricardo Haddad; Sandra Coccuzzo Sampaio Vessoni; Simone Kashima; Svetoslav Nanev Slavov; Vincent Louis Viala
EPI_ISL_2209094, EPI_ISL_2345645, EPI_ISL_2345744, EPI_ISL_2345782, EPI_ISL_2345787, EPI_ISL_2345869	VIGILANCIA EPIDEMIOLOGICA JARDINOPOLIS SP	Instituto Butantan / Mendelics	Antonio Jorge Martins; Claudia Renata dos Santos Barros; David Schlesinger; Debora Botequio Moretti; Dimas Tadeu Covas; Elaine Cristina Marqueze; Elaine Vieira Santos; Evandra Strazza Rodrigues; Heidge Fukumasu; Jayme Augusto de Souza-Neto; José Salvatore Leister Patané; Luiz Alcantara; Luiz Lehmann Coutinho; Maria Carolina Elias; Maurício Lacerda Nogueira; Rafael dos Santos Bezerra; Raul Machado Neto; Rejane Maria Tommasini Grotto; Ricardo Haddad; Sandra Coccuzzo Sampaio Vessoni; Simone Kashima; Svetoslav Nanev Slavov; Vincent Louis Viala
EPI_ISL_2445607	VIGILANCIA EPIDIMIOLOGICA DE ARACARIGUAMA	Instituto Butantan	Antonio Jorge Martins; Claudia Renata dos Santos Barros; David Schlesinger; Debora Botequio Moretti; Dimas Tadeu Covas; Elaine Cristina Marqueze; Elaine Vieira Santos; Evandra Strazza Rodrigues; Heidge Fukumasu; Jayme Augusto de Souza-Neto; José Salvatore Leister Patané; Luiz Alcantara; Luiz Lehmann Coutinho; Maria Carolina Elias; Maurício Lacerda Nogueira; Rafael dos Santos Bezerra; Raul Machado Neto; Rejane Maria Tommasini Grotto; Ricardo Haddad; Sandra Coccuzzo Sampaio Vessoni; Simone Kashima; Svetoslav Nanev Slavov; Vincent Louis Viala
EPI_ISL_2345668	VIGILANCIA EPIDIMIOLOGICA DE ARACARIGUAMA	Instituto Butantan / Mendelics	Antonio Jorge Martins; Claudia Renata dos Santos Barros; David Schlesinger; Debora Botequio Moretti; Dimas Tadeu Covas; Elaine Cristina Marqueze; Elaine Vieira Santos; Evandra Strazza Rodrigues; Heidge Fukumasu; Jayme Augusto de Souza-Neto; José Salvatore Leister Patané; Luiz Alcantara; Luiz Lehmann Coutinho; Maria Carolina Elias; Maurício Lacerda Nogueira; Rafael dos Santos Bezerra; Raul Machado Neto; Rejane Maria Tommasini Grotto; Ricardo Haddad; Sandra Coccuzzo Sampaio Vessoni; Simone Kashima; Svetoslav Nanev Slavov; Vincent Louis Viala
EPI_ISL_2445282, EPI_ISL_2445285, EPI_ISL_2445286, EPI_ISL_2445290, EPI_ISL_2445291, EPI_ISL_2445293, EPI_ISL_2445294, EPI_ISL_2445296, EPI_ISL_2445303, EPI_ISL_2473822			
see above	VIGILANCIA SANITARIA E VIG EPIDEMIOLOGICA DE ITAPEVI	Instituto Butantan	Antonio Jorge Martins; Claudia Renata dos Santos Barros; David Schlesinger; Debora Botequio Moretti; Dimas Tadeu Covas; Elaine Cristina Marqueze; Elaine Vieira Santos; Evandra Strazza Rodrigues; Heidge Fukumasu; Jayme Augusto de Souza-Neto; José Salvatore Leister Patané; Luiz Alcantara; Luiz Lehmann Coutinho; Maria Carolina Elias; Maurício Lacerda Nogueira; Rafael dos Santos Bezerra; Raul Machado Neto; Rejane Maria Tommasini Grotto; Ricardo Haddad; Sandra Coccuzzo Sampaio Vessoni; Simone Kashima; Svetoslav Nanev Slavov; Vincent Louis Viala
EPI_ISL_2109687	VIROLOGY LABORATORY-CHU NICE	VIROLOGY LABORATORY-CHU NICE	Aicha El Yakine; Geraldine Gonfrier; Jean Machowiak; Sebastien Vitale; Valerie Giordanengo; Virginie Flahou
EPI_ISL_2444192	VOTUPORANGA	Instituto Butantan / FAMERP	Antonio Jorge Martins; Claudia Renata dos Santos Barros; David Schlesinger; Debora Botequio Moretti; Dimas Tadeu Covas; Elaine Cristina Marqueze; Elaine Vieira Santos; Evandra Strazza Rodrigues; Heidge Fukumasu; Jayme Augusto de Souza-Neto; José Salvatore Leister Patané; Luiz Alcantara; Luiz Lehmann Coutinho; Maria Carolina Elias; Maurício Lacerda Nogueira; Rafael dos Santos Bezerra; Raul Machado Neto; Rejane Maria Tommasini Grotto; Ricardo Haddad; Sandra Coccuzzo Sampaio Vessoni; Simone Kashima; Svetoslav Nanev Slavov; Vincent Louis Viala
EPI_ISL_1914207	Viollier AG	Department of Biosystems Science and Engineering, ETH Zurich	Chaoran Chen; Christian Beisel; Christiane Beckmann; Christoph Noppen; David Dreifuss; Elodie Burcklen; Ina Nissen; Ivan Topolsky; Katharina Jahn; Lara Fuhrmann; Maurice Redondo; Mirjam Feldkamp; Natascha Santacroce; Niko Beerenwinkel; Noemie Santamaria de Souza; Olivier Kobel; Philipp Jablonski; Rebecca Denes; Sarah Nadeau; Sophie Seidel; Tanja Stadler
EPI_ISL_2151992, EPI_ISL_2212753, EPI_ISL_2307457, EPI_ISL_2307461, EPI_ISL_2307612, EPI_ISL_2307706, EPI_ISL_2307750, EPI_ISL_2307771, EPI_ISL_2307844, EPI_ISL_2307864, EPI_ISL_2308026, EPI_ISL_2308074			
see above	Viollier AG	Department of Biosystems Science and Engineering, ETH Zürich	Andrea Patrignani; Andreia Cabral de Gouvea; Catharine Aquino; Chaoran Chen; Christian Beisel; Christiane Beckmann; Christoph Noppen; Daniel Ehrsam; David Dreifuss; Doris Popovic; Elodie Burcklen; Griffin White; Ina Nissen; Isabel Stürmer; Ivan Topolsky; Jay Tracy; Katharina Jahn; Lara Fuhrmann; Laura Neff; Lennart Opitz; Maria Domenica Moccia; Maurice Redondo; Mirjam Feldkamp; Natascha Santacroce; Niko Beerenwinkel; Noemie Santamaria de Souza; Olivier Kobel; Philipp Jablonski; Ralph Schlapbach; Rebecca Denes; Sarah Nadeau; Simon Grüter; Sophie Seidel; Tanja Stadler; Timothy Sykes
EPI_ISL_2226439	Viollier AG	Viollier AG	Andrea Patrizia Salzmann; Christiane Beckmann; Christoph Noppen; Henriette Kurth; Maurice Redondo; Olivier Kobel
EPI_ISL_2003686, EPI_ISL_2101937, EPI_ISL_2101940, EPI_ISL_2344478, EPI_ISL_2344479, EPI_ISL_2344485, EPI_ISL_2344490, EPI_ISL_2344505, EPI_ISL_2603207, EPI_ISL_2603208, EPI_ISL_2603217			
see above	Virginia Division of Consolidated Laboratory Services	Virginia Division of Consolidated Laboratory Services	Virginia DCLS
EPI_ISL_2105163, EPI_ISL_2451955	WA State Department of Health	Centers for Disease Control and Prevention Division of Viral Diseases, Pathogen Discovery	Alison Laufer Halpin; Ben L. Rambo-Martin; Clinton R. Paden; Dakota Howard; Darlene Wagner; Dave Wentworth; Dhwani Batra; Jasmine Padilla; Justin Lee; Katie Dillon; Krista Queen; Kristen Knipe; Kristine Lacek; Mark Burroughs; Matthew Schmerer; Mili Sheth; Peter Cook; Sam Shepard; Sarah Nobles; Shoshona Le; Suxiang Tong; Vivien Dugan; Yvette Unoarumhi
EPI_ISL_2097314, EPI_ISL_2274650, EPI_ISL_2627011	WESTCHESTER MEDICAL CENTER	Wadsworth Center, New York State Department of Health	Alexis Russell; Catharine Prussing; Daryl M. Lamson; Erasmus Schneider; Erica Lasek-Nesselquist; John Kelly; Jonathan Plitnick; Kirsten St. George; Matthew Shudt; Melissa A Leisner; Navjot Singh
EPI_ISL_2385106	WHO National Influenza Centre Russian Federation	WHO National Influenza Centre Russian Federation	Andrey Komissarov; Artem Fadeev; Daria Danilenko; Dmitry Lioznov; Elena Nabieva; Georgii Bazykin; Kirill Varchenko; Ksenia Safina; Kseniya Komissarova; Maria Pisareva; Maria Timofeeva; Mikhail Bakaev; Nikita Yolshin; Oula Masour; Tamila Musaeva; Veronika Eder
EPI_ISL_2248729, EPI_ISL_2452015	WI State Laboratory of Hygiene	Centers for Disease Control and Prevention Division of Viral Diseases, Pathogen Discovery	Alison Laufer Halpin; Ben L. Rambo-Martin; Clinton R. Paden; Dakota Howard; Darlene Wagner; Dave Wentworth; Dhwani Batra; Jasmine Padilla; Justin Lee; Katie Dillon; Krista Queen; Kristen Knipe; Kristine Lacek; Mark Burroughs; Matthew Schmerer; Mili Sheth; Peter Cook; Sam Shepard; Sarah Nobles; Shoshona Le; Suxiang Tong; Vivien Dugan; Yvette Unoarumhi
EPI_ISL_2533758	WLA VAMC	Genomics and Discovery, Respiratory Viruses Branch, Division of Viral Diseases, Centers for Disease Control and Prevention	Adam Retchless; Anna Kelleher; Anna Uehara; Brian Lynch; Clinton R. Paden; Dhwani Batra; Haibin Wang; Han Jia Justin Ng; Jasmine Padilla; Jing Zhang; Justin Lee; Krista Queen; Mark Burroughs; Mili Sheth; Morgan Davis; Peter Cook; Rachel Marine; Sarah Nobles; Suxiang Tong; Tara Coalter; Yan Li; Ying Tao
EPI_ISL_2452097	WY Public Health Laboratory	Centers for Disease Control and Prevention Division of Viral Diseases, Pathogen Discovery	Alison Laufer Halpin; Ben L. Rambo-Martin; Clinton R. Paden; Dakota Howard; Darlene Wagner; Dave Wentworth; Dhwani Batra; Jasmine Padilla; Justin Lee; Katie Dillon; Krista Queen; Kristen Knipe; Kristine Lacek; Mark Burroughs; Matthew Schmerer; Mili Sheth; Peter Cook; Sam Shepard; Sarah Nobles; Shoshona Le; Suxiang Tong; Vivien Dugan; Yvette Unoarumhi
EPI_ISL_1897403, EPI_ISL_1897404, EPI_ISL_1897405, EPI_ISL_1897407, EPI_ISL_1897408, EPI_ISL_1897409, EPI_ISL_1897416, EPI_ISL_1897429, EPI_ISL_2096103, EPI_ISL_2096104, EPI_ISL_2096105, EPI_ISL_2096109, EPI_ISL_2096110, EPI_ISL_2096111, EPI_ISL_2096112, EPI_ISL_2096113, EPI_ISL_2336157, EPI_ISL_2336164, EPI_ISL_2336170, EPI_ISL_2336171, EPI_ISL_2336173, EPI_ISL_2336196, EPI_ISL_2336207, EPI_ISL_2336212, EPI_ISL_2336242, EPI_ISL_2336244, EPI_ISL_2336245, EPI_ISL_2378831, EPI_ISL_2378838, EPI_ISL_2378842, EPI_ISL_2378843, EPI_ISL_2378844, EPI_ISL_2378845, EPI_ISL_2378846, EPI_ISL_2378847, EPI_ISL_2378848, EPI_ISL_2378849, EPI_ISL_2378881, EPI_ISL_2378883, EPI_ISL_2378884, EPI_ISL_2378885, EPI_ISL_2378886, EPI_ISL_2378891, EPI_ISL_2443465, EPI_ISL_2557050, EPI_ISL_2651041			
see above	Washington State Department of Health Public Health Laboratories	Washington State Department of Health Public Health Laboratories	Avi Singh; Darren Lucas; Denny Russell; Drew MacKellar; Geoff Melly; Hannah Gray; Joenice Gonzalez; JohnAric Peterson; Philip Dykema; Rebecca Cao; Vanessa De Los Santos
EPI_ISL_2402825	Weill Cornell Medicine	New York Genome Center	Andre Corvelo; Arryn Craney; Chris Mason; Dayna M. Oschwald; Hanna Rennert; Lars F Westblade; Margaret Elizabeth Ross; Melissa Cushing; Michael Zody; Olivier Elemento; Priya Velu; Samantha Fennessey; Tom Maniatis
EPI_ISL_2376267, EPI_ISL_2399759, EPI_ISL_2657692, EPI_ISL_2657693, EPI_ISL_2657695	Wexner Medical Center	The Ohio State University College of Medicine	Koenig, S.; Seminetta, J.
EPI_ISL_1760181, EPI_ISL_1805458, EPI_ISL_1805780, EPI_ISL_1807191, EPI_ISL_1807199, EPI_ISL_1819295, EPI_ISL_1819296, EPI_ISL_1854743, EPI_ISL_1854746, EPI_ISL_1854748, EPI_ISL_1854751, EPI_ISL_1857044, EPI_ISL_1857061, EPI_ISL_1857070, EPI_ISL_1857087, EPI_ISL_1940143, EPI_ISL_1961015, EPI_ISL_2023321, EPI_ISL_2023341, EPI_ISL_2023343, EPI_ISL_2086433, EPI_ISL_2086435,			

EPI_ISL_2092839, EPI_ISL_2156025, EPI_ISL_2156026, EPI_ISL_2156034, EPI_ISL_2227391, EPI_ISL_2248756, EPI_ISL_2276085, EPI_ISL_2284856, EPI_ISL_2284866			
see above	Wichita State University - Molecular Diagnostics Lab	Kansas Health and Environmental Lab	Ben Olsen; Jonathan Barnell; Katherine Wiggins; Mike Grose; and Phil Adam
EPI_ISL_2279001, EPI_ISL_2279054, EPI_ISL_2279087, EPI_ISL_2279132, EPI_ISL_2279137, EPI_ISL_2279143, EPI_ISL_2279180, EPI_ISL_2279286, EPI_ISL_2279292, EPI_ISL_2279316, EPI_ISL_2279327, EPI_ISL_2279337, EPI_ISL_2279367, EPI_ISL_2279394, EPI_ISL_2279430, EPI_ISL_2279435, EPI_ISL_2279446, EPI_ISL_2279454, EPI_ISL_2279460, EPI_ISL_2279464, EPI_ISL_2279466, EPI_ISL_2279470, EPI_ISL_2279474, EPI_ISL_2279482, EPI_ISL_2279497, EPI_ISL_2558085, EPI_ISL_2558092, EPI_ISL_2558094, EPI_ISL_2558097, EPI_ISL_2558150, EPI_ISL_2558168, EPI_ISL_2558171, EPI_ISL_2558174, EPI_ISL_2558222, EPI_ISL_2558243, EPI_ISL_2558268, EPI_ISL_2558327, EPI_ISL_2558332, EPI_ISL_2558340, EPI_ISL_2558359, EPI_ISL_2558361, EPI_ISL_2558375, EPI_ISL_2558379, EPI_ISL_2558384, EPI_ISL_2558436, EPI_ISL_2558447, EPI_ISL_2558481, EPI_ISL_2558484, EPI_ISL_2558562, EPI_ISL_2558569, EPI_ISL_2558605, EPI_ISL_2558627			
see above	Wisconsin State Laboratory of Hygiene Communicable Disease Division	Wisconsin State Laboratory of Hygiene Communicable Disease Division	Abigail C. Shockey; Alicia J. Mooney; Erika M. Hanson; Kelsey R. Florek; Richard Griesser; Sara Wagner; Tonya Danz
EPI_ISL_2023097, EPI_ISL_2023184, EPI_ISL_2023211, EPI_ISL_2296383, EPI_ISL_2296387, EPI_ISL_2296389, EPI_ISL_2426129, EPI_ISL_2501685, EPI_ISL_2501703, EPI_ISL_2501704, EPI_ISL_2501705			
see above	Yale Clinical Virology Lab	Grubaugh Lab - Yale School of Public Health	Anderson Brito; Annie Watkins; Chaney Kalinich; Chantal Vogels; Isabel Ott; Jessica Rothman; Joseph Fauver; Mallery Breban; Marie L. Landry; Mary Petrone; Nathan Grubaugh; Tara Alpert
EPI_ISL_2360232	Zentrallabor Zürich	Institute of Medical Virology	Alexandra Trkola; Annette Audigé; Cyril Shah; Gabriela Ziltener; Guido Bloemberg; Jon Huder; Jürg Böni; Kevin Steiner; Maria Grünberg; Maryam Zaheri; Michael Huber; Riccarda Capaul; Stefan Schmutz; Verena Kufner
EPI_ISL_2445858	unknown	Instituto Nacional de Saude (INSA)	Borges et al
EPI_ISL_2488813	Evandro Chagas Institute	Evandro Chagas Institute	A.M.; Barbagelata; E.C.; E.M.A.; Ferreira; J.A.; Junior; K.C.; L.C.; L.S.; M.C.; P.S.; Pinheiro; Santos; Silva; Sousa; Sousa Junior; W.D.C.; da Silva

We gratefully acknowledge the following Authors from the Originating laboratories responsible for obtaining the specimens, as well as the Submitting laboratories where the genome data were generated and shared via GISAID, on which this research is based.

All Submitters of data may be contacted directly via www.gisaid.org

Authors are sorted alphabetically.

Accession ID	Originating Laboratory	Submitting Laboratory	Authors
EPI_ISL_3354027	"AK State Public Health Lab, State Health Department"	Centers for Disease Control and Prevention Division of Viral Diseases, Pathogen Discovery	Alex Burgin; Ben Rambo-Martin; Clinton Paden; Dakota Howard; Dave Wentworth; Dhwani Batra; Jasmine Padilla; Justin Lee; Krista Queen; Kristen Knipe; Kristine Lacek; Mark Burroughs; Matthew Schmerer; Meghan Bentz; Mili Sheth; Peter Cook; Sam Shepard; Sarah Nobles; Suxiang Tong; Vivien Dugan; Yvette Unoarumhi
EPI_ISL_2645302, EPI_ISL_2645305	"CO Dept. of Public Health and Environment, Lab Services Division"	Centers for Disease Control and Prevention Division of Viral Diseases, Pathogen Discovery	Alison Laufer Halpin; Ben L. Rambo-Martin; Clinton R. Paden; Dakota Howard; Darlene Wagner; Dave Wentworth; Dhwani Batra; Jasmine Padilla; Justin Lee; Katie Dillon; Krista Queen; Kristen Knipe; Kristine Lacek; Mark Burroughs; Matthew Schmerer; Mili Sheth; Peter Cook; Sam Shepard; Sarah Nobles; Shoshona Le; Suxiang Tong; Vivien Dugan; Yvette Unoarumhi
EPI_ISL_1711723	"NM Dept. Health, Scientific Laboratory Division "	Centers for Disease Control and Prevention Division of Viral Diseases, Pathogen Discovery	Alison Laufer Halpin; Ben L. Rambo-Martin; Clinton R. Paden; Dakota Howard; Darlene Wagner; Dave Wentworth; Dhwani Batra; Jasmine Padilla; Justin Lee; Katie Dillon; Krista Queen; Kristen Knipe; Kristine Lacek; Mark Burroughs; Matthew Schmerer; Mili Sheth; Peter Cook; Sam Shepard; Sarah Nobles; Shoshona Le; Suxiang Tong; Vivien Dugan; Yvette Unoarumhi
EPI_ISL_2720456	A.S.L. VERCELLI	Fondazione del Piemonte per l'Oncologia IRCCS	Antonino Sottile; Giorgio Giardina; Paola Marino; Silvia Brossa
EPI_ISL_1499576, EPI_ISL_1499577	ABC Algarve	Instituto Nacional de Saude (INSA)	Borges et al
EPI_ISL_3347192, EPI_ISL_3347193	ADILAB	Laboratorio Departamental de Salud Publica de Antioquia	Andres F. Cardona-Rios; Gloria Isabel Escobar; Idabely Betancur Ortiz; Juan P. Hernandez-Ortiz; Maria Stella López
EPI_ISL_3354677	AL Dept. of Public Health Bureau of Clinical Laboratories	Centers for Disease Control and Prevention Division of Viral Diseases, Pathogen Discovery	Alex Burgin; Ben Rambo-Martin; Clinton Paden; Dakota Howard; Dave Wentworth; Dhwani Batra; Jasmine Padilla; Justin Lee; Krista Queen; Kristen Knipe; Kristine Lacek; Mark Burroughs; Matthew Schmerer; Meghan Bentz; Mili Sheth; Peter Cook; Sam Shepard; Sarah Nobles; Suxiang Tong; Vivien Dugan; Yvette Unoarumhi
EPI_ISL_1756002, EPI_ISL_1756003, EPI_ISL_1756015	ALBANY MEDICAL CENTER	Wadsworth Center, New York State Department of Health	Alexis Russell; Catharine Prussing; Daryl M. Lamson; Erasmus Schneider; Erica Lasek-Nesselquist; John Kelly; Jonathan Plitnick; Kirsten St. George; Matthew Shudt; Melissa A Leisner; Navjot Singh
EPI_ISL_1336264	ALGEMEEN MEDISCH LABO	UAntwerp, Laboratory of Medical Microbiology	Basil Britto Xavier; Christine Lammens; Herman Goossens; Jasmine Coppens; Marie Le Mercier; Veerle Matheeussen
EPI_ISL_1795113, EPI_ISL_2345312	AMBULATORIO DE ATENDIMENTO DE DST DE GUARIBA	Instituto Butantan / ESALQ-Piracicaba	Antonio Jorge Martins; Bianca Cechetto Carlos. Mendelics: Bibiana Santos; Claudia Renata dos Santos Barros; David Schlesinger; David Schlesinger. Hemocentro Ribeirão Preto: Simone Kashima; Debora Botequio Moretti; Debora Botequio Moretti. Centro de Genômica Funcional da ESALQ: Luiz Lehmann Coutinho; Dimas Tadeu Covas; Elaine Cristina Marqueze; Elaine Vieira Santos; Elaine Vieira dos Santos; Elisangela Chicaroni Mattos; Erika Freitas; Evandra Strazza Rodrigues; Felipe Allan da Silva da Costa; Flavia Aburjaile; Guilherme Targino Valente; Heidge Fukumasu; Heidge Fukumasu. USP-Botucatu: Rejane Maria Tommasini Grotto; Instituto Butantan: Alexander Roberto Precioso; Jayme A. Souza-Neto; Jayme Augusto de Souza-Neto; Jessika Cristina Chagas Lesbon; José Salvatore Leister Patané; João Paulo Kitajima; Luiz Alcantara; Luiz Carlos Junior de Alcantara; Luiz Lehmann Coutinho; Maria Carolina Elias; Marta Giovanetti; Maurício Lacerda Nogueira; Patricia Akemi Assato; Rafael dos Santos Bezerra; Raquel de Lello Rocha Campos Cassano. NGS Soluções Genômicas: Pilar Drummond Sampaio Corrêa Mariani. FZEA-USP Pirassununga: Mirele Daiana Poleti; Raul Machado Neto; Rejane Maria Tommasini Grotto; Ricardo Augusto Brassaloti; Ricardo Haddad; Rodrigo Tocantins Calado.; Sandra Coccuzzo Sampaio; Sandra Coccuzzo Sampaio Vessoni; Simone Kashima; Svetoslav Nanev Slavov; Vagner Fonseca; Vincent Louis Viala
EPI_ISL_1795200, EPI_ISL_1795207, EPI_ISL_1795292, EPI_ISL_2345423, EPI_ISL_2345430, EPI_ISL_2345532	AMBULATORIO DE ESPECIALIDADE V E MOGI MIRIM	Instituto Butantan / ESALQ-Piracicaba	Antonio Jorge Martins; Bianca Cechetto Carlos. Mendelics: Bibiana Santos; Claudia Renata dos Santos Barros; David Schlesinger; David Schlesinger. Hemocentro Ribeirão Preto: Simone Kashima; Debora Botequio Moretti; Debora Botequio Moretti. Centro de Genômica Funcional da ESALQ: Luiz Lehmann Coutinho; Dimas Tadeu Covas; Elaine Cristina Marqueze; Elaine Vieira Santos; Elaine Vieira dos Santos; Elisangela Chicaroni Mattos; Erika Freitas; Evandra Strazza Rodrigues; Felipe Allan da Silva da Costa; Flavia Aburjaile; Guilherme Targino Valente; Heidge Fukumasu; Heidge Fukumasu. USP-Botucatu: Rejane Maria Tommasini Grotto; Instituto Butantan: Alexander Roberto Precioso; Jayme A. Souza-Neto; Jayme Augusto de Souza-Neto; Jessika Cristina Chagas Lesbon; José Salvatore Leister Patané; João Paulo Kitajima; Luiz Alcantara; Luiz Carlos Junior de Alcantara; Luiz Lehmann Coutinho; Maria Carolina Elias; Marta Giovanetti; Maurício Lacerda Nogueira; Patricia Akemi Assato; Rafael dos Santos Bezerra; Raquel de Lello Rocha Campos Cassano. NGS Soluções Genômicas: Pilar Drummond Sampaio Corrêa Mariani. FZEA-USP Pirassununga: Mirele Daiana Poleti; Raul Machado Neto; Rejane Maria Tommasini Grotto; Ricardo Augusto Brassaloti; Ricardo Haddad; Rodrigo Tocantins Calado.; Sandra Coccuzzo Sampaio; Sandra Coccuzzo Sampaio Vessoni; Simone Kashima; Svetoslav Nanev Slavov; Vagner Fonseca; Vincent Louis Viala
EPI_ISL_1967297	AMBULATORIO DE ESPECIALIDADE V E MOGI MIRIM	Instituto Butantan / Mendelics	Antonio Jorge Martins; Bianca Cechetto Carlos. Mendelics: Bibiana Santos; Claudia Renata dos Santos Barros; Cíntia Bittar; David Schlesinger. Hemocentro Ribeirão Preto: Simone Kashima; Debora Botequio Moretti; Elaine Cristina Marqueze; Elaine Vieira dos Santos; Elisangela Chicaroni Mattos; Erika Freitas; Evandra Strazza Rodrigues; Felipe Allan da Silva da Costa; Flavia Aburjaile; Fábio Sossai Possebon; Guilherme Campos; Guilherme Targino Valente; Heidge Fukumasu. USP-Botucatu: Rejane Maria Tommasini Grotto; Helena Lage Ferreira; Instituto Butantan: Dimas Tadeu Covas; Jardelina de Souza Todao Bernardino; Jayme A. Souza-Neto; Jessika Cristina Chagas Lesbon; Jorge A. Petroli Marchesi; José Salvatore Leister Patané; João Paulo Kitajima; João Pessoa Araújo Jr.; Leila Sabrina Ullmann; Loyze Paola Oliveira de Lima; Luiz Aurelio de Campos Crispin. Centro de Genômica Funcional da ESALQ: Luiz Lehmann Coutinho; Luiz Carlos Junior de Alcantara; Lívia Sacchetto; Maisa C. Pereira Parra; Maria Carolina Elias; Marta Giovanetti; Marília Moraes; Maurício Lacerda Nogueira. Prefeitura de Sao Paulo: Melissa Palmieri.; Patricia Akemi Assato; Paula Rahal; Paulo Inacio da Costa; Rafael dos Santos Bezerra; Raquel de Lello Rocha Campos Cassano. NGS Soluções Genômicas: Pilar Drummond Sampaio Corrêa Mariani. FZEA-USP Pirassununga: Mirele Daiana Poleti; Raul Machado Neto; Ricardo Augusto Brassaloti; Ricardo Haddad; Rodrigo Tocantins Calado. FAMERP-SJRP: Cecília Artico Banho; Sandra Coccuzzo Sampaio; Svetoslav Nanev Slavov; Vagner Fonseca; Vincent Louis Viala
EPI_ISL_1967028	AMBULATORIO DE MOLESTIAS INFECCIOSAS DE SALTO	Instituto Butantan / ESALQ-USP (Piracicaba)	Antonio Jorge Martins; Bianca Cechetto Carlos. Mendelics: Bibiana Santos; Claudia Renata dos Santos Barros; Cíntia Bittar; David Schlesinger. Hemocentro Ribeirão Preto: Simone Kashima; Debora Botequio Moretti; Elaine Cristina Marqueze; Elaine Vieira dos Santos; Elisangela Chicaroni Mattos; Erika Freitas; Evandra Strazza Rodrigues; Felipe Allan da Silva da Costa; Flavia Aburjaile; Fábio Sossai Possebon; Guilherme Campos; Guilherme Targino Valente; Heidge Fukumasu. USP-Botucatu: Rejane Maria Tommasini Grotto; Helena Lage Ferreira; Instituto Butantan: Dimas Tadeu Covas; Jardelina de Souza Todao Bernardino; Jayme A. Souza-Neto; Jessika Cristina Chagas Lesbon; Jorge A. Petroli Marchesi; José Salvatore Leister Patané; João Paulo Kitajima; João Pessoa Araújo Jr.; Leila Sabrina Ullmann; Loyze Paola Oliveira de Lima; Luiz Aurelio de Campos Crispin. Centro de Genômica Funcional da ESALQ: Luiz Lehmann Coutinho; Luiz Carlos Junior de Alcantara; Lívia Sacchetto; Maisa C. Pereira Parra; Maria Carolina Elias; Marta Giovanetti; Marília Moraes; Maurício Lacerda Nogueira. Prefeitura de Sao Paulo: Melissa Palmieri.; Patricia Akemi Assato; Paula Rahal; Paulo Inacio da Costa; Rafael dos Santos Bezerra; Raquel de Lello Rocha Campos Cassano. NGS Soluções Genômicas: Pilar Drummond Sampaio Corrêa Mariani. FZEA-USP Pirassununga: Mirele Daiana Poleti; Raul Machado Neto; Ricardo Augusto Brassaloti; Ricardo Haddad; Rodrigo Tocantins Calado. FAMERP-SJRP: Cecília Artico Banho; Sandra Coccuzzo Sampaio; Svetoslav Nanev Slavov; Vagner Fonseca; Vincent Louis Viala
EPI_ISL_1966095	AMBULATORIO MEDICO DE ESPECIALIDADES DE PERUIBE	Instituto Butantan / Mendelics	Antonio Jorge Martins; Bianca Cechetto Carlos. Mendelics: Bibiana Santos; Claudia Renata dos Santos Barros; Cíntia Bittar; David Schlesinger. Hemocentro Ribeirão Preto: Simone Kashima; Debora Botequio Moretti; Elaine Cristina Marqueze; Elaine Vieira dos Santos; Elisangela Chicaroni Mattos; Erika Freitas; Evandra Strazza Rodrigues; Felipe Allan da Silva da Costa; Flavia Aburjaile; Fábio Sossai Possebon; Guilherme Campos; Guilherme Targino Valente; Heidge Fukumasu. USP-Botucatu: Rejane Maria Tommasini Grotto; Helena Lage Ferreira; Instituto Butantan: Dimas Tadeu Covas; Jardelina de Souza Todao Bernardino; Jayme A. Souza-Neto; Jessika Cristina Chagas Lesbon; Jorge A. Petroli Marchesi; José Salvatore Leister Patané; João Paulo Kitajima; João Pessoa Araújo Jr.; Leila Sabrina Ullmann; Loyze Paola Oliveira de Lima; Luiz Aurelio de Campos Crispin. Centro de Genômica Funcional da ESALQ: Luiz Lehmann Coutinho; Luiz Carlos Junior de Alcantara; Lívia Sacchetto; Maisa C. Pereira Parra; Maria Carolina Elias; Marta Giovanetti; Marília Moraes; Maurício Lacerda Nogueira. Prefeitura de Sao Paulo: Melissa Palmieri.; Patricia Akemi Assato; Paula Rahal; Paulo Inacio da Costa; Rafael dos Santos Bezerra; Raquel de Lello Rocha Campos Cassano. NGS Soluções Genômicas: Pilar Drummond Sampaio Corrêa Mariani. FZEA-USP Pirassununga: Mirele Daiana Poleti; Raul Machado Neto; Ricardo Augusto Brassaloti; Ricardo Haddad; Rodrigo Tocantins Calado. FAMERP-SJRP: Cecília Artico Banho; Sandra Coccuzzo Sampaio; Svetoslav Nanev Slavov; Vagner Fonseca; Vincent Louis Viala
EPI_ISL_1795367, EPI_ISL_2345940	AMBULATORIO MEDICO MUNICIPAL DE AGUDOS	Instituto Butantan / ESALQ-Piracicaba	Antonio Jorge Martins; Bianca Cechetto Carlos. Mendelics: Bibiana Santos; Claudia Renata dos Santos Barros; David Schlesinger; David Schlesinger. Hemocentro Ribeirão Preto: Simone Kashima; Debora Botequio Moretti; Debora Botequio Moretti. Centro de Genômica Funcional da ESALQ: Luiz Lehmann Coutinho; Dimas Tadeu Covas; Elaine Cristina Marqueze; Elaine Vieira Santos; Elaine Vieira dos Santos; Elisangela Chicaroni Mattos; Erika Freitas; Evandra Strazza Rodrigues; Felipe Allan da Silva da Costa; Flavia Aburjaile; Guilherme Targino Valente; Heidge Fukumasu; Heidge Fukumasu. USP-Botucatu: Rejane Maria Tommasini Grotto; Instituto Butantan: Alexander Roberto Precioso; Jayme A. Souza-Neto; Jayme Augusto de Souza-Neto; Jessika Cristina Chagas Lesbon; José Salvatore Leister Patané; João Paulo Kitajima; Luiz Alcantara; Luiz Carlos Junior de Alcantara; Luiz Lehmann Coutinho; Maria Carolina Elias; Marta Giovanetti; Maurício Lacerda Nogueira; Patricia Akemi Assato; Rafael dos Santos Bezerra; Raquel de Lello Rocha Campos Cassano. NGS Soluções Genômicas: Pilar Drummond Sampaio Corrêa Mariani. FZEA-USP Pirassununga: Mirele Daiana Poleti; Raul Machado Neto; Rejane Maria Tommasini Grotto; Ricardo Augusto Brassaloti; Ricardo Haddad; Rodrigo Tocantins Calado.; Sandra Coccuzzo Sampaio; Sandra Coccuzzo Sampaio Vessoni; Simone Kashima; Svetoslav Nanev Slavov; Vagner Fonseca; Vincent Louis Viala
EPI_ISL_2018198, EPI_ISL_2018203	AR Dept. of Health-PHL, Molecular Diagnostics	Centers for Disease Control and Prevention Division of Viral Diseases, Pathogen Discovery	Alison Laufer Halpin; Ben L. Rambo-Martin; Clinton R. Paden; Dakota Howard; Darlene Wagner; Dave Wentworth; Dhwani Batra; Jasmine Padilla; Justin Lee; Katie Dillon; Krista Queen; Kristen Knipe; Kristine Lacek; Mark Burroughs; Matthew Schmerer; Mili Sheth; Peter Cook; Sam Shepard; Sarah Nobles; Shoshona Le; Suxiang Tong; Vivien Dugan; Yvette Unoarumhi
EPI_ISL_2272980, EPI_ISL_2272981	AREA DE SALUD CARRILLO	Inciensa, Instituto Costarricense de Investigación y Enseñanza en Nutrición y Salud	Adriana Godínez; Claudio Soto-Garita; Estela Cordero; Francisco Duarte; Hebleen Porras; Joselyn Prado & Adriana Bemúdez Espinoza; José Luis Vargas; Mariela Gutiérrez; Melany Calderón
EPI_ISL_1827536	AREA DE SALUD COTO BRUS	Inciensa, Instituto Costarricense de Investigación y Enseñanza en Nutrición y Salud	Adriana Godínez; Claudio Soto-Garita; Estela Cordero; Francisco Duarte; Hebleen Porras; Joselyn Prado & Fabricio Aguilar; José Luis Vargas; Mariela Gutiérrez; Melany Calderón
EPI_ISL_1827523	AREA DE SALUD CURRIDABAT 2	Inciensa, Instituto Costarricense de Investigación y Enseñanza en Nutrición y Salud	Adriana Godínez; Claudio Soto-Garita; Estela Cordero; Francisco Duarte; Hebleen Porras; José Luis Vargas; Mariela Gutiérrez & Joselyn Prado; Melany Calderón
EPI_ISL_2272999	AREA DE SALUD DESAMPARADOS 1 - CLINICA DR. MARCIAL FALLAS	Inciensa, Instituto Costarricense de Investigación y Enseñanza en Nutrición	Adriana Godínez; Claudio Soto-Garita; Estela Cordero; Francisco Duarte; Hebleen Porras; Joselyn Prado & Anthony Cordero-Jiménez; José Luis Vargas; Mariela Gutiérrez; Melany Calderón

		y Salud	
EPI_ISL_2103398	AREA DE SALUD GOICOECHEA 2 - CLINICA DR. JIMENEZ NUÑEZ	Inciensa, Instituto Costarricense de Investigación y Enseñanza en Nutrición y Salud	Adriana Godínez; Caterina Guzmán; Claudio Soto-Garita; Estela Cordero; Francisco Duarte; Hebleen Porras; Joselyn Prado; José Luis Vargas; Mariela Gutiérrez; Melany Calderón; Nazareth Ruiz & Mariel López
EPI_ISL_2272960	AREA DE SALUD LA CRUZ	Inciensa, Instituto Costarricense de Investigación y Enseñanza en Nutrición y Salud	Adriana Godínez; Claudio Soto-Garita; Estela Cordero; Francisco Duarte; Hebleen Porras; Joselyn Prado & Arnoldo Zamora-Villegas; José Luis Vargas; Mariela Gutiérrez; Melany Calderón
EPI_ISL_1827525	AREA DE SALUD LOS CHILES	Inciensa, Instituto Costarricense de Investigación y Enseñanza en Nutrición y Salud	Adriana Godínez; Claudio Soto-Garita; Estela Cordero; Francisco Duarte; Hebleen Porras; Joselyn Prado & Teresita Somogyi; José Luis Vargas; Mariela Gutiérrez; Melany Calderón
EPI_ISL_1117051	ARS Algarve - Laboratorio Laura Ayres	Instituto Nacional de Saude (INSA)	Borges et al
EPI_ISL_1811234	AS Santa Barbara	Inciensa, Instituto Costarricense de Investigación y Enseñanza en Nutrición y Salud	Pérez-Corrales C & Aguilar-Monge R
EPI_ISL_1418241	AX BIO OCEAN	CNR Virus des Infections Respiratoires - France SUD	Antonin Bal; Bruno Lina; Gregory Destras; Gwendolyne Burfin; Hadrien Regue; Laurence Josset; Martine Valette; Quentin Semanas
EPI_ISL_1176304, EPI_ISL_1824637	AZ Klina	AZ Klina	Carl Vael - Lynsey Berckmans; Dr. C. Vael
EPI_ISL_1493499, EPI_ISL_1493500, EPI_ISL_1493501, EPI_ISL_1493553, EPI_ISL_1550407, EPI_ISL_1550762, EPI_ISL_1550831, EPI_ISL_1550859, EPI_ISL_1551112, EPI_ISL_1551279, EPI_ISL_1551325, EPI_ISL_1551366, EPI_ISL_1551415, EPI_ISL_1551610, EPI_ISL_1559815, EPI_ISL_1559994, EPI_ISL_1560203, EPI_ISL_1560507, EPI_ISL_1560534, EPI_ISL_1560587, EPI_ISL_1560646, EPI_ISL_1560762, EPI_ISL_1560783, EPI_ISL_1560846, EPI_ISL_1560857, EPI_ISL_1560865, EPI_ISL_1561024, EPI_ISL_1561065, EPI_ISL_1561280, EPI_ISL_1561621, EPI_ISL_1561714, EPI_ISL_1562168, EPI_ISL_1562577, EPI_ISL_1562578, EPI_ISL_1562633, EPI_ISL_1562647, EPI_ISL_1562648, EPI_ISL_1562745, EPI_ISL_1562882, EPI_ISL_1563030, EPI_ISL_1563037, EPI_ISL_1563197, EPI_ISL_1563451, EPI_ISL_1563484, EPI_ISL_1563485, EPI_ISL_1563567, EPI_ISL_1648968, EPI_ISL_1649227, EPI_ISL_1649229, EPI_ISL_1649311, EPI_ISL_1649343, EPI_ISL_1649457, EPI_ISL_1649748, EPI_ISL_1649929, EPI_ISL_1649947, EPI_ISL_1649978, EPI_ISL_1649984, EPI_ISL_1650057, EPI_ISL_1650105, EPI_ISL_1650172, EPI_ISL_1650219, EPI_ISL_1650262, EPI_ISL_1650314, EPI_ISL_1650376, EPI_ISL_1650389, EPI_ISL_1650398, EPI_ISL_1650399, EPI_ISL_1650441, EPI_ISL_1650469, EPI_ISL_1650489, EPI_ISL_1650624, EPI_ISL_1650644, EPI_ISL_1650668, EPI_ISL_1650750, EPI_ISL_1651013, EPI_ISL_1651035, EPI_ISL_1651095, EPI_ISL_1651186, EPI_ISL_1667450, EPI_ISL_1667530, EPI_ISL_1667626, EPI_ISL_1667658, EPI_ISL_1667693, EPI_ISL_1667694, EPI_ISL_1667718, EPI_ISL_1667793, EPI_ISL_1667799, EPI_ISL_1667800, EPI_ISL_1667935, EPI_ISL_1667953, EPI_ISL_1668005, EPI_ISL_1668007, EPI_ISL_1668010, EPI_ISL_1668012, EPI_ISL_1668078, EPI_ISL_1668099, EPI_ISL_1668107, EPI_ISL_1668208, EPI_ISL_1668233, EPI_ISL_1668276, EPI_ISL_1668327, EPI_ISL_1668426, EPI_ISL_1668442, EPI_ISL_1685525, EPI_ISL_1685548, EPI_ISL_1685650, EPI_ISL_1685679, EPI_ISL_1685686, EPI_ISL_1685735, EPI_ISL_1685870, EPI_ISL_1685896, EPI_ISL_1685935, EPI_ISL_1685956, EPI_ISL_1685971, EPI_ISL_1685973, EPI_ISL_1686002, EPI_ISL_1686005, EPI_ISL_1686186, EPI_ISL_1686249, EPI_ISL_1686252, EPI_ISL_1686319, EPI_ISL_1686339, EPI_ISL_1686355, EPI_ISL_1686356, EPI_ISL_1686391, EPI_ISL_1686412, EPI_ISL_1686484, EPI_ISL_1686487, EPI_ISL_1686489, EPI_ISL_1686554, EPI_ISL_1686581, EPI_ISL_1686609, EPI_ISL_1686626, EPI_ISL_1686670, EPI_ISL_1686678, EPI_ISL_1686683, EPI_ISL_1686692, EPI_ISL_1686858, EPI_ISL_1686866, EPI_ISL_1686870, EPI_ISL_1686946, EPI_ISL_1686996, EPI_ISL_1687001, EPI_ISL_1687015, EPI_ISL_1687097, EPI_ISL_1687245, EPI_ISL_1687312, EPI_ISL_1687428, EPI_ISL_1687429, EPI_ISL_1687449, EPI_ISL_1687541, EPI_ISL_1687693, EPI_ISL_1687700, EPI_ISL_1687884, EPI_ISL_1688102, EPI_ISL_1688174, EPI_ISL_1688305, EPI_ISL_1688362, EPI_ISL_1688422, EPI_ISL_1688611, EPI_ISL_1688647, EPI_ISL_1688668, EPI_ISL_1688712, EPI_ISL_1688775, EPI_ISL_1688803, EPI_ISL_1688843, EPI_ISL_1688846, EPI_ISL_1688852, EPI_ISL_1688970, EPI_ISL_1689070, EPI_ISL_1689089, EPI_ISL_1689214, EPI_ISL_1689314, EPI_ISL_1689333, EPI_ISL_1689343, EPI_ISL_1689544, EPI_ISL_1689616, EPI_ISL_1689783, EPI_ISL_1689795, EPI_ISL_1689800, EPI_ISL_1689835, EPI_ISL_1689902, EPI_ISL_1689933, EPI_ISL_1690148, EPI_ISL_1690158, EPI_ISL_1695136, EPI_ISL_1695204, EPI_ISL_1695299, EPI_ISL_1695330, EPI_ISL_1695521, EPI_ISL_1701897, EPI_ISL_1701962, EPI_ISL_1701964, EPI_ISL_1702109, EPI_ISL_1702112, EPI_ISL_1702120, EPI_ISL_1702722, EPI_ISL_1702841, EPI_ISL_1702935, EPI_ISL_1702936, EPI_ISL_1702937, EPI_ISL_1702943, EPI_ISL_1702959, EPI_ISL_1711975, EPI_ISL_1735706, EPI_ISL_1735708, EPI_ISL_1735737, EPI_ISL_1735739, EPI_ISL_1735779, EPI_ISL_1735796, EPI_ISL_1735828, EPI_ISL_1735838, EPI_ISL_1735874, EPI_ISL_1735883, EPI_ISL_1735889, EPI_ISL_1735953, EPI_ISL_1735979, EPI_ISL_1735987, EPI_ISL_1736123, EPI_ISL_1736134, EPI_ISL_1736224, EPI_ISL_1736249, EPI_ISL_1736312, EPI_ISL_1736359, EPI_ISL_1736432, EPI_ISL_1736463, EPI_ISL_1736522, EPI_ISL_1736543, EPI_ISL_1736594, EPI_ISL_1736597, EPI_ISL_1736604, EPI_ISL_1736638, EPI_ISL_1736657, EPI_ISL_1736712, EPI_ISL_1736755, EPI_ISL_1736757, EPI_ISL_1736761, EPI_ISL_1736762, EPI_ISL_1736801, EPI_ISL_1736807, EPI_ISL_1736812, EPI_ISL_1736861, EPI_ISL_1736876, EPI_ISL_1736879, EPI_ISL_1736973, EPI_ISL_1737044, EPI_ISL_1737060, EPI_ISL_1737113, EPI_ISL_1737163, EPI_ISL_1737358, EPI_ISL_1737385, EPI_ISL_1737414, EPI_ISL_1737459, EPI_ISL_1737476, EPI_ISL_1737492, EPI_ISL_1737531, EPI_ISL_1737532, EPI_ISL_1737575, EPI_ISL_1737587, EPI_ISL_1737599, EPI_ISL_1737616, EPI_ISL_1737640, EPI_ISL_1737662, EPI_ISL_1737673, EPI_ISL_1753545, EPI_ISL_1753558, EPI_ISL_1834452, EPI_ISL_1834477, EPI_ISL_1834479, EPI_ISL_1834524, EPI_ISL_1834597, EPI_ISL_1834789, EPI_ISL_1834822, EPI_ISL_1834853, EPI_ISL_1834865, EPI_ISL_1834874, EPI_ISL_1834944, EPI_ISL_1834993, EPI_ISL_1835043, EPI_ISL_1835044, EPI_ISL_1835045, EPI_ISL_1835046, EPI_ISL_1835092, EPI_ISL_1835153, EPI_ISL_1835185, EPI_ISL_1835258, EPI_ISL_1835267, EPI_ISL_1835310, EPI_ISL_1835388, EPI_ISL_1835395, EPI_ISL_1835396, EPI_ISL_1835413, EPI_ISL_1835419, EPI_ISL_1835420, EPI_ISL_1835421, EPI_ISL_1835423, EPI_ISL_1835424, EPI_ISL_1835425, EPI_ISL_1835427, EPI_ISL_1835445, EPI_ISL_1835462, EPI_ISL_1835466, EPI_ISL_1835567, EPI_ISL_1835602, EPI_ISL_1835614, EPI_ISL_1835826, EPI_ISL_1835857, EPI_ISL_1835915, EPI_ISL_1835982, EPI_ISL_1835992, EPI_ISL_1836083, EPI_ISL_1836086, EPI_ISL_1836115, EPI_ISL_1836213, EPI_ISL_1836270, EPI_ISL_1836279, EPI_ISL_1836317, EPI_ISL_1836323, EPI_ISL_1836326, EPI_ISL_1836464, EPI_ISL_1836649, EPI_ISL_1836670, EPI_ISL_1836731, EPI_ISL_1836735, EPI_ISL_1836839, EPI_ISL_1836841, EPI_ISL_1836879, EPI_ISL_1836897, EPI_ISL_1836955, EPI_ISL_1837138, EPI_ISL_1837172, EPI_ISL_1837218, EPI_ISL_1837308, EPI_ISL_1837343, EPI_ISL_1837427, EPI_ISL_1837492, EPI_ISL_1837525, EPI_ISL_1837526, EPI_ISL_1837537, EPI_ISL_1837677, EPI_ISL_1837729, EPI_ISL_1837747, EPI_ISL_1837794, EPI_ISL_1837806, EPI_ISL_1837819, EPI_ISL_1837820, EPI_ISL_1837822, EPI_ISL_1837948, EPI_ISL_1838108, EPI_ISL_1838177, EPI_ISL_1838181, EPI_ISL_1838300, EPI_ISL_1838394, EPI_ISL_1900196, EPI_ISL_1900212, EPI_ISL_1900275, EPI_ISL_1900345, EPI_ISL_1900379, EPI_ISL_1900384, EPI_ISL_1900402, EPI_ISL_1900415, EPI_ISL_1900417, EPI_ISL_1922902, EPI_ISL_1922930, EPI_ISL_1922950, EPI_ISL_1922958, EPI_ISL_1922976, EPI_ISL_1922977, EPI_ISL_1923120, EPI_ISL_1923146, EPI_ISL_1925151, EPI_ISL_1925241, EPI_ISL_1925284, EPI_ISL_1925476, EPI_ISL_1925492, EPI_ISL_1994977, EPI_ISL_1994999, EPI_ISL_1995021, EPI_ISL_1995094, EPI_ISL_1995155, EPI_ISL_1995255, EPI_ISL_1995285, EPI_ISL_1995293, EPI_ISL_1995305, EPI_ISL_1995325, EPI_ISL_1995348, EPI_ISL_1995362, EPI_ISL_1995418, EPI_ISL_1995434, EPI_ISL_1995438, EPI_ISL_1995476, EPI_ISL_1995521, EPI_ISL_1995533, EPI_ISL_1995561, EPI_ISL_1995565, EPI_ISL_1995588, EPI_ISL_1995612, EPI_ISL_1995615, EPI_ISL_1995635, EPI_ISL_1995664, EPI_ISL_1995683, EPI_ISL_1995699, EPI_ISL_1995811, EPI_ISL_1995838, EPI_ISL_1995846, EPI_ISL_1995879, EPI_ISL_1995922, EPI_ISL_1995926, EPI_ISL_1995939, EPI_ISL_1995986, EPI_ISL_1996000, EPI_ISL_1996001, EPI_ISL_1996015, EPI_ISL_1996026, EPI_ISL_1996030, EPI_ISL_1996071, EPI_ISL_1996121, EPI_ISL_1996152, EPI_ISL_1996203, EPI_ISL_1996222, EPI_ISL_1996269, EPI_ISL_1996340, EPI_ISL_1996351, EPI_ISL_1996361, EPI_ISL_1996373, EPI_ISL_1996378, EPI_ISL_1996379, EPI_ISL_1996455, EPI_ISL_1996469, EPI_ISL_1996480, EPI_ISL_1996496, EPI_ISL_1996524, EPI_ISL_1996624, EPI_ISL_1996685, EPI_ISL_1996690, EPI_ISL_1996701, EPI_ISL_1996714, EPI_ISL_1996772, EPI_ISL_1996773, EPI_ISL_1996785, EPI_ISL_1996834, EPI_ISL_1996835, EPI_ISL_1996836, EPI_ISL_1996858, EPI_ISL_1996872, EPI_ISL_1996874, EPI_ISL_1996881, EPI_ISL_1996887, EPI_ISL_1996942, EPI_ISL_1997051, EPI_ISL_1997205, EPI_ISL_1997207, EPI_ISL_1997252, EPI_ISL_1997345, EPI_ISL_1997360, EPI_ISL_1997461, EPI_ISL_1997492, EPI_ISL_1997602, EPI_ISL_1997607, EPI_ISL_1997640, EPI_ISL_1997642, EPI_ISL_1997738, EPI_ISL_1997769, EPI_ISL_1997791, EPI_ISL_1997838, EPI_ISL_1997887, EPI_ISL_1997889, EPI_ISL_1997913, EPI_ISL_1998098, EPI_ISL_1998179, EPI_ISL_1998188, EPI_ISL_1998194, EPI_ISL_1998195, EPI_ISL_1998241, EPI_ISL_1998348, EPI_ISL_1998350, EPI_ISL_1998374, EPI_ISL_1998518, EPI_ISL_1998555, EPI_ISL_1998563, EPI_ISL_1998564, EPI_ISL_1998573, EPI_ISL_1998612, EPI_ISL_1998636, EPI_ISL_1998679, EPI_ISL_1998735, EPI_ISL_1998787, EPI_ISL_1998795, EPI_ISL_1998802, EPI_ISL_1998808, EPI_ISL_1998810, EPI_ISL_1998855, EPI_ISL_1998868, EPI_ISL_1998955, EPI_ISL_1999015, EPI_ISL_1999034, EPI_ISL_1999085, EPI_ISL_1999086, EPI_ISL_1999161, EPI_ISL_1999177, EPI_ISL_1999178, EPI_ISL_1999239, EPI_ISL_1999267, EPI_ISL_1999322, EPI_ISL_1999334, EPI_ISL_1999488, EPI_ISL_1999582, EPI_ISL_1999607, EPI_ISL_1999668, EPI_ISL_1999879, EPI_ISL_1999949, EPI_ISL_2000154, EPI_ISL_2000155, EPI_ISL_2000167, EPI_ISL_2000195, EPI_ISL_2000208, EPI_ISL_2000336, EPI_ISL_2000345, EPI_ISL_2000349, EPI_ISL_2000370, EPI_ISL_2000373, EPI_ISL_2000413, EPI_ISL_2000425, EPI_ISL_2000427, EPI_ISL_2000458, EPI_ISL_2000489, EPI_ISL_2000539, EPI_ISL_2035114, EPI_ISL_2035138, EPI_ISL_2035156, EPI_ISL_2035204, EPI_ISL_2035219, EPI_ISL_2035227, EPI_ISL_2038984, EPI_ISL_2039001, EPI_ISL_2039174, EPI_ISL_2039191, EPI_ISL_2039192, EPI_ISL_2039194, EPI_ISL_2039297, EPI_ISL_2039301, EPI_ISL_2039314, EPI_ISL_2039404, EPI_ISL_2039551, EPI_ISL_2039579, EPI_ISL_2039716, EPI_ISL_2040012, EPI_ISL_2040080, EPI_ISL_2040085, EPI_ISL_2040104, EPI_ISL_2040108, EPI_ISL_2040423, EPI_ISL_2040513, EPI_ISL_2040629, EPI_ISL_2040657, EPI_ISL_2040689, EPI_ISL_2040725, EPI_ISL_2040851, EPI_ISL_2040885, EPI_ISL_2040899, EPI_ISL_2040900, EPI_ISL_2041010, EPI_ISL_2041012, EPI_ISL_2041018, EPI_ISL_2041046, EPI_ISL_2041123, EPI_ISL_2041155, EPI_ISL_2041179, EPI_ISL_2041215, EPI_ISL_2041216, EPI_ISL_2041280, EPI_ISL_2041362, EPI_ISL_2041377, EPI_ISL_2041564, EPI_ISL_2041601, EPI_ISL_2041633, EPI_ISL_2041721, EPI_ISL_2041723, EPI_ISL_2041730, EPI_ISL_2041739, EPI_ISL_2041980, EPI_ISL_2041984, EPI_ISL_2042007, EPI_ISL_2042009, EPI_ISL_2042128, EPI_ISL_2042129, EPI_ISL_2042150, EPI_ISL_2042170, EPI_ISL_2042171, EPI_ISL_2042180, EPI_ISL_2042293, EPI_ISL_2042390, EPI_ISL_2042393, EPI_ISL_2042423, EPI_ISL_2042466, EPI_ISL_2042470, EPI_ISL_2042482, EPI_ISL_2149527, EPI_ISL_2149536, EPI_ISL_2149538, EPI_ISL_2149539, EPI_ISL_2149971, EPI_ISL_2150312, EPI_ISL_2186024, EPI_ISL_2186039, EPI_ISL_2186091, EPI_ISL_2186157, EPI_ISL_2201173, EPI_ISL_2201195, EPI_ISL_2201369, EPI_ISL_2201449, EPI_ISL_2201547, EPI_ISL_2201774, EPI_ISL_2201864, EPI_ISL_2201886, EPI_ISL_2201920, EPI_ISL_2201925, EPI_ISL_2201942, EPI_ISL_2202002, EPI_ISL_2202004, EPI_ISL_2202051, EPI_ISL_2202058, EPI_ISL_2202110, EPI_ISL_2202115, EPI_ISL_2202284, EPI_ISL_2202565, EPI_ISL_2202609, EPI_ISL_2202618, EPI_ISL_2202699, EPI_ISL_2202760, EPI_ISL_2202821, EPI_ISL_2202961, EPI_ISL_2241697, EPI_ISL_2241753, EPI_ISL_2241758, EPI_ISL_2241761, EPI_ISL_2241780, EPI_ISL_2241821, EPI_ISL_2242042, EPI_ISL_2242079, EPI_ISL_2242099, EPI_ISL_2242148, EPI_ISL_2242150, EPI_ISL_2242182, EPI_ISL_2242265, EPI_ISL_2242272, EPI_ISL_2242344, EPI_ISL_2242362, EPI_ISL_2242422, EPI_ISL_2242903, EPI_ISL_2242974, EPI_ISL_2242976, EPI_ISL_2242991, EPI_ISL_2421304, EPI_ISL_2421336, EPI_ISL_2421341, EPI_ISL_2421365, EPI_ISL_2421418, EPI_ISL_2421490, EPI_ISL_2421516, EPI_ISL_2421551, EPI_ISL_2421556, EPI_ISL_2421586, EPI_ISL_2421598, EPI_ISL_2421614, EPI_ISL_2421641, EPI_ISL_2421733, EPI_ISL_2421740, EPI_ISL_2421767, EPI_ISL_2442303, EPI_ISL_2442316, EPI_ISL_2442319, EPI_ISL_2442321, EPI_ISL_2482363, EPI_ISL_2482383, EPI_ISL_2482422, EPI_ISL_2600021, EPI_ISL_2600207, EPI_ISL_2649903, EPI_ISL_2649914			
see above	Aegis Sciences Corporation	Centers for Disease Control and Prevention Division of Viral Diseases, Pathogen Discovery	Adrian Paskey; Alec Vest; Benjamin Rambo-Martin; Christopher Gulvick; Clinton R. Paden; Cyndi Clark; Dakota Howard; Darlene Wagner; Dhwani Batra; Dillon Nall; Duncan MacCannell; Ethan Sanders; Holly Houdeshell; Jason Caravas; Kara Moser; Matthew Hardison; Matthew Schmerer; Ola Kvalvaag; Patrick Campbell; Peter W. Cook; Rob Case; Scott Sammons; Shatavia Morrison; Shaun Westlund; Vikramsinha Ghorpade; Yvette Unoarumhi
EPI_ISL_1201884, EPI_ISL_1219029, EPI_ISL_1219030, EPI_ISL_1219033, EPI_ISL_1219036	Aeroporto Internacional de Guarulhos	Instituto Adolfo Lutz, Interdiciplinary Procedures Center, Strategic Laboratory	Caio Vinicius Dias Lopes; Claudia Regina Gonçalves; Claudio Tavares Sacchi; Erica Valessa Ramos Gomes; Karoline Rodrigues Campos
EPI_ISL_1739043	Affidea	Instituto Nacional de Saude (INSA)	Borges et al
EPI_ISL_1854251	Affidea	Instituto Nacional de Saude (INSA) and Institute of Biomedicine (iBiMed), Universidade de Aveiro	Borges et al
EPI_ISL_1061282, EPI_ISL_1182734, EPI_ISL_1182768, EPI_ISL_1182769, EPI_ISL_1483589, EPI_ISL_1652201, EPI_ISL_1653895, EPI_ISL_1675103, EPI_ISL_1789630, EPI_ISL_1893796			
see above	Alaska State Virology Laboratory	Alaska State Virology Laboratory	Elva House; Jack Chen; Jacob Zidek; Lisa Smith; Ph.D.; Stephanie DeRonde
EPI_ISL_1587072	Altius Institute	Seattle Flu Study	Alex Nguyen; Amanda Adler; Andrew Meuser; Barry R. Lutz; Benjamin Pelle; Caitlin R. Wolf; Chris D. Frazar; Clem Green; Daniel Bates; Deborah A. Nickerson; Elisabeth Brandstetter; Erica Ryke; Hannah Petersen; Helen Y. Chu; Jacob Rodriguez; Janet A. Englund; Jay Shendure; Jessica Halow; John Stamatoyannopoulos; Joshua Richards; Jover Lee; Julia Wald; Kairsten Fay; Kirsten Lacombe; Kneshay Harper; Lea M. Starita; Mark J. Rieder; Matt Hartman; Matthew Richardson; Matthew Thompson; Melissa Truong; Michael Boeckh; Michael Famulare; Misja Ilcisin; Muhammad Halimun; Olivia Waltner; Peter D. Han; Rebecca Bruders; Ryan Alexander; Sadie Patraw; Sofia Olsson; Stephanie DeBaun; Thomas R. Sibley; Tobias Ragoczy; Trevor Bedford; Truong Nguyen
EPI_ISL_1625972	Ama J Angela	Instituto Adolfo Lutz, Interdiciplinary Procedures Center, Strategic Laboratory	Caio Vinicius Dias Lopes; Claudia Regina Gonçalves; Claudio Tavares Sacchi; Erica Valessa Ramos Gomes; Karoline Rodrigues Campos; Katia Correa de Oliveira Santos; Leonardo Jose Tadeu de Araujo
EPI_ISL_1966328, EPI_ISL_1966331, EPI_ISL_1966340, EPI_ISL_1966497, EPI_ISL_1966499, EPI_ISL_1966500, EPI_ISL_1966506			
see above	Ambulatório IB	Instituto Butantan / Mendelics	Antonio Jorge Martins; Bianca Cechetto Carlos. Mendelics: Bibiana Santos; Claudia Renata dos Santos Barros; Cíntia Bittar; David Schlesinger. Hemocentro Ribeirão Preto: Simone Kashima; Debora Botequio Moretti; Elaine Cristina Marqueze; Elaine Vieira dos Santos; Elisangela Chicaroni Mattos; Erika Freitas; Evandra Strazza Rodrigues; Felipe Allan da Silva da Costa; Flavia Aburjaile; Fábio Sossai Possebon; Guilherme Campos; Guilherme Targino Valente; Heidge Fukumasu. USP-Botucatu: Rejane Maria Tommasini Grotto; Helena Lage Ferreira; Instituto Butantan: Dimas Tadeu Covas; Jardelina de Souza Todao Bernardino; Jayme A. Souza-Neto; Jessika Cristina Chagas Lesbon; Jorge A. Petroli Marchesi; José Salvatore Leister Patané; João Paulo Kitajima; João Pessoa Araújo Jr.; Leila Sabrina Ullmann; Loyze Paola Oliveira de Lima; Luiz Aurelio de Campos Crispin. Centro de Genômica Funcional da ESALQ: Luiz Lehmann Coutinho; Luiz Carlos Junior de Alcantara; Lívia Sacchetto; Maisa C. Pereira Parra; Maria Carolina Elias; Marta Giovanetti; Marília Moraes; Maurício Lacerda Nogueira. Prefeitura de Sao Paulo: Melissa Palmieri.; Patricia Akemi Assato; Paula Rahal; Paulo Inacio da Costa; Rafael dos Santos Bezerra; Raquel de Lello Rocha Campos Cassano. NGS Soluções Genômicas: Pilar Drummond Sampaio Corrêa Mariani. FZEA-USP Pirassununga: Mirele Daiana Poleti; Raul Machado Neto; Ricardo Augusto Brassaloti; Ricardo Haddad; Rodrigo Tocantins Calado. FAMERP-SJRP: Cecília Artico Banho; Sandra Coccuzzo Sampaio; Svetoslav Nanev Slavov; Vagner Fonseca; Vincent Louis Viala
EPI_ISL_1574966	American Indian Health Service of Chicago	Illinois Department of Public Health - Chicago Lab	Ira Heimler; Vineet K. Dhiman
EPI_ISL_1121976	Area of Virology, Serology and Virology Division (SAViD), New South Wales Health Pathology Randwick	Virology Research Laboratory; Area of Virology, Serology and Virology Division (SAViD), New South	Au, J.; Bull, R.; Deveson, I.; Foster, C.; Rawlinson, W.; Ruiz Silva, M.; Van Hal, S.

		Wales Health Pathology Randwick	
EPI_ISL_2263452, EPI_ISL_2263453, EPI_ISL_2499835	Arizona State Public Health Laboratory	Arizona State Public Health Laboratory	Jessica Escobar; Katherine Fullerton; Linda Getsinger; Nobuko Fukushima; Stacy White; Trung Huynh; Victor Waddell
EPI_ISL_1743682, EPI_ISL_1743683, EPI_ISL_1743684, EPI_ISL_1743685, EPI_ISL_1743686, EPI_ISL_1910773, EPI_ISL_1910806, EPI_ISL_1910821, EPI_ISL_2007179, EPI_ISL_2153662, EPI_ISL_2716683			
see above	Arizona State University	Arizona State University	Efrem S. Lim; Joshua LaBaer; Joy M. Blain; LaRinda A. Holland; Matthew F. Smith; Nicholas J. Mellor; Peter T. Skidmore; Rabia Maqsood; Valerie Harris; Vel Murugan
EPI_ISL_2105735	Arión Genética	Instituto Nacional de Medicina Genomica	Cedro-Tanda A; Escobar-Arrazola M; Gonzalez-Barrera D; Herrera-Montalvo LA.; Hidalgo-Miranda A; Mendoza-Vargas A; Munguia-Garza P; Ramirez-Vega O; Rangel-DeLeon D; Reyes-Grajeda JP; Roldan-Castillo Magaly; Uribe-Figueroa Laura; Verea Jazmin
EPI_ISL_1365747	Associação Fundo de Incentivo a Pesquisa	Associação Fundo de Incentivo à Pesquisa (AFIP)	Debora Ribeiro Ramadan; Erika Rodrigues de Oliveira; Juliana Nogueira Martins Rodrigues; Priscila Farias Tempaku; Sergio Tufik; Soraya Sgambatti de Andrade
EPI_ISL_1499105	Associação Fundo de Incentivo à Pesquisa (AFIP)	Associação Fundo de Incentivo à Pesquisa (AFIP)	Debora R. Ramadan; Erika Rodrigues de Oliveira; Juliana Nogueira Martins Rodrigues; Priscila Farias Tempaku; Sergio Tufik.; Soraya Sgambatti de Andrade
EPI_ISL_1498380	Associação Fundo de Incentivo à Pesquisa (AFIP).	Associação Fundo de Incentivo à Pesquisa (AFIP)	Debora R. Ramadan; Erika Rodrigues de Oliveira; Juliana Nogueira Martins Rodrigues; Priscila Farias Tempaku; Sergio Tufik.; Soraya Sgambatti de Andrade
EPI_ISL_2423529	Atlanta VA Medical Center	Genomics and Discovery, Respiratory Viruses Branch, Division of Viral Diseases, Centers for Disease Control and Prevention	Adam Retchless; Anna Kelleher; Anna Uehara; Brian Lynch; Clinton R. Paden; Dhwani Batra; Haibin Wang; Han Jia Justin Ng; Jasmine Padilla; Jing Zhang; Justin Lee; Krista Queen; Mark Burroughs; Mili Sheth; Morgan Davis; Peter Cook; Rachel Marine; Sarah Nobles; Suxiang Tong; Tara Coalter; Yan Li; Ying Tao
EPI_ISL_1854228, EPI_ISL_1854244, EPI_ISL_1854245, EPI_ISL_1854246, EPI_ISL_1854247, EPI_ISL_1854248	Avelab	Instituto Nacional de Saude (INSA) and BioSystems & Integrative Sciences Institute (BioISI) Genomics Unit, FCUL	Borges et al
EPI_ISL_3341942	Azienda Ospedaliera Regionale San Carlo Potenza	Laboratorio di patologia clinica e microbiologia A.O.R - San Carlo Potenza	Angela Menchise; Anna Curci; Antonio Picerno; Chiara Autilio; Sara Pascale; Teresa Lopizzo
EPI_ISL_1745318, EPI_ISL_1745320, EPI_ISL_1840710, EPI_ISL_1840728, EPI_ISL_1941565, EPI_ISL_1941574, EPI_ISL_2007319			
see above	Azienda Sanitaria dell'Alto Adige Laboratorio Aziendale di Microbiologia e Virologia	Istituto di Genomica Applicata	Davide Scaglione; Eleonora Paparelli; Elisa Masi; Elisabetta Giacobazzi; Elisabetta Pagani; Gabriele Magris; Irena Jurman; Irene Bianconi; Michele Morgante; Stefanie Wieser; Vera Vendramin
EPI_ISL_2525481, EPI_ISL_2525549, EPI_ISL_2527711, EPI_ISL_2529847, EPI_ISL_2653581, EPI_ISL_2718844	BCCDC Public Health Laboratory	BCCDC Public Health Laboratory	Ana Pacagnella; Corrinne Ng; Dan Fornika; John Tyson; Kim Macdonald; Kimia Kamelian; Linda Hoang; Loretta Janz; Mel Krajden; Prystajecky Natalie; Robert Azana; Shannon Russell
EPI_ISL_1756356	BIO-REFERENCE LABORATORIES	Wadsworth Center, New York State Department of Health	Alexis Russell; Catharine Prussing; Daryl M. Lamson; Erasmus Schneider; Erica Lasek-Nesselquist; John Kelly; Jonathan Plitnick; Kirsten St. George; Matthew Shudt; Melissa A Leisner; Navjot Singh
EPI_ISL_1359892	BIOLAC - SIEGE	CNR Virus des Infections Respiratoires - France SUD	Antonin Bal; Bruno Lina; Gregory Destras; Gwendolyne Burfin; Hadrien Regue; Laurence Josset; Martine Valette; Quentin Semanas
EPI_ISL_1200661, EPI_ISL_1313158, EPI_ISL_1418249, EPI_ISL_1418250	BIOMNIS LYON	CNR Virus des Infections Respiratoires - France SUD	Antonin Bal; Bruno Lina; Gregory Destras; Gwendolyne Burfin; Hadrien Regue; Laurence Josset; Martine Valette; Quentin Semanas
EPI_ISL_1538414	Basurto University Hospital	Biocruces	Ana de la Hoz; Estibaliz Ugalde Zarraga; José Luís Díaz de Tuesta del Arco; Mikel Gallego Rodrigo; Mikel Urrutikoetxea-Gutierrez; Mª Carmen Nieto Toboso
EPI_ISL_1739331	Basurto University Hospital	Biocruces Bizkaia	Ana de la Hoz; Estibaliz Ugalde Zarraga; Jose Luis Díaz de Tuesta del Arco; Mikel Gallego Rodrigo; Mikel Urrutikoetxea-Gutiérrez; Mº Carmen Nieto Toboso
EPI_ISL_1706360	Basurto University Hospital Clinical Microbiology Lab	Biocruces Bizkaia	Ana de la Hoz; Estibaliz Ugalde Zarraga; José Luis Díaz de Tuesta; Mikel Gallego Rodrigo; Mikel Urrutikoetxea-Gutierrez; Mª Carmen Nieto Toboso
EPI_ISL_1706277	Basurto University Hospital Clinical Microbiology Laboratory	Biocruces Bizkaia	Ana de la Hoz; Estibaliz Ugalde Zarraga; José Luis Díaz de Tuesta del Arco.; Mikel Gallego Rodrigo; Mikel Urrutikoetxea-Gutierrez; Mª Carmen Nieto Toboso
EPI_ISL_1538413	Basurto University Hospital Microbiology Lab	Biocruces	Ana de la Hoz; Estibalez Ugalde Zarrraga; Jose Luis Díaz de Tuesta del Arco; Mikel Gallego Rodrigo; Mikel Urrutikoetxea-Gutierrez; Mª Carmen Nieto Toboso
EPI_ISL_1736905, EPI_ISL_1739330	Basurto University Hospital Microbiology Lab	Biocruces Bizkaia	Ana de la Hoz; Estibaliz Ugalde Zarraga; Jose Luis Díaz de Tuesta del Arco; Jose Luís Díaz de Tuesta del Arco; Mikel Gallego Rodrigo; Mikel Urrutikoetxea-Gutierrez; Mº Carmen Nieto Toboso
EPI_ISL_1615556, EPI_ISL_1615557, EPI_ISL_2037414, EPI_ISL_2037443	Basurto University Hospital: Clinical Microbiology Laboratory	Biocruces Bizkaia	Ana de la Hoz; Estibaliz Ugalde Zarraga; José Luis Díaz de Tuesta del Arco; José Luis Díaz de Tuesta del Arco.; Mikel Gallego Rodrigo; Mikel Urrutikoetxea-Gutierrez; Mikel Urrutikoetxea-Gutiérrez; Mª Carmen Nieto Toboso
EPI_ISL_1280655	Bayerisches Landesamt für Gesundheit und Lebensmittelsicherheit (LGL)	Robert Koch Institute	
EPI_ISL_1627453, EPI_ISL_1627454	Baylor Scott & White-Temple	Baylor Scott & White-Temple	Ari Rao; Caitlin Maloney; Kimberly Walker; Linden Morales; Marcus Volz; Shelby Hendrickson
EPI_ISL_1747611	Bioanalytika AG	Clinical Bacteriology	Adrian Egli; Adrian Härri; Alfredo Mari; Hans Hirsch; Helena MB Seth-Smith; Julia Bielicki; Karoline Leuzinger; Madlen Stange; Manuel Battegay; Tim Roloff
EPI_ISL_1756620, EPI_ISL_1757461	Biogroup Bio Lam-LCD Saint-Denis	Department of Virology, Henri Mondor University Hospital, Assistance Publique Hôpitaux de Paris, Université Paris-Est Créteil, INSERM U955	Alexandre Soulier; Christophe Rodriguez; Elisabeth Trawinski; Guillaume Gricourt; Jean-Michel Pawlotsky; Melissa N'Debi; Slim Fourati; Vanessa Demontant
EPI_ISL_1406151, EPI_ISL_1406179, EPI_ISL_1406183, EPI_ISL_4053500, EPI_ISL_4053502, EPI_ISL_4053505, EPI_ISL_4053520, EPI_ISL_4053522			
see above	Biolab Diagnostic Laboratories	Biolab Diagnostic Laboratories	Ahmad Tibi; Amid Abdelnour; Badia Saddedin; Eiad Atwa; Issa Abu-Dayyeh; Lama Hussein; Shaima Ali; Shayma Ali

EPI_ISL_1749270	Biology Lab, HIA PERCY	IRBA, 2MI	CHAPUS C.; DEPEILLE A.; FOISSAUD V.; GORGE O.; GRANDPERRET V.; JARJAVAL F.; NOLENT F.; SARILAR V.; VERGUET N.
EPI_ISL_2698100, EPI_ISL_2698101	Biology, UFLA, Universidade Federal de Lavras	Biology, UFLA	Barcante, J.; Cherem, J.; Fernandes, G.; Luciano, P.; Melo, D.; Pylro, V.
EPI_ISL_2106184	Biology, UFLA, Universidade Federal de Lavras	Biology, UFLA, Universidade Federal de Lavras	Barcante, J.; Cherem, J.; Fernandes, G.; Luciano, P.; Melo, D.; Pylro, V.
EPI_ISL_1917064	BioneXt Lab	Laboratoire national de sante, Microbiology, Microbial Genomics Platform	Anke Wienecke-Baldacchino; Catherine Ragimbeau; Fatu Djabi; Jessica Tapp; Lise Pignon; Raoul Salmon; Tamir Abdelrahman; Thibault Ferrandon
EPI_ISL_3494231, EPI_ISL_3494238, EPI_ISL_3494241	Boston University CTL	Boston University/National Emerging Infectious Disease Laboratories	Catherine Klapperich; Jacquelyn Turcinovic; John H. Connor; Lena Landeverde; Lynn Doucette-Stamm
EPI_ISL_1413799, EPI_ISL_1413803, EPI_ISL_1413805, EPI_ISL_1413813, EPI_ISL_1413823, EPI_ISL_1413829, EPI_ISL_1413837, EPI_ISL_1413840, EPI_ISL_1413842, EPI_ISL_1413845, EPI_ISL_1413851, EPI_ISL_1413856, EPI_ISL_1413859, EPI_ISL_1413861, EPI_ISL_1413864, EPI_ISL_1516491, EPI_ISL_1516492, EPI_ISL_1516498, EPI_ISL_1516700, EPI_ISL_1516701, EPI_ISL_1516720, EPI_ISL_1578342, EPI_ISL_1578346, EPI_ISL_1578347, EPI_ISL_1578348, EPI_ISL_1757215, EPI_ISL_1757235, EPI_ISL_1757243, EPI_ISL_1919016, EPI_ISL_2016413, EPI_ISL_2016415			
see above	Broad Institute Clinical Research Sequencing Platform	Infectious Disease Program, Broad Institute of Harvard and MIT	Adams, G.; B.L.; B.W.; Bauer, M.; Birren; Blumenstiel, B.; Brown, C.; Carter, A.; Chaluvadi, S.; D.J.; DeFelice, M.; DeRuff, K.; Dodge, S.; Gabriel, S.; Gallagher, G.; Gladden-Young, A.; Granger, B.; J.E.; K.J.; Lagerborg, K.; Larkin, K.; Lee, M.; Lemieux; Lennon, N.; Loreth, C.; Madoff, L.; McGovern, S.; Meldrim, J.; Normandin, E.; P.C.; Park; Pearlman, L.; Reilly, S.; Rudy, M.; Sabeti; Siddle; Smole, S.; Tomkins-Tinch, C.; Vicente, G.; and MacInnis
EPI_ISL_1501135, EPI_ISL_1501265	Broussais	HEGP - Laboratoire de Virologie	David Veyer
EPI_ISL_1701128, EPI_ISL_2103132, EPI_ISL_2103137	CA DPH Viral and Rickettsial Disease Laboratory	Chan-Zuckerberg Biohub	CZB Cliahub Consortium
EPI_ISL_1937829	CA-Los Angeles County Public Health Laboratory	Centers for Disease Control and Prevention Division of Viral Diseases, Pathogen Discovery	Alison Laufer Halpin; Ben L. Rambo-Martin; Clinton R. Paden; Dakota Howard; Darlene Wagner; Dave Wentworth; Dhwani Batra; Jasmine Padilla; Justin Lee; Katie Dillon; Krista Queen; Kristen Knipe; Kristine Lacek; Mark Burroughs; Matthew Schmerer; Mili Sheth; Peter Cook; Sam Shepard; Sarah Nobles; Shoshona Le; Suxiang Tong; Vivien Dugan; Yvette Unoarumhi
EPI_ISL_1761303	CAP EL REMEI	Banc de Sang i Teixits	Carlos Hobeich; Francisco Vidal; Irene Corrales; Lorena Ramírez; Maria Glòria Soria; Natàlia Comes; Nina Borràs; Noemí Gonzalez; Silvia Sauleda
EPI_ISL_1761184, EPI_ISL_1761216	CAP SANTA EUGÈNIA DE BERGA	Banc de Sang i Teixits	Carlos Hobeich; Francisco Vidal; Irene Corrales; Lorena Ramírez; Maria Glòria Soria; Natàlia Comes; Nina Borràs; Noemí Gonzalez; Silvia Sauleda
EPI_ISL_2825836, EPI_ISL_2825838, EPI_ISL_2825846, EPI_ISL_2825854, EPI_ISL_2825874, EPI_ISL_2858320, EPI_ISL_2858570, EPI_ISL_2882791, EPI_ISL_2882859, EPI_ISL_2882880, EPI_ISL_2882953, EPI_ISL_2883042, EPI_ISL_2890356, EPI_ISL_2890358, EPI_ISL_2890362, EPI_ISL_2891028, EPI_ISL_2891189			
see above	CDPH VBL	California Department of Public Health	CDPH-COVIDNet; UCLA Technology Center for Genomics & Bioinformatics
EPI_ISL_1798770, EPI_ISL_2018315	CDPH, Viral and Rickettsial Disease Laboratory	Centers for Disease Control and Prevention Division of Viral Diseases, Pathogen Discovery	Alison Laufer Halpin; Ben L. Rambo-Martin; Clinton R. Paden; Dakota Howard; Darlene Wagner; Dave Wentworth; Dhwani Batra; Jasmine Padilla; Justin Lee; Katie Dillon; Krista Queen; Kristen Knipe; Kristine Lacek; Mark Burroughs; Matthew Schmerer; Mili Sheth; Peter Cook; Sam Shepard; Sarah Nobles; Shoshona Le; Suxiang Tong; Vivien Dugan; Yvette Unoarumhi
EPI_ISL_3102254	CENTRAL DE TRANSPLANTES DO CEARA	Analytical Competence Molecular Epidemiology Lab/ACME, Oswaldo Cruz Foundation, Ceara (FIOCRUZ CE)	Cleber Furtado Aksenen; Fabio Miyajima; Fernando Braga Stehling; Francisco Eder de Moura Lopes; Jamille Maria Mendes Bezerra; Joaquim César do Nascimento Sousa Junior; Pedro Miguel Carneiro Jeronimo; Suzana Porto Almeida e Lucas Delerino; Thais Ferreira de Oliveira; Thais de Oliveira Costa; Ticiane Cavalcante de Souza; Veridiana Pessoa Miyajima
EPI_ISL_1966895, EPI_ISL_1966896, EPI_ISL_1966897, EPI_ISL_1966898, EPI_ISL_1966899, EPI_ISL_1966900, EPI_ISL_1966902, EPI_ISL_1966903, EPI_ISL_1966904			
see above	CENTRO DE APOIO EPIDEMIOLOGICO	Instituto Butantan / ESALQ-USP (Piracicaba)	Antonio Jorge Martins; Bianca Cechetto Carlos. Mendelics: Bibiana Santos; Claudia Renata dos Santos Barros; Cíntia Bittar; David Schlesinger. Hemocentro Ribeirão Preto: Simone Kashima; Debora Botequio Moretti; Elaine Cristina Marqueze; Elaine Vieira dos Santos; Elisangela Chicaroni Mattos; Erika Freitas; Evandra Strazza Rodrigues; Felipe Allan da Silva da Costa; Flavia Aburjaile; Fábio Sossai Possebon; Guilherme Campos; Guilherme Targino Valente; Heidge Fukumasu. USP-Botucatu: Rejane Maria Tommasini Grotto; Helena Lage Ferreira; Instituto Butantan: Dimas Tadeu Covas; Jardelina de Souza Todao Bernardino; Jayme A. Souza-Neto; Jessika Cristina Chagas Lesbon; Jorge A. Petroli Marchesi; José Salvatore Leister Patané; João Paulo Kitajima; João Pessoa Araújo Jr.; Leila Sabrina Ullmann; Loyze Paola Oliveira de Lima; Luiz Aurelio de Campos Crispin. Centro de Genômica Funcional da ESALQ: Luiz Lehmann Coutinho; Luiz Carlos Junior de Alcantara; Lívia Sacchetto; Maisa C. Pereira Parra; Maria Carolina Elias; Marta Giovanetti; Marília Moraes; Maurício Lacerda Nogueira. Prefeitura de Sao Paulo: Melissa Palmieri.; Patricia Akemi Assato; Paula Rahal; Paulo Inacio da Costa; Rafael dos Santos Bezerra; Raquel de Lello Rocha Campos Cassano. NGS Soluções Genômicas: Pilar Drummond Sampaio Corrêa Mariani. FZEA-USP Pirassununga: Mirele Daiana Poleti; Raul Machado Neto; Ricardo Augusto Brassaloti; Ricardo Haddad; Rodrigo Tocantins Calado. FAMERP-SJRP: Cecília Artico Banho; Sandra Coccuzzo Sampaio; Svetoslav Nanev Slavov; Vagner Fonseca; Vincent Louis Viala
EPI_ISL_3912133	CENTRO DE ATENDIMENTO COVID 19 TEREZINHA NUNES DA ROCHA	Analytical Competence Molecular Epidemiology Lab/ACME, Oswaldo Cruz Foundation, Ceara (FIOCRUZ CE)	Cleber Furtado Aksenen; Fabio Miyajima; Fernando Braga Stehling; Francisco Eder de Moura Lopes; Jamille Maria Mendes Bezerra; Joaquim Cesar do Nascimento Sousa Junior; Pedro Miguel Carneiro Jeronimo; Suzana Porto Almeida & Lucas Delerino on behalf of COVID-19 FIOCRUZ Genomic Network; Thais Ferreira de Oliveira; Thais de Oliveira Costa; Ticiane Cavalcante de Souza; Veridiana Pessoa Miyajima
EPI_ISL_3102322, EPI_ISL_3102538	CENTRO DE ATENDIMENTO PARA ENFRENTAMENTO AO COVID 19	Analytical Competence Molecular Epidemiology Lab/ACME, Oswaldo Cruz Foundation, Ceara (FIOCRUZ CE)	Cleber Furtado Aksenen; Fabio Miyajima; Fernando Braga Stehling; Francisco Eder de Moura Lopes; Jamille Maria Mendes Bezerra; Joaquim César do Nascimento Sousa Junior; Pedro Miguel Carneiro Jeronimo; Suzana Porto Almeida e Lucas Delerino; Thais Ferreira de Oliveira; Thais de Oliveira Costa; Ticiane Cavalcante de Souza; Veridiana Pessoa Miyajima
EPI_ISL_2344786, EPI_ISL_2344792	CENTRO DE ESPECIALIDADES DE AGUAI AGUAI	Instituto Butantan / ESALQ-Piracicaba	Antonio Jorge Martins; Claudia Renata dos Santos Barros; David Schlesinger; Debora Botequio Moretti; Dimas Tadeu Covas; Elaine Cristina Marqueze; Elaine Vieira Santos; Evandra Strazza Rodrigues; Heidge Fukumasu; Jayme Augusto de Souza-Neto; José Salvatore Leister Patané; Luiz Alcantara; Luiz Lehmann Coutinho; Maria Carolina Elias; Maurício Lacerda Nogueira; Rafael dos Santos Bezerra; Raul Machado Neto; Rejane Maria Tommasini Grotto; Ricardo Haddad; Sandra Coccuzzo Sampaio Vessoni; Simone Kashima; Svetoslav Nanev Slavov; Vincent Louis Viala
EPI_ISL_1795325, EPI_ISL_1795333, EPI_ISL_2345586, EPI_ISL_2345594	CENTRO DE ESPECIALIDADES DE PRIMAVERA	Instituto Butantan / ESALQ-Piracicaba	Antonio Jorge Martins; Bianca Cechetto Carlos. Mendelics: Bibiana Santos; Claudia Renata dos Santos Barros; David Schlesinger; David Schlesinger. Hemocentro Ribeirão Preto: Simone Kashima; Debora Botequio Moretti; Debora Botequio Moretti. Centro de Genômica Funcional da ESALQ: Luiz Lehmann Coutinho; Dimas Tadeu Covas; Elaine Cristina Marqueze; Elaine Vieira Santos; Elaine Vieira dos Santos; Elisangela Chicaroni Mattos; Erika Freitas; Evandra Strazza Rodrigues; Felipe Allan da Silva da Costa; Flavia Aburjaile; Guilherme Targino Valente; Heidge Fukumasu; Heidge Fukumasu. USP-Botucatu: Rejane Maria Tommasini Grotto; Instituto Butantan: Alexander Roberto Precioso; Jayme A. Souza-Neto; Jayme Augusto de Souza-Neto; Jessika Cristina Chagas Lesbon; José Salvatore Leister Patané; João Paulo Kitajima; Luiz Alcantara; Luiz Carlos Junior de Alcantara; Luiz Lehmann Coutinho; Maria Carolina Elias; Marta Giovanetti; Maurício Lacerda Nogueira; Patricia Akemi Assato; Rafael dos Santos Bezerra; Raquel de Lello Rocha Campos Cassano. NGS Soluções Genômicas: Pilar Drummond Sampaio Corrêa Mariani. FZEA-USP Pirassununga: Mirele Daiana Poleti; Raul Machado Neto; Rejane Maria Tommasini Grotto; Ricardo Augusto Brassaloti; Ricardo Haddad; Rodrigo Tocantins Calado.; Sandra Coccuzzo Sampaio; Sandra Coccuzzo Sampaio Vessoni; Simone Kashima; Svetoslav Nanev Slavov; Vagner Fonseca; Vincent Louis Viala
EPI_ISL_3912145	CENTRO DE ESPECIALIDADES MEDICAS DR NESTOR DE PAULA PESSOA	Analytical Competence Molecular Epidemiology Lab/ACME, Oswaldo Cruz Foundation, Ceara (FIOCRUZ CE)	Cleber Furtado Aksenen; Fabio Miyajima; Fernando Braga Stehling; Francisco Eder de Moura Lopes; Jamille Maria Mendes Bezerra; Joaquim Cesar do Nascimento Sousa Junior; Pedro Miguel Carneiro Jeronimo; Suzana Porto Almeida & Lucas Delerino on behalf of COVID-19 FIOCRUZ Genomic Network; Thais Ferreira de Oliveira; Thais de Oliveira Costa; Ticiane Cavalcante de Souza; Veridiana Pessoa Miyajima
EPI_ISL_1795109, EPI_ISL_2344683	CENTRO DE ESPECIALIDDS MEDICAS IRMA LEOPOLDINA PIRASSUNUNGA	Instituto Butantan / ESALQ-Piracicaba	Antonio Jorge Martins; Bianca Cechetto Carlos. Mendelics: Bibiana Santos; Claudia Renata dos Santos Barros; David Schlesinger; David Schlesinger. Hemocentro Ribeirão Preto: Simone Kashima; Debora Botequio Moretti; Debora Botequio Moretti. Centro de Genômica Funcional da ESALQ: Luiz Lehmann Coutinho; Dimas Tadeu Covas; Elaine Cristina Marqueze; Elaine Vieira Santos; Elaine Vieira dos Santos; Elisangela Chicaroni Mattos; Erika Freitas; Evandra Strazza Rodrigues; Felipe Allan da Silva da Costa; Flavia Aburjaile; Guilherme Targino Valente; Heidge Fukumasu; Heidge Fukumasu. USP-Botucatu: Rejane Maria Tommasini Grotto; Instituto Butantan: Alexander Roberto Precioso; Jayme A. Souza-Neto; Jayme Augusto de Souza-Neto; Jessika Cristina Chagas Lesbon; José Salvatore Leister Patané; João Paulo Kitajima; Luiz Alcantara; Luiz Carlos Junior de Alcantara; Luiz Lehmann Coutinho; Maria Carolina Elias; Marta Giovanetti; Maurício Lacerda Nogueira; Patricia Akemi Assato; Rafael dos Santos Bezerra; Raquel de Lello Rocha Campos Cassano. NGS Soluções Genômicas: Pilar Drummond Sampaio Corrêa Mariani. FZEA-USP Pirassununga: Mirele Daiana Poleti; Raul Machado Neto; Rejane Maria Tommasini Grotto; Ricardo Augusto Brassaloti; Ricardo Haddad; Rodrigo Tocantins Calado.; Sandra Coccuzzo Sampaio; Sandra Coccuzzo Sampaio Vessoni; Simone Kashima; Svetoslav Nanev Slavov; Vagner Fonseca; Vincent Louis Viala
EPI_ISL_2344665, EPI_ISL_2344666, EPI_ISL_2344678, EPI_ISL_2344679, EPI_ISL_2344680	CENTRO DE ESPECIALIDDS MEDICAS IRMA LEOPOLDINA PIRASSUNUNGA	Instituto Butantan / FZEA-USP-Pirassununga	Antonio Jorge Martins; Claudia Renata dos Santos Barros; David Schlesinger; Debora Botequio Moretti; Dimas Tadeu Covas; Elaine Cristina Marqueze; Elaine Vieira Santos; Evandra Strazza Rodrigues; Heidge Fukumasu; Jayme Augusto de Souza-Neto; José Salvatore Leister Patané; Luiz Alcantara; Luiz Lehmann Coutinho; Maria Carolina Elias; Maurício Lacerda Nogueira; Rafael dos Santos Bezerra; Raul Machado Neto; Rejane Maria Tommasini Grotto; Ricardo Haddad; Sandra Coccuzzo Sampaio Vessoni; Simone Kashima; Svetoslav Nanev Slavov; Vincent Louis Viala
EPI_ISL_1795228, EPI_ISL_1795229, EPI_ISL_2345459, EPI_ISL_2345460	CENTRO DE REFERENCIA DO IDOSO DR HUMBERTO MENDES DE CARVALHO	Instituto Butantan / ESALQ-Piracicaba	Antonio Jorge Martins; Bianca Cechetto Carlos. Mendelics: Bibiana Santos; Claudia Renata dos Santos Barros; David Schlesinger; David Schlesinger. Hemocentro Ribeirão Preto: Simone Kashima; Debora Botequio Moretti; Debora Botequio Moretti. Centro de Genômica Funcional da ESALQ: Luiz Lehmann Coutinho; Dimas Tadeu Covas; Elaine Cristina Marqueze; Elaine Vieira Santos; Elaine Vieira dos Santos; Elisangela Chicaroni Mattos; Erika Freitas; Evandra Strazza Rodrigues; Felipe Allan da Silva da Costa; Flavia Aburjaile; Guilherme Targino Valente; Heidge Fukumasu; Heidge Fukumasu. USP-Botucatu: Rejane Maria Tommasini Grotto; Instituto Butantan: Alexander Roberto Precioso; Jayme A. Souza-Neto; Jayme Augusto de Souza-Neto; Jessika Cristina Chagas Lesbon; José Salvatore Leister Patané; João Paulo Kitajima; Luiz Alcantara; Luiz Carlos Junior de Alcantara; Luiz Lehmann Coutinho; Maria Carolina Elias; Marta Giovanetti; Maurício Lacerda Nogueira; Patricia Akemi Assato; Rafael dos Santos Bezerra; Raquel de Lello Rocha Campos Cassano. NGS Soluções Genômicas: Pilar Drummond Sampaio Corrêa Mariani. FZEA-USP Pirassununga: Mirele Daiana Poleti; Raul Machado Neto; Rejane Maria Tommasini Grotto; Ricardo Augusto Brassaloti; Ricardo Haddad; Rodrigo Tocantins Calado.; Sandra Coccuzzo Sampaio; Sandra Coccuzzo Sampaio Vessoni; Simone Kashima; Svetoslav Nanev Slavov; Vagner Fonseca; Vincent Louis Viala
EPI_ISL_1966522	CENTRO DE SAUDE DE ADAMANTINA	Instituto Butantan / FZEA-USP (Pirassununga)	Antonio Jorge Martins; Bianca Cechetto Carlos. Mendelics: Bibiana Santos; Claudia Renata dos Santos Barros; Cíntia Bittar; David Schlesinger. Hemocentro Ribeirão Preto: Simone Kashima; Debora Botequio Moretti; Elaine Cristina Marqueze; Elaine Vieira dos Santos; Elisangela Chicaroni Mattos; Erika Freitas; Evandra Strazza Rodrigues; Felipe Allan da Silva da Costa; Flavia Aburjaile; Fábio Sossai Possebon; Guilherme Campos; Guilherme Targino Valente; Heidge Fukumasu. USP-Botucatu: Rejane Maria Tommasini Grotto; Helena Lage Ferreira; Instituto Butantan: Dimas Tadeu Covas; Jardelina de Souza Todao Bernardino; Jayme A. Souza-Neto; Jessika Cristina Chagas Lesbon; Jorge A. Petroli Marchesi; José Salvatore Leister Patané; João Paulo Kitajima; João Pessoa Araújo Jr.; Leila Sabrina Ullmann; Loyze Paola Oliveira de Lima; Luiz Aurelio de Campos Crispin. Centro de Genômica Funcional da ESALQ: Luiz Lehmann Coutinho; Luiz Carlos Junior de Alcantara; Lívia Sacchetto; Maisa C. Pereira Parra; Maria Carolina Elias; Marta Giovanetti; Marília Moraes; Maurício Lacerda Nogueira. Prefeitura de Sao Paulo: Melissa Palmieri.; Patricia Akemi Assato; Paula Rahal; Paulo Inacio da Costa; Rafael dos Santos Bezerra; Raquel de Lello Rocha Campos Cassano. NGS Soluções Genômicas: Pilar Drummond Sampaio Corrêa Mariani. FZEA-USP Pirassununga: Mirele Daiana Poleti; Raul Machado Neto; Ricardo Augusto Brassaloti; Ricardo Haddad; Rodrigo Tocantins Calado. FAMERP-SJRP: Cecília Artico Banho; Sandra Coccuzzo Sampaio; Svetoslav Nanev Slavov; Vagner Fonseca; Vincent Louis Viala

EPI_ISL_2344774	CENTRO DE SAUDE DE CHAVANTES	Instituto Butantan / ESALQ-Piracicaba	Antonio Jorge Martins; Claudia Renata dos Santos Barros; David Schlesinger; Debora Botequio Moretti; Dimas Tadeu Covas; Elaine Cristina Marqueze; Elaine Vieira Santos; Evandra Strazza Rodrigues; Heidge Fukumasu; Jayme Augusto de Souza-Neto; José Salvatore Leister Patané; Luiz Alcantara; Luiz Lehmann Coutinho; Maria Carolina Elias; Maurício Lacerda Nogueira; Rafael dos Santos Bezerra; Raul Machado Neto; Rejane Maria Tommasini Grotto; Ricardo Haddad; Sandra Coccuzzo Sampaio Vessoni; Simone Kashima; Svetoslav Nanev Slavov; Vincent Louis Viala
EPI_ISL_1795167, EPI_ISL_1795168, EPI_ISL_1795169, EPI_ISL_2345378, EPI_ISL_2345379, EPI_ISL_2345380	CENTRO DE SAUDE DE FLORINEA	Instituto Butantan / ESALQ-Piracicaba	Antonio Jorge Martins; Bianca Cechetto Carlos. Mendelics: Bibiana Santos; Claudia Renata dos Santos Barros; David Schlesinger; David Schlesinger. Hemocentro Ribeirão Preto: Simone Kashima; Debora Botequio Moretti; Debora Botequio Moretti. Centro de Genômica Funcional da ESALQ: Luiz Lehmann Coutinho; Dimas Tadeu Covas; Elaine Cristina Marqueze; Elaine Vieira Santos; Elaine Vieira dos Santos; Elisangela Chicaroni Mattos; Erika Freitas; Evandra Strazza Rodrigues; Felipe Allan da Silva da Costa; Flavia Aburjaile; Guilherme Targino Valente; Heidge Fukumasu; Heidge Fukumasu. USP-Botucatu: Rejane Maria Tommasini Grotto; Instituto Butantan: Alexander Roberto Precioso; Jayme A. Souza-Neto; Jayme Augusto de Souza-Neto; Jessika Cristina Chagas Lesbon; José Salvatore Leister Patané; João Paulo Kitajima; Luiz Alcantara; Luiz Carlos Junior de Alcantara; Luiz Lehmann Coutinho; Maria Carolina Elias; Marta Giovanetti; Maurício Lacerda Nogueira; Patricia Akemi Assato; Rafael dos Santos Bezerra; Raquel de Lello Rocha Campos Cassano. NGS Soluções Genômicas: Pilar Drummond Sampaio Corrêa Mariani. FZEA-USP Pirassununga: Mirele Daiana Poleti; Raul Machado Neto; Rejane Maria Tommasini Grotto; Ricardo Augusto Brassaloti; Ricardo Haddad; Rodrigo Tocantins Calado.; Sandra Coccuzzo Sampaio; Sandra Coccuzzo Sampaio Vessoni; Simone Kashima; Svetoslav Nanev Slavov; Vagner Fonseca; Vincent Louis Viala
EPI_ISL_2209396	CENTRO DE SAUDE DE IBIRAREMA	Instituto Butantan / ESALQ-Piracicaba	Antonio Jorge Martins; Claudia Renata dos Santos Barros; David Schlesinger; Debora Botequio Moretti; Dimas Tadeu Covas; Elaine Cristina Marqueze; Elaine Vieira Santos; Evandra Strazza Rodrigues; Heidge Fukumasu; Jayme Augusto de Souza-Neto; José Salvatore Leister Patané; Luiz Alcantara; Luiz Lehmann Coutinho; Maria Carolina Elias; Maurício Lacerda Nogueira; Rafael dos Santos Bezerra; Raul Machado Neto; Rejane Maria Tommasini Grotto; Ricardo Haddad; Sandra Coccuzzo Sampaio Vessoni; Simone Kashima; Svetoslav Nanev Slavov; Vincent Louis Viala
EPI_ISL_2344787	CENTRO DE SAUDE DE LUTECIA	Instituto Butantan / ESALQ-Piracicaba	Antonio Jorge Martins; Claudia Renata dos Santos Barros; David Schlesinger; Debora Botequio Moretti; Dimas Tadeu Covas; Elaine Cristina Marqueze; Elaine Vieira Santos; Evandra Strazza Rodrigues; Heidge Fukumasu; Jayme Augusto de Souza-Neto; José Salvatore Leister Patané; Luiz Alcantara; Luiz Lehmann Coutinho; Maria Carolina Elias; Maurício Lacerda Nogueira; Rafael dos Santos Bezerra; Raul Machado Neto; Rejane Maria Tommasini Grotto; Ricardo Haddad; Sandra Coccuzzo Sampaio Vessoni; Simone Kashima; Svetoslav Nanev Slavov; Vincent Louis Viala
EPI_ISL_1795170, EPI_ISL_1795172, EPI_ISL_2345381, EPI_ISL_2345387	CENTRO DE SAUDE DE MARACAI	Instituto Butantan / ESALQ-Piracicaba	Antonio Jorge Martins; Bianca Cechetto Carlos. Mendelics: Bibiana Santos; Claudia Renata dos Santos Barros; David Schlesinger; David Schlesinger. Hemocentro Ribeirão Preto: Simone Kashima; Debora Botequio Moretti; Debora Botequio Moretti. Centro de Genômica Funcional da ESALQ: Luiz Lehmann Coutinho; Dimas Tadeu Covas; Elaine Cristina Marqueze; Elaine Vieira Santos; Elaine Vieira dos Santos; Elisangela Chicaroni Mattos; Erika Freitas; Evandra Strazza Rodrigues; Felipe Allan da Silva da Costa; Flavia Aburjaile; Guilherme Targino Valente; Heidge Fukumasu; Heidge Fukumasu. USP-Botucatu: Rejane Maria Tommasini Grotto; Instituto Butantan: Alexander Roberto Precioso; Jayme A. Souza-Neto; Jayme Augusto de Souza-Neto; Jessika Cristina Chagas Lesbon; José Salvatore Leister Patané; João Paulo Kitajima; Luiz Alcantara; Luiz Carlos Junior de Alcantara; Luiz Lehmann Coutinho; Maria Carolina Elias; Marta Giovanetti; Maurício Lacerda Nogueira; Patricia Akemi Assato; Rafael dos Santos Bezerra; Raquel de Lello Rocha Campos Cassano. NGS Soluções Genômicas: Pilar Drummond Sampaio Corrêa Mariani. FZEA-USP Pirassununga: Mirele Daiana Poleti; Raul Machado Neto; Rejane Maria Tommasini Grotto; Ricardo Augusto Brassaloti; Ricardo Haddad; Rodrigo Tocantins Calado.; Sandra Coccuzzo Sampaio; Sandra Coccuzzo Sampaio Vessoni; Simone Kashima; Svetoslav Nanev Slavov; Vagner Fonseca; Vincent Louis Viala
EPI_ISL_1966957, EPI_ISL_1966959, EPI_ISL_1966960	CENTRO DE SAUDE DE MARACAI	Instituto Butantan / ESALQ-USP (Piracicaba)	Antonio Jorge Martins; Bianca Cechetto Carlos. Mendelics: Bibiana Santos; Claudia Renata dos Santos Barros; Cíntia Bittar; David Schlesinger. Hemocentro Ribeirão Preto: Simone Kashima; Debora Botequio Moretti; Elaine Cristina Marqueze; Elaine Vieira dos Santos; Elisangela Chicaroni Mattos; Erika Freitas; Evandra Strazza Rodrigues; Felipe Allan da Silva da Costa; Flavia Aburjaile; Fábio Sossai Possebon; Guilherme Campos; Guilherme Targino Valente; Heidge Fukumasu. USP-Botucatu: Rejane Maria Tommasini Grotto; Helena Lage Ferreira; Instituto Butantan: Dimas Tadeu Covas; Jardelina de Souza Todao Bernardino; Jayme A. Souza-Neto; Jessika Cristina Chagas Lesbon; Jorge A. Petroli Marchesi; José Salvatore Leister Patané; João Paulo Kitajima; João Pessoa Araújo Jr.; Leila Sabrina Ullmann; Loyze Paola Oliveira de Lima; Luiz Aurelio de Campos Crispin. Centro de Genômica Funcional da ESALQ: Luiz Lehmann Coutinho; Luiz Carlos Junior de Alcantara; Lívia Sacchetto; Maisa C. Pereira Parra; Maria Carolina Elias; Marta Giovanetti; Marília Moraes; Maurício Lacerda Nogueira. Prefeitura de Sao Paulo: Melissa Palmieri.; Patricia Akemi Assato; Paula Rahal; Paulo Inacio da Costa; Rafael dos Santos Bezerra; Raquel de Lello Rocha Campos Cassano. NGS Soluções Genômicas: Pilar Drummond Sampaio Corrêa Mariani. FZEA-USP Pirassununga: Mirele Daiana Poleti; Raul Machado Neto; Ricardo Augusto Brassaloti; Ricardo Haddad; Rodrigo Tocantins Calado. FAMERP-SJRP: Cecília Artico Banho; Sandra Coccuzzo Sampaio; Svetoslav Nanev Slavov; Vagner Fonseca; Vincent Louis Viala
EPI_ISL_2344785	CENTRO DE SAUDE DE OCAUCU	Instituto Butantan / ESALQ-Piracicaba	Antonio Jorge Martins; Claudia Renata dos Santos Barros; David Schlesinger; Debora Botequio Moretti; Dimas Tadeu Covas; Elaine Cristina Marqueze; Elaine Vieira Santos; Evandra Strazza Rodrigues; Heidge Fukumasu; Jayme Augusto de Souza-Neto; José Salvatore Leister Patané; Luiz Alcantara; Luiz Lehmann Coutinho; Maria Carolina Elias; Maurício Lacerda Nogueira; Rafael dos Santos Bezerra; Raul Machado Neto; Rejane Maria Tommasini Grotto; Ricardo Haddad; Sandra Coccuzzo Sampaio Vessoni; Simone Kashima; Svetoslav Nanev Slavov; Vincent Louis Viala
EPI_ISL_2344791	CENTRO DE SAUDE DE SPEDRO DO TURVO	Instituto Butantan / ESALQ-Piracicaba	Antonio Jorge Martins; Claudia Renata dos Santos Barros; David Schlesinger; Debora Botequio Moretti; Dimas Tadeu Covas; Elaine Cristina Marqueze; Elaine Vieira Santos; Evandra Strazza Rodrigues; Heidge Fukumasu; Jayme Augusto de Souza-Neto; José Salvatore Leister Patané; Luiz Alcantara; Luiz Lehmann Coutinho; Maria Carolina Elias; Maurício Lacerda Nogueira; Rafael dos Santos Bezerra; Raul Machado Neto; Rejane Maria Tommasini Grotto; Ricardo Haddad; Sandra Coccuzzo Sampaio Vessoni; Simone Kashima; Svetoslav Nanev Slavov; Vincent Louis Viala
EPI_ISL_2344744	CENTRO DE SAUDE DO INDAIA DO AGUAPEI FLORIDA PAULISTA	Instituto Butantan / ESALQ-Piracicaba	Antonio Jorge Martins; Claudia Renata dos Santos Barros; David Schlesinger; Debora Botequio Moretti; Dimas Tadeu Covas; Elaine Cristina Marqueze; Elaine Vieira Santos; Evandra Strazza Rodrigues; Heidge Fukumasu; Jayme Augusto de Souza-Neto; José Salvatore Leister Patané; Luiz Alcantara; Luiz Lehmann Coutinho; Maria Carolina Elias; Maurício Lacerda Nogueira; Rafael dos Santos Bezerra; Raul Machado Neto; Rejane Maria Tommasini Grotto; Ricardo Haddad; Sandra Coccuzzo Sampaio Vessoni; Simone Kashima; Svetoslav Nanev Slavov; Vincent Louis Viala
EPI_ISL_3912146, EPI_ISL_3912147	CENTRO DE SAUDE DR EDIJANIR GARCIA DA SILVA	Analytical Competence Molecular Epidemiology Lab/ACME, Oswaldo Cruz Foundation, Ceara (FIOCRUZ CE)	Cleber Furtado Aksenen; Fabio Miyajima; Fernando Braga Stehling; Francisco Eder de Moura Lopes; Jamille Maria Mendes Bezerra; Joaquim Cesar do Nascimento Sousa Junior; Pedro Miguel Carneiro Jeronimo; Suzana Porto Almeida & Lucas Delerino on behalf of COVID-19 FIOCRUZ Genomic Network; Thais Ferreira de Oliveira; Thais de Oliveira Costa; Ticiane Cavalcante de Souza; Veridiana Pessoa Miyajima
EPI_ISL_3912148	CENTRO DE SAUDE DR GUARANY MONTALVERNE	Analytical Competence Molecular Epidemiology Lab/ACME, Oswaldo Cruz Foundation, Ceara (FIOCRUZ CE)	Cleber Furtado Aksenen; Fabio Miyajima; Fernando Braga Stehling; Francisco Eder de Moura Lopes; Jamille Maria Mendes Bezerra; Joaquim Cesar do Nascimento Sousa Junior; Pedro Miguel Carneiro Jeronimo; Suzana Porto Almeida & Lucas Delerino on behalf of COVID-19 FIOCRUZ Genomic Network; Thais Ferreira de Oliveira; Thais de Oliveira Costa; Ticiane Cavalcante de Souza; Veridiana Pessoa Miyajima
EPI_ISL_2209265, EPI_ISL_2344710	CENTRO DE SAUDE DR MARIO DIAS DE AGUIAR CAPIVARI	Instituto Butantan / FZEA-USP-Pirassununga	Antonio Jorge Martins; Claudia Renata dos Santos Barros; David Schlesinger; Debora Botequio Moretti; Dimas Tadeu Covas; Elaine Cristina Marqueze; Elaine Vieira Santos; Evandra Strazza Rodrigues; Heidge Fukumasu; Jayme Augusto de Souza-Neto; José Salvatore Leister Patané; Luiz Alcantara; Luiz Lehmann Coutinho; Maria Carolina Elias; Maurício Lacerda Nogueira; Rafael dos Santos Bezerra; Raul Machado Neto; Rejane Maria Tommasini Grotto; Ricardo Haddad; Sandra Coccuzzo Sampaio Vessoni; Simone Kashima; Svetoslav Nanev Slavov; Vincent Louis Viala
EPI_ISL_3912149	CENTRO DE SAUDE DR MIRANDA TAVARES	Analytical Competence Molecular Epidemiology Lab/ACME, Oswaldo Cruz Foundation, Ceara (FIOCRUZ CE)	Cleber Furtado Aksenen; Fabio Miyajima; Fernando Braga Stehling; Francisco Eder de Moura Lopes; Jamille Maria Mendes Bezerra; Joaquim Cesar do Nascimento Sousa Junior; Pedro Miguel Carneiro Jeronimo; Suzana Porto Almeida & Lucas Delerino on behalf of COVID-19 FIOCRUZ Genomic Network; Thais Ferreira de Oliveira; Thais de Oliveira Costa; Ticiane Cavalcante de Souza; Veridiana Pessoa Miyajima
EPI_ISL_2344797	CENTRO DE SAUDE DR NELSON DA CUNHA BASTOS	Instituto Butantan / ESALQ-Piracicaba	Antonio Jorge Martins; Claudia Renata dos Santos Barros; David Schlesinger; Debora Botequio Moretti; Dimas Tadeu Covas; Elaine Cristina Marqueze; Elaine Vieira Santos; Evandra Strazza Rodrigues; Heidge Fukumasu; Jayme Augusto de Souza-Neto; José Salvatore Leister Patané; Luiz Alcantara; Luiz Lehmann Coutinho; Maria Carolina Elias; Maurício Lacerda Nogueira; Rafael dos Santos Bezerra; Raul Machado Neto; Rejane Maria Tommasini Grotto; Ricardo Haddad; Sandra Coccuzzo Sampaio Vessoni; Simone Kashima; Svetoslav Nanev Slavov; Vincent Louis Viala
EPI_ISL_2208950	CENTRO DE SAUDE DR RENATO DE CARVALHO RIBEIRO ANGATUBA	Instituto Butantan	Antonio Jorge Martins; Claudia Renata dos Santos Barros; David Schlesinger; Debora Botequio Moretti; Dimas Tadeu Covas; Elaine Cristina Marqueze; Elaine Vieira Santos; Evandra Strazza Rodrigues; Heidge Fukumasu; Jayme Augusto de Souza-Neto; José Salvatore Leister Patané; Luiz Alcantara; Luiz Lehmann Coutinho; Maria Carolina Elias; Maurício Lacerda Nogueira; Rafael dos Santos Bezerra; Raul Machado Neto; Rejane Maria Tommasini Grotto; Ricardo Haddad; Sandra Coccuzzo Sampaio Vessoni; Simone Kashima; Svetoslav Nanev Slavov; Vincent Louis Viala.
EPI_ISL_1966824, EPI_ISL_1966825, EPI_ISL_1966826, EPI_ISL_1966827, EPI_ISL_1966828, EPI_ISL_1966829	CENTRO DE SAUDE DR RENATO DE CARVALHO RIBEIRO ANGATUBA	Instituto Butantan / ESALQ-USP (Piracicaba)	Antonio Jorge Martins; Bianca Cechetto Carlos. Mendelics: Bibiana Santos; Claudia Renata dos Santos Barros; Cíntia Bittar; David Schlesinger. Hemocentro Ribeirão Preto: Simone Kashima; Debora Botequio Moretti; Elaine Cristina Marqueze; Elaine Vieira dos Santos; Elisangela Chicaroni Mattos; Erika Freitas; Evandra Strazza Rodrigues; Felipe Allan da Silva da Costa; Flavia Aburjaile; Fábio Sossai Possebon; Guilherme Campos; Guilherme Targino Valente; Heidge Fukumasu. USP-Botucatu: Rejane Maria Tommasini Grotto; Helena Lage Ferreira; Instituto Butantan: Dimas Tadeu Covas; Jardelina de Souza Todao Bernardino; Jayme A. Souza-Neto; Jessika Cristina Chagas Lesbon; Jorge A. Petroli Marchesi; José Salvatore Leister Patané; João Paulo Kitajima; João Pessoa Araújo Jr.; Leila Sabrina Ullmann; Loyze Paola Oliveira de Lima; Luiz Aurelio de Campos Crispin. Centro de Genômica Funcional da ESALQ: Luiz Lehmann Coutinho; Luiz Carlos Junior de Alcantara; Lívia Sacchetto; Maisa C. Pereira Parra; Maria Carolina Elias; Marta Giovanetti; Marília Moraes; Maurício Lacerda Nogueira. Prefeitura de Sao Paulo: Melissa Palmieri.; Patricia Akemi Assato; Paula Rahal; Paulo Inacio da Costa; Rafael dos Santos Bezerra; Raquel de Lello Rocha Campos Cassano. NGS Soluções Genômicas: Pilar Drummond Sampaio Corrêa Mariani. FZEA-USP Pirassununga: Mirele Daiana Poleti; Raul Machado Neto; Ricardo Augusto Brassaloti; Ricardo Haddad; Rodrigo Tocantins Calado. FAMERP-SJRP: Cecília Artico Banho; Sandra Coccuzzo Sampaio; Svetoslav Nanev Slavov; Vagner Fonseca; Vincent Louis Viala
EPI_ISL_2344587	CENTRO DE SAUDE DR RENATO DE CARVALHO RIBEIRO ANGATUBA	Instituto Butantan / UNESP-Botucatu	Antonio Jorge Martins; Claudia Renata dos Santos Barros; David Schlesinger; Debora Botequio Moretti; Dimas Tadeu Covas; Elaine Cristina Marqueze; Elaine Vieira Santos; Evandra Strazza Rodrigues; Heidge Fukumasu; Jayme Augusto de Souza-Neto; José Salvatore Leister Patané; Luiz Alcantara; Luiz Lehmann Coutinho; Maria Carolina Elias; Maurício Lacerda Nogueira; Rafael dos Santos Bezerra; Raul Machado Neto; Rejane Maria Tommasini Grotto; Ricardo Haddad; Sandra Coccuzzo Sampaio Vessoni; Simone Kashima; Svetoslav Nanev Slavov; Vincent Louis Viala
EPI_ISL_2344779	CENTRO DE SAUDE ESF IV ZONA RURAL DOMINGOS DE S SJRIOPARDO	Instituto Butantan / ESALQ-Piracicaba	Antonio Jorge Martins; Claudia Renata dos Santos Barros; David Schlesinger; Debora Botequio Moretti; Dimas Tadeu Covas; Elaine Cristina Marqueze; Elaine Vieira Santos; Evandra Strazza Rodrigues; Heidge Fukumasu; Jayme Augusto de Souza-Neto; José Salvatore Leister Patané; Luiz Alcantara; Luiz Lehmann Coutinho; Maria Carolina Elias; Maurício Lacerda Nogueira; Rafael dos Santos Bezerra; Raul Machado Neto; Rejane Maria Tommasini Grotto; Ricardo Haddad; Sandra Coccuzzo Sampaio Vessoni; Simone Kashima; Svetoslav Nanev Slavov; Vincent Louis Viala
EPI_ISL_1967059	CENTRO DE SAUDE ESF IV ZONA RURAL DOMINGOS DE S SJRIOPARDO	Instituto Butantan / ESALQ-USP (Piracicaba)	Antonio Jorge Martins; Bianca Cechetto Carlos. Mendelics: Bibiana Santos; Claudia Renata dos Santos Barros; Cíntia Bittar; David Schlesinger. Hemocentro Ribeirão Preto: Simone Kashima; Debora Botequio Moretti; Elaine Cristina Marqueze; Elaine Vieira dos Santos; Elisangela Chicaroni Mattos; Erika Freitas; Evandra Strazza Rodrigues; Felipe Allan da Silva da Costa; Flavia Aburjaile; Fábio Sossai Possebon; Guilherme Campos; Guilherme Targino Valente; Heidge Fukumasu. USP-Botucatu: Rejane Maria Tommasini Grotto; Helena Lage Ferreira; Instituto Butantan: Dimas Tadeu Covas; Jardelina de Souza Todao Bernardino; Jayme A. Souza-Neto; Jessika Cristina Chagas Lesbon; Jorge A. Petroli Marchesi; José Salvatore Leister Patané; João Paulo Kitajima; João Pessoa Araújo Jr.; Leila Sabrina Ullmann; Loyze Paola Oliveira de Lima; Luiz Aurelio de Campos Crispin. Centro de Genômica Funcional da ESALQ: Luiz Lehmann Coutinho; Luiz Carlos Junior de Alcantara; Lívia Sacchetto; Maisa C. Pereira Parra; Maria Carolina Elias; Marta Giovanetti; Marília Moraes; Maurício Lacerda Nogueira. Prefeitura de Sao Paulo: Melissa Palmieri.; Patricia Akemi Assato; Paula Rahal; Paulo Inacio da Costa; Rafael dos Santos Bezerra; Raquel de Lello Rocha Campos Cassano. NGS Soluções Genômicas: Pilar Drummond Sampaio Corrêa Mariani. FZEA-USP Pirassununga: Mirele Daiana Poleti; Raul Machado Neto; Ricardo Augusto Brassaloti; Ricardo Haddad; Rodrigo Tocantins Calado. FAMERP-SJRP: Cecília Artico Banho; Sandra Coccuzzo Sampaio; Svetoslav Nanev Slavov; Vagner Fonseca; Vincent Louis Viala
EPI_ISL_1966536, EPI_ISL_1966538, EPI_ISL_1966539, EPI_ISL_1966540	CENTRO DE SAUDE I TACITO LEITE DE CARVALHO E SILVA	Instituto Butantan / FZEA-USP (Pirassununga)	Antonio Jorge Martins; Bianca Cechetto Carlos. Mendelics: Bibiana Santos; Claudia Renata dos Santos Barros; Cíntia Bittar; David Schlesinger. Hemocentro Ribeirão Preto: Simone Kashima; Debora Botequio Moretti; Elaine Cristina Marqueze; Elaine Vieira dos Santos; Elisangela Chicaroni Mattos; Erika Freitas; Evandra Strazza Rodrigues; Felipe Allan da Silva da Costa; Flavia Aburjaile; Fábio Sossai Possebon; Guilherme Campos; Guilherme Targino Valente; Heidge Fukumasu. USP-Botucatu: Rejane Maria Tommasini Grotto; Helena Lage Ferreira; Instituto Butantan: Dimas Tadeu Covas; Jardelina de Souza Todao Bernardino; Jayme A. Souza-Neto; Jessika Cristina Chagas Lesbon; Jorge A. Petroli Marchesi; José Salvatore Leister Patané; João Paulo Kitajima; João Pessoa Araújo Jr.; Leila Sabrina Ullmann; Loyze Paola Oliveira de Lima; Luiz Aurelio de Campos Crispin. Centro de Genômica Funcional da ESALQ: Luiz Lehmann Coutinho; Luiz Carlos Junior de Alcantara; Lívia Sacchetto; Maisa C. Pereira Parra; Maria Carolina Elias; Marta Giovanetti; Marília Moraes; Maurício Lacerda Nogueira. Prefeitura de Sao Paulo: Melissa Palmieri.; Patricia Akemi Assato; Paula Rahal; Paulo Inacio da Costa; Rafael dos Santos Bezerra; Raquel de Lello Rocha Campos Cassano. NGS Soluções Genômicas: Pilar Drummond Sampaio Corrêa Mariani. FZEA-USP Pirassununga: Mirele Daiana Poleti; Raul Machado Neto; Ricardo Augusto Brassaloti; Ricardo Haddad; Rodrigo Tocantins Calado. FAMERP-SJRP: Cecília Artico Banho; Sandra Coccuzzo Sampaio; Svetoslav Nanev Slavov; Vagner Fonseca; Vincent Louis Viala
EPI_ISL_1795212, EPI_ISL_2345440	CENTRO DE SAUDE II DR ALCIDES FACUNDO ARROYO	Instituto Butantan / ESALQ-Piracicaba	Antonio Jorge Martins; Bianca Cechetto Carlos. Mendelics: Bibiana Santos; Claudia Renata dos Santos Barros; David Schlesinger; David Schlesinger. Hemocentro Ribeirão Preto: Simone Kashima; Debora Botequio Moretti; Debora Botequio Moretti. Centro de Genômica Funcional da ESALQ: Luiz Lehmann Coutinho; Dimas Tadeu Covas; Elaine Cristina Marqueze; Elaine Vieira Santos; Elaine Vieira dos Santos; Elisangela Chicaroni Mattos; Erika Freitas; Evandra Strazza Rodrigues; Felipe Allan da Silva da Costa; Flavia Aburjaile; Guilherme Targino Valente; Heidge Fukumasu; Heidge Fukumasu. USP-Botucatu: Rejane Maria Tommasini Grotto; Instituto Butantan: Alexander Roberto Precioso; Jayme A. Souza-Neto; Jayme Augusto de Souza-Neto; Jessika Cristina Chagas Lesbon; José Salvatore Leister Patané; João Paulo Kitajima; Luiz Alcantara; Luiz Carlos Junior de Alcantara; Luiz Lehmann Coutinho; Maria Carolina Elias; Marta Giovanetti; Maurício Lacerda Nogueira; Patricia Akemi Assato; Rafael dos Santos Bezerra; Raquel de Lello Rocha Campos Cassano. NGS Soluções Genômicas: Pilar Drummond Sampaio Corrêa Mariani. FZEA-USP Pirassununga: Mirele Daiana Poleti; Raul Machado Neto; Rejane Maria Tommasini Grotto; Ricardo Augusto Brassaloti; Ricardo Haddad; Rodrigo Tocantins Calado.; Sandra Coccuzzo Sampaio; Sandra Coccuzzo Sampaio Vessoni; Simone Kashima; Svetoslav Nanev Slavov; Vagner Fonseca; Vincent Louis Viala
EPI_ISL_1795327, EPI_ISL_1795331, EPI_ISL_2345588, EPI_ISL_2345592	CENTRO DE SAUDE II JUNQUEIROPOLIS	Instituto Butantan / ESALQ-Piracicaba	Antonio Jorge Martins; Bianca Cechetto Carlos. Mendelics: Bibiana Santos; Claudia Renata dos Santos Barros; David Schlesinger; David Schlesinger. Hemocentro Ribeirão Preto: Simone Kashima; Debora Botequio Moretti; Debora Botequio Moretti. Centro de Genômica Funcional da ESALQ: Luiz Lehmann Coutinho; Dimas Tadeu Covas; Elaine Cristina Marqueze; Elaine Vieira Santos; Elaine Vieira dos Santos; Elisangela Chicaroni Mattos; Erika Freitas; Evandra Strazza Rodrigues; Felipe Allan da Silva da Costa; Flavia Aburjaile; Guilherme Targino Valente; Heidge Fukumasu; Heidge Fukumasu. USP-Botucatu: Rejane Maria Tommasini Grotto; Instituto Butantan: Alexander Roberto Precioso; Jayme A. Souza-Neto; Jayme Augusto de Souza-Neto; Jessika Cristina Chagas Lesbon; José Salvatore Leister Patané; João Paulo Kitajima; Luiz Alcantara; Luiz Carlos Junior de Alcantara; Luiz Lehmann Coutinho; Maria Carolina Elias; Marta Giovanetti; Maurício Lacerda Nogueira; Patricia Akemi Assato; Rafael dos Santos Bezerra; Raquel de Lello Rocha Campos Cassano. NGS Soluções Genômicas: Pilar Drummond Sampaio Corrêa Mariani. FZEA-USP Pirassununga: Mirele Daiana Poleti; Raul Machado Neto; Rejane Maria Tommasini Grotto; Ricardo Augusto Brassaloti; Ricardo Haddad; Rodrigo Tocantins Calado.; Sandra Coccuzzo Sampaio; Sandra Coccuzzo Sampaio Vessoni; Simone Kashima; Svetoslav Nanev Slavov; Vagner Fonseca; Vincent Louis Viala
EPI_ISL_1966955	CENTRO DE SAUDE II MAIRINQUE MAIRINQUE	Instituto Butantan / ESALQ-USP (Piracicaba)	Antonio Jorge Martins; Bianca Cechetto Carlos. Mendelics: Bibiana Santos; Claudia Renata dos Santos Barros; Cíntia Bittar; David Schlesinger. Hemocentro Ribeirão Preto: Simone Kashima; Debora Botequio Moretti; Elaine Cristina Marqueze; Elaine Vieira dos Santos; Elisangela Chicaroni Mattos; Erika Freitas; Evandra Strazza Rodrigues; Felipe Allan da Silva da Costa; Flavia Aburjaile; Fábio Sossai Possebon; Guilherme Campos; Guilherme Targino Valente; Heidge Fukumasu. USP-Botucatu: Rejane Maria Tommasini Grotto; Helena Lage Ferreira; Instituto Butantan: Dimas Tadeu Covas; Jardelina de Souza Todao Bernardino; Jayme A. Souza-Neto; Jessika Cristina Chagas Lesbon; Jorge A. Petroli Marchesi; José Salvatore Leister Patané; João Paulo Kitajima; João Pessoa Araújo Jr.; Leila Sabrina Ullmann; Loyze Paola Oliveira de Lima; Luiz Aurelio de Campos Crispin. Centro de Genômica Funcional da ESALQ: Luiz Lehmann Coutinho; Luiz Carlos Junior de Alcantara; Lívia Sacchetto; Maisa C. Pereira Parra; Maria Carolina Elias; Marta Giovanetti; Marília Moraes; Maurício Lacerda Nogueira. Prefeitura de Sao Paulo: Melissa Palmieri.; Patricia Akemi Assato; Paula Rahal; Paulo Inacio da Costa; Rafael dos Santos Bezerra; Raquel de Lello Rocha Campos Cassano. NGS Soluções Genômicas: Pilar Drummond Sampaio Corrêa Mariani. FZEA-USP Pirassununga: Mirele Daiana Poleti; Raul Machado Neto; Ricardo Augusto Brassaloti; Ricardo Haddad; Rodrigo Tocantins Calado. FAMERP-SJRP: Cecília Artico Banho; Sandra Coccuzzo Sampaio; Svetoslav Nanev Slavov; Vagner Fonseca; Vincent Louis Viala
EPI_ISL_1967062, EPI_ISL_1967063, EPI_ISL_1967064	CENTRO DE SAUDE II SAO MIGUEL ARCANJO	Instituto Butantan / ESALQ-USP (Piracicaba)	Antonio Jorge Martins; Bianca Cechetto Carlos. Mendelics: Bibiana Santos; Claudia Renata dos Santos Barros; Cíntia Bittar; David Schlesinger. Hemocentro Ribeirão Preto: Simone Kashima; Debora Botequio Moretti; Elaine Cristina Marqueze; Elaine Vieira dos Santos; Elisangela Chicaroni Mattos; Erika Freitas; Evandra Strazza Rodrigues; Felipe Allan da Silva da Costa; Flavia Aburjaile; Fábio Sossai Possebon; Guilherme Campos; Guilherme Targino Valente; Heidge Fukumasu. USP-Botucatu: Rejane Maria Tommasini Grotto; Helena Lage Ferreira; Instituto Butantan: Dimas Tadeu Covas; Jardelina de Souza Todao Bernardino; Jayme A. Souza-Neto; Jessika Cristina Chagas Lesbon; Jorge A. Petroli Marchesi; José Salvatore Leister Patané; João Paulo Kitajima; João Pessoa Araújo Jr.; Leila Sabrina Ullmann; Loyze Paola Oliveira de Lima; Luiz Aurelio de Campos Crispin. Centro de Genômica Funcional da ESALQ: Luiz Lehmann Coutinho; Luiz Carlos Junior de Alcantara; Lívia Sacchetto; Maisa C. Pereira Parra; Maria Carolina Elias; Marta Giovanetti; Marília Moraes; Maurício Lacerda Nogueira. Prefeitura de Sao Paulo: Melissa Palmieri.; Patricia Akemi Assato; Paula Rahal; Paulo Inacio da Costa; Rafael dos Santos Bezerra; Raquel de Lello Rocha Campos Cassano. NGS Soluções Genômicas: Pilar Drummond Sampaio Corrêa Mariani. FZEA-USP Pirassununga: Mirele Daiana Poleti; Raul Machado Neto; Ricardo Augusto Brassaloti; Ricardo Haddad; Rodrigo Tocantins Calado. FAMERP-SJRP: Cecília Artico Banho; Sandra Coccuzzo Sampaio; Svetoslav Nanev Slavov; Vagner Fonseca; Vincent Louis Viala

EPI_ISL_1795081, EPI_ISL_1795297, EPI_ISL_1795299, EPI_ISL_2345539, EPI_ISL_2345542, EPI_ISL_2345546	CENTRO DE SAUDE III AFFONSO LUZZI SANTA CRUZ DAS PALMEIRAS	Instituto Butantan / ESALQ-Piracicaba	Antonio Jorge Martins; Bianca Cechetto Carlos. Mendelics: Bibiana Santos; Claudia Renata dos Santos Barros; David Schlesinger; David Schlesinger. Hemocentro Ribeirão Preto: Simone Kashima; Debora Botequio Moretti; Debora Botequio Moretti. Centro de Genômica Funcional da ESALQ: Luiz Lehmann Coutinho; Dimas Tadeu Covas; Elaine Cristina Marqueze; Elaine Vieira Santos; Elaine Vieira dos Santos; Elisangela Chicaroni Mattos; Erika Freitas; Evandra Strazza Rodrigues; Felipe Allan da Silva da Costa; Flavia Aburjaile; Guilherme Targino Valente; Heidge Fukumasu; Heidge Fukumasu. USP-Botucatu: Rejane Maria Tommasini Grotto; Instituto Butantan: Alexander Roberto Precioso; Jayme A. Souza-Neto; Jayme Augusto de Souza-Neto; Jessika Cristina Chagas Lesbon; José Salvatore Leister Patané; João Paulo Kitajima; Luiz Alcantara; Luiz Carlos Junior de Alcantara; Luiz Lehmann Coutinho; Maria Carolina Elias; Marta Giovanetti; Maurício Lacerda Nogueira; Patricia Akemi Assato; Rafael dos Santos Bezerra; Raquel de Lello Rocha Campos Cassano. NGS Soluções Genômicas: Pilar Drummond Sampaio Corrêa Mariani. FZEA-USP Pirassununga: Mirele Daiana Poleti; Raul Machado Neto; Rejane Maria Tommasini Grotto; Ricardo Augusto Brassaloti; Ricardo Haddad; Rodrigo Tocantins Calado.; Sandra Coccuzzo Sampaio; Sandra Coccuzzo Sampaio Vessoni; Simone Kashima; Svetoslav Nanev Slavov; Vagner Fonseca; Vincent Louis Viala
EPI_ISL_2344567	CENTRO DE SAUDE III AFFONSO LUZZI SANTA CRUZ DAS PALMEIRAS	Instituto Butantan / FZEA-USP-Pirassununga	Antonio Jorge Martins; Claudia Renata dos Santos Barros; David Schlesinger; Debora Botequio Moretti; Dimas Tadeu Covas; Elaine Cristina Marqueze; Elaine Vieira Santos; Evandra Strazza Rodrigues; Heidge Fukumasu; Jayme Augusto de Souza-Neto; José Salvatore Leister Patané; Luiz Alcantara; Luiz Lehmann Coutinho; Maria Carolina Elias; Maurício Lacerda Nogueira; Rafael dos Santos Bezerra; Raul Machado Neto; Rejane Maria Tommasini Grotto; Ricardo Haddad; Sandra Coccuzzo Sampaio Vessoni; Simone Kashima; Svetoslav Nanev Slavov; Vincent Louis Viala
EPI_ISL_1795114, EPI_ISL_1795115, EPI_ISL_2345313, EPI_ISL_2345314	CENTRO DE SAUDE III BORBOREMA	Instituto Butantan / ESALQ-Piracicaba	Antonio Jorge Martins; Bianca Cechetto Carlos. Mendelics: Bibiana Santos; Claudia Renata dos Santos Barros; David Schlesinger; David Schlesinger. Hemocentro Ribeirão Preto: Simone Kashima; Debora Botequio Moretti; Debora Botequio Moretti. Centro de Genômica Funcional da ESALQ: Luiz Lehmann Coutinho; Dimas Tadeu Covas; Elaine Cristina Marqueze; Elaine Vieira Santos; Elaine Vieira dos Santos; Elisangela Chicaroni Mattos; Erika Freitas; Evandra Strazza Rodrigues; Felipe Allan da Silva da Costa; Flavia Aburjaile; Guilherme Targino Valente; Heidge Fukumasu; Heidge Fukumasu. USP-Botucatu: Rejane Maria Tommasini Grotto; Instituto Butantan: Alexander Roberto Precioso; Jayme A. Souza-Neto; Jayme Augusto de Souza-Neto; Jessika Cristina Chagas Lesbon; José Salvatore Leister Patané; João Paulo Kitajima; Luiz Alcantara; Luiz Carlos Junior de Alcantara; Luiz Lehmann Coutinho; Maria Carolina Elias; Marta Giovanetti; Maurício Lacerda Nogueira; Patricia Akemi Assato; Rafael dos Santos Bezerra; Raquel de Lello Rocha Campos Cassano. NGS Soluções Genômicas: Pilar Drummond Sampaio Corrêa Mariani. FZEA-USP Pirassununga: Mirele Daiana Poleti; Raul Machado Neto; Rejane Maria Tommasini Grotto; Ricardo Augusto Brassaloti; Ricardo Haddad; Rodrigo Tocantins Calado.; Sandra Coccuzzo Sampaio; Sandra Coccuzzo Sampaio Vessoni; Simone Kashima; Svetoslav Nanev Slavov; Vagner Fonseca; Vincent Louis Viala
EPI_ISL_1795125, EPI_ISL_1795127, EPI_ISL_2345327, EPI_ISL_2345329	CENTRO DE SAUDE III CANDIDO RODRIGUES	Instituto Butantan / ESALQ-Piracicaba	Antonio Jorge Martins; Bianca Cechetto Carlos. Mendelics: Bibiana Santos; Claudia Renata dos Santos Barros; David Schlesinger; David Schlesinger. Hemocentro Ribeirão Preto: Simone Kashima; Debora Botequio Moretti; Debora Botequio Moretti. Centro de Genômica Funcional da ESALQ: Luiz Lehmann Coutinho; Dimas Tadeu Covas; Elaine Cristina Marqueze; Elaine Vieira Santos; Elaine Vieira dos Santos; Elisangela Chicaroni Mattos; Erika Freitas; Evandra Strazza Rodrigues; Felipe Allan da Silva da Costa; Flavia Aburjaile; Guilherme Targino Valente; Heidge Fukumasu; Heidge Fukumasu. USP-Botucatu: Rejane Maria Tommasini Grotto; Instituto Butantan: Alexander Roberto Precioso; Jayme A. Souza-Neto; Jayme Augusto de Souza-Neto; Jessika Cristina Chagas Lesbon; José Salvatore Leister Patané; João Paulo Kitajima; Luiz Alcantara; Luiz Carlos Junior de Alcantara; Luiz Lehmann Coutinho; Maria Carolina Elias; Marta Giovanetti; Maurício Lacerda Nogueira; Patricia Akemi Assato; Rafael dos Santos Bezerra; Raquel de Lello Rocha Campos Cassano. NGS Soluções Genômicas: Pilar Drummond Sampaio Corrêa Mariani. FZEA-USP Pirassununga: Mirele Daiana Poleti; Raul Machado Neto; Rejane Maria Tommasini Grotto; Ricardo Augusto Brassaloti; Ricardo Haddad; Rodrigo Tocantins Calado.; Sandra Coccuzzo Sampaio; Sandra Coccuzzo Sampaio Vessoni; Simone Kashima; Svetoslav Nanev Slavov; Vagner Fonseca; Vincent Louis Viala
EPI_ISL_2344689	CENTRO DE SAUDE III DE AGUAS DE SAO PEDRO	Instituto Butantan / FZEA-USP-Pirassununga	Antonio Jorge Martins; Claudia Renata dos Santos Barros; David Schlesinger; Debora Botequio Moretti; Dimas Tadeu Covas; Elaine Cristina Marqueze; Elaine Vieira Santos; Evandra Strazza Rodrigues; Heidge Fukumasu; Jayme Augusto de Souza-Neto; José Salvatore Leister Patané; Luiz Alcantara; Luiz Lehmann Coutinho; Maria Carolina Elias; Maurício Lacerda Nogueira; Rafael dos Santos Bezerra; Raul Machado Neto; Rejane Maria Tommasini Grotto; Ricardo Haddad; Sandra Coccuzzo Sampaio Vessoni; Simone Kashima; Svetoslav Nanev Slavov; Vincent Louis Viala
EPI_ISL_1795126, EPI_ISL_2345328	CENTRO DE SAUDE III SALES OLIVEIRA	Instituto Butantan / ESALQ-Piracicaba	Antonio Jorge Martins; Bianca Cechetto Carlos. Mendelics: Bibiana Santos; Claudia Renata dos Santos Barros; David Schlesinger; David Schlesinger. Hemocentro Ribeirão Preto: Simone Kashima; Debora Botequio Moretti; Debora Botequio Moretti. Centro de Genômica Funcional da ESALQ: Luiz Lehmann Coutinho; Dimas Tadeu Covas; Elaine Cristina Marqueze; Elaine Vieira Santos; Elaine Vieira dos Santos; Elisangela Chicaroni Mattos; Erika Freitas; Evandra Strazza Rodrigues; Felipe Allan da Silva da Costa; Flavia Aburjaile; Guilherme Targino Valente; Heidge Fukumasu; Heidge Fukumasu. USP-Botucatu: Rejane Maria Tommasini Grotto; Instituto Butantan: Alexander Roberto Precioso; Jayme A. Souza-Neto; Jayme Augusto de Souza-Neto; Jessika Cristina Chagas Lesbon; José Salvatore Leister Patané; João Paulo Kitajima; Luiz Alcantara; Luiz Carlos Junior de Alcantara; Luiz Lehmann Coutinho; Maria Carolina Elias; Marta Giovanetti; Maurício Lacerda Nogueira; Patricia Akemi Assato; Rafael dos Santos Bezerra; Raquel de Lello Rocha Campos Cassano. NGS Soluções Genômicas: Pilar Drummond Sampaio Corrêa Mariani. FZEA-USP Pirassununga: Mirele Daiana Poleti; Raul Machado Neto; Rejane Maria Tommasini Grotto; Ricardo Augusto Brassaloti; Ricardo Haddad; Rodrigo Tocantins Calado.; Sandra Coccuzzo Sampaio; Sandra Coccuzzo Sampaio Vessoni; Simone Kashima; Svetoslav Nanev Slavov; Vagner Fonseca; Vincent Louis Viala
EPI_ISL_1966345	CENTRO DE SAUDE III TABATINGA	Instituto Butantan / Mendelics	Antonio Jorge Martins; Bianca Cechetto Carlos. Mendelics: Bibiana Santos; Claudia Renata dos Santos Barros; Cíntia Bittar; David Schlesinger. Hemocentro Ribeirão Preto: Simone Kashima; Debora Botequio Moretti; Elaine Cristina Marqueze; Elaine Vieira dos Santos; Elisangela Chicaroni Mattos; Erika Freitas; Evandra Strazza Rodrigues; Felipe Allan da Silva da Costa; Flavia Aburjaile; Fábio Sossai Possebon; Guilherme Campos; Guilherme Targino Valente; Heidge Fukumasu. USP-Botucatu: Rejane Maria Tommasini Grotto; Helena Lage Ferreira; Instituto Butantan: Dimas Tadeu Covas; Jardelina de Souza Todao Bernardino; Jayme A. Souza-Neto; Jessika Cristina Chagas Lesbon; Jorge A. Petroli Marchesi; José Salvatore Leister Patané; João Paulo Kitajima; João Pessoa Araújo Jr.; Leila Sabrina Ullmann; Loyze Paola Oliveira de Lima; Luiz Aurelio de Campos Crispin. Centro de Genômica Funcional da ESALQ: Luiz Lehmann Coutinho; Luiz Carlos Junior de Alcantara; Lívia Sacchetto; Maisa C. Pereira Parra; Maria Carolina Elias; Marta Giovanetti; Marília Moraes; Maurício Lacerda Nogueira. Prefeitura de Sao Paulo: Melissa Palmieri.; Patricia Akemi Assato; Paula Rahal; Paulo Inacio da Costa; Rafael dos Santos Bezerra; Raquel de Lello Rocha Campos Cassano. NGS Soluções Genômicas: Pilar Drummond Sampaio Corrêa Mariani. FZEA-USP Pirassununga: Mirele Daiana Poleti; Raul Machado Neto; Ricardo Augusto Brassaloti; Ricardo Haddad; Rodrigo Tocantins Calado. FAMERP-SJRP: Cecília Artico Banho; Sandra Coccuzzo Sampaio; Svetoslav Nanev Slavov; Vagner Fonseca; Vincent Louis Viala
EPI_ISL_2344735	CENTRO DE SAUDE PE CESAR CORNEO	Instituto Butantan / ESALQ-Piracicaba	Antonio Jorge Martins; Claudia Renata dos Santos Barros; David Schlesinger; Debora Botequio Moretti; Dimas Tadeu Covas; Elaine Cristina Marqueze; Elaine Vieira Santos; Evandra Strazza Rodrigues; Heidge Fukumasu; Jayme Augusto de Souza-Neto; José Salvatore Leister Patané; Luiz Alcantara; Luiz Lehmann Coutinho; Maria Carolina Elias; Maurício Lacerda Nogueira; Rafael dos Santos Bezerra; Raul Machado Neto; Rejane Maria Tommasini Grotto; Ricardo Haddad; Sandra Coccuzzo Sampaio Vessoni; Simone Kashima; Svetoslav Nanev Slavov; Vincent Louis Viala
EPI_ISL_1967096, EPI_ISL_1967097	CENTRO DE SAUDE SAO ROQUE DR JOSE CARVALHO BRITO	Instituto Butantan / ESALQ-USP (Piracicaba)	Antonio Jorge Martins; Bianca Cechetto Carlos. Mendelics: Bibiana Santos; Claudia Renata dos Santos Barros; Cíntia Bittar; David Schlesinger. Hemocentro Ribeirão Preto: Simone Kashima; Debora Botequio Moretti; Elaine Cristina Marqueze; Elaine Vieira dos Santos; Elisangela Chicaroni Mattos; Erika Freitas; Evandra Strazza Rodrigues; Felipe Allan da Silva da Costa; Flavia Aburjaile; Fábio Sossai Possebon; Guilherme Campos; Guilherme Targino Valente; Heidge Fukumasu. USP-Botucatu: Rejane Maria Tommasini Grotto; Helena Lage Ferreira; Instituto Butantan: Dimas Tadeu Covas; Jardelina de Souza Todao Bernardino; Jayme A. Souza-Neto; Jessika Cristina Chagas Lesbon; Jorge A. Petroli Marchesi; José Salvatore Leister Patané; João Paulo Kitajima; João Pessoa Araújo Jr.; Leila Sabrina Ullmann; Loyze Paola Oliveira de Lima; Luiz Aurelio de Campos Crispin. Centro de Genômica Funcional da ESALQ: Luiz Lehmann Coutinho; Luiz Carlos Junior de Alcantara; Lívia Sacchetto; Maisa C. Pereira Parra; Maria Carolina Elias; Marta Giovanetti; Marília Moraes; Maurício Lacerda Nogueira. Prefeitura de Sao Paulo: Melissa Palmieri.; Patricia Akemi Assato; Paula Rahal; Paulo Inacio da Costa; Rafael dos Santos Bezerra; Raquel de Lello Rocha Campos Cassano. NGS Soluções Genômicas: Pilar Drummond Sampaio Corrêa Mariani. FZEA-USP Pirassununga: Mirele Daiana Poleti; Raul Machado Neto; Ricardo Augusto Brassaloti; Ricardo Haddad; Rodrigo Tocantins Calado. FAMERP-SJRP: Cecília Artico Banho; Sandra Coccuzzo Sampaio; Svetoslav Nanev Slavov; Vagner Fonseca; Vincent Louis Viala
EPI_ISL_2344749, EPI_ISL_2344752, EPI_ISL_2344757, EPI_ISL_2344759	CENTRO DE TRIAGEM COVID 19	Instituto Butantan / ESALQ-Piracicaba	Antonio Jorge Martins; Claudia Renata dos Santos Barros; David Schlesinger; Debora Botequio Moretti; Dimas Tadeu Covas; Elaine Cristina Marqueze; Elaine Vieira Santos; Evandra Strazza Rodrigues; Heidge Fukumasu; Jayme Augusto de Souza-Neto; José Salvatore Leister Patané; Luiz Alcantara; Luiz Lehmann Coutinho; Maria Carolina Elias; Maurício Lacerda Nogueira; Rafael dos Santos Bezerra; Raul Machado Neto; Rejane Maria Tommasini Grotto; Ricardo Haddad; Sandra Coccuzzo Sampaio Vessoni; Simone Kashima; Svetoslav Nanev Slavov; Vincent Louis Viala
EPI_ISL_2344690	CENTRO MEDICO DR NELSON SALOME DE CONCHAL	Instituto Butantan / FZEA-USP-Pirassununga	Antonio Jorge Martins; Claudia Renata dos Santos Barros; David Schlesinger; Debora Botequio Moretti; Dimas Tadeu Covas; Elaine Cristina Marqueze; Elaine Vieira Santos; Evandra Strazza Rodrigues; Heidge Fukumasu; Jayme Augusto de Souza-Neto; José Salvatore Leister Patané; Luiz Alcantara; Luiz Lehmann Coutinho; Maria Carolina Elias; Maurício Lacerda Nogueira; Rafael dos Santos Bezerra; Raul Machado Neto; Rejane Maria Tommasini Grotto; Ricardo Haddad; Sandra Coccuzzo Sampaio Vessoni; Simone Kashima; Svetoslav Nanev Slavov; Vincent Louis Viala
EPI_ISL_1795274, EPI_ISL_1795275, EPI_ISL_2345510, EPI_ISL_2345513	CENTRO MEDICO PMESP	Instituto Butantan / ESALQ-Piracicaba	Antonio Jorge Martins; Bianca Cechetto Carlos. Mendelics: Bibiana Santos; Claudia Renata dos Santos Barros; David Schlesinger; David Schlesinger. Hemocentro Ribeirão Preto: Simone Kashima; Debora Botequio Moretti; Debora Botequio Moretti. Centro de Genômica Funcional da ESALQ: Luiz Lehmann Coutinho; Dimas Tadeu Covas; Elaine Cristina Marqueze; Elaine Vieira Santos; Elaine Vieira dos Santos; Elisangela Chicaroni Mattos; Erika Freitas; Evandra Strazza Rodrigues; Felipe Allan da Silva da Costa; Flavia Aburjaile; Guilherme Targino Valente; Heidge Fukumasu; Heidge Fukumasu. USP-Botucatu: Rejane Maria Tommasini Grotto; Instituto Butantan: Alexander Roberto Precioso; Jayme A. Souza-Neto; Jayme Augusto de Souza-Neto; Jessika Cristina Chagas Lesbon; José Salvatore Leister Patané; João Paulo Kitajima; Luiz Alcantara; Luiz Carlos Junior de Alcantara; Luiz Lehmann Coutinho; Maria Carolina Elias; Marta Giovanetti; Maurício Lacerda Nogueira; Patricia Akemi Assato; Rafael dos Santos Bezerra; Raquel de Lello Rocha Campos Cassano. NGS Soluções Genômicas: Pilar Drummond Sampaio Corrêa Mariani. FZEA-USP Pirassununga: Mirele Daiana Poleti; Raul Machado Neto; Rejane Maria Tommasini Grotto; Ricardo Augusto Brassaloti; Ricardo Haddad; Rodrigo Tocantins Calado.; Sandra Coccuzzo Sampaio; Sandra Coccuzzo Sampaio Vessoni; Simone Kashima; Svetoslav Nanev Slavov; Vagner Fonseca; Vincent Louis Viala
EPI_ISL_2209074	CENTRO MUNICIPAL DE EPIDEMIOLOGIA E IMUNIZACOES LIMEIRA	Instituto Butantan	Antonio Jorge Martins; Claudia Renata dos Santos Barros; David Schlesinger; Debora Botequio Moretti; Dimas Tadeu Covas; Elaine Cristina Marqueze; Elaine Vieira Santos; Evandra Strazza Rodrigues; Heidge Fukumasu; Jayme Augusto de Souza-Neto; José Salvatore Leister Patané; Luiz Alcantara; Luiz Lehmann Coutinho; Maria Carolina Elias; Maurício Lacerda Nogueira; Rafael dos Santos Bezerra; Raul Machado Neto; Rejane Maria Tommasini Grotto; Ricardo Haddad; Sandra Coccuzzo Sampaio Vessoni; Simone Kashima; Svetoslav Nanev Slavov; Vincent Louis Viala.
EPI_ISL_2209982	CENTRO MUNICIPAL DE EPIDEMIOLOGIA E IMUNIZACOES LIMEIRA	Instituto Butantan / FZEA-USP-Pirassununga	Antonio Jorge Martins; Claudia Renata dos Santos Barros; David Schlesinger; Debora Botequio Moretti; Dimas Tadeu Covas; Elaine Cristina Marqueze; Elaine Vieira Santos; Evandra Strazza Rodrigues; Heidge Fukumasu; Jayme Augusto de Souza-Neto; José Salvatore Leister Patané; Luiz Alcantara; Luiz Lehmann Coutinho; Maria Carolina Elias; Maurício Lacerda Nogueira; Rafael dos Santos Bezerra; Raul Machado Neto; Rejane Maria Tommasini Grotto; Ricardo Haddad; Sandra Coccuzzo Sampaio Vessoni; Simone Kashima; Svetoslav Nanev Slavov; Vincent Louis Viala
EPI_ISL_1265833	CERBALLIANCE PACA	CNR Virus des Infections Respiratoires - France SUD	Antonin Bal; Bruno Lina; Gregory Destras; Gwendolyne Burfin; Hadrien Regue; Laurence Josset; Martine Valette; Quentin Semanas
EPI_ISL_1526351	CH ANTIBES - JUAN LES PINS	CNR Virus des Infections Respiratoires - France SUD	Antonin Bal; Bruno Lina; Gregory Destras; Gwendolyne Burfin; Hadrien Regue; Laurence Josset; Martine Valette; Quentin Semanas
EPI_ISL_2562029, EPI_ISL_2562030	CH Franck Joly	Cerba lab	Aude Lessenne; Bénédicte Roquebert; Emmanuel Lecorche; Kader Merah; Laura Verdurme; Patrice Herisson; Sabine Trombert-Paolantoni; Stéphanie Haïm-Boukobza; Thierry Collin
EPI_ISL_1399630	CH Porto - H Sto Antonio	Instituto Nacional de Saude (INSA) and BioSystems & Integrative Sciences Institute (BioISI) Genomics Unit, FCUL	Borges et al
EPI_ISL_2226343, EPI_ISL_2226354, EPI_ISL_2226358	CH SAINTONGE	CHU Poitiers	Agnes BEBY-DEFAUX; Birama N'DIAYE; Caroline MICHAUD; Magali GARCIA; Manon PRAT; Maxime PICHON; Nicolas LEVEQUE; Valentin BON-BARET
EPI_ISL_1593466, EPI_ISL_1628339, EPI_ISL_1628340, EPI_ISL_1628342	CH de l'Ouest Guyanais	Institut Pasteur de la Guyane	A. Salmier; Anne Lavergne; Dominique Rousset
EPI_ISL_1085358, EPI_ISL_1085912, EPI_ISL_1672684, EPI_ISL_1672741, EPI_ISL_1706628, EPI_ISL_1706945, EPI_ISL_1706948, EPI_ISL_1757696			
see above	CH.INTERCOMMUNAL DE CRETEIL	Department of Virology, Henri Mondor University Hospital, Assistance Publique Hôpitaux de Paris, Université Paris-Est Créteil, INSERM U955	Alexandre Soulier; Christophe Rodriguez; Elisabeth Trawinski; Guillaume Gricourt; Jean-Michel Pawlotsky; Melissa N'Debi; Slim Fourati; Vanessa Demontant
EPI_ISL_1593463, EPI_ISL_1593464, EPI_ISL_1593980, EPI_ISL_1627097, EPI_ISL_1627102, EPI_ISL_1627104, EPI_ISL_1627105, EPI_ISL_1628334, EPI_ISL_1628335, EPI_ISL_1628343, EPI_ISL_2227910			
see above	CHC Andrée Rosemon	Institut Pasteur de la Guyane	A. Salmier; Anne Lavergne; Dominique Rousset
EPI_ISL_1738809, EPI_ISL_1739049	CHLC	Instituto Nacional de Saude (INSA)	Borges et al
EPI_ISL_1171967	CHLN	Instituto Nacional de Saude (INSA)	Borges et al
EPI_ISL_2194032	CHU Nantes Virology	CHU Nantes Virology	Audrey Rodallec; Berthe-Marie Imbert-Marcille; Celine Bressollette-Bodin; Thomas Drumel
EPI_ISL_1399631, EPI_ISL_1399632	CHU Sao Joao, Porto	Instituto Nacional de Saude (INSA) and Instituto Gulbenkian de	Borges et al

		Ciencia (IGC)	
EPI_ISL_1854234, EPI_ISL_1854235, EPI_ISL_1854236	CHULN - H Santa Maria	Instituto Nacional de Saude (INSA)	Borges et al
EPI_ISL_1963930, EPI_ISL_1963933, EPI_ISL_1963936	CLILAB	Microbiology Department	Aida Gonzalez-Diaz; Carmen Ardanuy; Jordi Camara; Jordi Niubó; Laura Calatayud; M Angeles Domínguez; Miguel Fernandez-Huerta; Sara Marti
EPI_ISL_3912150	CLINICA ESCOLA DE SAUDE	Analytical Competence Molecular Epidemiology Lab/ACME, Oswaldo Cruz Foundation, Ceara (FIOCRUZ CE)	Cleber Furtado Aksenen; Fabio Miyajima; Fernando Braga Stehling; Francisco Eder de Moura Lopes; Jamille Maria Mendes Bezerra; Joaquim Cesar do Nascimento Sousa Junior; Pedro Miguel Carneiro Jeronimo; Suzana Porto Almeida & Lucas Delerino on behalf of COVID-19 FIOCRUZ Genomic Network; Thais Ferreira de Oliveira; Thais de Oliveira Costa; Ticiane Cavalcante de Souza; Veridiana Pessoa Miyajima
EPI_ISL_1593470, EPI_ISL_1593471, EPI_ISL_1627100, EPI_ISL_1627101, EPI_ISL_1628336, EPI_ISL_1628337, EPI_ISL_1628338, EPI_ISL_2227911, EPI_ISL_2227912, EPI_ISL_2227913, EPI_ISL_2227914, EPI_ISL_2227915, EPI_ISL_2227917, EPI_ISL_2227918			
see above	CNR Institut Pasteur de la Guyane	Institut Pasteur de la Guyane	A. Salmier; Anne Lavergne; Dominique Rousset
EPI_ISL_1418235, EPI_ISL_1695421	CNR Virus des Infections Respiratoires - France SUD	CNR Virus des Infections Respiratoires - France SUD	Antonin Bal; Bruno Lina; Gregory Destras; Gwendolyne Burfin; Hadrien Regue; Laurence Josset; Martine Valette; Quentin Semanas
EPI_ISL_2383912	CO Dept. of Public Health and Environment, Lab Services Division	Centers for Disease Control and Prevention Division of Viral Diseases, Pathogen Discovery	Alison Laufer Halpin; Ben L. Rambo-Martin; Clinton R. Paden; Dakota Howard; Darlene Wagner; Dave Wentworth; Dhwani Batra; Jasmine Padilla; Justin Lee; Katie Dillon; Krista Queen; Kristen Knipe; Kristine Lacek; Mark Burroughs; Matthew Schmerer; Mili Sheth; Peter Cook; Sam Shepard; Sarah Nobles; Shoshona Le; Suxiang Tong; Vivien Dugan; Yvette Unoarumhi
EPI_ISL_1445109	COMPLEXO HOSPITALAR OURO VERDE DE CAMPINAS	Instituto Butantan / Mendelics	Antonio Jorge Martins; Bibiana Santos; Claudia Renata dos Santos Barros; David Schlesinger; Debora Botequio Moretti; Dimas Tadeu Covas; Elaine Cristina Marqueze; Elaine Vieira dos Santos; Erika Freitas; Evandra Strazza Rodrigues; Flavia Aburjaile; José Salvatore Leister Patané; João Paulo Kitajima; Luiz Carlos Junior de Alcantara; Maria Carolina Elias; Marta Giovanetti; Rafael dos Santos Bezerra; Raul Machado Neto; Ricardo Haddad; Rodrigo Tocantins Calado.; Sandra Coccuzzo Sampaio; Simone Kashima; Svetoslav Nanev Slavov; Vagner Fonseca; Vincent Louis Viala
EPI_ISL_2209080	COORDENADORIA MUNICIPAL DE SAUDE DE IRACEMAPOLIS	Instituto Butantan	Antonio Jorge Martins; Claudia Renata dos Santos Barros; David Schlesinger; Debora Botequio Moretti; Dimas Tadeu Covas; Elaine Cristina Marqueze; Elaine Vieira Santos; Evandra Strazza Rodrigues; Heidge Fukumasu; Jayme Augusto de Souza-Neto; José Salvatore Leister Patané; Luiz Alcantara; Luiz Lehmann Coutinho; Maria Carolina Elias; Maurício Lacerda Nogueira; Rafael dos Santos Bezerra; Raul Machado Neto; Rejane Maria Tommasini Grotto; Ricardo Haddad; Sandra Coccuzzo Sampaio Vessoni; Simone Kashima; Svetoslav Nanev Slavov; Vincent Louis Viala.
EPI_ISL_1795107, EPI_ISL_2344660	COORDENADORIA MUNICIPAL DE SAUDE DE IRACEMAPOLIS	Instituto Butantan / ESALQ-Piracicaba	Antonio Jorge Martins; Bianca Cechetto Carlos. Mendelics: Bibiana Santos; Claudia Renata dos Santos Barros; David Schlesinger; David Schlesinger. Hemocentro Ribeirão Preto: Simone Kashima; Debora Botequio Moretti; Debora Botequio Moretti. Centro de Genômica Funcional da ESALQ: Luiz Lehmann Coutinho; Dimas Tadeu Covas; Elaine Cristina Marqueze; Elaine Vieira Santos; Elaine Vieira dos Santos; Elisangela Chicaroni Mattos; Erika Freitas; Evandra Strazza Rodrigues; Felipe Allan da Silva da Costa; Flavia Aburjaile; Guilherme Targino Valente; Heidge Fukumasu; Heidge Fukumasu. USP-Botucatu: Rejane Maria Tommasini Grotto; Instituto Butantan: Alexander Roberto Precioso; Jayme A. Souza-Neto; Jayme Augusto de Souza-Neto; Jessika Cristina Chagas Lesbon; José Salvatore Leister Patané; João Paulo Kitajima; Luiz Alcantara; Luiz Carlos Junior de Alcantara; Luiz Lehmann Coutinho; Maria Carolina Elias; Marta Giovanetti; Maurício Lacerda Nogueira; Patricia Akemi Assato; Rafael dos Santos Bezerra; Raquel de Lello Rocha Campos Cassano. NGS Soluções Genômicas: Pilar Drummond Sampaio Corrêa Mariani. FZEA-USP Pirassununga: Mirele Daiana Poleti; Raul Machado Neto; Rejane Maria Tommasini Grotto; Ricardo Augusto Brassaloti; Ricardo Haddad; Rodrigo Tocantins Calado.; Sandra Coccuzzo Sampaio; Sandra Coccuzzo Sampaio Vessoni; Simone Kashima; Svetoslav Nanev Slavov; Vagner Fonseca; Vincent Louis Viala
EPI_ISL_2209275, EPI_ISL_2344685, EPI_ISL_2344702	COORDENADORIA MUNICIPAL DE SAUDE DE IRACEMAPOLIS	Instituto Butantan / FZEA-USP-Pirassununga	Antonio Jorge Martins; Claudia Renata dos Santos Barros; David Schlesinger; Debora Botequio Moretti; Dimas Tadeu Covas; Elaine Cristina Marqueze; Elaine Vieira Santos; Evandra Strazza Rodrigues; Heidge Fukumasu; Jayme Augusto de Souza-Neto; José Salvatore Leister Patané; Luiz Alcantara; Luiz Lehmann Coutinho; Maria Carolina Elias; Maurício Lacerda Nogueira; Rafael dos Santos Bezerra; Raul Machado Neto; Rejane Maria Tommasini Grotto; Ricardo Haddad; Sandra Coccuzzo Sampaio Vessoni; Simone Kashima; Svetoslav Nanev Slavov; Vincent Louis Viala
EPI_ISL_1795234, EPI_ISL_1795235, EPI_ISL_1795236, EPI_ISL_1795237, EPI_ISL_1795238, EPI_ISL_1795241, EPI_ISL_1795389, EPI_ISL_2345466, EPI_ISL_2345467, EPI_ISL_2345468, EPI_ISL_2345469, EPI_ISL_2345470, EPI_ISL_2345473, EPI_ISL_2345475			
see above	CS DE BALSAMO	Instituto Butantan / ESALQ-Piracicaba	Antonio Jorge Martins; Bianca Cechetto Carlos. Mendelics: Bibiana Santos; Claudia Renata dos Santos Barros; David Schlesinger; David Schlesinger. Hemocentro Ribeirão Preto: Simone Kashima; Debora Botequio Moretti; Debora Botequio Moretti. Centro de Genômica Funcional da ESALQ: Luiz Lehmann Coutinho; Dimas Tadeu Covas; Elaine Cristina Marqueze; Elaine Vieira Santos; Elaine Vieira dos Santos; Elisangela Chicaroni Mattos; Erika Freitas; Evandra Strazza Rodrigues; Felipe Allan da Silva da Costa; Flavia Aburjaile; Guilherme Targino Valente; Heidge Fukumasu; Heidge Fukumasu. USP-Botucatu: Rejane Maria Tommasini Grotto; Instituto Butantan: Alexander Roberto Precioso; Jayme A. Souza-Neto; Jayme Augusto de Souza-Neto; Jessika Cristina Chagas Lesbon; José Salvatore Leister Patané; João Paulo Kitajima; Luiz Alcantara; Luiz Carlos Junior de Alcantara; Luiz Lehmann Coutinho; Maria Carolina Elias; Marta Giovanetti; Maurício Lacerda Nogueira; Patricia Akemi Assato; Rafael dos Santos Bezerra; Raquel de Lello Rocha Campos Cassano. NGS Soluções Genômicas: Pilar Drummond Sampaio Corrêa Mariani. FZEA-USP Pirassununga: Mirele Daiana Poleti; Raul Machado Neto; Rejane Maria Tommasini Grotto; Ricardo Augusto Brassaloti; Ricardo Haddad; Rodrigo Tocantins Calado.; Sandra Coccuzzo Sampaio; Sandra Coccuzzo Sampaio Vessoni; Simone Kashima; Svetoslav Nanev Slavov; Vagner Fonseca; Vincent Louis Viala
EPI_ISL_1966834	CS DE BALSAMO	Instituto Butantan / ESALQ-USP (Piracicaba)	Antonio Jorge Martins; Bianca Cechetto Carlos. Mendelics: Bibiana Santos; Claudia Renata dos Santos Barros; Cíntia Bittar; David Schlesinger. Hemocentro Ribeirão Preto: Simone Kashima; Debora Botequio Moretti; Elaine Cristina Marqueze; Elaine Vieira dos Santos; Elisangela Chicaroni Mattos; Erika Freitas; Evandra Strazza Rodrigues; Felipe Allan da Silva da Costa; Flavia Aburjaile; Fábio Sossai Possebon; Guilherme Campos; Guilherme Targino Valente; Heidge Fukumasu. USP-Botucatu: Rejane Maria Tommasini Grotto; Helena Lage Ferreira; Instituto Butantan: Dimas Tadeu Covas; Jardelina de Souza Todao Bernardino; Jayme A. Souza-Neto; Jessika Cristina Chagas Lesbon; Jorge A. Petroli Marchesi; José Salvatore Leister Patané; João Paulo Kitajima; João Pessoa Araújo Jr.; Leila Sabrina Ullmann; Loyze Paola Oliveira de Lima; Luiz Aurelio de Campos Crispin. Centro de Genômica Funcional da ESALQ: Luiz Lehmann Coutinho; Luiz Carlos Junior de Alcantara; Lívia Sacchetto; Maisa C. Pereira Parra; Maria Carolina Elias; Marta Giovanetti; Marília Moraes; Maurício Lacerda Nogueira. Prefeitura de Sao Paulo: Melissa Palmieri.; Patricia Akemi Assato; Paula Rahal; Paulo Inacio da Costa; Rafael dos Santos Bezerra; Raquel de Lello Rocha Campos Cassano. NGS Soluções Genômicas: Pilar Drummond Sampaio Corrêa Mariani. FZEA-USP Pirassununga: Mirele Daiana Poleti; Raul Machado Neto; Ricardo Augusto Brassaloti; Ricardo Haddad; Rodrigo Tocantins Calado. FAMERP-SJRP: Cecília Artico Banho; Sandra Coccuzzo Sampaio; Svetoslav Nanev Slavov; Vagner Fonseca; Vincent Louis Viala
EPI_ISL_1966524	CS DE BALSAMO	Instituto Butantan / FZEA-USP (Pirassununga)	Antonio Jorge Martins; Bianca Cechetto Carlos. Mendelics: Bibiana Santos; Claudia Renata dos Santos Barros; Cíntia Bittar; David Schlesinger. Hemocentro Ribeirão Preto: Simone Kashima; Debora Botequio Moretti; Elaine Cristina Marqueze; Elaine Vieira dos Santos; Elisangela Chicaroni Mattos; Erika Freitas; Evandra Strazza Rodrigues; Felipe Allan da Silva da Costa; Flavia Aburjaile; Fábio Sossai Possebon; Guilherme Campos; Guilherme Targino Valente; Heidge Fukumasu. USP-Botucatu: Rejane Maria Tommasini Grotto; Helena Lage Ferreira; Instituto Butantan: Dimas Tadeu Covas; Jardelina de Souza Todao Bernardino; Jayme A. Souza-Neto; Jessika Cristina Chagas Lesbon; Jorge A. Petroli Marchesi; José Salvatore Leister Patané; João Paulo Kitajima; João Pessoa Araújo Jr.; Leila Sabrina Ullmann; Loyze Paola Oliveira de Lima; Luiz Aurelio de Campos Crispin. Centro de Genômica Funcional da ESALQ: Luiz Lehmann Coutinho; Luiz Carlos Junior de Alcantara; Lívia Sacchetto; Maisa C. Pereira Parra; Maria Carolina Elias; Marta Giovanetti; Marília Moraes; Maurício Lacerda Nogueira. Prefeitura de Sao Paulo: Melissa Palmieri.; Patricia Akemi Assato; Paula Rahal; Paulo Inacio da Costa; Rafael dos Santos Bezerra; Raquel de Lello Rocha Campos Cassano. NGS Soluções Genômicas: Pilar Drummond Sampaio Corrêa Mariani. FZEA-USP Pirassununga: Mirele Daiana Poleti; Raul Machado Neto; Ricardo Augusto Brassaloti; Ricardo Haddad; Rodrigo Tocantins Calado. FAMERP-SJRP: Cecília Artico Banho; Sandra Coccuzzo Sampaio; Svetoslav Nanev Slavov; Vagner Fonseca; Vincent Louis Viala
EPI_ISL_1966868, EPI_ISL_1966869	CS DE CATIGUA	Instituto Butantan / ESALQ-USP (Piracicaba)	Antonio Jorge Martins; Bianca Cechetto Carlos. Mendelics: Bibiana Santos; Claudia Renata dos Santos Barros; Cíntia Bittar; David Schlesinger. Hemocentro Ribeirão Preto: Simone Kashima; Debora Botequio Moretti; Elaine Cristina Marqueze; Elaine Vieira dos Santos; Elisangela Chicaroni Mattos; Erika Freitas; Evandra Strazza Rodrigues; Felipe Allan da Silva da Costa; Flavia Aburjaile; Fábio Sossai Possebon; Guilherme Campos; Guilherme Targino Valente; Heidge Fukumasu. USP-Botucatu: Rejane Maria Tommasini Grotto; Helena Lage Ferreira; Instituto Butantan: Dimas Tadeu Covas; Jardelina de Souza Todao Bernardino; Jayme A. Souza-Neto; Jessika Cristina Chagas Lesbon; Jorge A. Petroli Marchesi; José Salvatore Leister Patané; João Paulo Kitajima; João Pessoa Araújo Jr.; Leila Sabrina Ullmann; Loyze Paola Oliveira de Lima; Luiz Aurelio de Campos Crispin. Centro de Genômica Funcional da ESALQ: Luiz Lehmann Coutinho; Luiz Carlos Junior de Alcantara; Lívia Sacchetto; Maisa C. Pereira Parra; Maria Carolina Elias; Marta Giovanetti; Marília Moraes; Maurício Lacerda Nogueira. Prefeitura de Sao Paulo: Melissa Palmieri.; Patricia Akemi Assato; Paula Rahal; Paulo Inacio da Costa; Rafael dos Santos Bezerra; Raquel de Lello Rocha Campos Cassano. NGS Soluções Genômicas: Pilar Drummond Sampaio Corrêa Mariani. FZEA-USP Pirassununga: Mirele Daiana Poleti; Raul Machado Neto; Ricardo Augusto Brassaloti; Ricardo Haddad; Rodrigo Tocantins Calado. FAMERP-SJRP: Cecília Artico Banho; Sandra Coccuzzo Sampaio; Svetoslav Nanev Slavov; Vagner Fonseca; Vincent Louis Viala
EPI_ISL_1795259, EPI_ISL_1795260, EPI_ISL_1795262, EPI_ISL_1795263, EPI_ISL_2345493, EPI_ISL_2345494, EPI_ISL_2345496, EPI_ISL_2345497			
see above	CS DE ICEM	Instituto Butantan / ESALQ-Piracicaba	Antonio Jorge Martins; Bianca Cechetto Carlos. Mendelics: Bibiana Santos; Claudia Renata dos Santos Barros; David Schlesinger; David Schlesinger. Hemocentro Ribeirão Preto: Simone Kashima; Debora Botequio Moretti; Debora Botequio Moretti. Centro de Genômica Funcional da ESALQ: Luiz Lehmann Coutinho; Dimas Tadeu Covas; Elaine Cristina Marqueze; Elaine Vieira Santos; Elaine Vieira dos Santos; Elisangela Chicaroni Mattos; Erika Freitas; Evandra Strazza Rodrigues; Felipe Allan da Silva da Costa; Flavia Aburjaile; Guilherme Targino Valente; Heidge Fukumasu; Heidge Fukumasu. USP-Botucatu: Rejane Maria Tommasini Grotto; Instituto Butantan: Alexander Roberto Precioso; Jayme A. Souza-Neto; Jayme Augusto de Souza-Neto; Jessika Cristina Chagas Lesbon; José Salvatore Leister Patané; João Paulo Kitajima; Luiz Alcantara; Luiz Carlos Junior de Alcantara; Luiz Lehmann Coutinho; Maria Carolina Elias; Marta Giovanetti; Maurício Lacerda Nogueira; Patricia Akemi Assato; Rafael dos Santos Bezerra; Raquel de Lello Rocha Campos Cassano. NGS Soluções Genômicas: Pilar Drummond Sampaio Corrêa Mariani. FZEA-USP Pirassununga: Mirele Daiana Poleti; Raul Machado Neto; Rejane Maria Tommasini Grotto; Ricardo Augusto Brassaloti; Ricardo Haddad; Rodrigo Tocantins Calado.; Sandra Coccuzzo Sampaio; Sandra Coccuzzo Sampaio Vessoni; Simone Kashima; Svetoslav Nanev Slavov; Vagner Fonseca; Vincent Louis Viala
EPI_ISL_1966785	CS DE MIRASSOLANDIA	Instituto Butantan / Mendelics	Antonio Jorge Martins; Bianca Cechetto Carlos. Mendelics: Bibiana Santos; Claudia Renata dos Santos Barros; Cíntia Bittar; David Schlesinger. Hemocentro Ribeirão Preto: Simone Kashima; Debora Botequio Moretti; Elaine Cristina Marqueze; Elaine Vieira dos Santos; Elisangela Chicaroni Mattos; Erika Freitas; Evandra Strazza Rodrigues; Felipe Allan da Silva da Costa; Flavia Aburjaile; Fábio Sossai Possebon; Guilherme Campos; Guilherme Targino Valente; Heidge Fukumasu. USP-Botucatu: Rejane Maria Tommasini Grotto; Helena Lage Ferreira; Instituto Butantan: Dimas Tadeu Covas; Jardelina de Souza Todao Bernardino; Jayme A. Souza-Neto; Jessika Cristina Chagas Lesbon; Jorge A. Petroli Marchesi; José Salvatore Leister Patané; João Paulo Kitajima; João Pessoa Araújo Jr.; Leila Sabrina Ullmann; Loyze Paola Oliveira de Lima; Luiz Aurelio de Campos Crispin. Centro de Genômica Funcional da ESALQ: Luiz Lehmann Coutinho; Luiz Carlos Junior de Alcantara; Lívia Sacchetto; Maisa C. Pereira Parra; Maria Carolina Elias; Marta Giovanetti; Marília Moraes; Maurício Lacerda Nogueira. Prefeitura de Sao Paulo: Melissa Palmieri.; Patricia Akemi Assato; Paula Rahal; Paulo Inacio da Costa; Rafael dos Santos Bezerra; Raquel de Lello Rocha Campos Cassano. NGS Soluções Genômicas: Pilar Drummond Sampaio Corrêa Mariani. FZEA-USP Pirassununga: Mirele Daiana Poleti; Raul Machado Neto; Ricardo Augusto Brassaloti; Ricardo Haddad; Rodrigo Tocantins Calado. FAMERP-SJRP: Cecília Artico Banho; Sandra Coccuzzo Sampaio; Svetoslav Nanev Slavov; Vagner Fonseca; Vincent Louis Viala
EPI_ISL_1966961	CS DE MONTE APRAZIVEL	Instituto Butantan / ESALQ-USP (Piracicaba)	Antonio Jorge Martins; Bianca Cechetto Carlos. Mendelics: Bibiana Santos; Claudia Renata dos Santos Barros; Cíntia Bittar; David Schlesinger. Hemocentro Ribeirão Preto: Simone Kashima; Debora Botequio Moretti; Elaine Cristina Marqueze; Elaine Vieira dos Santos; Elisangela Chicaroni Mattos; Erika Freitas; Evandra Strazza Rodrigues; Felipe Allan da Silva da Costa; Flavia Aburjaile; Fábio Sossai Possebon; Guilherme Campos; Guilherme Targino Valente; Heidge Fukumasu. USP-Botucatu: Rejane Maria Tommasini Grotto; Helena Lage Ferreira; Instituto Butantan: Dimas Tadeu Covas; Jardelina de Souza Todao Bernardino; Jayme A. Souza-Neto; Jessika Cristina Chagas Lesbon; Jorge A. Petroli Marchesi; José Salvatore Leister Patané; João Paulo Kitajima; João Pessoa Araújo Jr.; Leila Sabrina Ullmann; Loyze Paola Oliveira de Lima; Luiz Aurelio de Campos Crispin. Centro de Genômica Funcional da ESALQ: Luiz Lehmann Coutinho; Luiz Carlos Junior de Alcantara; Lívia Sacchetto; Maisa C. Pereira Parra; Maria Carolina Elias; Marta Giovanetti; Marília Moraes; Maurício Lacerda Nogueira. Prefeitura de Sao Paulo: Melissa Palmieri.; Patricia Akemi Assato; Paula Rahal; Paulo Inacio da Costa; Rafael dos Santos Bezerra; Raquel de Lello Rocha Campos Cassano. NGS Soluções Genômicas: Pilar Drummond Sampaio Corrêa Mariani. FZEA-USP Pirassununga: Mirele Daiana Poleti; Raul Machado Neto; Ricardo Augusto Brassaloti; Ricardo Haddad; Rodrigo Tocantins Calado. FAMERP-SJRP: Cecília Artico Banho; Sandra Coccuzzo Sampaio; Svetoslav Nanev Slavov; Vagner Fonseca; Vincent Louis Viala
EPI_ISL_1966984, EPI_ISL_1966985	CS DE NEVES PAULISTA	Instituto Butantan / ESALQ-USP (Piracicaba)	Antonio Jorge Martins; Bianca Cechetto Carlos. Mendelics: Bibiana Santos; Claudia Renata dos Santos Barros; Cíntia Bittar; David Schlesinger. Hemocentro Ribeirão Preto: Simone Kashima; Debora Botequio Moretti; Elaine Cristina Marqueze; Elaine Vieira dos Santos; Elisangela Chicaroni Mattos; Erika Freitas; Evandra Strazza Rodrigues; Felipe Allan da Silva da Costa; Flavia Aburjaile; Fábio Sossai Possebon; Guilherme Campos; Guilherme Targino Valente; Heidge Fukumasu. USP-Botucatu: Rejane Maria Tommasini Grotto; Helena Lage Ferreira; Instituto Butantan: Dimas Tadeu Covas; Jardelina de Souza Todao Bernardino; Jayme A. Souza-Neto; Jessika Cristina Chagas Lesbon; Jorge A. Petroli Marchesi; José Salvatore Leister Patané; João Paulo Kitajima; João Pessoa Araújo Jr.; Leila Sabrina Ullmann; Loyze Paola Oliveira de Lima; Luiz Aurelio de Campos Crispin. Centro de Genômica Funcional da ESALQ: Luiz Lehmann Coutinho; Luiz Carlos Junior de Alcantara; Lívia Sacchetto; Maisa C. Pereira Parra; Maria Carolina Elias; Marta Giovanetti; Marília Moraes; Maurício Lacerda Nogueira. Prefeitura de Sao Paulo: Melissa Palmieri.; Patricia Akemi Assato; Paula Rahal; Paulo Inacio da Costa; Rafael dos Santos Bezerra; Raquel de Lello Rocha Campos Cassano. NGS Soluções Genômicas: Pilar Drummond Sampaio Corrêa Mariani. FZEA-USP Pirassununga: Mirele Daiana Poleti; Raul Machado Neto; Ricardo Augusto Brassaloti; Ricardo Haddad; Rodrigo Tocantins Calado. FAMERP-SJRP: Cecília Artico Banho; Sandra Coccuzzo Sampaio; Svetoslav Nanev Slavov; Vagner Fonseca; Vincent Louis Viala
EPI_ISL_1795288, EPI_ISL_2345526	CS DE NIPOA	Instituto Butantan / ESALQ-Piracicaba	Antonio Jorge Martins; Bianca Cechetto Carlos. Mendelics: Bibiana Santos; Claudia Renata dos Santos Barros; David Schlesinger; David Schlesinger. Hemocentro Ribeirão Preto: Simone Kashima; Debora Botequio Moretti; Debora Botequio Moretti. Centro de Genômica Funcional da ESALQ: Luiz Lehmann Coutinho; Dimas Tadeu Covas; Elaine Cristina Marqueze; Elaine Vieira Santos; Elaine Vieira dos Santos; Elisangela Chicaroni Mattos; Erika Freitas; Evandra Strazza Rodrigues; Felipe Allan da Silva da Costa; Flavia Aburjaile; Guilherme Targino Valente; Heidge Fukumasu; Heidge Fukumasu. USP-Botucatu: Rejane Maria Tommasini Grotto; Instituto Butantan: Alexander Roberto Precioso; Jayme A. Souza-Neto; Jayme Augusto de Souza-Neto; Jessika Cristina Chagas Lesbon; José Salvatore Leister Patané; João Paulo Kitajima; Luiz Alcantara; Luiz Carlos Junior de Alcantara; Luiz Lehmann Coutinho; Maria Carolina Elias; Marta Giovanetti; Maurício Lacerda Nogueira; Patricia Akemi Assato; Rafael dos Santos Bezerra; Raquel de Lello Rocha Campos Cassano. NGS Soluções Genômicas: Pilar Drummond Sampaio Corrêa Mariani. FZEA-USP Pirassununga: Mirele Daiana Poleti; Raul Machado Neto; Rejane Maria Tommasini Grotto; Ricardo Augusto Brassaloti; Ricardo Haddad; Rodrigo Tocantins Calado.; Sandra Coccuzzo Sampaio; Sandra Coccuzzo Sampaio Vessoni; Simone Kashima; Svetoslav Nanev Slavov; Vagner Fonseca; Vincent Louis Viala
EPI_ISL_1966788, EPI_ISL_1966789	CS DE NIPOA	Instituto Butantan / Mendelics	Antonio Jorge Martins; Bianca Cechetto Carlos. Mendelics: Bibiana Santos; Claudia Renata dos Santos Barros; Cíntia Bittar; David Schlesinger. Hemocentro Ribeirão Preto: Simone Kashima; Debora Botequio Moretti; Elaine Cristina Marqueze; Elaine Vieira dos Santos; Elisangela Chicaroni Mattos; Erika Freitas; Evandra Strazza Rodrigues; Felipe Allan da Silva da Costa; Flavia Aburjaile; Fábio Sossai Possebon; Guilherme Campos; Guilherme Targino Valente; Heidge Fukumasu. USP-Botucatu: Rejane Maria Tommasini Grotto; Helena Lage Ferreira; Instituto Butantan: Dimas Tadeu Covas; Jardelina de Souza Todao Bernardino; Jayme A. Souza-Neto; Jessika Cristina Chagas Lesbon; Jorge A. Petroli Marchesi; José Salvatore Leister Patané; João Paulo Kitajima; João Pessoa Araújo Jr.; Leila Sabrina Ullmann; Loyze Paola Oliveira de Lima; Luiz Aurelio de Campos Crispin. Centro de Genômica Funcional da ESALQ: Luiz Lehmann Coutinho; Luiz Carlos Junior de Alcantara; Lívia Sacchetto; Maisa C. Pereira Parra; Maria Carolina Elias; Marta Giovanetti; Marília Moraes; Maurício Lacerda Nogueira. Prefeitura de Sao Paulo: Melissa Palmieri.; Patricia Akemi Assato; Paula Rahal; Paulo Inacio da Costa; Rafael dos Santos Bezerra; Raquel de Lello Rocha Campos Cassano. NGS Soluções Genômicas: Pilar Drummond Sampaio Corrêa Mariani. FZEA-USP Pirassununga: Mirele Daiana Poleti; Raul Machado Neto; Ricardo Augusto Brassaloti; Ricardo Haddad; Rodrigo Tocantins Calado. FAMERP-SJRP: Cecília Artico Banho; Sandra Coccuzzo Sampaio; Svetoslav Nanev Slavov; Vagner Fonseca; Vincent Louis Viala
EPI_ISL_1966988	CS DE ORINDIUVA	Instituto Butantan / ESALQ-USP (Piracicaba)	Antonio Jorge Martins; Bianca Cechetto Carlos. Mendelics: Bibiana Santos; Claudia Renata dos Santos Barros; Cíntia Bittar; David Schlesinger. Hemocentro Ribeirão Preto: Simone Kashima; Debora Botequio Moretti; Elaine Cristina Marqueze; Elaine Vieira dos Santos; Elisangela Chicaroni Mattos; Erika Freitas; Evandra Strazza Rodrigues; Felipe Allan da Silva da Costa; Flavia Aburjaile; Fábio Sossai Possebon; Guilherme Campos; Guilherme Targino Valente; Heidge Fukumasu. USP-Botucatu: Rejane Maria Tommasini Grotto; Helena Lage Ferreira; Instituto Butantan: Dimas Tadeu Covas; Jardelina de Souza Todao Bernardino; Jayme A. Souza-Neto; Jessika Cristina Chagas Lesbon; Jorge A. Petroli Marchesi; José Salvatore Leister Patané; João Paulo Kitajima; João Pessoa Araújo Jr.; Leila Sabrina Ullmann; Loyze Paola Oliveira de Lima; Luiz Aurelio de Campos Crispin. Centro de Genômica Funcional da ESALQ: Luiz Lehmann Coutinho; Luiz Carlos Junior de Alcantara; Lívia Sacchetto; Maisa C. Pereira Parra; Maria Carolina Elias; Marta Giovanetti; Marília Moraes; Maurício Lacerda Nogueira. Prefeitura de Sao Paulo: Melissa Palmieri.; Patricia Akemi Assato; Paula Rahal; Paulo Inacio da Costa; Rafael dos Santos Bezerra; Raquel de Lello Rocha Campos Cassano. NGS Soluções Genômicas: Pilar Drummond Sampaio Corrêa Mariani. FZEA-USP Pirassununga: Mirele Daiana Poleti; Raul Machado Neto; Ricardo Augusto Brassaloti; Ricardo Haddad; Rodrigo Tocantins Calado. FAMERP-SJRP: Cecília Artico Banho; Sandra Coccuzzo Sampaio; Svetoslav Nanev Slavov; Vagner Fonseca; Vincent Louis Viala
EPI_ISL_1795171, EPI_ISL_1795173, EPI_ISL_1795390, EPI_ISL_2345383,	CS DE OSCAR BRESSANE PSF	Instituto Butantan / ESALQ-Piracicaba	Antonio Jorge Martins; Bianca Cechetto Carlos. Mendelics: Bibiana Santos; Claudia Renata dos Santos Barros; David Schlesinger; David Schlesinger. Hemocentro Ribeirão Preto: Simone Kashima; Debora Botequio Moretti; Debora Botequio Moretti. Centro de Genômica Funcional da ESALQ: Luiz Lehmann Coutinho; Dimas Tadeu Covas; Elaine Cristina Marqueze; Elaine Vieira Santos; Elaine Vieira dos Santos; Elisangela Chicaroni Mattos; Erika Freitas; Evandra Strazza Rodrigues; Felipe Allan da Silva da Costa; Flavia Aburjaile; Guilherme Targino Valente; Heidge Fukumasu; Heidge Fukumasu. USP-Botucatu: Rejane Maria Tommasini Grotto; Instituto Butantan: Alexander Roberto Precioso; Jayme A. Souza-Neto; Jayme Augusto de Souza-Neto; Jessika Cristina Chagas Lesbon; José Salvatore Leister Patané; João Paulo Kitajima; Luiz Alcantara; Luiz Carlos Junior de Alcantara; Luiz Lehmann Coutinho; Maria Carolina Elias; Marta Giovanetti; Maurício Lacerda Nogueira; Patricia Akemi Assato; Rafael dos Santos Bezerra; Raquel de Lello Rocha Campos Cassano. NGS Soluções Genômicas: Pilar Drummond Sampaio Corrêa Mariani. FZEA-USP Pirassununga: Mirele Daiana Poleti; Raul Machado Neto; Rejane Maria Tommasini Grotto; Ricardo Augusto Brassaloti; Ricardo Haddad; Rodrigo Tocantins Calado.; Sandra Coccuzzo Sampaio; Sandra Coccuzzo

EPI_ISL_2345388, EPI_ISL_2345389			Sampaio Vessoni; Simone Kashima; Svetoslav Nanev Slavov; Vagner Fonseca; Vincent Louis Viala
EPI_ISL_1795276, EPI_ISL_1795277, EPI_ISL_1795278, EPI_ISL_1795279, EPI_ISL_1795280, EPI_ISL_1795281, EPI_ISL_1795282, EPI_ISL_1795283, EPI_ISL_1795284, EPI_ISL_1795285, EPI_ISL_1795286, EPI_ISL_2345514, EPI_ISL_2345515, EPI_ISL_2345516, EPI_ISL_2345517, EPI_ISL_2345518, EPI_ISL_2345519, EPI_ISL_2345520, EPI_ISL_2345521, EPI_ISL_2345522, EPI_ISL_2345523, EPI_ISL_2345524			
see above	CS DE PALESTINA	Instituto Butantan / ESALQ-Piracicaba	Antonio Jorge Martins; Bianca Cechetto Carlos. Mendelics: Bibiana Santos; Claudia Renata dos Santos Barros; David Schlesinger. Hemocentro Ribeirão Preto: Simone Kashima; Debora Botequio Moretti. Centro de Genômica Funcional da ESALQ: Luiz Lehmann Coutinho; Dimas Tadeu Covas; Elaine Cristina Marqueze; Elaine Vieira Santos; Elaine Vieira dos Santos; Elisangela Chicaroni Mattos; Erika Freitas; Evandra Strazza Rodrigues; Felipe Allan da Silva da Costa; Flavia Aburjaile; Guilherme Targino Valente; Heidge Fukumasu; Heidge Fukumasu. USP-Botucatu: Rejane Maria Tommasini Grotto; Instituto Butantan: Alexander Roberto Precioso; Jayme A. Souza-Neto; Jayme Augusto de Souza-Neto; Jessika Cristina Chagas Lesbon; José Salvatore Leister Patané; João Paulo Kitajima; Luiz Alcantara; Luiz Carlos Junior de Alcantara; Luiz Lehmann Coutinho; Maria Carolina Elias; Marta Giovanetti; Maurício Lacerda Nogueira; Patricia Akemi Assato; Rafael dos Santos Bezerra; Raquel de Lello Rocha Campos Cassano. NGS Soluções Genômicas: Pilar Drummond Sampaio Corrêa Mariani. FZEA-USP Pirassununga: Mirele Daiana Poleti; Raul Machado Neto; Rejane Maria Tommasini Grotto; Ricardo Augusto Brassaloti; Ricardo Haddad; Rodrigo Tocantins Calado.; Sandra Coccuzzo Sampaio; Sandra Coccuzzo Sampaio Vessoni; Simone Kashima; Svetoslav Nanev Slavov; Vagner Fonseca; Vincent Louis Viala
EPI_ISL_1795264, EPI_ISL_1795271, EPI_ISL_2345498, EPI_ISL_2345506	CS DE PARANAPUA	Instituto Butantan / ESALQ-Piracicaba	Antonio Jorge Martins; Bianca Cechetto Carlos. Mendelics: Bibiana Santos; Claudia Renata dos Santos Barros; David Schlesinger. Hemocentro Ribeirão Preto: Simone Kashima; Debora Botequio Moretti. Centro de Genômica Funcional da ESALQ: Luiz Lehmann Coutinho; Dimas Tadeu Covas; Elaine Cristina Marqueze; Elaine Vieira Santos; Elaine Vieira dos Santos; Elisangela Chicaroni Mattos; Erika Freitas; Evandra Strazza Rodrigues; Felipe Allan da Silva da Costa; Flavia Aburjaile; Guilherme Targino Valente; Heidge Fukumasu; Heidge Fukumasu. USP-Botucatu: Rejane Maria Tommasini Grotto; Instituto Butantan: Alexander Roberto Precioso; Jayme A. Souza-Neto; Jayme Augusto de Souza-Neto; Jessika Cristina Chagas Lesbon; José Salvatore Leister Patané; João Paulo Kitajima; Luiz Alcantara; Luiz Carlos Junior de Alcantara; Luiz Lehmann Coutinho; Maria Carolina Elias; Marta Giovanetti; Maurício Lacerda Nogueira; Patricia Akemi Assato; Rafael dos Santos Bezerra; Raquel de Lello Rocha Campos Cassano. NGS Soluções Genômicas: Pilar Drummond Sampaio Corrêa Mariani. FZEA-USP Pirassununga: Mirele Daiana Poleti; Raul Machado Neto; Rejane Maria Tommasini Grotto; Ricardo Augusto Brassaloti; Ricardo Haddad; Rodrigo Tocantins Calado.; Sandra Coccuzzo Sampaio; Sandra Coccuzzo Sampaio Vessoni; Simone Kashima; Svetoslav Nanev Slavov; Vagner Fonseca; Vincent Louis Viala
EPI_ISL_1795258, EPI_ISL_2345492	CS DE PLANALTO	Instituto Butantan / ESALQ-Piracicaba	Antonio Jorge Martins; Bianca Cechetto Carlos. Mendelics: Bibiana Santos; Claudia Renata dos Santos Barros; David Schlesinger. Hemocentro Ribeirão Preto: Simone Kashima; Debora Botequio Moretti. Centro de Genômica Funcional da ESALQ: Luiz Lehmann Coutinho; Dimas Tadeu Covas; Elaine Cristina Marqueze; Elaine Vieira Santos; Elaine Vieira dos Santos; Elisangela Chicaroni Mattos; Erika Freitas; Evandra Strazza Rodrigues; Felipe Allan da Silva da Costa; Flavia Aburjaile; Guilherme Targino Valente; Heidge Fukumasu; Heidge Fukumasu. USP-Botucatu: Rejane Maria Tommasini Grotto; Instituto Butantan: Alexander Roberto Precioso; Jayme A. Souza-Neto; Jayme Augusto de Souza-Neto; Jessika Cristina Chagas Lesbon; José Salvatore Leister Patané; João Paulo Kitajima; Luiz Alcantara; Luiz Carlos Junior de Alcantara; Luiz Lehmann Coutinho; Maria Carolina Elias; Marta Giovanetti; Maurício Lacerda Nogueira; Patricia Akemi Assato; Rafael dos Santos Bezerra; Raquel de Lello Rocha Campos Cassano. NGS Soluções Genômicas: Pilar Drummond Sampaio Corrêa Mariani. FZEA-USP Pirassununga: Mirele Daiana Poleti; Raul Machado Neto; Rejane Maria Tommasini Grotto; Ricardo Augusto Brassaloti; Ricardo Haddad; Rodrigo Tocantins Calado.; Sandra Coccuzzo Sampaio; Sandra Coccuzzo Sampaio Vessoni; Simone Kashima; Svetoslav Nanev Slavov; Vagner Fonseca; Vincent Louis Viala
EPI_ISL_2344769, EPI_ISL_2344772	CS DE RINOPOLIS	Instituto Butantan / ESALQ-Piracicaba	Antonio Jorge Martins; Claudia Renata dos Santos Barros; David Schlesinger; Debora Botequio Moretti; Dimas Tadeu Covas; Elaine Cristina Marqueze; Elaine Vieira Santos; Evandra Strazza Rodrigues; Heidge Fukumasu; Jayme Augusto de Souza-Neto; José Salvatore Leister Patané; Luiz Alcantara; Luiz Lehmann Coutinho; Maria Carolina Elias; Maurício Lacerda Nogueira; Rafael dos Santos Bezerra; Raul Machado Neto; Rejane Maria Tommasini Grotto; Ricardo Haddad; Sandra Coccuzzo Sampaio Vessoni; Simone Kashima; Svetoslav Nanev Slavov; Vincent Louis Viala
EPI_ISL_1966257	CS DE RUBINEIA	Instituto Butantan / Mendelics	Antonio Jorge Martins; Bianca Cechetto Carlos. Mendelics: Bibiana Santos; Claudia Renata dos Santos Barros; Cíntia Bittar; David Schlesinger. Hemocentro Ribeirão Preto: Simone Kashima; Debora Botequio Moretti; Elaine Cristina Marqueze; Elaine Vieira dos Santos; Elisangela Chicaroni Mattos; Erika Freitas; Evandra Strazza Rodrigues; Felipe Allan da Silva da Costa; Flavia Aburjaile; Fábio Sossai Possebon; Guilherme Campos; Guilherme Targino Valente; Heidge Fukumasu. USP-Botucatu: Rejane Maria Tommasini Grotto; Helena Lage Ferreira; Instituto Butantan: Dimas Tadeu Covas; Jardelina de Souza Todao Bernardino; Jayme A. Souza-Neto; Jessika Cristina Chagas Lesbon; Jorge A. Petroli Marchesi; José Salvatore Leister Patané; João Paulo Kitajima; João Pessoa Araújo Jr.; Leila Sabrina Ullmann; Loyze Paola Oliveira de Lima; Luiz Aurelio de Campos Crispin. Centro de Genômica Funcional da ESALQ: Luiz Lehmann Coutinho; Luiz Carlos Junior de Alcantara; Lívia Sacchetto; Maisa C. Pereira Parra; Maria Carolina Elias; Marta Giovanetti; Marília Moraes; Maurício Lacerda Nogueira. Prefeitura de Sao Paulo: Melissa Palmieri.; Patricia Akemi Assato; Paula Rahal; Paulo Inacio da Costa; Rafael dos Santos Bezerra; Raquel de Lello Rocha Campos Cassano. NGS Soluções Genômicas: Pilar Drummond Sampaio Corrêa Mariani. FZEA-USP Pirassununga: Mirele Daiana Poleti; Raul Machado Neto; Ricardo Augusto Brassaloti; Ricardo Haddad; Rodrigo Tocantins Calado. FAMERP-SJRP: Cecília Artico Banho; Sandra Coccuzzo Sampaio; Svetoslav Nanev Slavov; Vagner Fonseca; Vincent Louis Viala
EPI_ISL_1967050	CS DE SANTA RITA DOESTE	Instituto Butantan / ESALQ-USP (Piracicaba)	Antonio Jorge Martins; Bianca Cechetto Carlos. Mendelics: Bibiana Santos; Claudia Renata dos Santos Barros; Cíntia Bittar; David Schlesinger. Hemocentro Ribeirão Preto: Simone Kashima; Debora Botequio Moretti; Elaine Cristina Marqueze; Elaine Vieira dos Santos; Elisangela Chicaroni Mattos; Erika Freitas; Evandra Strazza Rodrigues; Felipe Allan da Silva da Costa; Flavia Aburjaile; Fábio Sossai Possebon; Guilherme Campos; Guilherme Targino Valente; Heidge Fukumasu. USP-Botucatu: Rejane Maria Tommasini Grotto; Helena Lage Ferreira; Instituto Butantan: Dimas Tadeu Covas; Jardelina de Souza Todao Bernardino; Jayme A. Souza-Neto; Jessika Cristina Chagas Lesbon; Jorge A. Petroli Marchesi; José Salvatore Leister Patané; João Paulo Kitajima; João Pessoa Araújo Jr.; Leila Sabrina Ullmann; Loyze Paola Oliveira de Lima; Luiz Aurelio de Campos Crispin. Centro de Genômica Funcional da ESALQ: Luiz Lehmann Coutinho; Luiz Carlos Junior de Alcantara; Lívia Sacchetto; Maisa C. Pereira Parra; Maria Carolina Elias; Marta Giovanetti; Marília Moraes; Maurício Lacerda Nogueira. Prefeitura de Sao Paulo: Melissa Palmieri.; Patricia Akemi Assato; Paula Rahal; Paulo Inacio da Costa; Rafael dos Santos Bezerra; Raquel de Lello Rocha Campos Cassano. NGS Soluções Genômicas: Pilar Drummond Sampaio Corrêa Mariani. FZEA-USP Pirassununga: Mirele Daiana Poleti; Raul Machado Neto; Ricardo Augusto Brassaloti; Ricardo Haddad; Rodrigo Tocantins Calado. FAMERP-SJRP: Cecília Artico Banho; Sandra Coccuzzo Sampaio; Svetoslav Nanev Slavov; Vagner Fonseca; Vincent Louis Viala
EPI_ISL_1966547	CS DE SANTA RITA DOESTE	Instituto Butantan / FZEA-USP (Pirassununga)	Antonio Jorge Martins; Bianca Cechetto Carlos. Mendelics: Bibiana Santos; Claudia Renata dos Santos Barros; Cíntia Bittar; David Schlesinger. Hemocentro Ribeirão Preto: Simone Kashima; Debora Botequio Moretti; Elaine Cristina Marqueze; Elaine Vieira dos Santos; Elisangela Chicaroni Mattos; Erika Freitas; Evandra Strazza Rodrigues; Felipe Allan da Silva da Costa; Flavia Aburjaile; Fábio Sossai Possebon; Guilherme Campos; Guilherme Targino Valente; Heidge Fukumasu. USP-Botucatu: Rejane Maria Tommasini Grotto; Helena Lage Ferreira; Instituto Butantan: Dimas Tadeu Covas; Jardelina de Souza Todao Bernardino; Jayme A. Souza-Neto; Jessika Cristina Chagas Lesbon; Jorge A. Petroli Marchesi; José Salvatore Leister Patané; João Paulo Kitajima; João Pessoa Araújo Jr.; Leila Sabrina Ullmann; Loyze Paola Oliveira de Lima; Luiz Aurelio de Campos Crispin. Centro de Genômica Funcional da ESALQ: Luiz Lehmann Coutinho; Luiz Carlos Junior de Alcantara; Lívia Sacchetto; Maisa C. Pereira Parra; Maria Carolina Elias; Marta Giovanetti; Marília Moraes; Maurício Lacerda Nogueira. Prefeitura de Sao Paulo: Melissa Palmieri.; Patricia Akemi Assato; Paula Rahal; Paulo Inacio da Costa; Rafael dos Santos Bezerra; Raquel de Lello Rocha Campos Cassano. NGS Soluções Genômicas: Pilar Drummond Sampaio Corrêa Mariani. FZEA-USP Pirassununga: Mirele Daiana Poleti; Raul Machado Neto; Ricardo Augusto Brassaloti; Ricardo Haddad; Rodrigo Tocantins Calado. FAMERP-SJRP: Cecília Artico Banho; Sandra Coccuzzo Sampaio; Svetoslav Nanev Slavov; Vagner Fonseca; Vincent Louis Viala
EPI_ISL_1795240, EPI_ISL_2345472	CS DE SEBASTIANOPOLIS DO SUL	Instituto Butantan / ESALQ-Piracicaba	Antonio Jorge Martins; Bianca Cechetto Carlos. Mendelics: Bibiana Santos; Claudia Renata dos Santos Barros; David Schlesinger. Hemocentro Ribeirão Preto: Simone Kashima; Debora Botequio Moretti. Centro de Genômica Funcional da ESALQ: Luiz Lehmann Coutinho; Dimas Tadeu Covas; Elaine Cristina Marqueze; Elaine Vieira Santos; Elaine Vieira dos Santos; Elisangela Chicaroni Mattos; Erika Freitas; Evandra Strazza Rodrigues; Felipe Allan da Silva da Costa; Flavia Aburjaile; Guilherme Targino Valente; Heidge Fukumasu; Heidge Fukumasu. USP-Botucatu: Rejane Maria Tommasini Grotto; Instituto Butantan: Alexander Roberto Precioso; Jayme A. Souza-Neto; Jayme Augusto de Souza-Neto; Jessika Cristina Chagas Lesbon; José Salvatore Leister Patané; João Paulo Kitajima; Luiz Alcantara; Luiz Carlos Junior de Alcantara; Luiz Lehmann Coutinho; Maria Carolina Elias; Marta Giovanetti; Maurício Lacerda Nogueira; Patricia Akemi Assato; Rafael dos Santos Bezerra; Raquel de Lello Rocha Campos Cassano. NGS Soluções Genômicas: Pilar Drummond Sampaio Corrêa Mariani. FZEA-USP Pirassununga: Mirele Daiana Poleti; Raul Machado Neto; Rejane Maria Tommasini Grotto; Ricardo Augusto Brassaloti; Ricardo Haddad; Rodrigo Tocantins Calado.; Sandra Coccuzzo Sampaio; Sandra Coccuzzo Sampaio Vessoni; Simone Kashima; Svetoslav Nanev Slavov; Vagner Fonseca; Vincent Louis Viala
EPI_ISL_1795232, EPI_ISL_1795233, EPI_ISL_1795239, EPI_ISL_1795242, EPI_ISL_1795243, EPI_ISL_1795244, EPI_ISL_2345464, EPI_ISL_2345465, EPI_ISL_2345471, EPI_ISL_2345474, EPI_ISL_2345476, EPI_ISL_2345477			
see above	CS DE URUPES	Instituto Butantan / ESALQ-Piracicaba	Antonio Jorge Martins; Bianca Cechetto Carlos. Mendelics: Bibiana Santos; Claudia Renata dos Santos Barros; David Schlesinger. Hemocentro Ribeirão Preto: Simone Kashima; Debora Botequio Moretti. Centro de Genômica Funcional da ESALQ: Luiz Lehmann Coutinho; Dimas Tadeu Covas; Elaine Cristina Marqueze; Elaine Vieira Santos; Elaine Vieira dos Santos; Elisangela Chicaroni Mattos; Erika Freitas; Evandra Strazza Rodrigues; Felipe Allan da Silva da Costa; Flavia Aburjaile; Guilherme Targino Valente; Heidge Fukumasu; Heidge Fukumasu. USP-Botucatu: Rejane Maria Tommasini Grotto; Instituto Butantan: Alexander Roberto Precioso; Jayme A. Souza-Neto; Jayme Augusto de Souza-Neto; Jessika Cristina Chagas Lesbon; José Salvatore Leister Patané; João Paulo Kitajima; Luiz Alcantara; Luiz Carlos Junior de Alcantara; Luiz Lehmann Coutinho; Maria Carolina Elias; Marta Giovanetti; Maurício Lacerda Nogueira; Patricia Akemi Assato; Rafael dos Santos Bezerra; Raquel de Lello Rocha Campos Cassano. NGS Soluções Genômicas: Pilar Drummond Sampaio Corrêa Mariani. FZEA-USP Pirassununga: Mirele Daiana Poleti; Raul Machado Neto; Rejane Maria Tommasini Grotto; Ricardo Augusto Brassaloti; Ricardo Haddad; Rodrigo Tocantins Calado.; Sandra Coccuzzo Sampaio; Sandra Coccuzzo Sampaio Vessoni; Simone Kashima; Svetoslav Nanev Slavov; Vagner Fonseca; Vincent Louis Viala
EPI_ISL_1966992, EPI_ISL_1966993	CS I DE OURINHOS	Instituto Butantan / ESALQ-USP (Piracicaba)	Antonio Jorge Martins; Bianca Cechetto Carlos. Mendelics: Bibiana Santos; Claudia Renata dos Santos Barros; Cíntia Bittar; David Schlesinger. Hemocentro Ribeirão Preto: Simone Kashima; Debora Botequio Moretti; Elaine Cristina Marqueze; Elaine Vieira dos Santos; Elisangela Chicaroni Mattos; Erika Freitas; Evandra Strazza Rodrigues; Felipe Allan da Silva da Costa; Flavia Aburjaile; Fábio Sossai Possebon; Guilherme Campos; Guilherme Targino Valente; Heidge Fukumasu. USP-Botucatu: Rejane Maria Tommasini Grotto; Helena Lage Ferreira; Instituto Butantan: Dimas Tadeu Covas; Jardelina de Souza Todao Bernardino; Jayme A. Souza-Neto; Jessika Cristina Chagas Lesbon; Jorge A. Petroli Marchesi; José Salvatore Leister Patané; João Paulo Kitajima; João Pessoa Araújo Jr.; Leila Sabrina Ullmann; Loyze Paola Oliveira de Lima; Luiz Aurelio de Campos Crispin. Centro de Genômica Funcional da ESALQ: Luiz Lehmann Coutinho; Luiz Carlos Junior de Alcantara; Lívia Sacchetto; Maisa C. Pereira Parra; Maria Carolina Elias; Marta Giovanetti; Marília Moraes; Maurício Lacerda Nogueira. Prefeitura de Sao Paulo: Melissa Palmieri.; Patricia Akemi Assato; Paula Rahal; Paulo Inacio da Costa; Rafael dos Santos Bezerra; Raquel de Lello Rocha Campos Cassano. NGS Soluções Genômicas: Pilar Drummond Sampaio Corrêa Mariani. FZEA-USP Pirassununga: Mirele Daiana Poleti; Raul Machado Neto; Ricardo Augusto Brassaloti; Ricardo Haddad; Rodrigo Tocantins Calado. FAMERP-SJRP: Cecília Artico Banho; Sandra Coccuzzo Sampaio; Svetoslav Nanev Slavov; Vagner Fonseca; Vincent Louis Viala
EPI_ISL_2344778	CS IACRI	Instituto Butantan / ESALQ-Piracicaba	Antonio Jorge Martins; Claudia Renata dos Santos Barros; David Schlesinger; Debora Botequio Moretti; Dimas Tadeu Covas; Elaine Cristina Marqueze; Elaine Vieira Santos; Evandra Strazza Rodrigues; Heidge Fukumasu; Jayme Augusto de Souza-Neto; José Salvatore Leister Patané; Luiz Alcantara; Luiz Lehmann Coutinho; Maria Carolina Elias; Maurício Lacerda Nogueira; Rafael dos Santos Bezerra; Raul Machado Neto; Rejane Maria Tommasini Grotto; Ricardo Haddad; Sandra Coccuzzo Sampaio Vessoni; Simone Kashima; Svetoslav Nanev Slavov; Vincent Louis Viala
EPI_ISL_1966909	CS II DR JAHYR DE PAULA RIBEIRO GUARA	Instituto Butantan / ESALQ-USP (Piracicaba)	Antonio Jorge Martins; Bianca Cechetto Carlos. Mendelics: Bibiana Santos; Claudia Renata dos Santos Barros; Cíntia Bittar; David Schlesinger. Hemocentro Ribeirão Preto: Simone Kashima; Debora Botequio Moretti; Elaine Cristina Marqueze; Elaine Vieira dos Santos; Elisangela Chicaroni Mattos; Erika Freitas; Evandra Strazza Rodrigues; Felipe Allan da Silva da Costa; Flavia Aburjaile; Fábio Sossai Possebon; Guilherme Campos; Guilherme Targino Valente; Heidge Fukumasu. USP-Botucatu: Rejane Maria Tommasini Grotto; Helena Lage Ferreira; Instituto Butantan: Dimas Tadeu Covas; Jardelina de Souza Todao Bernardino; Jayme A. Souza-Neto; Jessika Cristina Chagas Lesbon; Jorge A. Petroli Marchesi; José Salvatore Leister Patané; João Paulo Kitajima; João Pessoa Araújo Jr.; Leila Sabrina Ullmann; Loyze Paola Oliveira de Lima; Luiz Aurelio de Campos Crispin. Centro de Genômica Funcional da ESALQ: Luiz Lehmann Coutinho; Luiz Carlos Junior de Alcantara; Lívia Sacchetto; Maisa C. Pereira Parra; Maria Carolina Elias; Marta Giovanetti; Marília Moraes; Maurício Lacerda Nogueira. Prefeitura de Sao Paulo: Melissa Palmieri.; Patricia Akemi Assato; Paula Rahal; Paulo Inacio da Costa; Rafael dos Santos Bezerra; Raquel de Lello Rocha Campos Cassano. NGS Soluções Genômicas: Pilar Drummond Sampaio Corrêa Mariani. FZEA-USP Pirassununga: Mirele Daiana Poleti; Raul Machado Neto; Ricardo Augusto Brassaloti; Ricardo Haddad; Rodrigo Tocantins Calado. FAMERP-SJRP: Cecília Artico Banho; Sandra Coccuzzo Sampaio; Svetoslav Nanev Slavov; Vagner Fonseca; Vincent Louis Viala
EPI_ISL_1966808	CS II DR JOSE CARQUEIJO SCRPARDO	Instituto Butantan / Mendelics	Antonio Jorge Martins; Bianca Cechetto Carlos. Mendelics: Bibiana Santos; Claudia Renata dos Santos Barros; Cíntia Bittar; David Schlesinger. Hemocentro Ribeirão Preto: Simone Kashima; Debora Botequio Moretti; Elaine Cristina Marqueze; Elaine Vieira dos Santos; Elisangela Chicaroni Mattos; Erika Freitas; Evandra Strazza Rodrigues; Felipe Allan da Silva da Costa; Flavia Aburjaile; Fábio Sossai Possebon; Guilherme Campos; Guilherme Targino Valente; Heidge Fukumasu. USP-Botucatu: Rejane Maria Tommasini Grotto; Helena Lage Ferreira; Instituto Butantan: Dimas Tadeu Covas; Jardelina de Souza Todao Bernardino; Jayme A. Souza-Neto; Jessika Cristina Chagas Lesbon; Jorge A. Petroli Marchesi; José Salvatore Leister Patané; João Paulo Kitajima; João Pessoa Araújo Jr.; Leila Sabrina Ullmann; Loyze Paola Oliveira de Lima; Luiz Aurelio de Campos Crispin. Centro de Genômica Funcional da ESALQ: Luiz Lehmann Coutinho; Luiz Carlos Junior de Alcantara; Lívia Sacchetto; Maisa C. Pereira Parra; Maria Carolina Elias; Marta Giovanetti; Marília Moraes; Maurício Lacerda Nogueira. Prefeitura de Sao Paulo: Melissa Palmieri.; Patricia Akemi Assato; Paula Rahal; Paulo Inacio da Costa; Rafael dos Santos Bezerra; Raquel de Lello Rocha Campos Cassano. NGS Soluções Genômicas: Pilar Drummond Sampaio Corrêa Mariani. FZEA-USP Pirassununga: Mirele Daiana Poleti; Raul Machado Neto; Ricardo Augusto Brassaloti; Ricardo Haddad; Rodrigo Tocantins Calado. FAMERP-SJRP: Cecília Artico Banho; Sandra Coccuzzo Sampaio; Svetoslav Nanev Slavov; Vagner Fonseca; Vincent Louis Viala
EPI_ISL_1966989	CS II DR MIGUEL VITALIANO ORLANDIA	Instituto Butantan / ESALQ-USP (Piracicaba)	Antonio Jorge Martins; Bianca Cechetto Carlos. Mendelics: Bibiana Santos; Claudia Renata dos Santos Barros; Cíntia Bittar; David Schlesinger. Hemocentro Ribeirão Preto: Simone Kashima; Debora Botequio Moretti; Elaine Cristina Marqueze; Elaine Vieira dos Santos; Elisangela Chicaroni Mattos; Erika Freitas; Evandra Strazza Rodrigues; Felipe Allan da Silva da Costa; Flavia Aburjaile; Fábio Sossai Possebon; Guilherme Campos; Guilherme Targino Valente; Heidge Fukumasu. USP-Botucatu: Rejane Maria Tommasini Grotto; Helena Lage Ferreira; Instituto Butantan: Dimas Tadeu Covas; Jardelina de Souza Todao Bernardino; Jayme A. Souza-Neto; Jessika Cristina Chagas Lesbon; Jorge A. Petroli Marchesi; José Salvatore Leister Patané; João Paulo Kitajima; João Pessoa Araújo Jr.; Leila Sabrina Ullmann; Loyze Paola Oliveira de Lima; Luiz Aurelio de Campos Crispin. Centro de Genômica Funcional da ESALQ: Luiz Lehmann Coutinho; Luiz Carlos Junior de Alcantara; Lívia Sacchetto; Maisa C. Pereira Parra; Maria Carolina Elias; Marta Giovanetti; Marília Moraes; Maurício Lacerda Nogueira. Prefeitura de Sao Paulo: Melissa Palmieri.; Patricia Akemi Assato; Paula Rahal; Paulo Inacio da Costa; Rafael dos Santos Bezerra; Raquel de Lello Rocha Campos Cassano. NGS Soluções Genômicas: Pilar Drummond Sampaio Corrêa Mariani. FZEA-USP Pirassununga: Mirele Daiana Poleti; Raul Machado Neto; Ricardo Augusto Brassaloti; Ricardo Haddad; Rodrigo Tocantins Calado. FAMERP-SJRP: Cecília Artico Banho; Sandra Coccuzzo Sampaio; Svetoslav Nanev Slavov; Vagner Fonseca; Vincent Louis Viala
EPI_ISL_1625975	CS II Dr Antonio Vicoso Moreira De Rezende Sumare	Instituto Adolfo Lutz, Interdiciplinary Procedures Center, Strategic Laboratory	Caio Vinicius Dias Lopes; Claudia Regina Gonçalves; Claudio Tavares Sacchi; Erica Valessa Ramos Gomes; Karoline Rodrigues Campos; Katia Correa de Oliveira Santos; Leonardo Jose Tadeu de Araujo
EPI_ISL_1715135, EPI_ISL_1715136	CS II Dr Jose Ferreira Telles	Instituto Adolfo Lutz, Interdiciplinary Procedures Center, Strategic Laboratory	Caio Vinicius Dias Lopes; Claudia Regina Gonçalves; Claudio Tavares Sacchi; Erica Valessa Ramos Gomes; Karoline Rodrigues Campos; Katia Correa de Oliveira Santos; Leonardo Jose Tadeu de Araujo
EPI_ISL_1493580, EPI_ISL_1493582	CS II Dr Miguel Vitaliano Orlandia	Instituto Adolfo Lutz, Interdiciplinary Procedures Center, Strategic Laboratory	Caio Vinicius Dias Lopes; Claudia Regina Gonçalves; Claudio Tavares Sacchi; Erica Valessa Ramos Gomes; Karoline Rodrigues Campos
EPI_ISL_1966965, EPI_ISL_1966966, EPI_ISL_1966967, EPI_ISL_1966968, EPI_ISL_1966970, EPI_ISL_1966971, EPI_ISL_1966972, EPI_ISL_1966973, EPI_ISL_1966974, EPI_ISL_1966975, EPI_ISL_1966977, EPI_ISL_1966978, EPI_ISL_1966979, EPI_ISL_1966980, EPI_ISL_1966981, EPI_ISL_1966982, EPI_ISL_1966983			
see above	CS II EGIDIO BRUNHARA MORRO AGUDO	Instituto Butantan / ESALQ-USP (Piracicaba)	Antonio Jorge Martins; Bianca Cechetto Carlos. Mendelics: Bibiana Santos; Claudia Renata dos Santos Barros; Cíntia Bittar; David Schlesinger. Hemocentro Ribeirão Preto: Simone Kashima; Debora Botequio Moretti; Elaine Cristina Marqueze; Elaine Vieira dos Santos; Elisangela Chicaroni Mattos; Erika Freitas; Evandra Strazza Rodrigues; Felipe Allan da Silva da Costa; Flavia Aburjaile; Fábio Sossai Possebon; Guilherme Campos; Guilherme Targino Valente; Heidge Fukumasu. USP-Botucatu: Rejane Maria Tommasini Grotto; Helena Lage Ferreira; Instituto Butantan: Dimas Tadeu Covas; Jardelina de Souza Todao Bernardino; Jayme A. Souza-Neto; Jessika Cristina Chagas Lesbon; Jorge A. Petroli Marchesi; José Salvatore Leister Patané; João Paulo Kitajima; João Pessoa Araújo Jr.; Leila Sabrina Ullmann; Loyze Paola Oliveira de Lima; Luiz Aurelio de Campos Crispin. Centro de Genômica Funcional da ESALQ: Luiz Lehmann Coutinho; Luiz Carlos Junior de Alcantara; Lívia Sacchetto; Maisa C. Pereira Parra; Maria Carolina Elias; Marta Giovanetti; Marília Moraes; Maurício Lacerda Nogueira. Prefeitura de Sao Paulo: Melissa Palmieri.; Patricia Akemi Assato; Paula Rahal; Paulo Inacio da Costa; Rafael dos Santos Bezerra; Raquel de Lello Rocha Campos Cassano. NGS Soluções Genômicas: Pilar Drummond Sampaio Corrêa Mariani. FZEA-USP Pirassununga: Mirele Daiana Poleti; Raul Machado Neto; Ricardo Augusto Brassaloti; Ricardo Haddad; Rodrigo Tocantins Calado. FAMERP-SJRP: Cecília Artico Banho; Sandra Coccuzzo Sampaio; Svetoslav Nanev Slavov; Vagner Fonseca; Vincent Louis Viala
EPI_ISL_1966480	CS III SANTOPOLIS DO AGUAPEI	Instituto Butantan / Mendelics	Antonio Jorge Martins; Bianca Cechetto Carlos. Mendelics: Bibiana Santos; Claudia Renata dos Santos Barros; Cíntia Bittar; David Schlesinger. Hemocentro Ribeirão Preto: Simone Kashima; Debora Botequio Moretti; Elaine Cristina Marqueze; Elaine Vieira dos Santos; Elisangela Chicaroni Mattos; Erika Freitas; Evandra Strazza Rodrigues; Felipe Allan da Silva da Costa; Flavia Aburjaile; Fábio Sossai Possebon; Guilherme Campos; Guilherme Targino Valente; Heidge Fukumasu. USP-Botucatu: Rejane Maria Tommasini Grotto; Helena Lage Ferreira; Instituto Butantan: Dimas Tadeu Covas; Jardelina de Souza Todao Bernardino; Jayme A. Souza-Neto; Jessika Cristina Chagas Lesbon; Jorge A. Petroli Marchesi; José Salvatore Leister Patané; João Paulo Kitajima; João Pessoa Araújo Jr.; Leila Sabrina Ullmann; Loyze Paola Oliveira de Lima; Luiz Aurelio de Campos Crispin. Centro de Genômica Funcional da ESALQ: Luiz Lehmann Coutinho; Luiz Carlos Junior de Alcantara; Lívia Sacchetto; Maisa C. Pereira Parra; Maria Carolina Elias; Marta Giovanetti; Marília Moraes; Maurício Lacerda Nogueira. Prefeitura de Sao Paulo: Melissa Palmieri.; Patricia Akemi Assato; Paula Rahal; Paulo Inacio da Costa; Rafael dos Santos Bezerra; Raquel de Lello Rocha Campos Cassano. NGS Soluções Genômicas: Pilar Drummond Sampaio Corrêa Mariani. FZEA-USP Pirassununga: Mirele Daiana Poleti; Raul Machado Neto; Ricardo Augusto Brassaloti; Ricardo Haddad; Rodrigo Tocantins Calado. FAMERP-SJRP: Cecília Artico Banho; Sandra Coccuzzo Sampaio; Svetoslav Nanev Slavov; Vagner Fonseca; Vincent Louis Viala
EPI_ISL_1795161, EPI_ISL_2345372	CS III VILA ODILON	Instituto Butantan / ESALQ-Piracicaba	Antonio Jorge Martins; Bianca Cechetto Carlos. Mendelics: Bibiana Santos; Claudia Renata dos Santos Barros; David Schlesinger. Hemocentro Ribeirão Preto: Simone Kashima; Debora Botequio Moretti. Centro de Genômica Funcional da ESALQ: Luiz Lehmann Coutinho; Dimas Tadeu Covas; Elaine Cristina Marqueze; Elaine Vieira Santos; Elaine Vieira dos Santos; Elisangela Chicaroni Mattos; Erika Freitas; Evandra Strazza Rodrigues; Felipe Allan da Silva da Costa; Flavia Aburjaile; Guilherme Targino Valente; Heidge Fukumasu; Heidge Fukumasu. USP-Botucatu: Rejane Maria Tommasini Grotto; Instituto Butantan: Alexander Roberto Precioso; Jayme A. Souza-Neto; Jayme Augusto de Souza-Neto; Jessika Cristina Chagas Lesbon; José Salvatore Leister Patané; João Paulo Kitajima; Luiz Alcantara; Luiz Carlos Junior de Alcantara; Luiz Lehmann Coutinho; Maria Carolina Elias; Marta Giovanetti; Maurício Lacerda Nogueira; Patricia Akemi Assato; Rafael dos Santos Bezerra; Raquel de Lello Rocha Campos Cassano. NGS Soluções Genômicas: Pilar Drummond Sampaio Corrêa Mariani. FZEA-USP Pirassununga: Mirele Daiana Poleti; Raul Machado Neto; Rejane Maria Tommasini Grotto; Ricardo Augusto Brassaloti; Ricardo Haddad; Rodrigo Tocantins Calado.; Sandra Coccuzzo Sampaio; Sandra Coccuzzo Sampaio Vessoni; Simone Kashima; Svetoslav Nanev Slavov; Vagner Fonseca; Vincent Louis Viala
EPI_ISL_1628366, EPI_ISL_1715142	CS III de Patrocinio Paulista	Instituto Adolfo Lutz, Interdiciplinary Procedures Center,	Caio Vinicius Dias Lopes; Claudia Regina Gonçalves; Claudio Tavares Sacchi; Erica Valessa Ramos Gomes; Karoline Rodrigues Campos; Katia Correa de Oliveira Santos; Leonardo Jose Tadeu de Araujo

		Strategic Laboratory	
EPI_ISL_1821209, EPI_ISL_2003112	CS de Paulo de Faria	Instituto Adolfo Lutz, Interdiciplinary Procedures Center, Strategic Laboratory	Caio Vinicius Dias Lopes; Claudia Regina Gonçalves; Claudio Tavares Sacchi; Erica Valessa Ramos Gomes; Karoline Rodrigues Campos; Leonardo Jose Tadeu de Araujo
EPI_ISL_3912154	CSF COHAB II	Analytical Competence Molecular Epidemiology Lab/ACME, Oswaldo Cruz Foundation, Ceara (FIOCRUZ CE)	Cleber Furtado Aksenen; Fabio Miyajima; Fernando Braga Stehling; Francisco Eder de Moura Lopes; Jamille Maria Mendes Bezerra; Joaquim Cesar do Nascimento Sousa Junior; Pedro Miguel Carneiro Jeronimo; Suzana Porto Almeida & Lucas Delerino on behalf of COVID-19 FIOCRUZ Genomic Network; Thais Ferreira de Oliveira; Thais de Oliveira Costa; Ticiane Cavalcante de Souza; Veridiana Pessoa Miyajima
EPI_ISL_3912156	CSF SINHA SABOIA	Analytical Competence Molecular Epidemiology Lab/ACME, Oswaldo Cruz Foundation, Ceara (FIOCRUZ CE)	Cleber Furtado Aksenen; Fabio Miyajima; Fernando Braga Stehling; Francisco Eder de Moura Lopes; Jamille Maria Mendes Bezerra; Joaquim Cesar do Nascimento Sousa Junior; Pedro Miguel Carneiro Jeronimo; Suzana Porto Almeida & Lucas Delerino on behalf of COVID-19 FIOCRUZ Genomic Network; Thais Ferreira de Oliveira; Thais de Oliveira Costa; Ticiane Cavalcante de Souza; Veridiana Pessoa Miyajima
EPI_ISL_3912157	CSF TAPERUABA	Analytical Competence Molecular Epidemiology Lab/ACME, Oswaldo Cruz Foundation, Ceara (FIOCRUZ CE)	Cleber Furtado Aksenen; Fabio Miyajima; Fernando Braga Stehling; Francisco Eder de Moura Lopes; Jamille Maria Mendes Bezerra; Joaquim Cesar do Nascimento Sousa Junior; Pedro Miguel Carneiro Jeronimo; Suzana Porto Almeida & Lucas Delerino on behalf of COVID-19 FIOCRUZ Genomic Network; Thais Ferreira de Oliveira; Thais de Oliveira Costa; Ticiane Cavalcante de Souza; Veridiana Pessoa Miyajima
EPI_ISL_1967004, EPI_ISL_1967006, EPI_ISL_1967007, EPI_ISL_1967008, EPI_ISL_1967010, EPI_ISL_1967011, EPI_ISL_1967012, EPI_ISL_1967013			
see above	CSII DR WASHINGTON LUIS M RODRIGUES DA SILVA PITANGUEIRAS	Instituto Butantan / ESALQ-USP (Piracicaba)	Antonio Jorge Martins; Bianca Cechetto Carlos. Mendelics: Bibiana Santos; Claudia Renata dos Santos Barros; Cíntia Bittar; David Schlesinger. Hemocentro Ribeirão Preto: Simone Kashima; Debora Botequio Moretti; Elaine Cristina Marqueze; Elaine Vieira dos Santos; Elisangela Chicaroni Mattos; Erika Freitas; Evandra Strazza Rodrigues; Felipe Allan da Silva da Costa; Flavia Aburjaile; Fábio Sossai Possebon; Guilherme Campos; Guilherme Targino Valente; Heidge Fukumasu. USP-Botucatu: Rejane Maria Tommasini Grotto; Helena Lage Ferreira; Instituto Butantan: Dimas Tadeu Covas; Jardelina de Souza Todao Bernardino; Jayme A. Souza-Neto; Jessika Cristina Chagas Lesbon; Jorge A. Petroli Marchesi; José Salvatore Leister Patané; João Paulo Kitajima; João Pessoa Araújo Jr.; Leila Sabrina Ullmann; Loyze Paola Oliveira de Lima; Luiz Aurelio de Campos Crispin. Centro de Genômica Funcional da ESALQ: Luiz Lehmann Coutinho; Luiz Carlos Junior de Alcantara; Lívia Sacchetto; Maisa C. Pereira Parra; Maria Carolina Elias; Marta Giovanetti; Marília Moraes; Maurício Lacerda Nogueira. Prefeitura de Sao Paulo: Melissa Palmieri.; Patricia Akemi Assato; Paula Rahal; Paulo Inacio da Costa; Rafael dos Santos Bezerra; Raquel de Lello Rocha Campos Cassano. NGS Soluções Genômicas: Pilar Drummond Sampaio Corrêa Mariani. FZEA-USP Pirassununga: Mirele Daiana Poleti; Raul Machado Neto; Ricardo Augusto Brassaloti; Ricardo Haddad; Rodrigo Tocantins Calado. FAMERP-SJRP: Cecília Artico Banho; Sandra Coccuzzo Sampaio; Svetoslav Nanev Slavov; Vagner Fonseca; Vincent Louis Viala
EPI_ISL_2105160	CT-Dr. Katherine A. Kelley State Public Health Lab	Centers for Disease Control and Prevention Division of Viral Diseases, Pathogen Discovery	Alison Laufer Halpin; Ben L. Rambo-Martin; Clinton R. Paden; Dakota Howard; Darlene Wagner; Dave Wentworth; Dhwani Batra; Jasmine Padilla; Justin Lee; Katie Dillon; Krista Queen; Kristen Knipe; Kristine Lacek; Mark Burroughs; Matthew Schmerer; Mili Sheth; Peter Cook; Sam Shepard; Sarah Nobles; Shoshona Le; Suxiang Tong; Vivien Dugan; Yvette Unoarumhi
EPI_ISL_2234349, EPI_ISL_2537462	California Department of Public Health	California Department of Public Health	CDPH IDLB COVIDNet et al
EPI_ISL_2837494, EPI_ISL_2837516, EPI_ISL_2837724, EPI_ISL_2837733, EPI_ISL_2837789, EPI_ISL_2837807, EPI_ISL_2837808, EPI_ISL_2837809, EPI_ISL_2837817, EPI_ISL_2837820, EPI_ISL_2837908			
see above	California Department of Public Health Valencia Branch Laboratory (CDPH VBL)	California Department of Public Health	CDPH-COVIDNet
EPI_ISL_1731575, EPI_ISL_1752638	Casa de Caridade Sao Vicente de Paulo Cajuru	Instituto Adolfo Lutz, Interdiciplinary Procedures Center, Strategic Laboratory	Caio Vinicius Dias Lopes; Claudia Regina Gonçalves; Claudio Tavares Sacchi; Erica Valessa Ramos Gomes; Karoline Rodrigues Campos; Katia Correa de Oliveira Santos; Leonardo Jose Tadeu de Araujo
EPI_ISL_1067728, EPI_ISL_1067736	Center for Biotechnology and Cell Therapy, São Rafael Hospital, Salvador, Brazil	Central Public Health Laboratory - LACEN - Bahia, Salvador, Brazil	Arabela Leal; Breno Dominguez; Felicidade Pereira; Jaqueline Gomes; Luciana Oliveira; Luiz Alcantara; Marcela Gómez; Marta Giovanetti; Patrícia Cajado; Stephane Tosta; Vagner Fonseca; Vanessa Nardy
EPI_ISL_1760267, EPI_ISL_1760280, EPI_ISL_1760281, EPI_ISL_1760284	Centers for Disease Control and Prevention, Dengue Branch	Centers for Disease Control and Prevention, Dengue Branch	Betzabel Flores; Gabriela Paz-Bailey; Gilberto A. Santiago; Glenda Gonzalez; Jorge L. Munoz-Jordan; Keyla Charriez
EPI_ISL_2108582	Centogene; Dr. Bauer Laboratoriums GmbH	Robert Koch Institute	
EPI_ISL_984619, EPI_ISL_984620, EPI_ISL_984621	Central Laboratory of Public Health of Rio Grande do Sul (Lacen-RS)	State Center for Health Surveillance of the Health Department of the State of Rio Grande do Sul (CEVS/SES-RS)	Aline Campos; Cynthia Molina; Lara Crescente; Leticia Garay; Ludmila Fiorenzano Baethgen; Richard Salvato; Tatiana Gregianini
EPI_ISL_2502442, EPI_ISL_2502443, EPI_ISL_2502444, EPI_ISL_2502445, EPI_ISL_2502446, EPI_ISL_2502447, EPI_ISL_2502448, EPI_ISL_2502449, EPI_ISL_2502450, EPI_ISL_2502451, EPI_ISL_2502452, EPI_ISL_2502453, EPI_ISL_2502454, EPI_ISL_2502455, EPI_ISL_2502456, EPI_ISL_2502457, EPI_ISL_2502458, EPI_ISL_2502459, EPI_ISL_2502460, EPI_ISL_2502461, EPI_ISL_2502462, EPI_ISL_2502463, EPI_ISL_2502464, EPI_ISL_2502465, EPI_ISL_2502466, EPI_ISL_2502467, EPI_ISL_2502468, EPI_ISL_2502469, EPI_ISL_2502470, EPI_ISL_2502471, EPI_ISL_2502472, EPI_ISL_2502473, EPI_ISL_2502474, EPI_ISL_2502475, EPI_ISL_2549287			
see above	Central Laboratory, Bureau of Public Health (BOG) and Academic Hospital Paramaribo	Erasmus Medical Center	Bas B Oude Munnink; Cherise Beek; Consuella Partowidjojo; Dion Gajadin; Ed PF IJzerman; Emmanuelle Munger; Gary Gummels; Ingrid SK Krishnadath; Lycke Woittiez; Marion PG Koopmans; Mireille Van de Veer; Phyllis Pinas; Princes Wongsowidjojo; Radjesh Ori; Ranisha Doerbalie; Rohma Banwari; Soeradj Harkisoen; Stephen Vreden; Tilotmadebie Ramlal; Verne Nanhoe
EPI_ISL_1067729, EPI_ISL_1067730, EPI_ISL_1067731, EPI_ISL_1067733, EPI_ISL_1067734, EPI_ISL_1067735, EPI_ISL_1067737, EPI_ISL_1067738, EPI_ISL_1583650, EPI_ISL_1583656, EPI_ISL_1583665, EPI_ISL_1583673, EPI_ISL_1583674, EPI_ISL_1583676, EPI_ISL_1583677, EPI_ISL_1583679, EPI_ISL_1583680, EPI_ISL_1583681, EPI_ISL_1583682, EPI_ISL_1583683, EPI_ISL_1583694, EPI_ISL_1583697, EPI_ISL_1583700, EPI_ISL_1583719, EPI_ISL_1583722, EPI_ISL_1583725, EPI_ISL_1583730, EPI_ISL_1583733, EPI_ISL_3255057, EPI_ISL_3255058, EPI_ISL_3255061, EPI_ISL_3255064, EPI_ISL_3255077, EPI_ISL_3255078, EPI_ISL_3255079, EPI_ISL_3255134, EPI_ISL_3255187, EPI_ISL_3255193, EPI_ISL_3255195, EPI_ISL_3266079, EPI_ISL_3266081, EPI_ISL_3266087, EPI_ISL_3266089, EPI_ISL_3266090, EPI_ISL_3266091, EPI_ISL_3266092, EPI_ISL_3266094, EPI_ISL_3266099, EPI_ISL_3266103, EPI_ISL_3266106, EPI_ISL_3266107, EPI_ISL_3266108, EPI_ISL_3266109, EPI_ISL_3266110, EPI_ISL_3266111, EPI_ISL_3266112, EPI_ISL_3266113, EPI_ISL_3266114, EPI_ISL_3266117, EPI_ISL_3266118, EPI_ISL_3944576			
see above	Central Public Health Laboratory - LACEN - Bahia, Salvador, Brazil	Central Public Health Laboratory - LACEN - Bahia, Salvador, Brazil	Arabela Leal; Breno Dominguez; Felicidade Pereira; Gabriela Menezes; Jaqueline Gomes; Lenisa Dandara; Luciana Oliveira; Luiz Alcantara; Marcela Gómez; Marta Giovanetti; Patrícia Cajado; Stephane Tosta; Vagner Fonseca; Vanessa Nardy
EPI_ISL_1547454	Centre De Prelevement COVID RIOM	CHU Clermont-Ferrand, service de virologie	Bisseux Maxime; Combes Patricia; Henquell Cécile; Mirand Audrey
EPI_ISL_1483773, EPI_ISL_1623086	Centre Hospitalier Universitaire Clermont-Ferrand	CHU Clermont-Ferrand, service de virologie	Bisseux Maxime; Combes Patricia; Henquell Cécile; Mirand Audrey
EPI_ISL_1628377	Centro De Saude II Ibitinga	Instituto Adolfo Lutz, Interdiciplinary Procedures Center, Strategic Laboratory	Caio Vinicius Dias Lopes; Claudia Regina Gonçalves; Claudio Tavares Sacchi; Erica Valessa Ramos Gomes; Karoline Rodrigues Campos; Katia Correa de Oliveira Santos; Leonardo Jose Tadeu de Araujo
EPI_ISL_1628368	Centro Medico Social Comunitario Januario Teodoro de Souza	Instituto Adolfo Lutz, Interdiciplinary Procedures Center, Strategic Laboratory	Caio Vinicius Dias Lopes; Claudia Regina Gonçalves; Claudio Tavares Sacchi; Erica Valessa Ramos Gomes; Karoline Rodrigues Campos; Katia Correa de Oliveira Santos; Leonardo Jose Tadeu de Araujo
EPI_ISL_2274030, EPI_ISL_2274031, EPI_ISL_2274032, EPI_ISL_2274034, EPI_ISL_2274035, EPI_ISL_2274037, EPI_ISL_2274038, EPI_ISL_2274039			
see above	Centro Nacional de Enfermidades Tropicales (CENETROP)	Laboratory of Respiratory Viruses and Measles, Oswaldo Cruz Institute, FIOCRUZ	Alice Sampaio Rocha; Ana Carolina Mendonca; Anna Carolina Paixao; Cinthia Avila; Elisa Cavalcante Pereira; Fernando Motta; Luciana Appolinario; Marilda Siqueira on behalf of the Fiocruz COVID-19 Genomic Surveillance Network; Paola Resende; Renata Serrano Lopes; Roxana Loayza; Taina Venas
EPI_ISL_2612325, EPI_ISL_2612358, EPI_ISL_2612363, EPI_ISL_2612365, EPI_ISL_2612366, EPI_ISL_2612372, EPI_ISL_2612373, EPI_ISL_2612377, EPI_ISL_2612378, EPI_ISL_2612379, EPI_ISL_2612380, EPI_ISL_2612391, EPI_ISL_2612392, EPI_ISL_2612398, EPI_ISL_2612399, EPI_ISL_2612400, EPI_ISL_2612402, EPI_ISL_2612407, EPI_ISL_2612408			
see above	Centro de Infectologia Charles Mérieux/ Laboratório Rodolphe Mérieux, FUNDHACRE	Bioinformatics Laboratory / LNCC	Alessandra P Lamarca; Alexandra L Gerber; Ana Paula de C Guimarães; Ana Tereza R Vasconcelos; Andreas Stocker; Cirley Maria de Oliveira Lobato; Douglas Terra Machado; Luiz Fellype Alves de Souza; Luiz G P de Almeida; Ronaldo da Silva F Jr
EPI_ISL_2031715, EPI_ISL_2031719, EPI_ISL_2031720, EPI_ISL_2031721, EPI_ISL_2031722, EPI_ISL_2031725, EPI_ISL_2031727, EPI_ISL_2031728, EPI_ISL_2031737, EPI_ISL_2031739, EPI_ISL_2031740, EPI_ISL_2031742, EPI_ISL_2031744			

see above	Centro de Innovación en Vigilancia Epidemiológica (CiVE), Institut Pasteur Montevideo, Uruguay	Centro de Innovación en Vigilancia Epidemiológica (CiVE), Institut Pasteur Montevideo, Uruguay	Alicia Costábile; Alvaro Fajardo; Andrés Lizosaín; Belén González; Bernardina Rivera; Cecilia Alonso; Cecilia Salazar; Gonzalo Moratorio; Gregorio Iraola; Henry Albornoz; Ignacio Ferrés; Inés Bellini; Juan Zanetti; Julio Medina; Lucia Bilbao; Luciana Griffero; Lucía Spangenberg; Ma Noel Bentancor; Ma Pía Techera; Mailen Arleo; Martina Alonso; María José Benítez; Matías Maidana; Mauricio Méndez; Melissa Duquía; Mercedes Paz; Natalia Rego; Natalia Reyes; Odhille Chappos; Paula Perbolianachis; Pilar Moreno; Rodney Colina; Rodrigo Arce; Tamara Fernández; Tania Possi
EPI_ISL_2091312, EPI_ISL_2091317	Centro de Investigación Biomédica de Occidente (CIBO)	Centro de Investigación en Enfermedades Infecciosas (CIENI), Instituto Nacional de Enfermedades Respiratorias (INER)	Alejandro Sanchez-Flores; Alfredo Herrera-Estrella; Alicia Ocaña-Mondragón; Angel Gustavo Salas-Lais; Bernardo Martínez-Miguel; Blanca Taboada; Brenda Irasema Maldonado-Meza; Carla Ivón Herrera-Najera; Carlos F. Arias; Celia Boukadida; Clara Esperanza Santacruz-Tinoco; Concepción Grajales-Muñiz; Consorcio Mexicano de Vigilancia Genómica (CoViGen-Mex). Authors (in alphabetical order): Julio Elias Alvarado-Yaah; Célida Duque Molina; Fernando Fontove-Herrera; Francisco Pulido; Gloria Elena Espinosa-Ayala; Gloria María Molina-Salinas; Gloria Vazquez; Hector Esteban Paz-Juárez; Hector Montoya-Fuentes; Helen Haydee Fernanda Ramírez-Plascencia; José Antonio Enciso-Moreno; José Esteban Muñoz-Medina; José de Jesús Nuñez-Contreras; Juan Bautista Chale-Dzul; Luis Alberto Ochoa-Carrera; Margarita Matías-Florentino; María Guadalupe Santiago-Mauricio; María Guadalupe de Jesús Mireles-Rivera; Nelly Sélem-Mojica; Pavel Isa; Ricardo Grande; Santiago Ávila-Ríos; Victor Eduardo Garcia-Arias; Víctor Hugo Borja-Aburto
EPI_ISL_2402153	Centro de Investigación Biomédica de Occidente (CIBO)	Unidad de Genomica Avanzada	Alejandro Sanchez-Flores; Alfredo Herrera-Estrella; Alicia Ocana-Mondragon; Angel Gustavo Salas-Lais; Bernardo Martinez-Miguel; Blanca Taboada; Brenda Irasema Maldonado-Meza; Carla Ivon Herrera-Najera; Carlos F. Arias; Celia Boukadida; Clara Esperanza Santacruz-Tinoco; Concepcion Grajales-Muniz; Consorcio Mexicano de Vigilancia Genomica (CoViGen-Mex). Authors (in alphabetical order): Julio Elias Alvarado-Yaah; Fernando Fontove-Herrera; Francisco Pulido; Gloria Elena Espinoza-Ayala; Gloria Maria Molina-Salinas; Gloria Vazquez; Hector Esteban Paz-Juarez; Hector Montoya-Fuentes; Helen Haydee Fernanda Ramirez-Plascencia; Jorge Ivan Salinal-Nevarez; Jose Antonio Enciso-Moreno; Jose Esteban Munoz-Medina; Jose de Jesus Nunez-Contreras; Juan Bautista Chale-Dzul; Luis Alberto Ochoa-Carrera; Margarita Matias-Florentino; Maria Guadalupe Santiago-Mauricio; Maria Guadalupe de Jesus Mireles-Rivera; Nelly Selem-Mojica; Pavel Isa; Ricardo Grande; Santiago Avila-Rios; Victor Hugo Borja-Aburto
EPI_ISL_1279276	Centro de Investigación Biomédica del Noreste (CIBIN)	Instituto Nacional de Enfermedades Respiratorias (INER): Centro de Investigación en Enfermedades Infecciosas (CIENI)	Alejandro Sanchez-Flores; Alfredo Herrera-Estrella; Alicia Ocaña-Mondragón; Angel Gustavo Salas-Lais; Bernardo Martínez-Miguel; Blanca Taboada; Brenda Irasema Maldonado-Meza; Carla Ivón Herrera-Najera; Carlos F. Arias; Celia Boukadida; Clara Esperanza Santacruz-Tinoco; Concepción Grajales-Muñiz; Consorcio Mexicano de Vigilancia Genómica (CoViGen-Mex). Authors (in alphabetical order): Julio Elias Alvarado-Yaah; Fernando Fontove-Herrera; Francisco Pulido; Gloria Elena Espinoza-Ayala; Gloria María Molina-Salinas; Gloria Vazquez; Hector Esteban Paz-Juárez; Hector Montoya-Fuentes; Helen Haydee Fernanda Ramírez-Plascencia; Jorge Ivan Salinal-Nevarez; José Antonio Enciso-Moreno; José Esteban Muñoz-Medina; José de Jesús Nuñez-Contreras; Juan Bautista Chale-Dzul; Luis Alberto Ochoa-Carrera; Margarita Matías-Florentino; María Guadalupe Santiago-Mauricio; María Guadalupe de Jesús Mireles-Rivera; Nelly Sélem-Mojica; Pavel Isa; Ricardo Grande; Santiago Ávila-Ríos; Víctor Hugo Borja-Aburto
EPI_ISL_2491713, EPI_ISL_2491714, EPI_ISL_2491715, EPI_ISL_2491716, EPI_ISL_2491717, EPI_ISL_2491718	Centro de Pesquisa Gonçalo Moniz (CPqGM - FIOCRUZ/BA)	Laboratory of Respiratory Viruses and Measles, Oswaldo Cruz Institute, FIOCRUZ	Alice Sampaio Rocha; Ana Carolina Mendonca; Anna Carolina Paixao; Camila I. de Oliveira; Elisa Cavalcante Pereira; Fernando Motta; Luciana Appolinario; Marilda Siqueira on behalf of the Fiocruz COVID-19 Genomic Surveillance Network; Paola Resende; Renata Serrano Lopes; Ricardo Khouri; Taina Venas
EPI_ISL_1171648, EPI_ISL_1171649, EPI_ISL_1171650	Centro de Saude Dr. Jose Paione em Mococa	Instituto Adolfo Lutz, Interdiciplinary Procedures Center, Strategic Laboratory	Caio Vinicius Dias Lopes; Claudia Regina Gonçalves; Claudio Tavares Sacchi; Erica Valessa Ramos Gomes; Karoline Rodrigues Campos
EPI_ISL_1520129	Centro de Saude II Dr Jose Paione Mococa	Instituto Adolfo Lutz, Interdiciplinary Procedures Center, Strategic Laboratory	Caio Vinicius Dias Lopes; Claudia Regina Gonçalves; Claudio Tavares Sacchi; Erica Valessa Ramos Gomes; Karoline Rodrigues Campos
EPI_ISL_1196296	Centro de Saude II Dr. Jose Paione Mococa	Instituto Adolfo Lutz, Interdiciplinary Procedures Center, Strategic Laboratory	Caio Vinicius Dias Lopes; Claudia Regina Gonçalves; Claudio Tavares Sacchi; Erica Valessa Ramos Gomes; Karoline Rodrigues Campos
EPI_ISL_1715134	Centro de Saude II Ibitinga	Instituto Adolfo Lutz, Interdiciplinary Procedures Center, Strategic Laboratory	Caio Vinicius Dias Lopes; Claudia Regina Gonçalves; Claudio Tavares Sacchi; Erica Valessa Ramos Gomes; Karoline Rodrigues Campos; Katia Correa de Oliveira Santos; Leonardo Jose Tadeu de Araujo
EPI_ISL_1468427	Centro de Saude II Matao	Instituto Adolfo Lutz, Interdiciplinary Procedures Center, Strategic Laboratory	Caio Vinicius Dias Lopes; Claudia Regina Gonçalves; Claudio Tavares Sacchi; Erica Valessa Ramos Gomes; Karoline Rodrigues Campos
EPI_ISL_1358285	Centro de Treinamento e Referencia DST AIDS	Instituto Adolfo Lutz, Interdiciplinary Procedures Center, Strategic Laboratory	Caio Vinicius Dias Lopes; Claudia Regina Gonçalves; Claudio Tavares Sacchi; Erica Valessa Ramos Gomes; Karoline Rodrigues Campos
EPI_ISL_1005684	Cerballiance Monthléry	Cerba	Benazra M; Collin T; Delaplace E; Gresset N; Haim-Boukobza S; Lecorche E; Lesenne A; Olivi M; Ricard B; Roquebert B; Trombert-Paolantoni S
EPI_ISL_1005685	Cerballiance Montlhéry	Cerba	Benazra M; Collin T; Delaplace E; Gresset N; Haim-Boukobza S; Lecorche E; Lesenne A; Olivi M; Ricard B; Roquebert B; Trombert-Paolantoni S
EPI_ISL_1738812, EPI_ISL_1738813, EPI_ISL_1854239	Cintramedica	Instituto Nacional de Saude (INSA)	Borges et al
EPI_ISL_2234845	City of Milwaukee Health Department Laboratory	City of Milwaukee Health Department Laboratory	Amy Bauer; Jennifer Lentz; Manjeet Khubbar; Samantha Scott; Sanjib Bhattacharyya
EPI_ISL_1164183	Clinical Microbiology, Nationwide Children's Hospital	James Molecular Laboratory	Amy Leber; Dan Jones; Huanyu Wang; Pam Snyder; Zachary Rudy
EPI_ISL_1664592, EPI_ISL_2098779	Clinical Molecular Microbiology Laboratory, UNC Hospitals	Jeremy Wang	Alexander Rubinsteyn; Brianna de la Houssaye; Colleen Rice; Corbin Jones; Jason Smedberg; Jeremy Wang; Jose Luis Torres Castillo; Melissa Miller; Robert Hagan; Shawn Hawken
EPI_ISL_1617331, EPI_ISL_1617332, EPI_ISL_1665881, EPI_ISL_2195081	Colorado Department of Public Health and Environment	Colorado Department of Public Health and Environment	Alexandria Rossheim; Diana Ir; Emily A. Travanty; Laura Bankers; Molly C. Hetherington-Rauth; Sarah Elizabeth Totten; Shannon Ely; Shannon R. Matzinger
EPI_ISL_1582265, EPI_ISL_1582267, EPI_ISL_1755861, EPI_ISL_1855286	Columbia University Irving Medical Center	Wadsworth Center, New York State Department of Health	Alexis Russell; Catharine Prussing; Daryl M. Lamson; Erasmus Schneider; Erica Lasek-Nesselquist; John Kelly; Jonathan Plitnick; Kirsten St. George; Matthew Shudt; Melissa A Leisner; Navjot Singh
EPI_ISL_1595844	Compass Laboratory Services, LLC	Compass Laboratory Services	PhD; William Budd
EPI_ISL_872191, EPI_ISL_872192, EPI_ISL_1381068	Conjunto Hospitalar do Mandaqui de Sao Paulo	Instituto Adolfo Lutz, Interdiciplinary Procedures Center, Strategic Laboratory	Ana Lucia de Carvalho Avelino; Caio Vinicius Dias Lopes; Claudia Regina Gonçalves; Claudio Tavares Sacchi; Erica Valessa Ramos Gomes; Fabiana Cristina Pereira dos Santos; Karoline Rodrigues Campos; Katia Correa de Oliveira Santos
EPI_ISL_2034730	Connecticut DPH	Yale Center for Genomic Analysis	Anderson Brito; Annie Watkins; Anthony Muyombwe; Brooke Sullivan; Chaney Kalinich; Chantal Vogels; Curt Scharfe; Irina Tikhonova; Isabel Ott; Jafar Razeq; Jessica Rothman; Joseph Fauver; Kaya Bilguvar; Mallery Breban; Mary Petrone; Nathan Grubaugh; Randy Downing; Shrikant Mane; Stephen M. Bart; Tara Alpert
EPI_ISL_2497667	Contra Costa County Public Health Lab	Chan-Zuckerberg Biohub	CZB Cliahub Consortium
EPI_ISL_2102890	County of San Luis Obispo Public Health Laboratory	Chan-Zuckerberg Biohub	CZB Cliahub Consortium
EPI_ISL_2658458, EPI_ISL_2658466	Croatian Institute of Public Health	Croatian Institute of Public Health	Irena Tabain; Ivana Ferenčak
EPI_ISL_1575080	Cruces University Hospital	Biocruces Bizkaia	Ana Belen de la Hoz Rastrollo; Mikel Gallego Rodrigo
EPI_ISL_3243124, EPI_ISL_3452406	Curative Labs	New Mexico Department of Health Scientific Laboratory	Anastacia Griego-Fisher; D'eldra Malone; Ellie Johnson; Jennifer Benoit; Justin Griego; Linda Salazar; Linda Salzar; Michael Sam; Monica Manginell; Ratheesh Rajan; T. Mark Willmon
EPI_ISL_3664118, EPI_ISL_3664123,	DASA	DASA	Adriano Bonaldi; Angelica Hristov; Annelise Lopes; Bianca Cota; Cristina Oliveira; Jose Levi; Lidia Yamamoto; Paulo Pierry; Rodrigo Guarischi; Rodrigo Salazar

EPI_ISL_3664137			
EPI_ISL_833167, EPI_ISL_833169, EPI_ISL_833170, EPI_ISL_833171, EPI_ISL_833172, EPI_ISL_833173, EPI_ISL_833174			
see above	DB Diagnosticos do Brasil	Instituto Adolfo Lutz, Interdiciplinary Procedures Center, Strategic Laboratory	Claudia Regina Gonçalves; Claudio Tavares Sacchi; Erica Valessa Ramos Gomes; Karoline Rodrigues Campos
EPI_ISL_3482011	DC Public Health Lab/ Dept. of Forensic Sciences	DC Public Health Lab/ Dept. of Forensic Sciences	Brittany Hamilton; Connie Maza; David Payne; Elizabeth Zelaya; Janis Doss; Jocelyn Hauser; Monica Mann; Sarah Scott; Scott Nguyen
EPI_ISL_2209152	DEPARTAMENTO DE SAUDE COLETIVA	Instituto Butantan	Antonio Jorge Martins; Claudia Renata dos Santos Barros; David Schlesinger; Debora Botequio Moretti; Dimas Tadeu Covas; Elaine Cristina Marqueze; Elaine Vieira Santos; Evandra Strazza Rodrigues; Heidge Fukumasu; Jayme Augusto de Souza-Neto; José Salvatore Leister Patané; Luiz Alcantara; Luiz Lehmann Coutinho; Maria Carolina Elias; Maurício Lacerda Nogueira; Rafael dos Santos Bezerra; Raul Machado Neto; Rejane Maria Tommasini Grotto; Ricardo Haddad; Sandra Coccuzzo Sampaio Vessoni; Simone Kashima; Svetoslav Nanev Slavov; Vincent Louis Viala.
EPI_ISL_1967197	DEPARTAMENTO DE SAUDE COLETIVA	Instituto Butantan / ESALQ-USP (Piracicaba)	Antonio Jorge Martins; Bianca Cechetto Carlos. Mendelics: Bibiana Santos; Claudia Renata dos Santos Barros; Cíntia Bittar; David Schlesinger. Hemocentro Ribeirão Preto: Simone Kashima; Debora Botequio Moretti; Elaine Cristina Marqueze; Elaine Vieira dos Santos; Elisangela Chicaroni Mattos; Erika Freitas; Evandra Strazza Rodrigues; Felipe Allan da Silva da Costa; Flavia Aburjaile; Fábio Sossai Possebon; Guilherme Campos; Guilherme Targino Valente; Heidge Fukumasu. USP-Botucatu: Rejane Maria Tommasini Grotto; Helena Lage Ferreira; Instituto Butantan: Dimas Tadeu Covas; Jardelina de Souza Todao Bernardino; Jayme A. Souza-Neto; Jessika Cristina Chagas Lesbon; Jorge A. Petroli Marchesi; José Salvatore Leister Patané; João Paulo Kitajima; João Pessoa Araújo Jr.; Leila Sabrina Ullmann; Loyze Paola Oliveira de Lima; Luiz Aurelio de Campos Crispin. Centro de Genômica Funcional da ESALQ: Luiz Lehmann Coutinho; Luiz Carlos Junior de Alcantara; Lívia Sacchetto; Maisa C. Pereira Parra; Maria Carolina Elias; Marta Giovanetti; Marília Moraes; Maurício Lacerda Nogueira. Prefeitura de Sao Paulo: Melissa Palmieri.; Patricia Akemi Assato; Paula Rahal; Paulo Inacio da Costa; Rafael dos Santos Bezerra; Raquel de Lello Rocha Campos Cassano. NGS Soluções Genômicas: Pilar Drummond Sampaio Corrêa Mariani. FZEA-USP Pirassununga: Mirele Daiana Poleti; Raul Machado Neto; Ricardo Augusto Brassaloti; Ricardo Haddad; Rodrigo Tocantins Calado. FAMERP-SJRP: Cecília Artico Banho; Sandra Coccuzzo Sampaio; Svetoslav Nanev Slavov; Vagner Fonseca; Vincent Louis Viala
EPI_ISL_2344545	DEPARTAMENTO DE SAUDE COLETIVA	Instituto Butantan / UNESP-Botucatu	Antonio Jorge Martins; Claudia Renata dos Santos Barros; David Schlesinger; Debora Botequio Moretti; Dimas Tadeu Covas; Elaine Cristina Marqueze; Elaine Vieira Santos; Evandra Strazza Rodrigues; Heidge Fukumasu; Jayme Augusto de Souza-Neto; José Salvatore Leister Patané; Luiz Alcantara; Luiz Lehmann Coutinho; Maria Carolina Elias; Maurício Lacerda Nogueira; Rafael dos Santos Bezerra; Raul Machado Neto; Rejane Maria Tommasini Grotto; Ricardo Haddad; Sandra Coccuzzo Sampaio Vessoni; Simone Kashima; Svetoslav Nanev Slavov; Vincent Louis Viala
EPI_ISL_1795182, EPI_ISL_2345400	DEPARTAMENTO DE SAUDE MUNICIPAL SOCORRO SP	Instituto Butantan / ESALQ-Piracicaba	Antonio Jorge Martins; Bianca Cechetto Carlos. Mendelics: Bibiana Santos; Claudia Renata dos Santos Barros; David Schlesinger; David Schlesinger. Hemocentro Ribeirão Preto: Simone Kashima; Debora Botequio Moretti; Debora Botequio Moretti. Centro de Genômica Funcional da ESALQ: Luiz Lehmann Coutinho; Dimas Tadeu Covas; Elaine Cristina Marqueze; Elaine Vieira Santos; Elaine Vieira dos Santos; Elisangela Chicaroni Mattos; Erika Freitas; Evandra Strazza Rodrigues; Felipe Allan da Silva da Costa; Flavia Aburjaile; Guilherme Targino Valente; Heidge Fukumasu; Heidge Fukumasu. USP-Botucatu: Rejane Maria Tommasini Grotto; Instituto Butantan: Alexander Roberto Precioso; Jayme A. Souza-Neto; Jayme Augusto de Souza-Neto; Jessika Cristina Chagas Lesbon; José Salvatore Leister Patané; João Paulo Kitajima; Luiz Alcantara; Luiz Carlos Junior de Alcantara; Luiz Lehmann Coutinho; Maria Carolina Elias; Marta Giovanetti; Maurício Lacerda Nogueira; Patricia Akemi Assato; Rafael dos Santos Bezerra; Raquel de Lello Rocha Campos Cassano. NGS Soluções Genômicas: Pilar Drummond Sampaio Corrêa Mariani. FZEA-USP Pirassununga: Mirele Daiana Poleti; Raul Machado Neto; Rejane Maria Tommasini Grotto; Ricardo Augusto Brassaloti; Ricardo Haddad; Rodrigo Tocantins Calado.; Sandra Coccuzzo Sampaio; Sandra Coccuzzo Sampaio Vessoni; Simone Kashima; Svetoslav Nanev Slavov; Vagner Fonseca; Vincent Louis Viala
EPI_ISL_2362237	DIRETORIA MUNICIPAL DE SAUDE DE ENGENHEIRO COELHO	Instituto Butantan / FZEA-USP-Pirassununga	Antonio Jorge Martins; Claudia Renata dos Santos Barros; David Schlesinger; Debora Botequio Moretti; Dimas Tadeu Covas; Elaine Cristina Marqueze; Elaine Vieira Santos; Evandra Strazza Rodrigues; Heidge Fukumasu; Jayme Augusto de Souza-Neto; José Salvatore Leister Patané; Luiz Alcantara; Luiz Lehmann Coutinho; Maria Carolina Elias; Maurício Lacerda Nogueira; Rafael dos Santos Bezerra; Raul Machado Neto; Rejane Maria Tommasini Grotto; Ricardo Haddad; Sandra Coccuzzo Sampaio Vessoni; Simone Kashima; Svetoslav Nanev Slavov; Vincent Louis Viala
EPI_ISL_2820233	DNAGYN	LGBio - Laboratório de Genética e Biodiversidade	Alex Honda Bernardes; Amanda Alves de Melo; Aparecido Divino da Cruz; Cintia Pelegrineti Targueta de Azevedo Brito; Daniela de Melo e Silva; Elisangela de Paula Silveira Lacerda; Juliana Santana de Curcio; Luiz Augusto Pereira; Marc Alexandre Duarte Gigonzac; Mariana Pires de Campos Telles; Ramilla dos Santos Braga; Renata de Oliveira Dias; Rhewter Nunes; Thais Cidália Vieira Gigonzac; Thais Guimarães Castro; Thays Millena Alves Pedroso
EPI_ISL_1583675	DNA Laboratory	Central Public Health Laboratory - LACEN - Bahia, Salvador, Brazil	Arabela Leal; Breno Dominguez; Felicidade Pereira; Jaqueline Gomes; Luciana Oliveira; Luiz Alcantara; Marcela Gómez; Marta Giovanetti; Patrícia Cajado; Stephane Tosta; Vagner Fonseca; Vanessa Nardy
EPI_ISL_1447144	DOHMH Corona	New York City Public Health Laboratory	Jade Wang; et al.
EPI_ISL_2262606	DOHMH PHL	New York City Public Health Laboratory	Jade Wang; et al.
EPI_ISL_1908950	DPHL	Delaware Public Health Lab	Rebecca Savage
EPI_ISL_1012923	Department of Infectious Diseases, Istituto Superiore di Sanità, Rome, Italy; ASST Sette Laghi, Varese, Italy	Istituto Superiore di Sanità (ISS)	Alessandra Lo Presti; Andreina Baj; Angela Di Martino; Angelo Genoni; Fabrizio Maggi; Federica Novazzi; Manuela Marra; Marco Crescenzi; Maria Carollo; Paola Stefanelli; Stefano Fiore
EPI_ISL_1864209, EPI_ISL_1868237	Department of Virus and Microbiological Special Diagnostics, Statens Serum Institut, Copenhagen, Denmark	Aalborg University	Danish Covid-19 Genome Consortium
EPI_ISL_1086034, EPI_ISL_1086037, EPI_ISL_1086038, EPI_ISL_1086039, EPI_ISL_1086040, EPI_ISL_1086041, EPI_ISL_1086042, EPI_ISL_1086043, EPI_ISL_1096121, EPI_ISL_1096135			
see above	Diagnosticos da America - DASA	Instituto Adolfo Lutz, Interdiciplinary Procedures Center, Strategic Laboratory	Caio Vinicius Dias Lopes; Claudia Regina Gonçalves; Claudio Tavares Sacchi; Erica Valessa Ramos Gomes; Karoline Rodrigues Campos
EPI_ISL_1821211, EPI_ISL_1821212, EPI_ISL_1821213	Diagnóstico da America S/A	Instituto Adolfo Lutz, Interdiciplinary Procedures Center, Strategic Laboratory	Caio Vinicius Dias Lopes; Claudia Regina Gonçalves; Claudio Tavares Sacchi; Erica Valessa Ramos Gomes; Karoline Rodrigues Campos; Leonardo Jose Tadeu de Araujo
EPI_ISL_1622497	Division of Emerging Infectious Diseases, Bureau of Infectious Diseases Diagnosis Control, Korea Disease Control and Prevention Agency	Division of Emerging Infectious Diseases, Bureau of Infectious Diseases Diagnosis Control, Korea Disease Control and Prevention Agency	Ae Kyung Park; Chae Young Lee; Eun-Jin Kim; Heui Man Kim; Il-Hwan Kim; Jeong-Ah Kim; Jeong-Min Kim; Jin Sun No
EPI_ISL_2145333, EPI_ISL_2145336, EPI_ISL_2145338, EPI_ISL_2145424, EPI_ISL_2145438, EPI_ISL_2145475, EPI_ISL_2145478, EPI_ISL_2145479, EPI_ISL_2145481, EPI_ISL_2145588, EPI_ISL_2145589, EPI_ISL_2145630, EPI_ISL_2145632, EPI_ISL_2449986			
see above	Dutch COVID-19 response team	Erasmus Medical Center	Anne van der Linden; Annemiek van der Eijk; Bas Oude Munnink; Corine GeurtsvanKessel; David Nieuwenhuijse; Emmanuelle Munger; Irina Chestakova; Marion Koopmans; Marjan Boter; Reina Sikkema; Richard Molenkamp; on behalf of the Dutch national COVID-19 respo
EPI_ISL_2030599	Dutch COVID-19 response team	Medical Microbiology, Maastricht University Medical Centre	Brian van der Veer*; Carmen Reumkens; Christian Hoebe; Erik Beuken; Jozef Dingemans*; Lieke van Alphen; Paul Savelkoul
EPI_ISL_943045, EPI_ISL_943046, EPI_ISL_1014338, EPI_ISL_1035203, EPI_ISL_1165565, EPI_ISL_1289219, EPI_ISL_1289220, EPI_ISL_1289221, EPI_ISL_1289222, EPI_ISL_1289224, EPI_ISL_1370577, EPI_ISL_1370615, EPI_ISL_1370834, EPI_ISL_1370893, EPI_ISL_1370903, EPI_ISL_1371081, EPI_ISL_1371282, EPI_ISL_1371513, EPI_ISL_1371702, EPI_ISL_1371705, EPI_ISL_1456631, EPI_ISL_1456632, EPI_ISL_1456635, EPI_ISL_1456640, EPI_ISL_1456641, EPI_ISL_1456642, EPI_ISL_1456643, EPI_ISL_1456644, EPI_ISL_1456645, EPI_ISL_1456653, EPI_ISL_1456654, EPI_ISL_1456655, EPI_ISL_1456659, EPI_ISL_1456660, EPI_ISL_1456661, EPI_ISL_1521295, EPI_ISL_1521296, EPI_ISL_1521297, EPI_ISL_1521298, EPI_ISL_1521299, EPI_ISL_1521300, EPI_ISL_1521301, EPI_ISL_1521302, EPI_ISL_1521303, EPI_ISL_1521304, EPI_ISL_1521306, EPI_ISL_1521321, EPI_ISL_1521322, EPI_ISL_1521323, EPI_ISL_1521324, EPI_ISL_1521325, EPI_ISL_1521326, EPI_ISL_1521327, EPI_ISL_1521328, EPI_ISL_1521331, EPI_ISL_1521332, EPI_ISL_1521337, EPI_ISL_1596022, EPI_ISL_1596027, EPI_ISL_1596162, EPI_ISL_1596280, EPI_ISL_1596609, EPI_ISL_1596777, EPI_ISL_1596936, EPI_ISL_1597044, EPI_ISL_1597233, EPI_ISL_1597244, EPI_ISL_1597245, EPI_ISL_1597256, EPI_ISL_1597268, EPI_ISL_1597291, EPI_ISL_1597302, EPI_ISL_1597313, EPI_ISL_1597412, EPI_ISL_1597424, EPI_ISL_1597436, EPI_ISL_1597446, EPI_ISL_1597522, EPI_ISL_1597525, EPI_ISL_1597590, EPI_ISL_1703183, EPI_ISL_1703184, EPI_ISL_1703193, EPI_ISL_1703204, EPI_ISL_1703214, EPI_ISL_1703215, EPI_ISL_1703225, EPI_ISL_1703226, EPI_ISL_1703251, EPI_ISL_1703252, EPI_ISL_1703253, EPI_ISL_1703260, EPI_ISL_1703269, EPI_ISL_1703279, EPI_ISL_1703289, EPI_ISL_1703313, EPI_ISL_1703324, EPI_ISL_1703330, EPI_ISL_1703336, EPI_ISL_1703620, EPI_ISL_1704983, EPI_ISL_1705088, EPI_ISL_1705097, EPI_ISL_1705115, EPI_ISL_1705388, EPI_ISL_1705398, EPI_ISL_1705409, EPI_ISL_1705413, EPI_ISL_1705414, EPI_ISL_1705443, EPI_ISL_1705477, EPI_ISL_1705502, EPI_ISL_1705513, EPI_ISL_1705525, EPI_ISL_1705536, EPI_ISL_1705556, EPI_ISL_1705566, EPI_ISL_1705853, EPI_ISL_1705864, EPI_ISL_1792429, EPI_ISL_1792430, EPI_ISL_1792431, EPI_ISL_1792433, EPI_ISL_1792435, EPI_ISL_1792436, EPI_ISL_1792438, EPI_ISL_1792447, EPI_ISL_1792448, EPI_ISL_1792449, EPI_ISL_1792450, EPI_ISL_1792451, EPI_ISL_1792452, EPI_ISL_1792453, EPI_ISL_1961155, EPI_ISL_1962254, EPI_ISL_1962256, EPI_ISL_1962272, EPI_ISL_1962273, EPI_ISL_1962274, EPI_ISL_1962275, EPI_ISL_2093857, EPI_ISL_2475704			
see above	Dutch COVID-19 response team	National Institute for Public Health and the Environment (RIVM)	Adam Meijer; AnneMarie van den Brandt; Annelies Kroneman; Bas van der Veer; Chantal Reusken; Dennis Schmitz; Dirk Eggink; Euníce Then; Florian Zwagemaker; Harry Vennema; James Groot; Jeroen Cremer; Jolienke Hardeman; Karim Hajji; Kim Freriks; Linda van de Nes; Lisa Wijsman; Lynn Aarts; Melissa van Tuil; Robert Kohl; Ryanne Jaarsma; Sanne Bos; Sharon van den Brink; Sjoerd Kuiling; on behalf of the national COVID-19 response team
EPI_ISL_4060096, EPI_ISL_4060097, EPI_ISL_4060098	Dynacare Manitoba	Manitoba Cadham Provincial Laboratory	David Alexander; Hilary Racher; Hongbin Li; Melissa Desaulnier; Tintu Abraham
EPI_ISL_1795245, EPI_ISL_1795246, EPI_ISL_1795247, EPI_ISL_2345478, EPI_ISL_2345480, EPI_ISL_2345481	EMERGENCIA RESPIRATORIA DE NOVA GRANADA	Instituto Butantan / ESALQ-Piracicaba	Antonio Jorge Martins; Bianca Cechetto Carlos. Mendelics: Bibiana Santos; Claudia Renata dos Santos Barros; David Schlesinger; David Schlesinger. Hemocentro Ribeirão Preto: Simone Kashima; Debora Botequio Moretti; Debora Botequio Moretti. Centro de Genômica Funcional da ESALQ: Luiz Lehmann Coutinho; Dimas Tadeu Covas; Elaine Cristina Marqueze; Elaine Vieira Santos; Elaine Vieira dos Santos; Elisangela Chicaroni Mattos; Erika Freitas; Evandra Strazza Rodrigues; Felipe Allan da Silva da Costa; Flavia Aburjaile; Guilherme Targino Valente; Heidge Fukumasu; Heidge Fukumasu. USP-Botucatu: Rejane Maria Tommasini Grotto; Instituto Butantan: Alexander Roberto Precioso; Jayme A. Souza-Neto; Jayme Augusto de Souza-Neto; Jessika Cristina Chagas Lesbon; José Salvatore Leister Patané; João Paulo Kitajima; Luiz Alcantara; Luiz Carlos Junior de Alcantara; Luiz Lehmann Coutinho; Maria Carolina Elias; Marta Giovanetti; Maurício Lacerda Nogueira; Patricia Akemi Assato; Rafael dos Santos Bezerra; Raquel de Lello Rocha Campos Cassano. NGS Soluções Genômicas: Pilar Drummond Sampaio Corrêa Mariani. FZEA-USP Pirassununga: Mirele Daiana Poleti; Raul Machado Neto; Rejane Maria Tommasini Grotto; Ricardo Augusto Brassaloti; Ricardo Haddad; Rodrigo Tocantins Calado.; Sandra Coccuzzo Sampaio; Sandra Coccuzzo Sampaio Vessoni; Simone Kashima; Svetoslav Nanev Slavov; Vagner Fonseca; Vincent Louis Viala
EPI_ISL_1966533	EMERGENCIA RESPIRATORIA DE NOVA GRANADA	Instituto Butantan / FZEA-USP (Pirassununga)	Antonio Jorge Martins; Bianca Cechetto Carlos. Mendelics: Bibiana Santos; Claudia Renata dos Santos Barros; Cíntia Bittar; David Schlesinger. Hemocentro Ribeirão Preto: Simone Kashima; Debora Botequio Moretti; Elaine Cristina Marqueze; Elaine Vieira dos Santos; Elisangela Chicaroni Mattos; Erika Freitas; Evandra Strazza Rodrigues; Felipe Allan da Silva da Costa; Flavia Aburjaile; Fábio Sossai Possebon; Guilherme Campos; Guilherme Targino Valente; Heidge Fukumasu. USP-Botucatu: Rejane Maria Tommasini Grotto; Helena Lage Ferreira; Instituto Butantan: Dimas Tadeu Covas; Jardelina de Souza Todao Bernardino; Jayme A. Souza-Neto; Jessika Cristina Chagas Lesbon; Jorge A. Petroli Marchesi; José Salvatore Leister Patané; João Paulo Kitajima; João Pessoa Araújo Jr.; Leila Sabrina Ullmann; Loyze Paola Oliveira de Lima; Luiz Aurelio de Campos Crispin. Centro de Genômica Funcional da ESALQ: Luiz Lehmann Coutinho; Luiz Carlos Junior de Alcantara; Lívia Sacchetto; Maisa C. Pereira Parra; Maria Carolina Elias; Marta Giovanetti; Marília Moraes; Maurício Lacerda Nogueira. Prefeitura de Sao Paulo: Melissa Palmieri.; Patricia Akemi Assato; Paula Rahal; Paulo Inacio da Costa; Rafael dos Santos Bezerra; Raquel de Lello Rocha Campos Cassano. NGS Soluções Genômicas: Pilar Drummond Sampaio Corrêa Mariani. FZEA-USP Pirassununga: Mirele Daiana Poleti; Raul Machado Neto; Ricardo Augusto Brassaloti; Ricardo Haddad; Rodrigo Tocantins Calado. FAMERP-SJRP: Cecília Artico Banho; Sandra Coccuzzo Sampaio; Svetoslav Nanev Slavov; Vagner Fonseca; Vincent Louis Viala
EPI_ISL_1795098,	ESALQ	Instituto Butantan /	Antonio Jorge Martins; Bianca Cechetto Carlos. Mendelics: Bibiana Santos; Claudia Renata dos Santos Barros; David Schlesinger; David Schlesinger. Hemocentro Ribeirão Preto: Simone Kashima; Debora Botequio Moretti; Debora Botequio Moretti. Centro de Genômica Funcional da ESALQ: Luiz Lehmann Coutinho; Dimas Tadeu Covas;

EPI_ISL_1795099, EPI_ISL_2344544, EPI_ISL_2344547		ESALQ-Piracicaba	Elaine Cristina Marqueze; Elaine Vieira Santos; Elaine Vieira dos Santos; Elisangela Chicaroni Mattos; Erika Freitas; Evandra Strazza Rodrigues; Felipe Allan da Silva da Costa; Flavia Aburjaile; Guilherme Targino Valente; Heidge Fukumasu; Heidge Fukumasu. USP-Botucatu: Rejane Maria Tommasini Grotto; Instituto Butantan: Alexander Roberto Precioso; Jayme A. Souza-Neto; Jayme Augusto de Souza-Neto; Jessika Cristina Chagas Lesbon; José Salvatore Leister Patané; João Paulo Kitajima; Luiz Alcantara; Luiz Carlos Junior de Alcantara; Luiz Lehmann Coutinho; Maria Carolina Elias; Marta Giovanetti; Maurício Lacerda Nogueira; Patricia Akemi Assato; Rafael dos Santos Bezerra; Raquel de Lello Rocha Campos Cassano. NGS Soluções Genômicas: Pilar Drummond Sampaio Corrêa Mariani. FZEA-USP Pirassununga: Mirele Daiana Poleti; Raul Machado Neto; Rejane Maria Tommasini Grotto; Ricardo Augusto Brassaloti; Ricardo Haddad; Rodrigo Tocantins Calado.; Sandra Coccuzzo Sampaio; Sandra Coccuzzo Sampaio Vessoni; Simone Kashima; Svetoslav Nanev Slavov; Vagner Fonseca; Vincent Louis Viala
EPI_ISL_1966251	ESF DR LUIS ERNESTO SANDI MORI JALES	Instituto Butantan / Mendelics	Antonio Jorge Martins; Bianca Cechetto Carlos. Mendelics: Bibiana Santos; Claudia Renata dos Santos Barros; Cíntia Bittar; David Schlesinger. Hemocentro Ribeirão Preto: Simone Kashima; Debora Botequio Moretti; Elaine Cristina Marqueze; Elaine Vieira dos Santos; Elisangela Chicaroni Mattos; Erika Freitas; Evandra Strazza Rodrigues; Felipe Allan da Silva da Costa; Flavia Aburjaile; Fábio Sossai Possebon; Guilherme Campos; Guilherme Targino Valente; Heidge Fukumasu. USP-Botucatu: Rejane Maria Tommasini Grotto; Helena Lage Ferreira; Instituto Butantan: Dimas Tadeu Covas; Jardelina de Souza Todao Bernardino; Jayme A. Souza-Neto; Jessika Cristina Chagas Lesbon; Jorge A. Petroli Marchesi; José Salvatore Leister Patané; João Paulo Kitajima; João Pessoa Araújo Jr.; Leila Sabrina Ullmann; Loyze Paola Oliveira de Lima; Luiz Aurelio de Campos Crispin. Centro de Genômica Funcional da ESALQ: Luiz Lehmann Coutinho; Luiz Carlos Junior de Alcantara; Lívia Sacchetto; Maisa C. Pereira Parra; Maria Carolina Elias; Marta Giovanetti; Marília Moraes; Maurício Lacerda Nogueira. Prefeitura de Sao Paulo: Melissa Palmieri.; Patricia Akemi Assato; Paula Rahal; Paulo Inacio da Costa; Rafael dos Santos Bezerra; Raquel de Lello Rocha Campos Cassano. NGS Soluções Genômicas: Pilar Drummond Sampaio Corrêa Mariani. FZEA-USP Pirassununga: Mirele Daiana Poleti; Raul Machado Neto; Ricardo Augusto Brassaloti; Ricardo Haddad; Rodrigo Tocantins Calado. FAMERP-SJRP: Cecília Artico Banho; Sandra Coccuzzo Sampaio; Svetoslav Nanev Slavov; Vagner Fonseca; Vincent Louis Viala
EPI_ISL_1795376, EPI_ISL_1795377, EPI_ISL_2345952, EPI_ISL_2345953	ESF MINEIROS DO TIETE	Instituto Butantan / ESALQ-Piracicaba	Antonio Jorge Martins; Bianca Cechetto Carlos. Mendelics: Bibiana Santos; Claudia Renata dos Santos Barros; David Schlesinger; David Schlesinger. Hemocentro Ribeirão Preto: Simone Kashima; Debora Botequio Moretti; Debora Botequio Moretti. Centro de Genômica Funcional da ESALQ: Luiz Lehmann Coutinho; Dimas Tadeu Covas; Elaine Cristina Marqueze; Elaine Vieira Santos; Elaine Vieira dos Santos; Elisangela Chicaroni Mattos; Erika Freitas; Evandra Strazza Rodrigues; Felipe Allan da Silva da Costa; Flavia Aburjaile; Guilherme Targino Valente; Heidge Fukumasu; Heidge Fukumasu. USP-Botucatu: Rejane Maria Tommasini Grotto; Instituto Butantan: Alexander Roberto Precioso; Jayme A. Souza-Neto; Jayme Augusto de Souza-Neto; Jessika Cristina Chagas Lesbon; José Salvatore Leister Patané; João Paulo Kitajima; Luiz Alcantara; Luiz Carlos Junior de Alcantara; Luiz Lehmann Coutinho; Maria Carolina Elias; Marta Giovanetti; Maurício Lacerda Nogueira; Patricia Akemi Assato; Rafael dos Santos Bezerra; Raquel de Lello Rocha Campos Cassano. NGS Soluções Genômicas: Pilar Drummond Sampaio Corrêa Mariani. FZEA-USP Pirassununga: Mirele Daiana Poleti; Raul Machado Neto; Rejane Maria Tommasini Grotto; Ricardo Augusto Brassaloti; Ricardo Haddad; Rodrigo Tocantins Calado.; Sandra Coccuzzo Sampaio; Sandra Coccuzzo Sampaio Vessoni; Simone Kashima; Svetoslav Nanev Slavov; Vagner Fonseca; Vincent Louis Viala
EPI_ISL_1795248, EPI_ISL_1795249, EPI_ISL_1795250, EPI_ISL_2345482, EPI_ISL_2345483, EPI_ISL_2345484	ESF NOVA TANABI II	Instituto Butantan / ESALQ-Piracicaba	Antonio Jorge Martins; Bianca Cechetto Carlos. Mendelics: Bibiana Santos; Claudia Renata dos Santos Barros; David Schlesinger; David Schlesinger. Hemocentro Ribeirão Preto: Simone Kashima; Debora Botequio Moretti; Debora Botequio Moretti. Centro de Genômica Funcional da ESALQ: Luiz Lehmann Coutinho; Dimas Tadeu Covas; Elaine Cristina Marqueze; Elaine Vieira Santos; Elaine Vieira dos Santos; Elisangela Chicaroni Mattos; Erika Freitas; Evandra Strazza Rodrigues; Felipe Allan da Silva da Costa; Flavia Aburjaile; Guilherme Targino Valente; Heidge Fukumasu; Heidge Fukumasu. USP-Botucatu: Rejane Maria Tommasini Grotto; Instituto Butantan: Alexander Roberto Precioso; Jayme A. Souza-Neto; Jayme Augusto de Souza-Neto; Jessika Cristina Chagas Lesbon; José Salvatore Leister Patané; João Paulo Kitajima; Luiz Alcantara; Luiz Carlos Junior de Alcantara; Luiz Lehmann Coutinho; Maria Carolina Elias; Marta Giovanetti; Maurício Lacerda Nogueira; Patricia Akemi Assato; Rafael dos Santos Bezerra; Raquel de Lello Rocha Campos Cassano. NGS Soluções Genômicas: Pilar Drummond Sampaio Corrêa Mariani. FZEA-USP Pirassununga: Mirele Daiana Poleti; Raul Machado Neto; Rejane Maria Tommasini Grotto; Ricardo Augusto Brassaloti; Ricardo Haddad; Rodrigo Tocantins Calado.; Sandra Coccuzzo Sampaio; Sandra Coccuzzo Sampaio Vessoni; Simone Kashima; Svetoslav Nanev Slavov; Vagner Fonseca; Vincent Louis Viala
EPI_ISL_1966853	ESF ROSA ROSSI MAIA	Instituto Butantan / ESALQ-USP (Piracicaba)	Antonio Jorge Martins; Bianca Cechetto Carlos. Mendelics: Bibiana Santos; Claudia Renata dos Santos Barros; Cíntia Bittar; David Schlesinger. Hemocentro Ribeirão Preto: Simone Kashima; Debora Botequio Moretti; Elaine Cristina Marqueze; Elaine Vieira dos Santos; Elisangela Chicaroni Mattos; Erika Freitas; Evandra Strazza Rodrigues; Felipe Allan da Silva da Costa; Flavia Aburjaile; Fábio Sossai Possebon; Guilherme Campos; Guilherme Targino Valente; Heidge Fukumasu. USP-Botucatu: Rejane Maria Tommasini Grotto; Helena Lage Ferreira; Instituto Butantan: Dimas Tadeu Covas; Jardelina de Souza Todao Bernardino; Jayme A. Souza-Neto; Jessika Cristina Chagas Lesbon; Jorge A. Petroli Marchesi; José Salvatore Leister Patané; João Paulo Kitajima; João Pessoa Araújo Jr.; Leila Sabrina Ullmann; Loyze Paola Oliveira de Lima; Luiz Aurelio de Campos Crispin. Centro de Genômica Funcional da ESALQ: Luiz Lehmann Coutinho; Luiz Carlos Junior de Alcantara; Lívia Sacchetto; Maisa C. Pereira Parra; Maria Carolina Elias; Marta Giovanetti; Marília Moraes; Maurício Lacerda Nogueira. Prefeitura de Sao Paulo: Melissa Palmieri.; Patricia Akemi Assato; Paula Rahal; Paulo Inacio da Costa; Rafael dos Santos Bezerra; Raquel de Lello Rocha Campos Cassano. NGS Soluções Genômicas: Pilar Drummond Sampaio Corrêa Mariani. FZEA-USP Pirassununga: Mirele Daiana Poleti; Raul Machado Neto; Ricardo Augusto Brassaloti; Ricardo Haddad; Rodrigo Tocantins Calado. FAMERP-SJRP: Cecília Artico Banho; Sandra Coccuzzo Sampaio; Svetoslav Nanev Slavov; Vagner Fonseca; Vincent Louis Viala
EPI_ISL_3215463, EPI_ISL_3215466, EPI_ISL_3215596, EPI_ISL_3215666, EPI_ISL_3215740	EXCITE Lab	Andersen lab at Scripps Research	Abigail Schnapper; Angela Scioscia; Chip Schooley; David Pride; Helena Tubb; Natasha Martin Cheryl Anderson; Sawyer Farmer; Sharon Reed; Tommy Valles + SEARCH
EPI_ISL_2477521	Edmonton Provincial Lab	Public Health Agency of Canada (PHAC) National Microbiology Laboratory	Buss; Croxen M; Deo A; Dieu P; E; Ferrato C; Gill K; Khan F; Koleva P; Li V; Lloyd C; Lynch T; Ma R; Murphy S; Pabbaraju K; Shokoples S; Thayer J; Tipples G; Whitehouse M; Wong A; Yu C; Zelyas N
EPI_ISL_2107410, EPI_ISL_2107414, EPI_ISL_2107415	Emory Molecular Diagnostics Laboratory, Emory Healthcare	Piantadosi Lab, Emory Department of Pathology	Ahmed Babiker; Anne Piantadosi
EPI_ISL_2112527	Epidemiology of Microbial Diseases, Yale School of Public Health	Epidemiology of Microbial Diseases, Yale School of Public Health	A.E.; A.F.; Alpert, T.; Breban; Brito; C.B.; C.C.; Fauver; Grubaugh; I.M.; Iwasaki, A.; J.E.; J.R.; Kalinich; Landry; Lucas, C.; M.E.; M.I.; M.L.; N.D.; Ott; Petrone; Rothman; Vogels; Watkins
EPI_ISL_1571326, EPI_ISL_1845999, EPI_ISL_2110509, EPI_ISL_2110530, EPI_ISL_2110534, EPI_ISL_2110542, EPI_ISL_2110571, EPI_ISL_2110576, EPI_ISL_2123732			
see above	Eurofins LifeCodexx GmbH	Robert Koch Institute	
EPI_ISL_3354635	FL Bur. of Public Health Laboratories-Jacksonville	Centers for Disease Control and Prevention Division of Viral Diseases, Pathogen Discovery	Alex Burgin; Ben Rambo-Martin; Clinton Paden; Dakota Howard; Dave Wentworth; Dhwani Batra; Jasmine Padilla; Justin Lee; Krista Queen; Kristen Knipe; Kristine Lacek; Mark Burroughs; Matthew Schmerer; Meghan Bentz; Mili Sheth; Peter Cook; Sam Shepard; Sarah Nobles; Suxiang Tong; Vivien Dugan; Yvette Unoarumhi
EPI_ISL_2018168, EPI_ISL_3354607	FL Bureau of Public Health Laboratories-Miami	Centers for Disease Control and Prevention Division of Viral Diseases, Pathogen Discovery	Alex Burgin; Alison Laufer Halpin; Ben L. Rambo-Martin; Ben Rambo-Martin; Clinton Paden; Clinton R. Paden; Dakota Howard; Darlene Wagner; Dave Wentworth; Dhwani Batra; Jasmine Padilla; Justin Lee; Katie Dillon; Krista Queen; Kristen Knipe; Kristine Lacek; Mark Burroughs; Matthew Schmerer; Meghan Bentz; Mili Sheth; Peter Cook; Sam Shepard; Sarah Nobles; Shoshona Le; Suxiang Tong; Vivien Dugan; Yvette Unoarumhi
EPI_ISL_854594	Faroese National Reference Laboratory for Fish and Animal Diseases	Faroese National Reference Laboratory for Fish and Animal Diseases	Arnfinnur Kallsberg Junior; Debes Hammershaimb Christiansen; Maria Marjunardóttir Dahl; Petra Elisabeth Petersen
EPI_ISL_2156293	First Aide Diagnostic Center	Philippine Genome Center	Alethea R. de Guzman; Anna Ong-Lim; Arianne A. Zamora; Asia Louisa U. Chong; Benedict A. Maralit; Candice Francheska B. Tambaoan; Carlo M. Lapid; Celia Carlos; Devon Ray Pacial; Edsel Maurice Salvaña; El King D. Morado; Elcid Aaron R. Pangilinan; Eva Maria Cutiongco-de la Paz; Francis A. Tablizo; Irish Coleen A. Asin; Jaime C. Montoya; Jan Michael C. Yap; Jo-Hannah S. Llames; John Q. Wong; Joshua Gregor A. Dizon; Juan Antonio R. Magalang; Karol Sophia Agape R. Padilla; Kenneth M. Kim; Kris P. Punayan; Marc Edsel C. Ayes; Marc Jerrone R. Castro; Maria Rosario Singh-Vergeire and Cynthia P. Saloma; Maria Sofia L. Yangzon; Marissa Alejandria; Razel Nikka M. Hao; Rianna Patricia S. Cruz; Sheila Mae M. Araiza
EPI_ISL_1652237	Fleury	Instituto Butantan / Mendelics	Alexander Roberto Precioso; Antonio Jorge Martins; Bibiana Santos; Claudia Renata dos Santos Barros; David Schlesinger; Debora Botequio Moretti; Dimas Tadeu Covas; Elaine Cristina Marqueze; Elaine Vieira dos Santos; Erika Freitas; Evandra Strazza Rodrigues; Flavia Aburjaile; José Salvatore Leister Patané; João Paulo Kitajima; Luiz Carlos Junior de Alcantara; Maria Carolina Elias; Marta Giovanetti; Rafael dos Santos Bezerra; Raul Machado Neto; Ricardo Haddad; Rodrigo Tocantins Calado.; Sandra Coccuzzo Sampaio; Simone Kashima; Svetoslav Nanev Slavov; Vagner Fonseca; Vincent Louis Viala
EPI_ISL_1239974, EPI_ISL_1531237, EPI_ISL_1651496, EPI_ISL_1651531, EPI_ISL_2008652, EPI_ISL_2008659, EPI_ISL_2008709, EPI_ISL_2008742, EPI_ISL_2008744, EPI_ISL_2008745, EPI_ISL_2008827, EPI_ISL_2229962, EPI_ISL_2229989, EPI_ISL_2277955, EPI_ISL_2442426, EPI_ISL_2836047, EPI_ISL_2836377			
see above	Florida Bureau of Public Health Laboratories	Florida Bureau of Public Health Laboratories	Jason Blanton; Sarah Schmedes
EPI_ISL_1554964, EPI_ISL_1554965, EPI_ISL_1555018, EPI_ISL_1555035, EPI_ISL_1555060, EPI_ISL_1555064, EPI_ISL_1555082, EPI_ISL_1555099, EPI_ISL_1555137, EPI_ISL_1555146, EPI_ISL_1555187, EPI_ISL_1555264, EPI_ISL_1555282, EPI_ISL_1555330, EPI_ISL_1555406, EPI_ISL_1555504, EPI_ISL_1555538, EPI_ISL_1555591, EPI_ISL_1555674, EPI_ISL_1555688, EPI_ISL_1555693, EPI_ISL_1555728, EPI_ISL_1555759, EPI_ISL_1555775, EPI_ISL_1555901, EPI_ISL_1555910, EPI_ISL_1555974, EPI_ISL_1556010, EPI_ISL_1556163, EPI_ISL_1556170, EPI_ISL_1556202, EPI_ISL_1556218, EPI_ISL_1556224, EPI_ISL_1556227, EPI_ISL_1556267, EPI_ISL_1556324, EPI_ISL_1556395, EPI_ISL_1556396, EPI_ISL_1556452, EPI_ISL_1556486, EPI_ISL_1556646, EPI_ISL_1556649, EPI_ISL_1556682, EPI_ISL_1556720, EPI_ISL_1556729, EPI_ISL_1556737, EPI_ISL_1556787, EPI_ISL_1556805, EPI_ISL_1556907, EPI_ISL_1556918, EPI_ISL_1556919, EPI_ISL_1556924, EPI_ISL_1556947, EPI_ISL_1556998, EPI_ISL_1556999, EPI_ISL_1557007, EPI_ISL_1557095, EPI_ISL_1557096, EPI_ISL_1612943, EPI_ISL_1612964, EPI_ISL_1613001, EPI_ISL_1613006, EPI_ISL_1613068, EPI_ISL_1613339, EPI_ISL_1613410, EPI_ISL_1613534, EPI_ISL_1613543, EPI_ISL_1613836, EPI_ISL_1613957, EPI_ISL_1614028, EPI_ISL_1614045, EPI_ISL_1614083, EPI_ISL_1614101, EPI_ISL_1614119, EPI_ISL_1614154, EPI_ISL_1614179, EPI_ISL_1614213, EPI_ISL_1614415, EPI_ISL_1614436, EPI_ISL_1614500, EPI_ISL_1614512, EPI_ISL_1614589, EPI_ISL_1614598, EPI_ISL_1614667, EPI_ISL_1614680, EPI_ISL_1614698, EPI_ISL_1614734, EPI_ISL_1614747, EPI_ISL_1614905, EPI_ISL_1665906, EPI_ISL_1665945, EPI_ISL_1666026, EPI_ISL_1666085, EPI_ISL_1666100, EPI_ISL_1666101, EPI_ISL_1666131, EPI_ISL_1666224, EPI_ISL_1666274, EPI_ISL_1666322, EPI_ISL_1666323, EPI_ISL_1666325, EPI_ISL_1666346, EPI_ISL_1666376, EPI_ISL_1666383, EPI_ISL_1666389, EPI_ISL_1666391, EPI_ISL_1666396, EPI_ISL_1666432, EPI_ISL_1666492, EPI_ISL_1666515, EPI_ISL_1666657, EPI_ISL_1666808, EPI_ISL_1667066, EPI_ISL_1667068, EPI_ISL_1667118, EPI_ISL_1667119, EPI_ISL_1667186, EPI_ISL_1667199, EPI_ISL_1667221, EPI_ISL_1667226, EPI_ISL_1667245, EPI_ISL_1667284, EPI_ISL_1667360, EPI_ISL_1733818, EPI_ISL_1733866, EPI_ISL_1733903, EPI_ISL_1733971, EPI_ISL_1734014, EPI_ISL_1734124, EPI_ISL_1734136, EPI_ISL_1734154, EPI_ISL_1734267, EPI_ISL_1734447, EPI_ISL_1734481, EPI_ISL_1734512, EPI_ISL_1734526, EPI_ISL_1734530, EPI_ISL_1734540, EPI_ISL_1802677, EPI_ISL_1802694, EPI_ISL_1839089, EPI_ISL_1839119, EPI_ISL_1839128, EPI_ISL_1839145, EPI_ISL_1839146, EPI_ISL_1839284, EPI_ISL_1839305, EPI_ISL_1839313, EPI_ISL_1839328, EPI_ISL_1839333, EPI_ISL_1839379, EPI_ISL_1839384, EPI_ISL_1839386, EPI_ISL_1839392, EPI_ISL_1839393, EPI_ISL_1839414, EPI_ISL_1839513, EPI_ISL_1839525, EPI_ISL_1839530, EPI_ISL_1839538, EPI_ISL_1839656, EPI_ISL_1839662, EPI_ISL_1839693, EPI_ISL_1839736, EPI_ISL_1839743, EPI_ISL_1839870, EPI_ISL_1839910, EPI_ISL_1839937, EPI_ISL_1839938, EPI_ISL_1840157, EPI_ISL_1992484, EPI_ISL_1992524, EPI_ISL_1992528, EPI_ISL_1992529, EPI_ISL_2037730, EPI_ISL_2037765, EPI_ISL_2037773, EPI_ISL_2037774, EPI_ISL_2037776, EPI_ISL_2037784, EPI_ISL_2037786, EPI_ISL_2037823, EPI_ISL_2037875, EPI_ISL_2037951, EPI_ISL_2037956, EPI_ISL_2037963, EPI_ISL_2037966, EPI_ISL_2037999, EPI_ISL_2038010, EPI_ISL_2038036, EPI_ISL_2038042, EPI_ISL_2038059, EPI_ISL_2038062, EPI_ISL_2038083, EPI_ISL_2038087, EPI_ISL_2038103, EPI_ISL_2038129, EPI_ISL_2038141, EPI_ISL_2038145, EPI_ISL_2038155, EPI_ISL_2038161, EPI_ISL_2038174, EPI_ISL_2038214, EPI_ISL_2038232, EPI_ISL_2038235, EPI_ISL_2038255, EPI_ISL_2038310, EPI_ISL_2038398, EPI_ISL_2038399, EPI_ISL_2038400, EPI_ISL_2038434, EPI_ISL_2038440, EPI_ISL_2038456, EPI_ISL_2038473, EPI_ISL_2038491, EPI_ISL_2038549, EPI_ISL_2038576, EPI_ISL_2038645, EPI_ISL_2038670, EPI_ISL_2038683, EPI_ISL_2038712, EPI_ISL_2038734, EPI_ISL_2038761			
see above	Fulgent Genetics	Centers for Disease Control and Prevention Division of Viral Diseases, Pathogen Discovery	Adrian Paskey; Becky Tsai; Benafsh Sapra; Benjamin Rambo-Martin; Christopher Gulvick; Clinton R. Paden; Dakota Howard; Darlene Wagner; Dhwani Batra; Doreen Ng; Duncan MacCannell; Harry Gao; James Xie; Jason Caravas; John Gao; Joseph Fierro; Kara Moser; Matthew Schmerer; Mickey Li; Peter W. Cook; Scott Sammons; Shatavia Morrison; Yan Meng; Yvette Unoarumhi
EPI_ISL_1937097, EPI_ISL_1937161, EPI_ISL_1937397	Fulgent Genetics	Fulgent Genetics	Becky Tsai; Benafsh Sapra; Doreen Ng; Harry Gao; James Xie; John Gao; Joseph Fierro; Mickey Li; Yan Meng
EPI_ISL_1239137, EPI_ISL_1239138	Fundação Ezequiel Dias	Coordenação Geral de Laboratórios de Saúde Pública (CGLAB)	; Vagner Fonseca et al
EPI_ISL_2249397, EPI_ISL_2293021, EPI_ISL_2293022	Fundação Ezequiel Dias	Coordenação Geral de Laboratórios de Saúde Pública (CGLAB/DAEVS/SVS/MS)	Vagner Fonseca; et al.
EPI_ISL_1182543, EPI_ISL_1182544, EPI_ISL_1182545	Fundação Ezequiel Dias (FUNED)	Coordenação Geral de Laboratórios de Saúde Pública (CGLAB/DAEVS/SVS/MS)	Vagner Fonseca; et al.
EPI_ISL_3553531, EPI_ISL_3553532, EPI_ISL_3553533, EPI_ISL_3553534, EPI_ISL_3553535, EPI_ISL_3553536, EPI_ISL_3553537, EPI_ISL_3553538, EPI_ISL_3553539, EPI_ISL_3553540, EPI_ISL_3553541, EPI_ISL_3553542, EPI_ISL_3553543, EPI_ISL_3553544, EPI_ISL_3553545, EPI_ISL_3553546, EPI_ISL_3553547, EPI_ISL_3553548, EPI_ISL_3553549, EPI_ISL_3553550, EPI_ISL_3553551, EPI_ISL_3553552,			

EPI_ISL_3553553, EPI_ISL_3553554, EPI_ISL_3553555, EPI_ISL_3553556, EPI_ISL_3553557, EPI_ISL_3553558, EPI_ISL_3553559, EPI_ISL_3553560, EPI_ISL_3553561, EPI_ISL_3553562, EPI_ISL_3553563, EPI_ISL_3553564, EPI_ISL_3553565, EPI_ISL_3553566, EPI_ISL_3553567, EPI_ISL_3553568, EPI_ISL_3553569, EPI_ISL_3553570, EPI_ISL_3553571, EPI_ISL_3553572, EPI_ISL_3553573, EPI_ISL_3553574, EPI_ISL_3553575, EPI_ISL_3553576, EPI_ISL_3553577, EPI_ISL_3553578, EPI_ISL_3553579, EPI_ISL_3553580, EPI_ISL_3553581, EPI_ISL_3553582, EPI_ISL_3553583, EPI_ISL_3553688			
see above	Fundação Ezequiel Dias (FUNED)	Fundação Ezequiel Dias	Andre Leal; Cynthia Vazquez; Elaine Cristina; Felipe Iani; Flavia Aburjaile; Gislene Garcia de Castro Lichs; Glauco Carvalho; Hegger Fritsch; Joilson Xavier; Luiz Alcantara.; Luiz Henrique Ferraz Demarchi; Luiz Takao Watanabe; Marina Castilhos Souza Umaki Zardin; Marta Giovanetti; Natalia Guimaraes; Raquel da Silva Ferreira; Talita Adelino; Vagner Fonseca; de Oliveira
EPI_ISL_2551523, EPI_ISL_2551524, EPI_ISL_2551530	Fundação Ezequiel Dias - FUNED	Laboratório de Virologia Clínica e Molecular	Alex Fiorini; Ana Paula Salles Fernandes; Bruna Larotonda Telezynski; Danielle Bruna Leal Oliveira; Edison Luiz Durigon; Erick Gustavo Dorlass; Flavio Fonseca e Santuza Teixeira; Guilherme Pereira Scagion; Helena Perez Coelho; Hugo Sato; Karine Lima Lourenço; Luciano Matsumiya Thomazelli; Renata Peixoto; Rubens Daniel Miserani Magalhães; Tatiana Ometto
EPI_ISL_1708318, EPI_ISL_2499748	Fundação Hospitalar de Hematologia e Hemoterapia do Amazonas	Laboratório de Estudos de Vírus Emergentes	Adriana S. S. Duarte; Alessandro S. Farias; Arilson Bernardo S. P. Gomes; Audrey B. Zangirolami; Bruno D. Benites; Camila A.M. Silva; Camila L. Simeoni; Camila Lopes Simeoni; Carolina Costa-Lima; Cecilia C. Camilo; Chieh-Hsi Wu; Christopher Dye; Clarice W. Arns; Daniel A. Toledo-Teixeira; Daniel Augusto de Toledo-Teixeira; Darlan S. Candido; Erika R. Manuli; Esmenia C. Rocha; Ester C. Sabino; Ester Cerdeira Sabino; Fabia; Fabiana Granja; Fernando R. Spilki; Flavia C. Sales; Gabriela Fabiano de Souza; Giulia M. Ferreira; Grazielle C. Maktura; Henrique Marques-Souza; Ingra M. Claro; Jaqueline G. de Jesus; José Luiz Proença Módena; Julia Forato; Karina Bispo dos Santos; Karina Bispo-dos-Santos; Lais D. Coimbra; Lais Durço Coimbra; Leandro M. Souza; Lucas A.M. Franco; Lucas I. Buscaratti; Luciana S. Mofatto; Luciana Souto Mofatto; Magnun N.N. Santos; Marcelo A.S. Mori; Marcelo Addas-Carvalho; Maria L. Moretti; Maria Luiza Moretti; Mariana C. Pinho; Mariana S. Ramundo; Mariene R. Amorim; Mariene Ribeiro Amorim; Michael S. Diamond; Myuki A.E. Crispim; Natalia S. Brunetti; Nelson Gaburo; Nuno R. Faria; Nuno Rodrigues Faria; Oliver G. Pybus; Pamela S. Andrade; Pierina L. Parise; Pierina Lorencini Parise; Priscilla P. Barbosa; Priscilla Paschoal Barbosa; Rafael E. Marques; Renata Sesti-Costa; Rodrigo N. Angerami; Rodrigo Nogueira Angerami; Stefanie Primon Muraro; Thais M. Coletti; Vitor A. Costa; William M. Souza; William Marciel de Souza
EPI_ISL_1579172	Furst Medical Laboratory	Norwegian Institute of Public Health, Department of Virology	Atiya R Ali; Debech Nadia; Engebretsen Serina Beate; Garcia Llorente Ignacio; Hilde Elshaug; Hilde Vollan; Jon Bråte; Kamilla Heddeland Instefjord; Karoline Bragstad; Kathrine Stene-Johansen; Marie Paulsen Madsen; Olav Hungnes; Pedersen Benedikte Nevjen; Rasmus Riis Kopperud
EPI_ISL_1678367, EPI_ISL_1937673	GA Department of Public Health Laboratory	Centers for Disease Control and Prevention Division of Viral Diseases, Pathogen Discovery	Alison Laufer Halpin; Ben L. Rambo-Martin; Clinton R. Paden; Dakota Howard; Darlene Wagner; Dave Wentworth; Dhwani Batra; Jasmine Padilla; Justin Lee; Katie Dillon; Krista Queen; Kristen Knipe; Kristine Lacek; Mark Burroughs; Matthew Schmerer; Mili Sheth; Peter Cook; Sam Shepard; Sarah Nobles; Shoshona Le; Suxiang Tong; Vivien Dugan; Yvette Unoarumhi
EPI_ISL_1754367, EPI_ISL_1754385, EPI_ISL_1754388, EPI_ISL_1756229, EPI_ISL_1756495, EPI_ISL_1756508	GH A.CHENEVIER-H.MONDOR	Department of Virology, Henri Mondor University Hospital, Assistance Publique Hôpitaux de Paris, Université Paris-Est Créteil, INSERM U955	Alexandre Soulier; Christophe Rodriguez; Elisabeth Trawinski; Guillaume Gricourt; Jean-Michel Pawlotsky; Melissa N'Debi; Slim Fourati; Vanessa Demontant
EPI_ISL_1755025	GROUPE HOSPITALIER SUD ILE DE FRANCE	Department of Virology, Henri Mondor University Hospital, Assistance Publique Hôpitaux de Paris, Université Paris-Est Créteil, INSERM U955	Alexandre Soulier; Christophe Rodriguez; Elisabeth Trawinski; Guillaume Gricourt; Jean-Michel Pawlotsky; Melissa N'Debi; Slim Fourati; Vanessa Demontant
EPI_ISL_1966724	GRUPO TECNICO DE VIGILANCIA SANITARIA E EPIDEMIOLOGICA	Instituto Butantan	Antonio Jorge Martins; Bianca Cechetto Carlos. Mendelics: Bibiana Santos; Claudia Renata dos Santos Barros; Cíntia Bittar; David Schlesinger. Hemocentro Ribeirão Preto: Simone Kashima; Debora Botequio Moretti; Elaine Cristina Marqueze; Elaine Vieira dos Santos; Elisangela Chicaroni Mattos; Erika Freitas; Evandra Strazza Rodrigues; Felipe Allan da Silva da Costa; Flavia Aburjaile; Fábio Sossai Possebon; Guilherme Campos; Guilherme Targino Valente; Heidge Fukumasu. USP-Botucatu: Rejane Maria Tommasini Grotto; Helena Lage Ferreira; Instituto Butantan: Dimas Tadeu Covas; Jardelina de Souza Todao Bernardino; Jayme A. Souza-Neto; Jessika Cristina Chagas Lesbon; Jorge A. Petroli Marchesi; José Salvatore Leister Patané; João Paulo Kitajima; João Pessoa Araújo Jr.; Leila Sabrina Ullmann; Loyze Paola Oliveira de Lima; Luiz Aurelio de Campos Crispin. Centro de Genômica Funcional da ESALQ: Luiz Lehmann Coutinho; Luiz Carlos Junior de Alcantara; Lívia Sacchetto; Maisa C. Pereira Parra; Maria Carolina Elias; Marta Giovanetti; Marília Moraes; Maurício Lacerda Nogueira. Prefeitura de Sao Paulo: Melissa Palmieri.; Patricia Akemi Assato; Paula Rahal; Paulo Inacio da Costa; Rafael dos Santos Bezerra; Raquel de Lello Rocha Campos Cassano. NGS Soluções Genômicas: Pilar Drummond Sampaio Corrêa Mariani. FZEA-USP Pirassununga: Mirele Daiana Poleti; Raul Machado Neto; Ricardo Augusto Brassaloti; Ricardo Haddad; Rodrigo Tocantins Calado. FAMERP-SJRP: Cecília Artico Banho; Sandra Coccuzzo Sampaio; Svetoslav Nanev Slavov; Vagner Fonseca; Vincent Louis Viala
EPI_ISL_1966835, EPI_ISL_1966836, EPI_ISL_1966838, EPI_ISL_1966839	GRUPO TECNICO DE VIGILANCIA SANITARIA E EPIDEMIOLOGICA	Instituto Butantan / ESALQ-USP (Piracicaba)	Antonio Jorge Martins; Bianca Cechetto Carlos. Mendelics: Bibiana Santos; Claudia Renata dos Santos Barros; Cíntia Bittar; David Schlesinger. Hemocentro Ribeirão Preto: Simone Kashima; Debora Botequio Moretti; Elaine Cristina Marqueze; Elaine Vieira dos Santos; Elisangela Chicaroni Mattos; Erika Freitas; Evandra Strazza Rodrigues; Felipe Allan da Silva da Costa; Flavia Aburjaile; Fábio Sossai Possebon; Guilherme Campos; Guilherme Targino Valente; Heidge Fukumasu. USP-Botucatu: Rejane Maria Tommasini Grotto; Helena Lage Ferreira; Instituto Butantan: Dimas Tadeu Covas; Jardelina de Souza Todao Bernardino; Jayme A. Souza-Neto; Jessika Cristina Chagas Lesbon; Jorge A. Petroli Marchesi; José Salvatore Leister Patané; João Paulo Kitajima; João Pessoa Araújo Jr.; Leila Sabrina Ullmann; Loyze Paola Oliveira de Lima; Luiz Aurelio de Campos Crispin. Centro de Genômica Funcional da ESALQ: Luiz Lehmann Coutinho; Luiz Carlos Junior de Alcantara; Lívia Sacchetto; Maisa C. Pereira Parra; Maria Carolina Elias; Marta Giovanetti; Marília Moraes; Maurício Lacerda Nogueira. Prefeitura de Sao Paulo: Melissa Palmieri.; Patricia Akemi Assato; Paula Rahal; Paulo Inacio da Costa; Rafael dos Santos Bezerra; Raquel de Lello Rocha Campos Cassano. NGS Soluções Genômicas: Pilar Drummond Sampaio Corrêa Mariani. FZEA-USP Pirassununga: Mirele Daiana Poleti; Raul Machado Neto; Ricardo Augusto Brassaloti; Ricardo Haddad; Rodrigo Tocantins Calado. FAMERP-SJRP: Cecília Artico Banho; Sandra Coccuzzo Sampaio; Svetoslav Nanev Slavov; Vagner Fonseca; Vincent Louis Viala
EPI_ISL_2345835, EPI_ISL_2362271	GRUPO TECNICO DE VIGILANCIA SANITARIA E EPIDEMIOLOGICA	Instituto Butantan / Mendelics	Antonio Jorge Martins; Claudia Renata dos Santos Barros; David Schlesinger; Debora Botequio Moretti; Dimas Tadeu Covas; Elaine Cristina Marqueze; Elaine Vieira Santos; Evandra Strazza Rodrigues; Heidge Fukumasu; Jayme Augusto de Souza-Neto; José Salvatore Leister Patané; Luiz Alcantara; Luiz Lehmann Coutinho; Maria Carolina Elias; Maurício Lacerda Nogueira; Rafael dos Santos Bezerra; Raul Machado Neto; Rejane Maria Tommasini Grotto; Ricardo Haddad; Sandra Coccuzzo Sampaio Vessoni; Simone Kashima; Svetoslav Nanev Slavov; Vincent Louis Viala
EPI_ISL_1626613, EPI_ISL_1626615, EPI_ISL_1626616, EPI_ISL_2157195, EPI_ISL_2157196, EPI_ISL_2157197, EPI_ISL_2157198, EPI_ISL_2157201			
see above	Gencore - Universidad de los Andes	Gencore - Universidad de los Andes	Ana Maria Palacio; Cristian Barrera; David Gonzalez; Erica Salguero; Gabriela Ariza; Luisa Sacristan; Marcela Guevara; Silvia Restrepo
EPI_ISL_1167929, EPI_ISL_1321468, EPI_ISL_1470430, EPI_ISL_1470440, EPI_ISL_1470451, EPI_ISL_1470457, EPI_ISL_1470478, EPI_ISL_1470501, EPI_ISL_1470517, EPI_ISL_1470531, EPI_ISL_1470548, EPI_ISL_1470549, EPI_ISL_1470550, EPI_ISL_1470551, EPI_ISL_1470554, EPI_ISL_1534575, EPI_ISL_1534590, EPI_ISL_1534603, EPI_ISL_1541015, EPI_ISL_1541055, EPI_ISL_1633360, EPI_ISL_1633365, EPI_ISL_1633367, EPI_ISL_1633368, EPI_ISL_1633379, EPI_ISL_1633388, EPI_ISL_1633392, EPI_ISL_1633430, EPI_ISL_1633433, EPI_ISL_1633523, EPI_ISL_1633524, EPI_ISL_1633525, EPI_ISL_1633529, EPI_ISL_1633544, EPI_ISL_1712326, EPI_ISL_1712329, EPI_ISL_1712347, EPI_ISL_1712351, EPI_ISL_1712353, EPI_ISL_1712354, EPI_ISL_1712359, EPI_ISL_1712360, EPI_ISL_1712362, EPI_ISL_1712364, EPI_ISL_1712367, EPI_ISL_1712368, EPI_ISL_1712370, EPI_ISL_1712372, EPI_ISL_2009215, EPI_ISL_2009227, EPI_ISL_2009236, EPI_ISL_2009237, EPI_ISL_2009239, EPI_ISL_2009245, EPI_ISL_2009255, EPI_ISL_2009266, EPI_ISL_2009287, EPI_ISL_2009603, EPI_ISL_2009604, EPI_ISL_2009605, EPI_ISL_2009608, EPI_ISL_2009611, EPI_ISL_2009617, EPI_ISL_2009620, EPI_ISL_2009624, EPI_ISL_2597248, EPI_ISL_2597252, EPI_ISL_2597260, EPI_ISL_2597299, EPI_ISL_2597301, EPI_ISL_3546385			
see above	Genetica Molecular and Subdepartamento de Virologia ISP Chile	Instituto de Salud Publica de Chile	Andres Castillo; Barbara Parra; Constanza Campano; Gisselle Barra; Jaime Lagos; Javier Tognarelli; Jorge Fernandez; Karen Orostica; Loredana Arata; Patricia Bustos; Rodrigo Fasce; Soledad Ulloa
EPI_ISL_1511643	Genomic and molecular Biology Group, A.C.Camargo Cancer Center	Laboratory of Bioinformatics and Computational Biology, A.C.Camargo Cancer Center	Alexandre Defelicibus; Dirce Carraro; Giovana Torrezan; Israel Tojal
EPI_ISL_1399624, EPI_ISL_1399625, EPI_ISL_1399626, EPI_ISL_1739105, EPI_ISL_1739106, EPI_ISL_1739107, EPI_ISL_1739135, EPI_ISL_1739136			
see above	Germano de Sousa	Instituto Nacional de Saude (INSA)	Borges et al
EPI_ISL_1854229	Germano de Sousa	Instituto Nacional de Saude (INSA) and Instituto Gulbenkian de Ciencia (IGC)	Borges et al
EPI_ISL_1219136	Gonçalo Moniz Institute, FIOCRUZ, Bahia	Laboratory of Respiratory Viruses and Measles, Oswaldo Cruz Institute, FIOCRUZ	Alice Sampaio Rocha; Ana Carolina Mendonca; Anna Carolina Paixao; Fernando Motta; Luciana Appolinario; Marilda Siqueira on behalf of the Fiocruz COVID-19 Genomic Surveillance Network; Paola Resende; Renata Serrano Lopes; Ricardo Khouri; Tiago Graf
EPI_ISL_2550797	Grady Memorial Hospital, Clinical Microbiology Laboratory	Piantadosi Lab, Emory Department of Pathology	Anne Piantadosi; Dara Khosravi; Yun F (Wayne) Wang
EPI_ISL_2339821, EPI_ISL_2339822, EPI_ISL_2339835, EPI_ISL_2339841, EPI_ISL_2603133, EPI_ISL_2603138, EPI_ISL_2603147, EPI_ISL_2603153, EPI_ISL_2603156			
see above	Grupo de Investigación en Enfermedades Tropicales del Ejército (GINETEJ), Laboratorio de Referencia e Investigación, Dirección de Sanidad Ejército, Bogotá, Colombia	Centro de Investigaciones en Microbiología y Biotecnología-UR (CIMBIUR), Facultad de Ciencias Naturales, Universidad del Rosario, Bogotá, Colombia	Camilo A. Correa-Cárdenas; Carolina Oliveros; Claudia Méndez; Elizabeth K. Márquez; Frank de los Santos Ortiz; Juan David Ramírez; Julie Pérez; Lorena Albarracín; Luz H. Patiño; Maria Clara Duque; Marina Muñoz; María Teresa Alvarado; Nathalia Ballesteros; Sergio Castañeda; Sergio Gutiérrez-Riveros; Yanira Romero; Zulma Cucunubá
EPI_ISL_1632710	Gundersen Clinical Microbiology Laboratory	Kabara Cancer Research Institute	Craig S. Richmond; Paraic A. Kenny
EPI_ISL_1854240	H Evora	Instituto Nacional de Saude (INSA)	Borges et al
EPI_ISL_1854241	H Garcia de Orta	Instituto Nacional de Saude (INSA)	Borges et al
EPI_ISL_3102237, EPI_ISL_3102238, EPI_ISL_3102458,	H J M A HOSPITAL JOSE MARTINIANO DE ALENCAR	Analytical Competence Molecular Epidemiology Lab/ACME, Oswaldo	Cleber Furtado Aksenen; Fabio Miyajima; Fernando Braga Stehling; Francisco Eder de Moura Lopes; Jamille Maria Mendes Bezerra; Joaquim Cesar do Nascimento Sousa Junior; Joaquim César do Nascimento Sousa Junior; Pedro Miguel Carneiro Jeronimo; Suzana Porto Almeida & Lucas Delerino on behalf of COVID-19 FIOCRUZ Genomic Network; Suzana Porto Almeida e Lucas Delerino; Thais Ferreira de Oliveira; Thais de Oliveira Costa; Ticiane Cavalcante de Souza; Veridiana Pessoa Miyajima

EPI_ISL_3912159, EPI_ISL_3912160		Cruz Foundation, Ceara (FIOCRUZ CE)	
EPI_ISL_1854249, EPI_ISL_1854250	H Vila Franca Xira	Instituto Nacional de Saude (INSA) and Institute of Biomedicine (iBiMed), Universidade de Aveiro	Borges et al
EPI_ISL_3102216, EPI_ISL_3102218, EPI_ISL_3102219, EPI_ISL_3102220	HEMOCE CENTRO DE HEMATOLOGIA E HEMOTERAPIA	Analytical Competence Molecular Epidemiology Lab/ACME, Oswaldo Cruz Foundation, Ceara (FIOCRUZ CE)	Cleber Furtado Aksenen; Fabio Miyajima; Fernando Braga Stehling; Francisco Eder de Moura Lopes; Jamille Maria Mendes Bezerra; Joaquim César do Nascimento Sousa Junior; Pedro Miguel Carneiro Jeronimo; Suzana Porto Almeida e Lucas Delerino; Thais Ferreira de Oliveira; Thais de Oliveira Costa; Ticiane Cavalcante de Souza; Veridiana Pessoa Miyajima
EPI_ISL_2801308, EPI_ISL_2801309, EPI_ISL_2801322, EPI_ISL_2801365, EPI_ISL_3102332, EPI_ISL_3102338, EPI_ISL_3102362, EPI_ISL_3102371, EPI_ISL_3102402			
see above	HEMOCE CENTRO DE HEMATOLOGIA E HEMOTERAPIA DO CEARA	Analytical Competence Molecular Epidemiology Lab/ACME, Oswaldo Cruz Foundation, Ceara (FIOCRUZ CE)	Cleber Furtado Aksenen; Cleber Furtado Aksenen e Suzana Porto Almeida; Fabio Miyajima; Fernando Braga Stehling; Francisco Eder de Moura Lopes; Jamille Maria Mendes Bezerra; Joaquim César do Nascimento Sousa Junior; Pedro Miguel Carneiro Jeronimo; Suzana Porto Almeida e Lucas Delerino; Thais Ferreira de Oliveira; Thais de Oliveira Costa; Ticiane Cavalcante de Souza; Veridiana Pessoa Miyajima
EPI_ISL_2801307, EPI_ISL_3102380, EPI_ISL_3102388, EPI_ISL_3102404, EPI_ISL_3912163, EPI_ISL_3912164	HGCC HOSPITAL GERAL DR CESAR CALS	Analytical Competence Molecular Epidemiology Lab/ACME, Oswaldo Cruz Foundation, Ceara (FIOCRUZ CE)	Cleber Furtado Aksenen; Cleber Furtado Aksenen e Suzana Porto Almeida; Fabio Miyajima; Fernando Braga Stehling; Francisco Eder de Moura Lopes; Jamille Maria Mendes Bezerra; Joaquim Cesar do Nascimento Sousa Junior; Joaquim César do Nascimento Sousa Junior; Pedro Miguel Carneiro Jeronimo; Suzana Porto Almeida & Lucas Delerino on behalf of COVID-19 FIOCRUZ Genomic Network; Suzana Porto Almeida e Lucas Delerino; Thais Ferreira de Oliveira; Thais de Oliveira Costa; Ticiane Cavalcante de Souza; Veridiana Pessoa Miyajima
EPI_ISL_3102344, EPI_ISL_3912166	HGF HOSPITAL GERAL DE FORTALEZA	Analytical Competence Molecular Epidemiology Lab/ACME, Oswaldo Cruz Foundation, Ceara (FIOCRUZ CE)	Cleber Furtado Aksenen; Fabio Miyajima; Fernando Braga Stehling; Francisco Eder de Moura Lopes; Jamille Maria Mendes Bezerra; Joaquim Cesar do Nascimento Sousa Junior; Joaquim César do Nascimento Sousa Junior; Pedro Miguel Carneiro Jeronimo; Suzana Porto Almeida & Lucas Delerino on behalf of COVID-19 FIOCRUZ Genomic Network; Suzana Porto Almeida e Lucas Delerino; Thais Ferreira de Oliveira; Thais de Oliveira Costa; Ticiane Cavalcante de Souza; Veridiana Pessoa Miyajima
EPI_ISL_3102350, EPI_ISL_3102358, EPI_ISL_3102406	HIAS HOSPITAL INFANTIL ALBERT SABIN	Analytical Competence Molecular Epidemiology Lab/ACME, Oswaldo Cruz Foundation, Ceara (FIOCRUZ CE)	Cleber Furtado Aksenen; Fabio Miyajima; Fernando Braga Stehling; Francisco Eder de Moura Lopes; Jamille Maria Mendes Bezerra; Joaquim César do Nascimento Sousa Junior; Pedro Miguel Carneiro Jeronimo; Suzana Porto Almeida e Lucas Delerino; Thais Ferreira de Oliveira; Thais de Oliveira Costa; Ticiane Cavalcante de Souza; Veridiana Pessoa Miyajima
EPI_ISL_2017282, EPI_ISL_2017283, EPI_ISL_2017284, EPI_ISL_2017285, EPI_ISL_2017286, EPI_ISL_2017287, EPI_ISL_2017288, EPI_ISL_2017290, EPI_ISL_2017292, EPI_ISL_2017293, EPI_ISL_2017294, EPI_ISL_2017295, EPI_ISL_2017296, EPI_ISL_2017298, EPI_ISL_2017299, EPI_ISL_2017300, EPI_ISL_2017301, EPI_ISL_2017303, EPI_ISL_2017304, EPI_ISL_2017305, EPI_ISL_2017324, EPI_ISL_2017325, EPI_ISL_2017326, EPI_ISL_2017327, EPI_ISL_2017328, EPI_ISL_2017329, EPI_ISL_2017330, EPI_ISL_2017331, EPI_ISL_2017332, EPI_ISL_2017333, EPI_ISL_2017334, EPI_ISL_2017335, EPI_ISL_2017336, EPI_ISL_2017338, EPI_ISL_2017339, EPI_ISL_2017340, EPI_ISL_2017341, EPI_ISL_2017342, EPI_ISL_2017343, EPI_ISL_2017344, EPI_ISL_2017345, EPI_ISL_2017346, EPI_ISL_2017347, EPI_ISL_2017348, EPI_ISL_2017349, EPI_ISL_2017350, EPI_ISL_2017351, EPI_ISL_2017352, EPI_ISL_2017353, EPI_ISL_2017354, EPI_ISL_2017355, EPI_ISL_2017356, EPI_ISL_2017357, EPI_ISL_2017358, EPI_ISL_2017359, EPI_ISL_2017360, EPI_ISL_2017361, EPI_ISL_2017362, EPI_ISL_2017365, EPI_ISL_2017368, EPI_ISL_2017371, EPI_ISL_2017373, EPI_ISL_2017374, EPI_ISL_2017375, EPI_ISL_2017376, EPI_ISL_2017377, EPI_ISL_2017378, EPI_ISL_2017379, EPI_ISL_2017380, EPI_ISL_2017382, EPI_ISL_2017393, EPI_ISL_2017395, EPI_ISL_2017396, EPI_ISL_2017399, EPI_ISL_2017404, EPI_ISL_2017407, EPI_ISL_2017408, EPI_ISL_2017413, EPI_ISL_2017414, EPI_ISL_2017417, EPI_ISL_2017418, EPI_ISL_2017419, EPI_ISL_2017421, EPI_ISL_2017424, EPI_ISL_2017425, EPI_ISL_2017426, EPI_ISL_2017427, EPI_ISL_2017428, EPI_ISL_2017429, EPI_ISL_2017446, EPI_ISL_2017448, EPI_ISL_2017451, EPI_ISL_2017452, EPI_ISL_2017453, EPI_ISL_2017454, EPI_ISL_2017456, EPI_ISL_2017459, EPI_ISL_2017462, EPI_ISL_2017463, EPI_ISL_2017464, EPI_ISL_2017467, EPI_ISL_2017468, EPI_ISL_2017469, EPI_ISL_2017470, EPI_ISL_2017471, EPI_ISL_2017472, EPI_ISL_2017473, EPI_ISL_2017474, EPI_ISL_2017475, EPI_ISL_2017477, EPI_ISL_2017479, EPI_ISL_2017483, EPI_ISL_2017484, EPI_ISL_2102518, EPI_ISL_2102519, EPI_ISL_2102520, EPI_ISL_2102521, EPI_ISL_2187689, EPI_ISL_2187713, EPI_ISL_2187722, EPI_ISL_2187723, EPI_ISL_2187733, EPI_ISL_2187734, EPI_ISL_2187741, EPI_ISL_2187748, EPI_ISL_2187749, EPI_ISL_2187754, EPI_ISL_2187757, EPI_ISL_2187762, EPI_ISL_2187763, EPI_ISL_2187764, EPI_ISL_2187768, EPI_ISL_2187769, EPI_ISL_2187770, EPI_ISL_2187771, EPI_ISL_2187773, EPI_ISL_2187775, EPI_ISL_2187779, EPI_ISL_2187780, EPI_ISL_2187782, EPI_ISL_2187784, EPI_ISL_2187785, EPI_ISL_2187787, EPI_ISL_2187792, EPI_ISL_2187794, EPI_ISL_2187796, EPI_ISL_2187797, EPI_ISL_2187798, EPI_ISL_2187803, EPI_ISL_2187804, EPI_ISL_2187806, EPI_ISL_2187807, EPI_ISL_2187808, EPI_ISL_2187809, EPI_ISL_2187811, EPI_ISL_2187812, EPI_ISL_2187813, EPI_ISL_2187815, EPI_ISL_2187816, EPI_ISL_2187817, EPI_ISL_2187820, EPI_ISL_2187821, EPI_ISL_2187822, EPI_ISL_2187824, EPI_ISL_2187825, EPI_ISL_2187826, EPI_ISL_2187829, EPI_ISL_2187830, EPI_ISL_2187832, EPI_ISL_2187833, EPI_ISL_2187834, EPI_ISL_2187835, EPI_ISL_2187836, EPI_ISL_2187837, EPI_ISL_2187838, EPI_ISL_2187839, EPI_ISL_2187840, EPI_ISL_2187842, EPI_ISL_2187845, EPI_ISL_2187846, EPI_ISL_2187847, EPI_ISL_2187848, EPI_ISL_2187849, EPI_ISL_2187851, EPI_ISL_2187852, EPI_ISL_2187853, EPI_ISL_2187855, EPI_ISL_2187856, EPI_ISL_2187857, EPI_ISL_2187858, EPI_ISL_2187859, EPI_ISL_2187861, EPI_ISL_2187862, EPI_ISL_2187863, EPI_ISL_2187864, EPI_ISL_2187865, EPI_ISL_2187866, EPI_ISL_2187867, EPI_ISL_2187870, EPI_ISL_2187873, EPI_ISL_2187874, EPI_ISL_2187876, EPI_ISL_2187877, EPI_ISL_2187878, EPI_ISL_2187880, EPI_ISL_2187884, EPI_ISL_2187885, EPI_ISL_2187886, EPI_ISL_2187887, EPI_ISL_2187888, EPI_ISL_2187891, EPI_ISL_2187892, EPI_ISL_2187893, EPI_ISL_2187894, EPI_ISL_2187895, EPI_ISL_2187898, EPI_ISL_2187899, EPI_ISL_2187900, EPI_ISL_2187901, EPI_ISL_2187902, EPI_ISL_2187905, EPI_ISL_2187906, EPI_ISL_2187907, EPI_ISL_2187912, EPI_ISL_2187913, EPI_ISL_2187914, EPI_ISL_2187915, EPI_ISL_2187919, EPI_ISL_2187920, EPI_ISL_2187921, EPI_ISL_2187925, EPI_ISL_2187926, EPI_ISL_2187927, EPI_ISL_2187928, EPI_ISL_2187929, EPI_ISL_2187931, EPI_ISL_2187932, EPI_ISL_2187933, EPI_ISL_2187934, EPI_ISL_2187935, EPI_ISL_2187936, EPI_ISL_2187937, EPI_ISL_2187938, EPI_ISL_2187939, EPI_ISL_2187940, EPI_ISL_2187941, EPI_ISL_2187942, EPI_ISL_2187944, EPI_ISL_2187945, EPI_ISL_2187946, EPI_ISL_2187947, EPI_ISL_2187948, EPI_ISL_2187950, EPI_ISL_2187954, EPI_ISL_2187955, EPI_ISL_2187956, EPI_ISL_2187957, EPI_ISL_2187958, EPI_ISL_2187960, EPI_ISL_2187961, EPI_ISL_2187962, EPI_ISL_2187963, EPI_ISL_2187964, EPI_ISL_2187966, EPI_ISL_2187967, EPI_ISL_2187968, EPI_ISL_2187971, EPI_ISL_2187973, EPI_ISL_2187975, EPI_ISL_2187976, EPI_ISL_2187977, EPI_ISL_2187978, EPI_ISL_2187979, EPI_ISL_2187980, EPI_ISL_2187981, EPI_ISL_2187982, EPI_ISL_2187983, EPI_ISL_2187984, EPI_ISL_2222885, EPI_ISL_2222894, EPI_ISL_2227562, EPI_ISL_2227563, EPI_ISL_2348599, EPI_ISL_2348600, EPI_ISL_2348601, EPI_ISL_2348602, EPI_ISL_2348603, EPI_ISL_2466444, EPI_ISL_2466445, EPI_ISL_2466467, EPI_ISL_2497436			
see above	HLAGYN - Laboratorio de Imunologia de Transplantes de Goias	HLAGYN - Laboratorio de Imunologia de Transplantes de Goias	Alessandro Leonardo Alvares Magalhaes; Daniel Ferreira de Sousa; Danielle de Paiva Rezende; Erika Lopes Rocha Batista; Fernando Antonio Vinhal dos Santos; Frederico Rodrigues Vinhal; Lucas Carlos Gomes Pereira; Paola Cristina Resende Silva; Raphael Bessa Parmigiane; Sabrina Sara Moreira Duarte
EPI_ISL_2801311, EPI_ISL_2801323, EPI_ISL_3102226, EPI_ISL_3102228, EPI_ISL_3102229, EPI_ISL_3102230, EPI_ISL_3102231, EPI_ISL_3102232, EPI_ISL_3102233, EPI_ISL_3102234, EPI_ISL_3102235, EPI_ISL_3102236, EPI_ISL_3102271, EPI_ISL_3102415, EPI_ISL_3102416, EPI_ISL_3102418, EPI_ISL_3102425, EPI_ISL_3102433, EPI_ISL_3102443, EPI_ISL_3102445, EPI_ISL_3102447, EPI_ISL_3102452, EPI_ISL_3102467, EPI_ISL_3102468, EPI_ISL_3102470, EPI_ISL_3102473, EPI_ISL_3912174, EPI_ISL_3912175, EPI_ISL_3912176, EPI_ISL_3912177, EPI_ISL_3912178, EPI_ISL_3912179, EPI_ISL_3912180, EPI_ISL_3912181, EPI_ISL_3912182, EPI_ISL_3912183, EPI_ISL_3912185, EPI_ISL_3912186, EPI_ISL_3912187			
see above	HM HOSPITAL DE MESSEJANA DR CARLOS ALBERTO STUDART GOMES	Analytical Competence Molecular Epidemiology Lab/ACME, Oswaldo Cruz Foundation, Ceara (FIOCRUZ CE)	Cleber Furtado Aksenen; Cleber Furtado Aksenen e Suzana Porto Almeida; Fabio Miyajima; Fernando Braga Stehling; Francisco Eder de Moura Lopes; Jamille Maria Mendes Bezerra; Joaquim Cesar do Nascimento Sousa Junior; Joaquim César do Nascimento Sousa Junior; Pedro Miguel Carneiro Jeronimo; Suzana Porto Almeida & Lucas Delerino on behalf of COVID-19 FIOCRUZ Genomic Network; Suzana Porto Almeida e Lucas Delerino; Thais Ferreira de Oliveira; Thais de Oliveira Costa; Ticiane Cavalcante de Souza; Veridiana Pessoa Miyajima
EPI_ISL_1811236	HNN	Inciensa, Instituto Costarricense de Investigación y Enseñanza en Nutrición y Salud	Pérez-Corrales C & Catalán-Marín F
EPI_ISL_2142666	HOPITAL SAINT ANDRE	CNR Virus des Infections Respiratoires - France SUD	Antonin Bal; Bruno Lina; Gregory Destras; Gwendolyne Burfin; Hadrien Regue; Laurence Josset; Martine Valette; Quentin Semanas
EPI_ISL_2345972	HOSP DO SERV PUB ESTFCO MORATO DE OLIVEIRA SAO PAULO	Instituto Butantan	Antonio Jorge Martins; Claudia Renata dos Santos Barros; David Schlesinger; Debora Botequio Moretti; Dimas Tadeu Covas; Elaine Cristina Marqueze; Elaine Vieira Santos; Evandra Strazza Rodrigues; Heidge Fukumasu; Jayme Augusto de Souza-Neto; José Salvatore Leister Patané; Luiz Alcantara; Luiz Lehmann Coutinho; Maria Carolina Elias; Maurício Lacerda Nogueira; Rafael dos Santos Bezerra; Raul Machado Neto; Rejane Maria Tommasini Grotto; Ricardo Haddad; Sandra Coccuzzo Sampaio Vessoni; Simone Kashima; Svetoslav Nanev Slavov; Vincent Louis Viala
EPI_ISL_1967093, EPI_ISL_1967094, EPI_ISL_1967095	HOSP DO SERV PUB ESTFCO MORATO DE OLIVEIRA SAO PAULO	Instituto Butantan / ESALQ-USP (Piracicaba)	Antonio Jorge Martins; Bianca Cechetto Carlos. Mendelics: Bibiana Santos; Claudia Renata dos Santos Barros; Cíntia Bittar; David Schlesinger. Hemocentro Ribeirão Preto: Simone Kashima; Debora Botequio Moretti; Elaine Cristina Marqueze; Elaine Vieira dos Santos; Elisangela Chicaroni Mattos; Erika Freitas; Evandra Strazza Rodrigues; Felipe Allan da Silva da Costa; Flavia Aburjaile; Fábio Sossai Possebon; Guilherme Campos; Guilherme Targino Valente; Heidge Fukumasu. USP-Botucatu: Rejane Maria Tommasini Grotto; Helena Lage Ferreira; Instituto Butantan: Dimas Tadeu Covas; Jardelina de Souza Todao Bernardino; Jayme A. Souza-Neto; Jessika Cristina Chagas Lesbon; Jorge A. Petroli Marchesi; José Salvatore Leister Patané; João Paulo Kitajima; João Pessoa Araújo Jr.; Leila Sabrina Ullmann; Loyze Paola Oliveira de Lima; Luiz Aurelio de Campos Crispin. Centro de Genômica Funcional da ESALQ: Luiz Lehmann Coutinho; Luiz Carlos Junior de Alcantara; Lívia Sacchetto; Maisa C. Pereira Parra; Maria Carolina Elias; Marta Giovanetti; Marília Moraes; Maurício Lacerda Nogueira. Prefeitura de Sao Paulo: Melissa Palmieri.; Patricia Akemi Assato; Paula Rahal; Paulo Inacio da Costa; Rafael dos Santos Bezerra; Raquel de Lello Rocha Campos Cassano. NGS Soluções Genômicas: Pilar Drummond Sampaio Corrêa Mariani. FZEA-USP Pirassununga: Mirele Daiana Poleti; Raul Machado Neto; Ricardo Augusto Brassaloti; Ricardo Haddad; Rodrigo Tocantins Calado. FAMERP-SJRP: Cecília Artico Banho; Sandra Coccuzzo Sampaio; Svetoslav Nanev Slavov; Vagner Fonseca; Vincent Louis Viala
EPI_ISL_2209204	HOSP E MATERNIDADE MUNICIPAL N SRA MONTE SERRAT	Instituto Butantan	Antonio Jorge Martins; Claudia Renata dos Santos Barros; David Schlesinger; Debora Botequio Moretti; Dimas Tadeu Covas; Elaine Cristina Marqueze; Elaine Vieira Santos; Evandra Strazza Rodrigues; Heidge Fukumasu; Jayme Augusto de Souza-Neto; José Salvatore Leister Patané; Luiz Alcantara; Luiz Lehmann Coutinho; Maria Carolina Elias; Maurício Lacerda Nogueira; Rafael dos Santos Bezerra; Raul Machado Neto; Rejane Maria Tommasini Grotto; Ricardo Haddad; Sandra Coccuzzo Sampaio Vessoni; Simone Kashima; Svetoslav Nanev Slavov; Vincent Louis Viala.
EPI_ISL_2344947	HOSP E MATERNIDADE MUNICIPAL N SRA MONTE SERRAT	Instituto Butantan / ESALQ-Piracicaba	Antonio Jorge Martins; Claudia Renata dos Santos Barros; David Schlesinger; Debora Botequio Moretti; Dimas Tadeu Covas; Elaine Cristina Marqueze; Elaine Vieira Santos; Evandra Strazza Rodrigues; Heidge Fukumasu; Jayme Augusto de Souza-Neto; José Salvatore Leister Patané; Luiz Alcantara; Luiz Lehmann Coutinho; Maria Carolina Elias; Maurício Lacerda Nogueira; Rafael dos Santos Bezerra; Raul Machado Neto; Rejane Maria Tommasini Grotto; Ricardo Haddad; Sandra Coccuzzo Sampaio Vessoni; Simone Kashima; Svetoslav Nanev Slavov; Vincent Louis Viala
EPI_ISL_1967029, EPI_ISL_1967030, EPI_ISL_1967031	HOSP E MATERNIDADE MUNICIPAL N SRA MONTE SERRAT	Instituto Butantan / ESALQ-USP (Piracicaba)	Antonio Jorge Martins; Bianca Cechetto Carlos. Mendelics: Bibiana Santos; Claudia Renata dos Santos Barros; Cíntia Bittar; David Schlesinger. Hemocentro Ribeirão Preto: Simone Kashima; Debora Botequio Moretti; Elaine Cristina Marqueze; Elaine Vieira dos Santos; Elisangela Chicaroni Mattos; Erika Freitas; Evandra Strazza Rodrigues; Felipe Allan da Silva da Costa; Flavia Aburjaile; Fábio Sossai Possebon; Guilherme Campos; Guilherme Targino Valente; Heidge Fukumasu. USP-Botucatu: Rejane Maria Tommasini Grotto; Helena Lage Ferreira; Instituto Butantan: Dimas Tadeu Covas; Jardelina de Souza Todao Bernardino; Jayme A. Souza-Neto; Jessika Cristina Chagas Lesbon; Jorge A. Petroli Marchesi; José Salvatore Leister Patané; João Paulo Kitajima; João Pessoa Araújo Jr.; Leila Sabrina Ullmann; Loyze Paola Oliveira de Lima; Luiz Aurelio de Campos Crispin. Centro de Genômica Funcional da ESALQ: Luiz Lehmann Coutinho; Luiz Carlos Junior de Alcantara; Lívia Sacchetto; Maisa C. Pereira Parra; Maria Carolina Elias; Marta Giovanetti; Marília Moraes; Maurício Lacerda Nogueira. Prefeitura de Sao Paulo: Melissa Palmieri.; Patricia Akemi Assato; Paula Rahal; Paulo Inacio da Costa; Rafael dos Santos Bezerra; Raquel de Lello Rocha Campos Cassano. NGS Soluções Genômicas: Pilar Drummond Sampaio Corrêa Mariani. FZEA-USP Pirassununga: Mirele Daiana Poleti; Raul Machado Neto; Ricardo Augusto Brassaloti; Ricardo Haddad; Rodrigo Tocantins Calado. FAMERP-SJRP: Cecília Artico Banho; Sandra Coccuzzo Sampaio; Svetoslav Nanev Slavov; Vagner Fonseca; Vincent Louis Viala
EPI_ISL_2170937	HOSP E MATERNIDADE MUNICIPAL N SRA MONTE SERRAT	Instituto Butantan / Mendelics	Antonio Jorge Martins; Bianca Cechetto Carlos. Mendelics: Bibiana Santos; Claudia Renata dos Santos Barros; Cíntia Bittar; David Schlesinger. Hemocentro Ribeirão Preto: Simone Kashima; Debora Botequio Moretti; Elaine Cristina Marqueze; Elaine Vieira dos Santos; Elisangela Chicaroni Mattos; Erika Freitas; Evandra Strazza Rodrigues; Felipe Allan da Silva da Costa; Flavia Aburjaile; Fábio Sossai Possebon; Guilherme Campos; Guilherme Targino Valente; Heidge Fukumasu. USP-Botucatu: Rejane Maria Tommasini Grotto; Helena Lage Ferreira; Instituto Butantan: Dimas Tadeu Covas; Jardelina de Souza Todao Bernardino; Jayme A. Souza-Neto; Jessika Cristina Chagas Lesbon; Jorge A. Petroli Marchesi; José Salvatore Leister Patané; João Paulo Kitajima; João Pessoa Araújo Jr.; Leila Sabrina Ullmann; Loyze Paola Oliveira de Lima; Luiz Aurelio de Campos Crispin. Centro de Genômica Funcional da ESALQ: Luiz Lehmann Coutinho; Luiz Carlos Junior de Alcantara; Lívia Sacchetto; Maisa C. Pereira Parra; Maria Carolina Elias; Marta Giovanetti; Marília Moraes; Maurício Lacerda Nogueira. Prefeitura de Sao Paulo: Melissa Palmieri.; Patricia Akemi Assato; Paula Rahal; Paulo Inacio da Costa; Rafael dos Santos Bezerra; Raquel de Lello Rocha Campos Cassano. NGS Soluções Genômicas: Pilar Drummond Sampaio Corrêa Mariani. FZEA-USP Pirassununga: Mirele Daiana Poleti; Raul Machado Neto; Ricardo Augusto Brassaloti; Ricardo Haddad; Rodrigo Tocantins Calado. FAMERP-SJRP: Cecília Artico Banho; Sandra Coccuzzo Sampaio; Svetoslav Nanev Slavov; Vagner Fonseca; Vincent Louis Viala
EPI_ISL_2344546	HOSP E MATERNIDADE MUNICIPAL N SRA MONTE SERRAT	Instituto Butantan / UNESP-Botucatu	Antonio Jorge Martins; Claudia Renata dos Santos Barros; David Schlesinger; Debora Botequio Moretti; Dimas Tadeu Covas; Elaine Cristina Marqueze; Elaine Vieira Santos; Evandra Strazza Rodrigues; Heidge Fukumasu; Jayme Augusto de Souza-Neto; José Salvatore Leister Patané; Luiz Alcantara; Luiz Lehmann Coutinho; Maria Carolina Elias; Maurício Lacerda Nogueira; Rafael dos Santos Bezerra; Raul Machado Neto; Rejane Maria Tommasini Grotto; Ricardo Haddad; Sandra Coccuzzo Sampaio Vessoni; Simone Kashima; Svetoslav Nanev Slavov; Vincent Louis Viala
EPI_ISL_3912189	HOSP MATERN JULIA JORGE	Analytical Competence Molecular Epidemiology Lab/ACME, Oswaldo Cruz Foundation, Ceara (FIOCRUZ CE)	Cleber Furtado Aksenen; Fabio Miyajima; Fernando Braga Stehling; Francisco Eder de Moura Lopes; Jamille Maria Mendes Bezerra; Joaquim Cesar do Nascimento Sousa Junior; Pedro Miguel Carneiro Jeronimo; Suzana Porto Almeida & Lucas Delerino on behalf of COVID-19 FIOCRUZ Genomic Network; Thais Ferreira de Oliveira; Thais de Oliveira Costa; Ticiane Cavalcante de Souza; Veridiana Pessoa Miyajima

EPI_ISL_3102259	HOSP MATERN SANTA IZABEL ARACOIABA	Analytical Competence Molecular Epidemiology Lab/ACME, Oswaldo Cruz Foundation, Ceara (FIOCRUZ CE)	Cleber Furtado Aksenen; Fabio Miyajima; Fernando Braga Stehling; Francisco Eder de Moura Lopes; Jamille Maria Mendes Bezerra; Joaquim César do Nascimento Sousa Junior; Pedro Miguel Carneiro Jeronimo; Suzana Porto Almeida e Lucas Delerino; Thais Ferreira de Oliveira; Thais de Oliveira Costa; Ticiane Cavalcante de Souza; Veridiana Pessoa Miyajima
EPI_ISL_3912190	HOSP MATERNIDADE MARIA TEREZA DE JESUS MATEUS	Analytical Competence Molecular Epidemiology Lab/ACME, Oswaldo Cruz Foundation, Ceara (FIOCRUZ CE)	Cleber Furtado Aksenen; Fabio Miyajima; Fernando Braga Stehling; Francisco Eder de Moura Lopes; Jamille Maria Mendes Bezerra; Joaquim Cesar do Nascimento Sousa Junior; Pedro Miguel Carneiro Jeronimo; Suzana Porto Almeida & Lucas Delerino on behalf of COVID-19 FIOCRUZ Genomic Network; Thais Ferreira de Oliveira; Thais de Oliveira Costa; Ticiane Cavalcante de Souza; Veridiana Pessoa Miyajima
EPI_ISL_3912192	HOSP MUN ABELARDO GADELHA DA ROCHA	Analytical Competence Molecular Epidemiology Lab/ACME, Oswaldo Cruz Foundation, Ceara (FIOCRUZ CE)	Cleber Furtado Aksenen; Fabio Miyajima; Fernando Braga Stehling; Francisco Eder de Moura Lopes; Jamille Maria Mendes Bezerra; Joaquim Cesar do Nascimento Sousa Junior; Pedro Miguel Carneiro Jeronimo; Suzana Porto Almeida & Lucas Delerino on behalf of COVID-19 FIOCRUZ Genomic Network; Thais Ferreira de Oliveira; Thais de Oliveira Costa; Ticiane Cavalcante de Souza; Veridiana Pessoa Miyajima
EPI_ISL_2345997, EPI_ISL_2346003, EPI_ISL_2346004	HOSP MUN CIDADE TIRADENTES CARMEN PRUDENTE	Instituto Butantan	Antonio Jorge Martins; Claudia Renata dos Santos Barros; David Schlesinger; Debora Botequio Moretti; Dimas Tadeu Covas; Elaine Cristina Marqueze; Elaine Vieira Santos; Evandra Strazza Rodrigues; Heidge Fukumasu; Jayme Augusto de Souza-Neto; José Salvatore Leister Patané; Luiz Alcantara; Luiz Lehmann Coutinho; Maria Carolina Elias; Maurício Lacerda Nogueira; Rafael dos Santos Bezerra; Raul Machado Neto; Rejane Maria Tommasini Grotto; Ricardo Haddad; Sandra Coccuzzo Sampaio Vessoni; Simone Kashima; Svetoslav Nanev Slavov; Vincent Louis Viala
EPI_ISL_1967070, EPI_ISL_1967071, EPI_ISL_1967072	HOSP MUN CIDADE TIRADENTES CARMEN PRUDENTE	Instituto Butantan / ESALQ-USP (Piracicaba)	Antonio Jorge Martins; Bianca Cechetto Carlos. Mendelics: Bibiana Santos; Claudia Renata dos Santos Barros; Cíntia Bittar; David Schlesinger. Hemocentro Ribeirão Preto: Simone Kashima; Debora Botequio Moretti; Elaine Cristina Marqueze; Elaine Vieira dos Santos; Elisangela Chicaroni Mattos; Erika Freitas; Evandra Strazza Rodrigues; Felipe Allan da Silva da Costa; Flavia Aburjaile; Fábio Sossai Possebon; Guilherme Campos; Guilherme Targino Valente; Heidge Fukumasu. USP-Botucatu: Rejane Maria Tommasini Grotto; Helena Lage Ferreira; Instituto Butantan: Dimas Tadeu Covas; Jardelina de Souza Todao Bernardino; Jayme A. Souza-Neto; Jessika Cristina Chagas Lesbon; Jorge A. Petroli Marchesi; José Salvatore Leister Patané; João Paulo Kitajima; João Pessoa Araújo Jr.; Leila Sabrina Ullmann; Loyze Paola Oliveira de Lima; Luiz Aurelio de Campos Crispin. Centro de Genômica Funcional da ESALQ: Luiz Lehmann Coutinho; Luiz Carlos Junior de Alcantara; Lívia Sacchetto; Maisa C. Pereira Parra; Maria Carolina Elias; Marta Giovanetti; Marília Moraes; Maurício Lacerda Nogueira. Prefeitura de Sao Paulo: Melissa Palmieri.; Patricia Akemi Assato; Paula Rahal; Paulo Inacio da Costa; Rafael dos Santos Bezerra; Raquel de Lello Rocha Campos Cassano. NGS Soluções Genômicas: Pilar Drummond Sampaio Corrêa Mariani. FZEA-USP Pirassununga: Mirele Daiana Poleti; Raul Machado Neto; Ricardo Augusto Brassaloti; Ricardo Haddad; Rodrigo Tocantins Calado. FAMERP-SJRP: Cecília Artico Banho; Sandra Coccuzzo Sampaio; Svetoslav Nanev Slavov; Vagner Fonseca; Vincent Louis Viala
EPI_ISL_1966737	HOSP MUN JOSANIAS CASTANHA BRAGA	Instituto Butantan	Antonio Jorge Martins; Bianca Cechetto Carlos. Mendelics: Bibiana Santos; Claudia Renata dos Santos Barros; Cíntia Bittar; David Schlesinger. Hemocentro Ribeirão Preto: Simone Kashima; Debora Botequio Moretti; Elaine Cristina Marqueze; Elaine Vieira dos Santos; Elisangela Chicaroni Mattos; Erika Freitas; Evandra Strazza Rodrigues; Felipe Allan da Silva da Costa; Flavia Aburjaile; Fábio Sossai Possebon; Guilherme Campos; Guilherme Targino Valente; Heidge Fukumasu. USP-Botucatu: Rejane Maria Tommasini Grotto; Helena Lage Ferreira; Instituto Butantan: Dimas Tadeu Covas; Jardelina de Souza Todao Bernardino; Jayme A. Souza-Neto; Jessika Cristina Chagas Lesbon; Jorge A. Petroli Marchesi; José Salvatore Leister Patané; João Paulo Kitajima; João Pessoa Araújo Jr.; Leila Sabrina Ullmann; Loyze Paola Oliveira de Lima; Luiz Aurelio de Campos Crispin. Centro de Genômica Funcional da ESALQ: Luiz Lehmann Coutinho; Luiz Carlos Junior de Alcantara; Lívia Sacchetto; Maisa C. Pereira Parra; Maria Carolina Elias; Marta Giovanetti; Marília Moraes; Maurício Lacerda Nogueira. Prefeitura de Sao Paulo: Melissa Palmieri.; Patricia Akemi Assato; Paula Rahal; Paulo Inacio da Costa; Rafael dos Santos Bezerra; Raquel de Lello Rocha Campos Cassano. NGS Soluções Genômicas: Pilar Drummond Sampaio Corrêa Mariani. FZEA-USP Pirassununga: Mirele Daiana Poleti; Raul Machado Neto; Ricardo Augusto Brassaloti; Ricardo Haddad; Rodrigo Tocantins Calado. FAMERP-SJRP: Cecília Artico Banho; Sandra Coccuzzo Sampaio; Svetoslav Nanev Slavov; Vagner Fonseca; Vincent Louis Viala
EPI_ISL_3912193	HOSP MUNIC ANTONIO NERY FILHO	Analytical Competence Molecular Epidemiology Lab/ACME, Oswaldo Cruz Foundation, Ceara (FIOCRUZ CE)	Cleber Furtado Aksenen; Fabio Miyajima; Fernando Braga Stehling; Francisco Eder de Moura Lopes; Jamille Maria Mendes Bezerra; Joaquim Cesar do Nascimento Sousa Junior; Pedro Miguel Carneiro Jeronimo; Suzana Porto Almeida & Lucas Delerino on behalf of COVID-19 FIOCRUZ Genomic Network; Thais Ferreira de Oliveira; Thais de Oliveira Costa; Ticiane Cavalcante de Souza; Veridiana Pessoa Miyajima
EPI_ISL_1967067	HOSP PRONTO SOCORRO ITAQUERA	Instituto Butantan / ESALQ-USP (Piracicaba)	Antonio Jorge Martins; Bianca Cechetto Carlos. Mendelics: Bibiana Santos; Claudia Renata dos Santos Barros; Cíntia Bittar; David Schlesinger. Hemocentro Ribeirão Preto: Simone Kashima; Debora Botequio Moretti; Elaine Cristina Marqueze; Elaine Vieira dos Santos; Elisangela Chicaroni Mattos; Erika Freitas; Evandra Strazza Rodrigues; Felipe Allan da Silva da Costa; Flavia Aburjaile; Fábio Sossai Possebon; Guilherme Campos; Guilherme Targino Valente; Heidge Fukumasu. USP-Botucatu: Rejane Maria Tommasini Grotto; Helena Lage Ferreira; Instituto Butantan: Dimas Tadeu Covas; Jardelina de Souza Todao Bernardino; Jayme A. Souza-Neto; Jessika Cristina Chagas Lesbon; Jorge A. Petroli Marchesi; José Salvatore Leister Patané; João Paulo Kitajima; João Pessoa Araújo Jr.; Leila Sabrina Ullmann; Loyze Paola Oliveira de Lima; Luiz Aurelio de Campos Crispin. Centro de Genômica Funcional da ESALQ: Luiz Lehmann Coutinho; Luiz Carlos Junior de Alcantara; Lívia Sacchetto; Maisa C. Pereira Parra; Maria Carolina Elias; Marta Giovanetti; Marília Moraes; Maurício Lacerda Nogueira. Prefeitura de Sao Paulo: Melissa Palmieri.; Patricia Akemi Assato; Paula Rahal; Paulo Inacio da Costa; Rafael dos Santos Bezerra; Raquel de Lello Rocha Campos Cassano. NGS Soluções Genômicas: Pilar Drummond Sampaio Corrêa Mariani. FZEA-USP Pirassununga: Mirele Daiana Poleti; Raul Machado Neto; Ricardo Augusto Brassaloti; Ricardo Haddad; Rodrigo Tocantins Calado. FAMERP-SJRP: Cecília Artico Banho; Sandra Coccuzzo Sampaio; Svetoslav Nanev Slavov; Vagner Fonseca; Vincent Louis Viala
EPI_ISL_3102217	HOSPITAL ANTONIO PRUDENTE	Analytical Competence Molecular Epidemiology Lab/ACME, Oswaldo Cruz Foundation, Ceara (FIOCRUZ CE)	Cleber Furtado Aksenen; Fabio Miyajima; Fernando Braga Stehling; Francisco Eder de Moura Lopes; Jamille Maria Mendes Bezerra; Joaquim César do Nascimento Sousa Junior; Pedro Miguel Carneiro Jeronimo; Suzana Porto Almeida e Lucas Delerino; Thais Ferreira de Oliveira; Thais de Oliveira Costa; Ticiane Cavalcante de Souza; Veridiana Pessoa Miyajima
EPI_ISL_2801321	HOSPITAL ANTONIO ROSENO DE MATOS	Analytical Competence Molecular Epidemiology Lab/ACME, Oswaldo Cruz Foundation, Ceara (FIOCRUZ CE)	Cleber Furtado Aksenen e Suzana Porto Almeida; Fabio Miyajima; Fernando Braga Stehling; Francisco Eder de Moura Lopes; Jamille Maria Mendes Bezerra; Joaquim César do Nascimento Sousa Junior; Pedro Miguel Carneiro Jeronimo; Thais Ferreira de Oliveira; Thais de Oliveira Costa; Ticiane Cavalcante de Souza; Veridiana Pessoa Miyajima
EPI_ISL_2020126	HOSPITAL CAN MISSES	HOSPITAL UNIVERSITARIO SON ESPASES	Antonio Oliver; Carla López-Causapé; Pablo Fraile-Ribot; SeqCovid
EPI_ISL_3102308, EPI_ISL_3102316	HOSPITAL CURA DARS	Analytical Competence Molecular Epidemiology Lab/ACME, Oswaldo Cruz Foundation, Ceara (FIOCRUZ CE)	Cleber Furtado Aksenen; Fabio Miyajima; Fernando Braga Stehling; Francisco Eder de Moura Lopes; Jamille Maria Mendes Bezerra; Joaquim César do Nascimento Sousa Junior; Pedro Miguel Carneiro Jeronimo; Suzana Porto Almeida e Lucas Delerino; Thais Ferreira de Oliveira; Thais de Oliveira Costa; Ticiane Cavalcante de Souza; Veridiana Pessoa Miyajima
EPI_ISL_2346002	HOSPITAL DE CAMPANHA COVID 19 CAIEIRAS	Instituto Butantan	Antonio Jorge Martins; Claudia Renata dos Santos Barros; David Schlesinger; Debora Botequio Moretti; Dimas Tadeu Covas; Elaine Cristina Marqueze; Elaine Vieira Santos; Evandra Strazza Rodrigues; Heidge Fukumasu; Jayme Augusto de Souza-Neto; José Salvatore Leister Patané; Luiz Alcantara; Luiz Lehmann Coutinho; Maria Carolina Elias; Maurício Lacerda Nogueira; Rafael dos Santos Bezerra; Raul Machado Neto; Rejane Maria Tommasini Grotto; Ricardo Haddad; Sandra Coccuzzo Sampaio Vessoni; Simone Kashima; Svetoslav Nanev Slavov; Vincent Louis Viala
EPI_ISL_2210253, EPI_ISL_2362266	HOSPITAL DE CAMPANHA COVID 19 CAIEIRAS	Instituto Butantan / Mendelics	Antonio Jorge Martins; Claudia Renata dos Santos Barros; David Schlesinger; Debora Botequio Moretti; Dimas Tadeu Covas; Elaine Cristina Marqueze; Elaine Vieira Santos; Evandra Strazza Rodrigues; Heidge Fukumasu; Jayme Augusto de Souza-Neto; José Salvatore Leister Patané; Luiz Alcantara; Luiz Lehmann Coutinho; Maria Carolina Elias; Maurício Lacerda Nogueira; Rafael dos Santos Bezerra; Raul Machado Neto; Rejane Maria Tommasini Grotto; Ricardo Haddad; Sandra Coccuzzo Sampaio Vessoni; Simone Kashima; Svetoslav Nanev Slavov; Vincent Louis Viala
EPI_ISL_1795345, EPI_ISL_2345612	HOSPITAL DE CAMPANHA COVID 19 MUNICIPIO DE TAUBATE	Instituto Butantan / ESALQ-Piracicaba	Antonio Jorge Martins; Bianca Cechetto Carlos. Mendelics: Bibiana Santos; Claudia Renata dos Santos Barros; David Schlesinger; David Schlesinger. Hemocentro Ribeirão Preto: Simone Kashima; Debora Botequio Moretti; Debora Botequio Moretti. Centro de Genômica Funcional da ESALQ: Luiz Lehmann Coutinho; Dimas Tadeu Covas; Elaine Cristina Marqueze; Elaine Vieira Santos; Elaine Vieira dos Santos; Elisangela Chicaroni Mattos; Erika Freitas; Evandra Strazza Rodrigues; Felipe Allan da Silva da Costa; Flavia Aburjaile; Guilherme Targino Valente; Heidge Fukumasu; Heidge Fukumasu. USP-Botucatu: Rejane Maria Tommasini Grotto; Instituto Butantan: Alexander Roberto Precioso; Jayme A. Souza-Neto; Jayme Augusto de Souza-Neto; Jessika Cristina Chagas Lesbon; José Salvatore Leister Patané; João Paulo Kitajima; Luiz Alcantara; Luiz Carlos Junior de Alcantara; Luiz Lehmann Coutinho; Maria Carolina Elias; Marta Giovanetti; Maurício Lacerda Nogueira; Patricia Akemi Assato; Rafael dos Santos Bezerra; Raquel de Lello Rocha Campos Cassano. NGS Soluções Genômicas: Pilar Drummond Sampaio Corrêa Mariani. FZEA-USP Pirassununga: Mirele Daiana Poleti; Raul Machado Neto; Rejane Maria Tommasini Grotto; Ricardo Augusto Brassaloti; Ricardo Haddad; Rodrigo Tocantins Calado.; Sandra Coccuzzo Sampaio; Sandra Coccuzzo Sampaio Vessoni; Simone Kashima; Svetoslav Nanev Slavov; Vagner Fonseca; Vincent Louis Viala
EPI_ISL_1967165, EPI_ISL_1967166, EPI_ISL_1967167, EPI_ISL_1967168, EPI_ISL_1967169, EPI_ISL_1967170, EPI_ISL_1967171, EPI_ISL_1967172, EPI_ISL_1967173, EPI_ISL_1967174, EPI_ISL_1967175, EPI_ISL_1967176, EPI_ISL_1967178, EPI_ISL_1967179, EPI_ISL_1967180, EPI_ISL_1967181, EPI_ISL_1967182, EPI_ISL_1967183, EPI_ISL_1967185, EPI_ISL_1967186, EPI_ISL_1967187, EPI_ISL_1967188, EPI_ISL_1967189, EPI_ISL_1967190, EPI_ISL_1967191			
see above	HOSPITAL DE CAMPANHA COVID 19 MUNICIPIO DE TAUBATE	Instituto Butantan / ESALQ-USP (Piracicaba)	Antonio Jorge Martins; Bianca Cechetto Carlos. Mendelics: Bibiana Santos; Claudia Renata dos Santos Barros; Cíntia Bittar; David Schlesinger. Hemocentro Ribeirão Preto: Simone Kashima; Debora Botequio Moretti; Elaine Cristina Marqueze; Elaine Vieira dos Santos; Elisangela Chicaroni Mattos; Erika Freitas; Evandra Strazza Rodrigues; Felipe Allan da Silva da Costa; Flavia Aburjaile; Fábio Sossai Possebon; Guilherme Campos; Guilherme Targino Valente; Heidge Fukumasu. USP-Botucatu: Rejane Maria Tommasini Grotto; Helena Lage Ferreira; Instituto Butantan: Dimas Tadeu Covas; Jardelina de Souza Todao Bernardino; Jayme A. Souza-Neto; Jessika Cristina Chagas Lesbon; Jorge A. Petroli Marchesi; José Salvatore Leister Patané; João Paulo Kitajima; João Pessoa Araújo Jr.; Leila Sabrina Ullmann; Loyze Paola Oliveira de Lima; Luiz Aurelio de Campos Crispin. Centro de Genômica Funcional da ESALQ: Luiz Lehmann Coutinho; Luiz Carlos Junior de Alcantara; Lívia Sacchetto; Maisa C. Pereira Parra; Maria Carolina Elias; Marta Giovanetti; Marília Moraes; Maurício Lacerda Nogueira. Prefeitura de Sao Paulo: Melissa Palmieri.; Patricia Akemi Assato; Paula Rahal; Paulo Inacio da Costa; Rafael dos Santos Bezerra; Raquel de Lello Rocha Campos Cassano. NGS Soluções Genômicas: Pilar Drummond Sampaio Corrêa Mariani. FZEA-USP Pirassununga: Mirele Daiana Poleti; Raul Machado Neto; Ricardo Augusto Brassaloti; Ricardo Haddad; Rodrigo Tocantins Calado. FAMERP-SJRP: Cecília Artico Banho; Sandra Coccuzzo Sampaio; Svetoslav Nanev Slavov; Vagner Fonseca; Vincent Louis Viala
EPI_ISL_3102272	HOSPITAL DE PEQUENO PORTE SAO FRANCISCO SALITRE	Analytical Competence Molecular Epidemiology Lab/ACME, Oswaldo Cruz Foundation, Ceara (FIOCRUZ CE)	Cleber Furtado Aksenen; Fabio Miyajima; Fernando Braga Stehling; Francisco Eder de Moura Lopes; Jamille Maria Mendes Bezerra; Joaquim César do Nascimento Sousa Junior; Pedro Miguel Carneiro Jeronimo; Suzana Porto Almeida e Lucas Delerino; Thais Ferreira de Oliveira; Thais de Oliveira Costa; Ticiane Cavalcante de Souza; Veridiana Pessoa Miyajima
EPI_ISL_1795101, EPI_ISL_1795106, EPI_ISL_2344550, EPI_ISL_2344556	HOSPITAL DOS FORNCEDORES	Instituto Butantan / ESALQ-Piracicaba	Antonio Jorge Martins; Bianca Cechetto Carlos. Mendelics: Bibiana Santos; Claudia Renata dos Santos Barros; David Schlesinger; David Schlesinger. Hemocentro Ribeirão Preto: Simone Kashima; Debora Botequio Moretti; Debora Botequio Moretti. Centro de Genômica Funcional da ESALQ: Luiz Lehmann Coutinho; Dimas Tadeu Covas; Elaine Cristina Marqueze; Elaine Vieira Santos; Elaine Vieira dos Santos; Elisangela Chicaroni Mattos; Erika Freitas; Evandra Strazza Rodrigues; Felipe Allan da Silva da Costa; Flavia Aburjaile; Guilherme Targino Valente; Heidge Fukumasu; Heidge Fukumasu. USP-Botucatu: Rejane Maria Tommasini Grotto; Instituto Butantan: Alexander Roberto Precioso; Jayme A. Souza-Neto; Jayme Augusto de Souza-Neto; Jessika Cristina Chagas Lesbon; José Salvatore Leister Patané; João Paulo Kitajima; Luiz Alcantara; Luiz Carlos Junior de Alcantara; Luiz Lehmann Coutinho; Maria Carolina Elias; Marta Giovanetti; Maurício Lacerda Nogueira; Patricia Akemi Assato; Rafael dos Santos Bezerra; Raquel de Lello Rocha Campos Cassano. NGS Soluções Genômicas: Pilar Drummond Sampaio Corrêa Mariani. FZEA-USP Pirassununga: Mirele Daiana Poleti; Raul Machado Neto; Rejane Maria Tommasini Grotto; Ricardo Augusto Brassaloti; Ricardo Haddad; Rodrigo Tocantins Calado.; Sandra Coccuzzo Sampaio; Sandra Coccuzzo Sampaio Vessoni; Simone Kashima; Svetoslav Nanev Slavov; Vagner Fonseca; Vincent Louis Viala
EPI_ISL_3102321	HOSPITAL DR ALBERTO FEITOSA LIMA	Analytical Competence Molecular Epidemiology Lab/ACME, Oswaldo Cruz Foundation, Ceara (FIOCRUZ CE)	Cleber Furtado Aksenen; Fabio Miyajima; Fernando Braga Stehling; Francisco Eder de Moura Lopes; Jamille Maria Mendes Bezerra; Joaquim César do Nascimento Sousa Junior; Pedro Miguel Carneiro Jeronimo; Suzana Porto Almeida e Lucas Delerino; Thais Ferreira de Oliveira; Thais de Oliveira Costa; Ticiane Cavalcante de Souza; Veridiana Pessoa Miyajima
EPI_ISL_1712404, EPI_ISL_2272959	HOSPITAL DR. ENRIQUE BALTODANO BRICEÑO	Inciensa, Instituto Costarricense de Investigación y Enseñanza en Nutrición y Salud	Adriana Godínez; Claudio Soto-Garita; Estela Cordero; Francisco Duarte; Hebleen Porras; Joselyn Prado & Adriana Bermúdez-Espinoza; Joselyn Prado & Arnoldo Zamora-Villegas; José Luis Vargas; Mariela Gutiérrez; Melany Calderón
EPI_ISL_2272986	HOSPITAL DR. MAX PERALTA JIMENEZ	Inciensa, Instituto Costarricense de Investigación y Enseñanza en Nutrición y Salud	Adriana Godínez; Claudio Soto-Garita; Estela Cordero; Francisco Duarte; Hebleen Porras; Joselyn Prado & Mariana Piedra Calderón; José Luis Vargas; Mariela Gutiérrez; Melany Calderón
EPI_ISL_3102479	HOSPITAL E	Analytical Competence	Cleber Furtado Aksenen; Fabio Miyajima; Fernando Braga Stehling; Francisco Eder de Moura Lopes; Jamille Maria Mendes Bezerra; Joaquim César do Nascimento Sousa Junior; Pedro Miguel Carneiro Jeronimo; Suzana Porto Almeida e Lucas Delerino; Thais Ferreira de Oliveira; Thais de Oliveira Costa; Ticiane Cavalcante de Souza;

	MATERNIDADE ADOLFO BEZERRA DE MENEZES	Molecular Epidemiology Lab/ACME, Oswaldo Cruz Foundation, Ceara (FIOCRUZ CE)	Veridiana Pessoa Miyajima
EPI_ISL_2801310, EPI_ISL_3102221, EPI_ISL_3102222, EPI_ISL_3102223, EPI_ISL_3102224, EPI_ISL_3102333, EPI_ISL_3102343, EPI_ISL_3102373, EPI_ISL_3102377, EPI_ISL_3102379, EPI_ISL_3102390, EPI_ISL_3912196, EPI_ISL_3912197			
see above	HOSPITAL E MATERNIDADE DRA ZILDA ARNS NEUMANN	Analytical Competence Molecular Epidemiology Lab/ACME, Oswaldo Cruz Foundation, Ceara (FIOCRUZ CE)	Cleber Furtado Aksenen; Cleber Furtado Aksenen e Suzana Porto Almeida; Fabio Miyajima; Fernando Braga Stehling; Francisco Eder de Moura Lopes; Jamille Maria Mendes Bezerra; Joaquim Cesar do Nascimento Sousa Junior; Joaquim César do Nascimento Sousa Junior; Pedro Miguel Carneiro Jeronimo; Suzana Porto Almeida & Lucas Delerino on behalf of COVID-19 FIOCRUZ Genomic Network; Suzana Porto Almeida e Lucas Delerino; Thais Ferreira de Oliveira; Thais de Oliveira Costa; Ticiane Cavalcante de Souza; Veridiana Pessoa Miyajima
EPI_ISL_3102478	HOSPITAL E MATERNIDADE GASTROCLINICA	Analytical Competence Molecular Epidemiology Lab/ACME, Oswaldo Cruz Foundation, Ceara (FIOCRUZ CE)	Cleber Furtado Aksenen; Fabio Miyajima; Fernando Braga Stehling; Francisco Eder de Moura Lopes; Jamille Maria Mendes Bezerra; Joaquim César do Nascimento Sousa Junior; Pedro Miguel Carneiro Jeronimo; Suzana Porto Almeida e Lucas Delerino; Thais Ferreira de Oliveira; Thais de Oliveira Costa; Ticiane Cavalcante de Souza; Veridiana Pessoa Miyajima
EPI_ISL_3912198	HOSPITAL E MATERNIDADE JOSE GRANJA RIBEIRO	Analytical Competence Molecular Epidemiology Lab/ACME, Oswaldo Cruz Foundation, Ceara (FIOCRUZ CE)	Cleber Furtado Aksenen; Fabio Miyajima; Fernando Braga Stehling; Francisco Eder de Moura Lopes; Jamille Maria Mendes Bezerra; Joaquim Cesar do Nascimento Sousa Junior; Pedro Miguel Carneiro Jeronimo; Suzana Porto Almeida & Lucas Delerino on behalf of COVID-19 FIOCRUZ Genomic Network; Thais Ferreira de Oliveira; Thais de Oliveira Costa; Ticiane Cavalcante de Souza; Veridiana Pessoa Miyajima
EPI_ISL_1795344, EPI_ISL_2345610	HOSPITAL E MATERNIDADE NOSSA SENHORA DA AJUDA	Instituto Butantan / ESALQ-Piracicaba	Antonio Jorge Martins; Bianca Cechetto Carlos. Mendelics: Bibiana Santos; Claudia Renata dos Santos Barros; David Schlesinger; David Schlesinger. Hemocentro Ribeirão Preto: Simone Kashima; Debora Botequio Moretti; Debora Botequio Moretti. Centro de Genômica Funcional da ESALQ: Luiz Lehmann Coutinho; Dimas Tadeu Covas; Elaine Cristina Marqueze; Elaine Vieira Santos; Elaine Vieira dos Santos; Elisangela Chicaroni Mattos; Erika Freitas; Evandra Strazza Rodrigues; Felipe Allan da Silva da Costa; Flavia Aburjaile; Guilherme Targino Valente; Heidge Fukumasu; Heidge Fukumasu. USP-Botucatu: Rejane Maria Tommasini Grotto; Instituto Butantan: Alexander Roberto Precioso; Jayme A. Souza-Neto; Jayme Augusto de Souza-Neto; Jessika Cristina Chagas Lesbon; José Salvatore Leister Patané; João Paulo Kitajima; Luiz Alcantara; Luiz Carlos Junior de Alcantara; Luiz Lehmann Coutinho; Maria Carolina Elias; Marta Giovanetti; Maurício Lacerda Nogueira; Patricia Akemi Assato; Rafael dos Santos Bezerra; Raquel de Lello Rocha Campos Cassano. NGS Soluções Genômicas: Pilar Drummond Sampaio Corrêa Mariani. FZEA-USP Pirassununga: Mirele Daiana Poleti; Raul Machado Neto; Rejane Maria Tommasini Grotto; Ricardo Augusto Brassaloti; Ricardo Haddad; Rodrigo Tocantins Calado.; Sandra Coccuzzo Sampaio; Sandra Coccuzzo Sampaio Vessoni; Simone Kashima; Svetoslav Nanev Slavov; Vagner Fonseca; Vincent Louis Viala
EPI_ISL_3102214, EPI_ISL_3102355, EPI_ISL_3102367, EPI_ISL_3102385, EPI_ISL_3102389, EPI_ISL_3102391, EPI_ISL_3102407, EPI_ISL_3102493, EPI_ISL_3102529, EPI_ISL_3102531, EPI_ISL_3102532, EPI_ISL_3102535, EPI_ISL_3912208, EPI_ISL_3912210			
see above	HOSPITAL ESTADUAL LEONARDO DA VINCI	Analytical Competence Molecular Epidemiology Lab/ACME, Oswaldo Cruz Foundation, Ceara (FIOCRUZ CE)	Cleber Furtado Aksenen; Fabio Miyajima; Fernando Braga Stehling; Francisco Eder de Moura Lopes; Jamille Maria Mendes Bezerra; Joaquim Cesar do Nascimento Sousa Junior; Joaquim César do Nascimento Sousa Junior; Pedro Miguel Carneiro Jeronimo; Suzana Porto Almeida & Lucas Delerino on behalf of COVID-19 FIOCRUZ Genomic Network; Suzana Porto Almeida e Lucas Delerino; Thais Ferreira de Oliveira; Thais de Oliveira Costa; Ticiane Cavalcante de Souza; Veridiana Pessoa Miyajima
EPI_ISL_2150636	HOSPITAL GENERAL UNIVERSITARIO DE GUADALAJARA	Instituto de Salud Carlos III	A. Monzón; ALEJANDRO; F. Casas; I. Jiménez; I.GONZALEZ PRAETORIUS; M. Sandonís; P. Zaballos; S. Cuesta; S. Iglesias-Caballero; S. Pozo; S. Varona; V. Camarero; Vázquez-Morón
EPI_ISL_2150639	HOSPITAL GENERAL VIRGEN DE LA LUZ	Instituto de Salud Carlos III	A. Monzón; F. Casas; I. Jiménez; I.RODRIGUEZ ESCUDERO; Iglesias-Caballero; M. Sandonís; MARIA JOSE; P. Zaballos; S. Camarero; S. Cuesta; S. Pozo; S. Varona; V. Vázquez-Morón
EPI_ISL_1966738	HOSPITAL GERAL DE VILA NOVA CACHOEIRINHA SAO PAULO	Instituto Butantan	Antonio Jorge Martins; Bianca Cechetto Carlos. Mendelics: Bibiana Santos; Claudia Renata dos Santos Barros; Cíntia Bittar; David Schlesinger. Hemocentro Ribeirão Preto: Simone Kashima; Debora Botequio Moretti; Elaine Cristina Marqueze; Elaine Vieira dos Santos; Elisangela Chicaroni Mattos; Erika Freitas; Evandra Strazza Rodrigues; Felipe Allan da Silva da Costa; Flavia Aburjaile; Fábio Sossai Possebon; Guilherme Campos; Guilherme Targino Valente; Heidge Fukumasu. USP-Botucatu: Rejane Maria Tommasini Grotto; Helena Lage Ferreira; Instituto Butantan: Dimas Tadeu Covas; Jardelina de Souza Todao Bernardino; Jayme A. Souza-Neto; Jessika Cristina Chagas Lesbon; Jorge A. Petroli Marchesi; José Salvatore Leister Patané; João Paulo Kitajima; João Pessoa Araújo Jr.; Leila Sabrina Ullmann; Loyze Paola Oliveira de Lima; Luiz Aurelio de Campos Crispin. Centro de Genômica Funcional da ESALQ: Luiz Lehmann Coutinho; Luiz Carlos Junior de Alcantara; Lívia Sacchetto; Maisa C. Pereira Parra; Maria Carolina Elias; Marta Giovanetti; Marília Moraes; Maurício Lacerda Nogueira. Prefeitura de Sao Paulo: Melissa Palmieri.; Patricia Akemi Assato; Paula Rahal; Paulo Inacio da Costa; Rafael dos Santos Bezerra; Raquel de Lello Rocha Campos Cassano. NGS Soluções Genômicas: Pilar Drummond Sampaio Corrêa Mariani. FZEA-USP Pirassununga: Mirele Daiana Poleti; Raul Machado Neto; Ricardo Augusto Brassaloti; Ricardo Haddad; Rodrigo Tocantins Calado. FAMERP-SJRP: Cecília Artico Banho; Sandra Coccuzzo Sampaio; Svetoslav Nanev Slavov; Vagner Fonseca; Vincent Louis Viala
EPI_ISL_1967078, EPI_ISL_1967080, EPI_ISL_1967081, EPI_ISL_1967084, EPI_ISL_1967086, EPI_ISL_1967088	HOSPITAL GERAL JESUS TEIXEIRA DA COSTA GUAIANASES SAO PAULO	Instituto Butantan / ESALQ-USP (Piracicaba)	Antonio Jorge Martins; Bianca Cechetto Carlos. Mendelics: Bibiana Santos; Claudia Renata dos Santos Barros; Cíntia Bittar; David Schlesinger. Hemocentro Ribeirão Preto: Simone Kashima; Debora Botequio Moretti; Elaine Cristina Marqueze; Elaine Vieira dos Santos; Elisangela Chicaroni Mattos; Erika Freitas; Evandra Strazza Rodrigues; Felipe Allan da Silva da Costa; Flavia Aburjaile; Fábio Sossai Possebon; Guilherme Campos; Guilherme Targino Valente; Heidge Fukumasu. USP-Botucatu: Rejane Maria Tommasini Grotto; Helena Lage Ferreira; Instituto Butantan: Dimas Tadeu Covas; Jardelina de Souza Todao Bernardino; Jayme A. Souza-Neto; Jessika Cristina Chagas Lesbon; Jorge A. Petroli Marchesi; José Salvatore Leister Patané; João Paulo Kitajima; João Pessoa Araújo Jr.; Leila Sabrina Ullmann; Loyze Paola Oliveira de Lima; Luiz Aurelio de Campos Crispin. Centro de Genômica Funcional da ESALQ: Luiz Lehmann Coutinho; Luiz Carlos Junior de Alcantara; Lívia Sacchetto; Maisa C. Pereira Parra; Maria Carolina Elias; Marta Giovanetti; Marília Moraes; Maurício Lacerda Nogueira. Prefeitura de Sao Paulo: Melissa Palmieri.; Patricia Akemi Assato; Paula Rahal; Paulo Inacio da Costa; Rafael dos Santos Bezerra; Raquel de Lello Rocha Campos Cassano. NGS Soluções Genômicas: Pilar Drummond Sampaio Corrêa Mariani. FZEA-USP Pirassununga: Mirele Daiana Poleti; Raul Machado Neto; Ricardo Augusto Brassaloti; Ricardo Haddad; Rodrigo Tocantins Calado. FAMERP-SJRP: Cecília Artico Banho; Sandra Coccuzzo Sampaio; Svetoslav Nanev Slavov; Vagner Fonseca; Vincent Louis Viala
EPI_ISL_2801306	HOSPITAL INFANTIL ALBERT SABIN	Analytical Competence Molecular Epidemiology Lab/ACME, Oswaldo Cruz Foundation, Ceara (FIOCRUZ CE)	Cleber Furtado Aksenen e Suzana Porto Almeida; Fabio Miyajima; Fernando Braga Stehling; Francisco Eder de Moura Lopes; Jamille Maria Mendes Bezerra; Joaquim César do Nascimento Sousa Junior; Pedro Miguel Carneiro Jeronimo; Thais Ferreira de Oliveira; Thais de Oliveira Costa; Ticiane Cavalcante de Souza; Veridiana Pessoa Miyajima
EPI_ISL_2020122	HOSPITAL MANACOR	HOSPITAL UNIVERSITARIO SON ESPASES	Antonio Oliver; Carla López-Causapé; Pablo Fraile-Ribot; SeqCovid
EPI_ISL_2346037	HOSPITAL MUN DR GUIDO GUIDA	Instituto Butantan	Antonio Jorge Martins; Claudia Renata dos Santos Barros; David Schlesinger; Debora Botequio Moretti; Dimas Tadeu Covas; Elaine Cristina Marqueze; Elaine Vieira Santos; Evandra Strazza Rodrigues; Heidge Fukumasu; Jayme Augusto de Souza-Neto; José Salvatore Leister Patané; Luiz Alcantara; Luiz Lehmann Coutinho; Maria Carolina Elias; Maurício Lacerda Nogueira; Rafael dos Santos Bezerra; Raul Machado Neto; Rejane Maria Tommasini Grotto; Ricardo Haddad; Sandra Coccuzzo Sampaio Vessoni; Simone Kashima; Svetoslav Nanev Slavov; Vincent Louis Viala
EPI_ISL_1966913, EPI_ISL_1966914	HOSPITAL MUNICIPAL DE IBIUNA IBIUNA SP	Instituto Butantan / ESALQ-USP (Piracicaba)	Antonio Jorge Martins; Bianca Cechetto Carlos. Mendelics: Bibiana Santos; Claudia Renata dos Santos Barros; Cíntia Bittar; David Schlesinger. Hemocentro Ribeirão Preto: Simone Kashima; Debora Botequio Moretti; Elaine Cristina Marqueze; Elaine Vieira dos Santos; Elisangela Chicaroni Mattos; Erika Freitas; Evandra Strazza Rodrigues; Felipe Allan da Silva da Costa; Flavia Aburjaile; Fábio Sossai Possebon; Guilherme Campos; Guilherme Targino Valente; Heidge Fukumasu. USP-Botucatu: Rejane Maria Tommasini Grotto; Helena Lage Ferreira; Instituto Butantan: Dimas Tadeu Covas; Jardelina de Souza Todao Bernardino; Jayme A. Souza-Neto; Jessika Cristina Chagas Lesbon; Jorge A. Petroli Marchesi; José Salvatore Leister Patané; João Paulo Kitajima; João Pessoa Araújo Jr.; Leila Sabrina Ullmann; Loyze Paola Oliveira de Lima; Luiz Aurelio de Campos Crispin. Centro de Genômica Funcional da ESALQ: Luiz Lehmann Coutinho; Luiz Carlos Junior de Alcantara; Lívia Sacchetto; Maisa C. Pereira Parra; Maria Carolina Elias; Marta Giovanetti; Marília Moraes; Maurício Lacerda Nogueira. Prefeitura de Sao Paulo: Melissa Palmieri.; Patricia Akemi Assato; Paula Rahal; Paulo Inacio da Costa; Rafael dos Santos Bezerra; Raquel de Lello Rocha Campos Cassano. NGS Soluções Genômicas: Pilar Drummond Sampaio Corrêa Mariani. FZEA-USP Pirassununga: Mirele Daiana Poleti; Raul Machado Neto; Ricardo Augusto Brassaloti; Ricardo Haddad; Rodrigo Tocantins Calado. FAMERP-SJRP: Cecília Artico Banho; Sandra Coccuzzo Sampaio; Svetoslav Nanev Slavov; Vagner Fonseca; Vincent Louis Viala
EPI_ISL_1966919, EPI_ISL_1966920, EPI_ISL_1966921	HOSPITAL MUNICIPAL DE ITABERA	Instituto Butantan / ESALQ-USP (Piracicaba)	Antonio Jorge Martins; Bianca Cechetto Carlos. Mendelics: Bibiana Santos; Claudia Renata dos Santos Barros; Cíntia Bittar; David Schlesinger. Hemocentro Ribeirão Preto: Simone Kashima; Debora Botequio Moretti; Elaine Cristina Marqueze; Elaine Vieira dos Santos; Elisangela Chicaroni Mattos; Erika Freitas; Evandra Strazza Rodrigues; Felipe Allan da Silva da Costa; Flavia Aburjaile; Fábio Sossai Possebon; Guilherme Campos; Guilherme Targino Valente; Heidge Fukumasu. USP-Botucatu: Rejane Maria Tommasini Grotto; Helena Lage Ferreira; Instituto Butantan: Dimas Tadeu Covas; Jardelina de Souza Todao Bernardino; Jayme A. Souza-Neto; Jessika Cristina Chagas Lesbon; Jorge A. Petroli Marchesi; José Salvatore Leister Patané; João Paulo Kitajima; João Pessoa Araújo Jr.; Leila Sabrina Ullmann; Loyze Paola Oliveira de Lima; Luiz Aurelio de Campos Crispin. Centro de Genômica Funcional da ESALQ: Luiz Lehmann Coutinho; Luiz Carlos Junior de Alcantara; Lívia Sacchetto; Maisa C. Pereira Parra; Maria Carolina Elias; Marta Giovanetti; Marília Moraes; Maurício Lacerda Nogueira. Prefeitura de Sao Paulo: Melissa Palmieri.; Patricia Akemi Assato; Paula Rahal; Paulo Inacio da Costa; Rafael dos Santos Bezerra; Raquel de Lello Rocha Campos Cassano. NGS Soluções Genômicas: Pilar Drummond Sampaio Corrêa Mariani. FZEA-USP Pirassununga: Mirele Daiana Poleti; Raul Machado Neto; Ricardo Augusto Brassaloti; Ricardo Haddad; Rodrigo Tocantins Calado. FAMERP-SJRP: Cecília Artico Banho; Sandra Coccuzzo Sampaio; Svetoslav Nanev Slavov; Vagner Fonseca; Vincent Louis Viala
EPI_ISL_2344767, EPI_ISL_2344788	HOSPITAL MUNICIPAL DE ITAPIRA	Instituto Butantan / ESALQ-Piracicaba	Antonio Jorge Martins; Claudia Renata dos Santos Barros; David Schlesinger; Debora Botequio Moretti; Dimas Tadeu Covas; Elaine Cristina Marqueze; Elaine Vieira Santos; Evandra Strazza Rodrigues; Heidge Fukumasu; Jayme Augusto de Souza-Neto; José Salvatore Leister Patané; Luiz Alcantara; Luiz Lehmann Coutinho; Maria Carolina Elias; Maurício Lacerda Nogueira; Rafael dos Santos Bezerra; Raul Machado Neto; Rejane Maria Tommasini Grotto; Ricardo Haddad; Sandra Coccuzzo Sampaio Vessoni; Simone Kashima; Svetoslav Nanev Slavov; Vincent Louis Viala
EPI_ISL_1966942, EPI_ISL_1966945, EPI_ISL_1966946, EPI_ISL_1966947, EPI_ISL_1966949	HOSPITAL MUNICIPAL DE ITAPIRA	Instituto Butantan / ESALQ-USP (Piracicaba)	Antonio Jorge Martins; Bianca Cechetto Carlos. Mendelics: Bibiana Santos; Claudia Renata dos Santos Barros; Cíntia Bittar; David Schlesinger. Hemocentro Ribeirão Preto: Simone Kashima; Debora Botequio Moretti; Elaine Cristina Marqueze; Elaine Vieira dos Santos; Elisangela Chicaroni Mattos; Erika Freitas; Evandra Strazza Rodrigues; Felipe Allan da Silva da Costa; Flavia Aburjaile; Fábio Sossai Possebon; Guilherme Campos; Guilherme Targino Valente; Heidge Fukumasu. USP-Botucatu: Rejane Maria Tommasini Grotto; Helena Lage Ferreira; Instituto Butantan: Dimas Tadeu Covas; Jardelina de Souza Todao Bernardino; Jayme A. Souza-Neto; Jessika Cristina Chagas Lesbon; Jorge A. Petroli Marchesi; José Salvatore Leister Patané; João Paulo Kitajima; João Pessoa Araújo Jr.; Leila Sabrina Ullmann; Loyze Paola Oliveira de Lima; Luiz Aurelio de Campos Crispin. Centro de Genômica Funcional da ESALQ: Luiz Lehmann Coutinho; Luiz Carlos Junior de Alcantara; Lívia Sacchetto; Maisa C. Pereira Parra; Maria Carolina Elias; Marta Giovanetti; Marília Moraes; Maurício Lacerda Nogueira. Prefeitura de Sao Paulo: Melissa Palmieri.; Patricia Akemi Assato; Paula Rahal; Paulo Inacio da Costa; Rafael dos Santos Bezerra; Raquel de Lello Rocha Campos Cassano. NGS Soluções Genômicas: Pilar Drummond Sampaio Corrêa Mariani. FZEA-USP Pirassununga: Mirele Daiana Poleti; Raul Machado Neto; Ricardo Augusto Brassaloti; Ricardo Haddad; Rodrigo Tocantins Calado. FAMERP-SJRP: Cecília Artico Banho; Sandra Coccuzzo Sampaio; Svetoslav Nanev Slavov; Vagner Fonseca; Vincent Louis Viala
EPI_ISL_2344571, EPI_ISL_2344573, EPI_ISL_2344574, EPI_ISL_2344578, EPI_ISL_2344579	HOSPITAL MUNICIPAL DE ITAPIRA	Instituto Butantan / FZEA-USP-Pirassununga	Antonio Jorge Martins; Claudia Renata dos Santos Barros; David Schlesinger; Debora Botequio Moretti; Dimas Tadeu Covas; Elaine Cristina Marqueze; Elaine Vieira Santos; Evandra Strazza Rodrigues; Heidge Fukumasu; Jayme Augusto de Souza-Neto; José Salvatore Leister Patané; Luiz Alcantara; Luiz Lehmann Coutinho; Maria Carolina Elias; Maurício Lacerda Nogueira; Rafael dos Santos Bezerra; Raul Machado Neto; Rejane Maria Tommasini Grotto; Ricardo Haddad; Sandra Coccuzzo Sampaio Vessoni; Simone Kashima; Svetoslav Nanev Slavov; Vincent Louis Viala
EPI_ISL_1795211, EPI_ISL_2345439	HOSPITAL MUNICIPAL DR MARIO GATTI CAMPINAS	Instituto Butantan / ESALQ-Piracicaba	Antonio Jorge Martins; Bianca Cechetto Carlos. Mendelics: Bibiana Santos; Claudia Renata dos Santos Barros; David Schlesinger; David Schlesinger. Hemocentro Ribeirão Preto: Simone Kashima; Debora Botequio Moretti; Debora Botequio Moretti. Centro de Genômica Funcional da ESALQ: Luiz Lehmann Coutinho; Dimas Tadeu Covas; Elaine Cristina Marqueze; Elaine Vieira Santos; Elaine Vieira dos Santos; Elisangela Chicaroni Mattos; Erika Freitas; Evandra Strazza Rodrigues; Felipe Allan da Silva da Costa; Flavia Aburjaile; Guilherme Targino Valente; Heidge Fukumasu; Heidge Fukumasu. USP-Botucatu: Rejane Maria Tommasini Grotto; Instituto Butantan: Alexander Roberto Precioso; Jayme A. Souza-Neto; Jayme Augusto de Souza-Neto; Jessika Cristina Chagas Lesbon; José Salvatore Leister Patané; João Paulo Kitajima; Luiz Alcantara; Luiz Carlos Junior de Alcantara; Luiz Lehmann Coutinho; Maria Carolina Elias; Marta Giovanetti; Maurício Lacerda Nogueira; Patricia Akemi Assato; Rafael dos Santos Bezerra; Raquel de Lello Rocha Campos Cassano. NGS Soluções Genômicas: Pilar Drummond Sampaio Corrêa Mariani. FZEA-USP Pirassununga: Mirele Daiana Poleti; Raul Machado Neto; Rejane Maria Tommasini Grotto; Ricardo Augusto Brassaloti; Ricardo Haddad; Rodrigo Tocantins Calado.; Sandra Coccuzzo Sampaio; Sandra Coccuzzo Sampaio Vessoni; Simone Kashima; Svetoslav Nanev Slavov; Vagner Fonseca; Vincent Louis Viala
EPI_ISL_2344800	HOSPITAL MUNICIPAL DR TABAJARA RAMOS	Instituto Butantan / ESALQ-Piracicaba	Antonio Jorge Martins; Claudia Renata dos Santos Barros; David Schlesinger; Debora Botequio Moretti; Dimas Tadeu Covas; Elaine Cristina Marqueze; Elaine Vieira Santos; Evandra Strazza Rodrigues; Heidge Fukumasu; Jayme Augusto de Souza-Neto; José Salvatore Leister Patané; Luiz Alcantara; Luiz Lehmann Coutinho; Maria Carolina Elias; Maurício Lacerda Nogueira; Rafael dos Santos Bezerra; Raul Machado Neto; Rejane Maria Tommasini Grotto; Ricardo Haddad; Sandra Coccuzzo Sampaio Vessoni; Simone Kashima; Svetoslav Nanev Slavov; Vincent Louis Viala
EPI_ISL_1966645	HOSPITAL MUNICIPAL DR WALDEMAR TEBALDI	Instituto Butantan / Mendelics	Antonio Jorge Martins; Bianca Cechetto Carlos. Mendelics: Bibiana Santos; Claudia Renata dos Santos Barros; Cíntia Bittar; David Schlesinger. Hemocentro Ribeirão Preto: Simone Kashima; Debora Botequio Moretti; Elaine Cristina Marqueze; Elaine Vieira dos Santos; Elisangela Chicaroni Mattos; Erika Freitas; Evandra Strazza Rodrigues; Felipe Allan da Silva da Costa; Flavia Aburjaile; Fábio Sossai Possebon; Guilherme Campos; Guilherme Targino Valente; Heidge Fukumasu. USP-Botucatu: Rejane Maria Tommasini Grotto; Helena Lage Ferreira; Instituto Butantan: Dimas Tadeu Covas; Jardelina de Souza Todao Bernardino; Jayme A. Souza-Neto; Jessika Cristina Chagas Lesbon; Jorge A. Petroli Marchesi; José Salvatore Leister Patané; João Paulo Kitajima; João Pessoa Araújo Jr.; Leila Sabrina Ullmann; Loyze Paola Oliveira de Lima; Luiz Aurelio de Campos Crispin. Centro de Genômica Funcional da ESALQ: Luiz Lehmann Coutinho; Luiz Carlos Junior de Alcantara; Lívia Sacchetto; Maisa C. Pereira Parra; Maria Carolina Elias; Marta Giovanetti; Marília Moraes; Maurício Lacerda Nogueira. Prefeitura de Sao Paulo: Melissa Palmieri.; Patricia Akemi Assato; Paula Rahal; Paulo Inacio da Costa; Rafael dos Santos Bezerra; Raquel de Lello Rocha Campos Cassano. NGS Soluções Genômicas: Pilar Drummond Sampaio Corrêa Mariani. FZEA-USP Pirassununga: Mirele Daiana Poleti; Raul Machado Neto; Ricardo Augusto Brassaloti; Ricardo Haddad; Rodrigo Tocantins Calado. FAMERP-SJRP: Cecília Artico Banho; Sandra Coccuzzo Sampaio; Svetoslav Nanev Slavov; Vagner Fonseca; Vincent Louis Viala
EPI_ISL_3102490	HOSPITAL MUNICIPAL GOVERNADOR ADAUTO BEZERRA	Analytical Competence Molecular Epidemiology Lab/ACME, Oswaldo Cruz Foundation, Ceara (FIOCRUZ CE)	Cleber Furtado Aksenen; Fabio Miyajima; Fernando Braga Stehling; Francisco Eder de Moura Lopes; Jamille Maria Mendes Bezerra; Joaquim César do Nascimento Sousa Junior; Pedro Miguel Carneiro Jeronimo; Suzana Porto Almeida e Lucas Delerino; Thais Ferreira de Oliveira; Thais de Oliveira Costa; Ticiane Cavalcante de Souza; Veridiana Pessoa Miyajima
EPI_ISL_3912212, EPI_ISL_3912213	HOSPITAL MUNICIPAL JOAO LEOPOLDO	Analytical Competence Molecular Epidemiology	Cleber Furtado Aksenen; Fabio Miyajima; Fernando Braga Stehling; Francisco Eder de Moura Lopes; Jamille Maria Mendes Bezerra; Joaquim Cesar do Nascimento Sousa Junior; Pedro Miguel Carneiro Jeronimo; Suzana Porto Almeida & Lucas Delerino on behalf of COVID-19 FIOCRUZ Genomic Network; Thais Ferreira de Oliveira; Thais de Oliveira Costa; Ticiane Cavalcante de Souza; Veridiana Pessoa Miyajima

	PINHEIRO LANDIM	Lab/ACME, Oswaldo Cruz Foundation, Ceara (FIOCRUZ CE)	
EPI_ISL_1967025, EPI_ISL_1967026	HOSPITAL MUNICIPAL MARIA ROSA CARDOSO	Instituto Butantan / ESALQ-USP (Piracicaba)	Antonio Jorge Martins; Bianca Cechetto Carlos. Mendelics: Bibiana Santos; Claudia Renata dos Santos Barros; Cíntia Bittar; David Schlesinger. Hemocentro Ribeirão Preto: Simone Kashima; Debora Botequio Moretti; Elaine Cristina Marqueze; Elaine Vieira dos Santos; Elisangela Chicaroni Mattos; Erika Freitas; Evandra Strazza Rodrigues; Felipe Allan da Silva da Costa; Flavia Aburjaile; Fábio Sossai Possebon; Guilherme Campos; Guilherme Targino Valente; Heidge Fukumasu. USP-Botucatu: Rejane Maria Tommasini Grotto; Helena Lage Ferreira; Instituto Butantan: Dimas Tadeu Covas; Jardelina de Souza Todao Bernardino; Jayme A. Souza-Neto; Jessika Cristina Chagas Lesbon; Jorge A. Petroli Marchesi; José Salvatore Leister Patané; João Paulo Kitajima; João Pessoa Araújo Jr.; Leila Sabrina Ullmann; Loyze Paola Oliveira de Lima; Luiz Aurelio de Campos Crispin. Centro de Genômica Funcional da ESALQ: Luiz Lehmann Coutinho; Luiz Carlos Junior de Alcantara; Lívia Sacchetto; Maisa C. Pereira Parra; Maria Carolina Elias; Marta Giovanetti; Marília Moraes; Maurício Lacerda Nogueira. Prefeitura de Sao Paulo: Melissa Palmieri.; Patricia Akemi Assato; Paula Rahal; Paulo Inacio da Costa; Rafael dos Santos Bezerra; Raquel de Lello Rocha Campos Cassano. NGS Soluções Genômicas: Pilar Drummond Sampaio Corrêa Mariani. FZEA-USP Pirassununga: Mirele Daiana Poleti; Raul Machado Neto; Ricardo Augusto Brassaloti; Ricardo Haddad; Rodrigo Tocantins Calado. FAMERP-SJRP: Cecília Artico Banho; Sandra Coccuzzo Sampaio; Svetoslav Nanev Slavov; Vagner Fonseca; Vincent Louis Viala
EPI_ISL_1795337, EPI_ISL_1795338, EPI_ISL_1795341, EPI_ISL_2345603, EPI_ISL_2345604, EPI_ISL_2345607	HOSPITAL MUNICIPAL REYNALDO GUERRA CAJATI	Instituto Butantan / ESALQ-Piracicaba	Antonio Jorge Martins; Bianca Cechetto Carlos. Mendelics: Bibiana Santos; Claudia Renata dos Santos Barros; David Schlesinger; David Schlesinger. Hemocentro Ribeirão Preto: Simone Kashima; Debora Botequio Moretti; Debora Botequio Moretti. Centro de Genômica Funcional da ESALQ: Luiz Lehmann Coutinho; Dimas Tadeu Covas; Elaine Cristina Marqueze; Elaine Vieira Santos; Elaine Vieira dos Santos; Elisangela Chicaroni Mattos; Erika Freitas; Evandra Strazza Rodrigues; Felipe Allan da Silva da Costa; Flavia Aburjaile; Guilherme Targino Valente; Heidge Fukumasu; Heidge Fukumasu. USP-Botucatu: Rejane Maria Tommasini Grotto; Instituto Butantan: Alexander Roberto Precioso; Jayme A. Souza-Neto; Jayme Augusto de Souza-Neto; Jessika Cristina Chagas Lesbon; José Salvatore Leister Patané; João Paulo Kitajima; Luiz Alcantara; Luiz Carlos Junior de Alcantara; Luiz Lehmann Coutinho; Maria Carolina Elias; Marta Giovanetti; Maurício Lacerda Nogueira; Patricia Akemi Assato; Rafael dos Santos Bezerra; Raquel de Lello Rocha Campos Cassano. NGS Soluções Genômicas: Pilar Drummond Sampaio Corrêa Mariani. FZEA-USP Pirassununga: Mirele Daiana Poleti; Raul Machado Neto; Rejane Maria Tommasini Grotto; Ricardo Augusto Brassaloti; Ricardo Haddad; Rodrigo Tocantins Calado.; Sandra Coccuzzo Sampaio; Sandra Coccuzzo Sampaio Vessoni; Simone Kashima; Svetoslav Nanev Slavov; Vagner Fonseca; Vincent Louis Viala
EPI_ISL_1966846	HOSPITAL MUNICIPAL REYNALDO GUERRA CAJATI	Instituto Butantan / ESALQ-USP (Piracicaba)	Antonio Jorge Martins; Bianca Cechetto Carlos. Mendelics: Bibiana Santos; Claudia Renata dos Santos Barros; Cíntia Bittar; David Schlesinger. Hemocentro Ribeirão Preto: Simone Kashima; Debora Botequio Moretti; Elaine Cristina Marqueze; Elaine Vieira dos Santos; Elisangela Chicaroni Mattos; Erika Freitas; Evandra Strazza Rodrigues; Felipe Allan da Silva da Costa; Flavia Aburjaile; Fábio Sossai Possebon; Guilherme Campos; Guilherme Targino Valente; Heidge Fukumasu. USP-Botucatu: Rejane Maria Tommasini Grotto; Helena Lage Ferreira; Instituto Butantan: Dimas Tadeu Covas; Jardelina de Souza Todao Bernardino; Jayme A. Souza-Neto; Jessika Cristina Chagas Lesbon; Jorge A. Petroli Marchesi; José Salvatore Leister Patané; João Paulo Kitajima; João Pessoa Araújo Jr.; Leila Sabrina Ullmann; Loyze Paola Oliveira de Lima; Luiz Aurelio de Campos Crispin. Centro de Genômica Funcional da ESALQ: Luiz Lehmann Coutinho; Luiz Carlos Junior de Alcantara; Lívia Sacchetto; Maisa C. Pereira Parra; Maria Carolina Elias; Marta Giovanetti; Marília Moraes; Maurício Lacerda Nogueira. Prefeitura de Sao Paulo: Melissa Palmieri.; Patricia Akemi Assato; Paula Rahal; Paulo Inacio da Costa; Rafael dos Santos Bezerra; Raquel de Lello Rocha Campos Cassano. NGS Soluções Genômicas: Pilar Drummond Sampaio Corrêa Mariani. FZEA-USP Pirassununga: Mirele Daiana Poleti; Raul Machado Neto; Ricardo Augusto Brassaloti; Ricardo Haddad; Rodrigo Tocantins Calado. FAMERP-SJRP: Cecília Artico Banho; Sandra Coccuzzo Sampaio; Svetoslav Nanev Slavov; Vagner Fonseca; Vincent Louis Viala
EPI_ISL_3102323, EPI_ISL_3912221	HOSPITAL OTOCLINICA	Analytical Competence Molecular Epidemiology Lab/ACME, Oswaldo Cruz Foundation, Ceara (FIOCRUZ CE)	Cleber Furtado Aksenen; Fabio Miyajima; Fernando Braga Stehling; Francisco Eder de Moura Lopes; Jamille Maria Mendes Bezerra; Joaquim Cesar do Nascimento Sousa Junior; Joaquim César do Nascimento Sousa Junior; Pedro Miguel Carneiro Jeronimo; Suzana Porto Almeida & Lucas Delerino on behalf of COVID-19 FIOCRUZ Genomic Network; Suzana Porto Almeida e Lucas Delerino; Thais Ferreira de Oliveira; Thais de Oliveira Costa; Ticiane Cavalcante de Souza; Veridiana Pessoa Miyajima
EPI_ISL_3912222	HOSPITAL REGIONAL DE IGUATU	Analytical Competence Molecular Epidemiology Lab/ACME, Oswaldo Cruz Foundation, Ceara (FIOCRUZ CE)	Cleber Furtado Aksenen; Fabio Miyajima; Fernando Braga Stehling; Francisco Eder de Moura Lopes; Jamille Maria Mendes Bezerra; Joaquim Cesar do Nascimento Sousa Junior; Pedro Miguel Carneiro Jeronimo; Suzana Porto Almeida & Lucas Delerino on behalf of COVID-19 FIOCRUZ Genomic Network; Thais Ferreira de Oliveira; Thais de Oliveira Costa; Ticiane Cavalcante de Souza; Veridiana Pessoa Miyajima
EPI_ISL_1795221, EPI_ISL_2345451	HOSPITAL REGIONAL DE ITAPETININGA	Instituto Butantan / ESALQ-Piracicaba	Antonio Jorge Martins; Bianca Cechetto Carlos. Mendelics: Bibiana Santos; Claudia Renata dos Santos Barros; David Schlesinger; David Schlesinger. Hemocentro Ribeirão Preto: Simone Kashima; Debora Botequio Moretti; Debora Botequio Moretti. Centro de Genômica Funcional da ESALQ: Luiz Lehmann Coutinho; Dimas Tadeu Covas; Elaine Cristina Marqueze; Elaine Vieira Santos; Elaine Vieira dos Santos; Elisangela Chicaroni Mattos; Erika Freitas; Evandra Strazza Rodrigues; Felipe Allan da Silva da Costa; Flavia Aburjaile; Guilherme Targino Valente; Heidge Fukumasu; Heidge Fukumasu. USP-Botucatu: Rejane Maria Tommasini Grotto; Instituto Butantan: Alexander Roberto Precioso; Jayme A. Souza-Neto; Jayme Augusto de Souza-Neto; Jessika Cristina Chagas Lesbon; José Salvatore Leister Patané; João Paulo Kitajima; Luiz Alcantara; Luiz Carlos Junior de Alcantara; Luiz Lehmann Coutinho; Maria Carolina Elias; Marta Giovanetti; Maurício Lacerda Nogueira; Patricia Akemi Assato; Rafael dos Santos Bezerra; Raquel de Lello Rocha Campos Cassano. NGS Soluções Genômicas: Pilar Drummond Sampaio Corrêa Mariani. FZEA-USP Pirassununga: Mirele Daiana Poleti; Raul Machado Neto; Rejane Maria Tommasini Grotto; Ricardo Augusto Brassaloti; Ricardo Haddad; Rodrigo Tocantins Calado.; Sandra Coccuzzo Sampaio; Sandra Coccuzzo Sampaio Vessoni; Simone Kashima; Svetoslav Nanev Slavov; Vagner Fonseca; Vincent Louis Viala
EPI_ISL_1966923, EPI_ISL_1966924, EPI_ISL_1966925	HOSPITAL REGIONAL DE ITAPETININGA	Instituto Butantan / ESALQ-USP (Piracicaba)	Antonio Jorge Martins; Bianca Cechetto Carlos. Mendelics: Bibiana Santos; Claudia Renata dos Santos Barros; Cíntia Bittar; David Schlesinger. Hemocentro Ribeirão Preto: Simone Kashima; Debora Botequio Moretti; Elaine Cristina Marqueze; Elaine Vieira dos Santos; Elisangela Chicaroni Mattos; Erika Freitas; Evandra Strazza Rodrigues; Felipe Allan da Silva da Costa; Flavia Aburjaile; Fábio Sossai Possebon; Guilherme Campos; Guilherme Targino Valente; Heidge Fukumasu. USP-Botucatu: Rejane Maria Tommasini Grotto; Helena Lage Ferreira; Instituto Butantan: Dimas Tadeu Covas; Jardelina de Souza Todao Bernardino; Jayme A. Souza-Neto; Jessika Cristina Chagas Lesbon; Jorge A. Petroli Marchesi; José Salvatore Leister Patané; João Paulo Kitajima; João Pessoa Araújo Jr.; Leila Sabrina Ullmann; Loyze Paola Oliveira de Lima; Luiz Aurelio de Campos Crispin. Centro de Genômica Funcional da ESALQ: Luiz Lehmann Coutinho; Luiz Carlos Junior de Alcantara; Lívia Sacchetto; Maisa C. Pereira Parra; Maria Carolina Elias; Marta Giovanetti; Marília Moraes; Maurício Lacerda Nogueira. Prefeitura de Sao Paulo: Melissa Palmieri.; Patricia Akemi Assato; Paula Rahal; Paulo Inacio da Costa; Rafael dos Santos Bezerra; Raquel de Lello Rocha Campos Cassano. NGS Soluções Genômicas: Pilar Drummond Sampaio Corrêa Mariani. FZEA-USP Pirassununga: Mirele Daiana Poleti; Raul Machado Neto; Ricardo Augusto Brassaloti; Ricardo Haddad; Rodrigo Tocantins Calado. FAMERP-SJRP: Cecília Artico Banho; Sandra Coccuzzo Sampaio; Svetoslav Nanev Slavov; Vagner Fonseca; Vincent Louis Viala
EPI_ISL_2801320	HOSPITAL REGIONAL DO CARIRI	Analytical Competence Molecular Epidemiology Lab/ACME, Oswaldo Cruz Foundation, Ceara (FIOCRUZ CE)	Cleber Furtado Aksenen e Suzana Porto Almeida; Fabio Miyajima; Fernando Braga Stehling; Francisco Eder de Moura Lopes; Jamille Maria Mendes Bezerra; Joaquim César do Nascimento Sousa Junior; Pedro Miguel Carneiro Jeronimo; Thais Ferreira de Oliveira; Thais de Oliveira Costa; Ticiane Cavalcante de Souza; Veridiana Pessoa Miyajima
EPI_ISL_3102326, EPI_ISL_3912224	HOSPITAL REGIONAL DO SERTAO CENTRAL	Analytical Competence Molecular Epidemiology Lab/ACME, Oswaldo Cruz Foundation, Ceara (FIOCRUZ CE)	Cleber Furtado Aksenen; Fabio Miyajima; Fernando Braga Stehling; Francisco Eder de Moura Lopes; Jamille Maria Mendes Bezerra; Joaquim Cesar do Nascimento Sousa Junior; Joaquim César do Nascimento Sousa Junior; Pedro Miguel Carneiro Jeronimo; Suzana Porto Almeida & Lucas Delerino on behalf of COVID-19 FIOCRUZ Genomic Network; Suzana Porto Almeida e Lucas Delerino; Thais Ferreira de Oliveira; Thais de Oliveira Costa; Ticiane Cavalcante de Souza; Veridiana Pessoa Miyajima
EPI_ISL_2272969	HOSPITAL SAN VICENTE DE PAUL	Inciensa, Instituto Costarricense de Investigación y Enseñanza en Nutrición y Salud	Adriana Godínez; Claudio Soto-Garita; Estela Cordero; Francisco Duarte; Hebleen Porras; Joselyn Prado & Silvia Sáenz-García; José Luis Vargas; Mariela Gutiérrez; Melany Calderón
EPI_ISL_1795372, EPI_ISL_2345946	HOSPITAL SANTA THEREZINHA BROTAS	Instituto Butantan / ESALQ-Piracicaba	Antonio Jorge Martins; Bianca Cechetto Carlos. Mendelics: Bibiana Santos; Claudia Renata dos Santos Barros; David Schlesinger; David Schlesinger. Hemocentro Ribeirão Preto: Simone Kashima; Debora Botequio Moretti; Debora Botequio Moretti. Centro de Genômica Funcional da ESALQ: Luiz Lehmann Coutinho; Dimas Tadeu Covas; Elaine Cristina Marqueze; Elaine Vieira Santos; Elaine Vieira dos Santos; Elisangela Chicaroni Mattos; Erika Freitas; Evandra Strazza Rodrigues; Felipe Allan da Silva da Costa; Flavia Aburjaile; Guilherme Targino Valente; Heidge Fukumasu; Heidge Fukumasu. USP-Botucatu: Rejane Maria Tommasini Grotto; Instituto Butantan: Alexander Roberto Precioso; Jayme A. Souza-Neto; Jayme Augusto de Souza-Neto; Jessika Cristina Chagas Lesbon; José Salvatore Leister Patané; João Paulo Kitajima; Luiz Alcantara; Luiz Carlos Junior de Alcantara; Luiz Lehmann Coutinho; Maria Carolina Elias; Marta Giovanetti; Maurício Lacerda Nogueira; Patricia Akemi Assato; Rafael dos Santos Bezerra; Raquel de Lello Rocha Campos Cassano. NGS Soluções Genômicas: Pilar Drummond Sampaio Corrêa Mariani. FZEA-USP Pirassununga: Mirele Daiana Poleti; Raul Machado Neto; Rejane Maria Tommasini Grotto; Ricardo Augusto Brassaloti; Ricardo Haddad; Rodrigo Tocantins Calado.; Sandra Coccuzzo Sampaio; Sandra Coccuzzo Sampaio Vessoni; Simone Kashima; Svetoslav Nanev Slavov; Vagner Fonseca; Vincent Louis Viala
EPI_ISL_1966954	HOSPITAL SANTO ANTONIO DE JUQUIA JUQUIA	Instituto Butantan / ESALQ-USP (Piracicaba)	Antonio Jorge Martins; Bianca Cechetto Carlos. Mendelics: Bibiana Santos; Claudia Renata dos Santos Barros; Cíntia Bittar; David Schlesinger. Hemocentro Ribeirão Preto: Simone Kashima; Debora Botequio Moretti; Elaine Cristina Marqueze; Elaine Vieira dos Santos; Elisangela Chicaroni Mattos; Erika Freitas; Evandra Strazza Rodrigues; Felipe Allan da Silva da Costa; Flavia Aburjaile; Fábio Sossai Possebon; Guilherme Campos; Guilherme Targino Valente; Heidge Fukumasu. USP-Botucatu: Rejane Maria Tommasini Grotto; Helena Lage Ferreira; Instituto Butantan: Dimas Tadeu Covas; Jardelina de Souza Todao Bernardino; Jayme A. Souza-Neto; Jessika Cristina Chagas Lesbon; Jorge A. Petroli Marchesi; José Salvatore Leister Patané; João Paulo Kitajima; João Pessoa Araújo Jr.; Leila Sabrina Ullmann; Loyze Paola Oliveira de Lima; Luiz Aurelio de Campos Crispin. Centro de Genômica Funcional da ESALQ: Luiz Lehmann Coutinho; Luiz Carlos Junior de Alcantara; Lívia Sacchetto; Maisa C. Pereira Parra; Maria Carolina Elias; Marta Giovanetti; Marília Moraes; Maurício Lacerda Nogueira. Prefeitura de Sao Paulo: Melissa Palmieri.; Patricia Akemi Assato; Paula Rahal; Paulo Inacio da Costa; Rafael dos Santos Bezerra; Raquel de Lello Rocha Campos Cassano. NGS Soluções Genômicas: Pilar Drummond Sampaio Corrêa Mariani. FZEA-USP Pirassununga: Mirele Daiana Poleti; Raul Machado Neto; Ricardo Augusto Brassaloti; Ricardo Haddad; Rodrigo Tocantins Calado. FAMERP-SJRP: Cecília Artico Banho; Sandra Coccuzzo Sampaio; Svetoslav Nanev Slavov; Vagner Fonseca; Vincent Louis Viala
EPI_ISL_3102239, EPI_ISL_3102240, EPI_ISL_3102241, EPI_ISL_3102242, EPI_ISL_3102244, EPI_ISL_3102245, EPI_ISL_3102246, EPI_ISL_3102247, EPI_ISL_3102249, EPI_ISL_3102250, EPI_ISL_3102251, EPI_ISL_3102410, EPI_ISL_3102411, EPI_ISL_3102414, EPI_ISL_3102421, EPI_ISL_3102423, EPI_ISL_3102437, EPI_ISL_3102438, EPI_ISL_3102439, EPI_ISL_3102441, EPI_ISL_3102446, EPI_ISL_3102456, EPI_ISL_3102459			
see above	HOSPITAL SAO JOSE DE DOENCAS INFECCIOSAS	Analytical Competence Molecular Epidemiology Lab/ACME, Oswaldo Cruz Foundation, Ceara (FIOCRUZ CE)	Cleber Furtado Aksenen; Fabio Miyajima; Fernando Braga Stehling; Francisco Eder de Moura Lopes; Jamille Maria Mendes Bezerra; Joaquim César do Nascimento Sousa Junior; Pedro Miguel Carneiro Jeronimo; Suzana Porto Almeida e Lucas Delerino; Thais Ferreira de Oliveira; Thais de Oliveira Costa; Ticiane Cavalcante de Souza; Veridiana Pessoa Miyajima
EPI_ISL_2209319	HOSPITAL SAO JOSE ITIRAPINA	Instituto Butantan	Antonio Jorge Martins; Claudia Renata dos Santos Barros; David Schlesinger; Debora Botequio Moretti; Dimas Tadeu Covas; Elaine Cristina Marqueze; Elaine Vieira Santos; Evandra Strazza Rodrigues; Heidge Fukumasu; Jayme Augusto de Souza-Neto; José Salvatore Leister Patané; Luiz Alcantara; Luiz Lehmann Coutinho; Maria Carolina Elias; Maurício Lacerda Nogueira; Rafael dos Santos Bezerra; Raul Machado Neto; Rejane Maria Tommasini Grotto; Ricardo Haddad; Sandra Coccuzzo Sampaio Vessoni; Simone Kashima; Svetoslav Nanev Slavov; Vincent Louis Viala.
EPI_ISL_2209975, EPI_ISL_2210091	HOSPITAL SAO JOSE ITIRAPINA	Instituto Butantan / FZEA-USP-Pirassununga	Antonio Jorge Martins; Claudia Renata dos Santos Barros; David Schlesinger; Debora Botequio Moretti; Dimas Tadeu Covas; Elaine Cristina Marqueze; Elaine Vieira Santos; Evandra Strazza Rodrigues; Heidge Fukumasu; Jayme Augusto de Souza-Neto; José Salvatore Leister Patané; Luiz Alcantara; Luiz Lehmann Coutinho; Maria Carolina Elias; Maurício Lacerda Nogueira; Rafael dos Santos Bezerra; Raul Machado Neto; Rejane Maria Tommasini Grotto; Ricardo Haddad; Sandra Coccuzzo Sampaio Vessoni; Simone Kashima; Svetoslav Nanev Slavov; Vincent Louis Viala
EPI_ISL_3102489	HOSPITAL SAO LUCAS	Analytical Competence Molecular Epidemiology Lab/ACME, Oswaldo Cruz Foundation, Ceara (FIOCRUZ CE)	Cleber Furtado Aksenen; Fabio Miyajima; Fernando Braga Stehling; Francisco Eder de Moura Lopes; Jamille Maria Mendes Bezerra; Joaquim César do Nascimento Sousa Junior; Pedro Miguel Carneiro Jeronimo; Suzana Porto Almeida e Lucas Delerino; Thais Ferreira de Oliveira; Thais de Oliveira Costa; Ticiane Cavalcante de Souza; Veridiana Pessoa Miyajima
EPI_ISL_3102486, EPI_ISL_3102487	HOSPITAL SAO MATEUS	Analytical Competence Molecular Epidemiology Lab/ACME, Oswaldo Cruz Foundation, Ceara (FIOCRUZ CE)	Cleber Furtado Aksenen; Fabio Miyajima; Fernando Braga Stehling; Francisco Eder de Moura Lopes; Jamille Maria Mendes Bezerra; Joaquim César do Nascimento Sousa Junior; Pedro Miguel Carneiro Jeronimo; Suzana Porto Almeida e Lucas Delerino; Thais Ferreira de Oliveira; Thais de Oliveira Costa; Ticiane Cavalcante de Souza; Veridiana Pessoa Miyajima
EPI_ISL_1967065, EPI_ISL_1967066	HOSPITAL SAO MIGUEL ARCANJO	Instituto Butantan / ESALQ-USP (Piracicaba)	Antonio Jorge Martins; Bianca Cechetto Carlos. Mendelics: Bibiana Santos; Claudia Renata dos Santos Barros; Cíntia Bittar; David Schlesinger. Hemocentro Ribeirão Preto: Simone Kashima; Debora Botequio Moretti; Elaine Cristina Marqueze; Elaine Vieira dos Santos; Elisangela Chicaroni Mattos; Erika Freitas; Evandra Strazza Rodrigues; Felipe Allan da Silva da Costa; Flavia Aburjaile; Fábio Sossai Possebon; Guilherme Campos; Guilherme Targino Valente; Heidge Fukumasu. USP-Botucatu: Rejane Maria Tommasini Grotto; Helena Lage Ferreira; Instituto Butantan: Dimas Tadeu Covas; Jardelina de Souza Todao Bernardino; Jayme A. Souza-Neto; Jessika Cristina Chagas Lesbon; Jorge A. Petroli Marchesi; José Salvatore Leister Patané; João Paulo Kitajima; João Pessoa Araújo Jr.; Leila Sabrina Ullmann; Loyze Paola Oliveira de Lima; Luiz Aurelio de Campos Crispin. Centro de Genômica Funcional da ESALQ: Luiz Lehmann Coutinho; Luiz Carlos Junior de Alcantara; Lívia Sacchetto; Maisa C. Pereira Parra; Maria Carolina Elias; Marta Giovanetti; Marília Moraes; Maurício Lacerda Nogueira. Prefeitura de Sao Paulo: Melissa Palmieri.; Patricia Akemi Assato; Paula Rahal; Paulo Inacio da Costa; Rafael dos Santos Bezerra; Raquel de Lello Rocha Campos Cassano. NGS Soluções Genômicas: Pilar Drummond Sampaio Corrêa Mariani. FZEA-USP Pirassununga: Mirele Daiana Poleti; Raul Machado Neto; Ricardo Augusto Brassaloti; Ricardo Haddad; Rodrigo Tocantins Calado. FAMERP-SJRP: Cecília Artico Banho; Sandra Coccuzzo Sampaio; Svetoslav Nanev Slavov; Vagner Fonseca; Vincent Louis Viala
EPI_ISL_2801312, EPI_ISL_2801324	HOSPITAL SÃO JOSE DE DOENÇAS INFECCIOSAS	Analytical Competence Molecular Epidemiology Lab/ACME, Oswaldo Cruz Foundation, Ceara (FIOCRUZ CE)	Cleber Furtado Aksenen e Suzana Porto Almeida; Fabio Miyajima; Fernando Braga Stehling; Francisco Eder de Moura Lopes; Jamille Maria Mendes Bezerra; Joaquim César do Nascimento Sousa Junior; Pedro Miguel Carneiro Jeronimo; Thais Ferreira de Oliveira; Thais de Oliveira Costa; Ticiane Cavalcante de Souza; Veridiana Pessoa Miyajima
EPI_ISL_2801317	HOSPITAL SÃO MATEUS	Analytical Competence Molecular Epidemiology Lab/ACME, Oswaldo Cruz Foundation, Ceara (FIOCRUZ CE)	Cleber Furtado Aksenen e Suzana Porto Almeida; Fabio Miyajima; Fernando Braga Stehling; Francisco Eder de Moura Lopes; Jamille Maria Mendes Bezerra; Joaquim César do Nascimento Sousa Junior; Pedro Miguel Carneiro Jeronimo; Thais Ferreira de Oliveira; Thais de Oliveira Costa; Ticiane Cavalcante de Souza; Veridiana Pessoa Miyajima
EPI_ISL_2150638	HOSPITAL UNIV.	Instituto de Salud	A. Monzón; ANA MARIA; F. Casas; I. Jiménez; I.MILAGRO BEAMONTE; M. Sandonís; P. Zaballos; S. Cuesta; S. Iglesias-Caballero; S. Pozo; S. Varona; V. Camarero; Vázquez-Morón

	MIGUEL SERVET	Carlos III	
EPI_ISL_1908182	HOSPITAL UNIVERS MARQUES DE VALDECILLA	Instituto de Salud Carlos III	A. Monzón; CARLOS ANTONIO; F. Casas; I. Jiménez; I.SALAS VENERO; M. Sandonís; P. Zaballos; S. Cuesta; S. Iglesias-Caballero; S. Pozo; S. Varona; V. Camarero; Vázquez-Morón
EPI_ISL_3502764, EPI_ISL_3532482	HOSPITAL UNIVERSITARIO 12 DE OCTUBRE	HOSPITAL UNIVERSITARIO 12 DE OCTUBRE	Carmen Martín-Higuera; Esther Viedma; Irene Múñoz-Gallego; M.ª Dolores Folgueira; Mar Aguilera; Noelia Moral; Rafael Delgado; Sagrario Zurita
EPI_ISL_2020104, EPI_ISL_2020154, EPI_ISL_2020179, EPI_ISL_2100319	HOSPITAL UNIVERSITARIO SON ESPASES	HOSPITAL UNIVERSITARIO SON ESPASES	Antonio Oliver; Carla López-Causapé; Pablo Fraile-Ribot; SeqCovid
EPI_ISL_1510754, EPI_ISL_2150635, EPI_ISL_2150641	HOSPITAL UNIVERSITARIO VIRGEN DE LA ARRIXACA	Instituto de Salud Carlos III	A. Monzón; F. Casas; I. Jiménez; I. MORENO PARRADO; I.MORENO PARRADO; Iglesias-Caballero; LAURA; M. Camarero; M. Sandonís; P. Zaballos; S. Camarero; S. Cuesta; S. Iglesias-Caballero; S. Pozo; S. Varona; Sandonís; V. Vázquez-Morón
EPI_ISL_2801325	HOSPITAL UNIVERSITÁRIO WALTER CANTIDIO	Analytical Competence Molecular Epidemiology Lab/ACME, Oswaldo Cruz Foundation, Ceara (FIOCRUZ CE)	Cleber Furtado Aksenen e Suzana Porto Almeida; Fabio Miyajima; Fernando Braga Stehling; Francisco Eder de Moura Lopes; Jamille Maria Mendes Bezerra; Joaquim César do Nascimento Sousa Junior; Pedro Miguel Carneiro Jeronimo; Thais Ferreira de Oliveira; Thais de Oliveira Costa; Ticiane Cavalcante de Souza; Veridiana Pessoa Miyajima
EPI_ISL_3102262	HPP LUIZ ROBERTO PESSOA AIRES	Analytical Competence Molecular Epidemiology Lab/ACME, Oswaldo Cruz Foundation, Ceara (FIOCRUZ CE)	Cleber Furtado Aksenen; Fabio Miyajima; Fernando Braga Stehling; Francisco Eder de Moura Lopes; Jamille Maria Mendes Bezerra; Joaquim César do Nascimento Sousa Junior; Pedro Miguel Carneiro Jeronimo; Suzana Porto Almeida e Lucas Delerino; Thais Ferreira de Oliveira; Thais de Oliveira Costa; Ticiane Cavalcante de Souza; Veridiana Pessoa Miyajima

EPI_ISL_1971337, EPI_ISL_2081100, EPI_ISL_2081106, EPI_ISL_2081109, EPI_ISL_2081119, EPI_ISL_2483366, EPI_ISL_2483379, EPI_ISL_2483424, EPI_ISL_2483426, EPI_ISL_2483427, EPI_ISL_2483428, EPI_ISL_2483430, EPI_ISL_2483432, EPI_ISL_2483438, EPI_ISL_2483443, EPI_ISL_2738475

see above	Hackensack Medical Center	New York Genome Center	Andre Corvelo; Barry Kreiswirth; David Perlin; Dayna M. Oschwald; Jose Mediavilla; Kaelea Composto; Kar Chow; Liang Chen; Marcus Cunningham; Michael Zody; Samantha Fennessey; Tom Maniatis

EPI_ISL_1194849, EPI_ISL_1194852, EPI_ISL_1337165, EPI_ISL_1337333, EPI_ISL_1337346, EPI_ISL_1337348, EPI_ISL_1480020, EPI_ISL_1480124, EPI_ISL_1480193, EPI_ISL_1480204, EPI_ISL_1480211, EPI_ISL_1480288, EPI_ISL_1480420, EPI_ISL_1480437, EPI_ISL_1553319, EPI_ISL_1553399, EPI_ISL_1553453, EPI_ISL_1553537, EPI_ISL_1553576, EPI_ISL_1553622, EPI_ISL_1553640, EPI_ISL_1553671, EPI_ISL_1553907, EPI_ISL_1553916, EPI_ISL_1553947, EPI_ISL_1554010, EPI_ISL_1554059, EPI_ISL_1554095, EPI_ISL_1554100, EPI_ISL_1554122, EPI_ISL_1554156, EPI_ISL_1554167, EPI_ISL_1554231, EPI_ISL_1554323, EPI_ISL_1554341, EPI_ISL_1554396, EPI_ISL_1554596, EPI_ISL_1554602, EPI_ISL_1554637, EPI_ISL_1554734, EPI_ISL_1554751, EPI_ISL_1554758, EPI_ISL_1554779, EPI_ISL_1554811, EPI_ISL_1554823, EPI_ISL_1575765, EPI_ISL_1575901, EPI_ISL_1575923, EPI_ISL_1575934, EPI_ISL_1576009, EPI_ISL_1576082, EPI_ISL_1576104, EPI_ISL_1576139, EPI_ISL_1576253, EPI_ISL_1576393, EPI_ISL_1576405, EPI_ISL_1576414, EPI_ISL_1576530, EPI_ISL_1576599, EPI_ISL_1576618, EPI_ISL_1576647, EPI_ISL_1576731, EPI_ISL_1580837, EPI_ISL_1580975, EPI_ISL_1580989, EPI_ISL_1580995, EPI_ISL_1581008, EPI_ISL_1581042, EPI_ISL_1581070, EPI_ISL_1581109, EPI_ISL_1581249, EPI_ISL_1581305, EPI_ISL_1581378, EPI_ISL_1581490, EPI_ISL_1581546, EPI_ISL_1581567, EPI_ISL_1581626, EPI_ISL_1581731, EPI_ISL_1581760, EPI_ISL_1581778, EPI_ISL_1581803, EPI_ISL_1592426, EPI_ISL_1592434, EPI_ISL_1592486, EPI_ISL_1592539, EPI_ISL_1592666, EPI_ISL_1592803, EPI_ISL_1592825, EPI_ISL_1592894, EPI_ISL_1593008, EPI_ISL_1593020, EPI_ISL_1593159, EPI_ISL_1593163, EPI_ISL_1593172, EPI_ISL_1615255, EPI_ISL_1615324, EPI_ISL_1615327, EPI_ISL_1615338, EPI_ISL_1615350, EPI_ISL_1615371, EPI_ISL_1678891, EPI_ISL_1678910, EPI_ISL_1678978, EPI_ISL_1679026, EPI_ISL_1679027, EPI_ISL_1679074, EPI_ISL_1679117, EPI_ISL_1679121, EPI_ISL_1679207, EPI_ISL_1679340, EPI_ISL_1679452, EPI_ISL_1679551, EPI_ISL_1679579, EPI_ISL_1679595, EPI_ISL_1679599, EPI_ISL_1679862, EPI_ISL_1679942, EPI_ISL_1679959, EPI_ISL_1679973, EPI_ISL_1680144, EPI_ISL_1680182, EPI_ISL_1680227, EPI_ISL_1680266, EPI_ISL_1680271, EPI_ISL_1680325, EPI_ISL_1690332, EPI_ISL_1690441, EPI_ISL_1690458, EPI_ISL_1690564, EPI_ISL_1690718, EPI_ISL_1690748, EPI_ISL_1690877, EPI_ISL_1690982, EPI_ISL_1691032, EPI_ISL_1691069, EPI_ISL_1691081, EPI_ISL_1691095, EPI_ISL_1691114, EPI_ISL_1691123, EPI_ISL_1691348, EPI_ISL_1691353, EPI_ISL_1694681, EPI_ISL_1701841, EPI_ISL_1701864, EPI_ISL_1734583, EPI_ISL_1734594, EPI_ISL_1734697, EPI_ISL_1734717, EPI_ISL_1734718, EPI_ISL_1734724, EPI_ISL_1734793, EPI_ISL_1734946, EPI_ISL_1734976, EPI_ISL_1734985, EPI_ISL_1734993, EPI_ISL_1735009, EPI_ISL_1735113, EPI_ISL_1735114, EPI_ISL_1735219, EPI_ISL_1735276, EPI_ISL_1735289, EPI_ISL_1735431, EPI_ISL_1735449, EPI_ISL_1735480, EPI_ISL_1735492, EPI_ISL_1735628, EPI_ISL_1735647, EPI_ISL_1735676, EPI_ISL_1753502, EPI_ISL_1753511, EPI_ISL_1796142, EPI_ISL_1796210, EPI_ISL_1796288, EPI_ISL_1796386, EPI_ISL_1796438, EPI_ISL_1796446, EPI_ISL_1796488, EPI_ISL_1796491, EPI_ISL_1796498, EPI_ISL_1796516, EPI_ISL_1796524, EPI_ISL_1796547, EPI_ISL_1796608, EPI_ISL_1796655, EPI_ISL_1796677, EPI_ISL_1796724, EPI_ISL_1796737, EPI_ISL_1796769, EPI_ISL_1796776, EPI_ISL_1796789, EPI_ISL_1796801, EPI_ISL_1796817, EPI_ISL_1796839, EPI_ISL_1796856, EPI_ISL_1796866, EPI_ISL_1796942, EPI_ISL_1796971, EPI_ISL_1796997, EPI_ISL_1797023, EPI_ISL_1797033, EPI_ISL_1797131, EPI_ISL_1797138, EPI_ISL_1797191, EPI_ISL_1797209, EPI_ISL_1797219, EPI_ISL_1797237, EPI_ISL_1797262, EPI_ISL_1797381, EPI_ISL_1797398, EPI_ISL_1797409, EPI_ISL_1803536, EPI_ISL_1803537, EPI_ISL_1803553, EPI_ISL_1803568, EPI_ISL_1803580, EPI_ISL_1803603, EPI_ISL_1803624, EPI_ISL_1803625, EPI_ISL_1803642, EPI_ISL_1803656, EPI_ISL_1803673, EPI_ISL_1803720, EPI_ISL_1803777, EPI_ISL_1803800, EPI_ISL_1803802, EPI_ISL_1803816, EPI_ISL_1803855, EPI_ISL_1803888, EPI_ISL_1803946, EPI_ISL_1803999, EPI_ISL_1804036, EPI_ISL_1804110, EPI_ISL_1804112, EPI_ISL_1804117, EPI_ISL_1804120, EPI_ISL_1804144, EPI_ISL_1804146, EPI_ISL_1804160, EPI_ISL_1804216, EPI_ISL_1804339, EPI_ISL_1804434, EPI_ISL_1804524, EPI_ISL_1804544, EPI_ISL_1804573, EPI_ISL_1804581, EPI_ISL_1804583, EPI_ISL_1804584, EPI_ISL_1804594, EPI_ISL_1804607, EPI_ISL_1804613, EPI_ISL_1804615, EPI_ISL_1804678, EPI_ISL_1804721, EPI_ISL_1804727, EPI_ISL_1817999, EPI_ISL_1818024, EPI_ISL_1818042, EPI_ISL_1906887, EPI_ISL_1906925, EPI_ISL_1907002, EPI_ISL_1907008, EPI_ISL_1907016, EPI_ISL_1907090, EPI_ISL_1907118, EPI_ISL_1907124, EPI_ISL_1907164, EPI_ISL_1907165, EPI_ISL_1907181, EPI_ISL_1907197, EPI_ISL_1907235, EPI_ISL_1907265, EPI_ISL_1907293, EPI_ISL_1907351, EPI_ISL_1907373, EPI_ISL_1907406, EPI_ISL_1907412, EPI_ISL_1907420, EPI_ISL_1907450, EPI_ISL_1907508, EPI_ISL_1907514, EPI_ISL_1907517, EPI_ISL_1907538, EPI_ISL_1907561, EPI_ISL_1907579, EPI_ISL_1907627, EPI_ISL_1907660, EPI_ISL_1907729, EPI_ISL_1907743, EPI_ISL_1907806, EPI_ISL_1907808, EPI_ISL_1907826, EPI_ISL_1907867, EPI_ISL_1907889, EPI_ISL_1907985, EPI_ISL_1908017, EPI_ISL_1908053, EPI_ISL_1908071, EPI_ISL_1908076, EPI_ISL_1908116, EPI_ISL_1908137, EPI_ISL_1923204, EPI_ISL_1923216, EPI_ISL_1923224, EPI_ISL_1923261, EPI_ISL_1923376, EPI_ISL_1923383, EPI_ISL_1923474, EPI_ISL_1923487, EPI_ISL_1923513, EPI_ISL_1923524, EPI_ISL_1923525, EPI_ISL_1923542, EPI_ISL_1923577, EPI_ISL_1923605, EPI_ISL_1923608, EPI_ISL_1923629, EPI_ISL_1923648, EPI_ISL_1923652, EPI_ISL_1923683, EPI_ISL_1923747, EPI_ISL_1923749, EPI_ISL_1923758, EPI_ISL_1923771, EPI_ISL_1923775, EPI_ISL_1923804, EPI_ISL_1923816, EPI_ISL_1923844, EPI_ISL_1923875, EPI_ISL_1923894, EPI_ISL_1923917, EPI_ISL_1923929, EPI_ISL_1923989, EPI_ISL_1924003, EPI_ISL_1924035, EPI_ISL_1924042, EPI_ISL_1924048, EPI_ISL_1924079, EPI_ISL_1924114, EPI_ISL_1924148, EPI_ISL_1924156, EPI_ISL_1924167, EPI_ISL_1924255, EPI_ISL_1924258, EPI_ISL_1935596, EPI_ISL_1942151, EPI_ISL_1942160, EPI_ISL_1990577, EPI_ISL_1990589, EPI_ISL_1990612, EPI_ISL_1990887, EPI_ISL_1990933, EPI_ISL_1990973, EPI_ISL_1991018, EPI_ISL_1991027, EPI_ISL_1991232, EPI_ISL_1991243, EPI_ISL_1991422, EPI_ISL_1991526, EPI_ISL_1991540, EPI_ISL_1991564, EPI_ISL_1991702, EPI_ISL_1991705, EPI_ISL_1991711, EPI_ISL_1991716, EPI_ISL_1991721, EPI_ISL_1991805

see above	Helix/Illumina	Centers for Disease Control and Prevention Division of Viral Diseases, Pathogen Discovery	Adrian Paskey; Alexandre Bolze; Ary Ascencio; Ben L. Rambo-Martin; Benjamin Rambo-Martin; Brad Sickler; Charlotte Rivera-Garcia; Christine Tran; Christopher Gulvick; Clinton R. Paden; Dakota Howard; Darlene Wagner; David Becker; Dhwani Batra; Duncan MacCannell; Efren Sandoval; Eileen de Feo; Elizabeth Cirulli; Eric Allen; Geraint Levan; James Lu; Jan Antico; Jason Caravas; Jason Nguyen; Jimmy Ramirez; Jingtao Liu; Kara Moser; Kelly Schiabor Barrett; Kim Gietzen; Magnus Isaksson; Marc Laurent; Matthew Schmerer; Matthew Tolentino; Nicole L. Washington; Peter W. Cook; Phil Febbo; Ryan Cho; Scott Sammons; Shannon Wickline; Shatavia Morrison; Sherry Wang; Simon White; Summer Galloway; Suxiang Tong; Tyler Cassens; William Lee; Yvette Unoarumhi

EPI_ISL_3149997, EPI_ISL_3149998, EPI_ISL_3149999, EPI_ISL_3150000, EPI_ISL_3150001, EPI_ISL_3150002, EPI_ISL_3150003, EPI_ISL_3150004, EPI_ISL_3150005, EPI_ISL_3150006, EPI_ISL_3150007, EPI_ISL_3150008, EPI_ISL_3150009, EPI_ISL_3150010, EPI_ISL_3150011, EPI_ISL_3150012, EPI_ISL_3150013, EPI_ISL_3150014, EPI_ISL_3150015, EPI_ISL_3150016, EPI_ISL_3150017

see above	Hermes Pardini	Universidade Federal de Ciencias da Saude de Porto Alegre	Andrea Roberto de Souza; Andy Goren; Carlos Gustavo Wambier; Claudia Elizabeth Thompson; Daniel do Nascimento Fonseca; Emilyn Oliveira Guerreiro; Flávio Adsuara Cadegiani; Liane Nanci Rotta; Patrícia Aline Gröhs Ferrareze; Ricardo Ariel Zimerman; Zhihua Ren
EPI_ISL_1262988	Hopital	National Reference Center for Viruses of Respiratory Infections, Institut Pasteur, Paris	Angela Brisebarre; Camille Capel; Etienne Simon-Lorière; Marion Barbet; Maud Vanpeene; Méline Bizard; Sylvie Behillil; Sylvie van der Werf; Vincent Enouf; Woercel Isabelle
EPI_ISL_1811228	Horquetas Rio Frio	Inciensa, Instituto Costarricense de Investigación y Enseñanza en Nutrición y Salud	Pérez-Corrales C & Montiel-Ulloa G
EPI_ISL_1533707	Hosp Mun Planalto Waldomiro de Paula	Instituto Adolfo Lutz, Interdiciplinary Procedures Center, Strategic Laboratory	Caio Vinicius Dias Lopes; Claudia Regina Gonçalves; Claudio Tavares Sacchi; Erica Valessa Ramos Gomes; Karoline Rodrigues Campos; Leonardo Jose Tadeu de Araujo

EPI_ISL_1381213, EPI_ISL_1381216, EPI_ISL_1381217, EPI_ISL_1516921, EPI_ISL_1554911, EPI_ISL_1583049, EPI_ISL_1621510, EPI_ISL_1621822, EPI_ISL_1675020, EPI_ISL_1700617, EPI_ISL_1700647, EPI_ISL_1700648, EPI_ISL_1700650, EPI_ISL_1821591, EPI_ISL_2029664, EPI_ISL_2030088, EPI_ISL_2279592, EPI_ISL_2279593, EPI_ISL_2790909

see above	Hospital	National Reference Center for Viruses of Respiratory Infections, Institut Pasteur, Paris	Adrien Pain; Amaury Vaysse; Angela Brisebarre; Camille Capel; Christophe Malabat; Corinne Maufrais; CéLine Ramanantsoa; Damien Mornico; Eleha Guillotel; Emmanuelle Permal; Etienne Simon-Lorière; Frédéric Lemoine; Gael Millot; Isabelle Woercel; JéRome Guinard; Laurence Louvet; Louise Lefrançois; Louvet Laurence; Marianne Burgard; Marion Barbet; Maud Vanpeene; Méline Bizard; Nabil Gastli; Pascale Martres; Pierre Lechat; Rousset Dominique; Serazin ValéRie; StéPhanie Marque Juillet; Sylvie Behillil; Sylvie Van der Werf; Sylvie van der Werf; Vincent Enouf
EPI_ISL_1443196, EPI_ISL_1443197, EPI_ISL_1443198	Hospital Aliança	Hospital São Rafael - IDOR	Aquiles Assunção Camelier; Aurea Angelica Paste; Bruno Solano de Freitas Souza; Carolina Kymie Vasques Nonaka; Elves A.P. Maciel; Isadora Cristina de Siqueira; Karoline Almeida Félix de Sousa; Margarida Celia L. C. Neves; Tiago Gräf; Victor Costa Araujo; Yasmin Santos Freitas Macêdo
EPI_ISL_1219021	Hospital Aviccena	Instituto Adolfo Lutz, Interdiciplinary Procedures Center, Strategic Laboratory	Caio Vinicius Dias Lopes; Claudia Regina Gonçalves; Claudio Tavares Sacchi; Erica Valessa Ramos Gomes; Karoline Rodrigues Campos
EPI_ISL_906080, EPI_ISL_906081	Hospital Beneficiencia Portuguesa	Instituto Adolfo Lutz, Interdiciplinary Procedures Center, Strategic Laboratory	Claudia Regina Gonçalves; Claudio Tavares Sacchi; Erica Valessa Ramos Gomes; Karoline Rodrigues Campos
EPI_ISL_940626, EPI_ISL_940627	Hospital Central Sao Caetano do Sul	Instituto Adolfo Lutz, Interdiciplinary Procedures Center, Strategic Laboratory	Claudia Regina Gonçalves; Claudio Tavares Sacchi; Erica Valessa Ramos Gomes; Karoline Rodrigues Campos
EPI_ISL_1121307	Hospital E Antonio Policarpo de Oliveira	Instituto Adolfo Lutz, Interdiciplinary Procedures Center, Strategic Laboratory	Caio Vinicius Dias Lopes; Claudia Regina Gonçalves; Claudio Tavares Sacchi; Erica Valessa Ramos Gomes; Karoline Rodrigues Campos
EPI_ISL_1303542	Hospital Estadual de Campanha Barradas	Instituto Adolfo Lutz, Interdiciplinary Procedures Center, Strategic Laboratory	Caio Vinicius Dias Lopes; Claudia Regina Gonçalves; Claudio Tavares Sacchi; Erica Valessa Ramos Gomes; Karoline Rodrigues Campos
EPI_ISL_1498917	Hospital Estadual de Mirandopolis	Instituto Adolfo Lutz, Interdiciplinary Procedures Center, Strategic Laboratory	Caio Vinicius Dias Lopes; Claudia Regina Gonçalves; Claudio Tavares Sacchi; Erica Valessa Ramos Gomes; Karoline Rodrigues Campos

EPI_ISL_1303537	Hospital Estadual de Vila Alpina	Instituto Adolfo Lutz, Interdiciplinary Procedures Center, Strategic Laboratory	Caio Vinicius Dias Lopes; Claudia Regina Gonçalves; Claudio Tavares Sacchi; Erica Valessa Ramos Gomes; Karoline Rodrigues Campos
EPI_ISL_1533709	Hospital Estadual de Vila Alpina Org Social Seconci Sao Paulo	Instituto Adolfo Lutz, Interdiciplinary Procedures Center, Strategic Laboratory	Caio Vinicius Dias Lopes; Claudia Regina Gonçalves; Claudio Tavares Sacchi; Erica Valessa Ramos Gomes; Karoline Rodrigues Campos; Leonardo Jose Tadeu de Araujo
EPI_ISL_2016620, EPI_ISL_2016621, EPI_ISL_2016622, EPI_ISL_2016623	Hospital Francesc de Borja (Gandia)	SeqCOVID-SPAIN consortium/IBV(CSIC)	Inmaculada Vidal Catalá; Isabel García Nava and SeqCOVID-SPAIN consortium; Mª Rosa Navarro Cots; Nieves Orta Mira
EPI_ISL_985404, EPI_ISL_1307715, EPI_ISL_1381872, EPI_ISL_1401467, EPI_ISL_1476981, EPI_ISL_1477061, EPI_ISL_1524839, EPI_ISL_1524840, EPI_ISL_1524841, EPI_ISL_1524842, EPI_ISL_1647783, EPI_ISL_1647841, EPI_ISL_1647852, EPI_ISL_1647854, EPI_ISL_1647855, EPI_ISL_1647858, EPI_ISL_1647859, EPI_ISL_1647945, EPI_ISL_1647946, EPI_ISL_1914363, EPI_ISL_1914364, EPI_ISL_1914366, EPI_ISL_1914367, EPI_ISL_1914368, EPI_ISL_3505904			
see above	Hospital General Universitario Gregorio Marañón	Hospital General Universitario Gregorio Marañón	Cristina Rodriguez-Grande; Darío García de Viedma; Darío García de Viedma.; Julia Suárez; Laura Pérez-Lago; Marta Herranz Martin; Patricia Muñoz; Pedro Sola Campoy; Pilar Catalán; Sergio Buenestado Serrano; Victor Manuel de la Cueva
EPI_ISL_2271475	Hospital General Universitario de Alicante - Instituto de Investigación Sanitaria y Biomédica de Alicante	SeqCOVID-SPAIN consortium/IBV(CSIC)	Carmen Molina Pardines and SeqCOVID-SPAIN consortium; Maripaz Ventero Martín
EPI_ISL_2614600	Hospital Geral de Guaianazes	Instituto Adolfo Lutz, Interdiciplinary Procedures Center, Strategic Laboratory	Caio Vinicius Dias Lopes; Claudia Regina Gonçalves; Claudio Tavares Sacchi; Erica Valessa Ramos Gomes; Karoline Rodrigues Campos; Leonardo Jose Tadeu de Araujo
EPI_ISL_1533725	Hospital Geral de Guarulhos	Instituto Adolfo Lutz, Interdiciplinary Procedures Center, Strategic Laboratory	Caio Vinicius Dias Lopes; Claudia Regina Gonçalves; Claudio Tavares Sacchi; Erica Valessa Ramos Gomes; Karoline Rodrigues Campos; Leonardo Jose Tadeu de Araujo
EPI_ISL_1533723	Hospital Geral de Itaquaquecetuba	Instituto Adolfo Lutz, Interdiciplinary Procedures Center, Strategic Laboratory	Caio Vinicius Dias Lopes; Claudia Regina Gonçalves; Claudio Tavares Sacchi; Erica Valessa Ramos Gomes; Karoline Rodrigues Campos; Leonardo Jose Tadeu de Araujo
EPI_ISL_2614580	Hospital Geral de Pedreira	Instituto Adolfo Lutz, Interdiciplinary Procedures Center, Strategic Laboratory	Caio Vinicius Dias Lopes; Claudia Regina Gonçalves; Claudio Tavares Sacchi; Erica Valessa Ramos Gomes; Karoline Rodrigues Campos; Leonardo Jose Tadeu de Araujo
EPI_ISL_940630, EPI_ISL_943967, EPI_ISL_943968, EPI_ISL_943969, EPI_ISL_943970, EPI_ISL_943971	Hospital Geral de Sao Paulo	Instituto Adolfo Lutz, Interdiciplinary Procedures Center, Strategic Laboratory	Claudia Regina Gonçalves; Claudio Tavares Sacchi; Erica Valessa Ramos Gomes; Karoline Rodrigues Campos
EPI_ISL_906075	Hospital Geral de Vila Penteado Dr Jose Pangella Sao Paulo	Instituto Adolfo Lutz, Interdiciplinary Procedures Center, Strategic Laboratory	Claudia Regina Gonçalves; Claudio Tavares Sacchi; Erica Valessa Ramos Gomes; Karoline Rodrigues Campos
EPI_ISL_1381069	Hospital Heliopolis	Instituto Adolfo Lutz, Interdiciplinary Procedures Center, Strategic Laboratory	Caio Vinicius Dias Lopes; Claudia Regina Gonçalves; Claudio Tavares Sacchi; Erica Valessa Ramos Gomes; Karoline Rodrigues Campos
EPI_ISL_2551531	Hospital Infantil Menino Jesus	Laboratório de Virologia Clínica e Molecular	Alex Fiorini; Ana Paula Salles Fernandes; Bruna Larotonda Telezynski; Danielle Bruna Leal Oliveira; Edison Luiz Durigon; Erick Gustavo Dorlass; Flavio Fonseca e Santuza Teixeira; Guilherme Pereira Scagion; Helena Perez Coelho; Hugo Sato; Karine Lima Lourenço; Luciano Matsumiya Thomazelli; Renata Peixoto; Rubens Daniel Miserani Magalhães; Tatiana Ometto
EPI_ISL_2551532	Hospital Infantnil Darcy Vargas	Laboratório de Virologia Clínica e Molecular	Alex Fiorini; Ana Paula Salles Fernandes; Bruna Larotonda Telezynski; Danielle Bruna Leal Oliveira; Edison Luiz Durigon; Erick Gustavo Dorlass; Flavio Fonseca e Santuza Teixeira; Guilherme Pereira Scagion; Helena Perez Coelho; Hugo Sato; Karine Lima Lourenço; Luciano Matsumiya Thomazelli; Renata Peixoto; Rubens Daniel Miserani Magalhães; Tatiana Ometto
EPI_ISL_2363535, EPI_ISL_2363555, EPI_ISL_2363558, EPI_ISL_2363563, EPI_ISL_2363564, EPI_ISL_2363565	Hospital Jaime Ferre - SAMCO Rafaela	Grupo de Genómica y Bioinformática del Instituto de Investigación de la Cadena Láctea CONICET-INTA on behalf of 'Proyecto Argentino Interinstitucional de genomica de SARS-CoV-2' (PAIS Consortium)	AF; Amadio; C; Eberhardt; Irazoqui; Isaia; JF; JM; MF; Pandolfi; Quaranta; V
EPI_ISL_3031345, EPI_ISL_3031346	Hospital Metropolitano Dr. Célio de Castro	Instituto René Rachou / Fiocruz Minas	Anna Salim; Cristina Fonseca; Gabriel Fernandes; Mariana Melo; Núbia Fernandes; Pedro Alves; Rosiane Pereira; Rubens do Monte Neto; Sandra Gava; Thaís Santos; Thaís Silva; Wilma Patrícia Bernardes
EPI_ISL_2614577	Hospital Municipal Antonio Giglio	Instituto Adolfo Lutz, Interdiciplinary Procedures Center, Strategic Laboratory	Caio Vinicius Dias Lopes; Claudia Regina Gonçalves; Claudio Tavares Sacchi; Erica Valessa Ramos Gomes; Karoline Rodrigues Campos; Leonardo Jose Tadeu de Araujo
EPI_ISL_1381070	Hospital Municipal Cidade Tiradentes Carmem Prudente	Instituto Adolfo Lutz, Interdiciplinary Procedures Center, Strategic Laboratory	Caio Vinicius Dias Lopes; Claudia Regina Gonçalves; Claudio Tavares Sacchi; Erica Valessa Ramos Gomes; Karoline Rodrigues Campos
EPI_ISL_1303541	Hospital Municipal Cidade Tiradentes Carmen Prudente	Instituto Adolfo Lutz, Interdiciplinary Procedures Center, Strategic Laboratory	Caio Vinicius Dias Lopes; Claudia Regina Gonçalves; Claudio Tavares Sacchi; Erica Valessa Ramos Gomes; Karoline Rodrigues Campos
EPI_ISL_1533695	Hospital Municipal Dr Mario Gatti	Instituto Adolfo Lutz, Interdiciplinary Procedures Center, Strategic Laboratory	Caio Vinicius Dias Lopes; Claudia Regina Gonçalves; Claudio Tavares Sacchi; Erica Valessa Ramos Gomes; Karoline Rodrigues Campos; Leonardo Jose Tadeu de Araujo
EPI_ISL_1731577, EPI_ISL_2003113	Hospital Municipal Dr Mario Gatti Campinas	Instituto Adolfo Lutz, Interdiciplinary Procedures Center, Strategic Laboratory	Caio Vinicius Dias Lopes; Claudia Regina Gonçalves; Claudio Tavares Sacchi; Erica Valessa Ramos Gomes; Karoline Rodrigues Campos; Katia Correa de Oliveira Santos; Leonardo Jose Tadeu de Araujo
EPI_ISL_2614590	Hospital Municipal Dr. Guido Guida	Instituto Adolfo Lutz, Interdiciplinary Procedures Center, Strategic Laboratory	Caio Vinicius Dias Lopes; Claudia Regina Gonçalves; Claudio Tavares Sacchi; Erica Valessa Ramos Gomes; Karoline Rodrigues Campos; Leonardo Jose Tadeu de Araujo
EPI_ISL_1620638	Hospital Municipal Dr. Ignacio Proença de Gouvea	Instituto Butantan / Mendelics	Alexander Roberto Precioso; Antonio Jorge Martins; Bibiana Santos; Claudia Renata dos Santos Barros; David Schlesinger; Debora Botequio Moretti; Dimas Tadeu Covas; Elaine Cristina Marqueze; Elaine Vieira dos Santos; Erika Freitas; Evandra Strazza Rodrigues; Flavia Aburjaile; José Salvatore Leister Patané; João Paulo Kitajima; Luiz Carlos Junior de Alcantara; Maria Carolina Elias; Marta Giovanetti; Rafael dos Santos Bezerra; Raul Machado Neto; Ricardo Haddad; Rodrigo Tocantins Calado.; Sandra Coccuzzo Sampaio; Simone Kashima; Svetoslav Nanev Slavov; Vagner Fonseca; Vincent Louis Viala
EPI_ISL_2614572	Hospital Municipal Ermelino Matarazzo	Instituto Adolfo Lutz, Interdiciplinary	Caio Vinicius Dias Lopes; Claudia Regina Gonçalves; Claudio Tavares Sacchi; Erica Valessa Ramos Gomes; Karoline Rodrigues Campos; Leonardo Jose Tadeu de Araujo

		Procedures Center, Strategic Laboratory	
EPI_ISL_2614597	Hospital Municipal de Mogi das Cruzes	Instituto Adolfo Lutz, Interdiciplinary Procedures Center, Strategic Laboratory	Caio Vinicius Dias Lopes; Claudia Regina Gonçalves; Claudio Tavares Sacchi; Erica Valessa Ramos Gomes; Karoline Rodrigues Campos; Leonardo Jose Tadeu de Araujo
EPI_ISL_1533696	Hospital Municipal de Pedreira	Instituto Adolfo Lutz, Interdiciplinary Procedures Center, Strategic Laboratory	Caio Vinicius Dias Lopes; Claudia Regina Gonçalves; Claudio Tavares Sacchi; Erica Valessa Ramos Gomes; Karoline Rodrigues Campos; Leonardo Jose Tadeu de Araujo
EPI_ISL_1358287	Hospital Nipo Brasileiro	Instituto Adolfo Lutz, Interdiciplinary Procedures Center, Strategic Laboratory	Caio Vinicius Dias Lopes; Claudia Regina Gonçalves; Claudio Tavares Sacchi; Erica Valessa Ramos Gomes; Karoline Rodrigues Campos
EPI_ISL_2614594	Hospital Regional de Cotia	Instituto Adolfo Lutz, Interdiciplinary Procedures Center, Strategic Laboratory	Caio Vinicius Dias Lopes; Claudia Regina Gonçalves; Claudio Tavares Sacchi; Erica Valessa Ramos Gomes; Karoline Rodrigues Campos; Leonardo Jose Tadeu de Araujo
EPI_ISL_940619, EPI_ISL_940620, EPI_ISL_940621, EPI_ISL_940622, EPI_ISL_940623, EPI_ISL_940624, EPI_ISL_940625			
see above	Hospital Sao Joaquim - Beneficiencia Portuguesa	Instituto Adolfo Lutz, Interdiciplinary Procedures Center, Strategic Laboratory	Claudia Regina Gonçalves; Claudio Tavares Sacchi; Erica Valessa Ramos Gomes; Karoline Rodrigues Campos
EPI_ISL_906076, EPI_ISL_906077	Hospital Sao Luiz Sao Caetano	Instituto Adolfo Lutz, Interdiciplinary Procedures Center, Strategic Laboratory	Claudia Regina Gonçalves; Claudio Tavares Sacchi; Erica Valessa Ramos Gomes; Karoline Rodrigues Campos
EPI_ISL_1628372	Hospital Sao Marcos da Sama Morro Agudo	Instituto Adolfo Lutz, Interdiciplinary Procedures Center, Strategic Laboratory	Caio Vinicius Dias Lopes; Claudia Regina Gonçalves; Claudio Tavares Sacchi; Erica Valessa Ramos Gomes; Karoline Rodrigues Campos; Katia Correa de Oliveira Santos; Leonardo Jose Tadeu de Araujo
EPI_ISL_3860415	Hospital Señor del Milagro	Instituto de Patologia Vegetal (CIAP-INTA) on behalf of 'Proyecto Argentino Interinstitucional de genomica de SARS-CoV-2' (PAIS Consortium)	Debat; FD; Fernanda Agüero; Fernández; HJ; Lavaque Esteban; Marquez, N.; Pamela Cajal; Raskovsky Viviana; Veronica Lesser
EPI_ISL_1608161, EPI_ISL_1608162, EPI_ISL_1608163, EPI_ISL_1608164, EPI_ISL_1608165, EPI_ISL_1608166, EPI_ISL_1608167, EPI_ISL_1608168, EPI_ISL_1608169, EPI_ISL_1608170, EPI_ISL_1608171			
see above	Hospital São Rafael - IDOR	Hospital São Rafael - IDOR	Ana Verena Almeida Mendes; Bruno Solano de Freitas Souza; Camila Araújo de Lorenzo Barcia; Carolina Kymie Vasques Nonaka; Clarissa Araújo Gurgel Rocha; Ian Marinho Santos; Iasmin Nogueira Bastos; Isadora Cristina de Siqueira; Janderson Lopes de Oliveira; Karoline Almeida Felix de Sousa; Maria Clara Brito de Santana; Rogério da Hora Passos; Thamires Gomes Lopes Weber; Tiago Gräf; Vanessa Ferreira Costa
EPI_ISL_1963916	Hospital Universitari Bellvitge	Microbiology Department	Aida Gonzalez-Diaz; Carmen Ardanuy; Jordi Camara; Jordi Niubó; Laura Calatayud; M Angeles Domínguez; Miguel Fernandez-Huerta; Sara Marti
EPI_ISL_1250798	Hospital Universitari Vall d'Hebron - Vall d'Hebron Institut de Recerca	Hospital Universitari Vall d'Hebron - Vall d'Hebron Institut de Recerca	Andrés Antón; Ariadna Rando; Carla Castillo; Cristina Andrés; Damir Garcia-Cehic; Josep F Abril; Josep Quer; Juliana Esperalba; Maria Carmen Martin; Maria Gema Codina; Maria Piñana; Tomàs Pumarola
EPI_ISL_1538412	Hospital Universitario Basurto Clinical Microbiology Service	Biocruces Bizkaia	Ana Belen de la Hoz; Estibaliza Ugalde Zarraga; Jose Luis Díaz de Tuesta del Arco; Mikel Gallego Rodrigo; Mikel Joseba Urrutikoetxea-Gutierrez; Mª Carmen Nieto Toboso
EPI_ISL_1533702, EPI_ISL_1533712	Hospital Universitario da USP Sao Paulo	Instituto Adolfo Lutz, Interdiciplinary Procedures Center, Strategic Laboratory	Caio Vinicius Dias Lopes; Claudia Regina Gonçalves; Claudio Tavares Sacchi; Erica Valessa Ramos Gomes; Karoline Rodrigues Campos; Leonardo Jose Tadeu de Araujo
EPI_ISL_1821210	Hospital Universitario de Marilia	Instituto Adolfo Lutz, Interdiciplinary Procedures Center, Strategic Laboratory	Caio Vinicius Dias Lopes; Claudia Regina Gonçalves; Claudio Tavares Sacchi; Erica Valessa Ramos Gomes; Karoline Rodrigues Campos; Leonardo Jose Tadeu de Araujo
EPI_ISL_2614569	Hospital Universitário da USP	Instituto Adolfo Lutz, Interdiciplinary Procedures Center, Strategic Laboratory	Caio Vinicius Dias Lopes; Claudia Regina Gonçalves; Claudio Tavares Sacchi; Erica Valessa Ramos Gomes; Karoline Rodrigues Campos; Leonardo Jose Tadeu de Araujo
EPI_ISL_3031312, EPI_ISL_3031316, EPI_ISL_3031317, EPI_ISL_3031318	Hospital da Baleia	Instituto René Rachou / Fiocruz Minas	Alana Oliveira; Anna Salim; Camila Corsini; Daniel Miranda; Gabriel Fernandes; Mozar de Castro; Nathalie Almeida; Pedro Alves; Priscilla Filgueiras; Rafaella Fortini; Raphael Silva; Raquel Vilela; Rubens do Monte Neto; Sarah Gomes; Thaís Silva; Wander Jeremias
EPI_ISL_1533699, EPI_ISL_2614575	Hospital das Clinicas Luzia de Pinho Melo	Instituto Adolfo Lutz, Interdiciplinary Procedures Center, Strategic Laboratory	Caio Vinicius Dias Lopes; Claudia Regina Gonçalves; Claudio Tavares Sacchi; Erica Valessa Ramos Gomes; Karoline Rodrigues Campos; Leonardo Jose Tadeu de Araujo
EPI_ISL_2614598	Hospital de Campanha Covid-19 Mairipora	Instituto Adolfo Lutz, Interdiciplinary Procedures Center, Strategic Laboratory	Caio Vinicius Dias Lopes; Claudia Regina Gonçalves; Claudio Tavares Sacchi; Erica Valessa Ramos Gomes; Karoline Rodrigues Campos; Leonardo Jose Tadeu de Araujo
EPI_ISL_875689	Hospital do Servidor Publico	Instituto Adolfo Lutz, Interdiciplinary Procedures Center, Strategic Laboratory	Claudia Regina Gonçalves; Claudio Tavares Sacchi; Erica Valessa Ramos Gomes; Karoline Rodrigues Campos
EPI_ISL_1121308	Hospital e Pronto Socorro Portinari	Instituto Adolfo Lutz, Interdiciplinary Procedures Center, Strategic Laboratory	Caio Vinicius Dias Lopes; Claudia Regina Gonçalves; Claudio Tavares Sacchi; Erica Valessa Ramos Gomes; Karoline Rodrigues Campos
EPI_ISL_2003926, EPI_ISL_2003956, EPI_ISL_2003966, EPI_ISL_2597572, EPI_ISL_2597577, EPI_ISL_2653036, EPI_ISL_3022828, EPI_ISL_3022865, EPI_ISL_3023231			
see above	Hospital of the University of Pennsylvania Molecular Pathology Lab	Bushman Lab - University of Pennsylvania	Abigail Glascock; Aoife M. Roche; Arupa Ganguly; Ayannah S. Fitzgerald; Brendan Kelly; Jevon Graham-Wooten; John Everett; John K. Everett; Kyle Rodino; Layla A. Khatib; Mike Feldman; Pascha Hokama; Ronald G. Collman and Frederic Bushan; Ronald G. Collman and Frederic Bushman; Samantha A. Whiteside; Scott Sherrill-Mix; Shantan Reddy; Young Hwang
EPI_ISL_1479113, EPI_ISL_1672871	Houston Health Dept.	Houston Health Dept.	Adolpho Lara; Pamela Brown; Ryker Penn; Yanlai Lai
EPI_ISL_1078954, EPI_ISL_1079206, EPI_ISL_1079246, EPI_ISL_1079917, EPI_ISL_1237447, EPI_ISL_1238196, EPI_ISL_1305221, EPI_ISL_1305222, EPI_ISL_1305226, EPI_ISL_2190495, EPI_ISL_2191170, EPI_ISL_2191231, EPI_ISL_2200291, EPI_ISL_2201494, EPI_ISL_2203377, EPI_ISL_2204974, EPI_ISL_2205669, EPI_ISL_2211977, EPI_ISL_2212003, EPI_ISL_2212042, EPI_ISL_2213726, EPI_ISL_2216599, EPI_ISL_2216645, EPI_ISL_2216933, EPI_ISL_2216950, EPI_ISL_2217100, EPI_ISL_2221466, EPI_ISL_2221525, EPI_ISL_2222823, EPI_ISL_2222893, EPI_ISL_2223003, EPI_ISL_2223017, EPI_ISL_2224440			
see above	Houston Methodist Hospital	Houston Methodist Hospital	Ilya J. Finkelstein; James J. Davis; Jessica Cambric; Jimmy Gollihar; Kristina Reppond; Layne Pruitt; Madison N. Shyer; Marcus Nguyen; Matthew Ojeda Saavedra; Paul A. Christensen; Prasanti Yerramilli; Randall J. Olsen; Robert Olson; Ryan Gadd; S. Wesley Long; Sishir Subedi; and James M. Musser
EPI_ISL_1756322	Hôpital Ambroise-Paré	Department of Virology, Henri Mondor University Hospital,	Alexandre Soulier; Christophe Rodriguez; Elisabeth Trawinski; Guillaume Gricourt; Jean-Michel Pawlotsky; Melissa N'Debi; Slim Fourati; Vanessa Demontant

		Assistance Publique Hôpitaux de Paris, Université Paris-Est Créteil, INSERM U955	
EPI_ISL_1754996, EPI_ISL_1755133, EPI_ISL_1755809, EPI_ISL_1757613	Hôpital Avicenne	Department of Virology, Henri Mondor University Hospital, Assistance Publique Hôpitaux de Paris, Université Paris-Est Créteil, INSERM U955	Alexandre Soulier; Christophe Rodriguez; Elisabeth Trawinski; Guillaume Gricourt; Jean-Michel Pawlotsky; Melissa N'Debi; Slim Fourati; Vanessa Demontant
EPI_ISL_1757581	Hôpital Cochin	Department of Virology, Henri Mondor University Hospital, Assistance Publique Hôpitaux de Paris, Université Paris-Est Créteil, INSERM U955	Alexandre Soulier; Christophe Rodriguez; Elisabeth Trawinski; Guillaume Gricourt; Jean-Michel Pawlotsky; Melissa N'Debi; Slim Fourati; Vanessa Demontant
EPI_ISL_1404536, EPI_ISL_1404538	Hôpital Henri Mondor	Department of Virology, Henri Mondor University Hospital, Assistance Publique Hôpitaux de Paris, Université Paris-Est Créteil, INSERM U955	Alexandre Soulier; Christophe Rodriguez; Elisabeth Trawinski; Guillaume Gricourt; Jean-Michel Pawlotsky; Melissa N'Debi; Slim Fourati; Vanessa Demontant
EPI_ISL_2614544, EPI_ISL_2614545, EPI_ISL_2614546, EPI_ISL_2614547, EPI_ISL_2614549, EPI_ISL_2614550, EPI_ISL_2614551, EPI_ISL_2614552, EPI_ISL_2614553, EPI_ISL_2614555, EPI_ISL_2614557, EPI_ISL_2614558, EPI_ISL_2614559, EPI_ISL_2614560, EPI_ISL_2614561, EPI_ISL_2614562, EPI_ISL_2614563			
see above	IAL Presidente Prudente	Instituto Adolfo Lutz, Interdiciplinary Procedures Center, Strategic Laboratory	Caio Vinicius Dias Lopes; Claudia Regina Gonçalves; Claudio Tavares Sacchi; Erica Valessa Ramos Gomes; Karoline Rodrigues Campos; Leonardo Jose Tadeu de Araujo
EPI_ISL_1358288, EPI_ISL_1358289, EPI_ISL_1358290	IAL Regional de Aracatuba	Instituto Adolfo Lutz, Interdiciplinary Procedures Center, Strategic Laboratory	Caio Vinicius Dias Lopes; Claudia Regina Gonçalves; Claudio Tavares Sacchi; Erica Valessa Ramos Gomes; Karoline Rodrigues Campos
EPI_ISL_1086044, EPI_ISL_1096131, EPI_ISL_1096132, EPI_ISL_1096134, EPI_ISL_1121310, EPI_ISL_1121313, EPI_ISL_1121314			
see above	IAL Regional de Bauru	Instituto Adolfo Lutz, Interdiciplinary Procedures Center, Strategic Laboratory	Caio Vinicius Dias Lopes; Claudia Regina Gonçalves; Claudio Tavares Sacchi; Erica Valessa Ramos Gomes; Karoline Rodrigues Campos
EPI_ISL_1171619, EPI_ISL_1171653, EPI_ISL_1171656, EPI_ISL_1171657, EPI_ISL_1171658, EPI_ISL_1171666, EPI_ISL_1171672, EPI_ISL_1171674			
see above	IAL Regional de Presidente Prudente	Instituto Adolfo Lutz, Interdiciplinary Procedures Center, Strategic Laboratory	Caio Vinicius Dias Lopes; Claudia Regina Gonçalves; Claudio Tavares Sacchi; Erica Valessa Ramos Gomes; Karoline Rodrigues Campos
EPI_ISL_1139070	IAL Regional de Ribeirao Preto	Instituto Adolfo Lutz, Interdiciplinary Procedures Center, Strategic Laboratory	Caio Vinicius Dias Lopes; Claudia Regina Gonçalves; Claudio Tavares Sacchi; Erica Valessa Ramos Gomes; Karoline Rodrigues Campos
EPI_ISL_1381048, EPI_ISL_1381061, EPI_ISL_1381062, EPI_ISL_1381065	IAL Regional de Santo Andre	Instituto Adolfo Lutz, Interdiciplinary Procedures Center, Strategic Laboratory	Caio Vinicius Dias Lopes; Claudia Regina Gonçalves; Claudio Tavares Sacchi; Erica Valessa Ramos Gomes; Karoline Rodrigues Campos
EPI_ISL_1219025, EPI_ISL_1293059, EPI_ISL_1293063, EPI_ISL_1293067, EPI_ISL_1293077, EPI_ISL_1293080	IAL Regional de Sorocaba	Instituto Adolfo Lutz, Interdiciplinary Procedures Center, Strategic Laboratory	Caio Vinicius Dias Lopes; Claudia Regina Gonçalves; Claudio Tavares Sacchi; Erica Valessa Ramos Gomes; Karoline Rodrigues Campos
EPI_ISL_1303520, EPI_ISL_1303522, EPI_ISL_1303525, EPI_ISL_1303529	IAL Regional de São Jose do Rio Preto	Instituto Adolfo Lutz, Interdiciplinary Procedures Center, Strategic Laboratory	Caio Vinicius Dias Lopes; Claudia Regina Gonçalves; Claudio Tavares Sacchi; Erica Valessa Ramos Gomes; Karoline Rodrigues Campos
EPI_ISL_1960074	ICMT-APARTADO	Universidad Nacional de Colombia - Laboratorio Genómico One Health	Andres F. Cardona-Rios; Carlos Franco-Muñoz; Carolina Muñoz-Arango; Celeny Ortiz; Daniel O. Maldonado-Perez; Diego A. Álvarez-Díaz; Hector Alejandro Ruiz-Moreno; Idabely Betancur Ortiz; Jorge E. Osorio; Juan P. Hernandez-Ortiz; Karl A Ciuoderis; Katherine Laiton-Donato; Laura Silvana Perez; Lina M. Hurtado; Marcela Mercado-Reyes; Maria Angélica Maya; Maria Stella López; Rita Almanza Payares; Sandra Ines Cano; Simón Villegas Velásquez
EPI_ISL_2234873, EPI_ISL_2234874, EPI_ISL_2234875, EPI_ISL_2234886, EPI_ISL_2234900, EPI_ISL_2234903, EPI_ISL_2234905, EPI_ISL_2444776, EPI_ISL_2444778, EPI_ISL_2444779, EPI_ISL_2444781, EPI_ISL_2444782, EPI_ISL_2444787, EPI_ISL_2444789, EPI_ISL_2444790, EPI_ISL_2444791, EPI_ISL_2444798, EPI_ISL_2444801, EPI_ISL_2444805, EPI_ISL_2444808, EPI_ISL_2444809, EPI_ISL_2444811, EPI_ISL_2444816, EPI_ISL_2444818, EPI_ISL_2444831, EPI_ISL_2444834, EPI_ISL_2444837, EPI_ISL_4071892, EPI_ISL_4071893			
see above	IICS-UNA	IICS-UNA	Adriana Valenzuela; Alejandra Rojas; Chyntia Diaz; Eva Nara; Fatima Cardozo; Florencia del Puerto; Joel Ortiz; Jonas Fernandez; Laura Franco; Laura Mendoza; Leticia Rojas; Magaly Martinez; Maria Eugenia Galeano.
EPI_ISL_3912230	IJF INSTITUTO DR JOSE FROTA CENTRAL	Analytical Competence Molecular Epidemiology Lab/ACME, Oswaldo Cruz Foundation, Ceara (FIOCRUZ CE)	Cleber Furtado Aksenen; Fabio Miyajima; Fernando Braga Stehling; Francisco Eder de Moura Lopes; Jamille Maria Mendes Bezerra; Joaquim Cesar do Nascimento Sousa Junior; Pedro Miguel Carneiro Jeronimo; Suzana Porto Almeida & Lucas Delerino on behalf of COVID-19 FIOCRUZ Genomic Network; Thais Ferreira de Oliveira; Thais de Oliveira Costa; Ticiane Cavalcante de Souza; Veridiana Pessoa Miyajima
EPI_ISL_1446979, EPI_ISL_1823782, EPI_ISL_1823788	IL Department of Public Health Chicago Laboratory	Centers for Disease Control and Prevention Division of Viral Diseases, Pathogen Discovery	Alison Laufer Halpin; Ben L. Rambo-Martin; Clinton R. Paden; Dakota Howard; Darlene Wagner; Dave Wentworth; Dhwani Batra; Jasmine Padilla; Justin Lee; Katie Dillon; Krista Queen; Kristen Knipe; Kristine Lacek; Mark Burroughs; Matthew Schmerer; Mili Sheth; Peter Cook; Sam Shepard; Sarah Nobles; Shoshona Le; Suxiang Tong; Vivien Dugan; Yvette Unoarumhi
EPI_ISL_1509934	IL Department of Public Health Chicago Laboratory	Genomics and Discovery, Respiratory Viruses Branch, Division of Viral Diseases, Centers for Disease Control and Prevention	Adam Retchless; Anna Kelleher; Anna Montmayeur; Anna Uehara; Brian Lynch; Clinton R. Paden; Haibin Wang; Han Jia Justin Ng; Jing Zhang; Justin Lee; Krista Queen; Mark Burroughs; Peter Cook; Rachel Marine; Suxiang Tong; Yan Li; Ying Tao
EPI_ISL_1798496, EPI_ISL_1798498, EPI_ISL_1798508, EPI_ISL_1798561, EPI_ISL_1798583, EPI_ISL_1937768, EPI_ISL_1937770, EPI_ISL_1937771, EPI_ISL_1937777, EPI_ISL_1937780, EPI_ISL_1937781, EPI_ISL_1937783, EPI_ISL_1937784, EPI_ISL_1937785, EPI_ISL_1989977, EPI_ISL_3354527, EPI_ISL_3354528, EPI_ISL_3354530, EPI_ISL_3354531, EPI_ISL_3354532, EPI_ISL_3354536, EPI_ISL_3354545			
see above	IL Dept. of Public Health Springfield Laboratory	Centers for Disease Control and Prevention Division of Viral Diseases, Pathogen Discovery	Alex Burgin; Alison Laufer Halpin; Ben L. Rambo-Martin; Ben Rambo-Martin; Clinton Paden; Clinton R. Paden; Dakota Howard; Darlene Wagner; Dave Wentworth; Dhwani Batra; Jasmine Padilla; Justin Lee; Katie Dillon; Krista Queen; Kristen Knipe; Kristine Lacek; Mark Burroughs; Matthew Schmerer; Meghan Bentz; Mili Sheth; Peter Cook; Sam Shepard; Sarah Nobles; Shoshona Le; Suxiang Tong; Vivien Dugan; Yvette Unoarumhi
EPI_ISL_1408896, EPI_ISL_1509935	IL Dept. of Public Health Springfield Laboratory	Genomics and Discovery, Respiratory Viruses Branch, Division of Viral Diseases, Centers for Disease Control and Prevention	Adam Retchless; Anna Kelleher; Anna Montmayeur; Anna Uehara; Brian Lynch; Clinton R. Paden; Haibin Wang; Han Jia Justin Ng; Jing Zhang; Justin Lee; Krista Queen; Mark Burroughs; Peter Cook; Rachel Marine; Suxiang Tong; Yan Li; Ying Tao
EPI_ISL_1639313,	IMD - Medizinisches	Robert Koch Institute	

EPI_ISL_1639325, EPI_ISL_1722204, EPI_ISL_1722214, EPI_ISL_1722223	Labor Rostock		
EPI_ISL_1399627	IMM	Instituto Nacional de Saude (INSA)	Borges et al
EPI_ISL_1213173, EPI_ISL_1213175, EPI_ISL_1213177, EPI_ISL_1213178, EPI_ISL_1213180, EPI_ISL_1213182, EPI_ISL_1213183, EPI_ISL_1213185, EPI_ISL_1213187, EPI_ISL_1213192, EPI_ISL_1213194, EPI_ISL_1213196, EPI_ISL_1213197, EPI_ISL_1213199			
see above	IMT-UFRN/RN	Bioinformatics Laboratory / LNCC	Alessandra P Lamarca; Alexandra L Gerber; Ana Paula Melo Mariano; Ana Paula de C Guimarães; Ana Tereza R Vasconcelos; Angela Maria Guimarães Santos; Bianca Mendes Maciel; Danielle Angst Secco; Eduardo Sérgio Soares Sousa; Eloiza Helena Campana; Francisco Paulo Freire Neto; George Rego Albuquerque; Kátia Castanho Scortecci; Lucymara Fassarella Agnez Lima; Luiz G P de Almeida; Luís Cristóvão Porto; Otavio J. Brustolini; Paulo Ricardo Nascimento; Ronaldo da Silva Francisco Jr; Sandra Rocha Gadelha; Selma Maria Bezerra Jeronimo; Vinícius Pietta Perez
EPI_ISL_1824049	IN State Department of Health Laboratory Services	Centers for Disease Control and Prevention Division of Viral Diseases, Pathogen Discovery	Alison Laufer Halpin; Ben L. Rambo-Martin; Clinton R. Paden; Dakota Howard; Darlene Wagner; Dave Wentworth; Dhwani Batra; Jasmine Padilla; Justin Lee; Katie Dillon; Krista Queen; Kristen Knipe; Kristine Lacek; Mark Burroughs; Matthew Schmerer; Mili Sheth; Peter Cook; Sam Shepard; Sarah Nobles; Shoshona Le; Suxiang Tong; Vivien Dugan; Yvette Unoarumhi
EPI_ISL_1648085, EPI_ISL_1818776, EPI_ISL_1841017, EPI_ISL_1841026, EPI_ISL_1841032	IN State Department of Health Laboratory Services	IN State Department of Health Laboratory Services	Ankita Kashikar; Brian Pope; Cassandra Campion; Jamie Yeadon; Kyle Brownlee; Lixia Liu; Mark Glazier; Melissa Hindenlang
EPI_ISL_2628036, EPI_ISL_2628126, EPI_ISL_2628194, EPI_ISL_2628208	INHRR	Laboratorio de Virología Molecular	Carmen L Loureiro; CoViVen Group; Domingo J Garzaro; Esmeralda Vizzi; Flor H Pujol; Héctor R Rangel; José Luis Zambrano; Lieska Rodríguez; Mariana Hidalgo; Pierina D´Angelo; Rossana C Jaspe; Víctor Alarcón; Yoneira Sulbaran; Zoila Moros
EPI_ISL_1055003, EPI_ISL_1547381	INSA	Instituto Nacional de Saude (INSA)	Borges et al
EPI_ISL_1589853	INSACOG-WB	National Institute of Biomedical Genomics – INSACOG	Ajay Chakraborti; Arindam Maitra; Bhaswati Bandyopadhyay; Nidhan Kumar Biswas; Saumitra Das; Sreedhar Chinnaswamy; Tamal Ghosh
EPI_ISL_1854237	IPVC	Instituto Nacional de Saude (INSA)	Borges et al
EPI_ISL_2612488	ISDH	Quantigen Biosciences	Paul J. Childress
EPI_ISL_2628271, EPI_ISL_2628298, EPI_ISL_2628301, EPI_ISL_2628302, EPI_ISL_2628303, EPI_ISL_2628304	IVIC	Laboratorio de Virología Molecular	Carmen L Loureiro; CoViVen Group; Domingo J Garzaro; Esmeralda Vizzi; Flor H Pujol; Héctor R Rangel; José Luis Zambrano; Lieska Rodríguez; Mariana Hidalgo; Pierina D´Angelo; Rossana C Jaspe; Víctor Alarcón; Yoneira Sulbaran; Zoila Moros
EPI_ISL_2335108, EPI_ISL_2335136	IVY3 Central Lab, Vanderbilt University Medical Center	Lauring Lab, University of Michigan, Department of Microbiology and Immunology	Gilbert
EPI_ISL_1663079, EPI_ISL_1663095, EPI_ISL_1663107, EPI_ISL_1663111, EPI_ISL_1663114, EPI_ISL_1663121, EPI_ISL_1663124, EPI_ISL_1663138, EPI_ISL_1663139, EPI_ISL_1742340, EPI_ISL_1742365, EPI_ISL_1742374, EPI_ISL_1742380, EPI_ISL_1742386, EPI_ISL_1742388, EPI_ISL_1742389, EPI_ISL_1742396, EPI_ISL_1742420, EPI_ISL_1809453, EPI_ISL_1809465, EPI_ISL_1809503, EPI_ISL_1809515, EPI_ISL_1809572, EPI_ISL_1809580, EPI_ISL_1809623, EPI_ISL_1809635, EPI_ISL_1809677, EPI_ISL_1809701, EPI_ISL_1809732, EPI_ISL_1964997, EPI_ISL_1964998, EPI_ISL_1965078, EPI_ISL_2800847			
see above	Illinois Department of Public Health	Illinois Department of Public Health - Chicago Lab	Ira Heimler; Joel Price; Vineet K. Dhiman
EPI_ISL_1478879, EPI_ISL_1478906, EPI_ISL_1478920, EPI_ISL_1500158, EPI_ISL_1500175, EPI_ISL_1500177, EPI_ISL_1500184, EPI_ISL_1500221, EPI_ISL_1500223, EPI_ISL_1500224, EPI_ISL_1500225, EPI_ISL_1500233, EPI_ISL_1500243, EPI_ISL_1553175, EPI_ISL_1553176, EPI_ISL_1553179, EPI_ISL_1553184, EPI_ISL_1553205, EPI_ISL_1553207, EPI_ISL_1553209, EPI_ISL_1553227, EPI_ISL_1553236, EPI_ISL_1553238, EPI_ISL_1553239, EPI_ISL_1595524, EPI_ISL_1595531, EPI_ISL_1595533, EPI_ISL_1595535, EPI_ISL_1595554, EPI_ISL_1595560, EPI_ISL_1595569, EPI_ISL_1595578, EPI_ISL_1595583, EPI_ISL_1595585, EPI_ISL_1595592, EPI_ISL_1624091, EPI_ISL_1624095, EPI_ISL_1624117, EPI_ISL_1624119, EPI_ISL_1624127, EPI_ISL_1624129, EPI_ISL_1624142, EPI_ISL_1624158, EPI_ISL_1708240, EPI_ISL_1708241, EPI_ISL_1708243, EPI_ISL_1708244, EPI_ISL_1708248, EPI_ISL_1708254, EPI_ISL_1708257, EPI_ISL_1708258, EPI_ISL_1708259, EPI_ISL_1708262, EPI_ISL_1708274, EPI_ISL_1708280, EPI_ISL_1708281, EPI_ISL_1708282, EPI_ISL_1708285, EPI_ISL_1708304, EPI_ISL_1708309, EPI_ISL_1732553, EPI_ISL_1732558, EPI_ISL_1732562, EPI_ISL_1732575, EPI_ISL_1732578, EPI_ISL_1732601, EPI_ISL_1732603, EPI_ISL_1732606, EPI_ISL_1732608, EPI_ISL_1732612, EPI_ISL_1732615, EPI_ISL_1732617, EPI_ISL_1732618, EPI_ISL_1732627, EPI_ISL_1732628, EPI_ISL_1789122, EPI_ISL_1789141, EPI_ISL_1789152, EPI_ISL_1789174, EPI_ISL_1789175, EPI_ISL_1789176, EPI_ISL_1789177, EPI_ISL_1789200, EPI_ISL_1825694, EPI_ISL_1825695, EPI_ISL_1825696, EPI_ISL_1825701, EPI_ISL_1825704, EPI_ISL_1825705, EPI_ISL_1825707, EPI_ISL_1825714, EPI_ISL_1825716, EPI_ISL_1825720, EPI_ISL_1825723, EPI_ISL_1825726, EPI_ISL_1825731, EPI_ISL_1825742, EPI_ISL_1825743, EPI_ISL_1825744, EPI_ISL_1825748, EPI_ISL_1825749, EPI_ISL_1825750, EPI_ISL_1825758, EPI_ISL_1825761, EPI_ISL_1825762, EPI_ISL_1825763, EPI_ISL_1825764, EPI_ISL_1825766, EPI_ISL_1825771, EPI_ISL_1825776, EPI_ISL_1825779, EPI_ISL_1825781, EPI_ISL_1825783, EPI_ISL_1825786, EPI_ISL_1825792, EPI_ISL_1825795, EPI_ISL_1825796, EPI_ISL_1825797, EPI_ISL_1825804, EPI_ISL_1825806, EPI_ISL_1825810, EPI_ISL_1825811, EPI_ISL_1825812, EPI_ISL_1825821, EPI_ISL_1825847, EPI_ISL_1825858, EPI_ISL_1825863, EPI_ISL_1825864, EPI_ISL_1825866, EPI_ISL_1825869, EPI_ISL_1825873, EPI_ISL_1825874, EPI_ISL_1938496, EPI_ISL_1938501, EPI_ISL_1938521, EPI_ISL_1938540, EPI_ISL_2000783, EPI_ISL_2000811, EPI_ISL_2086537, EPI_ISL_2086538, EPI_ISL_2086547, EPI_ISL_2086566, EPI_ISL_2203511, EPI_ISL_2203512, EPI_ISL_2203516, EPI_ISL_2203518, EPI_ISL_2203534			
see above	Illinois Department of Public Health - Springfield Lab	Illinois Department of Public Health - Springfield Lab	Bryan Sim; Gordon McCall
EPI_ISL_1628603, EPI_ISL_1810847	InDRE	Instituto Nacional de Medicina Genomica	Alcaraz N; Canseco Mendez JC; Cedro-Tanda A; Garcia-Cardenas FJ; Gisela Barrera-Badillo; Gonzalez-Barrera D; Gonzalez-Woge MA; Herrera-Montalvo LA.; Hidalgo-Miranda A; Irma Lopez-Martinez; Jose Ernesto Ramirez González; Mendoza-Vargas A; Miranda-Ortiz H; Munguia-Garza P; Ramirez-Vega O; Rangel-DeLeon D; Reyes-Grajeda JP; Rosas-Escobar P
EPI_ISL_1517432, EPI_ISL_1517433	Inciensa, Instituto Costarricense de Investigación y Enseñanza en Nutrición y Salud	Inciensa, Instituto Costarricense de Investigación y Enseñanza en Nutrición y Salud	Barboza-Arguedas E & Centeno-Miranda M; Cristian Peréz-Corrales; Cristian Peréz-Corrales & Barboza-Arguedas E
EPI_ISL_2011473, EPI_ISL_2011497, EPI_ISL_2011499, EPI_ISL_2011527, EPI_ISL_2136470, EPI_ISL_2136502, EPI_ISL_2136512			
see above	Incyte Diagnostics Spokane	Seattle Flu Study	Amanda Adler; Barry R. Lutz; Benjamin Pelle; Brian Hiatt; Caitlin R. Wolf; Chris D. Frazar; Deborah A. Nickerson; Elisabeth Brandstetter; Erica Ryke; Geoff Melly; Helen Y. Chu; Janet A. Englund; Jay Shendure; Jover Lee; Kairsten Fay; Kirsten Lacombe; Lea M. Starita; Mark J. Rieder; Matthew Richardson; Matthew Thompson; Melissa Truong; Michael Boeckh; Michael Famulare; Misja Ilcisin; Peter D. Han; Philip Dykema; Romesh Gautom; Scott Lindquist; Thomas R. Sibley; Trevor Bedford
EPI_ISL_1691733, EPI_ISL_1691981, EPI_ISL_1692039, EPI_ISL_1693117, EPI_ISL_1693319, EPI_ISL_1693672, EPI_ISL_1693959, EPI_ISL_1694045, EPI_ISL_1694128, EPI_ISL_1694246, EPI_ISL_1694530, EPI_ISL_1702242, EPI_ISL_1702287, EPI_ISL_1732753, EPI_ISL_1732776, EPI_ISL_1732822, EPI_ISL_1732836, EPI_ISL_1795455, EPI_ISL_1795478, EPI_ISL_1795595, EPI_ISL_1802742, EPI_ISL_1802760, EPI_ISL_1802932, EPI_ISL_1803059, EPI_ISL_1803119, EPI_ISL_1803442, EPI_ISL_1817991, EPI_ISL_2013564, EPI_ISL_2013828, EPI_ISL_2013856, EPI_ISL_2014055, EPI_ISL_2014084, EPI_ISL_2014277, EPI_ISL_2014479, EPI_ISL_2014563, EPI_ISL_2047287, EPI_ISL_2047488, EPI_ISL_2283321, EPI_ISL_3305237			
see above	Infinity Biologix	Centers for Disease Control and Prevention Division of Viral Diseases, Pathogen Discovery	Adrian Paskey; Benjamin Rambo-Martin; Chirayu Goswami; Christian Bixby; Christopher Gulvick; Clinton R. Paden; Dakota Howard; Darlene Wagner; Dhwani Batra; Duncan MacCannell; Jason Caravas; Jonathan Schultz; Kara Moser; Matthew Schmerer; Peter W. Cook; Robin Grimwood; Russ Hager; Scott Sammons; Shatavia Morrison; Yihe Wang; Yvette Unoarumhi
EPI_ISL_2280284	Institut Pasteur de Guyane	Institut Pasteur	Angela Brisebarre; Camille Capel; Christophe Malabat; Corinne Maufrais; Dominique Rousset; Emmanuelle Permal; Etienne Simon-Lorière; Frédéric Lemoine; Louise Lefrançois; Marion Barbet; Maud Vanpeene; Méline Bizard; Sylvie Behillil; Sylvie Van der Werf; Vincent Enouf
EPI_ISL_1656147, EPI_ISL_1738807	Institute of Microbiology, Universidad San Francisco de Quito	Institute of Microbiology, Universidad San Francisco de Quito	Belén Prado-Vivar; Bernardo Gutiérrez; Freddy Saldarriaga-Mera; Gabriel Trueba; Juan José Guadalupe; Manuel Jaramillo; Mayra Perero Intriago; Michelle Grunauer; Miguel Sacoto Mazini; Monica Becerra-Wong; Patricio Rojas-Silva; Paúl Cárdenas; Sully Márquez; Verónica Barragán
EPI_ISL_2245179, EPI_ISL_2245184, EPI_ISL_2245185	Instituto Adolfo Lutz	Coordenação Geral de Laboratórios de Saúde Pública (CGLAB/DAEVS/SVS/MS)	Vagner Fonseca; et al.
EPI_ISL_1715146, EPI_ISL_1715147, EPI_ISL_1731578, EPI_ISL_1731579, EPI_ISL_1731580, EPI_ISL_1752642, EPI_ISL_1752643, EPI_ISL_1821214, EPI_ISL_1821215, EPI_ISL_1821216, EPI_ISL_1821217, EPI_ISL_1821218, EPI_ISL_1821219, EPI_ISL_1821220, EPI_ISL_1821221, EPI_ISL_1821222, EPI_ISL_1821223, EPI_ISL_1821224			
see above	Instituto Adolfo Lutz - Regional de Aracatuba	Instituto Adolfo Lutz, Interdiciplinary Procedures Center, Strategic Laboratory	Caio Vinicius Dias Lopes; Claudia Regina Gonçalves; Claudio Tavares Sacchi; Erica Valessa Ramos Gomes; Karoline Rodrigues Campos; Katia Correa de Oliveira Santos; Leonardo Jose Tadeu de Araujo
EPI_ISL_2691109, EPI_ISL_2919236, EPI_ISL_2919239	Instituto Adolfo Lutz - Regional de Bauru	Instituto Adolfo Lutz, Interdiciplinary Procedures Center, Strategic Laboratory	Caio Vinicius Dias Lopes; Claudia Regina Gonçalves; Claudio Tavares Sacchi; Erica Valessa Ramos Gomes; Karoline Rodrigues Campos; Leonardo Jose Tadeu de Araujo
EPI_ISL_906068, EPI_ISL_906069, EPI_ISL_2003162, EPI_ISL_2003163, EPI_ISL_2003167, EPI_ISL_2003168, EPI_ISL_2614520, EPI_ISL_2614521, EPI_ISL_2614525, EPI_ISL_2614529, EPI_ISL_2614530, EPI_ISL_2614531, EPI_ISL_2614533, EPI_ISL_2614534, EPI_ISL_2614535, EPI_ISL_2614536, EPI_ISL_2614537, EPI_ISL_2614538, EPI_ISL_2828688, EPI_ISL_2828689			
see above	Instituto Adolfo Lutz - Regional de Campinas	Instituto Adolfo Lutz, Interdiciplinary Procedures Center,	Caio Vinicius Dias Lopes; Claudia Regina Gonçalves; Claudio Tavares Sacchi; Erica Valessa Ramos Gomes; Karoline Rodrigues Campos; Leonardo Jose Tadeu de Araujo

		Strategic Laboratory	
EPI_ISL_1821234, EPI_ISL_1821235, EPI_ISL_1821236, EPI_ISL_1821237, EPI_ISL_1821239, EPI_ISL_1821240, EPI_ISL_1821241, EPI_ISL_1821242, EPI_ISL_2003152, EPI_ISL_2614626, EPI_ISL_2691096, EPI_ISL_2691097, EPI_ISL_2691100, EPI_ISL_2691101, EPI_ISL_2691102, EPI_ISL_2691103, EPI_ISL_2691104, EPI_ISL_2691105, EPI_ISL_2691106			
see above	Instituto Adolfo Lutz - Regional de Marilia	Instituto Adolfo Lutz, Interdiciplinary Procedures Center, Strategic Laboratory	Caio Vinicius Dias Lopes; Claudia Regina Gonçalves; Claudio Tavares Sacchi; Erica Valessa Ramos Gomes; Karoline Rodrigues Campos; Leonardo Jose Tadeu de Araujo
EPI_ISL_2614614, EPI_ISL_2614615, EPI_ISL_2614616	Instituto Adolfo Lutz - Regional de Presidente Prudente	Instituto Adolfo Lutz, Interdiciplinary Procedures Center, Strategic Laboratory	Caio Vinicius Dias Lopes; Claudia Regina Gonçalves; Claudio Tavares Sacchi; Erica Valessa Ramos Gomes; Karoline Rodrigues Campos; Leonardo Jose Tadeu de Araujo
EPI_ISL_1715148, EPI_ISL_1731581, EPI_ISL_1731582, EPI_ISL_1752645, EPI_ISL_1821246, EPI_ISL_1821247, EPI_ISL_1821248, EPI_ISL_1821249, EPI_ISL_1821250, EPI_ISL_1821251, EPI_ISL_1821252, EPI_ISL_1821253, EPI_ISL_1821254, EPI_ISL_1821255, EPI_ISL_1821256, EPI_ISL_1821258, EPI_ISL_1821259, EPI_ISL_1821260, EPI_ISL_1821261, EPI_ISL_1821262, EPI_ISL_1821263, EPI_ISL_1821264, EPI_ISL_1821265			
see above	Instituto Adolfo Lutz - Regional de Ribeirao Preto	Instituto Adolfo Lutz, Interdiciplinary Procedures Center, Strategic Laboratory	Caio Vinicius Dias Lopes; Claudia Regina Gonçalves; Claudio Tavares Sacchi; Erica Valessa Ramos Gomes; Karoline Rodrigues Campos; Katia Correa de Oliveira Santos; Leonardo Jose Tadeu de Araujo
EPI_ISL_1625978, EPI_ISL_1625986, EPI_ISL_1625987, EPI_ISL_1625988, EPI_ISL_1625989, EPI_ISL_1625992, EPI_ISL_1625993, EPI_ISL_1625994, EPI_ISL_1625995, EPI_ISL_1625997, EPI_ISL_1625998, EPI_ISL_1625999, EPI_ISL_1626001, EPI_ISL_1626002, EPI_ISL_1626003, EPI_ISL_1626004, EPI_ISL_1626006, EPI_ISL_1626606			
see above	Instituto Adolfo Lutz - Regional de Rio Claro	Instituto Adolfo Lutz, Interdiciplinary Procedures Center, Strategic Laboratory	Caio Vinicius Dias Lopes; Claudia Regina Gonçalves; Claudio Tavares Sacchi; Erica Valessa Ramos Gomes; Karoline Rodrigues Campos; Katia Correa de Oliveira Santos; Leonardo Jose Tadeu de Araujo
EPI_ISL_2003135, EPI_ISL_2003137, EPI_ISL_2003138, EPI_ISL_2003139, EPI_ISL_2003140, EPI_ISL_2003142, EPI_ISL_2003143, EPI_ISL_2003145, EPI_ISL_2003146, EPI_ISL_2003147, EPI_ISL_2003148			
see above	Instituto Adolfo Lutz - Regional de Santos	Instituto Adolfo Lutz, Interdiciplinary Procedures Center, Strategic Laboratory	Caio Vinicius Dias Lopes; Claudia Regina Gonçalves; Claudio Tavares Sacchi; Erica Valessa Ramos Gomes; Karoline Rodrigues Campos; Leonardo Jose Tadeu de Araujo
EPI_ISL_2958842	Instituto Adolfo Lutz - Regional de Sorocaba	Instituto Adolfo Lutz, Interdiciplinary Procedures Center, Strategic Laboratory	Caio Vinicius Dias Lopes; Claudia Regina Gonçalves; Claudio Tavares Sacchi; Erica Valessa Ramos Gomes; Karoline Rodrigues Campos
EPI_ISL_1628347, EPI_ISL_1628350, EPI_ISL_1628351, EPI_ISL_1628354, EPI_ISL_1628355, EPI_ISL_1628357, EPI_ISL_1628358, EPI_ISL_1628360, EPI_ISL_1628361, EPI_ISL_1628362, EPI_ISL_1715150, EPI_ISL_1715151, EPI_ISL_1715152, EPI_ISL_1715153, EPI_ISL_1715154, EPI_ISL_1715155, EPI_ISL_1715156, EPI_ISL_1715157, EPI_ISL_1715158, EPI_ISL_1731583, EPI_ISL_1731584, EPI_ISL_1731586, EPI_ISL_1731588, EPI_ISL_1731589, EPI_ISL_1731590, EPI_ISL_1731592, EPI_ISL_1731597, EPI_ISL_1731599, EPI_ISL_1731600, EPI_ISL_1731601, EPI_ISL_1731603, EPI_ISL_1731604, EPI_ISL_1731605, EPI_ISL_1731607, EPI_ISL_1731608, EPI_ISL_1731609, EPI_ISL_1731610, EPI_ISL_1731611, EPI_ISL_1731612, EPI_ISL_1752647, EPI_ISL_1752651, EPI_ISL_1752652, EPI_ISL_1752654, EPI_ISL_1752655, EPI_ISL_1752658, EPI_ISL_1752660, EPI_ISL_1752662, EPI_ISL_1752665, EPI_ISL_1752666, EPI_ISL_1752667, EPI_ISL_1752668, EPI_ISL_1821267, EPI_ISL_1821268, EPI_ISL_1821269, EPI_ISL_1821271, EPI_ISL_1821272, EPI_ISL_2003114, EPI_ISL_2003115, EPI_ISL_2003116, EPI_ISL_2003117, EPI_ISL_2003118, EPI_ISL_2003119, EPI_ISL_2003121, EPI_ISL_2003125, EPI_ISL_2003126, EPI_ISL_2003127, EPI_ISL_2003128, EPI_ISL_2003129, EPI_ISL_2003130, EPI_ISL_2003132, EPI_ISL_2756432, EPI_ISL_2756433, EPI_ISL_2756434, EPI_ISL_2756435, EPI_ISL_2756436, EPI_ISL_2756440, EPI_ISL_2756441, EPI_ISL_2756443, EPI_ISL_2756445, EPI_ISL_2756446, EPI_ISL_2756447, EPI_ISL_2756449, EPI_ISL_2756454, EPI_ISL_2756455, EPI_ISL_2756456, EPI_ISL_2756457, EPI_ISL_2756459, EPI_ISL_2756460, EPI_ISL_2756461, EPI_ISL_2756462, EPI_ISL_2756463, EPI_ISL_2756464, EPI_ISL_2756465, EPI_ISL_2756466, EPI_ISL_2756467, EPI_ISL_2756468, EPI_ISL_2756469, EPI_ISL_2756470, EPI_ISL_2756471, EPI_ISL_2756472, EPI_ISL_2756473, EPI_ISL_2756474, EPI_ISL_2756475, EPI_ISL_2756476, EPI_ISL_2756479, EPI_ISL_2756480, EPI_ISL_2756482, EPI_ISL_2756485, EPI_ISL_2756486, EPI_ISL_2756488, EPI_ISL_2756489, EPI_ISL_2756490, EPI_ISL_2756491, EPI_ISL_2756492, EPI_ISL_2756493			
see above	Instituto Adolfo Lutz Central	Instituto Adolfo Lutz, Interdiciplinary Procedures Center, Strategic Laboratory	Caio Vinicius Dias Lopes; Claudia Regina Gonçalves; Claudio Tavares Sacchi; Erica Valessa Ramos Gomes; Karoline Rodrigues Campos; Katia Correa de Oliveira Santos; Leonardo Jose Tadeu de Araujo
EPI_ISL_2614602, EPI_ISL_2614603, EPI_ISL_2614604, EPI_ISL_2614605, EPI_ISL_2614606, EPI_ISL_2614608, EPI_ISL_2614609, EPI_ISL_2614610, EPI_ISL_2841607, EPI_ISL_2841609, EPI_ISL_2841611, EPI_ISL_2841612, EPI_ISL_2958833			
see above	Instituto Biologico	Instituto Adolfo Lutz, Interdiciplinary Procedures Center, Strategic Laboratory	Caio Vinicius Dias Lopes; Claudia Regina Gonçalves; Claudio Tavares Sacchi; Erica Valessa Ramos Gomes; Karoline Rodrigues Campos; Leonardo Jose Tadeu de Araujo
EPI_ISL_4104674, EPI_ISL_4104678, EPI_ISL_4104680, EPI_ISL_4104682, EPI_ISL_4104686, EPI_ISL_4104687, EPI_ISL_4104689, EPI_ISL_4104690, EPI_ISL_4104694, EPI_ISL_4104698, EPI_ISL_4104699, EPI_ISL_4104701, EPI_ISL_4104703, EPI_ISL_4104708, EPI_ISL_4104717, EPI_ISL_4104718, EPI_ISL_4104722, EPI_ISL_4104724, EPI_ISL_4104728, EPI_ISL_4104729			
see above	Instituto Butantan	Instituto Butantan	Antonio Jorge Martins; Claudia Renata dos Santos Barros; David Schlesinger; Debora Botequio Moretti; Dimas Tadeu Covas; Elaine Cristina Marqueze; Elaine Vieira Santos; Evandra Strazza Rodrigues; Heidge Fukumasu; Jayme Augusto de Souza-Neto; José Salvatore Leister Patané; Luiz Alcantara; Luiz Lehmann Coutinho; Maria Carolina Elias; Maurício Lacerda Nogueira; Rafael dos Santos Bezerra; Raul Machado Neto; Rejane Maria Tommasini Grotto; Ricardo Haddad; Sandra Coccuzzo Sampaio Vessoni; Simone Kashima; Svetoslav Nanev Slavov; Vincent Louis Viala
EPI_ISL_2344420	Instituto Butantan	Instituto de Medicina Tropical de Sao Paulo	Brazil-UK Centre for Arbovirus Discovery Diagnosis Genomics and Epidemiology (CADDE) Genomic Network - Instituto de Medicina Tropical
EPI_ISL_3418671, EPI_ISL_3418673, EPI_ISL_3418676, EPI_ISL_3418678, EPI_ISL_3418679, EPI_ISL_3418680, EPI_ISL_3418681, EPI_ISL_3418682, EPI_ISL_3418683			
see above	Instituto Carlos Chagas - ICC, FIOCRUZ Parana	Instituto Carlos Chagas - ICC, FIOCRUZ Parana	A.A.; A.M.; A.R.; Aguiar; Albrecht, L.; Alves; Avila; Balsanelli, E.; Becker, G.; Blanes, L.; Dallagiovanna, B.; Debur; E.M.; F.K.; F.O.; Faoro, H.; Graef, T.; H.G.; I.N.; L.G.; L.R.; M.M.; M.O.; Marchini; Md.C.; Morello; Nardeli; Oliveira; P.C.; Passetti, F.; Pedrosa; Resende; Riediger; S.C.; Schemberger; Suzukawa; V.A.; Zanette, D.; de Baura; de Souza; dos Santos
EPI_ISL_2493026	Instituto Nacional de Investigação em Saúde	CERI, Centre for Epidemic Response and Innvoation, Stellenbosch University and KRISP, KZN Research Innovation and Sequencing Platform, UKZN.	Afonso P; David K; Emmanuel SJ; Freitas RH; Giandhari J; Inglês L; Lutucuta S; Miranda J; Morais J; Mufinda M; Naidoo Y; Neto Z; Paulo A Carralero RR Paixão JP; Pereira A; Pillay S; Tegally H; Wilkinson E; de Oliveira T
EPI_ISL_1824485, EPI_ISL_2160668	Instituto Nacional de Medicina Genomica	Centro de Investigación en Enfermedades Infecciosas (CIENI), Instituto Nacional de Enfermedades Respiratorias (INER)	Arriaga-Canon C; Avila-Rios S; Boukadida C; Cedro-Tanda A; Herrera-Montalvo LA; Hidalgo-Miranda A; Matias-Florentino M; Mendoza-Vargas A; Perez-Garcia M; Reyes-Grajeda JP; Reyes-Teran G
EPI_ISL_1585916	Instituto Nacional de Medicina Genomica	Instituto Nacional de Medicina Genomica	Alcaraz N; Canseco Mendez JC; Cedro-Tanda A; Garcia-Cardenas FJ; Gonzalez-Barrera D; Gonzalez-Woge MA; Herrera-Montalvo LA; Hidalgo-Miranda A; Mendoza-Vargas A; Miranda-Ortiz H; Munguia-Garza P; Ramirez-Vega O; Rangel-DeLeon D; Reyes-Grajeda JP; Rosas-Escobar P
EPI_ISL_956287, EPI_ISL_956289, EPI_ISL_956297	Instituto Nacional de Salud- Dirección de Redes de Laboratorios de Salud Pública	Instituto Nacional de Salud- Dirección de Investigación en Salud Pública	Carlos Franco-Muñoz; Diego A. Álvarez-Díaz; Diego Andrés Prada; Gerardo Santamaría; Hector Alejandro Ruiz-Moreno; Jhonnatan Reales-González; Julian Naizaque; Katherine Laiton-Donato; Magdalena Wiesner; Marcela Mercado-Reyes; Maria T. Herrera-Sepúlveda; Martha Lucia Ospina Martinez; Mauricio Pacheco-Montealegre; Sheryll Corchuelo
EPI_ISL_1854230, EPI_ISL_1854231	Instituto Nacional de Saude (INSA) and Instituto Gulbenkian de Ciencia (IGC)	Instituto Nacional de Saude (INSA) and Instituto Gulbenkian de Ciencia (IGC)	Borges et al
EPI_ISL_2004134, EPI_ISL_2004192	Instituto Nacional de Saude (INSA) and i3S - Instituto de Investigação e Inovação em Saúde	Instituto Nacional de Saude (INSA) and i3S - Instituto de Investigação e Inovação em Saúde	Borges et al
EPI_ISL_2758988, EPI_ISL_2758992, EPI_ISL_2758994, EPI_ISL_2758995, EPI_ISL_2758997, EPI_ISL_2759001, EPI_ISL_2759002, EPI_ISL_2759004, EPI_ISL_2759006, EPI_ISL_2759011, EPI_ISL_2759012, EPI_ISL_2759016, EPI_ISL_2759017, EPI_ISL_2759020, EPI_ISL_2759021, EPI_ISL_2759022, EPI_ISL_2759024, EPI_ISL_2759025, EPI_ISL_2759026, EPI_ISL_2759028, EPI_ISL_2759033, EPI_ISL_2759035, EPI_ISL_2759037, EPI_ISL_2759041, EPI_ISL_2759045, EPI_ISL_2759046, EPI_ISL_2759047, EPI_ISL_2759050, EPI_ISL_2759051, EPI_ISL_2759052, EPI_ISL_2759054, EPI_ISL_2759056, EPI_ISL_2759057, EPI_ISL_2759059, EPI_ISL_2759061, EPI_ISL_2759063, EPI_ISL_2759065, EPI_ISL_2759066, EPI_ISL_2759068			
see above	Instituto de Biologia Molecular do Paraná (LAC)	Instituto Carlos Chagas - Fiocruz	Alessandra De Melo Aguiar; Andreia Akemi Suzukawa; Andréa Rodrigues Ávila; Bruno Dallagiovanna; Dalila Zanette; Eduardo Balsanelli; Emanuel Maltempi de Souza; Fabio Passetti; Fabricio Klerynton Marchini; Fábio de Oliveira Pedrosa; Guilherme Becker; Helisson Faoro; Hellen Geremias dos Santos; Irina Nastassja Riediger; Letusa Albrecht; Lucas Blanes; Luis Gustavo Morello; Lysangela Ronalte Alves; Maria do Carmo Debur; Mauro de Medeiros Oliveira; Michelle Orane Schemberger; Paola Cristina Resende; Sheila Cristina Nardeli; Tiago Gräf; Valter Antônio de Baura
EPI_ISL_1000671, EPI_ISL_1000673, EPI_ISL_1734843, EPI_ISL_1734844, EPI_ISL_1734852, EPI_ISL_1734858, EPI_ISL_1734866, EPI_ISL_1734872, EPI_ISL_1734874, EPI_ISL_1734883, EPI_ISL_1911958, EPI_ISL_1911960, EPI_ISL_1911961, EPI_ISL_1911962, EPI_ISL_1911964, EPI_ISL_2223443, EPI_ISL_2223446, EPI_ISL_2223452, EPI_ISL_2295388, EPI_ISL_2295389, EPI_ISL_2295396, EPI_ISL_2295399, EPI_ISL_2295403, EPI_ISL_2295404, EPI_ISL_2295405, EPI_ISL_2295406, EPI_ISL_2295408, EPI_ISL_2295409, EPI_ISL_2295410, EPI_ISL_2295413, EPI_ISL_2295414, EPI_ISL_2295416, EPI_ISL_2308609, EPI_ISL_3118795, EPI_ISL_3118796, EPI_ISL_3118797, EPI_ISL_3118804, EPI_ISL_3118809, EPI_ISL_3118811, EPI_ISL_3118813			
see above	Instituto de Biotecnologia - UNESP-Botucatu-SP	Instituto de Biotecnologia - UNESP-Botucatu-SP	Camila Dantas Malossi; Cecília Artico Banho; Cíntia Bittar; Fábio Sossai Possebon; Guilherme Campos; Helena Lage Ferreira; Jorge A. Petroli Marchesi; João Pessoa Araújo Jr.; Leila Sabrina Ullmann; Lívia Sacchetto; Maisa C. Pereira Parra; Marília Moraes; Maurício L. Nogueira; Paula Rahal; Paulo Inacio da Costa
EPI_ISL_2776166, EPI_ISL_2776171, EPI_ISL_2776172, EPI_ISL_2776174,	Instituto de Medicina Tropical & Salud Global (IMTSAG)	Grubaugh Lab - Yale School of Public Health	Alejandaro Vallejo Degaudenzi; Anderson Brito; Annie Watkins; Chaney Kalinich; Chantal Vogels; Elisa Contreras; Esperanza Mendoza; Isabel Ott; Jessica Rothman; Joseph Fauver; Kendall Billig; Mallery Breban; Mary Petrone; Nathan Grubaugh; Robert Paulino-Ramirez; Tara Alpert; Tobias Koch; Victor Virgilio Calderon

EPI_ISL_2776178, EPI_ISL_2776179			
EPI_ISL_2037442	Instituto de Virologia "Dr. J. M. Vanella", Facultad de Ciencias Médicas, Universidad Nacional de Córdoba.	Centro de Investigaciones Agropecuarias (CIAP), Instituto Nacional de Tecnologia Agropecuaria (INTA) Cordoba, Argentina, on behalf of 'Proyecto Argentino Interinstitucional de Genomica de SARS-CoV-2 (PAIS Consortium)	Adrian Diaz; Brenda Konigheim; Franco Fernandez; Gabriela Barbas; Gonzalo Castro; Humberto Debat; Javier Aguilar; Lorena Spinsanti; Maria Belen Pisano; Maria Elisa Rivarola; Mauricio Beranek; Nathalie Marquez; Sandra Gallego.; Sebastian Blanco; Viviana Re
EPI_ISL_2691149, EPI_ISL_2691150	Intituto Adolfo Lutz - Regional de Sorocaba	Instituto Adolfo Lutz, Interdiciplinary Procedures Center, Strategic Laboratory	Caio Vinicius Dias Lopes; Claudia Regina Gonçalves; Claudio Tavares Sacchi; Erica Valessa Ramos Gomes; Karoline Rodrigues Campos; Leonardo Jose Tadeu de Araujo
EPI_ISL_1061413	Istanbul University-Cerrahpasa, Cerrahpasa School of Medicine, COVID-19 Laboratory	Istanbul University-Cerrahpasa, Cerrahpasa School of Medicine, COVID-19 Laboratory	Ebru Yucebag; Fusun Can; Haluk Eraksoy; Kenan Midilli; Mert Ahmet Kuskucu; Okan Kadir Nohut; Serap Şimşek Yavuz; Yesim Tuyji Tok; Zarifa Abullayeva
EPI_ISL_1854242, EPI_ISL_1854243	Joaquim Chaves Saude	Instituto Nacional de Saude (INSA)	Borges et al
EPI_ISL_1739011, EPI_ISL_1739012, EPI_ISL_1739013, EPI_ISL_1739041	Joaquim Chaves Saude	Instituto Nacional de Saude (INSA) and Institute of Biomedicine (iBiMed), Universidade de Aveiro	Borges et al
EPI_ISL_2246720, EPI_ISL_2246721, EPI_ISL_2246725, EPI_ISL_2246726, EPI_ISL_2246728, EPI_ISL_2246731	John H Stroger Jr Hospital of Cook County	RIPHL at Rush University Medical Center	Ah-Young Sung; Felix Araujo Perez; Kevin Kunstman; Laura Furtado; Marieta Hyde; Max Kolton; Sharon Welbel; Stefan Green
EPI_ISL_1973456, EPI_ISL_1973458	Johns Hopkins Hospital Department of Pathology	Johns Hopkins Hospital Department of Pathology	Adannaya Amadi; C. Paul Morris; Chun Huai Luo; Heba H. Mostafa; Matthew Schwartz; Nicholas Gallagher
EPI_ISL_1312267, EPI_ISL_2404238, EPI_ISL_2404654, EPI_ISL_2425143, EPI_ISL_2425168, EPI_ISL_2425189, EPI_ISL_2425221, EPI_ISL_2832285, EPI_ISL_2832900			
see above	KU Leuven, Rega Institute, Clinical and Epidemiological Virology	KU Leuven, Rega Institute, Clinical and Epidemiological Virology	Bert Vanmechelen; Joan Marti-Carerras; Piet Maes; Tony Wawina-Bokalanga
EPI_ISL_1823805, EPI_ISL_1823809	LA Office of Public Health Laboratories	Centers for Disease Control and Prevention Division of Viral Diseases, Pathogen Discovery	Alison Laufer Halpin; Ben L. Rambo-Martin; Clinton R. Paden; Dakota Howard; Darlene Wagner; Dave Wentworth; Dhwani Batra; Jasmine Padilla; Justin Lee; Katie Dillon; Krista Queen; Kristen Knipe; Kristine Lacek; Mark Burroughs; Matthew Schmerer; Mili Sheth; Peter Cook; Sam Shepard; Sarah Nobles; Shoshona Le; Suxiang Tong; Vivien Dugan; Yvette Unoarumhi
EPI_ISL_1788362, EPI_ISL_2142664	LABORATOIRE DE BIOLOGIE MEDICALE	CNR Virus des Infections Respiratoires - France SUD	Antonin Bal; Bruno Lina; Gregory Destras; Gwendolyne Burfin; Hadrien Regue; Laurence Josset; Martine Valette; Quentin Semanas
EPI_ISL_3102494, EPI_ISL_3102526	LABORATORIO CLEMENTINO FRAGA	Analytical Competence Molecular Epidemiology Lab/ACME, Oswaldo Cruz Foundation, Ceara (FIOCRUZ CE)	Cleber Furtado Aksenen; Fabio Miyajima; Fernando Braga Stehling; Francisco Eder de Moura Lopes; Jamille Maria Mendes Bezerra; Joaquim César do Nascimento Sousa Junior; Pedro Miguel Carneiro Jeronimo; Suzana Porto Almeida e Lucas Delerino; Thais Ferreira de Oliveira; Thais de Oliveira Costa; Ticiane Cavalcante de Souza; Veridiana Pessoa Miyajima
EPI_ISL_1795303, EPI_ISL_1795306, EPI_ISL_1795308, EPI_ISL_1795309, EPI_ISL_1795310, EPI_ISL_1795311, EPI_ISL_1795313, EPI_ISL_1795316, EPI_ISL_1795317, EPI_ISL_1795319, EPI_ISL_1795320, EPI_ISL_1795322, EPI_ISL_1795323, EPI_ISL_2344795, EPI_ISL_2344796, EPI_ISL_2344828, EPI_ISL_2344836, EPI_ISL_2344840, EPI_ISL_2344841, EPI_ISL_2344842, EPI_ISL_2344843, EPI_ISL_2345549, EPI_ISL_2345554, EPI_ISL_2345557, EPI_ISL_2345558, EPI_ISL_2345559, EPI_ISL_2345560, EPI_ISL_2345563, EPI_ISL_2345568, EPI_ISL_2345569, EPI_ISL_2345570, EPI_ISL_2345576, EPI_ISL_2345578, EPI_ISL_2345582, EPI_ISL_2345584			
see above	LABORATORIO DE FRANCA	Instituto Butantan / ESALQ-Piracicaba	Antonio Jorge Martins; Bianca Cechetto Carlos. Mendelics: Bibiana Santos; Claudia Renata dos Santos Barros; David Schlesinger; David Schlesinger. Hemocentro Ribeirão Preto: Simone Kashima; Debora Botequio Moretti; Debora Botequio Moretti. Centro de Genômica Funcional da ESALQ: Luiz Lehmann Coutinho; Dimas Tadeu Covas; Elaine Cristina Marqueze; Elaine Vieira Santos; Elaine Vieira dos Santos; Elisangela Chicaroni Mattos; Erika Freitas; Evandra Strazza Rodrigues; Felipe Allan da Silva da Costa; Flavia Aburjaile; Guilherme Targino Valente; Heidge Fukumasu; Heidge Fukumasu. USP-Botucatu: Rejane Maria Tommasini Grotto; Instituto Butantan: Alexander Roberto Precioso; Jayme A. Souza-Neto; Jayme Augusto de Souza-Neto; Jessika Cristina Chagas Lesbon; José Salvatore Leister Patané; João Paulo Kitajima; Luiz Alcantara; Luiz Carlos Junior de Alcantara; Luiz Lehmann Coutinho; Maria Carolina Elias; Marta Giovanetti; Maurício Lacerda Nogueira; Patricia Akemi Assato; Rafael dos Santos Bezerra; Raquel de Lello Rocha Campos Cassano. NGS Soluções Genômicas: Pilar Drummond Sampaio Corrêa Mariani. FZEA-USP Pirassununga: Mirele Daiana Poleti; Raul Machado Neto; Rejane Maria Tommasini Grotto; Ricardo Augusto Brassaloti; Ricardo Haddad; Rodrigo Tocantins Calado.; Sandra Coccuzzo Sampaio; Sandra Coccuzzo Sampaio Vessoni; Simone Kashima; Svetoslav Nanev Slavov; Vagner Fonseca; Vincent Louis Viala
EPI_ISL_2344583	LABORATORIO DE FRANCA	Instituto Butantan / FZEA-USP-Pirassununga	Antonio Jorge Martins; Claudia Renata dos Santos Barros; David Schlesinger; Debora Botequio Moretti; Dimas Tadeu Covas; Elaine Cristina Marqueze; Elaine Vieira Santos; Evandra Strazza Rodrigues; Heidge Fukumasu; Jayme Augusto de Souza-Neto; José Salvatore Leister Patané; Luiz Alcantara; Luiz Lehmann Coutinho; Maria Carolina Elias; Maurício Lacerda Nogueira; Rafael dos Santos Bezerra; Raul Machado Neto; Rejane Maria Tommasini Grotto; Ricardo Haddad; Sandra Coccuzzo Sampaio Vessoni; Simone Kashima; Svetoslav Nanev Slavov; Vincent Louis Viala
EPI_ISL_1795346, EPI_ISL_1795347, EPI_ISL_1795348, EPI_ISL_1795349, EPI_ISL_1795350, EPI_ISL_1795351, EPI_ISL_1795352, EPI_ISL_1795353, EPI_ISL_1795354, EPI_ISL_1795355, EPI_ISL_1795356, EPI_ISL_1795358, EPI_ISL_1795359, EPI_ISL_1795365, EPI_ISL_1795366, EPI_ISL_2345613, EPI_ISL_2345615, EPI_ISL_2345616, EPI_ISL_2345617, EPI_ISL_2345618, EPI_ISL_2345619, EPI_ISL_2345621, EPI_ISL_2345622, EPI_ISL_2345627, EPI_ISL_2345628, EPI_ISL_2345629, EPI_ISL_2345633, EPI_ISL_2345634, EPI_ISL_2345640, EPI_ISL_2345641			
see above	LABORATORIO DR PAULO EMILIO DALESSANDRO PINDAMONHANGABA	Instituto Butantan / ESALQ-Piracicaba	Antonio Jorge Martins; Bianca Cechetto Carlos. Mendelics: Bibiana Santos; Claudia Renata dos Santos Barros; David Schlesinger; David Schlesinger. Hemocentro Ribeirão Preto: Simone Kashima; Debora Botequio Moretti; Debora Botequio Moretti. Centro de Genômica Funcional da ESALQ: Luiz Lehmann Coutinho; Dimas Tadeu Covas; Elaine Cristina Marqueze; Elaine Vieira Santos; Elaine Vieira dos Santos; Elisangela Chicaroni Mattos; Erika Freitas; Evandra Strazza Rodrigues; Felipe Allan da Silva da Costa; Flavia Aburjaile; Guilherme Targino Valente; Heidge Fukumasu; Heidge Fukumasu. USP-Botucatu: Rejane Maria Tommasini Grotto; Instituto Butantan: Alexander Roberto Precioso; Jayme A. Souza-Neto; Jayme Augusto de Souza-Neto; Jessika Cristina Chagas Lesbon; José Salvatore Leister Patané; João Paulo Kitajima; Luiz Alcantara; Luiz Carlos Junior de Alcantara; Luiz Lehmann Coutinho; Maria Carolina Elias; Marta Giovanetti; Maurício Lacerda Nogueira; Patricia Akemi Assato; Rafael dos Santos Bezerra; Raquel de Lello Rocha Campos Cassano. NGS Soluções Genômicas: Pilar Drummond Sampaio Corrêa Mariani. FZEA-USP Pirassununga: Mirele Daiana Poleti; Raul Machado Neto; Rejane Maria Tommasini Grotto; Ricardo Augusto Brassaloti; Ricardo Haddad; Rodrigo Tocantins Calado.; Sandra Coccuzzo Sampaio; Sandra Coccuzzo Sampaio Vessoni; Simone Kashima; Svetoslav Nanev Slavov; Vagner Fonseca; Vincent Louis Viala
EPI_ISL_2345653, EPI_ISL_2345658, EPI_ISL_2345698, EPI_ISL_2362241, EPI_ISL_2362242, EPI_ISL_2362253, EPI_ISL_2362263, EPI_ISL_2362274			
see above	LABORATORIO DR PAULO EMILIO DALESSANDRO PINDAMONHANGABA	Instituto Butantan / Mendelics	Antonio Jorge Martins; Claudia Renata dos Santos Barros; David Schlesinger; Debora Botequio Moretti; Dimas Tadeu Covas; Elaine Cristina Marqueze; Elaine Vieira Santos; Evandra Strazza Rodrigues; Heidge Fukumasu; Jayme Augusto de Souza-Neto; José Salvatore Leister Patané; Luiz Alcantara; Luiz Lehmann Coutinho; Maria Carolina Elias; Maurício Lacerda Nogueira; Rafael dos Santos Bezerra; Raul Machado Neto; Rejane Maria Tommasini Grotto; Ricardo Haddad; Sandra Coccuzzo Sampaio Vessoni; Simone Kashima; Svetoslav Nanev Slavov; Vincent Louis Viala
EPI_ISL_2209338, EPI_ISL_2209345	LABORATORIO LOCAL DE ITAPECERICA DA SERRA	Instituto Butantan	Antonio Jorge Martins; Claudia Renata dos Santos Barros; David Schlesinger; Debora Botequio Moretti; Dimas Tadeu Covas; Elaine Cristina Marqueze; Elaine Vieira Santos; Evandra Strazza Rodrigues; Heidge Fukumasu; Jayme Augusto de Souza-Neto; José Salvatore Leister Patané; Luiz Alcantara; Luiz Lehmann Coutinho; Maria Carolina Elias; Maurício Lacerda Nogueira; Rafael dos Santos Bezerra; Raul Machado Neto; Rejane Maria Tommasini Grotto; Ricardo Haddad; Sandra Coccuzzo Sampaio Vessoni; Simone Kashima; Svetoslav Nanev Slavov; Vincent Louis Viala.
EPI_ISL_2345891, EPI_ISL_2345895, EPI_ISL_2345896, EPI_ISL_2345898	LABORATORIO LOCAL DE ITAPECERICA DA SERRA	Instituto Butantan / FZEA-USP-Pirassununga	Antonio Jorge Martins; Claudia Renata dos Santos Barros; David Schlesinger; Debora Botequio Moretti; Dimas Tadeu Covas; Elaine Cristina Marqueze; Elaine Vieira Santos; Evandra Strazza Rodrigues; Heidge Fukumasu; Jayme Augusto de Souza-Neto; José Salvatore Leister Patané; Luiz Alcantara; Luiz Lehmann Coutinho; Maria Carolina Elias; Maurício Lacerda Nogueira; Rafael dos Santos Bezerra; Raul Machado Neto; Rejane Maria Tommasini Grotto; Ricardo Haddad; Sandra Coccuzzo Sampaio Vessoni; Simone Kashima; Svetoslav Nanev Slavov; Vincent Louis Viala
EPI_ISL_3102475, EPI_ISL_3102477, EPI_ISL_3102543	LABORATORIO MUN ANALISES CLINICAS	Analytical Competence Molecular Epidemiology Lab/ACME, Oswaldo Cruz Foundation, Ceara (FIOCRUZ CE)	Cleber Furtado Aksenen; Fabio Miyajima; Fernando Braga Stehling; Francisco Eder de Moura Lopes; Jamille Maria Mendes Bezerra; Joaquim César do Nascimento Sousa Junior; Pedro Miguel Carneiro Jeronimo; Suzana Porto Almeida e Lucas Delerino; Thais Ferreira de Oliveira; Thais de Oliveira Costa; Ticiane Cavalcante de Souza; Veridiana Pessoa Miyajima
EPI_ISL_1966611	LABORATORIO MUNICIPAL DE ANALISES CLINICAS DE ITANHAEM	Instituto Butantan / Mendelics	Antonio Jorge Martins; Bianca Cechetto Carlos. Mendelics: Bibiana Santos; Claudia Renata dos Santos Barros; Cíntia Bittar; David Schlesinger. Hemocentro Ribeirão Preto: Simone Kashima; Debora Botequio Moretti; Elaine Cristina Marqueze; Elaine Vieira dos Santos; Elisangela Chicaroni Mattos; Erika Freitas; Evandra Strazza Rodrigues; Felipe Allan da Silva da Costa; Flavia Aburjaile; Fábio Sossai Possebon; Guilherme Campos; Guilherme Targino Valente; Heidge Fukumasu. USP-Botucatu: Rejane Maria Tommasini Grotto; Helena Lage Ferreira; Instituto Butantan: Dimas Tadeu Covas; Jardelina de Souza Todao Bernardino; Jayme A. Souza-Neto; Jessika Cristina Chagas Lesbon; Jorge A. Petroli Marchesi; José Salvatore Leister Patané; João Paulo Kitajima; João Pessoa Araújo Jr.; Leila Sabrina Ullmann; Loyze Paola Oliveira de Lima; Luiz Aurelio de Campos Crispin. Centro de Genômica Funcional da ESALQ: Luiz Lehmann Coutinho; Luiz Carlos Junior de Alcantara; Lívia Sacchetto; Maisa C. Pereira Parra; Maria Carolina Elias; Marta Giovanetti; Marília Moraes; Maurício Lacerda Nogueira. Prefeitura de Sao Paulo: Melissa Palmieri.; Patricia Akemi Assato; Paula Rahal; Paulo Inacio da Costa; Rafael dos Santos Bezerra; Raquel de Lello Rocha Campos Cassano. NGS Soluções Genômicas: Pilar Drummond Sampaio Corrêa Mariani. FZEA-USP Pirassununga: Mirele Daiana Poleti; Raul Machado Neto; Ricardo Augusto Brassaloti; Ricardo Haddad; Rodrigo Tocantins Calado. FAMERP-SJRP: Cecília Artico Banho; Sandra Coccuzzo Sampaio; Svetoslav Nanev Slavov; Vagner Fonseca; Vincent Louis Viala
EPI_ISL_2209360	LABORATORIO MUNICIPAL DE	Instituto Butantan	Antonio Jorge Martins; Claudia Renata dos Santos Barros; David Schlesinger; Debora Botequio Moretti; Dimas Tadeu Covas; Elaine Cristina Marqueze; Elaine Vieira Santos; Evandra Strazza Rodrigues; Heidge Fukumasu; Jayme Augusto de Souza-Neto; José Salvatore Leister Patané; Luiz Alcantara; Luiz Lehmann Coutinho; Maria Carolina Elias; Maurício Lacerda Nogueira; Rafael dos Santos Bezerra; Raul Machado Neto; Rejane Maria Tommasini Grotto; Ricardo Haddad; Sandra Coccuzzo Sampaio Vessoni; Simone Kashima; Svetoslav Nanev Slavov; Vincent Louis Viala.

	ANALISES CLINICAS DE RIO CLARO		
EPI_ISL_2209122, EPI_ISL_2209252, EPI_ISL_2209278, EPI_ISL_2210088, EPI_ISL_2344728, EPI_ISL_2344729	LABORATORIO MUNICIPAL DE ANALISES CLINICAS DE RIO CLARO	Instituto Butantan / FZEA-USP-Pirassununga	Antonio Jorge Martins; Claudia Renata dos Santos Barros; David Schlesinger; Debora Botequio Moretti; Dimas Tadeu Covas; Elaine Cristina Marqueze; Elaine Vieira Santos; Evandra Strazza Rodrigues; Heidge Fukumasu; Jayme Augusto de Souza-Neto; José Salvatore Leister Patané; Luiz Alcantara; Luiz Lehmann Coutinho; Maria Carolina Elias; Maurício Lacerda Nogueira; Rafael dos Santos Bezerra; Raul Machado Neto; Rejane Maria Tommasini Grotto; Ricardo Haddad; Sandra Coccuzzo Sampaio Vessoni; Simone Kashima; Svetoslav Nanev Slavov; Vincent Louis Viala
EPI_ISL_1795100, EPI_ISL_2344549, EPI_ISL_2344734, EPI_ISL_2344801, EPI_ISL_2344826, EPI_ISL_2344884	LABORATORIO MUNICIPAL DE PIRACICABA	Instituto Butantan / ESALQ-Piracicaba	Antonio Jorge Martins; Bianca Cechetto Carlos. Mendelics: Bibiana Santos; Claudia Renata dos Santos Barros; David Schlesinger; David Schlesinger. Hemocentro Ribeirão Preto: Simone Kashima; Debora Botequio Moretti; Debora Botequio Moretti. Centro de Genômica Funcional da ESALQ: Luiz Lehmann Coutinho; Dimas Tadeu Covas; Elaine Cristina Marqueze; Elaine Vieira Santos; Elaine Vieira dos Santos; Elisangela Chicaroni Mattos; Erika Freitas; Evandra Strazza Rodrigues; Felipe Allan da Silva da Costa; Flavia Aburjaile; Guilherme Targino Valente; Heidge Fukumasu; Heidge Fukumasu. USP-Botucatu: Rejane Maria Tommasini Grotto; Instituto Butantan: Alexander Roberto Precioso; Jayme A. Souza-Neto; Jayme Augusto de Souza-Neto; Jessika Cristina Chagas Lesbon; José Salvatore Leister Patané; João Paulo Kitajima; Luiz Alcantara; Luiz Carlos Junior de Alcantara; Luiz Lehmann Coutinho; Maria Carolina Elias; Marta Giovanetti; Maurício Lacerda Nogueira; Patricia Akemi Assato; Rafael dos Santos Bezerra; Raquel de Lello Rocha Campos Cassano. NGS Soluções Genômicas: Pilar Drummond Sampaio Corrêa Mariani. FZEA-USP Pirassununga: Mirele Daiana Poleti; Raul Machado Neto; Rejane Maria Tommasini Grotto; Ricardo Augusto Brassaloti; Ricardo Haddad; Rodrigo Tocantins Calado.; Sandra Coccuzzo Sampaio; Sandra Coccuzzo Sampaio Vessoni; Simone Kashima; Svetoslav Nanev Slavov; Vagner Fonseca; Vincent Louis Viala
EPI_ISL_1966996, EPI_ISL_1966998, EPI_ISL_1966999, EPI_ISL_1967001, EPI_ISL_1967002, EPI_ISL_1967003	LABORATORIO MUNICIPAL DE PIRACICABA	Instituto Butantan / ESALQ-USP (Piracicaba)	Antonio Jorge Martins; Bianca Cechetto Carlos. Mendelics: Bibiana Santos; Claudia Renata dos Santos Barros; Cíntia Bittar; David Schlesinger. Hemocentro Ribeirão Preto: Simone Kashima; Debora Botequio Moretti; Elaine Cristina Marqueze; Elaine Vieira dos Santos; Elisangela Chicaroni Mattos; Erika Freitas; Evandra Strazza Rodrigues; Felipe Allan da Silva da Costa; Flavia Aburjaile; Fábio Sossai Possebon; Guilherme Campos; Guilherme Targino Valente; Heidge Fukumasu. USP-Botucatu: Rejane Maria Tommasini Grotto; Helena Lage Ferreira; Instituto Butantan: Dimas Tadeu Covas; Jardelina de Souza Todao Bernardino; Jayme A. Souza-Neto; Jessika Cristina Chagas Lesbon; Jorge A. Petroli Marchesi; José Salvatore Leister Patané; João Paulo Kitajima; João Pessoa Araújo Jr.; Leila Sabrina Ullmann; Loyze Paola Oliveira de Lima; Luiz Aurelio de Campos Crispin. Centro de Genômica Funcional da ESALQ: Luiz Lehmann Coutinho; Luiz Carlos Junior de Alcantara; Lívia Sacchetto; Maisa C. Pereira Parra; Maria Carolina Elias; Marta Giovanetti; Marília Moraes; Maurício Lacerda Nogueira. Prefeitura de Sao Paulo: Melissa Palmieri.; Patricia Akemi Assato; Paula Rahal; Paulo Inacio da Costa; Rafael dos Santos Bezerra; Raquel de Lello Rocha Campos Cassano. NGS Soluções Genômicas: Pilar Drummond Sampaio Corrêa Mariani. FZEA-USP Pirassununga: Mirele Daiana Poleti; Raul Machado Neto; Ricardo Augusto Brassaloti; Ricardo Haddad; Rodrigo Tocantins Calado. FAMERP-SJRP: Cecília Artico Banho; Sandra Coccuzzo Sampaio; Svetoslav Nanev Slavov; Vagner Fonseca; Vincent Louis Viala
EPI_ISL_1967149, EPI_ISL_1967150, EPI_ISL_1967154, EPI_ISL_1967155	LABORATORIO MUNICIPAL DE SUZANO	Instituto Butantan / ESALQ-USP (Piracicaba)	Antonio Jorge Martins; Bianca Cechetto Carlos. Mendelics: Bibiana Santos; Claudia Renata dos Santos Barros; Cíntia Bittar; David Schlesinger. Hemocentro Ribeirão Preto: Simone Kashima; Debora Botequio Moretti; Elaine Cristina Marqueze; Elaine Vieira dos Santos; Elisangela Chicaroni Mattos; Erika Freitas; Evandra Strazza Rodrigues; Felipe Allan da Silva da Costa; Flavia Aburjaile; Fábio Sossai Possebon; Guilherme Campos; Guilherme Targino Valente; Heidge Fukumasu. USP-Botucatu: Rejane Maria Tommasini Grotto; Helena Lage Ferreira; Instituto Butantan: Dimas Tadeu Covas; Jardelina de Souza Todao Bernardino; Jayme A. Souza-Neto; Jessika Cristina Chagas Lesbon; Jorge A. Petroli Marchesi; José Salvatore Leister Patané; João Paulo Kitajima; João Pessoa Araújo Jr.; Leila Sabrina Ullmann; Loyze Paola Oliveira de Lima; Luiz Aurelio de Campos Crispin. Centro de Genômica Funcional da ESALQ: Luiz Lehmann Coutinho; Luiz Carlos Junior de Alcantara; Lívia Sacchetto; Maisa C. Pereira Parra; Maria Carolina Elias; Marta Giovanetti; Marília Moraes; Maurício Lacerda Nogueira. Prefeitura de Sao Paulo: Melissa Palmieri.; Patricia Akemi Assato; Paula Rahal; Paulo Inacio da Costa; Rafael dos Santos Bezerra; Raquel de Lello Rocha Campos Cassano. NGS Soluções Genômicas: Pilar Drummond Sampaio Corrêa Mariani. FZEA-USP Pirassununga: Mirele Daiana Poleti; Raul Machado Neto; Ricardo Augusto Brassaloti; Ricardo Haddad; Rodrigo Tocantins Calado. FAMERP-SJRP: Cecília Artico Banho; Sandra Coccuzzo Sampaio; Svetoslav Nanev Slavov; Vagner Fonseca; Vincent Louis Viala
EPI_ISL_2801314	LABORATÓRIO SÃO CARLOS	Analytical Competence Molecular Epidemiology Lab/ACME, Oswaldo Cruz Foundation, Ceara (FIOCRUZ CE)	Cleber Furtado Aksenen e Suzana Porto Almeida; Fabio Miyajima; Fernando Braga Stehling; Francisco Eder de Moura Lopes; Jamille Maria Mendes Bezerra; Joaquim César do Nascimento Sousa Junior; Pedro Miguel Carneiro Jeronimo; Thais Ferreira de Oliveira; Thais de Oliveira Costa; Ticiane Cavalcante de Souza; Veridiana Pessoa Miyajima
EPI_ISL_2488779, EPI_ISL_2488787, EPI_ISL_2488788, EPI_ISL_2488789, EPI_ISL_2488790	LACEN - Laboratório Central de Saúde Pública de Pernambuco	Evandro Chagas Institute	A.M.; Barbagelata; E.C.; E.M.A.; Ferreira; J.A.; Junior; K.C.; L.C.; L.S.; M.C.; P.S.; Pinheiro; Santos; Silva; Sousa; Sousa Junior; W.D.C.; da Silva
EPI_ISL_1261687	LACEN - Laboratório Central de Saúde Pública de Roraima	Evandro Chagas Institute	A.M.; Barbagelata; E.C.; E.M.A.; Ferreira; J.A.; Junior; K.C.; L.C.; L.S.; M.C.; P.S.; Pinheiro; Santos; Silva; Sousa; Sousa Junior; W.D.C.; da Silva
EPI_ISL_1164984, EPI_ISL_1261686, EPI_ISL_1261688, EPI_ISL_1261689, EPI_ISL_1261692, EPI_ISL_2488776, EPI_ISL_2488792, EPI_ISL_2488793, EPI_ISL_2488794, EPI_ISL_2488804, EPI_ISL_2488805, EPI_ISL_2488806, EPI_ISL_2488807			
see above	LACEN - Laboratório Central de Saúde Pública do Amapá	Evandro Chagas Institute	A.M.; Barbagelata; E.C.; E.M.A.; Ferreira; J.A.; Junior; K.C.; L.C.; L.S.; M.C.; P.S.; Pinheiro; Santos; Silva; Sousa; Sousa Junior; W.D.C.; da Silva
EPI_ISL_918499, EPI_ISL_918500, EPI_ISL_918501, EPI_ISL_918502, EPI_ISL_918503, EPI_ISL_918504, EPI_ISL_918505, EPI_ISL_918506, EPI_ISL_918507, EPI_ISL_918508, EPI_ISL_918509, EPI_ISL_918510, EPI_ISL_918511, EPI_ISL_1261683, EPI_ISL_1261685, EPI_ISL_1261690, EPI_ISL_1261694, EPI_ISL_2488774, EPI_ISL_2488775			
see above	LACEN - Laboratório Central de Saúde Pública do Amazonas	Evandro Chagas Institute	A.M.; Barbagelata; E.C.; E.M.A.; Ferreira; J.A.; Junior; K.C.; L.C.; L.S.; M.C.; P.S.; Pinheiro; Santos; Silva; Sousa; Sousa Junior; W.D.C.; da Silva
EPI_ISL_1164970, EPI_ISL_1164971, EPI_ISL_1164973, EPI_ISL_1164980, EPI_ISL_1261684, EPI_ISL_1261693, EPI_ISL_2488799, EPI_ISL_2488800, EPI_ISL_2488801, EPI_ISL_2488802, EPI_ISL_2488803			
see above	LACEN - Laboratório Central de Saúde Pública do Ceará	Evandro Chagas Institute	A.M.; Barbagelata; E.C.; E.M.A.; Ferreira; J.A.; Junior; K.C.; L.C.; L.S.; M.C.; P.S.; Pinheiro; Santos; Silva; Sousa; Sousa Junior; W.D.C.; da Silva
EPI_ISL_2488808, EPI_ISL_2488809, EPI_ISL_2488811	LACEN - Laboratório Central de Saúde Pública do Maranhao	Evandro Chagas Institute	A.M.; Barbagelata; E.C.; E.M.A.; Ferreira; J.A.; Junior; K.C.; L.C.; L.S.; M.C.; P.S.; Pinheiro; Santos; Silva; Sousa; Sousa Junior; W.D.C.; da Silva
EPI_ISL_1164991	LACEN - Laboratório Central de Saúde Pública do Paraiba	Evandro Chagas Institute	A.M.; Barbagelata; E.C.; E.M.A.; Ferreira; J.A.; Junior; K.C.; L.C.; L.S.; M.C.; P.S.; Pinheiro; Santos; Silva; Sousa; Sousa Junior; W.D.C.; da Silva
EPI_ISL_904120, EPI_ISL_904121, EPI_ISL_1164972, EPI_ISL_1164978	LACEN - Laboratório Central de Saúde Pública do Pará	Evandro Chagas Institute	A.M.; Barbagelata; E.C.; E.M.A.; Ferreira; J.A.; Junior; K.C.; L.C.; L.S.; M.C.; P.S.; Pinheiro; Santos; Silva; Sousa; Sousa Junior; W.D.C.; da Silva
EPI_ISL_1166615	LACEN - Laboratório Central de Saúde Pública do Rio Grande do Norte	Evandro Chagas Institute Virology	A.M.; Barbagelata; E.C.; E.M.A.; Ferreira; J.A.; Junior; K.C.; L.C.; L.S.; M.C.; P.S.; Pinheiro; Santos; Silva; Sousa; Sousa Junior; W.D.C.; da Silva
EPI_ISL_2488812	LACEN - Laboratório Central de Saúde Pública do Rio Grande do Norte	Evandro Chagas Institute	A.M.; Barbagelata; E.C.; E.M.A.; Ferreira; J.A.; Junior; K.C.; L.C.; L.S.; M.C.; P.S.; Pinheiro; Santos; Silva; Sousa; Sousa Junior; W.D.C.; da Silva
EPI_ISL_2488772, EPI_ISL_2488780, EPI_ISL_2488781, EPI_ISL_2488782, EPI_ISL_2488783, EPI_ISL_2488784, EPI_ISL_2488785, EPI_ISL_2488786			
see above	LACEN - Laboratório Central de Saúde Pública do Roraima	Evandro Chagas Institute	A.M.; Barbagelata; E.C.; E.M.A.; Ferreira; J.A.; Junior; K.C.; L.C.; L.S.; M.C.; P.S.; Pinheiro; Santos; Silva; Sousa; Sousa Junior; W.D.C.; da Silva
EPI_ISL_1293053, EPI_ISL_1293054, EPI_ISL_1293055, EPI_ISL_1303502, EPI_ISL_1303503	LACEN de Rondonia	Instituto Adolfo Lutz, Interdiciplinary Procedures Center, Strategic Laboratory	Caio Vinicius Dias Lopes; Claudia Regina Gonçalves; Claudio Tavares Sacchi; Erica Valessa Ramos Gomes; Karoline Rodrigues Campos
EPI_ISL_985318, EPI_ISL_985319	LACEN de Santa Catarina	Instituto Adolfo Lutz, Interdiciplinary Procedures Center, Strategic Laboratory	Claudia Regina Gonçalves; Claudio Tavares Sacchi; Erica Valessa Ramos Gomes; Karoline Rodrigues Campos
EPI_ISL_1303506	LACEN do Distrito Federal	Instituto Adolfo Lutz, Interdiciplinary Procedures Center, Strategic Laboratory	Caio Vinicius Dias Lopes; Claudia Regina Gonçalves; Claudio Tavares Sacchi; Erica Valessa Ramos Gomes; Karoline Rodrigues Campos
EPI_ISL_943990, EPI_ISL_985303, EPI_ISL_985304, EPI_ISL_985305, EPI_ISL_985306, EPI_ISL_985307, EPI_ISL_985308, EPI_ISL_985309, EPI_ISL_985310, EPI_ISL_985311, EPI_ISL_985312, EPI_ISL_985313, EPI_ISL_985314, EPI_ISL_985315, EPI_ISL_985316, EPI_ISL_985317, EPI_ISL_1039691, EPI_ISL_1039692, EPI_ISL_1039693, EPI_ISL_1039694, EPI_ISL_1039695, EPI_ISL_1041509, EPI_ISL_1303512, EPI_ISL_1303513, EPI_ISL_1303514, EPI_ISL_1303517, EPI_ISL_1468413, EPI_ISL_1468414, EPI_ISL_1468415, EPI_ISL_1493574, EPI_ISL_1493575, EPI_ISL_1493576, EPI_ISL_1493577, EPI_ISL_1628363, EPI_ISL_1628364, EPI_ISL_1628365, EPI_ISL_2614541, EPI_ISL_2614542, EPI_ISL_2691214, EPI_ISL_2691215, EPI_ISL_2828706, EPI_ISL_2919228, EPI_ISL_2919229, EPI_ISL_2919230, EPI_ISL_2919231, EPI_ISL_2919232			
see above	LACEN do Estado de Goias	Instituto Adolfo Lutz, Interdiciplinary Procedures Center, Strategic Laboratory	Caio Vinicius Dias Lopes; Claudia Regina Gonçalves; Claudio Tavares Sacchi; Erica Valessa Ramos Gomes; Karoline Rodrigues Campos; Katia Correa de Oliveira Santos; Leonardo Jose Tadeu de Araujo
EPI_ISL_2958884, EPI_ISL_2958885, EPI_ISL_2958886, EPI_ISL_2958888, EPI_ISL_2958889, EPI_ISL_2958890, EPI_ISL_3316168, EPI_ISL_3316173, EPI_ISL_3316186, EPI_ISL_3316187, EPI_ISL_3316191, EPI_ISL_3316194, EPI_ISL_3316195, EPI_ISL_3316209, EPI_ISL_3316210, EPI_ISL_3316214, EPI_ISL_3316215, EPI_ISL_3316218, EPI_ISL_3316220, EPI_ISL_3316224			
see above	LACEN do Estado de Mato Grosso	Instituto Adolfo Lutz, Interdiciplinary Procedures Center, Strategic Laboratory	Caio Vinicius Dias Lopes; Claudia Regina Gonçalves; Claudio Tavares Sacchi; Erica Valessa Ramos Gomes; Karoline Rodrigues Campos; Leonardo Tadeu de Araujo; Marlon Benedito Nascimento Santos
EPI_ISL_1493578,	LACEN do Estado de	Instituto Adolfo Lutz,	Caio Vinicius Dias Lopes; Claudia Regina Gonçalves; Claudio Tavares Sacchi; Erica Valessa Ramos Gomes; Karoline Rodrigues Campos

EPI_ISL_1493579, EPI_ISL_1494924, EPI_ISL_1520108, EPI_ISL_1520109	Rondonia	Interdiciplinary Procedures Center, Strategic Laboratory	
EPI_ISL_943987, EPI_ISL_1303509, EPI_ISL_2919216, EPI_ISL_2919217, EPI_ISL_2919219, EPI_ISL_2919220, EPI_ISL_2919222, EPI_ISL_2919223, EPI_ISL_2919224, EPI_ISL_2919227			
see above	LACEN do Estado de Tocantins	Instituto Adolfo Lutz, Interdiciplinary Procedures Center, Strategic Laboratory	Caio Vinicius Dias Lopes; Claudia Regina Gonçalves; Claudio Tavares Sacchi; Erica Valessa Ramos Gomes; Karoline Rodrigues Campos
EPI_ISL_3671908, EPI_ISL_3671909, EPI_ISL_3671911, EPI_ISL_3671912, EPI_ISL_3671914, EPI_ISL_3671915, EPI_ISL_3671916, EPI_ISL_3671917, EPI_ISL_3671918, EPI_ISL_3691382, EPI_ISL_3691383, EPI_ISL_3691384, EPI_ISL_3691385, EPI_ISL_3691386, EPI_ISL_3691387, EPI_ISL_3691388, EPI_ISL_3691389, EPI_ISL_3691390, EPI_ISL_3946597			
see above	LACEN do Estado do Mato Grosso do Sul	Instituto Adolfo Lutz, Interdiciplinary Procedures Center, Strategic Laboratory	Caio Vinicius Dias Lopes; Claudia Regina Gonçalves; Claudio Tavares Sacchi; Karoline Rodrigues Campos; Leonardo Tadeu de Araujo; Marlon Benedito Nascomento Santos
EPI_ISL_3691681, EPI_ISL_3691682, EPI_ISL_3691683, EPI_ISL_3691684	LACEN do Estado do Piaui	Instituto Adolfo Lutz, Interdiciplinary Procedures Center, Strategic Laboratory	Caio Vinicius Dias Lopes; Claudia Regina Gonçalves; Claudio Tavares Sacchi; Karoline Rodrigues Campos; Leonardo Tadeu de Araujo; Marlon Benedito Nascomento Santos
EPI_ISL_1358304, EPI_ISL_2761936, EPI_ISL_2958855, EPI_ISL_2958856	LACEN do Mato Grosso do Sul	Instituto Adolfo Lutz, Interdiciplinary Procedures Center, Strategic Laboratory	Caio Vinicius Dias Lopes; Claudia Regina Gonçalves; Claudio Tavares Sacchi; Erica Valessa Ramos Gomes; Karoline Rodrigues Campos; Leonardo Jose Tadeu de Araujo
EPI_ISL_1121316	LACEN do Rio Grande do Sul	Instituto Adolfo Lutz, Interdiciplinary Procedures Center, Strategic Laboratory	Caio Vinicius Dias Lopes; Claudia Regina Gonçalves; Claudio Tavares Sacchi; Erica Valessa Ramos Gomes; Karoline Rodrigues Campos
EPI_ISL_906071, EPI_ISL_940614, EPI_ISL_940615, EPI_ISL_940616, EPI_ISL_940617, EPI_ISL_940618	LACEN-PI DR. Costa Alvarenga	Instituto Adolfo Lutz, Interdiciplinary Procedures Center, Strategic Laboratory	Claudia Regina Gonçalves; Claudio Tavares Sacchi; Erica Valessa Ramos Gomes; Karoline Rodrigues Campos
EPI_ISL_2221885, EPI_ISL_2821263, EPI_ISL_2821265, EPI_ISL_2821268, EPI_ISL_2821269, EPI_ISL_2821270, EPI_ISL_2821271, EPI_ISL_2821273, EPI_ISL_3134631, EPI_ISL_3703554			
see above	LACEN/PE	WallauLab on behalf of Fiocruz COVID-19 Genomic Surveillance Network	Alexandre Freitas da Silva; Antonio Mauro Rezende; Cassia Docena; Constância Flávia Junqueira Ayres; Cássia Docena; Duschinka Ribeiro Duarte Guedes; Elisama Helvecio; Filipe Zimmer Dezordi; Gabriel Luz Wallau; Gustavo Barbosa de Lima; Lais Ceschini Machado; Larissa Krokovsky; Laís Ceschini Machado; Lilian Caroliny Amorim Silva; Marcelo Henrique dos Santos Paiva; Matheus Filgueira Bezerra; Sinval Pinto Brandão Filho
EPI_ISL_1213168	LAFEM/UESC	Bioinformatics Laboratory / LNCC	Alessandra P Lamarca; Alexandra L Gerber; Ana Paula Melo Mariano; Ana Paula de C Guimarães; Ana Tereza R Vasconcelos; Angela Maria Guimarães Santos; Bianca Mendes Maciel; Danielle Angst Secco; Eduardo Sérgio Soares Sousa; Eloiza Helena Campana; Francisco Paulo Freire Neto; George Rego Albuquerque; Kátia Castanho Scortecci; Lucymara Fassarella Agnez Lima; Luiz G P de Almeida; Luís Cristóvão Porto; Otavio J. Brustolini; Paulo Ricardo Nascimento; Ronaldo da Silva Francisco Jr; Sandra Rocha Gadelha; Selma Maria Bezerra Jeronimo; Vinícius Pietta Perez
EPI_ISL_3134973	LATE - Laboratório de Técnicas Especiais - Hospital Israelita Albert Einstein	LATE - Laboratório de Técnicas Especiais - Hospital Israelita Albert Einstein	Alexandre Hideaki Takara; Ana Paula Moreira Salles; Anelisie da Silva Santos; Deyvid Amgarten; Erick Gustavo Dorlass; Fernanda de Mello Malta; João Renato Rebello Pinho; Marcio Anunciacao Menezes; Pedro Henrique Sebe Rodrigues; Raquel Riyuzo
EPI_ISL_1313008	LBA	CNR Virus des Infections Respiratoires - France SUD	Antonin Bal; Bruno Lina; Gregory Destras; Gwendolyne Burfin; Hadrien Regue; Laurence Josset; Martine Valette; Quentin Semanas
EPI_ISL_1608139	LBM ALPHABIO, Marseille	LBM ALPHABIO, Marseille	Vincent GARCIA
EPI_ISL_1213189, EPI_ISL_1213190, EPI_ISL_1213201, EPI_ISL_1213202, EPI_ISL_1213204, EPI_ISL_1213206, EPI_ISL_1213207			
see above	LBM/UFPB	Bioinformatics Laboratory / LNCC	Alessandra P Lamarca; Alexandra L Gerber; Ana Paula Melo Mariano; Ana Paula de C Guimarães; Ana Tereza R Vasconcelos; Angela Maria Guimarães Santos; Bianca Mendes Maciel; Danielle Angst Secco; Eduardo Sérgio Soares Sousa; Eloiza Helena Campana; Francisco Paulo Freire Neto; George Rego Albuquerque; Kátia Castanho Scortecci; Lucymara Fassarella Agnez Lima; Luiz G P de Almeida; Luís Cristóvão Porto; Otavio J. Brustolini; Paulo Ricardo Nascimento; Ronaldo da Silva Francisco Jr; Sandra Rocha Gadelha; Selma Maria Bezerra Jeronimo; Vinícius Pietta Perez
EPI_ISL_1960077, EPI_ISL_1960078	LDSP	Universidad Nacional de Colombia - Laboratorio Genómico One Health	Andres F. Cardona-Rios; Carlos Franco-Muñoz; Carolina Muñoz-Arango; Celeny Ortiz; Daniel O. Maldonado-Perez; Diego A. Álvarez-Díaz; Hector Alejandro Ruiz-Moreno; Idabely Betancur Ortiz; Jorge E. Osorio; Juan P. Hernandez-Ortiz; Karl A Ciuoderis; Katherine Laiton-Donato; Laura Silvana Perez; Lina M. Hurtado; Marcela Mercado-Reyes; Maria Angélica Maya; Maria Stella López; Rita Almanza Payares; Sandra Ines Cano; Simón Villegas Velásquez
EPI_ISL_1805639	LDSP FRONTERIZO	Instituto Nacional de Salud- Dirección de Investigación en Salud Pública	Carlos Franco-Muñoz; Carmen Osorio; Diana Malo; Diego A. Álvarez-Díaz; Diego Andrés Prada; Gerardo Santamaría; Hector Alejandro Ruiz-Moreno; Jhonnatan Reales-González; Jorge Rivera; Juan Camilo Martinez; Julian Naizaque; Katherine Laiton-Donato; Lisseth Pardo; Magdalena Wiesner; Marcela Mercado-Reyes; Maria T. Herrera-Sepúlveda; Marta Lopez Blanco; Martha Lucia Ospina Martinez; Paola Rojas; Sergio Gomez; Sheryll Corchuelo; Ángela Alarcon Cruz
EPI_ISL_1469941, EPI_ISL_2296079, EPI_ISL_2296080	LESP Ciudad de Mexico	Instituto de Diagnostico y Referencia Epidemiologicos (INDRE)	Abril Rodriguez-Maldonado; Ariadna Medina-Benitez; Claudia Wong-Arambula; Ernesto Ramirez-Gonzalez.; Gisela Barrera-Badillo; Irma Lopez-Martinez; Joaquin Quiroz-Mercado; Lucia Hernandez-Rivas; Natividad Cruz-Ortiz; Sergio Rangel-Guerrero; Tatiana Nunez-Garcia; Vanessa Rivero-Arredondo
EPI_ISL_2296082	LESP Estado de Mexico	Instituto de Diagnostico y Referencia Epidemiologicos (INDRE)	Abril Rodriguez-Maldonado; Ariadna Medina-Benitez; Claudia Wong-Arambula; Ernesto Ramirez-Gonzalez.; Gisela Barrera-Badillo; Irma Lopez-Martinez; Joaquin Quiroz-Mercado; Lucia Hernandez-Rivas; Natividad Cruz-Ortiz; Sergio Rangel-Guerrero; Tatiana Nunez-Garcia; Vanessa Rivero-Arredondo
EPI_ISL_2105682, EPI_ISL_2296069	LESP Guanajuato	Instituto de Diagnostico y Referencia Epidemiologicos (INDRE)	Abril Rodriguez-Maldonado; Ariadna Medina-Benitez; Claudia Wong-Arambula; Ernesto Ramirez-Gonzalez.; Gisela Barrera-Badillo; Irma Lopez-Martinez; Joaquin Quiroz-Mercado; Lucia Hernandez-Rivas; Natividad Cruz-Ortiz; Sergio Rangel-Guerrero; Tatiana Nunez-Garcia; Vanessa Rivero-Arredondo
EPI_ISL_1805500, EPI_ISL_1805503, EPI_ISL_1805504	LESP Nuevo Leon	Instituto de Diagnostico y Referencia Epidemiologicos (INDRE)	Abril Rodriguez-Maldonado; Ariadna Medina-Benitez; Claudia Wong-Arambula; Ernesto Ramirez-Gonzalez.; Gisela Barrera-Badillo; Irma Lopez-Martinez; Joaquin Quiroz-Mercado; Lucia Hernandez-Rivas; Natividad Cruz-Ortiz; Sergio Rangel-Guerrero; Tatiana Nunez-Garcia; Vanessa Rivero-Arredondo
EPI_ISL_2105693	LESP Quintana Roo	Instituto de Diagnostico y Referencia Epidemiologicos (INDRE)	Abril Rodriguez-Maldonado; Ariadna Medina-Benitez; Claudia Wong-Arambula; Ernesto Ramirez-Gonzalez.; Gisela Barrera-Badillo; Irma Lopez-Martinez; Joaquin Quiroz-Mercado; Lucia Hernandez-Rivas; Natividad Cruz-Ortiz; Sergio Rangel-Guerrero; Tatiana Nunez-Garcia; Vanessa Rivero-Arredondo
EPI_ISL_2296084	LESP Tamaulipas	Instituto de Diagnostico y Referencia Epidemiologicos (INDRE)	Abril Rodriguez-Maldonado; Ariadna Medina-Benitez; Claudia Wong-Arambula; Ernesto Ramirez-Gonzalez.; Gisela Barrera-Badillo; Irma Lopez-Martinez; Joaquin Quiroz-Mercado; Lucia Hernandez-Rivas; Natividad Cruz-Ortiz; Sergio Rangel-Guerrero; Tatiana Nunez-Garcia; Vanessa Rivero-Arredondo
EPI_ISL_1608054	LHUB-ULB	Labo Klinische Biologie, UZA	Basil Britto Xavier; Christine Lammens; Herman Goossens; Jasmine Coppens; Marie Le Mercier; Veerle Matheeussen
EPI_ISL_1250700	LabPLUS	Institute of Environmental Science and Research (ESR)	Anja Werno; Antje van der Linden; Arlo Upton; Chris Mansell; David Hammer; Dragana Drinkovic; Erasmus Smit; Gary McAuliffe; Hana Sofia Andersson; Hermes Perez; James Ussher; Jill Sherwood; Jing Wang; Joep de Ligt; Josh Freeman; Julia Howard; Juliet Elvy; Lauren Jelly; Mary DeAlmeida; Matt Blakiston; Matt Storey; Matthew Rogers; Max Bloomfield; Michael Addidle; Michelle Balm; Muhammad Faisal; Nikki Freed; Olin Silander; Olivia Stroeven; Rachel Boyle; Sally Roberts; SallyAnn Harbison; Sarah Jefferies; Sharmini Muttaiyah; Susan Morpeth; Susan Taylor; Timothy Blackmore; Vani Sathyendran; Veronica Playle; Virginia Hope; Xiaoyun Ren
EPI_ISL_1738825, EPI_ISL_1739172, EPI_ISL_1739174	Labeto - CAB - Leiria	Instituto Nacional de Saude (INSA)	Borges et al
EPI_ISL_1381222, EPI_ISL_1417191, EPI_ISL_1417204, EPI_ISL_1417205, EPI_ISL_1594450, EPI_ISL_1788821, EPI_ISL_1788822, EPI_ISL_1788823, EPI_ISL_1788824, EPI_ISL_1788825, EPI_ISL_1788826, EPI_ISL_1788827, EPI_ISL_1788829, EPI_ISL_1788830, EPI_ISL_1788831, EPI_ISL_1788832, EPI_ISL_1788833, EPI_ISL_1788834, EPI_ISL_1788835, EPI_ISL_1788836, EPI_ISL_1788837, EPI_ISL_1788838, EPI_ISL_1821633, EPI_ISL_1821634, EPI_ISL_1821635, EPI_ISL_1821636, EPI_ISL_1821637, EPI_ISL_1821638, EPI_ISL_1821639, EPI_ISL_1821640, EPI_ISL_1821641, EPI_ISL_1821642, EPI_ISL_1821643, EPI_ISL_1821644, EPI_ISL_1821645, EPI_ISL_1821646, EPI_ISL_1821647, EPI_ISL_1821648, EPI_ISL_1821649, EPI_ISL_1821650, EPI_ISL_1821651, EPI_ISL_1821652, EPI_ISL_1821653, EPI_ISL_1821654, EPI_ISL_1821657			
see above	Labo Analyses Med	National Reference Center for Viruses of Respiratory Infections,	Amaury Vaysse; Angela Brisebarre; Camille Capel; Catherine Coignard; Christophe Malabat; Corinne Maufrais; Dominique Rousset; Emmanuelle Permal; Etienne Simon-Lorière; Frédéric Lemoine; Louise Lefrançois; Marion Barbet; Maud Vanpeene; Méline Bizard; OphéLie Said-Delattre; Pierre-Yves Leonard; Rousset Dominique; Sylvie Behillil; Sylvie Van der Werf; Sylvie van der Werf; Vincent Enouf

		Institut Pasteur, Paris	
EPI_ISL_1593984	Labo Carage	Institut Pasteur de la Guyane	Anne Lavergne; Dominique Rousset
EPI_ISL_1727096	Labor Dr. Heidrich & Kollegen MVZ GmbH Hamburg	Robert Koch Institute	
EPI_ISL_1217110	Labor Dr. Wisplinghoff - Köln	Robert Koch Institute	
EPI_ISL_1153503	Labor Lübeck bzw. Laborärztliche Gemeinschaftspraxis Lübeck	Robert Koch Institute	
EPI_ISL_1573258, EPI_ISL_1573260	Labor Prof. Dr. G. Enders MVZ GbR	Robert Koch Institute	
EPI_ISL_1627106, EPI_ISL_1627107, EPI_ISL_1628330, EPI_ISL_1628331, EPI_ISL_2227919, EPI_ISL_2227920, EPI_ISL_2227921, EPI_ISL_2227922, EPI_ISL_2227923, EPI_ISL_2227924, EPI_ISL_2227925, EPI_ISL_2227926			
see above	Laboratoire Carage	Institut Pasteur de la Guyane	A. Salmier; Anne Lavergne; Dominique Rousset
EPI_ISL_1259270	Laboratoire Hospitalier Universitaire de Bruxelles (LHUB-ULB)	UAntwerp, Laboratory of Medical Microbiology	Basil Britto Xavier; Christine Lammens; Herman Goossens; Jasmine Coppens; Marie Le Mercier; Veerle Matheeussen
EPI_ISL_2374083, EPI_ISL_2374084, EPI_ISL_2374085, EPI_ISL_2374086	Laboratoire Virologie Saint Louis APHP	Laboratoire Virologie Saint Louis APHP	Constance Delauguerre; Jérome Le Goff; Linda Feghoul; Marie Laure Chaix; Marie Laure Néré; Maud Salmona; Severine Mercier Delarue; Sophia Achaibou
EPI_ISL_1384156	Laboratoire national de sante, Microbiology, Virology	Laboratoire national de sante, Microbiology, Microbial Genomics Platform	Anke Wienecke-Baldacchino; Catherine Ragimbeau; Fatu Djabi; Jessica Tapp; Lise Pignon; Raoul Salmon; Tamir Abdelrahman; Trung Nguyen Nguyen
EPI_ISL_1384478	Laboratoires d'analyses medicales - Ketterthill	Laboratoire national de sante, Microbiology, Microbial Genomics Platform	Anke Wienecke-Baldacchino; Caroline Scheiber; Catherine Ragimbeau; Fatu Djabi; Jessica Tapp; Lise Pignon; Raoul Salmon; Serge Vedy; Tamir Abdelrahman
EPI_ISL_2289143, EPI_ISL_2308280	Laboratori de Referencia de Catalunya	Laboratori de Referencia de Catalunya	Bellosillo B.; Canal M.; Hernandez JJ.; Padilla E.; Ramirez A.; Vilas A.
EPI_ISL_2157480, EPI_ISL_2157481, EPI_ISL_2157491, EPI_ISL_2196258, EPI_ISL_2196270, EPI_ISL_2196271, EPI_ISL_2196272, EPI_ISL_2196273, EPI_ISL_2196274, EPI_ISL_2196275, EPI_ISL_2196276, EPI_ISL_2196277, EPI_ISL_2196278, EPI_ISL_2196279, EPI_ISL_2196280, EPI_ISL_2557354, EPI_ISL_2557356			
see above	Laboratorio Central de Saude Publica do Estado de Minas Gerais (LACEN/MG)	Laboratory of Respiratory Viruses and Measles, Oswaldo Cruz Institute, FIOCRUZ	Alice Sampaio Rocha; Ana Carolina Mendonca; Andre Felipe Leal Bernardes; Anna Carolina Paixao; Elisa Cavalcante Pereira; Fernando Motta; Luciana Appolinario; Marilda Siqueira on behalf of the Fiocruz COVID-19 Genomic Surveillance Network; Paola Resende; Renata Serrano Lopes; Taina Venas
EPI_ISL_2196243, EPI_ISL_2196281, EPI_ISL_2196282, EPI_ISL_2196283, EPI_ISL_2196284, EPI_ISL_2196285, EPI_ISL_2196286, EPI_ISL_2196287, EPI_ISL_2196288, EPI_ISL_2196289, EPI_ISL_2196290, EPI_ISL_2196291, EPI_ISL_2196292, EPI_ISL_2196293, EPI_ISL_2196294, EPI_ISL_2196295, EPI_ISL_2196296, EPI_ISL_2196297, EPI_ISL_2196298, EPI_ISL_2196299, EPI_ISL_2196300, EPI_ISL_2196301, EPI_ISL_2196302, EPI_ISL_2196303, EPI_ISL_2443557, EPI_ISL_2443558, EPI_ISL_2443559, EPI_ISL_2443560, EPI_ISL_2443563, EPI_ISL_2443564, EPI_ISL_2645819, EPI_ISL_2645820, EPI_ISL_2645821, EPI_ISL_2645822, EPI_ISL_2645823, EPI_ISL_2645824, EPI_ISL_2645826, EPI_ISL_2645827, EPI_ISL_2645828, EPI_ISL_2645829, EPI_ISL_2645830, EPI_ISL_2645831, EPI_ISL_2645832, EPI_ISL_2645838, EPI_ISL_2645840, EPI_ISL_2645842, EPI_ISL_2645844, EPI_ISL_2645845, EPI_ISL_2645847, EPI_ISL_2645848, EPI_ISL_2645849, EPI_ISL_2645850, EPI_ISL_2645851, EPI_ISL_2645852, EPI_ISL_2645853, EPI_ISL_2645854, EPI_ISL_2645856, EPI_ISL_2645862, EPI_ISL_2645863, EPI_ISL_2645864, EPI_ISL_2645865, EPI_ISL_2645866, EPI_ISL_2645867, EPI_ISL_2645868, EPI_ISL_2645869, EPI_ISL_2645871, EPI_ISL_2645872, EPI_ISL_2645873, EPI_ISL_2645874, EPI_ISL_2645875, EPI_ISL_2645876, EPI_ISL_2645877, EPI_ISL_2645878, EPI_ISL_2645879, EPI_ISL_2645880, EPI_ISL_2645883, EPI_ISL_2645884, EPI_ISL_2645886, EPI_ISL_2645887, EPI_ISL_2645888, EPI_ISL_2645889, EPI_ISL_2645890, EPI_ISL_2645891, EPI_ISL_2645892, EPI_ISL_2645893, EPI_ISL_2645894, EPI_ISL_2645895, EPI_ISL_2645896, EPI_ISL_2645897, EPI_ISL_2645898, EPI_ISL_2645899, EPI_ISL_2645900, EPI_ISL_2645901, EPI_ISL_2645902, EPI_ISL_2645903, EPI_ISL_2645905, EPI_ISL_2645906, EPI_ISL_2645907, EPI_ISL_2645908, EPI_ISL_2645910, EPI_ISL_2645911, EPI_ISL_2645912, EPI_ISL_2645913, EPI_ISL_2645914, EPI_ISL_2645915, EPI_ISL_2645916, EPI_ISL_2645918, EPI_ISL_2645919, EPI_ISL_2645920, EPI_ISL_2645921, EPI_ISL_2645922, EPI_ISL_2645923, EPI_ISL_2645924, EPI_ISL_2645925, EPI_ISL_2645926, EPI_ISL_2645930, EPI_ISL_2645931, EPI_ISL_2645932, EPI_ISL_4080768			
see above	Laboratorio Central de Saude Publica do Estado do Para (LACEN/PA)	Laboratory of Respiratory Viruses and Measles, Oswaldo Cruz Institute, FIOCRUZ	Agatha Soares; Alice Sampaio Rocha; Ana Carolina Mendonca; Anna Carolina Paixao; Elisa Cavalcante Pereira; Fernando Motta; Ighor Arantes; Luciana Appolinario; Marilda Siqueira on behalf of the Fiocruz COVID-19 Genomic Surveillance Network; Paola Resende; Renata Serrano Lopes; Taina Venas; Valnete Andrade
EPI_ISL_3982691	Laboratorio Antonello Pelotas Rio Grande do Sul	Hemocentro de Ribeirao Preto FMRP USP	Antonio Jorge Martins; Claudia Renata dos Santos Barros; David Schlesinger; Debora Botequio Moretti; Dimas Tadeu Covas; Elaine Cristina Marqueze; Elaine Vieira Santos; Evandra Strazza Rodrigues; Heidge Fukumasu; Jayme Augusto de Souza-Neto; José Salvatore Leister Patané; Luiz Alcantara; Luiz Lehmann Coutinho; Maria Carolina Elias; Maurício Lacerda Nogueira; Rafael dos Santos Bezerra; Raul Machado Neto; Rejane Maria Tommasini Grotto; Ricardo Haddad; Rodrigo Proto de Siqueira; Sandra Coccuzzo Sampaio Vessoni; Simone Kashima; Svetoslav Nanev Slavov; VV Cantarelli; Vincent Louis Viala
EPI_ISL_3982692	Laboratorio Antonello Pelotas, Rio Grande do Sul	Hemocentro de Ribeirao Preto FMRP USP	Antonio Jorge Martins; Claudia Renata dos Santos Barros; David Schlesinger; Debora Botequio Moretti; Dimas Tadeu Covas; Elaine Cristina Marqueze; Elaine Vieira Santos; Evandra Strazza Rodrigues; Heidge Fukumasu; Jayme Augusto de Souza-Neto; José Salvatore Leister Patané; Luiz Alcantara; Luiz Lehmann Coutinho; Maria Carolina Elias; Maurício Lacerda Nogueira; Rafael dos Santos Bezerra; Raul Machado Neto; Rejane Maria Tommasini Grotto; Ricardo Haddad; Rodrigo Proto de Siqueira; Sandra Coccuzzo Sampaio Vessoni; Simone Kashima; Svetoslav Nanev Slavov; VV Cantarelli; Vincent Louis Viala
EPI_ISL_3982703, EPI_ISL_3982710, EPI_ISL_3982716, EPI_ISL_3982718, EPI_ISL_3982719, EPI_ISL_3982722, EPI_ISL_3982728, EPI_ISL_3982732, EPI_ISL_3982734, EPI_ISL_3982735, EPI_ISL_3982736, EPI_ISL_3982737, EPI_ISL_3982738, EPI_ISL_3982739, EPI_ISL_3982743, EPI_ISL_3982746, EPI_ISL_3982747, EPI_ISL_3982750, EPI_ISL_3982753, EPI_ISL_3982755, EPI_ISL_3982757, EPI_ISL_3982758, EPI_ISL_3982760, EPI_ISL_3982765			
see above	Laboratorio Antonello, Pelotas, Rio Grande do Sul	Hemocentro de Ribeirao Preto FMRP USP	Antonio Jorge Martins; Claudia Renata dos Santos Barros; David Schlesinger; Debora Botequio Moretti; Dimas Tadeu Covas; Elaine Cristina Marqueze; Elaine Vieira Santos; Evandra Strazza Rodrigues; Heidge Fukumasu; Jayme Augusto de Souza-Neto; José Salvatore Leister Patané; Luiz Alcantara; Luiz Lehmann Coutinho; Maria Carolina Elias; Maurício Lacerda Nogueira; Rafael dos Santos Bezerra; Raul Machado Neto; Rejane Maria Tommasini Grotto; Ricardo Haddad; Rodrigo Proto de Siqueira; Sandra Coccuzzo Sampaio Vessoni; Simone Kashima; Svetoslav Nanev Slavov; VV Cantarelli; Vincent Louis Viala
EPI_ISL_3982713, EPI_ISL_3982733	Laboratorio Antonello, Pelotas, Rio Grande do Sul	Hemocentro de Ribeirao Preto/FMRP-USP	Antonio Jorge Martins; Claudia Renata dos Santos Barros; David Schlesinger; Debora Botequio Moretti; Dimas Tadeu Covas; Elaine Cristina Marqueze; Elaine Vieira Santos; Evandra Strazza Rodrigues; Heidge Fukumasu; Jayme Augusto de Souza-Neto; José Salvatore Leister Patané; Luiz Alcantara; Luiz Lehmann Coutinho; Maria Carolina Elias; Maurício Lacerda Nogueira; Rafael dos Santos Bezerra; Raul Machado Neto; Rejane Maria Tommasini Grotto; Ricardo Haddad; Rodrigo Proto de Siqueira; Sandra Coccuzzo Sampaio Vessoni; Simone Kashima; Svetoslav Nanev Slavov; VV Cantarelli; Vincent Louis Viala
EPI_ISL_1804917	Laboratorio Aziendale di Microbiologia e Virologia, Azienda Sanitaria dell'Alto Adige	Laboratorio Aziendale di Microbiologia e Virologia, Azienda Sanitaria dell'Alto Adige	Anne Picard; Bartolomeo Mobilio Rodriguez; Chiara Cantaloni; Claudia Volpato; Elisa Masi; Elisabetta Giacobazzi; Elisabetta Pagani; Irene Bianconi; Stefanie Wieser
[truncated: 487,636 more chars]
